# Responses to Reduced Feeding Frequency in Captive-Born Cheetahs (*Acinonyx jubatus*): Implications for Behavioural and Physiological Stress and Gastrointestinal Health

**DOI:** 10.3390/ani13172783

**Published:** 2023-08-31

**Authors:** Kelsey Lee Brown, André Ganswindt, Gerhard Steenkamp, Adrian Stephen Wolferstan Tordiffe

**Affiliations:** 1 Department of Paraclinical Sciences, Faculty of Veterinary Science, University of Pretoria, Pretoria 0110, South Africa; 2 Centre for Veterinary Wildlife Studies, Faculty of Veterinary Science, University of Pretoria, Pretoria 0110, South Africa; 3 Department of Zoology and Entomology, Faculty of Natural and Agricultural Sciences, Mammal Research Institute, University of Pretoria, Pretoria 0028, South Africa; 4 Department of Companion Animal Clinical Studies, Faculty of Veterinary Science, University of Pretoria, Pretoria 0110, South Africa

**Keywords:** cheetah, captive diet, wildlife husbandry, gastrointestinal health, stress

## Abstract

**Simple Summary:**

This study examined how offering larger quantities of food less frequently to better replicate their natural feeding pattern could affect the health of captive-born cheetahs. For three weeks, six hand-reared cheetahs were fed four once-daily meals per week, followed by three weeks in which they were fed two daily rations six days a week for the same duration while maintaining their total weekly food intake. The studied cheetahs showed higher faecal consistency scores and activity levels when fed less frequently. The results indicate that reducing feeding frequency could benefit captive cheetahs’ gastrointestinal health without causing significant stress.

**Abstract:**

Unnatural diet composition and frequent feeding regimes may play an aetiological role in the multiple diseases prevalent in captive cheetahs. This study investigated the responses of captive-born (hand-reared) cheetahs (*n* = 6) to a reduced feeding frequency schedule distinguished by offering larger quantities of food less frequently. The study cheetahs were fed four once-daily meals per week during the 3-week treatment period, followed by a 3-week control period in which they were fed two daily rations six days a week. Total weekly food intake was maintained throughout the study. Variations in behaviour, faecal consistency score (FCS), and faecal glucocorticoid metabolite concentration were measured. Less frequent feeding resulted in higher FCS (*p* < 0.01) and locomotory behaviour (*p* < 0.05) among the studied cheetahs. Faecal glucocorticoid metabolite concentration demonstrated an initial acute stress response to the change in feeding frequency (*p* < 0.05) and subsequent adaptation. The results of the FCS analysis suggest that the more natural feeding pattern could have benefited the studied cheetahs’ gastrointestinal health without a significant behavioural or physiological stress response overall to the change in feeding frequency.

## 1. Introduction

In the wild, cheetahs (*Acinonyx jubatus*) typically prey on small to medium-sized antelope species with a body mass of between 23 and 56 kg [1] and, if left undisturbed, can consume a large proportion of the carcass [2]. They seldom eat daily or at a fixed interval; cheetahs exhibit a feeding pattern alternating between consuming large meals and periods of limited or no food influenced by irregular prey availability in their natural habitat [3]. However, captive cheetahs are routinely fed a nonvarying diet of skinned muscle meat from livestock species, commercially prepared carnivore diets, carcass parts, or a combination [4,5] offered at fixed intervals once or twice daily, with only one fast day per week. A whole carcass diet of the cheetah’s natural prey species and the associated feeding habits are challenging to replicate in captivity [6]. Some facilities argue that frequent feeding allows for daily monitoring of the animals’ appetites as an indicator of health and reduces boredom-induced stress [7]. However, unnatural diet composition and frequent feeding regimes may play an aetiological role in the prevalence of gastrointestinal (GI) and metabolic diseases in captive cheetahs [5,8].

One of these diseases, *Helicobacter*-associated gastritis, causes significant morbidity and mortality in captive cheetahs worldwide [9,10,11]. *Helicobacter* species, spiral bacteria colonising the stomach, infect most captive and wild cheetahs [12]. Captive cheetahs typically have some degree of inflammation (gastritis) that can be asymptomatic or associated with regurgitation, vomiting, the passage of undigested food, and weight loss [13,14]. However, in free-ranging cheetahs, there is colonisation by abundant spiral bacteria but little to no associated inflammation [10,15], demonstrating the likely multifactorial aetiopathogenesis of gastritis.

Diet, as a potential risk factor for GI pathology in captive cheetahs, was previously dismissed for the most part [10]. More recently, Whitehouse-Tedd et al. [5] found that feeding horsemeat has a significant (detrimental) relationship with gastritis risk in captive cheetahs. They attributed this to its high protein content [5] and/or digestibility [16] relative to other meat types fed to captive cheetahs. Crude protein is anticipated to be high in all carnivorous diets, including that of free-ranging cheetahs. However, the frequency with which it is fed in captivity and its quality could affect GI health by changing the amount of protein reaching the large intestine. Colonic fermentation of poorly digested dietary protein modifies microbiota composition in favour of proteolytic bacteria, some of which can be pathogenic in high concentrations [17,18] and produce putrefactive compounds (e.g., ammonia, indoles, phenols) associated with various disease states [19,20,21]. Moreover, horse (in particular) is commonly fed as muscle meat without low to nondigestible collagen-rich matter (e.g., bone, tendons, cartilage); therefore, its relative lack of ‘animal fibre’ may further increase putrefaction of digesta in the intestine [19,20,22,23].

Transforming gut bacteria-derived putrefactants into toxic metabolites negatively affects multiple other organ systems and metabolic pathways. Uraemic toxicity of indoxyl sulphate is associated with the progression of chronic renal failure [24], a significant cause of death in captive cheetahs [25,26,27,28]. The renal lesions in captive cheetahs resemble diabetic glomerulopathy in humans and chronic progressive nephropathy in rats [25]. High-protein diets, particularly when fed ad libitum and continually, accelerate glomerulosclerosis in rats [29,30,31] and could be a comparable dietary risk factor for kidney damage in captive cheetahs.

A growing body of evidence suggests that feeding restrictions shape the gut ecosystem, function, and interaction with the host. Intermittent fasting has beneficial regulatory effects on immune homeostasis and intestinal microbiota composition in human and rodent models [32,33,34,35]. Furthermore, intermittent fasting attenuates the colon tissue inflammatory response and oxidative stress [32,36]. Following the adaption of captive lions (*Panthera leo*) from a conventional zoo feeding programme of predictable, fixed, small daily meals to a more natural gorge and fast feeding schedule of larger, more infrequent meals, Altman et al. [37] reported improved digestibility of a horsemeat-based diet. Considering similarities in the species’ natural feeding ecology, fasting conditions could have digestive health benefits for the cheetah as they did for the lion.

This study’s overall aim was to investigate the responses of captive-born (hand-reared) cheetahs to a reduced feeding frequency schedule distinguished by offering larger quantities of food less frequently. We hypothesised that a more natural feeding pattern would beneficially impact the GI ecosystem, including the microbial fermentation process. In previous studies, faecal consistency scoring has been used as a noninvasive method of measuring GI health in cheetahs, other exotic felids [5,38], and domestic carnivore species [39,40]. We also hypothesised that changing the feeding frequency may result in a behavioural and/or physiological stress response. Poor faecal consistency has been linked to GI stress in captive carnivores [38]. Behavioural observations [41] and faecal glucocorticoid metabolite (fGCM) analysis [42] were used as more established stress-related markers in the cheetah. In addition, we explored the use of biologging technology to record body temperature (T_b_), heart rate (HR), and locomotor activity (LA) simultaneously (refer to Appendix A). We predicted that higher fGCM concentrations, T_b_, and HR would indicate a physiological stress response.

## 2. Materials and Methods

### 2.1. Study Site and Animals

The experimental trials took place between April and September 2019 at the Cango Wildlife Ranch and Conservation Centre (33°33′ S, 22°12′ E), 4 km north of Oudtshoorn, a semiarid region in the Western Cape of South Africa. Study months (autumn and mainly winter) were distinguished by short photoperiods and cold air temperatures (T_a_), ranging from 13 to 17 °C.

Three male (CH-2205, -2206, and -2271) and three female (CH-2207, -2276, and -2277) adult cheetahs (Table 1) habituated to human presence and interacting daily with the facility’s caretakers in the absence of restraint were assigned to this study. The study cheetahs were housed off-exhibit at the Jill Bryden-Fayers Reserve, neighbouring the Cango Wildlife Ranch. They were held in outdoor enclosures ranging from 400 to 1350 m^2^, adjoining conspecifics. The enclosures’ topography was varied and naturalistic, consisting of a dirt substrate, vantage points and marking areas (e.g., rocks, tree stumps), sufficient vegetation to hide, and a wooden shed for shelter. Enclosures were cleaned once or twice daily.

### 2.2. Experimental Design

The experimental trials commenced following a ≥12-day washout after surgical implantation of the biologgers (refer to Appendix A.1.2. Surgical procedures). In a pilot study conducted on the study cheetahs, T_b_, HR, LA, faecal consistency score (FCS), and fGCM concentration data demonstrated temporal recovery by postoperative day 10 [43]; therefore, the authors felt ≥12 days after surgery to be sufficiently long to commence the experimental trials. They were conducted using a within-subject experimental design, where each cheetah in the study served as their own control. Grouped by their respective ages, the study cheetahs received the treatment, i.e., a reduced feeding frequency schedule in an initial 3-week period followed by a 3-week control period, against which the effects of the treatment were measured (Table 2).

At the Cango Wildlife Ranch, cheetahs are fed a horsemeat-based diet prepared on-site, weighed, and recorded. To supplement the nutritional composition of horsemeat, i.e., moisture: 71.9%, dry matter: 29.1%, crude protein: 19.8%, and crude fat: 6.63% [44], 7.5 g of predator powder (V-Tech Pty Ltd., Midrand, South Africa; containing 35 g of calcium per 100 g of powder) and 20 g of glycine (WildCat Nutrition Pty Ltd., Pretoria, South Africa) is added per 1 kg of meat [45]. Regarding paired housing (Table 1), 1 tbsp of uncooked (nondigestible) rice was thoroughly mixed into the diet of study cheetahs CH-2206 and CH-2277 once daily to assign individual faecal samples—the facility’s caretakers separate cheetahs during feeding to reduce competition for food and prevent meal sharing. Meals are offered at variable intervals to prevent food anticipatory behavioural activity [46]. Once weekly at random, cheetahs are fed horse shank or rib bones with some meat intact equivalent to the weight of their daily ration in place of the day’s meals to maintain variety and provide periodontal stimulation. The bones are not consumed by the cheetahs and are removed and discarded. Leftover meat is removed, weighed, recorded, and discarded. Water is available ad libitum.

During the 3-week treatment period, the study cheetahs were fed on a reduced feeding frequency schedule, where meals were offered once daily between 08.00 and 17.00 h, Monday, Tuesday, Thursday, and Friday (Table 2). Weekly fasting days were assigned to Wednesday, Saturday, and Sunday. Larger than regular meals were offered on feed days to maintain total weekly food intake despite additional fast days. The three-year-old study cheetahs (CH-2205, -2206, and -2207) were fed 2.7 kg per day four days a week, and the two-year-old study cheetahs (CH-2271, -2276, and -27) were fed 2.5 kg per day four days a week. During the following 3-week control period, the study cheetahs were fed on a feeding schedule routinely used at the Cango Wildlife Ranch, where meals were offered twice daily between 08.00–12.00 and 15.00–17.00 h, Monday to Saturday. Weekly fasting days are assigned to Sundays. The three-year-old study cheetahs were fed 1.8 kg per day portioned into two rations six days a week, and the two-year-old study cheetahs were fed 1.6 kg per day portioned into two daily rations six days a week. There was a washout between the treatment and control, during which the study cheetahs were fed on the routine feeding schedule.

Other than the specific intervention being investigated, i.e., a reduced feeding frequency schedule, the study cheetahs’ environment, housing, and management (refer to Section 2.1. Study Site and Animals) were maintained across the treatment and control, including bones offered randomly once weekly in place of the day’s meals.

Throughout the study, i.e., in the treatment and control, each cheetah was monitored regarding (i) behaviour, (ii) FCS, and (iii) fGCM concentration.

### 2.3. Behavioural Data Collection

Each cheetah in the study was observed 15 times during the treatment and control, respectively. These observations were conducted between 07.00 and 17.00 h, Monday to Sunday, within the operating hours of the Cango Wildlife Ranch. During five weekly 60 min observation sessions, the principal investigator (KLB) carried out 12 instantaneous scan samples [47] with a 5 min interscan interval per enclosure. Regarding paired housing (Table 1), the study cheetahs were observed together using physical identifiers to assign individual behaviour. Sampling was conducted on a variable day-and-time basis between enclosures, randomly selected to prevent time-of-day effects.

The study recorded 15 behaviours categorised as ‘inactive’, ‘active’, and ‘not observed’ (Table 3), informed by the previous literature on felid behaviour (specifically cheetahs) [37,48,49,50] and initial observations of the cheetahs being studied. Time spent out of sight (hiding or staying away from the human observer) was noted as its performance has been linked to a psychological stress response in felids [51,52,53,54,55].

### 2.4. Faecal Sample Collection and Consistency Scoring

During the operating hours of the Cango Wildlife Ranch (08.00–17.00 h), faeces were collected within one hour after defecation. Faeces excreted between 17.00–08.00 h, when the study cheetahs’ enclosures could not be entered, were collected within 16 h after defecation (4–10 °C T_a_) [56].

Following sample collection, the principal investigator (KLB) assigned FCS using a five-point faecal scoring system adapted from that developed by Whitehouse-Tedd et al. [5]. In this study, the five-point faecal scoring system used (grades ranging from 1 to 5, where grade 1 was the lowest and grades 2–5 were progressively higher) included two points (grade 4: firm and dry, and grade 5: firm) considered to be ‘normal,’ and three points (grades 1–3: liquid, soft without shape, and soft with shape) considered to be ‘suboptimal’ according to free-ranging cheetah scat. As a species inhabiting semiarid regions (such as Oudtshoorn), dry faecal consistency was not considered dissimilar to faeces found in free-ranging cheetahs [45].

Afterwards, the samples were deposited into appropriately labelled (sample collection date, study cheetah, and sample identification number) 50 mL polypropylene specimen containers and frozen at −20 °C.

### 2.5. Faecal Steroid Extraction and Quantification

Following completion of the experimental trials, faecal samples were transported frozen to the Endocrine Research Laboratory, University of Pretoria, South Africa. Faecal steroids were extracted and subsequently analysed for fGCM concentration.

Frozen faecal samples were lyophilised, and the resultant dry faeces were pulverised and sieved through a mesh strainer to remove fibrous material [57]. Between 0.050 and 0.055 g of faecal powder was weighed per sample and extracted using 3 mL of 80% ethanol. The suspensions were vortexed for 15 min and centrifuged at 1500× *g* for 10 min [58]. Supernatants were decanted into 1.5 mL safe-lock microcentrifuge tubes, labelled, and frozen at −20 °C until further analysis.

Immunoreactive fGCM concentrations were quantified using a corticosterone-3-CMO enzyme immunoassay (EIA) [56,59] according to procedures described by Ganswindt et al. [60]. Detailed assay characteristics, including full descriptions of the assay components and antibody cross-reactivities, are provided by Palme and Möstl [59]. The sensitivity of the EIA used at 90% binding was 3.6 ng/g faecal dry weight (DW). Interassay coefficients of variation (CV), determined by repeated measurements of low- and high-quality controls, were 11.74% and 12.91%, respectively, and intraassay CV were 5.59% and 6.61%, respectively. Faecal steroid concentrations are presented as µg/g faecal DW.

### 2.6. Data Preparation

#### 2.6.1. Behavioural Observations

The frequency with which each cheetah in the study performed each behaviour during each observation session was calculated as a proportion of the total number of scan samples carried out during that observation session per study cheetah [48,49]. The resulting data highlighted the proportion of scan samples in which each behaviour was observed during the treatment and control and on feeding and fasting days for each study cheetah.

#### 2.6.2. Faecal Consistency Scores and Glucocorticoid Metabolite Concentrations

Cheetahs typically defecate once daily, attenuating diurnal and pulsatory glucocorticoid (GC) secretion variations in the faeces [61]. However, differences in species and individual traits can affect hormone concentrations and GI transit time [62]. In a study by Terio et al. [42], peak concentrations of GC metabolites were found in the first faecal sample collected from cheetahs after administering adrenocorticotropic hormone. This was comparable to domestic cats’ faecal cortisol excretion rate [63]. This study assumed FCS and fGCM concentrations to reflect the previous day’s intervention to account for cheetahs’ specific 24 h gut passage rate and excretory pattern. The within-subject experimental design, where each cheetah served as their own control, eliminated interindividual variability [64].

### 2.7. Statistical Analysis

Statistical analysis was performed using Microsoft Excel (version 16.0) and JMP Pro software (version 16.0) for Windows, developed by SAS Institute Inc (Cary, NC, USA). The variables measured were explored for univariate outliers greater than three interquartile ranges (IQR) away from the 99.5th or 0.05th percentiles. No outlying values were detected. Normal distribution and homogeneity of variance were explored using Anderson-Darling [65] and Levene’s tests [66], respectively. Behaviour, FCS, and fGCM concentration data were Box–Cox transformed [67] to satisfy the assumption of normality and homogeneity due to their departure. The data were back-transformed for descriptive statistics and visual representation to maintain statistical integrity. In this study, a mixed model for repeated measures (MMRM) analysis [68] was used to investigate the independent fixed effects of the study period and feed versus fast day on (i) behaviour, (ii) FCS, and (iii) fGCM concentration and their interaction to test the moderator effect of the study period on feed versus fast day. An MMRM analysis investigated the independent fixed effect of treatment week (one, two, and three) on (i) behaviour, (ii) FCS, and (iii) fGCM concentration. The study cheetah was included as the random effect in the analyses.

Multiple pairwise comparisons were explored using Tukey’s honestly significant difference (HSD) post-hoc tests [69]. Effect sizes of pairwise comparisons were calculated using the following formula:

*d* = *x*_1_ − *x*_2_/√*SD*_1_^2^ + *SD*_2_^2^/2, (1)

where *d* = Cohen’s d effect size; *x*_1_ and *x*_2_ = means of the two groups; and *SD*_1_ and *SD*_2_ = standard deviation of the two groups [70]. Root mean square standardised effects (RMSSE) were interpreted as small (*d* = 0.2), medium (*d* = 0.5), and large (*d* = 0.8) based on Cohen’s d effect size criteria. Descriptive statistics were reported as median (IQR), and the significance level, alpha, was set at 0.05.

## 3. Results

### 3.1. Behavioural Observations

Three hundred and sixty scan samples per study cheetah were collected during the treatment (*n* = 180) and control (*n* = 180). The study cheetahs spent most of their time inactive (for the treatment: 40.37% (week one: 37.02%, week two: 40.61%, and week three: 42.13%) and control: 43.72%, and for feeding: 40.11% and fasting days: 51.30%) (Table S1). The MMRM analysis revealed that the fixed effects of the study period and feed versus fast day on each behaviour failed to achieve statistical significance; therefore, post-hoc testing was not performed.

The MMRM analysis revealed that the fixed effect of treatment week on locomotion was significant (*F*_3,99.87_ = 2.90, *p* = 0.037). Post-hoc comparisons using Tukey’s HSD test revealed that locomotion was significantly higher (*t*_99.9_ = 2.94, *p* = 0.021) during week three of the treatment (24.84%) than the control (14.80%) (Table S1).

Effect size calculations using Cohen’s d revealed a medium RMSSE (*d* = 0.77; *t*_101_ = 2.99, *p* = 0.004) on locomotion between week three of the treatment and the control (Table 4).

### 3.2. Faecal Consistency Scores

**Figure 1:**
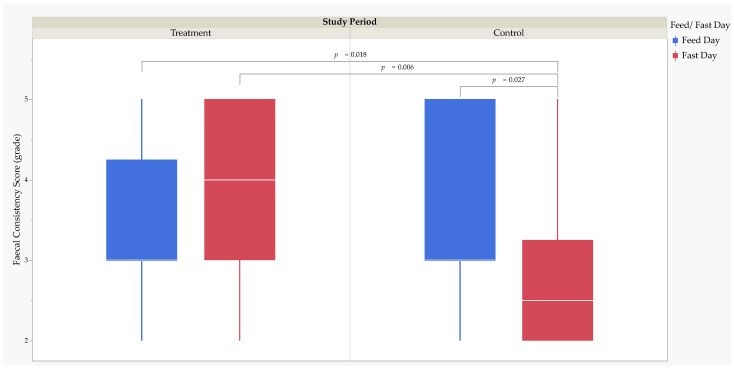
Box and whisker plot of faecal consistency score (grade) for the study cheetahs (CH-2205, -2206, -2207, -2271, -2276, and -2277). Effect of feed versus fast day by the study period. Statistics were performed using Tukey’s honestly significant difference post-hoc test.

Two hundred and thirteen faecal samples were collected from the study cheetahs during the treatment (*n* = 105) and control (*n* = 108). The soft with shape faecal grade was the most frequently recorded in the study cheetahs (for the treatment: 45.71% (week one: 45.45%, week two: 45.95%, and week three: 45.71%) and the control: 48.15%, and for feeding: 48.81% and fasting days: 40.00%) (Table S2). The MMRM analysis revealed that FCS was significantly higher (*F*_1,205.2_ = 10.22, *p =* 0.002) during the treatment (3 (2)) than the control (3 (1)). The MMRM analysis revealed that the fixed effect of feed versus fast day on FCS failed to achieve statistical significance. Post-hoc comparisons using Tukey’s HSD test revealed that FCS was significantly lower on control fasting days (2.5 (1.25)) than on treatment feeding days (3 (1.25); *t*_205.4_ = −2.96, *p =* 0.018), treatment fasting days (4 (2); *t*_205.4_ = −3.31, *p =* 0.006), and control feeding days (3 (2); *t*_205.4_ = −2.83, *p =* 0.027) (Figure 1).

The MMRM analysis revealed that the fixed effect of treatment week on FCS failed to achieve statistical significance; therefore, post-hoc testing was not performed.

Effect size calculations using Cohen’s d revealed a medium RMSSE (*d* = 0.65; *t*_209_ = 3.31, *p* = 0.001) on FCS between the treatment and control (Table 4). The RMSSE of feed versus fast day on FCS by the study period was large between control fasting days and treatment feeding days (*d* = 1.07; *t*_209_ = 3.17, *p* = 0.002), treatment fasting days (*d* = 1.23; *t*_209_ = 3.43, *p* = 0.001), and control feeding days (*d* = 1.01; *t*_209_ = 3.04, *p* = 0.003).

### 3.3. Faecal Glucocorticoid Metabolite Concentrations

The MMRM analysis revealed that the fixed effects of the study period and feed versus fast day on fGCM concentration failed to achieve statistical significance; therefore, post-hoc testing was not performed (Figure S1).

**Figure 2:**
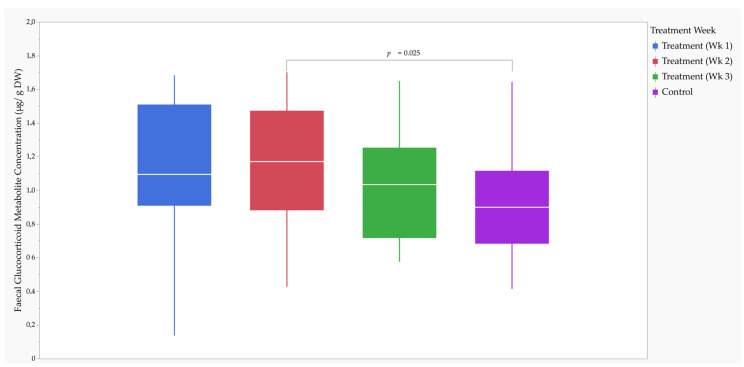
Box and whisker plot of faecal glucocorticoid metabolite concentration (µg/g dry weight (DW)) for the study cheetahs (CH-2205, -2206, -2207, -2271, -2276, and -2277). Effect of treatment week (Wk; one, two, and three). Statistics were performed using Tukey’s honestly significant difference post-hoc test.

The MMRM analysis revealed that the fixed effect of treatment week on fGCM concentration was significant (*F*_3,166.3_ = 3.14, *p* = 0.027). Post-hoc comparisons using Tukey’s HSD test revealed that fGCM concentration was significantly higher (*t*_166.3_ = 2.85, *p* = 0.025) during week two of the treatment (1.17 (0.59) µg/g DW) than the control (0.90 (0.43) µg/g DW) (Figure 2).

Effect size calculations using Cohen’s d revealed a medium RMSSE (*d* = 0.58; *t*_170_ = 2.76, *p* = 0.006) on fGCM concentration between week two of the treatment and the control (Table 4).

## 4. Discussion

This study aimed to investigate the responses of captive-born (hand-reared) cheetahs to a reduced feeding frequency. The results of the FCS analysis support, to some extent, the researchers’ hypothesis that the more natural feeding pattern would beneficially impact the GI ecosystem, including the microbial fermentation process. Overall, the findings indicate that the change in feeding frequency did not result in a significant behavioural or physiological stress response, contrary to what was predicted.

Animals’ GI tracts harbour essential gut microbes serving various functions [18]. However, disturbance-related deviation in the microbial diversity and abundance pattern beyond a natural range, i.e., gut dysbiosis, can advance pathophysiology and affect host health [71]. Considering the purported link with intestinal microbiota composition [72,73], higher FCS indicates that less frequent feeding could have benefited the studied cheetahs’ GI health. The data present here is consistent with Altman et al.’s [37] work concerning the impact of a random gorge and fast feeding schedule on the digestion of a horsemeat-based diet in captive lions.

Studies have shown that chronic or repeated exposure to stressors can disrupt gut homeostasis [74,75]; therefore, an alternative interpretation of this result may be the stress-reducing effects of less frequent feeding as a potential form of environmental enrichment (EE). In animal husbandry, the principle of EE is widely used to provide species-appropriate challenges to captive animals that lack adequate stimuli. This encourages them to engage actively with their environments, reducing stress and stereotypical behaviour [76,77]. One common type of enrichment is food-based, which also applies to the cheetah [41,48,49,78,79].

Absent hunting opportunities, offering carnivores predictable, fixed, and small daily meals can worsen their tendency to be inactive in captivity. This can result in obesity and affect their wellbeing [22]. In this study, the cheetahs spent most of their time inactive, consistent with previous research on captive cheetahs [79] and other felids [80,81,82,83,84,85,86]. The reduced feeding frequency schedule resulted in numerically lower inactivity and higher locomotion (the latter significantly so during week three of the treatment). Increased activity has similarly been reported in captive lions following the adaption from a conventional zoo feeding programme to a randomised feeding schedule [37].

Stress reduction using EE extends to sympathoadrenal responses. The two branches: the sympathetic nervous system (SNS) and the hypothalamic–pituitary–adrenal (HPA) axis, work together to maintain or re-establish homeostasis by orchestrating behavioural and physiological adaptations to the stressor [87]. The SNS provides the immediate first wave of the stress response, mediating the rapid release of the catecholamine hormones epinephrine (E) and norepinephrine from the adrenal medulla [88]. The second wave is more gradual and involves GCs, the product of the hormonal cascade along the HPA axis [89]. Multiple hypotheses have been proposed to explain the stress-reducing effects of EE, some of which are based on the contention that EE itself acts as a mild stressor [90]. By providing challenges appropriate to an animal’s sensory, physical, and cognitive capacities [91], EE is thought to enable arousal and activation of the physiological stress response without pushing the animal into high, maladaptive stress levels [92,93]. In this manner, EE is adaptive, improving animals’ capacity to cope with stressors; that is, stress resilience [94,95,96]. Numerically and significantly higher fGCM concentration during weeks one and two of the treatment, followed by lower values during week three, suggests the studied cheetahs experienced an acute stress response to the change in feeding frequency to which they adapted. Having previously been provided EE may have increased the captive-born cheetahs in this study’s resilience to enrichment-induced arousal.

More work remains to be carried out before fully understanding the optimal feeding regime(s) to beneficially modulate cheetahs’ intestinal microbiota composition. This study has at least four potential limitations to be considered. Firstly, faecal consistency scoring lacks specificity as a discrete measure of GI health and should not be interpreted as providing empirical evidence of diet suitability for maintaining cheetahs in captivity. Further research is needed to validate FCS against other measurements of microbiome health effects, such as digestibility, pH, the incidence of vomiting or diarrhoea, veterinary diagnosis of GI disease, fermentation byproducts, faecal frequency, dry weight percentage, and gut microbiota and short-chain fatty acids.

It must also be acknowledged that the present study was conducted on hand-reared cheetahs. In mammals, there is evidence that the high microbial diversity of infants’ gut communities may be inherited from their biological mothers [97]. Studies have shown that the rearing method (hand- versus mother-reared) can affect animal gut microbiota composition [98,99]. Therefore, the findings should be generalised to only some captive cheetahs.

Secondly, the duration of each period, i.e., treatment, control, and washout, was selected to accommodate the inverse relationship between the number of T_b_, HR, and LA recordings made using the biologgers and the lifespan of their batteries (refer to Appendix A). The 3-week treatment and control periods may have needed to be longer to produce a definitive response in the studied cheetahs, limiting the conclusions that can be made from the results. For example, in mice, stress-related alterations in the composition and function of faecal microbiota were described after eight days, while they appeared after ten days in rats [100,101]. It would be recommendable for future research to lengthen the current study period and washout to quantify the treatment effects better and prevent possible carryover effects.

A third potential limitation to consider is GI transit time. Measuring fGCM concentrations provides an integrated measure of adrenocortical activity. It reflects the cumulative secretion of plasma GCs over several hours (6–24 h, depending on species-specific defecation rate), attenuating fluctuations due to secretory patterns [102,103,104,105]. However, dietary intake could affect the faecal excretion of steroid hormone metabolites independent of a stress response [106]. Due to accelerated GI transit time, larger quantities of food may decrease the accumulation time of faeces in the intestine and increase metabolite concentration variability [107].

By design, there were fewer fasting days during the routine feeding schedule than during the reduced feeding frequency schedule. The days on which the studied cheetahs’ behaviour was observed were not adapted to maintain fast-day observation sessions equivalent between the treatment and control; therefore, it is possible that fasting days were over- and underrepresented during the treatment and control, respectively.

## 5. Conclusions

Though the validity of FCS as a measure of GI health must be established by further research, these results provided preliminary evidence for a reduced feeding frequency schedule to mediate the unnatural composition of horsemeat-based diets routinely fed to captive cheetahs and as an effective EE strategy. While previous studies have mainly examined the epidemiological relationship between diet composition and GI disease [5,19,20,22,23], the findings presented here indicate that feeding regimes may also play a significant role. This study expands on existing research by Whitehouse-Tedd et al. [5] in developing a global standard by which captive facilities can score their cheetahs’ faecal consistency.

## Figures and Tables

**Table 1:** Demographic information of six captive-born (hand-reared) cheetahs.

Group	Identification Number	Housing	DOB ^1^ (YY-MM-DD)	BM1 ^2^ (kg)	BM2 ^3^ (kg)	Sex
1	CH-2205	Paired (with CH-2206)	9 June 2016	45.0	45.0	M ^4^
	CH-2206	Paired (with CH-2205)	9 June 2016	47.15	47.65	M ^4^
	CH-2207	Single	9 June 2016	36.6	37.1	F ^5^
2	CH-2271	Single	28 August 2017	37.65	41.75	M ^4^
	CH-2276	Paired (with CH-2277)	16 September 2017	39.9	39.35	F ^5^
	CH-2277	Paired (with CH-2276)	16 September 2017	43.05	43.55	F ^5^

^1^ DOB: date of birth; ^2^ BM1: body mass at the start of the experimental trials; ^3^ BM2: body mass at the end of the experimental trials; ^4^ M: male; ^5^ F: female.

**Table 2:** The experimental design used in the study.

Group	Identification Number	Reduced Feeding Frequency Schedule (Treatment)					Washout	Routine Feeding Schedule (Control)				
		Period	Feeding Days	Feeding Time(s)	Amount Fed/Day	Fasting Day(s)		Period	Feeding Days	Feeding Time(s)	Amount Fed/Day	Fasting Day(s)
1	CH-2205	15 May 2019 to 4 June 2019	Mon ^1^, Tues ^2^, Thurs ^3^, Fri ^4^	0800–1700 h	2.7 kg	Wed ^5^, Sat ^6^, Sun ^7^	1 day	6 June 2019 to 26 June 2019	Mon ^1^, Tues ^2^, Wed ^5^, Thurs ^3^, Fri ^4^, Sat ^6^	0800–1200 h, 1500–1700 h	1.8 kg	Sun ^7^
	CH-2206											
	CH-2207	23 April 2019 to 13 May 2019					24 days					
2	CH-2271	31 July 2019 to 20 August 2019	Mon ^1^, Tues ^2^, Thurs ^3^, Fri ^4^	0800–1700 h	2.5 kg	Wed ^5^, Sat ^6^, Sun ^7^	1 day	22 August 2019 to 11 September 2019	Mon ^1^, Tues ^2^, Wed ^5^, Thurs ^3^, Fri ^4^, Sat ^6^	0800–1200 h, 1500–1700 h	1.6 kg	Sun ^7^
	CH-2276	9 July 2019 to 29 July 2019					24 days					
	CH-2277											

^1^ Mon: Monday; ^2^ Tues: Tuesday; ^3^ Thurs: Thursday; ^4^ Fri: Friday; ^5^ Wed: Wednesday; ^6^ Sat: Saturday; ^7^ Sun: Sunday.

**Table 3:** The behaviours recorded in the study and their definitions.

Inactive	
Inactive	Laying/asleep, laying/awake, sitting (stationary in a bipedal position)
Active	
Individual behaviour	
Appetitive behaviour	Feeding, food anticipatory activity, stalking
Attention	Staring at one area or paying attention to any visual or auditory stimulus
Autogrooming	Licking or scratching of the own body
Environmental enrichment	Interacting with an enrichment device by biting, dragging, scratching, or carrying it in the mouth
Locomotion	Jumping, running, solitary play, walking
Maintenance	Drinking, defecating/urinating, yawning
Olfactory exploration	Sniffing the air, an object, or the substrate; performing flehmen
Scent marking	Marking substrates or objects in the enclosure by urine-spraying (releasing urine backwards against a vertical surface or object while standing with tail raised vertically), rolling, and rubbing (leaving scents on the substrate or on any object, respectively)
Standing	Stationary in a quadrupedal position
Stereotypical	Pacing (repetitive, apparently functionless locomotory movement along a given route uninterrupted by other behaviours)
Vocalisation	Chirping, growling, purring, stutter-barking, or yowling
Social behaviour	
Affiliative behaviour *	Social play (play-fight, chasing, or playing together with an enrichment item), pawing, or rubbing on a conspecific, social grooming (licking a conspecific or being licked), paying attention to conspecifics by observing them with interest, and interacting with human caretakers
Agnostic behaviour *	Aggression, dominance mount, threat display
Interspecific behaviour	Paying attention to another species’ presence
Not observed	
Out of sight	Focal animal is not visible from the point of observation/behaviour unknown

Informed by the previous literature on felid behaviour (specifically cheetahs) [37,49,50] and initial observations of the cheetahs being studied. * Includes actions performed or received by the focal animal.

**Table 4:** Root mean square standardised effects on behaviour, faecal consistency score (grade), and faecal glucocorticoid metabolite concentration (µg/g dry weight (DW)) for the study cheetahs (CH-2205, -2206, -2207, -2271, -2276, and -2277). Effect sizes were calculated using Cohen’s d.

Variable	Effect	Level	-Level	*t*	*p*	df ^1^	Cohen’s d	95% CI ^2^ for Cohen’s d	
								Lower	Upper
Behaviour									
Locomotion	Treatment Wk ^3^	Treatment (Wk ^3^ 3)	Control	2.99	0.004	101	*0.77*	0.25	1.29
Faecal consistency score (grade)	Study period	Treatment	Control	3.31	0.001	209	*0.65*	0.26	1.03
	Study period*feed/fast day	Treatment, fast day	Control, fast day	3.43	0.001	209	**1.23**	0.51	1.94
	Study period*feed/fast day	Treatment, feed day	Control, fast day	3.17	0.002	209	**1.07**	0.40	1.74
	Study period*feed/fast day	Control, feed day	Control, fast day	3.04	0.003	209	**1.01**	0.35	1.66
Faecal glucocorticoid metabolite concentration (µg/g DW)	Treatment Wk ^3^	Treatment (Wk ^3^ 2)	Control	2.76	0.006	170	*0.58*	0.16	1.00

Numbers in italics represent a medium magnitude of effect (*d* = 0.5), while bold numbers represent a large magnitude of effect (*d* = 0.8). ^1^ df: degrees of freedom; ^2^ CI: confidence interval; ^3^ Wk: week.

**Figure A1:**
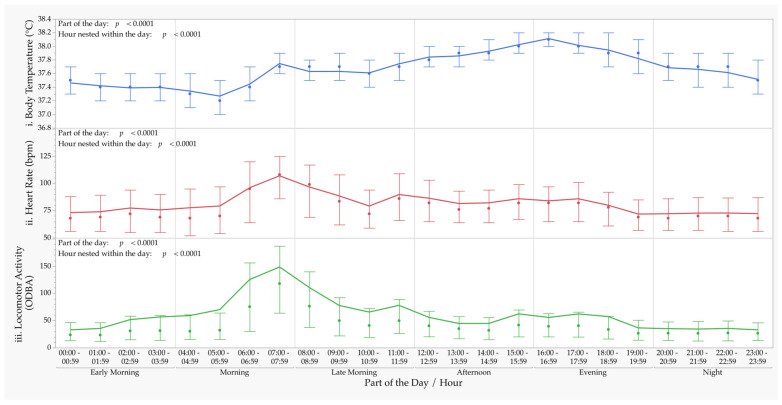
Circadian rhythm of (i) body temperature (°C), (ii) heart rate (beats per minute (bpm)), and (iii) locomotor activity (overall dynamic body acceleration (ODBA)) (median, interquartile range) for the study cheetahs (CH-2205, -2206, and -2276) throughout the study, i.e., in the treatment and control. Statistics were performed using a mixed model for repeated measures.

**Figure A2:**
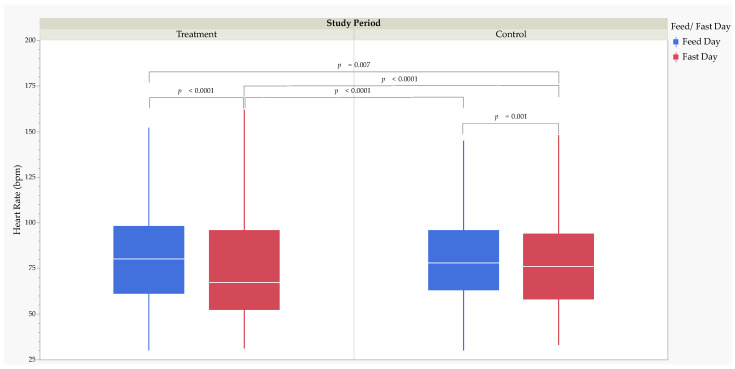
Box and whisker plot of heart rate (beats per minute (bpm)) for study cheetahs (CH-2205, -2206, and -2276). Effect of feed versus fast day by the study period. Statistics were performed using Tukey’s honestly significant difference post-hoc test.

**Figure A3:**
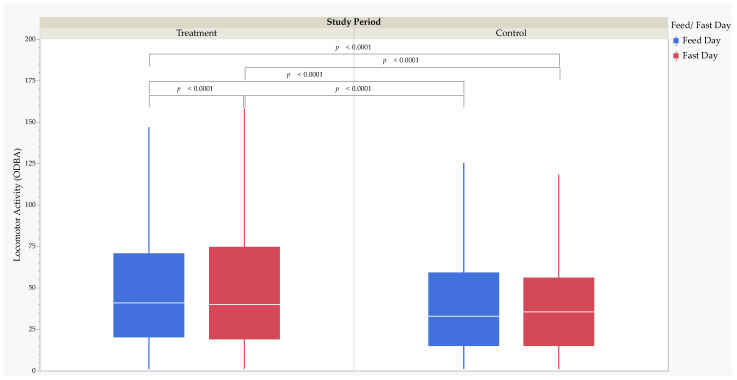
Box and whisker plot of locomotor activity (overall dynamic body acceleration (ODBA)) for study cheetahs (CH-2205, -2206, and -2276). Effect of feed versus fast day by the study period. Statistics were performed using Tukey’s honestly significant difference post-hoc test.

**Figure A4:**
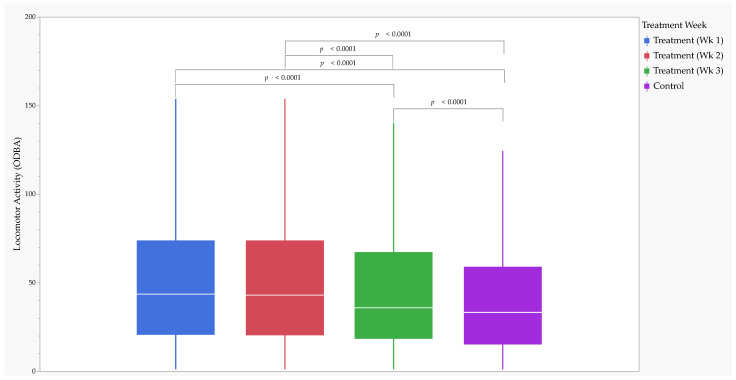
Box and whisker plot of locomotor activity (overall dynamic body acceleration (ODBA)) for the study cheetahs (CH-2205, -2206, and -2276). Effect of treatment week (Wk; one, two, and three). Statistics were performed using Tukey’s honestly significant difference post-hoc test.

## Data Availability

The data presented in this study are available in Supplementary Materials.

## Supplementary Materials

The following supporting information can be downloaded at: https://www.mdpi.com/article/10.3390/ani13172783/s1, Table S1. The proportion of scan samples in which each behaviour was observed for the study cheetahs (CH-2205, -2206, -2207, -2271, -2276, and -2277) during the treatment (week (Wk) one, two, and three) and control and on feed versus fast day; Table S2. The proportion of faecal samples collected of each faecal consistency score for the study cheetahs (CH-2205, -2206, -2207, -2271, -2276, and -2277) during the treatment (week (Wk) one, two, and three) and control and on feed versus fast day; Table S3. Biologger recordings for the study cheetahs during the treatment and control; Figure S1. Box and whisker plot of faecal glucocorticoid metabolite concentration (µg/g dry weight (DW)) for the study cheetahs (CH-2205, -2206, -2207, -2271, -2276, and -2277). Effect of feed versus fast day by the study period; Figure S2. Box and whisker plot of body temperature (°C) for the study cheetahs (CH-2205, -2206, and -2276). Effect of feed versus fast day by the study period; Figure S3. Box and whisker plot of body temperature (°C) for the study cheetahs (CH-2205, -2206, and -2276). Effect of treatment week (Wk; one, two, and three). Statistics were performed using Tukey’s honestly significant difference post-hoc test; Figure S4. Box and whisker plot of heart rate (beats per minute (bpm)) for the study cheetahs (CH-2205, -2206, and -2276). Effect of treatment week (Wk; one, two, and three). Statistics were performed using Tukey’s honestly significant difference post-hoc test; Dataset S1.

## Appendix A

### Appendix A.1. Materials and Methods

#### Appendix A.1.1. Body Temperature, Heart Rate, and Locomotor Activity Recordings

Each cheetah in the study had a single cardiac-, temperature-, and movement-sensitive biologging unit (DST centi-HRT ACT, Star-Oddi, Gardabaer, Iceland) implanted, measuring 46 mm × 15 mm × 15 mm, and weighing approximately 19 g. The biologgers were calibrated against a high-accuracy thermometer (1504 Tweener Thermometer Readout, Fluke, Everett, WA, USA) to within 0.1 °C. They were set to record triaxial LA, i.e., heave, surge, sway, for the overall dynamic body acceleration (ODBA) [108] every minute, 24 h a day throughout the study, i.e., in the treatment and control, and T_b_ (°C) and leadless single-channel electrocardiogram (ECG)-derived HR (beats per minute (bpm)) at 200 Hz every 5 min. Representative traces of raw ECG recordings were saved every 24 h for validating the HR measurements’ quality index (QI) (where QI_0_ was the highest quality and QI_1_, QI_2_, and QI_3_ were progressively reduced).

#### Appendix A.1.2. Surgical Procedures

Before surgical implantation, the biologgers were sterilised using ethylene oxide. Each study cheetah received a combination of 0.03 mg/kg of medetomidine (Medetomidine 20-mg/mL, Kyron Laboratories, Johannesburg, South Africa) and 0.8 mg/kg of tiletamine–zolazepam (Zoletil, Virbac Animal Health, Johannesburg, South Africa), administered intramuscularly (IM) by hand injection while in their enclosures. Once recumbent, the study cheetahs were placed in crates to which they were habituated and transported by vehicle within 500 m to an onsite clinic at the Cango Wildlife Ranch. There, they were intubated with an endotracheal tube of appropriate size and maintained under anaesthesia with 2–3% isoflurane (Forane, Abbott, Weltevreden Park, South Africa), administered in 100% oxygen. The depth of anaesthesia and standard anaesthetic parameters were monitored throughout the procedure. Intravenous lactated Ringer’s solution (B Braun, Johannesburg, South Africa) was administered to maintain intraoperative normovolemia.

At the commencement of surgical procedures, 5 mL of blood was collected from the jugular vein of each study cheetah. Blood samples were divided between lithium heparin and EDTA tubes. Following this, a comprehensive haematological evaluation (using an onsite HM5 analyser; Abaxis Veterinary Diagnostics, Union City, CA, USA) and plasma biochemistry profile (using an onsite VetScan VS2 analyser; Abaxis Veterinary Diagnostics, Union City, CA, USA) were conducted to determine the health status of the study cheetahs before the onset of the field research. Urine samples were collected via a 6 FG dog urinary catheter and evaluated using a standard multiparameter dipstick and refractometer (Jorgensen Laboratories, Loveland, CO, USA).

The study cheetahs were placed in a left lateral recumbent position, and a thoracic incision site was shaved and sterilised with chlorhexidine gluconate (Hibitane, Zeneca, Johannesburg, South Africa). A 50 mm cranial–caudal incision was made in the skin, and a single biologging unit was implanted IM between the deep and superficial pectoralis major muscles without tethering. Wounds were closed with continuous subcutaneous and intradermal sutures (5/0 Monosyn, B-Braun, Barcelona, Spain) and treated with a topical antiseptic and ectoparasiticide spray (F10 Germicidal Wound Spray, Health and Hygiene Pty Ltd., Roodepoort, South Africa).

The study cheetahs were returned to the crates, where the effects of the anaesthesia were reversed with 0.1 mg/kg atipamezole (Antisedan, Zoetis, Johannesburg, South Africa) IM. Once recovered from the anaesthetic, they were transported back to their enclosures. The caretakers conducted daily observations to monitor for signs of surgical site infection or other postoperative complications.

Following the completion of the experimental trials, the same anaesthesia surgical protocols were used to remove the biologgers.

#### Appendix A.1.3. Data Preparation

Battery malfunction of the biologgers implanted in four of the six study cheetahs (CH-2207, -2271, -2276, and -2277) prevented those units from recording partial T_b_, HR, and LA data for CH-2276 during the control, and whole datasets for CH-2207, CH-2271, and CH-2277 (Table S3).

The initial examination of the raw HR data for the study cheetahs with functional biologgers (CH-2205 and -2206), and partial data for CH-2276, revealed values ranging from 0 to 1005 bpm, the extremes of which were likely due to incomplete, low-quality readings or implant movement within the pectoral muscle when the study cheetahs were active. To remove erroneous measurements, ensuring only plausible values were included in the analyses, upper and lower thresholds were created. At the low end of the range, all readings of 0 bpm were removed, establishing a new minimum of 30 bpm. For the upper end, where most unlikely measurements occurred, EGC data available in the literature on the cheetah [109,110] informed the HR values to be filtered. The values to which QI_3_ was assigned (indicating the lowest quality) were also removed. Once filtered, all HR recordings (*n* = 30,225/33,443, which represented over 90% of the raw data initially captured) fell within the 30 and 200 bpm threshold set. As T_b_ and LA recordings were not sensitive to implant movement within the pectoral muscle, they were not filtered, in addition to the lack of extreme T_b_ values.

To investigate the circadian patterns of T_b_, HR, and LA, data were assigned to six part-of-the-day categories based on the hour they were recorded during the day, namely (i) early morning (00.00–04.00 h), (ii) morning (04.00–08.00 h), (iii) late morning (08.00–12.00 h), (iv) afternoon (12.00–16.00 h), (v) evening (16.00–20.00 h), and (vi) night (20.00–00.00 h).

#### Appendix A.1.4. Statistical Analysis

Outlying values were detected for LA (*n* = 40) and subsequently excluded from descriptive statistics and analyses. Body temperature, HR, and LA data were Box–Cox transformed to satisfy the assumption of normality and homogeneity due to their departure. The data were back-transformed for descriptive statistics and visual representation to maintain statistical integrity. In this study, an MMRM analysis was used to investigate the independent fixed effects of part of the day and the hour within part of the day on (i) T_b_, (ii) HR, and (iii) LA. An MMRM analysis investigated the independent fixed effects of the study period and feed versus fast day on (i) T_b_, (ii) HR, and (iii) LA and their interaction to test the moderator effect of the study period on feed versus fast day. An MMRM analysis investigated the independent fixed effect of treatment week (one, two, and three) on (i) T_b_, (ii) HR, and (iii) LA. An MMRM analysis investigated the independent fixed effects of when the study cheetahs were feeding versus when they were not (i.e., feeding time (yes versus no)) and part of the day during which they were offered meals (late morning, afternoon, and evening) on (i) T_b_, (ii) HR, and (iii) LA and their interaction to test the moderator effect of part of the day on feeding time. The study cheetah was included as the random effect in the analyses.

Multiple pairwise comparisons were explored using Tukey’s HSD post-hoc tests. Effect sizes of pairwise comparisons were calculated using Cohen’s d (Equation (1)).

### Appendix A.2. Results

The MMRM analysis revealed that the circadian rhythm of T_b_ fluctuated significantly during the 24 h day (for part of the day: *F*_5,33417_ = 2886.93, *p* < 0.0001 and for the hour nested within part of the day: *F*_18,33417_ = 171.90, *p* < 0.0001) (Figure A1 (i)). Throughout this study, the overall median T_b_ was higher during the evening (38.0 (0.4) °C) between 16.00–16.59 h (38.1 (0.2) °C) than the other parts of the day and hours.

The MMRM analysis revealed that the fixed effects of the study period and feed versus fast day on T_b_ failed to achieve statistical significance; therefore, post-hoc testing was not performed (Figure S2).

The MMRM analysis revealed that the fixed effect of treatment week on T_b_ was significant (*F*_3,33418_ = 18.34, *p* < 0.0001). Post-hoc comparisons using Tukey’s HSD test revealed that T_b_ was significantly higher during week three of the treatment (37.7 (0.6) °C) than in weeks one (37.7 (0.5) °C; *t*_33418_ = −4.37, *p* < 0.0001) and two (37.7 (0.5) °C; *t*_33418_ = −6.94, *p* < 0.0001) of the treatment and the control (37.7 (0.5) °C; *t*_33418_ = 6.24, *p* < 0.0001) (Figure S3).

The MMRM analysis revealed that T_b_ was significantly higher (*F*_1,16698_ = 30.41, *p* < 0.0001) when the study cheetahs were feeding (37.7 (0.5) °C) than when they were not (37.9 [0.3] °C). Post-hoc comparisons using Tukey’s HSD test revealed that T_b_ was significantly higher at feeding time during the afternoon (yes: 38.0 (0.2) °C versus no: 37.9 (0.3) °C; *t*_16697_ = 3.26, *p* = 0.014) and the evening (yes: 38.2 (0.3) °C versus no: 38.0 (0.4) °C; *t*_16697_ = 4.53, *p* < 0.0001).

Effect size calculations using Cohen’s d revealed a small RMSSE on T_b_ between week three of the treatment and weeks one (*d* = 0.13; *t*_33439_ = 6.93, *p* < 0.0001) and two (*d* = 0.08; *t*_33439_ = 4.37, *p* < 0.0001) of the treatment and the control (*d* = 0.10; *t*_33439_ = 6.60, *p* < 0.0001) (Table A1). Effect size calculations using Cohen’s d revealed a small RMSSE (*d* = 0.41; *t*_16698_ = 9.46, *p* < 0.0001) of feeding time on T_b_. The RMSSE of feeding time on T_b_ by part of the day was medium during the afternoon (*d* = 0.54; *t*_16698_ = 6.22, *p* < 0.0001) and the evening (*d* = 0.65; *t*_16698_ = 7.24, *p* < 0.0001).

**Table A1:** Root mean square standardised effects on body temperature (°C), heart rate (beats per minute (bpm)), and locomotor activity (overall dynamic body acceleration (ODBA)) for the study cheetahs (CH-2205, -2206, and -2276). Effect sizes were calculated using Cohen’s d.

Variable	Effect	Level	-Level	*t*	*p*	df ^1^	Cohen’s d	95% CI ^2^ for Cohen’s d	
								Lower	Upper
Body temperature (°C)	Feeding time	Yes	No	9.46	<0.0001	16698	0.41	0.32	0.49
	Feeding time*part of the day	Yes, evening	No, evening	7.24	<0.0001	16698	*0.65*	0.48	0.83
	Feeding time*part of the day	Yes, afternoon	No, afternoon	6.22	<0.0001	16698	*0.54*	0.37	0.71
	Treatment Wk ^3^	Treatment (Wk ^3^ 3)	Treatment (Wk ^3^ 2)	6.93	<0.0001	33439	0.13	0.09	0.16
	Treatment Wk ^3^	Treatment (Wk ^3^ 3)	Control	6.60	<0.0001	33439	0.10	0.07	0.13
	Treatment Wk ^3^	Treatment (Wk ^3^ 3)	Treatment (Wk ^3^ 1)	4.37	<0.0001	33439	0.08	0.04	0.12
Heart rate (bpm)	Study period	Control	Treatment	7.11	<0.0001	30221	0.11	0.08	0.14
	Feed/fast day	Feed day	Fast day	13.26	<0.0001	30221	0.20	0.17	0.23
	Study period*feed/fast day	Control, feed day	Treatment, fast day	18.97	<0.0001	30221	0.31	0.27	0.34
	Study period*feed/fast day	Treatment, feed day	Treatment, fast day	18.05	<0.0001	30221	0.30	0.26	0.33
	Study period*feed/fast day	Control, fast day	Treatment, fast day	7.59	<0.0001	30221	0.20	0.15	0.26
	Study period*feed/fast day	Control, feed day	Control, fast day	4.03	<0.0001	30221	0.10	0.05	0.15
	Study period*feed/fast day	Treatment, feed day	Control, fast day	3.64	0.000	30221	0.09	0.04	0.14
	Treatment Wk ^3^	Control	Treatment (Wk ^3^ 2)	10.33	<0.0001	30221	0.17	0.13	0.20
	Treatment Wk ^3^	Treatment (Wk ^3^ 1)	Treatment (Wk ^3^ 2)	6.25	<0.0001	30221	0.12	0.08	0.16
	Treatment Wk ^3^	Treatment (Wk ^3^ 3)	Treatment (Wk ^3^ 2)	4.34	<0.0001	30221	0.08	0.05	0.12
	Treatment Wk ^3^	Control	Treatment (Wk ^3^ 3)	5.12	<0.0001	30221	0.08	0.05	0.11
	Treatment Wk ^3^	Control	Treatment (Wk ^3^ 1)	2.85	0.004	30221	0.05	0.01	0.08
	Feeding time	Yes	No	1.97	0.048	14834	0.09	0.00	0.18
	Feeding time*part of the day	No, late morning	Yes, late morning	3.69	0.000	14834	0.14	0.07	0.21
Locomotor activity (ODBA)	Study period	Treatment	Control	15.48	<0.0001	33399	0.22	0.19	0.25
	Feed/fast day	Feed day	Fast day	0.38	0.707	33399	0.01	−0.02	0.03
	Study period*feed/fast day	Treatment, fast day	Control, fast day	10.13	<0.0001	33399	0.26	0.21	0.31
	Study period*feed/fast day	Treatment, feed day	Control, fast day	9.39	<0.0001	33399	0.23	0.18	0.27
	Study period*feed/fast day	Treatment, fast day	Control, feed day	14.11	<0.0001	33399	0.22	0.19	0.25
	Study period*feed/fast day	Treatment, feed day	Control, feed day	14.44	<0.0001	33399	0.18	0.16	0.21
	Study period*feed/fast day	Treatment, fast day	Treatment, feed day	2.11	0.035	33399	0.03	0.00	0.06
	Treatment Wk ^3^	Treatment (Wk ^3^ 1)	Control	16.66	<0.0001	33399	0.25	0.22	0.28
	Treatment Wk ^3^	Treatment (Wk ^3^ 2)	Control	14.14	<0.0001	33399	0.21	0.19	0.24
	Treatment Wk ^3^	Treatment (Wk ^3^ 3)	Control	8.68	<0.0001	33399	0.13	0.10	0.16
	Treatment Wk ^3^	Treatment (Wk ^3^ 1)	Treatment (Wk ^3^ 3)	6.66	<0.0001	33399	0.12	0.09	0.16
	Treatment Wk ^3^	Treatment (Wk ^3^ 2)	Treatment (Wk ^3^ 3)	4.56	<0.0001	33399	0.08	0.05	0.12
	Feeding time	Yes	No	7.74	<0.0001	16686	0.33	0.25	0.42

Numbers in italics represent a medium magnitude of effect (*d* = 0.5). ^1^ df: degrees of freedom; ^2^ CI: confidence interval; ^3^ Wk: week.

The MMRM analysis revealed that the circadian rhythm of HR fluctuated significantly during the 24 h day (for part of the day: *F*_5,30199_ = 300.96, *p* < 0.0001 and for the hour nested within part of the day: *F*_18,30199_ = 81.77, *p* < 0.0001) (Figure A1 (ii)). Throughout the study, the overall median HR was higher during the morning (85 (55) bpm) between 07.00–07.59 h (108 (39) bpm) than the other parts of the day and hours.

The MMRM analysis revealed that HR was significantly lower (*F*_1,30210_ = −56.85, *p* < 0.0001) during the treatment (76 (39) bpm) than the control (78 (34) bpm). The MMRM analysis revealed that HR was significantly higher (*F*_1,30221_ = 170.83, *p* < 0.0001) on feeding days (79 (35) bpm) than on fasting days (69 (41) bpm). Post-hoc comparisons using Tukey’s HSD test revealed that HR was significantly higher on treatment feeding days (80 (37) bpm) than on treatment fasting days (67 (44) bpm; *t*_30219_ = 17.94, *p* < 0.0001) and control fasting days (76 (36) bpm; *t*_30219_ = 3.24, *p* = 0.007) and significantly higher on control feeding days (78 (33) bpm) than on treatment fasting days (*t*_30219_ = 19.26, *p* < 0.0001) and control fasting days (*t*_30219_ = 3.89, *p* = 0.001) (Figure A2).

The MMRM analysis revealed that the fixed effect of treatment week on HR was significant (*F*_3,30218_ = 41.46, *p* < 0.0001). Post-hoc comparisons using Tukey’s HSD test revealed that HR was significantly lower during weeks one (79 (39) bpm; *t*_30218_ = −3.37, *p* = 0.004), two (73 (40) bpm; *t*_30218_ = −10.85, *p* < 0.0001), and three (74 (38) bpm; *t*_30218_ = −5.57, *p* < 0.0001) of the treatment than the control (78 (34) bpm) (Figure S4).

The MMRM analysis revealed that HR was significantly higher (*F*_1,14832_ = 6.56, *p* = 0.010) when the study cheetahs were feeding (81 (39) bpm) than when they were not (80 (35) bpm). Post-hoc comparisons using Tukey’s HSD test revealed that HR was significantly lower (*t*_14833_ = −3.69, *p* = 0.003) at feeding time during the late morning (yes: 80 (44) bpm versus no: 84 (46) bpm).

Effect size calculations using Cohen’s d revealed a small RMSSE of the study period (*d* = 0.11; *t*_30221_ = 7.11, *p* < 0.0001) and feed versus fast day (*d* = 0.20; *t*_30221_ = 13.26, *p* < 0.0001) on HR (Table A1). The RMSSE of feed versus fast day on HR by the study period was small between treatment feeding days and treatment fasting days (*d* = 0.30; *t*_30221_ = 18.05, *p* < 0.0001) and control fasting days (*d* = 0.09; *t*_30221_ = 3.64, *p* = 0.000) and between control feeding days and treatment fasting days (*d* = 0.31; *t*_30221_ = 18.97, *p* < 0.0001) and control fasting days (*d* = 0.10; *t*_30221_ = 4.03, *p* < 0.0001). Effect size calculations using Cohen’s d revealed a small RMSSE on HR between weeks one (*d* = 0.05; *t*_30221_ = 2.85, *p* = 0.004), two (*d* = 0.17; *t*_30221_ = 10.33, *p* < 0.0001), and three (*d* = 0.08; *t*_30221_ = 5.12, *p* < 0.0001) of the treatment and the control (Table A1). Effect size calculations using Cohen’s d revealed a small RMSSE of feeding time (*d* = 0.09; *t*_14834_ = 1.97, *p* = 0.048) on HR. The RMSSE of feeding time on HR by part of the day was small during the late morning (*d* = 0.14; *t*_14834_ = 3.69, *p* = 0.000).

The MMRM analysis revealed that the circadian rhythm of LA fluctuated significantly during the 24 h day (for part of the day: *F*_5,33377_ = 723.70, *p* < 0.0001 and for the hour nested within part of the day: *F*_18,33377_ = 138.37, *p* < 0.0001) (Figure A1 (iii)). Throughout the study, the overall median LA was higher during the morning (55.0 (106.4) ODBA) between 07.00–07.59 h (117.0 (121.6) ODBA) than the other parts of the day and hours.

The MMRM analysis revealed that LA was significantly higher (*F*_1,33397_ = 115.50, *p* < 0.0001) during the treatment (40.5 (52.0) ODBA) than the control (33.2 (43.6) ODBA). The MMRM analysis revealed that LA was significantly higher (*F*_1,33397_ = 10.79, *p* = 0.001) on fasting days (38.6 (49.6) ODBA) than on feeding days (36.6 (47.6) ODBA). Post-hoc comparisons using Tukey’s HSD test revealed that LA was significantly higher on treatment fasting days (39.8 (55.3) ODBA) than on treatment feeding days (40.8 (50.6) ODBA; *t*_33397_ = 5.99, *p* < 0.0001) and control feeding days (33.0 (44.2) ODBA; *t*_33397_ = 13.18, *p* < 0.0001) (Figure A3).

The MMRM analysis revealed that the fixed effect of treatment week on LA was significant (*F*_3,33397_ = 72.22, *p* < 0.0001). Post-hoc comparisons using Tukey’s HSD test revealed that LA was significantly higher during week one of the treatment (43.4 (53.2) ODBA) than in week three of the treatment (35.8 (48.8) ODBA; *t*_33397_ = 7.22, *p* < 0.0001) and the control (33.2 (43.8) ODBA; *t*_33397_ = 13.02, *p* < 0.0001), significantly higher during week two of the treatment (42.8 (53.4) ODBA) than in week three of the treatment (*t*_33397_ = 4.94, *p* < 0.0001) and the control (*t*_33397_ = 10.30, *p* < 0.0001), and significantly higher during week three of the treatment than the control (*t*_33397_ = 4.41, *p* < 0.0001) (Figure A4).

The MMRM analysis revealed that LA was significantly higher (*F*_1,16685_ = 9.38, *p* = 0.002) when the study cheetahs were feeding (52.6 (70.4) ODBA) than when they were not (39.8 (49.0) ODBA). Post-hoc comparisons using Tukey’s HSD test revealed that the interaction effect of feeding time on LA by part of the day failed to achieve statistical significance during the late morning, the afternoon, and the evening.

Effect size calculations using Cohen’s d revealed a small RMSSE of the study period (*d* = 0.22; *t*_33399_ = 15.48, *p* < 0.0001) and feed versus fast day (*d* = 0.01; *t*_33399_ = 0.38, *p* = 0.707) on LA (Table A1). The RMSSE of feed versus fast day on LA by the study period was small between treatment fasting days and treatment feeding days (*d* = 0.03; *t*_33399_ = 2.11, *p* = 0.035) and control feeding days (*d* = 0.22; *t*_33399_ = 14.11, *p* < 0.0001). Effect size calculations using Cohen’s d revealed a small RMSSE between week one of the treatment and week three of the treatment (*d* = 0.12; *t*_33399_ = 6.66, *p* < 0.0001) and the control (*d* = 0.25; *t*_33399_ = 16.66, *p* < 0.0001), a small RMSSE between week two of the treatment and week three of the treatment (*d* = 0.08; *t*_33399_ = 4.56, *p* < 0.0001) and the control (*d* = 0.21; *t*_33399_ = 14.14, *p* < 0.0001), and a small RMSSE between week three of the treatment and the control (*d* = 0.21; *t*_33399_ = 14.14, *p* < 0.0001). Effect size calculations using Cohen’s d revealed a small RMSSE of feeding time (*d* = 0.13; *t*_16686_ = 8.68, *p* < 0.0001) on LA.

### Appendix A.3. Discussion

This appendix contains the study’s secondary aim, exploring the use of biologging technology to record T_b_, HR, and LA simultaneously.

Consistent with the reduced feeding frequency schedule as a potential food-based enrichment was higher LA. It is also possible to interpret the observed increase in activity among the studied cheetahs as behavioural agitation. Cheetahs and other carnivores in the wild spend a significant proportion of their activity budget resting [111]. While reducing the risk of obesity, the motivation for increased activity levels, possibly hunger, could have negative welfare implications. A lack of satiety is further supported by higher LA on treatment fasting days (in particular). These results highlight the importance of offering sufficiently large meals if additional fasting days are introduced so as not to be contrary to animals’ wellbeing. By week three of the treatment, LA demonstrated temporal adaptation to the change in feeding frequency.

Environmental enrichment blunts E release [112] and, as a result, reduces stress-related increases in HR [113,114,115,116,117]. When the studied cheetahs were fed less frequently, they had a lower HR, indicating stress reduction [118] to a greater degree as it cooccurred with increased activity. During physical activity, requirements for oxygen and gluconeogenetic substrates in skeletal muscle are increased, as are the removal of metabolites and carbon dioxide [119]. The cardiac output is increased to meet the demand for blood flow by contracting muscles, attributed to sympathetically mediated increases in HR and stroke volume. Increased T_b_ follows an increase in HR due to heat generated during nutrient conversion to muscular work.

Physiologically, enrichment-induced arousal was attended by higher HR (a salient feature of the SNS-mediated reaction to stress) on days when the studied cheetahs were fed. Additionally, higher T_b_ and HR were observed when they were feeding than when they were not. Stress-induced hyperthermia has been shown to some extent by a study in which the increase in T_b_ of free-ranging cheetahs following a successful hunt was double that of an unsuccessful chase (mean ± standard deviation (SD); 1.38 ± 0.28 °C vs. 0.58 ± 0.18 °C), despite comparable levels of physical activity [120,121]. The authors attributed the hyperthermia experienced by hunting cheetahs to the stress associated with vulnerability to attack and kleptoparasitism by more dominant intraguild predators [120,121]. In this study, the cheetahs were fed in a space free of interference competition; therefore, the thermal and cardiac response related to feeding suggests psychological excitation to food consumption rather than a predator-induced stress response. Alternatively, as higher T_b_ and HR at feeding time cooccurred with higher LA, these results could be interpreted as the demands of increased physical activity.

Recording simultaneous T_b_, HR, and LA using biologging technology has tremendous potential to measure stress in the cheetah; however, a significant limitation of this study was the battery malfunction of the biologgers implanted in four of the six cheetahs, preventing those units from recording partial T_b_, HR, and LA data or whole datasets. As with any emerging technological advance, there is a need for further development, including more reliable and affordable devices, novel attachment or implantation and retrieval methods, and remote data transmission, before the routine application of biologgers in stress and animal welfare studies.

It must also be acknowledged that the treatment effects on the measured variables, though at times statistically significant, were likely irrelevant to the biology of the cheetahs studied. The magnitude of the differences in numeric values for T_b_, HR, and LA may not have been sufficient for biological significance. For example, Hetem et al. [120,121] reported a significant difference in T_b_ increase between successful and unsuccessful hunts (mean ± SD; 1.38 ± 0.28 °C vs. 0.58 ± 0.18 °C; *p* < 0.0001), while the difference in T_b_ between when the study cheetahs were feeding and when they were not (median (IQR); 37.7 (0.5) °C vs. 37.9 (0.3) °C; *p* < 0.0001) significant here was comparatively less. This study’s reliance on only six study subjects, made worse by the battery malfunction of four of the six biologgers, is likely to have reduced our power to detect a biologically relevant effect. Cheetahs and other large carnivores are typically kept in low population densities at facilities like the Cango Wildlife Ranch. Future research should increase sample size through multi-institutional studies to improve the findings’ quality, as Swaisgood and Shepherdson previously recommended [77].
