# Supplementary material for: Responses to Reduced Feeding Frequency in Captive-Born Cheetahs (Acinonyx jubatus): Implications for Behavioural and Physiological Stress and Gastrointestinal Health
Source: Animals (Basel). 2023 Aug 31;13(17):2783. doi: 10.3390/ani13172783 (PMC10486355; doi:10.3390/ani13172783)
Supplement: Supplementary file 1 [file animals-13-02783-s001.zip › animals-2565889-supplementary.pdf]

Table S1

Table S1. The proportion of scan samples in which each behaviour was observed for the study cheetahs (CH-2205, -2206, -2207, -2271, -2276, and -2277) during the treatment (week [Wk] one, two, and three) and control and on feed versus fast day.

**Behaviour**	**Study Period**					**Feed/Fast Day**	
	**Treatment (%)**	**Treatment (Wk 1) (%)**	**Treatment (Wk 2) (%)**	**Treatment (Wk 3) (%)**	**Control (%)**	**Feeding Days (%)**	**Fasting Days (%)**
Inactive	40.37	37.02	40.61	42.13	43.72	40.11	51.30
Appetitive behaviour	10.74	8.17	14.33	9.27	13.45	12.70	9.42
Attention	7.82	12.50	6.14	6.46	6.84	7.70	5.52
Auto-grooming	2.33	0.48	2.73	3.09	2.47	2.22	3.25
Locomotion	21.35	18.27	18.09	25.84	14.80	18.32	16.56
Maintenance	0.35	0.00	0.34	0.56	0.22	0.21	0.65
Olfactory exploration	1.63	1.92	0.68	2.25	1.23	1.46	1.30
Scent-marking	3.73	6.25	3.07	2.81	2.91	3.33	3.25
Standing	0.35	0.48	0.34	0.28	0.22	0.35	0.00
Stereotypical	0.23	0.96	0.00	0.00	0.11	0.21	0.00
Vocalisation	0.47	0.00	1.02	0.28	0.22	0.14	1.30
Affiliative behaviour	1.40	1.44	2.39	0.56	2.13	1.80	1.62
Agnostic behaviour	0.23	0.00	0.68	0.00	0.45	0.28	0.65
Interspecific behaviour	8.98	12.50	9.56	6.46	7.51	8.88	5.19
Out of sight	0.00	0.00	0.00	0.00	3.70	2.29	0.00

Table S2

Table S2. The proportion of faecal samples collected of each faecal consistency score for the study cheetahs (CH-2205, -2206, -2207, -2271, -2276, and -2277) during the treatment (week [Wk] one, two, and three) and control and on feed versus fast day.

Faecal Consistency Score	Study Period					Feed/Fast Day	
	Treatment (%)	Treatment (Wk 1) (%)	Treatment (Wk 2) (%)	Treatment (Wk 3) (%)	Control (%)	Feeding Days (%)	Fasting Days (%)
Firm	29.52	30.30	18.92	40.00	24.07	25.00	33.33
Firm and dry	20.00	18.18	29.73	11.43	17.59	20.83	11.11
Soft with shape	45.71	45.45	45.95	45.71	48.15	48.81	40.00
Soft without shape	4.76	6.06	5.41	2.86	10.19	5.36	15.56
Liquid	0.00	0.00	0.00	0.00	0.00	0.00	0.00

Table S3

Table S3. Biologger recordings for the study cheetahs during the treatment and control.

	**Treatment**				**Control**			
	**T_b [1]**	**HR [2]**	**LA [3]**	**Filtered HR [2]**	**T_b [1]**	**HR [2]**	**LA [3]**	**Filtered HR [2]**
CH-2205	6048	6048	30240	5075	6048	6048	30240	5222
CH-2206	6048	6048	30240	5744	6048	6048	30240	5705
CH-2207	0	0	0	0	0	0	0	0
CH-2271	0	0	0	0	0	0	0	0
CH-2276	6048	6048	30240	5525	3203	3203	15967	2954
CH-2277	0	0	0	0	0	0	0	0

[1] T_b, body temperature; [2] HR, heart rate; [3] LA, locomotor activity.

Figure S1

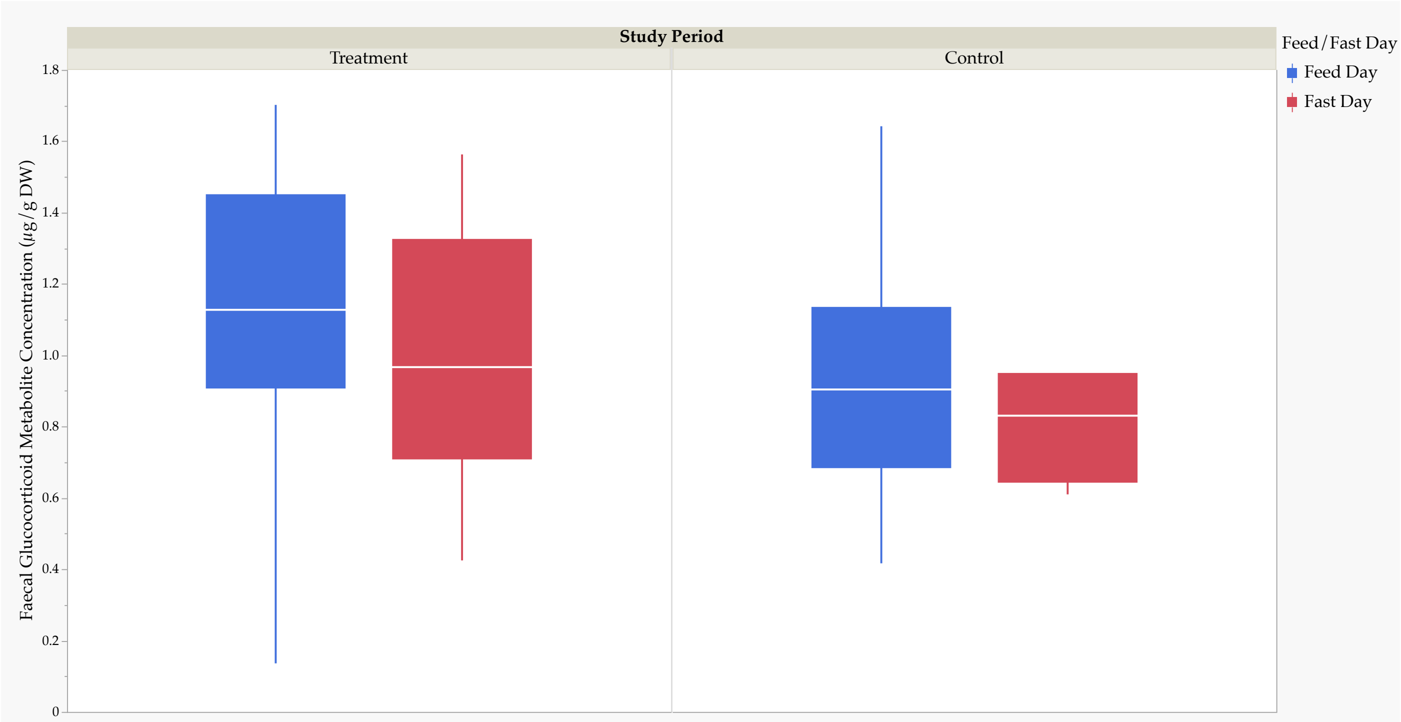

Figure S1. Box and whisker plot of faecal glucocorticoid metabolite concentration (μg/g dry weight [DW]) for the study cheetahs (CH-2205, -2206, -2207, -2271, -2276, and -2277). Effect of feed versus fast day by the study period.

Figure S2

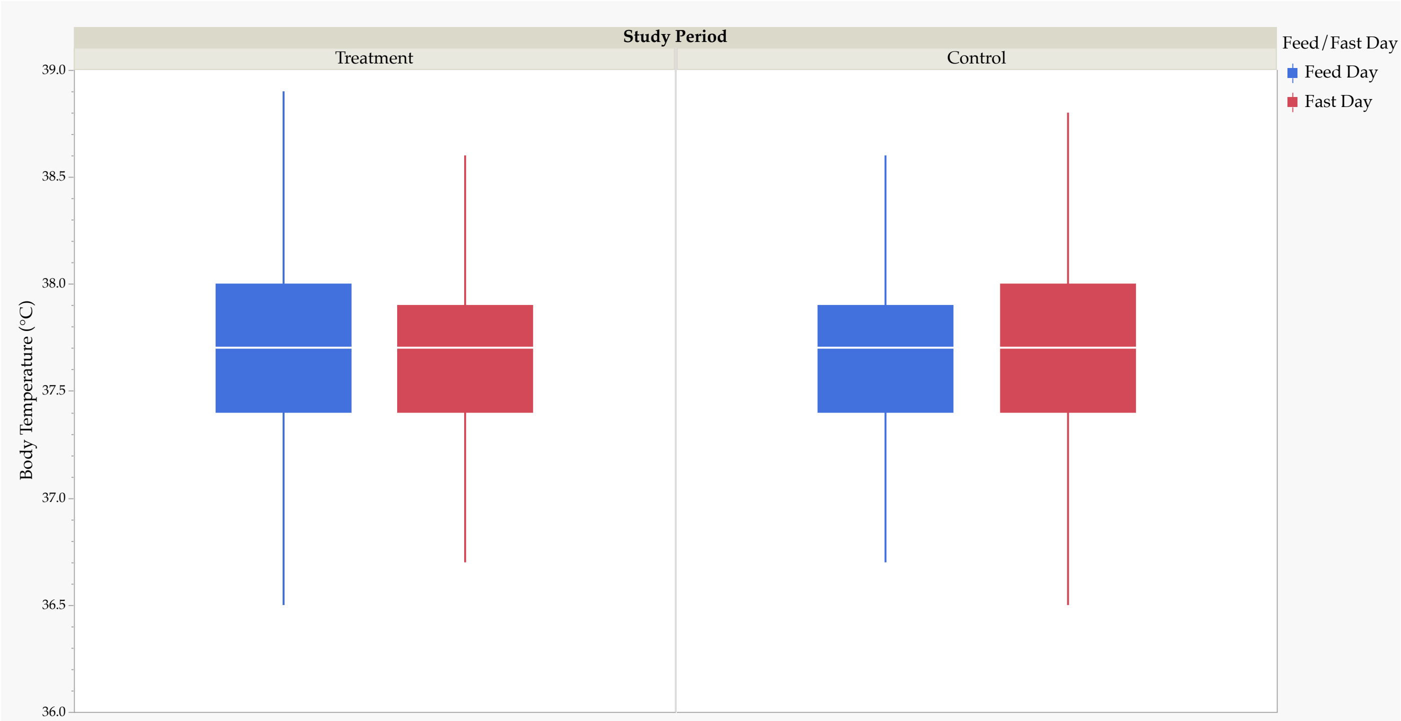

Figure S2. Box and whisker plot of body temperature (°C) for the study cheetahs (CH-2205, -2206, and -2276). Effect of feed versus fast day by the study period.

Figure S3

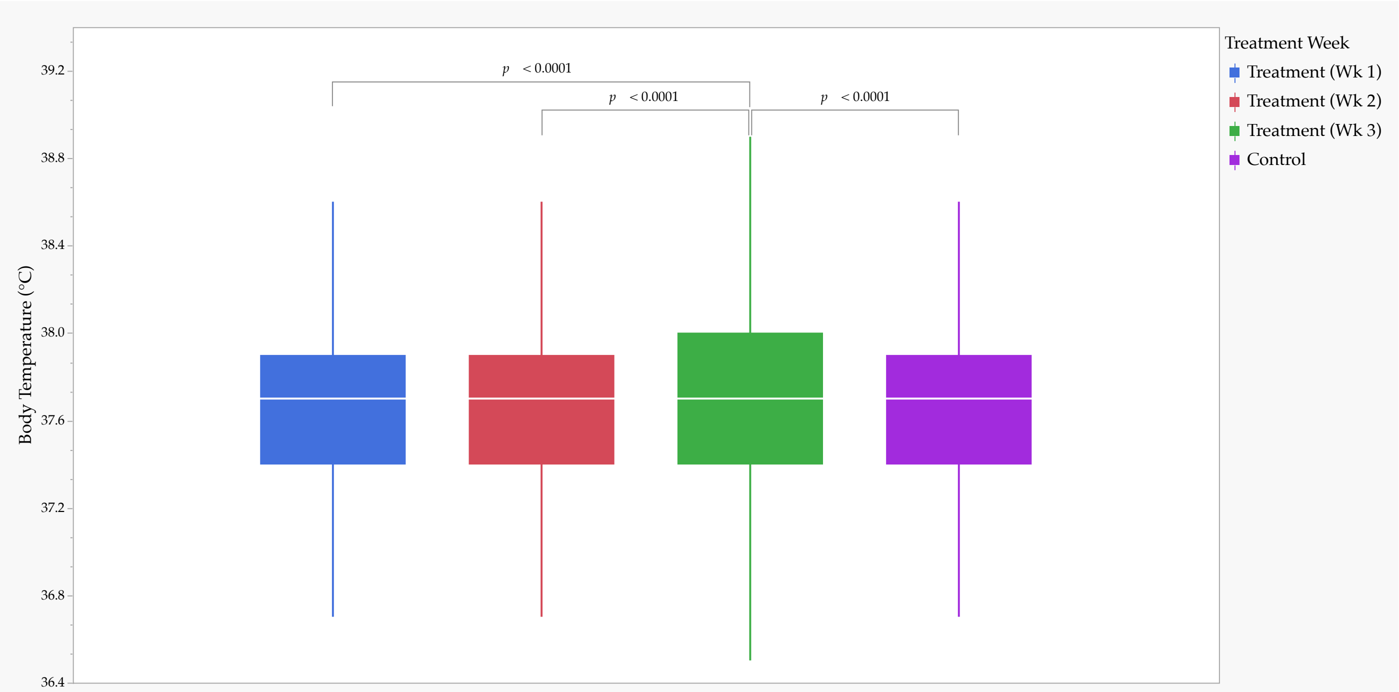

Figure S3. Box and whisker plot of body temperature (°C) for the study cheetahs (CH-2205, -2206, and -2276). Effect of treatment week (Wk; one, two, and three). Statistics were performed using Tukey's honestly significant difference post hoc test.

Figure S4

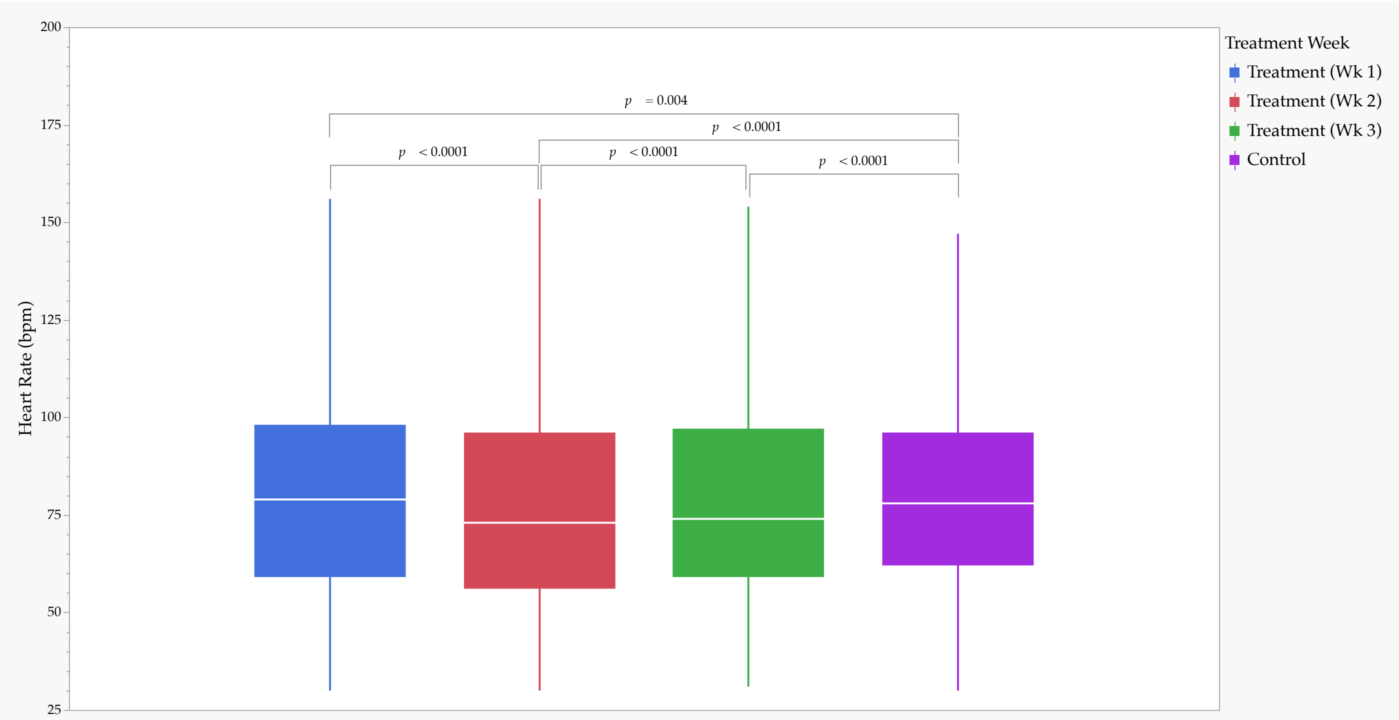

Figure S4. Box and whisker plot of heart rate (beats per minute [bpm]) for the study cheetahs (CH-2205, -2206, and -2276). Effect of treatment week (Wk; one, two, and three). Statistics were performed using Tukey's honestly significant difference post hoc test.

Dataset S1: BEHAVIOUR_1

Date	Cheetah ID	Sex	Age Group	Week	Study Period	Feed/Fast Day	Minute	Hour	Part of the Day	Behaviour
2019/04/24	CH2207	Female	Three y/o	Treatment (Wk 1)	Treatment	Fast Day	07:45	07:00 - 07:59	Morning	Locomotion
2019/04/24	CH2207	Female	Three y/o	Treatment (Wk 1)	Treatment	Fast Day	07:50	07:00 - 07:59	Morning	Auto-grooming
2019/04/24	CH2207	Female	Three y/o	Treatment (Wk 1)	Treatment	Fast Day	07:55	07:00 - 07:59	Morning	Locomotion
2019/04/24	CH2207	Female	Three y/o	Treatment (Wk 1)	Treatment	Fast Day	08:00	08:00 - 08:59	Late Morning	Scent-marking
2019/04/24	CH2207	Female	Three y/o	Treatment (Wk 1)	Treatment	Fast Day	08:05	08:00 - 08:59	Late Morning	Locomotion
2019/04/24	CH2207	Female	Three y/o	Treatment (Wk 1)	Treatment	Fast Day	08:10	08:00 - 08:59	Late Morning	Appetitive behaviour
2019/04/24	CH2207	Female	Three y/o	Treatment (Wk 1)	Treatment	Fast Day	08:15	08:00 - 08:59	Late Morning	Appetitive behaviour
2019/04/24	CH2207	Female	Three y/o	Treatment (Wk 1)	Treatment	Fast Day	08:20	08:00 - 08:59	Late Morning	Scent-marking
2019/04/24	CH2207	Female	Three y/o	Treatment (Wk 1)	Treatment	Fast Day	08:25	08:00 - 08:59	Late Morning	Appetitive behaviour
2019/04/24	CH2207	Female	Three y/o	Treatment (Wk 1)	Treatment	Fast Day	08:30	08:00 - 08:59	Late Morning	Appetitive behaviour
2019/04/24	CH2207	Female	Three y/o	Treatment (Wk 1)	Treatment	Fast Day	08:35	08:00 - 08:59	Late Morning	Scent-marking
2019/04/24	CH2207	Female	Three y/o	Treatment (Wk 1)	Treatment	Fast Day	08:40	08:00 - 08:59	Late Morning	Scent-marking
2019/04/30	CH2207	Female	Three y/o	Treatment (Wk 2)	Treatment	Feed Day	08:10	08:00 - 08:59	Late Morning	Inactive
2019/04/30	CH2207	Female	Three y/o	Treatment (Wk 2)	Treatment	Feed Day	08:15	08:00 - 08:59	Late Morning	Inactive
2019/04/30	CH2207	Female	Three y/o	Treatment (Wk 2)	Treatment	Feed Day	08:20	08:00 - 08:59	Late Morning	Appetitive behaviour
2019/04/30	CH2207	Female	Three y/o	Treatment (Wk 2)	Treatment	Feed Day	08:25	08:00 - 08:59	Late Morning	Locomotion
2019/04/30	CH2207	Female	Three y/o	Treatment (Wk 2)	Treatment	Feed Day	08:30	08:00 - 08:59	Late Morning	Appetitive behaviour
2019/04/30	CH2207	Female	Three y/o	Treatment (Wk 2)	Treatment	Feed Day	08:35	08:00 - 08:59	Late Morning	Appetitive behaviour
2019/04/30	CH2207	Female	Three y/o	Treatment (Wk 2)	Treatment	Feed Day	08:40	08:00 - 08:59	Late Morning	Appetitive behaviour
2019/04/30	CH2207	Female	Three y/o	Treatment (Wk 2)	Treatment	Feed Day	08:45	08:00 - 08:59	Late Morning	Appetitive behaviour
2019/04/30	CH2207	Female	Three y/o	Treatment (Wk 2)	Treatment	Feed Day	08:50	08:00 - 08:59	Late Morning	Appetitive behaviour
2019/04/30	CH2207	Female	Three y/o	Treatment (Wk 2)	Treatment	Feed Day	08:55	08:00 - 08:59	Late Morning	Locomotion
2019/04/30	CH2207	Female	Three y/o	Treatment (Wk 2)	Treatment	Feed Day	09:00	09:00 - 09:59	Late Morning	Auto-grooming
2019/04/30	CH2207	Female	Three y/o	Treatment (Wk 2)	Treatment	Feed Day	09:05	09:00 - 09:59	Late Morning	Inactive
2019/05/01	CH2207	Female	Three y/o	Treatment (Wk 2)	Treatment	Fast Day	09:05	09:00 - 09:59	Late Morning	Appetitive behaviour
2019/05/01	CH2207	Female	Three y/o	Treatment (Wk 2)	Treatment	Fast Day	09:10	09:00 - 09:59	Late Morning	Locomotion
2019/05/01	CH2207	Female	Three y/o	Treatment (Wk 2)	Treatment	Fast Day	09:15	09:00 - 09:59	Late Morning	Locomotion
2019/05/01	CH2207	Female	Three y/o	Treatment (Wk 2)	Treatment	Fast Day	09:20	09:00 - 09:59	Late Morning	Inactive
2019/05/01	CH2207	Female	Three y/o	Treatment (Wk 2)	Treatment	Fast Day	09:25	09:00 - 09:59	Late Morning	Inactive
2019/05/01	CH2207	Female	Three y/o	Treatment (Wk 2)	Treatment	Fast Day	09:30	09:00 - 09:59	Late Morning	Appetitive behaviour
2019/05/01	CH2207	Female	Three y/o	Treatment (Wk 2)	Treatment	Fast Day	09:35	09:00 - 09:59	Late Morning	Locomotion
2019/05/01	CH2207	Female	Three y/o	Treatment (Wk 2)	Treatment	Fast Day	09:40	09:00 - 09:59	Late Morning	Locomotion
2019/05/01	CH2207	Female	Three y/o	Treatment (Wk 2)	Treatment	Fast Day	09:45	09:00 - 09:59	Late Morning	Locomotion
2019/05/01	CH2207	Female	Three y/o	Treatment (Wk 2)	Treatment	Fast Day	09:50	09:00 - 09:59	Late Morning	Locomotion
2019/05/01	CH2207	Female	Three y/o	Treatment (Wk 2)	Treatment	Fast Day	09:55	09:00 - 09:59	Late Morning	Inactive

2019/05/01	CH2207	Female	Three y/o	Treatment (Wk 2)	Treatment	Fast Day	10:00	10:00 - 10:59	Late Morning	Inactive
2019/05/05	CH2207	Female	Three y/o	Treatment (Wk 2)	Treatment	Fast Day	08:35	08:00 - 08:59	Late Morning	Inactive
2019/05/05	CH2207	Female	Three y/o	Treatment (Wk 2)	Treatment	Fast Day	08:40	08:00 - 08:59	Late Morning	Scent-marking
2019/05/05	CH2207	Female	Three y/o	Treatment (Wk 2)	Treatment	Fast Day	08:45	08:00 - 08:59	Late Morning	Inactive
2019/05/05	CH2207	Female	Three y/o	Treatment (Wk 2)	Treatment	Fast Day	08:50	08:00 - 08:59	Late Morning	Inactive
2019/05/05	CH2207	Female	Three y/o	Treatment (Wk 2)	Treatment	Fast Day	08:55	08:00 - 08:59	Late Morning	Appetitive behaviour
2019/05/05	CH2207	Female	Three y/o	Treatment (Wk 2)	Treatment	Fast Day	09:00	09:00 - 09:59	Late Morning	Locomotion
2019/05/05	CH2207	Female	Three y/o	Treatment (Wk 2)	Treatment	Fast Day	09:05	09:00 - 09:59	Late Morning	Inactive
2019/05/05	CH2207	Female	Three y/o	Treatment (Wk 2)	Treatment	Fast Day	09:10	09:00 - 09:59	Late Morning	Inactive
2019/05/05	CH2207	Female	Three y/o	Treatment (Wk 2)	Treatment	Fast Day	09:15	09:00 - 09:59	Late Morning	Auto-grooming
2019/05/05	CH2207	Female	Three y/o	Treatment (Wk 2)	Treatment	Fast Day	09:20	09:00 - 09:59	Late Morning	Locomotion
2019/05/05	CH2207	Female	Three y/o	Treatment (Wk 2)	Treatment	Fast Day	09:25	09:00 - 09:59	Late Morning	Locomotion
2019/05/05	CH2207	Female	Three y/o	Treatment (Wk 2)	Treatment	Fast Day	09:30	09:00 - 09:59	Late Morning	Locomotion
2019/05/07	CH2207	Female	Three y/o	Treatment (Wk 3)	Treatment	Feed Day	09:05	09:00 - 09:59	Late Morning	Locomotion
2019/05/07	CH2207	Female	Three y/o	Treatment (Wk 3)	Treatment	Feed Day	09:10	09:00 - 09:59	Late Morning	Inactive
2019/05/07	CH2207	Female	Three y/o	Treatment (Wk 3)	Treatment	Feed Day	09:15	09:00 - 09:59	Late Morning	Inactive
2019/05/07	CH2207	Female	Three y/o	Treatment (Wk 3)	Treatment	Feed Day	09:20	09:00 - 09:59	Late Morning	Auto-grooming
2019/05/07	CH2207	Female	Three y/o	Treatment (Wk 3)	Treatment	Feed Day	09:25	09:00 - 09:59	Late Morning	Locomotion
2019/05/07	CH2207	Female	Three y/o	Treatment (Wk 3)	Treatment	Feed Day	09:30	09:00 - 09:59	Late Morning	Locomotion
2019/05/07	CH2207	Female	Three y/o	Treatment (Wk 3)	Treatment	Feed Day	09:35	09:00 - 09:59	Late Morning	Locomotion
2019/05/07	CH2207	Female	Three y/o	Treatment (Wk 3)	Treatment	Feed Day	09:40	09:00 - 09:59	Late Morning	Locomotion
2019/05/07	CH2207	Female	Three y/o	Treatment (Wk 3)	Treatment	Feed Day	09:45	09:00 - 09:59	Late Morning	Locomotion
2019/05/07	CH2207	Female	Three y/o	Treatment (Wk 3)	Treatment	Feed Day	09:50	09:00 - 09:59	Late Morning	Inactive
2019/05/07	CH2207	Female	Three y/o	Treatment (Wk 3)	Treatment	Feed Day	09:55	09:00 - 09:59	Late Morning	Inactive
2019/05/07	CH2207	Female	Three y/o	Treatment (Wk 3)	Treatment	Feed Day	10:00	10:00 - 10:59	Late Morning	Inactive
2019/05/08	CH2207	Female	Three y/o	Treatment (Wk 3)	Treatment	Fast Day	10:30	10:00 - 10:59	Late Morning	Inactive
2019/05/08	CH2207	Female	Three y/o	Treatment (Wk 3)	Treatment	Fast Day	10:35	10:00 - 10:59	Late Morning	Attention
2019/05/08	CH2207	Female	Three y/o	Treatment (Wk 3)	Treatment	Fast Day	10:40	10:00 - 10:59	Late Morning	Inactive
2019/05/08	CH2207	Female	Three y/o	Treatment (Wk 3)	Treatment	Fast Day	10:45	10:00 - 10:59	Late Morning	Inactive
2019/05/08	CH2207	Female	Three y/o	Treatment (Wk 3)	Treatment	Fast Day	10:50	10:00 - 10:59	Late Morning	Inactive
2019/05/08	CH2207	Female	Three y/o	Treatment (Wk 3)	Treatment	Fast Day	10:55	10:00 - 10:59	Late Morning	Inactive
2019/05/08	CH2207	Female	Three y/o	Treatment (Wk 3)	Treatment	Fast Day	11:00	11:00 - 11:59	Late Morning	Inactive
2019/05/08	CH2207	Female	Three y/o	Treatment (Wk 3)	Treatment	Fast Day	11:05	11:00 - 11:59	Late Morning	Inactive
2019/05/08	CH2207	Female	Three y/o	Treatment (Wk 3)	Treatment	Fast Day	11:10	11:00 - 11:59	Late Morning	Inactive
2019/05/08	CH2207	Female	Three y/o	Treatment (Wk 3)	Treatment	Fast Day	11:15	11:00 - 11:59	Late Morning	Inactive
2019/05/08	CH2207	Female	Three y/o	Treatment (Wk 3)	Treatment	Fast Day	11:20	11:00 - 11:59	Late Morning	Inactive
2019/05/08	CH2207	Female	Three y/o	Treatment (Wk 3)	Treatment	Fast Day	11:25	11:00 - 11:59	Late Morning	Appetitive behaviour

2019/05/10	CH2207	Female	Three y/o	Treatment (Wk 3)	Treatment	Feed Day	10:05	10:00 - 10:59	Late Morning	Inactive
2019/05/10	CH2207	Female	Three y/o	Treatment (Wk 3)	Treatment	Feed Day	10:10	10:00 - 10:59	Late Morning	Inactive
2019/05/10	CH2207	Female	Three y/o	Treatment (Wk 3)	Treatment	Feed Day	10:15	10:00 - 10:59	Late Morning	Inactive
2019/05/10	CH2207	Female	Three y/o	Treatment (Wk 3)	Treatment	Feed Day	10:20	10:00 - 10:59	Late Morning	Inactive
2019/05/10	CH2207	Female	Three y/o	Treatment (Wk 3)	Treatment	Feed Day	10:25	10:00 - 10:59	Late Morning	Inactive
2019/05/10	CH2207	Female	Three y/o	Treatment (Wk 3)	Treatment	Feed Day	10:30	10:00 - 10:59	Late Morning	Inactive
2019/05/10	CH2207	Female	Three y/o	Treatment (Wk 3)	Treatment	Feed Day	10:35	10:00 - 10:59	Late Morning	Inactive
2019/05/10	CH2207	Female	Three y/o	Treatment (Wk 3)	Treatment	Feed Day	10:40	10:00 - 10:59	Late Morning	Inactive
2019/05/10	CH2207	Female	Three y/o	Treatment (Wk 3)	Treatment	Feed Day	10:45	10:00 - 10:59	Late Morning	Inactive
2019/05/10	CH2207	Female	Three y/o	Treatment (Wk 3)	Treatment	Feed Day	10:50	10:00 - 10:59	Late Morning	Inactive
2019/05/10	CH2207	Female	Three y/o	Treatment (Wk 3)	Treatment	Feed Day	10:55	10:00 - 10:59	Late Morning	Inactive
2019/05/10	CH2207	Female	Three y/o	Treatment (Wk 3)	Treatment	Feed Day	11:00	11:00 - 11:59	Late Morning	Inactive
2019/05/11	CH2207	Female	Three y/o	Treatment (Wk 3)	Treatment	Fast Day	07:35	07:00 - 07:59	Morning	Inactive
2019/05/11	CH2207	Female	Three y/o	Treatment (Wk 3)	Treatment	Fast Day	07:40	07:00 - 07:59	Morning	Locomotion
2019/05/11	CH2207	Female	Three y/o	Treatment (Wk 3)	Treatment	Fast Day	07:45	07:00 - 07:59	Morning	Scent-marking
2019/05/11	CH2207	Female	Three y/o	Treatment (Wk 3)	Treatment	Fast Day	07:50	07:00 - 07:59	Morning	Auto-grooming
2019/05/11	CH2207	Female	Three y/o	Treatment (Wk 3)	Treatment	Fast Day	07:55	07:00 - 07:59	Morning	Auto-grooming
2019/05/11	CH2207	Female	Three y/o	Treatment (Wk 3)	Treatment	Fast Day	08:00	08:00 - 08:59	Late Morning	Auto-grooming
2019/05/11	CH2207	Female	Three y/o	Treatment (Wk 3)	Treatment	Fast Day	08:05	08:00 - 08:59	Late Morning	Auto-grooming
2019/05/11	CH2207	Female	Three y/o	Treatment (Wk 3)	Treatment	Fast Day	08:10	08:00 - 08:59	Late Morning	Attention
2019/05/11	CH2207	Female	Three y/o	Treatment (Wk 3)	Treatment	Fast Day	08:15	08:00 - 08:59	Late Morning	Inactive
2019/05/11	CH2207	Female	Three y/o	Treatment (Wk 3)	Treatment	Fast Day	08:20	08:00 - 08:59	Late Morning	Inactive
2019/05/11	CH2207	Female	Three y/o	Treatment (Wk 3)	Treatment	Fast Day	08:25	08:00 - 08:59	Late Morning	Auto-grooming
2019/05/11	CH2207	Female	Three y/o	Treatment (Wk 3)	Treatment	Fast Day	08:30	08:00 - 08:59	Late Morning	Inactive
2019/05/12	CH2207	Female	Three y/o	Treatment (Wk 3)	Treatment	Fast Day	07:55	07:00 - 07:59	Morning	Locomotion
2019/05/12	CH2207	Female	Three y/o	Treatment (Wk 3)	Treatment	Fast Day	08:00	08:00 - 08:59	Late Morning	Locomotion
2019/05/12	CH2207	Female	Three y/o	Treatment (Wk 3)	Treatment	Fast Day	08:05	08:00 - 08:59	Late Morning	Locomotion
2019/05/12	CH2207	Female	Three y/o	Treatment (Wk 3)	Treatment	Fast Day	08:10	08:00 - 08:59	Late Morning	Locomotion
2019/05/12	CH2207	Female	Three y/o	Treatment (Wk 3)	Treatment	Fast Day	08:15	08:00 - 08:59	Late Morning	Locomotion
2019/05/12	CH2207	Female	Three y/o	Treatment (Wk 3)	Treatment	Fast Day	08:20	08:00 - 08:59	Late Morning	Locomotion
2019/05/12	CH2207	Female	Three y/o	Treatment (Wk 3)	Treatment	Fast Day	08:25	08:00 - 08:59	Late Morning	Maintenance
2019/05/12	CH2207	Female	Three y/o	Treatment (Wk 3)	Treatment	Fast Day	08:30	08:00 - 08:59	Late Morning	Attention
2019/05/12	CH2207	Female	Three y/o	Treatment (Wk 3)	Treatment	Fast Day	08:35	08:00 - 08:59	Late Morning	Attention
2019/05/12	CH2207	Female	Three y/o	Treatment (Wk 3)	Treatment	Fast Day	08:40	08:00 - 08:59	Late Morning	Inactive
2019/05/12	CH2207	Female	Three y/o	Treatment (Wk 3)	Treatment	Fast Day	08:45	08:00 - 08:59	Late Morning	Attention
2019/05/12	CH2207	Female	Three y/o	Treatment (Wk 3)	Treatment	Fast Day	08:50	08:00 - 08:59	Late Morning	Inactive
2019/05/13	CH2207	Female	Three y/o	Treatment (Wk 3)	Treatment	Feed Day	09:20	09:00 - 09:59	Late Morning	Inactive

2019/05/13	CH2207	Female	Three y/o	Treatment (Wk 3)	Treatment	Feed Day	09:25	09:00 - 09:59	Late Morning	Locomotion
2019/05/13	CH2207	Female	Three y/o	Treatment (Wk 3)	Treatment	Feed Day	09:30	09:00 - 09:59	Late Morning	Locomotion
2019/05/13	CH2207	Female	Three y/o	Treatment (Wk 3)	Treatment	Feed Day	09:35	09:00 - 09:59	Late Morning	Locomotion
2019/05/13	CH2207	Female	Three y/o	Treatment (Wk 3)	Treatment	Feed Day	09:40	09:00 - 09:59	Late Morning	Locomotion
2019/05/13	CH2207	Female	Three y/o	Treatment (Wk 3)	Treatment	Feed Day	09:45	09:00 - 09:59	Late Morning	Locomotion
2019/05/13	CH2207	Female	Three y/o	Treatment (Wk 3)	Treatment	Feed Day	09:50	09:00 - 09:59	Late Morning	Locomotion
2019/05/13	CH2207	Female	Three y/o	Treatment (Wk 3)	Treatment	Feed Day	09:55	09:00 - 09:59	Late Morning	Locomotion
2019/05/13	CH2207	Female	Three y/o	Treatment (Wk 3)	Treatment	Feed Day	10:00	10:00 - 10:59	Late Morning	Auto-grooming
2019/05/13	CH2207	Female	Three y/o	Treatment (Wk 3)	Treatment	Feed Day	10:05	10:00 - 10:59	Late Morning	Locomotion
2019/05/13	CH2207	Female	Three y/o	Treatment (Wk 3)	Treatment	Feed Day	10:10	10:00 - 10:59	Late Morning	Locomotion
2019/05/13	CH2207	Female	Three y/o	Treatment (Wk 3)	Treatment	Feed Day	10:15	10:00 - 10:59	Late Morning	Auto-grooming
2019/05/15	CH2205	Male	Three y/o	Treatment (Wk 1)	Treatment	Fast Day	09:50	9:00 - 9:59	Late Morning	Attention
2019/05/15	CH2206	Male	Three y/o	Treatment (Wk 1)	Treatment	Fast Day	09:50	9:00 - 9:59	Late Morning	Attention
2019/05/15	CH2205	Male	Three y/o	Treatment (Wk 1)	Treatment	Fast Day	09:55	9:00 - 9:59	Late Morning	Locomotion
2019/05/15	CH2206	Male	Three y/o	Treatment (Wk 1)	Treatment	Fast Day	09:55	9:00 - 9:59	Late Morning	Attention
2019/05/15	CH2205	Male	Three y/o	Treatment (Wk 1)	Treatment	Fast Day	10:00	10:00 - 10:59	Late Morning	Inactive
2019/05/15	CH2206	Male	Three y/o	Treatment (Wk 1)	Treatment	Fast Day	10:00	10:00 - 10:59	Late Morning	Attention
2019/05/15	CH2205	Male	Three y/o	Treatment (Wk 1)	Treatment	Fast Day	10:05	10:00 - 10:59	Late Morning	Inactive
2019/05/15	CH2206	Male	Three y/o	Treatment (Wk 1)	Treatment	Fast Day	10:05	10:00 - 10:59	Late Morning	Inactive
2019/05/15	CH2205	Male	Three y/o	Treatment (Wk 1)	Treatment	Fast Day	10:10	10:00 - 10:59	Late Morning	Inactive
2019/05/15	CH2206	Male	Three y/o	Treatment (Wk 1)	Treatment	Fast Day	10:10	10:00 - 10:59	Late Morning	Attention
2019/05/15	CH2205	Male	Three y/o	Treatment (Wk 1)	Treatment	Fast Day	10:15	10:00 - 10:59	Late Morning	Inactive
2019/05/15	CH2206	Male	Three y/o	Treatment (Wk 1)	Treatment	Fast Day	10:15	10:00 - 10:59	Late Morning	Inactive
2019/05/15	CH2205	Male	Three y/o	Treatment (Wk 1)	Treatment	Fast Day	10:20	10:00 - 10:59	Late Morning	Locomotion
2019/05/15	CH2206	Male	Three y/o	Treatment (Wk 1)	Treatment	Fast Day	10:20	10:00 - 10:59	Late Morning	Inactive
2019/05/15	CH2205	Male	Three y/o	Treatment (Wk 1)	Treatment	Fast Day	10:25	10:00 - 10:59	Late Morning	Inactive
2019/05/15	CH2206	Male	Three y/o	Treatment (Wk 1)	Treatment	Fast Day	10:25	10:00 - 10:59	Late Morning	Inactive
2019/05/15	CH2205	Male	Three y/o	Treatment (Wk 1)	Treatment	Fast Day	10:30	10:00 - 10:59	Late Morning	Interspecific behaviour
2019/05/15	CH2206	Male	Three y/o	Treatment (Wk 1)	Treatment	Fast Day	10:30	10:00 - 10:59	Late Morning	Inactive
2019/05/15	CH2205	Male	Three y/o	Treatment (Wk 1)	Treatment	Fast Day	10:35	10:00 - 10:59	Late Morning	Inactive
2019/05/15	CH2206	Male	Three y/o	Treatment (Wk 1)	Treatment	Fast Day	10:35	10:00 - 10:59	Late Morning	Inactive
2019/05/15	CH2205	Male	Three y/o	Treatment (Wk 1)	Treatment	Fast Day	10:40	10:00 - 10:59	Late Morning	Inactive
2019/05/15	CH2206	Male	Three y/o	Treatment (Wk 1)	Treatment	Fast Day	10:40	10:00 - 10:59	Late Morning	Inactive
2019/05/15	CH2205	Male	Three y/o	Treatment (Wk 1)	Treatment	Fast Day	10:45	10:00 - 10:59	Late Morning	Inactive
2019/05/15	CH2206	Male	Three y/o	Treatment (Wk 1)	Treatment	Fast Day	10:45	10:00 - 10:59	Late Morning	Inactive
2019/05/17	CH2205	Male	Three y/o	Treatment (Wk 1)	Treatment	Feed Day	08:05	08:00 - 08:59	Late Morning	Attention
2019/05/17	CH2206	Male	Three y/o	Treatment (Wk 1)	Treatment	Feed Day	08:05	08:00 - 08:59	Late Morning	Attention

2019/05/17	CH2205	Male	Three y/o	Treatment (Wk 1)	Treatment	Feed Day	08:10	08:00 - 08:59	Late Morning	Locomotion
2019/05/17	CH2206	Male	Three y/o	Treatment (Wk 1)	Treatment	Feed Day	08:10	08:00 - 08:59	Late Morning	Attention
2019/05/17	CH2205	Male	Three y/o	Treatment (Wk 1)	Treatment	Feed Day	08:15	08:00 - 08:59	Late Morning	Locomotion
2019/05/17	CH2206	Male	Three y/o	Treatment (Wk 1)	Treatment	Feed Day	08:15	08:00 - 08:59	Late Morning	Attention
2019/05/17	CH2205	Male	Three y/o	Treatment (Wk 1)	Treatment	Feed Day	08:20	08:00 - 08:59	Late Morning	Locomotion
2019/05/17	CH2206	Male	Three y/o	Treatment (Wk 1)	Treatment	Feed Day	08:20	08:00 - 08:59	Late Morning	Locomotion
2019/05/17	CH2205	Male	Three y/o	Treatment (Wk 1)	Treatment	Feed Day	08:25	08:00 - 08:59	Late Morning	Inactive
2019/05/17	CH2206	Male	Three y/o	Treatment (Wk 1)	Treatment	Feed Day	08:25	08:00 - 08:59	Late Morning	Olfactory exploration
2019/05/17	CH2205	Male	Three y/o	Treatment (Wk 1)	Treatment	Feed Day	08:30	08:00 - 08:59	Late Morning	Inactive
2019/05/17	CH2206	Male	Three y/o	Treatment (Wk 1)	Treatment	Feed Day	08:30	08:00 - 08:59	Late Morning	Locomotion
2019/05/17	CH2205	Male	Three y/o	Treatment (Wk 1)	Treatment	Feed Day	08:35	08:00 - 08:59	Late Morning	Attention
2019/05/17	CH2206	Male	Three y/o	Treatment (Wk 1)	Treatment	Feed Day	08:35	08:00 - 08:59	Late Morning	Locomotion
2019/05/17	CH2205	Male	Three y/o	Treatment (Wk 1)	Treatment	Feed Day	08:40	08:00 - 08:59	Late Morning	Olfactory exploration
2019/05/17	CH2206	Male	Three y/o	Treatment (Wk 1)	Treatment	Feed Day	08:40	08:00 - 08:59	Late Morning	Appetitive behaviour
2019/05/17	CH2205	Male	Three y/o	Treatment (Wk 1)	Treatment	Feed Day	08:45	08:00 - 08:59	Late Morning	Locomotion
2019/05/17	CH2206	Male	Three y/o	Treatment (Wk 1)	Treatment	Feed Day	08:45	08:00 - 08:59	Late Morning	Appetitive behaviour
2019/05/17	CH2205	Male	Three y/o	Treatment (Wk 1)	Treatment	Feed Day	08:50	08:00 - 08:59	Late Morning	Locomotion
2019/05/17	CH2206	Male	Three y/o	Treatment (Wk 1)	Treatment	Feed Day	08:50	08:00 - 08:59	Late Morning	Scent-marking
2019/05/17	CH2205	Male	Three y/o	Treatment (Wk 1)	Treatment	Feed Day	08:55	08:00 - 08:59	Late Morning	Locomotion
2019/05/17	CH2206	Male	Three y/o	Treatment (Wk 1)	Treatment	Feed Day	08:55	08:00 - 08:59	Late Morning	Appetitive behaviour
2019/05/17	CH2205	Male	Three y/o	Treatment (Wk 1)	Treatment	Feed Day	09:00	09:00 - 09:59	Late Morning	Locomotion
2019/05/17	CH2206	Male	Three y/o	Treatment (Wk 1)	Treatment	Feed Day	09:00	09:00 - 09:59	Late Morning	Inactive
2019/05/20	CH2205	Male	Three y/o	Treatment (Wk 1)	Treatment	Feed Day	09:35	09:00 - 09:59	Late Morning	Attention
2019/05/20	CH2206	Male	Three y/o	Treatment (Wk 1)	Treatment	Feed Day	09:35	09:00 - 09:59	Late Morning	Attention
2019/05/20	CH2205	Male	Three y/o	Treatment (Wk 1)	Treatment	Feed Day	09:40	09:00 - 09:59	Late Morning	Olfactory exploration
2019/05/20	CH2206	Male	Three y/o	Treatment (Wk 1)	Treatment	Feed Day	09:40	09:00 - 09:59	Late Morning	Olfactory exploration
2019/05/20	CH2205	Male	Three y/o	Treatment (Wk 1)	Treatment	Feed Day	09:45	09:00 - 09:59	Late Morning	Inactive
2019/05/20	CH2206	Male	Three y/o	Treatment (Wk 1)	Treatment	Feed Day	09:45	09:00 - 09:59	Late Morning	Locomotion
2019/05/20	CH2205	Male	Three y/o	Treatment (Wk 1)	Treatment	Feed Day	09:50	09:00 - 09:59	Late Morning	Attention
2019/05/20	CH2206	Male	Three y/o	Treatment (Wk 1)	Treatment	Feed Day	09:50	09:00 - 09:59	Late Morning	Appetitive behaviour
2019/05/20	CH2205	Male	Three y/o	Treatment (Wk 1)	Treatment	Feed Day	09:55	09:00 - 09:59	Late Morning	Attention
2019/05/20	CH2206	Male	Three y/o	Treatment (Wk 1)	Treatment	Feed Day	09:55	09:00 - 09:59	Late Morning	Attention
2019/05/20	CH2205	Male	Three y/o	Treatment (Wk 1)	Treatment	Feed Day	10:00	10:00 - 10:59	Late Morning	Inactive
2019/05/20	CH2206	Male	Three y/o	Treatment (Wk 1)	Treatment	Feed Day	10:00	10:00 - 10:59	Late Morning	Appetitive behaviour
2019/05/20	CH2205	Male	Three y/o	Treatment (Wk 1)	Treatment	Feed Day	10:05	10:00 - 10:59	Late Morning	Locomotion
2019/05/20	CH2206	Male	Three y/o	Treatment (Wk 1)	Treatment	Feed Day	10:05	10:00 - 10:59	Late Morning	Attention
2019/05/20	CH2205	Male	Three y/o	Treatment (Wk 1)	Treatment	Feed Day	10:10	10:00 - 10:59	Late Morning	Inactive

2019/05/20	CH2206	Male	Three y/o	Treatment (Wk 1)	Treatment	Feed Day	10:10	10:00 - 10:59	Late Morning	Appetitive behaviour
2019/05/20	CH2205	Male	Three y/o	Treatment (Wk 1)	Treatment	Feed Day	10:15	10:00 - 10:59	Late Morning	Attention
2019/05/20	CH2206	Male	Three y/o	Treatment (Wk 1)	Treatment	Feed Day	10:15	10:00 - 10:59	Late Morning	Appetitive behaviour
2019/05/20	CH2205	Male	Three y/o	Treatment (Wk 1)	Treatment	Feed Day	10:20	10:00 - 10:59	Late Morning	Locomotion
2019/05/20	CH2206	Male	Three y/o	Treatment (Wk 1)	Treatment	Feed Day	10:20	10:00 - 10:59	Late Morning	Locomotion
2019/05/20	CH2205	Male	Three y/o	Treatment (Wk 1)	Treatment	Feed Day	10:25	10:00 - 10:59	Late Morning	Appetitive behaviour
2019/05/20	CH2206	Male	Three y/o	Treatment (Wk 1)	Treatment	Feed Day	10:25	10:00 - 10:59	Late Morning	Appetitive behaviour
2019/05/20	CH2205	Male	Three y/o	Treatment (Wk 1)	Treatment	Feed Day	10:30	10:00 - 10:59	Late Morning	Inactive
2019/05/20	CH2206	Male	Three y/o	Treatment (Wk 1)	Treatment	Feed Day	10:30	10:00 - 10:59	Late Morning	Inactive
2019/05/22	CH2205	Male	Three y/o	Treatment (Wk 2)	Treatment	Fast Day	08:15	08:00 - 08:59	Late Morning	Locomotion
2019/05/22	CH2206	Male	Three y/o	Treatment (Wk 2)	Treatment	Fast Day	08:15	08:00 - 08:59	Late Morning	Attention
2019/05/22	CH2205	Male	Three y/o	Treatment (Wk 2)	Treatment	Fast Day	08:20	08:00 - 08:59	Late Morning	Locomotion
2019/05/22	CH2206	Male	Three y/o	Treatment (Wk 2)	Treatment	Fast Day	08:20	08:00 - 08:59	Late Morning	Locomotion
2019/05/22	CH2205	Male	Three y/o	Treatment (Wk 2)	Treatment	Fast Day	08:25	08:00 - 08:59	Late Morning	Inactive
2019/05/22	CH2206	Male	Three y/o	Treatment (Wk 2)	Treatment	Fast Day	08:25	08:00 - 08:59	Late Morning	Auto-grooming
2019/05/22	CH2205	Male	Three y/o	Treatment (Wk 2)	Treatment	Fast Day	08:30	08:00 - 08:59	Late Morning	Auto-grooming
2019/05/22	CH2206	Male	Three y/o	Treatment (Wk 2)	Treatment	Fast Day	08:30	08:00 - 08:59	Late Morning	Appetitive behaviour
2019/05/22	CH2205	Male	Three y/o	Treatment (Wk 2)	Treatment	Fast Day	08:35	08:00 - 08:59	Late Morning	Locomotion
2019/05/22	CH2206	Male	Three y/o	Treatment (Wk 2)	Treatment	Fast Day	08:35	08:00 - 08:59	Late Morning	Appetitive behaviour
2019/05/22	CH2205	Male	Three y/o	Treatment (Wk 2)	Treatment	Fast Day	08:40	08:00 - 08:59	Late Morning	Appetitive behaviour
2019/05/22	CH2206	Male	Three y/o	Treatment (Wk 2)	Treatment	Fast Day	08:40	08:00 - 08:59	Late Morning	Appetitive behaviour
2019/05/22	CH2205	Male	Three y/o	Treatment (Wk 2)	Treatment	Fast Day	08:45	08:00 - 08:59	Late Morning	Appetitive behaviour
2019/05/22	CH2206	Male	Three y/o	Treatment (Wk 2)	Treatment	Fast Day	08:45	08:00 - 08:59	Late Morning	Appetitive behaviour
2019/05/22	CH2205	Male	Three y/o	Treatment (Wk 2)	Treatment	Fast Day	08:50	08:00 - 08:59	Late Morning	Interspecific behaviour
2019/05/22	CH2206	Male	Three y/o	Treatment (Wk 2)	Treatment	Fast Day	08:50	08:00 - 08:59	Late Morning	Appetitive behaviour
2019/05/22	CH2205	Male	Three y/o	Treatment (Wk 2)	Treatment	Fast Day	08:55	08:00 - 08:59	Late Morning	Appetitive behaviour
2019/05/22	CH2206	Male	Three y/o	Treatment (Wk 2)	Treatment	Fast Day	08:55	08:00 - 08:59	Late Morning	Interspecific behaviour
2019/05/22	CH2205	Male	Three y/o	Treatment (Wk 2)	Treatment	Fast Day	09:00	09:00 - 09:59	Late Morning	Vocalisation
2019/05/22	CH2206	Male	Three y/o	Treatment (Wk 2)	Treatment	Fast Day	09:00	09:00 - 09:59	Late Morning	Locomotion
2019/05/22	CH2205	Male	Three y/o	Treatment (Wk 2)	Treatment	Fast Day	09:05	09:00 - 09:59	Late Morning	Vocalisation
2019/05/22	CH2206	Male	Three y/o	Treatment (Wk 2)	Treatment	Fast Day	09:05	09:00 - 09:59	Late Morning	Inactive
2019/05/22	CH2205	Male	Three y/o	Treatment (Wk 2)	Treatment	Fast Day	09:10	09:00 - 09:59	Late Morning	Vocalisation
2019/05/22	CH2206	Male	Three y/o	Treatment (Wk 2)	Treatment	Fast Day	09:10	09:00 - 09:59	Late Morning	Locomotion
2019/05/23	CH2205	Male	Three y/o	Treatment (Wk 2)	Treatment	Feed Day	09:10	09:00 - 09:59	Late Morning	Attention
2019/05/23	CH2206	Male	Three y/o	Treatment (Wk 2)	Treatment	Feed Day	09:10	09:00 - 09:59	Late Morning	Attention
2019/05/23	CH2205	Male	Three y/o	Treatment (Wk 2)	Treatment	Feed Day	09:15	09:00 - 09:59	Late Morning	Locomotion
2019/05/23	CH2206	Male	Three y/o	Treatment (Wk 2)	Treatment	Feed Day	09:15	09:00 - 09:59	Late Morning	Attention

2019/05/23	CH2205	Male	Three y/o	Treatment (Wk 2)	Treatment	Feed Day	09:20	09:00 - 09:59	Late Morning	Inactive
2019/05/23	CH2206	Male	Three y/o	Treatment (Wk 2)	Treatment	Feed Day	09:20	09:00 - 09:59	Late Morning	Auto-grooming
2019/05/23	CH2205	Male	Three y/o	Treatment (Wk 2)	Treatment	Feed Day	09:25	09:00 - 09:59	Late Morning	Inactive
2019/05/23	CH2206	Male	Three y/o	Treatment (Wk 2)	Treatment	Feed Day	09:25	09:00 - 09:59	Late Morning	Inactive
2019/05/23	CH2205	Male	Three y/o	Treatment (Wk 2)	Treatment	Feed Day	09:30	09:00 - 09:59	Late Morning	Attention
2019/05/23	CH2206	Male	Three y/o	Treatment (Wk 2)	Treatment	Feed Day	09:30	09:00 - 09:59	Late Morning	Attention
2019/05/23	CH2205	Male	Three y/o	Treatment (Wk 2)	Treatment	Feed Day	09:35	09:00 - 09:59	Late Morning	Inactive
2019/05/23	CH2206	Male	Three y/o	Treatment (Wk 2)	Treatment	Feed Day	09:35	09:00 - 09:59	Late Morning	Locomotion
2019/05/23	CH2205	Male	Three y/o	Treatment (Wk 2)	Treatment	Feed Day	09:40	09:00 - 09:59	Late Morning	Inactive
2019/05/23	CH2206	Male	Three y/o	Treatment (Wk 2)	Treatment	Feed Day	09:40	09:00 - 09:59	Late Morning	Inactive
2019/05/23	CH2205	Male	Three y/o	Treatment (Wk 2)	Treatment	Feed Day	09:45	09:00 - 09:59	Late Morning	Attention
2019/05/23	CH2206	Male	Three y/o	Treatment (Wk 2)	Treatment	Feed Day	09:45	09:00 - 09:59	Late Morning	Inactive
2019/05/23	CH2205	Male	Three y/o	Treatment (Wk 2)	Treatment	Feed Day	09:50	09:00 - 09:59	Late Morning	Scent-marking
2019/05/23	CH2206	Male	Three y/o	Treatment (Wk 2)	Treatment	Feed Day	09:50	09:00 - 09:59	Late Morning	Inactive
2019/05/23	CH2205	Male	Three y/o	Treatment (Wk 2)	Treatment	Feed Day	09:55	09:00 - 09:59	Late Morning	Inactive
2019/05/23	CH2206	Male	Three y/o	Treatment (Wk 2)	Treatment	Feed Day	09:55	09:00 - 09:59	Late Morning	Inactive
2019/05/23	CH2205	Male	Three y/o	Treatment (Wk 2)	Treatment	Feed Day	10:00	10:00 - 10:59	Late Morning	Inactive
2019/05/23	CH2206	Male	Three y/o	Treatment (Wk 2)	Treatment	Feed Day	10:00	10:00 - 10:59	Late Morning	Inactive
2019/05/23	CH2205	Male	Three y/o	Treatment (Wk 2)	Treatment	Feed Day	10:05	10:00 - 10:59	Late Morning	Inactive
2019/05/23	CH2206	Male	Three y/o	Treatment (Wk 2)	Treatment	Feed Day	10:05	10:00 - 10:59	Late Morning	Inactive
2019/05/26	CH2205	Male	Three y/o	Treatment (Wk 2)	Treatment	Fast Day	12:05	12:00 - 12:59	Afternoon	Inactive
2019/05/26	CH2206	Male	Three y/o	Treatment (Wk 2)	Treatment	Fast Day	12:05	12:00 - 12:59	Afternoon	Inactive
2019/05/26	CH2205	Male	Three y/o	Treatment (Wk 2)	Treatment	Fast Day	12:10	12:00 - 12:59	Afternoon	Inactive
2019/05/26	CH2206	Male	Three y/o	Treatment (Wk 2)	Treatment	Fast Day	12:10	12:00 - 12:59	Afternoon	Inactive
2019/05/26	CH2205	Male	Three y/o	Treatment (Wk 2)	Treatment	Fast Day	12:15	12:00 - 12:59	Afternoon	Attention
2019/05/26	CH2206	Male	Three y/o	Treatment (Wk 2)	Treatment	Fast Day	12:15	12:00 - 12:59	Afternoon	Inactive
2019/05/26	CH2205	Male	Three y/o	Treatment (Wk 2)	Treatment	Fast Day	12:20	12:00 - 12:59	Afternoon	Inactive
2019/05/26	CH2206	Male	Three y/o	Treatment (Wk 2)	Treatment	Fast Day	12:20	12:00 - 12:59	Afternoon	Inactive
2019/05/26	CH2205	Male	Three y/o	Treatment (Wk 2)	Treatment	Fast Day	12:25	12:00 - 12:59	Afternoon	Inactive
2019/05/26	CH2206	Male	Three y/o	Treatment (Wk 2)	Treatment	Fast Day	12:25	12:00 - 12:59	Afternoon	Inactive
2019/05/26	CH2205	Male	Three y/o	Treatment (Wk 2)	Treatment	Fast Day	12:30	12:00 - 12:59	Afternoon	Inactive
2019/05/26	CH2206	Male	Three y/o	Treatment (Wk 2)	Treatment	Fast Day	12:30	12:00 - 12:59	Afternoon	Inactive
2019/05/26	CH2205	Male	Three y/o	Treatment (Wk 2)	Treatment	Fast Day	12:35	12:00 - 12:59	Afternoon	Inactive
2019/05/26	CH2206	Male	Three y/o	Treatment (Wk 2)	Treatment	Fast Day	12:35	12:00 - 12:59	Afternoon	Inactive
2019/05/26	CH2205	Male	Three y/o	Treatment (Wk 2)	Treatment	Fast Day	12:40	12:00 - 12:59	Afternoon	Inactive
2019/05/26	CH2206	Male	Three y/o	Treatment (Wk 2)	Treatment	Fast Day	12:40	12:00 - 12:59	Afternoon	Inactive
2019/05/26	CH2205	Male	Three y/o	Treatment (Wk 2)	Treatment	Fast Day	12:45	12:00 - 12:59	Afternoon	Inactive

2019/05/26	CH2206	Male	Three y/o	Treatment (Wk 2)	Treatment	Fast Day	12:45	12:00 - 12:59	Afternoon	Inactive
2019/05/26	CH2205	Male	Three y/o	Treatment (Wk 2)	Treatment	Fast Day	12:50	12:00 - 12:59	Afternoon	Inactive
2019/05/26	CH2206	Male	Three y/o	Treatment (Wk 2)	Treatment	Fast Day	12:50	12:00 - 12:59	Afternoon	Inactive
2019/05/26	CH2205	Male	Three y/o	Treatment (Wk 2)	Treatment	Fast Day	12:55	12:00 - 12:59	Afternoon	Auto-grooming
2019/05/26	CH2206	Male	Three y/o	Treatment (Wk 2)	Treatment	Fast Day	12:55	12:00 - 12:59	Afternoon	Inactive
2019/05/26	CH2205	Male	Three y/o	Treatment (Wk 2)	Treatment	Fast Day	13:00	13:00 - 13:59	Afternoon	Inactive
2019/05/26	CH2206	Male	Three y/o	Treatment (Wk 2)	Treatment	Fast Day	13:00	13:00 - 13:59	Afternoon	Inactive
2019/05/27	CH2205	Male	Three y/o	Treatment (Wk 2)	Treatment	Feed Day	09:40	09:00 - 09:59	Late Morning	Standing
2019/05/27	CH2206	Male	Three y/o	Treatment (Wk 2)	Treatment	Feed Day	09:40	09:00 - 09:59	Late Morning	Auto-grooming
2019/05/27	CH2205	Male	Three y/o	Treatment (Wk 2)	Treatment	Feed Day	09:45	09:00 - 09:59	Late Morning	Interspecific behaviour
2019/05/27	CH2206	Male	Three y/o	Treatment (Wk 2)	Treatment	Feed Day	09:45	09:00 - 09:59	Late Morning	Appetitive behaviour
2019/05/27	CH2205	Male	Three y/o	Treatment (Wk 2)	Treatment	Feed Day	09:50	09:00 - 09:59	Late Morning	Inactive
2019/05/27	CH2206	Male	Three y/o	Treatment (Wk 2)	Treatment	Feed Day	09:50	09:00 - 09:59	Late Morning	Locomotion
2019/05/27	CH2205	Male	Three y/o	Treatment (Wk 2)	Treatment	Feed Day	09:55	09:00 - 09:59	Late Morning	Locomotion
2019/05/27	CH2206	Male	Three y/o	Treatment (Wk 2)	Treatment	Feed Day	09:55	09:00 - 09:59	Late Morning	Locomotion
2019/05/27	CH2205	Male	Three y/o	Treatment (Wk 2)	Treatment	Feed Day	10:00	10:00 - 10:59	Late Morning	Interspecific behaviour
2019/05/27	CH2206	Male	Three y/o	Treatment (Wk 2)	Treatment	Feed Day	10:00	10:00 - 10:59	Late Morning	Locomotion
2019/05/27	CH2205	Male	Three y/o	Treatment (Wk 2)	Treatment	Feed Day	10:05	10:00 - 10:59	Late Morning	Locomotion
2019/05/27	CH2206	Male	Three y/o	Treatment (Wk 2)	Treatment	Feed Day	10:05	10:00 - 10:59	Late Morning	Locomotion
2019/05/27	CH2205	Male	Three y/o	Treatment (Wk 2)	Treatment	Feed Day	10:10	10:00 - 10:59	Late Morning	Olfactory exploration
2019/05/27	CH2206	Male	Three y/o	Treatment (Wk 2)	Treatment	Feed Day	10:10	10:00 - 10:59	Late Morning	Appetitive behaviour
2019/05/27	CH2205	Male	Three y/o	Treatment (Wk 2)	Treatment	Feed Day	10:15	10:00 - 10:59	Late Morning	Interspecific behaviour
2019/05/27	CH2206	Male	Three y/o	Treatment (Wk 2)	Treatment	Feed Day	10:15	10:00 - 10:59	Late Morning	Appetitive behaviour
2019/05/27	CH2205	Male	Three y/o	Treatment (Wk 2)	Treatment	Feed Day	10:20	10:00 - 10:59	Late Morning	Interspecific behaviour
2019/05/27	CH2206	Male	Three y/o	Treatment (Wk 2)	Treatment	Feed Day	10:20	10:00 - 10:59	Late Morning	Appetitive behaviour
2019/05/27	CH2205	Male	Three y/o	Treatment (Wk 2)	Treatment	Feed Day	10:25	10:00 - 10:59	Late Morning	Inactive
2019/05/27	CH2206	Male	Three y/o	Treatment (Wk 2)	Treatment	Feed Day	10:25	10:00 - 10:59	Late Morning	Interspecific behaviour
2019/05/27	CH2205	Male	Three y/o	Treatment (Wk 2)	Treatment	Feed Day	10:30	10:00 - 10:59	Late Morning	Interspecific behaviour
2019/05/27	CH2206	Male	Three y/o	Treatment (Wk 2)	Treatment	Feed Day	10:30	10:00 - 10:59	Late Morning	Interspecific behaviour
2019/05/27	CH2205	Male	Three y/o	Treatment (Wk 2)	Treatment	Feed Day	10:35	10:00 - 10:59	Late Morning	Interspecific behaviour
2019/05/27	CH2206	Male	Three y/o	Treatment (Wk 2)	Treatment	Feed Day	10:35	10:00 - 10:59	Late Morning	Interspecific behaviour
2019/05/28	CH2205	Male	Three y/o	Treatment (Wk 2)	Treatment	Feed Day	10:05	10:00 - 10:59	Late Morning	Olfactory exploration
2019/05/28	CH2206	Male	Three y/o	Treatment (Wk 2)	Treatment	Feed Day	10:05	10:00 - 10:59	Late Morning	Locomotion
2019/05/28	CH2205	Male	Three y/o	Treatment (Wk 2)	Treatment	Feed Day	10:10	10:00 - 10:59	Late Morning	Locomotion
2019/05/28	CH2206	Male	Three y/o	Treatment (Wk 2)	Treatment	Feed Day	10:10	10:00 - 10:59	Late Morning	Appetitive behaviour
2019/05/28	CH2205	Male	Three y/o	Treatment (Wk 2)	Treatment	Feed Day	10:15	10:00 - 10:59	Late Morning	Scent-marking
2019/05/28	CH2206	Male	Three y/o	Treatment (Wk 2)	Treatment	Feed Day	10:15	10:00 - 10:59	Late Morning	Appetitive behaviour

2019/05/28	CH2205	Male	Three y/o	Treatment (Wk 2)	Treatment	Feed Day	10:20	10:00 - 10:59	Late Morning	Attention
2019/05/28	CH2206	Male	Three y/o	Treatment (Wk 2)	Treatment	Feed Day	10:20	10:00 - 10:59	Late Morning	Appetitive behaviour
2019/05/28	CH2205	Male	Three y/o	Treatment (Wk 2)	Treatment	Feed Day	10:25	10:00 - 10:59	Late Morning	Scent-marking
2019/05/28	CH2206	Male	Three y/o	Treatment (Wk 2)	Treatment	Feed Day	10:25	10:00 - 10:59	Late Morning	Appetitive behaviour
2019/05/28	CH2205	Male	Three y/o	Treatment (Wk 2)	Treatment	Feed Day	10:30	10:00 - 10:59	Late Morning	Attention
2019/05/28	CH2206	Male	Three y/o	Treatment (Wk 2)	Treatment	Feed Day	10:30	10:00 - 10:59	Late Morning	Scent-marking
2019/05/28	CH2205	Male	Three y/o	Treatment (Wk 2)	Treatment	Feed Day	10:35	10:00 - 10:59	Late Morning	Locomotion
2019/05/28	CH2206	Male	Three y/o	Treatment (Wk 2)	Treatment	Feed Day	10:35	10:00 - 10:59	Late Morning	Scent-marking
2019/05/28	CH2205	Male	Three y/o	Treatment (Wk 2)	Treatment	Feed Day	10:40	10:00 - 10:59	Late Morning	Attention
2019/05/28	CH2206	Male	Three y/o	Treatment (Wk 2)	Treatment	Feed Day	10:40	10:00 - 10:59	Late Morning	Attention
2019/05/28	CH2205	Male	Three y/o	Treatment (Wk 2)	Treatment	Feed Day	10:45	10:00 - 10:59	Late Morning	Attention
2019/05/28	CH2206	Male	Three y/o	Treatment (Wk 2)	Treatment	Feed Day	10:45	10:00 - 10:59	Late Morning	Locomotion
2019/05/28	CH2205	Male	Three y/o	Treatment (Wk 2)	Treatment	Feed Day	10:50	10:00 - 10:59	Late Morning	Interspecific behaviour
2019/05/28	CH2206	Male	Three y/o	Treatment (Wk 2)	Treatment	Feed Day	10:50	10:00 - 10:59	Late Morning	Locomotion
2019/05/28	CH2205	Male	Three y/o	Treatment (Wk 2)	Treatment	Feed Day	10:55	10:00 - 10:59	Late Morning	Locomotion
2019/05/28	CH2206	Male	Three y/o	Treatment (Wk 2)	Treatment	Feed Day	10:55	10:00 - 10:59	Late Morning	Appetitive behaviour
2019/05/28	CH2205	Male	Three y/o	Treatment (Wk 2)	Treatment	Feed Day	11:00	11:00 - 11:59	Late Morning	Interspecific behaviour
2019/05/28	CH2206	Male	Three y/o	Treatment (Wk 2)	Treatment	Feed Day	11:00	11:00 - 11:59	Late Morning	Appetitive behaviour
2019/05/29	CH2205	Male	Three y/o	Treatment (Wk 3)	Treatment	Fast Day	09:00	09:00 - 09:59	Late Morning	Locomotion
2019/05/29	CH2206	Male	Three y/o	Treatment (Wk 3)	Treatment	Fast Day	09:00	09:00 - 09:59	Late Morning	Appetitive behaviour
2019/05/29	CH2205	Male	Three y/o	Treatment (Wk 3)	Treatment	Fast Day	09:05	09:00 - 09:59	Late Morning	Attention
2019/05/29	CH2206	Male	Three y/o	Treatment (Wk 3)	Treatment	Fast Day	09:05	09:00 - 09:59	Late Morning	Appetitive behaviour
2019/05/29	CH2205	Male	Three y/o	Treatment (Wk 3)	Treatment	Fast Day	09:10	09:00 - 09:59	Late Morning	Locomotion
2019/05/29	CH2206	Male	Three y/o	Treatment (Wk 3)	Treatment	Fast Day	09:10	09:00 - 09:59	Late Morning	Olfactory exploration
2019/05/29	CH2205	Male	Three y/o	Treatment (Wk 3)	Treatment	Fast Day	09:15	09:00 - 09:59	Late Morning	Olfactory exploration
2019/05/29	CH2206	Male	Three y/o	Treatment (Wk 3)	Treatment	Fast Day	09:15	09:00 - 09:59	Late Morning	Locomotion
2019/05/29	CH2205	Male	Three y/o	Treatment (Wk 3)	Treatment	Fast Day	09:20	09:00 - 09:59	Late Morning	Locomotion
2019/05/29	CH2206	Male	Three y/o	Treatment (Wk 3)	Treatment	Fast Day	09:20	09:00 - 09:59	Late Morning	Appetitive behaviour
2019/05/29	CH2205	Male	Three y/o	Treatment (Wk 3)	Treatment	Fast Day	09:25	09:00 - 09:59	Late Morning	Olfactory exploration
2019/05/29	CH2206	Male	Three y/o	Treatment (Wk 3)	Treatment	Fast Day	09:25	09:00 - 09:59	Late Morning	Appetitive behaviour
2019/05/29	CH2205	Male	Three y/o	Treatment (Wk 3)	Treatment	Fast Day	09:30	09:00 - 09:59	Late Morning	Inactive
2019/05/29	CH2206	Male	Three y/o	Treatment (Wk 3)	Treatment	Fast Day	09:30	09:00 - 09:59	Late Morning	Appetitive behaviour
2019/05/29	CH2205	Male	Three y/o	Treatment (Wk 3)	Treatment	Fast Day	09:35	09:00 - 09:59	Late Morning	Interspecific behaviour
2019/05/29	CH2206	Male	Three y/o	Treatment (Wk 3)	Treatment	Fast Day	09:35	09:00 - 09:59	Late Morning	Locomotion
2019/05/29	CH2205	Male	Three y/o	Treatment (Wk 3)	Treatment	Fast Day	09:40	09:00 - 09:59	Late Morning	Vocalisation
2019/05/29	CH2206	Male	Three y/o	Treatment (Wk 3)	Treatment	Fast Day	09:40	09:00 - 09:59	Late Morning	Locomotion
2019/05/29	CH2205	Male	Three y/o	Treatment (Wk 3)	Treatment	Fast Day	09:45	09:00 - 09:59	Late Morning	Inactive

2019/05/29	CH2206	Male	Three y/o	Treatment (Wk 3)	Treatment	Fast Day	09:45	09:00 - 09:59	Late Morning	Locomotion
2019/05/29	CH2205	Male	Three y/o	Treatment (Wk 3)	Treatment	Fast Day	09:50	09:00 - 09:59	Late Morning	Locomotion
2019/05/29	CH2206	Male	Three y/o	Treatment (Wk 3)	Treatment	Fast Day	09:50	09:00 - 09:59	Late Morning	Locomotion
2019/05/29	CH2205	Male	Three y/o	Treatment (Wk 3)	Treatment	Fast Day	09:55	09:00 - 09:59	Late Morning	Olfactory exploration
2019/05/29	CH2206	Male	Three y/o	Treatment (Wk 3)	Treatment	Fast Day	09:55	09:00 - 09:59	Late Morning	Inactive
2019/05/30	CH2205	Male	Three y/o	Treatment (Wk 3)	Treatment	Feed Day	09:45	09:00 - 09:59	Late Morning	Locomotion
2019/05/30	CH2206	Male	Three y/o	Treatment (Wk 3)	Treatment	Feed Day	09:45	09:00 - 09:59	Late Morning	Inactive
2019/05/30	CH2205	Male	Three y/o	Treatment (Wk 3)	Treatment	Feed Day	09:50	09:00 - 09:59	Late Morning	Interspecific behaviour
2019/05/30	CH2206	Male	Three y/o	Treatment (Wk 3)	Treatment	Feed Day	09:50	09:00 - 09:59	Late Morning	Interspecific behaviour
2019/05/30	CH2205	Male	Three y/o	Treatment (Wk 3)	Treatment	Feed Day	09:55	09:00 - 09:59	Late Morning	Locomotion
2019/05/30	CH2206	Male	Three y/o	Treatment (Wk 3)	Treatment	Feed Day	09:55	09:00 - 09:59	Late Morning	Interspecific behaviour
2019/05/30	CH2205	Male	Three y/o	Treatment (Wk 3)	Treatment	Feed Day	10:00	10:00 - 10:59	Late Morning	Inactive
2019/05/30	CH2206	Male	Three y/o	Treatment (Wk 3)	Treatment	Feed Day	10:00	10:00 - 10:59	Late Morning	Locomotion
2019/05/30	CH2205	Male	Three y/o	Treatment (Wk 3)	Treatment	Feed Day	10:05	10:00 - 10:59	Late Morning	Inactive
2019/05/30	CH2206	Male	Three y/o	Treatment (Wk 3)	Treatment	Feed Day	10:05	10:00 - 10:59	Late Morning	Locomotion
2019/05/30	CH2205	Male	Three y/o	Treatment (Wk 3)	Treatment	Feed Day	10:10	10:00 - 10:59	Late Morning	Inactive
2019/05/30	CH2206	Male	Three y/o	Treatment (Wk 3)	Treatment	Feed Day	10:10	10:00 - 10:59	Late Morning	Inactive
2019/05/30	CH2205	Male	Three y/o	Treatment (Wk 3)	Treatment	Feed Day	10:15	10:00 - 10:59	Late Morning	Inactive
2019/05/30	CH2206	Male	Three y/o	Treatment (Wk 3)	Treatment	Feed Day	10:15	10:00 - 10:59	Late Morning	Inactive
2019/05/30	CH2205	Male	Three y/o	Treatment (Wk 3)	Treatment	Feed Day	10:20	10:00 - 10:59	Late Morning	Inactive
2019/05/30	CH2206	Male	Three y/o	Treatment (Wk 3)	Treatment	Feed Day	10:20	10:00 - 10:59	Late Morning	Locomotion
2019/05/30	CH2205	Male	Three y/o	Treatment (Wk 3)	Treatment	Feed Day	10:25	10:00 - 10:59	Late Morning	Locomotion
2019/05/30	CH2206	Male	Three y/o	Treatment (Wk 3)	Treatment	Feed Day	10:25	10:00 - 10:59	Late Morning	Inactive
2019/05/30	CH2205	Male	Three y/o	Treatment (Wk 3)	Treatment	Feed Day	10:30	10:00 - 10:59	Late Morning	Inactive
2019/05/30	CH2206	Male	Three y/o	Treatment (Wk 3)	Treatment	Feed Day	10:30	10:00 - 10:59	Late Morning	Inactive
2019/05/30	CH2205	Male	Three y/o	Treatment (Wk 3)	Treatment	Feed Day	10:35	10:00 - 10:59	Late Morning	Inactive
2019/05/30	CH2206	Male	Three y/o	Treatment (Wk 3)	Treatment	Feed Day	10:35	10:00 - 10:59	Late Morning	Inactive
2019/05/30	CH2205	Male	Three y/o	Treatment (Wk 3)	Treatment	Feed Day	10:40	10:00 - 10:59	Late Morning	Auto-grooming
2019/05/30	CH2206	Male	Three y/o	Treatment (Wk 3)	Treatment	Feed Day	10:40	10:00 - 10:59	Late Morning	Inactive
2019/05/31	CH2205	Male	Three y/o	Treatment (Wk 3)	Treatment	Feed Day	09:00	09:00 - 09:59	Late Morning	Locomotion
2019/05/31	CH2206	Male	Three y/o	Treatment (Wk 3)	Treatment	Feed Day	09:00	09:00 - 09:59	Late Morning	Inactive
2019/05/31	CH2205	Male	Three y/o	Treatment (Wk 3)	Treatment	Feed Day	09:05	09:00 - 09:59	Late Morning	Attention
2019/05/31	CH2206	Male	Three y/o	Treatment (Wk 3)	Treatment	Feed Day	09:05	09:00 - 09:59	Late Morning	Attention
2019/05/31	CH2205	Male	Three y/o	Treatment (Wk 3)	Treatment	Feed Day	09:10	09:00 - 09:59	Late Morning	Attention
2019/05/31	CH2206	Male	Three y/o	Treatment (Wk 3)	Treatment	Feed Day	09:10	09:00 - 09:59	Late Morning	Locomotion
2019/05/31	CH2205	Male	Three y/o	Treatment (Wk 3)	Treatment	Feed Day	09:15	09:00 - 09:59	Late Morning	Locomotion
2019/05/31	CH2206	Male	Three y/o	Treatment (Wk 3)	Treatment	Feed Day	09:15	09:00 - 09:59	Late Morning	Locomotion

2019/05/31	CH2205	Male	Three y/o	Treatment (Wk 3)	Treatment	Feed Day	09:20	09:00 - 09:59	Late Morning	Locomotion
2019/05/31	CH2206	Male	Three y/o	Treatment (Wk 3)	Treatment	Feed Day	09:20	09:00 - 09:59	Late Morning	Olfactory exploration
2019/05/31	CH2205	Male	Three y/o	Treatment (Wk 3)	Treatment	Feed Day	09:25	09:00 - 09:59	Late Morning	Inactive
2019/05/31	CH2206	Male	Three y/o	Treatment (Wk 3)	Treatment	Feed Day	09:25	09:00 - 09:59	Late Morning	Locomotion
2019/05/31	CH2205	Male	Three y/o	Treatment (Wk 3)	Treatment	Feed Day	09:30	09:00 - 09:59	Late Morning	Inactive
2019/05/31	CH2206	Male	Three y/o	Treatment (Wk 3)	Treatment	Feed Day	09:30	09:00 - 09:59	Late Morning	Appetitive behaviour
2019/05/31	CH2205	Male	Three y/o	Treatment (Wk 3)	Treatment	Feed Day	09:35	09:00 - 09:59	Late Morning	Interspecific behaviour
2019/05/31	CH2206	Male	Three y/o	Treatment (Wk 3)	Treatment	Feed Day	09:35	09:00 - 09:59	Late Morning	Inactive
2019/05/31	CH2205	Male	Three y/o	Treatment (Wk 3)	Treatment	Feed Day	09:40	09:00 - 09:59	Late Morning	Inactive
2019/05/31	CH2206	Male	Three y/o	Treatment (Wk 3)	Treatment	Feed Day	09:40	09:00 - 09:59	Late Morning	Locomotion
2019/05/31	CH2205	Male	Three y/o	Treatment (Wk 3)	Treatment	Feed Day	09:45	09:00 - 09:59	Late Morning	Inactive
2019/05/31	CH2206	Male	Three y/o	Treatment (Wk 3)	Treatment	Feed Day	09:45	09:00 - 09:59	Late Morning	Locomotion
2019/05/31	CH2205	Male	Three y/o	Treatment (Wk 3)	Treatment	Feed Day	09:50	09:00 - 09:59	Late Morning	Interspecific behaviour
2019/05/31	CH2206	Male	Three y/o	Treatment (Wk 3)	Treatment	Feed Day	09:50	09:00 - 09:59	Late Morning	Locomotion
2019/05/31	CH2205	Male	Three y/o	Treatment (Wk 3)	Treatment	Feed Day	09:55	09:00 - 09:59	Late Morning	Inactive
2019/05/31	CH2206	Male	Three y/o	Treatment (Wk 3)	Treatment	Feed Day	09:55	09:00 - 09:59	Late Morning	Inactive
2019/06/01	CH2205	Male	Three y/o	Treatment (Wk 3)	Treatment	Feed Day	12:45	12:00 - 12:59	Afternoon	Inactive
2019/06/01	CH2206	Male	Three y/o	Treatment (Wk 3)	Treatment	Feed Day	12:45	12:00 - 12:59	Afternoon	Inactive
2019/06/01	CH2205	Male	Three y/o	Treatment (Wk 3)	Treatment	Feed Day	12:50	12:00 - 12:59	Afternoon	Appetitive behaviour
2019/06/01	CH2206	Male	Three y/o	Treatment (Wk 3)	Treatment	Feed Day	12:50	12:00 - 12:59	Afternoon	Appetitive behaviour
2019/06/01	CH2205	Male	Three y/o	Treatment (Wk 3)	Treatment	Feed Day	12:55	12:00 - 12:59	Afternoon	Appetitive behaviour
2019/06/01	CH2206	Male	Three y/o	Treatment (Wk 3)	Treatment	Feed Day	12:55	12:00 - 12:59	Afternoon	Appetitive behaviour
2019/06/01	CH2205	Male	Three y/o	Treatment (Wk 3)	Treatment	Feed Day	13:00	13:00 - 13:59	Afternoon	Appetitive behaviour
2019/06/01	CH2206	Male	Three y/o	Treatment (Wk 3)	Treatment	Feed Day	13:00	13:00 - 13:59	Afternoon	Appetitive behaviour
2019/06/01	CH2205	Male	Three y/o	Treatment (Wk 3)	Treatment	Feed Day	13:05	13:00 - 13:59	Afternoon	Locomotion
2019/06/01	CH2206	Male	Three y/o	Treatment (Wk 3)	Treatment	Feed Day	13:05	13:00 - 13:59	Afternoon	Appetitive behaviour
2019/06/01	CH2205	Male	Three y/o	Treatment (Wk 3)	Treatment	Feed Day	13:10	13:00 - 13:59	Afternoon	Appetitive behaviour
2019/06/01	CH2206	Male	Three y/o	Treatment (Wk 3)	Treatment	Feed Day	13:10	13:00 - 13:59	Afternoon	Appetitive behaviour
2019/06/01	CH2205	Male	Three y/o	Treatment (Wk 3)	Treatment	Feed Day	13:15	13:00 - 13:59	Afternoon	Appetitive behaviour
2019/06/01	CH2206	Male	Three y/o	Treatment (Wk 3)	Treatment	Feed Day	13:15	13:00 - 13:59	Afternoon	Appetitive behaviour
2019/06/01	CH2205	Male	Three y/o	Treatment (Wk 3)	Treatment	Feed Day	13:20	13:00 - 13:59	Afternoon	Appetitive behaviour
2019/06/01	CH2206	Male	Three y/o	Treatment (Wk 3)	Treatment	Feed Day	13:20	13:00 - 13:59	Afternoon	Appetitive behaviour
2019/06/01	CH2205	Male	Three y/o	Treatment (Wk 3)	Treatment	Feed Day	13:25	13:00 - 13:59	Afternoon	Appetitive behaviour
2019/06/01	CH2206	Male	Three y/o	Treatment (Wk 3)	Treatment	Feed Day	13:25	13:00 - 13:59	Afternoon	Appetitive behaviour
2019/06/01	CH2205	Male	Three y/o	Treatment (Wk 3)	Treatment	Feed Day	13:30	13:00 - 13:59	Afternoon	Attention
2019/06/01	CH2206	Male	Three y/o	Treatment (Wk 3)	Treatment	Feed Day	13:30	13:00 - 13:59	Afternoon	Appetitive behaviour
2019/06/01	CH2205	Male	Three y/o	Treatment (Wk 3)	Treatment	Feed Day	13:35	13:00 - 13:59	Afternoon	Appetitive behaviour

2019/06/01	CH2206	Male	Three y/o	Treatment (Wk 3)	Treatment	Feed Day	13:35	13:00 - 13:59	Afternoon	Appetitive behaviour
2019/06/01	CH2205	Male	Three y/o	Treatment (Wk 3)	Treatment	Feed Day	13:40	13:00 - 13:59	Afternoon	Appetitive behaviour
2019/06/01	CH2206	Male	Three y/o	Treatment (Wk 3)	Treatment	Feed Day	13:40	13:00 - 13:59	Afternoon	Appetitive behaviour
2019/06/02	CH2205	Male	Three y/o	Treatment (Wk 3)	Treatment	Fast Day	11:55	11:00 - 11:59	Late Morning	Appetitive behaviour
2019/06/02	CH2206	Male	Three y/o	Treatment (Wk 3)	Treatment	Fast Day	11:55	11:00 - 11:59	Late Morning	Appetitive behaviour
2019/06/02	CH2205	Male	Three y/o	Treatment (Wk 3)	Treatment	Fast Day	12:00	12:00 - 12:59	Afternoon	Locomotion
2019/06/02	CH2206	Male	Three y/o	Treatment (Wk 3)	Treatment	Fast Day	12:00	12:00 - 12:59	Afternoon	Scent-marking
2019/06/02	CH2205	Male	Three y/o	Treatment (Wk 3)	Treatment	Fast Day	12:05	12:00 - 12:59	Afternoon	Interspecific behaviour
2019/06/02	CH2206	Male	Three y/o	Treatment (Wk 3)	Treatment	Fast Day	12:05	12:00 - 12:59	Afternoon	Interspecific behaviour
2019/06/02	CH2205	Male	Three y/o	Treatment (Wk 3)	Treatment	Fast Day	12:10	12:00 - 12:59	Afternoon	Scent-marking
2019/06/02	CH2206	Male	Three y/o	Treatment (Wk 3)	Treatment	Fast Day	12:10	12:00 - 12:59	Afternoon	Interspecific behaviour
2019/06/02	CH2205	Male	Three y/o	Treatment (Wk 3)	Treatment	Fast Day	12:15	12:00 - 12:59	Afternoon	Maintenance
2019/06/02	CH2206	Male	Three y/o	Treatment (Wk 3)	Treatment	Fast Day	12:15	12:00 - 12:59	Afternoon	Interspecific behaviour
2019/06/02	CH2205	Male	Three y/o	Treatment (Wk 3)	Treatment	Fast Day	12:20	12:00 - 12:59	Afternoon	Inactive
2019/06/02	CH2206	Male	Three y/o	Treatment (Wk 3)	Treatment	Fast Day	12:20	12:00 - 12:59	Afternoon	Inactive
2019/06/02	CH2205	Male	Three y/o	Treatment (Wk 3)	Treatment	Fast Day	12:25	12:00 - 12:59	Afternoon	Inactive
2019/06/02	CH2206	Male	Three y/o	Treatment (Wk 3)	Treatment	Fast Day	12:25	12:00 - 12:59	Afternoon	Inactive
2019/06/02	CH2205	Male	Three y/o	Treatment (Wk 3)	Treatment	Fast Day	12:30	12:00 - 12:59	Afternoon	Inactive
2019/06/02	CH2206	Male	Three y/o	Treatment (Wk 3)	Treatment	Fast Day	12:30	12:00 - 12:59	Afternoon	Inactive
2019/06/02	CH2205	Male	Three y/o	Treatment (Wk 3)	Treatment	Fast Day	12:35	12:00 - 12:59	Afternoon	Inactive
2019/06/02	CH2206	Male	Three y/o	Treatment (Wk 3)	Treatment	Fast Day	12:35	12:00 - 12:59	Afternoon	Inactive
2019/06/02	CH2205	Male	Three y/o	Treatment (Wk 3)	Treatment	Fast Day	12:40	12:00 - 12:59	Afternoon	Inactive
2019/06/02	CH2206	Male	Three y/o	Treatment (Wk 3)	Treatment	Fast Day	12:40	12:00 - 12:59	Afternoon	Inactive
2019/06/02	CH2205	Male	Three y/o	Treatment (Wk 3)	Treatment	Fast Day	12:45	12:00 - 12:59	Afternoon	Inactive
2019/06/02	CH2206	Male	Three y/o	Treatment (Wk 3)	Treatment	Fast Day	12:45	12:00 - 12:59	Afternoon	Inactive
2019/06/02	CH2205	Male	Three y/o	Treatment (Wk 3)	Treatment	Fast Day	12:50	12:00 - 12:59	Afternoon	Inactive
2019/06/02	CH2206	Male	Three y/o	Treatment (Wk 3)	Treatment	Fast Day	12:50	12:00 - 12:59	Afternoon	Inactive
2019/06/03	CH2205	Male	Three y/o	Treatment (Wk 3)	Treatment	Feed Day	09:50	09:00 - 09:59	Late Morning	Scent-marking
2019/06/03	CH2206	Male	Three y/o	Treatment (Wk 3)	Treatment	Feed Day	09:50	09:00 - 09:59	Late Morning	Attention
2019/06/03	CH2205	Male	Three y/o	Treatment (Wk 3)	Treatment	Feed Day	09:55	09:00 - 09:59	Late Morning	Olfactory exploration
2019/06/03	CH2206	Male	Three y/o	Treatment (Wk 3)	Treatment	Feed Day	09:55	09:00 - 09:59	Late Morning	Locomotion
2019/06/03	CH2205	Male	Three y/o	Treatment (Wk 3)	Treatment	Feed Day	10:00	10:00 - 10:59	Late Morning	Scent-marking
2019/06/03	CH2206	Male	Three y/o	Treatment (Wk 3)	Treatment	Feed Day	10:00	10:00 - 10:59	Late Morning	Attention
2019/06/03	CH2205	Male	Three y/o	Treatment (Wk 3)	Treatment	Feed Day	10:05	10:00 - 10:59	Late Morning	Locomotion
2019/06/03	CH2206	Male	Three y/o	Treatment (Wk 3)	Treatment	Feed Day	10:05	10:00 - 10:59	Late Morning	Locomotion
2019/06/03	CH2205	Male	Three y/o	Treatment (Wk 3)	Treatment	Feed Day	10:10	10:00 - 10:59	Late Morning	Locomotion
2019/06/03	CH2206	Male	Three y/o	Treatment (Wk 3)	Treatment	Feed Day	10:10	10:00 - 10:59	Late Morning	Locomotion

2019/06/03	CH2205	Male	Three y/o	Treatment (Wk 3)	Treatment	Feed Day	10:15	10:00 - 10:59	Late Morning	Attention
2019/06/03	CH2206	Male	Three y/o	Treatment (Wk 3)	Treatment	Feed Day	10:15	10:00 - 10:59	Late Morning	Locomotion
2019/06/03	CH2205	Male	Three y/o	Treatment (Wk 3)	Treatment	Feed Day	10:20	10:00 - 10:59	Late Morning	Attention
2019/06/03	CH2206	Male	Three y/o	Treatment (Wk 3)	Treatment	Feed Day	10:20	10:00 - 10:59	Late Morning	Auto-grooming
2019/06/03	CH2205	Male	Three y/o	Treatment (Wk 3)	Treatment	Feed Day	10:25	10:00 - 10:59	Late Morning	Locomotion
2019/06/03	CH2206	Male	Three y/o	Treatment (Wk 3)	Treatment	Feed Day	10:25	10:00 - 10:59	Late Morning	Locomotion
2019/06/03	CH2205	Male	Three y/o	Treatment (Wk 3)	Treatment	Feed Day	10:30	10:00 - 10:59	Late Morning	Locomotion
2019/06/03	CH2206	Male	Three y/o	Treatment (Wk 3)	Treatment	Feed Day	10:30	10:00 - 10:59	Late Morning	Locomotion
2019/06/03	CH2205	Male	Three y/o	Treatment (Wk 3)	Treatment	Feed Day	10:35	10:00 - 10:59	Late Morning	Inactive
2019/06/03	CH2206	Male	Three y/o	Treatment (Wk 3)	Treatment	Feed Day	10:35	10:00 - 10:59	Late Morning	Inactive
2019/06/03	CH2205	Male	Three y/o	Treatment (Wk 3)	Treatment	Feed Day	10:40	10:00 - 10:59	Late Morning	Inactive
2019/06/03	CH2206	Male	Three y/o	Treatment (Wk 3)	Treatment	Feed Day	10:40	10:00 - 10:59	Late Morning	Inactive
2019/06/03	CH2205	Male	Three y/o	Treatment (Wk 3)	Treatment	Feed Day	10:45	10:00 - 10:59	Late Morning	Inactive
2019/06/03	CH2206	Male	Three y/o	Treatment (Wk 3)	Treatment	Feed Day	10:45	10:00 - 10:59	Late Morning	Inactive
2019/06/04	CH2205	Male	Three y/o	Treatment (Wk 3)	Treatment	Feed Day	09:00	09:00 - 09:59	Late Morning	Olfactory exploration
2019/06/04	CH2206	Male	Three y/o	Treatment (Wk 3)	Treatment	Feed Day	09:00	09:00 - 09:59	Late Morning	Locomotion
2019/06/04	CH2205	Male	Three y/o	Treatment (Wk 3)	Treatment	Feed Day	09:05	09:00 - 09:59	Late Morning	Locomotion
2019/06/04	CH2206	Male	Three y/o	Treatment (Wk 3)	Treatment	Feed Day	09:05	09:00 - 09:59	Late Morning	Locomotion
2019/06/04	CH2205	Male	Three y/o	Treatment (Wk 3)	Treatment	Feed Day	09:10	09:00 - 09:59	Late Morning	Locomotion
2019/06/04	CH2206	Male	Three y/o	Treatment (Wk 3)	Treatment	Feed Day	09:10	09:00 - 09:59	Late Morning	Locomotion
2019/06/04	CH2205	Male	Three y/o	Treatment (Wk 3)	Treatment	Feed Day	09:15	09:00 - 09:59	Late Morning	Scent-marking
2019/06/04	CH2206	Male	Three y/o	Treatment (Wk 3)	Treatment	Feed Day	09:15	09:00 - 09:59	Late Morning	Locomotion
2019/06/04	CH2205	Male	Three y/o	Treatment (Wk 3)	Treatment	Feed Day	09:20	09:00 - 09:59	Late Morning	Appetitive behaviour
2019/06/04	CH2206	Male	Three y/o	Treatment (Wk 3)	Treatment	Feed Day	09:20	09:00 - 09:59	Late Morning	Locomotion
2019/06/04	CH2205	Male	Three y/o	Treatment (Wk 3)	Treatment	Feed Day	09:25	09:00 - 09:59	Late Morning	Locomotion
2019/06/04	CH2206	Male	Three y/o	Treatment (Wk 3)	Treatment	Feed Day	09:25	09:00 - 09:59	Late Morning	Locomotion
2019/06/04	CH2205	Male	Three y/o	Treatment (Wk 3)	Treatment	Feed Day	09:30	09:00 - 09:59	Late Morning	Inactive
2019/06/04	CH2206	Male	Three y/o	Treatment (Wk 3)	Treatment	Feed Day	09:30	09:00 - 09:59	Late Morning	Attention
2019/06/04	CH2205	Male	Three y/o	Treatment (Wk 3)	Treatment	Feed Day	09:35	09:00 - 09:59	Late Morning	Attention
2019/06/04	CH2206	Male	Three y/o	Treatment (Wk 3)	Treatment	Feed Day	09:35	09:00 - 09:59	Late Morning	Locomotion
2019/06/04	CH2205	Male	Three y/o	Treatment (Wk 3)	Treatment	Feed Day	09:40	09:00 - 09:59	Late Morning	Interspecific behaviour
2019/06/04	CH2206	Male	Three y/o	Treatment (Wk 3)	Treatment	Feed Day	09:40	09:00 - 09:59	Late Morning	Locomotion
2019/06/04	CH2205	Male	Three y/o	Treatment (Wk 3)	Treatment	Feed Day	09:45	09:00 - 09:59	Late Morning	Inactive
2019/06/04	CH2206	Male	Three y/o	Treatment (Wk 3)	Treatment	Feed Day	09:45	09:00 - 09:59	Late Morning	Inactive
2019/06/04	CH2205	Male	Three y/o	Treatment (Wk 3)	Treatment	Feed Day	09:50	09:00 - 09:59	Late Morning	Interspecific behaviour
2019/06/04	CH2206	Male	Three y/o	Treatment (Wk 3)	Treatment	Feed Day	09:50	09:00 - 09:59	Late Morning	Inactive
2019/06/04	CH2205	Male	Three y/o	Treatment (Wk 3)	Treatment	Feed Day	09:55	09:00 - 09:59	Late Morning	Interspecific behaviour

2019/06/04	CH2206	Male	Three y/o	Treatment (Wk 3)	Treatment	Feed Day	09:55	09:00 - 09:59	Late Morning	Inactive
2019/06/06	CH2207	Female	Three y/o	Control	Control	Feed Day	09:50	09:00 - 09:59	Late Morning	Appetitive behaviour
2019/06/06	CH2207	Female	Three y/o	Control	Control	Feed Day	09:55	09:00 - 09:59	Late Morning	Appetitive behaviour
2019/06/06	CH2207	Female	Three y/o	Control	Control	Feed Day	10:00	10:00 - 10:59	Late Morning	Locomotion
2019/06/06	CH2207	Female	Three y/o	Control	Control	Feed Day	10:05	10:00 - 10:59	Late Morning	Out of sight
2019/06/06	CH2207	Female	Three y/o	Control	Control	Feed Day	10:10	10:00 - 10:59	Late Morning	Out of sight
2019/06/06	CH2207	Female	Three y/o	Control	Control	Feed Day	10:15	10:00 - 10:59	Late Morning	Out of sight
2019/06/06	CH2207	Female	Three y/o	Control	Control	Feed Day	10:20	10:00 - 10:59	Late Morning	Out of sight
2019/06/06	CH2207	Female	Three y/o	Control	Control	Feed Day	10:25	10:00 - 10:59	Late Morning	Out of sight
2019/06/06	CH2207	Female	Three y/o	Control	Control	Feed Day	10:30	10:00 - 10:59	Late Morning	Out of sight
2019/06/06	CH2207	Female	Three y/o	Control	Control	Feed Day	10:35	10:00 - 10:59	Late Morning	Out of sight
2019/06/06	CH2207	Female	Three y/o	Control	Control	Feed Day	10:40	10:00 - 10:59	Late Morning	Out of sight
2019/06/06	CH2207	Female	Three y/o	Control	Control	Feed Day	10:45	10:00 - 10:59	Late Morning	Out of sight
2019/06/06	CH2205	Male	Three y/o	Control	Control	Feed Day	10:45	10:00 - 10:59	Late Morning	Attention
2019/06/06	CH2206	Male	Three y/o	Control	Control	Feed Day	10:45	10:00 - 10:59	Late Morning	Appetitive behaviour
2019/06/06	CH2205	Male	Three y/o	Control	Control	Feed Day	10:50	10:00 - 10:59	Late Morning	Attention
2019/06/06	CH2206	Male	Three y/o	Control	Control	Feed Day	10:50	10:00 - 10:59	Late Morning	Appetitive behaviour
2019/06/06	CH2205	Male	Three y/o	Control	Control	Feed Day	10:55	10:00 - 10:59	Late Morning	Appetitive behaviour
2019/06/06	CH2206	Male	Three y/o	Control	Control	Feed Day	10:55	10:00 - 10:59	Late Morning	Appetitive behaviour
2019/06/06	CH2205	Male	Three y/o	Control	Control	Feed Day	11:00	11:00 - 11:59	Late Morning	Inactive
2019/06/06	CH2206	Male	Three y/o	Control	Control	Feed Day	11:00	11:00 - 11:59	Late Morning	Appetitive behaviour
2019/06/06	CH2205	Male	Three y/o	Control	Control	Feed Day	11:05	11:00 - 11:59	Late Morning	Attention
2019/06/06	CH2206	Male	Three y/o	Control	Control	Feed Day	11:05	11:00 - 11:59	Late Morning	Attention
2019/06/06	CH2205	Male	Three y/o	Control	Control	Feed Day	11:10	11:00 - 11:59	Late Morning	Attention
2019/06/06	CH2206	Male	Three y/o	Control	Control	Feed Day	11:10	11:00 - 11:59	Late Morning	Appetitive behaviour
2019/06/06	CH2205	Male	Three y/o	Control	Control	Feed Day	11:15	11:00 - 11:59	Late Morning	Inactive
2019/06/06	CH2206	Male	Three y/o	Control	Control	Feed Day	11:15	11:00 - 11:59	Late Morning	Appetitive behaviour
2019/06/06	CH2205	Male	Three y/o	Control	Control	Feed Day	11:20	11:00 - 11:59	Late Morning	Inactive
2019/06/06	CH2206	Male	Three y/o	Control	Control	Feed Day	11:20	11:00 - 11:59	Late Morning	Appetitive behaviour
2019/06/06	CH2205	Male	Three y/o	Control	Control	Feed Day	11:25	11:00 - 11:59	Late Morning	Attention
2019/06/06	CH2206	Male	Three y/o	Control	Control	Feed Day	11:25	11:00 - 11:59	Late Morning	Appetitive behaviour
2019/06/06	CH2205	Male	Three y/o	Control	Control	Feed Day	11:30	11:00 - 11:59	Late Morning	Appetitive behaviour
2019/06/06	CH2206	Male	Three y/o	Control	Control	Feed Day	11:30	11:00 - 11:59	Late Morning	Inactive
2019/06/06	CH2205	Male	Three y/o	Control	Control	Feed Day	11:35	11:00 - 11:59	Late Morning	Appetitive behaviour
2019/06/06	CH2206	Male	Three y/o	Control	Control	Feed Day	11:35	11:00 - 11:59	Late Morning	Inactive
2019/06/06	CH2205	Male	Three y/o	Control	Control	Feed Day	11:40	11:00 - 11:59	Late Morning	Appetitive behaviour
2019/06/06	CH2206	Male	Three y/o	Control	Control	Feed Day	11:40	11:00 - 11:59	Late Morning	Inactive

2019/06/07	CH2205	Male	Three y/o	Control	Control	Feed Day	09:05	09:00 - 09:59	Late Morning	Scent-marking
2019/06/07	CH2206	Male	Three y/o	Control	Control	Feed Day	09:05	09:00 - 09:59	Late Morning	Attention
2019/06/07	CH2205	Male	Three y/o	Control	Control	Feed Day	09:10	09:00 - 09:59	Late Morning	Interspecific behaviour
2019/06/07	CH2206	Male	Three y/o	Control	Control	Feed Day	09:10	09:00 - 09:59	Late Morning	Auto-grooming
2019/06/07	CH2205	Male	Three y/o	Control	Control	Feed Day	09:15	09:00 - 09:59	Late Morning	Affiliative behaviour
2019/06/07	CH2206	Male	Three y/o	Control	Control	Feed Day	09:15	09:00 - 09:59	Late Morning	Affiliative behaviour
2019/06/07	CH2205	Male	Three y/o	Control	Control	Feed Day	09:20	09:00 - 09:59	Late Morning	Interspecific behaviour
2019/06/07	CH2206	Male	Three y/o	Control	Control	Feed Day	09:20	09:00 - 09:59	Late Morning	Interspecific behaviour
2019/06/07	CH2205	Male	Three y/o	Control	Control	Feed Day	09:25	09:00 - 09:59	Late Morning	Interspecific behaviour
2019/06/07	CH2206	Male	Three y/o	Control	Control	Feed Day	09:25	09:00 - 09:59	Late Morning	Interspecific behaviour
2019/06/07	CH2205	Male	Three y/o	Control	Control	Feed Day	09:30	09:00 - 09:59	Late Morning	Interspecific behaviour
2019/06/07	CH2206	Male	Three y/o	Control	Control	Feed Day	09:30	09:00 - 09:59	Late Morning	Olfactory exploration
2019/06/07	CH2205	Male	Three y/o	Control	Control	Feed Day	09:35	09:00 - 09:59	Late Morning	Scent-marking
2019/06/07	CH2206	Male	Three y/o	Control	Control	Feed Day	09:35	09:00 - 09:59	Late Morning	Locomotion
2019/06/07	CH2205	Male	Three y/o	Control	Control	Feed Day	09:40	09:00 - 09:59	Late Morning	Out of sight
2019/06/07	CH2206	Male	Three y/o	Control	Control	Feed Day	09:40	09:00 - 09:59	Late Morning	Out of sight
2019/06/07	CH2205	Male	Three y/o	Control	Control	Feed Day	09:45	09:00 - 09:59	Late Morning	Out of sight
2019/06/07	CH2206	Male	Three y/o	Control	Control	Feed Day	09:45	09:00 - 09:59	Late Morning	Out of sight
2019/06/07	CH2205	Male	Three y/o	Control	Control	Feed Day	09:50	09:00 - 09:59	Late Morning	Out of sight
2019/06/07	CH2206	Male	Three y/o	Control	Control	Feed Day	09:50	09:00 - 09:59	Late Morning	Out of sight
2019/06/07	CH2205	Male	Three y/o	Control	Control	Feed Day	09:55	09:00 - 09:59	Late Morning	Out of sight
2019/06/07	CH2206	Male	Three y/o	Control	Control	Feed Day	09:55	09:00 - 09:59	Late Morning	Out of sight
2019/06/07	CH2205	Male	Three y/o	Control	Control	Feed Day	10:00	10:00-10:59	Late Morning	Out of sight
2019/06/07	CH2206	Male	Three y/o	Control	Control	Feed Day	10:00	10:00-10:59	Late Morning	Out of sight
2019/06/07	CH2207	Female	Three y/o	Control	Control	Feed Day	10:00	10:00 - 10:59	Late Morning	Inactive
2019/06/07	CH2207	Female	Three y/o	Control	Control	Feed Day	10:05	10:00 - 10:59	Late Morning	Inactive
2019/06/07	CH2207	Female	Three y/o	Control	Control	Feed Day	10:10	10:00 - 10:59	Late Morning	Inactive
2019/06/07	CH2207	Female	Three y/o	Control	Control	Feed Day	10:15	10:00 - 10:59	Late Morning	Inactive
2019/06/07	CH2207	Female	Three y/o	Control	Control	Feed Day	10:20	10:00 - 10:59	Late Morning	Attention
2019/06/07	CH2207	Female	Three y/o	Control	Control	Feed Day	10:25	10:00 - 10:59	Late Morning	Inactive
2019/06/07	CH2207	Female	Three y/o	Control	Control	Feed Day	10:30	10:00 - 10:59	Late Morning	Attention
2019/06/07	CH2207	Female	Three y/o	Control	Control	Feed Day	10:35	10:00 - 10:59	Late Morning	Inactive
2019/06/07	CH2207	Female	Three y/o	Control	Control	Feed Day	10:40	10:00 - 10:59	Late Morning	Inactive
2019/06/07	CH2207	Female	Three y/o	Control	Control	Feed Day	10:45	10:00 - 10:59	Late Morning	Inactive
2019/06/07	CH2207	Female	Three y/o	Control	Control	Feed Day	10:50	10:00 - 10:59	Late Morning	Inactive
2019/06/07	CH2207	Female	Three y/o	Control	Control	Feed Day	10:55	10:00 - 10:59	Late Morning	Appetitive behaviour
2019/06/08	CH2207	Female	Three y/o	Control	Control	Feed Day	08:55	08:00 - 08:59	Late Morning	Attention

2019/06/08	CH2207	Female	Three y/o	Control	Control	Feed Day	09:00	09:00 - 09:59	Late Morning	Attention
2019/06/08	CH2207	Female	Three y/o	Control	Control	Feed Day	09:05	09:00 - 09:59	Late Morning	Attention
2019/06/08	CH2207	Female	Three y/o	Control	Control	Feed Day	09:10	09:00 - 09:59	Late Morning	Inactive
2019/06/08	CH2207	Female	Three y/o	Control	Control	Feed Day	09:15	09:00 - 09:59	Late Morning	Auto-grooming
2019/06/08	CH2207	Female	Three y/o	Control	Control	Feed Day	09:20	09:00 - 09:59	Late Morning	Attention
2019/06/08	CH2207	Female	Three y/o	Control	Control	Feed Day	09:25	09:00 - 09:59	Late Morning	Attention
2019/06/08	CH2207	Female	Three y/o	Control	Control	Feed Day	09:30	09:00 - 09:59	Late Morning	Attention
2019/06/08	CH2207	Female	Three y/o	Control	Control	Feed Day	09:35	09:00 - 09:59	Late Morning	Attention
2019/06/08	CH2207	Female	Three y/o	Control	Control	Feed Day	09:40	09:00 - 09:59	Late Morning	Scent-marking
2019/06/08	CH2207	Female	Three y/o	Control	Control	Feed Day	09:45	09:00 - 09:59	Late Morning	Locomotion
2019/06/08	CH2205	Male	Three y/o	Control	Control	Feed Day	09:50	09:00 - 09:59	Late Morning	Scent-marking
2019/06/08	CH2206	Male	Three y/o	Control	Control	Feed Day	09:50	09:00 - 09:59	Late Morning	Attention
2019/06/08	CH2207	Female	Three y/o	Control	Control	Feed Day	09:50	09:00 - 09:59	Late Morning	Locomotion
2019/06/08	CH2205	Male	Three y/o	Control	Control	Feed Day	09:55	09:00 - 09:59	Late Morning	Attention
2019/06/08	CH2206	Male	Three y/o	Control	Control	Feed Day	09:55	09:00 - 09:59	Late Morning	Locomotion
2019/06/08	CH2205	Male	Three y/o	Control	Control	Feed Day	10:00	10:00 - 10:59	Late Morning	Inactive
2019/06/08	CH2206	Male	Three y/o	Control	Control	Feed Day	10:00	10:00 - 10:59	Late Morning	Locomotion
2019/06/08	CH2205	Male	Three y/o	Control	Control	Feed Day	10:05	10:00 - 10:59	Late Morning	Inactive
2019/06/08	CH2206	Male	Three y/o	Control	Control	Feed Day	10:05	10:00 - 10:59	Late Morning	Auto-grooming
2019/06/08	CH2205	Male	Three y/o	Control	Control	Feed Day	10:10	10:00 - 10:59	Late Morning	Affiliative behaviour
2019/06/08	CH2206	Male	Three y/o	Control	Control	Feed Day	10:10	10:00 - 10:59	Late Morning	Affiliative behaviour
2019/06/08	CH2205	Male	Three y/o	Control	Control	Feed Day	10:15	10:00 - 10:59	Late Morning	Locomotion
2019/06/08	CH2206	Male	Three y/o	Control	Control	Feed Day	10:15	10:00 - 10:59	Late Morning	Attention
2019/06/08	CH2205	Male	Three y/o	Control	Control	Feed Day	10:20	10:00 - 10:59	Late Morning	Locomotion
2019/06/08	CH2206	Male	Three y/o	Control	Control	Feed Day	10:20	10:00 - 10:59	Late Morning	Inactive
2019/06/08	CH2205	Male	Three y/o	Control	Control	Feed Day	10:25	10:00 - 10:59	Late Morning	Inactive
2019/06/08	CH2206	Male	Three y/o	Control	Control	Feed Day	10:25	10:00 - 10:59	Late Morning	Inactive
2019/06/08	CH2205	Male	Three y/o	Control	Control	Feed Day	10:30	10:00 - 10:59	Late Morning	Inactive
2019/06/08	CH2206	Male	Three y/o	Control	Control	Feed Day	10:30	10:00 - 10:59	Late Morning	Inactive
2019/06/08	CH2205	Male	Three y/o	Control	Control	Feed Day	10:35	10:00 - 10:59	Late Morning	Inactive
2019/06/08	CH2206	Male	Three y/o	Control	Control	Feed Day	10:35	10:00 - 10:59	Late Morning	Inactive
2019/06/08	CH2205	Male	Three y/o	Control	Control	Feed Day	10:40	10:00 - 10:59	Late Morning	Inactive
2019/06/08	CH2206	Male	Three y/o	Control	Control	Feed Day	10:40	10:00 - 10:59	Late Morning	Inactive
2019/06/08	CH2205	Male	Three y/o	Control	Control	Feed Day	10:45	10:00 - 10:59	Late Morning	Inactive
2019/06/08	CH2206	Male	Three y/o	Control	Control	Feed Day	10:45	10:00 - 10:59	Late Morning	Inactive
2019/06/11	CH2207	Female	Three y/o	Control	Control	Feed Day	08:35	08:00 - 08:59	Late Morning	Appetitive behaviour
2019/06/11	CH2207	Female	Three y/o	Control	Control	Feed Day	08:40	08:00 - 08:59	Late Morning	Appetitive behaviour

2019/06/11	CH2207	Female	Three y/o	Control	Control	Feed Day	08:45	08:00 - 08:59	Late Morning	Appetitive behaviour
2019/06/11	CH2207	Female	Three y/o	Control	Control	Feed Day	08:50	08:00 - 08:59	Late Morning	Locomotion
2019/06/11	CH2207	Female	Three y/o	Control	Control	Feed Day	08:55	08:00 - 08:59	Late Morning	Locomotion
2019/06/11	CH2207	Female	Three y/o	Control	Control	Feed Day	09:00	09:00 - 09:59	Late Morning	Locomotion
2019/06/11	CH2207	Female	Three y/o	Control	Control	Feed Day	09:05	09:00 - 09:59	Late Morning	Locomotion
2019/06/11	CH2207	Female	Three y/o	Control	Control	Feed Day	09:10	09:00 - 09:59	Late Morning	Locomotion
2019/06/11	CH2207	Female	Three y/o	Control	Control	Feed Day	09:15	09:00 - 09:59	Late Morning	Appetitive behaviour
2019/06/11	CH2207	Female	Three y/o	Control	Control	Feed Day	09:20	09:00 - 09:59	Late Morning	Appetitive behaviour
2019/06/11	CH2207	Female	Three y/o	Control	Control	Feed Day	09:25	09:00 - 09:59	Late Morning	Inactive
2019/06/11	CH2205	Male	Three y/o	Control	Control	Feed Day	09:25	09:00 - 09:59	Late Morning	Appetitive behaviour
2019/06/11	CH2206	Male	Three y/o	Control	Control	Feed Day	09:25	09:00 - 09:59	Late Morning	Appetitive behaviour
2019/06/11	CH2207	Female	Three y/o	Control	Control	Feed Day	09:30	09:00 - 09:59	Late Morning	Inactive
2019/06/11	CH2205	Male	Three y/o	Control	Control	Feed Day	09:30	09:00 - 09:59	Late Morning	Vocalisation
2019/06/11	CH2206	Male	Three y/o	Control	Control	Feed Day	09:30	09:00 - 09:59	Late Morning	Appetitive behaviour
2019/06/11	CH2205	Male	Three y/o	Control	Control	Feed Day	09:35	09:00 - 09:59	Late Morning	Inactive
2019/06/11	CH2206	Male	Three y/o	Control	Control	Feed Day	09:35	09:00 - 09:59	Late Morning	Locomotion
2019/06/11	CH2205	Male	Three y/o	Control	Control	Feed Day	09:40	09:00 - 09:59	Late Morning	Locomotion
2019/06/11	CH2206	Male	Three y/o	Control	Control	Feed Day	09:40	09:00 - 09:59	Late Morning	Locomotion
2019/06/11	CH2205	Male	Three y/o	Control	Control	Feed Day	09:45	09:00 - 09:59	Late Morning	Locomotion
2019/06/11	CH2206	Male	Three y/o	Control	Control	Feed Day	09:45	09:00 - 09:59	Late Morning	Interspecific behaviour
2019/06/11	CH2205	Male	Three y/o	Control	Control	Feed Day	09:50	09:00 - 09:59	Late Morning	Inactive
2019/06/11	CH2206	Male	Three y/o	Control	Control	Feed Day	09:50	09:00 - 09:59	Late Morning	Inactive
2019/06/11	CH2205	Male	Three y/o	Control	Control	Feed Day	09:55	09:00 - 09:59	Late Morning	Inactive
2019/06/11	CH2206	Male	Three y/o	Control	Control	Feed Day	09:55	09:00 - 09:59	Late Morning	Inactive
2019/06/11	CH2205	Male	Three y/o	Control	Control	Feed Day	10:00	10:00 - 10:59	Late Morning	Inactive
2019/06/11	CH2206	Male	Three y/o	Control	Control	Feed Day	10:00	10:00 - 10:59	Late Morning	Inactive
2019/06/11	CH2205	Male	Three y/o	Control	Control	Feed Day	10:05	10:00 - 10:59	Late Morning	Affiliative behaviour
2019/06/11	CH2206	Male	Three y/o	Control	Control	Feed Day	10:05	10:00 - 10:59	Late Morning	Affiliative behaviour
2019/06/11	CH2205	Male	Three y/o	Control	Control	Feed Day	10:10	10:00 - 10:59	Late Morning	Interspecific behaviour
2019/06/11	CH2206	Male	Three y/o	Control	Control	Feed Day	10:10	10:00 - 10:59	Late Morning	Scent-marking
2019/06/11	CH2205	Male	Three y/o	Control	Control	Feed Day	10:15	10:00 - 10:59	Late Morning	Locomotion
2019/06/11	CH2206	Male	Three y/o	Control	Control	Feed Day	10:15	10:00 - 10:59	Late Morning	Locomotion
2019/06/11	CH2205	Male	Three y/o	Control	Control	Feed Day	10:20	10:00 - 10:59	Late Morning	Inactive
2019/06/11	CH2206	Male	Three y/o	Control	Control	Feed Day	10:20	10:00 - 10:59	Late Morning	Inactive
2019/06/12	CH2205	Male	Three y/o	Control	Control	Feed Day	08:45	08:00 - 08:59	Late Morning	Locomotion
2019/06/12	CH2206	Male	Three y/o	Control	Control	Feed Day	08:45	08:00 - 08:59	Late Morning	Locomotion
2019/06/12	CH2205	Male	Three y/o	Control	Control	Feed Day	08:50	08:00 - 08:59	Late Morning	Locomotion

2019/06/12	CH2206	Male	Three y/o	Control	Control	Feed Day	08:50	08:00 - 08:59	Late Morning	Olfactory exploration
2019/06/12	CH2205	Male	Three y/o	Control	Control	Feed Day	08:55	08:00 - 08:59	Late Morning	Locomotion
2019/06/12	CH2206	Male	Three y/o	Control	Control	Feed Day	08:55	08:00 - 08:59	Late Morning	Interspecific behaviour
2019/06/12	CH2205	Male	Three y/o	Control	Control	Feed Day	09:00	09:00 - 09:59	Late Morning	Attention
2019/06/12	CH2206	Male	Three y/o	Control	Control	Feed Day	09:00	09:00 - 09:59	Late Morning	Locomotion
2019/06/12	CH2205	Male	Three y/o	Control	Control	Feed Day	09:05	09:00 - 09:59	Late Morning	Scent-marking
2019/06/12	CH2206	Male	Three y/o	Control	Control	Feed Day	09:05	09:00 - 09:59	Late Morning	Interspecific behaviour
2019/06/12	CH2205	Male	Three y/o	Control	Control	Feed Day	09:10	09:00 - 09:59	Late Morning	Locomotion
2019/06/12	CH2206	Male	Three y/o	Control	Control	Feed Day	09:10	09:00 - 09:59	Late Morning	Interspecific behaviour
2019/06/12	CH2205	Male	Three y/o	Control	Control	Feed Day	09:15	09:00 - 09:59	Late Morning	Attention
2019/06/12	CH2206	Male	Three y/o	Control	Control	Feed Day	09:15	09:00 - 09:59	Late Morning	Inactive
2019/06/12	CH2205	Male	Three y/o	Control	Control	Feed Day	09:20	09:00 - 09:59	Late Morning	Inactive
2019/06/12	CH2206	Male	Three y/o	Control	Control	Feed Day	09:20	09:00 - 09:59	Late Morning	Inactive
2019/06/12	CH2205	Male	Three y/o	Control	Control	Feed Day	09:25	09:00 - 09:59	Late Morning	Attention
2019/06/12	CH2206	Male	Three y/o	Control	Control	Feed Day	09:25	09:00 - 09:59	Late Morning	Attention
2019/06/12	CH2205	Male	Three y/o	Control	Control	Feed Day	09:30	09:00 - 09:59	Late Morning	Scent-marking
2019/06/12	CH2206	Male	Three y/o	Control	Control	Feed Day	09:30	09:00 - 09:59	Late Morning	Locomotion
2019/06/12	CH2205	Male	Three y/o	Control	Control	Feed Day	09:35	09:00 - 09:59	Late Morning	Locomotion
2019/06/12	CH2206	Male	Three y/o	Control	Control	Feed Day	09:35	09:00 - 09:59	Late Morning	Interspecific behaviour
2019/06/12	CH2205	Male	Three y/o	Control	Control	Feed Day	09:40	09:00 - 09:59	Late Morning	Out of sight
2019/06/12	CH2206	Male	Three y/o	Control	Control	Feed Day	09:40	09:00 - 09:59	Late Morning	Inactive
2019/06/12	CH2207	Female	Three y/o	Control	Control	Feed Day	09:45	09:00 - 09:59	Late Morning	Locomotion
2019/06/12	CH2207	Female	Three y/o	Control	Control	Feed Day	09:50	09:00 - 09:59	Late Morning	Auto-grooming
2019/06/12	CH2207	Female	Three y/o	Control	Control	Feed Day	09:55	09:00 - 09:59	Late Morning	Attention
2019/06/12	CH2207	Female	Three y/o	Control	Control	Feed Day	10:00	10:00 - 10:59	Late Morning	Inactive
2019/06/12	CH2207	Female	Three y/o	Control	Control	Feed Day	10:05	10:00 - 10:59	Late Morning	Scent-marking
2019/06/12	CH2207	Female	Three y/o	Control	Control	Feed Day	10:10	10:00 - 10:59	Late Morning	Attention
2019/06/12	CH2207	Female	Three y/o	Control	Control	Feed Day	10:15	10:00 - 10:59	Late Morning	Attention
2019/06/12	CH2207	Female	Three y/o	Control	Control	Feed Day	10:20	10:00 - 10:59	Late Morning	Attention
2019/06/12	CH2207	Female	Three y/o	Control	Control	Feed Day	10:25	10:00 - 10:59	Late Morning	Attention
2019/06/12	CH2207	Female	Three y/o	Control	Control	Feed Day	10:30	10:00 - 10:59	Late Morning	Attention
2019/06/12	CH2207	Female	Three y/o	Control	Control	Feed Day	10:35	10:00 - 10:59	Late Morning	Attention
2019/06/12	CH2207	Female	Three y/o	Control	Control	Feed Day	10:40	10:00 - 10:59	Late Morning	Appetitive behaviour
2019/06/13	CH2207	Female	Three y/o	Control	Control	Feed Day	09:25	09:00 - 09:59	Late Morning	Inactive
2019/06/13	CH2207	Female	Three y/o	Control	Control	Feed Day	09:30	09:00 - 09:59	Late Morning	Locomotion
2019/06/13	CH2207	Female	Three y/o	Control	Control	Feed Day	09:35	09:00 - 09:59	Late Morning	Appetitive behaviour
2019/06/13	CH2207	Female	Three y/o	Control	Control	Feed Day	09:40	09:00 - 09:59	Late Morning	Affiliative behaviour

2019/06/13	CH2207	Female	Three y/o	Control	Control	Feed Day	09:45	09:00 - 09:59	Late Morning	Agnostic behaviour
2019/06/13	CH2207	Female	Three y/o	Control	Control	Feed Day	09:50	09:00 - 09:59	Late Morning	Inactive
2019/06/13	CH2207	Female	Three y/o	Control	Control	Feed Day	09:55	09:00 - 09:59	Late Morning	Inactive
2019/06/13	CH2207	Female	Three y/o	Control	Control	Feed Day	10:00	10:00 - 10:59	Late Morning	Auto-grooming
2019/06/13	CH2207	Female	Three y/o	Control	Control	Feed Day	10:05	10:00 - 10:59	Late Morning	Auto-grooming
2019/06/13	CH2207	Female	Three y/o	Control	Control	Feed Day	10:10	10:00 - 10:59	Late Morning	Inactive
2019/06/13	CH2207	Female	Three y/o	Control	Control	Feed Day	10:15	10:00 - 10:59	Late Morning	Attention
2019/06/13	CH2207	Female	Three y/o	Control	Control	Feed Day	10:20	10:00 - 10:59	Late Morning	Inactive
2019/06/13	CH2205	Male	Three y/o	Control	Control	Feed Day	10:20	10:00 - 10:59	Late Morning	Attention
2019/06/13	CH2206	Male	Three y/o	Control	Control	Feed Day	10:20	10:00 - 10:59	Late Morning	Attention
2019/06/13	CH2205	Male	Three y/o	Control	Control	Feed Day	10:25	10:00 - 10:59	Late Morning	Scent-marking
2019/06/13	CH2206	Male	Three y/o	Control	Control	Feed Day	10:25	10:00 - 10:59	Late Morning	Inactive
2019/06/13	CH2205	Male	Three y/o	Control	Control	Feed Day	10:30	10:00 - 10:59	Late Morning	Inactive
2019/06/13	CH2206	Male	Three y/o	Control	Control	Feed Day	10:30	10:00 - 10:59	Late Morning	Inactive
2019/06/13	CH2205	Male	Three y/o	Control	Control	Feed Day	10:35	10:00 - 10:59	Late Morning	Inactive
2019/06/13	CH2206	Male	Three y/o	Control	Control	Feed Day	10:35	10:00 - 10:59	Late Morning	Inactive
2019/06/13	CH2205	Male	Three y/o	Control	Control	Feed Day	10:40	10:00 - 10:59	Late Morning	Attention
2019/06/13	CH2206	Male	Three y/o	Control	Control	Feed Day	10:40	10:00 - 10:59	Late Morning	Attention
2019/06/13	CH2205	Male	Three y/o	Control	Control	Feed Day	10:45	10:00 - 10:59	Late Morning	Locomotion
2019/06/13	CH2206	Male	Three y/o	Control	Control	Feed Day	10:45	10:00 - 10:59	Late Morning	Inactive
2019/06/13	CH2205	Male	Three y/o	Control	Control	Feed Day	10:50	10:00 - 10:59	Late Morning	Locomotion
2019/06/13	CH2206	Male	Three y/o	Control	Control	Feed Day	10:50	10:00 - 10:59	Late Morning	Locomotion
2019/06/13	CH2205	Male	Three y/o	Control	Control	Feed Day	10:55	10:00 - 10:59	Late Morning	Locomotion
2019/06/13	CH2206	Male	Three y/o	Control	Control	Feed Day	10:55	10:00 - 10:59	Late Morning	Locomotion
2019/06/13	CH2205	Male	Three y/o	Control	Control	Feed Day	11:00	11:00 - 11:59	Late Morning	Locomotion
2019/06/13	CH2206	Male	Three y/o	Control	Control	Feed Day	11:00	11:00 - 11:59	Late Morning	Locomotion
2019/06/13	CH2205	Male	Three y/o	Control	Control	Feed Day	11:05	11:00 - 11:59	Late Morning	Scent-marking
2019/06/13	CH2206	Male	Three y/o	Control	Control	Feed Day	11:05	11:00 - 11:59	Late Morning	Locomotion
2019/06/13	CH2205	Male	Three y/o	Control	Control	Feed Day	11:10	11:00 - 11:59	Late Morning	Attention
2019/06/13	CH2206	Male	Three y/o	Control	Control	Feed Day	11:10	11:00 - 11:59	Late Morning	Attention
2019/06/13	CH2205	Male	Three y/o	Control	Control	Feed Day	11:15	11:00 - 11:59	Late Morning	Attention
2019/06/13	CH2206	Male	Three y/o	Control	Control	Feed Day	11:15	11:00 - 11:59	Late Morning	Locomotion
2019/06/14	CH2205	Male	Three y/o	Control	Control	Feed Day	08:55	08:00 - 08:59	Late Morning	Locomotion
2019/06/14	CH2206	Male	Three y/o	Control	Control	Feed Day	08:55	08:00 - 08:59	Late Morning	Locomotion
2019/06/14	CH2205	Male	Three y/o	Control	Control	Feed Day	09:00	09:00 - 09:59	Late Morning	Locomotion
2019/06/14	CH2206	Male	Three y/o	Control	Control	Feed Day	09:00	09:00 - 09:59	Late Morning	Locomotion
2019/06/14	CH2205	Male	Three y/o	Control	Control	Feed Day	09:05	09:00 - 09:59	Late Morning	Olfactory exploration

2019/06/14	CH2206	Male	Three y/o	Control	Control	Feed Day	09:05	09:00 - 09:59	Late Morning	Locomotion
2019/06/14	CH2205	Male	Three y/o	Control	Control	Feed Day	09:10	09:00 - 09:59	Late Morning	Locomotion
2019/06/14	CH2206	Male	Three y/o	Control	Control	Feed Day	09:10	09:00 - 09:59	Late Morning	Scent-marking
2019/06/14	CH2205	Male	Three y/o	Control	Control	Feed Day	09:15	09:00 - 09:59	Late Morning	Locomotion
2019/06/14	CH2206	Male	Three y/o	Control	Control	Feed Day	09:15	09:00 - 09:59	Late Morning	Attention
2019/06/14	CH2205	Male	Three y/o	Control	Control	Feed Day	09:20	09:00 - 09:59	Late Morning	Olfactory exploration
2019/06/14	CH2206	Male	Three y/o	Control	Control	Feed Day	09:20	09:00 - 09:59	Late Morning	Scent-marking
2019/06/14	CH2205	Male	Three y/o	Control	Control	Feed Day	09:25	09:00 - 09:59	Late Morning	Inactive
2019/06/14	CH2206	Male	Three y/o	Control	Control	Feed Day	09:25	09:00 - 09:59	Late Morning	Inactive
2019/06/14	CH2205	Male	Three y/o	Control	Control	Feed Day	09:30	09:00 - 09:59	Late Morning	Inactive
2019/06/14	CH2206	Male	Three y/o	Control	Control	Feed Day	09:30	09:00 - 09:59	Late Morning	Inactive
2019/06/14	CH2205	Male	Three y/o	Control	Control	Feed Day	09:35	09:00 - 09:59	Late Morning	Inactive
2019/06/14	CH2206	Male	Three y/o	Control	Control	Feed Day	09:35	09:00 - 09:59	Late Morning	Inactive
2019/06/14	CH2205	Male	Three y/o	Control	Control	Feed Day	09:40	09:00 - 09:59	Late Morning	Inactive
2019/06/14	CH2206	Male	Three y/o	Control	Control	Feed Day	09:40	09:00 - 09:59	Late Morning	Inactive
2019/06/14	CH2205	Male	Three y/o	Control	Control	Feed Day	09:45	09:00 - 09:59	Late Morning	Inactive
2019/06/14	CH2206	Male	Three y/o	Control	Control	Feed Day	09:45	09:00 - 09:59	Late Morning	Inactive
2019/06/14	CH2205	Male	Three y/o	Control	Control	Feed Day	09:50	09:00 - 09:59	Late Morning	Inactive
2019/06/14	CH2206	Male	Three y/o	Control	Control	Feed Day	09:50	09:00 - 09:59	Late Morning	Inactive
2019/06/14	CH2207	Female	Three y/o	Control	Control	Feed Day	11:00	11:00 - 11:59	Late Morning	Attention
2019/06/14	CH2207	Female	Three y/o	Control	Control	Feed Day	11:05	11:00 - 11:59	Late Morning	Appetitive behaviour
2019/06/14	CH2207	Female	Three y/o	Control	Control	Feed Day	11:10	11:00 - 11:59	Late Morning	Locomotion
2019/06/14	CH2207	Female	Three y/o	Control	Control	Feed Day	11:15	11:00 - 11:59	Late Morning	Locomotion
2019/06/14	CH2207	Female	Three y/o	Control	Control	Feed Day	11:20	11:00 - 11:59	Late Morning	Appetitive behaviour
2019/06/14	CH2207	Female	Three y/o	Control	Control	Feed Day	11:25	11:00 - 11:59	Late Morning	Appetitive behaviour
2019/06/14	CH2207	Female	Three y/o	Control	Control	Feed Day	11:30	11:00 - 11:59	Late Morning	Inactive
2019/06/14	CH2207	Female	Three y/o	Control	Control	Feed Day	11:35	11:00 - 11:59	Late Morning	Auto-grooming
2019/06/14	CH2207	Female	Three y/o	Control	Control	Feed Day	11:40	11:00 - 11:59	Late Morning	Inactive
2019/06/14	CH2207	Female	Three y/o	Control	Control	Feed Day	11:45	11:00 - 11:59	Late Morning	Appetitive behaviour
2019/06/14	CH2207	Female	Three y/o	Control	Control	Feed Day	11:50	11:00 - 11:59	Late Morning	Inactive
2019/06/14	CH2207	Female	Three y/o	Control	Control	Feed Day	11:55	11:00 - 11:59	Late Morning	Inactive
2019/06/15	CH2207	Female	Three y/o	Control	Control	Feed Day	09:05	09:00 - 09:59	Late Morning	Attention
2019/06/15	CH2207	Female	Three y/o	Control	Control	Feed Day	09:10	09:00 - 09:59	Late Morning	Locomotion
2019/06/15	CH2207	Female	Three y/o	Control	Control	Feed Day	09:15	09:00 - 09:59	Late Morning	Agnostic behaviour
2019/06/15	CH2207	Female	Three y/o	Control	Control	Feed Day	09:20	09:00 - 09:59	Late Morning	Auto-grooming
2019/06/15	CH2207	Female	Three y/o	Control	Control	Feed Day	09:25	09:00 - 09:59	Late Morning	Auto-grooming
2019/06/15	CH2207	Female	Three y/o	Control	Control	Feed Day	09:30	09:00 - 09:59	Late Morning	Appetitive behaviour

2019/06/15	CH2207	Female	Three y/o	Control	Control	Feed Day	09:35	09:00 - 09:59	Late Morning	Auto-grooming
2019/06/15	CH2207	Female	Three y/o	Control	Control	Feed Day	09:40	09:00 - 09:59	Late Morning	Appetitive behaviour
2019/06/15	CH2207	Female	Three y/o	Control	Control	Feed Day	09:45	09:00 - 09:59	Late Morning	Appetitive behaviour
2019/06/15	CH2207	Female	Three y/o	Control	Control	Feed Day	09:50	09:00 - 09:59	Late Morning	Appetitive behaviour
2019/06/15	CH2207	Female	Three y/o	Control	Control	Feed Day	09:55	09:00 - 09:59	Late Morning	Inactive
2019/06/15	CH2207	Female	Three y/o	Control	Control	Feed Day	10:00	10:00 - 10:59	Late Morning	Appetitive behaviour
2019/06/15	CH2205	Male	Three y/o	Control	Control	Feed Day	10:00	10:00 - 10:59	Late Morning	Vocalisation
2019/06/15	CH2206	Male	Three y/o	Control	Control	Feed Day	10:00	10:00 - 10:59	Late Morning	Locomotion
2019/06/15	CH2205	Male	Three y/o	Control	Control	Feed Day	10:05	10:00 - 10:59	Late Morning	Locomotion
2019/06/15	CH2206	Male	Three y/o	Control	Control	Feed Day	10:05	10:00 - 10:59	Late Morning	Olfactory exploration
2019/06/15	CH2205	Male	Three y/o	Control	Control	Feed Day	10:10	10:00 - 10:59	Late Morning	Locomotion
2019/06/15	CH2206	Male	Three y/o	Control	Control	Feed Day	10:10	10:00 - 10:59	Late Morning	Interspecific behaviour
2019/06/15	CH2205	Male	Three y/o	Control	Control	Feed Day	10:15	10:00 - 10:59	Late Morning	Interspecific behaviour
2019/06/15	CH2206	Male	Three y/o	Control	Control	Feed Day	10:15	10:00 - 10:59	Late Morning	Interspecific behaviour
2019/06/15	CH2205	Male	Three y/o	Control	Control	Feed Day	10:20	10:00 - 10:59	Late Morning	Locomotion
2019/06/15	CH2206	Male	Three y/o	Control	Control	Feed Day	10:20	10:00 - 10:59	Late Morning	Inactive
2019/06/15	CH2205	Male	Three y/o	Control	Control	Feed Day	10:25	10:00 - 10:59	Late Morning	Inactive
2019/06/15	CH2206	Male	Three y/o	Control	Control	Feed Day	10:25	10:00 - 10:59	Late Morning	Inactive
2019/06/15	CH2205	Male	Three y/o	Control	Control	Feed Day	10:30	10:00 - 10:59	Late Morning	Auto-grooming
2019/06/15	CH2206	Male	Three y/o	Control	Control	Feed Day	10:30	10:00 - 10:59	Late Morning	Inactive
2019/06/15	CH2205	Male	Three y/o	Control	Control	Feed Day	10:35	10:00 - 10:59	Late Morning	Inactive
2019/06/15	CH2206	Male	Three y/o	Control	Control	Feed Day	10:35	10:00 - 10:59	Late Morning	Inactive
2019/06/15	CH2205	Male	Three y/o	Control	Control	Feed Day	10:40	10:00 - 10:59	Late Morning	Inactive
2019/06/15	CH2206	Male	Three y/o	Control	Control	Feed Day	10:40	10:00 - 10:59	Late Morning	Inactive
2019/06/15	CH2205	Male	Three y/o	Control	Control	Feed Day	10:45	10:00 - 10:59	Late Morning	Inactive
2019/06/15	CH2206	Male	Three y/o	Control	Control	Feed Day	10:45	10:00 - 10:59	Late Morning	Inactive
2019/06/15	CH2205	Male	Three y/o	Control	Control	Feed Day	10:50	10:00 - 10:59	Late Morning	Inactive
2019/06/15	CH2206	Male	Three y/o	Control	Control	Feed Day	10:50	10:00 - 10:59	Late Morning	Inactive
2019/06/15	CH2205	Male	Three y/o	Control	Control	Feed Day	10:55	10:00 - 10:59	Late Morning	Inactive
2019/06/15	CH2206	Male	Three y/o	Control	Control	Feed Day	10:55	10:00 - 10:59	Late Morning	Inactive
2019/06/18	CH2205	Male	Three y/o	Control	Control	Feed Day	10:10	10:00 - 10:59	Late Morning	Olfactory exploration
2019/06/18	CH2206	Male	Three y/o	Control	Control	Feed Day	10:10	10:00 - 10:59	Late Morning	Olfactory exploration
2019/06/18	CH2205	Male	Three y/o	Control	Control	Feed Day	10:15	10:00 - 10:59	Late Morning	Attention
2019/06/18	CH2206	Male	Three y/o	Control	Control	Feed Day	10:15	10:00 - 10:59	Late Morning	Locomotion
2019/06/18	CH2205	Male	Three y/o	Control	Control	Feed Day	10:20	10:00 - 10:59	Late Morning	Inactive
2019/06/18	CH2206	Male	Three y/o	Control	Control	Feed Day	10:20	10:00 - 10:59	Late Morning	Inactive
2019/06/18	CH2205	Male	Three y/o	Control	Control	Feed Day	10:25	10:00 - 10:59	Late Morning	Inactive

2019/06/18	CH2206	Male	Three y/o	Control	Control	Feed Day	10:25	10:00 - 10:59	Late Morning	Inactive
2019/06/18	CH2205	Male	Three y/o	Control	Control	Feed Day	10:30	10:00 - 10:59	Late Morning	Inactive
2019/06/18	CH2206	Male	Three y/o	Control	Control	Feed Day	10:30	10:00 - 10:59	Late Morning	Locomotion
2019/06/18	CH2205	Male	Three y/o	Control	Control	Feed Day	10:35	10:00 - 10:59	Late Morning	Inactive
2019/06/18	CH2206	Male	Three y/o	Control	Control	Feed Day	10:35	10:00 - 10:59	Late Morning	Inactive
2019/06/18	CH2205	Male	Three y/o	Control	Control	Feed Day	10:40	10:00 - 10:59	Late Morning	Inactive
2019/06/18	CH2206	Male	Three y/o	Control	Control	Feed Day	10:40	10:00 - 10:59	Late Morning	Inactive
2019/06/18	CH2205	Male	Three y/o	Control	Control	Feed Day	10:45	10:00 - 10:59	Late Morning	Inactive
2019/06/18	CH2206	Male	Three y/o	Control	Control	Feed Day	10:45	10:00 - 10:59	Late Morning	Inactive
2019/06/18	CH2205	Male	Three y/o	Control	Control	Feed Day	10:50	10:00 - 10:59	Late Morning	Inactive
2019/06/18	CH2206	Male	Three y/o	Control	Control	Feed Day	10:50	10:00 - 10:59	Late Morning	Inactive
2019/06/18	CH2205	Male	Three y/o	Control	Control	Feed Day	10:55	10:00 - 10:59	Late Morning	Inactive
2019/06/18	CH2206	Male	Three y/o	Control	Control	Feed Day	10:55	10:00 - 10:59	Late Morning	Inactive
2019/06/18	CH2205	Male	Three y/o	Control	Control	Feed Day	11:00	11:00 - 11:59	Late Morning	Inactive
2019/06/18	CH2206	Male	Three y/o	Control	Control	Feed Day	11:00	11:00 - 11:59	Late Morning	Interspecific behaviour
2019/06/18	CH2205	Male	Three y/o	Control	Control	Feed Day	11:05	11:00 - 11:59	Late Morning	Inactive
2019/06/18	CH2206	Male	Three y/o	Control	Control	Feed Day	11:05	11:00 - 11:59	Late Morning	Interspecific behaviour
2019/06/18	CH2207	Female	Three y/o	Control	Control	Feed Day	11:05	11:00 - 11:59	Late Morning	Scent-marking
2019/06/18	CH2207	Female	Three y/o	Control	Control	Feed Day	11:10	11:00 - 11:59	Late Morning	Scent-marking
2019/06/18	CH2207	Female	Three y/o	Control	Control	Feed Day	11:15	11:00 - 11:59	Late Morning	Locomotion
2019/06/18	CH2207	Female	Three y/o	Control	Control	Feed Day	11:20	11:00 - 11:59	Late Morning	Appetitive behaviour
2019/06/18	CH2207	Female	Three y/o	Control	Control	Feed Day	11:25	11:00 - 11:59	Late Morning	Appetitive behaviour
2019/06/18	CH2207	Female	Three y/o	Control	Control	Feed Day	11:30	11:00 - 11:59	Late Morning	Appetitive behaviour
2019/06/18	CH2207	Female	Three y/o	Control	Control	Feed Day	11:35	11:00 - 11:59	Late Morning	Inactive
2019/06/18	CH2207	Female	Three y/o	Control	Control	Feed Day	11:40	11:00 - 11:59	Late Morning	Inactive
2019/06/18	CH2207	Female	Three y/o	Control	Control	Feed Day	11:45	11:00 - 11:59	Late Morning	Inactive
2019/06/18	CH2207	Female	Three y/o	Control	Control	Feed Day	11:50	11:00 - 11:59	Late Morning	Inactive
2019/06/18	CH2207	Female	Three y/o	Control	Control	Feed Day	11:55	11:00 - 11:59	Late Morning	Inactive
2019/06/18	CH2207	Female	Three y/o	Control	Control	Feed Day	12:00	12:00 - 12:59	Afternoon	Inactive
2019/06/19	CH2207	Female	Three y/o	Control	Control	Feed Day	08:45	08:00 - 08:59	Late Morning	Attention
2019/06/19	CH2207	Female	Three y/o	Control	Control	Feed Day	08:50	08:00 - 08:59	Late Morning	Attention
2019/06/19	CH2207	Female	Three y/o	Control	Control	Feed Day	08:55	08:00 - 08:59	Late Morning	Attention
2019/06/19	CH2207	Female	Three y/o	Control	Control	Feed Day	09:00	09:00 - 09:59	Late Morning	Inactive
2019/06/19	CH2207	Female	Three y/o	Control	Control	Feed Day	09:05	09:00 - 09:59	Late Morning	Scent-marking
2019/06/19	CH2207	Female	Three y/o	Control	Control	Feed Day	09:10	09:00 - 09:59	Late Morning	Auto-grooming
2019/06/19	CH2207	Female	Three y/o	Control	Control	Feed Day	09:15	09:00 - 09:59	Late Morning	Auto-grooming
2019/06/19	CH2207	Female	Three y/o	Control	Control	Feed Day	09:20	09:00 - 09:59	Late Morning	Auto-grooming

2019/06/19	CH2207	Female	Three y/o	Control	Control	Feed Day	09:25	09:00 - 09:59	Late Morning	Locomotion
2019/06/19	CH2207	Female	Three y/o	Control	Control	Feed Day	09:30	09:00 - 09:59	Late Morning	Attention
2019/06/19	CH2207	Female	Three y/o	Control	Control	Feed Day	09:35	09:00 - 09:59	Late Morning	Locomotion
2019/06/19	CH2207	Female	Three y/o	Control	Control	Feed Day	09:40	09:00 - 09:59	Late Morning	Inactive
2019/06/19	CH2205	Male	Three y/o	Control	Control	Feed Day	09:45	09:00 - 09:59	Late Morning	Inactive
2019/06/19	CH2206	Male	Three y/o	Control	Control	Feed Day	09:45	09:00 - 09:59	Late Morning	Olfactory exploration
2019/06/19	CH2205	Male	Three y/o	Control	Control	Feed Day	09:50	09:00 - 09:59	Late Morning	Affiliative behaviour
2019/06/19	CH2206	Male	Three y/o	Control	Control	Feed Day	09:50	09:00 - 09:59	Late Morning	Affiliative behaviour
2019/06/19	CH2205	Male	Three y/o	Control	Control	Feed Day	09:55	09:00 - 09:59	Late Morning	Locomotion
2019/06/19	CH2206	Male	Three y/o	Control	Control	Feed Day	09:55	09:00 - 09:59	Late Morning	Affiliative behaviour
2019/06/19	CH2205	Male	Three y/o	Control	Control	Feed Day	10:00	10:00 - 10:59	Late Morning	Scent-marking
2019/06/19	CH2206	Male	Three y/o	Control	Control	Feed Day	10:00	10:00 - 10:59	Late Morning	Auto-grooming
2019/06/19	CH2205	Male	Three y/o	Control	Control	Feed Day	10:05	10:00 - 10:59	Late Morning	Inactive
2019/06/19	CH2206	Male	Three y/o	Control	Control	Feed Day	10:05	10:00 - 10:59	Late Morning	Inactive
2019/06/19	CH2205	Male	Three y/o	Control	Control	Feed Day	10:10	10:00 - 10:59	Late Morning	Inactive
2019/06/19	CH2206	Male	Three y/o	Control	Control	Feed Day	10:10	10:00 - 10:59	Late Morning	Inactive
2019/06/19	CH2205	Male	Three y/o	Control	Control	Feed Day	10:15	10:00 - 10:59	Late Morning	Attention
2019/06/19	CH2206	Male	Three y/o	Control	Control	Feed Day	10:15	10:00 - 10:59	Late Morning	Inactive
2019/06/19	CH2205	Male	Three y/o	Control	Control	Feed Day	10:20	10:00 - 10:59	Late Morning	Inactive
2019/06/19	CH2206	Male	Three y/o	Control	Control	Feed Day	10:20	10:00 - 10:59	Late Morning	Olfactory exploration
2019/06/19	CH2205	Male	Three y/o	Control	Control	Feed Day	10:25	10:00 - 10:59	Late Morning	Interspecific behaviour
2019/06/19	CH2206	Male	Three y/o	Control	Control	Feed Day	10:25	10:00 - 10:59	Late Morning	Inactive
2019/06/19	CH2205	Male	Three y/o	Control	Control	Feed Day	10:30	10:00 - 10:59	Late Morning	Interspecific behaviour
2019/06/19	CH2206	Male	Three y/o	Control	Control	Feed Day	10:30	10:00 - 10:59	Late Morning	Inactive
2019/06/19	CH2205	Male	Three y/o	Control	Control	Feed Day	10:35	10:00 - 10:59	Late Morning	Inactive
2019/06/19	CH2206	Male	Three y/o	Control	Control	Feed Day	10:35	10:00 - 10:59	Late Morning	Inactive
2019/06/19	CH2205	Male	Three y/o	Control	Control	Feed Day	10:40	10:00 - 10:59	Late Morning	Inactive
2019/06/19	CH2206	Male	Three y/o	Control	Control	Feed Day	10:40	10:00 - 10:59	Late Morning	Inactive
2019/07/09	CH2271	Male	Two y/o	Treatment (Wk 1)	Treatment	Feed Day	11:35	11:00 - 11:59	Late Morning	Locomotion
2019/07/09	CH2271	Male	Two y/o	Treatment (Wk 1)	Treatment	Feed Day	11:40	11:00 - 11:59	Late Morning	Interspecific behaviour
2019/07/09	CH2271	Male	Two y/o	Treatment (Wk 1)	Treatment	Feed Day	11:45	11:00 - 11:59	Late Morning	Interspecific behaviour
2019/07/09	CH2271	Male	Two y/o	Treatment (Wk 1)	Treatment	Feed Day	11:50	11:00 - 11:59	Late Morning	Scent-marking
2019/07/09	CH2271	Male	Two y/o	Treatment (Wk 1)	Treatment	Feed Day	11:55	11:00 - 11:59	Late Morning	Interspecific behaviour
2019/07/09	CH2271	Male	Two y/o	Treatment (Wk 1)	Treatment	Feed Day	12:00	12:00 - 12:59	Afternoon	Inactive
2019/07/09	CH2271	Male	Two y/o	Treatment (Wk 1)	Treatment	Feed Day	12:05	12:00 - 12:59	Afternoon	Interspecific behaviour
2019/07/09	CH2271	Male	Two y/o	Treatment (Wk 1)	Treatment	Feed Day	12:10	12:00 - 12:59	Afternoon	Inactive
2019/07/09	CH2271	Male	Two y/o	Treatment (Wk 1)	Treatment	Feed Day	12:15	12:00 - 12:59	Afternoon	Interspecific behaviour

2019/07/09	CH2271	Male	Two y/o	Treatment (Wk 1)	Treatment	Feed Day	12:20	12:00 - 12:59	Afternoon	Interspecific behaviour
2019/07/09	CH2271	Male	Two y/o	Treatment (Wk 1)	Treatment	Feed Day	12:25	12:00 - 12:59	Afternoon	Inactive
2019/07/09	CH2271	Male	Two y/o	Treatment (Wk 1)	Treatment	Feed Day	12:30	12:00 - 12:59	Afternoon	Interspecific behaviour
2019/07/10	CH2271	Male	Two y/o	Treatment (Wk 1)	Treatment	Fast Day	11:55	11:00 - 11:59	Late Morning	Interspecific behaviour
2019/07/10	CH2271	Male	Two y/o	Treatment (Wk 1)	Treatment	Fast Day	12:00	12:00 - 12:59	Afternoon	Affiliative behaviour
2019/07/10	CH2271	Male	Two y/o	Treatment (Wk 1)	Treatment	Fast Day	12:05	12:00 - 12:59	Afternoon	Inactive
2019/07/10	CH2271	Male	Two y/o	Treatment (Wk 1)	Treatment	Fast Day	12:10	12:00 - 12:59	Afternoon	Inactive
2019/07/10	CH2271	Male	Two y/o	Treatment (Wk 1)	Treatment	Fast Day	12:15	12:00 - 12:59	Afternoon	Inactive
2019/07/10	CH2271	Male	Two y/o	Treatment (Wk 1)	Treatment	Fast Day	12:20	12:00 - 12:59	Afternoon	Locomotion
2019/07/10	CH2271	Male	Two y/o	Treatment (Wk 1)	Treatment	Fast Day	12:25	12:00 - 12:59	Afternoon	Inactive
2019/07/10	CH2271	Male	Two y/o	Treatment (Wk 1)	Treatment	Fast Day	12:30	12:00 - 12:59	Afternoon	Inactive
2019/07/10	CH2271	Male	Two y/o	Treatment (Wk 1)	Treatment	Fast Day	12:35	12:00 - 12:59	Afternoon	Inactive
2019/07/10	CH2271	Male	Two y/o	Treatment (Wk 1)	Treatment	Fast Day	12:40	12:00 - 12:59	Afternoon	Scent-marking
2019/07/10	CH2271	Male	Two y/o	Treatment (Wk 1)	Treatment	Fast Day	12:45	12:00 - 12:59	Afternoon	Attention
2019/07/10	CH2271	Male	Two y/o	Treatment (Wk 1)	Treatment	Fast Day	12:50	12:00 - 12:59	Afternoon	Inactive
2019/07/11	CH2271	Male	Two y/o	Treatment (Wk 1)	Treatment	Feed Day	08:35	08:00 - 08:59	Late Morning	Interspecific behaviour
2019/07/11	CH2271	Male	Two y/o	Treatment (Wk 1)	Treatment	Feed Day	08:40	08:00 - 08:59	Late Morning	Locomotion
2019/07/11	CH2271	Male	Two y/o	Treatment (Wk 1)	Treatment	Feed Day	08:45	08:00 - 08:59	Late Morning	Scent-marking
2019/07/11	CH2271	Male	Two y/o	Treatment (Wk 1)	Treatment	Feed Day	08:50	08:00 - 08:59	Late Morning	Interspecific behaviour
2019/07/11	CH2271	Male	Two y/o	Treatment (Wk 1)	Treatment	Feed Day	08:55	08:00 - 08:59	Late Morning	Appetitive behaviour
2019/07/11	CH2271	Male	Two y/o	Treatment (Wk 1)	Treatment	Feed Day	09:00	09:00 - 09:59	Late Morning	Appetitive behaviour
2019/07/11	CH2271	Male	Two y/o	Treatment (Wk 1)	Treatment	Feed Day	09:05	09:00 - 09:59	Late Morning	Interspecific behaviour
2019/07/11	CH2271	Male	Two y/o	Treatment (Wk 1)	Treatment	Feed Day	09:10	09:00 - 09:59	Late Morning	Appetitive behaviour
2019/07/11	CH2271	Male	Two y/o	Treatment (Wk 1)	Treatment	Feed Day	09:15	09:00 - 09:59	Late Morning	Appetitive behaviour
2019/07/11	CH2271	Male	Two y/o	Treatment (Wk 1)	Treatment	Feed Day	09:20	09:00 - 09:59	Late Morning	Scent-marking
2019/07/11	CH2271	Male	Two y/o	Treatment (Wk 1)	Treatment	Feed Day	09:25	09:00 - 09:59	Late Morning	Interspecific behaviour
2019/07/11	CH2271	Male	Two y/o	Treatment (Wk 1)	Treatment	Feed Day	09:30	09:00 - 09:59	Late Morning	Locomotion
2019/07/12	CH2271	Male	Two y/o	Treatment (Wk 1)	Treatment	Feed Day	08:40	08:00 - 08:59	Late Morning	Interspecific behaviour
2019/07/12	CH2271	Male	Two y/o	Treatment (Wk 1)	Treatment	Feed Day	08:45	08:00 - 08:59	Late Morning	Affiliative behaviour
2019/07/12	CH2271	Male	Two y/o	Treatment (Wk 1)	Treatment	Feed Day	08:50	08:00 - 08:59	Late Morning	Attention
2019/07/12	CH2271	Male	Two y/o	Treatment (Wk 1)	Treatment	Feed Day	08:55	08:00 - 08:59	Late Morning	Attention
2019/07/12	CH2271	Male	Two y/o	Treatment (Wk 1)	Treatment	Feed Day	09:00	09:00 - 09:59	Late Morning	Locomotion
2019/07/12	CH2271	Male	Two y/o	Treatment (Wk 1)	Treatment	Feed Day	09:05	08:00 - 08:59	Late Morning	Locomotion
2019/07/12	CH2271	Male	Two y/o	Treatment (Wk 1)	Treatment	Feed Day	09:10	08:00 - 08:59	Late Morning	Locomotion
2019/07/12	CH2271	Male	Two y/o	Treatment (Wk 1)	Treatment	Feed Day	09:15	09:00 - 09:59	Late Morning	Locomotion
2019/07/12	CH2271	Male	Two y/o	Treatment (Wk 1)	Treatment	Feed Day	09:20	09:00 - 09:59	Late Morning	Locomotion
2019/07/12	CH2271	Male	Two y/o	Treatment (Wk 1)	Treatment	Feed Day	09:25	09:00 - 09:59	Late Morning	Locomotion

2019/07/12	CH2271	Male	Two y/o	Treatment (Wk 1)	Treatment	Feed Day	09:30	09:00 - 09:59	Late Morning	Attention
2019/07/12	CH2271	Male	Two y/o	Treatment (Wk 1)	Treatment	Feed Day	09:35	09:00 - 09:59	Late Morning	Attention
2019/07/15	CH2271	Male	Two y/o	Treatment (Wk 1)	Treatment	Feed Day	08:35	08:00 - 08:59	Late Morning	Interspecific behaviour
2019/07/15	CH2271	Male	Two y/o	Treatment (Wk 1)	Treatment	Feed Day	08:40	08:00 - 08:59	Late Morning	Affiliative behaviour
2019/07/15	CH2271	Male	Two y/o	Treatment (Wk 1)	Treatment	Feed Day	08:45	08:00 - 08:59	Late Morning	Attention
2019/07/15	CH2271	Male	Two y/o	Treatment (Wk 1)	Treatment	Feed Day	08:50	08:00 - 08:59	Late Morning	Attention
2019/07/15	CH2271	Male	Two y/o	Treatment (Wk 1)	Treatment	Feed Day	08:55	08:00 - 08:59	Late Morning	Locomotion
2019/07/15	CH2271	Male	Two y/o	Treatment (Wk 1)	Treatment	Feed Day	09:00	09:00 - 09:59	Late Morning	Locomotion
2019/07/15	CH2271	Male	Two y/o	Treatment (Wk 1)	Treatment	Feed Day	09:05	09:00 - 09:59	Late Morning	Locomotion
2019/07/15	CH2271	Male	Two y/o	Treatment (Wk 1)	Treatment	Feed Day	09:10	09:00 - 09:59	Late Morning	Locomotion
2019/07/15	CH2271	Male	Two y/o	Treatment (Wk 1)	Treatment	Feed Day	09:15	09:00 - 09:59	Late Morning	Locomotion
2019/07/15	CH2271	Male	Two y/o	Treatment (Wk 1)	Treatment	Feed Day	09:20	09:00 - 09:59	Late Morning	Locomotion
2019/07/15	CH2271	Male	Two y/o	Treatment (Wk 1)	Treatment	Feed Day	09:25	09:00 - 09:59	Late Morning	Locomotion
2019/07/15	CH2271	Male	Two y/o	Treatment (Wk 1)	Treatment	Feed Day	09:30	09:00 - 09:59	Late Morning	Locomotion
2019/07/16	CH2271	Male	Two y/o	Treatment (Wk 2)	Treatment	Feed Day	10:00	10:00 - 10:59	Late Morning	Attention
2019/07/16	CH2271	Male	Two y/o	Treatment (Wk 2)	Treatment	Feed Day	10:05	10:00 - 10:59	Late Morning	Locomotion
2019/07/16	CH2271	Male	Two y/o	Treatment (Wk 2)	Treatment	Feed Day	10:10	10:00 - 10:59	Late Morning	Appetitive behaviour
2019/07/16	CH2271	Male	Two y/o	Treatment (Wk 2)	Treatment	Feed Day	10:15	10:00 - 10:59	Late Morning	Appetitive behaviour
2019/07/16	CH2271	Male	Two y/o	Treatment (Wk 2)	Treatment	Feed Day	10:20	10:00 - 10:59	Late Morning	Affiliative behaviour
2019/07/16	CH2271	Male	Two y/o	Treatment (Wk 2)	Treatment	Feed Day	10:25	10:00 - 10:59	Late Morning	Maintenance
2019/07/16	CH2271	Male	Two y/o	Treatment (Wk 2)	Treatment	Feed Day	10:30	10:00 - 10:59	Late Morning	Inactive
2019/07/16	CH2271	Male	Two y/o	Treatment (Wk 2)	Treatment	Feed Day	10:35	10:00 - 10:59	Late Morning	Inactive
2019/07/16	CH2271	Male	Two y/o	Treatment (Wk 2)	Treatment	Feed Day	10:40	10:00 - 10:59	Late Morning	Inactive
2019/07/16	CH2271	Male	Two y/o	Treatment (Wk 2)	Treatment	Feed Day	10:45	10:00 - 10:59	Late Morning	Locomotion
2019/07/16	CH2271	Male	Two y/o	Treatment (Wk 2)	Treatment	Feed Day	10:50	10:00 - 10:59	Late Morning	Affiliative behaviour
2019/07/16	CH2271	Male	Two y/o	Treatment (Wk 2)	Treatment	Feed Day	10:55	10:00 - 10:59	Late Morning	Affiliative behaviour
2019/07/17	CH2271	Male	Two y/o	Treatment (Wk 2)	Treatment	Fast Day	08:50	08:00 - 08:59	Late Morning	Inactive
2019/07/17	CH2271	Male	Two y/o	Treatment (Wk 2)	Treatment	Fast Day	08:55	08:00 - 08:59	Late Morning	Inactive
2019/07/17	CH2271	Male	Two y/o	Treatment (Wk 2)	Treatment	Fast Day	09:00	09:00 - 09:59	Late Morning	Inactive
2019/07/17	CH2271	Male	Two y/o	Treatment (Wk 2)	Treatment	Fast Day	09:05	09:00 - 09:59	Late Morning	Inactive
2019/07/17	CH2271	Male	Two y/o	Treatment (Wk 2)	Treatment	Fast Day	09:10	09:00 - 09:59	Late Morning	Inactive
2019/07/17	CH2271	Male	Two y/o	Treatment (Wk 2)	Treatment	Fast Day	09:15	09:00 - 09:59	Late Morning	Attention
2019/07/17	CH2271	Male	Two y/o	Treatment (Wk 2)	Treatment	Fast Day	09:20	09:00 - 09:59	Late Morning	Interspecific behaviour
2019/07/17	CH2271	Male	Two y/o	Treatment (Wk 2)	Treatment	Fast Day	09:25	09:00 - 09:59	Late Morning	Interspecific behaviour
2019/07/17	CH2271	Male	Two y/o	Treatment (Wk 2)	Treatment	Fast Day	09:30	09:00 - 09:59	Late Morning	Affiliative behaviour
2019/07/17	CH2271	Male	Two y/o	Treatment (Wk 2)	Treatment	Fast Day	09:35	09:00 - 09:59	Late Morning	Locomotion
2019/07/17	CH2271	Male	Two y/o	Treatment (Wk 2)	Treatment	Fast Day	09:40	09:00 - 09:59	Late Morning	Affiliative behaviour

2019/07/17	CH2271	Male	Two y/o	Treatment (Wk 2)	Treatment	Fast Day	09:45	09:00 - 09:59	Late Morning	Locomotion
2019/07/18	CH2271	Male	Two y/o	Treatment (Wk 2)	Treatment	Feed Day	09:55	09:00 - 09:59	Late Morning	Affiliative behaviour
2019/07/18	CH2271	Male	Two y/o	Treatment (Wk 2)	Treatment	Feed Day	10:00	10:00 - 10:59	Late Morning	Interspecific behaviour
2019/07/18	CH2271	Male	Two y/o	Treatment (Wk 2)	Treatment	Feed Day	10:05	10:00 - 10:59	Late Morning	Inactive
2019/07/18	CH2271	Male	Two y/o	Treatment (Wk 2)	Treatment	Feed Day	10:10	10:00 - 10:59	Late Morning	Inactive
2019/07/18	CH2271	Male	Two y/o	Treatment (Wk 2)	Treatment	Feed Day	10:15	10:00 - 10:59	Late Morning	Inactive
2019/07/18	CH2271	Male	Two y/o	Treatment (Wk 2)	Treatment	Feed Day	10:20	10:00 - 10:59	Late Morning	Inactive
2019/07/18	CH2271	Male	Two y/o	Treatment (Wk 2)	Treatment	Feed Day	10:25	10:00 - 10:59	Late Morning	Inactive
2019/07/18	CH2271	Male	Two y/o	Treatment (Wk 2)	Treatment	Feed Day	10:30	10:00 - 10:59	Late Morning	Attention
2019/07/18	CH2271	Male	Two y/o	Treatment (Wk 2)	Treatment	Feed Day	10:35	10:00 - 10:59	Late Morning	Inactive
2019/07/19	CH2271	Male	Two y/o	Treatment (Wk 2)	Treatment	Feed Day	09:30	09:00 - 09:59	Late Morning	Appetitive behaviour
2019/07/19	CH2271	Male	Two y/o	Treatment (Wk 2)	Treatment	Feed Day	09:35	09:00 - 09:59	Late Morning	Appetitive behaviour
2019/07/19	CH2271	Male	Two y/o	Treatment (Wk 2)	Treatment	Feed Day	09:40	09:00 - 09:59	Late Morning	Affiliative behaviour
2019/07/19	CH2271	Male	Two y/o	Treatment (Wk 2)	Treatment	Feed Day	09:45	09:00 - 09:59	Late Morning	Appetitive behaviour
2019/07/19	CH2271	Male	Two y/o	Treatment (Wk 2)	Treatment	Feed Day	09:50	09:00 - 09:59	Late Morning	Appetitive behaviour
2019/07/19	CH2271	Male	Two y/o	Treatment (Wk 2)	Treatment	Feed Day	09:55	09:00 - 09:59	Late Morning	Appetitive behaviour
2019/07/19	CH2271	Male	Two y/o	Treatment (Wk 2)	Treatment	Feed Day	10:00	10:00 - 10:59	Late Morning	Locomotion
2019/07/19	CH2271	Male	Two y/o	Treatment (Wk 2)	Treatment	Feed Day	10:05	10:00 - 10:59	Late Morning	Appetitive behaviour
2019/07/19	CH2271	Male	Two y/o	Treatment (Wk 2)	Treatment	Feed Day	10:10	10:00 - 10:59	Late Morning	Appetitive behaviour
2019/07/19	CH2271	Male	Two y/o	Treatment (Wk 2)	Treatment	Feed Day	10:15	10:00 - 10:59	Late Morning	Scent-marking
2019/07/19	CH2271	Male	Two y/o	Treatment (Wk 2)	Treatment	Feed Day	10:20	10:00 - 10:59	Late Morning	Interspecific behaviour
2019/07/19	CH2271	Male	Two y/o	Treatment (Wk 2)	Treatment	Feed Day	10:25	10:00 - 10:59	Late Morning	Locomotion
2019/07/22	CH2271	Male	Two y/o	Treatment (Wk 2)	Treatment	Feed Day	09:30	09:00 - 09:59	Late Morning	Interspecific behaviour
2019/07/22	CH2271	Male	Two y/o	Treatment (Wk 2)	Treatment	Feed Day	09:35	09:00 - 09:59	Late Morning	Locomotion
2019/07/22	CH2271	Male	Two y/o	Treatment (Wk 2)	Treatment	Feed Day	09:40	09:00 - 09:59	Late Morning	Scent-marking
2019/07/22	CH2271	Male	Two y/o	Treatment (Wk 2)	Treatment	Feed Day	09:45	09:00 - 09:59	Late Morning	Locomotion
2019/07/22	CH2271	Male	Two y/o	Treatment (Wk 2)	Treatment	Feed Day	09:50	09:00 - 09:59	Late Morning	Interspecific behaviour
2019/07/22	CH2271	Male	Two y/o	Treatment (Wk 2)	Treatment	Feed Day	09:55	09:00 - 09:59	Late Morning	Locomotion
2019/07/22	CH2271	Male	Two y/o	Treatment (Wk 2)	Treatment	Feed Day	10:00	10:00 - 10:59	Late Morning	Locomotion
2019/07/22	CH2271	Male	Two y/o	Treatment (Wk 2)	Treatment	Feed Day	10:05	10:00 - 10:59	Late Morning	Locomotion
2019/07/22	CH2271	Male	Two y/o	Treatment (Wk 2)	Treatment	Feed Day	10:10	10:00 - 10:59	Late Morning	Locomotion
2019/07/22	CH2271	Male	Two y/o	Treatment (Wk 2)	Treatment	Feed Day	10:15	10:00 - 10:59	Late Morning	Inactive
2019/07/22	CH2271	Male	Two y/o	Treatment (Wk 2)	Treatment	Feed Day	10:20	10:00 - 10:59	Late Morning	Inactive
2019/07/22	CH2271	Male	Two y/o	Treatment (Wk 2)	Treatment	Feed Day	10:25	10:00 - 10:59	Late Morning	Locomotion
2019/07/23	CH2271	Male	Two y/o	Treatment (Wk 3)	Treatment	Feed Day	08:30	08:00 - 08:59	Late Morning	Attention
2019/07/23	CH2271	Male	Two y/o	Treatment (Wk 3)	Treatment	Feed Day	08:35	08:00 - 08:59	Late Morning	Locomotion
2019/07/23	CH2271	Male	Two y/o	Treatment (Wk 3)	Treatment	Feed Day	08:40	08:00 - 08:59	Late Morning	Scent-marking

2019/07/23	CH2271	Male	Two y/o	Treatment (Wk 3)	Treatment	Feed Day	08:45	08:00 - 08:59	Late Morning	Attention
2019/07/23	CH2271	Male	Two y/o	Treatment (Wk 3)	Treatment	Feed Day	08:50	08:00 - 08:59	Late Morning	Locomotion
2019/07/23	CH2271	Male	Two y/o	Treatment (Wk 3)	Treatment	Feed Day	08:55	08:00 - 08:59	Late Morning	Locomotion
2019/07/23	CH2271	Male	Two y/o	Treatment (Wk 3)	Treatment	Feed Day	09:00	09:00 - 09:59	Late Morning	Scent-marking
2019/07/23	CH2271	Male	Two y/o	Treatment (Wk 3)	Treatment	Feed Day	09:05	09:00 - 09:59	Late Morning	Locomotion
2019/07/23	CH2271	Male	Two y/o	Treatment (Wk 3)	Treatment	Feed Day	09:10	09:00 - 09:59	Late Morning	Inactive
2019/07/23	CH2271	Male	Two y/o	Treatment (Wk 3)	Treatment	Feed Day	09:15	09:00 - 09:59	Late Morning	Locomotion
2019/07/23	CH2271	Male	Two y/o	Treatment (Wk 3)	Treatment	Feed Day	09:20	09:00 - 09:59	Late Morning	Locomotion
2019/07/23	CH2271	Male	Two y/o	Treatment (Wk 3)	Treatment	Feed Day	09:25	09:00 - 09:59	Late Morning	Locomotion
2019/07/24	CH2271	Male	Two y/o	Treatment (Wk 3)	Treatment	Fast Day	09:30	09:00 - 09:59	Late Morning	Locomotion
2019/07/24	CH2271	Male	Two y/o	Treatment (Wk 3)	Treatment	Fast Day	09:35	09:00 - 09:59	Late Morning	Inactive
2019/07/24	CH2271	Male	Two y/o	Treatment (Wk 3)	Treatment	Fast Day	09:40	09:00 - 09:59	Late Morning	Inactive
2019/07/24	CH2271	Male	Two y/o	Treatment (Wk 3)	Treatment	Fast Day	09:45	09:00 - 09:59	Late Morning	Inactive
2019/07/24	CH2271	Male	Two y/o	Treatment (Wk 3)	Treatment	Fast Day	09:50	09:00 - 09:59	Late Morning	Inactive
2019/07/24	CH2271	Male	Two y/o	Treatment (Wk 3)	Treatment	Fast Day	09:55	09:00 - 09:59	Late Morning	Inactive
2019/07/24	CH2271	Male	Two y/o	Treatment (Wk 3)	Treatment	Fast Day	10:00	10:00 - 10:59	Late Morning	Inactive
2019/07/24	CH2271	Male	Two y/o	Treatment (Wk 3)	Treatment	Fast Day	10:05	10:00 - 10:59	Late Morning	Inactive
2019/07/24	CH2271	Male	Two y/o	Treatment (Wk 3)	Treatment	Fast Day	10:10	10:00 - 10:59	Late Morning	Inactive
2019/07/24	CH2271	Male	Two y/o	Treatment (Wk 3)	Treatment	Fast Day	10:15	10:00 - 10:59	Late Morning	Inactive
2019/07/24	CH2271	Male	Two y/o	Treatment (Wk 3)	Treatment	Fast Day	10:20	10:00 - 10:59	Late Morning	Inactive
2019/07/24	CH2271	Male	Two y/o	Treatment (Wk 3)	Treatment	Fast Day	10:25	10:00 - 10:59	Late Morning	Interspecific behaviour
2019/07/25	CH2271	Male	Two y/o	Treatment (Wk 3)	Treatment	Feed Day	08:40	08:00 - 08:59	Late Morning	Attention
2019/07/25	CH2271	Male	Two y/o	Treatment (Wk 3)	Treatment	Feed Day	08:45	08:00 - 08:59	Late Morning	Locomotion
2019/07/25	CH2271	Male	Two y/o	Treatment (Wk 3)	Treatment	Feed Day	08:50	08:00 - 08:59	Late Morning	Olfactory exploration
2019/07/25	CH2271	Male	Two y/o	Treatment (Wk 3)	Treatment	Feed Day	08:55	08:00 - 08:59	Late Morning	Inactive
2019/07/25	CH2271	Male	Two y/o	Treatment (Wk 3)	Treatment	Feed Day	09:00	09:00 - 09:59	Late Morning	Appetitive behaviour
2019/07/25	CH2271	Male	Two y/o	Treatment (Wk 3)	Treatment	Feed Day	09:05	09:00 - 09:59	Late Morning	Appetitive behaviour
2019/07/25	CH2271	Male	Two y/o	Treatment (Wk 3)	Treatment	Feed Day	09:10	09:00 - 09:59	Late Morning	Affiliative behaviour
2019/07/25	CH2271	Male	Two y/o	Treatment (Wk 3)	Treatment	Feed Day	09:15	09:00 - 09:59	Late Morning	Locomotion
2019/07/25	CH2271	Male	Two y/o	Treatment (Wk 3)	Treatment	Feed Day	09:20	09:00 - 09:59	Late Morning	Locomotion
2019/07/25	CH2271	Male	Two y/o	Treatment (Wk 3)	Treatment	Feed Day	09:25	09:00 - 09:59	Late Morning	Affiliative behaviour
2019/07/25	CH2271	Male	Two y/o	Treatment (Wk 3)	Treatment	Feed Day	09:30	09:00 - 09:59	Late Morning	Locomotion
2019/07/25	CH2271	Male	Two y/o	Treatment (Wk 3)	Treatment	Feed Day	09:35	09:00 - 09:59	Late Morning	Attention
2019/07/26	CH2271	Male	Two y/o	Treatment (Wk 3)	Treatment	Feed Day	08:45	08:00 - 08:59	Late Morning	Locomotion
2019/07/26	CH2271	Male	Two y/o	Treatment (Wk 3)	Treatment	Feed Day	08:50	08:00 - 08:59	Late Morning	Attention
2019/07/26	CH2271	Male	Two y/o	Treatment (Wk 3)	Treatment	Feed Day	08:55	08:00 - 08:59	Late Morning	Attention
2019/07/26	CH2271	Male	Two y/o	Treatment (Wk 3)	Treatment	Feed Day	09:00	09:00 - 09:59	Late Morning	Locomotion

2019/07/26	CH2271	Male	Two y/o	Treatment (Wk 3)	Treatment	Feed Day	09:05	09:00 - 09:59	Late Morning	Locomotion
2019/07/26	CH2271	Male	Two y/o	Treatment (Wk 3)	Treatment	Feed Day	09:10	09:00 - 09:59	Late Morning	Locomotion
2019/07/26	CH2271	Male	Two y/o	Treatment (Wk 3)	Treatment	Feed Day	09:15	09:00 - 09:59	Late Morning	Locomotion
2019/07/26	CH2271	Male	Two y/o	Treatment (Wk 3)	Treatment	Feed Day	09:20	09:00 - 09:59	Late Morning	Locomotion
2019/07/26	CH2271	Male	Two y/o	Treatment (Wk 3)	Treatment	Feed Day	09:25	09:00 - 09:59	Late Morning	Locomotion
2019/07/26	CH2271	Male	Two y/o	Treatment (Wk 3)	Treatment	Feed Day	09:30	09:00 - 09:59	Late Morning	Locomotion
2019/07/26	CH2271	Male	Two y/o	Treatment (Wk 3)	Treatment	Feed Day	09:35	09:00 - 09:59	Late Morning	Locomotion
2019/07/26	CH2271	Male	Two y/o	Treatment (Wk 3)	Treatment	Feed Day	09:40	09:00 - 09:59	Late Morning	Attention
2019/07/29	CH2271	Male	Two y/o	Treatment (Wk 3)	Treatment	Feed Day	09:30	09:00 - 09:59	Late Morning	Locomotion
2019/07/29	CH2271	Male	Two y/o	Treatment (Wk 3)	Treatment	Feed Day	09:35	09:00 - 09:59	Late Morning	Appetitive behaviour
2019/07/29	CH2271	Male	Two y/o	Treatment (Wk 3)	Treatment	Feed Day	09:40	09:00 - 09:59	Late Morning	Interspecific behaviour
2019/07/29	CH2271	Male	Two y/o	Treatment (Wk 3)	Treatment	Feed Day	09:45	09:00 - 09:59	Late Morning	Inactive
2019/07/29	CH2271	Male	Two y/o	Treatment (Wk 3)	Treatment	Feed Day	09:50	09:00 - 09:59	Late Morning	Interspecific behaviour
2019/07/29	CH2271	Male	Two y/o	Treatment (Wk 3)	Treatment	Feed Day	09:55	09:00 - 09:59	Late Morning	Standing
2019/07/29	CH2271	Male	Two y/o	Treatment (Wk 3)	Treatment	Feed Day	10:00	10:00 - 10:59	Late Morning	Inactive
2019/07/29	CH2271	Male	Two y/o	Treatment (Wk 3)	Treatment	Feed Day	10:05	10:00 - 10:59	Late Morning	Inactive
2019/07/29	CH2271	Male	Two y/o	Treatment (Wk 3)	Treatment	Feed Day	10:10	10:00 - 10:59	Late Morning	Locomotion
2019/07/29	CH2271	Male	Two y/o	Treatment (Wk 3)	Treatment	Feed Day	10:15	10:00 - 10:59	Late Morning	Interspecific behaviour
2019/07/29	CH2271	Male	Two y/o	Treatment (Wk 3)	Treatment	Feed Day	10:20	10:00 - 10:59	Late Morning	Scent-marking
2019/07/29	CH2271	Male	Two y/o	Treatment (Wk 3)	Treatment	Feed Day	10:25	10:00 - 10:59	Late Morning	Inactive
2019/08/01	CH2276	Female	Two y/o	Treatment (Wk 1)	Treatment	Feed Day	11:50	11:00 - 11:59	Late Morning	Inactive
2019/08/01	CH2277	Female	Two y/o	Treatment (Wk 1)	Treatment	Feed Day	11:50	11:00 - 11:59	Late Morning	Inactive
2019/08/01	CH2276	Female	Two y/o	Treatment (Wk 1)	Treatment	Feed Day	11:55	11:00 - 11:59	Late Morning	Inactive
2019/08/01	CH2277	Female	Two y/o	Treatment (Wk 1)	Treatment	Feed Day	11:55	11:00 - 11:59	Late Morning	Inactive
2019/08/01	CH2276	Female	Two y/o	Treatment (Wk 1)	Treatment	Feed Day	12:00	12:00 - 12:59	Afternoon	Inactive
2019/08/01	CH2277	Female	Two y/o	Treatment (Wk 1)	Treatment	Feed Day	12:00	12:00 - 12:59	Afternoon	Inactive
2019/08/01	CH2276	Female	Two y/o	Treatment (Wk 1)	Treatment	Feed Day	12:05	12:00 - 12:59	Afternoon	Inactive
2019/08/01	CH2277	Female	Two y/o	Treatment (Wk 1)	Treatment	Feed Day	12:05	12:00 - 12:59	Afternoon	Inactive
2019/08/01	CH2276	Female	Two y/o	Treatment (Wk 1)	Treatment	Feed Day	12:10	12:00 - 12:59	Afternoon	Scent-marking
2019/08/01	CH2277	Female	Two y/o	Treatment (Wk 1)	Treatment	Feed Day	12:10	12:00 - 12:59	Afternoon	Scent-marking
2019/08/01	CH2276	Female	Two y/o	Treatment (Wk 1)	Treatment	Feed Day	12:15	12:00 - 12:59	Afternoon	Inactive
2019/08/01	CH2277	Female	Two y/o	Treatment (Wk 1)	Treatment	Feed Day	12:15	12:00 - 12:59	Afternoon	Inactive
2019/08/01	CH2276	Female	Two y/o	Treatment (Wk 1)	Treatment	Feed Day	12:20	12:00 - 12:59	Afternoon	Inactive
2019/08/01	CH2277	Female	Two y/o	Treatment (Wk 1)	Treatment	Feed Day	12:20	12:00 - 12:59	Afternoon	Inactive
2019/08/01	CH2276	Female	Two y/o	Treatment (Wk 1)	Treatment	Feed Day	12:25	12:00 - 12:59	Afternoon	Inactive
2019/08/01	CH2277	Female	Two y/o	Treatment (Wk 1)	Treatment	Feed Day	12:25	12:00 - 12:59	Afternoon	Inactive
2019/08/01	CH2276	Female	Two y/o	Treatment (Wk 1)	Treatment	Feed Day	12:30	12:00 - 12:59	Afternoon	Stereotypical

2019/08/01	CH2277	Female	Two y/o	Treatment (Wk 1)	Treatment	Feed Day	12:30	12:00 - 12:59	Afternoon	Stereotypical
2019/08/01	CH2276	Female	Two y/o	Treatment (Wk 1)	Treatment	Feed Day	12:35	12:00 - 12:59	Afternoon	Interspecific behaviour
2019/08/01	CH2277	Female	Two y/o	Treatment (Wk 1)	Treatment	Feed Day	12:35	12:00 - 12:59	Afternoon	Interspecific behaviour
2019/08/01	CH2276	Female	Two y/o	Treatment (Wk 1)	Treatment	Feed Day	12:40	12:00 - 12:59	Afternoon	Inactive
2019/08/01	CH2277	Female	Two y/o	Treatment (Wk 1)	Treatment	Feed Day	12:40	12:00 - 12:59	Afternoon	Inactive
2019/08/01	CH2276	Female	Two y/o	Treatment (Wk 1)	Treatment	Feed Day	12:45	12:00 - 12:59	Afternoon	Attention
2019/08/01	CH2277	Female	Two y/o	Treatment (Wk 1)	Treatment	Feed Day	12:45	12:00 - 12:59	Afternoon	Attention
2019/08/03	CH2276	Female	Two y/o	Treatment (Wk 1)	Treatment	Feed Day	11:50	11:00 - 11:59	Late Morning	Inactive
2019/08/03	CH2277	Female	Two y/o	Treatment (Wk 1)	Treatment	Feed Day	11:50	11:00 - 11:59	Late Morning	Inactive
2019/08/03	CH2276	Female	Two y/o	Treatment (Wk 1)	Treatment	Feed Day	11:55	11:00 - 11:59	Late Morning	Scent-marking
2019/08/03	CH2277	Female	Two y/o	Treatment (Wk 1)	Treatment	Feed Day	11:55	11:00 - 11:59	Late Morning	Standing
2019/08/03	CH2276	Female	Two y/o	Treatment (Wk 1)	Treatment	Feed Day	12:00	12:00 - 12:59	Afternoon	Inactive
2019/08/03	CH2277	Female	Two y/o	Treatment (Wk 1)	Treatment	Feed Day	12:00	12:00 - 12:59	Afternoon	Inactive
2019/08/03	CH2276	Female	Two y/o	Treatment (Wk 1)	Treatment	Feed Day	12:05	12:00 - 12:59	Afternoon	Inactive
2019/08/03	CH2277	Female	Two y/o	Treatment (Wk 1)	Treatment	Feed Day	12:05	12:00 - 12:59	Afternoon	Inactive
2019/08/03	CH2276	Female	Two y/o	Treatment (Wk 1)	Treatment	Feed Day	12:10	12:00 - 12:59	Afternoon	Inactive
2019/08/03	CH2277	Female	Two y/o	Treatment (Wk 1)	Treatment	Feed Day	12:10	12:00 - 12:59	Afternoon	Inactive
2019/08/03	CH2276	Female	Two y/o	Treatment (Wk 1)	Treatment	Feed Day	12:15	12:00 - 12:59	Afternoon	Inactive
2019/08/03	CH2277	Female	Two y/o	Treatment (Wk 1)	Treatment	Feed Day	12:15	12:00 - 12:59	Afternoon	Inactive
2019/08/03	CH2276	Female	Two y/o	Treatment (Wk 1)	Treatment	Feed Day	12:20	12:00 - 12:59	Afternoon	Interspecific behaviour
2019/08/03	CH2277	Female	Two y/o	Treatment (Wk 1)	Treatment	Feed Day	12:20	12:00 - 12:59	Afternoon	Interspecific behaviour
2019/08/03	CH2276	Female	Two y/o	Treatment (Wk 1)	Treatment	Feed Day	12:25	12:00 - 12:59	Afternoon	Interspecific behaviour
2019/08/03	CH2277	Female	Two y/o	Treatment (Wk 1)	Treatment	Feed Day	12:25	12:00 - 12:59	Afternoon	Interspecific behaviour
2019/08/03	CH2276	Female	Two y/o	Treatment (Wk 1)	Treatment	Feed Day	12:30	12:00 - 12:59	Afternoon	Interspecific behaviour
2019/08/03	CH2277	Female	Two y/o	Treatment (Wk 1)	Treatment	Feed Day	12:30	12:00 - 12:59	Afternoon	Interspecific behaviour
2019/08/03	CH2276	Female	Two y/o	Treatment (Wk 1)	Treatment	Feed Day	12:35	12:00 - 12:59	Afternoon	Interspecific behaviour
2019/08/03	CH2277	Female	Two y/o	Treatment (Wk 1)	Treatment	Feed Day	12:35	12:00 - 12:59	Afternoon	Interspecific behaviour
2019/08/03	CH2276	Female	Two y/o	Treatment (Wk 1)	Treatment	Feed Day	12:40	12:00 - 12:59	Afternoon	Inactive
2019/08/03	CH2277	Female	Two y/o	Treatment (Wk 1)	Treatment	Feed Day	12:40	12:00 - 12:59	Afternoon	Inactive
2019/08/03	CH2276	Female	Two y/o	Treatment (Wk 1)	Treatment	Feed Day	12:45	12:00 - 12:59	Afternoon	Inactive
2019/08/03	CH2277	Female	Two y/o	Treatment (Wk 1)	Treatment	Feed Day	12:45	12:00 - 12:59	Afternoon	Inactive
2019/08/05	CH2276	Female	Two y/o	Treatment (Wk 1)	Treatment	Feed Day	12:05	12:00 - 12:59	Afternoon	Inactive
2019/08/05	CH2277	Female	Two y/o	Treatment (Wk 1)	Treatment	Feed Day	12:05	12:00 - 12:59	Afternoon	Inactive
2019/08/05	CH2276	Female	Two y/o	Treatment (Wk 1)	Treatment	Feed Day	12:10	12:00 - 12:59	Afternoon	Inactive
2019/08/05	CH2277	Female	Two y/o	Treatment (Wk 1)	Treatment	Feed Day	12:10	12:00 - 12:59	Afternoon	Scent-marking
2019/08/05	CH2276	Female	Two y/o	Treatment (Wk 1)	Treatment	Feed Day	12:15	12:00 - 12:59	Afternoon	Inactive
2019/08/05	CH2277	Female	Two y/o	Treatment (Wk 1)	Treatment	Feed Day	12:15	12:00 - 12:59	Afternoon	Inactive

2019/08/05	CH2276	Female	Two y/o	Treatment (Wk 1)	Treatment	Feed Day	12:20	12:00 - 12:59	Afternoon	Locomotion
2019/08/05	CH2277	Female	Two y/o	Treatment (Wk 1)	Treatment	Feed Day	12:20	12:00 - 12:59	Afternoon	Interspecific behaviour
2019/08/05	CH2276	Female	Two y/o	Treatment (Wk 1)	Treatment	Feed Day	12:25	12:00 - 12:59	Afternoon	Inactive
2019/08/05	CH2277	Female	Two y/o	Treatment (Wk 1)	Treatment	Feed Day	12:25	12:00 - 12:59	Afternoon	Inactive
2019/08/05	CH2276	Female	Two y/o	Treatment (Wk 1)	Treatment	Feed Day	12:30	12:00 - 12:59	Afternoon	Inactive
2019/08/05	CH2277	Female	Two y/o	Treatment (Wk 1)	Treatment	Feed Day	12:30	12:00 - 12:59	Afternoon	Inactive
2019/08/05	CH2276	Female	Two y/o	Treatment (Wk 1)	Treatment	Feed Day	12:35	12:00 - 12:59	Afternoon	Inactive
2019/08/05	CH2277	Female	Two y/o	Treatment (Wk 1)	Treatment	Feed Day	12:35	12:00 - 12:59	Afternoon	Inactive
2019/08/05	CH2276	Female	Two y/o	Treatment (Wk 1)	Treatment	Feed Day	12:40	12:00 - 12:59	Afternoon	Inactive
2019/08/05	CH2277	Female	Two y/o	Treatment (Wk 1)	Treatment	Feed Day	12:40	12:00 - 12:59	Afternoon	Inactive
2019/08/07	CH2276	Female	Two y/o	Treatment (Wk 2)	Treatment	Fast Day	11:45	11:00 - 11:59	Late Morning	Locomotion
2019/08/07	CH2277	Female	Two y/o	Treatment (Wk 2)	Treatment	Fast Day	11:45	11:00 - 11:59	Late Morning	Locomotion
2019/08/07	CH2276	Female	Two y/o	Treatment (Wk 2)	Treatment	Fast Day	11:50	11:00 - 11:59	Late Morning	Interspecific behaviour
2019/08/07	CH2277	Female	Two y/o	Treatment (Wk 2)	Treatment	Fast Day	11:50	11:00 - 11:59	Late Morning	Scent-marking
2019/08/07	CH2276	Female	Two y/o	Treatment (Wk 2)	Treatment	Fast Day	11:55	11:00 - 11:59	Late Morning	Attention
2019/08/07	CH2277	Female	Two y/o	Treatment (Wk 2)	Treatment	Fast Day	11:55	11:00 - 11:59	Late Morning	Attention
2019/08/07	CH2276	Female	Two y/o	Treatment (Wk 2)	Treatment	Fast Day	12:00	12:00 - 12:59	Afternoon	Inactive
2019/08/07	CH2277	Female	Two y/o	Treatment (Wk 2)	Treatment	Fast Day	12:00	12:00 - 12:59	Afternoon	Inactive
2019/08/07	CH2276	Female	Two y/o	Treatment (Wk 2)	Treatment	Fast Day	12:05	12:00 - 12:59	Afternoon	Inactive
2019/08/07	CH2277	Female	Two y/o	Treatment (Wk 2)	Treatment	Fast Day	12:05	12:00 - 12:59	Afternoon	Inactive
2019/08/07	CH2276	Female	Two y/o	Treatment (Wk 2)	Treatment	Fast Day	12:10	12:00 - 12:59	Afternoon	Inactive
2019/08/07	CH2277	Female	Two y/o	Treatment (Wk 2)	Treatment	Fast Day	12:10	12:00 - 12:59	Afternoon	Inactive
2019/08/07	CH2276	Female	Two y/o	Treatment (Wk 2)	Treatment	Fast Day	12:15	12:00 - 12:59	Afternoon	Inactive
2019/08/07	CH2277	Female	Two y/o	Treatment (Wk 2)	Treatment	Fast Day	12:15	12:00 - 12:59	Afternoon	Inactive
2019/08/07	CH2276	Female	Two y/o	Treatment (Wk 2)	Treatment	Fast Day	12:20	12:00 - 12:59	Afternoon	Inactive
2019/08/07	CH2277	Female	Two y/o	Treatment (Wk 2)	Treatment	Fast Day	12:20	12:00 - 12:59	Afternoon	Inactive
2019/08/09	CH2276	Female	Two y/o	Treatment (Wk 2)	Treatment	Feed Day	12:40	12:00 - 12:59	Afternoon	Inactive
2019/08/09	CH2277	Female	Two y/o	Treatment (Wk 2)	Treatment	Feed Day	12:40	12:00 - 12:59	Afternoon	Inactive
2019/08/09	CH2276	Female	Two y/o	Treatment (Wk 2)	Treatment	Feed Day	12:45	12:00 - 12:59	Afternoon	Inactive
2019/08/09	CH2277	Female	Two y/o	Treatment (Wk 2)	Treatment	Feed Day	12:45	12:00 - 12:59	Afternoon	Inactive
2019/08/09	CH2276	Female	Two y/o	Treatment (Wk 2)	Treatment	Feed Day	12:50	12:00 - 12:59	Afternoon	Inactive
2019/08/09	CH2277	Female	Two y/o	Treatment (Wk 2)	Treatment	Feed Day	12:50	12:00 - 12:59	Afternoon	Inactive
2019/08/09	CH2276	Female	Two y/o	Treatment (Wk 2)	Treatment	Feed Day	12:55	12:00 - 12:59	Afternoon	Inactive
2019/08/09	CH2277	Female	Two y/o	Treatment (Wk 2)	Treatment	Feed Day	12:55	12:00 - 12:59	Afternoon	Inactive
2019/08/09	CH2276	Female	Two y/o	Treatment (Wk 2)	Treatment	Feed Day	13:00	13:00 - 13:59	Afternoon	Inactive
2019/08/09	CH2277	Female	Two y/o	Treatment (Wk 2)	Treatment	Feed Day	13:00	13:00 - 13:59	Afternoon	Inactive
2019/08/09	CH2276	Female	Two y/o	Treatment (Wk 2)	Treatment	Feed Day	13:05	13:00 - 13:59	Afternoon	Inactive

2019/08/09	CH2277	Female	Two y/o	Treatment (Wk 2)	Treatment	Feed Day	13:05	13:00 - 13:59	Afternoon	Inactive
2019/08/09	CH2276	Female	Two y/o	Treatment (Wk 2)	Treatment	Feed Day	13:10	13:00 - 13:59	Afternoon	Inactive
2019/08/09	CH2277	Female	Two y/o	Treatment (Wk 2)	Treatment	Feed Day	13:10	13:00 - 13:59	Afternoon	Auto-grooming
2019/08/09	CH2276	Female	Two y/o	Treatment (Wk 2)	Treatment	Feed Day	13:15	13:00 - 13:59	Afternoon	Inactive
2019/08/09	CH2277	Female	Two y/o	Treatment (Wk 2)	Treatment	Feed Day	13:15	13:00 - 13:59	Afternoon	Locomotion
2019/08/11	CH2276	Female	Two y/o	Treatment (Wk 2)	Treatment	Fast Day	11:55	11:00 - 11:59	Late Morning	Inactive
2019/08/11	CH2277	Female	Two y/o	Treatment (Wk 2)	Treatment	Fast Day	11:55	11:00 - 11:59	Late Morning	Inactive
2019/08/11	CH2276	Female	Two y/o	Treatment (Wk 2)	Treatment	Fast Day	12:00	12:00 - 12:59	Afternoon	Inactive
2019/08/11	CH2277	Female	Two y/o	Treatment (Wk 2)	Treatment	Fast Day	12:00	12:00 - 12:59	Afternoon	Inactive
2019/08/11	CH2276	Female	Two y/o	Treatment (Wk 2)	Treatment	Fast Day	12:05	12:00 - 12:59	Afternoon	Inactive
2019/08/11	CH2277	Female	Two y/o	Treatment (Wk 2)	Treatment	Fast Day	12:05	12:00 - 12:59	Afternoon	Inactive
2019/08/11	CH2276	Female	Two y/o	Treatment (Wk 2)	Treatment	Fast Day	12:10	12:00 - 12:59	Afternoon	Appetitive behaviour
2019/08/11	CH2277	Female	Two y/o	Treatment (Wk 2)	Treatment	Fast Day	12:10	12:00 - 12:59	Afternoon	Appetitive behaviour
2019/08/11	CH2276	Female	Two y/o	Treatment (Wk 2)	Treatment	Fast Day	12:15	12:00 - 12:59	Afternoon	Agnostic behaviour
2019/08/11	CH2277	Female	Two y/o	Treatment (Wk 2)	Treatment	Fast Day	12:15	12:00 - 12:59	Afternoon	Agnostic behaviour
2019/08/11	CH2276	Female	Two y/o	Treatment (Wk 2)	Treatment	Fast Day	12:20	12:00 - 12:59	Afternoon	Inactive
2019/08/11	CH2277	Female	Two y/o	Treatment (Wk 2)	Treatment	Fast Day	12:20	12:00 - 12:59	Afternoon	Inactive
2019/08/11	CH2276	Female	Two y/o	Treatment (Wk 2)	Treatment	Fast Day	12:25	12:00 - 12:59	Afternoon	Interspecific behaviour
2019/08/11	CH2277	Female	Two y/o	Treatment (Wk 2)	Treatment	Fast Day	12:25	12:00 - 12:59	Afternoon	Interspecific behaviour
2019/08/11	CH2276	Female	Two y/o	Treatment (Wk 2)	Treatment	Fast Day	12:30	12:00 - 12:59	Afternoon	Appetitive behaviour
2019/08/11	CH2277	Female	Two y/o	Treatment (Wk 2)	Treatment	Fast Day	12:30	12:00 - 12:59	Afternoon	Appetitive behaviour
2019/08/11	CH2276	Female	Two y/o	Treatment (Wk 2)	Treatment	Fast Day	12:35	12:00 - 12:59	Afternoon	Locomotion
2019/08/11	CH2277	Female	Two y/o	Treatment (Wk 2)	Treatment	Fast Day	12:35	12:00 - 12:59	Afternoon	Appetitive behaviour
2019/08/11	CH2276	Female	Two y/o	Treatment (Wk 2)	Treatment	Fast Day	12:40	12:00 - 12:59	Afternoon	Inactive
2019/08/11	CH2277	Female	Two y/o	Treatment (Wk 2)	Treatment	Fast Day	12:40	12:00 - 12:59	Afternoon	Appetitive behaviour
2019/08/11	CH2276	Female	Two y/o	Treatment (Wk 2)	Treatment	Fast Day	12:45	12:00 - 12:59	Afternoon	Locomotion
2019/08/11	CH2277	Female	Two y/o	Treatment (Wk 2)	Treatment	Fast Day	12:45	12:00 - 12:59	Afternoon	Locomotion
2019/08/11	CH2276	Female	Two y/o	Treatment (Wk 2)	Treatment	Fast Day	12:50	12:00 - 12:59	Afternoon	Locomotion
2019/08/11	CH2277	Female	Two y/o	Treatment (Wk 2)	Treatment	Fast Day	12:50	12:00 - 12:59	Afternoon	Locomotion
2019/08/13	CH2276	Female	Two y/o	Treatment (Wk 2)	Treatment	Feed Day	12:00	12:00 - 12:59	Afternoon	Interspecific behaviour
2019/08/13	CH2277	Female	Two y/o	Treatment (Wk 2)	Treatment	Feed Day	12:00	12:00 - 12:59	Afternoon	Interspecific behaviour
2019/08/13	CH2276	Female	Two y/o	Treatment (Wk 2)	Treatment	Feed Day	12:05	12:00 - 12:59	Afternoon	Inactive
2019/08/13	CH2277	Female	Two y/o	Treatment (Wk 2)	Treatment	Feed Day	12:05	12:00 - 12:59	Afternoon	Inactive
2019/08/13	CH2276	Female	Two y/o	Treatment (Wk 2)	Treatment	Feed Day	12:10	12:00 - 12:59	Afternoon	Inactive
2019/08/13	CH2277	Female	Two y/o	Treatment (Wk 2)	Treatment	Feed Day	12:10	12:00 - 12:59	Afternoon	Inactive
2019/08/13	CH2276	Female	Two y/o	Treatment (Wk 2)	Treatment	Feed Day	12:15	12:00 - 12:59	Afternoon	Interspecific behaviour
2019/08/13	CH2277	Female	Two y/o	Treatment (Wk 2)	Treatment	Feed Day	12:15	12:00 - 12:59	Afternoon	Interspecific behaviour

2019/08/13	CH2276	Female	Two y/o	Treatment (Wk 2)	Treatment	Feed Day	12:20	12:00 - 12:59	Afternoon	Interspecific behaviour
2019/08/13	CH2277	Female	Two y/o	Treatment (Wk 2)	Treatment	Feed Day	12:20	12:00 - 12:59	Afternoon	Interspecific behaviour
2019/08/13	CH2276	Female	Two y/o	Treatment (Wk 2)	Treatment	Feed Day	12:25	12:00 - 12:59	Afternoon	Inactive
2019/08/13	CH2277	Female	Two y/o	Treatment (Wk 2)	Treatment	Feed Day	12:25	12:00 - 12:59	Afternoon	Inactive
2019/08/13	CH2276	Female	Two y/o	Treatment (Wk 2)	Treatment	Feed Day	12:30	12:00 - 12:59	Afternoon	Inactive
2019/08/13	CH2277	Female	Two y/o	Treatment (Wk 2)	Treatment	Feed Day	12:30	12:00 - 12:59	Afternoon	Inactive
2019/08/13	CH2276	Female	Two y/o	Treatment (Wk 2)	Treatment	Feed Day	12:35	12:00 - 12:59	Afternoon	Inactive
2019/08/13	CH2277	Female	Two y/o	Treatment (Wk 2)	Treatment	Feed Day	12:35	12:00 - 12:59	Afternoon	Inactive
2019/08/13	CH2276	Female	Two y/o	Treatment (Wk 2)	Treatment	Feed Day	12:40	12:00 - 12:59	Afternoon	Inactive
2019/08/13	CH2277	Female	Two y/o	Treatment (Wk 2)	Treatment	Feed Day	12:40	12:00 - 12:59	Afternoon	Inactive
2019/08/13	CH2276	Female	Two y/o	Treatment (Wk 2)	Treatment	Feed Day	12:45	12:00 - 12:59	Afternoon	Inactive
2019/08/13	CH2277	Female	Two y/o	Treatment (Wk 2)	Treatment	Feed Day	12:45	12:00 - 12:59	Afternoon	Inactive
2019/08/13	CH2276	Female	Two y/o	Treatment (Wk 2)	Treatment	Feed Day	12:50	12:00 - 12:59	Afternoon	Inactive
2019/08/13	CH2277	Female	Two y/o	Treatment (Wk 2)	Treatment	Feed Day	12:50	12:00 - 12:59	Afternoon	Inactive
2019/08/13	CH2276	Female	Two y/o	Treatment (Wk 2)	Treatment	Feed Day	12:55	12:00 - 12:59	Afternoon	Inactive
2019/08/13	CH2277	Female	Two y/o	Treatment (Wk 2)	Treatment	Feed Day	12:55	12:00 - 12:59	Afternoon	Inactive
2019/08/14	CH2276	Female	Two y/o	Treatment (Wk 3)	Treatment	Fast Day	11:55	11:00 - 11:59	Late Morning	Inactive
2019/08/14	CH2277	Female	Two y/o	Treatment (Wk 3)	Treatment	Fast Day	11:55	11:00 - 11:59	Late Morning	Inactive
2019/08/14	CH2276	Female	Two y/o	Treatment (Wk 3)	Treatment	Fast Day	12:00	12:00 - 12:59	Afternoon	Inactive
2019/08/14	CH2277	Female	Two y/o	Treatment (Wk 3)	Treatment	Fast Day	12:00	12:00 - 12:59	Afternoon	Inactive
2019/08/14	CH2276	Female	Two y/o	Treatment (Wk 3)	Treatment	Fast Day	12:05	12:00 - 12:59	Afternoon	Inactive
2019/08/14	CH2277	Female	Two y/o	Treatment (Wk 3)	Treatment	Fast Day	12:05	12:00 - 12:59	Afternoon	Inactive
2019/08/14	CH2276	Female	Two y/o	Treatment (Wk 3)	Treatment	Fast Day	12:10	12:00 - 12:59	Afternoon	Inactive
2019/08/14	CH2277	Female	Two y/o	Treatment (Wk 3)	Treatment	Fast Day	12:10	12:00 - 12:59	Afternoon	Inactive
2019/08/14	CH2276	Female	Two y/o	Treatment (Wk 3)	Treatment	Fast Day	12:15	12:00 - 12:59	Afternoon	Inactive
2019/08/14	CH2277	Female	Two y/o	Treatment (Wk 3)	Treatment	Fast Day	12:15	12:00 - 12:59	Afternoon	Inactive
2019/08/14	CH2276	Female	Two y/o	Treatment (Wk 3)	Treatment	Fast Day	12:20	12:00 - 12:59	Afternoon	Inactive
2019/08/14	CH2277	Female	Two y/o	Treatment (Wk 3)	Treatment	Fast Day	12:20	12:00 - 12:59	Afternoon	Inactive
2019/08/14	CH2276	Female	Two y/o	Treatment (Wk 3)	Treatment	Fast Day	12:25	12:00 - 12:59	Afternoon	Inactive
2019/08/14	CH2277	Female	Two y/o	Treatment (Wk 3)	Treatment	Fast Day	12:25	12:00 - 12:59	Afternoon	Inactive
2019/08/14	CH2276	Female	Two y/o	Treatment (Wk 3)	Treatment	Fast Day	12:30	12:00 - 12:59	Afternoon	Inactive
2019/08/14	CH2277	Female	Two y/o	Treatment (Wk 3)	Treatment	Fast Day	12:30	12:00 - 12:59	Afternoon	Inactive
2019/08/14	CH2276	Female	Two y/o	Treatment (Wk 3)	Treatment	Fast Day	12:35	12:00 - 12:59	Afternoon	Inactive
2019/08/14	CH2277	Female	Two y/o	Treatment (Wk 3)	Treatment	Fast Day	12:35	12:00 - 12:59	Afternoon	Inactive
2019/08/14	CH2276	Female	Two y/o	Treatment (Wk 3)	Treatment	Fast Day	12:40	12:00 - 12:59	Afternoon	Inactive
2019/08/14	CH2277	Female	Two y/o	Treatment (Wk 3)	Treatment	Fast Day	12:40	12:00 - 12:59	Afternoon	Inactive
2019/08/14	CH2276	Female	Two y/o	Treatment (Wk 3)	Treatment	Fast Day	12:45	12:00 - 12:59	Afternoon	Inactive

2019/08/14	CH2277	Female	Two y/o	Treatment (Wk 3)	Treatment	Fast Day	12:45	12:00 - 12:59	Afternoon	Inactive
2019/08/14	CH2276	Female	Two y/o	Treatment (Wk 3)	Treatment	Fast Day	12:50	12:00 - 12:59	Afternoon	Inactive
2019/08/14	CH2277	Female	Two y/o	Treatment (Wk 3)	Treatment	Fast Day	12:50	12:00 - 12:59	Afternoon	Inactive
2019/08/19	CH2276	Female	Two y/o	Treatment (Wk 3)	Treatment	Feed Day	11:50	11:00 - 11:59	Late Morning	Inactive
2019/08/19	CH2277	Female	Two y/o	Treatment (Wk 3)	Treatment	Feed Day	11:50	11:00 - 11:59	Late Morning	Inactive
2019/08/19	CH2276	Female	Two y/o	Treatment (Wk 3)	Treatment	Feed Day	11:55	11:00 - 11:59	Late Morning	Scent-marking
2019/08/19	CH2277	Female	Two y/o	Treatment (Wk 3)	Treatment	Feed Day	11:55	11:00 - 11:59	Late Morning	Inactive
2019/08/19	CH2276	Female	Two y/o	Treatment (Wk 3)	Treatment	Feed Day	12:00	12:00 - 12:59	Afternoon	Interspecific behaviour
2019/08/19	CH2277	Female	Two y/o	Treatment (Wk 3)	Treatment	Feed Day	12:00	12:00 - 12:59	Afternoon	Interspecific behaviour
2019/08/19	CH2276	Female	Two y/o	Treatment (Wk 3)	Treatment	Feed Day	12:05	12:00 - 12:59	Afternoon	Inactive
2019/08/19	CH2277	Female	Two y/o	Treatment (Wk 3)	Treatment	Feed Day	12:05	12:00 - 12:59	Afternoon	Inactive
2019/08/19	CH2276	Female	Two y/o	Treatment (Wk 3)	Treatment	Feed Day	12:10	12:00 - 12:59	Afternoon	Inactive
2019/08/19	CH2277	Female	Two y/o	Treatment (Wk 3)	Treatment	Feed Day	12:10	12:00 - 12:59	Afternoon	Auto-grooming
2019/08/19	CH2276	Female	Two y/o	Treatment (Wk 3)	Treatment	Feed Day	12:15	12:00 - 12:59	Afternoon	Inactive
2019/08/19	CH2277	Female	Two y/o	Treatment (Wk 3)	Treatment	Feed Day	12:15	12:00 - 12:59	Afternoon	Inactive
2019/08/19	CH2276	Female	Two y/o	Treatment (Wk 3)	Treatment	Feed Day	12:20	12:00 - 12:59	Afternoon	Inactive
2019/08/19	CH2277	Female	Two y/o	Treatment (Wk 3)	Treatment	Feed Day	12:20	12:00 - 12:59	Afternoon	Inactive
2019/08/19	CH2276	Female	Two y/o	Treatment (Wk 3)	Treatment	Feed Day	12:25	12:00 - 12:59	Afternoon	Interspecific behaviour
2019/08/19	CH2277	Female	Two y/o	Treatment (Wk 3)	Treatment	Feed Day	12:25	12:00 - 12:59	Afternoon	Interspecific behaviour
2019/08/19	CH2276	Female	Two y/o	Treatment (Wk 3)	Treatment	Feed Day	12:30	12:00 - 12:59	Afternoon	Inactive
2019/08/19	CH2277	Female	Two y/o	Treatment (Wk 3)	Treatment	Feed Day	12:30	12:00 - 12:59	Afternoon	Inactive
2019/08/19	CH2276	Female	Two y/o	Treatment (Wk 3)	Treatment	Feed Day	12:35	12:00 - 12:59	Afternoon	Inactive
2019/08/19	CH2277	Female	Two y/o	Treatment (Wk 3)	Treatment	Feed Day	12:35	12:00 - 12:59	Afternoon	Inactive
2019/08/19	CH2276	Female	Two y/o	Treatment (Wk 3)	Treatment	Feed Day	12:40	12:00 - 12:59	Afternoon	Inactive
2019/08/19	CH2277	Female	Two y/o	Treatment (Wk 3)	Treatment	Feed Day	12:40	12:00 - 12:59	Afternoon	Inactive
2019/08/19	CH2276	Female	Two y/o	Treatment (Wk 3)	Treatment	Feed Day	12:45	12:00 - 12:59	Afternoon	Inactive
2019/08/19	CH2277	Female	Two y/o	Treatment (Wk 3)	Treatment	Feed Day	12:45	12:00 - 12:59	Afternoon	Inactive
2019/08/20	CH2276	Female	Two y/o	Treatment (Wk 3)	Treatment	Feed Day	11:45	11:00 - 11:59	Late Morning	Inactive
2019/08/20	CH2277	Female	Two y/o	Treatment (Wk 3)	Treatment	Feed Day	11:45	11:00 - 11:59	Late Morning	Inactive
2019/08/20	CH2276	Female	Two y/o	Treatment (Wk 3)	Treatment	Feed Day	11:50	11:00 - 11:59	Late Morning	Inactive
2019/08/20	CH2277	Female	Two y/o	Treatment (Wk 3)	Treatment	Feed Day	11:50	11:00 - 11:59	Late Morning	Inactive
2019/08/20	CH2276	Female	Two y/o	Treatment (Wk 3)	Treatment	Feed Day	11:55	11:00 - 11:59	Late Morning	Interspecific behaviour
2019/08/20	CH2277	Female	Two y/o	Treatment (Wk 3)	Treatment	Feed Day	11:55	11:00 - 11:59	Late Morning	Interspecific behaviour
2019/08/20	CH2276	Female	Two y/o	Treatment (Wk 3)	Treatment	Feed Day	12:00	12:00 - 12:59	Afternoon	Inactive
2019/08/20	CH2277	Female	Two y/o	Treatment (Wk 3)	Treatment	Feed Day	12:00	12:00 - 12:59	Afternoon	Inactive
2019/08/22	CH2271	Male	Two y/o	Control	Control	Feed Day	12:20	12:00 - 12:59	Afternoon	Inactive
2019/08/22	CH2271	Male	Two y/o	Control	Control	Feed Day	12:25	12:00 - 12:59	Afternoon	Locomotion

2019/08/22	CH2271	Male	Two y/o	Control	Control	Feed Day	12:30	12:00 - 12:59	Afternoon	Inactive
2019/08/22	CH2271	Male	Two y/o	Control	Control	Feed Day	12:35	12:00 - 12:59	Afternoon	Inactive
2019/08/22	CH2271	Male	Two y/o	Control	Control	Feed Day	12:40	12:00 - 12:59	Afternoon	Appetitive behaviour
2019/08/22	CH2271	Male	Two y/o	Control	Control	Feed Day	12:45	12:00 - 12:59	Afternoon	Appetitive behaviour
2019/08/22	CH2271	Male	Two y/o	Control	Control	Feed Day	12:50	12:00 - 12:59	Afternoon	Appetitive behaviour
2019/08/22	CH2271	Male	Two y/o	Control	Control	Feed Day	12:55	12:00 - 12:59	Afternoon	Maintenance
2019/08/22	CH2271	Male	Two y/o	Control	Control	Feed Day	13:00	13:00 - 13:59	Afternoon	Inactive
2019/08/22	CH2271	Male	Two y/o	Control	Control	Feed Day	13:05	13:00 - 13:59	Afternoon	Inactive
2019/08/22	CH2271	Male	Two y/o	Control	Control	Feed Day	13:10	13:00 - 13:59	Afternoon	Inactive
2019/08/22	CH2276	Female	Two y/o	Control	Control	Fast Day	13:10	13:00 - 13:59	Afternoon	Inactive
2019/08/22	CH2277	Female	Two y/o	Control	Control	Fast Day	13:10	13:00 - 13:59	Afternoon	Inactive
2019/08/22	CH2271	Male	Two y/o	Control	Control	Feed Day	13:15	13:00 - 13:59	Afternoon	Inactive
2019/08/22	CH2276	Female	Two y/o	Control	Control	Fast Day	13:15	13:00 - 13:59	Afternoon	Interspecific behaviour
2019/08/22	CH2277	Female	Two y/o	Control	Control	Fast Day	13:15	13:00 - 13:59	Afternoon	Locomotion
2019/08/22	CH2276	Female	Two y/o	Control	Control	Fast Day	13:20	13:00 - 13:59	Afternoon	Affiliative behaviour
2019/08/22	CH2277	Female	Two y/o	Control	Control	Fast Day	13:20	13:00 - 13:59	Afternoon	Affiliative behaviour
2019/08/22	CH2276	Female	Two y/o	Control	Control	Fast Day	13:25	13:00 - 13:59	Afternoon	Inactive
2019/08/22	CH2277	Female	Two y/o	Control	Control	Fast Day	13:25	13:00 - 13:59	Afternoon	Locomotion
2019/08/22	CH2276	Female	Two y/o	Control	Control	Fast Day	13:30	13:00 - 13:59	Afternoon	Inactive
2019/08/22	CH2277	Female	Two y/o	Control	Control	Fast Day	13:30	13:00 - 13:59	Afternoon	Inactive
2019/08/22	CH2276	Female	Two y/o	Control	Control	Fast Day	13:35	13:00 - 13:59	Afternoon	Inactive
2019/08/22	CH2277	Female	Two y/o	Control	Control	Fast Day	13:35	13:00 - 13:59	Afternoon	Inactive
2019/08/22	CH2276	Female	Two y/o	Control	Control	Fast Day	13:40	13:00 - 13:59	Afternoon	Inactive
2019/08/22	CH2277	Female	Two y/o	Control	Control	Fast Day	13:40	13:00 - 13:59	Afternoon	Inactive
2019/08/22	CH2276	Female	Two y/o	Control	Control	Fast Day	13:45	13:00 - 13:59	Afternoon	Inactive
2019/08/22	CH2277	Female	Two y/o	Control	Control	Fast Day	13:45	13:00 - 13:59	Afternoon	Inactive
2019/08/23	CH2276	Female	Two y/o	Control	Control	Feed Day	10:55	10:00 - 10:59	Late Morning	Inactive
2019/08/23	CH2277	Female	Two y/o	Control	Control	Feed Day	10:55	10:00 - 10:59	Late Morning	Inactive
2019/08/23	CH2276	Female	Two y/o	Control	Control	Feed Day	11:00	11:00 - 11:59	Late Morning	Inactive
2019/08/23	CH2277	Female	Two y/o	Control	Control	Feed Day	11:00	11:00 - 11:59	Late Morning	Inactive
2019/08/23	CH2276	Female	Two y/o	Control	Control	Feed Day	11:05	11:00 - 11:59	Late Morning	Scent-marking
2019/08/23	CH2277	Female	Two y/o	Control	Control	Feed Day	11:05	11:00 - 11:59	Late Morning	Locomotion
2019/08/23	CH2276	Female	Two y/o	Control	Control	Feed Day	11:10	11:00 - 11:59	Late Morning	Affiliative behaviour
2019/08/23	CH2277	Female	Two y/o	Control	Control	Feed Day	11:10	11:00 - 11:59	Late Morning	Affiliative behaviour
2019/08/23	CH2276	Female	Two y/o	Control	Control	Feed Day	11:15	11:00 - 11:59	Late Morning	Attention
2019/08/23	CH2277	Female	Two y/o	Control	Control	Feed Day	11:15	11:00 - 11:59	Late Morning	Auto-grooming
2019/08/23	CH2276	Female	Two y/o	Control	Control	Feed Day	11:20	11:00 - 11:59	Late Morning	Inactive

2019/08/23	CH2277	Female	Two y/o	Control	Control	Feed Day	11:20	11:00 - 11:59	Late Morning	Inactive
2019/08/23	CH2276	Female	Two y/o	Control	Control	Feed Day	11:25	11:00 - 11:59	Late Morning	Inactive
2019/08/23	CH2277	Female	Two y/o	Control	Control	Feed Day	11:25	11:00 - 11:59	Late Morning	Inactive
2019/08/23	CH2276	Female	Two y/o	Control	Control	Feed Day	11:30	11:00 - 11:59	Late Morning	Inactive
2019/08/23	CH2277	Female	Two y/o	Control	Control	Feed Day	11:30	11:00 - 11:59	Late Morning	Inactive
2019/08/23	CH2276	Female	Two y/o	Control	Control	Feed Day	11:35	11:00 - 11:59	Late Morning	Inactive
2019/08/23	CH2277	Female	Two y/o	Control	Control	Feed Day	11:35	11:00 - 11:59	Late Morning	Inactive
2019/08/23	CH2276	Female	Two y/o	Control	Control	Feed Day	11:40	11:00 - 11:59	Late Morning	Inactive
2019/08/23	CH2277	Female	Two y/o	Control	Control	Feed Day	11:40	11:00 - 11:59	Late Morning	Inactive
2019/08/23	CH2276	Female	Two y/o	Control	Control	Feed Day	11:45	11:00 - 11:59	Late Morning	Inactive
2019/08/23	CH2277	Female	Two y/o	Control	Control	Feed Day	11:45	11:00 - 11:59	Late Morning	Inactive
2019/08/23	CH2276	Female	Two y/o	Control	Control	Feed Day	11:50	11:00 - 11:59	Late Morning	Inactive
2019/08/23	CH2277	Female	Two y/o	Control	Control	Feed Day	11:50	11:00 - 11:59	Late Morning	Inactive
2019/08/23	CH2271	Male	Two y/o	Control	Control	Feed Day	11:50	11:00 - 11:59	Late Morning	Inactive
2019/08/23	CH2271	Male	Two y/o	Control	Control	Feed Day	11:55	11:00 - 11:59	Late Morning	Auto-grooming
2019/08/23	CH2271	Male	Two y/o	Control	Control	Feed Day	12:00	12:00 - 12:59	Afternoon	Inactive
2019/08/23	CH2271	Male	Two y/o	Control	Control	Feed Day	12:05	12:00 - 12:59	Afternoon	Inactive
2019/08/23	CH2271	Male	Two y/o	Control	Control	Feed Day	12:10	12:00 - 12:59	Afternoon	Inactive
2019/08/23	CH2271	Male	Two y/o	Control	Control	Feed Day	12:15	12:00 - 12:59	Afternoon	Inactive
2019/08/23	CH2271	Male	Two y/o	Control	Control	Feed Day	12:20	12:00 - 12:59	Afternoon	Inactive
2019/08/23	CH2271	Male	Two y/o	Control	Control	Feed Day	12:25	12:00 - 12:59	Afternoon	Inactive
2019/08/23	CH2271	Male	Two y/o	Control	Control	Feed Day	12:30	12:00 - 12:59	Afternoon	Inactive
2019/08/23	CH2271	Male	Two y/o	Control	Control	Feed Day	12:35	12:00 - 12:59	Afternoon	Inactive
2019/08/23	CH2271	Male	Two y/o	Control	Control	Feed Day	12:40	12:00 - 12:59	Afternoon	Inactive
2019/08/23	CH2271	Male	Two y/o	Control	Control	Feed Day	12:45	12:00 - 12:59	Afternoon	Inactive
2019/08/26	CH2271	Male	Two y/o	Control	Control	Feed Day	11:45	11:00 - 11:59	Late Morning	Locomotion
2019/08/26	CH2271	Male	Two y/o	Control	Control	Feed Day	11:50	11:00 - 11:59	Late Morning	Scent-marking
2019/08/26	CH2271	Male	Two y/o	Control	Control	Feed Day	11:55	11:00 - 11:59	Late Morning	Interspecific behaviour
2019/08/26	CH2271	Male	Two y/o	Control	Control	Feed Day	12:00	12:00 - 12:59	Afternoon	Inactive
2019/08/26	CH2271	Male	Two y/o	Control	Control	Feed Day	12:05	12:00 - 12:59	Afternoon	Inactive
2019/08/26	CH2271	Male	Two y/o	Control	Control	Feed Day	12:10	12:00 - 12:59	Afternoon	Inactive
2019/08/26	CH2271	Male	Two y/o	Control	Control	Feed Day	12:15	12:00 - 12:59	Afternoon	Inactive
2019/08/26	CH2271	Male	Two y/o	Control	Control	Feed Day	12:20	12:00 - 12:59	Afternoon	Inactive
2019/08/26	CH2271	Male	Two y/o	Control	Control	Feed Day	12:25	12:00 - 12:59	Afternoon	Inactive
2019/08/26	CH2271	Male	Two y/o	Control	Control	Feed Day	12:30	12:00 - 12:59	Afternoon	Inactive
2019/08/26	CH2271	Male	Two y/o	Control	Control	Feed Day	12:35	12:00 - 12:59	Afternoon	Inactive
2019/08/26	CH2276	Female	Two y/o	Control	Control	Feed Day	12:35	12:00 - 12:59	Afternoon	Inactive

2019/08/26	CH2277	Female	Two y/o	Control	Control	Feed Day	12:35	12:00 - 12:59	Afternoon	Inactive
2019/08/26	CH2271	Male	Two y/o	Control	Control	Feed Day	12:40	12:00 - 12:59	Afternoon	Inactive
2019/08/26	CH2276	Female	Two y/o	Control	Control	Feed Day	12:40	12:00 - 12:59	Afternoon	Inactive
2019/08/26	CH2277	Female	Two y/o	Control	Control	Feed Day	12:40	12:00 - 12:59	Afternoon	Inactive
2019/08/26	CH2276	Female	Two y/o	Control	Control	Feed Day	12:45	12:00 - 12:59	Afternoon	Inactive
2019/08/26	CH2277	Female	Two y/o	Control	Control	Feed Day	12:45	12:00 - 12:59	Afternoon	Inactive
2019/08/26	CH2276	Female	Two y/o	Control	Control	Feed Day	12:50	12:00 - 12:59	Afternoon	Inactive
2019/08/26	CH2277	Female	Two y/o	Control	Control	Feed Day	12:50	12:00 - 12:59	Afternoon	Inactive
2019/08/26	CH2276	Female	Two y/o	Control	Control	Feed Day	12:55	12:00 - 12:59	Afternoon	Inactive
2019/08/26	CH2277	Female	Two y/o	Control	Control	Feed Day	12:55	12:00 - 12:59	Afternoon	Inactive
2019/08/26	CH2276	Female	Two y/o	Control	Control	Feed Day	13:00	13:00 - 13:59	Afternoon	Inactive
2019/08/26	CH2277	Female	Two y/o	Control	Control	Feed Day	13:00	13:00 - 13:59	Afternoon	Inactive
2019/08/26	CH2276	Female	Two y/o	Control	Control	Feed Day	13:05	13:00 - 13:59	Afternoon	Inactive
2019/08/26	CH2277	Female	Two y/o	Control	Control	Feed Day	13:05	13:00 - 13:59	Afternoon	Inactive
2019/08/26	CH2276	Female	Two y/o	Control	Control	Feed Day	13:10	13:00 - 13:59	Afternoon	Inactive
2019/08/26	CH2277	Female	Two y/o	Control	Control	Feed Day	13:10	13:00 - 13:59	Afternoon	Inactive
2019/08/26	CH2276	Female	Two y/o	Control	Control	Feed Day	13:15	13:00 - 13:59	Afternoon	Inactive
2019/08/26	CH2277	Female	Two y/o	Control	Control	Feed Day	13:15	13:00 - 13:59	Afternoon	Inactive
2019/08/26	CH2276	Female	Two y/o	Control	Control	Feed Day	13:20	13:00 - 13:59	Afternoon	Inactive
2019/08/26	CH2277	Female	Two y/o	Control	Control	Feed Day	13:20	13:00 - 13:59	Afternoon	Inactive
2019/08/26	CH2276	Female	Two y/o	Control	Control	Feed Day	13:25	13:00 - 13:59	Afternoon	Inactive
2019/08/26	CH2277	Female	Two y/o	Control	Control	Feed Day	13:25	13:00 - 13:59	Afternoon	Inactive
2019/08/26	CH2276	Female	Two y/o	Control	Control	Feed Day	13:30	13:00 - 13:59	Afternoon	Inactive
2019/08/26	CH2277	Female	Two y/o	Control	Control	Feed Day	13:30	13:00 - 13:59	Afternoon	Inactive
2019/08/27	CH2276	Female	Two y/o	Control	Control	Feed Day	07:35	07:00 - 07:59	Morning	Interspecific behaviour
2019/08/27	CH2277	Female	Two y/o	Control	Control	Feed Day	07:35	07:00 - 07:59	Morning	Appetitive behaviour
2019/08/27	CH2276	Female	Two y/o	Control	Control	Feed Day	07:40	07:00 - 07:59	Morning	Inactive
2019/08/27	CH2277	Female	Two y/o	Control	Control	Feed Day	07:40	07:00 - 07:59	Morning	Interspecific behaviour
2019/08/27	CH2276	Female	Two y/o	Control	Control	Feed Day	07:45	07:00 - 07:59	Morning	Inactive
2019/08/27	CH2277	Female	Two y/o	Control	Control	Feed Day	07:45	07:00 - 07:59	Morning	Interspecific behaviour
2019/08/27	CH2276	Female	Two y/o	Control	Control	Feed Day	07:50	07:00 - 07:59	Morning	Inactive
2019/08/27	CH2277	Female	Two y/o	Control	Control	Feed Day	07:50	07:00 - 07:59	Morning	Interspecific behaviour
2019/08/27	CH2276	Female	Two y/o	Control	Control	Feed Day	07:55	07:00 - 07:59	Morning	Inactive
2019/08/27	CH2277	Female	Two y/o	Control	Control	Feed Day	07:55	07:00 - 07:59	Morning	Appetitive behaviour
2019/08/27	CH2276	Female	Two y/o	Control	Control	Feed Day	08:00	08:00 - 08:59	Late Morning	Attention
2019/08/27	CH2277	Female	Two y/o	Control	Control	Feed Day	08:00	08:00 - 08:59	Late Morning	Locomotion
2019/08/27	CH2276	Female	Two y/o	Control	Control	Feed Day	08:05	08:00 - 08:59	Late Morning	Locomotion

2019/08/27	CH2277	Female	Two y/o	Control	Control	Feed Day	08:05	08:00 - 08:59	Late Morning	Attention
2019/08/27	CH2276	Female	Two y/o	Control	Control	Feed Day	08:10	08:00 - 08:59	Late Morning	Agnostic behaviour
2019/08/27	CH2277	Female	Two y/o	Control	Control	Feed Day	08:10	08:00 - 08:59	Late Morning	Inactive
2019/08/27	CH2276	Female	Two y/o	Control	Control	Feed Day	08:15	08:00 - 08:59	Late Morning	Agnostic behaviour
2019/08/27	CH2277	Female	Two y/o	Control	Control	Feed Day	08:15	08:00 - 08:59	Late Morning	Inactive
2019/08/27	CH2276	Female	Two y/o	Control	Control	Feed Day	08:20	08:00 - 08:59	Late Morning	Inactive
2019/08/27	CH2277	Female	Two y/o	Control	Control	Feed Day	08:20	08:00 - 08:59	Late Morning	Auto-grooming
2019/08/27	CH2276	Female	Two y/o	Control	Control	Feed Day	08:25	08:00 - 08:59	Late Morning	Appetitive behaviour
2019/08/27	CH2277	Female	Two y/o	Control	Control	Feed Day	08:25	08:00 - 08:59	Late Morning	Appetitive behaviour
2019/08/27	CH2271	Male	Two y/o	Control	Control	Feed Day	08:30	08:00 - 08:59	Late Morning	Locomotion
2019/08/27	CH2276	Female	Two y/o	Control	Control	Feed Day	08:30	08:00 - 08:59	Late Morning	Appetitive behaviour
2019/08/27	CH2277	Female	Two y/o	Control	Control	Feed Day	08:30	08:00 - 08:59	Late Morning	Appetitive behaviour
2019/08/27	CH2271	Male	Two y/o	Control	Control	Feed Day	08:35	08:00 - 08:59	Late Morning	Inactive
2019/08/27	CH2271	Male	Two y/o	Control	Control	Feed Day	08:40	08:00 - 08:59	Late Morning	Inactive
2019/08/27	CH2271	Male	Two y/o	Control	Control	Feed Day	08:45	08:00 - 08:59	Late Morning	Inactive
2019/08/27	CH2271	Male	Two y/o	Control	Control	Feed Day	08:50	08:00 - 08:59	Late Morning	Appetitive behaviour
2019/08/27	CH2271	Male	Two y/o	Control	Control	Feed Day	08:55	08:00 - 08:59	Late Morning	Interspecific behaviour
2019/08/27	CH2271	Male	Two y/o	Control	Control	Feed Day	09:00	09:00 - 09:59	Late Morning	Inactive
2019/08/27	CH2271	Male	Two y/o	Control	Control	Feed Day	09:05	09:00 - 09:59	Late Morning	Locomotion
2019/08/27	CH2271	Male	Two y/o	Control	Control	Feed Day	09:10	09:00 - 09:59	Late Morning	Locomotion
2019/08/27	CH2271	Male	Two y/o	Control	Control	Feed Day	09:15	09:00 - 09:59	Late Morning	Locomotion
2019/08/27	CH2271	Male	Two y/o	Control	Control	Feed Day	09:20	09:00 - 09:59	Late Morning	Interspecific behaviour
2019/08/27	CH2271	Male	Two y/o	Control	Control	Feed Day	09:25	09:00 - 09:59	Late Morning	Locomotion
2019/08/28	CH2271	Male	Two y/o	Control	Control	Feed Day	07:40	07:00 - 07:59	Late Morning	Locomotion
2019/08/28	CH2271	Male	Two y/o	Control	Control	Feed Day	07:45	07:00 - 07:59	Late Morning	Inactive
2019/08/28	CH2271	Male	Two y/o	Control	Control	Feed Day	07:50	07:00 - 07:59	Late Morning	Inactive
2019/08/28	CH2271	Male	Two y/o	Control	Control	Feed Day	07:55	07:00 - 07:59	Late Morning	Inactive
2019/08/28	CH2271	Male	Two y/o	Control	Control	Feed Day	08:00	08:00 - 08:59	Late Morning	Locomotion
2019/08/28	CH2271	Male	Two y/o	Control	Control	Feed Day	08:05	08:00 - 08:59	Late Morning	Locomotion
2019/08/28	CH2271	Male	Two y/o	Control	Control	Feed Day	08:10	08:00 - 08:59	Late Morning	Locomotion
2019/08/28	CH2271	Male	Two y/o	Control	Control	Feed Day	08:15	08:00 - 08:59	Late Morning	Locomotion
2019/08/28	CH2271	Male	Two y/o	Control	Control	Feed Day	08:20	08:00 - 08:59	Late Morning	Appetitive behaviour
2019/08/28	CH2271	Male	Two y/o	Control	Control	Feed Day	08:25	08:00 - 08:59	Late Morning	Appetitive behaviour
2019/08/28	CH2271	Male	Two y/o	Control	Control	Feed Day	08:30	08:00 - 08:59	Late Morning	Appetitive behaviour
2019/08/28	CH2271	Male	Two y/o	Control	Control	Feed Day	08:35	08:00 - 08:59	Late Morning	Appetitive behaviour
2019/08/28	CH2276	Female	Two y/o	Control	Control	Feed Day	08:35	08:00 - 08:59	Late Morning	Inactive
2019/08/28	CH2277	Female	Two y/o	Control	Control	Feed Day	08:35	08:00 - 08:59	Late Morning	Locomotion

2019/08/28	CH2276	Female	Two y/o	Control	Control	Feed Day	08:40	08:00 - 08:59	Late Morning	Scent-marking
2019/08/28	CH2277	Female	Two y/o	Control	Control	Feed Day	08:40	08:00 - 08:59	Late Morning	Appetitive behaviour
2019/08/28	CH2276	Female	Two y/o	Control	Control	Feed Day	08:45	08:00 - 08:59	Late Morning	Appetitive behaviour
2019/08/28	CH2277	Female	Two y/o	Control	Control	Feed Day	08:45	08:00 - 08:59	Late Morning	Attention
2019/08/28	CH2276	Female	Two y/o	Control	Control	Feed Day	08:50	08:00 - 08:59	Late Morning	Locomotion
2019/08/28	CH2277	Female	Two y/o	Control	Control	Feed Day	08:50	08:00 - 08:59	Late Morning	Attention
2019/08/28	CH2276	Female	Two y/o	Control	Control	Feed Day	08:55	08:00 - 08:59	Late Morning	Locomotion
2019/08/28	CH2277	Female	Two y/o	Control	Control	Feed Day	08:55	08:00 - 08:59	Late Morning	Appetitive behaviour
2019/08/28	CH2276	Female	Two y/o	Control	Control	Feed Day	09:00	09:00 - 09:59	Late Morning	Scent-marking
2019/08/28	CH2277	Female	Two y/o	Control	Control	Feed Day	09:00	09:00 - 09:59	Late Morning	Appetitive behaviour
2019/08/28	CH2276	Female	Two y/o	Control	Control	Feed Day	09:05	09:00 - 09:59	Late Morning	Appetitive behaviour
2019/08/28	CH2277	Female	Two y/o	Control	Control	Feed Day	09:05	09:00 - 09:59	Late Morning	Appetitive behaviour
2019/08/28	CH2276	Female	Two y/o	Control	Control	Feed Day	09:10	09:00 - 09:59	Late Morning	Interspecific behaviour
2019/08/28	CH2277	Female	Two y/o	Control	Control	Feed Day	09:10	09:00 - 09:59	Late Morning	Interspecific behaviour
2019/08/28	CH2276	Female	Two y/o	Control	Control	Feed Day	09:15	09:00 - 09:59	Late Morning	Appetitive behaviour
2019/08/28	CH2277	Female	Two y/o	Control	Control	Feed Day	09:15	09:00 - 09:59	Late Morning	Appetitive behaviour
2019/08/28	CH2276	Female	Two y/o	Control	Control	Feed Day	09:20	09:00 - 09:59	Late Morning	Locomotion
2019/08/28	CH2277	Female	Two y/o	Control	Control	Feed Day	09:20	09:00 - 09:59	Late Morning	Appetitive behaviour
2019/08/28	CH2276	Female	Two y/o	Control	Control	Feed Day	09:25	09:00 - 09:59	Late Morning	Appetitive behaviour
2019/08/28	CH2277	Female	Two y/o	Control	Control	Feed Day	09:25	09:00 - 09:59	Late Morning	Appetitive behaviour
2019/08/28	CH2276	Female	Two y/o	Control	Control	Feed Day	09:30	09:00 - 09:59	Late Morning	Inactive
2019/08/28	CH2277	Female	Two y/o	Control	Control	Feed Day	09:30	09:00 - 09:59	Late Morning	Inactive
2019/08/29	CH2276	Female	Two y/o	Control	Control	Feed Day	07:50	07:00 - 07:59	Morning	Inactive
2019/08/29	CH2277	Female	Two y/o	Control	Control	Feed Day	07:50	07:00 - 07:59	Morning	Locomotion
2019/08/29	CH2276	Female	Two y/o	Control	Control	Feed Day	07:55	07:00 - 07:59	Morning	Inactive
2019/08/29	CH2277	Female	Two y/o	Control	Control	Feed Day	07:55	07:00 - 07:59	Morning	Interspecific behaviour
2019/08/29	CH2276	Female	Two y/o	Control	Control	Feed Day	08:00	08:00 - 08:59	Late Morning	Interspecific behaviour
2019/08/29	CH2277	Female	Two y/o	Control	Control	Feed Day	08:00	08:00 - 08:59	Late Morning	Locomotion
2019/08/29	CH2276	Female	Two y/o	Control	Control	Feed Day	08:05	08:00 - 08:59	Late Morning	Inactive
2019/08/29	CH2277	Female	Two y/o	Control	Control	Feed Day	08:05	08:00 - 08:59	Late Morning	Locomotion
2019/08/29	CH2276	Female	Two y/o	Control	Control	Feed Day	08:10	08:00 - 08:59	Late Morning	Inactive
2019/08/29	CH2277	Female	Two y/o	Control	Control	Feed Day	08:10	08:00 - 08:59	Late Morning	Appetitive behaviour
2019/08/29	CH2276	Female	Two y/o	Control	Control	Feed Day	08:15	08:00 - 08:59	Late Morning	Inactive
2019/08/29	CH2277	Female	Two y/o	Control	Control	Feed Day	08:15	08:00 - 08:59	Late Morning	Appetitive behaviour
2019/08/29	CH2276	Female	Two y/o	Control	Control	Feed Day	08:20	08:00 - 08:59	Late Morning	Locomotion
2019/08/29	CH2277	Female	Two y/o	Control	Control	Feed Day	08:20	08:00 - 08:59	Late Morning	Appetitive behaviour
2019/08/29	CH2276	Female	Two y/o	Control	Control	Feed Day	08:25	08:00 - 08:59	Late Morning	Locomotion

2019/08/29	CH2277	Female	Two y/o	Control	Control	Feed Day	08:25	08:00 - 08:59	Late Morning	Appetitive behaviour
2019/08/29	CH2276	Female	Two y/o	Control	Control	Feed Day	08:30	08:00 - 08:59	Late Morning	Interspecific behaviour
2019/08/29	CH2277	Female	Two y/o	Control	Control	Feed Day	08:30	08:00 - 08:59	Late Morning	Inactive
2019/08/29	CH2276	Female	Two y/o	Control	Control	Feed Day	08:35	08:00 - 08:59	Late Morning	Interspecific behaviour
2019/08/29	CH2277	Female	Two y/o	Control	Control	Feed Day	08:35	08:00 - 08:59	Late Morning	Appetitive behaviour
2019/08/29	CH2276	Female	Two y/o	Control	Control	Feed Day	08:40	08:00 - 08:59	Late Morning	Interspecific behaviour
2019/08/29	CH2277	Female	Two y/o	Control	Control	Feed Day	08:40	08:00 - 08:59	Late Morning	Appetitive behaviour
2019/08/29	CH2271	Male	Two y/o	Control	Control	Feed Day	08:45	08:00 - 08:59	Late Morning	Locomotion
2019/08/29	CH2276	Female	Two y/o	Control	Control	Feed Day	08:45	08:00 - 08:59	Late Morning	Inactive
2019/08/29	CH2277	Female	Two y/o	Control	Control	Feed Day	08:45	08:00 - 08:59	Late Morning	Interspecific behaviour
2019/08/29	CH2271	Male	Two y/o	Control	Control	Feed Day	08:50	08:00 - 08:59	Late Morning	Locomotion
2019/08/29	CH2271	Male	Two y/o	Control	Control	Feed Day	08:55	08:00 - 08:59	Late Morning	Affiliative behaviour
2019/08/29	CH2271	Male	Two y/o	Control	Control	Feed Day	09:00	09:00 - 09:59	Late Morning	Locomotion
2019/08/29	CH2271	Male	Two y/o	Control	Control	Feed Day	09:05	09:00 - 09:59	Late Morning	Locomotion
2019/08/29	CH2271	Male	Two y/o	Control	Control	Feed Day	09:10	09:00 - 09:59	Late Morning	Inactive
2019/08/29	CH2271	Male	Two y/o	Control	Control	Feed Day	09:15	09:00 - 09:59	Late Morning	Inactive
2019/08/29	CH2271	Male	Two y/o	Control	Control	Feed Day	09:20	09:00 - 09:59	Late Morning	Inactive
2019/08/29	CH2271	Male	Two y/o	Control	Control	Feed Day	09:25	09:00 - 09:59	Late Morning	Inactive
2019/08/29	CH2271	Male	Two y/o	Control	Control	Feed Day	09:30	09:00 - 09:59	Late Morning	Inactive
2019/08/29	CH2271	Male	Two y/o	Control	Control	Feed Day	09:35	09:00 - 09:59	Late Morning	Inactive
2019/08/29	CH2271	Male	Two y/o	Control	Control	Feed Day	09:40	09:00 - 09:59	Late Morning	Inactive
2019/08/30	CH2271	Male	Two y/o	Control	Control	Feed Day	07:55	07:00 - 07:59	Morning	Attention
2019/08/30	CH2271	Male	Two y/o	Control	Control	Feed Day	08:00	08:00 - 08:59	Late Morning	Auto-grooming
2019/08/30	CH2271	Male	Two y/o	Control	Control	Feed Day	08:05	08:00 - 08:59	Late Morning	Locomotion
2019/08/30	CH2271	Male	Two y/o	Control	Control	Feed Day	08:10	08:00 - 08:59	Late Morning	Attention
2019/08/30	CH2271	Male	Two y/o	Control	Control	Feed Day	08:15	08:00 - 08:59	Late Morning	Locomotion
2019/08/30	CH2271	Male	Two y/o	Control	Control	Feed Day	08:20	08:00 - 08:59	Late Morning	Locomotion
2019/08/30	CH2271	Male	Two y/o	Control	Control	Feed Day	08:25	08:00 - 08:59	Late Morning	Locomotion
2019/08/30	CH2271	Male	Two y/o	Control	Control	Feed Day	08:30	08:00 - 08:59	Late Morning	Appetitive behaviour
2019/08/30	CH2271	Male	Two y/o	Control	Control	Feed Day	08:35	08:00 - 08:59	Late Morning	Attention
2019/08/30	CH2271	Male	Two y/o	Control	Control	Feed Day	08:40	08:00 - 08:59	Late Morning	Appetitive behaviour
2019/08/30	CH2271	Male	Two y/o	Control	Control	Feed Day	08:45	08:00 - 08:59	Late Morning	Maintenance
2019/08/30	CH2276	Female	Two y/o	Control	Control	Feed Day	08:50	08:00 - 08:59	Late Morning	Inactive
2019/08/30	CH2277	Female	Two y/o	Control	Control	Feed Day	08:50	08:00 - 08:59	Late Morning	Locomotion
2019/08/30	CH2271	Male	Two y/o	Control	Control	Feed Day	08:50	08:00 - 08:59	Late Morning	Appetitive behaviour
2019/08/30	CH2276	Female	Two y/o	Control	Control	Feed Day	08:55	08:00 - 08:59	Late Morning	Inactive
2019/08/30	CH2277	Female	Two y/o	Control	Control	Feed Day	08:55	08:00 - 08:59	Late Morning	Inactive

2019/08/30	CH2276	Female	Two y/o	Control	Control	Feed Day	09:00	09:00 - 09:59	Late Morning	Inactive
2019/08/30	CH2277	Female	Two y/o	Control	Control	Feed Day	09:00	09:00 - 09:59	Late Morning	Inactive
2019/08/30	CH2276	Female	Two y/o	Control	Control	Feed Day	09:05	09:00 - 09:59	Late Morning	Inactive
2019/08/30	CH2277	Female	Two y/o	Control	Control	Feed Day	09:05	09:00 - 09:59	Late Morning	Inactive
2019/08/30	CH2276	Female	Two y/o	Control	Control	Feed Day	09:10	09:00 - 09:59	Late Morning	Inactive
2019/08/30	CH2277	Female	Two y/o	Control	Control	Feed Day	09:10	09:00 - 09:59	Late Morning	Inactive
2019/08/30	CH2276	Female	Two y/o	Control	Control	Feed Day	09:15	09:00 - 09:59	Late Morning	Inactive
2019/08/30	CH2277	Female	Two y/o	Control	Control	Feed Day	09:15	09:00 - 09:59	Late Morning	Inactive
2019/08/30	CH2276	Female	Two y/o	Control	Control	Feed Day	09:20	09:00 - 09:59	Late Morning	Inactive
2019/08/30	CH2277	Female	Two y/o	Control	Control	Feed Day	09:20	09:00 - 09:59	Late Morning	Inactive
2019/08/30	CH2276	Female	Two y/o	Control	Control	Feed Day	09:25	09:00 - 09:59	Late Morning	Inactive
2019/08/30	CH2277	Female	Two y/o	Control	Control	Feed Day	09:25	09:00 - 09:59	Late Morning	Inactive
2019/08/30	CH2276	Female	Two y/o	Control	Control	Feed Day	09:30	09:00 - 09:59	Late Morning	Inactive
2019/08/30	CH2277	Female	Two y/o	Control	Control	Feed Day	09:30	09:00 - 09:59	Late Morning	Inactive
2019/08/30	CH2276	Female	Two y/o	Control	Control	Feed Day	09:35	09:00 - 09:59	Late Morning	Inactive
2019/08/30	CH2277	Female	Two y/o	Control	Control	Feed Day	09:35	09:00 - 09:59	Late Morning	Locomotion
2019/08/30	CH2276	Female	Two y/o	Control	Control	Feed Day	09:40	09:00 - 09:59	Late Morning	Attention
2019/08/30	CH2277	Female	Two y/o	Control	Control	Feed Day	09:40	09:00 - 09:59	Late Morning	Appetitive behaviour
2019/08/30	CH2276	Female	Two y/o	Control	Control	Feed Day	09:45	09:00 - 09:59	Late Morning	Inactive
2019/08/30	CH2277	Female	Two y/o	Control	Control	Feed Day	09:45	09:00 - 09:59	Late Morning	Interspecific behaviour
2019/08/31	CH2276	Female	Two y/o	Control	Control	Feed Day	13:40	13:00 - 13:59	Afternoon	Inactive
2019/08/31	CH2277	Female	Two y/o	Control	Control	Feed Day	13:40	13:00 - 13:59	Afternoon	Inactive
2019/08/31	CH2276	Female	Two y/o	Control	Control	Feed Day	13:45	13:00 - 13:59	Afternoon	Inactive
2019/08/31	CH2277	Female	Two y/o	Control	Control	Feed Day	13:45	13:00 - 13:59	Afternoon	Inactive
2019/08/31	CH2276	Female	Two y/o	Control	Control	Feed Day	13:50	13:00 - 13:59	Afternoon	Inactive
2019/08/31	CH2277	Female	Two y/o	Control	Control	Feed Day	13:50	13:00 - 13:59	Afternoon	Inactive
2019/08/31	CH2276	Female	Two y/o	Control	Control	Feed Day	13:55	13:00 - 13:59	Afternoon	Inactive
2019/08/31	CH2277	Female	Two y/o	Control	Control	Feed Day	13:55	13:00 - 13:59	Afternoon	Inactive
2019/08/31	CH2276	Female	Two y/o	Control	Control	Feed Day	14:00	14:00 - 14:59	Afternoon	Inactive
2019/08/31	CH2277	Female	Two y/o	Control	Control	Feed Day	14:00	14:00 - 14:59	Afternoon	Inactive
2019/08/31	CH2276	Female	Two y/o	Control	Control	Feed Day	14:05	14:00 - 14:59	Afternoon	Inactive
2019/08/31	CH2277	Female	Two y/o	Control	Control	Feed Day	14:05	14:00 - 14:59	Afternoon	Inactive
2019/08/31	CH2276	Female	Two y/o	Control	Control	Feed Day	14:10	14:00 - 14:59	Afternoon	Inactive
2019/08/31	CH2277	Female	Two y/o	Control	Control	Feed Day	14:10	14:00 - 14:59	Afternoon	Inactive
2019/08/31	CH2276	Female	Two y/o	Control	Control	Feed Day	14:15	14:00 - 14:59	Afternoon	Inactive
2019/08/31	CH2277	Female	Two y/o	Control	Control	Feed Day	14:15	14:00 - 14:59	Afternoon	Interspecific behaviour
2019/08/31	CH2276	Female	Two y/o	Control	Control	Feed Day	14:20	14:00 - 14:59	Afternoon	Locomotion

2019/08/31	CH2277	Female	Two y/o	Control	Control	Feed Day	14:20	14:00 - 14:59	Afternoon	Interspecific behaviour
2019/08/31	CH2276	Female	Two y/o	Control	Control	Feed Day	14:25	14:00 - 14:59	Afternoon	Inactive
2019/08/31	CH2277	Female	Two y/o	Control	Control	Feed Day	14:25	14:00 - 14:59	Afternoon	Interspecific behaviour
2019/08/31	CH2276	Female	Two y/o	Control	Control	Feed Day	14:30	14:00 - 14:59	Afternoon	Inactive
2019/08/31	CH2277	Female	Two y/o	Control	Control	Feed Day	14:30	14:00 - 14:59	Afternoon	Inactive
2019/08/31	CH2271	Male	Two y/o	Control	Control	Feed Day	14:35	14:00 - 14:59	Afternoon	Locomotion
2019/08/31	CH2276	Female	Two y/o	Control	Control	Feed Day	14:35	14:00 - 14:59	Afternoon	Inactive
2019/08/31	CH2277	Female	Two y/o	Control	Control	Feed Day	14:35	14:00 - 14:59	Afternoon	Inactive
2019/08/31	CH2271	Male	Two y/o	Control	Control	Feed Day	14:40	14:00 - 14:59	Afternoon	Attention
2019/08/31	CH2271	Male	Two y/o	Control	Control	Feed Day	14:45	14:00 - 14:59	Afternoon	Stereotypical
2019/08/31	CH2271	Male	Two y/o	Control	Control	Feed Day	14:50	14:00 - 14:59	Afternoon	Locomotion
2019/08/31	CH2271	Male	Two y/o	Control	Control	Feed Day	14:55	14:00 - 14:59	Afternoon	Inactive
2019/08/31	CH2271	Male	Two y/o	Control	Control	Feed Day	15:00	15:00 - 15:59	Afternoon	Inactive
2019/08/31	CH2271	Male	Two y/o	Control	Control	Feed Day	15:05	15:00 - 15:59	Afternoon	Inactive
2019/08/31	CH2271	Male	Two y/o	Control	Control	Feed Day	15:10	15:00 - 15:59	Afternoon	Inactive
2019/08/31	CH2271	Male	Two y/o	Control	Control	Feed Day	15:15	15:00 - 15:59	Afternoon	Inactive
2019/08/31	CH2271	Male	Two y/o	Control	Control	Feed Day	15:20	15:00 - 15:59	Afternoon	Inactive
2019/08/31	CH2271	Male	Two y/o	Control	Control	Feed Day	15:25	15:00 - 15:59	Afternoon	Inactive
2019/08/31	CH2271	Male	Two y/o	Control	Control	Feed Day	15:30	15:00 - 15:59	Afternoon	Inactive
2019/09/03	CH2271	Male	Two y/o	Control	Control	Feed Day	11:05	11:00 - 11:59	Late Morning	Inactive
2019/09/03	CH2271	Male	Two y/o	Control	Control	Feed Day	11:10	11:00 - 11:59	Late Morning	Inactive
2019/09/03	CH2271	Male	Two y/o	Control	Control	Feed Day	11:15	11:00 - 11:59	Late Morning	Inactive
2019/09/03	CH2271	Male	Two y/o	Control	Control	Feed Day	11:20	11:00 - 11:59	Late Morning	Inactive
2019/09/03	CH2271	Male	Two y/o	Control	Control	Feed Day	11:25	11:00 - 11:59	Late Morning	Locomotion
2019/09/03	CH2271	Male	Two y/o	Control	Control	Feed Day	11:30	11:00 - 11:59	Late Morning	Locomotion
2019/09/03	CH2271	Male	Two y/o	Control	Control	Feed Day	11:35	11:00 - 11:59	Late Morning	Inactive
2019/09/03	CH2271	Male	Two y/o	Control	Control	Feed Day	11:40	11:00 - 11:59	Late Morning	Inactive
2019/09/03	CH2271	Male	Two y/o	Control	Control	Feed Day	11:45	11:00 - 11:59	Late Morning	Inactive
2019/09/03	CH2271	Male	Two y/o	Control	Control	Feed Day	11:50	11:00 - 11:59	Late Morning	Inactive
2019/09/03	CH2271	Male	Two y/o	Control	Control	Feed Day	11:55	11:00 - 11:59	Late Morning	Inactive
2019/09/03	CH2271	Male	Two y/o	Control	Control	Feed Day	12:00	12:00 - 12:59	Afternoon	Inactive
2019/09/03	CH2276	Female	Two y/o	Control	Control	Feed Day	12:00	12:00 - 12:59	Afternoon	Locomotion
2019/09/03	CH2277	Female	Two y/o	Control	Control	Feed Day	12:00	12:00 - 12:59	Afternoon	Inactive
2019/09/03	CH2276	Female	Two y/o	Control	Control	Feed Day	12:05	12:00 - 12:59	Afternoon	Inactive
2019/09/03	CH2277	Female	Two y/o	Control	Control	Feed Day	12:05	12:00 - 12:59	Afternoon	Inactive
2019/09/03	CH2276	Female	Two y/o	Control	Control	Feed Day	12:10	12:00 - 12:59	Afternoon	Inactive
2019/09/03	CH2277	Female	Two y/o	Control	Control	Feed Day	12:10	12:00 - 12:59	Afternoon	Inactive

2019/09/03	CH2276	Female	Two y/o	Control	Control	Feed Day	12:15	12:00 - 12:59	Afternoon	Inactive
2019/09/03	CH2277	Female	Two y/o	Control	Control	Feed Day	12:15	12:00 - 12:59	Afternoon	Inactive
2019/09/03	CH2276	Female	Two y/o	Control	Control	Feed Day	12:20	12:00 - 12:59	Afternoon	Inactive
2019/09/03	CH2277	Female	Two y/o	Control	Control	Feed Day	12:20	12:00 - 12:59	Afternoon	Inactive
2019/09/03	CH2276	Female	Two y/o	Control	Control	Feed Day	12:25	12:00 - 12:59	Afternoon	Inactive
2019/09/03	CH2277	Female	Two y/o	Control	Control	Feed Day	12:25	12:00 - 12:59	Afternoon	Inactive
2019/09/03	CH2276	Female	Two y/o	Control	Control	Feed Day	12:30	12:00 - 12:59	Afternoon	Inactive
2019/09/03	CH2277	Female	Two y/o	Control	Control	Feed Day	12:30	12:00 - 12:59	Afternoon	Inactive
2019/09/03	CH2276	Female	Two y/o	Control	Control	Feed Day	12:35	12:00 - 12:59	Afternoon	Inactive
2019/09/03	CH2277	Female	Two y/o	Control	Control	Feed Day	12:35	12:00 - 12:59	Afternoon	Inactive
2019/09/03	CH2276	Female	Two y/o	Control	Control	Feed Day	12:40	12:00 - 12:59	Afternoon	Inactive
2019/09/03	CH2277	Female	Two y/o	Control	Control	Feed Day	12:40	12:00 - 12:59	Afternoon	Inactive
2019/09/03	CH2276	Female	Two y/o	Control	Control	Feed Day	12:45	12:00 - 12:59	Afternoon	Inactive
2019/09/03	CH2277	Female	Two y/o	Control	Control	Feed Day	12:45	12:00 - 12:59	Afternoon	Inactive
2019/09/03	CH2276	Female	Two y/o	Control	Control	Feed Day	12:50	12:00 - 12:59	Afternoon	Inactive
2019/09/03	CH2277	Female	Two y/o	Control	Control	Feed Day	12:50	12:00 - 12:59	Afternoon	Inactive
2019/09/03	CH2276	Female	Two y/o	Control	Control	Feed Day	12:55	12:00 - 12:59	Afternoon	Inactive
2019/09/03	CH2277	Female	Two y/o	Control	Control	Feed Day	12:55	12:00 - 12:59	Afternoon	Inactive
2019/09/04	CH2276	Female	Two y/o	Control	Control	Feed Day	11:15	11:00 - 11:59	Late Morning	Inactive
2019/09/04	CH2277	Female	Two y/o	Control	Control	Feed Day	11:15	11:00 - 11:59	Late Morning	Inactive
2019/09/04	CH2276	Female	Two y/o	Control	Control	Feed Day	11:20	11:00 - 11:59	Late Morning	Inactive
2019/09/04	CH2277	Female	Two y/o	Control	Control	Feed Day	11:20	11:00 - 11:59	Late Morning	Inactive
2019/09/04	CH2276	Female	Two y/o	Control	Control	Feed Day	11:25	11:00 - 11:59	Late Morning	Inactive
2019/09/04	CH2277	Female	Two y/o	Control	Control	Feed Day	11:25	11:00 - 11:59	Late Morning	Inactive
2019/09/04	CH2276	Female	Two y/o	Control	Control	Feed Day	11:30	11:00 - 11:59	Late Morning	Inactive
2019/09/04	CH2277	Female	Two y/o	Control	Control	Feed Day	11:30	11:00 - 11:59	Late Morning	Inactive
2019/09/04	CH2276	Female	Two y/o	Control	Control	Feed Day	11:35	11:00 - 11:59	Late Morning	Inactive
2019/09/04	CH2277	Female	Two y/o	Control	Control	Feed Day	11:35	11:00 - 11:59	Late Morning	Inactive
2019/09/04	CH2276	Female	Two y/o	Control	Control	Feed Day	11:40	11:00 - 11:59	Late Morning	Out of sight
2019/09/04	CH2277	Female	Two y/o	Control	Control	Feed Day	11:40	11:00 - 11:59	Late Morning	Inactive
2019/09/04	CH2276	Female	Two y/o	Control	Control	Feed Day	11:45	11:00 - 11:59	Late Morning	Out of sight
2019/09/04	CH2277	Female	Two y/o	Control	Control	Feed Day	11:45	11:00 - 11:59	Late Morning	Inactive
2019/09/04	CH2276	Female	Two y/o	Control	Control	Feed Day	11:50	11:00 - 11:59	Late Morning	Out of sight
2019/09/04	CH2277	Female	Two y/o	Control	Control	Feed Day	11:50	11:00 - 11:59	Late Morning	Inactive
2019/09/04	CH2276	Female	Two y/o	Control	Control	Feed Day	11:55	11:00 - 11:59	Late Morning	Out of sight
2019/09/04	CH2277	Female	Two y/o	Control	Control	Feed Day	11:55	11:00 - 11:59	Late Morning	Inactive
2019/09/04	CH2276	Female	Two y/o	Control	Control	Feed Day	12:00	12:00 - 12:59	Afternoon	Out of sight

2019/09/04	CH2277	Female	Two y/o	Control	Control	Feed Day	12:00	12:00 - 12:59	Afternoon	Inactive
2019/09/04	CH2271	Male	Two y/o	Control	Control	Feed Day	12:05	12:00 - 12:59	Afternoon	Inactive
2019/09/04	CH2276	Female	Two y/o	Control	Control	Feed Day	12:05	12:00 - 12:59	Afternoon	Out of sight
2019/09/04	CH2277	Female	Two y/o	Control	Control	Feed Day	12:05	12:00 - 12:59	Afternoon	Inactive
2019/09/04	CH2271	Male	Two y/o	Control	Control	Feed Day	12:10	12:00 - 12:59	Afternoon	Auto-grooming
2019/09/04	CH2276	Female	Two y/o	Control	Control	Feed Day	12:10	12:00 - 12:59	Afternoon	Out of sight
2019/09/04	CH2277	Female	Two y/o	Control	Control	Feed Day	12:10	12:00 - 12:59	Afternoon	Inactive
2019/09/04	CH2271	Male	Two y/o	Control	Control	Feed Day	12:15	12:00 - 12:59	Afternoon	Inactive
2019/09/04	CH2271	Male	Two y/o	Control	Control	Feed Day	12:20	12:00 - 12:59	Afternoon	Scent-marking
2019/09/04	CH2271	Male	Two y/o	Control	Control	Feed Day	12:25	12:00 - 12:59	Afternoon	Inactive
2019/09/04	CH2271	Male	Two y/o	Control	Control	Feed Day	12:30	12:00 - 12:59	Afternoon	Inactive
2019/09/04	CH2271	Male	Two y/o	Control	Control	Feed Day	12:35	12:00 - 12:59	Afternoon	Inactive
2019/09/04	CH2271	Male	Two y/o	Control	Control	Feed Day	12:40	12:00 - 12:59	Afternoon	Inactive
2019/09/04	CH2271	Male	Two y/o	Control	Control	Feed Day	12:45	12:00 - 12:59	Afternoon	Inactive
2019/09/04	CH2271	Male	Two y/o	Control	Control	Feed Day	12:50	12:00 - 12:59	Afternoon	Inactive
2019/09/04	CH2271	Male	Two y/o	Control	Control	Feed Day	12:55	12:00 - 12:59	Afternoon	Inactive
2019/09/04	CH2271	Male	Two y/o	Control	Control	Feed Day	13:00	13:00 - 13:59	Afternoon	Inactive
2019/09/05	CH2271	Male	Two y/o	Control	Control	Feed Day	11:25	11:00 - 11:59	Late Morning	Locomotion
2019/09/05	CH2271	Male	Two y/o	Control	Control	Feed Day	11:30	11:00 - 11:59	Late Morning	Scent-marking
2019/09/05	CH2271	Male	Two y/o	Control	Control	Feed Day	11:35	11:00 - 11:59	Late Morning	Scent-marking
2019/09/05	CH2271	Male	Two y/o	Control	Control	Feed Day	11:40	11:00 - 11:59	Late Morning	Attention
2019/09/05	CH2271	Male	Two y/o	Control	Control	Feed Day	11:45	11:00 - 11:59	Late Morning	Locomotion
2019/09/05	CH2271	Male	Two y/o	Control	Control	Feed Day	11:50	11:00 - 11:59	Late Morning	Interspecific behaviour
2019/09/05	CH2271	Male	Two y/o	Control	Control	Feed Day	11:55	11:00 - 11:59	Late Morning	Interspecific behaviour
2019/09/05	CH2271	Male	Two y/o	Control	Control	Feed Day	12:00	12:00 - 12:59	Afternoon	Inactive
2019/09/05	CH2271	Male	Two y/o	Control	Control	Feed Day	12:05	12:00 - 12:59	Afternoon	Interspecific behaviour
2019/09/05	CH2276	Female	Two y/o	Control	Control	Feed Day	12:05	12:00 - 12:59	Afternoon	Inactive
2019/09/05	CH2277	Female	Two y/o	Control	Control	Feed Day	12:05	12:00 - 12:59	Afternoon	Inactive
2019/09/05	CH2271	Male	Two y/o	Control	Control	Feed Day	12:10	12:00 - 12:59	Afternoon	Locomotion
2019/09/05	CH2276	Female	Two y/o	Control	Control	Feed Day	12:10	12:00 - 12:59	Afternoon	Interspecific behaviour
2019/09/05	CH2277	Female	Two y/o	Control	Control	Feed Day	12:10	12:00 - 12:59	Afternoon	Interspecific behaviour
2019/09/05	CH2271	Male	Two y/o	Control	Control	Feed Day	12:15	12:00 - 12:59	Afternoon	Inactive
2019/09/05	CH2276	Female	Two y/o	Control	Control	Feed Day	12:15	12:00 - 12:59	Afternoon	Interspecific behaviour
2019/09/05	CH2277	Female	Two y/o	Control	Control	Feed Day	12:15	12:00 - 12:59	Afternoon	Interspecific behaviour
2019/09/05	CH2271	Male	Two y/o	Control	Control	Feed Day	12:20	12:00 - 12:59	Afternoon	Inactive
2019/09/05	CH2276	Female	Two y/o	Control	Control	Feed Day	12:20	12:00 - 12:59	Afternoon	Inactive
2019/09/05	CH2277	Female	Two y/o	Control	Control	Feed Day	12:20	12:00 - 12:59	Afternoon	Inactive

2019/09/05	CH2276	Female	Two y/o	Control	Control	Feed Day	12:25	12:00 - 12:59	Afternoon	Inactive
2019/09/05	CH2277	Female	Two y/o	Control	Control	Feed Day	12:25	12:00 - 12:59	Afternoon	Inactive
2019/09/05	CH2276	Female	Two y/o	Control	Control	Feed Day	12:30	12:00 - 12:59	Afternoon	Inactive
2019/09/05	CH2277	Female	Two y/o	Control	Control	Feed Day	12:30	12:00 - 12:59	Afternoon	Inactive
2019/09/05	CH2276	Female	Two y/o	Control	Control	Feed Day	12:35	12:00 - 12:59	Afternoon	Inactive
2019/09/05	CH2277	Female	Two y/o	Control	Control	Feed Day	12:35	12:00 - 12:59	Afternoon	Inactive
2019/09/05	CH2276	Female	Two y/o	Control	Control	Feed Day	12:40	12:00 - 12:59	Afternoon	Inactive
2019/09/05	CH2277	Female	Two y/o	Control	Control	Feed Day	12:40	12:00 - 12:59	Afternoon	Inactive
2019/09/05	CH2276	Female	Two y/o	Control	Control	Feed Day	12:45	12:00 - 12:59	Afternoon	Inactive
2019/09/05	CH2277	Female	Two y/o	Control	Control	Feed Day	12:45	12:00 - 12:59	Afternoon	Inactive
2019/09/05	CH2276	Female	Two y/o	Control	Control	Feed Day	12:50	12:00 - 12:59	Afternoon	Inactive
2019/09/05	CH2277	Female	Two y/o	Control	Control	Feed Day	12:50	12:00 - 12:59	Afternoon	Inactive
2019/09/05	CH2276	Female	Two y/o	Control	Control	Feed Day	12:55	12:00 - 12:59	Afternoon	Inactive
2019/09/05	CH2277	Female	Two y/o	Control	Control	Feed Day	12:55	12:00 - 12:59	Afternoon	Inactive
2019/09/05	CH2276	Female	Two y/o	Control	Control	Feed Day	13:00	13:00 - 13:59	Afternoon	Inactive
2019/09/05	CH2277	Female	Two y/o	Control	Control	Feed Day	13:00	13:00 - 13:59	Afternoon	Inactive
2019/09/06	CH2276	Female	Two y/o	Control	Control	Feed Day	07:40	07:00 - 07:59	Morning	Locomotion
2019/09/06	CH2277	Female	Two y/o	Control	Control	Feed Day	07:40	07:00 - 07:59	Morning	Interspecific behaviour
2019/09/06	CH2276	Female	Two y/o	Control	Control	Feed Day	07:45	07:00 - 07:59	Morning	Appetitive behaviour
2019/09/06	CH2277	Female	Two y/o	Control	Control	Feed Day	07:45	07:00 - 07:59	Morning	Attention
2019/09/06	CH2276	Female	Two y/o	Control	Control	Feed Day	07:50	07:00 - 07:59	Morning	Appetitive behaviour
2019/09/06	CH2277	Female	Two y/o	Control	Control	Feed Day	07:50	07:00 - 07:59	Morning	Attention
2019/09/06	CH2276	Female	Two y/o	Control	Control	Feed Day	07:55	07:00 - 07:59	Morning	Appetitive behaviour
2019/09/06	CH2277	Female	Two y/o	Control	Control	Feed Day	07:55	07:00 - 07:59	Morning	Locomotion
2019/09/06	CH2276	Female	Two y/o	Control	Control	Feed Day	08:00	08:00 - 08:59	Late Morning	Appetitive behaviour
2019/09/06	CH2277	Female	Two y/o	Control	Control	Feed Day	08:00	08:00 - 08:59	Late Morning	Appetitive behaviour
2019/09/06	CH2276	Female	Two y/o	Control	Control	Feed Day	08:05	08:00 - 08:59	Late Morning	Appetitive behaviour
2019/09/06	CH2277	Female	Two y/o	Control	Control	Feed Day	08:05	08:00 - 08:59	Late Morning	Appetitive behaviour
2019/09/06	CH2276	Female	Two y/o	Control	Control	Feed Day	08:10	08:00 - 08:59	Late Morning	Out of sight
2019/09/06	CH2277	Female	Two y/o	Control	Control	Feed Day	08:10	08:00 - 08:59	Late Morning	Locomotion
2019/09/06	CH2276	Female	Two y/o	Control	Control	Feed Day	08:15	08:00 - 08:59	Late Morning	Out of sight
2019/09/06	CH2277	Female	Two y/o	Control	Control	Feed Day	08:15	08:00 - 08:59	Late Morning	Auto-grooming
2019/09/06	CH2277	Female	Two y/o	Control	Control	Feed Day	08:20	08:00 - 08:59	Late Morning	Locomotion
2019/09/06	CH2276	Female	Two y/o	Control	Control	Feed Day	08:20	08:00 - 08:59	Late Morning	Locomotion
2019/09/06	CH2277	Female	Two y/o	Control	Control	Feed Day	08:25	08:00 - 08:59	Late Morning	Interspecific behaviour
2019/09/06	CH2276	Female	Two y/o	Control	Control	Feed Day	08:25	08:00 - 08:59	Late Morning	Interspecific behaviour
2019/09/06	CH2277	Female	Two y/o	Control	Control	Feed Day	08:30	08:00 - 08:59	Late Morning	Interspecific behaviour

2019/09/06	CH2276	Female	Two y/o	Control	Control	Feed Day	08:30	08:00 - 08:59	Late Morning	Interspecific behaviour
2019/09/06	CH2271	Male	Two y/o	Control	Control	Feed Day	08:35	08:00 - 08:59	Late Morning	Affiliative behaviour
2019/09/06	CH2277	Female	Two y/o	Control	Control	Feed Day	08:35	08:00 - 08:59	Late Morning	Appetitive behaviour
2019/09/06	CH2276	Female	Two y/o	Control	Control	Feed Day	08:35	08:00 - 08:59	Late Morning	Appetitive behaviour
2019/09/06	CH2271	Male	Two y/o	Control	Control	Feed Day	08:40	08:00 - 08:59	Late Morning	Locomotion
2019/09/06	CH2271	Male	Two y/o	Control	Control	Feed Day	08:45	08:00 - 08:59	Late Morning	Locomotion
2019/09/06	CH2271	Male	Two y/o	Control	Control	Feed Day	08:50	08:00 - 08:59	Late Morning	Locomotion
2019/09/06	CH2271	Male	Two y/o	Control	Control	Feed Day	08:55	08:00 - 08:59	Late Morning	Locomotion
2019/09/06	CH2271	Male	Two y/o	Control	Control	Feed Day	09:00	09:00 - 09:59	Late Morning	Locomotion
2019/09/06	CH2271	Male	Two y/o	Control	Control	Feed Day	09:05	09:00 - 09:59	Late Morning	Interspecific behaviour
2019/09/06	CH2271	Male	Two y/o	Control	Control	Feed Day	09:10	09:00 - 09:59	Late Morning	Locomotion
2019/09/06	CH2271	Male	Two y/o	Control	Control	Feed Day	09:15	09:00 - 09:59	Late Morning	Interspecific behaviour
2019/09/06	CH2271	Male	Two y/o	Control	Control	Feed Day	09:20	09:00 - 09:59	Late Morning	Appetitive behaviour
2019/09/06	CH2271	Male	Two y/o	Control	Control	Feed Day	09:25	09:00 - 09:59	Late Morning	Appetitive behaviour
2019/09/06	CH2271	Male	Two y/o	Control	Control	Feed Day	09:30	09:00 - 09:59	Late Morning	Appetitive behaviour
2019/09/07	CH2271	Male	Two y/o	Control	Control	Feed Day	07:45	07:00 - 07:59	Morning	Locomotion
2019/09/07	CH2271	Male	Two y/o	Control	Control	Feed Day	07:50	07:00 - 07:59	Morning	Inactive
2019/09/07	CH2271	Male	Two y/o	Control	Control	Feed Day	07:55	07:00 - 07:59	Morning	Inactive
2019/09/07	CH2271	Male	Two y/o	Control	Control	Feed Day	08:00	08:00 - 08:59	Late Morning	Inactive
2019/09/07	CH2271	Male	Two y/o	Control	Control	Feed Day	08:05	08:00 - 08:59	Late Morning	Inactive
2019/09/07	CH2271	Male	Two y/o	Control	Control	Feed Day	08:10	08:00 - 08:59	Late Morning	Inactive
2019/09/07	CH2271	Male	Two y/o	Control	Control	Feed Day	08:15	08:00 - 08:59	Late Morning	Inactive
2019/09/07	CH2271	Male	Two y/o	Control	Control	Feed Day	08:20	08:00 - 08:59	Late Morning	Scent-marking
2019/09/07	CH2271	Male	Two y/o	Control	Control	Feed Day	08:25	08:00 - 08:59	Late Morning	Auto-grooming
2019/09/07	CH2276	Female	Two y/o	Control	Control	Feed Day	08:25	08:00 - 08:59	Late Morning	Appetitive behaviour
2019/09/07	CH2277	Female	Two y/o	Control	Control	Feed Day	08:25	08:00 - 08:59	Late Morning	Inactive
2019/09/07	CH2271	Male	Two y/o	Control	Control	Feed Day	08:30	08:00 - 08:59	Late Morning	Interspecific behaviour
2019/09/07	CH2276	Female	Two y/o	Control	Control	Feed Day	08:30	08:00 - 08:59	Late Morning	Appetitive behaviour
2019/09/07	CH2277	Female	Two y/o	Control	Control	Feed Day	08:30	08:00 - 08:59	Late Morning	Appetitive behaviour
2019/09/07	CH2271	Male	Two y/o	Control	Control	Feed Day	08:35	08:00 - 08:59	Late Morning	Appetitive behaviour
2019/09/07	CH2276	Female	Two y/o	Control	Control	Feed Day	08:35	08:00 - 08:59	Late Morning	Appetitive behaviour
2019/09/07	CH2277	Female	Two y/o	Control	Control	Feed Day	08:35	08:00 - 08:59	Late Morning	Appetitive behaviour
2019/09/07	CH2271	Male	Two y/o	Control	Control	Feed Day	08:40	08:00 - 08:59	Late Morning	Appetitive behaviour
2019/09/07	CH2276	Female	Two y/o	Control	Control	Feed Day	08:40	08:00 - 08:59	Late Morning	Appetitive behaviour
2019/09/07	CH2277	Female	Two y/o	Control	Control	Feed Day	08:40	08:00 - 08:59	Late Morning	Appetitive behaviour
2019/09/07	CH2276	Female	Two y/o	Control	Control	Feed Day	08:45	08:00 - 08:59	Late Morning	Appetitive behaviour
2019/09/07	CH2277	Female	Two y/o	Control	Control	Feed Day	08:45	08:00 - 08:59	Late Morning	Appetitive behaviour

2019/09/07	CH2276	Female	Two y/o	Control	Control	Feed Day	08:50	08:00 - 08:59	Late Morning	Appetitive behaviour
2019/09/07	CH2277	Female	Two y/o	Control	Control	Feed Day	08:50	08:00 - 08:59	Late Morning	Appetitive behaviour
2019/09/07	CH2276	Female	Two y/o	Control	Control	Feed Day	08:55	08:00 - 08:59	Late Morning	Appetitive behaviour
2019/09/07	CH2277	Female	Two y/o	Control	Control	Feed Day	08:55	08:00 - 08:59	Late Morning	Appetitive behaviour
2019/09/07	CH2276	Female	Two y/o	Control	Control	Feed Day	09:00	09:00 - 09:59	Late Morning	Appetitive behaviour
2019/09/07	CH2277	Female	Two y/o	Control	Control	Feed Day	09:00	09:00 - 09:59	Late Morning	Appetitive behaviour
2019/09/07	CH2276	Female	Two y/o	Control	Control	Feed Day	09:05	09:00 - 09:59	Late Morning	Appetitive behaviour
2019/09/07	CH2277	Female	Two y/o	Control	Control	Feed Day	09:05	09:00 - 09:59	Late Morning	Standing
2019/09/07	CH2276	Female	Two y/o	Control	Control	Feed Day	09:10	09:00 - 09:59	Late Morning	Appetitive behaviour
2019/09/07	CH2277	Female	Two y/o	Control	Control	Feed Day	09:10	09:00 - 09:59	Late Morning	Interspecific behaviour
2019/09/07	CH2276	Female	Two y/o	Control	Control	Feed Day	09:15	09:00 - 09:59	Late Morning	Interspecific behaviour
2019/09/07	CH2277	Female	Two y/o	Control	Control	Feed Day	09:15	09:00 - 09:59	Late Morning	Interspecific behaviour
2019/09/07	CH2276	Female	Two y/o	Control	Control	Feed Day	09:20	09:00 - 09:59	Late Morning	Interspecific behaviour
2019/09/07	CH2277	Female	Two y/o	Control	Control	Feed Day	09:20	09:00 - 09:59	Late Morning	Interspecific behaviour
2019/09/10	CH2276	Female	Two y/o	Control	Control	Feed Day	07:45	07:00 - 07:59	Morning	Locomotion
2019/09/10	CH2277	Female	Two y/o	Control	Control	Feed Day	07:45	07:00 - 07:59	Morning	Attention
2019/09/10	CH2276	Female	Two y/o	Control	Control	Feed Day	07:50	07:00 - 07:59	Morning	Locomotion
2019/09/10	CH2277	Female	Two y/o	Control	Control	Feed Day	07:50	07:00 - 07:59	Morning	Locomotion
2019/09/10	CH2276	Female	Two y/o	Control	Control	Feed Day	07:55	07:00 - 07:59	Morning	Affiliative behaviour
2019/09/10	CH2277	Female	Two y/o	Control	Control	Feed Day	07:55	07:00 - 07:59	Morning	Affiliative behaviour
2019/09/10	CH2276	Female	Two y/o	Control	Control	Feed Day	08:00	08:00 - 08:59	Late Morning	Out of sight
2019/09/10	CH2277	Female	Two y/o	Control	Control	Feed Day	08:00	08:00 - 08:59	Late Morning	Appetitive behaviour
2019/09/10	CH2276	Female	Two y/o	Control	Control	Feed Day	08:05	08:00 - 08:59	Late Morning	Out of sight
2019/09/10	CH2277	Female	Two y/o	Control	Control	Feed Day	08:05	08:00 - 08:59	Late Morning	Locomotion
2019/09/10	CH2276	Female	Two y/o	Control	Control	Feed Day	08:10	08:00 - 08:59	Late Morning	Out of sight
2019/09/10	CH2277	Female	Two y/o	Control	Control	Feed Day	08:10	08:00 - 08:59	Late Morning	Inactive
2019/09/10	CH2276	Female	Two y/o	Control	Control	Feed Day	08:15	08:00 - 08:59	Late Morning	Out of sight
2019/09/10	CH2277	Female	Two y/o	Control	Control	Feed Day	08:15	08:00 - 08:59	Late Morning	Inactive
2019/09/10	CH2276	Female	Two y/o	Control	Control	Feed Day	08:20	08:00 - 08:59	Late Morning	Locomotion
2019/09/10	CH2277	Female	Two y/o	Control	Control	Feed Day	08:20	08:00 - 08:59	Late Morning	Interspecific behaviour
2019/09/10	CH2276	Female	Two y/o	Control	Control	Feed Day	08:25	08:00 - 08:59	Late Morning	Interspecific behaviour
2019/09/10	CH2277	Female	Two y/o	Control	Control	Feed Day	08:25	08:00 - 08:59	Late Morning	Appetitive behaviour
2019/09/10	CH2276	Female	Two y/o	Control	Control	Feed Day	08:30	08:00 - 08:59	Late Morning	Interspecific behaviour
2019/09/10	CH2277	Female	Two y/o	Control	Control	Feed Day	08:30	08:00 - 08:59	Late Morning	Interspecific behaviour
2019/09/10	CH2276	Female	Two y/o	Control	Control	Feed Day	08:35	08:00 - 08:59	Late Morning	Appetitive behaviour
2019/09/10	CH2277	Female	Two y/o	Control	Control	Feed Day	08:35	08:00 - 08:59	Late Morning	Appetitive behaviour
2019/09/10	CH2271	Male	Two y/o	Control	Control	Feed Day	08:40	08:00 - 08:59	Late Morning	Olfactory exploration

2019/09/10	CH2276	Female	Two y/o	Control	Control	Feed Day	08:40	08:00 - 08:59	Late Morning	Locomotion
2019/09/10	CH2277	Female	Two y/o	Control	Control	Feed Day	08:40	08:00 - 08:59	Late Morning	Locomotion
2019/09/10	CH2271	Male	Two y/o	Control	Control	Feed Day	08:45	08:00 - 08:59	Late Morning	Scent-marking
2019/09/10	CH2271	Male	Two y/o	Control	Control	Feed Day	08:50	08:00 - 08:59	Late Morning	Inactive
2019/09/10	CH2271	Male	Two y/o	Control	Control	Feed Day	08:55	08:00 - 08:59	Late Morning	Locomotion
2019/09/10	CH2271	Male	Two y/o	Control	Control	Feed Day	09:00	09:00 - 09:59	Late Morning	Locomotion
2019/09/10	CH2271	Male	Two y/o	Control	Control	Feed Day	09:05	09:00 - 09:59	Late Morning	Locomotion
2019/09/10	CH2271	Male	Two y/o	Control	Control	Feed Day	09:10	09:00 - 09:59	Late Morning	Affiliative behaviour
2019/09/10	CH2271	Male	Two y/o	Control	Control	Feed Day	09:15	09:00 - 09:59	Late Morning	Locomotion
2019/09/10	CH2271	Male	Two y/o	Control	Control	Feed Day	09:20	09:00 - 09:59	Late Morning	Appetitive behaviour
2019/09/10	CH2271	Male	Two y/o	Control	Control	Feed Day	09:25	09:00 - 09:59	Late Morning	Inactive
2019/09/10	CH2271	Male	Two y/o	Control	Control	Feed Day	09:30	09:00 - 09:59	Late Morning	Inactive
2019/09/10	CH2271	Male	Two y/o	Control	Control	Feed Day	09:35	09:00 - 09:59	Late Morning	Locomotion
2019/09/11	CH2271	Male	Two y/o	Control	Control	Feed Day	07:45	07:00 - 07:59	Morning	Locomotion
2019/09/11	CH2271	Male	Two y/o	Control	Control	Feed Day	07:50	07:00 - 07:59	Morning	Scent-marking
2019/09/11	CH2271	Male	Two y/o	Control	Control	Feed Day	07:55	07:00 - 07:59	Morning	Locomotion
2019/09/11	CH2271	Male	Two y/o	Control	Control	Feed Day	08:00	08:00 - 08:59	Late Morning	Locomotion
2019/09/11	CH2271	Male	Two y/o	Control	Control	Feed Day	08:05	08:00 - 08:59	Late Morning	Locomotion
2019/09/11	CH2271	Male	Two y/o	Control	Control	Feed Day	08:10	08:00 - 08:59	Late Morning	Locomotion
2019/09/11	CH2271	Male	Two y/o	Control	Control	Feed Day	08:15	08:00 - 08:59	Late Morning	Olfactory exploration
2019/09/11	CH2271	Male	Two y/o	Control	Control	Feed Day	08:20	08:00 - 08:59	Late Morning	Inactive
2019/09/11	CH2271	Male	Two y/o	Control	Control	Feed Day	08:25	08:00 - 08:59	Late Morning	Inactive
2019/09/11	CH2271	Male	Two y/o	Control	Control	Feed Day	08:30	08:00 - 08:59	Late Morning	Interspecific behaviour
2019/09/11	CH2271	Male	Two y/o	Control	Control	Feed Day	08:35	08:00 - 08:59	Late Morning	Appetitive behaviour
2019/09/11	CH2276	Female	Two y/o	Control	Control	Feed Day	08:35	08:00 - 08:59	Late Morning	Interspecific behaviour
2019/09/11	CH2277	Female	Two y/o	Control	Control	Feed Day	08:35	08:00 - 08:59	Late Morning	Interspecific behaviour
2019/09/11	CH2271	Male	Two y/o	Control	Control	Feed Day	08:40	08:00 - 08:59	Late Morning	Appetitive behaviour
2019/09/11	CH2276	Female	Two y/o	Control	Control	Feed Day	08:40	08:00 - 08:59	Late Morning	Appetitive behaviour
2019/09/11	CH2277	Female	Two y/o	Control	Control	Feed Day	08:40	08:00 - 08:59	Late Morning	Appetitive behaviour
2019/09/11	CH2276	Female	Two y/o	Control	Control	Feed Day	08:45	08:00 - 08:59	Late Morning	Appetitive behaviour
2019/09/11	CH2277	Female	Two y/o	Control	Control	Feed Day	08:45	08:00 - 08:59	Late Morning	Appetitive behaviour
2019/09/11	CH2276	Female	Two y/o	Control	Control	Feed Day	08:50	08:00 - 08:59	Late Morning	Appetitive behaviour
2019/09/11	CH2277	Female	Two y/o	Control	Control	Feed Day	08:50	08:00 - 08:59	Late Morning	Appetitive behaviour
2019/09/11	CH2276	Female	Two y/o	Control	Control	Feed Day	08:55	08:00 - 08:59	Late Morning	Appetitive behaviour
2019/09/11	CH2277	Female	Two y/o	Control	Control	Feed Day	08:55	08:00 - 08:59	Late Morning	Appetitive behaviour
2019/09/11	CH2276	Female	Two y/o	Control	Control	Feed Day	09:00	09:00 - 09:59	Late Morning	Appetitive behaviour
2019/09/11	CH2277	Female	Two y/o	Control	Control	Feed Day	09:00	09:00 - 09:59	Late Morning	Appetitive behaviour

Dataset S1: BEHAVIOUR_1

2019/09/11	CH2276	Female	Two y/o	Control	Control	Feed Day	09:05	09:00 - 09:59	Late Morning	Interspecific behaviour
2019/09/11	CH2277	Female	Two y/o	Control	Control	Feed Day	09:05	09:00 - 09:59	Late Morning	Standing
2019/09/11	CH2276	Female	Two y/o	Control	Control	Feed Day	09:10	09:00 - 09:59	Late Morning	Inactive
2019/09/11	CH2277	Female	Two y/o	Control	Control	Feed Day	09:10	09:00 - 09:59	Late Morning	Inactive
2019/09/11	CH2276	Female	Two y/o	Control	Control	Feed Day	09:15	09:00 - 09:59	Late Morning	Inactive
2019/09/11	CH2277	Female	Two y/o	Control	Control	Feed Day	09:15	09:00 - 09:59	Late Morning	Inactive
2019/09/11	CH2276	Female	Two y/o	Control	Control	Feed Day	09:20	09:00 - 09:59	Late Morning	Inactive
2019/09/11	CH2277	Female	Two y/o	Control	Control	Feed Day	09:20	09:00 - 09:59	Late Morning	Inactive
2019/09/11	CH2276	Female	Two y/o	Control	Control	Feed Day	09:25	09:00 - 09:59	Late Morning	Inactive
2019/09/11	CH2277	Female	Two y/o	Control	Control	Feed Day	09:25	09:00 - 09:59	Late Morning	Inactive
2019/09/11	CH2276	Female	Two y/o	Control	Control	Feed Day	09:30	09:00 - 09:59	Late Morning	Inactive
2019/09/11	CH2277	Female	Two y/o	Control	Control	Feed Day	09:30	09:00 - 09:59	Late Morning	Inactive

Date	Cheetah ID	Sex	Age Group	Week	Study Period	Feed/Fast Day	Inactive	Appetitve behaviour	Attention	Auto-grooming	Environmental enrichment	Locomotion	Maintenance	Olfactory exploration	Scent-marking	Standing	Stereotypical	Vocalisation	Affiliative behaviour	Agnostic behaviour	Interspecific behaviour	Out of sight	Inactive X	Appetitve behaviour X	Attention X	Auto-grooming X	Locomotion X	Maintenance X	Olfactory exploration X	Scent-marking X	Standing X	Stereotypical X	Vocalisation X	Affiliative behaviour X	Agnostic behaviour X	Interspecific behaviour X	Out of sight X
2019/04/24	CH2207	Female	Three y/o	Treatment (Wk 1)	Treatment	Fast Day		0,33		0,08		0,25			0,33										-0,28		-0,07	-0,28			0,00							
2019/04/30	CH2207	Female	Three y/o	Treatment (Wk 2)	Treatment	Feed Day	0,25	0,50		0,08		0,17											-0,64	-0,18		-0,07	-0,36										
2019/05/01	CH2207	Female	Three y/o	Treatment (Wk 2)	Treatment	Fast Day	0,33	0,17				0,50											-0,55	-0,43			-0,14										
2019/05/05	CH2207	Female	Three y/o	Treatment (Wk 2)	Treatment	Fast Day	0,42	0,08		0,08		0,33			0,08								-0,47	-0,58		-0,07	-0,22			-0,09							
2019/05/07	CH2207	Female	Three y/o	Treatment (Wk 3)	Treatment	Feed Day	0,42			0,08		0,50											-0,47			-0,07	-0,14										
2019/05/08	CH2207	Female	Three y/o	Treatment (Wk 3)	Treatment	Fast Day	0,83	0,08	0,08													-0,12	-0,58	-0,21												
2019/05/10	CH2207	Female	Three y/o	Treatment (Wk 3)	Treatment	Feed Day	1,00																0,00														
2019/05/11	CH2207	Female	Three y/o	Treatment (Wk 3)	Treatment	Fast Day	0,33		0,08	0,42		0,08			0,08								-0,55		-0,21	0,00	-0,51			-0,09							
2019/05/12	CH2207	Female	Three y/o	Treatment (Wk 3)	Treatment	Fast Day	0,17		0,25			0,50	0,08										-0,75		-0,06		-0,14	-0,21								
2019/05/13	CH2207	Female	Three y/o	Treatment (Wk 3)	Treatment	Feed Day	0,08			0,17		0,75											-0,88			-0,02	-0,06										
2019/05/15	CH2205	Male	Three y/o	Treatment (Wk 1)	Treatment	Fast Day	0,67		0,08			0,17								0,08		-0,25		-0,21		-0,36									-0,30	
2019/05/15	CH2206	Male	Three y/o	Treatment (Wk 1)	Treatment	Fast Day	0,67		0,33														-0,25		-0,04												
2019/05/17	CH2205	Male	Three y/o	Treatment (Wk 1)	Treatment	Feed Day	0,17		0,17			0,58		0,08										-0,75		-0,10		-0,11		-0,06								
2019/05/17	CH2206	Male	Three y/o	Treatment (Wk 1)	Treatment	Feed Day	0,08	0,25	0,25			0,25		0,08	0,08								-0,88	-0,34	-0,06		-0,28		-0,06	-0,09							
2019/05/20	CH2205	Male	Three y/o	Treatment (Wk 1)	Treatment	Feed Day	0,33	0,08	0,33			0,17		0,08										-0,55	-0,58	-0,04		-0,36		-0,06								
2019/05/20	CH2206	Male	Three y/o	Treatment (Wk 1)	Treatment	Feed Day	0,08	0,42	0,25			0,17		0,08										-0,88	-0,22	-0,06		-0,36		-0,06								
2019/05/22	CH2205	Male	Three y/o	Treatment (Wk 2)	Treatment	Fast Day	0,08	0,25		0,08		0,25						0,25			0,08		-0,88	-0,34		-0,07	-0,28							-0,01			-0,30	
2019/05/22	CH2206	Male	Three y/o	Treatment (Wk 2)	Treatment	Fast Day	0,08	0,42	0,08	0,08		0,25									0,08		-0,88	-0,22	-0,21	-0,07	-0,28									-0,30	
2019/05/23	CH2205	Male	Three y/o	Treatment (Wk 2)	Treatment	Feed Day	0,58		0,25			0,08			0,08								-0,32		-0,06		-0,51			-0,09							
2019/05/23	CH2206	Male	Three y/o	Treatment (Wk 2)	Treatment	Feed Day	0,58		0,25	0,08		0,08											-0,32		-0,06	-0,07	-0,51										
2019/05/26	CH2205	Male	Three y/o	Treatment (Wk 2)	Treatment	Fast Day	0,83		0,08	0,08												-0,12		-0,21	-0,07												
2019/05/26	CH2206	Male	Three y/o	Treatment (Wk 2)	Treatment	Fast Day	1,00																0,00														
2019/05/27	CH2205	Male	Three y/o	Treatment (Wk 2)	Treatment	Feed Day	0,17					0,17		0,08		0,08					0,50		-0,75				-0,36		-0,06		-0,21					-0,06	
2019/05/27	CH2206	Male	Three y/o	Treatment (Wk 2)	Treatment	Feed Day		0,33		0,08		0,33										0,25			-0,28		-0,07	-0,22									-0,13	
2019/05/28	CH2205	Male	Three y/o	Treatment (Wk 2)	Treatment	Feed Day			0,33			0,25		0,08	0,17						0,17				-0,04		-0,28		-0,06	-0,02						-0,18	
2019/05/28	CH2206	Male	Three y/o	Treatment (Wk 2)	Treatment	Feed Day		0,50	0,08			0,25			0,17									-0,18	-0,21		-0,28			-0,02							
2019/05/29	CH2205	Male	Three y/o	Treatment (Wk 3)	Treatment	Fast Day	0,17		0,08			0,33		0,25				0,08			0,08		-0,75		-0,21		-0,22		-0,01				-0,08			-0,30	
2019/05/29	CH2206	Male	Three y/o	Treatment (Wk 3)	Treatment	Fast Day	0,08	0,42				0,42		0,08										-0,88	-0,22			-0,17		-0,06								
2019/05/30	CH2205	Male	Three y/o	Treatment (Wk 3)	Treatment	Feed Day	0,58			0,08		0,25									0,08		-0,32			-0,07	-0,28									-0,30	
2019/05/30	CH2206	Male	Three y/o	Treatment (Wk 3)	Treatment	Feed Day	0,58					0,25									0,17		-0,32				-0,28									-0,18	
2019/05/31	CH2205	Male	Three y/o	Treatment (Wk 3)	Treatment	Feed Day	0,42		0,17			0,25									0,17		-0,47		-0,10		-0,28									-0,18	
2019/05/31	CH2206	Male	Three y/o	Treatment (Wk 3)	Treatment	Feed Day	0,25	0,08	0,08			0,50		0,08										-0,64	-0,58	-0,21		-0,14		-0,06								
2019/06/01	CH2205	Male	Three y/o	Treatment (Wk 3)	Treatment	Feed Day	0,08	0,75	0,08			0,08											-0,88	-0,07	-0,21		-0,51										
2019/06/01	CH2206	Male	Three y/o	Treatment (Wk 3)	Treatment	Feed Day	0,08	0,92															-0,88	-0,02													
2019/06/02	CH2205	Male	Three y/o	Treatment (Wk 3)	Treatment	Fast Day	0,58	0,08				0,08	0,08		0,08						0,08		-0,32	-0,58			-0,51	-0,21		-0,09						-0,30	
2019/06/02	CH2206	Male	Three y/o	Treatment (Wk 3)	Treatment	Fast Day	0,58	0,08							0,08						0,25		-0,32	-0,58						-0,09						-0,13	
2019/06/03	CH2205	Male	Three y/o	Treatment (Wk 3)	Treatment	Feed Day	0,25		0,17			0,33		0,08	0,17								-0,64		-0,10		-0,22		-0,06	-0,02							
2019/06/03	CH2206	Male	Three y/o	Treatment (Wk 3)	Treatment	Feed Day	0,25		0,17	0,08		0,50											-0,64		-0,10	-0,07	-0,14										
2019/06/04	CH2205	Male	Three y/o	Treatment (Wk 3)	Treatment	Feed Day	0,17	0,08	0,08			0,25		0,08	0,08						0,25		-0,75	-0,58	-0,21		-0,28		-0,06	-0,09						-0,13	
2019/06/04	CH2206	Male	Three y/o	Treatment (Wk 3)	Treatment	Feed Day	0,25		0,08			0,67											-0,64		-0,21		-0,08										
2019/06/06	CH2205	Male	Three y/o	Control	Control	Feed Day	0,25	0,33	0,42													-0,64	-0,28	-0,03												
2019/06/06	CH2206	Male	Three y/o	Control	Control	Feed Day	0,25	0,67	0,08													-0,64	-0,10	-0,21												
2019/06/06	CH2207	Female	Three y/o	Control	Control	Feed Day		0,17				0,08										0,75		-0,43			-0,51											-0,18
2019/06/07	CH2205	Male	Three y/o	Control	Control	Feed Day										0,17				0,08		0,33	0,42								-0,02					-0,07		-0,10	-0,46
2019/06/07	CH2206	Male	Three y/o	Control	Control	Feed Day			0,08	0,08		0,08		0,08							0,08		0,17	0,42			-0,21	-0,07	-0,51		-0,06					-0,07		-0,18	-0,46
2019/06/07	CH2207	Female	Three y/o	Control	Control	Feed Day	0,75	0,08	0,17													-0,18	-0,58	-0,10												
2019/06/08	CH2205	Male	Three y/o	Control	Control	Feed Day	0,58		0,08			0,17			0,08				0,08				-0,32		-0,21		-0,36			-0,09				-0,07			
2019/06/08	CH2206	Male	Three y/o	Control	Control	Feed Day	0,50		0,17	0,08		0,17							0,08				-0,39		-0,10	-0,07	-0,36							-0,07			
2019/06/08	CH2207	Female	Three y/o	Control	Control	Feed Day	0,08		0,58	0,08		0,17			0,08								-0,88		-0,01	-0,07	-0,36			-0,09							
2019/06/11	CH2205	Male	Three y/o	Control	Control	Feed Day	0,42	0,08				0,25						0,08	0,08		0,08		-0,47	-0,58			-0,28							-0,08	-0,07		-0,30	
2019/06/11	CH2206	Male	Three y/o	Control	Control	Feed Day	0,33	0,17				0,25			0,08				0,08		0,08		-0,55	-0,43			-0,28			-0,09				-0,07		-0,30	
2019/06/11	CH2207	Female	Three y/o	Control	Control	Feed Day	0,17	0,42				0,42											-0,75	-0,22			-0,17										
2019/06/12	CH2205	Male	Three y/o	Control	Control	Feed Day	0,08		0,25			0,42			0,17						0,08	-0,88		-0,06		-0,17			-0,02							-0,84
2019/06/12	CH2206	Male	Three y/o	Control	Control	Feed Day	0,25		0,08			0,25		0,08							0,33		-0,64		-0,21		-0,28		-0,06							-0,10	
2019/06/12	CH2207	Female	Three y/o	Control	Control	Feed Day	0,08	0,08	0,58	0,08		0,08			0,08								-0,88	-0,58	-0,01	-0,07	-0,51			-0,09							
2019/06/13	CH2205	Male	Three y/o	Control	Control	Feed Day	0,17		0,33			0,33			0,17								-0,75		-0,04		-0,22			-0,02							
2019/06/13	CH2206	Male	Three y/o	Control	Control	Feed Day	0,33		0,25			0,42											-0,55		-0,06		-0,17										
2019/06/13	CH2207	Female	Three y/o	Control	Control	Feed Day	0,42	0,08	0,08	0,17		0,08							0,08	0,08			-0,47	-0,58	-0,21	-0,02	-0,51							-0,07	-0,06		
2019/06/14	CH2205	Male	Three y/o	Control	Control	Feed Day	0,50					0,33		0,17										-0,39				-0,22		-0,01								
2019/06/14	CH2206	Male	Three y/o	Control	Control	Feed Day	0,50		0,08			0,25			0,17								-0,39		-0,21		-0,28			-0,02							
2019/06/14	CH2207	Female	Three y/o	Control	Control	Feed Day	0,33	0,33	0,08	0,08		0,17											-0,55	-0,28	-0,21	-0,07	-0,36										
2019/06/15	CH2205	Male	Three y/o	Control	Control	Feed Day	0,50			0,08		0,25						0,08			0,08		-0,39			-0,07	-0,28							-0,08			-0,30	
2019/06/15	CH2206	Male	Three y/o	Control	Control	Feed Day	0,67					0,08		0,08							0,17		-0,25				-0,51		-0,06							-0,18	
2019/06/15	CH2207	Female	Three y/o	Control	Control	Feed Day	0,08	0,42	0,08	0,25		0,08								0,08				-0,88	-0,22	-0,21	-0,01	-0,51								-0,06		
2019/06/18	CH2205	Male	Three y/o	Control	Control	Feed Day	0,83		0,08									0,08					-0,12		-0,21								-0,08				
2019/06/18	CH2206	Male	Three y/o	Control	Control	Feed Day	0,58					0,17		0,08							0,17		-0,32				-0,36		-0,06							-0,18	
2019/06/18	CH2207	Female	Three y/o	Control	Control	Feed Day	0,50	0,25				0,08			0,17								-0,39	-0,34			-0,51			-0,02							
2019/06/19	CH2205	Male	Three y/o	Control	Control	Feed Day	0,50		0,08			0,08			0,08				0,08		0,17		-0,39		-0,21		-0,51			-0,09				-0,07		-0,18	
2019/06/19	CH2206	Male	Three y/o	Control	Control	Feed Day	0,58			0,08				0,17					0,17				-0,32			-0,07			-0,01					-0,02			
2019/06/19	CH2207	Female	Three y/o	Control	Control	Feed Day	0,17		0,33	0,25		0,17			0,08								-0,75		-0,04	-0,01	-0,36			-0,09							
2019/07/09	CH2271	Male	Two y/o	Treatment (Wk 1)	Treatment	Feed Day	0,25					0,08			0,08						0,58		-0,64				-0,51			-0,09						-0,04	
2019/07/10	CH2271	Male	Two y/o	Treatment (Wk 1)	Treatment	Fast Day	0,58		0,08			0,08			0,08				0,08		0,08		-0,32		-0,21		-0,51			-0,09				-0,07		-0,30	
2019/07/11	CH2271	Male	Two y/o	Treatment (Wk 1)	Treatment	Feed Day		0,33				0,17			0,17						0,33			-0,28			-0,36			-0,02						-0,10	
2019/07/12	CH2271	Male	Two y/o	Treatment (Wk 1)	Treatment	Feed Day			0,33			0,50							0,08		0,08				-0,04		-0,14							-0,07		-0,30	
2019/07/15	CH2271	Male	Two y/o	Treatment (Wk 1)	Treatment	Feed Day			0,17			0,67							0,08		0,08				-0,10		-0,08							-0,07		-0,30	
2019/07/16	CH2271	Male	Two y/o	Treatment (Wk 2)	Treatment	Feed Day	0,25	0,17	0,08			0,17	0,08						0,25				-0,64	-0,43	-0,21		-0,36	-0,21						-0,01			
2019/07/17	CH2271	Male	Two y/o	Treatment (Wk 2)	Treatment	Fast Day	0,42		0,08			0,17							0,17		0,17		-0,47		-0,21		-0,36							-0,02		-0,18	
2019/07/18	CH2271	Male	Two y/o	Treatment (Wk 2)	Treatment	Feed Day	0,67		0,11											0,11		0,11		-0,25		-0,16									-0,04		-0,25	
2019/07/19	CH2271	Male	Two y/o	Treatment (Wk 2)	Treatment	Feed Day		0,58				0,17			0,08				0,08		0,08			-0,14			-0,36			-0,09				-0,07		-0,30	
2019/07/22	CH2271	Male	Two y/o	Treatment (Wk 2)	Treatment	Feed Day	0,17					0,58			0,08						0,17		-0,75				-0,11			-0,09						-0,18	
2019/07/23	CH2271	Male	Two y/o	Treatment (Wk 3)	Treatment	Feed Day	0,08		0,17			0,58			0,17								-0,88		-0,10		-0,11			-0,02							
2019/07/24	CH2271	Male	Two y/o	Treatment (Wk 3)	Treatment	Fast Day	0,83					0,08									0,08		-0,12				-0,51									-0,30	
2019/07/25	CH2271	Male	Two y/o	Treatment (Wk 3)	Treatment	Feed Day	0,08	0,17	0,17			0,33		0,08					0,17				-0,88	-0,43	-0,10		-0,22		-0,06					-0,02			
2019/07/26	CH2271	Male	Two y/o	Treatment (Wk 3)	Treatment	Feed Day			0,25			0,75														-0,06		-0,06										
2019/07/29	CH2271	Male	Two y/o	Treatment (Wk 3)	Treatment	Feed Day	0,33	0,08				0,17			0,08	0,08					0,25		-0,55	-0,58			-0,36			-0,09	-0,21					-0,13	
2019/08/01	CH2276	Female	Two y/o	Treatment (Wk 1)	Treatment	Feed Day	0,67		0,08						0,08		0,08				0,08		-0,25		-0,21					-0,09		-0,21				-0,30	
2019/08/01	CH2277	Female	Two y/o	Treatment (Wk 1)	Treatment	Feed Day	0,67		0,08						0,08		0,08				0,08		-0,25		-0,21					-0,09		-0,21				-0,30	
2019/08/03	CH2276	Female	Two y/o	Treatment (Wk 1)	Treatment	Feed Day	0,58								0,08						0,33		-0,32							-0,09						-0,10	
2019/08/03	CH2277	Female	Two y/o	Treatment (Wk 1)	Treatment	Feed Day	0,58									0,08					0,33		-0,32								-0,21					-0,10	
2019/08/05	CH2276	Female	Two y/o	Treatment (Wk 1)	Treatment	Feed Day	0,88					0,13											-0,09				-0,42										
2019/08/05	CH2277	Female	Two y/o	Treatment (Wk 1)	Treatment	Feed Day	0,75								0,13						0,13		-0,18							-0,04						-0,23	
2019/08/07	CH2276	Female	Two y/o	Treatment (Wk 2)	Treatment	Fast Day	0,63		0,13			0,13									0,13		-0,28		-0,14		-0,42									-0,23	
2019/08/07	CH2277	Female	Two y/o	Treatment (Wk 2)	Treatment	Fast Day	0,75					0,13			0,13								-0,18				-0,42			-0,04							
2019/08/09	CH2276	Female	Two y/o	Treatment (Wk 2)	Treatment	Feed Day	1,00																0,00														
2019/08/09	CH2277	Female	Two y/o	Treatment (Wk 2)	Treatment	Feed Day	0,75			0,13		0,13											-0,18			-0,03	-0,42										
2019/08/11	CH2276	Female	Two y/o	Treatment (Wk 2)	Treatment	Fast Day	0,42	0,17				0,25								0,08	0,08		-0,47	-0,43			-0,28								-0,06	-0,30	
2019/08/11	CH2277	Female	Two y/o	Treatment (Wk 2)	Treatment	Fast Day	0,33	0,33				0,17								0,08	0,08		-0,55	-0,28			-0,36								-0,06	-0,30	
2019/08/13	CH2276	Female	Two y/o	Treatment (Wk 2)	Treatment	Feed Day	0,75															0,25		-0,18													-0,13	
2019/08/13	CH2277	Female	Two y/o	Treatment (Wk 2)	Treatment	Feed Day	0,75															0,25		-0,18													-0,13	
2019/08/14	CH2276	Female	Two y/o	Treatment (Wk 3)	Treatment	Fast Day	1,00																0,00														
2019/08/14	CH2277	Female	Two y/o	Treatment (Wk 3)	Treatment	Fast Day	1,00																0,00														
2019/08/19	CH2276	Female	Two y/o	Treatment (Wk 3)	Treatment	Feed Day	0,75								0,08						0,17		-0,18							-0,09						-0,18	
2019/08/19	CH2277	Female	Two y/o	Treatment (Wk 3)	Treatment	Feed Day	0,75			0,08												0,17		-0,18			-0,07											-0,18	
2019/08/20	CH2276	Female	Two y/o	Treatment (Wk 3)	Treatment	Feed Day	0,75															0,25		-0,18													-0,13	
2019/08/20	CH2277	Female	Two y/o	Treatment (Wk 3)	Treatment	Feed Day	0,75															0,25		-0,18													-0,13	
2019/08/22	CH2271	Male	Two y/o	Control	Control	Feed Day	0,58	0,25				0,08	0,08										-0,32	-0,34			-0,51	-0,21										
2019/08/22	CH2276	Female	Two y/o	Control	Control	Fast Day	0,75												0,13		0,125		-0,18											-0,03		-0,23	
2019/08/22	CH2277	Female	Two y/o	Control	Control	Fast Day	0,63					0,25							0,13				-0,28				-0,28							-0,03			
2019/08/23	CH2271	Male	Two y/o	Control	Control	Feed Day	0,92		0,08														-0,06		-0,21												
2019/08/23	CH2276	Female	Two y/o	Control	Control	Feed Day	0,83								0,08					0,08				-0,12							-0,09				-0,07			
2019/08/23	CH2277	Female	Two y/o	Control	Control	Feed Day	0,75			0,08		0,08							0,08				-0,18			-0,07	-0,51							-0,07			
2019/08/26	CH2271	Male	Two y/o	Control	Control	Feed Day	0,75					0,08			0,08						0,08		-0,18				-0,51			-0,09						-0,30	
2019/08/26	CH2276	Female	Two y/o	Control	Control	Feed Day	1,00																0,00														
2019/08/26	CH2277	Female	Two y/o	Control	Control	Feed Day	1,00																0,00														
2019/08/27	CH2271	Male	Two y/o	Control	Control	Feed Day	0,33	0,08				0,42									0,17		-0,55	-0,58			-0,17									-0,18	
2019/08/27	CH2276	Female	Two y/o	Control	Control	Feed Day	0,42	0,17	0,08			0,08								0,17	0,08		-0,47	-0,43	-0,21		-0,51								-0,02	-0,30	
2019/08/27	CH2277	Female	Two y/o	Control	Control	Feed Day	0,17	0,33	0,08	0,08		0,08									0,25		-0,75	-0,28	-0,21	-0,07	-0,51									-0,13	
2019/08/28	CH2271	Male	Two y/o	Control	Control	Feed Day	0,25	0,33				0,42											-0,64	-0,28			-0,17										
2019/08/28	CH2276	Female	Two y/o	Control	Control	Feed Day	0,17	0,33				0,25			0,17						0,08		-0,75	-0,28			-0,28			-0,02						-0,30	
2019/08/28	CH2277	Female	Two y/o	Control	Control	Feed Day	0,08	0,58	0,17			0,08									0,08		-0,88	-0,14	-0,10		-0,51									-0,30	
2019/08/29	CH2271	Male	Two y/o	Control	Control	Feed Day	0,58					0,33							0,08				-0,32				-0,22							-0,07			
2019/08/29	CH2276	Female	Two y/o	Control	Control	Feed Day	0,50					0,17									0,33		-0,39				-0,36									-0,10	
2019/08/29	CH2277	Female	Two y/o	Control	Control	Feed Day	0,08	0,50				0,25									0,17		-0,88	-0,18			-0,28									-0,18	
2019/08/30	CH2271	Male	Two y/o	Control	Control	Feed Day		0,25	0,25	0,08		0,33	0,08											-0,34	-0,06	-0,07	-0,22	-0,21										
2019/08/30	CH2276	Female	Two y/o	Control	Control	Feed Day	0,92		0,08														-0,06		-0,21												
2019/08/30	CH2277	Female	Two y/o	Control	Control	Feed Day	0,67	0,08				0,17									0,08		-0,25	-0,58			-0,36									-0,30	

2019/08/31	CH2271	Male	Two y/o	Control	Control	Feed Day	0,67		0,08		0,17				0,08				-0,25		-0,21		-0,36				-0,21			
2019/08/31	CH2276	Female	Two y/o	Control	Control	Feed Day	0,92				0,08								-0,06				-0,51							
2019/08/31	CH2277	Female	Two y/o	Control	Control	Feed Day	0,75										0,25		-0,18										-0,13	
2019/09/03	CH2271	Male	Two y/o	Control	Control	Feed Day	0,83				0,17								-0,12				-0,36							
2019/09/03	CH2276	Female	Two y/o	Control	Control	Feed Day	0,92				0,08								-0,06				-0,51							
2019/09/03	CH2277	Female	Two y/o	Control	Control	Feed Day	1,00												0,00											
2019/09/04	CH2271	Male	Two y/o	Control	Control	Feed Day	0,83			0,08			0,08						-0,12			-0,07			-0,09					
2019/09/04	CH2276	Female	Two y/o	Control	Control	Feed Day	0,42											0,58	-0,47											-0,32
2019/09/04	CH2277	Female	Two y/o	Control	Control	Feed Day	1,00												0,00											
2019/09/05	CH2271	Male	Two y/o	Control	Control	Feed Day	0,25		0,08		0,25		0,17				0,25		-0,64		-0,21		-0,28		-0,02				-0,13	
2019/09/05	CH2276	Female	Two y/o	Control	Control	Feed Day	0,83										0,17		-0,12										-0,18	
2019/09/05	CH2277	Female	Two y/o	Control	Control	Feed Day	0,83										0,17		-0,12										-0,18	
2019/09/06	CH2271	Male	Two y/o	Control	Control	Feed Day		0,25			0,50					0,08	0,17			-0,34			-0,14					-0,07	-0,18	
2019/09/06	CH2276	Female	Two y/o	Control	Control	Feed Day		0,50			0,17						0,17	0,17		-0,18			-0,36						-0,18	-0,72
2019/09/06	CH2277	Female	Two y/o	Control	Control	Feed Day		0,25	0,17	0,08	0,25						0,25			-0,34	-0,10	-0,07	-0,28						-0,13	
2019/09/07	CH2271	Male	Two y/o	Control	Control	Feed Day	0,50	0,17		0,08	0,08		0,08				0,08		-0,39	-0,43		-0,07	-0,51		-0,09				-0,30	
2019/09/07	CH2276	Female	Two y/o	Control	Control	Feed Day		0,83									0,17			-0,05									-0,18	
2019/09/07	CH2277	Female	Two y/o	Control	Control	Feed Day	0,08	0,58						0,08			0,25		-0,88	-0,14						-0,21			-0,13	
2019/09/10	CH2271	Male	Two y/o	Control	Control	Feed Day	0,25	0,08			0,42	0,08	0,08			0,08			-0,64	-0,58			-0,17	-0,06	-0,09			-0,07		
2019/09/10	CH2276	Female	Two y/o	Control	Control	Feed Day		0,08			0,33					0,08	0,17	0,33		-0,58			-0,22					-0,07	-0,18	-0,54
2019/09/10	CH2277	Female	Two y/o	Control	Control	Feed Day	0,17	0,25	0,08		0,25					0,08	0,17		-0,75	-0,34	-0,21		-0,28					-0,07	-0,18	
2019/09/11	CH2271	Male	Two y/o	Control	Control	Feed Day	0,17	0,17			0,42	0,08	0,08				0,08		-0,75	-0,43			-0,17	-0,06	-0,09				-0,30	
2019/09/11	CH2276	Female	Two y/o	Control	Control	Feed Day	0,42	0,42									0,17		-0,47	-0,22									-0,18	
2019/09/11	CH2277	Female	Two y/o	Control	Control	Feed Day	0,42	0,42						0,08			0,08		-0,47	-0,22						-0,21			-0,30	

Dataset S1: FAECAL

Date	Cheetah ID	Sex	Age Group	Week	Study Period	Feed/Fast Day	Faecal Consistency Score	Faecal Consistency Score (grade)	Faecal Glucocorticoid Metabolite Concentration (µg/g DW)	Undigested Food	Faecal Consistency Score (grade) X	Faecal Glucocorticoid Metabolite Concentration (µg/g DW) X
2019/04/24	CH2207	Female	Three y/o	Treatment (Wk 1)	Treatment	Fast Day	Firm	5		No	4,97	
2019/04/25	CH2207	Female	Three y/o	Treatment (Wk 1)	Treatment	Feed Day	Soft with shape	3		No	3,11	
2019/04/26	CH2207	Female	Three y/o	Treatment (Wk 1)	Treatment	Feed Day	Firm and dry	4		No	4,12	
2019/04/27	CH2207	Female	Three y/o	Treatment (Wk 1)	Treatment	Fast Day	Soft without shape	2		No	1,84	
2019/04/28	CH2207	Female	Three y/o	Treatment (Wk 1)	Treatment	Fast Day	Firm	5		No	4,97	
2019/04/30	CH2207	Female	Three y/o	Treatment (Wk 2)	Treatment	Feed Day	Firm and dry	4		No	4,12	
2019/05/01	CH2207	Female	Three y/o	Treatment (Wk 2)	Treatment	Fast Day	Firm and dry	4		No	4,12	
2019/05/02	CH2207	Female	Three y/o	Treatment (Wk 2)	Treatment	Feed Day	Soft without shape	2		No	1,84	
2019/05/03	CH2207	Female	Three y/o	Treatment (Wk 2)	Treatment	Feed Day	Firm and dry	4		No	4,12	
2019/05/04	CH2207	Female	Three y/o	Treatment (Wk 2)	Treatment	Fast Day	Firm and dry	4		No	4,12	
2019/05/05	CH2207	Female	Three y/o	Treatment (Wk 2)	Treatment	Fast Day	Soft with shape	3		No	3,11	
2019/05/06	CH2207	Female	Three y/o	Treatment (Wk 2)	Treatment	Feed Day	Soft with shape	3		No	3,11	
2019/05/07	CH2207	Female	Three y/o	Treatment (Wk 3)	Treatment	Feed Day	Firm	5		Yes	4,97	
2019/05/08	CH2207	Female	Three y/o	Treatment (Wk 3)	Treatment	Fast Day	Firm	5		No	4,97	
2019/05/09	CH2207	Female	Three y/o	Treatment (Wk 3)	Treatment	Feed Day	Firm and dry	4		No	4,12	
2019/05/10	CH2207	Female	Three y/o	Treatment (Wk 3)	Treatment	Feed Day	Firm	5		No	4,97	
2019/05/11	CH2207	Female	Three y/o	Treatment (Wk 3)	Treatment	Fast Day	Soft with shape	3		No	3,11	
2019/05/12	CH2207	Female	Three y/o	Treatment (Wk 3)	Treatment	Fast Day	Firm	5		No	4,97	
2019/05/13	CH2207	Female	Three y/o	Treatment (Wk 3)	Treatment	Feed Day	Firm and dry	4		No	4,12	
2019/05/15	CH2205	Male	Three y/o	Treatment (Wk 1)	Treatment	Fast Day	Firm	5	0,96	No	4,97	-0,04
2019/05/15	CH2206	Male	Three y/o	Treatment (Wk 1)	Treatment	Fast Day	Firm	5	1,56	No	4,97	0,47
2019/05/16	CH2205	Male	Three y/o	Treatment (Wk 1)	Treatment	Feed Day	Soft with shape	3	1,21	No	3,11	0,19
2019/05/16	CH2206	Male	Three y/o	Treatment (Wk 1)	Treatment	Feed Day	Firm and dry	4	1,07	No	4,12	0,06
2019/05/17	CH2205	Male	Three y/o	Treatment (Wk 1)	Treatment	Feed Day	Firm	5	0,99	No	4,97	-0,01
2019/05/17	CH2206	Male	Three y/o	Treatment (Wk 1)	Treatment	Feed Day	Firm and dry	4	1,01	No	4,12	0,01
2019/05/18	CH2206	Male	Three y/o	Treatment (Wk 1)	Treatment	Fast Day	Soft with shape	3	1,35	Yes	3,11	0,31
2019/05/19	CH2205	Male	Three y/o	Treatment (Wk 1)	Treatment	Fast Day	Firm and dry	4	0,83	No	4,12	-0,18
2019/05/19	CH2206	Male	Three y/o	Treatment (Wk 1)	Treatment	Fast Day	Soft with shape	3	1,32	No	3,11	0,28
2019/05/21	CH2205	Male	Three y/o	Treatment (Wk 1)	Treatment	Feed Day	Soft with shape	3	1,60	No	3,11	0,50
2019/05/21	CH2206	Male	Three y/o	Treatment (Wk 1)	Treatment	Feed Day	Soft with shape	3	1,68	No	3,11	0,56
2019/05/22	CH2205	Male	Three y/o	Treatment (Wk 2)	Treatment	Fast Day	Soft with shape	3	1,19	Yes	3,11	0,17
2019/05/22	CH2206	Male	Three y/o	Treatment (Wk 2)	Treatment	Fast Day	Firm and dry	4	1,10	No	4,12	0,09
2019/05/23	CH2205	Male	Three y/o	Treatment (Wk 2)	Treatment	Feed Day	Firm and dry	4	0,71	No	4,12	-0,32
2019/05/23	CH2206	Male	Three y/o	Treatment (Wk 2)	Treatment	Feed Day	Firm and dry	4	1,15	No	4,12	0,14
2019/05/24	CH2205	Male	Three y/o	Treatment (Wk 2)	Treatment	Feed Day	Firm	5	1,47	No	4,97	0,40
2019/05/24	CH2206	Male	Three y/o	Treatment (Wk 2)	Treatment	Feed Day	Firm and dry	4	1,70	No	4,12	0,57
2019/05/25	CH2205	Male	Three y/o	Treatment (Wk 2)	Treatment	Fast Day	Firm	5	0,97	No	4,97	-0,03
2019/05/25	CH2206	Male	Three y/o	Treatment (Wk 2)	Treatment	Fast Day	Soft with shape	3	1,35	No	3,11	0,31
2019/05/27	CH2206	Male	Three y/o	Treatment (Wk 2)	Treatment	Feed Day	Soft with shape	3	3,35	No	3,11	1,47
2019/05/28	CH2205	Male	Three y/o	Treatment (Wk 2)	Treatment	Feed Day	Soft with shape	3	1,41	Yes	3,11	0,35
2019/05/29	CH2205	Male	Three y/o	Treatment (Wk 3)	Treatment	Feed Day	Soft with shape	3	1,18	No	3,11	0,17
2019/05/30	CH2206	Male	Three y/o	Treatment (Wk 3)	Treatment	Feed Day	Soft with shape	3	0,62	Yes	3,11	-0,43
2019/05/31	CH2205	Male	Three y/o	Treatment (Wk 3)	Treatment	Feed Day	Soft with shape	3	1,40	No	3,11	0,35
2019/05/31	CH2206	Male	Three y/o	Treatment (Wk 3)	Treatment	Feed Day	Soft with shape	3	1,32	Yes	3,11	0,29

2019/06/01	CH2205	Male	Three y/o	Treatment (Wk 3)	Treatment	Feed Day	Soft with shape	3	0,80	Yes	3,11	-0,22
2019/06/01	CH2206	Male	Three y/o	Treatment (Wk 3)	Treatment	Feed Day	Soft with shape	3	1,04	Yes	3,11	0,04
2019/06/02	CH2205	Male	Three y/o	Treatment (Wk 3)	Treatment	Fast Day	Soft with shape	3	0,74	No	3,11	-0,28
2019/06/02	CH2206	Male	Three y/o	Treatment (Wk 3)	Treatment	Fast Day	Firm	5	0,93	No	4,97	-0,07
2019/06/03	CH2206	Male	Three y/o	Treatment (Wk 3)	Treatment	Feed Day	Soft with shape	3	1,08	No	3,11	0,08
2019/06/04	CH2205	Male	Three y/o	Treatment (Wk 3)	Treatment	Feed Day	Soft without shape	2	1,65	Yes	1,84	0,54
2019/06/04	CH2206	Male	Three y/o	Treatment (Wk 3)	Treatment	Feed Day	Soft with shape	3	1,40	No	3,11	0,35
2019/06/06	CH2205	Male	Three y/o	Control	Control	Feed Day	Soft without shape	2	1,54	No	1,84	0,45
2019/06/06	CH2206	Male	Three y/o	Control	Control	Feed Day	Soft with shape	3	0,74	Yes	3,11	-0,28
2019/06/06	CH2207	Female	Three y/o	Control	Control	Feed Day	Firm	5		No	4,97	
2019/06/07	CH2205	Male	Three y/o	Control	Control	Feed Day	Soft without shape	2	0,58	Yes	1,84	-0,49
2019/06/07	CH2206	Male	Three y/o	Control	Control	Feed Day	Firm	5	0,90	No	4,97	-0,10
2019/06/07	CH2207	Female	Three y/o	Control	Control	Feed Day	Soft with shape	3		No	3,11	
2019/06/08	CH2205	Male	Three y/o	Control	Control	Feed Day	Firm	5	0,53	No	4,97	-0,56
2019/06/08	CH2206	Male	Three y/o	Control	Control	Feed Day	Soft with shape	3	0,53	Yes	3,11	-0,56
2019/06/08	CH2207	Female	Three y/o	Control	Control	Feed Day	Soft with shape	3		No	3,11	
2019/06/09	CH2205	Male	Three y/o	Control	Control	Feed Day	Soft with shape	3	0,93	No	3,11	-0,07
2019/06/09	CH2207	Female	Three y/o	Control	Control	Feed Day	Soft with shape	3		No	3,11	
2019/06/10	CH2206	Male	Three y/o	Control	Control	Fast Day	Soft without shape	2	0,64	No	1,84	-0,40
2019/06/10	CH2207	Female	Three y/o	Control	Control	Fast Day	Soft with shape	3		Yes	3,11	
2019/06/11	CH2206	Male	Three y/o	Control	Control	Feed Day	Firm	5	0,55	No	4,97	-0,53
2019/06/11	CH2207	Female	Three y/o	Control	Control	Feed Day	Soft with shape	3		No	3,11	
2019/06/12	CH2205	Male	Three y/o	Control	Control	Feed Day	Soft with shape	3	1,00	No	3,11	0,00
2019/06/12	CH2206	Male	Three y/o	Control	Control	Feed Day	Soft with shape	3	1,60	No	3,11	0,50
2019/06/12	CH2207	Female	Three y/o	Control	Control	Feed Day	Firm and dry	4		No	4,12	
2019/06/13	CH2205	Male	Three y/o	Control	Control	Feed Day	Soft with shape	3	1,04	Yes	3,11	0,04
2019/06/13	CH2207	Female	Three y/o	Control	Control	Feed Day	Soft with shape	3		No	3,11	
2019/06/14	CH2205	Male	Three y/o	Control	Control	Feed Day	Soft with shape	3	1,29	No	3,11	0,26
2019/06/14	CH2206	Male	Three y/o	Control	Control	Feed Day	Soft with shape	3	1,11	No	3,11	0,10
2019/06/14	CH2207	Female	Three y/o	Control	Control	Feed Day	Soft without shape	2		No	1,84	
2019/06/15	CH2205	Male	Three y/o	Control	Control	Feed Day	Soft with shape	3	1,34	No	3,11	0,30
2019/06/15	CH2207	Female	Three y/o	Control	Control	Feed Day	Soft with shape	3		No	3,11	
2019/06/16	CH2205	Male	Three y/o	Control	Control	Feed Day	Soft with shape	3	2,04	No	3,11	0,79
2019/06/16	CH2206	Male	Three y/o	Control	Control	Feed Day	Soft with shape	3	1,64	No	3,11	0,53
2019/06/16	CH2207	Female	Three y/o	Control	Control	Feed Day	Soft with shape	3		No	3,11	
2019/06/17	CH2205	Male	Three y/o	Control	Control	Fast Day	Soft without shape	2	1,42	No	1,84	0,37
2019/06/17	CH2206	Male	Three y/o	Control	Control	Fast Day	Firm	5	0,95	No	4,97	-0,05
2019/06/17	CH2207	Female	Three y/o	Control	Control	Fast Day	Firm and dry	4		No	4,12	
2019/06/18	CH2205	Male	Three y/o	Control	Control	Feed Day	Soft with shape	3	0,63	Yes	3,11	-0,42
2019/06/18	CH2206	Male	Three y/o	Control	Control	Feed Day	Firm and dry	4	0,62	No	4,12	-0,43
2019/06/18	CH2207	Female	Three y/o	Control	Control	Feed Day	Soft with shape	3		No	3,11	
2019/06/19	CH2205	Male	Three y/o	Control	Control	Feed Day	Soft with shape	3	0,68	Yes	3,11	-0,36
2019/06/19	CH2206	Male	Three y/o	Control	Control	Feed Day	Soft with shape	3	0,69	No	3,11	-0,34
2019/06/19	CH2207	Female	Three y/o	Control	Control	Feed Day	Firm	5		No	4,97	
2019/06/20	CH2205	Male	Three y/o	Control	Control	Feed Day	Soft with shape	3	1,02	No	3,11	0,02
2019/06/20	CH2206	Male	Three y/o	Control	Control	Feed Day	Firm and dry	4	0,67	Yes	4,12	-0,36
2019/06/20	CH2207	Female	Three y/o	Control	Control	Feed Day	Soft without shape	2		No	1,84	
2019/06/21	CH2205	Male	Three y/o	Control	Control	Feed Day	Soft without shape	2	0,81	No	1,84	-0,19
2019/06/21	CH2207	Female	Three y/o	Control	Control	Feed Day	Firm and dry	4		No	4,12	

2019/06/22	CH2205	Male	Three y/o	Control	Control	Feed Day	Firm	5	0,96	No	4,97	-0,04
2019/06/22	CH2207	Female	Three y/o	Control	Control	Feed Day	Soft with shape	3		No	3,11	
2019/06/23	CH2205	Male	Three y/o	Control	Control	Feed Day	Firm and dry	4	0,42	No	4,12	-0,74
2019/06/23	CH2206	Male	Three y/o	Control	Control	Feed Day	Soft without shape	2	1,50	No	1,84	0,42
2019/06/23	CH2207	Female	Three y/o	Control	Control	Feed Day	Soft with shape	3		No	3,11	
2019/06/24	CH2205	Male	Three y/o	Control	Control	Fast Day	Soft without shape	2	0,89	Yes	1,84	-0,11
2019/06/24	CH2206	Male	Three y/o	Control	Control	Fast Day	Soft without shape	2	0,61	Yes	1,84	-0,44
2019/06/24	CH2207	Female	Three y/o	Control	Control	Fast Day	Soft without shape	2		Yes	1,84	
2019/06/25	CH2207	Female	Three y/o	Control	Control	Feed Day	Soft with shape	3		No	3,11	
2019/06/26	CH2205	Male	Three y/o	Control	Control	Feed Day	Soft with shape	3	1,11	No	3,11	0,10
2019/06/26	CH2206	Male	Three y/o	Control	Control	Feed Day	Soft with shape	3	1,51	No	3,11	0,43
2019/07/09	CH2271	Male	Two y/o	Treatment (Wk 1)	Treatment	Feed Day	Firm	5	0,14	Yes	4,97	-1,41
2019/07/11	CH2271	Male	Two y/o	Treatment (Wk 1)	Treatment	Feed Day	Firm	5	1,21	No	4,97	0,19
2019/07/12	CH2271	Male	Two y/o	Treatment (Wk 1)	Treatment	Feed Day	Soft with shape	3	0,90	No	3,11	-0,10
2019/07/13	CH2271	Male	Two y/o	Treatment (Wk 1)	Treatment	Feed Day	Soft with shape	3	1,33	No	3,11	0,29
2019/07/14	CH2271	Male	Two y/o	Treatment (Wk 1)	Treatment	Fast Day	Soft with shape	3	1,23	No	3,11	0,21
2019/07/15	CH2271	Male	Two y/o	Treatment (Wk 1)	Treatment	Feed Day	Firm	5	1,56	No	4,97	0,47
2019/07/16	CH2271	Male	Two y/o	Treatment (Wk 2)	Treatment	Feed Day	Soft with shape	3	1,49	No	3,11	0,42
2019/07/17	CH2271	Male	Two y/o	Treatment (Wk 2)	Treatment	Fast Day	Firm	5	1,16	No	4,97	0,15
2019/07/18	CH2271	Male	Two y/o	Treatment (Wk 2)	Treatment	Feed Day	Soft with shape	3	1,49	No	3,11	0,42
2019/07/19	CH2271	Male	Two y/o	Treatment (Wk 2)	Treatment	Feed Day	Soft with shape	3	1,07	Yes	3,11	0,06
2019/07/20	CH2271	Male	Two y/o	Treatment (Wk 2)	Treatment	Feed Day	Soft with shape	3	1,40	No	3,11	0,35
2019/07/21	CH2271	Male	Two y/o	Treatment (Wk 2)	Treatment	Fast Day	Soft with shape	3	0,57	No	3,11	-0,50
2019/07/22	CH2271	Male	Two y/o	Treatment (Wk 2)	Treatment	Feed Day	Soft with shape	3	0,89	No	3,11	-0,11
2019/07/23	CH2271	Male	Two y/o	Treatment (Wk 3)	Treatment	Feed Day	Soft with shape	3	1,22	No	3,11	0,20
2019/07/24	CH2271	Male	Two y/o	Treatment (Wk 3)	Treatment	Fast Day	Firm	5	1,17	No	4,97	0,15
2019/07/25	CH2271	Male	Two y/o	Treatment (Wk 3)	Treatment	Feed Day	Firm	5	1,18	No	4,97	0,17
2019/07/26	CH2271	Male	Two y/o	Treatment (Wk 3)	Treatment	Feed Day	Soft with shape	3	1,47	No	3,11	0,40
2019/07/27	CH2271	Male	Two y/o	Treatment (Wk 3)	Treatment	Feed Day	Soft with shape	3	0,58	No	3,11	-0,49
2019/07/28	CH2271	Male	Two y/o	Treatment (Wk 3)	Treatment	Fast Day	Firm	5	1,43	No	4,97	0,37
2019/07/31	CH2277	Female	Two y/o	Treatment (Wk 1)	Treatment	Fast Day	Soft with shape	3	0,69	Yes	3,11	-0,34
2019/08/01	CH2276	Female	Two y/o	Treatment (Wk 1)	Treatment	Feed Day	Soft with shape	3	1,66	No	3,11	0,54
2019/08/02	CH2276	Female	Two y/o	Treatment (Wk 1)	Treatment	Feed Day	Soft with shape	3	1,62	No	3,11	0,51
2019/08/02	CH2277	Female	Two y/o	Treatment (Wk 1)	Treatment	Feed Day	Soft with shape	3	0,92	No	3,11	-0,08
2019/08/03	CH2276	Female	Two y/o	Treatment (Wk 1)	Treatment	Feed Day	Soft without shape	2	0,47	No	1,84	-0,65
2019/08/04	CH2276	Female	Two y/o	Treatment (Wk 1)	Treatment	Fast Day	Soft with shape	3	0,65	No	3,11	-0,40
2019/08/04	CH2277	Female	Two y/o	Treatment (Wk 1)	Treatment	Fast Day	Firm	5	0,45	No	4,97	-0,68
2019/08/05	CH2276	Female	Two y/o	Treatment (Wk 1)	Treatment	Feed Day	Firm and dry	4	1,08	No	4,12	0,08
2019/08/05	CH2277	Female	Two y/o	Treatment (Wk 1)	Treatment	Feed Day	Firm and dry	4	0,96	No	4,12	-0,04
2019/08/06	CH2276	Female	Two y/o	Treatment (Wk 1)	Treatment	Feed Day	Soft with shape	3	2,54	No	3,11	1,07
2019/08/06	CH2277	Female	Two y/o	Treatment (Wk 1)	Treatment	Feed Day	Firm	5	1,10	No	4,97	0,10
2019/08/07	CH2276	Female	Two y/o	Treatment (Wk 2)	Treatment	Fast Day	Soft without shape	2	1,54	No	1,84	0,46
2019/08/07	CH2277	Female	Two y/o	Treatment (Wk 2)	Treatment	Fast Day	Soft with shape	3	1,46	No	3,11	0,40
2019/08/08	CH2276	Female	Two y/o	Treatment (Wk 2)	Treatment	Feed Day	Firm	5	1,61	No	4,97	0,51
2019/08/08	CH2277	Female	Two y/o	Treatment (Wk 2)	Treatment	Feed Day	Firm	5	1,25	No	4,97	0,22
2019/08/09	CH2277	Female	Two y/o	Treatment (Wk 2)	Treatment	Feed Day	Soft with shape	3	1,60	No	3,11	0,50
2019/08/10	CH2276	Female	Two y/o	Treatment (Wk 2)	Treatment	Feed Day	Firm	5	1,41	No	4,97	0,36
2019/08/10	CH2277	Female	Two y/o	Treatment (Wk 2)	Treatment	Feed Day	Firm	5	1,06	No	4,97	0,06
2019/08/11	CH2276	Female	Two y/o	Treatment (Wk 2)	Treatment	Fast Day	Soft with shape	3	0,43	Yes	3,11	-0,72

2019/08/11	CH2277	Female	Two y/o	Treatment (Wk 2)	Treatment	Fast Day	Soft with shape	3	0,85	Yes	3,11	-0,16
2019/08/12	CH2276	Female	Two y/o	Treatment (Wk 2)	Treatment	Feed Day	Firm and dry	4	0,43	No	4,12	-0,71
2019/08/12	CH2277	Female	Two y/o	Treatment (Wk 2)	Treatment	Feed Day	Firm and dry	4	0,54	No	4,12	-0,55
2019/08/13	CH2276	Female	Two y/o	Treatment (Wk 2)	Treatment	Feed Day	Firm and dry	4	1,09	No	4,12	0,09
2019/08/13	CH2277	Female	Two y/o	Treatment (Wk 2)	Treatment	Feed Day	Soft with shape	3	0,79	No	3,11	-0,23
2019/08/14	CH2276	Female	Two y/o	Treatment (Wk 3)	Treatment	Fast Day	Soft with shape	3	0,83	Yes	3,11	-0,18
2019/08/14	CH2277	Female	Two y/o	Treatment (Wk 3)	Treatment	Fast Day	Firm	5	0,59	No	4,97	-0,47
2019/08/15	CH2276	Female	Two y/o	Treatment (Wk 3)	Treatment	Feed Day	Firm	5	1,26	No	4,97	0,24
2019/08/15	CH2277	Female	Two y/o	Treatment (Wk 3)	Treatment	Feed Day	Firm	5	0,67	No	4,97	-0,36
2019/08/16	CH2276	Female	Two y/o	Treatment (Wk 3)	Treatment	Feed Day	Firm	5	1,08	Yes	4,97	0,07
2019/08/17	CH2276	Female	Two y/o	Treatment (Wk 3)	Treatment	Feed Day	Firm	5	1,03	No	4,97	0,03
2019/08/17	CH2277	Female	Two y/o	Treatment (Wk 3)	Treatment	Feed Day	Firm and dry	4	0,69	No	4,12	-0,34
2019/08/18	CH2277	Female	Two y/o	Treatment (Wk 3)	Treatment	Fast Day	Firm	5	0,72	No	4,97	-0,31
2019/08/19	CH2277	Female	Two y/o	Treatment (Wk 3)	Treatment	Feed Day	Firm and dry	4	0,72	No	4,12	-0,31
2019/08/20	CH2276	Female	Two y/o	Treatment (Wk 3)	Treatment	Feed Day	Soft with shape	3	0,58	No	3,11	-0,48
2019/08/20	CH2277	Female	Two y/o	Treatment (Wk 3)	Treatment	Feed Day	Soft with shape	3	0,94	No	3,11	-0,06
2019/08/22	CH2271	Male	Two y/o	Control	Control	Feed Day	Firm and dry	4	0,98	No	4,12	-0,02
2019/08/22	CH2276	Female	Two y/o	Control	Control	Fast Day	Soft with shape	3	0,80	No	3,11	-0,21
2019/08/22	CH2277	Female	Two y/o	Control	Control	Fast Day	Soft with shape	3	0,83	No	3,11	-0,18
2019/08/23	CH2271	Male	Two y/o	Control	Control	Feed Day	Firm and dry	4	0,98	No	4,12	-0,02
2019/08/24	CH2276	Female	Two y/o	Control	Control	Feed Day	Firm	5	1,41	No	4,97	0,36
2019/08/24	CH2277	Female	Two y/o	Control	Control	Feed Day	Firm	5	0,62	No	4,97	-0,43
2019/08/25	CH2271	Male	Two y/o	Control	Control	Feed Day	Firm and dry	4	0,74	No	4,12	-0,27
2019/08/25	CH2277	Female	Two y/o	Control	Control	Feed Day	Firm	5	0,85	No	4,97	-0,15
2019/08/26	CH2271	Male	Two y/o	Control	Control	Feed Day	Firm	5	0,65	Yes	4,97	-0,39
2019/08/26	CH2276	Female	Two y/o	Control	Control	Feed Day	Firm	5	1,64	No	4,97	0,53
2019/08/26	CH2277	Female	Two y/o	Control	Control	Feed Day	Soft with shape	3	1,25	No	3,11	0,23
2019/08/27	CH2271	Male	Two y/o	Control	Control	Feed Day	Soft with shape	3	0,96	Yes	3,11	-0,04
2019/08/27	CH2276	Female	Two y/o	Control	Control	Feed Day	Soft with shape	3	0,86	No	3,11	-0,14
2019/08/28	CH2271	Male	Two y/o	Control	Control	Feed Day	Soft with shape	3	1,33	No	3,11	0,29
2019/08/28	CH2276	Female	Two y/o	Control	Control	Feed Day	Firm	5	1,30	Yes	4,97	0,27
2019/08/28	CH2277	Female	Two y/o	Control	Control	Feed Day	Firm	5	1,47	No	4,97	0,40
2019/08/29	CH2271	Male	Two y/o	Control	Control	Feed Day	Firm and dry	4	1,01	No	4,12	0,01
2019/08/29	CH2277	Female	Two y/o	Control	Control	Feed Day	Firm	5	1,09	No	4,97	0,09
2019/08/30	CH2271	Male	Two y/o	Control	Control	Feed Day	Firm and dry	4	0,92	No	4,12	-0,08
2019/08/30	CH2276	Female	Two y/o	Control	Control	Feed Day	Firm	5	1,39	Yes	4,97	0,34
2019/08/30	CH2277	Female	Two y/o	Control	Control	Feed Day	Firm and dry	4	0,59	No	4,12	-0,47
2019/08/31	CH2271	Male	Two y/o	Control	Control	Feed Day	Firm	5	0,96	Yes	4,97	-0,04
2019/08/31	CH2276	Female	Two y/o	Control	Control	Feed Day	Firm and dry	4	0,80	No	4,12	-0,22
2019/08/31	CH2277	Female	Two y/o	Control	Control	Feed Day	Firm	5	0,63	No	4,97	-0,42
2019/09/01	CH2271	Male	Two y/o	Control	Control	Feed Day	Soft with shape	3	1,12	Yes	3,11	0,11
2019/09/01	CH2276	Female	Two y/o	Control	Control	Feed Day	Firm and dry	4	0,68	No	4,12	-0,35
2019/09/01	CH2277	Female	Two y/o	Control	Control	Feed Day	Firm	5	0,74	No	4,97	-0,28
2019/09/02	CH2271	Male	Two y/o	Control	Control	Feed Day	Soft with shape	3	0,52	No	3,11	-0,57
2019/09/02	CH2276	Female	Two y/o	Control	Control	Feed Day	Soft with shape	3	0,90	No	3,11	-0,10
2019/09/02	CH2277	Female	Two y/o	Control	Control	Feed Day	Soft with shape	3	0,52	No	3,11	-0,57
2019/09/03	CH2271	Male	Two y/o	Control	Control	Feed Day	Firm	5	0,64	No	4,97	-0,41
2019/09/03	CH2277	Female	Two y/o	Control	Control	Feed Day	Soft with shape	3	0,69	No	3,11	-0,34
2019/09/04	CH2271	Male	Two y/o	Control	Control	Feed Day	Soft with shape	3	1,15	No	3,11	0,14

2019/09/04	CH2276	Female	Two y/o	Control	Control	Feed Day	Soft with shape	3	1,20	No	3,11	0,18
2019/09/04	CH2277	Female	Two y/o	Control	Control	Feed Day	Soft with shape	3	0,66	Yes	3,11	-0,38
2019/09/05	CH2271	Male	Two y/o	Control	Control	Feed Day	Firm and dry	4	0,59	No	4,12	-0,47
2019/09/05	CH2276	Female	Two y/o	Control	Control	Feed Day	Firm	5	1,11	No	4,97	0,10
2019/09/05	CH2277	Female	Two y/o	Control	Control	Feed Day	Firm	5	0,85	No	4,97	-0,15
2019/09/06	CH2271	Male	Two y/o	Control	Control	Feed Day	Soft with shape	3	0,87	Yes	3,11	-0,13
2019/09/06	CH2276	Female	Two y/o	Control	Control	Feed Day	Firm	5	1,01	Yes	4,97	0,01
2019/09/06	CH2277	Female	Two y/o	Control	Control	Feed Day	Soft with shape	3	0,60	No	3,11	-0,46
2019/09/07	CH2271	Male	Two y/o	Control	Control	Feed Day	Firm and dry	4	0,88	No	4,12	-0,13
2019/09/07	CH2276	Female	Two y/o	Control	Control	Feed Day	Soft with shape	3	1,08	Yes	3,11	0,08
2019/09/07	CH2277	Female	Two y/o	Control	Control	Feed Day	Firm	5	0,75	No	4,97	-0,27
2019/09/08	CH2276	Female	Two y/o	Control	Control	Feed Day	Firm	5	0,87	No	4,97	-0,13
2019/09/08	CH2277	Female	Two y/o	Control	Control	Feed Day	Firm and dry	4	0,79	No	4,12	-0,22
2019/09/09	CH2271	Male	Two y/o	Control	Control	Feed Day	Firm	5	0,94	No	4,97	-0,06
2019/09/09	CH2276	Female	Two y/o	Control	Control	Feed Day	Firm and dry	4	0,85	No	4,12	-0,16
2019/09/09	CH2277	Female	Two y/o	Control	Control	Feed Day	Firm and dry	4	0,73	No	4,12	-0,30
2019/09/10	CH2271	Male	Two y/o	Control	Control	Feed Day	Soft with shape	3	1,39	No	3,11	0,34
2019/09/10	CH2276	Female	Two y/o	Control	Control	Feed Day	Soft with shape	3	0,78	No	3,11	-0,23
2019/09/10	CH2277	Female	Two y/o	Control	Control	Feed Day	Soft with shape	3	0,90	No	3,11	-0,10
2019/09/11	CH2271	Male	Two y/o	Control	Control	Feed Day	Soft with shape	3	1,47	No	3,11	0,41
2019/09/11	CH2276	Female	Two y/o	Control	Control	Feed Day	Soft with shape	3	1,05	Yes	3,11	0,05
2019/09/11	CH2277	Female	Two y/o	Control	Control	Feed Day	Soft with shape	3	1,32	No	3,11	0,28

#	Date	Cheetah ID	Sex	Age Group	Week	Study Period	Feed/Fast Day	Feeding Time	Minute	Hour	Part of the Day	Body Temperature (°C)	Heart Rate (bpm)	Locomotor Activity (ODBA)	Locomotor Activity (ODBA) - Outliers	Body Temperature (ºC) X	Heart Rate (bpm) X	Locomotor Activity (ODBA) - Outliers X
1	2019/05/15	CH2205	Male	Three y/o	Treatment (Wk 1)	Treatment	Fast Day	No	00:00	00:00 - 00:59	Early Morning	37,6	55	7,2	7,2	18,75	547,52	65,66
	2019/05/15	CH2206	Male	Three y/o	Treatment (Wk 1)	Treatment	Fast Day	No	00:00	00:00 - 00:59	Early Morning	37,6	87	33,2	33,2	18,75	583,64	117,68
	2019/05/15	CH2205	Male	Three y/o	Treatment (Wk 1)	Treatment	Fast Day	No	00:05	00:00 - 00:59	Early Morning	37,6	41	9,4	9,4	18,75	522,29	74,66
	2019/05/15	CH2206	Male	Three y/o	Treatment (Wk 1)	Treatment	Fast Day	No	00:05	00:00 - 00:59	Early Morning	37,5	57	28,2	28,2	18,65	550,47	112,07
	2019/05/15	CH2205	Male	Three y/o	Treatment (Wk 1)	Treatment	Fast Day	No	00:10	00:00 - 00:59	Early Morning	37,6	84	7,4	7,4	18,75	581,01	66,59
	2019/05/15	CH2206	Male	Three y/o	Treatment (Wk 1)	Treatment	Fast Day	No	00:10	00:00 - 00:59	Early Morning	37,5	75	15,2	15,2	18,65	572,36	90,96
	2019/05/15	CH2205	Male	Three y/o	Treatment (Wk 1)	Treatment	Fast Day	No	00:15	00:00 - 00:59	Early Morning	37,7	61	6,2	6,2	18,85	556,01	60,63
	2019/05/15	CH2206	Male	Three y/o	Treatment (Wk 1)	Treatment	Fast Day	No	00:15	00:00 - 00:59	Early Morning	37,7	47	19,8	19,8	18,85	534,23	99,97
	2019/05/15	CH2205	Male	Three y/o	Treatment (Wk 1)	Treatment	Fast Day	No	00:20	00:00 - 00:59	Early Morning	37,7	62	11,4	11,4	18,85	557,33	81,19
	2019/05/15	CH2206	Male	Three y/o	Treatment (Wk 1)	Treatment	Fast Day	No	00:20	00:00 - 00:59	Early Morning	37,7	95	21,4	21,4	18,85	590,14	102,63
	2019/05/15	CH2205	Male	Three y/o	Treatment (Wk 1)	Treatment	Fast Day	No	00:25	00:00 - 00:59	Early Morning	37,7	71	9,6	9,6	18,85	568,10	75,37
	2019/05/15	CH2206	Male	Three y/o	Treatment (Wk 1)	Treatment	Fast Day	No	00:25	00:00 - 00:59	Early Morning	37,7	53	19,2	19,2	18,85	544,43	98,92
	2019/05/15	CH2205	Male	Three y/o	Treatment (Wk 1)	Treatment	Fast Day	No	00:30	00:00 - 00:59	Early Morning	37,7	50	8,0	8,0	18,85	539,52	69,22
	2019/05/15	CH2206	Male	Three y/o	Treatment (Wk 1)	Treatment	Fast Day	No	00:30	00:00 - 00:59	Early Morning	37,7	96	22,8	22,8	18,85	590,91	104,79
	2019/05/15	CH2205	Male	Three y/o	Treatment (Wk 1)	Treatment	Fast Day	No	00:35	00:00 - 00:59	Early Morning	37,7	45	9,4	9,4	18,85	530,47	74,66
	2019/05/15	CH2206	Male	Three y/o	Treatment (Wk 1)	Treatment	Fast Day	No	00:35	00:00 - 00:59	Early Morning	37,7	52	25,2	25,2	18,85	542,83	108,22
	2019/05/15	CH2205	Male	Three y/o	Treatment (Wk 1)	Treatment	Fast Day	No	00:40	00:00 - 00:59	Early Morning	37,7	64	8,8	8,8	18,85	559,88	72,43
	2019/05/15	CH2206	Male	Three y/o	Treatment (Wk 1)	Treatment	Fast Day	No	00:40	00:00 - 00:59	Early Morning	37,5	80	29,4	29,4	18,65	577,31	113,50
	2019/05/15	CH2205	Male	Three y/o	Treatment (Wk 1)	Treatment	Fast Day	No	00:45	00:00 - 00:59	Early Morning	37,7	56	20,8	20,8	18,85	549,01	101,65
	2019/05/15	CH2206	Male	Three y/o	Treatment (Wk 1)	Treatment	Fast Day	No	00:45	00:00 - 00:59	Early Morning	37,5	53	29,2	29,2	18,65	544,43	113,27
	2019/05/15	CH2205	Male	Three y/o	Treatment (Wk 1)	Treatment	Fast Day	No	00:50	00:00 - 00:59	Early Morning	37,7	57	12,2	12,2	18,85	550,47	83,49
	2019/05/15	CH2206	Male	Three y/o	Treatment (Wk 1)	Treatment	Fast Day	No	00:50	00:00 - 00:59	Early Morning	37,7	84	23,0	23,0	18,85	581,01	105,09
	2019/05/15	CH2205	Male	Three y/o	Treatment (Wk 1)	Treatment	Fast Day	No	00:55	00:00 - 00:59	Early Morning	37,6	92	7,6	7,6	18,75	587,78	67,49
	2019/05/15	CH2206	Male	Three y/o	Treatment (Wk 1)	Treatment	Fast Day	No	00:55	00:00 - 00:59	Early Morning	37,7	86	23,0	23,0	18,85	582,77	105,09
	2019/05/15	CH2205	Male	Three y/o	Treatment (Wk 1)	Treatment	Fast Day	No	01:00	01:00 - 01:59	Early Morning	37,6	81	27,8	27,8	18,75	578,26	111,58
	2019/05/15	CH2206	Male	Three y/o	Treatment (Wk 1)	Treatment	Fast Day	No	01:00	01:00 - 01:59	Early Morning	37,7	94	28,2	28,2	18,85	589,37	112,07
	2019/05/15	CH2205	Male	Three y/o	Treatment (Wk 1)	Treatment	Fast Day	No	01:05	01:00 - 01:59	Early Morning	37,6	61	27,4	27,4	18,75	556,01	111,09
	2019/05/15	CH2206	Male	Three y/o	Treatment (Wk 1)	Treatment	Fast Day	No	01:05	01:00 - 01:59	Early Morning	37,7	89	39,4	39,4	18,85	585,33	123,57
	2019/05/15	CH2205	Male	Three y/o	Treatment (Wk 1)	Treatment	Fast Day	No	01:10	01:00 - 01:59	Early Morning	37,6	64	11,0	11,0	18,75	559,88	79,98
	2019/05/15	CH2206	Male	Three y/o	Treatment (Wk 1)	Treatment	Fast Day	No	01:10	01:00 - 01:59	Early Morning	37,5	92	60,4	60,4	18,65	587,78	138,32
	2019/05/15	CH2205	Male	Three y/o	Treatment (Wk 1)	Treatment	Fast Day	No	01:15	01:00 - 01:59	Early Morning	37,5	53	7,0	7,0	18,65	544,43	64,72
	2019/05/15	CH2206	Male	Three y/o	Treatment (Wk 1)	Treatment	Fast Day	No	01:15	01:00 - 01:59	Early Morning	37,4	56	57,2	57,2	18,55	549,01	136,44
	2019/05/15	CH2205	Male	Three y/o	Treatment (Wk 1)	Treatment	Fast Day	No	01:20	01:00 - 01:59	Early Morning	37,5	45	6,6	6,6	18,65	530,47	62,73
	2019/05/15	CH2206	Male	Three y/o	Treatment (Wk 1)	Treatment	Fast Day	No	01:20	01:00 - 01:59	Early Morning	37,5	80	55,2	55,2	18,65	577,31	135,21
	2019/05/15	CH2205	Male	Three y/o	Treatment (Wk 1)	Treatment	Fast Day	No	01:25	01:00 - 01:59	Early Morning	37,5	55	21,4	21,4	18,65	547,52	102,63
	2019/05/15	CH2206	Male	Three y/o	Treatment (Wk 1)	Treatment	Fast Day	No	01:25	01:00 - 01:59	Early Morning	37,5	61	56,8	56,8	18,65	556,01	136,20
	2019/05/15	CH2205	Male	Three y/o	Treatment (Wk 1)	Treatment	Fast Day	No	01:30	01:00 - 01:59	Early Morning	37,5	58	17,2	17,2	18,65	551,90	95,17
	2019/05/15	CH2206	Male	Three y/o	Treatment (Wk 1)	Treatment	Fast Day	No	01:30	01:00 - 01:59	Early Morning	37,5	87	56,4	56,4	18,65	583,64	135,95
	2019/05/15	CH2205	Male	Three y/o	Treatment (Wk 1)	Treatment	Fast Day	No	01:35	01:00 - 01:59	Early Morning	37,4	32	14,0	14,0	18,55	499,63	88,17
	2019/05/15	CH2206	Male	Three y/o	Treatment (Wk 1)	Treatment	Fast Day	No	01:35	01:00 - 01:59	Early Morning	37,5	63	39,4	39,4	18,65	558,62	123,57
	2019/05/15	CH2205	Male	Three y/o	Treatment (Wk 1)	Treatment	Fast Day	No	01:40	01:00 - 01:59	Early Morning	37,3	44	15,4	15,4	18,46	528,51	91,41
	2019/05/15	CH2206	Male	Three y/o	Treatment (Wk 1)	Treatment	Fast Day	No	01:40	01:00 - 01:59	Early Morning	37,5	54	16,6	16,6	18,65	545,99	93,96
	2019/05/15	CH2205	Male	Three y/o	Treatment (Wk 1)	Treatment	Fast Day	No	01:45	01:00 - 01:59	Early Morning	37,3	47	30,6	30,6	18,46	534,23	114,87
	2019/05/15	CH2206	Male	Three y/o	Treatment (Wk 1)	Treatment	Fast Day	No	01:45	01:00 - 01:59	Early Morning	37,6	73	21,2	21,2	18,75	570,27	102,31
	2019/05/15	CH2205	Male	Three y/o	Treatment (Wk 1)	Treatment	Fast Day	No	01:50	01:00 - 01:59	Early Morning	37,2	50	17,2	17,2	18,36	539,52	95,17
	2019/05/15	CH2206	Male	Three y/o	Treatment (Wk 1)	Treatment	Fast Day	No	01:50	01:00 - 01:59	Early Morning	37,6	65	12,6	12,6	18,75	561,12	84,59
	2019/05/15	CH2205	Male	Three y/o	Treatment (Wk 1)	Treatment	Fast Day	No	01:55	01:00 - 01:59	Early Morning	37,3	86	18,0	18,0	18,46	582,77	96,72
	2019/05/15	CH2206	Male	Three y/o	Treatment (Wk 1)	Treatment	Fast Day	No	01:55	01:00 - 01:59	Early Morning	37,5	68	36,4	36,4	18,65	564,71	120,84
	2019/05/15	CH2205	Male	Three y/o	Treatment (Wk 1)	Treatment	Fast Day	No	02:00	02:00 - 02:59	Early Morning	37,4	105	12,2	12,2	18,55	597,38	83,49
	2019/05/15	CH2206	Male	Three y/o	Treatment (Wk 1)	Treatment	Fast Day	No	02:00	02:00 - 02:59	Early Morning	37,7	131	37,6	37,6	18,85	612,80	121,96
	2019/05/15	CH2205	Male	Three y/o	Treatment (Wk 1)	Treatment	Fast Day	No	02:05	02:00 - 02:59	Early Morning	37,4	46	10,6	10,6	18,55	532,38	78,73
	2019/05/15	CH2206	Male	Three y/o	Treatment (Wk 1)	Treatment	Fast Day	No	02:05	02:00 - 02:59	Early Morning	37,5	110	27,2	27,2	18,65	600,69	110,83
	2019/05/15	CH2205	Male	Three y/o	Treatment (Wk 1)	Treatment	Fast Day	No	02:10	02:00 - 02:59	Early Morning	37,2	91	27,6	27,6	18,36	586,98	111,33
	2019/05/15	CH2206	Male	Three y/o	Treatment (Wk 1)	Treatment	Fast Day	No	02:10	02:00 - 02:59	Early Morning	37,5	109	53,6	53,6	18,65	600,04	134,19
	2019/05/15	CH2205	Male	Three y/o	Treatment (Wk 1)	Treatment	Fast Day	No	02:15	02:00 - 02:59	Early Morning	37,3	49	290,2	290,2	18,46	537,80	193,25
	2019/05/15	CH2206	Male	Three y/o	Treatment (Wk 1)	Treatment	Fast Day	No	02:15	02:00 - 02:59	Early Morning	37,4	98	95,2	95,2	18,55	592,41	154,13
	2019/05/15	CH2205	Male	Three y/o	Treatment (Wk 1)	Treatment	Fast Day	No	02:20	02:00 - 02:59	Early Morning	37,3	137	49,6	49,6	18,46	615,83	131,51
	2019/05/15	CH2206	Male	Three y/o	Treatment (Wk 1)	Treatment	Fast Day	No	02:20	02:00 - 02:59	Early Morning	37,4	120	51,0	51,0	18,55	606,78	132,47
	2019/05/15	CH2205	Male	Three y/o	Treatment (Wk 1)	Treatment	Fast Day	No	02:25	02:00 - 02:59	Early Morning	37,3		95,4	95,4	18,46		154,20
	2019/05/15	CH2206	Male	Three y/o	Treatment (Wk 1)	Treatment	Fast Day	No	02:25	02:00 - 02:59	Early Morning	37,3	112	104,2	104,2	18,46	601,96	157,28
	2019/05/15	CH2205	Male	Three y/o	Treatment (Wk 1)	Treatment	Fast Day	No	02:30	02:00 - 02:59	Early Morning	37,4	107	56,6	56,6	18,55	598,73	136,07
	2019/05/15	CH2206	Male	Three y/o	Treatment (Wk 1)	Treatment	Fast Day	No	02:30	02:00 - 02:59	Early Morning	37,3	59	35,2	35,2	18,46	553,30	119,69
	2019/05/15	CH2205	Male	Three y/o	Treatment (Wk 1)	Treatment	Fast Day	No	02:35	02:00 - 02:59	Early Morning	37,5		7,6	7,6	18,65		67,49
	2019/05/15	CH2206	Male	Three y/o	Treatment (Wk 1)	Treatment	Fast Day	No	02:35	02:00 - 02:59	Early Morning	37,3	55	42,2	42,2	18,46	547,52	125,93
	2019/05/15	CH2205	Male	Three y/o	Treatment (Wk 1)	Treatment	Fast Day	No	02:40	02:00 - 02:59	Early Morning	37,3		3,6	3,6	18,46		42,42
	2019/05/15	CH2206	Male	Three y/o	Treatment (Wk 1)	Treatment	Fast Day	No	02:40	02:00 - 02:59	Early Morning	37,2	66	48,0	48,0	18,36	562,34	130,38
	2019/05/15	CH2205	Male	Three y/o	Treatment (Wk 1)	Treatment	Fast Day	No	02:45	02:00 - 02:59	Early Morning	37,3	49	4,4	4,4	18,46	537,80	49,12

2019/05/15	CH2206	Male	Three y/o	Treatment (Wk 1)	Treatment	Fast Day	No	02:45	02:00 - 02:59	Early Morning	37,1	73	42,4	42,4	18,26	570,27	126,10
2019/05/15	CH2205	Male	Three y/o	Treatment (Wk 1)	Treatment	Fast Day	No	02:50	02:00 - 02:59	Early Morning	37,3	60	1,2	1,2	18,46	554,67	5,99
2019/05/15	CH2206	Male	Three y/o	Treatment (Wk 1)	Treatment	Fast Day	No	02:50	02:00 - 02:59	Early Morning	37,1		63,2	63,2	18,26		139,89
2019/05/15	CH2205	Male	Three y/o	Treatment (Wk 1)	Treatment	Fast Day	No	02:55	02:00 - 02:59	Early Morning	37,3		12,8	12,8	18,46		85,12
2019/05/15	CH2206	Male	Three y/o	Treatment (Wk 1)	Treatment	Fast Day	No	02:55	02:00 - 02:59	Early Morning	37,1	96	45,2	45,2	18,26	590,91	128,30
2019/05/15	CH2205	Male	Three y/o	Treatment (Wk 1)	Treatment	Fast Day	No	03:00	03:00 - 03:59	Early Morning	37,3	55	13,6	13,6	18,46	547,52	87,18
2019/05/15	CH2206	Male	Three y/o	Treatment (Wk 1)	Treatment	Fast Day	No	03:00	03:00 - 03:59	Early Morning	37,0	81	51,6	51,6	18,16	578,26	132,87
2019/05/15	CH2205	Male	Three y/o	Treatment (Wk 1)	Treatment	Fast Day	No	03:05	03:00 - 03:59	Early Morning	37,3	73	10,2	10,2	18,46	570,27	77,43
2019/05/15	CH2206	Male	Three y/o	Treatment (Wk 1)	Treatment	Fast Day	No	03:05	03:00 - 03:59	Early Morning	36,8	44	44,2	44,2	17,96	528,51	127,53
2019/05/15	CH2205	Male	Three y/o	Treatment (Wk 1)	Treatment	Fast Day	No	03:10	03:00 - 03:59	Early Morning	37,4	61	8,2	8,2	18,55	556,01	70,05
2019/05/15	CH2206	Male	Three y/o	Treatment (Wk 1)	Treatment	Fast Day	No	03:10	03:00 - 03:59	Early Morning	36,6	85	45,8	45,8	17,77	581,90	128,76
2019/05/15	CH2205	Male	Three y/o	Treatment (Wk 1)	Treatment	Fast Day	No	03:15	03:00 - 03:59	Early Morning	37,4	49	5,4	5,4	18,55	537,80	55,99
2019/05/15	CH2206	Male	Three y/o	Treatment (Wk 1)	Treatment	Fast Day	No	03:15	03:00 - 03:59	Early Morning	36,7	62	48,8	48,8	17,87	557,33	130,95
2019/05/15	CH2205	Male	Three y/o	Treatment (Wk 1)	Treatment	Fast Day	No	03:20	03:00 - 03:59	Early Morning	37,4	78	13,2	13,2	18,55	575,38	86,17
2019/05/15	CH2206	Male	Three y/o	Treatment (Wk 1)	Treatment	Fast Day	No	03:20	03:00 - 03:59	Early Morning	36,8	68	54,4	54,4	17,96	564,71	134,70
2019/05/15	CH2205	Male	Three y/o	Treatment (Wk 1)	Treatment	Fast Day	No	03:25	03:00 - 03:59	Early Morning	37,4	42	16,6	16,6	18,55	524,42	93,96
2019/05/15	CH2206	Male	Three y/o	Treatment (Wk 1)	Treatment	Fast Day	No	03:25	03:00 - 03:59	Early Morning	36,6	52	55,0	55,0	17,77	542,83	135,08
2019/05/15	CH2205	Male	Three y/o	Treatment (Wk 1)	Treatment	Fast Day	No	03:30	03:00 - 03:59	Early Morning	37,3	62	12,2	12,2	18,46	557,33	83,49
2019/05/15	CH2206	Male	Three y/o	Treatment (Wk 1)	Treatment	Fast Day	No	03:30	03:00 - 03:59	Early Morning	36,5	41	54,2	54,2	17,67	522,29	134,57
2019/05/15	CH2205	Male	Three y/o	Treatment (Wk 1)	Treatment	Fast Day	No	03:35	03:00 - 03:59	Early Morning	37,2	38	13,0	13,0	18,36	515,48	85,65
2019/05/15	CH2206	Male	Three y/o	Treatment (Wk 1)	Treatment	Fast Day	No	03:35	03:00 - 03:59	Early Morning	36,9	53	55,0	55,0	18,06	544,43	135,08
2019/05/15	CH2205	Male	Three y/o	Treatment (Wk 1)	Treatment	Fast Day	No	03:40	03:00 - 03:59	Early Morning	37,2	40	12,8	12,8	18,36	520,09	85,12
2019/05/15	CH2206	Male	Three y/o	Treatment (Wk 1)	Treatment	Fast Day	No	03:40	03:00 - 03:59	Early Morning	37,0	52	53,4	53,4	18,16	542,83	134,06
2019/05/15	CH2205	Male	Three y/o	Treatment (Wk 1)	Treatment	Fast Day	No	03:45	03:00 - 03:59	Early Morning	37,2	40	18,8	18,8	18,36	520,09	98,20
2019/05/15	CH2206	Male	Three y/o	Treatment (Wk 1)	Treatment	Fast Day	No	03:45	03:00 - 03:59	Early Morning	37,0	84	58,2	58,2	18,16	581,01	137,04
2019/05/15	CH2205	Male	Three y/o	Treatment (Wk 1)	Treatment	Fast Day	No	03:50	03:00 - 03:59	Early Morning	37,1	77	16,6	16,6	18,26	574,39	93,96
2019/05/15	CH2206	Male	Three y/o	Treatment (Wk 1)	Treatment	Fast Day	No	03:50	03:00 - 03:59	Early Morning	37,0	98	55,8	55,8	18,16	592,41	135,58
2019/05/15	CH2205	Male	Three y/o	Treatment (Wk 1)	Treatment	Fast Day	No	03:55	03:00 - 03:59	Early Morning	37,1	58	21,4	21,4	18,26	551,90	102,63
2019/05/15	CH2206	Male	Three y/o	Treatment (Wk 1)	Treatment	Fast Day	No	03:55	03:00 - 03:59	Early Morning	36,9	47	60,2	60,2	18,06	534,23	138,21
2019/05/15	CH2205	Male	Three y/o	Treatment (Wk 1)	Treatment	Fast Day	No	04:00	04:00 - 04:59	Morning	37,1		22,4	22,4	18,26		104,19
2019/05/15	CH2206	Male	Three y/o	Treatment (Wk 1)	Treatment	Fast Day	No	04:00	04:00 - 04:59	Morning	36,9	48	53,2	53,2	18,06	536,04	133,93
2019/05/15	CH2205	Male	Three y/o	Treatment (Wk 1)	Treatment	Fast Day	No	04:05	04:00 - 04:59	Morning	37,1		15,6	15,6	18,26		91,85
2019/05/15	CH2206	Male	Three y/o	Treatment (Wk 1)	Treatment	Fast Day	No	04:05	04:00 - 04:59	Morning	36,9	74	60,2	60,2	18,06	571,33	138,21
2019/05/15	CH2205	Male	Three y/o	Treatment (Wk 1)	Treatment	Fast Day	No	04:10	04:00 - 04:59	Morning	37,1	46	16,4	16,4	18,26	532,38	93,55
2019/05/15	CH2206	Male	Three y/o	Treatment (Wk 1)	Treatment	Fast Day	No	04:10	04:00 - 04:59	Morning	36,9	41	46,6	46,6	18,06	522,29	129,35
2019/05/15	CH2205	Male	Three y/o	Treatment (Wk 1)	Treatment	Fast Day	No	04:15	04:00 - 04:59	Morning	37,0	45	16,6	16,6	18,16	530,47	93,96
2019/05/15	CH2206	Male	Three y/o	Treatment (Wk 1)	Treatment	Fast Day	No	04:15	04:00 - 04:59	Morning	36,9	84	49,6	49,6	18,06	581,01	131,51
2019/05/15	CH2205	Male	Three y/o	Treatment (Wk 1)	Treatment	Fast Day	No	04:20	04:00 - 04:59	Morning	37,0	62	13,6	13,6	18,16	557,33	87,18
2019/05/15	CH2206	Male	Three y/o	Treatment (Wk 1)	Treatment	Fast Day	No	04:20	04:00 - 04:59	Morning	36,9	46	57,8	57,8	18,06	532,38	136,80
2019/05/15	CH2205	Male	Three y/o	Treatment (Wk 1)	Treatment	Fast Day	No	04:25	04:00 - 04:59	Morning	37,0	47	14,6	14,6	18,16	534,23	89,59
2019/05/15	CH2206	Male	Three y/o	Treatment (Wk 1)	Treatment	Fast Day	No	04:25	04:00 - 04:59	Morning	36,9	55	57,4	57,4	18,06	547,52	136,56
2019/05/15	CH2205	Male	Three y/o	Treatment (Wk 1)	Treatment	Fast Day	No	04:30	04:00 - 04:59	Morning	37,0	52	17,0	17,0	18,16	542,83	94,77
2019/05/15	CH2206	Male	Three y/o	Treatment (Wk 1)	Treatment	Fast Day	No	04:30	04:00 - 04:59	Morning	36,9	84	13,4	13,4	18,06	581,01	86,68
2019/05/15	CH2205	Male	Three y/o	Treatment (Wk 1)	Treatment	Fast Day	No	04:35	04:00 - 04:59	Morning	37,0		18,2	18,2	18,16		97,10
2019/05/15	CH2206	Male	Three y/o	Treatment (Wk 1)	Treatment	Fast Day	No	04:35	04:00 - 04:59	Morning	37,0	78	4,8	4,8	18,16	575,38	52,04
2019/05/15	CH2205	Male	Three y/o	Treatment (Wk 1)	Treatment	Fast Day	No	04:40	04:00 - 04:59	Morning	37,0	80	15,4	15,4	18,16	577,31	91,41
2019/05/15	CH2206	Male	Three y/o	Treatment (Wk 1)	Treatment	Fast Day	No	04:40	04:00 - 04:59	Morning	37,0	47	15,4	15,4	18,16	534,23	91,41
2019/05/15	CH2205	Male	Three y/o	Treatment (Wk 1)	Treatment	Fast Day	No	04:45	04:00 - 04:59	Morning	37,0	44	10,6	10,6	18,16	528,51	78,73
2019/05/15	CH2206	Male	Three y/o	Treatment (Wk 1)	Treatment	Fast Day	No	04:45	04:00 - 04:59	Morning	36,9	47	21,0	21,0	18,06	534,23	101,98
2019/05/15	CH2205	Male	Three y/o	Treatment (Wk 1)	Treatment	Fast Day	No	04:50	04:00 - 04:59	Morning	37,0		8,2	8,2	18,16		70,05
2019/05/15	CH2206	Male	Three y/o	Treatment (Wk 1)	Treatment	Fast Day	No	04:50	04:00 - 04:59	Morning	36,8	47	17,2	17,2	17,96	534,23	95,17
2019/05/15	CH2205	Male	Three y/o	Treatment (Wk 1)	Treatment	Fast Day	No	04:55	04:00 - 04:59	Morning	36,9	60	9,6	9,6	18,06	554,67	75,37
2019/05/15	CH2206	Male	Three y/o	Treatment (Wk 1)	Treatment	Fast Day	No	04:55	04:00 - 04:59	Morning	36,8	42	12,8	12,8	17,96	524,42	85,12
2019/05/15	CH2205	Male	Three y/o	Treatment (Wk 1)	Treatment	Fast Day	No	05:00	05:00 - 05:59	Morning	36,8	65	8,0	8,0	17,96	561,12	69,22
2019/05/15	CH2206	Male	Three y/o	Treatment (Wk 1)	Treatment	Fast Day	No	05:00	05:00 - 05:59	Morning	36,8	48	18,0	18,0	17,96	536,04	96,72
2019/05/15	CH2205	Male	Three y/o	Treatment (Wk 1)	Treatment	Fast Day	No	05:05	05:00 - 05:59	Morning	36,8		29,0	29,0	17,96		113,03
2019/05/15	CH2206	Male	Three y/o	Treatment (Wk 1)	Treatment	Fast Day	No	05:05	05:00 - 05:59	Morning	36,8	64	23,8	23,8	17,96	559,88	106,26
2019/05/15	CH2205	Male	Three y/o	Treatment (Wk 1)	Treatment	Fast Day	No	05:10	05:00 - 05:59	Morning	37,0	125	16,8	16,8	18,16	609,60	94,37
2019/05/15	CH2206	Male	Three y/o	Treatment (Wk 1)	Treatment	Fast Day	No	05:10	05:00 - 05:59	Morning	36,8	156	88,4	88,4	17,96	624,43	151,55
2019/05/15	CH2205	Male	Three y/o	Treatment (Wk 1)	Treatment	Fast Day	No	05:15	05:00 - 05:59	Morning	37,0	60	7,6	7,6	18,16	554,67	67,49
2019/05/15	CH2206	Male	Three y/o	Treatment (Wk 1)	Treatment	Fast Day	No	05:15	05:00 - 05:59	Morning	37,0	101	126,6	126,6	18,16	594,59	164,08
2019/05/15	CH2205	Male	Three y/o	Treatment (Wk 1)	Treatment	Fast Day	No	05:20	05:00 - 05:59	Morning	37,0	64	4,4	4,4	18,16	559,88	49,12
2019/05/15	CH2206	Male	Three y/o	Treatment (Wk 1)	Treatment	Fast Day	No	05:20	05:00 - 05:59	Morning	37,0		52,2	52,2	18,16		133,27
2019/05/15	CH2205	Male	Three y/o	Treatment (Wk 1)	Treatment	Fast Day	No	05:25	05:00 - 05:59	Morning	36,8		2,8	2,8	17,96		34,04
2019/05/15	CH2206	Male	Three y/o	Treatment (Wk 1)	Treatment	Fast Day	No	05:25	05:00 - 05:59	Morning	36,8	53	25,0	25,0	17,96	544,43	107,94
2019/05/15	CH2205	Male	Three y/o	Treatment (Wk 1)	Treatment	Fast Day	No	05:30	05:00 - 05:59	Morning	36,8	61	5,0	5,0	17,96	556,01	53,41
2019/05/15	CH2206	Male	Three y/o	Treatment (Wk 1)	Treatment	Fast Day	No	05:30	05:00 - 05:59	Morning	36,5	68	28,0	28,0	17,67	564,71	111,83
2019/05/15	CH2205	Male	Three y/o	Treatment (Wk 1)	Treatment	Fast Day	No	05:35	05:00 - 05:59	Morning	36,8	41	7,2	7,2	17,96	522,29	65,66
2019/05/15	CH2206	Male	Three y/o	Treatment (Wk 1)	Treatment	Fast Day	No	05:35	05:00 - 05:59	Morning	36,4	83	28,8	28,8	17,57	580,11	112,79
2019/05/15	CH2205	Male	Three y/o	Treatment (Wk 1)	Treatment	Fast Day	No	05:40	05:00 - 05:59	Morning	36,8	51	13,8	13,8	17,96	541,20	87,68

2019/05/15	CH2206	Male	Three y/o	Treatment (Wk 1)	Treatment	Fast Day	No	05:40	05:00 - 05:59	Morning	36,3	97	35,0	35,0	17,48	591,66	119,49
2019/05/15	CH2205	Male	Three y/o	Treatment (Wk 1)	Treatment	Fast Day	No	05:45	05:00 - 05:59	Morning	36,8	43	3,0	3,0	17,96	526,50	36,34
2019/05/15	CH2206	Male	Three y/o	Treatment (Wk 1)	Treatment	Fast Day	No	05:45	05:00 - 05:59	Morning	36,3	46	35,4	35,4	17,48	532,38	119,88
2019/05/15	CH2205	Male	Three y/o	Treatment (Wk 1)	Treatment	Fast Day	No	05:50	05:00 - 05:59	Morning	36,7	50	5,6	5,6	17,87	539,52	57,21
2019/05/15	CH2206	Male	Three y/o	Treatment (Wk 1)	Treatment	Fast Day	No	05:50	05:00 - 05:59	Morning	36,3	44	37,4	37,4	17,48	528,51	121,77
2019/05/15	CH2205	Male	Three y/o	Treatment (Wk 1)	Treatment	Fast Day	No	05:55	05:00 - 05:59	Morning	36,6	46	8,8	8,8	17,77	532,38	72,43
2019/05/15	CH2206	Male	Three y/o	Treatment (Wk 1)	Treatment	Fast Day	No	05:55	05:00 - 05:59	Morning	36,1	47	51,2	51,2	17,29	534,23	132,61
2019/05/15	CH2205	Male	Three y/o	Treatment (Wk 1)	Treatment	Fast Day	No	06:00	06:00 - 06:59	Morning	36,6	54	11,4	11,4	17,77	545,99	81,19
2019/05/15	CH2206	Male	Three y/o	Treatment (Wk 1)	Treatment	Fast Day	No	06:00	06:00 - 06:59	Morning	36,4	44	44,6	44,6	17,57	528,51	127,84
2019/05/15	CH2205	Male	Three y/o	Treatment (Wk 1)	Treatment	Fast Day	No	06:05	06:00 - 06:59	Morning	36,5		116,2	116,2	17,67		161,08
2019/05/15	CH2206	Male	Three y/o	Treatment (Wk 1)	Treatment	Fast Day	No	06:05	06:00 - 06:59	Morning	36,4	114	105,8	105,8	17,57	603,20	157,81
2019/05/15	CH2205	Male	Three y/o	Treatment (Wk 1)	Treatment	Fast Day	No	06:10	06:00 - 06:59	Morning	36,6		230,8	230,8	17,77		185,16
2019/05/15	CH2206	Male	Three y/o	Treatment (Wk 1)	Treatment	Fast Day	No	06:10	06:00 - 06:59	Morning	36,6		235,0	235,0	17,77		185,80
2019/05/15	CH2205	Male	Three y/o	Treatment (Wk 1)	Treatment	Fast Day	No	06:15	06:00 - 06:59	Morning	36,8	94	47,0	47,0	17,96	589,37	129,65
2019/05/15	CH2206	Male	Three y/o	Treatment (Wk 1)	Treatment	Fast Day	No	06:15	06:00 - 06:59	Morning	36,8		57,8	57,8	17,96		136,80
2019/05/15	CH2205	Male	Three y/o	Treatment (Wk 1)	Treatment	Fast Day	No	06:20	06:00 - 06:59	Morning	36,9		79,8	79,8	18,06		147,99
2019/05/15	CH2206	Male	Three y/o	Treatment (Wk 1)	Treatment	Fast Day	No	06:20	06:00 - 06:59	Morning	37,1	121	78,2	78,2	18,26	607,35	147,28
2019/05/15	CH2205	Male	Three y/o	Treatment (Wk 1)	Treatment	Fast Day	No	06:25	06:00 - 06:59	Morning	37,0	73	153,8	153,8	18,16	570,27	170,89
2019/05/15	CH2206	Male	Three y/o	Treatment (Wk 1)	Treatment	Fast Day	No	06:25	06:00 - 06:59	Morning	37,1		32,2	32,2	18,26		116,63
2019/05/15	CH2205	Male	Three y/o	Treatment (Wk 1)	Treatment	Fast Day	No	06:30	06:00 - 06:59	Morning	37,1		125,0	125,0	18,26		163,63
2019/05/15	CH2206	Male	Three y/o	Treatment (Wk 1)	Treatment	Fast Day	No	06:30	06:00 - 06:59	Morning	37,3	92	248,6	248,6	18,46	587,78	187,78
2019/05/15	CH2205	Male	Three y/o	Treatment (Wk 1)	Treatment	Fast Day	No	06:35	06:00 - 06:59	Morning	37,3		158,4	158,4	18,46		171,93
2019/05/15	CH2206	Male	Three y/o	Treatment (Wk 1)	Treatment	Fast Day	No	06:35	06:00 - 06:59	Morning	37,4	129	61,0	61,0	18,55	611,75	138,67
2019/05/15	CH2205	Male	Three y/o	Treatment (Wk 1)	Treatment	Fast Day	No	06:40	06:00 - 06:59	Morning	37,3	115	106,6	106,6	18,46	603,82	158,07
2019/05/15	CH2206	Male	Three y/o	Treatment (Wk 1)	Treatment	Fast Day	No	06:40	06:00 - 06:59	Morning	37,4	115	78,6	78,6	18,55	603,82	147,46
2019/05/15	CH2205	Male	Three y/o	Treatment (Wk 1)	Treatment	Fast Day	No	06:45	06:00 - 06:59	Morning	37,4	121	34,8	34,8	18,55	607,35	119,29
2019/05/15	CH2206	Male	Three y/o	Treatment (Wk 1)	Treatment	Fast Day	No	06:45	06:00 - 06:59	Morning	37,5	126	353,4	353,4	18,65	610,14	200,22
2019/05/15	CH2205	Male	Three y/o	Treatment (Wk 1)	Treatment	Fast Day	No	06:50	06:00 - 06:59	Morning	37,4	85	172,8	172,8	18,55	581,90	174,98
2019/05/15	CH2206	Male	Three y/o	Treatment (Wk 1)	Treatment	Fast Day	No	06:50	06:00 - 06:59	Morning	37,5	89	47,2	47,2	18,65	585,33	129,80
2019/05/15	CH2205	Male	Three y/o	Treatment (Wk 1)	Treatment	Fast Day	No	06:55	06:00 - 06:59	Morning	37,5	66	76,6	76,6	18,65	562,34	146,57
2019/05/15	CH2206	Male	Three y/o	Treatment (Wk 1)	Treatment	Fast Day	No	06:55	06:00 - 06:59	Morning	37,5	101	121,2	121,2	18,65	594,59	162,56
2019/05/15	CH2205	Male	Three y/o	Treatment (Wk 1)	Treatment	Fast Day	No	07:00	07:00 - 07:59	Morning	37,6		93,0	93,0	18,75		153,31
2019/05/15	CH2206	Male	Three y/o	Treatment (Wk 1)	Treatment	Fast Day	No	07:00	07:00 - 07:59	Morning	37,5	117	119,0	119,0	18,65	605,02	161,92
2019/05/15	CH2205	Male	Three y/o	Treatment (Wk 1)	Treatment	Fast Day	No	07:05	07:00 - 07:59	Morning	37,5		42,4	42,4	18,65		126,10
2019/05/15	CH2206	Male	Three y/o	Treatment (Wk 1)	Treatment	Fast Day	No	07:05	07:00 - 07:59	Morning	37,5	105	122,6	122,6	18,65	597,38	162,96
2019/05/15	CH2205	Male	Three y/o	Treatment (Wk 1)	Treatment	Fast Day	No	07:10	07:00 - 07:59	Morning	37,3		190,8	190,8	18,46		178,46
2019/05/15	CH2206	Male	Three y/o	Treatment (Wk 1)	Treatment	Fast Day	No	07:10	07:00 - 07:59	Morning	37,7	94	726,0	726,0	18,85	589,37	225,86
2019/05/15	CH2205	Male	Three y/o	Treatment (Wk 1)	Treatment	Fast Day	No	07:15	07:00 - 07:59	Morning	37,5	109	59,2	59,2	18,65	600,04	137,63
2019/05/15	CH2206	Male	Three y/o	Treatment (Wk 1)	Treatment	Fast Day	No	07:15	07:00 - 07:59	Morning	37,7	110	89,6	89,6	18,85	600,69	152,02
2019/05/15	CH2205	Male	Three y/o	Treatment (Wk 1)	Treatment	Fast Day	No	07:20	07:00 - 07:59	Morning	37,5		96,2	96,2	18,65		154,49
2019/05/15	CH2206	Male	Three y/o	Treatment (Wk 1)	Treatment	Fast Day	No	07:20	07:00 - 07:59	Morning	37,7	124	101,4	101,4	18,85	609,04	156,33
2019/05/15	CH2205	Male	Three y/o	Treatment (Wk 1)	Treatment	Fast Day	No	07:25	07:00 - 07:59	Morning	37,6		37,0	37,0	18,75		121,40
2019/05/15	CH2206	Male	Three y/o	Treatment (Wk 1)	Treatment	Fast Day	No	07:25	07:00 - 07:59	Morning	37,7	79	236,6	236,6	18,85	576,36	186,04
2019/05/15	CH2205	Male	Three y/o	Treatment (Wk 1)	Treatment	Fast Day	No	07:30	07:00 - 07:59	Morning	37,5	90	7,2	7,2	18,65	586,16	65,66
2019/05/15	CH2206	Male	Three y/o	Treatment (Wk 1)	Treatment	Fast Day	No	07:30	07:00 - 07:59	Morning	37,7	115	107,8	107,8	18,85	603,82	158,46
2019/05/15	CH2205	Male	Three y/o	Treatment (Wk 1)	Treatment	Fast Day	No	07:35	07:00 - 07:59	Morning	37,4		148,8	148,8	18,55		169,74
2019/05/15	CH2206	Male	Three y/o	Treatment (Wk 1)	Treatment	Fast Day	No	07:35	07:00 - 07:59	Morning	37,7	117	77,2	77,2	18,85	605,02	146,84
2019/05/15	CH2205	Male	Three y/o	Treatment (Wk 1)	Treatment	Fast Day	No	07:40	07:00 - 07:59	Morning	37,5	98	227,8	227,8	18,65	592,41	184,70
2019/05/15	CH2206	Male	Three y/o	Treatment (Wk 1)	Treatment	Fast Day	No	07:40	07:00 - 07:59	Morning	37,7	109	224,4	224,4	18,85	600,04	184,17
2019/05/15	CH2205	Male	Three y/o	Treatment (Wk 1)	Treatment	Fast Day	No	07:45	07:00 - 07:59	Morning	37,6	141	288,0	288,0	18,75	617,75	192,98
2019/05/15	CH2206	Male	Three y/o	Treatment (Wk 1)	Treatment	Fast Day	No	07:45	07:00 - 07:59	Morning	37,8	162	955,8		18,95	626,88	
2019/05/15	CH2205	Male	Three y/o	Treatment (Wk 1)	Treatment	Fast Day	No	07:50	07:00 - 07:59	Morning	37,5	82	6,8	6,8	18,65	579,19	63,74
2019/05/15	CH2206	Male	Three y/o	Treatment (Wk 1)	Treatment	Fast Day	No	07:50	07:00 - 07:59	Morning	37,9	51	50,2	50,2	19,05	541,20	131,92
2019/05/15	CH2205	Male	Three y/o	Treatment (Wk 1)	Treatment	Fast Day	No	07:55	07:00 - 07:59	Morning	37,6	66	8,6	8,6	18,75	562,34	71,66
2019/05/15	CH2206	Male	Three y/o	Treatment (Wk 1)	Treatment	Fast Day	No	07:55	07:00 - 07:59	Morning	37,7	54	306,0	306,0	18,85	545,99	195,12
2019/05/15	CH2205	Male	Three y/o	Treatment (Wk 1)	Treatment	Fast Day	No	08:00	08:00 - 08:59	Late Morning	37,6	152	14,8	14,8	18,75	622,73	90,05
2019/05/15	CH2206	Male	Three y/o	Treatment (Wk 1)	Treatment	Fast Day	No	08:00	08:00 - 08:59	Late Morning	37,7	37	75,4	75,4	18,85	513,06	146,02
2019/05/15	CH2205	Male	Three y/o	Treatment (Wk 1)	Treatment	Fast Day	No	08:05	08:00 - 08:59	Late Morning	37,6	49	6,8	6,8	18,75	537,80	63,74
2019/05/15	CH2206	Male	Three y/o	Treatment (Wk 1)	Treatment	Fast Day	No	08:05	08:00 - 08:59	Late Morning	37,7	99	254,4	254,4	18,85	593,14	188,60
2019/05/15	CH2205	Male	Three y/o	Treatment (Wk 1)	Treatment	Fast Day	No	08:10	08:00 - 08:59	Late Morning	37,5	96	73,2	73,2	18,65	590,91	144,99
2019/05/15	CH2206	Male	Three y/o	Treatment (Wk 1)	Treatment	Fast Day	No	08:10	08:00 - 08:59	Late Morning	37,7	123	96,4	96,4	18,85	608,49	154,57
2019/05/15	CH2205	Male	Three y/o	Treatment (Wk 1)	Treatment	Fast Day	No	08:15	08:00 - 08:59	Late Morning	37,5		95,6	95,6	18,65		154,28
2019/05/15	CH2206	Male	Three y/o	Treatment (Wk 1)	Treatment	Fast Day	No	08:15	08:00 - 08:59	Late Morning	37,6	115	174,6	174,6	18,75	603,82	175,34
2019/05/15	CH2205	Male	Three y/o	Treatment (Wk 1)	Treatment	Fast Day	No	08:20	08:00 - 08:59	Late Morning	37,5	67	79,4	79,4	18,65	563,53	147,81
2019/05/15	CH2206	Male	Three y/o	Treatment (Wk 1)	Treatment	Fast Day	No	08:20	08:00 - 08:59	Late Morning	37,7	112	163,2	163,2	18,85	601,96	172,97
2019/05/15	CH2205	Male	Three y/o	Treatment (Wk 1)	Treatment	Fast Day	No	08:25	08:00 - 08:59	Late Morning	37,5	96	185,0	185,0	18,65	590,91	177,38
2019/05/15	CH2206	Male	Three y/o	Treatment (Wk 1)	Treatment	Fast Day	No	08:25	08:00 - 08:59	Late Morning	37,7	118	52,6	52,6	18,85	605,61	133,54
2019/05/15	CH2205	Male	Three y/o	Treatment (Wk 1)	Treatment	Fast Day	No	08:30	08:00 - 08:59	Late Morning	37,5	84	75,8	75,8	18,65	581,01	146,20
2019/05/15	CH2206	Male	Three y/o	Treatment (Wk 1)	Treatment	Fast Day	No	08:30	08:00 - 08:59	Late Morning	37,7	128	26,2	26,2	18,85	611,22	109,55
2019/05/15	CH2205	Male	Three y/o	Treatment (Wk 1)	Treatment	Fast Day	No	08:35	08:00 - 08:59	Late Morning	37,6	132	62,2	62,2	18,75	613,32	139,34

2019/05/15	CH2206	Male	Three y/o	Treatment (Wk 1)	Treatment	Fast Day	No	08:35	08:00 - 08:59	Late Morning	37,5	131	110,0	110,0	18,65	612,80	159,17
2019/05/15	CH2205	Male	Three y/o	Treatment (Wk 1)	Treatment	Fast Day	No	08:40	08:00 - 08:59	Late Morning	37,7	100	80,2	80,2	18,85	593,87	148,16
2019/05/15	CH2206	Male	Three y/o	Treatment (Wk 1)	Treatment	Fast Day	No	08:40	08:00 - 08:59	Late Morning	37,7	104	154,6	154,6	18,85	596,69	171,08
2019/05/15	CH2205	Male	Three y/o	Treatment (Wk 1)	Treatment	Fast Day	No	08:45	08:00 - 08:59	Late Morning	37,7	107	134,8	134,8	18,85	598,73	166,28
2019/05/15	CH2206	Male	Three y/o	Treatment (Wk 1)	Treatment	Fast Day	No	08:45	08:00 - 08:59	Late Morning	37,7	111	113,6	113,6	18,85	601,33	160,29
2019/05/15	CH2205	Male	Three y/o	Treatment (Wk 1)	Treatment	Fast Day	No	08:50	08:00 - 08:59	Late Morning	37,7	83	3,6	3,6	18,85	580,11	42,42
2019/05/15	CH2206	Male	Three y/o	Treatment (Wk 1)	Treatment	Fast Day	No	08:50	08:00 - 08:59	Late Morning	37,7	111	124,0	124,0	18,85	601,33	163,35
2019/05/15	CH2205	Male	Three y/o	Treatment (Wk 1)	Treatment	Fast Day	No	08:55	08:00 - 08:59	Late Morning	37,7		128,2	128,2	18,85		164,52
2019/05/15	CH2206	Male	Three y/o	Treatment (Wk 1)	Treatment	Fast Day	No	08:55	08:00 - 08:59	Late Morning	37,5	114	284,6	284,6	18,65	603,20	192,56
2019/05/15	CH2205	Male	Three y/o	Treatment (Wk 1)	Treatment	Fast Day	No	09:00	09:00 - 09:59	Late Morning	37,8	68	11,4	11,4	18,95	564,71	81,19
2019/05/15	CH2206	Male	Three y/o	Treatment (Wk 1)	Treatment	Fast Day	No	09:00	09:00 - 09:59	Late Morning	37,7	67	40,2	40,2	18,85	563,53	124,26
2019/05/15	CH2205	Male	Three y/o	Treatment (Wk 1)	Treatment	Fast Day	No	09:05	09:00 - 09:59	Late Morning	37,7	73	7,8	7,8	18,85	570,27	68,36
2019/05/15	CH2206	Male	Three y/o	Treatment (Wk 1)	Treatment	Fast Day	No	09:05	09:00 - 09:59	Late Morning	37,5	52	43,4	43,4	18,65	542,83	126,90
2019/05/15	CH2205	Male	Three y/o	Treatment (Wk 1)	Treatment	Fast Day	No	09:10	09:00 - 09:59	Late Morning	37,7		12,6	12,6	18,85		84,59
2019/05/15	CH2206	Male	Three y/o	Treatment (Wk 1)	Treatment	Fast Day	No	09:10	09:00 - 09:59	Late Morning	37,5	56	63,0	63,0	18,65	549,01	139,78
2019/05/15	CH2205	Male	Three y/o	Treatment (Wk 1)	Treatment	Fast Day	No	09:15	09:00 - 09:59	Late Morning	37,7		81,6	81,6	18,85		148,76
2019/05/15	CH2206	Male	Three y/o	Treatment (Wk 1)	Treatment	Fast Day	No	09:15	09:00 - 09:59	Late Morning	37,7	60	69,0	69,0	18,85	554,67	142,94
2019/05/15	CH2205	Male	Three y/o	Treatment (Wk 1)	Treatment	Fast Day	No	09:20	09:00 - 09:59	Late Morning	37,7	78	19,4	19,4	18,85	575,38	99,28
2019/05/15	CH2206	Male	Three y/o	Treatment (Wk 1)	Treatment	Fast Day	No	09:20	09:00 - 09:59	Late Morning	37,5	126	80,0	80,0	18,65	610,14	148,08
2019/05/15	CH2205	Male	Three y/o	Treatment (Wk 1)	Treatment	Fast Day	No	09:25	09:00 - 09:59	Late Morning	37,6	88	75,0	75,0	18,75	584,49	145,83
2019/05/15	CH2206	Male	Three y/o	Treatment (Wk 1)	Treatment	Fast Day	No	09:25	09:00 - 09:59	Late Morning	37,5	76	130,4	130,4	18,65	573,39	165,11
2019/05/15	CH2205	Male	Three y/o	Treatment (Wk 1)	Treatment	Fast Day	No	09:30	09:00 - 09:59	Late Morning	37,6	103	35,0	35,0	18,75	596,00	119,49
2019/05/15	CH2206	Male	Three y/o	Treatment (Wk 1)	Treatment	Fast Day	No	09:30	09:00 - 09:59	Late Morning	37,4	80	297,4	297,4	18,55	577,31	194,11
2019/05/15	CH2205	Male	Three y/o	Treatment (Wk 1)	Treatment	Fast Day	No	09:35	09:00 - 09:59	Late Morning	37,6	191	41,8	41,8	18,75	637,33	125,60
2019/05/15	CH2206	Male	Three y/o	Treatment (Wk 1)	Treatment	Fast Day	No	09:35	09:00 - 09:59	Late Morning	37,5	92	71,0	71,0	18,65	587,78	143,93
2019/05/15	CH2205	Male	Three y/o	Treatment (Wk 1)	Treatment	Fast Day	No	09:40	09:00 - 09:59	Late Morning	37,6		114,6	114,6	18,75		160,60
2019/05/15	CH2206	Male	Three y/o	Treatment (Wk 1)	Treatment	Fast Day	No	09:40	09:00 - 09:59	Late Morning	37,4	54	48,8	48,8	18,55	545,99	130,95
2019/05/15	CH2205	Male	Three y/o	Treatment (Wk 1)	Treatment	Fast Day	No	09:45	09:00 - 09:59	Late Morning	37,6	77	18,0	18,0	18,75	574,39	96,72
2019/05/15	CH2206	Male	Three y/o	Treatment (Wk 1)	Treatment	Fast Day	No	09:45	09:00 - 09:59	Late Morning	37,4	52	52,0	52,0	18,55	542,83	133,14
2019/05/15	CH2205	Male	Three y/o	Treatment (Wk 1)	Treatment	Fast Day	No	09:50	09:00 - 09:59	Late Morning	37,6	68	11,0	11,0	18,75	564,71	79,98
2019/05/15	CH2206	Male	Three y/o	Treatment (Wk 1)	Treatment	Fast Day	No	09:50	09:00 - 09:59	Late Morning	37,3	46	116,4	116,4	18,46	532,38	161,14
2019/05/15	CH2205	Male	Three y/o	Treatment (Wk 1)	Treatment	Fast Day	No	09:55	09:00 - 09:59	Late Morning	37,6	165	171,4	171,4	18,75	628,06	174,70
2019/05/15	CH2206	Male	Three y/o	Treatment (Wk 1)	Treatment	Fast Day	No	09:55	09:00 - 09:59	Late Morning	37,2	137	46,4	46,4	18,36	615,83	129,21
2019/05/15	CH2205	Male	Three y/o	Treatment (Wk 1)	Treatment	Fast Day	No	10:00	10:00 - 10:59	Late Morning	37,7	85	30,0	30,0	18,85	581,90	114,20
2019/05/15	CH2206	Male	Three y/o	Treatment (Wk 1)	Treatment	Fast Day	No	10:00	10:00 - 10:59	Late Morning	37,1	46	55,4	55,4	18,26	532,38	135,33
2019/05/15	CH2205	Male	Three y/o	Treatment (Wk 1)	Treatment	Fast Day	No	10:05	10:00 - 10:59	Late Morning	37,6	64	25,8	25,8	18,75	559,88	109,02
2019/05/15	CH2206	Male	Three y/o	Treatment (Wk 1)	Treatment	Fast Day	No	10:05	10:00 - 10:59	Late Morning	37,0	84	39,2	39,2	18,16	581,01	123,39
2019/05/15	CH2205	Male	Three y/o	Treatment (Wk 1)	Treatment	Fast Day	No	10:10	10:00 - 10:59	Late Morning	37,7		19,2	19,2	18,85		98,92
2019/05/15	CH2206	Male	Three y/o	Treatment (Wk 1)	Treatment	Fast Day	No	10:10	10:00 - 10:59	Late Morning	37,1	61	31,0	31,0	18,26	556,01	115,32
2019/05/15	CH2205	Male	Three y/o	Treatment (Wk 1)	Treatment	Fast Day	No	10:15	10:00 - 10:59	Late Morning	37,7		48,2	48,2	18,85		130,52
2019/05/15	CH2206	Male	Three y/o	Treatment (Wk 1)	Treatment	Fast Day	No	10:15	10:00 - 10:59	Late Morning	37,2	45	20,8	20,8	18,36	530,47	101,65
2019/05/15	CH2205	Male	Three y/o	Treatment (Wk 1)	Treatment	Fast Day	No	10:20	10:00 - 10:59	Late Morning	37,7	72	14,2	14,2	18,85	569,20	88,65
2019/05/15	CH2206	Male	Three y/o	Treatment (Wk 1)	Treatment	Fast Day	No	10:20	10:00 - 10:59	Late Morning	37,2	44	19,6	19,6	18,36	528,51	99,63
2019/05/15	CH2205	Male	Three y/o	Treatment (Wk 1)	Treatment	Fast Day	No	10:25	10:00 - 10:59	Late Morning	37,7	61	10,4	10,4	18,85	556,01	78,08
2019/05/15	CH2206	Male	Three y/o	Treatment (Wk 1)	Treatment	Fast Day	No	10:25	10:00 - 10:59	Late Morning	37,3	51	47,0	47,0	18,46	541,20	129,65
2019/05/15	CH2205	Male	Three y/o	Treatment (Wk 1)	Treatment	Fast Day	No	10:30	10:00 - 10:59	Late Morning	37,5		11,2	11,2	18,65		80,59
2019/05/15	CH2206	Male	Three y/o	Treatment (Wk 1)	Treatment	Fast Day	No	10:30	10:00 - 10:59	Late Morning	37,2	54	61,6	61,6	18,36	545,99	139,00
2019/05/15	CH2205	Male	Three y/o	Treatment (Wk 1)	Treatment	Fast Day	No	10:35	10:00 - 10:59	Late Morning	37,5	63	8,2	8,2	18,65	558,62	70,05
2019/05/15	CH2206	Male	Three y/o	Treatment (Wk 1)	Treatment	Fast Day	No	10:35	10:00 - 10:59	Late Morning	36,9	74	54,4	54,4	18,06	571,33	134,70
2019/05/15	CH2205	Male	Three y/o	Treatment (Wk 1)	Treatment	Fast Day	No	10:40	10:00 - 10:59	Late Morning	37,5	76	13,8	13,8	18,65	573,39	87,68
2019/05/15	CH2206	Male	Three y/o	Treatment (Wk 1)	Treatment	Fast Day	No	10:40	10:00 - 10:59	Late Morning	36,8	108	93,2	93,2	17,96	599,39	153,39
2019/05/15	CH2205	Male	Three y/o	Treatment (Wk 1)	Treatment	Fast Day	No	10:45	10:00 - 10:59	Late Morning	37,5	90	43,4	43,4	18,65	586,16	126,90
2019/05/15	CH2206	Male	Three y/o	Treatment (Wk 1)	Treatment	Fast Day	No	10:45	10:00 - 10:59	Late Morning	36,9	83	76,4	76,4	18,06	580,11	146,48
2019/05/15	CH2205	Male	Three y/o	Treatment (Wk 1)	Treatment	Fast Day	No	10:50	10:00 - 10:59	Late Morning	37,5		21,2	21,2	18,65		102,31
2019/05/15	CH2206	Male	Three y/o	Treatment (Wk 1)	Treatment	Fast Day	No	10:50	10:00 - 10:59	Late Morning	37,0	54	24,0	24,0	18,16	545,99	106,55
2019/05/15	CH2205	Male	Three y/o	Treatment (Wk 1)	Treatment	Fast Day	No	10:55	10:00 - 10:59	Late Morning	37,4	118	16,8	16,8	18,55	605,61	94,37
2019/05/15	CH2206	Male	Three y/o	Treatment (Wk 1)	Treatment	Fast Day	No	10:55	10:00 - 10:59	Late Morning	37,0	53	28,6	28,6	18,16	544,43	112,56
2019/05/15	CH2205	Male	Three y/o	Treatment (Wk 1)	Treatment	Fast Day	No	11:00	11:00 - 11:59	Late Morning	37,4	61	77,0	77,0	18,55	556,01	146,75
2019/05/15	CH2206	Male	Three y/o	Treatment (Wk 1)	Treatment	Fast Day	No	11:00	11:00 - 11:59	Late Morning	37,1	70	20,6	20,6	18,26	566,99	101,32
2019/05/15	CH2205	Male	Three y/o	Treatment (Wk 1)	Treatment	Fast Day	No	11:05	11:00 - 11:59	Late Morning	37,4	78	23,4	23,4	18,55	575,38	105,68
2019/05/15	CH2206	Male	Three y/o	Treatment (Wk 1)	Treatment	Fast Day	No	11:05	11:00 - 11:59	Late Morning	37,2	54	6,6	6,6	18,36	545,99	62,73
2019/05/15	CH2205	Male	Three y/o	Treatment (Wk 1)	Treatment	Fast Day	No	11:10	11:00 - 11:59	Late Morning	37,4	76	132,2	132,2	18,55	573,39	165,59
2019/05/15	CH2206	Male	Three y/o	Treatment (Wk 1)	Treatment	Fast Day	No	11:10	11:00 - 11:59	Late Morning	37,2	93	533,8	533,8	18,36	588,58	214,88
2019/05/15	CH2205	Male	Three y/o	Treatment (Wk 1)	Treatment	Fast Day	No	11:15	11:00 - 11:59	Late Morning	37,4		675,0	675,0	18,55		223,25
2019/05/15	CH2206	Male	Three y/o	Treatment (Wk 1)	Treatment	Fast Day	No	11:15	11:00 - 11:59	Late Morning	37,3	56	124,0	124,0	18,46	549,01	163,35
2019/05/15	CH2205	Male	Three y/o	Treatment (Wk 1)	Treatment	Fast Day	No	11:20	11:00 - 11:59	Late Morning	37,5	90	150,6	150,6	18,65	586,16	170,16
2019/05/15	CH2206	Male	Three y/o	Treatment (Wk 1)	Treatment	Fast Day	No	11:20	11:00 - 11:59	Late Morning	37,3	85	26,2	26,2	18,46	581,90	109,55
2019/05/15	CH2205	Male	Three y/o	Treatment (Wk 1)	Treatment	Fast Day	No	11:25	11:00 - 11:59	Late Morning	37,5	102	37,8	37,8	18,65	595,30	122,14
2019/05/15	CH2206	Male	Three y/o	Treatment (Wk 1)	Treatment	Fast Day	No	11:25	11:00 - 11:59	Late Morning	37,3	57	78,2	78,2	18,46	550,47	147,28
2019/05/15	CH2205	Male	Three y/o	Treatment (Wk 1)	Treatment	Fast Day	No	11:30	11:00 - 11:59	Late Morning	37,6		41,8	41,8	18,75		125,60

2019/05/15	CH2206	Male	Three y/o	Treatment (Wk 1)	Treatment	Fast Day	No	11:30	11:00 - 11:59	Late Morning	37,4	120	48,4	48,4	18,55	606,78	130,66
2019/05/15	CH2205	Male	Three y/o	Treatment (Wk 1)	Treatment	Fast Day	No	11:35	11:00 - 11:59	Late Morning	37,7		48,2	48,2	18,85		130,52
2019/05/15	CH2206	Male	Three y/o	Treatment (Wk 1)	Treatment	Fast Day	No	11:35	11:00 - 11:59	Late Morning	37,5	51	8,8	8,8	18,65	541,20	72,43
2019/05/15	CH2205	Male	Three y/o	Treatment (Wk 1)	Treatment	Fast Day	No	11:40	11:00 - 11:59	Late Morning	37,6	86	44,0	44,0	18,75	582,77	127,37
2019/05/15	CH2206	Male	Three y/o	Treatment (Wk 1)	Treatment	Fast Day	No	11:40	11:00 - 11:59	Late Morning	37,4	120	49,4	49,4	18,55	606,78	131,37
2019/05/15	CH2205	Male	Three y/o	Treatment (Wk 1)	Treatment	Fast Day	No	11:45	11:00 - 11:59	Late Morning	37,5	94	90,4	90,4	18,65	589,37	152,33
2019/05/15	CH2206	Male	Three y/o	Treatment (Wk 1)	Treatment	Fast Day	No	11:45	11:00 - 11:59	Late Morning	37,4	96	32,0	32,0	18,55	590,91	116,41
2019/05/15	CH2205	Male	Three y/o	Treatment (Wk 1)	Treatment	Fast Day	No	11:50	11:00 - 11:59	Late Morning	37,6	70	24,6	24,6	18,75	566,99	107,39
2019/05/15	CH2206	Male	Three y/o	Treatment (Wk 1)	Treatment	Fast Day	No	11:50	11:00 - 11:59	Late Morning	37,4	48	37,8	37,8	18,55	536,04	122,14
2019/05/15	CH2205	Male	Three y/o	Treatment (Wk 1)	Treatment	Fast Day	No	11:55	11:00 - 11:59	Late Morning	37,5		44,2	44,2	18,65		127,53
2019/05/15	CH2206	Male	Three y/o	Treatment (Wk 1)	Treatment	Fast Day	No	11:55	11:00 - 11:59	Late Morning	37,4	108	39,0	39,0	18,55	599,39	123,22
2019/05/15	CH2205	Male	Three y/o	Treatment (Wk 1)	Treatment	Fast Day	No	12:00	12:00 - 12:59	Afternoon	37,6	197	21,8	21,8	18,75	639,25	103,26
2019/05/15	CH2206	Male	Three y/o	Treatment (Wk 1)	Treatment	Fast Day	No	12:00	12:00 - 12:59	Afternoon	37,4	68	34,2	34,2	18,55	564,71	118,70
2019/05/15	CH2205	Male	Three y/o	Treatment (Wk 1)	Treatment	Fast Day	No	12:05	12:00 - 12:59	Afternoon	37,5	70	6,8	6,8	18,65	566,99	63,74
2019/05/15	CH2206	Male	Three y/o	Treatment (Wk 1)	Treatment	Fast Day	No	12:05	12:00 - 12:59	Afternoon	37,4	58	31,8	31,8	18,55	551,90	116,20
2019/05/15	CH2205	Male	Three y/o	Treatment (Wk 1)	Treatment	Fast Day	No	12:10	12:00 - 12:59	Afternoon	37,5	59	9,4	9,4	18,65	553,30	74,66
2019/05/15	CH2206	Male	Three y/o	Treatment (Wk 1)	Treatment	Fast Day	No	12:10	12:00 - 12:59	Afternoon	37,4	125	46,0	46,0	18,55	609,60	128,91
2019/05/15	CH2205	Male	Three y/o	Treatment (Wk 1)	Treatment	Fast Day	No	12:15	12:00 - 12:59	Afternoon	37,5	65	11,4	11,4	18,65	561,12	81,19
2019/05/15	CH2206	Male	Three y/o	Treatment (Wk 1)	Treatment	Fast Day	No	12:15	12:00 - 12:59	Afternoon	37,5	73	48,0	48,0	18,65	570,27	130,38
2019/05/15	CH2205	Male	Three y/o	Treatment (Wk 1)	Treatment	Fast Day	No	12:20	12:00 - 12:59	Afternoon	37,5	64	13,6	13,6	18,65	559,88	87,18
2019/05/15	CH2206	Male	Three y/o	Treatment (Wk 1)	Treatment	Fast Day	No	12:20	12:00 - 12:59	Afternoon	37,5	76	47,0	47,0	18,65	573,39	129,65
2019/05/15	CH2205	Male	Three y/o	Treatment (Wk 1)	Treatment	Fast Day	No	12:25	12:00 - 12:59	Afternoon	37,5	56	9,2	9,2	18,65	549,01	73,94
2019/05/15	CH2206	Male	Three y/o	Treatment (Wk 1)	Treatment	Fast Day	No	12:25	12:00 - 12:59	Afternoon	37,5	60	51,8	51,8	18,65	554,67	133,01
2019/05/15	CH2205	Male	Three y/o	Treatment (Wk 1)	Treatment	Fast Day	No	12:30	12:00 - 12:59	Afternoon	37,5	68	17,2	17,2	18,65	564,71	95,17
2019/05/15	CH2206	Male	Three y/o	Treatment (Wk 1)	Treatment	Fast Day	No	12:30	12:00 - 12:59	Afternoon	37,5	66	50,6	50,6	18,65	562,34	132,20
2019/05/15	CH2205	Male	Three y/o	Treatment (Wk 1)	Treatment	Fast Day	No	12:35	12:00 - 12:59	Afternoon	37,5	86	10,4	10,4	18,65	582,77	78,08
2019/05/15	CH2206	Male	Three y/o	Treatment (Wk 1)	Treatment	Fast Day	No	12:35	12:00 - 12:59	Afternoon	37,7	59	51,2	51,2	18,85	553,30	132,61
2019/05/15	CH2205	Male	Three y/o	Treatment (Wk 1)	Treatment	Fast Day	No	12:40	12:00 - 12:59	Afternoon	37,5	62	15,2	15,2	18,65	557,33	90,96
2019/05/15	CH2206	Male	Three y/o	Treatment (Wk 1)	Treatment	Fast Day	No	12:40	12:00 - 12:59	Afternoon	37,7	82	50,4	50,4	18,85	579,19	132,06
2019/05/15	CH2205	Male	Three y/o	Treatment (Wk 1)	Treatment	Fast Day	No	12:45	12:00 - 12:59	Afternoon	37,5	76	18,4	18,4	18,65	573,39	97,47
2019/05/15	CH2206	Male	Three y/o	Treatment (Wk 1)	Treatment	Fast Day	No	12:45	12:00 - 12:59	Afternoon	37,7	77	53,2	53,2	18,85	574,39	133,93
2019/05/15	CH2205	Male	Three y/o	Treatment (Wk 1)	Treatment	Fast Day	No	12:50	12:00 - 12:59	Afternoon	37,5	64	14,2	14,2	18,65	559,88	88,65
2019/05/15	CH2206	Male	Three y/o	Treatment (Wk 1)	Treatment	Fast Day	No	12:50	12:00 - 12:59	Afternoon	37,7	68	49,8	49,8	18,85	564,71	131,65
2019/05/15	CH2205	Male	Three y/o	Treatment (Wk 1)	Treatment	Fast Day	No	12:55	12:00 - 12:59	Afternoon	37,6	120	18,4	18,4	18,75	606,78	97,47
2019/05/15	CH2206	Male	Three y/o	Treatment (Wk 1)	Treatment	Fast Day	No	12:55	12:00 - 12:59	Afternoon	37,7	90	51,2	51,2	18,85	586,16	132,61
2019/05/15	CH2205	Male	Three y/o	Treatment (Wk 1)	Treatment	Fast Day	No	13:00	13:00 - 13:59	Afternoon	37,6	73	15,8	15,8	18,75	570,27	92,28
2019/05/15	CH2206	Male	Three y/o	Treatment (Wk 1)	Treatment	Fast Day	No	13:00	13:00 - 13:59	Afternoon	37,7	62	52,6	52,6	18,85	557,33	133,54
2019/05/15	CH2205	Male	Three y/o	Treatment (Wk 1)	Treatment	Fast Day	No	13:05	13:00 - 13:59	Afternoon	37,6	67	18,4	18,4	18,75	563,53	97,47
2019/05/15	CH2206	Male	Three y/o	Treatment (Wk 1)	Treatment	Fast Day	No	13:05	13:00 - 13:59	Afternoon	37,7	77	48,8	48,8	18,85	574,39	130,95
2019/05/15	CH2205	Male	Three y/o	Treatment (Wk 1)	Treatment	Fast Day	No	13:10	13:00 - 13:59	Afternoon	37,7	60	21,6	21,6	18,85	554,67	102,94
2019/05/15	CH2206	Male	Three y/o	Treatment (Wk 1)	Treatment	Fast Day	No	13:10	13:00 - 13:59	Afternoon	37,7	97	41,4	41,4	18,85	591,66	125,27
2019/05/15	CH2205	Male	Three y/o	Treatment (Wk 1)	Treatment	Fast Day	No	13:15	13:00 - 13:59	Afternoon	37,7	64	19,6	19,6	18,85	559,88	99,63
2019/05/15	CH2206	Male	Three y/o	Treatment (Wk 1)	Treatment	Fast Day	No	13:15	13:00 - 13:59	Afternoon	37,7	78	19,8	19,8	18,85	575,38	99,97
2019/05/15	CH2205	Male	Three y/o	Treatment (Wk 1)	Treatment	Fast Day	No	13:20	13:00 - 13:59	Afternoon	37,7	64	20,6	20,6	18,85	559,88	101,32
2019/05/15	CH2206	Male	Three y/o	Treatment (Wk 1)	Treatment	Fast Day	No	13:20	13:00 - 13:59	Afternoon	37,7	69	17,6	17,6	18,85	565,86	95,95
2019/05/15	CH2205	Male	Three y/o	Treatment (Wk 1)	Treatment	Fast Day	No	13:25	13:00 - 13:59	Afternoon	37,7	76	37,6	37,6	18,85	573,39	121,96
2019/05/15	CH2206	Male	Three y/o	Treatment (Wk 1)	Treatment	Fast Day	No	13:25	13:00 - 13:59	Afternoon	37,7	63	41,4	41,4	18,85	558,62	125,27
2019/05/15	CH2205	Male	Three y/o	Treatment (Wk 1)	Treatment	Fast Day	No	13:30	13:00 - 13:59	Afternoon	37,7	60	15,0	15,0	18,85	554,67	90,51
2019/05/15	CH2206	Male	Three y/o	Treatment (Wk 1)	Treatment	Fast Day	No	13:30	13:00 - 13:59	Afternoon	37,7	53	58,2	58,2	18,85	544,43	137,04
2019/05/15	CH2205	Male	Three y/o	Treatment (Wk 1)	Treatment	Fast Day	No	13:35	13:00 - 13:59	Afternoon	37,7	58	12,2	12,2	18,85	551,90	83,49
2019/05/15	CH2206	Male	Three y/o	Treatment (Wk 1)	Treatment	Fast Day	No	13:35	13:00 - 13:59	Afternoon	37,7	83	62,4	62,4	18,85	580,11	139,45
2019/05/15	CH2205	Male	Three y/o	Treatment (Wk 1)	Treatment	Fast Day	No	13:40	13:00 - 13:59	Afternoon	37,7	84	13,8	13,8	18,85	581,01	87,68
2019/05/15	CH2206	Male	Three y/o	Treatment (Wk 1)	Treatment	Fast Day	No	13:40	13:00 - 13:59	Afternoon	37,8	100	39,0	39,0	18,95	593,87	123,22
2019/05/15	CH2205	Male	Three y/o	Treatment (Wk 1)	Treatment	Fast Day	No	13:45	13:00 - 13:59	Afternoon	37,7	57	13,2	13,2	18,85	550,47	86,17
2019/05/15	CH2206	Male	Three y/o	Treatment (Wk 1)	Treatment	Fast Day	No	13:45	13:00 - 13:59	Afternoon	37,8	79	48,6	48,6	18,95	576,36	130,81
2019/05/15	CH2205	Male	Three y/o	Treatment (Wk 1)	Treatment	Fast Day	No	13:50	13:00 - 13:59	Afternoon	37,7	102	17,8	17,8	18,85	595,30	96,34
2019/05/15	CH2206	Male	Three y/o	Treatment (Wk 1)	Treatment	Fast Day	No	13:50	13:00 - 13:59	Afternoon	37,8	98	29,0	29,0	18,95	592,41	113,03
2019/05/15	CH2205	Male	Three y/o	Treatment (Wk 1)	Treatment	Fast Day	No	13:55	13:00 - 13:59	Afternoon	37,7	59	18,8	18,8	18,85	553,30	98,20
2019/05/15	CH2206	Male	Three y/o	Treatment (Wk 1)	Treatment	Fast Day	No	13:55	13:00 - 13:59	Afternoon	37,8	80	21,0	21,0	18,95	577,31	101,98
2019/05/15	CH2205	Male	Three y/o	Treatment (Wk 1)	Treatment	Fast Day	No	14:00	14:00 - 14:59	Afternoon	37,7	74	14,0	14,0	18,85	571,33	88,17
2019/05/15	CH2206	Male	Three y/o	Treatment (Wk 1)	Treatment	Fast Day	No	14:00	14:00 - 14:59	Afternoon	37,8	77	5,6	5,6	18,95	574,39	57,21
2019/05/15	CH2205	Male	Three y/o	Treatment (Wk 1)	Treatment	Fast Day	No	14:05	14:00 - 14:59	Afternoon	37,7	69	19,2	19,2	18,85	565,86	98,92
2019/05/15	CH2206	Male	Three y/o	Treatment (Wk 1)	Treatment	Fast Day	No	14:05	14:00 - 14:59	Afternoon	37,8	77	13,8	13,8	18,95	574,39	87,68
2019/05/15	CH2205	Male	Three y/o	Treatment (Wk 1)	Treatment	Fast Day	No	14:10	14:00 - 14:59	Afternoon	37,9	61	17,8	17,8	19,05	556,01	96,34
2019/05/15	CH2206	Male	Three y/o	Treatment (Wk 1)	Treatment	Fast Day	No	14:10	14:00 - 14:59	Afternoon	37,8	94	3,4	3,4	18,95	589,37	40,51
2019/05/15	CH2205	Male	Three y/o	Treatment (Wk 1)	Treatment	Fast Day	No	14:15	14:00 - 14:59	Afternoon	37,9	95	14,6	14,6	19,05	590,14	89,59
2019/05/15	CH2206	Male	Three y/o	Treatment (Wk 1)	Treatment	Fast Day	No	14:15	14:00 - 14:59	Afternoon	37,7	67	6,2	6,2	18,85	563,53	60,63
2019/05/15	CH2205	Male	Three y/o	Treatment (Wk 1)	Treatment	Fast Day	No	14:20	14:00 - 14:59	Afternoon	37,9	73	16,2	16,2	19,05	570,27	93,13
2019/05/15	CH2206	Male	Three y/o	Treatment (Wk 1)	Treatment	Fast Day	No	14:20	14:00 - 14:59	Afternoon	37,7	87	6,6	6,6	18,85	583,64	62,73
2019/05/15	CH2205	Male	Three y/o	Treatment (Wk 1)	Treatment	Fast Day	No	14:25	14:00 - 14:59	Afternoon	37,9	64	22,2	22,2	19,05	559,88	103,88

2019/05/15	CH2206	Male	Three y/o	Treatment (Wk 1)	Treatment	Fast Day	No	14:25	14:00 - 14:59	Afternoon	37,8	70	27,4	27,4	18,95	566,99	111,09
2019/05/15	CH2205	Male	Three y/o	Treatment (Wk 1)	Treatment	Fast Day	No	14:30	14:00 - 14:59	Afternoon	37,9	63	26,0	26,0	19,05	558,62	109,29
2019/05/15	CH2206	Male	Three y/o	Treatment (Wk 1)	Treatment	Fast Day	No	14:30	14:00 - 14:59	Afternoon	37,8	77	34,2	34,2	18,95	574,39	118,70
2019/05/15	CH2205	Male	Three y/o	Treatment (Wk 1)	Treatment	Fast Day	No	14:35	14:00 - 14:59	Afternoon	37,9	67	22,6	22,6	19,05	563,53	104,49
2019/05/15	CH2206	Male	Three y/o	Treatment (Wk 1)	Treatment	Fast Day	No	14:35	14:00 - 14:59	Afternoon	37,8	57	28,8	28,8	18,95	550,47	112,79
2019/05/15	CH2205	Male	Three y/o	Treatment (Wk 1)	Treatment	Fast Day	No	14:40	14:00 - 14:59	Afternoon	37,9	63	20,8	20,8	19,05	558,62	101,65
2019/05/15	CH2206	Male	Three y/o	Treatment (Wk 1)	Treatment	Fast Day	No	14:40	14:00 - 14:59	Afternoon	37,8	71	27,0	27,0	18,95	568,10	110,58
2019/05/15	CH2205	Male	Three y/o	Treatment (Wk 1)	Treatment	Fast Day	No	14:45	14:00 - 14:59	Afternoon	37,9	68	33,2	33,2	19,05	564,71	117,68
2019/05/15	CH2206	Male	Three y/o	Treatment (Wk 1)	Treatment	Fast Day	No	14:45	14:00 - 14:59	Afternoon	37,8	63	29,6	29,6	18,95	558,62	113,73
2019/05/15	CH2205	Male	Three y/o	Treatment (Wk 1)	Treatment	Fast Day	No	14:50	14:00 - 14:59	Afternoon	37,9	67	21,2	21,2	19,05	563,53	102,31
2019/05/15	CH2206	Male	Three y/o	Treatment (Wk 1)	Treatment	Fast Day	No	14:50	14:00 - 14:59	Afternoon	37,9	75	18,4	18,4	19,05	572,36	97,47
2019/05/15	CH2205	Male	Three y/o	Treatment (Wk 1)	Treatment	Fast Day	No	14:55	14:00 - 14:59	Afternoon	37,9	65	21,6	21,6	19,05	561,12	102,94
2019/05/15	CH2206	Male	Three y/o	Treatment (Wk 1)	Treatment	Fast Day	No	14:55	14:00 - 14:59	Afternoon	37,9	85	9,2	9,2	19,05	581,90	73,94
2019/05/15	CH2205	Male	Three y/o	Treatment (Wk 1)	Treatment	Fast Day	No	15:00	15:00 - 15:59	Afternoon	37,9	73	22,6	22,6	19,05	570,27	104,49
2019/05/15	CH2206	Male	Three y/o	Treatment (Wk 1)	Treatment	Fast Day	No	15:00	15:00 - 15:59	Afternoon	37,9	94	10,0	10,0	19,05	589,37	76,76
2019/05/15	CH2205	Male	Three y/o	Treatment (Wk 1)	Treatment	Fast Day	No	15:05	15:00 - 15:59	Afternoon	37,9	68	18,8	18,8	19,05	564,71	98,20
2019/05/15	CH2206	Male	Three y/o	Treatment (Wk 1)	Treatment	Fast Day	No	15:05	15:00 - 15:59	Afternoon	37,9	92	24,2	24,2	19,05	587,78	106,83
2019/05/15	CH2205	Male	Three y/o	Treatment (Wk 1)	Treatment	Fast Day	No	15:10	15:00 - 15:59	Afternoon	37,9	57	17,4	17,4	19,05	550,47	95,56
2019/05/15	CH2206	Male	Three y/o	Treatment (Wk 1)	Treatment	Fast Day	No	15:10	15:00 - 15:59	Afternoon	37,9	88	20,0	20,0	19,05	584,49	100,31
2019/05/15	CH2205	Male	Three y/o	Treatment (Wk 1)	Treatment	Fast Day	No	15:15	15:00 - 15:59	Afternoon	37,9	62	17,6	17,6	19,05	557,33	95,95
2019/05/15	CH2206	Male	Three y/o	Treatment (Wk 1)	Treatment	Fast Day	No	15:15	15:00 - 15:59	Afternoon	37,9	77	28,0	28,0	19,05	574,39	111,83
2019/05/15	CH2205	Male	Three y/o	Treatment (Wk 1)	Treatment	Fast Day	No	15:20	15:00 - 15:59	Afternoon	37,9	67	15,6	15,6	19,05	563,53	91,85
2019/05/15	CH2206	Male	Three y/o	Treatment (Wk 1)	Treatment	Fast Day	No	15:20	15:00 - 15:59	Afternoon	37,8	61	20,8	20,8	18,95	556,01	101,65
2019/05/15	CH2205	Male	Three y/o	Treatment (Wk 1)	Treatment	Fast Day	No	15:25	15:00 - 15:59	Afternoon	37,9	69	21,2	21,2	19,05	565,86	102,31
2019/05/15	CH2206	Male	Three y/o	Treatment (Wk 1)	Treatment	Fast Day	No	15:25	15:00 - 15:59	Afternoon	37,8	80	16,8	16,8	18,95	577,31	94,37
2019/05/15	CH2205	Male	Three y/o	Treatment (Wk 1)	Treatment	Fast Day	No	15:30	15:00 - 15:59	Afternoon	37,9		22,0	22,0	19,05		103,57
2019/05/15	CH2206	Male	Three y/o	Treatment (Wk 1)	Treatment	Fast Day	No	15:30	15:00 - 15:59	Afternoon	37,8	90	36,8	36,8	18,95	586,16	121,22
2019/05/15	CH2205	Male	Three y/o	Treatment (Wk 1)	Treatment	Fast Day	No	15:35	15:00 - 15:59	Afternoon	37,9	158	30,8	30,8	19,05	625,26	115,10
2019/05/15	CH2206	Male	Three y/o	Treatment (Wk 1)	Treatment	Fast Day	No	15:35	15:00 - 15:59	Afternoon	37,9	134	234,6	234,6	19,05	614,33	185,74
2019/05/15	CH2205	Male	Three y/o	Treatment (Wk 1)	Treatment	Fast Day	No	15:40	15:00 - 15:59	Afternoon	37,9	98	25,2	25,2	19,05	592,41	108,22
2019/05/15	CH2206	Male	Three y/o	Treatment (Wk 1)	Treatment	Fast Day	No	15:40	15:00 - 15:59	Afternoon	37,9		519,4	519,4	19,05		213,90
2019/05/15	CH2205	Male	Three y/o	Treatment (Wk 1)	Treatment	Fast Day	No	15:45	15:00 - 15:59	Afternoon	37,9	53	52,2	52,2	19,05	544,43	133,27
2019/05/15	CH2206	Male	Three y/o	Treatment (Wk 1)	Treatment	Fast Day	No	15:45	15:00 - 15:59	Afternoon	38,0	117	415,6	415,6	19,15	605,02	205,97
2019/05/15	CH2205	Male	Three y/o	Treatment (Wk 1)	Treatment	Fast Day	No	15:50	15:00 - 15:59	Afternoon	37,9	91	23,0	23,0	19,05	586,98	105,09
2019/05/15	CH2206	Male	Three y/o	Treatment (Wk 1)	Treatment	Fast Day	No	15:50	15:00 - 15:59	Afternoon	38,3		36,8	36,8	19,46		121,22
2019/05/15	CH2205	Male	Three y/o	Treatment (Wk 1)	Treatment	Fast Day	No	15:55	15:00 - 15:59	Afternoon	37,9	73	7,0	7,0	19,05	570,27	64,72
2019/05/15	CH2206	Male	Three y/o	Treatment (Wk 1)	Treatment	Fast Day	No	15:55	15:00 - 15:59	Afternoon	38,3	125	40,6	40,6	19,46	609,60	124,60
2019/05/15	CH2205	Male	Three y/o	Treatment (Wk 1)	Treatment	Fast Day	No	16:00	16:00 - 16:59	Evening	37,9		26,6	26,6	19,05		110,07
2019/05/15	CH2206	Male	Three y/o	Treatment (Wk 1)	Treatment	Fast Day	No	16:00	16:00 - 16:59	Evening	38,2		100,2	100,2	19,36		155,91
2019/05/15	CH2205	Male	Three y/o	Treatment (Wk 1)	Treatment	Fast Day	No	16:05	16:00 - 16:59	Evening	37,9	72	12,2	12,2	19,05	569,20	83,49
2019/05/15	CH2206	Male	Three y/o	Treatment (Wk 1)	Treatment	Fast Day	No	16:05	16:00 - 16:59	Evening	38,2	98	39,0	39,0	19,36	592,41	123,22
2019/05/15	CH2205	Male	Three y/o	Treatment (Wk 1)	Treatment	Fast Day	No	16:10	16:00 - 16:59	Evening	37,9	88	9,8	9,8	19,05	584,49	76,07
2019/05/15	CH2206	Male	Three y/o	Treatment (Wk 1)	Treatment	Fast Day	No	16:10	16:00 - 16:59	Evening	38,2	120	75,2	75,2	19,36	606,78	145,93
2019/05/15	CH2205	Male	Three y/o	Treatment (Wk 1)	Treatment	Fast Day	No	16:15	16:00 - 16:59	Evening	37,9	67	10,2	10,2	19,05	563,53	77,43
2019/05/15	CH2206	Male	Three y/o	Treatment (Wk 1)	Treatment	Fast Day	No	16:15	16:00 - 16:59	Evening	38,2	70	38,0	38,0	19,36	566,99	122,32
2019/05/15	CH2205	Male	Three y/o	Treatment (Wk 1)	Treatment	Fast Day	No	16:20	16:00 - 16:59	Evening	38,0	60	11,2	11,2	19,15	554,67	80,59
2019/05/15	CH2206	Male	Three y/o	Treatment (Wk 1)	Treatment	Fast Day	No	16:20	16:00 - 16:59	Evening	38,2		144,2	144,2	19,36		168,64
2019/05/15	CH2205	Male	Three y/o	Treatment (Wk 1)	Treatment	Fast Day	No	16:25	16:00 - 16:59	Evening	38,0	54	6,0	6,0	19,15	545,99	59,53
2019/05/15	CH2206	Male	Three y/o	Treatment (Wk 1)	Treatment	Fast Day	No	16:25	16:00 - 16:59	Evening	38,2	92	46,4	46,4	19,36	587,78	129,21
2019/05/15	CH2205	Male	Three y/o	Treatment (Wk 1)	Treatment	Fast Day	No	16:30	16:00 - 16:59	Evening	38,0	65	6,6	6,6	19,15	561,12	62,73
2019/05/15	CH2206	Male	Three y/o	Treatment (Wk 1)	Treatment	Fast Day	No	16:30	16:00 - 16:59	Evening	38,2	86	31,0	31,0	19,36	582,77	115,32
2019/05/15	CH2205	Male	Three y/o	Treatment (Wk 1)	Treatment	Fast Day	No	16:35	16:00 - 16:59	Evening	38,1	53	3,8	3,8	19,26	544,43	44,22
2019/05/15	CH2206	Male	Three y/o	Treatment (Wk 1)	Treatment	Fast Day	No	16:35	16:00 - 16:59	Evening	38,2	87	32,4	32,4	19,36	583,64	116,84
2019/05/15	CH2205	Male	Three y/o	Treatment (Wk 1)	Treatment	Fast Day	No	16:40	16:00 - 16:59	Evening	38,1	61	8,0	8,0	19,26	556,01	69,22
2019/05/15	CH2206	Male	Three y/o	Treatment (Wk 1)	Treatment	Fast Day	No	16:40	16:00 - 16:59	Evening	38,2	48	34,6	34,6	19,36	536,04	119,10
2019/05/15	CH2205	Male	Three y/o	Treatment (Wk 1)	Treatment	Fast Day	No	16:45	16:00 - 16:59	Evening	38,1	71	1,4	1,4	19,26	568,10	11,07
2019/05/15	CH2206	Male	Three y/o	Treatment (Wk 1)	Treatment	Fast Day	No	16:45	16:00 - 16:59	Evening	38,2	70	44,2	44,2	19,36	566,99	127,53
2019/05/15	CH2205	Male	Three y/o	Treatment (Wk 1)	Treatment	Fast Day	No	16:50	16:00 - 16:59	Evening	38,1	67	5,6	5,6	19,26	563,53	57,21
2019/05/15	CH2206	Male	Three y/o	Treatment (Wk 1)	Treatment	Fast Day	No	16:50	16:00 - 16:59	Evening	38,1	73	49,2	49,2	19,26	570,27	131,23
2019/05/15	CH2205	Male	Three y/o	Treatment (Wk 1)	Treatment	Fast Day	No	16:55	16:00 - 16:59	Evening	38,1	64	11,0	11,0	19,26	559,88	79,98
2019/05/15	CH2206	Male	Three y/o	Treatment (Wk 1)	Treatment	Fast Day	No	16:55	16:00 - 16:59	Evening	38,1	73	39,8	39,8	19,26	570,27	123,91
2019/05/15	CH2205	Male	Three y/o	Treatment (Wk 1)	Treatment	Fast Day	No	17:00	17:00 - 17:59	Evening	38,1	81	6,0	6,0	19,26	578,26	59,53
2019/05/15	CH2206	Male	Three y/o	Treatment (Wk 1)	Treatment	Fast Day	No	17:00	17:00 - 17:59	Evening	38,1	86	18,6	18,6	19,26	582,77	97,84
2019/05/15	CH2205	Male	Three y/o	Treatment (Wk 1)	Treatment	Fast Day	No	17:05	17:00 - 17:59	Evening	38,1	52	7,2	7,2	19,26	542,83	65,66
2019/05/15	CH2206	Male	Three y/o	Treatment (Wk 1)	Treatment	Fast Day	No	17:05	17:00 - 17:59	Evening	38,0	76	58,0	58,0	19,15	573,39	136,92
2019/05/15	CH2205	Male	Three y/o	Treatment (Wk 1)	Treatment	Fast Day	No	17:10	17:00 - 17:59	Evening	38,1	148	11,6	11,6	19,26	620,97	81,78
2019/05/15	CH2206	Male	Three y/o	Treatment (Wk 1)	Treatment	Fast Day	No	17:10	17:00 - 17:59	Evening	38,1	97	65,4	65,4	19,26	591,66	141,08
2019/05/15	CH2205	Male	Three y/o	Treatment (Wk 1)	Treatment	Fast Day	No	17:15	17:00 - 17:59	Evening	38,1	76	10,8	10,8	19,26	573,39	79,36
2019/05/15	CH2206	Male	Three y/o	Treatment (Wk 1)	Treatment	Fast Day	No	17:15	17:00 - 17:59	Evening	38,0	105	56,4	56,4	19,15	597,38	135,95
2019/05/15	CH2205	Male	Three y/o	Treatment (Wk 1)	Treatment	Fast Day	No	17:20	17:00 - 17:59	Evening	38,1		5,2	5,2	19,26		54,72

2019/05/15	CH2206	Male	Three y/o	Treatment (Wk 1)	Treatment	Fast Day	No	17:20	17:00 - 17:59	Evening	38,1	86	58,0	58,0	19,26	582,77	136,92
2019/05/15	CH2205	Male	Three y/o	Treatment (Wk 1)	Treatment	Fast Day	No	17:25	17:00 - 17:59	Evening	38,1		6,6	6,6	19,26		62,73
2019/05/15	CH2206	Male	Three y/o	Treatment (Wk 1)	Treatment	Fast Day	No	17:25	17:00 - 17:59	Evening	38,0	98	55,4	55,4	19,15	592,41	135,33
2019/05/15	CH2205	Male	Three y/o	Treatment (Wk 1)	Treatment	Fast Day	No	17:30	17:00 - 17:59	Evening	38,1	84	7,8	7,8	19,26	581,01	68,36
2019/05/15	CH2206	Male	Three y/o	Treatment (Wk 1)	Treatment	Fast Day	No	17:30	17:00 - 17:59	Evening	38,1	188	44,8	44,8	19,26	636,34	127,99
2019/05/15	CH2205	Male	Three y/o	Treatment (Wk 1)	Treatment	Fast Day	No	17:35	17:00 - 17:59	Evening	38,1	75	5,6	5,6	19,26	572,36	57,21
2019/05/15	CH2206	Male	Three y/o	Treatment (Wk 1)	Treatment	Fast Day	No	17:35	17:00 - 17:59	Evening	38,0	85	57,2	57,2	19,15	581,90	136,44
2019/05/15	CH2205	Male	Three y/o	Treatment (Wk 1)	Treatment	Fast Day	No	17:40	17:00 - 17:59	Evening	38,1		8,8	8,8	19,26		72,43
2019/05/15	CH2206	Male	Three y/o	Treatment (Wk 1)	Treatment	Fast Day	No	17:40	17:00 - 17:59	Evening	38,0	65	60,6	60,6	19,15	561,12	138,44
2019/05/15	CH2205	Male	Three y/o	Treatment (Wk 1)	Treatment	Fast Day	No	17:45	17:00 - 17:59	Evening	38,0		11,6	11,6	19,15		81,78
2019/05/15	CH2206	Male	Three y/o	Treatment (Wk 1)	Treatment	Fast Day	No	17:45	17:00 - 17:59	Evening	38,0	60	60,6	60,6	19,15	554,67	138,44
2019/05/15	CH2205	Male	Three y/o	Treatment (Wk 1)	Treatment	Fast Day	No	17:50	17:00 - 17:59	Evening	37,9		51,0	51,0	19,05		132,47
2019/05/15	CH2206	Male	Three y/o	Treatment (Wk 1)	Treatment	Fast Day	No	17:50	17:00 - 17:59	Evening	38,0	59	63,0	63,0	19,15	553,30	139,78
2019/05/15	CH2205	Male	Three y/o	Treatment (Wk 1)	Treatment	Fast Day	No	17:55	17:00 - 17:59	Evening	37,9	94	27,6	27,6	19,05	589,37	111,33
2019/05/15	CH2206	Male	Three y/o	Treatment (Wk 1)	Treatment	Fast Day	No	17:55	17:00 - 17:59	Evening	38,0	95	33,0	33,0	19,15	590,14	117,47
2019/05/15	CH2205	Male	Three y/o	Treatment (Wk 1)	Treatment	Fast Day	No	18:00	18:00 - 18:59	Evening	37,7		26,0	26,0	18,85		109,29
2019/05/15	CH2206	Male	Three y/o	Treatment (Wk 1)	Treatment	Fast Day	No	18:00	18:00 - 18:59	Evening	38,0	99	37,6	37,6	19,15	593,14	121,96
2019/05/15	CH2205	Male	Three y/o	Treatment (Wk 1)	Treatment	Fast Day	No	18:05	18:00 - 18:59	Evening	37,6		25,4	25,4	18,75		108,49
2019/05/15	CH2206	Male	Three y/o	Treatment (Wk 1)	Treatment	Fast Day	No	18:05	18:00 - 18:59	Evening	37,9		45,2	45,2	19,05		128,30
2019/05/15	CH2205	Male	Three y/o	Treatment (Wk 1)	Treatment	Fast Day	No	18:10	18:00 - 18:59	Evening	37,7	82	28,6	28,6	18,85	579,19	112,56
2019/05/15	CH2206	Male	Three y/o	Treatment (Wk 1)	Treatment	Fast Day	No	18:10	18:00 - 18:59	Evening	37,9	55	15,4	15,4	19,05	547,52	91,41
2019/05/15	CH2205	Male	Three y/o	Treatment (Wk 1)	Treatment	Fast Day	No	18:15	18:00 - 18:59	Evening	37,9	79	26,0	26,0	19,05	576,36	109,29
2019/05/15	CH2206	Male	Three y/o	Treatment (Wk 1)	Treatment	Fast Day	No	18:15	18:00 - 18:59	Evening	37,7	94	23,4	23,4	18,85	589,37	105,68
2019/05/15	CH2205	Male	Three y/o	Treatment (Wk 1)	Treatment	Fast Day	No	18:20	18:00 - 18:59	Evening	37,9	61	30,0	30,0	19,05	556,01	114,20
2019/05/15	CH2206	Male	Three y/o	Treatment (Wk 1)	Treatment	Fast Day	No	18:20	18:00 - 18:59	Evening	37,7	94	31,2	31,2	18,85	589,37	115,54
2019/05/15	CH2205	Male	Three y/o	Treatment (Wk 1)	Treatment	Fast Day	No	18:25	18:00 - 18:59	Evening	38,0	57	25,6	25,6	19,15	550,47	108,76
2019/05/15	CH2206	Male	Three y/o	Treatment (Wk 1)	Treatment	Fast Day	No	18:25	18:00 - 18:59	Evening	37,5	48	29,4	29,4	18,65	536,04	113,50
2019/05/15	CH2205	Male	Three y/o	Treatment (Wk 1)	Treatment	Fast Day	No	18:30	18:00 - 18:59	Evening	38,0	49	27,4	27,4	19,15	537,80	111,09
2019/05/15	CH2206	Male	Three y/o	Treatment (Wk 1)	Treatment	Fast Day	No	18:30	18:00 - 18:59	Evening	37,3	83	24,6	24,6	18,46	580,11	107,39
2019/05/15	CH2205	Male	Three y/o	Treatment (Wk 1)	Treatment	Fast Day	No	18:35	18:00 - 18:59	Evening	38,0	92	28,2	28,2	19,15	587,78	112,07
2019/05/15	CH2206	Male	Three y/o	Treatment (Wk 1)	Treatment	Fast Day	No	18:35	18:00 - 18:59	Evening	37,2	90	26,4	26,4	18,36	586,16	109,81
2019/05/15	CH2205	Male	Three y/o	Treatment (Wk 1)	Treatment	Fast Day	No	18:40	18:00 - 18:59	Evening	38,1	84	26,0	26,0	19,26	581,01	109,29
2019/05/15	CH2206	Male	Three y/o	Treatment (Wk 1)	Treatment	Fast Day	No	18:40	18:00 - 18:59	Evening	37,2	83	35,2	35,2	18,36	580,11	119,69
2019/05/15	CH2205	Male	Three y/o	Treatment (Wk 1)	Treatment	Fast Day	No	18:45	18:00 - 18:59	Evening	38,1	52	23,6	23,6	19,26	542,83	105,97
2019/05/15	CH2206	Male	Three y/o	Treatment (Wk 1)	Treatment	Fast Day	No	18:45	18:00 - 18:59	Evening	37,5	84	39,2	39,2	18,65	581,01	123,39
2019/05/15	CH2205	Male	Three y/o	Treatment (Wk 1)	Treatment	Fast Day	No	18:50	18:00 - 18:59	Evening	38,1	53	24,0	24,0	19,26	544,43	106,55
2019/05/15	CH2206	Male	Three y/o	Treatment (Wk 1)	Treatment	Fast Day	No	18:50	18:00 - 18:59	Evening	37,7	47	40,4	40,4	18,85	534,23	124,43
2019/05/15	CH2205	Male	Three y/o	Treatment (Wk 1)	Treatment	Fast Day	No	18:55	18:00 - 18:59	Evening	38,0	62	18,8	18,8	19,15	557,33	98,20
2019/05/15	CH2206	Male	Three y/o	Treatment (Wk 1)	Treatment	Fast Day	No	18:55	18:00 - 18:59	Evening	37,7	145	38,2	38,2	18,85	619,62	122,50
2019/05/15	CH2205	Male	Three y/o	Treatment (Wk 1)	Treatment	Fast Day	No	19:00	19:00 - 19:59	Evening	38,0	51	21,2	21,2	19,15	541,20	102,31
2019/05/15	CH2206	Male	Three y/o	Treatment (Wk 1)	Treatment	Fast Day	No	19:00	19:00 - 19:59	Evening	37,8	49	33,2	33,2	18,95	537,80	117,68
2019/05/15	CH2205	Male	Three y/o	Treatment (Wk 1)	Treatment	Fast Day	No	19:05	19:00 - 19:59	Evening	37,9	53	18,4	18,4	19,05	544,43	97,47
2019/05/15	CH2206	Male	Three y/o	Treatment (Wk 1)	Treatment	Fast Day	No	19:05	19:00 - 19:59	Evening	37,8	92	35,2	35,2	18,95	587,78	119,69
2019/05/15	CH2205	Male	Three y/o	Treatment (Wk 1)	Treatment	Fast Day	No	19:10	19:00 - 19:59	Evening	37,9	73	19,8	19,8	19,05	570,27	99,97
2019/05/15	CH2206	Male	Three y/o	Treatment (Wk 1)	Treatment	Fast Day	No	19:10	19:00 - 19:59	Evening	37,8	83	35,2	35,2	18,95	580,11	119,69
2019/05/15	CH2205	Male	Three y/o	Treatment (Wk 1)	Treatment	Fast Day	No	19:15	19:00 - 19:59	Evening	38,0	66	22,0	22,0	19,15	562,34	103,57
2019/05/15	CH2206	Male	Three y/o	Treatment (Wk 1)	Treatment	Fast Day	No	19:15	19:00 - 19:59	Evening	37,8	53	32,4	32,4	18,95	544,43	116,84
2019/05/15	CH2205	Male	Three y/o	Treatment (Wk 1)	Treatment	Fast Day	No	19:20	19:00 - 19:59	Evening	38,0	56	19,2	19,2	19,15	549,01	98,92
2019/05/15	CH2206	Male	Three y/o	Treatment (Wk 1)	Treatment	Fast Day	No	19:20	19:00 - 19:59	Evening	37,8	90	37,0	37,0	18,95	586,16	121,40
2019/05/15	CH2205	Male	Three y/o	Treatment (Wk 1)	Treatment	Fast Day	No	19:25	19:00 - 19:59	Evening	38,0	47	23,2	23,2	19,15	534,23	105,39
2019/05/15	CH2206	Male	Three y/o	Treatment (Wk 1)	Treatment	Fast Day	No	19:25	19:00 - 19:59	Evening	37,8	75	36,4	36,4	18,95	572,36	120,84
2019/05/15	CH2205	Male	Three y/o	Treatment (Wk 1)	Treatment	Fast Day	No	19:30	19:00 - 19:59	Evening	38,0	68	23,6	23,6	19,15	564,71	105,97
2019/05/15	CH2206	Male	Three y/o	Treatment (Wk 1)	Treatment	Fast Day	No	19:30	19:00 - 19:59	Evening	37,8	67	24,2	24,2	18,95	563,53	106,83
2019/05/15	CH2205	Male	Three y/o	Treatment (Wk 1)	Treatment	Fast Day	No	19:35	19:00 - 19:59	Evening	38,0	50	21,2	21,2	19,15	539,52	102,31
2019/05/15	CH2206	Male	Three y/o	Treatment (Wk 1)	Treatment	Fast Day	No	19:35	19:00 - 19:59	Evening	37,9	51	24,8	24,8	19,05	541,20	107,67
2019/05/15	CH2205	Male	Three y/o	Treatment (Wk 1)	Treatment	Fast Day	No	19:40	19:00 - 19:59	Evening	38,0	73	18,0	18,0	19,15	570,27	96,72
2019/05/15	CH2206	Male	Three y/o	Treatment (Wk 1)	Treatment	Fast Day	No	19:40	19:00 - 19:59	Evening	37,9	45	42,0	42,0	19,05	530,47	125,77
2019/05/15	CH2205	Male	Three y/o	Treatment (Wk 1)	Treatment	Fast Day	No	19:45	19:00 - 19:59	Evening	38,0	54	25,2	25,2	19,15	545,99	108,22
2019/05/15	CH2206	Male	Three y/o	Treatment (Wk 1)	Treatment	Fast Day	No	19:45	19:00 - 19:59	Evening	37,9	48	35,6	35,6	19,05	536,04	120,08
2019/05/15	CH2205	Male	Three y/o	Treatment (Wk 1)	Treatment	Fast Day	No	19:50	19:00 - 19:59	Evening	38,0	44	19,6	19,6	19,15	528,51	99,63
2019/05/15	CH2206	Male	Three y/o	Treatment (Wk 1)	Treatment	Fast Day	No	19:50	19:00 - 19:59	Evening	37,9	61	36,6	36,6	19,05	556,01	121,03
2019/05/15	CH2205	Male	Three y/o	Treatment (Wk 1)	Treatment	Fast Day	No	19:55	19:00 - 19:59	Evening	38,0	90	23,2	23,2	19,15	586,16	105,39
2019/05/15	CH2206	Male	Three y/o	Treatment (Wk 1)	Treatment	Fast Day	No	19:55	19:00 - 19:59	Evening	37,9	43	29,8	29,8	19,05	526,50	113,97
2019/05/15	CH2205	Male	Three y/o	Treatment (Wk 1)	Treatment	Fast Day	No	20:00	20:00 - 20:59	Night	38,0	57	24,2	24,2	19,15	550,47	106,83
2019/05/15	CH2206	Male	Three y/o	Treatment (Wk 1)	Treatment	Fast Day	No	20:00	20:00 - 20:59	Night	37,9	84	27,0	27,0	19,05	581,01	110,58
2019/05/15	CH2205	Male	Three y/o	Treatment (Wk 1)	Treatment	Fast Day	No	20:05	20:00 - 20:59	Night	38,0	53	24,4	24,4	19,15	544,43	107,11
2019/05/15	CH2206	Male	Three y/o	Treatment (Wk 1)	Treatment	Fast Day	No	20:05	20:00 - 20:59	Night	37,9	93	26,6	26,6	19,05	588,58	110,07
2019/05/15	CH2205	Male	Three y/o	Treatment (Wk 1)	Treatment	Fast Day	No	20:10	20:00 - 20:59	Night	38,0	84	24,0	24,0	19,15	581,01	106,55
2019/05/15	CH2206	Male	Three y/o	Treatment (Wk 1)	Treatment	Fast Day	No	20:10	20:00 - 20:59	Night	37,9	47	3,8	3,8	19,05	534,23	44,22
2019/05/15	CH2205	Male	Three y/o	Treatment (Wk 1)	Treatment	Fast Day	No	20:15	20:00 - 20:59	Night	38,0	44	24,2	24,2	19,15	528,51	106,83

2019/05/15	CH2206	Male	Three y/o	Treatment (Wk 1)	Treatment	Fast Day	No	20:15	20:00 - 20:59	Night	37,9	81	8,2	8,2	19,05	578,26	70,05
2019/05/15	CH2205	Male	Three y/o	Treatment (Wk 1)	Treatment	Fast Day	No	20:20	20:00 - 20:59	Night	38,0	96	20,4	20,4	19,15	590,91	100,99
2019/05/15	CH2206	Male	Three y/o	Treatment (Wk 1)	Treatment	Fast Day	No	20:20	20:00 - 20:59	Night	37,9	77	3,4	3,4	19,05	574,39	40,51
2019/05/15	CH2205	Male	Three y/o	Treatment (Wk 1)	Treatment	Fast Day	No	20:25	20:00 - 20:59	Night	38,0	89	21,2	21,2	19,15	585,33	102,31
2019/05/15	CH2206	Male	Three y/o	Treatment (Wk 1)	Treatment	Fast Day	No	20:25	20:00 - 20:59	Night	37,9	89	6,2	6,2	19,05	585,33	60,63
2019/05/15	CH2205	Male	Three y/o	Treatment (Wk 1)	Treatment	Fast Day	No	20:30	20:00 - 20:59	Night	38,0	51	22,4	22,4	19,15	541,20	104,19
2019/05/15	CH2206	Male	Three y/o	Treatment (Wk 1)	Treatment	Fast Day	No	20:30	20:00 - 20:59	Night	37,9	85	9,8	9,8	19,05	581,90	76,07
2019/05/15	CH2205	Male	Three y/o	Treatment (Wk 1)	Treatment	Fast Day	No	20:35	20:00 - 20:59	Night	38,0	77	21,4	21,4	19,15	574,39	102,63
2019/05/15	CH2206	Male	Three y/o	Treatment (Wk 1)	Treatment	Fast Day	No	20:35	20:00 - 20:59	Night	37,9	58	18,4	18,4	19,05	551,90	97,47
2019/05/15	CH2205	Male	Three y/o	Treatment (Wk 1)	Treatment	Fast Day	No	20:40	20:00 - 20:59	Night	37,9	62	21,0	21,0	19,05	557,33	101,98
2019/05/15	CH2206	Male	Three y/o	Treatment (Wk 1)	Treatment	Fast Day	No	20:40	20:00 - 20:59	Night	37,9	45	24,0	24,0	19,05	530,47	106,55
2019/05/15	CH2205	Male	Three y/o	Treatment (Wk 1)	Treatment	Fast Day	No	20:45	20:00 - 20:59	Night	37,9	60	24,0	24,0	19,05	554,67	106,55
2019/05/15	CH2206	Male	Three y/o	Treatment (Wk 1)	Treatment	Fast Day	No	20:45	20:00 - 20:59	Night	37,9	63	19,8	19,8	19,05	558,62	99,97
2019/05/15	CH2205	Male	Three y/o	Treatment (Wk 1)	Treatment	Fast Day	No	20:50	20:00 - 20:59	Night	37,9	119	20,6	20,6	19,05	606,20	101,32
2019/05/15	CH2206	Male	Three y/o	Treatment (Wk 1)	Treatment	Fast Day	No	20:50	20:00 - 20:59	Night	37,9	70	6,4	6,4	19,05	566,99	61,70
2019/05/15	CH2205	Male	Three y/o	Treatment (Wk 1)	Treatment	Fast Day	No	20:55	20:00 - 20:59	Night	37,9	54	17,6	17,6	19,05	545,99	95,95
2019/05/15	CH2206	Male	Three y/o	Treatment (Wk 1)	Treatment	Fast Day	No	20:55	20:00 - 20:59	Night	37,8	57	3,4	3,4	18,95	550,47	40,51
2019/05/15	CH2205	Male	Three y/o	Treatment (Wk 1)	Treatment	Fast Day	No	21:00	21:00 - 21:59	Night	37,9	43	15,4	15,4	19,05	526,50	91,41
2019/05/15	CH2206	Male	Three y/o	Treatment (Wk 1)	Treatment	Fast Day	No	21:00	21:00 - 21:59	Night	37,7	58	8,2	8,2	18,85	551,90	70,05
2019/05/15	CH2205	Male	Three y/o	Treatment (Wk 1)	Treatment	Fast Day	No	21:05	21:00 - 21:59	Night	37,7	92	19,6	19,6	18,85	587,78	99,63
2019/05/15	CH2206	Male	Three y/o	Treatment (Wk 1)	Treatment	Fast Day	No	21:05	21:00 - 21:59	Night	37,5	86	14,8	14,8	18,65	582,77	90,05
2019/05/15	CH2205	Male	Three y/o	Treatment (Wk 1)	Treatment	Fast Day	No	21:10	21:00 - 21:59	Night	37,7	54	19,0	19,0	18,85	545,99	98,56
2019/05/15	CH2206	Male	Three y/o	Treatment (Wk 1)	Treatment	Fast Day	No	21:10	21:00 - 21:59	Night	37,7	108	34,4	34,4	18,85	599,39	118,90
2019/05/15	CH2205	Male	Three y/o	Treatment (Wk 1)	Treatment	Fast Day	No	21:15	21:00 - 21:59	Night	37,7	83	17,2	17,2	18,85	580,11	95,17
2019/05/15	CH2206	Male	Three y/o	Treatment (Wk 1)	Treatment	Fast Day	No	21:15	21:00 - 21:59	Night	37,7	50	20,4	20,4	18,85	539,52	100,99
2019/05/15	CH2205	Male	Three y/o	Treatment (Wk 1)	Treatment	Fast Day	No	21:20	21:00 - 21:59	Night	37,7	61	21,2	21,2	18,85	556,01	102,31
2019/05/15	CH2206	Male	Three y/o	Treatment (Wk 1)	Treatment	Fast Day	No	21:20	21:00 - 21:59	Night	37,8	82	11,8	11,8	18,95	579,19	82,36
2019/05/15	CH2205	Male	Three y/o	Treatment (Wk 1)	Treatment	Fast Day	No	21:25	21:00 - 21:59	Night	37,6	93	13,8	13,8	18,75	588,58	87,68
2019/05/15	CH2206	Male	Three y/o	Treatment (Wk 1)	Treatment	Fast Day	No	21:25	21:00 - 21:59	Night	37,8	94	42,4	42,4	18,95	589,37	126,10
2019/05/15	CH2205	Male	Three y/o	Treatment (Wk 1)	Treatment	Fast Day	No	21:30	21:00 - 21:59	Night	37,6	68	8,6	8,6	18,75	564,71	71,66
2019/05/15	CH2206	Male	Three y/o	Treatment (Wk 1)	Treatment	Fast Day	No	21:30	21:00 - 21:59	Night	37,8	115	23,8	23,8	18,95	603,82	106,26
2019/05/15	CH2205	Male	Three y/o	Treatment (Wk 1)	Treatment	Fast Day	No	21:35	21:00 - 21:59	Night	37,6	50	7,0	7,0	18,75	539,52	64,72
2019/05/15	CH2206	Male	Three y/o	Treatment (Wk 1)	Treatment	Fast Day	No	21:35	21:00 - 21:59	Night	37,4	64	22,2	22,2	18,55	559,88	103,88
2019/05/15	CH2205	Male	Three y/o	Treatment (Wk 1)	Treatment	Fast Day	No	21:40	21:00 - 21:59	Night	37,6	83	2,6	2,6	18,75	580,11	31,57
2019/05/15	CH2206	Male	Three y/o	Treatment (Wk 1)	Treatment	Fast Day	No	21:40	21:00 - 21:59	Night	37,0	50	18,4	18,4	18,16	539,52	97,47
2019/05/15	CH2205	Male	Three y/o	Treatment (Wk 1)	Treatment	Fast Day	No	21:45	21:00 - 21:59	Night	37,7	51	3,6	3,6	18,85	541,20	42,42
2019/05/15	CH2206	Male	Three y/o	Treatment (Wk 1)	Treatment	Fast Day	No	21:45	21:00 - 21:59	Night	36,6	88	42,6	42,6	17,77	584,49	126,26
2019/05/15	CH2205	Male	Three y/o	Treatment (Wk 1)	Treatment	Fast Day	No	21:50	21:00 - 21:59	Night	37,7	60	14,4	14,4	18,85	554,67	89,12
2019/05/15	CH2206	Male	Three y/o	Treatment (Wk 1)	Treatment	Fast Day	No	21:50	21:00 - 21:59	Night	36,4	68	48,4	48,4	17,57	564,71	130,66
2019/05/15	CH2205	Male	Three y/o	Treatment (Wk 1)	Treatment	Fast Day	No	21:55	21:00 - 21:59	Night	37,7	86	17,0	17,0	18,85	582,77	94,77
2019/05/15	CH2206	Male	Three y/o	Treatment (Wk 1)	Treatment	Fast Day	No	21:55	21:00 - 21:59	Night	36,4	73	48,6	48,6	17,57	570,27	130,81
2019/05/15	CH2205	Male	Three y/o	Treatment (Wk 1)	Treatment	Fast Day	No	22:00	22:00 - 22:59	Night	37,8	60	16,6	16,6	18,95	554,67	93,96
2019/05/15	CH2206	Male	Three y/o	Treatment (Wk 1)	Treatment	Fast Day	No	22:00	22:00 - 22:59	Night	36,5	63	47,0	47,0	17,67	558,62	129,65
2019/05/15	CH2205	Male	Three y/o	Treatment (Wk 1)	Treatment	Fast Day	No	22:05	22:00 - 22:59	Night	37,9	56	17,6	17,6	19,05	549,01	95,95
2019/05/15	CH2206	Male	Three y/o	Treatment (Wk 1)	Treatment	Fast Day	No	22:05	22:00 - 22:59	Night	36,6	69	43,6	43,6	17,77	565,86	127,06
2019/05/15	CH2205	Male	Three y/o	Treatment (Wk 1)	Treatment	Fast Day	No	22:10	22:00 - 22:59	Night	37,9	88	20,4	20,4	19,05	584,49	100,99
2019/05/15	CH2206	Male	Three y/o	Treatment (Wk 1)	Treatment	Fast Day	No	22:10	22:00 - 22:59	Night	36,8	49	58,2	58,2	17,96	537,80	137,04
2019/05/15	CH2205	Male	Three y/o	Treatment (Wk 1)	Treatment	Fast Day	No	22:15	22:00 - 22:59	Night	37,9	60	17,8	17,8	19,05	554,67	96,34
2019/05/15	CH2206	Male	Three y/o	Treatment (Wk 1)	Treatment	Fast Day	No	22:15	22:00 - 22:59	Night	36,9	70	62,0	62,0	18,06	566,99	139,23
2019/05/15	CH2205	Male	Three y/o	Treatment (Wk 1)	Treatment	Fast Day	No	22:20	22:00 - 22:59	Night	37,9	60	19,4	19,4	19,05	554,67	99,28
2019/05/15	CH2206	Male	Three y/o	Treatment (Wk 1)	Treatment	Fast Day	No	22:20	22:00 - 22:59	Night	37,0	58	63,8	63,8	18,16	551,90	140,22
2019/05/15	CH2205	Male	Three y/o	Treatment (Wk 1)	Treatment	Fast Day	No	22:25	22:00 - 22:59	Night	37,9	85	19,4	19,4	19,05	581,90	99,28
2019/05/15	CH2206	Male	Three y/o	Treatment (Wk 1)	Treatment	Fast Day	No	22:25	22:00 - 22:59	Night	37,1	93	54,6	54,6	18,26	588,58	134,83
2019/05/15	CH2205	Male	Three y/o	Treatment (Wk 1)	Treatment	Fast Day	No	22:30	22:00 - 22:59	Night	37,9	61	19,2	19,2	19,05	556,01	98,92
2019/05/15	CH2206	Male	Three y/o	Treatment (Wk 1)	Treatment	Fast Day	No	22:30	22:00 - 22:59	Night	37,2	90	50,2	50,2	18,36	586,16	131,92
2019/05/15	CH2205	Male	Three y/o	Treatment (Wk 1)	Treatment	Fast Day	No	22:35	22:00 - 22:59	Night	37,7	59	15,6	15,6	18,85	553,30	91,85
2019/05/15	CH2206	Male	Three y/o	Treatment (Wk 1)	Treatment	Fast Day	No	22:35	22:00 - 22:59	Night	37,3	40	49,0	49,0	18,46	520,09	131,09
2019/05/15	CH2205	Male	Three y/o	Treatment (Wk 1)	Treatment	Fast Day	No	22:40	22:00 - 22:59	Night	37,7		20,0	20,0	18,85		100,31
2019/05/15	CH2206	Male	Three y/o	Treatment (Wk 1)	Treatment	Fast Day	No	22:40	22:00 - 22:59	Night	37,4	91	40,2	40,2	18,55	586,98	124,26
2019/05/15	CH2205	Male	Three y/o	Treatment (Wk 1)	Treatment	Fast Day	No	22:45	22:00 - 22:59	Night	37,7	90	15,6	15,6	18,85	586,16	91,85
2019/05/15	CH2206	Male	Three y/o	Treatment (Wk 1)	Treatment	Fast Day	No	22:45	22:00 - 22:59	Night	37,5	51	45,2	45,2	18,65	541,20	128,30
2019/05/15	CH2205	Male	Three y/o	Treatment (Wk 1)	Treatment	Fast Day	No	22:50	22:00 - 22:59	Night	37,7		19,8	19,8	18,85		99,97
2019/05/15	CH2206	Male	Three y/o	Treatment (Wk 1)	Treatment	Fast Day	No	22:50	22:00 - 22:59	Night	37,5	41	49,4	49,4	18,65	522,29	131,37
2019/05/15	CH2205	Male	Three y/o	Treatment (Wk 1)	Treatment	Fast Day	No	22:55	22:00 - 22:59	Night	37,7		12,8	12,8	18,85		85,12
2019/05/15	CH2206	Male	Three y/o	Treatment (Wk 1)	Treatment	Fast Day	No	22:55	22:00 - 22:59	Night	37,5	57	47,2	47,2	18,65	550,47	129,80
2019/05/15	CH2205	Male	Three y/o	Treatment (Wk 1)	Treatment	Fast Day	No	23:00	23:00 - 23:59	Night	37,7		15,6	15,6	18,85		91,85
2019/05/15	CH2206	Male	Three y/o	Treatment (Wk 1)	Treatment	Fast Day	No	23:00	23:00 - 23:59	Night	37,7	82	47,0	47,0	18,85	579,19	129,65
2019/05/15	CH2205	Male	Three y/o	Treatment (Wk 1)	Treatment	Fast Day	No	23:05	23:00 - 23:59	Night	37,7	46	16,0	16,0	18,85	532,38	92,71
2019/05/15	CH2206	Male	Three y/o	Treatment (Wk 1)	Treatment	Fast Day	No	23:05	23:00 - 23:59	Night	37,7	46	39,4	39,4	18,85	532,38	123,57
2019/05/15	CH2205	Male	Three y/o	Treatment (Wk 1)	Treatment	Fast Day	No	23:10	23:00 - 23:59	Night	37,7		17,4	17,4	18,85		95,56

	2019/05/15	CH2206	Male	Three y/o	Treatment (Wk 1)	Treatment	Fast Day	No	23:10	23:00 - 23:59	Night	37,7	98	33,4	33,4	18,85	592,41	117,88
	2019/05/15	CH2205	Male	Three y/o	Treatment (Wk 1)	Treatment	Fast Day	No	23:15	23:00 - 23:59	Night	37,7		8,6	8,6	18,85		71,66
	2019/05/15	CH2206	Male	Three y/o	Treatment (Wk 1)	Treatment	Fast Day	No	23:15	23:00 - 23:59	Night	37,7	81	25,4	25,4	18,85	578,26	108,49
	2019/05/15	CH2205	Male	Three y/o	Treatment (Wk 1)	Treatment	Fast Day	No	23:20	23:00 - 23:59	Night	37,7	79	7,8	7,8	18,85	576,36	68,36
	2019/05/15	CH2206	Male	Three y/o	Treatment (Wk 1)	Treatment	Fast Day	No	23:20	23:00 - 23:59	Night	37,7	93	24,0	24,0	18,85	588,58	106,55
	2019/05/15	CH2205	Male	Three y/o	Treatment (Wk 1)	Treatment	Fast Day	No	23:25	23:00 - 23:59	Night	37,7	89	10,4	10,4	18,85	585,33	78,08
	2019/05/15	CH2206	Male	Three y/o	Treatment (Wk 1)	Treatment	Fast Day	No	23:25	23:00 - 23:59	Night	37,7	67	36,0	36,0	18,85	563,53	120,46
	2019/05/15	CH2205	Male	Three y/o	Treatment (Wk 1)	Treatment	Fast Day	No	23:30	23:00 - 23:59	Night	37,7	96	20,4	20,4	18,85	590,91	100,99
	2019/05/15	CH2206	Male	Three y/o	Treatment (Wk 1)	Treatment	Fast Day	No	23:30	23:00 - 23:59	Night	37,5	47	17,8	17,8	18,65	534,23	96,34
	2019/05/15	CH2205	Male	Three y/o	Treatment (Wk 1)	Treatment	Fast Day	No	23:35	23:00 - 23:59	Night	37,9	61	17,4	17,4	19,05	556,01	95,56
	2019/05/15	CH2206	Male	Three y/o	Treatment (Wk 1)	Treatment	Fast Day	No	23:35	23:00 - 23:59	Night	37,7	67	19,4	19,4	18,85	563,53	99,28
	2019/05/15	CH2205	Male	Three y/o	Treatment (Wk 1)	Treatment	Fast Day	No	23:40	23:00 - 23:59	Night	37,9	58	18,6	18,6	19,05	551,90	97,84
	2019/05/15	CH2206	Male	Three y/o	Treatment (Wk 1)	Treatment	Fast Day	No	23:40	23:00 - 23:59	Night	37,7	57	30,0	30,0	18,85	550,47	114,20
	2019/05/15	CH2205	Male	Three y/o	Treatment (Wk 1)	Treatment	Fast Day	No	23:45	23:00 - 23:59	Night	37,9	81	23,0	23,0	19,05	578,26	105,09
	2019/05/15	CH2206	Male	Three y/o	Treatment (Wk 1)	Treatment	Fast Day	No	23:45	23:00 - 23:59	Night	37,5	86	28,8	28,8	18,65	582,77	112,79
	2019/05/15	CH2205	Male	Three y/o	Treatment (Wk 1)	Treatment	Fast Day	No	23:50	23:00 - 23:59	Night	37,9	95	20,2	20,2	19,05	590,14	100,65
	2019/05/15	CH2206	Male	Three y/o	Treatment (Wk 1)	Treatment	Fast Day	No	23:50	23:00 - 23:59	Night	37,5	58	23,8	23,8	18,65	551,90	106,26
	2019/05/15	CH2205	Male	Three y/o	Treatment (Wk 1)	Treatment	Fast Day	No	23:55	23:00 - 23:59	Night	37,9		14,8	14,8	19,05		90,05
	2019/05/15	CH2206	Male	Three y/o	Treatment (Wk 1)	Treatment	Fast Day	No	23:55	23:00 - 23:59	Night	37,4	90	29,0	29,0	18,55	586,16	113,03
2	2019/05/16	CH2205	Male	Three y/o	Treatment (Wk 1)	Treatment	Feed Day	No	00:00	00:00 - 00:59	Early Morning	37,9	114	20,2	20,2	19,05	603,20	100,65
	2019/05/16	CH2206	Male	Three y/o	Treatment (Wk 1)	Treatment	Feed Day	No	00:00	00:00 - 00:59	Early Morning	37,4	48	32,0	32,0	18,55	536,04	116,41
	2019/05/16	CH2205	Male	Three y/o	Treatment (Wk 1)	Treatment	Feed Day	No	00:05	00:00 - 00:59	Early Morning	37,7		17,6	17,6	18,85		95,95
	2019/05/16	CH2206	Male	Three y/o	Treatment (Wk 1)	Treatment	Feed Day	No	00:05	00:00 - 00:59	Early Morning	37,4	66	35,4	35,4	18,55	562,34	119,88
	2019/05/16	CH2205	Male	Three y/o	Treatment (Wk 1)	Treatment	Feed Day	No	00:10	00:00 - 00:59	Early Morning	37,7	58	26,2	26,2	18,85	551,90	109,55
	2019/05/16	CH2206	Male	Three y/o	Treatment (Wk 1)	Treatment	Feed Day	No	00:10	00:00 - 00:59	Early Morning	37,3	47	31,8	31,8	18,46	534,23	116,20
	2019/05/16	CH2205	Male	Three y/o	Treatment (Wk 1)	Treatment	Feed Day	No	00:15	00:00 - 00:59	Early Morning	37,7	56	26,4	26,4	18,85	549,01	109,81
	2019/05/16	CH2206	Male	Three y/o	Treatment (Wk 1)	Treatment	Feed Day	No	00:15	00:00 - 00:59	Early Morning	37,3	69	39,0	39,0	18,46	565,86	123,22
	2019/05/16	CH2205	Male	Three y/o	Treatment (Wk 1)	Treatment	Feed Day	No	00:20	00:00 - 00:59	Early Morning	37,7	85	24,6	24,6	18,85	581,90	107,39
	2019/05/16	CH2206	Male	Three y/o	Treatment (Wk 1)	Treatment	Feed Day	No	00:20	00:00 - 00:59	Early Morning	37,3	80	36,6	36,6	18,46	577,31	121,03
	2019/05/16	CH2205	Male	Three y/o	Treatment (Wk 1)	Treatment	Feed Day	No	00:25	00:00 - 00:59	Early Morning	37,6	50	29,0	29,0	18,75	539,52	113,03
	2019/05/16	CH2206	Male	Three y/o	Treatment (Wk 1)	Treatment	Feed Day	No	00:25	00:00 - 00:59	Early Morning	37,4	68	37,0	37,0	18,55	564,71	121,40
	2019/05/16	CH2205	Male	Three y/o	Treatment (Wk 1)	Treatment	Feed Day	No	00:30	00:00 - 00:59	Early Morning	37,5	85	26,0	26,0	18,65	581,90	109,29
	2019/05/16	CH2206	Male	Three y/o	Treatment (Wk 1)	Treatment	Feed Day	No	00:30	00:00 - 00:59	Early Morning	37,5	92	35,2	35,2	18,65	587,78	119,69
	2019/05/16	CH2205	Male	Three y/o	Treatment (Wk 1)	Treatment	Feed Day	No	00:35	00:00 - 00:59	Early Morning	37,5	50	16,2	16,2	18,65	539,52	93,13
	2019/05/16	CH2206	Male	Three y/o	Treatment (Wk 1)	Treatment	Feed Day	No	00:35	00:00 - 00:59	Early Morning	37,5	66	40,0	40,0	18,65	562,34	124,09
	2019/05/16	CH2205	Male	Three y/o	Treatment (Wk 1)	Treatment	Feed Day	No	00:40	00:00 - 00:59	Early Morning	37,5	50	17,4	17,4	18,65	539,52	95,56
	2019/05/16	CH2206	Male	Three y/o	Treatment (Wk 1)	Treatment	Feed Day	No	00:40	00:00 - 00:59	Early Morning	37,5	72	39,0	39,0	18,65	569,20	123,22
	2019/05/16	CH2205	Male	Three y/o	Treatment (Wk 1)	Treatment	Feed Day	No	00:45	00:00 - 00:59	Early Morning	37,5	197	16,0	16,0	18,65	639,25	92,71
	2019/05/16	CH2206	Male	Three y/o	Treatment (Wk 1)	Treatment	Feed Day	No	00:45	00:00 - 00:59	Early Morning	37,5	84	35,6	35,6	18,65	581,01	120,08
	2019/05/16	CH2205	Male	Three y/o	Treatment (Wk 1)	Treatment	Feed Day	No	00:50	00:00 - 00:59	Early Morning	37,5		6,2	6,2	18,65		60,63
	2019/05/16	CH2206	Male	Three y/o	Treatment (Wk 1)	Treatment	Feed Day	No	00:50	00:00 - 00:59	Early Morning	37,5	68	35,2	35,2	18,65	564,71	119,69
	2019/05/16	CH2205	Male	Three y/o	Treatment (Wk 1)	Treatment	Feed Day	No	00:55	00:00 - 00:59	Early Morning	37,5		3,4	3,4	18,65		40,51
	2019/05/16	CH2206	Male	Three y/o	Treatment (Wk 1)	Treatment	Feed Day	No	00:55	00:00 - 00:59	Early Morning	37,5	110	39,6	39,6	18,65	600,69	123,74
	2019/05/16	CH2205	Male	Three y/o	Treatment (Wk 1)	Treatment	Feed Day	No	01:00	01:00 - 01:59	Early Morning	37,4		6,6	6,6	18,55		62,73
	2019/05/16	CH2206	Male	Three y/o	Treatment (Wk 1)	Treatment	Feed Day	No	01:00	01:00 - 01:59	Early Morning	37,5	110	39,4	39,4	18,65	600,69	123,57
	2019/05/16	CH2205	Male	Three y/o	Treatment (Wk 1)	Treatment	Feed Day	No	01:05	01:00 - 01:59	Early Morning	37,3		10,8	10,8	18,46		79,36
	2019/05/16	CH2206	Male	Three y/o	Treatment (Wk 1)	Treatment	Feed Day	No	01:05	01:00 - 01:59	Early Morning	37,5	90	40,0	40,0	18,65	586,16	124,09
	2019/05/16	CH2205	Male	Three y/o	Treatment (Wk 1)	Treatment	Feed Day	No	01:10	01:00 - 01:59	Early Morning	37,3	56	20,8	20,8	18,46	549,01	101,65
	2019/05/16	CH2206	Male	Three y/o	Treatment (Wk 1)	Treatment	Feed Day	No	01:10	01:00 - 01:59	Early Morning	37,5	51	36,8	36,8	18,65	541,20	121,22
	2019/05/16	CH2205	Male	Three y/o	Treatment (Wk 1)	Treatment	Feed Day	No	01:15	01:00 - 01:59	Early Morning	37,3	46	20,8	20,8	18,46	532,38	101,65
	2019/05/16	CH2206	Male	Three y/o	Treatment (Wk 1)	Treatment	Feed Day	No	01:15	01:00 - 01:59	Early Morning	37,4	93	44,0	44,0	18,55	588,58	127,37
	2019/05/16	CH2205	Male	Three y/o	Treatment (Wk 1)	Treatment	Feed Day	No	01:20	01:00 - 01:59	Early Morning	37,3	98	18,2	18,2	18,46	592,41	97,10
	2019/05/16	CH2206	Male	Three y/o	Treatment (Wk 1)	Treatment	Feed Day	No	01:20	01:00 - 01:59	Early Morning	37,5	135	53,0	53,0	18,65	614,84	133,80
	2019/05/16	CH2205	Male	Three y/o	Treatment (Wk 1)	Treatment	Feed Day	No	01:25	01:00 - 01:59	Early Morning	37,3	98	14,2	14,2	18,46	592,41	88,65
	2019/05/16	CH2206	Male	Three y/o	Treatment (Wk 1)	Treatment	Feed Day	No	01:25	01:00 - 01:59	Early Morning	37,4	124	33,4	33,4	18,55	609,04	117,88
	2019/05/16	CH2205	Male	Three y/o	Treatment (Wk 1)	Treatment	Feed Day	No	01:30	01:00 - 01:59	Early Morning	37,3	68	13,4	13,4	18,46	564,71	86,68
	2019/05/16	CH2206	Male	Three y/o	Treatment (Wk 1)	Treatment	Feed Day	No	01:30	01:00 - 01:59	Early Morning	37,3	94	41,6	41,6	18,46	589,37	125,44
	2019/05/16	CH2205	Male	Three y/o	Treatment (Wk 1)	Treatment	Feed Day	No	01:35	01:00 - 01:59	Early Morning	37,3	77	12,4	12,4	18,46	574,39	84,04
	2019/05/16	CH2206	Male	Three y/o	Treatment (Wk 1)	Treatment	Feed Day	No	01:35	01:00 - 01:59	Early Morning	36,9	71	42,2	42,2	18,06	568,10	125,93
	2019/05/16	CH2205	Male	Three y/o	Treatment (Wk 1)	Treatment	Feed Day	No	01:40	01:00 - 01:59	Early Morning	37,3	57	11,8	11,8	18,46	550,47	82,36
	2019/05/16	CH2206	Male	Three y/o	Treatment (Wk 1)	Treatment	Feed Day	No	01:40	01:00 - 01:59	Early Morning	36,5	50	29,4	29,4	17,67	539,52	113,50
	2019/05/16	CH2205	Male	Three y/o	Treatment (Wk 1)	Treatment	Feed Day	No	01:45	01:00 - 01:59	Early Morning	37,3	70	4,0	4,0	18,46	566,99	45,94
	2019/05/16	CH2206	Male	Three y/o	Treatment (Wk 1)	Treatment	Feed Day	No	01:45	01:00 - 01:59	Early Morning	36,8	59	13,8	13,8	17,96	553,30	87,68
	2019/05/16	CH2205	Male	Three y/o	Treatment (Wk 1)	Treatment	Feed Day	No	01:50	01:00 - 01:59	Early Morning	37,5	68	6,4	6,4	18,65	564,71	61,70
	2019/05/16	CH2206	Male	Three y/o	Treatment (Wk 1)	Treatment	Feed Day	No	01:50	01:00 - 01:59	Early Morning	37,0	51	7,8	7,8	18,16	541,20	68,36
	2019/05/16	CH2205	Male	Three y/o	Treatment (Wk 1)	Treatment	Feed Day	No	01:55	01:00 - 01:59	Early Morning	37,5	93	7,2	7,2	18,65	588,58	65,66
	2019/05/16	CH2206	Male	Three y/o	Treatment (Wk 1)	Treatment	Feed Day	No	01:55	01:00 - 01:59	Early Morning	37,1	64	27,2	27,2	18,26	559,88	110,83
	2019/05/16	CH2205	Male	Three y/o	Treatment (Wk 1)	Treatment	Feed Day	No	02:00	02:00 - 02:59	Early Morning	37,6	87	8,0	8,0	18,75	583,64	69,22
	2019/05/16	CH2206	Male	Three y/o	Treatment (Wk 1)	Treatment	Feed Day	No	02:00	02:00 - 02:59	Early Morning	37,2	79	10,4	10,4	18,36	576,36	78,08
	2019/05/16	CH2205	Male	Three y/o	Treatment (Wk 1)	Treatment	Feed Day	No	02:05	02:00 - 02:59	Early Morning	37,5	67	6,8	6,8	18,65	563,53	63,74

2019/05/16	CH2206	Male	Three y/o	Treatment (Wk 1)	Treatment	Feed Day	No	02:05	02:00 - 02:59	Early Morning	37,3	83	5,2	5,2	18,46	580,11	54,72
2019/05/16	CH2205	Male	Three y/o	Treatment (Wk 1)	Treatment	Feed Day	No	02:10	02:00 - 02:59	Early Morning	37,6	77	8,8	8,8	18,75	574,39	72,43
2019/05/16	CH2206	Male	Three y/o	Treatment (Wk 1)	Treatment	Feed Day	No	02:10	02:00 - 02:59	Early Morning	37,3	109	4,0	4,0	18,46	600,04	45,94
2019/05/16	CH2205	Male	Three y/o	Treatment (Wk 1)	Treatment	Feed Day	No	02:15	02:00 - 02:59	Early Morning	37,6	86	8,4	8,4	18,75	582,77	70,86
2019/05/16	CH2206	Male	Three y/o	Treatment (Wk 1)	Treatment	Feed Day	No	02:15	02:00 - 02:59	Early Morning	37,4	86	5,0	5,0	18,55	582,77	53,41
2019/05/16	CH2205	Male	Three y/o	Treatment (Wk 1)	Treatment	Feed Day	No	02:20	02:00 - 02:59	Early Morning	37,6	67	4,0	4,0	18,75	563,53	45,94
2019/05/16	CH2206	Male	Three y/o	Treatment (Wk 1)	Treatment	Feed Day	No	02:20	02:00 - 02:59	Early Morning	37,4	81	3,2	3,2	18,55	578,26	38,49
2019/05/16	CH2205	Male	Three y/o	Treatment (Wk 1)	Treatment	Feed Day	No	02:25	02:00 - 02:59	Early Morning	37,6	95	7,0	7,0	18,75	590,14	64,72
2019/05/16	CH2206	Male	Three y/o	Treatment (Wk 1)	Treatment	Feed Day	No	02:25	02:00 - 02:59	Early Morning	37,4	58	4,4	4,4	18,55	551,90	49,12
2019/05/16	CH2205	Male	Three y/o	Treatment (Wk 1)	Treatment	Feed Day	No	02:30	02:00 - 02:59	Early Morning	37,5	54	5,8	5,8	18,65	545,99	58,39
2019/05/16	CH2206	Male	Three y/o	Treatment (Wk 1)	Treatment	Feed Day	No	02:30	02:00 - 02:59	Early Morning	37,5	86	7,2	7,2	18,65	582,77	65,66
2019/05/16	CH2205	Male	Three y/o	Treatment (Wk 1)	Treatment	Feed Day	No	02:35	02:00 - 02:59	Early Morning	37,5	89	7,6	7,6	18,65	585,33	67,49
2019/05/16	CH2206	Male	Three y/o	Treatment (Wk 1)	Treatment	Feed Day	No	02:35	02:00 - 02:59	Early Morning	37,5	60	14,6	14,6	18,65	554,67	89,59
2019/05/16	CH2205	Male	Three y/o	Treatment (Wk 1)	Treatment	Feed Day	No	02:40	02:00 - 02:59	Early Morning	37,5	92	7,0	7,0	18,65	587,78	64,72
2019/05/16	CH2206	Male	Three y/o	Treatment (Wk 1)	Treatment	Feed Day	No	02:40	02:00 - 02:59	Early Morning	37,5	89	12,0	12,0	18,65	585,33	82,93
2019/05/16	CH2205	Male	Three y/o	Treatment (Wk 1)	Treatment	Feed Day	No	02:45	02:00 - 02:59	Early Morning	37,6	89	4,0	4,0	18,75	585,33	45,94
2019/05/16	CH2206	Male	Three y/o	Treatment (Wk 1)	Treatment	Feed Day	No	02:45	02:00 - 02:59	Early Morning	37,5	50	6,6	6,6	18,65	539,52	62,73
2019/05/16	CH2205	Male	Three y/o	Treatment (Wk 1)	Treatment	Feed Day	No	02:50	02:00 - 02:59	Early Morning	37,5	53	8,0	8,0	18,65	544,43	69,22
2019/05/16	CH2206	Male	Three y/o	Treatment (Wk 1)	Treatment	Feed Day	No	02:50	02:00 - 02:59	Early Morning	37,5	88	11,2	11,2	18,65	584,49	80,59
2019/05/16	CH2205	Male	Three y/o	Treatment (Wk 1)	Treatment	Feed Day	No	02:55	02:00 - 02:59	Early Morning	37,5	53	4,8	4,8	18,65	544,43	52,04
2019/05/16	CH2206	Male	Three y/o	Treatment (Wk 1)	Treatment	Feed Day	No	02:55	02:00 - 02:59	Early Morning	37,5	98	8,6	8,6	18,65	592,41	71,66
2019/05/16	CH2205	Male	Three y/o	Treatment (Wk 1)	Treatment	Feed Day	No	03:00	03:00 - 03:59	Early Morning	37,5	53	5,4	5,4	18,65	544,43	55,99
2019/05/16	CH2206	Male	Three y/o	Treatment (Wk 1)	Treatment	Feed Day	No	03:00	03:00 - 03:59	Early Morning	37,5	77	13,6	13,6	18,65	574,39	87,18
2019/05/16	CH2205	Male	Three y/o	Treatment (Wk 1)	Treatment	Feed Day	No	03:05	03:00 - 03:59	Early Morning	37,5	60	4,8	4,8	18,65	554,67	52,04
2019/05/16	CH2206	Male	Three y/o	Treatment (Wk 1)	Treatment	Feed Day	No	03:05	03:00 - 03:59	Early Morning	37,6	90	13,2	13,2	18,75	586,16	86,17
2019/05/16	CH2205	Male	Three y/o	Treatment (Wk 1)	Treatment	Feed Day	No	03:10	03:00 - 03:59	Early Morning	37,5	63	5,0	5,0	18,65	558,62	53,41
2019/05/16	CH2206	Male	Three y/o	Treatment (Wk 1)	Treatment	Feed Day	No	03:10	03:00 - 03:59	Early Morning	37,7	80	10,4	10,4	18,85	577,31	78,08
2019/05/16	CH2205	Male	Three y/o	Treatment (Wk 1)	Treatment	Feed Day	No	03:15	03:00 - 03:59	Early Morning	37,6	69	3,6	3,6	18,75	565,86	42,42
2019/05/16	CH2206	Male	Three y/o	Treatment (Wk 1)	Treatment	Feed Day	No	03:15	03:00 - 03:59	Early Morning	37,7	60	6,2	6,2	18,85	554,67	60,63
2019/05/16	CH2205	Male	Three y/o	Treatment (Wk 1)	Treatment	Feed Day	No	03:20	03:00 - 03:59	Early Morning	37,6	82	4,8	4,8	18,75	579,19	52,04
2019/05/16	CH2206	Male	Three y/o	Treatment (Wk 1)	Treatment	Feed Day	No	03:20	03:00 - 03:59	Early Morning	37,7	48	10,4	10,4	18,85	536,04	78,08
2019/05/16	CH2205	Male	Three y/o	Treatment (Wk 1)	Treatment	Feed Day	No	03:25	03:00 - 03:59	Early Morning	37,6	51	14,4	14,4	18,75	541,20	89,12
2019/05/16	CH2206	Male	Three y/o	Treatment (Wk 1)	Treatment	Feed Day	No	03:25	03:00 - 03:59	Early Morning	37,7	42	10,4	10,4	18,85	524,42	78,08
2019/05/16	CH2205	Male	Three y/o	Treatment (Wk 1)	Treatment	Feed Day	No	03:30	03:00 - 03:59	Early Morning	37,6	51	8,0	8,0	18,75	541,20	69,22
2019/05/16	CH2206	Male	Three y/o	Treatment (Wk 1)	Treatment	Feed Day	No	03:30	03:00 - 03:59	Early Morning	37,7	47	15,0	15,0	18,85	534,23	90,51
2019/05/16	CH2205	Male	Three y/o	Treatment (Wk 1)	Treatment	Feed Day	No	03:35	03:00 - 03:59	Early Morning	37,5	82	13,6	13,6	18,65	579,19	87,18
2019/05/16	CH2206	Male	Three y/o	Treatment (Wk 1)	Treatment	Feed Day	No	03:35	03:00 - 03:59	Early Morning	37,7	86	15,2	15,2	18,85	582,77	90,96
2019/05/16	CH2205	Male	Three y/o	Treatment (Wk 1)	Treatment	Feed Day	No	03:40	03:00 - 03:59	Early Morning	37,5		13,4	13,4	18,65		86,68
2019/05/16	CH2206	Male	Three y/o	Treatment (Wk 1)	Treatment	Feed Day	No	03:40	03:00 - 03:59	Early Morning	37,7	75	21,4	21,4	18,85	572,36	102,63
2019/05/16	CH2205	Male	Three y/o	Treatment (Wk 1)	Treatment	Feed Day	No	03:45	03:00 - 03:59	Early Morning	37,4	57	15,0	15,0	18,55	550,47	90,51
2019/05/16	CH2206	Male	Three y/o	Treatment (Wk 1)	Treatment	Feed Day	No	03:45	03:00 - 03:59	Early Morning	37,7	73	16,0	16,0	18,85	570,27	92,71
2019/05/16	CH2205	Male	Three y/o	Treatment (Wk 1)	Treatment	Feed Day	No	03:50	03:00 - 03:59	Early Morning	37,3	50	18,2	18,2	18,46	539,52	97,10
2019/05/16	CH2206	Male	Three y/o	Treatment (Wk 1)	Treatment	Feed Day	No	03:50	03:00 - 03:59	Early Morning	37,6	74	18,8	18,8	18,75	571,33	98,20
2019/05/16	CH2205	Male	Three y/o	Treatment (Wk 1)	Treatment	Feed Day	No	03:55	03:00 - 03:59	Early Morning	37,3	53	11,8	11,8	18,46	544,43	82,36
2019/05/16	CH2206	Male	Three y/o	Treatment (Wk 1)	Treatment	Feed Day	No	03:55	03:00 - 03:59	Early Morning	37,7	43	27,2	27,2	18,85	526,50	110,83
2019/05/16	CH2205	Male	Three y/o	Treatment (Wk 1)	Treatment	Feed Day	No	04:00	04:00 - 04:59	Morning	37,4	78	17,8	17,8	18,55	575,38	96,34
2019/05/16	CH2206	Male	Three y/o	Treatment (Wk 1)	Treatment	Feed Day	No	04:00	04:00 - 04:59	Morning	37,7	52	25,8	25,8	18,85	542,83	109,02
2019/05/16	CH2205	Male	Three y/o	Treatment (Wk 1)	Treatment	Feed Day	No	04:05	04:00 - 04:59	Morning	37,4	97	19,4	19,4	18,55	591,66	99,28
2019/05/16	CH2206	Male	Three y/o	Treatment (Wk 1)	Treatment	Feed Day	No	04:05	04:00 - 04:59	Morning	37,7	47	26,8	26,8	18,85	534,23	110,33
2019/05/16	CH2205	Male	Three y/o	Treatment (Wk 1)	Treatment	Feed Day	No	04:10	04:00 - 04:59	Morning	37,3		9,2	9,2	18,46		73,94
2019/05/16	CH2206	Male	Three y/o	Treatment (Wk 1)	Treatment	Feed Day	No	04:10	04:00 - 04:59	Morning	37,7	73	22,2	22,2	18,85	570,27	103,88
2019/05/16	CH2205	Male	Three y/o	Treatment (Wk 1)	Treatment	Feed Day	No	04:15	04:00 - 04:59	Morning	37,4		11,0	11,0	18,55		79,98
2019/05/16	CH2206	Male	Three y/o	Treatment (Wk 1)	Treatment	Feed Day	No	04:15	04:00 - 04:59	Morning	37,7	52	28,6	28,6	18,85	542,83	112,56
2019/05/16	CH2205	Male	Three y/o	Treatment (Wk 1)	Treatment	Feed Day	No	04:20	04:00 - 04:59	Morning	37,5	102	8,2	8,2	18,65	595,30	70,05
2019/05/16	CH2206	Male	Three y/o	Treatment (Wk 1)	Treatment	Feed Day	No	04:20	04:00 - 04:59	Morning	37,7	121	59,0	59,0	18,85	607,35	137,51
2019/05/16	CH2205	Male	Three y/o	Treatment (Wk 1)	Treatment	Feed Day	No	04:25	04:00 - 04:59	Morning	37,5	191	10,8	10,8	18,65	637,33	79,36
2019/05/16	CH2206	Male	Three y/o	Treatment (Wk 1)	Treatment	Feed Day	No	04:25	04:00 - 04:59	Morning	37,7	99	44,6	44,6	18,85	593,14	127,84
2019/05/16	CH2205	Male	Three y/o	Treatment (Wk 1)	Treatment	Feed Day	No	04:30	04:00 - 04:59	Morning	37,5	99	20,2	20,2	18,65	593,14	100,65
2019/05/16	CH2206	Male	Three y/o	Treatment (Wk 1)	Treatment	Feed Day	No	04:30	04:00 - 04:59	Morning	37,7	98	113,8	113,8	18,85	592,41	160,35
2019/05/16	CH2205	Male	Three y/o	Treatment (Wk 1)	Treatment	Feed Day	No	04:35	04:00 - 04:59	Morning	37,5	106	33,6	33,6	18,65	598,06	118,09
2019/05/16	CH2206	Male	Three y/o	Treatment (Wk 1)	Treatment	Feed Day	No	04:35	04:00 - 04:59	Morning	37,7	132	119,8	119,8	18,85	613,32	162,15
2019/05/16	CH2205	Male	Three y/o	Treatment (Wk 1)	Treatment	Feed Day	No	04:40	04:00 - 04:59	Morning	37,5		128,0	128,0	18,65		164,46
2019/05/16	CH2206	Male	Three y/o	Treatment (Wk 1)	Treatment	Feed Day	No	04:40	04:00 - 04:59	Morning	37,5	131	57,0	57,0	18,65	612,80	136,32
2019/05/16	CH2205	Male	Three y/o	Treatment (Wk 1)	Treatment	Feed Day	No	04:45	04:00 - 04:59	Morning	37,4	61	180,6	180,6	18,55	556,01	176,53
2019/05/16	CH2206	Male	Three y/o	Treatment (Wk 1)	Treatment	Feed Day	No	04:45	04:00 - 04:59	Morning	37,4	88	63,2	63,2	18,55	584,49	139,89
2019/05/16	CH2205	Male	Three y/o	Treatment (Wk 1)	Treatment	Feed Day	No	04:50	04:00 - 04:59	Morning	37,4		12,4	12,4	18,55		84,04
2019/05/16	CH2206	Male	Three y/o	Treatment (Wk 1)	Treatment	Feed Day	No	04:50	04:00 - 04:59	Morning	37,4	92	33,0	33,0	18,55	587,78	117,47
2019/05/16	CH2205	Male	Three y/o	Treatment (Wk 1)	Treatment	Feed Day	No	04:55	04:00 - 04:59	Morning	37,3		17,8	17,8	18,46		96,34
2019/05/16	CH2206	Male	Three y/o	Treatment (Wk 1)	Treatment	Feed Day	No	04:55	04:00 - 04:59	Morning	37,1	78	35,2	35,2	18,26	575,38	119,69
2019/05/16	CH2205	Male	Three y/o	Treatment (Wk 1)	Treatment	Feed Day	No	05:00	05:00 - 05:59	Morning	37,2	43	21,0	21,0	18,36	526,50	101,98

2019/05/16	CH2206	Male	Three y/o	Treatment (Wk 1)	Treatment	Feed Day	No	05:00	05:00 - 05:59	Morning	36,8	64	42,0	42,0	17,96	559,88	125,77
2019/05/16	CH2205	Male	Three y/o	Treatment (Wk 1)	Treatment	Feed Day	No	05:05	05:00 - 05:59	Morning	37,3	73	11,8	11,8	18,46	570,27	82,36
2019/05/16	CH2206	Male	Three y/o	Treatment (Wk 1)	Treatment	Feed Day	No	05:05	05:00 - 05:59	Morning	36,5	113	47,6	47,6	17,67	602,58	130,09
2019/05/16	CH2205	Male	Three y/o	Treatment (Wk 1)	Treatment	Feed Day	No	05:10	05:00 - 05:59	Morning	37,3	75	9,8	9,8	18,46	572,36	76,07
2019/05/16	CH2206	Male	Three y/o	Treatment (Wk 1)	Treatment	Feed Day	No	05:10	05:00 - 05:59	Morning	36,5	73	61,2	61,2	17,67	570,27	138,78
2019/05/16	CH2205	Male	Three y/o	Treatment (Wk 1)	Treatment	Feed Day	No	05:15	05:00 - 05:59	Morning	37,3	44	8,2	8,2	18,46	528,51	70,05
2019/05/16	CH2206	Male	Three y/o	Treatment (Wk 1)	Treatment	Feed Day	No	05:15	05:00 - 05:59	Morning	36,1	72	59,2	59,2	17,29	569,20	137,63
2019/05/16	CH2205	Male	Three y/o	Treatment (Wk 1)	Treatment	Feed Day	No	05:20	05:00 - 05:59	Morning	37,3	79	5,8	5,8	18,46	576,36	58,39
2019/05/16	CH2206	Male	Three y/o	Treatment (Wk 1)	Treatment	Feed Day	No	05:20	05:00 - 05:59	Morning	35,9	57	41,4	41,4	17,09	550,47	125,27
2019/05/16	CH2205	Male	Three y/o	Treatment (Wk 1)	Treatment	Feed Day	No	05:25	05:00 - 05:59	Morning	37,2	52	2,4	2,4	18,36	542,83	28,91
2019/05/16	CH2206	Male	Three y/o	Treatment (Wk 1)	Treatment	Feed Day	No	05:25	05:00 - 05:59	Morning	36,4	88	28,6	28,6	17,57	584,49	112,56
2019/05/16	CH2205	Male	Three y/o	Treatment (Wk 1)	Treatment	Feed Day	No	05:30	05:00 - 05:59	Morning	37,2	52	1,2	1,2	18,36	542,83	5,99
2019/05/16	CH2206	Male	Three y/o	Treatment (Wk 1)	Treatment	Feed Day	No	05:30	05:00 - 05:59	Morning	36,5	74	25,8	25,8	17,67	571,33	109,02
2019/05/16	CH2205	Male	Three y/o	Treatment (Wk 1)	Treatment	Feed Day	No	05:35	05:00 - 05:59	Morning	37,1	85	7,8	7,8	18,26	581,90	68,36
2019/05/16	CH2206	Male	Three y/o	Treatment (Wk 1)	Treatment	Feed Day	No	05:35	05:00 - 05:59	Morning	36,6	77	29,6	29,6	17,77	574,39	113,73
2019/05/16	CH2205	Male	Three y/o	Treatment (Wk 1)	Treatment	Feed Day	No	05:40	05:00 - 05:59	Morning	37,2	55	4,4	4,4	18,36	547,52	49,12
2019/05/16	CH2206	Male	Three y/o	Treatment (Wk 1)	Treatment	Feed Day	No	05:40	05:00 - 05:59	Morning	36,6	47	40,8	40,8	17,77	534,23	124,77
2019/05/16	CH2205	Male	Three y/o	Treatment (Wk 1)	Treatment	Feed Day	No	05:45	05:00 - 05:59	Morning	37,3	82	7,6	7,6	18,46	579,19	67,49
2019/05/16	CH2206	Male	Three y/o	Treatment (Wk 1)	Treatment	Feed Day	No	05:45	05:00 - 05:59	Morning	36,8	80	48,8	48,8	17,96	577,31	130,95
2019/05/16	CH2205	Male	Three y/o	Treatment (Wk 1)	Treatment	Feed Day	No	05:50	05:00 - 05:59	Morning	37,3	60	9,8	9,8	18,46	554,67	76,07
2019/05/16	CH2206	Male	Three y/o	Treatment (Wk 1)	Treatment	Feed Day	No	05:50	05:00 - 05:59	Morning	36,9	50	46,6	46,6	18,06	539,52	129,35
2019/05/16	CH2205	Male	Three y/o	Treatment (Wk 1)	Treatment	Feed Day	No	05:55	05:00 - 05:59	Morning	37,3	73	18,2	18,2	18,46	570,27	97,10
2019/05/16	CH2206	Male	Three y/o	Treatment (Wk 1)	Treatment	Feed Day	No	05:55	05:00 - 05:59	Morning	37,1	80	39,8	39,8	18,26	577,31	123,91
2019/05/16	CH2205	Male	Three y/o	Treatment (Wk 1)	Treatment	Feed Day	No	06:00	06:00 - 06:59	Morning	37,3	117	3,6	3,6	18,46	605,02	42,42
2019/05/16	CH2206	Male	Three y/o	Treatment (Wk 1)	Treatment	Feed Day	No	06:00	06:00 - 06:59	Morning	37,1	75	43,8	43,8	18,26	572,36	127,22
2019/05/16	CH2205	Male	Three y/o	Treatment (Wk 1)	Treatment	Feed Day	No	06:05	06:00 - 06:59	Morning	37,1	110	97,8	97,8	18,26	600,69	155,07
2019/05/16	CH2206	Male	Three y/o	Treatment (Wk 1)	Treatment	Feed Day	No	06:05	06:00 - 06:59	Morning	37,1	121	183,0	183,0	18,26	607,35	177,00
2019/05/16	CH2205	Male	Three y/o	Treatment (Wk 1)	Treatment	Feed Day	No	06:10	06:00 - 06:59	Morning	37,2	172	37,4	37,4	18,36	630,73	121,77
2019/05/16	CH2206	Male	Three y/o	Treatment (Wk 1)	Treatment	Feed Day	No	06:10	06:00 - 06:59	Morning	37,2		113,2	113,2	18,36		160,17
2019/05/16	CH2205	Male	Three y/o	Treatment (Wk 1)	Treatment	Feed Day	No	06:15	06:00 - 06:59	Morning	37,3	128	100,2	100,2	18,46	611,22	155,91
2019/05/16	CH2206	Male	Three y/o	Treatment (Wk 1)	Treatment	Feed Day	No	06:15	06:00 - 06:59	Morning	37,2	111	13,8	13,8	18,36	601,33	87,68
2019/05/16	CH2205	Male	Three y/o	Treatment (Wk 1)	Treatment	Feed Day	No	06:20	06:00 - 06:59	Morning	37,4	140	92,8	92,8	18,55	617,28	153,24
2019/05/16	CH2206	Male	Three y/o	Treatment (Wk 1)	Treatment	Feed Day	No	06:20	06:00 - 06:59	Morning	37,1	104	164,8	164,8	18,26	596,69	173,32
2019/05/16	CH2205	Male	Three y/o	Treatment (Wk 1)	Treatment	Feed Day	No	06:25	06:00 - 06:59	Morning	37,4		124,8	124,8	18,55		163,58
2019/05/16	CH2206	Male	Three y/o	Treatment (Wk 1)	Treatment	Feed Day	No	06:25	06:00 - 06:59	Morning	37,2	117	104,4	104,4	18,36	605,02	157,35
2019/05/16	CH2205	Male	Three y/o	Treatment (Wk 1)	Treatment	Feed Day	No	06:30	06:00 - 06:59	Morning	37,5	115	92,8	92,8	18,65	603,82	153,24
2019/05/16	CH2206	Male	Three y/o	Treatment (Wk 1)	Treatment	Feed Day	No	06:30	06:00 - 06:59	Morning	37,4	118	113,6	113,6	18,55	605,61	160,29
2019/05/16	CH2205	Male	Three y/o	Treatment (Wk 1)	Treatment	Feed Day	No	06:35	06:00 - 06:59	Morning	37,7	63	218,0	218,0	18,85	558,62	183,15
2019/05/16	CH2206	Male	Three y/o	Treatment (Wk 1)	Treatment	Feed Day	No	06:35	06:00 - 06:59	Morning	37,7	114	165,8	165,8	18,85	603,20	173,53
2019/05/16	CH2205	Male	Three y/o	Treatment (Wk 1)	Treatment	Feed Day	No	06:40	06:00 - 06:59	Morning	37,7	125	227,4	227,4	18,85	609,60	184,64
2019/05/16	CH2206	Male	Three y/o	Treatment (Wk 1)	Treatment	Feed Day	No	06:40	06:00 - 06:59	Morning	37,7	109	121,6	121,6	18,85	600,04	162,67
2019/05/16	CH2205	Male	Three y/o	Treatment (Wk 1)	Treatment	Feed Day	No	06:45	06:00 - 06:59	Morning	37,6	57	143,8	143,8	18,75	550,47	168,54
2019/05/16	CH2206	Male	Three y/o	Treatment (Wk 1)	Treatment	Feed Day	No	06:45	06:00 - 06:59	Morning	37,5	106	100,6	100,6	18,65	598,06	156,05
2019/05/16	CH2205	Male	Three y/o	Treatment (Wk 1)	Treatment	Feed Day	No	06:50	06:00 - 06:59	Morning	37,6	188	225,0	225,0	18,75	636,34	184,27
2019/05/16	CH2206	Male	Three y/o	Treatment (Wk 1)	Treatment	Feed Day	No	06:50	06:00 - 06:59	Morning	37,5	114	121,2	121,2	18,65	603,20	162,56
2019/05/16	CH2205	Male	Three y/o	Treatment (Wk 1)	Treatment	Feed Day	No	06:55	06:00 - 06:59	Morning	37,6	73	242,0	242,0	18,75	570,27	186,83
2019/05/16	CH2206	Male	Three y/o	Treatment (Wk 1)	Treatment	Feed Day	No	06:55	06:00 - 06:59	Morning	37,5	104	80,8	80,8	18,65	596,69	148,42
2019/05/16	CH2205	Male	Three y/o	Treatment (Wk 1)	Treatment	Feed Day	No	07:00	07:00 - 07:59	Morning	37,6	140	93,6	93,6	18,75	617,28	153,54
2019/05/16	CH2206	Male	Three y/o	Treatment (Wk 1)	Treatment	Feed Day	No	07:00	07:00 - 07:59	Morning	37,5	125	478,8	478,8	18,65	609,60	211,00
2019/05/16	CH2205	Male	Three y/o	Treatment (Wk 1)	Treatment	Feed Day	No	07:05	07:00 - 07:59	Morning	37,6	79	295,6	295,6	18,75	576,36	193,90
2019/05/16	CH2206	Male	Three y/o	Treatment (Wk 1)	Treatment	Feed Day	No	07:05	07:00 - 07:59	Morning	37,5	137	403,6	403,6	18,65	615,83	204,93
2019/05/16	CH2205	Male	Three y/o	Treatment (Wk 1)	Treatment	Feed Day	No	07:10	07:00 - 07:59	Morning	37,6	103	351,8	351,8	18,75	596,00	200,06
2019/05/16	CH2206	Male	Three y/o	Treatment (Wk 1)	Treatment	Feed Day	No	07:10	07:00 - 07:59	Morning	37,7		153,4	153,4	18,85		170,80
2019/05/16	CH2205	Male	Three y/o	Treatment (Wk 1)	Treatment	Feed Day	No	07:15	07:00 - 07:59	Morning	37,7		254,4	254,4	18,85		188,60
2019/05/16	CH2206	Male	Three y/o	Treatment (Wk 1)	Treatment	Feed Day	No	07:15	07:00 - 07:59	Morning	37,7		44,8	44,8	18,85		127,99
2019/05/16	CH2205	Male	Three y/o	Treatment (Wk 1)	Treatment	Feed Day	No	07:20	07:00 - 07:59	Morning	37,6	103	17,6	17,6	18,75	596,00	95,95
2019/05/16	CH2206	Male	Three y/o	Treatment (Wk 1)	Treatment	Feed Day	No	07:20	07:00 - 07:59	Morning	37,4	75	54,4	54,4	18,55	572,36	134,70
2019/05/16	CH2205	Male	Three y/o	Treatment (Wk 1)	Treatment	Feed Day	No	07:25	07:00 - 07:59	Morning	37,6		6,4	6,4	18,75		61,70
2019/05/16	CH2206	Male	Three y/o	Treatment (Wk 1)	Treatment	Feed Day	No	07:25	07:00 - 07:59	Morning	37,1	98	150,6	150,6	18,26	592,41	170,16
2019/05/16	CH2205	Male	Three y/o	Treatment (Wk 1)	Treatment	Feed Day	No	07:30	07:00 - 07:59	Morning	37,6	64	76,8	76,8	18,75	559,88	146,66
2019/05/16	CH2206	Male	Three y/o	Treatment (Wk 1)	Treatment	Feed Day	No	07:30	07:00 - 07:59	Morning	37,2		89,8	89,8	18,36		152,10
2019/05/16	CH2205	Male	Three y/o	Treatment (Wk 1)	Treatment	Feed Day	No	07:35	07:00 - 07:59	Morning	37,6	54	104,0	104,0	18,75	545,99	157,21
2019/05/16	CH2206	Male	Three y/o	Treatment (Wk 1)	Treatment	Feed Day	No	07:35	07:00 - 07:59	Morning	37,3	103	175,6	175,6	18,46	596,00	175,55
2019/05/16	CH2205	Male	Three y/o	Treatment (Wk 1)	Treatment	Feed Day	No	07:40	07:00 - 07:59	Morning	37,6	91	95,2	95,2	18,75	586,98	154,13
2019/05/16	CH2206	Male	Three y/o	Treatment (Wk 1)	Treatment	Feed Day	No	07:40	07:00 - 07:59	Morning	37,4	110	376,0	376,0	18,55	600,69	202,42
2019/05/16	CH2205	Male	Three y/o	Treatment (Wk 1)	Treatment	Feed Day	No	07:45	07:00 - 07:59	Morning	37,5	115	270,2	270,2	18,65	603,82	190,72
2019/05/16	CH2206	Male	Three y/o	Treatment (Wk 1)	Treatment	Feed Day	No	07:45	07:00 - 07:59	Morning	37,5	125	158,0	158,0	18,65	609,60	171,84
2019/05/16	CH2205	Male	Three y/o	Treatment (Wk 1)	Treatment	Feed Day	No	07:50	07:00 - 07:59	Morning	37,6		58,2	58,2	18,75		137,04
2019/05/16	CH2206	Male	Three y/o	Treatment (Wk 1)	Treatment	Feed Day	No	07:50	07:00 - 07:59	Morning	37,5	172	69,6	69,6	18,65	630,73	143,24
2019/05/16	CH2205	Male	Three y/o	Treatment (Wk 1)	Treatment	Feed Day	No	07:55	07:00 - 07:59	Morning	37,6	90	119,4	119,4	18,75	586,16	162,03

2019/05/16	CH2206	Male	Three y/o	Treatment (Wk 1)	Treatment	Feed Day	No	07:55	07:00 - 07:59	Morning	37,5	113	95,2	95,2	18,65	602,58	154,13
2019/05/16	CH2205	Male	Three y/o	Treatment (Wk 1)	Treatment	Feed Day	No	08:00	08:00 - 08:59	Late Morning	37,5	98	45,0	45,0	18,65	592,41	128,15
2019/05/16	CH2206	Male	Three y/o	Treatment (Wk 1)	Treatment	Feed Day	No	08:00	08:00 - 08:59	Late Morning	37,5	106	131,0	131,0	18,65	598,06	165,27
2019/05/16	CH2205	Male	Three y/o	Treatment (Wk 1)	Treatment	Feed Day	No	08:05	08:00 - 08:59	Late Morning	37,5		33,6	33,6	18,65		118,09
2019/05/16	CH2206	Male	Three y/o	Treatment (Wk 1)	Treatment	Feed Day	No	08:05	08:00 - 08:59	Late Morning	37,5	124	114,4	114,4	18,65	609,04	160,54
2019/05/16	CH2205	Male	Three y/o	Treatment (Wk 1)	Treatment	Feed Day	No	08:10	08:00 - 08:59	Late Morning	37,4	61	24,8	24,8	18,55	556,01	107,67
2019/05/16	CH2206	Male	Three y/o	Treatment (Wk 1)	Treatment	Feed Day	No	08:10	08:00 - 08:59	Late Morning	37,5	109	80,0	80,0	18,65	600,04	148,08
2019/05/16	CH2205	Male	Three y/o	Treatment (Wk 1)	Treatment	Feed Day	No	08:15	08:00 - 08:59	Late Morning	37,4		20,0	20,0	18,55		100,31
2019/05/16	CH2206	Male	Three y/o	Treatment (Wk 1)	Treatment	Feed Day	No	08:15	08:00 - 08:59	Late Morning	37,7	108	87,0	87,0	18,85	599,39	150,99
2019/05/16	CH2205	Male	Three y/o	Treatment (Wk 1)	Treatment	Feed Day	No	08:20	08:00 - 08:59	Late Morning	37,3	55	16,2	16,2	18,46	547,52	93,13
2019/05/16	CH2206	Male	Three y/o	Treatment (Wk 1)	Treatment	Feed Day	No	08:20	08:00 - 08:59	Late Morning	37,7	94	106,4	106,4	18,85	589,37	158,01
2019/05/16	CH2205	Male	Three y/o	Treatment (Wk 1)	Treatment	Feed Day	No	08:25	08:00 - 08:59	Late Morning	37,2		16,6	16,6	18,36		93,96
2019/05/16	CH2206	Male	Three y/o	Treatment (Wk 1)	Treatment	Feed Day	No	08:25	08:00 - 08:59	Late Morning	37,7	113	126,6	126,6	18,85	602,58	164,08
2019/05/16	CH2205	Male	Three y/o	Treatment (Wk 1)	Treatment	Feed Day	No	08:30	08:00 - 08:59	Late Morning	37,2	53	84,4	84,4	18,36	544,43	149,94
2019/05/16	CH2206	Male	Three y/o	Treatment (Wk 1)	Treatment	Feed Day	No	08:30	08:00 - 08:59	Late Morning	37,6	141	33,6	33,6	18,75	617,75	118,09
2019/05/16	CH2205	Male	Three y/o	Treatment (Wk 1)	Treatment	Feed Day	No	08:35	08:00 - 08:59	Late Morning	37,3		128,8	128,8	18,46		164,68
2019/05/16	CH2206	Male	Three y/o	Treatment (Wk 1)	Treatment	Feed Day	No	08:35	08:00 - 08:59	Late Morning	37,7	64	275,4	275,4	18,85	559,88	191,40
2019/05/16	CH2205	Male	Three y/o	Treatment (Wk 1)	Treatment	Feed Day	No	08:40	08:00 - 08:59	Late Morning	37,3	97	109,2	109,2	18,46	591,66	158,91
2019/05/16	CH2206	Male	Three y/o	Treatment (Wk 1)	Treatment	Feed Day	No	08:40	08:00 - 08:59	Late Morning	37,9		157,8	157,8	19,05		171,79
2019/05/16	CH2205	Male	Three y/o	Treatment (Wk 1)	Treatment	Feed Day	No	08:45	08:00 - 08:59	Late Morning	37,3	88	43,2	43,2	18,46	584,49	126,74
2019/05/16	CH2206	Male	Three y/o	Treatment (Wk 1)	Treatment	Feed Day	No	08:45	08:00 - 08:59	Late Morning	37,9	105	152,0	152,0	19,05	597,38	170,48
2019/05/16	CH2205	Male	Three y/o	Treatment (Wk 1)	Treatment	Feed Day	No	08:50	08:00 - 08:59	Late Morning	37,3	83	59,0	59,0	18,46	580,11	137,51
2019/05/16	CH2206	Male	Three y/o	Treatment (Wk 1)	Treatment	Feed Day	No	08:50	08:00 - 08:59	Late Morning	37,8		111,6	111,6	18,95		159,67
2019/05/16	CH2205	Male	Three y/o	Treatment (Wk 1)	Treatment	Feed Day	No	08:55	08:00 - 08:59	Late Morning	37,4		18,8	18,8	18,55		98,20
2019/05/16	CH2206	Male	Three y/o	Treatment (Wk 1)	Treatment	Feed Day	No	08:55	08:00 - 08:59	Late Morning	37,8	141	101,0	101,0	18,95	617,75	156,19
2019/05/16	CH2205	Male	Three y/o	Treatment (Wk 1)	Treatment	Feed Day	No	09:00	09:00 - 09:59	Late Morning	37,5	60	87,4	87,4	18,65	554,67	151,15
2019/05/16	CH2206	Male	Three y/o	Treatment (Wk 1)	Treatment	Feed Day	No	09:00	09:00 - 09:59	Late Morning	37,8	124	145,8	145,8	18,95	609,04	169,02
2019/05/16	CH2205	Male	Three y/o	Treatment (Wk 1)	Treatment	Feed Day	No	09:05	09:00 - 09:59	Late Morning	37,6	73	40,8	40,8	18,75	570,27	124,77
2019/05/16	CH2206	Male	Three y/o	Treatment (Wk 1)	Treatment	Feed Day	No	09:05	09:00 - 09:59	Late Morning	37,7	119	97,4	97,4	18,85	606,20	154,93
2019/05/16	CH2205	Male	Three y/o	Treatment (Wk 1)	Treatment	Feed Day	No	09:10	09:00 - 09:59	Late Morning	37,6	51	13,6	13,6	18,75	541,20	87,18
2019/05/16	CH2206	Male	Three y/o	Treatment (Wk 1)	Treatment	Feed Day	No	09:10	09:00 - 09:59	Late Morning	37,7	96	18,2	18,2	18,85	590,91	97,10
2019/05/16	CH2205	Male	Three y/o	Treatment (Wk 1)	Treatment	Feed Day	No	09:15	09:00 - 09:59	Late Morning	37,6	58	54,0	54,0	18,75	551,90	134,45
2019/05/16	CH2206	Male	Three y/o	Treatment (Wk 1)	Treatment	Feed Day	No	09:15	09:00 - 09:59	Late Morning	37,7	90	14,0	14,0	18,85	586,16	88,17
2019/05/16	CH2205	Male	Three y/o	Treatment (Wk 1)	Treatment	Feed Day	No	09:20	09:00 - 09:59	Late Morning	37,5	68	15,6	15,6	18,65	564,71	91,85
2019/05/16	CH2206	Male	Three y/o	Treatment (Wk 1)	Treatment	Feed Day	No	09:20	09:00 - 09:59	Late Morning	37,7	67	17,0	17,0	18,85	563,53	94,77
2019/05/16	CH2205	Male	Three y/o	Treatment (Wk 1)	Treatment	Feed Day	No	09:25	09:00 - 09:59	Late Morning	37,4	117	19,6	19,6	18,55	605,02	99,63
2019/05/16	CH2206	Male	Three y/o	Treatment (Wk 1)	Treatment	Feed Day	No	09:25	09:00 - 09:59	Late Morning	37,7	47	14,6	14,6	18,85	534,23	89,59
2019/05/16	CH2205	Male	Three y/o	Treatment (Wk 1)	Treatment	Feed Day	No	09:30	09:00 - 09:59	Late Morning	37,4	53	13,0	13,0	18,55	544,43	85,65
2019/05/16	CH2206	Male	Three y/o	Treatment (Wk 1)	Treatment	Feed Day	No	09:30	09:00 - 09:59	Late Morning	37,7	54	22,8	22,8	18,85	545,99	104,79
2019/05/16	CH2205	Male	Three y/o	Treatment (Wk 1)	Treatment	Feed Day	No	09:35	09:00 - 09:59	Late Morning	37,4	44	20,4	20,4	18,55	528,51	100,99
2019/05/16	CH2206	Male	Three y/o	Treatment (Wk 1)	Treatment	Feed Day	No	09:35	09:00 - 09:59	Late Morning	37,5	54	26,8	26,8	18,65	545,99	110,33
2019/05/16	CH2205	Male	Three y/o	Treatment (Wk 1)	Treatment	Feed Day	No	09:40	09:00 - 09:59	Late Morning	37,4	86	75,2	75,2	18,55	582,77	145,93
2019/05/16	CH2206	Male	Three y/o	Treatment (Wk 1)	Treatment	Feed Day	No	09:40	09:00 - 09:59	Late Morning	37,5		32,4	32,4	18,65		116,84
2019/05/16	CH2205	Male	Three y/o	Treatment (Wk 1)	Treatment	Feed Day	No	09:45	09:00 - 09:59	Late Morning	37,4	56	149,8	149,8	18,55	549,01	169,97
2019/05/16	CH2206	Male	Three y/o	Treatment (Wk 1)	Treatment	Feed Day	No	09:45	09:00 - 09:59	Late Morning	37,5	113	106,0	106,0	18,65	602,58	157,88
2019/05/16	CH2205	Male	Three y/o	Treatment (Wk 1)	Treatment	Feed Day	No	09:50	09:00 - 09:59	Late Morning	37,4		31,6	31,6	18,55		115,98
2019/05/16	CH2206	Male	Three y/o	Treatment (Wk 1)	Treatment	Feed Day	No	09:50	09:00 - 09:59	Late Morning	37,5	117	52,2	52,2	18,65	605,02	133,27
2019/05/16	CH2205	Male	Three y/o	Treatment (Wk 1)	Treatment	Feed Day	No	09:55	09:00 - 09:59	Late Morning	37,3	49	9,0	9,0	18,46	537,80	73,19
2019/05/16	CH2206	Male	Three y/o	Treatment (Wk 1)	Treatment	Feed Day	No	09:55	09:00 - 09:59	Late Morning	37,5	77	38,4	38,4	18,65	574,39	122,68
2019/05/16	CH2205	Male	Three y/o	Treatment (Wk 1)	Treatment	Feed Day	No	10:00	10:00 - 10:59	Late Morning	37,4	47	9,4	9,4	18,55	534,23	74,66
2019/05/16	CH2206	Male	Three y/o	Treatment (Wk 1)	Treatment	Feed Day	No	10:00	10:00 - 10:59	Late Morning	37,5	50	28,8	28,8	18,65	539,52	112,79
2019/05/16	CH2205	Male	Three y/o	Treatment (Wk 1)	Treatment	Feed Day	No	10:05	10:00 - 10:59	Late Morning	37,4	48	8,2	8,2	18,55	536,04	70,05
2019/05/16	CH2206	Male	Three y/o	Treatment (Wk 1)	Treatment	Feed Day	No	10:05	10:00 - 10:59	Late Morning	37,5	56	25,8	25,8	18,65	549,01	109,02
2019/05/16	CH2205	Male	Three y/o	Treatment (Wk 1)	Treatment	Feed Day	No	10:10	10:00 - 10:59	Late Morning	37,4	43	7,6	7,6	18,55	526,50	67,49
2019/05/16	CH2206	Male	Three y/o	Treatment (Wk 1)	Treatment	Feed Day	No	10:10	10:00 - 10:59	Late Morning	37,5	44	28,6	28,6	18,65	528,51	112,56
2019/05/16	CH2205	Male	Three y/o	Treatment (Wk 1)	Treatment	Feed Day	No	10:15	10:00 - 10:59	Late Morning	37,4	53	8,8	8,8	18,55	544,43	72,43
2019/05/16	CH2206	Male	Three y/o	Treatment (Wk 1)	Treatment	Feed Day	No	10:15	10:00 - 10:59	Late Morning	37,4	59	24,6	24,6	18,55	553,30	107,39
2019/05/16	CH2205	Male	Three y/o	Treatment (Wk 1)	Treatment	Feed Day	No	10:20	10:00 - 10:59	Late Morning	37,4	48	7,0	7,0	18,55	536,04	64,72
2019/05/16	CH2206	Male	Three y/o	Treatment (Wk 1)	Treatment	Feed Day	No	10:20	10:00 - 10:59	Late Morning	37,4	51	22,6	22,6	18,55	541,20	104,49
2019/05/16	CH2205	Male	Three y/o	Treatment (Wk 1)	Treatment	Feed Day	No	10:25	10:00 - 10:59	Late Morning	37,4	38	8,4	8,4	18,55	515,48	70,86
2019/05/16	CH2206	Male	Three y/o	Treatment (Wk 1)	Treatment	Feed Day	No	10:25	10:00 - 10:59	Late Morning	37,4	64	16,2	16,2	18,55	559,88	93,13
2019/05/16	CH2205	Male	Three y/o	Treatment (Wk 1)	Treatment	Feed Day	Yes	10:30	10:00 - 10:59	Late Morning	37,4	42	8,2	8,2	18,55	524,42	70,05
2019/05/16	CH2206	Male	Three y/o	Treatment (Wk 1)	Treatment	Feed Day	Yes	10:30	10:00 - 10:59	Late Morning	37,4	48	29,8	29,8	18,55	536,04	113,97
2019/05/16	CH2205	Male	Three y/o	Treatment (Wk 1)	Treatment	Feed Day	Yes	10:35	10:00 - 10:59	Late Morning	37,3	46	5,6	5,6	18,46	532,38	57,21
2019/05/16	CH2206	Male	Three y/o	Treatment (Wk 1)	Treatment	Feed Day	Yes	10:35	10:00 - 10:59	Late Morning	37,4	44	57,8	57,8	18,55	528,51	136,80
2019/05/16	CH2205	Male	Three y/o	Treatment (Wk 1)	Treatment	Feed Day	Yes	10:40	10:00 - 10:59	Late Morning	37,3	44	7,4	7,4	18,46	528,51	66,59
2019/05/16	CH2206	Male	Three y/o	Treatment (Wk 1)	Treatment	Feed Day	Yes	10:40	10:00 - 10:59	Late Morning	37,4	65	47,4	47,4	18,55	561,12	129,94
2019/05/16	CH2205	Male	Three y/o	Treatment (Wk 1)	Treatment	Feed Day	Yes	10:45	10:00 - 10:59	Late Morning	37,3	41	6,4	6,4	18,46	522,29	61,70
2019/05/16	CH2206	Male	Three y/o	Treatment (Wk 1)	Treatment	Feed Day	Yes	10:45	10:00 - 10:59	Late Morning	37,4	42	47,2	47,2	18,55	524,42	129,80
2019/05/16	CH2205	Male	Three y/o	Treatment (Wk 1)	Treatment	Feed Day	Yes	10:50	10:00 - 10:59	Late Morning	37,3	40	5,6	5,6	18,46	520,09	57,21

2019/05/16	CH2206	Male	Three y/o	Treatment (Wk 1)	Treatment	Feed Day	Yes	10:50	10:00 - 10:59	Late Morning	37,4	44	47,4	47,4	18,55	528,51	129,94
2019/05/16	CH2205	Male	Three y/o	Treatment (Wk 1)	Treatment	Feed Day	Yes	10:55	10:00 - 10:59	Late Morning	37,2	39	7,0	7,0	18,36	517,82	64,72
2019/05/16	CH2206	Male	Three y/o	Treatment (Wk 1)	Treatment	Feed Day	Yes	10:55	10:00 - 10:59	Late Morning	37,4	44	26,8	26,8	18,55	528,51	110,33
2019/05/16	CH2205	Male	Three y/o	Treatment (Wk 1)	Treatment	Feed Day	Yes	11:00	11:00 - 11:59	Late Morning	37,2	61	9,0	9,0	18,36	556,01	73,19
2019/05/16	CH2206	Male	Three y/o	Treatment (Wk 1)	Treatment	Feed Day	Yes	11:00	11:00 - 11:59	Late Morning	37,4	42	28,2	28,2	18,55	524,42	112,07
2019/05/16	CH2205	Male	Three y/o	Treatment (Wk 1)	Treatment	Feed Day	Yes	11:05	11:00 - 11:59	Late Morning	37,2	96	17,6	17,6	18,36	590,91	95,95
2019/05/16	CH2206	Male	Three y/o	Treatment (Wk 1)	Treatment	Feed Day	Yes	11:05	11:00 - 11:59	Late Morning	37,3	43	27,2	27,2	18,46	526,50	110,83
2019/05/16	CH2205	Male	Three y/o	Treatment (Wk 1)	Treatment	Feed Day	Yes	11:10	11:00 - 11:59	Late Morning	37,2	59	20,8	20,8	18,36	553,30	101,65
2019/05/16	CH2206	Male	Three y/o	Treatment (Wk 1)	Treatment	Feed Day	Yes	11:10	11:00 - 11:59	Late Morning	37,3	67	55,4	55,4	18,46	563,53	135,33
2019/05/16	CH2205	Male	Three y/o	Treatment (Wk 1)	Treatment	Feed Day	Yes	11:15	11:00 - 11:59	Late Morning	37,3	58	29,4	29,4	18,46	551,90	113,50
2019/05/16	CH2206	Male	Three y/o	Treatment (Wk 1)	Treatment	Feed Day	Yes	11:15	11:00 - 11:59	Late Morning	37,3	104	36,8	36,8	18,46	596,69	121,22
2019/05/16	CH2205	Male	Three y/o	Treatment (Wk 1)	Treatment	Feed Day	Yes	11:20	11:00 - 11:59	Late Morning	37,3	82	16,8	16,8	18,46	579,19	94,37
2019/05/16	CH2206	Male	Three y/o	Treatment (Wk 1)	Treatment	Feed Day	Yes	11:20	11:00 - 11:59	Late Morning	37,3	78	46,0	46,0	18,46	575,38	128,91
2019/05/16	CH2205	Male	Three y/o	Treatment (Wk 1)	Treatment	Feed Day	Yes	11:25	11:00 - 11:59	Late Morning	37,2	152	73,0	73,0	18,36	622,73	144,89
2019/05/16	CH2206	Male	Three y/o	Treatment (Wk 1)	Treatment	Feed Day	Yes	11:25	11:00 - 11:59	Late Morning	37,4	51	105,2	105,2	18,55	541,20	157,61
2019/05/16	CH2205	Male	Three y/o	Treatment (Wk 1)	Treatment	Feed Day	No	11:30	11:00 - 11:59	Late Morning	37,2	99	20,6	20,6	18,36	593,14	101,32
2019/05/16	CH2206	Male	Three y/o	Treatment (Wk 1)	Treatment	Feed Day	No	11:30	11:00 - 11:59	Late Morning	37,3	109	187,2	187,2	18,46	600,04	177,79
2019/05/16	CH2205	Male	Three y/o	Treatment (Wk 1)	Treatment	Feed Day	No	11:35	11:00 - 11:59	Late Morning	37,2	100	132,4	132,4	18,36	593,87	165,65
2019/05/16	CH2206	Male	Three y/o	Treatment (Wk 1)	Treatment	Feed Day	No	11:35	11:00 - 11:59	Late Morning	37,4		35,6	35,6	18,55		120,08
2019/05/16	CH2205	Male	Three y/o	Treatment (Wk 1)	Treatment	Feed Day	No	11:40	11:00 - 11:59	Late Morning	37,5		68,4	68,4	18,65		142,64
2019/05/16	CH2206	Male	Three y/o	Treatment (Wk 1)	Treatment	Feed Day	No	11:40	11:00 - 11:59	Late Morning	37,4		26,4	26,4	18,55		109,81
2019/05/16	CH2205	Male	Three y/o	Treatment (Wk 1)	Treatment	Feed Day	No	11:45	11:00 - 11:59	Late Morning	37,4		9,4	9,4	18,55		74,66
2019/05/16	CH2206	Male	Three y/o	Treatment (Wk 1)	Treatment	Feed Day	No	11:45	11:00 - 11:59	Late Morning	37,3	92	12,4	12,4	18,46	587,78	84,04
2019/05/16	CH2205	Male	Three y/o	Treatment (Wk 1)	Treatment	Feed Day	No	11:50	11:00 - 11:59	Late Morning	37,3		211,6	211,6	18,46		182,10
2019/05/16	CH2206	Male	Three y/o	Treatment (Wk 1)	Treatment	Feed Day	No	11:50	11:00 - 11:59	Late Morning	37,3		42,6	42,6	18,46		126,26
2019/05/16	CH2205	Male	Three y/o	Treatment (Wk 1)	Treatment	Feed Day	No	11:55	11:00 - 11:59	Late Morning	37,4	152	257,4	257,4	18,55	622,73	189,01
2019/05/16	CH2206	Male	Three y/o	Treatment (Wk 1)	Treatment	Feed Day	No	11:55	11:00 - 11:59	Late Morning	37,2	177	12,6	12,6	18,36	632,55	84,59
2019/05/16	CH2205	Male	Three y/o	Treatment (Wk 1)	Treatment	Feed Day	No	12:00	12:00 - 12:59	Afternoon	37,5	111	162,4	162,4	18,65	601,33	172,80
2019/05/16	CH2206	Male	Three y/o	Treatment (Wk 1)	Treatment	Feed Day	No	12:00	12:00 - 12:59	Afternoon	37,3	90	12,0	12,0	18,46	586,16	82,93
2019/05/16	CH2205	Male	Three y/o	Treatment (Wk 1)	Treatment	Feed Day	No	12:05	12:00 - 12:59	Afternoon	37,6	104	87,8	87,8	18,75	596,69	151,31
2019/05/16	CH2206	Male	Three y/o	Treatment (Wk 1)	Treatment	Feed Day	No	12:05	12:00 - 12:59	Afternoon	37,3	103	82,4	82,4	18,46	596,00	149,10
2019/05/16	CH2205	Male	Three y/o	Treatment (Wk 1)	Treatment	Feed Day	No	12:10	12:00 - 12:59	Afternoon	37,7	85	134,0	134,0	18,85	581,90	166,07
2019/05/16	CH2206	Male	Three y/o	Treatment (Wk 1)	Treatment	Feed Day	No	12:10	12:00 - 12:59	Afternoon	37,4	87	10,0	10,0	18,55	583,64	76,76
2019/05/16	CH2205	Male	Three y/o	Treatment (Wk 1)	Treatment	Feed Day	No	12:15	12:00 - 12:59	Afternoon	37,9	94	129,6	129,6	19,05	589,37	164,90
2019/05/16	CH2206	Male	Three y/o	Treatment (Wk 1)	Treatment	Feed Day	No	12:15	12:00 - 12:59	Afternoon	37,4	88	15,8	15,8	18,55	584,49	92,28
2019/05/16	CH2205	Male	Three y/o	Treatment (Wk 1)	Treatment	Feed Day	No	12:20	12:00 - 12:59	Afternoon	37,9	114	52,0	52,0	19,05	603,20	133,14
2019/05/16	CH2206	Male	Three y/o	Treatment (Wk 1)	Treatment	Feed Day	No	12:20	12:00 - 12:59	Afternoon	37,3	82	27,0	27,0	18,46	579,19	110,58
2019/05/16	CH2205	Male	Three y/o	Treatment (Wk 1)	Treatment	Feed Day	No	12:25	12:00 - 12:59	Afternoon	37,7	89	71,2	71,2	18,85	585,33	144,03
2019/05/16	CH2206	Male	Three y/o	Treatment (Wk 1)	Treatment	Feed Day	No	12:25	12:00 - 12:59	Afternoon	37,4	182	65,0	65,0	18,55	634,31	140,87
2019/05/16	CH2205	Male	Three y/o	Treatment (Wk 1)	Treatment	Feed Day	No	12:30	12:00 - 12:59	Afternoon	37,7	69	24,8	24,8	18,85	565,86	107,67
2019/05/16	CH2206	Male	Three y/o	Treatment (Wk 1)	Treatment	Feed Day	No	12:30	12:00 - 12:59	Afternoon	37,5	95	37,4	37,4	18,65	590,14	121,77
2019/05/16	CH2205	Male	Three y/o	Treatment (Wk 1)	Treatment	Feed Day	No	12:35	12:00 - 12:59	Afternoon	37,7	61	26,8	26,8	18,85	556,01	110,33
2019/05/16	CH2206	Male	Three y/o	Treatment (Wk 1)	Treatment	Feed Day	No	12:35	12:00 - 12:59	Afternoon	37,5	69	35,6	35,6	18,65	565,86	120,08
2019/05/16	CH2205	Male	Three y/o	Treatment (Wk 1)	Treatment	Feed Day	No	12:40	12:00 - 12:59	Afternoon	37,7	67	88,6	88,6	18,85	563,53	151,63
2019/05/16	CH2206	Male	Three y/o	Treatment (Wk 1)	Treatment	Feed Day	No	12:40	12:00 - 12:59	Afternoon	37,5	84	36,2	36,2	18,65	581,01	120,65
2019/05/16	CH2205	Male	Three y/o	Treatment (Wk 1)	Treatment	Feed Day	No	12:45	12:00 - 12:59	Afternoon	37,7	60	4,6	4,6	18,85	554,67	50,61
2019/05/16	CH2206	Male	Three y/o	Treatment (Wk 1)	Treatment	Feed Day	No	12:45	12:00 - 12:59	Afternoon	37,5	79	52,0	52,0	18,65	576,36	133,14
2019/05/16	CH2205	Male	Three y/o	Treatment (Wk 1)	Treatment	Feed Day	No	12:50	12:00 - 12:59	Afternoon	37,7	63	7,0	7,0	18,85	558,62	64,72
2019/05/16	CH2206	Male	Three y/o	Treatment (Wk 1)	Treatment	Feed Day	No	12:50	12:00 - 12:59	Afternoon	37,5	90	40,2	40,2	18,65	586,16	124,26
2019/05/16	CH2205	Male	Three y/o	Treatment (Wk 1)	Treatment	Feed Day	No	12:55	12:00 - 12:59	Afternoon	37,7	52	4,4	4,4	18,85	542,83	49,12
2019/05/16	CH2206	Male	Three y/o	Treatment (Wk 1)	Treatment	Feed Day	No	12:55	12:00 - 12:59	Afternoon	37,7	62	36,0	36,0	18,85	557,33	120,46
2019/05/16	CH2205	Male	Three y/o	Treatment (Wk 1)	Treatment	Feed Day	No	13:00	13:00 - 13:59	Afternoon	37,7	91	15,4	15,4	18,85	586,98	91,41
2019/05/16	CH2206	Male	Three y/o	Treatment (Wk 1)	Treatment	Feed Day	No	13:00	13:00 - 13:59	Afternoon	37,7		78,0	78,0	18,85		147,20
2019/05/16	CH2205	Male	Three y/o	Treatment (Wk 1)	Treatment	Feed Day	No	13:05	13:00 - 13:59	Afternoon	37,7	64	12,8	12,8	18,85	559,88	85,12
2019/05/16	CH2206	Male	Three y/o	Treatment (Wk 1)	Treatment	Feed Day	No	13:05	13:00 - 13:59	Afternoon	37,7	65	12,2	12,2	18,85	561,12	83,49
2019/05/16	CH2205	Male	Three y/o	Treatment (Wk 1)	Treatment	Feed Day	No	13:10	13:00 - 13:59	Afternoon	37,8	70	13,6	13,6	18,95	566,99	87,18
2019/05/16	CH2206	Male	Three y/o	Treatment (Wk 1)	Treatment	Feed Day	No	13:10	13:00 - 13:59	Afternoon	37,8	89	10,6	10,6	18,95	585,33	78,73
2019/05/16	CH2205	Male	Three y/o	Treatment (Wk 1)	Treatment	Feed Day	No	13:15	13:00 - 13:59	Afternoon	37,8	66	13,8	13,8	18,95	562,34	87,68
2019/05/16	CH2206	Male	Three y/o	Treatment (Wk 1)	Treatment	Feed Day	No	13:15	13:00 - 13:59	Afternoon	37,8	87	14,4	14,4	18,95	583,64	89,12
2019/05/16	CH2205	Male	Three y/o	Treatment (Wk 1)	Treatment	Feed Day	No	13:20	13:00 - 13:59	Afternoon	37,8	55	15,6	15,6	18,95	547,52	91,85
2019/05/16	CH2206	Male	Three y/o	Treatment (Wk 1)	Treatment	Feed Day	No	13:20	13:00 - 13:59	Afternoon	37,8	87	11,2	11,2	18,95	583,64	80,59
2019/05/16	CH2205	Male	Three y/o	Treatment (Wk 1)	Treatment	Feed Day	No	13:25	13:00 - 13:59	Afternoon	37,7		16,6	16,6	18,85		93,96
2019/05/16	CH2206	Male	Three y/o	Treatment (Wk 1)	Treatment	Feed Day	No	13:25	13:00 - 13:59	Afternoon	37,8	78	15,8	15,8	18,95	575,38	92,28
2019/05/16	CH2205	Male	Three y/o	Treatment (Wk 1)	Treatment	Feed Day	No	13:30	13:00 - 13:59	Afternoon	37,7	78	16,8	16,8	18,85	575,38	94,37
2019/05/16	CH2206	Male	Three y/o	Treatment (Wk 1)	Treatment	Feed Day	No	13:30	13:00 - 13:59	Afternoon	37,8	78	11,0	11,0	18,95	575,38	79,98
2019/05/16	CH2205	Male	Three y/o	Treatment (Wk 1)	Treatment	Feed Day	No	13:35	13:00 - 13:59	Afternoon	37,7	60	20,2	20,2	18,85	554,67	100,65
2019/05/16	CH2206	Male	Three y/o	Treatment (Wk 1)	Treatment	Feed Day	No	13:35	13:00 - 13:59	Afternoon	37,8	76	13,2	13,2	18,95	573,39	86,17
2019/05/16	CH2205	Male	Three y/o	Treatment (Wk 1)	Treatment	Feed Day	No	13:40	13:00 - 13:59	Afternoon	37,7	57	15,8	15,8	18,85	550,47	92,28
2019/05/16	CH2206	Male	Three y/o	Treatment (Wk 1)	Treatment	Feed Day	No	13:40	13:00 - 13:59	Afternoon	37,8	78	16,8	16,8	18,95	575,38	94,37
2019/05/16	CH2205	Male	Three y/o	Treatment (Wk 1)	Treatment	Feed Day	No	13:45	13:00 - 13:59	Afternoon	37,7		20,8	20,8	18,85		101,65

2019/05/16	CH2206	Male	Three y/o	Treatment (Wk 1)	Treatment	Feed Day	No	13:45	13:00 - 13:59	Afternoon	37,8	94	23,2	23,2	18,95	589,37	105,39
2019/05/16	CH2205	Male	Three y/o	Treatment (Wk 1)	Treatment	Feed Day	No	13:50	13:00 - 13:59	Afternoon	37,7	89	27,2	27,2	18,85	585,33	110,83
2019/05/16	CH2206	Male	Three y/o	Treatment (Wk 1)	Treatment	Feed Day	No	13:50	13:00 - 13:59	Afternoon	37,8	90	35,6	35,6	18,95	586,16	120,08
2019/05/16	CH2205	Male	Three y/o	Treatment (Wk 1)	Treatment	Feed Day	No	13:55	13:00 - 13:59	Afternoon	37,7	61	27,2	27,2	18,85	556,01	110,83
2019/05/16	CH2206	Male	Three y/o	Treatment (Wk 1)	Treatment	Feed Day	No	13:55	13:00 - 13:59	Afternoon	37,8	90	40,2	40,2	18,95	586,16	124,26
2019/05/16	CH2205	Male	Three y/o	Treatment (Wk 1)	Treatment	Feed Day	No	14:00	14:00 - 14:59	Afternoon	37,7	88	29,0	29,0	18,85	584,49	113,03
2019/05/16	CH2206	Male	Three y/o	Treatment (Wk 1)	Treatment	Feed Day	No	14:00	14:00 - 14:59	Afternoon	37,8	112	12,4	12,4	18,95	601,96	84,04
2019/05/16	CH2205	Male	Three y/o	Treatment (Wk 1)	Treatment	Feed Day	No	14:05	14:00 - 14:59	Afternoon	37,7	64	25,2	25,2	18,85	559,88	108,22
2019/05/16	CH2206	Male	Three y/o	Treatment (Wk 1)	Treatment	Feed Day	No	14:05	14:00 - 14:59	Afternoon	37,9	96	18,8	18,8	19,05	590,91	98,20
2019/05/16	CH2205	Male	Three y/o	Treatment (Wk 1)	Treatment	Feed Day	No	14:10	14:00 - 14:59	Afternoon	37,7	65	27,8	27,8	18,85	561,12	111,58
2019/05/16	CH2206	Male	Three y/o	Treatment (Wk 1)	Treatment	Feed Day	No	14:10	14:00 - 14:59	Afternoon	37,9	92	8,2	8,2	19,05	587,78	70,05
2019/05/16	CH2205	Male	Three y/o	Treatment (Wk 1)	Treatment	Feed Day	No	14:15	14:00 - 14:59	Afternoon	37,7	71	25,4	25,4	18,85	568,10	108,49
2019/05/16	CH2206	Male	Three y/o	Treatment (Wk 1)	Treatment	Feed Day	No	14:15	14:00 - 14:59	Afternoon	37,9	86	10,6	10,6	19,05	582,77	78,73
2019/05/16	CH2205	Male	Three y/o	Treatment (Wk 1)	Treatment	Feed Day	No	14:20	14:00 - 14:59	Afternoon	37,7	75	29,4	29,4	18,85	572,36	113,50
2019/05/16	CH2206	Male	Three y/o	Treatment (Wk 1)	Treatment	Feed Day	No	14:20	14:00 - 14:59	Afternoon	37,9	100	8,6	8,6	19,05	593,87	71,66
2019/05/16	CH2205	Male	Three y/o	Treatment (Wk 1)	Treatment	Feed Day	No	14:25	14:00 - 14:59	Afternoon	37,7	63	25,6	25,6	18,85	558,62	108,76
2019/05/16	CH2206	Male	Three y/o	Treatment (Wk 1)	Treatment	Feed Day	No	14:25	14:00 - 14:59	Afternoon	37,9	74	14,4	14,4	19,05	571,33	89,12
2019/05/16	CH2205	Male	Three y/o	Treatment (Wk 1)	Treatment	Feed Day	No	14:30	14:00 - 14:59	Afternoon	37,7	58	27,4	27,4	18,85	551,90	111,09
2019/05/16	CH2206	Male	Three y/o	Treatment (Wk 1)	Treatment	Feed Day	No	14:30	14:00 - 14:59	Afternoon	37,9	90	24,0	24,0	19,05	586,16	106,55
2019/05/16	CH2205	Male	Three y/o	Treatment (Wk 1)	Treatment	Feed Day	No	14:35	14:00 - 14:59	Afternoon	37,7	66	56,2	56,2	18,85	562,34	135,83
2019/05/16	CH2206	Male	Three y/o	Treatment (Wk 1)	Treatment	Feed Day	No	14:35	14:00 - 14:59	Afternoon	37,9	83	29,8	29,8	19,05	580,11	113,97
2019/05/16	CH2205	Male	Three y/o	Treatment (Wk 1)	Treatment	Feed Day	No	14:40	14:00 - 14:59	Afternoon	37,9	67	16,2	16,2	19,05	563,53	93,13
2019/05/16	CH2206	Male	Three y/o	Treatment (Wk 1)	Treatment	Feed Day	No	14:40	14:00 - 14:59	Afternoon	37,9	83	52,6	52,6	19,05	580,11	133,54
2019/05/16	CH2205	Male	Three y/o	Treatment (Wk 1)	Treatment	Feed Day	No	14:45	14:00 - 14:59	Afternoon	37,9	61	20,4	20,4	19,05	556,01	100,99
2019/05/16	CH2206	Male	Three y/o	Treatment (Wk 1)	Treatment	Feed Day	No	14:45	14:00 - 14:59	Afternoon	37,9	94	43,4	43,4	19,05	589,37	126,90
2019/05/16	CH2205	Male	Three y/o	Treatment (Wk 1)	Treatment	Feed Day	No	14:50	14:00 - 14:59	Afternoon	37,9	70	20,2	20,2	19,05	566,99	100,65
2019/05/16	CH2206	Male	Three y/o	Treatment (Wk 1)	Treatment	Feed Day	No	14:50	14:00 - 14:59	Afternoon	37,9	81	30,2	30,2	19,05	578,26	114,42
2019/05/16	CH2205	Male	Three y/o	Treatment (Wk 1)	Treatment	Feed Day	No	14:55	14:00 - 14:59	Afternoon	37,9	78	20,4	20,4	19,05	575,38	100,99
2019/05/16	CH2206	Male	Three y/o	Treatment (Wk 1)	Treatment	Feed Day	No	14:55	14:00 - 14:59	Afternoon	37,9	85	15,0	15,0	19,05	581,90	90,51
2019/05/16	CH2205	Male	Three y/o	Treatment (Wk 1)	Treatment	Feed Day	No	15:00	15:00 - 15:59	Afternoon	37,9	45	18,6	18,6	19,05	530,47	97,84
2019/05/16	CH2206	Male	Three y/o	Treatment (Wk 1)	Treatment	Feed Day	No	15:00	15:00 - 15:59	Afternoon	37,9	92	24,6	24,6	19,05	587,78	107,39
2019/05/16	CH2205	Male	Three y/o	Treatment (Wk 1)	Treatment	Feed Day	No	15:05	15:00 - 15:59	Afternoon	37,9	66	22,2	22,2	19,05	562,34	103,88
2019/05/16	CH2206	Male	Three y/o	Treatment (Wk 1)	Treatment	Feed Day	No	15:05	15:00 - 15:59	Afternoon	37,9	75	27,8	27,8	19,05	572,36	111,58
2019/05/16	CH2205	Male	Three y/o	Treatment (Wk 1)	Treatment	Feed Day	No	15:10	15:00 - 15:59	Afternoon	37,9	66	14,8	14,8	19,05	562,34	90,05
2019/05/16	CH2206	Male	Three y/o	Treatment (Wk 1)	Treatment	Feed Day	No	15:10	15:00 - 15:59	Afternoon	37,9	85	26,6	26,6	19,05	581,90	110,07
2019/05/16	CH2205	Male	Three y/o	Treatment (Wk 1)	Treatment	Feed Day	No	15:15	15:00 - 15:59	Afternoon	37,9	61	21,0	21,0	19,05	556,01	101,98
2019/05/16	CH2206	Male	Three y/o	Treatment (Wk 1)	Treatment	Feed Day	No	15:15	15:00 - 15:59	Afternoon	37,9	80	28,2	28,2	19,05	577,31	112,07
2019/05/16	CH2205	Male	Three y/o	Treatment (Wk 1)	Treatment	Feed Day	No	15:20	15:00 - 15:59	Afternoon	37,9	45	17,8	17,8	19,05	530,47	96,34
2019/05/16	CH2206	Male	Three y/o	Treatment (Wk 1)	Treatment	Feed Day	No	15:20	15:00 - 15:59	Afternoon	37,9	63	33,0	33,0	19,05	558,62	117,47
2019/05/16	CH2205	Male	Three y/o	Treatment (Wk 1)	Treatment	Feed Day	No	15:25	15:00 - 15:59	Afternoon	37,9	64	18,8	18,8	19,05	559,88	98,20
2019/05/16	CH2206	Male	Three y/o	Treatment (Wk 1)	Treatment	Feed Day	No	15:25	15:00 - 15:59	Afternoon	37,9	88	33,8	33,8	19,05	584,49	118,29
2019/05/16	CH2205	Male	Three y/o	Treatment (Wk 1)	Treatment	Feed Day	No	15:30	15:00 - 15:59	Afternoon	37,9	70	19,6	19,6	19,05	566,99	99,63
2019/05/16	CH2206	Male	Three y/o	Treatment (Wk 1)	Treatment	Feed Day	No	15:30	15:00 - 15:59	Afternoon	37,9	57	31,6	31,6	19,05	550,47	115,98
2019/05/16	CH2205	Male	Three y/o	Treatment (Wk 1)	Treatment	Feed Day	No	15:35	15:00 - 15:59	Afternoon	37,9	79	19,8	19,8	19,05	576,36	99,97
2019/05/16	CH2206	Male	Three y/o	Treatment (Wk 1)	Treatment	Feed Day	No	15:35	15:00 - 15:59	Afternoon	38,0	50	37,4	37,4	19,15	539,52	121,77
2019/05/16	CH2205	Male	Three y/o	Treatment (Wk 1)	Treatment	Feed Day	No	15:40	15:00 - 15:59	Afternoon	37,9	64	19,2	19,2	19,05	559,88	98,92
2019/05/16	CH2206	Male	Three y/o	Treatment (Wk 1)	Treatment	Feed Day	No	15:40	15:00 - 15:59	Afternoon	38,0	86	38,4	38,4	19,15	582,77	122,68
2019/05/16	CH2205	Male	Three y/o	Treatment (Wk 1)	Treatment	Feed Day	No	15:45	15:00 - 15:59	Afternoon	37,9	61	18,4	18,4	19,05	556,01	97,47
2019/05/16	CH2206	Male	Three y/o	Treatment (Wk 1)	Treatment	Feed Day	No	15:45	15:00 - 15:59	Afternoon	38,0	118	48,4	48,4	19,15	605,61	130,66
2019/05/16	CH2205	Male	Three y/o	Treatment (Wk 1)	Treatment	Feed Day	No	15:50	15:00 - 15:59	Afternoon	37,9	68	15,4	15,4	19,05	564,71	91,41
2019/05/16	CH2206	Male	Three y/o	Treatment (Wk 1)	Treatment	Feed Day	No	15:50	15:00 - 15:59	Afternoon	38,0	68	40,8	40,8	19,15	564,71	124,77
2019/05/16	CH2205	Male	Three y/o	Treatment (Wk 1)	Treatment	Feed Day	No	15:55	15:00 - 15:59	Afternoon	38,0	76	9,6	9,6	19,15	573,39	75,37
2019/05/16	CH2206	Male	Three y/o	Treatment (Wk 1)	Treatment	Feed Day	No	15:55	15:00 - 15:59	Afternoon	38,0	73	25,2	25,2	19,15	570,27	108,22
2019/05/16	CH2205	Male	Three y/o	Treatment (Wk 1)	Treatment	Feed Day	No	16:00	16:00 - 16:59	Evening	38,0	43	3,8	3,8	19,15	526,50	44,22
2019/05/16	CH2206	Male	Three y/o	Treatment (Wk 1)	Treatment	Feed Day	No	16:00	16:00 - 16:59	Evening	38,0	77	20,2	20,2	19,15	574,39	100,65
2019/05/16	CH2205	Male	Three y/o	Treatment (Wk 1)	Treatment	Feed Day	No	16:05	16:00 - 16:59	Evening	38,0	102	12,4	12,4	19,15	595,30	84,04
2019/05/16	CH2206	Male	Three y/o	Treatment (Wk 1)	Treatment	Feed Day	No	16:05	16:00 - 16:59	Evening	38,0	86	48,4	48,4	19,15	582,77	130,66
2019/05/16	CH2205	Male	Three y/o	Treatment (Wk 1)	Treatment	Feed Day	No	16:10	16:00 - 16:59	Evening	38,0	68	19,6	19,6	19,15	564,71	99,63
2019/05/16	CH2206	Male	Three y/o	Treatment (Wk 1)	Treatment	Feed Day	No	16:10	16:00 - 16:59	Evening	38,0	88	40,2	40,2	19,15	584,49	124,26
2019/05/16	CH2205	Male	Three y/o	Treatment (Wk 1)	Treatment	Feed Day	No	16:15	16:00 - 16:59	Evening	38,0	75	49,8	49,8	19,15	572,36	131,65
2019/05/16	CH2206	Male	Three y/o	Treatment (Wk 1)	Treatment	Feed Day	No	16:15	16:00 - 16:59	Evening	37,9	86	41,8	41,8	19,05	582,77	125,60
2019/05/16	CH2205	Male	Three y/o	Treatment (Wk 1)	Treatment	Feed Day	No	16:20	16:00 - 16:59	Evening	38,0	35	113,2	113,2	19,15	507,97	160,17
2019/05/16	CH2206	Male	Three y/o	Treatment (Wk 1)	Treatment	Feed Day	No	16:20	16:00 - 16:59	Evening	38,0	101	101,4	101,4	19,15	594,59	156,33
2019/05/16	CH2205	Male	Three y/o	Treatment (Wk 1)	Treatment	Feed Day	No	16:25	16:00 - 16:59	Evening	37,9	145	119,8	119,8	19,05	619,62	162,15
2019/05/16	CH2206	Male	Three y/o	Treatment (Wk 1)	Treatment	Feed Day	No	16:25	16:00 - 16:59	Evening	37,9	84	114,8	114,8	19,05	581,01	160,66
2019/05/16	CH2205	Male	Three y/o	Treatment (Wk 1)	Treatment	Feed Day	No	16:30	16:00 - 16:59	Evening	37,7		56,0	56,0	18,85		135,70
2019/05/16	CH2206	Male	Three y/o	Treatment (Wk 1)	Treatment	Feed Day	No	16:30	16:00 - 16:59	Evening	37,8	104	34,4	34,4	18,95	596,69	118,90
2019/05/16	CH2205	Male	Three y/o	Treatment (Wk 1)	Treatment	Feed Day	No	16:35	16:00 - 16:59	Evening	37,7	100	168,2	168,2	18,85	593,87	174,03
2019/05/16	CH2206	Male	Three y/o	Treatment (Wk 1)	Treatment	Feed Day	No	16:35	16:00 - 16:59	Evening	37,9	96	51,8	51,8	19,05	590,91	133,01
2019/05/16	CH2205	Male	Three y/o	Treatment (Wk 1)	Treatment	Feed Day	No	16:40	16:00 - 16:59	Evening	37,7		128,4	128,4	18,85		164,57

2019/05/16	CH2206	Male	Three y/o	Treatment (Wk 1)	Treatment	Feed Day	No	16:40	16:00 - 16:59	Evening	37,8	73	26,0	26,0	18,95	570,27	109,29
2019/05/16	CH2205	Male	Three y/o	Treatment (Wk 1)	Treatment	Feed Day	No	16:45	16:00 - 16:59	Evening	37,9	129	67,6	67,6	19,05	611,75	142,23
2019/05/16	CH2206	Male	Three y/o	Treatment (Wk 1)	Treatment	Feed Day	No	16:45	16:00 - 16:59	Evening	37,9	74	54,8	54,8	19,05	571,33	134,96
2019/05/16	CH2205	Male	Three y/o	Treatment (Wk 1)	Treatment	Feed Day	No	16:50	16:00 - 16:59	Evening	37,9	70	29,6	29,6	19,05	566,99	113,73
2019/05/16	CH2206	Male	Three y/o	Treatment (Wk 1)	Treatment	Feed Day	No	16:50	16:00 - 16:59	Evening	37,9	68	38,4	38,4	19,05	564,71	122,68
2019/05/16	CH2205	Male	Three y/o	Treatment (Wk 1)	Treatment	Feed Day	No	16:55	16:00 - 16:59	Evening	37,9	95	24,2	24,2	19,05	590,14	106,83
2019/05/16	CH2206	Male	Three y/o	Treatment (Wk 1)	Treatment	Feed Day	No	16:55	16:00 - 16:59	Evening	37,9	77	29,2	29,2	19,05	574,39	113,27
2019/05/16	CH2205	Male	Three y/o	Treatment (Wk 1)	Treatment	Feed Day	No	17:00	17:00 - 17:59	Evening	37,9	145	95,4	95,4	19,05	619,62	154,20
2019/05/16	CH2206	Male	Three y/o	Treatment (Wk 1)	Treatment	Feed Day	No	17:00	17:00 - 17:59	Evening	38,0	120	25,2	25,2	19,15	606,78	108,22
2019/05/16	CH2205	Male	Three y/o	Treatment (Wk 1)	Treatment	Feed Day	No	17:05	17:00 - 17:59	Evening	37,9	69	14,8	14,8	19,05	565,86	90,05
2019/05/16	CH2206	Male	Three y/o	Treatment (Wk 1)	Treatment	Feed Day	No	17:05	17:00 - 17:59	Evening	37,9	112	64,6	64,6	19,05	601,96	140,65
2019/05/16	CH2205	Male	Three y/o	Treatment (Wk 1)	Treatment	Feed Day	No	17:10	17:00 - 17:59	Evening	37,9	61	10,8	10,8	19,05	556,01	79,36
2019/05/16	CH2206	Male	Three y/o	Treatment (Wk 1)	Treatment	Feed Day	No	17:10	17:00 - 17:59	Evening	37,9	73	27,8	27,8	19,05	570,27	111,58
2019/05/16	CH2205	Male	Three y/o	Treatment (Wk 1)	Treatment	Feed Day	No	17:15	17:00 - 17:59	Evening	38,0	88	207,4	207,4	19,15	584,49	181,40
2019/05/16	CH2206	Male	Three y/o	Treatment (Wk 1)	Treatment	Feed Day	No	17:15	17:00 - 17:59	Evening	37,9	92	63,4	63,4	19,05	587,78	140,00
2019/05/16	CH2205	Male	Three y/o	Treatment (Wk 1)	Treatment	Feed Day	No	17:20	17:00 - 17:59	Evening	38,0	80	56,6	56,6	19,15	577,31	136,07
2019/05/16	CH2206	Male	Three y/o	Treatment (Wk 1)	Treatment	Feed Day	No	17:20	17:00 - 17:59	Evening	37,9	120	65,4	65,4	19,05	606,78	141,08
2019/05/16	CH2205	Male	Three y/o	Treatment (Wk 1)	Treatment	Feed Day	No	17:25	17:00 - 17:59	Evening	38,0	88	6,6	6,6	19,15	584,49	62,73
2019/05/16	CH2206	Male	Three y/o	Treatment (Wk 1)	Treatment	Feed Day	No	17:25	17:00 - 17:59	Evening	37,9	82	42,6	42,6	19,05	579,19	126,26
2019/05/16	CH2205	Male	Three y/o	Treatment (Wk 1)	Treatment	Feed Day	No	17:30	17:00 - 17:59	Evening	37,9		7,6	7,6	19,05		67,49
2019/05/16	CH2206	Male	Three y/o	Treatment (Wk 1)	Treatment	Feed Day	No	17:30	17:00 - 17:59	Evening	37,9	51	54,8	54,8	19,05	541,20	134,96
2019/05/16	CH2205	Male	Three y/o	Treatment (Wk 1)	Treatment	Feed Day	No	17:35	17:00 - 17:59	Evening	37,9	73	5,8	5,8	19,05	570,27	58,39
2019/05/16	CH2206	Male	Three y/o	Treatment (Wk 1)	Treatment	Feed Day	No	17:35	17:00 - 17:59	Evening	37,8	84	52,4	52,4	18,95	581,01	133,41
2019/05/16	CH2205	Male	Three y/o	Treatment (Wk 1)	Treatment	Feed Day	No	17:40	17:00 - 17:59	Evening	37,9	63	7,4	7,4	19,05	558,62	66,59
2019/05/16	CH2206	Male	Three y/o	Treatment (Wk 1)	Treatment	Feed Day	No	17:40	17:00 - 17:59	Evening	37,8	50	45,2	45,2	18,95	539,52	128,30
2019/05/16	CH2205	Male	Three y/o	Treatment (Wk 1)	Treatment	Feed Day	No	17:45	17:00 - 17:59	Evening	38,0	54	6,8	6,8	19,15	545,99	63,74
2019/05/16	CH2206	Male	Three y/o	Treatment (Wk 1)	Treatment	Feed Day	No	17:45	17:00 - 17:59	Evening	37,8	62	44,6	44,6	18,95	557,33	127,84
2019/05/16	CH2205	Male	Three y/o	Treatment (Wk 1)	Treatment	Feed Day	No	17:50	17:00 - 17:59	Evening	38,0	78	6,0	6,0	19,15	575,38	59,53
2019/05/16	CH2206	Male	Three y/o	Treatment (Wk 1)	Treatment	Feed Day	No	17:50	17:00 - 17:59	Evening	37,8	82	41,0	41,0	18,95	579,19	124,94
2019/05/16	CH2205	Male	Three y/o	Treatment (Wk 1)	Treatment	Feed Day	No	17:55	17:00 - 17:59	Evening	38,0	74	5,8	5,8	19,15	571,33	58,39
2019/05/16	CH2206	Male	Three y/o	Treatment (Wk 1)	Treatment	Feed Day	No	17:55	17:00 - 17:59	Evening	37,9	51	26,0	26,0	19,05	541,20	109,29
2019/05/16	CH2205	Male	Three y/o	Treatment (Wk 1)	Treatment	Feed Day	No	18:00	18:00 - 18:59	Evening	38,0	86	2,8	2,8	19,15	582,77	34,04
2019/05/16	CH2206	Male	Three y/o	Treatment (Wk 1)	Treatment	Feed Day	No	18:00	18:00 - 18:59	Evening	38,0	87	30,4	30,4	19,15	583,64	114,65
2019/05/16	CH2205	Male	Three y/o	Treatment (Wk 1)	Treatment	Feed Day	No	18:05	18:00 - 18:59	Evening	38,0	48	3,4	3,4	19,15	536,04	40,51
2019/05/16	CH2206	Male	Three y/o	Treatment (Wk 1)	Treatment	Feed Day	No	18:05	18:00 - 18:59	Evening	38,0	86	26,6	26,6	19,15	582,77	110,07
2019/05/16	CH2205	Male	Three y/o	Treatment (Wk 1)	Treatment	Feed Day	No	18:10	18:00 - 18:59	Evening	37,9	90	3,6	3,6	19,05	586,16	42,42
2019/05/16	CH2206	Male	Three y/o	Treatment (Wk 1)	Treatment	Feed Day	No	18:10	18:00 - 18:59	Evening	38,0	57	19,2	19,2	19,15	550,47	98,92
2019/05/16	CH2205	Male	Three y/o	Treatment (Wk 1)	Treatment	Feed Day	No	18:15	18:00 - 18:59	Evening	37,9	53	5,2	5,2	19,05	544,43	54,72
2019/05/16	CH2206	Male	Three y/o	Treatment (Wk 1)	Treatment	Feed Day	No	18:15	18:00 - 18:59	Evening	38,0	101	12,8	12,8	19,15	594,59	85,12
2019/05/16	CH2205	Male	Three y/o	Treatment (Wk 1)	Treatment	Feed Day	No	18:20	18:00 - 18:59	Evening	38,0	57	4,2	4,2	19,15	550,47	47,57
2019/05/16	CH2206	Male	Three y/o	Treatment (Wk 1)	Treatment	Feed Day	No	18:20	18:00 - 18:59	Evening	38,1	102	49,4	49,4	19,26	595,30	131,37
2019/05/16	CH2205	Male	Three y/o	Treatment (Wk 1)	Treatment	Feed Day	No	18:25	18:00 - 18:59	Evening	38,0	64	3,2	3,2	19,15	559,88	38,49
2019/05/16	CH2206	Male	Three y/o	Treatment (Wk 1)	Treatment	Feed Day	No	18:25	18:00 - 18:59	Evening	38,0	72	39,2	39,2	19,15	569,20	123,39
2019/05/16	CH2205	Male	Three y/o	Treatment (Wk 1)	Treatment	Feed Day	No	18:30	18:00 - 18:59	Evening	38,0	64	5,4	5,4	19,15	559,88	55,99
2019/05/16	CH2206	Male	Three y/o	Treatment (Wk 1)	Treatment	Feed Day	No	18:30	18:00 - 18:59	Evening	37,9	77	35,2	35,2	19,05	574,39	119,69
2019/05/16	CH2205	Male	Three y/o	Treatment (Wk 1)	Treatment	Feed Day	No	18:35	18:00 - 18:59	Evening	38,0	54	7,6	7,6	19,15	545,99	67,49
2019/05/16	CH2206	Male	Three y/o	Treatment (Wk 1)	Treatment	Feed Day	No	18:35	18:00 - 18:59	Evening	38,0	88	33,2	33,2	19,15	584,49	117,68
2019/05/16	CH2205	Male	Three y/o	Treatment (Wk 1)	Treatment	Feed Day	No	18:40	18:00 - 18:59	Evening	38,0	61	6,4	6,4	19,15	556,01	61,70
2019/05/16	CH2206	Male	Three y/o	Treatment (Wk 1)	Treatment	Feed Day	No	18:40	18:00 - 18:59	Evening	38,0	73	26,8	26,8	19,15	570,27	110,33
2019/05/16	CH2205	Male	Three y/o	Treatment (Wk 1)	Treatment	Feed Day	No	18:45	18:00 - 18:59	Evening	38,1	46	10,6	10,6	19,26	532,38	78,73
2019/05/16	CH2206	Male	Three y/o	Treatment (Wk 1)	Treatment	Feed Day	No	18:45	18:00 - 18:59	Evening	37,9	52	47,6	47,6	19,05	542,83	130,09
2019/05/16	CH2205	Male	Three y/o	Treatment (Wk 1)	Treatment	Feed Day	No	18:50	18:00 - 18:59	Evening	38,1	65	9,2	9,2	19,26	561,12	73,94
2019/05/16	CH2206	Male	Three y/o	Treatment (Wk 1)	Treatment	Feed Day	No	18:50	18:00 - 18:59	Evening	37,9	68	59,0	59,0	19,05	564,71	137,51
2019/05/16	CH2205	Male	Three y/o	Treatment (Wk 1)	Treatment	Feed Day	No	18:55	18:00 - 18:59	Evening	38,0		3,8	3,8	19,15		44,22
2019/05/16	CH2206	Male	Three y/o	Treatment (Wk 1)	Treatment	Feed Day	No	18:55	18:00 - 18:59	Evening	37,9	99	57,4	57,4	19,05	593,14	136,56
2019/05/16	CH2205	Male	Three y/o	Treatment (Wk 1)	Treatment	Feed Day	No	19:00	19:00 - 19:59	Evening	38,0		8,4	8,4	19,15		70,86
2019/05/16	CH2206	Male	Three y/o	Treatment (Wk 1)	Treatment	Feed Day	No	19:00	19:00 - 19:59	Evening	37,9	87	50,0	50,0	19,05	583,64	131,79
2019/05/16	CH2205	Male	Three y/o	Treatment (Wk 1)	Treatment	Feed Day	No	19:05	19:00 - 19:59	Evening	38,0	75	12,4	12,4	19,15	572,36	84,04
2019/05/16	CH2206	Male	Three y/o	Treatment (Wk 1)	Treatment	Feed Day	No	19:05	19:00 - 19:59	Evening	37,9	47	52,6	52,6	19,05	534,23	133,54
2019/05/16	CH2205	Male	Three y/o	Treatment (Wk 1)	Treatment	Feed Day	No	19:10	19:00 - 19:59	Evening	38,0	83	8,6	8,6	19,15	580,11	71,66
2019/05/16	CH2206	Male	Three y/o	Treatment (Wk 1)	Treatment	Feed Day	No	19:10	19:00 - 19:59	Evening	37,9	90	49,6	49,6	19,05	586,16	131,51
2019/05/16	CH2205	Male	Three y/o	Treatment (Wk 1)	Treatment	Feed Day	No	19:15	19:00 - 19:59	Evening	37,9	53	8,8	8,8	19,05	544,43	72,43
2019/05/16	CH2206	Male	Three y/o	Treatment (Wk 1)	Treatment	Feed Day	No	19:15	19:00 - 19:59	Evening	37,9	79	50,8	50,8	19,05	576,36	132,33
2019/05/16	CH2205	Male	Three y/o	Treatment (Wk 1)	Treatment	Feed Day	No	19:20	19:00 - 19:59	Evening	37,9	82	9,6	9,6	19,05	579,19	75,37
2019/05/16	CH2206	Male	Three y/o	Treatment (Wk 1)	Treatment	Feed Day	No	19:20	19:00 - 19:59	Evening	38,0	90	60,2	60,2	19,15	586,16	138,21
2019/05/16	CH2205	Male	Three y/o	Treatment (Wk 1)	Treatment	Feed Day	No	19:25	19:00 - 19:59	Evening	38,0	65	15,2	15,2	19,15	561,12	90,96
2019/05/16	CH2206	Male	Three y/o	Treatment (Wk 1)	Treatment	Feed Day	No	19:25	19:00 - 19:59	Evening	37,9	78	52,6	52,6	19,05	575,38	133,54
2019/05/16	CH2205	Male	Three y/o	Treatment (Wk 1)	Treatment	Feed Day	No	19:30	19:00 - 19:59	Evening	38,0	73	14,6	14,6	19,15	570,27	89,59
2019/05/16	CH2206	Male	Three y/o	Treatment (Wk 1)	Treatment	Feed Day	No	19:30	19:00 - 19:59	Evening	37,8	123	53,0	53,0	18,95	608,49	133,80
2019/05/16	CH2205	Male	Three y/o	Treatment (Wk 1)	Treatment	Feed Day	No	19:35	19:00 - 19:59	Evening	38,1	59	11,2	11,2	19,26	553,30	80,59

2019/05/16	CH2206	Male	Three y/o	Treatment (Wk 1)	Treatment	Feed Day	No	19:35	19:00 - 19:59	Evening	37,6	71	61,0	61,0	18,75	568,10	138,67
2019/05/16	CH2205	Male	Three y/o	Treatment (Wk 1)	Treatment	Feed Day	No	19:40	19:00 - 19:59	Evening	38,1	72	13,0	13,0	19,26	569,20	85,65
2019/05/16	CH2206	Male	Three y/o	Treatment (Wk 1)	Treatment	Feed Day	No	19:40	19:00 - 19:59	Evening	37,5	88	62,2	62,2	18,65	584,49	139,34
2019/05/16	CH2205	Male	Three y/o	Treatment (Wk 1)	Treatment	Feed Day	No	19:45	19:00 - 19:59	Evening	38,1	64	8,8	8,8	19,26	559,88	72,43
2019/05/16	CH2206	Male	Three y/o	Treatment (Wk 1)	Treatment	Feed Day	No	19:45	19:00 - 19:59	Evening	37,4	79	60,6	60,6	18,55	576,36	138,44
2019/05/16	CH2205	Male	Three y/o	Treatment (Wk 1)	Treatment	Feed Day	No	19:50	19:00 - 19:59	Evening	38,1	80	10,8	10,8	19,26	577,31	79,36
2019/05/16	CH2206	Male	Three y/o	Treatment (Wk 1)	Treatment	Feed Day	No	19:50	19:00 - 19:59	Evening	37,4	88	60,4	60,4	18,55	584,49	138,32
2019/05/16	CH2205	Male	Three y/o	Treatment (Wk 1)	Treatment	Feed Day	No	19:55	19:00 - 19:59	Evening	38,2	89	9,4	9,4	19,36	585,33	74,66
2019/05/16	CH2206	Male	Three y/o	Treatment (Wk 1)	Treatment	Feed Day	No	19:55	19:00 - 19:59	Evening	37,4	77	51,8	51,8	18,55	574,39	133,01
2019/05/16	CH2205	Male	Three y/o	Treatment (Wk 1)	Treatment	Feed Day	No	20:00	20:00 - 20:59	Night	38,2	75	7,6	7,6	19,36	572,36	67,49
2019/05/16	CH2206	Male	Three y/o	Treatment (Wk 1)	Treatment	Feed Day	No	20:00	20:00 - 20:59	Night	37,7	98	37,0	37,0	18,85	592,41	121,40
2019/05/16	CH2205	Male	Three y/o	Treatment (Wk 1)	Treatment	Feed Day	No	20:05	20:00 - 20:59	Night	38,2	53	7,4	7,4	19,36	544,43	66,59
2019/05/16	CH2206	Male	Three y/o	Treatment (Wk 1)	Treatment	Feed Day	No	20:05	20:00 - 20:59	Night	37,9	51	38,6	38,6	19,05	541,20	122,86
2019/05/16	CH2205	Male	Three y/o	Treatment (Wk 1)	Treatment	Feed Day	No	20:10	20:00 - 20:59	Night	38,2	59	14,8	14,8	19,36	553,30	90,05
2019/05/16	CH2206	Male	Three y/o	Treatment (Wk 1)	Treatment	Feed Day	No	20:10	20:00 - 20:59	Night	37,9	84	19,6	19,6	19,05	581,01	99,63
2019/05/16	CH2205	Male	Three y/o	Treatment (Wk 1)	Treatment	Feed Day	No	20:15	20:00 - 20:59	Night	38,2	61	8,2	8,2	19,36	556,01	70,05
2019/05/16	CH2206	Male	Three y/o	Treatment (Wk 1)	Treatment	Feed Day	No	20:15	20:00 - 20:59	Night	37,9	46	9,0	9,0	19,05	532,38	73,19
2019/05/16	CH2205	Male	Three y/o	Treatment (Wk 1)	Treatment	Feed Day	No	20:20	20:00 - 20:59	Night	38,2	49	10,6	10,6	19,36	537,80	78,73
2019/05/16	CH2206	Male	Three y/o	Treatment (Wk 1)	Treatment	Feed Day	No	20:20	20:00 - 20:59	Night	38,0	60	12,0	12,0	19,15	554,67	82,93
2019/05/16	CH2205	Male	Three y/o	Treatment (Wk 1)	Treatment	Feed Day	No	20:25	20:00 - 20:59	Night	38,2	86	16,8	16,8	19,36	582,77	94,37
2019/05/16	CH2206	Male	Three y/o	Treatment (Wk 1)	Treatment	Feed Day	No	20:25	20:00 - 20:59	Night	38,0	95	15,2	15,2	19,15	590,14	90,96
2019/05/16	CH2205	Male	Three y/o	Treatment (Wk 1)	Treatment	Feed Day	No	20:30	20:00 - 20:59	Night	38,2	61	15,8	15,8	19,36	556,01	92,28
2019/05/16	CH2206	Male	Three y/o	Treatment (Wk 1)	Treatment	Feed Day	No	20:30	20:00 - 20:59	Night	37,9	49	17,6	17,6	19,05	537,80	95,95
2019/05/16	CH2205	Male	Three y/o	Treatment (Wk 1)	Treatment	Feed Day	No	20:35	20:00 - 20:59	Night	38,1	90	11,8	11,8	19,26	586,16	82,36
2019/05/16	CH2206	Male	Three y/o	Treatment (Wk 1)	Treatment	Feed Day	No	20:35	20:00 - 20:59	Night	37,9	96	17,8	17,8	19,05	590,91	96,34
2019/05/16	CH2205	Male	Three y/o	Treatment (Wk 1)	Treatment	Feed Day	No	20:40	20:00 - 20:59	Night	38,1	53	13,6	13,6	19,26	544,43	87,18
2019/05/16	CH2206	Male	Three y/o	Treatment (Wk 1)	Treatment	Feed Day	No	20:40	20:00 - 20:59	Night	38,0	84	14,2	14,2	19,15	581,01	88,65
2019/05/16	CH2205	Male	Three y/o	Treatment (Wk 1)	Treatment	Feed Day	No	20:45	20:00 - 20:59	Night	38,1	83	10,4	10,4	19,26	580,11	78,08
2019/05/16	CH2206	Male	Three y/o	Treatment (Wk 1)	Treatment	Feed Day	No	20:45	20:00 - 20:59	Night	37,9	65	13,2	13,2	19,05	561,12	86,17
2019/05/16	CH2205	Male	Three y/o	Treatment (Wk 1)	Treatment	Feed Day	No	20:50	20:00 - 20:59	Night	38,1	68	12,6	12,6	19,26	564,71	84,59
2019/05/16	CH2206	Male	Three y/o	Treatment (Wk 1)	Treatment	Feed Day	No	20:50	20:00 - 20:59	Night	37,9	45	27,0	27,0	19,05	530,47	110,58
2019/05/16	CH2205	Male	Three y/o	Treatment (Wk 1)	Treatment	Feed Day	No	20:55	20:00 - 20:59	Night	38,1	63	12,6	12,6	19,26	558,62	84,59
2019/05/16	CH2206	Male	Three y/o	Treatment (Wk 1)	Treatment	Feed Day	No	20:55	20:00 - 20:59	Night	37,9	65	29,8	29,8	19,05	561,12	113,97
2019/05/16	CH2205	Male	Three y/o	Treatment (Wk 1)	Treatment	Feed Day	No	21:00	21:00 - 21:59	Night	38,1	89	7,6	7,6	19,26	585,33	67,49
2019/05/16	CH2206	Male	Three y/o	Treatment (Wk 1)	Treatment	Feed Day	No	21:00	21:00 - 21:59	Night	38,0	64	23,8	23,8	19,15	559,88	106,26
2019/05/16	CH2205	Male	Three y/o	Treatment (Wk 1)	Treatment	Feed Day	No	21:05	21:00 - 21:59	Night	38,1	63	13,4	13,4	19,26	558,62	86,68
2019/05/16	CH2206	Male	Three y/o	Treatment (Wk 1)	Treatment	Feed Day	No	21:05	21:00 - 21:59	Night	38,0	54	17,8	17,8	19,15	545,99	96,34
2019/05/16	CH2205	Male	Three y/o	Treatment (Wk 1)	Treatment	Feed Day	No	21:10	21:00 - 21:59	Night	38,1	111	13,0	13,0	19,26	601,33	85,65
2019/05/16	CH2206	Male	Three y/o	Treatment (Wk 1)	Treatment	Feed Day	No	21:10	21:00 - 21:59	Night	38,0	108	55,6	55,6	19,15	599,39	135,46
2019/05/16	CH2205	Male	Three y/o	Treatment (Wk 1)	Treatment	Feed Day	No	21:15	21:00 - 21:59	Night	38,1	95	9,4	9,4	19,26	590,14	74,66
2019/05/16	CH2206	Male	Three y/o	Treatment (Wk 1)	Treatment	Feed Day	No	21:15	21:00 - 21:59	Night	37,9	98	60,8	60,8	19,05	592,41	138,55
2019/05/16	CH2205	Male	Three y/o	Treatment (Wk 1)	Treatment	Feed Day	No	21:20	21:00 - 21:59	Night	38,1	56	8,4	8,4	19,26	549,01	70,86
2019/05/16	CH2206	Male	Three y/o	Treatment (Wk 1)	Treatment	Feed Day	No	21:20	21:00 - 21:59	Night	37,8	83	61,2	61,2	18,95	580,11	138,78
2019/05/16	CH2205	Male	Three y/o	Treatment (Wk 1)	Treatment	Feed Day	No	21:25	21:00 - 21:59	Night	38,0	54	9,0	9,0	19,15	545,99	73,19
2019/05/16	CH2206	Male	Three y/o	Treatment (Wk 1)	Treatment	Feed Day	No	21:25	21:00 - 21:59	Night	37,8	85	56,6	56,6	18,95	581,90	136,07
2019/05/16	CH2205	Male	Three y/o	Treatment (Wk 1)	Treatment	Feed Day	No	21:30	21:00 - 21:59	Night	38,0	174	10,2	10,2	19,15	631,46	77,43
2019/05/16	CH2206	Male	Three y/o	Treatment (Wk 1)	Treatment	Feed Day	No	21:30	21:00 - 21:59	Night	37,7	83	47,2	47,2	18,85	580,11	129,80
2019/05/16	CH2205	Male	Three y/o	Treatment (Wk 1)	Treatment	Feed Day	No	21:35	21:00 - 21:59	Night	37,9		6,0	6,0	19,05		59,53
2019/05/16	CH2206	Male	Three y/o	Treatment (Wk 1)	Treatment	Feed Day	No	21:35	21:00 - 21:59	Night	37,8	54	47,6	47,6	18,95	545,99	130,09
2019/05/16	CH2205	Male	Three y/o	Treatment (Wk 1)	Treatment	Feed Day	No	21:40	21:00 - 21:59	Night	37,9	45	9,6	9,6	19,05	530,47	75,37
2019/05/16	CH2206	Male	Three y/o	Treatment (Wk 1)	Treatment	Feed Day	No	21:40	21:00 - 21:59	Night	37,8	83	44,2	44,2	18,95	580,11	127,53
2019/05/16	CH2205	Male	Three y/o	Treatment (Wk 1)	Treatment	Feed Day	No	21:45	21:00 - 21:59	Night	37,9	69	9,4	9,4	19,05	565,86	74,66
2019/05/16	CH2206	Male	Three y/o	Treatment (Wk 1)	Treatment	Feed Day	No	21:45	21:00 - 21:59	Night	37,7	50	10,4	10,4	18,85	539,52	78,08
2019/05/16	CH2205	Male	Three y/o	Treatment (Wk 1)	Treatment	Feed Day	No	21:50	21:00 - 21:59	Night	38,0	49	5,6	5,6	19,15	537,80	57,21
2019/05/16	CH2206	Male	Three y/o	Treatment (Wk 1)	Treatment	Feed Day	No	21:50	21:00 - 21:59	Night	37,5	90	6,6	6,6	18,65	586,16	62,73
2019/05/16	CH2205	Male	Three y/o	Treatment (Wk 1)	Treatment	Feed Day	No	21:55	21:00 - 21:59	Night	38,0	51	8,4	8,4	19,15	541,20	70,86
2019/05/16	CH2206	Male	Three y/o	Treatment (Wk 1)	Treatment	Feed Day	No	21:55	21:00 - 21:59	Night	37,5	61	6,8	6,8	18,65	556,01	63,74
2019/05/16	CH2205	Male	Three y/o	Treatment (Wk 1)	Treatment	Feed Day	No	22:00	22:00 - 22:59	Night	38,0	62	7,0	7,0	19,15	557,33	64,72
2019/05/16	CH2206	Male	Three y/o	Treatment (Wk 1)	Treatment	Feed Day	No	22:00	22:00 - 22:59	Night	37,5	83	9,0	9,0	18,65	580,11	73,19
2019/05/16	CH2205	Male	Three y/o	Treatment (Wk 1)	Treatment	Feed Day	No	22:05	22:00 - 22:59	Night	38,0	72	9,6	9,6	19,15	569,20	75,37
2019/05/16	CH2206	Male	Three y/o	Treatment (Wk 1)	Treatment	Feed Day	No	22:05	22:00 - 22:59	Night	37,5	79	10,8	10,8	18,65	576,36	79,36
2019/05/16	CH2205	Male	Three y/o	Treatment (Wk 1)	Treatment	Feed Day	No	22:10	22:00 - 22:59	Night	38,0	68	14,0	14,0	19,15	564,71	88,17
2019/05/16	CH2206	Male	Three y/o	Treatment (Wk 1)	Treatment	Feed Day	No	22:10	22:00 - 22:59	Night	37,7	89	13,8	13,8	18,85	585,33	87,68
2019/05/16	CH2205	Male	Three y/o	Treatment (Wk 1)	Treatment	Feed Day	No	22:15	22:00 - 22:59	Night	38,1	49	9,2	9,2	19,26	537,80	73,94
2019/05/16	CH2206	Male	Three y/o	Treatment (Wk 1)	Treatment	Feed Day	No	22:15	22:00 - 22:59	Night	37,7	84	10,4	10,4	18,85	581,01	78,08
2019/05/16	CH2205	Male	Three y/o	Treatment (Wk 1)	Treatment	Feed Day	No	22:20	22:00 - 22:59	Night	38,1	83	9,0	9,0	19,26	580,11	73,19
2019/05/16	CH2206	Male	Three y/o	Treatment (Wk 1)	Treatment	Feed Day	No	22:20	22:00 - 22:59	Night	37,7	47	21,6	21,6	18,85	534,23	102,94
2019/05/16	CH2205	Male	Three y/o	Treatment (Wk 1)	Treatment	Feed Day	No	22:25	22:00 - 22:59	Night	38,1	48	8,6	8,6	19,26	536,04	71,66
2019/05/16	CH2206	Male	Three y/o	Treatment (Wk 1)	Treatment	Feed Day	No	22:25	22:00 - 22:59	Night	37,8	80	18,0	18,0	18,95	577,31	96,72
2019/05/16	CH2205	Male	Three y/o	Treatment (Wk 1)	Treatment	Feed Day	No	22:30	22:00 - 22:59	Night	38,1	46	9,8	9,8	19,26	532,38	76,07

	2019/05/16	CH2206	Male	Three y/o	Treatment (Wk 1)	Treatment	Feed Day	No	22:30	22:00 - 22:59	Night	37,8	51	21,0	21,0	18,95	541,20	101,98
	2019/05/16	CH2205	Male	Three y/o	Treatment (Wk 1)	Treatment	Feed Day	No	22:35	22:00 - 22:59	Night	38,1	83	7,2	7,2	19,26	580,11	65,66
	2019/05/16	CH2206	Male	Three y/o	Treatment (Wk 1)	Treatment	Feed Day	No	22:35	22:00 - 22:59	Night	37,8	55	17,8	17,8	18,95	547,52	96,34
	2019/05/16	CH2205	Male	Three y/o	Treatment (Wk 1)	Treatment	Feed Day	No	22:40	22:00 - 22:59	Night	38,1	92	9,0	9,0	19,26	587,78	73,19
	2019/05/16	CH2206	Male	Three y/o	Treatment (Wk 1)	Treatment	Feed Day	No	22:40	22:00 - 22:59	Night	37,8	117	52,0	52,0	18,95	605,02	133,14
	2019/05/16	CH2205	Male	Three y/o	Treatment (Wk 1)	Treatment	Feed Day	No	22:45	22:00 - 22:59	Night	38,1	56	12,2	12,2	19,26	549,01	83,49
	2019/05/16	CH2206	Male	Three y/o	Treatment (Wk 1)	Treatment	Feed Day	No	22:45	22:00 - 22:59	Night	37,7	79	53,4	53,4	18,85	576,36	134,06
	2019/05/16	CH2205	Male	Three y/o	Treatment (Wk 1)	Treatment	Feed Day	No	22:50	22:00 - 22:59	Night	38,1	59	12,2	12,2	19,26	553,30	83,49
	2019/05/16	CH2206	Male	Three y/o	Treatment (Wk 1)	Treatment	Feed Day	No	22:50	22:00 - 22:59	Night	37,7	84	36,4	36,4	18,85	581,01	120,84
	2019/05/16	CH2205	Male	Three y/o	Treatment (Wk 1)	Treatment	Feed Day	No	22:55	22:00 - 22:59	Night	38,1	42	8,8	8,8	19,26	524,42	72,43
	2019/05/16	CH2206	Male	Three y/o	Treatment (Wk 1)	Treatment	Feed Day	No	22:55	22:00 - 22:59	Night	37,7	45	39,4	39,4	18,85	530,47	123,57
	2019/05/16	CH2205	Male	Three y/o	Treatment (Wk 1)	Treatment	Feed Day	No	23:00	23:00 - 23:59	Night	38,1	65	13,6	13,6	19,26	561,12	87,18
	2019/05/16	CH2206	Male	Three y/o	Treatment (Wk 1)	Treatment	Feed Day	No	23:00	23:00 - 23:59	Night	37,7	78	42,0	42,0	18,85	575,38	125,77
	2019/05/16	CH2205	Male	Three y/o	Treatment (Wk 1)	Treatment	Feed Day	No	23:05	23:00 - 23:59	Night	38,1	82	9,2	9,2	19,26	579,19	73,94
	2019/05/16	CH2206	Male	Three y/o	Treatment (Wk 1)	Treatment	Feed Day	No	23:05	23:00 - 23:59	Night	37,7	72	54,0	54,0	18,85	569,20	134,45
	2019/05/16	CH2205	Male	Three y/o	Treatment (Wk 1)	Treatment	Feed Day	No	23:10	23:00 - 23:59	Night	38,0	64	9,6	9,6	19,15	559,88	75,37
	2019/05/16	CH2206	Male	Three y/o	Treatment (Wk 1)	Treatment	Feed Day	No	23:10	23:00 - 23:59	Night	37,8	109	53,0	53,0	18,95	600,04	133,80
	2019/05/16	CH2205	Male	Three y/o	Treatment (Wk 1)	Treatment	Feed Day	No	23:15	23:00 - 23:59	Night	38,0	71	9,4	9,4	19,15	568,10	74,66
	2019/05/16	CH2206	Male	Three y/o	Treatment (Wk 1)	Treatment	Feed Day	No	23:15	23:00 - 23:59	Night	37,7	84	52,4	52,4	18,85	581,01	133,41
	2019/05/16	CH2205	Male	Three y/o	Treatment (Wk 1)	Treatment	Feed Day	No	23:20	23:00 - 23:59	Night	38,0	46	8,6	8,6	19,15	532,38	71,66
	2019/05/16	CH2206	Male	Three y/o	Treatment (Wk 1)	Treatment	Feed Day	No	23:20	23:00 - 23:59	Night	37,7	89	47,4	47,4	18,85	585,33	129,94
	2019/05/16	CH2205	Male	Three y/o	Treatment (Wk 1)	Treatment	Feed Day	No	23:25	23:00 - 23:59	Night	38,0	66	10,0	10,0	19,15	562,34	76,76
	2019/05/16	CH2206	Male	Three y/o	Treatment (Wk 1)	Treatment	Feed Day	No	23:25	23:00 - 23:59	Night	37,7	60	48,0	48,0	18,85	554,67	130,38
	2019/05/16	CH2205	Male	Three y/o	Treatment (Wk 1)	Treatment	Feed Day	No	23:30	23:00 - 23:59	Night	38,0	94	11,4	11,4	19,15	589,37	81,19
	2019/05/16	CH2206	Male	Three y/o	Treatment (Wk 1)	Treatment	Feed Day	No	23:30	23:00 - 23:59	Night	37,7	68	39,6	39,6	18,85	564,71	123,74
	2019/05/16	CH2205	Male	Three y/o	Treatment (Wk 1)	Treatment	Feed Day	No	23:35	23:00 - 23:59	Night	38,0	79	10,6	10,6	19,15	576,36	78,73
	2019/05/16	CH2206	Male	Three y/o	Treatment (Wk 1)	Treatment	Feed Day	No	23:35	23:00 - 23:59	Night	37,7	94	35,6	35,6	18,85	589,37	120,08
	2019/05/16	CH2205	Male	Three y/o	Treatment (Wk 1)	Treatment	Feed Day	No	23:40	23:00 - 23:59	Night	38,0	55	9,8	9,8	19,15	547,52	76,07
	2019/05/16	CH2206	Male	Three y/o	Treatment (Wk 1)	Treatment	Feed Day	No	23:40	23:00 - 23:59	Night	37,5	81	47,2	47,2	18,65	578,26	129,80
	2019/05/16	CH2205	Male	Three y/o	Treatment (Wk 1)	Treatment	Feed Day	No	23:45	23:00 - 23:59	Night	38,0	51	14,6	14,6	19,15	541,20	89,59
	2019/05/16	CH2206	Male	Three y/o	Treatment (Wk 1)	Treatment	Feed Day	No	23:45	23:00 - 23:59	Night	37,5	47	47,0	47,0	18,65	534,23	129,65
	2019/05/16	CH2205	Male	Three y/o	Treatment (Wk 1)	Treatment	Feed Day	No	23:50	23:00 - 23:59	Night	38,0	52	16,6	16,6	19,15	542,83	93,96
	2019/05/16	CH2206	Male	Three y/o	Treatment (Wk 1)	Treatment	Feed Day	No	23:50	23:00 - 23:59	Night	37,5	88	48,8	48,8	18,65	584,49	130,95
	2019/05/16	CH2205	Male	Three y/o	Treatment (Wk 1)	Treatment	Feed Day	No	23:55	23:00 - 23:59	Night	38,0	46	14,8	14,8	19,15	532,38	90,05
	2019/05/16	CH2206	Male	Three y/o	Treatment (Wk 1)	Treatment	Feed Day	No	23:55	23:00 - 23:59	Night	37,5	93	52,0	52,0	18,65	588,58	133,14
3	2019/05/17	CH2205	Male	Three y/o	Treatment (Wk 1)	Treatment	Feed Day	No	00:00	00:00 - 00:59	Early Morning	38,0	44	12,0	12,0	19,15	528,51	82,93
	2019/05/17	CH2206	Male	Three y/o	Treatment (Wk 1)	Treatment	Feed Day	No	00:00	00:00 - 00:59	Early Morning	37,5	88	53,6	53,6	18,65	584,49	134,19
	2019/05/17	CH2205	Male	Three y/o	Treatment (Wk 1)	Treatment	Feed Day	No	00:05	00:00 - 00:59	Early Morning	38,0	49	305,2	305,2	19,15	537,80	195,03
	2019/05/17	CH2206	Male	Three y/o	Treatment (Wk 1)	Treatment	Feed Day	No	00:05	00:00 - 00:59	Early Morning	37,5	96	31,8	31,8	18,65	590,91	116,20
	2019/05/17	CH2205	Male	Three y/o	Treatment (Wk 1)	Treatment	Feed Day	No	00:10	00:00 - 00:59	Early Morning	38,0		23,0	23,0	19,15		105,09
	2019/05/17	CH2206	Male	Three y/o	Treatment (Wk 1)	Treatment	Feed Day	No	00:10	00:00 - 00:59	Early Morning	37,7	51	10,8	10,8	18,85	541,20	79,36
	2019/05/17	CH2205	Male	Three y/o	Treatment (Wk 1)	Treatment	Feed Day	No	00:15	00:00 - 00:59	Early Morning	38,0	194	26,4	26,4	19,15	638,30	109,81
	2019/05/17	CH2206	Male	Three y/o	Treatment (Wk 1)	Treatment	Feed Day	No	00:15	00:00 - 00:59	Early Morning	37,7	97	10,0	10,0	18,85	591,66	76,76
	2019/05/17	CH2205	Male	Three y/o	Treatment (Wk 1)	Treatment	Feed Day	No	00:20	00:00 - 00:59	Early Morning	37,9	73	4,4	4,4	19,05	570,27	49,12
	2019/05/17	CH2206	Male	Three y/o	Treatment (Wk 1)	Treatment	Feed Day	No	00:20	00:00 - 00:59	Early Morning	37,7	56	12,4	12,4	18,85	549,01	84,04
	2019/05/17	CH2205	Male	Three y/o	Treatment (Wk 1)	Treatment	Feed Day	No	00:25	00:00 - 00:59	Early Morning	37,9	98	7,0	7,0	19,05	592,41	64,72
	2019/05/17	CH2206	Male	Three y/o	Treatment (Wk 1)	Treatment	Feed Day	No	00:25	00:00 - 00:59	Early Morning	37,7	76	7,0	7,0	18,85	573,39	64,72
	2019/05/17	CH2205	Male	Three y/o	Treatment (Wk 1)	Treatment	Feed Day	No	00:30	00:00 - 00:59	Early Morning	37,7	62	3,4	3,4	18,85	557,33	40,51
	2019/05/17	CH2206	Male	Three y/o	Treatment (Wk 1)	Treatment	Feed Day	No	00:30	00:00 - 00:59	Early Morning	37,7	78	13,8	13,8	18,85	575,38	87,68
	2019/05/17	CH2205	Male	Three y/o	Treatment (Wk 1)	Treatment	Feed Day	No	00:35	00:00 - 00:59	Early Morning	37,7	88	8,6	8,6	18,85	584,49	71,66
	2019/05/17	CH2206	Male	Three y/o	Treatment (Wk 1)	Treatment	Feed Day	No	00:35	00:00 - 00:59	Early Morning	37,7	88	19,6	19,6	18,85	584,49	99,63
	2019/05/17	CH2205	Male	Three y/o	Treatment (Wk 1)	Treatment	Feed Day	No	00:40	00:00 - 00:59	Early Morning	37,7	91	6,8	6,8	18,85	586,98	63,74
	2019/05/17	CH2206	Male	Three y/o	Treatment (Wk 1)	Treatment	Feed Day	No	00:40	00:00 - 00:59	Early Morning	37,7	80	20,6	20,6	18,85	577,31	101,32
	2019/05/17	CH2205	Male	Three y/o	Treatment (Wk 1)	Treatment	Feed Day	No	00:45	00:00 - 00:59	Early Morning	37,7	42	7,2	7,2	18,85	524,42	65,66
	2019/05/17	CH2206	Male	Three y/o	Treatment (Wk 1)	Treatment	Feed Day	No	00:45	00:00 - 00:59	Early Morning	37,7	47	19,2	19,2	18,85	534,23	98,92
	2019/05/17	CH2205	Male	Three y/o	Treatment (Wk 1)	Treatment	Feed Day	No	00:50	00:00 - 00:59	Early Morning	37,7	79	5,2	5,2	18,85	576,36	54,72
	2019/05/17	CH2206	Male	Three y/o	Treatment (Wk 1)	Treatment	Feed Day	No	00:50	00:00 - 00:59	Early Morning	37,7	60	12,8	12,8	18,85	554,67	85,12
	2019/05/17	CH2205	Male	Three y/o	Treatment (Wk 1)	Treatment	Feed Day	No	00:55	00:00 - 00:59	Early Morning	37,7	57	6,6	6,6	18,85	550,47	62,73
	2019/05/17	CH2206	Male	Three y/o	Treatment (Wk 1)	Treatment	Feed Day	No	00:55	00:00 - 00:59	Early Morning	37,8	53	21,0	21,0	18,95	544,43	101,98
	2019/05/17	CH2205	Male	Three y/o	Treatment (Wk 1)	Treatment	Feed Day	No	01:00	01:00 - 01:59	Early Morning	37,7	55	9,2	9,2	18,85	547,52	73,94
	2019/05/17	CH2206	Male	Three y/o	Treatment (Wk 1)	Treatment	Feed Day	No	01:00	01:00 - 01:59	Early Morning	37,8	45	26,4	26,4	18,95	530,47	109,81
	2019/05/17	CH2205	Male	Three y/o	Treatment (Wk 1)	Treatment	Feed Day	No	01:05	01:00 - 01:59	Early Morning	37,7	98	7,4	7,4	18,85	592,41	66,59
	2019/05/17	CH2206	Male	Three y/o	Treatment (Wk 1)	Treatment	Feed Day	No	01:05	01:00 - 01:59	Early Morning	37,7	83	31,8	31,8	18,85	580,11	116,20
	2019/05/17	CH2205	Male	Three y/o	Treatment (Wk 1)	Treatment	Feed Day	No	01:10	01:00 - 01:59	Early Morning	37,7	88	6,0	6,0	18,85	584,49	59,53
	2019/05/17	CH2206	Male	Three y/o	Treatment (Wk 1)	Treatment	Feed Day	No	01:10	01:00 - 01:59	Early Morning	37,5	56	29,8	29,8	18,65	549,01	113,97
	2019/05/17	CH2205	Male	Three y/o	Treatment (Wk 1)	Treatment	Feed Day	No	01:15	01:00 - 01:59	Early Morning	37,7	49	6,8	6,8	18,85	537,80	63,74
	2019/05/17	CH2206	Male	Three y/o	Treatment (Wk 1)	Treatment	Feed Day	No	01:15	01:00 - 01:59	Early Morning	37,3	46	17,6	17,6	18,46	532,38	95,95
	2019/05/17	CH2205	Male	Three y/o	Treatment (Wk 1)	Treatment	Feed Day	No	01:20	01:00 - 01:59	Early Morning	37,7	62	4,8	4,8	18,85	557,33	52,04
	2019/05/17	CH2206	Male	Three y/o	Treatment (Wk 1)	Treatment	Feed Day	No	01:20	01:00 - 01:59	Early Morning	37,3	80	14,6	14,6	18,46	577,31	89,59
	2019/05/17	CH2205	Male	Three y/o	Treatment (Wk 1)	Treatment	Feed Day	No	01:25	01:00 - 01:59	Early Morning	37,7	80	10,0	10,0	18,85	577,31	76,76

2019/05/17	CH2206	Male	Three y/o	Treatment (Wk 1)	Treatment	Feed Day	No	01:25	01:00 - 01:59	Early Morning	37,3	45	20,6	20,6	18,46	530,47	101,32
2019/05/17	CH2205	Male	Three y/o	Treatment (Wk 1)	Treatment	Feed Day	No	01:30	01:00 - 01:59	Early Morning	37,9	57	11,4	11,4	19,05	550,47	81,19
2019/05/17	CH2206	Male	Three y/o	Treatment (Wk 1)	Treatment	Feed Day	No	01:30	01:00 - 01:59	Early Morning	37,4	62	19,2	19,2	18,55	557,33	98,92
2019/05/17	CH2205	Male	Three y/o	Treatment (Wk 1)	Treatment	Feed Day	No	01:35	01:00 - 01:59	Early Morning	37,9	45	15,8	15,8	19,05	530,47	92,28
2019/05/17	CH2206	Male	Three y/o	Treatment (Wk 1)	Treatment	Feed Day	No	01:35	01:00 - 01:59	Early Morning	37,5	55	15,4	15,4	18,65	547,52	91,41
2019/05/17	CH2205	Male	Three y/o	Treatment (Wk 1)	Treatment	Feed Day	No	01:40	01:00 - 01:59	Early Morning	37,8	49	15,4	15,4	18,95	537,80	91,41
2019/05/17	CH2206	Male	Three y/o	Treatment (Wk 1)	Treatment	Feed Day	No	01:40	01:00 - 01:59	Early Morning	37,5	77	17,8	17,8	18,65	574,39	96,34
2019/05/17	CH2205	Male	Three y/o	Treatment (Wk 1)	Treatment	Feed Day	No	01:45	01:00 - 01:59	Early Morning	37,7	54	20,8	20,8	18,85	545,99	101,65
2019/05/17	CH2206	Male	Three y/o	Treatment (Wk 1)	Treatment	Feed Day	No	01:45	01:00 - 01:59	Early Morning	37,5	94	36,2	36,2	18,65	589,37	120,65
2019/05/17	CH2205	Male	Three y/o	Treatment (Wk 1)	Treatment	Feed Day	No	01:50	01:00 - 01:59	Early Morning	37,9	128	65,8	65,8	19,05	611,22	141,29
2019/05/17	CH2206	Male	Three y/o	Treatment (Wk 1)	Treatment	Feed Day	No	01:50	01:00 - 01:59	Early Morning	37,3	96	37,6	37,6	18,46	590,91	121,96
2019/05/17	CH2205	Male	Three y/o	Treatment (Wk 1)	Treatment	Feed Day	No	01:55	01:00 - 01:59	Early Morning	37,9	131	95,4	95,4	19,05	612,80	154,20
2019/05/17	CH2206	Male	Three y/o	Treatment (Wk 1)	Treatment	Feed Day	No	01:55	01:00 - 01:59	Early Morning	37,5	117	198,8	198,8	18,65	605,02	179,91
2019/05/17	CH2205	Male	Three y/o	Treatment (Wk 1)	Treatment	Feed Day	No	02:00	02:00 - 02:59	Early Morning	37,7	120	67,0	67,0	18,85	606,78	141,92
2019/05/17	CH2206	Male	Three y/o	Treatment (Wk 1)	Treatment	Feed Day	No	02:00	02:00 - 02:59	Early Morning	37,5	108	48,2	48,2	18,65	599,39	130,52
2019/05/17	CH2205	Male	Three y/o	Treatment (Wk 1)	Treatment	Feed Day	No	02:05	02:00 - 02:59	Early Morning	37,6	85	183,6	183,6	18,75	581,90	177,11
2019/05/17	CH2206	Male	Three y/o	Treatment (Wk 1)	Treatment	Feed Day	No	02:05	02:00 - 02:59	Early Morning	37,4	110	84,2	84,2	18,55	600,69	149,85
2019/05/17	CH2205	Male	Three y/o	Treatment (Wk 1)	Treatment	Feed Day	No	02:10	02:00 - 02:59	Early Morning	37,7	114	28,0	28,0	18,85	603,20	111,83
2019/05/17	CH2206	Male	Three y/o	Treatment (Wk 1)	Treatment	Feed Day	No	02:10	02:00 - 02:59	Early Morning	37,5	119	68,2	68,2	18,65	606,20	142,53
2019/05/17	CH2205	Male	Three y/o	Treatment (Wk 1)	Treatment	Feed Day	No	02:15	02:00 - 02:59	Early Morning	37,6	118	93,0	93,0	18,75	605,61	153,31
2019/05/17	CH2206	Male	Three y/o	Treatment (Wk 1)	Treatment	Feed Day	No	02:15	02:00 - 02:59	Early Morning	37,5	94	186,8	186,8	18,65	589,37	177,72
2019/05/17	CH2205	Male	Three y/o	Treatment (Wk 1)	Treatment	Feed Day	No	02:20	02:00 - 02:59	Early Morning	37,7	107	59,6	59,6	18,85	598,73	137,86
2019/05/17	CH2206	Male	Three y/o	Treatment (Wk 1)	Treatment	Feed Day	No	02:20	02:00 - 02:59	Early Morning	37,4	111	7,2	7,2	18,55	601,33	65,66
2019/05/17	CH2205	Male	Three y/o	Treatment (Wk 1)	Treatment	Feed Day	No	02:25	02:00 - 02:59	Early Morning	37,6		26,4	26,4	18,75		109,81
2019/05/17	CH2206	Male	Three y/o	Treatment (Wk 1)	Treatment	Feed Day	No	02:25	02:00 - 02:59	Early Morning	37,1	48	5,4	5,4	18,26	536,04	55,99
2019/05/17	CH2205	Male	Three y/o	Treatment (Wk 1)	Treatment	Feed Day	No	02:30	02:00 - 02:59	Early Morning	37,3		21,0	21,0	18,46		101,98
2019/05/17	CH2206	Male	Three y/o	Treatment (Wk 1)	Treatment	Feed Day	No	02:30	02:00 - 02:59	Early Morning	36,8	169	3,0	3,0	17,96	629,60	36,34
2019/05/17	CH2205	Male	Three y/o	Treatment (Wk 1)	Treatment	Feed Day	No	02:35	02:00 - 02:59	Early Morning	37,2	109	50,0	50,0	18,36	600,04	131,79
2019/05/17	CH2206	Male	Three y/o	Treatment (Wk 1)	Treatment	Feed Day	No	02:35	02:00 - 02:59	Early Morning	36,6	182	12,0	12,0	17,77	634,31	82,93
2019/05/17	CH2205	Male	Three y/o	Treatment (Wk 1)	Treatment	Feed Day	No	02:40	02:00 - 02:59	Early Morning	37,3	109	85,8	85,8	18,46	600,04	150,51
2019/05/17	CH2206	Male	Three y/o	Treatment (Wk 1)	Treatment	Feed Day	No	02:40	02:00 - 02:59	Early Morning	36,8	165	229,2	229,2	17,96	628,06	184,92
2019/05/17	CH2205	Male	Three y/o	Treatment (Wk 1)	Treatment	Feed Day	No	02:45	02:00 - 02:59	Early Morning	37,5	112	33,0	33,0	18,65	601,96	117,47
2019/05/17	CH2206	Male	Three y/o	Treatment (Wk 1)	Treatment	Feed Day	No	02:45	02:00 - 02:59	Early Morning	37,1	126	155,4	155,4	18,26	610,14	171,26
2019/05/17	CH2205	Male	Three y/o	Treatment (Wk 1)	Treatment	Feed Day	No	02:50	02:00 - 02:59	Early Morning	37,6		96,2	96,2	18,75		154,49
2019/05/17	CH2206	Male	Three y/o	Treatment (Wk 1)	Treatment	Feed Day	No	02:50	02:00 - 02:59	Early Morning	37,2	109	101,0	101,0	18,36	600,04	156,19
2019/05/17	CH2205	Male	Three y/o	Treatment (Wk 1)	Treatment	Feed Day	No	02:55	02:00 - 02:59	Early Morning	37,6	52	59,6	59,6	18,75	542,83	137,86
2019/05/17	CH2206	Male	Three y/o	Treatment (Wk 1)	Treatment	Feed Day	No	02:55	02:00 - 02:59	Early Morning	37,2	113	34,4	34,4	18,36	602,58	118,90
2019/05/17	CH2205	Male	Three y/o	Treatment (Wk 1)	Treatment	Feed Day	No	03:00	03:00 - 03:59	Early Morning	37,5	73	3,4	3,4	18,65	570,27	40,51
2019/05/17	CH2206	Male	Three y/o	Treatment (Wk 1)	Treatment	Feed Day	No	03:00	03:00 - 03:59	Early Morning	37,1	78	57,8	57,8	18,26	575,38	136,80
2019/05/17	CH2205	Male	Three y/o	Treatment (Wk 1)	Treatment	Feed Day	No	03:05	03:00 - 03:59	Early Morning	37,3	51	4,0	4,0	18,46	541,20	45,94
2019/05/17	CH2206	Male	Three y/o	Treatment (Wk 1)	Treatment	Feed Day	No	03:05	03:00 - 03:59	Early Morning	36,8	47	33,8	33,8	17,96	534,23	118,29
2019/05/17	CH2205	Male	Three y/o	Treatment (Wk 1)	Treatment	Feed Day	No	03:10	03:00 - 03:59	Early Morning	37,2	53	14,2	14,2	18,36	544,43	88,65
2019/05/17	CH2206	Male	Three y/o	Treatment (Wk 1)	Treatment	Feed Day	No	03:10	03:00 - 03:59	Early Morning	36,5	48	30,6	30,6	17,67	536,04	114,87
2019/05/17	CH2205	Male	Three y/o	Treatment (Wk 1)	Treatment	Feed Day	No	03:15	03:00 - 03:59	Early Morning	37,2	72	14,4	14,4	18,36	569,20	89,12
2019/05/17	CH2206	Male	Three y/o	Treatment (Wk 1)	Treatment	Feed Day	No	03:15	03:00 - 03:59	Early Morning	36,5	47	21,4	21,4	17,67	534,23	102,63
2019/05/17	CH2205	Male	Three y/o	Treatment (Wk 1)	Treatment	Feed Day	No	03:20	03:00 - 03:59	Early Morning	37,3	87	18,8	18,8	18,46	583,64	98,20
2019/05/17	CH2206	Male	Three y/o	Treatment (Wk 1)	Treatment	Feed Day	No	03:20	03:00 - 03:59	Early Morning	37,3	55	13,6	13,6	18,46	547,52	87,18
2019/05/17	CH2205	Male	Three y/o	Treatment (Wk 1)	Treatment	Feed Day	No	03:25	03:00 - 03:59	Early Morning	37,4	84	19,8	19,8	18,55	581,01	99,97
2019/05/17	CH2206	Male	Three y/o	Treatment (Wk 1)	Treatment	Feed Day	No	03:25	03:00 - 03:59	Early Morning	37,4	60	17,4	17,4	18,55	554,67	95,56
2019/05/17	CH2205	Male	Three y/o	Treatment (Wk 1)	Treatment	Feed Day	No	03:30	03:00 - 03:59	Early Morning	37,4	50	21,4	21,4	18,55	539,52	102,63
2019/05/17	CH2206	Male	Three y/o	Treatment (Wk 1)	Treatment	Feed Day	No	03:30	03:00 - 03:59	Early Morning	37,4	180	57,2	57,2	18,55	633,61	136,44
2019/05/17	CH2205	Male	Three y/o	Treatment (Wk 1)	Treatment	Feed Day	No	03:35	03:00 - 03:59	Early Morning	37,5	58	13,6	13,6	18,65	551,90	87,18
2019/05/17	CH2206	Male	Three y/o	Treatment (Wk 1)	Treatment	Feed Day	No	03:35	03:00 - 03:59	Early Morning	37,3	79	58,2	58,2	18,46	576,36	137,04
2019/05/17	CH2205	Male	Three y/o	Treatment (Wk 1)	Treatment	Feed Day	No	03:40	03:00 - 03:59	Early Morning	37,5	79	18,6	18,6	18,65	576,36	97,84
2019/05/17	CH2206	Male	Three y/o	Treatment (Wk 1)	Treatment	Feed Day	No	03:40	03:00 - 03:59	Early Morning	37,3	86	47,4	47,4	18,46	582,77	129,94
2019/05/17	CH2205	Male	Three y/o	Treatment (Wk 1)	Treatment	Feed Day	No	03:45	03:00 - 03:59	Early Morning	37,4	93	4,4	4,4	18,55	588,58	49,12
2019/05/17	CH2206	Male	Three y/o	Treatment (Wk 1)	Treatment	Feed Day	No	03:45	03:00 - 03:59	Early Morning	37,3	83	65,0	65,0	18,46	580,11	140,87
2019/05/17	CH2205	Male	Three y/o	Treatment (Wk 1)	Treatment	Feed Day	No	03:50	03:00 - 03:59	Early Morning	37,6	61	5,6	5,6	18,75	556,01	57,21
2019/05/17	CH2206	Male	Three y/o	Treatment (Wk 1)	Treatment	Feed Day	No	03:50	03:00 - 03:59	Early Morning	37,2	57	63,6	63,6	18,36	550,47	140,11
2019/05/17	CH2205	Male	Three y/o	Treatment (Wk 1)	Treatment	Feed Day	No	03:55	03:00 - 03:59	Early Morning	37,6	68	6,6	6,6	18,75	564,71	62,73
2019/05/17	CH2206	Male	Three y/o	Treatment (Wk 1)	Treatment	Feed Day	No	03:55	03:00 - 03:59	Early Morning	37,2	59	60,8	60,8	18,36	553,30	138,55
2019/05/17	CH2205	Male	Three y/o	Treatment (Wk 1)	Treatment	Feed Day	No	04:00	04:00 - 04:59	Morning	37,6	80	5,4	5,4	18,75	577,31	55,99
2019/05/17	CH2206	Male	Three y/o	Treatment (Wk 1)	Treatment	Feed Day	No	04:00	04:00 - 04:59	Morning	37,2	80	54,6	54,6	18,36	577,31	134,83
2019/05/17	CH2205	Male	Three y/o	Treatment (Wk 1)	Treatment	Feed Day	No	04:05	04:00 - 04:59	Morning	37,6	55	3,4	3,4	18,75	547,52	40,51
2019/05/17	CH2206	Male	Three y/o	Treatment (Wk 1)	Treatment	Feed Day	No	04:05	04:00 - 04:59	Morning	37,3	85	46,4	46,4	18,46	581,90	129,21
2019/05/17	CH2205	Male	Three y/o	Treatment (Wk 1)	Treatment	Feed Day	No	04:10	04:00 - 04:59	Morning	37,6	154	12,8	12,8	18,75	623,58	85,12
2019/05/17	CH2206	Male	Three y/o	Treatment (Wk 1)	Treatment	Feed Day	No	04:10	04:00 - 04:59	Morning	37,3	106	60,2	60,2	18,46	598,06	138,21
2019/05/17	CH2205	Male	Three y/o	Treatment (Wk 1)	Treatment	Feed Day	No	04:15	04:00 - 04:59	Morning	37,5	92	4,2	4,2	18,65	587,78	47,57
2019/05/17	CH2206	Male	Three y/o	Treatment (Wk 1)	Treatment	Feed Day	No	04:15	04:00 - 04:59	Morning	37,3	73	62,0	62,0	18,46	570,27	139,23
2019/05/17	CH2205	Male	Three y/o	Treatment (Wk 1)	Treatment	Feed Day	No	04:20	04:00 - 04:59	Morning	37,4	57	3,2	3,2	18,55	550,47	38,49

2019/05/17	CH2206	Male	Three y/o	Treatment (Wk 1)	Treatment	Feed Day	No	04:20	04:00 - 04:59	Morning	37,4	83	45,2	45,2	18,55	580,11	128,30
2019/05/17	CH2205	Male	Three y/o	Treatment (Wk 1)	Treatment	Feed Day	No	04:25	04:00 - 04:59	Morning	37,4	64	5,4	5,4	18,55	559,88	55,99
2019/05/17	CH2206	Male	Three y/o	Treatment (Wk 1)	Treatment	Feed Day	No	04:25	04:00 - 04:59	Morning	37,4	57	38,4	38,4	18,55	550,47	122,68
2019/05/17	CH2205	Male	Three y/o	Treatment (Wk 1)	Treatment	Feed Day	No	04:30	04:00 - 04:59	Morning	37,5	88	4,4	4,4	18,65	584,49	49,12
2019/05/17	CH2206	Male	Three y/o	Treatment (Wk 1)	Treatment	Feed Day	No	04:30	04:00 - 04:59	Morning	37,4	39	56,4	56,4	18,55	517,82	135,95
2019/05/17	CH2205	Male	Three y/o	Treatment (Wk 1)	Treatment	Feed Day	No	04:35	04:00 - 04:59	Morning	37,5	55	6,2	6,2	18,65	547,52	60,63
2019/05/17	CH2206	Male	Three y/o	Treatment (Wk 1)	Treatment	Feed Day	No	04:35	04:00 - 04:59	Morning	37,4	45	56,2	56,2	18,55	530,47	135,83
2019/05/17	CH2205	Male	Three y/o	Treatment (Wk 1)	Treatment	Feed Day	No	04:40	04:00 - 04:59	Morning	37,6	86	4,2	4,2	18,75	582,77	47,57
2019/05/17	CH2206	Male	Three y/o	Treatment (Wk 1)	Treatment	Feed Day	No	04:40	04:00 - 04:59	Morning	37,4	73	57,6	57,6	18,55	570,27	136,68
2019/05/17	CH2205	Male	Three y/o	Treatment (Wk 1)	Treatment	Feed Day	No	04:45	04:00 - 04:59	Morning	37,6	48	20,4	20,4	18,75	536,04	100,99
2019/05/17	CH2206	Male	Three y/o	Treatment (Wk 1)	Treatment	Feed Day	No	04:45	04:00 - 04:59	Morning	37,3	58	59,0	59,0	18,46	551,90	137,51
2019/05/17	CH2205	Male	Three y/o	Treatment (Wk 1)	Treatment	Feed Day	No	04:50	04:00 - 04:59	Morning	37,6	86	5,2	5,2	18,75	582,77	54,72
2019/05/17	CH2206	Male	Three y/o	Treatment (Wk 1)	Treatment	Feed Day	No	04:50	04:00 - 04:59	Morning	37,3	50	67,2	67,2	18,46	539,52	142,02
2019/05/17	CH2205	Male	Three y/o	Treatment (Wk 1)	Treatment	Feed Day	No	04:55	04:00 - 04:59	Morning	37,6	62	6,6	6,6	18,75	557,33	62,73
2019/05/17	CH2206	Male	Three y/o	Treatment (Wk 1)	Treatment	Feed Day	No	04:55	04:00 - 04:59	Morning	37,3	53	60,0	60,0	18,46	544,43	138,09
2019/05/17	CH2205	Male	Three y/o	Treatment (Wk 1)	Treatment	Feed Day	No	05:00	05:00 - 05:59	Morning	37,6	46	5,0	5,0	18,75	532,38	53,41
2019/05/17	CH2206	Male	Three y/o	Treatment (Wk 1)	Treatment	Feed Day	No	05:00	05:00 - 05:59	Morning	37,2	89	62,0	62,0	18,36	585,33	139,23
2019/05/17	CH2205	Male	Three y/o	Treatment (Wk 1)	Treatment	Feed Day	No	05:05	05:00 - 05:59	Morning	37,6	90	3,8	3,8	18,75	586,16	44,22
2019/05/17	CH2206	Male	Three y/o	Treatment (Wk 1)	Treatment	Feed Day	No	05:05	05:00 - 05:59	Morning	37,2	43	8,6	8,6	18,36	526,50	71,66
2019/05/17	CH2205	Male	Three y/o	Treatment (Wk 1)	Treatment	Feed Day	No	05:10	05:00 - 05:59	Morning	37,7	53	3,6	3,6	18,85	544,43	42,42
2019/05/17	CH2206	Male	Three y/o	Treatment (Wk 1)	Treatment	Feed Day	No	05:10	05:00 - 05:59	Morning	37,0	52	20,0	20,0	18,16	542,83	100,31
2019/05/17	CH2205	Male	Three y/o	Treatment (Wk 1)	Treatment	Feed Day	No	05:15	05:00 - 05:59	Morning	37,7	61	12,2	12,2	18,85	556,01	83,49
2019/05/17	CH2206	Male	Three y/o	Treatment (Wk 1)	Treatment	Feed Day	No	05:15	05:00 - 05:59	Morning	37,1	99	62,0	62,0	18,26	593,14	139,23
2019/05/17	CH2205	Male	Three y/o	Treatment (Wk 1)	Treatment	Feed Day	No	05:20	05:00 - 05:59	Morning	37,6	43	7,2	7,2	18,75	526,50	65,66
2019/05/17	CH2206	Male	Three y/o	Treatment (Wk 1)	Treatment	Feed Day	No	05:20	05:00 - 05:59	Morning	37,2	83	60,0	60,0	18,36	580,11	138,09
2019/05/17	CH2205	Male	Three y/o	Treatment (Wk 1)	Treatment	Feed Day	No	05:25	05:00 - 05:59	Morning	37,6	78	17,4	17,4	18,75	575,38	95,56
2019/05/17	CH2206	Male	Three y/o	Treatment (Wk 1)	Treatment	Feed Day	No	05:25	05:00 - 05:59	Morning	37,2	53	55,8	55,8	18,36	544,43	135,58
2019/05/17	CH2205	Male	Three y/o	Treatment (Wk 1)	Treatment	Feed Day	No	05:30	05:00 - 05:59	Morning	37,5	54	8,6	8,6	18,65	545,99	71,66
2019/05/17	CH2206	Male	Three y/o	Treatment (Wk 1)	Treatment	Feed Day	No	05:30	05:00 - 05:59	Morning	37,1	66	52,8	52,8	18,26	562,34	133,67
2019/05/17	CH2205	Male	Three y/o	Treatment (Wk 1)	Treatment	Feed Day	No	05:35	05:00 - 05:59	Morning	37,6	60	9,4	9,4	18,75	554,67	74,66
2019/05/17	CH2206	Male	Three y/o	Treatment (Wk 1)	Treatment	Feed Day	No	05:35	05:00 - 05:59	Morning	37,1	49	73,2	73,2	18,26	537,80	144,99
2019/05/17	CH2205	Male	Three y/o	Treatment (Wk 1)	Treatment	Feed Day	No	05:40	05:00 - 05:59	Morning	37,6	61	21,0	21,0	18,75	556,01	101,98
2019/05/17	CH2206	Male	Three y/o	Treatment (Wk 1)	Treatment	Feed Day	No	05:40	05:00 - 05:59	Morning	37,2	111	96,0	96,0	18,36	601,33	154,42
2019/05/17	CH2205	Male	Three y/o	Treatment (Wk 1)	Treatment	Feed Day	No	05:45	05:00 - 05:59	Morning	37,6	112	12,4	12,4	18,75	601,96	84,04
2019/05/17	CH2206	Male	Three y/o	Treatment (Wk 1)	Treatment	Feed Day	No	05:45	05:00 - 05:59	Morning	37,2	138	104,8	104,8	18,36	616,31	157,48
2019/05/17	CH2205	Male	Three y/o	Treatment (Wk 1)	Treatment	Feed Day	No	05:50	05:00 - 05:59	Morning	37,5		8,2	8,2	18,65		70,05
2019/05/17	CH2206	Male	Three y/o	Treatment (Wk 1)	Treatment	Feed Day	No	05:50	05:00 - 05:59	Morning	37,3	128	101,4	101,4	18,46	611,22	156,33
2019/05/17	CH2205	Male	Three y/o	Treatment (Wk 1)	Treatment	Feed Day	No	05:55	05:00 - 05:59	Morning	37,3		15,4	15,4	18,46		91,41
2019/05/17	CH2206	Male	Three y/o	Treatment (Wk 1)	Treatment	Feed Day	No	05:55	05:00 - 05:59	Morning	37,0	84	59,2	59,2	18,16	581,01	137,63
2019/05/17	CH2205	Male	Three y/o	Treatment (Wk 1)	Treatment	Feed Day	No	06:00	06:00 - 06:59	Morning	37,3	79	13,8	13,8	18,46	576,36	87,68
2019/05/17	CH2206	Male	Three y/o	Treatment (Wk 1)	Treatment	Feed Day	No	06:00	06:00 - 06:59	Morning	36,7	110	46,0	46,0	17,87	600,69	128,91
2019/05/17	CH2205	Male	Three y/o	Treatment (Wk 1)	Treatment	Feed Day	No	06:05	06:00 - 06:59	Morning	37,2	70	19,4	19,4	18,36	566,99	99,28
2019/05/17	CH2206	Male	Three y/o	Treatment (Wk 1)	Treatment	Feed Day	No	06:05	06:00 - 06:59	Morning	36,6	76	95,6	95,6	17,77	573,39	154,28
2019/05/17	CH2205	Male	Three y/o	Treatment (Wk 1)	Treatment	Feed Day	No	06:10	06:00 - 06:59	Morning	37,3	98	68,2	68,2	18,46	592,41	142,53
2019/05/17	CH2206	Male	Three y/o	Treatment (Wk 1)	Treatment	Feed Day	No	06:10	06:00 - 06:59	Morning	37,0	113	118,6	118,6	18,16	602,58	161,80
2019/05/17	CH2205	Male	Three y/o	Treatment (Wk 1)	Treatment	Feed Day	No	06:15	06:00 - 06:59	Morning	37,3	118	179,2	179,2	18,46	605,61	176,26
2019/05/17	CH2206	Male	Three y/o	Treatment (Wk 1)	Treatment	Feed Day	No	06:15	06:00 - 06:59	Morning	37,1	121	324,8	324,8	18,26	607,35	197,23
2019/05/17	CH2205	Male	Three y/o	Treatment (Wk 1)	Treatment	Feed Day	No	06:20	06:00 - 06:59	Morning	37,4	191	95,8	95,8	18,55	637,33	154,35
2019/05/17	CH2206	Male	Three y/o	Treatment (Wk 1)	Treatment	Feed Day	No	06:20	06:00 - 06:59	Morning	37,3	132	117,6	117,6	18,46	613,32	161,50
2019/05/17	CH2205	Male	Three y/o	Treatment (Wk 1)	Treatment	Feed Day	No	06:25	06:00 - 06:59	Morning	37,4	123	103,4	103,4	18,55	608,49	157,01
2019/05/17	CH2206	Male	Three y/o	Treatment (Wk 1)	Treatment	Feed Day	No	06:25	06:00 - 06:59	Morning	37,4	103	104,4	104,4	18,55	596,00	157,35
2019/05/17	CH2205	Male	Three y/o	Treatment (Wk 1)	Treatment	Feed Day	No	06:30	06:00 - 06:59	Morning	37,4	162	111,4	111,4	18,55	626,88	159,61
2019/05/17	CH2206	Male	Three y/o	Treatment (Wk 1)	Treatment	Feed Day	No	06:30	06:00 - 06:59	Morning	37,5		128,4	128,4	18,65		164,57
2019/05/17	CH2205	Male	Three y/o	Treatment (Wk 1)	Treatment	Feed Day	No	06:35	06:00 - 06:59	Morning	37,4	117	93,6	93,6	18,55	605,02	153,54
2019/05/17	CH2206	Male	Three y/o	Treatment (Wk 1)	Treatment	Feed Day	No	06:35	06:00 - 06:59	Morning	37,5	123	112,2	112,2	18,65	608,49	159,86
2019/05/17	CH2205	Male	Three y/o	Treatment (Wk 1)	Treatment	Feed Day	No	06:40	06:00 - 06:59	Morning	37,5	108	381,2	381,2	18,65	599,39	202,91
2019/05/17	CH2206	Male	Three y/o	Treatment (Wk 1)	Treatment	Feed Day	No	06:40	06:00 - 06:59	Morning	37,5	102	163,8	163,8	18,65	595,30	173,10
2019/05/17	CH2205	Male	Three y/o	Treatment (Wk 1)	Treatment	Feed Day	No	06:45	06:00 - 06:59	Morning	37,6	111	150,0	150,0	18,75	601,33	170,02
2019/05/17	CH2206	Male	Three y/o	Treatment (Wk 1)	Treatment	Feed Day	No	06:45	06:00 - 06:59	Morning	37,5	121	280,8	280,8	18,65	607,35	192,08
2019/05/17	CH2205	Male	Three y/o	Treatment (Wk 1)	Treatment	Feed Day	No	06:50	06:00 - 06:59	Morning	37,5		44,8	44,8	18,65		127,99
2019/05/17	CH2206	Male	Three y/o	Treatment (Wk 1)	Treatment	Feed Day	No	06:50	06:00 - 06:59	Morning	37,5	62	99,0	99,0	18,65	557,33	155,49
2019/05/17	CH2205	Male	Three y/o	Treatment (Wk 1)	Treatment	Feed Day	No	06:55	06:00 - 06:59	Morning	37,6	112	29,2	29,2	18,75	601,96	113,27
2019/05/17	CH2206	Male	Three y/o	Treatment (Wk 1)	Treatment	Feed Day	No	06:55	06:00 - 06:59	Morning	37,5	115	90,0	90,0	18,65	603,82	152,17
2019/05/17	CH2205	Male	Three y/o	Treatment (Wk 1)	Treatment	Feed Day	No	07:00	07:00 - 07:59	Morning	37,5	90	43,4	43,4	18,65	586,16	126,90
2019/05/17	CH2206	Male	Three y/o	Treatment (Wk 1)	Treatment	Feed Day	No	07:00	07:00 - 07:59	Morning	37,6	103	105,0	105,0	18,75	596,00	157,55
2019/05/17	CH2205	Male	Three y/o	Treatment (Wk 1)	Treatment	Feed Day	No	07:05	07:00 - 07:59	Morning	37,6	160	195,2	195,2	18,75	626,07	179,26
2019/05/17	CH2206	Male	Three y/o	Treatment (Wk 1)	Treatment	Feed Day	No	07:05	07:00 - 07:59	Morning	37,5	106	100,6	100,6	18,65	598,06	156,05
2019/05/17	CH2205	Male	Three y/o	Treatment (Wk 1)	Treatment	Feed Day	No	07:10	07:00 - 07:59	Morning	37,7	103	79,4	79,4	18,85	596,00	147,81
2019/05/17	CH2206	Male	Three y/o	Treatment (Wk 1)	Treatment	Feed Day	No	07:10	07:00 - 07:59	Morning	37,7		182,8	182,8	18,85		176,96
2019/05/17	CH2205	Male	Three y/o	Treatment (Wk 1)	Treatment	Feed Day	No	07:15	07:00 - 07:59	Morning	37,8	103	23,6	23,6	18,95	596,00	105,97

2019/05/17	CH2206	Male	Three y/o	Treatment (Wk 1)	Treatment	Feed Day	No	07:15	07:00 - 07:59	Morning	37,7	111	254,2	254,2	18,85	601,33	188,57
2019/05/17	CH2205	Male	Three y/o	Treatment (Wk 1)	Treatment	Feed Day	No	07:20	07:00 - 07:59	Morning	37,7	86	60,8	60,8	18,85	582,77	138,55
2019/05/17	CH2206	Male	Three y/o	Treatment (Wk 1)	Treatment	Feed Day	No	07:20	07:00 - 07:59	Morning	37,8	121	121,8	121,8	18,95	607,35	162,73
2019/05/17	CH2205	Male	Three y/o	Treatment (Wk 1)	Treatment	Feed Day	No	07:25	07:00 - 07:59	Morning	37,9	134	176,8	176,8	19,05	614,33	175,78
2019/05/17	CH2206	Male	Three y/o	Treatment (Wk 1)	Treatment	Feed Day	No	07:25	07:00 - 07:59	Morning	37,9		110,6	110,6	19,05		159,36
2019/05/17	CH2205	Male	Three y/o	Treatment (Wk 1)	Treatment	Feed Day	No	07:30	07:00 - 07:59	Morning	37,9	53	336,6	336,6	19,05	544,43	198,49
2019/05/17	CH2206	Male	Three y/o	Treatment (Wk 1)	Treatment	Feed Day	No	07:30	07:00 - 07:59	Morning	37,8	177	127,2	127,2	18,95	632,55	164,24
2019/05/17	CH2205	Male	Three y/o	Treatment (Wk 1)	Treatment	Feed Day	No	07:35	07:00 - 07:59	Morning	37,7	94	262,2	262,2	18,85	589,37	189,66
2019/05/17	CH2206	Male	Three y/o	Treatment (Wk 1)	Treatment	Feed Day	No	07:35	07:00 - 07:59	Morning	37,8	129	271,8	271,8	18,95	611,75	190,93
2019/05/17	CH2205	Male	Three y/o	Treatment (Wk 1)	Treatment	Feed Day	No	07:40	07:00 - 07:59	Morning	37,7	103	70,8	70,8	18,85	596,00	143,83
2019/05/17	CH2206	Male	Three y/o	Treatment (Wk 1)	Treatment	Feed Day	No	07:40	07:00 - 07:59	Morning	37,7	58	161,4	161,4	18,85	551,90	172,59
2019/05/17	CH2205	Male	Three y/o	Treatment (Wk 1)	Treatment	Feed Day	No	07:45	07:00 - 07:59	Morning	37,6	90	135,0	135,0	18,75	586,16	166,33
2019/05/17	CH2206	Male	Three y/o	Treatment (Wk 1)	Treatment	Feed Day	No	07:45	07:00 - 07:59	Morning	37,7	180	153,6	153,6	18,85	633,61	170,85
2019/05/17	CH2205	Male	Three y/o	Treatment (Wk 1)	Treatment	Feed Day	No	07:50	07:00 - 07:59	Morning	37,7	101	932,6		18,85	594,59	
2019/05/17	CH2206	Male	Three y/o	Treatment (Wk 1)	Treatment	Feed Day	No	07:50	07:00 - 07:59	Morning	37,7	134	129,4	129,4	18,85	614,33	164,84
2019/05/17	CH2205	Male	Three y/o	Treatment (Wk 1)	Treatment	Feed Day	No	07:55	07:00 - 07:59	Morning	37,9	103	5,6	5,6	19,05	596,00	57,21
2019/05/17	CH2206	Male	Three y/o	Treatment (Wk 1)	Treatment	Feed Day	No	07:55	07:00 - 07:59	Morning	37,8		138,4	138,4	18,95		167,20
2019/05/17	CH2205	Male	Three y/o	Treatment (Wk 1)	Treatment	Feed Day	No	08:00	08:00 - 08:59	Late Morning	37,6	71	5,2	5,2	18,75	568,10	54,72
2019/05/17	CH2206	Male	Three y/o	Treatment (Wk 1)	Treatment	Feed Day	No	08:00	08:00 - 08:59	Late Morning	37,8	104	263,2	263,2	18,95	596,69	189,80
2019/05/17	CH2205	Male	Three y/o	Treatment (Wk 1)	Treatment	Feed Day	No	08:05	08:00 - 08:59	Late Morning	37,7		61,6	61,6	18,85		139,00
2019/05/17	CH2206	Male	Three y/o	Treatment (Wk 1)	Treatment	Feed Day	No	08:05	08:00 - 08:59	Late Morning	37,8	126	58,8	58,8	18,95	610,14	137,39
2019/05/17	CH2205	Male	Three y/o	Treatment (Wk 1)	Treatment	Feed Day	No	08:10	08:00 - 08:59	Late Morning	37,7	93	119,6	119,6	18,85	588,58	162,09
2019/05/17	CH2206	Male	Three y/o	Treatment (Wk 1)	Treatment	Feed Day	No	08:10	08:00 - 08:59	Late Morning	37,5	71	29,6	29,6	18,65	568,10	113,73
2019/05/17	CH2205	Male	Three y/o	Treatment (Wk 1)	Treatment	Feed Day	No	08:15	08:00 - 08:59	Late Morning	37,7	105	79,8	79,8	18,85	597,38	147,99
2019/05/17	CH2206	Male	Three y/o	Treatment (Wk 1)	Treatment	Feed Day	No	08:15	08:00 - 08:59	Late Morning	37,5	117	126,2	126,2	18,65	605,02	163,97
2019/05/17	CH2205	Male	Three y/o	Treatment (Wk 1)	Treatment	Feed Day	No	08:20	08:00 - 08:59	Late Morning	37,6	40	21,4	21,4	18,75	520,09	102,63
2019/05/17	CH2206	Male	Three y/o	Treatment (Wk 1)	Treatment	Feed Day	No	08:20	08:00 - 08:59	Late Morning	37,7	104	150,4	150,4	18,85	596,69	170,11
2019/05/17	CH2205	Male	Three y/o	Treatment (Wk 1)	Treatment	Feed Day	No	08:25	08:00 - 08:59	Late Morning	37,5	72	5,6	5,6	18,65	569,20	57,21
2019/05/17	CH2206	Male	Three y/o	Treatment (Wk 1)	Treatment	Feed Day	No	08:25	08:00 - 08:59	Late Morning	37,6	108	118,4	118,4	18,75	599,39	161,74
2019/05/17	CH2205	Male	Three y/o	Treatment (Wk 1)	Treatment	Feed Day	No	08:30	08:00 - 08:59	Late Morning	37,6		26,0	26,0	18,75		109,29
2019/05/17	CH2206	Male	Three y/o	Treatment (Wk 1)	Treatment	Feed Day	No	08:30	08:00 - 08:59	Late Morning	37,7	108	178,4	178,4	18,85	599,39	176,10
2019/05/17	CH2205	Male	Three y/o	Treatment (Wk 1)	Treatment	Feed Day	No	08:35	08:00 - 08:59	Late Morning	37,7		18,6	18,6	18,85		97,84
2019/05/17	CH2206	Male	Three y/o	Treatment (Wk 1)	Treatment	Feed Day	No	08:35	08:00 - 08:59	Late Morning	37,5	135	198,0	198,0	18,65	614,84	179,77
2019/05/17	CH2205	Male	Three y/o	Treatment (Wk 1)	Treatment	Feed Day	No	08:40	08:00 - 08:59	Late Morning	37,7	75	80,2	80,2	18,85	572,36	148,16
2019/05/17	CH2206	Male	Three y/o	Treatment (Wk 1)	Treatment	Feed Day	No	08:40	08:00 - 08:59	Late Morning	37,7	104	138,2	138,2	18,85	596,69	167,15
2019/05/17	CH2205	Male	Three y/o	Treatment (Wk 1)	Treatment	Feed Day	No	08:45	08:00 - 08:59	Late Morning	37,7	92	76,0	76,0	18,85	587,78	146,29
2019/05/17	CH2206	Male	Three y/o	Treatment (Wk 1)	Treatment	Feed Day	No	08:45	08:00 - 08:59	Late Morning	37,8	110	154,8	154,8	18,95	600,69	171,12
2019/05/17	CH2205	Male	Three y/o	Treatment (Wk 1)	Treatment	Feed Day	No	08:50	08:00 - 08:59	Late Morning	37,7	70	85,2	85,2	18,85	566,99	150,27
2019/05/17	CH2206	Male	Three y/o	Treatment (Wk 1)	Treatment	Feed Day	No	08:50	08:00 - 08:59	Late Morning	37,8	100	132,6	132,6	18,95	593,87	165,70
2019/05/17	CH2205	Male	Three y/o	Treatment (Wk 1)	Treatment	Feed Day	No	08:55	08:00 - 08:59	Late Morning	37,9	66	254,8	254,8	19,05	562,34	188,65
2019/05/17	CH2206	Male	Three y/o	Treatment (Wk 1)	Treatment	Feed Day	No	08:55	08:00 - 08:59	Late Morning	37,9	105	58,2	58,2	19,05	597,38	137,04
2019/05/17	CH2205	Male	Three y/o	Treatment (Wk 1)	Treatment	Feed Day	No	09:00	09:00 - 09:59	Late Morning	37,7	67	14,2	14,2	18,85	563,53	88,65
2019/05/17	CH2206	Male	Three y/o	Treatment (Wk 1)	Treatment	Feed Day	No	09:00	09:00 - 09:59	Late Morning	37,8	150	333,8	333,8	18,95	621,86	198,20
2019/05/17	CH2205	Male	Three y/o	Treatment (Wk 1)	Treatment	Feed Day	No	09:05	09:00 - 09:59	Late Morning	37,6		56,2	56,2	18,75		135,83
2019/05/17	CH2206	Male	Three y/o	Treatment (Wk 1)	Treatment	Feed Day	No	09:05	09:00 - 09:59	Late Morning	37,8	141	75,6	75,6	18,95	617,75	146,11
2019/05/17	CH2205	Male	Three y/o	Treatment (Wk 1)	Treatment	Feed Day	No	09:10	09:00 - 09:59	Late Morning	37,6		12,6	12,6	18,75		84,59
2019/05/17	CH2206	Male	Three y/o	Treatment (Wk 1)	Treatment	Feed Day	No	09:10	09:00 - 09:59	Late Morning	37,8	103	103,4	103,4	18,95	596,00	157,01
2019/05/17	CH2205	Male	Three y/o	Treatment (Wk 1)	Treatment	Feed Day	No	09:15	09:00 - 09:59	Late Morning	37,5	57	14,4	14,4	18,65	550,47	89,12
2019/05/17	CH2206	Male	Three y/o	Treatment (Wk 1)	Treatment	Feed Day	No	09:15	09:00 - 09:59	Late Morning	37,8		112,2	112,2	18,95		159,86
2019/05/17	CH2205	Male	Three y/o	Treatment (Wk 1)	Treatment	Feed Day	No	09:20	09:00 - 09:59	Late Morning	37,6		17,6	17,6	18,75		95,95
2019/05/17	CH2206	Male	Three y/o	Treatment (Wk 1)	Treatment	Feed Day	No	09:20	09:00 - 09:59	Late Morning	37,8	121	75,6	75,6	18,95	607,35	146,11
2019/05/17	CH2205	Male	Three y/o	Treatment (Wk 1)	Treatment	Feed Day	No	09:25	09:00 - 09:59	Late Morning	37,5		81,2	81,2	18,65		148,59
2019/05/17	CH2206	Male	Three y/o	Treatment (Wk 1)	Treatment	Feed Day	No	09:25	09:00 - 09:59	Late Morning	37,7	120	181,2	181,2	18,85	606,78	176,65
2019/05/17	CH2205	Male	Three y/o	Treatment (Wk 1)	Treatment	Feed Day	No	09:30	09:00 - 09:59	Late Morning	37,6	99	298,4	298,4	18,75	593,14	194,23
2019/05/17	CH2206	Male	Three y/o	Treatment (Wk 1)	Treatment	Feed Day	No	09:30	09:00 - 09:59	Late Morning	37,8	115	108,2	108,2	18,95	603,82	158,59
2019/05/17	CH2205	Male	Three y/o	Treatment (Wk 1)	Treatment	Feed Day	No	09:35	09:00 - 09:59	Late Morning	37,7		19,8	19,8	18,85		99,97
2019/05/17	CH2206	Male	Three y/o	Treatment (Wk 1)	Treatment	Feed Day	No	09:35	09:00 - 09:59	Late Morning	37,8	131	146,4	146,4	18,95	612,80	169,17
2019/05/17	CH2205	Male	Three y/o	Treatment (Wk 1)	Treatment	Feed Day	No	09:40	09:00 - 09:59	Late Morning	37,7	103	63,2	63,2	18,85	596,00	139,89
2019/05/17	CH2206	Male	Three y/o	Treatment (Wk 1)	Treatment	Feed Day	No	09:40	09:00 - 09:59	Late Morning	37,8	125	62,2	62,2	18,95	609,60	139,34
2019/05/17	CH2205	Male	Three y/o	Treatment (Wk 1)	Treatment	Feed Day	No	09:45	09:00 - 09:59	Late Morning	37,9	97	62,2	62,2	19,05	591,66	139,34
2019/05/17	CH2206	Male	Three y/o	Treatment (Wk 1)	Treatment	Feed Day	No	09:45	09:00 - 09:59	Late Morning	37,9	104	76,4	76,4	19,05	596,69	146,48
2019/05/17	CH2205	Male	Three y/o	Treatment (Wk 1)	Treatment	Feed Day	No	09:50	09:00 - 09:59	Late Morning	37,8	70	5,6	5,6	18,95	566,99	57,21
2019/05/17	CH2206	Male	Three y/o	Treatment (Wk 1)	Treatment	Feed Day	No	09:50	09:00 - 09:59	Late Morning	37,9	123	26,8	26,8	19,05	608,49	110,33
2019/05/17	CH2205	Male	Three y/o	Treatment (Wk 1)	Treatment	Feed Day	No	09:55	09:00 - 09:59	Late Morning	37,7	66	34,4	34,4	18,85	562,34	118,90
2019/05/17	CH2206	Male	Three y/o	Treatment (Wk 1)	Treatment	Feed Day	No	09:55	09:00 - 09:59	Late Morning	37,8	72	29,2	29,2	18,95	569,20	113,27
2019/05/17	CH2205	Male	Three y/o	Treatment (Wk 1)	Treatment	Feed Day	No	10:00	10:00 - 10:59	Late Morning	37,7	75	15,4	15,4	18,85	572,36	91,41
2019/05/17	CH2206	Male	Three y/o	Treatment (Wk 1)	Treatment	Feed Day	No	10:00	10:00 - 10:59	Late Morning	37,7	51	25,8	25,8	18,85	541,20	109,02
2019/05/17	CH2205	Male	Three y/o	Treatment (Wk 1)	Treatment	Feed Day	No	10:05	10:00 - 10:59	Late Morning	37,7	52	17,4	17,4	18,85	542,83	95,56
2019/05/17	CH2206	Male	Three y/o	Treatment (Wk 1)	Treatment	Feed Day	No	10:05	10:00 - 10:59	Late Morning	37,8	71	23,2	23,2	18,95	568,10	105,39
2019/05/17	CH2205	Male	Three y/o	Treatment (Wk 1)	Treatment	Feed Day	No	10:10	10:00 - 10:59	Late Morning	37,7	60	20,6	20,6	18,85	554,67	101,32

2019/05/17	CH2206	Male	Three y/o	Treatment (Wk 1)	Treatment	Feed Day	No	10:10	10:00 - 10:59	Late Morning	37,7	57	29,2	29,2	18,85	550,47	113,27
2019/05/17	CH2205	Male	Three y/o	Treatment (Wk 1)	Treatment	Feed Day	No	10:15	10:00 - 10:59	Late Morning	37,6	63	22,0	22,0	18,75	558,62	103,57
2019/05/17	CH2206	Male	Three y/o	Treatment (Wk 1)	Treatment	Feed Day	No	10:15	10:00 - 10:59	Late Morning	37,7	83	40,4	40,4	18,85	580,11	124,43
2019/05/17	CH2205	Male	Three y/o	Treatment (Wk 1)	Treatment	Feed Day	No	10:20	10:00 - 10:59	Late Morning	37,6	44	25,0	25,0	18,75	528,51	107,94
2019/05/17	CH2206	Male	Three y/o	Treatment (Wk 1)	Treatment	Feed Day	No	10:20	10:00 - 10:59	Late Morning	37,7	61	21,4	21,4	18,85	556,01	102,63
2019/05/17	CH2205	Male	Three y/o	Treatment (Wk 1)	Treatment	Feed Day	No	10:25	10:00 - 10:59	Late Morning	37,6	47	20,8	20,8	18,75	534,23	101,65
2019/05/17	CH2206	Male	Three y/o	Treatment (Wk 1)	Treatment	Feed Day	No	10:25	10:00 - 10:59	Late Morning	37,8	62	26,8	26,8	18,95	557,33	110,33
2019/05/17	CH2205	Male	Three y/o	Treatment (Wk 1)	Treatment	Feed Day	Yes	10:30	10:00 - 10:59	Late Morning	37,6	45	23,8	23,8	18,75	530,47	106,26
2019/05/17	CH2206	Male	Three y/o	Treatment (Wk 1)	Treatment	Feed Day	Yes	10:30	10:00 - 10:59	Late Morning	37,7	72	40,6	40,6	18,85	569,20	124,60
2019/05/17	CH2205	Male	Three y/o	Treatment (Wk 1)	Treatment	Feed Day	Yes	10:35	10:00 - 10:59	Late Morning	37,6	57	20,8	20,8	18,75	550,47	101,65
2019/05/17	CH2206	Male	Three y/o	Treatment (Wk 1)	Treatment	Feed Day	Yes	10:35	10:00 - 10:59	Late Morning	37,7	69	40,0	40,0	18,85	565,86	124,09
2019/05/17	CH2205	Male	Three y/o	Treatment (Wk 1)	Treatment	Feed Day	Yes	10:40	10:00 - 10:59	Late Morning	37,5	40	26,4	26,4	18,65	520,09	109,81
2019/05/17	CH2206	Male	Three y/o	Treatment (Wk 1)	Treatment	Feed Day	Yes	10:40	10:00 - 10:59	Late Morning	37,7	55	34,0	34,0	18,85	547,52	118,49
2019/05/17	CH2205	Male	Three y/o	Treatment (Wk 1)	Treatment	Feed Day	Yes	10:45	10:00 - 10:59	Late Morning	37,5	39	20,2	20,2	18,65	517,82	100,65
2019/05/17	CH2206	Male	Three y/o	Treatment (Wk 1)	Treatment	Feed Day	Yes	10:45	10:00 - 10:59	Late Morning	37,7	51	67,0	67,0	18,85	541,20	141,92
2019/05/17	CH2205	Male	Three y/o	Treatment (Wk 1)	Treatment	Feed Day	Yes	10:50	10:00 - 10:59	Late Morning	37,5	53	15,0	15,0	18,65	544,43	90,51
2019/05/17	CH2206	Male	Three y/o	Treatment (Wk 1)	Treatment	Feed Day	Yes	10:50	10:00 - 10:59	Late Morning	37,7	59	63,4	63,4	18,85	553,30	140,00
2019/05/17	CH2205	Male	Three y/o	Treatment (Wk 1)	Treatment	Feed Day	Yes	10:55	10:00 - 10:59	Late Morning	37,5	62	21,0	21,0	18,65	557,33	101,98
2019/05/17	CH2206	Male	Three y/o	Treatment (Wk 1)	Treatment	Feed Day	Yes	10:55	10:00 - 10:59	Late Morning	37,7	56	64,8	64,8	18,85	549,01	140,76
2019/05/17	CH2205	Male	Three y/o	Treatment (Wk 1)	Treatment	Feed Day	Yes	11:00	11:00 - 11:59	Late Morning	37,5	53	17,6	17,6	18,65	544,43	95,95
2019/05/17	CH2206	Male	Three y/o	Treatment (Wk 1)	Treatment	Feed Day	Yes	11:00	11:00 - 11:59	Late Morning	37,7	55	40,6	40,6	18,85	547,52	124,60
2019/05/17	CH2205	Male	Three y/o	Treatment (Wk 1)	Treatment	Feed Day	Yes	11:05	11:00 - 11:59	Late Morning	37,5	52	25,2	25,2	18,65	542,83	108,22
2019/05/17	CH2206	Male	Three y/o	Treatment (Wk 1)	Treatment	Feed Day	Yes	11:05	11:00 - 11:59	Late Morning	37,7	44	45,8	45,8	18,85	528,51	128,76
2019/05/17	CH2205	Male	Three y/o	Treatment (Wk 1)	Treatment	Feed Day	Yes	11:10	11:00 - 11:59	Late Morning	37,5	73	53,8	53,8	18,65	570,27	134,32
2019/05/17	CH2206	Male	Three y/o	Treatment (Wk 1)	Treatment	Feed Day	Yes	11:10	11:00 - 11:59	Late Morning	37,5	98	74,4	74,4	18,65	592,41	145,55
2019/05/17	CH2205	Male	Three y/o	Treatment (Wk 1)	Treatment	Feed Day	Yes	11:15	11:00 - 11:59	Late Morning	37,6		65,4	65,4	18,75		141,08
2019/05/17	CH2206	Male	Three y/o	Treatment (Wk 1)	Treatment	Feed Day	Yes	11:15	11:00 - 11:59	Late Morning	37,5	112	108,2	108,2	18,65	601,96	158,59
2019/05/17	CH2205	Male	Three y/o	Treatment (Wk 1)	Treatment	Feed Day	Yes	11:20	11:00 - 11:59	Late Morning	37,5	152	52,4	52,4	18,65	622,73	133,41
2019/05/17	CH2206	Male	Three y/o	Treatment (Wk 1)	Treatment	Feed Day	Yes	11:20	11:00 - 11:59	Late Morning	37,5	91	33,6	33,6	18,65	586,98	118,09
2019/05/17	CH2205	Male	Three y/o	Treatment (Wk 1)	Treatment	Feed Day	Yes	11:25	11:00 - 11:59	Late Morning	37,5	103	185,2	185,2	18,65	596,00	177,42
2019/05/17	CH2206	Male	Three y/o	Treatment (Wk 1)	Treatment	Feed Day	Yes	11:25	11:00 - 11:59	Late Morning	37,5	129	41,8	41,8	18,65	611,75	125,60
2019/05/17	CH2205	Male	Three y/o	Treatment (Wk 1)	Treatment	Feed Day	No	11:30	11:00 - 11:59	Late Morning	37,5		65,4	65,4	18,65		141,08
2019/05/17	CH2206	Male	Three y/o	Treatment (Wk 1)	Treatment	Feed Day	No	11:30	11:00 - 11:59	Late Morning	37,5	104	82,2	82,2	18,65	596,69	149,02
2019/05/17	CH2205	Male	Three y/o	Treatment (Wk 1)	Treatment	Feed Day	No	11:35	11:00 - 11:59	Late Morning	37,5	98	30,4	30,4	18,65	592,41	114,65
2019/05/17	CH2206	Male	Three y/o	Treatment (Wk 1)	Treatment	Feed Day	No	11:35	11:00 - 11:59	Late Morning	37,5		53,4	53,4	18,65		134,06
2019/05/17	CH2205	Male	Three y/o	Treatment (Wk 1)	Treatment	Feed Day	No	11:40	11:00 - 11:59	Late Morning	37,4	78	29,0	29,0	18,55	575,38	113,03
2019/05/17	CH2206	Male	Three y/o	Treatment (Wk 1)	Treatment	Feed Day	No	11:40	11:00 - 11:59	Late Morning	37,4	99	84,8	84,8	18,55	593,14	150,10
2019/05/17	CH2205	Male	Three y/o	Treatment (Wk 1)	Treatment	Feed Day	No	11:45	11:00 - 11:59	Late Morning	37,5	75	234,4	234,4	18,65	572,36	185,71
2019/05/17	CH2206	Male	Three y/o	Treatment (Wk 1)	Treatment	Feed Day	No	11:45	11:00 - 11:59	Late Morning	37,4	102	28,4	28,4	18,55	595,30	112,31
2019/05/17	CH2205	Male	Three y/o	Treatment (Wk 1)	Treatment	Feed Day	No	11:50	11:00 - 11:59	Late Morning	37,5		76,2	76,2	18,65		146,38
2019/05/17	CH2206	Male	Three y/o	Treatment (Wk 1)	Treatment	Feed Day	No	11:50	11:00 - 11:59	Late Morning	37,4	84	37,2	37,2	18,55	581,01	121,59
2019/05/17	CH2205	Male	Three y/o	Treatment (Wk 1)	Treatment	Feed Day	No	11:55	11:00 - 11:59	Late Morning	37,4	174	33,0	33,0	18,55	631,46	117,47
2019/05/17	CH2206	Male	Three y/o	Treatment (Wk 1)	Treatment	Feed Day	No	11:55	11:00 - 11:59	Late Morning	37,5	80	41,0	41,0	18,65	577,31	124,94
2019/05/17	CH2205	Male	Three y/o	Treatment (Wk 1)	Treatment	Feed Day	No	12:00	12:00 - 12:59	Afternoon	37,4	102	51,6	51,6	18,55	595,30	132,87
2019/05/17	CH2206	Male	Three y/o	Treatment (Wk 1)	Treatment	Feed Day	No	12:00	12:00 - 12:59	Afternoon	37,5	112	42,2	42,2	18,65	601,96	125,93
2019/05/17	CH2205	Male	Three y/o	Treatment (Wk 1)	Treatment	Feed Day	No	12:05	12:00 - 12:59	Afternoon	37,5	92	97,6	97,6	18,65	587,78	155,00
2019/05/17	CH2206	Male	Three y/o	Treatment (Wk 1)	Treatment	Feed Day	No	12:05	12:00 - 12:59	Afternoon	37,5	73	27,6	27,6	18,65	570,27	111,33
2019/05/17	CH2205	Male	Three y/o	Treatment (Wk 1)	Treatment	Feed Day	No	12:10	12:00 - 12:59	Afternoon	37,6	83	8,4	8,4	18,75	580,11	70,86
2019/05/17	CH2206	Male	Three y/o	Treatment (Wk 1)	Treatment	Feed Day	No	12:10	12:00 - 12:59	Afternoon	37,5	55	41,4	41,4	18,65	547,52	125,27
2019/05/17	CH2205	Male	Three y/o	Treatment (Wk 1)	Treatment	Feed Day	No	12:15	12:00 - 12:59	Afternoon	37,6		16,4	16,4	18,75		93,55
2019/05/17	CH2206	Male	Three y/o	Treatment (Wk 1)	Treatment	Feed Day	No	12:15	12:00 - 12:59	Afternoon	37,5	72	22,2	22,2	18,65	569,20	103,88
2019/05/17	CH2205	Male	Three y/o	Treatment (Wk 1)	Treatment	Feed Day	No	12:20	12:00 - 12:59	Afternoon	37,6	38	34,4	34,4	18,75	515,48	118,90
2019/05/17	CH2206	Male	Three y/o	Treatment (Wk 1)	Treatment	Feed Day	No	12:20	12:00 - 12:59	Afternoon	37,5	99	10,2	10,2	18,65	593,14	77,43
2019/05/17	CH2205	Male	Three y/o	Treatment (Wk 1)	Treatment	Feed Day	No	12:25	12:00 - 12:59	Afternoon	37,6	68	16,0	16,0	18,75	564,71	92,71
2019/05/17	CH2206	Male	Three y/o	Treatment (Wk 1)	Treatment	Feed Day	No	12:25	12:00 - 12:59	Afternoon	37,5	57	6,6	6,6	18,65	550,47	62,73
2019/05/17	CH2205	Male	Three y/o	Treatment (Wk 1)	Treatment	Feed Day	No	12:30	12:00 - 12:59	Afternoon	37,6	65	17,4	17,4	18,75	561,12	95,56
2019/05/17	CH2206	Male	Three y/o	Treatment (Wk 1)	Treatment	Feed Day	No	12:30	12:00 - 12:59	Afternoon	37,5	67	6,4	6,4	18,65	563,53	61,70
2019/05/17	CH2205	Male	Three y/o	Treatment (Wk 1)	Treatment	Feed Day	No	12:35	12:00 - 12:59	Afternoon	37,6	62	15,2	15,2	18,75	557,33	90,96
2019/05/17	CH2206	Male	Three y/o	Treatment (Wk 1)	Treatment	Feed Day	No	12:35	12:00 - 12:59	Afternoon	37,5	86	11,8	11,8	18,65	582,77	82,36
2019/05/17	CH2205	Male	Three y/o	Treatment (Wk 1)	Treatment	Feed Day	No	12:40	12:00 - 12:59	Afternoon	37,6	70	22,6	22,6	18,75	566,99	104,49
2019/05/17	CH2206	Male	Three y/o	Treatment (Wk 1)	Treatment	Feed Day	No	12:40	12:00 - 12:59	Afternoon	37,5	72	27,8	27,8	18,65	569,20	111,58
2019/05/17	CH2205	Male	Three y/o	Treatment (Wk 1)	Treatment	Feed Day	No	12:45	12:00 - 12:59	Afternoon	37,6	123	25,6	25,6	18,75	608,49	108,76
2019/05/17	CH2206	Male	Three y/o	Treatment (Wk 1)	Treatment	Feed Day	No	12:45	12:00 - 12:59	Afternoon	37,7	104	8,4	8,4	18,85	596,69	70,86
2019/05/17	CH2205	Male	Three y/o	Treatment (Wk 1)	Treatment	Feed Day	No	12:50	12:00 - 12:59	Afternoon	37,6	70	23,4	23,4	18,75	566,99	105,68
2019/05/17	CH2206	Male	Three y/o	Treatment (Wk 1)	Treatment	Feed Day	No	12:50	12:00 - 12:59	Afternoon	37,7	81	3,2	3,2	18,85	578,26	38,49
2019/05/17	CH2205	Male	Three y/o	Treatment (Wk 1)	Treatment	Feed Day	No	12:55	12:00 - 12:59	Afternoon	37,6	63	17,2	17,2	18,75	558,62	95,17
2019/05/17	CH2206	Male	Three y/o	Treatment (Wk 1)	Treatment	Feed Day	No	12:55	12:00 - 12:59	Afternoon	37,7	48	7,6	7,6	18,85	536,04	67,49
2019/05/17	CH2205	Male	Three y/o	Treatment (Wk 1)	Treatment	Feed Day	No	13:00	13:00 - 13:59	Afternoon	37,6	53	20,0	20,0	18,75	544,43	100,31
2019/05/17	CH2206	Male	Three y/o	Treatment (Wk 1)	Treatment	Feed Day	No	13:00	13:00 - 13:59	Afternoon	37,7	79	9,2	9,2	18,85	576,36	73,94
2019/05/17	CH2205	Male	Three y/o	Treatment (Wk 1)	Treatment	Feed Day	No	13:05	13:00 - 13:59	Afternoon	37,6	84	18,2	18,2	18,75	581,01	97,10

2019/05/17	CH2206	Male	Three y/o	Treatment (Wk 1)	Treatment	Feed Day	No	13:05	13:00 - 13:59	Afternoon	37,7	64	11,2	11,2	18,85	559,88	80,59
2019/05/17	CH2205	Male	Three y/o	Treatment (Wk 1)	Treatment	Feed Day	No	13:10	13:00 - 13:59	Afternoon	37,6	52	22,4	22,4	18,75	542,83	104,19
2019/05/17	CH2206	Male	Three y/o	Treatment (Wk 1)	Treatment	Feed Day	No	13:10	13:00 - 13:59	Afternoon	37,7	77	12,8	12,8	18,85	574,39	85,12
2019/05/17	CH2205	Male	Three y/o	Treatment (Wk 1)	Treatment	Feed Day	No	13:15	13:00 - 13:59	Afternoon	37,6	64	14,6	14,6	18,75	559,88	89,59
2019/05/17	CH2206	Male	Three y/o	Treatment (Wk 1)	Treatment	Feed Day	No	13:15	13:00 - 13:59	Afternoon	37,7	75	13,4	13,4	18,85	572,36	86,68
2019/05/17	CH2205	Male	Three y/o	Treatment (Wk 1)	Treatment	Feed Day	No	13:20	13:00 - 13:59	Afternoon	37,6	56	24,0	24,0	18,75	549,01	106,55
2019/05/17	CH2206	Male	Three y/o	Treatment (Wk 1)	Treatment	Feed Day	No	13:20	13:00 - 13:59	Afternoon	37,7	45	19,8	19,8	18,85	530,47	99,97
2019/05/17	CH2205	Male	Three y/o	Treatment (Wk 1)	Treatment	Feed Day	No	13:25	13:00 - 13:59	Afternoon	37,7	46	27,6	27,6	18,85	532,38	111,33
2019/05/17	CH2206	Male	Three y/o	Treatment (Wk 1)	Treatment	Feed Day	No	13:25	13:00 - 13:59	Afternoon	37,7	137	31,8	31,8	18,85	615,83	116,20
2019/05/17	CH2205	Male	Three y/o	Treatment (Wk 1)	Treatment	Feed Day	No	13:30	13:00 - 13:59	Afternoon	37,6	62	15,2	15,2	18,75	557,33	90,96
2019/05/17	CH2206	Male	Three y/o	Treatment (Wk 1)	Treatment	Feed Day	No	13:30	13:00 - 13:59	Afternoon	37,8	94	39,0	39,0	18,95	589,37	123,22
2019/05/17	CH2205	Male	Three y/o	Treatment (Wk 1)	Treatment	Feed Day	No	13:35	13:00 - 13:59	Afternoon	37,5	86	16,8	16,8	18,65	582,77	94,37
2019/05/17	CH2206	Male	Three y/o	Treatment (Wk 1)	Treatment	Feed Day	No	13:35	13:00 - 13:59	Afternoon	37,7	90	44,6	44,6	18,85	586,16	127,84
2019/05/17	CH2205	Male	Three y/o	Treatment (Wk 1)	Treatment	Feed Day	No	13:40	13:00 - 13:59	Afternoon	37,5	51	16,0	16,0	18,65	541,20	92,71
2019/05/17	CH2206	Male	Three y/o	Treatment (Wk 1)	Treatment	Feed Day	No	13:40	13:00 - 13:59	Afternoon	37,8	84	46,6	46,6	18,95	581,01	129,35
2019/05/17	CH2205	Male	Three y/o	Treatment (Wk 1)	Treatment	Feed Day	No	13:45	13:00 - 13:59	Afternoon	37,6	57	1,2	1,2	18,75	550,47	5,99
2019/05/17	CH2206	Male	Three y/o	Treatment (Wk 1)	Treatment	Feed Day	No	13:45	13:00 - 13:59	Afternoon	37,8	91	39,6	39,6	18,95	586,98	123,74
2019/05/17	CH2205	Male	Three y/o	Treatment (Wk 1)	Treatment	Feed Day	No	13:50	13:00 - 13:59	Afternoon	37,7	57	3,4	3,4	18,85	550,47	40,51
2019/05/17	CH2206	Male	Three y/o	Treatment (Wk 1)	Treatment	Feed Day	No	13:50	13:00 - 13:59	Afternoon	37,8	103	39,8	39,8	18,95	596,00	123,91
2019/05/17	CH2205	Male	Three y/o	Treatment (Wk 1)	Treatment	Feed Day	No	13:55	13:00 - 13:59	Afternoon	37,7	58	4,2	4,2	18,85	551,90	47,57
2019/05/17	CH2206	Male	Three y/o	Treatment (Wk 1)	Treatment	Feed Day	No	13:55	13:00 - 13:59	Afternoon	37,8	86	17,4	17,4	18,95	582,77	95,56
2019/05/17	CH2205	Male	Three y/o	Treatment (Wk 1)	Treatment	Feed Day	No	14:00	14:00 - 14:59	Afternoon	37,7	84	3,0	3,0	18,85	581,01	36,34
2019/05/17	CH2206	Male	Three y/o	Treatment (Wk 1)	Treatment	Feed Day	No	14:00	14:00 - 14:59	Afternoon	37,8	91	15,8	15,8	18,95	586,98	92,28
2019/05/17	CH2205	Male	Three y/o	Treatment (Wk 1)	Treatment	Feed Day	No	14:05	14:00 - 14:59	Afternoon	37,7	69	4,2	4,2	18,85	565,86	47,57
2019/05/17	CH2206	Male	Three y/o	Treatment (Wk 1)	Treatment	Feed Day	No	14:05	14:00 - 14:59	Afternoon	37,8	100	11,2	11,2	18,95	593,87	80,59
2019/05/17	CH2205	Male	Three y/o	Treatment (Wk 1)	Treatment	Feed Day	No	14:10	14:00 - 14:59	Afternoon	37,7	80	8,6	8,6	18,85	577,31	71,66
2019/05/17	CH2206	Male	Three y/o	Treatment (Wk 1)	Treatment	Feed Day	No	14:10	14:00 - 14:59	Afternoon	37,9	95	11,0	11,0	19,05	590,14	79,98
2019/05/17	CH2205	Male	Three y/o	Treatment (Wk 1)	Treatment	Feed Day	No	14:15	14:00 - 14:59	Afternoon	37,7		14,2	14,2	18,85		88,65
2019/05/17	CH2206	Male	Three y/o	Treatment (Wk 1)	Treatment	Feed Day	No	14:15	14:00 - 14:59	Afternoon	37,9	86	13,6	13,6	19,05	582,77	87,18
2019/05/17	CH2205	Male	Three y/o	Treatment (Wk 1)	Treatment	Feed Day	No	14:20	14:00 - 14:59	Afternoon	37,7		11,4	11,4	18,85		81,19
2019/05/17	CH2206	Male	Three y/o	Treatment (Wk 1)	Treatment	Feed Day	No	14:20	14:00 - 14:59	Afternoon	37,9	113	4,8	4,8	19,05	602,58	52,04
2019/05/17	CH2205	Male	Three y/o	Treatment (Wk 1)	Treatment	Feed Day	No	14:25	14:00 - 14:59	Afternoon	37,7		20,0	20,0	18,85		100,31
2019/05/17	CH2206	Male	Three y/o	Treatment (Wk 1)	Treatment	Feed Day	No	14:25	14:00 - 14:59	Afternoon	37,9	92	14,8	14,8	19,05	587,78	90,05
2019/05/17	CH2205	Male	Three y/o	Treatment (Wk 1)	Treatment	Feed Day	No	14:30	14:00 - 14:59	Afternoon	37,9		4,8	4,8	19,05		52,04
2019/05/17	CH2206	Male	Three y/o	Treatment (Wk 1)	Treatment	Feed Day	No	14:30	14:00 - 14:59	Afternoon	37,9	120	31,2	31,2	19,05	606,78	115,54
2019/05/17	CH2205	Male	Three y/o	Treatment (Wk 1)	Treatment	Feed Day	No	14:35	14:00 - 14:59	Afternoon	37,9	88	32,4	32,4	19,05	584,49	116,84
2019/05/17	CH2206	Male	Three y/o	Treatment (Wk 1)	Treatment	Feed Day	No	14:35	14:00 - 14:59	Afternoon	37,9	91	97,8	97,8	19,05	586,98	155,07
2019/05/17	CH2205	Male	Three y/o	Treatment (Wk 1)	Treatment	Feed Day	No	14:40	14:00 - 14:59	Afternoon	37,7		15,8	15,8	18,85		92,28
2019/05/17	CH2206	Male	Three y/o	Treatment (Wk 1)	Treatment	Feed Day	No	14:40	14:00 - 14:59	Afternoon	37,9	103	34,4	34,4	19,05	596,00	118,90
2019/05/17	CH2205	Male	Three y/o	Treatment (Wk 1)	Treatment	Feed Day	No	14:45	14:00 - 14:59	Afternoon	37,7	77	14,2	14,2	18,85	574,39	88,65
2019/05/17	CH2206	Male	Three y/o	Treatment (Wk 1)	Treatment	Feed Day	No	14:45	14:00 - 14:59	Afternoon	37,9	112	33,2	33,2	19,05	601,96	117,68
2019/05/17	CH2205	Male	Three y/o	Treatment (Wk 1)	Treatment	Feed Day	No	14:50	14:00 - 14:59	Afternoon	37,7		42,2	42,2	18,85		125,93
2019/05/17	CH2206	Male	Three y/o	Treatment (Wk 1)	Treatment	Feed Day	No	14:50	14:00 - 14:59	Afternoon	37,9	125	194,4	194,4	19,05	609,60	179,12
2019/05/17	CH2205	Male	Three y/o	Treatment (Wk 1)	Treatment	Feed Day	No	14:55	14:00 - 14:59	Afternoon	37,7	167	24,6	24,6	18,85	628,84	107,39
2019/05/17	CH2206	Male	Three y/o	Treatment (Wk 1)	Treatment	Feed Day	No	14:55	14:00 - 14:59	Afternoon	37,9	115	45,8	45,8	19,05	603,82	128,76
2019/05/17	CH2205	Male	Three y/o	Treatment (Wk 1)	Treatment	Feed Day	No	15:00	15:00 - 15:59	Afternoon	37,7		10,8	10,8	18,85		79,36
2019/05/17	CH2206	Male	Three y/o	Treatment (Wk 1)	Treatment	Feed Day	No	15:00	15:00 - 15:59	Afternoon	37,9	111	16,4	16,4	19,05	601,33	93,55
2019/05/17	CH2205	Male	Three y/o	Treatment (Wk 1)	Treatment	Feed Day	No	15:05	15:00 - 15:59	Afternoon	37,7	75	22,6	22,6	18,85	572,36	104,49
2019/05/17	CH2206	Male	Three y/o	Treatment (Wk 1)	Treatment	Feed Day	No	15:05	15:00 - 15:59	Afternoon	37,8	84	77,8	77,8	18,95	581,01	147,11
2019/05/17	CH2205	Male	Three y/o	Treatment (Wk 1)	Treatment	Feed Day	No	15:10	15:00 - 15:59	Afternoon	37,9	65	32,2	32,2	19,05	561,12	116,63
2019/05/17	CH2206	Male	Three y/o	Treatment (Wk 1)	Treatment	Feed Day	No	15:10	15:00 - 15:59	Afternoon	37,9	115	35,2	35,2	19,05	603,82	119,69
2019/05/17	CH2205	Male	Three y/o	Treatment (Wk 1)	Treatment	Feed Day	No	15:15	15:00 - 15:59	Afternoon	37,9	76	24,8	24,8	19,05	573,39	107,67
2019/05/17	CH2206	Male	Three y/o	Treatment (Wk 1)	Treatment	Feed Day	No	15:15	15:00 - 15:59	Afternoon	37,9	86	19,6	19,6	19,05	582,77	99,63
2019/05/17	CH2205	Male	Three y/o	Treatment (Wk 1)	Treatment	Feed Day	No	15:20	15:00 - 15:59	Afternoon	37,9	71	21,8	21,8	19,05	568,10	103,26
2019/05/17	CH2206	Male	Three y/o	Treatment (Wk 1)	Treatment	Feed Day	No	15:20	15:00 - 15:59	Afternoon	37,9	81	15,2	15,2	19,05	578,26	90,96
2019/05/17	CH2205	Male	Three y/o	Treatment (Wk 1)	Treatment	Feed Day	No	15:25	15:00 - 15:59	Afternoon	37,9	55	25,0	25,0	19,05	547,52	107,94
2019/05/17	CH2206	Male	Three y/o	Treatment (Wk 1)	Treatment	Feed Day	No	15:25	15:00 - 15:59	Afternoon	37,9	79	8,4	8,4	19,05	576,36	70,86
2019/05/17	CH2205	Male	Three y/o	Treatment (Wk 1)	Treatment	Feed Day	No	15:30	15:00 - 15:59	Afternoon	38,0	56	19,4	19,4	19,15	549,01	99,28
2019/05/17	CH2206	Male	Three y/o	Treatment (Wk 1)	Treatment	Feed Day	No	15:30	15:00 - 15:59	Afternoon	37,9	80	5,2	5,2	19,05	577,31	54,72
2019/05/17	CH2205	Male	Three y/o	Treatment (Wk 1)	Treatment	Feed Day	No	15:35	15:00 - 15:59	Afternoon	38,0	54	25,0	25,0	19,15	545,99	107,94
2019/05/17	CH2206	Male	Three y/o	Treatment (Wk 1)	Treatment	Feed Day	No	15:35	15:00 - 15:59	Afternoon	37,9	77	3,6	3,6	19,05	574,39	42,42
2019/05/17	CH2205	Male	Three y/o	Treatment (Wk 1)	Treatment	Feed Day	No	15:40	15:00 - 15:59	Afternoon	38,0	86	171,6	171,6	19,15	582,77	174,74
2019/05/17	CH2206	Male	Three y/o	Treatment (Wk 1)	Treatment	Feed Day	No	15:40	15:00 - 15:59	Afternoon	37,9	108	55,0	55,0	19,05	599,39	135,08
2019/05/17	CH2205	Male	Three y/o	Treatment (Wk 1)	Treatment	Feed Day	No	15:45	15:00 - 15:59	Afternoon	37,9	64	29,0	29,0	19,05	559,88	113,03
2019/05/17	CH2206	Male	Three y/o	Treatment (Wk 1)	Treatment	Feed Day	No	15:45	15:00 - 15:59	Afternoon	37,9	82	39,0	39,0	19,05	579,19	123,22
2019/05/17	CH2205	Male	Three y/o	Treatment (Wk 1)	Treatment	Feed Day	No	15:50	15:00 - 15:59	Afternoon	37,9	118	21,4	21,4	19,05	605,61	102,63
2019/05/17	CH2206	Male	Three y/o	Treatment (Wk 1)	Treatment	Feed Day	No	15:50	15:00 - 15:59	Afternoon	37,9	82	36,8	36,8	19,05	579,19	121,22
2019/05/17	CH2205	Male	Three y/o	Treatment (Wk 1)	Treatment	Feed Day	No	15:55	15:00 - 15:59	Afternoon	37,9	73	19,6	19,6	19,05	570,27	99,63
2019/05/17	CH2206	Male	Three y/o	Treatment (Wk 1)	Treatment	Feed Day	No	15:55	15:00 - 15:59	Afternoon	37,9		39,6	39,6	19,05		123,74
2019/05/17	CH2205	Male	Three y/o	Treatment (Wk 1)	Treatment	Feed Day	No	16:00	16:00 - 16:59	Evening	37,9	62	16,6	16,6	19,05	557,33	93,96

2019/05/17	CH2206	Male	Three y/o	Treatment (Wk 1)	Treatment	Feed Day	No	16:00	16:00 - 16:59	Evening	37,9	82	41,6	41,6	19,05	579,19	125,44
2019/05/17	CH2205	Male	Three y/o	Treatment (Wk 1)	Treatment	Feed Day	No	16:05	16:00 - 16:59	Evening	38,0	64	4,6	4,6	19,15	559,88	50,61
2019/05/17	CH2206	Male	Three y/o	Treatment (Wk 1)	Treatment	Feed Day	No	16:05	16:00 - 16:59	Evening	37,9	90	40,2	40,2	19,05	586,16	124,26
2019/05/17	CH2205	Male	Three y/o	Treatment (Wk 1)	Treatment	Feed Day	No	16:10	16:00 - 16:59	Evening	38,0	74	6,0	6,0	19,15	571,33	59,53
2019/05/17	CH2206	Male	Three y/o	Treatment (Wk 1)	Treatment	Feed Day	No	16:10	16:00 - 16:59	Evening	37,9	68	45,2	45,2	19,05	564,71	128,30
2019/05/17	CH2205	Male	Three y/o	Treatment (Wk 1)	Treatment	Feed Day	No	16:15	16:00 - 16:59	Evening	38,0	75	7,4	7,4	19,15	572,36	66,59
2019/05/17	CH2206	Male	Three y/o	Treatment (Wk 1)	Treatment	Feed Day	No	16:15	16:00 - 16:59	Evening	38,0	86	42,4	42,4	19,15	582,77	126,10
2019/05/17	CH2205	Male	Three y/o	Treatment (Wk 1)	Treatment	Feed Day	No	16:20	16:00 - 16:59	Evening	38,1	55	6,6	6,6	19,26	547,52	62,73
2019/05/17	CH2206	Male	Three y/o	Treatment (Wk 1)	Treatment	Feed Day	No	16:20	16:00 - 16:59	Evening	38,0	94	45,4	45,4	19,15	589,37	128,45
2019/05/17	CH2205	Male	Three y/o	Treatment (Wk 1)	Treatment	Feed Day	No	16:25	16:00 - 16:59	Evening	38,1		20,2	20,2	19,26		100,65
2019/05/17	CH2206	Male	Three y/o	Treatment (Wk 1)	Treatment	Feed Day	No	16:25	16:00 - 16:59	Evening	38,0	70	42,2	42,2	19,15	566,99	125,93
2019/05/17	CH2205	Male	Three y/o	Treatment (Wk 1)	Treatment	Feed Day	No	16:30	16:00 - 16:59	Evening	38,1	78	22,4	22,4	19,26	575,38	104,19
2019/05/17	CH2206	Male	Three y/o	Treatment (Wk 1)	Treatment	Feed Day	No	16:30	16:00 - 16:59	Evening	38,0	86	44,0	44,0	19,15	582,77	127,37
2019/05/17	CH2205	Male	Three y/o	Treatment (Wk 1)	Treatment	Feed Day	No	16:35	16:00 - 16:59	Evening	38,1	63	27,6	27,6	19,26	558,62	111,33
2019/05/17	CH2206	Male	Three y/o	Treatment (Wk 1)	Treatment	Feed Day	No	16:35	16:00 - 16:59	Evening	38,0	74	49,2	49,2	19,15	571,33	131,23
2019/05/17	CH2205	Male	Three y/o	Treatment (Wk 1)	Treatment	Feed Day	No	16:40	16:00 - 16:59	Evening	38,2	72	23,6	23,6	19,36	569,20	105,97
2019/05/17	CH2206	Male	Three y/o	Treatment (Wk 1)	Treatment	Feed Day	No	16:40	16:00 - 16:59	Evening	38,0	83	47,0	47,0	19,15	580,11	129,65
2019/05/17	CH2205	Male	Three y/o	Treatment (Wk 1)	Treatment	Feed Day	No	16:45	16:00 - 16:59	Evening	38,2	70	27,4	27,4	19,36	566,99	111,09
2019/05/17	CH2206	Male	Three y/o	Treatment (Wk 1)	Treatment	Feed Day	No	16:45	16:00 - 16:59	Evening	38,0	86	42,4	42,4	19,15	582,77	126,10
2019/05/17	CH2205	Male	Three y/o	Treatment (Wk 1)	Treatment	Feed Day	No	16:50	16:00 - 16:59	Evening	38,2	58	21,0	21,0	19,36	551,90	101,98
2019/05/17	CH2206	Male	Three y/o	Treatment (Wk 1)	Treatment	Feed Day	No	16:50	16:00 - 16:59	Evening	38,0	94	14,6	14,6	19,15	589,37	89,59
2019/05/17	CH2205	Male	Three y/o	Treatment (Wk 1)	Treatment	Feed Day	No	16:55	16:00 - 16:59	Evening	38,2	61	27,4	27,4	19,36	556,01	111,09
2019/05/17	CH2206	Male	Three y/o	Treatment (Wk 1)	Treatment	Feed Day	No	16:55	16:00 - 16:59	Evening	38,0	86	8,0	8,0	19,15	582,77	69,22
2019/05/17	CH2205	Male	Three y/o	Treatment (Wk 1)	Treatment	Feed Day	No	17:00	17:00 - 17:59	Evening	38,2	94	25,4	25,4	19,36	589,37	108,49
2019/05/17	CH2206	Male	Three y/o	Treatment (Wk 1)	Treatment	Feed Day	No	17:00	17:00 - 17:59	Evening	38,0	76	8,6	8,6	19,15	573,39	71,66
2019/05/17	CH2205	Male	Three y/o	Treatment (Wk 1)	Treatment	Feed Day	No	17:05	17:00 - 17:59	Evening	38,2	79	15,0	15,0	19,36	576,36	90,51
2019/05/17	CH2206	Male	Three y/o	Treatment (Wk 1)	Treatment	Feed Day	No	17:05	17:00 - 17:59	Evening	38,0	86	6,6	6,6	19,15	582,77	62,73
2019/05/17	CH2205	Male	Three y/o	Treatment (Wk 1)	Treatment	Feed Day	No	17:10	17:00 - 17:59	Evening	38,2	59	13,4	13,4	19,36	553,30	86,68
2019/05/17	CH2206	Male	Three y/o	Treatment (Wk 1)	Treatment	Feed Day	No	17:10	17:00 - 17:59	Evening	38,0	88	15,0	15,0	19,15	584,49	90,51
2019/05/17	CH2205	Male	Three y/o	Treatment (Wk 1)	Treatment	Feed Day	No	17:15	17:00 - 17:59	Evening	38,2	73	28,4	28,4	19,36	570,27	112,31
2019/05/17	CH2206	Male	Three y/o	Treatment (Wk 1)	Treatment	Feed Day	No	17:15	17:00 - 17:59	Evening	38,0	102	15,6	15,6	19,15	595,30	91,85
2019/05/17	CH2205	Male	Three y/o	Treatment (Wk 1)	Treatment	Feed Day	No	17:20	17:00 - 17:59	Evening	38,2	64	26,0	26,0	19,36	559,88	109,29
2019/05/17	CH2206	Male	Three y/o	Treatment (Wk 1)	Treatment	Feed Day	No	17:20	17:00 - 17:59	Evening	38,0	83	27,8	27,8	19,15	580,11	111,58
2019/05/17	CH2205	Male	Three y/o	Treatment (Wk 1)	Treatment	Feed Day	No	17:25	17:00 - 17:59	Evening	38,2	81	24,6	24,6	19,36	578,26	107,39
2019/05/17	CH2206	Male	Three y/o	Treatment (Wk 1)	Treatment	Feed Day	No	17:25	17:00 - 17:59	Evening	38,0	80	9,8	9,8	19,15	577,31	76,07
2019/05/17	CH2205	Male	Three y/o	Treatment (Wk 1)	Treatment	Feed Day	No	17:30	17:00 - 17:59	Evening	38,1	60	6,6	6,6	19,26	554,67	62,73
2019/05/17	CH2206	Male	Three y/o	Treatment (Wk 1)	Treatment	Feed Day	No	17:30	17:00 - 17:59	Evening	38,0	49	7,8	7,8	19,15	537,80	68,36
2019/05/17	CH2205	Male	Three y/o	Treatment (Wk 1)	Treatment	Feed Day	No	17:35	17:00 - 17:59	Evening	38,0	59	6,4	6,4	19,15	553,30	61,70
2019/05/17	CH2206	Male	Three y/o	Treatment (Wk 1)	Treatment	Feed Day	No	17:35	17:00 - 17:59	Evening	38,0	88	13,8	13,8	19,15	584,49	87,68
2019/05/17	CH2205	Male	Three y/o	Treatment (Wk 1)	Treatment	Feed Day	No	17:40	17:00 - 17:59	Evening	38,0	55	3,8	3,8	19,15	547,52	44,22
2019/05/17	CH2206	Male	Three y/o	Treatment (Wk 1)	Treatment	Feed Day	No	17:40	17:00 - 17:59	Evening	38,0	89	20,8	20,8	19,15	585,33	101,65
2019/05/17	CH2205	Male	Three y/o	Treatment (Wk 1)	Treatment	Feed Day	No	17:45	17:00 - 17:59	Evening	38,0	48	4,2	4,2	19,15	536,04	47,57
2019/05/17	CH2206	Male	Three y/o	Treatment (Wk 1)	Treatment	Feed Day	No	17:45	17:00 - 17:59	Evening	38,0	69	13,6	13,6	19,15	565,86	87,18
2019/05/17	CH2205	Male	Three y/o	Treatment (Wk 1)	Treatment	Feed Day	No	17:50	17:00 - 17:59	Evening	38,0	86	14,0	14,0	19,15	582,77	88,17
2019/05/17	CH2206	Male	Three y/o	Treatment (Wk 1)	Treatment	Feed Day	No	17:50	17:00 - 17:59	Evening	38,0	78	22,8	22,8	19,15	575,38	104,79
2019/05/17	CH2205	Male	Three y/o	Treatment (Wk 1)	Treatment	Feed Day	No	17:55	17:00 - 17:59	Evening	38,1	94	143,4	143,4	19,26	589,37	168,44
2019/05/17	CH2206	Male	Three y/o	Treatment (Wk 1)	Treatment	Feed Day	No	17:55	17:00 - 17:59	Evening	38,0		43,8	43,8	19,15		127,22
2019/05/17	CH2205	Male	Three y/o	Treatment (Wk 1)	Treatment	Feed Day	No	18:00	18:00 - 18:59	Evening	37,9		8,6	8,6	19,05		71,66
2019/05/17	CH2206	Male	Three y/o	Treatment (Wk 1)	Treatment	Feed Day	No	18:00	18:00 - 18:59	Evening	38,0	108	71,4	71,4	19,15	599,39	144,12
2019/05/17	CH2205	Male	Three y/o	Treatment (Wk 1)	Treatment	Feed Day	No	18:05	18:00 - 18:59	Evening	37,8		65,4	65,4	18,95		141,08
2019/05/17	CH2206	Male	Three y/o	Treatment (Wk 1)	Treatment	Feed Day	No	18:05	18:00 - 18:59	Evening	37,9	138	48,6	48,6	19,05	616,31	130,81
2019/05/17	CH2205	Male	Three y/o	Treatment (Wk 1)	Treatment	Feed Day	No	18:10	18:00 - 18:59	Evening	37,7		39,8	39,8	18,85		123,91
2019/05/17	CH2206	Male	Three y/o	Treatment (Wk 1)	Treatment	Feed Day	No	18:10	18:00 - 18:59	Evening	37,8	131	58,6	58,6	18,95	612,80	137,28
2019/05/17	CH2205	Male	Three y/o	Treatment (Wk 1)	Treatment	Feed Day	No	18:15	18:00 - 18:59	Evening	37,7	108	18,6	18,6	18,85	599,39	97,84
2019/05/17	CH2206	Male	Three y/o	Treatment (Wk 1)	Treatment	Feed Day	No	18:15	18:00 - 18:59	Evening	37,9		106,2	106,2	19,05		157,94
2019/05/17	CH2205	Male	Three y/o	Treatment (Wk 1)	Treatment	Feed Day	No	18:20	18:00 - 18:59	Evening	37,7	138	24,2	24,2	18,85	616,31	106,83
2019/05/17	CH2206	Male	Three y/o	Treatment (Wk 1)	Treatment	Feed Day	No	18:20	18:00 - 18:59	Evening	37,9	110	42,8	42,8	19,05	600,69	126,42
2019/05/17	CH2205	Male	Three y/o	Treatment (Wk 1)	Treatment	Feed Day	No	18:25	18:00 - 18:59	Evening	37,7	174	34,8	34,8	18,85	631,46	119,29
2019/05/17	CH2206	Male	Three y/o	Treatment (Wk 1)	Treatment	Feed Day	No	18:25	18:00 - 18:59	Evening	37,8	103	51,4	51,4	18,95	596,00	132,74
2019/05/17	CH2205	Male	Three y/o	Treatment (Wk 1)	Treatment	Feed Day	No	18:30	18:00 - 18:59	Evening	37,6		21,2	21,2	18,75		102,31
2019/05/17	CH2206	Male	Three y/o	Treatment (Wk 1)	Treatment	Feed Day	No	18:30	18:00 - 18:59	Evening	37,8	75	55,6	55,6	18,95	572,36	135,46
2019/05/17	CH2205	Male	Three y/o	Treatment (Wk 1)	Treatment	Feed Day	No	18:35	18:00 - 18:59	Evening	37,7	76	14,2	14,2	18,85	573,39	88,65
2019/05/17	CH2206	Male	Three y/o	Treatment (Wk 1)	Treatment	Feed Day	No	18:35	18:00 - 18:59	Evening	37,8	87	59,4	59,4	18,95	583,64	137,75
2019/05/17	CH2205	Male	Three y/o	Treatment (Wk 1)	Treatment	Feed Day	No	18:40	18:00 - 18:59	Evening	37,9	64	14,0	14,0	19,05	559,88	88,17
2019/05/17	CH2206	Male	Three y/o	Treatment (Wk 1)	Treatment	Feed Day	No	18:40	18:00 - 18:59	Evening	37,8	82	51,4	51,4	18,95	579,19	132,74
2019/05/17	CH2205	Male	Three y/o	Treatment (Wk 1)	Treatment	Feed Day	No	18:45	18:00 - 18:59	Evening	38,0	82	13,4	13,4	19,15	579,19	86,68
2019/05/17	CH2206	Male	Three y/o	Treatment (Wk 1)	Treatment	Feed Day	No	18:45	18:00 - 18:59	Evening	37,8	82	53,4	53,4	18,95	579,19	134,06
2019/05/17	CH2205	Male	Three y/o	Treatment (Wk 1)	Treatment	Feed Day	No	18:50	18:00 - 18:59	Evening	38,0	84	13,2	13,2	19,15	581,01	86,17
2019/05/17	CH2206	Male	Three y/o	Treatment (Wk 1)	Treatment	Feed Day	No	18:50	18:00 - 18:59	Evening	37,8	81	52,8	52,8	18,95	578,26	133,67
2019/05/17	CH2205	Male	Three y/o	Treatment (Wk 1)	Treatment	Feed Day	No	18:55	18:00 - 18:59	Evening	38,1	82	12,8	12,8	19,26	579,19	85,12

2019/05/17	CH2206	Male	Three y/o	Treatment (Wk 1)	Treatment	Feed Day	No	18:55	18:00 - 18:59	Evening	37,9	89	61,4	61,4	19,05	585,33	138,89
2019/05/17	CH2205	Male	Three y/o	Treatment (Wk 1)	Treatment	Feed Day	No	19:00	19:00 - 19:59	Evening	38,1	87	8,6	8,6	19,26	583,64	71,66
2019/05/17	CH2206	Male	Three y/o	Treatment (Wk 1)	Treatment	Feed Day	No	19:00	19:00 - 19:59	Evening	37,9	90	58,6	58,6	19,05	586,16	137,28
2019/05/17	CH2205	Male	Three y/o	Treatment (Wk 1)	Treatment	Feed Day	No	19:05	19:00 - 19:59	Evening	38,1	84	12,4	12,4	19,26	581,01	84,04
2019/05/17	CH2206	Male	Three y/o	Treatment (Wk 1)	Treatment	Feed Day	No	19:05	19:00 - 19:59	Evening	37,9	80	52,4	52,4	19,05	577,31	133,41
2019/05/17	CH2205	Male	Three y/o	Treatment (Wk 1)	Treatment	Feed Day	No	19:10	19:00 - 19:59	Evening	38,2	51	10,2	10,2	19,36	541,20	77,43
2019/05/17	CH2206	Male	Three y/o	Treatment (Wk 1)	Treatment	Feed Day	No	19:10	19:00 - 19:59	Evening	37,9	89	52,8	52,8	19,05	585,33	133,67
2019/05/17	CH2205	Male	Three y/o	Treatment (Wk 1)	Treatment	Feed Day	No	19:15	19:00 - 19:59	Evening	38,2	80	12,8	12,8	19,36	577,31	85,12
2019/05/17	CH2206	Male	Three y/o	Treatment (Wk 1)	Treatment	Feed Day	No	19:15	19:00 - 19:59	Evening	37,9	77	50,2	50,2	19,05	574,39	131,92
2019/05/17	CH2205	Male	Three y/o	Treatment (Wk 1)	Treatment	Feed Day	No	19:20	19:00 - 19:59	Evening	38,2	69	11,4	11,4	19,36	565,86	81,19
2019/05/17	CH2206	Male	Three y/o	Treatment (Wk 1)	Treatment	Feed Day	No	19:20	19:00 - 19:59	Evening	37,9	89	54,4	54,4	19,05	585,33	134,70
2019/05/17	CH2205	Male	Three y/o	Treatment (Wk 1)	Treatment	Feed Day	No	19:25	19:00 - 19:59	Evening	38,2	67	13,8	13,8	19,36	563,53	87,68
2019/05/17	CH2206	Male	Three y/o	Treatment (Wk 1)	Treatment	Feed Day	No	19:25	19:00 - 19:59	Evening	37,9	84	51,4	51,4	19,05	581,01	132,74
2019/05/17	CH2205	Male	Three y/o	Treatment (Wk 1)	Treatment	Feed Day	No	19:30	19:00 - 19:59	Evening	38,2	50	8,4	8,4	19,36	539,52	70,86
2019/05/17	CH2206	Male	Three y/o	Treatment (Wk 1)	Treatment	Feed Day	No	19:30	19:00 - 19:59	Evening	37,9	66	57,8	57,8	19,05	562,34	136,80
2019/05/17	CH2205	Male	Three y/o	Treatment (Wk 1)	Treatment	Feed Day	No	19:35	19:00 - 19:59	Evening	38,1	53	5,4	5,4	19,26	544,43	55,99
2019/05/17	CH2206	Male	Three y/o	Treatment (Wk 1)	Treatment	Feed Day	No	19:35	19:00 - 19:59	Evening	38,0	109	57,2	57,2	19,15	600,04	136,44
2019/05/17	CH2205	Male	Three y/o	Treatment (Wk 1)	Treatment	Feed Day	No	19:40	19:00 - 19:59	Evening	38,1	92	6,6	6,6	19,26	587,78	62,73
2019/05/17	CH2206	Male	Three y/o	Treatment (Wk 1)	Treatment	Feed Day	No	19:40	19:00 - 19:59	Evening	38,0	63	51,6	51,6	19,15	558,62	132,87
2019/05/17	CH2205	Male	Three y/o	Treatment (Wk 1)	Treatment	Feed Day	No	19:45	19:00 - 19:59	Evening	38,1	68	3,2	3,2	19,26	564,71	38,49
2019/05/17	CH2206	Male	Three y/o	Treatment (Wk 1)	Treatment	Feed Day	No	19:45	19:00 - 19:59	Evening	38,0	56	46,4	46,4	19,15	549,01	129,21
2019/05/17	CH2205	Male	Three y/o	Treatment (Wk 1)	Treatment	Feed Day	No	19:50	19:00 - 19:59	Evening	38,1	94	9,8	9,8	19,26	589,37	76,07
2019/05/17	CH2206	Male	Three y/o	Treatment (Wk 1)	Treatment	Feed Day	No	19:50	19:00 - 19:59	Evening	38,0	64	45,8	45,8	19,15	559,88	128,76
2019/05/17	CH2205	Male	Three y/o	Treatment (Wk 1)	Treatment	Feed Day	No	19:55	19:00 - 19:59	Evening	38,1	102	10,2	10,2	19,26	595,30	77,43
2019/05/17	CH2206	Male	Three y/o	Treatment (Wk 1)	Treatment	Feed Day	No	19:55	19:00 - 19:59	Evening	38,0	75	51,8	51,8	19,15	572,36	133,01
2019/05/17	CH2205	Male	Three y/o	Treatment (Wk 1)	Treatment	Feed Day	No	20:00	20:00 - 20:59	Night	38,1	97	8,8	8,8	19,26	591,66	72,43
2019/05/17	CH2206	Male	Three y/o	Treatment (Wk 1)	Treatment	Feed Day	No	20:00	20:00 - 20:59	Night	38,0	59	53,0	53,0	19,15	553,30	133,80
2019/05/17	CH2205	Male	Three y/o	Treatment (Wk 1)	Treatment	Feed Day	No	20:05	20:00 - 20:59	Night	38,1	89	11,4	11,4	19,26	585,33	81,19
2019/05/17	CH2206	Male	Three y/o	Treatment (Wk 1)	Treatment	Feed Day	No	20:05	20:00 - 20:59	Night	38,0	47	48,4	48,4	19,15	534,23	130,66
2019/05/17	CH2205	Male	Three y/o	Treatment (Wk 1)	Treatment	Feed Day	No	20:10	20:00 - 20:59	Night	38,1	103	5,0	5,0	19,26	596,00	53,41
2019/05/17	CH2206	Male	Three y/o	Treatment (Wk 1)	Treatment	Feed Day	No	20:10	20:00 - 20:59	Night	38,0	79	47,6	47,6	19,15	576,36	130,09
2019/05/17	CH2205	Male	Three y/o	Treatment (Wk 1)	Treatment	Feed Day	No	20:15	20:00 - 20:59	Night	38,1	51	17,8	17,8	19,26	541,20	96,34
2019/05/17	CH2206	Male	Three y/o	Treatment (Wk 1)	Treatment	Feed Day	No	20:15	20:00 - 20:59	Night	38,0	91	15,4	15,4	19,15	586,98	91,41
2019/05/17	CH2205	Male	Three y/o	Treatment (Wk 1)	Treatment	Feed Day	No	20:20	20:00 - 20:59	Night	38,1	73	12,4	12,4	19,26	570,27	84,04
2019/05/17	CH2206	Male	Three y/o	Treatment (Wk 1)	Treatment	Feed Day	No	20:20	20:00 - 20:59	Night	38,0	50	10,2	10,2	19,15	539,52	77,43
2019/05/17	CH2205	Male	Three y/o	Treatment (Wk 1)	Treatment	Feed Day	No	20:25	20:00 - 20:59	Night	38,1		27,2	27,2	19,26		110,83
2019/05/17	CH2206	Male	Three y/o	Treatment (Wk 1)	Treatment	Feed Day	No	20:25	20:00 - 20:59	Night	38,0	86	3,6	3,6	19,15	582,77	42,42
2019/05/17	CH2205	Male	Three y/o	Treatment (Wk 1)	Treatment	Feed Day	No	20:30	20:00 - 20:59	Night	38,1		33,0	33,0	19,26		117,47
2019/05/17	CH2206	Male	Three y/o	Treatment (Wk 1)	Treatment	Feed Day	No	20:30	20:00 - 20:59	Night	37,9	84	6,2	6,2	19,05	581,01	60,63
2019/05/17	CH2205	Male	Three y/o	Treatment (Wk 1)	Treatment	Feed Day	No	20:35	20:00 - 20:59	Night	38,0		25,8	25,8	19,15		109,02
2019/05/17	CH2206	Male	Three y/o	Treatment (Wk 1)	Treatment	Feed Day	No	20:35	20:00 - 20:59	Night	37,9	85	6,0	6,0	19,05	581,90	59,53
2019/05/17	CH2205	Male	Three y/o	Treatment (Wk 1)	Treatment	Feed Day	No	20:40	20:00 - 20:59	Night	38,0	71	10,0	10,0	19,15	568,10	76,76
2019/05/17	CH2206	Male	Three y/o	Treatment (Wk 1)	Treatment	Feed Day	No	20:40	20:00 - 20:59	Night	37,9	46	4,0	4,0	19,05	532,38	45,94
2019/05/17	CH2205	Male	Three y/o	Treatment (Wk 1)	Treatment	Feed Day	No	20:45	20:00 - 20:59	Night	38,0	68	9,0	9,0	19,15	564,71	73,19
2019/05/17	CH2206	Male	Three y/o	Treatment (Wk 1)	Treatment	Feed Day	No	20:45	20:00 - 20:59	Night	37,9	86	13,2	13,2	19,05	582,77	86,17
2019/05/17	CH2205	Male	Three y/o	Treatment (Wk 1)	Treatment	Feed Day	No	20:50	20:00 - 20:59	Night	38,0	88	6,4	6,4	19,15	584,49	61,70
2019/05/17	CH2206	Male	Three y/o	Treatment (Wk 1)	Treatment	Feed Day	No	20:50	20:00 - 20:59	Night	37,9	82	16,2	16,2	19,05	579,19	93,13
2019/05/17	CH2205	Male	Three y/o	Treatment (Wk 1)	Treatment	Feed Day	No	20:55	20:00 - 20:59	Night	38,1	93	12,4	12,4	19,26	588,58	84,04
2019/05/17	CH2206	Male	Three y/o	Treatment (Wk 1)	Treatment	Feed Day	No	20:55	20:00 - 20:59	Night	37,9	48	21,8	21,8	19,05	536,04	103,26
2019/05/17	CH2205	Male	Three y/o	Treatment (Wk 1)	Treatment	Feed Day	No	21:00	21:00 - 21:59	Night	38,1	62	10,4	10,4	19,26	557,33	78,08
2019/05/17	CH2206	Male	Three y/o	Treatment (Wk 1)	Treatment	Feed Day	No	21:00	21:00 - 21:59	Night	37,9		34,8	34,8	19,05		119,29
2019/05/17	CH2205	Male	Three y/o	Treatment (Wk 1)	Treatment	Feed Day	No	21:05	21:00 - 21:59	Night	38,1	56	5,6	5,6	19,26	549,01	57,21
2019/05/17	CH2206	Male	Three y/o	Treatment (Wk 1)	Treatment	Feed Day	No	21:05	21:00 - 21:59	Night	37,8	91	33,8	33,8	18,95	586,98	118,29
2019/05/17	CH2205	Male	Three y/o	Treatment (Wk 1)	Treatment	Feed Day	No	21:10	21:00 - 21:59	Night	38,1	65	9,8	9,8	19,26	561,12	76,07
2019/05/17	CH2206	Male	Three y/o	Treatment (Wk 1)	Treatment	Feed Day	No	21:10	21:00 - 21:59	Night	37,7	96	63,4	63,4	18,85	590,91	140,00
2019/05/17	CH2205	Male	Three y/o	Treatment (Wk 1)	Treatment	Feed Day	No	21:15	21:00 - 21:59	Night	38,1	58	12,0	12,0	19,26	551,90	82,93
2019/05/17	CH2206	Male	Three y/o	Treatment (Wk 1)	Treatment	Feed Day	No	21:15	21:00 - 21:59	Night	37,7	87	64,4	64,4	18,85	583,64	140,55
2019/05/17	CH2205	Male	Three y/o	Treatment (Wk 1)	Treatment	Feed Day	No	21:20	21:00 - 21:59	Night	38,1	95	12,6	12,6	19,26	590,14	84,59
2019/05/17	CH2206	Male	Three y/o	Treatment (Wk 1)	Treatment	Feed Day	No	21:20	21:00 - 21:59	Night	37,7	58	64,2	64,2	18,85	551,90	140,44
2019/05/17	CH2205	Male	Three y/o	Treatment (Wk 1)	Treatment	Feed Day	No	21:25	21:00 - 21:59	Night	38,1	60	12,6	12,6	19,26	554,67	84,59
2019/05/17	CH2206	Male	Three y/o	Treatment (Wk 1)	Treatment	Feed Day	No	21:25	21:00 - 21:59	Night	37,8	73	63,2	63,2	18,95	570,27	139,89
2019/05/17	CH2205	Male	Three y/o	Treatment (Wk 1)	Treatment	Feed Day	No	21:30	21:00 - 21:59	Night	38,0	46	11,0	11,0	19,15	532,38	79,98
2019/05/17	CH2206	Male	Three y/o	Treatment (Wk 1)	Treatment	Feed Day	No	21:30	21:00 - 21:59	Night	37,8	64	61,6	61,6	18,95	559,88	139,00
2019/05/17	CH2205	Male	Three y/o	Treatment (Wk 1)	Treatment	Feed Day	No	21:35	21:00 - 21:59	Night	38,0	79	11,2	11,2	19,15	576,36	80,59
2019/05/17	CH2206	Male	Three y/o	Treatment (Wk 1)	Treatment	Feed Day	No	21:35	21:00 - 21:59	Night	37,9	80	67,2	67,2	19,05	577,31	142,02
2019/05/17	CH2205	Male	Three y/o	Treatment (Wk 1)	Treatment	Feed Day	No	21:40	21:00 - 21:59	Night	38,0	90	15,0	15,0	19,15	586,16	90,51
2019/05/17	CH2206	Male	Three y/o	Treatment (Wk 1)	Treatment	Feed Day	No	21:40	21:00 - 21:59	Night	37,9	66	60,4	60,4	19,05	562,34	138,32
2019/05/17	CH2205	Male	Three y/o	Treatment (Wk 1)	Treatment	Feed Day	No	21:45	21:00 - 21:59	Night	38,0	57	14,8	14,8	19,15	550,47	90,05
2019/05/17	CH2206	Male	Three y/o	Treatment (Wk 1)	Treatment	Feed Day	No	21:45	21:00 - 21:59	Night	37,9	70	65,4	65,4	19,05	566,99	141,08
2019/05/17	CH2205	Male	Three y/o	Treatment (Wk 1)	Treatment	Feed Day	No	21:50	21:00 - 21:59	Night	38,0	89	19,2	19,2	19,15	585,33	98,92

	2019/05/17	CH2206	Male	Three y/o	Treatment (Wk 1)	Treatment	Feed Day	No	21:50	21:00 - 21:59	Night	37,9	83	59,8	59,8	19,05	580,11	137,98
	2019/05/17	CH2205	Male	Three y/o	Treatment (Wk 1)	Treatment	Feed Day	No	21:55	21:00 - 21:59	Night	38,0	72	17,8	17,8	19,15	569,20	96,34
	2019/05/17	CH2206	Male	Three y/o	Treatment (Wk 1)	Treatment	Feed Day	No	21:55	21:00 - 21:59	Night	37,9	84	60,6	60,6	19,05	581,01	138,44
	2019/05/17	CH2205	Male	Three y/o	Treatment (Wk 1)	Treatment	Feed Day	No	22:00	22:00 - 22:59	Night	38,0	95	19,4	19,4	19,15	590,14	99,28
	2019/05/17	CH2206	Male	Three y/o	Treatment (Wk 1)	Treatment	Feed Day	No	22:00	22:00 - 22:59	Night	37,9	54	60,8	60,8	19,05	545,99	138,55
	2019/05/17	CH2205	Male	Three y/o	Treatment (Wk 1)	Treatment	Feed Day	No	22:05	22:00 - 22:59	Night	38,0	73	21,0	21,0	19,15	570,27	101,98
	2019/05/17	CH2206	Male	Three y/o	Treatment (Wk 1)	Treatment	Feed Day	No	22:05	22:00 - 22:59	Night	37,9	65	58,6	58,6	19,05	561,12	137,28
	2019/05/17	CH2205	Male	Three y/o	Treatment (Wk 1)	Treatment	Feed Day	No	22:10	22:00 - 22:59	Night	38,0	74	17,6	17,6	19,15	571,33	95,95
	2019/05/17	CH2206	Male	Three y/o	Treatment (Wk 1)	Treatment	Feed Day	No	22:10	22:00 - 22:59	Night	38,0	81	61,2	61,2	19,15	578,26	138,78
	2019/05/17	CH2205	Male	Three y/o	Treatment (Wk 1)	Treatment	Feed Day	No	22:15	22:00 - 22:59	Night	38,0	68	19,2	19,2	19,15	564,71	98,92
	2019/05/17	CH2206	Male	Three y/o	Treatment (Wk 1)	Treatment	Feed Day	No	22:15	22:00 - 22:59	Night	38,0	79	62,0	62,0	19,15	576,36	139,23
	2019/05/17	CH2205	Male	Three y/o	Treatment (Wk 1)	Treatment	Feed Day	No	22:20	22:00 - 22:59	Night	38,0	72	24,4	24,4	19,15	569,20	107,11
	2019/05/17	CH2206	Male	Three y/o	Treatment (Wk 1)	Treatment	Feed Day	No	22:20	22:00 - 22:59	Night	38,0	80	61,4	61,4	19,15	577,31	138,89
	2019/05/17	CH2205	Male	Three y/o	Treatment (Wk 1)	Treatment	Feed Day	No	22:25	22:00 - 22:59	Night	37,9	59	17,6	17,6	19,05	553,30	95,95
	2019/05/17	CH2206	Male	Three y/o	Treatment (Wk 1)	Treatment	Feed Day	No	22:25	22:00 - 22:59	Night	38,0	84	62,2	62,2	19,15	581,01	139,34
	2019/05/17	CH2205	Male	Three y/o	Treatment (Wk 1)	Treatment	Feed Day	No	22:30	22:00 - 22:59	Night	37,9	54	21,0	21,0	19,05	545,99	101,98
	2019/05/17	CH2206	Male	Three y/o	Treatment (Wk 1)	Treatment	Feed Day	No	22:30	22:00 - 22:59	Night	38,0	80	55,2	55,2	19,15	577,31	135,21
	2019/05/17	CH2205	Male	Three y/o	Treatment (Wk 1)	Treatment	Feed Day	No	22:35	22:00 - 22:59	Night	37,9	88	17,8	17,8	19,05	584,49	96,34
	2019/05/17	CH2206	Male	Three y/o	Treatment (Wk 1)	Treatment	Feed Day	No	22:35	22:00 - 22:59	Night	38,0	83	63,0	63,0	19,15	580,11	139,78
	2019/05/17	CH2205	Male	Three y/o	Treatment (Wk 1)	Treatment	Feed Day	No	22:40	22:00 - 22:59	Night	37,9	37	20,0	20,0	19,05	513,06	100,31
	2019/05/17	CH2206	Male	Three y/o	Treatment (Wk 1)	Treatment	Feed Day	No	22:40	22:00 - 22:59	Night	38,0	65	61,6	61,6	19,15	561,12	139,00
	2019/05/17	CH2205	Male	Three y/o	Treatment (Wk 1)	Treatment	Feed Day	No	22:45	22:00 - 22:59	Night	37,9	93	27,4	27,4	19,05	588,58	111,09
	2019/05/17	CH2206	Male	Three y/o	Treatment (Wk 1)	Treatment	Feed Day	No	22:45	22:00 - 22:59	Night	38,0	58	57,6	57,6	19,15	551,90	136,68
	2019/05/17	CH2205	Male	Three y/o	Treatment (Wk 1)	Treatment	Feed Day	No	22:50	22:00 - 22:59	Night	37,9	50	28,6	28,6	19,05	539,52	112,56
	2019/05/17	CH2206	Male	Three y/o	Treatment (Wk 1)	Treatment	Feed Day	No	22:50	22:00 - 22:59	Night	38,0	102	57,0	57,0	19,15	595,30	136,32
	2019/05/17	CH2205	Male	Three y/o	Treatment (Wk 1)	Treatment	Feed Day	No	22:55	22:00 - 22:59	Night	37,9	72	30,6	30,6	19,05	569,20	114,87
	2019/05/17	CH2206	Male	Three y/o	Treatment (Wk 1)	Treatment	Feed Day	No	22:55	22:00 - 22:59	Night	38,0	83	55,2	55,2	19,15	580,11	135,21
	2019/05/17	CH2205	Male	Three y/o	Treatment (Wk 1)	Treatment	Feed Day	No	23:00	23:00 - 23:59	Night	37,9	68	27,6	27,6	19,05	564,71	111,33
	2019/05/17	CH2206	Male	Three y/o	Treatment (Wk 1)	Treatment	Feed Day	No	23:00	23:00 - 23:59	Night	38,0	94	60,8	60,8	19,15	589,37	138,55
	2019/05/17	CH2205	Male	Three y/o	Treatment (Wk 1)	Treatment	Feed Day	No	23:05	23:00 - 23:59	Night	37,9	89	21,6	21,6	19,05	585,33	102,94
	2019/05/17	CH2206	Male	Three y/o	Treatment (Wk 1)	Treatment	Feed Day	No	23:05	23:00 - 23:59	Night	38,0	91	58,6	58,6	19,15	586,98	137,28
	2019/05/17	CH2205	Male	Three y/o	Treatment (Wk 1)	Treatment	Feed Day	No	23:10	23:00 - 23:59	Night	37,7	117	33,2	33,2	18,85	605,02	117,68
	2019/05/17	CH2206	Male	Three y/o	Treatment (Wk 1)	Treatment	Feed Day	No	23:10	23:00 - 23:59	Night	38,0	78	55,4	55,4	19,15	575,38	135,33
	2019/05/17	CH2205	Male	Three y/o	Treatment (Wk 1)	Treatment	Feed Day	No	23:15	23:00 - 23:59	Night	37,4		33,6	33,6	18,55		118,09
	2019/05/17	CH2206	Male	Three y/o	Treatment (Wk 1)	Treatment	Feed Day	No	23:15	23:00 - 23:59	Night	38,0	79	52,4	52,4	19,15	576,36	133,41
	2019/05/17	CH2205	Male	Three y/o	Treatment (Wk 1)	Treatment	Feed Day	No	23:20	23:00 - 23:59	Night	37,2		32,4	32,4	18,36		116,84
	2019/05/17	CH2206	Male	Three y/o	Treatment (Wk 1)	Treatment	Feed Day	No	23:20	23:00 - 23:59	Night	38,0	78	50,0	50,0	19,15	575,38	131,79
	2019/05/17	CH2205	Male	Three y/o	Treatment (Wk 1)	Treatment	Feed Day	No	23:25	23:00 - 23:59	Night	37,3		21,4	21,4	18,46		102,63
	2019/05/17	CH2206	Male	Three y/o	Treatment (Wk 1)	Treatment	Feed Day	No	23:25	23:00 - 23:59	Night	38,0	69	56,2	56,2	19,15	565,86	135,83
	2019/05/17	CH2205	Male	Three y/o	Treatment (Wk 1)	Treatment	Feed Day	No	23:30	23:00 - 23:59	Night	37,3	54	20,2	20,2	18,46	545,99	100,65
	2019/05/17	CH2206	Male	Three y/o	Treatment (Wk 1)	Treatment	Feed Day	No	23:30	23:00 - 23:59	Night	37,9	67	59,0	59,0	19,05	563,53	137,51
	2019/05/17	CH2205	Male	Three y/o	Treatment (Wk 1)	Treatment	Feed Day	No	23:35	23:00 - 23:59	Night	37,4	73	11,4	11,4	18,55	570,27	81,19
	2019/05/17	CH2206	Male	Three y/o	Treatment (Wk 1)	Treatment	Feed Day	No	23:35	23:00 - 23:59	Night	37,9	81	44,2	44,2	19,05	578,26	127,53
	2019/05/17	CH2205	Male	Three y/o	Treatment (Wk 1)	Treatment	Feed Day	No	23:40	23:00 - 23:59	Night	37,5	50	13,4	13,4	18,65	539,52	86,68
	2019/05/17	CH2206	Male	Three y/o	Treatment (Wk 1)	Treatment	Feed Day	No	23:40	23:00 - 23:59	Night	37,8	65	36,8	36,8	18,95	561,12	121,22
	2019/05/17	CH2205	Male	Three y/o	Treatment (Wk 1)	Treatment	Feed Day	No	23:45	23:00 - 23:59	Night	37,6	92	7,4	7,4	18,75	587,78	66,59
	2019/05/17	CH2206	Male	Three y/o	Treatment (Wk 1)	Treatment	Feed Day	No	23:45	23:00 - 23:59	Night	37,8	42	41,6	41,6	18,95	524,42	125,44
	2019/05/17	CH2205	Male	Three y/o	Treatment (Wk 1)	Treatment	Feed Day	No	23:50	23:00 - 23:59	Night	37,6	59	13,2	13,2	18,75	553,30	86,17
	2019/05/17	CH2206	Male	Three y/o	Treatment (Wk 1)	Treatment	Feed Day	No	23:50	23:00 - 23:59	Night	37,8	88	59,6	59,6	18,95	584,49	137,86
	2019/05/17	CH2205	Male	Three y/o	Treatment (Wk 1)	Treatment	Feed Day	No	23:55	23:00 - 23:59	Night	37,7	51	12,2	12,2	18,85	541,20	83,49
	2019/05/17	CH2206	Male	Three y/o	Treatment (Wk 1)	Treatment	Feed Day	No	23:55	23:00 - 23:59	Night	37,8	81	38,4	38,4	18,95	578,26	122,68
4	2019/05/18	CH2205	Male	Three y/o	Treatment (Wk 1)	Treatment	Fast Day	No	00:00	00:00 - 00:59	Early Morning	37,7	88	11,0	11,0	18,85	584,49	79,98
	2019/05/18	CH2206	Male	Three y/o	Treatment (Wk 1)	Treatment	Fast Day	No	00:00	00:00 - 00:59	Early Morning	37,7	80	33,6	33,6	18,85	577,31	118,09
	2019/05/18	CH2205	Male	Three y/o	Treatment (Wk 1)	Treatment	Fast Day	No	00:05	00:00 - 00:59	Early Morning	37,7	56	10,0	10,0	18,85	549,01	76,76
	2019/05/18	CH2206	Male	Three y/o	Treatment (Wk 1)	Treatment	Fast Day	No	00:05	00:00 - 00:59	Early Morning	37,7	104	40,4	40,4	18,85	596,69	124,43
	2019/05/18	CH2205	Male	Three y/o	Treatment (Wk 1)	Treatment	Fast Day	No	00:10	00:00 - 00:59	Early Morning	37,7	64	13,2	13,2	18,85	559,88	86,17
	2019/05/18	CH2206	Male	Three y/o	Treatment (Wk 1)	Treatment	Fast Day	No	00:10	00:00 - 00:59	Early Morning	37,7	44	44,2	44,2	18,85	528,51	127,53
	2019/05/18	CH2205	Male	Three y/o	Treatment (Wk 1)	Treatment	Fast Day	No	00:15	00:00 - 00:59	Early Morning	37,7	63	10,4	10,4	18,85	558,62	78,08
	2019/05/18	CH2206	Male	Three y/o	Treatment (Wk 1)	Treatment	Fast Day	No	00:15	00:00 - 00:59	Early Morning	37,7	50	41,4	41,4	18,85	539,52	125,27
	2019/05/18	CH2205	Male	Three y/o	Treatment (Wk 1)	Treatment	Fast Day	No	00:20	00:00 - 00:59	Early Morning	37,7	43	13,2	13,2	18,85	526,50	86,17
	2019/05/18	CH2206	Male	Three y/o	Treatment (Wk 1)	Treatment	Fast Day	No	00:20	00:00 - 00:59	Early Morning	37,7	45	38,6	38,6	18,85	530,47	122,86
	2019/05/18	CH2205	Male	Three y/o	Treatment (Wk 1)	Treatment	Fast Day	No	00:25	00:00 - 00:59	Early Morning	37,7	73	9,6	9,6	18,85	570,27	75,37
	2019/05/18	CH2206	Male	Three y/o	Treatment (Wk 1)	Treatment	Fast Day	No	00:25	00:00 - 00:59	Early Morning	37,5	84	56,0	56,0	18,65	581,01	135,70
	2019/05/18	CH2205	Male	Three y/o	Treatment (Wk 1)	Treatment	Fast Day	No	00:30	00:00 - 00:59	Early Morning	37,7	95	8,8	8,8	18,85	590,14	72,43
	2019/05/18	CH2206	Male	Three y/o	Treatment (Wk 1)	Treatment	Fast Day	No	00:30	00:00 - 00:59	Early Morning	37,5	86	59,4	59,4	18,65	582,77	137,75
	2019/05/18	CH2205	Male	Three y/o	Treatment (Wk 1)	Treatment	Fast Day	No	00:35	00:00 - 00:59	Early Morning	37,7	92	8,4	8,4	18,85	587,78	70,86
	2019/05/18	CH2206	Male	Three y/o	Treatment (Wk 1)	Treatment	Fast Day	No	00:35	00:00 - 00:59	Early Morning	37,5	75	52,8	52,8	18,65	572,36	133,67
	2019/05/18	CH2205	Male	Three y/o	Treatment (Wk 1)	Treatment	Fast Day	No	00:40	00:00 - 00:59	Early Morning	37,7	115	7,0	7,0	18,85	603,82	64,72
	2019/05/18	CH2206	Male	Three y/o	Treatment (Wk 1)	Treatment	Fast Day	No	00:40	00:00 - 00:59	Early Morning	37,5	135	51,4	51,4	18,65	614,84	132,74
	2019/05/18	CH2205	Male	Three y/o	Treatment (Wk 1)	Treatment	Fast Day	No	00:45	00:00 - 00:59	Early Morning	37,7	56	13,8	13,8	18,85	549,01	87,68

Dataset S1: LOGGER

2019/05/18	CH2206	Male	Three y/o	Treatment (Wk 1)	Treatment	Fast Day	No	00:45	00:00 - 00:59	Early Morning	37,5	72	28,8	28,8	18,65	569,20	112,79
2019/05/18	CH2205	Male	Three y/o	Treatment (Wk 1)	Treatment	Fast Day	No	00:50	00:00 - 00:59	Early Morning	37,7	93	10,8	10,8	18,85	588,58	79,36
2019/05/18	CH2206	Male	Three y/o	Treatment (Wk 1)	Treatment	Fast Day	No	00:50	00:00 - 00:59	Early Morning	37,5	113	29,6	29,6	18,65	602,58	113,73
2019/05/18	CH2205	Male	Three y/o	Treatment (Wk 1)	Treatment	Fast Day	No	00:55	00:00 - 00:59	Early Morning	37,7	50	12,0	12,0	18,85	539,52	82,93
2019/05/18	CH2206	Male	Three y/o	Treatment (Wk 1)	Treatment	Fast Day	No	00:55	00:00 - 00:59	Early Morning	37,5	111	22,4	22,4	18,65	601,33	104,19
2019/05/18	CH2205	Male	Three y/o	Treatment (Wk 1)	Treatment	Fast Day	No	01:00	01:00 - 01:59	Early Morning	37,7	100	10,4	10,4	18,85	593,87	78,08
2019/05/18	CH2206	Male	Three y/o	Treatment (Wk 1)	Treatment	Fast Day	No	01:00	01:00 - 01:59	Early Morning	37,5	131	84,2	84,2	18,65	612,80	149,85
2019/05/18	CH2205	Male	Three y/o	Treatment (Wk 1)	Treatment	Fast Day	No	01:05	01:00 - 01:59	Early Morning	37,7		30,0	30,0	18,85		114,20
2019/05/18	CH2206	Male	Three y/o	Treatment (Wk 1)	Treatment	Fast Day	No	01:05	01:00 - 01:59	Early Morning	37,4		43,8	43,8	18,55		127,22
2019/05/18	CH2205	Male	Three y/o	Treatment (Wk 1)	Treatment	Fast Day	No	01:10	01:00 - 01:59	Early Morning	37,6		32,6	32,6	18,75		117,05
2019/05/18	CH2206	Male	Three y/o	Treatment (Wk 1)	Treatment	Fast Day	No	01:10	01:00 - 01:59	Early Morning	37,3	82	46,2	46,2	18,46	579,19	129,06
2019/05/18	CH2205	Male	Three y/o	Treatment (Wk 1)	Treatment	Fast Day	No	01:15	01:00 - 01:59	Early Morning	37,5		30,0	30,0	18,65		114,20
2019/05/18	CH2206	Male	Three y/o	Treatment (Wk 1)	Treatment	Fast Day	No	01:15	01:00 - 01:59	Early Morning	37,2	70	49,0	49,0	18,36	566,99	131,09
2019/05/18	CH2205	Male	Three y/o	Treatment (Wk 1)	Treatment	Fast Day	No	01:20	01:00 - 01:59	Early Morning	37,6		33,0	33,0	18,75		117,47
2019/05/18	CH2206	Male	Three y/o	Treatment (Wk 1)	Treatment	Fast Day	No	01:20	01:00 - 01:59	Early Morning	37,1	61	53,4	53,4	18,26	556,01	134,06
2019/05/18	CH2205	Male	Three y/o	Treatment (Wk 1)	Treatment	Fast Day	No	01:25	01:00 - 01:59	Early Morning	37,6	86	26,6	26,6	18,75	582,77	110,07
2019/05/18	CH2206	Male	Three y/o	Treatment (Wk 1)	Treatment	Fast Day	No	01:25	01:00 - 01:59	Early Morning	37,0	44	54,0	54,0	18,16	528,51	134,45
2019/05/18	CH2205	Male	Three y/o	Treatment (Wk 1)	Treatment	Fast Day	No	01:30	01:00 - 01:59	Early Morning	37,5	58	20,2	20,2	18,65	551,90	100,65
2019/05/18	CH2206	Male	Three y/o	Treatment (Wk 1)	Treatment	Fast Day	No	01:30	01:00 - 01:59	Early Morning	37,0	49	53,4	53,4	18,16	537,80	134,06
2019/05/18	CH2205	Male	Three y/o	Treatment (Wk 1)	Treatment	Fast Day	No	01:35	01:00 - 01:59	Early Morning	37,5	83	17,8	17,8	18,65	580,11	96,34
2019/05/18	CH2206	Male	Three y/o	Treatment (Wk 1)	Treatment	Fast Day	No	01:35	01:00 - 01:59	Early Morning	37,0	80	39,2	39,2	18,16	577,31	123,39
2019/05/18	CH2205	Male	Three y/o	Treatment (Wk 1)	Treatment	Fast Day	No	01:40	01:00 - 01:59	Early Morning	37,4	51	15,4	15,4	18,55	541,20	91,41
2019/05/18	CH2206	Male	Three y/o	Treatment (Wk 1)	Treatment	Fast Day	No	01:40	01:00 - 01:59	Early Morning	36,9	79	35,4	35,4	18,06	576,36	119,88
2019/05/18	CH2205	Male	Three y/o	Treatment (Wk 1)	Treatment	Fast Day	No	01:45	01:00 - 01:59	Early Morning	37,3	83	21,4	21,4	18,46	580,11	102,63
2019/05/18	CH2206	Male	Three y/o	Treatment (Wk 1)	Treatment	Fast Day	No	01:45	01:00 - 01:59	Early Morning	36,8	45	36,6	36,6	17,96	530,47	121,03
2019/05/18	CH2205	Male	Three y/o	Treatment (Wk 1)	Treatment	Fast Day	No	01:50	01:00 - 01:59	Early Morning	37,3	83	27,8	27,8	18,46	580,11	111,58
2019/05/18	CH2206	Male	Three y/o	Treatment (Wk 1)	Treatment	Fast Day	No	01:50	01:00 - 01:59	Early Morning	36,8	108	34,2	34,2	17,96	599,39	118,70
2019/05/18	CH2205	Male	Three y/o	Treatment (Wk 1)	Treatment	Fast Day	No	01:55	01:00 - 01:59	Early Morning	37,2	52	26,8	26,8	18,36	542,83	110,33
2019/05/18	CH2206	Male	Three y/o	Treatment (Wk 1)	Treatment	Fast Day	No	01:55	01:00 - 01:59	Early Morning	36,8	83	35,8	35,8	17,96	580,11	120,27
2019/05/18	CH2205	Male	Three y/o	Treatment (Wk 1)	Treatment	Fast Day	No	02:00	02:00 - 02:59	Early Morning	37,2	67	25,4	25,4	18,36	563,53	108,49
2019/05/18	CH2206	Male	Three y/o	Treatment (Wk 1)	Treatment	Fast Day	No	02:00	02:00 - 02:59	Early Morning	36,8	87	37,6	37,6	17,96	583,64	121,96
2019/05/18	CH2205	Male	Three y/o	Treatment (Wk 1)	Treatment	Fast Day	No	02:05	02:00 - 02:59	Early Morning	37,2	41	25,8	25,8	18,36	522,29	109,02
2019/05/18	CH2206	Male	Three y/o	Treatment (Wk 1)	Treatment	Fast Day	No	02:05	02:00 - 02:59	Early Morning	36,8	45	35,4	35,4	17,96	530,47	119,88
2019/05/18	CH2205	Male	Three y/o	Treatment (Wk 1)	Treatment	Fast Day	No	02:10	02:00 - 02:59	Early Morning	37,3	75	33,4	33,4	18,46	572,36	117,88
2019/05/18	CH2206	Male	Three y/o	Treatment (Wk 1)	Treatment	Fast Day	No	02:10	02:00 - 02:59	Early Morning	36,8	76	35,6	35,6	17,96	573,39	120,08
2019/05/18	CH2205	Male	Three y/o	Treatment (Wk 1)	Treatment	Fast Day	No	02:15	02:00 - 02:59	Early Morning	37,2	63	25,8	25,8	18,36	558,62	109,02
2019/05/18	CH2206	Male	Three y/o	Treatment (Wk 1)	Treatment	Fast Day	No	02:15	02:00 - 02:59	Early Morning	36,8	44	42,0	42,0	17,96	528,51	125,77
2019/05/18	CH2205	Male	Three y/o	Treatment (Wk 1)	Treatment	Fast Day	No	02:20	02:00 - 02:59	Early Morning	37,3	58	23,0	23,0	18,46	551,90	105,09
2019/05/18	CH2206	Male	Three y/o	Treatment (Wk 1)	Treatment	Fast Day	No	02:20	02:00 - 02:59	Early Morning	36,8	93	14,2	14,2	17,96	588,58	88,65
2019/05/18	CH2205	Male	Three y/o	Treatment (Wk 1)	Treatment	Fast Day	No	02:25	02:00 - 02:59	Early Morning	37,4	85	28,2	28,2	18,55	581,90	112,07
2019/05/18	CH2206	Male	Three y/o	Treatment (Wk 1)	Treatment	Fast Day	No	02:25	02:00 - 02:59	Early Morning	36,9	55	13,0	13,0	18,06	547,52	85,65
2019/05/18	CH2205	Male	Three y/o	Treatment (Wk 1)	Treatment	Fast Day	No	02:30	02:00 - 02:59	Early Morning	37,5	53	26,6	26,6	18,65	544,43	110,07
2019/05/18	CH2206	Male	Three y/o	Treatment (Wk 1)	Treatment	Fast Day	No	02:30	02:00 - 02:59	Early Morning	36,9	92	20,8	20,8	18,06	587,78	101,65
2019/05/18	CH2205	Male	Three y/o	Treatment (Wk 1)	Treatment	Fast Day	No	02:35	02:00 - 02:59	Early Morning	37,5	61	24,4	24,4	18,65	556,01	107,11
2019/05/18	CH2206	Male	Three y/o	Treatment (Wk 1)	Treatment	Fast Day	No	02:35	02:00 - 02:59	Early Morning	36,9	80	5,8	5,8	18,06	577,31	58,39
2019/05/18	CH2205	Male	Three y/o	Treatment (Wk 1)	Treatment	Fast Day	No	02:40	02:00 - 02:59	Early Morning	37,6	48	15,8	15,8	18,75	536,04	92,28
2019/05/18	CH2206	Male	Three y/o	Treatment (Wk 1)	Treatment	Fast Day	No	02:40	02:00 - 02:59	Early Morning	36,9	63	6,4	6,4	18,06	558,62	61,70
2019/05/18	CH2205	Male	Three y/o	Treatment (Wk 1)	Treatment	Fast Day	No	02:45	02:00 - 02:59	Early Morning	37,5	45	14,6	14,6	18,65	530,47	89,59
2019/05/18	CH2206	Male	Three y/o	Treatment (Wk 1)	Treatment	Fast Day	No	02:45	02:00 - 02:59	Early Morning	36,9	53	15,8	15,8	18,06	544,43	92,28
2019/05/18	CH2205	Male	Three y/o	Treatment (Wk 1)	Treatment	Fast Day	No	02:50	02:00 - 02:59	Early Morning	37,4	82	13,2	13,2	18,55	579,19	86,17
2019/05/18	CH2206	Male	Three y/o	Treatment (Wk 1)	Treatment	Fast Day	No	02:50	02:00 - 02:59	Early Morning	37,0	57	17,8	17,8	18,16	550,47	96,34
2019/05/18	CH2205	Male	Three y/o	Treatment (Wk 1)	Treatment	Fast Day	No	02:55	02:00 - 02:59	Early Morning	37,4	92	14,6	14,6	18,55	587,78	89,59
2019/05/18	CH2206	Male	Three y/o	Treatment (Wk 1)	Treatment	Fast Day	No	02:55	02:00 - 02:59	Early Morning	37,1	44	12,6	12,6	18,26	528,51	84,59
2019/05/18	CH2205	Male	Three y/o	Treatment (Wk 1)	Treatment	Fast Day	No	03:00	03:00 - 03:59	Early Morning	37,3		33,8	33,8	18,46		118,29
2019/05/18	CH2206	Male	Three y/o	Treatment (Wk 1)	Treatment	Fast Day	No	03:00	03:00 - 03:59	Early Morning	37,2	49	60,0	60,0	18,36	537,80	138,09
2019/05/18	CH2205	Male	Three y/o	Treatment (Wk 1)	Treatment	Fast Day	No	03:05	03:00 - 03:59	Early Morning	37,4		32,2	32,2	18,55		116,63
2019/05/18	CH2206	Male	Three y/o	Treatment (Wk 1)	Treatment	Fast Day	No	03:05	03:00 - 03:59	Early Morning	37,2	72	54,4	54,4	18,36	569,20	134,70
2019/05/18	CH2205	Male	Three y/o	Treatment (Wk 1)	Treatment	Fast Day	No	03:10	03:00 - 03:59	Early Morning	37,4		33,8	33,8	18,55		118,29
2019/05/18	CH2206	Male	Three y/o	Treatment (Wk 1)	Treatment	Fast Day	No	03:10	03:00 - 03:59	Early Morning	37,2	48	58,6	58,6	18,36	536,04	137,28
2019/05/18	CH2205	Male	Three y/o	Treatment (Wk 1)	Treatment	Fast Day	No	03:15	03:00 - 03:59	Early Morning	37,4		29,2	29,2	18,55		113,27
2019/05/18	CH2206	Male	Three y/o	Treatment (Wk 1)	Treatment	Fast Day	No	03:15	03:00 - 03:59	Early Morning	37,1	84	54,2	54,2	18,26	581,01	134,57
2019/05/18	CH2205	Male	Three y/o	Treatment (Wk 1)	Treatment	Fast Day	No	03:20	03:00 - 03:59	Early Morning	37,4		27,0	27,0	18,55		110,58
2019/05/18	CH2206	Male	Three y/o	Treatment (Wk 1)	Treatment	Fast Day	No	03:20	03:00 - 03:59	Early Morning	37,1	79	62,2	62,2	18,26	576,36	139,34
2019/05/18	CH2205	Male	Three y/o	Treatment (Wk 1)	Treatment	Fast Day	No	03:25	03:00 - 03:59	Early Morning	37,4		27,0	27,0	18,55		110,58
2019/05/18	CH2206	Male	Three y/o	Treatment (Wk 1)	Treatment	Fast Day	No	03:25	03:00 - 03:59	Early Morning	37,1	78	58,4	58,4	18,26	575,38	137,16
2019/05/18	CH2205	Male	Three y/o	Treatment (Wk 1)	Treatment	Fast Day	No	03:30	03:00 - 03:59	Early Morning	37,4	69	27,0	27,0	18,55	565,86	110,58
2019/05/18	CH2206	Male	Three y/o	Treatment (Wk 1)	Treatment	Fast Day	No	03:30	03:00 - 03:59	Early Morning	37,2	58	60,4	60,4	18,36	551,90	138,32
2019/05/18	CH2205	Male	Three y/o	Treatment (Wk 1)	Treatment	Fast Day	No	03:35	03:00 - 03:59	Early Morning	37,4	56	23,2	23,2	18,55	549,01	105,39
2019/05/18	CH2206	Male	Three y/o	Treatment (Wk 1)	Treatment	Fast Day	No	03:35	03:00 - 03:59	Early Morning	37,2	46	61,4	61,4	18,36	532,38	138,89
2019/05/18	CH2205	Male	Three y/o	Treatment (Wk 1)	Treatment	Fast Day	No	03:40	03:00 - 03:59	Early Morning	37,4	62	23,2	23,2	18,55	557,33	105,39

2019/05/18	CH2206	Male	Three y/o	Treatment (Wk 1)	Treatment	Fast Day	No	03:40	03:00 - 03:59	Early Morning	37,3	57	64,0	64,0	18,46	550,47	140,33
2019/05/18	CH2205	Male	Three y/o	Treatment (Wk 1)	Treatment	Fast Day	No	03:45	03:00 - 03:59	Early Morning	37,4	50	14,4	14,4	18,55	539,52	89,12
2019/05/18	CH2206	Male	Three y/o	Treatment (Wk 1)	Treatment	Fast Day	No	03:45	03:00 - 03:59	Early Morning	37,2	55	66,4	66,4	18,36	547,52	141,61
2019/05/18	CH2205	Male	Three y/o	Treatment (Wk 1)	Treatment	Fast Day	No	03:50	03:00 - 03:59	Early Morning	37,4	55	10,2	10,2	18,55	547,52	77,43
2019/05/18	CH2206	Male	Three y/o	Treatment (Wk 1)	Treatment	Fast Day	No	03:50	03:00 - 03:59	Early Morning	37,2	55	59,2	59,2	18,36	547,52	137,63
2019/05/18	CH2205	Male	Three y/o	Treatment (Wk 1)	Treatment	Fast Day	No	03:55	03:00 - 03:59	Early Morning	37,4	82	7,6	7,6	18,55	579,19	67,49
2019/05/18	CH2206	Male	Three y/o	Treatment (Wk 1)	Treatment	Fast Day	No	03:55	03:00 - 03:59	Early Morning	37,2	71	63,0	63,0	18,36	568,10	139,78
2019/05/18	CH2205	Male	Three y/o	Treatment (Wk 1)	Treatment	Fast Day	No	04:00	04:00 - 04:59	Morning	37,4	47	6,6	6,6	18,55	534,23	62,73
2019/05/18	CH2206	Male	Three y/o	Treatment (Wk 1)	Treatment	Fast Day	No	04:00	04:00 - 04:59	Morning	37,2	48	66,4	66,4	18,36	536,04	141,61
2019/05/18	CH2205	Male	Three y/o	Treatment (Wk 1)	Treatment	Fast Day	No	04:05	04:00 - 04:59	Morning	37,4	52	66,4	66,4	18,55	542,83	141,61
2019/05/18	CH2206	Male	Three y/o	Treatment (Wk 1)	Treatment	Fast Day	No	04:05	04:00 - 04:59	Morning	37,2	57	51,4	51,4	18,36	550,47	132,74
2019/05/18	CH2205	Male	Three y/o	Treatment (Wk 1)	Treatment	Fast Day	No	04:10	04:00 - 04:59	Morning	37,5	162	98,8	98,8	18,65	626,88	155,42
2019/05/18	CH2206	Male	Three y/o	Treatment (Wk 1)	Treatment	Fast Day	No	04:10	04:00 - 04:59	Morning	37,3	118	153,2	153,2	18,46	605,61	170,76
2019/05/18	CH2205	Male	Three y/o	Treatment (Wk 1)	Treatment	Fast Day	No	04:15	04:00 - 04:59	Morning	37,4		18,8	18,8	18,55		98,20
2019/05/18	CH2206	Male	Three y/o	Treatment (Wk 1)	Treatment	Fast Day	No	04:15	04:00 - 04:59	Morning	37,3	110	140,2	140,2	18,46	600,69	167,65
2019/05/18	CH2205	Male	Three y/o	Treatment (Wk 1)	Treatment	Fast Day	No	04:20	04:00 - 04:59	Morning	37,3	132	57,6	57,6	18,46	613,32	136,68
2019/05/18	CH2206	Male	Three y/o	Treatment (Wk 1)	Treatment	Fast Day	No	04:20	04:00 - 04:59	Morning	37,2	98	226,0	226,0	18,36	592,41	184,42
2019/05/18	CH2205	Male	Three y/o	Treatment (Wk 1)	Treatment	Fast Day	No	04:25	04:00 - 04:59	Morning	37,3	121	250,4	250,4	18,46	607,35	188,04
2019/05/18	CH2206	Male	Three y/o	Treatment (Wk 1)	Treatment	Fast Day	No	04:25	04:00 - 04:59	Morning	37,2	118	244,8	244,8	18,36	605,61	187,24
2019/05/18	CH2205	Male	Three y/o	Treatment (Wk 1)	Treatment	Fast Day	No	04:30	04:00 - 04:59	Morning	37,3	177	80,2	80,2	18,46	632,55	148,16
2019/05/18	CH2206	Male	Three y/o	Treatment (Wk 1)	Treatment	Fast Day	No	04:30	04:00 - 04:59	Morning	37,3	112	116,6	116,6	18,46	601,96	161,20
2019/05/18	CH2205	Male	Three y/o	Treatment (Wk 1)	Treatment	Fast Day	No	04:35	04:00 - 04:59	Morning	37,4	107	25,0	25,0	18,55	598,73	107,94
2019/05/18	CH2206	Male	Three y/o	Treatment (Wk 1)	Treatment	Fast Day	No	04:35	04:00 - 04:59	Morning	37,3	102	205,2	205,2	18,46	595,30	181,02
2019/05/18	CH2205	Male	Three y/o	Treatment (Wk 1)	Treatment	Fast Day	No	04:40	04:00 - 04:59	Morning	37,1	72	21,0	21,0	18,26	569,20	101,98
2019/05/18	CH2206	Male	Three y/o	Treatment (Wk 1)	Treatment	Fast Day	No	04:40	04:00 - 04:59	Morning	37,4	120	53,2	53,2	18,55	606,78	133,93
2019/05/18	CH2205	Male	Three y/o	Treatment (Wk 1)	Treatment	Fast Day	No	04:45	04:00 - 04:59	Morning	37,0	98	23,0	23,0	18,16	592,41	105,09
2019/05/18	CH2206	Male	Three y/o	Treatment (Wk 1)	Treatment	Fast Day	No	04:45	04:00 - 04:59	Morning	37,2	48	63,8	63,8	18,36	536,04	140,22
2019/05/18	CH2205	Male	Three y/o	Treatment (Wk 1)	Treatment	Fast Day	No	04:50	04:00 - 04:59	Morning	36,9	80	24,2	24,2	18,06	577,31	106,83
2019/05/18	CH2206	Male	Three y/o	Treatment (Wk 1)	Treatment	Fast Day	No	04:50	04:00 - 04:59	Morning	37,0	54	113,2	113,2	18,16	545,99	160,17
2019/05/18	CH2205	Male	Three y/o	Treatment (Wk 1)	Treatment	Fast Day	No	04:55	04:00 - 04:59	Morning	36,9		35,4	35,4	18,06		119,88
2019/05/18	CH2206	Male	Three y/o	Treatment (Wk 1)	Treatment	Fast Day	No	04:55	04:00 - 04:59	Morning	37,2	114	117,6	117,6	18,36	603,20	161,50
2019/05/18	CH2205	Male	Three y/o	Treatment (Wk 1)	Treatment	Fast Day	No	05:00	05:00 - 05:59	Morning	37,1		30,0	30,0	18,26		114,20
2019/05/18	CH2206	Male	Three y/o	Treatment (Wk 1)	Treatment	Fast Day	No	05:00	05:00 - 05:59	Morning	37,3	107	99,0	99,0	18,46	598,73	155,49
2019/05/18	CH2205	Male	Three y/o	Treatment (Wk 1)	Treatment	Fast Day	No	05:05	05:00 - 05:59	Morning	37,0	160	243,8	243,8	18,16	626,07	187,09
2019/05/18	CH2206	Male	Three y/o	Treatment (Wk 1)	Treatment	Fast Day	No	05:05	05:00 - 05:59	Morning	37,3	110	46,2	46,2	18,46	600,69	129,06
2019/05/18	CH2205	Male	Three y/o	Treatment (Wk 1)	Treatment	Fast Day	No	05:10	05:00 - 05:59	Morning	37,1	109	143,0	143,0	18,26	600,04	168,34
2019/05/18	CH2206	Male	Three y/o	Treatment (Wk 1)	Treatment	Fast Day	No	05:10	05:00 - 05:59	Morning	37,4	103	180,8	180,8	18,55	596,00	176,57
2019/05/18	CH2205	Male	Three y/o	Treatment (Wk 1)	Treatment	Fast Day	No	05:15	05:00 - 05:59	Morning	37,2		145,6	145,6	18,36		168,97
2019/05/18	CH2206	Male	Three y/o	Treatment (Wk 1)	Treatment	Fast Day	No	05:15	05:00 - 05:59	Morning	37,4	137	187,6	187,6	18,55	615,83	177,87
2019/05/18	CH2205	Male	Three y/o	Treatment (Wk 1)	Treatment	Fast Day	No	05:20	05:00 - 05:59	Morning	37,2		116,6	116,6	18,36		161,20
2019/05/18	CH2206	Male	Three y/o	Treatment (Wk 1)	Treatment	Fast Day	No	05:20	05:00 - 05:59	Morning	37,5	117	140,2	140,2	18,65	605,02	167,65
2019/05/18	CH2205	Male	Three y/o	Treatment (Wk 1)	Treatment	Fast Day	No	05:25	05:00 - 05:59	Morning	37,3	99	130,4	130,4	18,46	593,14	165,11
2019/05/18	CH2206	Male	Three y/o	Treatment (Wk 1)	Treatment	Fast Day	No	05:25	05:00 - 05:59	Morning	37,4	84	225,8	225,8	18,55	581,01	184,39
2019/05/18	CH2205	Male	Three y/o	Treatment (Wk 1)	Treatment	Fast Day	No	05:30	05:00 - 05:59	Morning	37,1	117	22,0	22,0	18,26	605,02	103,57
2019/05/18	CH2206	Male	Three y/o	Treatment (Wk 1)	Treatment	Fast Day	No	05:30	05:00 - 05:59	Morning	37,5	108	156,6	156,6	18,65	599,39	171,53
2019/05/18	CH2205	Male	Three y/o	Treatment (Wk 1)	Treatment	Fast Day	No	05:35	05:00 - 05:59	Morning	37,0		24,0	24,0	18,16		106,55
2019/05/18	CH2206	Male	Three y/o	Treatment (Wk 1)	Treatment	Fast Day	No	05:35	05:00 - 05:59	Morning	37,5	101	148,4	148,4	18,65	594,59	169,64
2019/05/18	CH2205	Male	Three y/o	Treatment (Wk 1)	Treatment	Fast Day	No	05:40	05:00 - 05:59	Morning	37,0	115	72,8	72,8	18,16	603,82	144,80
2019/05/18	CH2206	Male	Three y/o	Treatment (Wk 1)	Treatment	Fast Day	No	05:40	05:00 - 05:59	Morning	37,5	110	137,0	137,0	18,65	600,69	166,84
2019/05/18	CH2205	Male	Three y/o	Treatment (Wk 1)	Treatment	Fast Day	No	05:45	05:00 - 05:59	Morning	37,1		53,0	53,0	18,26		133,80
2019/05/18	CH2206	Male	Three y/o	Treatment (Wk 1)	Treatment	Fast Day	No	05:45	05:00 - 05:59	Morning	37,7	104	134,8	134,8	18,85	596,69	166,28
2019/05/18	CH2205	Male	Three y/o	Treatment (Wk 1)	Treatment	Fast Day	No	05:50	05:00 - 05:59	Morning	37,3	78	73,8	73,8	18,46	575,38	145,27
2019/05/18	CH2206	Male	Three y/o	Treatment (Wk 1)	Treatment	Fast Day	No	05:50	05:00 - 05:59	Morning	37,7	174	64,0	64,0	18,85	631,46	140,33
2019/05/18	CH2205	Male	Three y/o	Treatment (Wk 1)	Treatment	Fast Day	No	05:55	05:00 - 05:59	Morning	37,3	79	278,8	278,8	18,46	576,36	191,83
2019/05/18	CH2206	Male	Three y/o	Treatment (Wk 1)	Treatment	Fast Day	No	05:55	05:00 - 05:59	Morning	37,7	103	176,0	176,0	18,85	596,00	175,63
2019/05/18	CH2205	Male	Three y/o	Treatment (Wk 1)	Treatment	Fast Day	No	06:00	06:00 - 06:59	Morning	37,4	98	112,2	112,2	18,55	592,41	159,86
2019/05/18	CH2206	Male	Three y/o	Treatment (Wk 1)	Treatment	Fast Day	No	06:00	06:00 - 06:59	Morning	37,7	137	84,8	84,8	18,85	615,83	150,10
2019/05/18	CH2205	Male	Three y/o	Treatment (Wk 1)	Treatment	Fast Day	No	06:05	06:00 - 06:59	Morning	37,4	120	71,0	71,0	18,55	606,78	143,93
2019/05/18	CH2206	Male	Three y/o	Treatment (Wk 1)	Treatment	Fast Day	No	06:05	06:00 - 06:59	Morning	37,8	125	55,4	55,4	18,95	609,60	135,33
2019/05/18	CH2205	Male	Three y/o	Treatment (Wk 1)	Treatment	Fast Day	No	06:10	06:00 - 06:59	Morning	37,6		102,0	102,0	18,75		156,53
2019/05/18	CH2206	Male	Three y/o	Treatment (Wk 1)	Treatment	Fast Day	No	06:10	06:00 - 06:59	Morning	38,0	106	15,8	15,8	19,15	598,06	92,28
2019/05/18	CH2205	Male	Three y/o	Treatment (Wk 1)	Treatment	Fast Day	No	06:15	06:00 - 06:59	Morning	37,6	91	55,4	55,4	18,75	586,98	135,33
2019/05/18	CH2206	Male	Three y/o	Treatment (Wk 1)	Treatment	Fast Day	No	06:15	06:00 - 06:59	Morning	38,0	98	43,6	43,6	19,15	592,41	127,06
2019/05/18	CH2205	Male	Three y/o	Treatment (Wk 1)	Treatment	Fast Day	No	06:20	06:00 - 06:59	Morning	37,5		20,6	20,6	18,65		101,32
2019/05/18	CH2206	Male	Three y/o	Treatment (Wk 1)	Treatment	Fast Day	No	06:20	06:00 - 06:59	Morning	37,9	113	147,8	147,8	19,05	602,58	169,50
2019/05/18	CH2205	Male	Three y/o	Treatment (Wk 1)	Treatment	Fast Day	No	06:25	06:00 - 06:59	Morning	37,3		15,6	15,6	18,46		91,85
2019/05/18	CH2206	Male	Three y/o	Treatment (Wk 1)	Treatment	Fast Day	No	06:25	06:00 - 06:59	Morning	37,8	117	70,8	70,8	18,95	605,02	143,83
2019/05/18	CH2205	Male	Three y/o	Treatment (Wk 1)	Treatment	Fast Day	No	06:30	06:00 - 06:59	Morning	37,4	89	104,8	104,8	18,55	585,33	157,48
2019/05/18	CH2206	Male	Three y/o	Treatment (Wk 1)	Treatment	Fast Day	No	06:30	06:00 - 06:59	Morning	37,8	131	207,8	207,8	18,95	612,80	181,47
2019/05/18	CH2205	Male	Three y/o	Treatment (Wk 1)	Treatment	Fast Day	No	06:35	06:00 - 06:59	Morning	37,4	180	375,6	375,6	18,55	633,61	202,38

2019/05/18	CH2206	Male	Three y/o	Treatment (Wk 1)	Treatment	Fast Day	No	06:35	06:00 - 06:59	Morning	37,7	167	193,2	193,2	18,85	628,84	178,90
2019/05/18	CH2205	Male	Three y/o	Treatment (Wk 1)	Treatment	Fast Day	No	06:40	06:00 - 06:59	Morning	37,4	107	68,6	68,6	18,55	598,73	142,74
2019/05/18	CH2206	Male	Three y/o	Treatment (Wk 1)	Treatment	Fast Day	No	06:40	06:00 - 06:59	Morning	37,7	113	156,2	156,2	18,85	602,58	171,44
2019/05/18	CH2205	Male	Three y/o	Treatment (Wk 1)	Treatment	Fast Day	No	06:45	06:00 - 06:59	Morning	37,5	45	105,2	105,2	18,65	530,47	157,61
2019/05/18	CH2206	Male	Three y/o	Treatment (Wk 1)	Treatment	Fast Day	No	06:45	06:00 - 06:59	Morning	37,7		180,0	180,0	18,85		176,41
2019/05/18	CH2205	Male	Three y/o	Treatment (Wk 1)	Treatment	Fast Day	No	06:50	06:00 - 06:59	Morning	37,4	49	70,2	70,2	18,55	537,80	143,54
2019/05/18	CH2206	Male	Three y/o	Treatment (Wk 1)	Treatment	Fast Day	No	06:50	06:00 - 06:59	Morning	37,7	128	81,4	81,4	18,85	611,22	148,68
2019/05/18	CH2205	Male	Three y/o	Treatment (Wk 1)	Treatment	Fast Day	No	06:55	06:00 - 06:59	Morning	37,5	126	160,2	160,2	18,65	610,14	172,32
2019/05/18	CH2206	Male	Three y/o	Treatment (Wk 1)	Treatment	Fast Day	No	06:55	06:00 - 06:59	Morning	37,7	111	156,2	156,2	18,85	601,33	171,44
2019/05/18	CH2205	Male	Three y/o	Treatment (Wk 1)	Treatment	Fast Day	No	07:00	07:00 - 07:59	Morning	37,5	68	310,8	310,8	18,65	564,71	195,67
2019/05/18	CH2206	Male	Three y/o	Treatment (Wk 1)	Treatment	Fast Day	No	07:00	07:00 - 07:59	Morning	37,7		132,4	132,4	18,85		165,65
2019/05/18	CH2205	Male	Three y/o	Treatment (Wk 1)	Treatment	Fast Day	No	07:05	07:00 - 07:59	Morning	37,5	95	119,0	119,0	18,65	590,14	161,92
2019/05/18	CH2206	Male	Three y/o	Treatment (Wk 1)	Treatment	Fast Day	No	07:05	07:00 - 07:59	Morning	37,7	115	96,2	96,2	18,85	603,82	154,49
2019/05/18	CH2205	Male	Three y/o	Treatment (Wk 1)	Treatment	Fast Day	No	07:10	07:00 - 07:59	Morning	37,5	115	125,6	125,6	18,65	603,82	163,80
2019/05/18	CH2206	Male	Three y/o	Treatment (Wk 1)	Treatment	Fast Day	No	07:10	07:00 - 07:59	Morning	37,7	102	262,8	262,8	18,85	595,30	189,74
2019/05/18	CH2205	Male	Three y/o	Treatment (Wk 1)	Treatment	Fast Day	No	07:15	07:00 - 07:59	Morning	37,5	51	63,0	63,0	18,65	541,20	139,78
2019/05/18	CH2206	Male	Three y/o	Treatment (Wk 1)	Treatment	Fast Day	No	07:15	07:00 - 07:59	Morning	37,7	118	167,0	167,0	18,85	605,61	173,78
2019/05/18	CH2205	Male	Three y/o	Treatment (Wk 1)	Treatment	Fast Day	No	07:20	07:00 - 07:59	Morning	37,6	101	91,2	91,2	18,75	594,59	152,63
2019/05/18	CH2206	Male	Three y/o	Treatment (Wk 1)	Treatment	Fast Day	No	07:20	07:00 - 07:59	Morning	37,7	174	127,0	127,0	18,85	631,46	164,19
2019/05/18	CH2205	Male	Three y/o	Treatment (Wk 1)	Treatment	Fast Day	No	07:25	07:00 - 07:59	Morning	37,6	80	113,0	113,0	18,75	577,31	160,11
2019/05/18	CH2206	Male	Three y/o	Treatment (Wk 1)	Treatment	Fast Day	No	07:25	07:00 - 07:59	Morning	37,7	103	78,4	78,4	18,85	596,00	147,37
2019/05/18	CH2205	Male	Three y/o	Treatment (Wk 1)	Treatment	Fast Day	No	07:30	07:00 - 07:59	Morning	37,6	103	162,0	162,0	18,75	596,00	172,72
2019/05/18	CH2206	Male	Three y/o	Treatment (Wk 1)	Treatment	Fast Day	No	07:30	07:00 - 07:59	Morning	37,7	113	83,8	83,8	18,85	602,58	149,69
2019/05/18	CH2205	Male	Three y/o	Treatment (Wk 1)	Treatment	Fast Day	No	07:35	07:00 - 07:59	Morning	37,6	101	138,8	138,8	18,75	594,59	167,30
2019/05/18	CH2206	Male	Three y/o	Treatment (Wk 1)	Treatment	Fast Day	No	07:35	07:00 - 07:59	Morning	37,8	111	206,2	206,2	18,95	601,33	181,19
2019/05/18	CH2205	Male	Three y/o	Treatment (Wk 1)	Treatment	Fast Day	No	07:40	07:00 - 07:59	Morning	37,6		62,4	62,4	18,75		139,45
2019/05/18	CH2206	Male	Three y/o	Treatment (Wk 1)	Treatment	Fast Day	No	07:40	07:00 - 07:59	Morning	37,8		318,0	318,0	18,95		196,48
2019/05/18	CH2205	Male	Three y/o	Treatment (Wk 1)	Treatment	Fast Day	No	07:45	07:00 - 07:59	Morning	37,6		59,2	59,2	18,75		137,63
2019/05/18	CH2206	Male	Three y/o	Treatment (Wk 1)	Treatment	Fast Day	No	07:45	07:00 - 07:59	Morning	37,8	128	102,2	102,2	18,95	611,22	156,60
2019/05/18	CH2205	Male	Three y/o	Treatment (Wk 1)	Treatment	Fast Day	No	07:50	07:00 - 07:59	Morning	37,6	104	262,6	262,6	18,75	596,69	189,72
2019/05/18	CH2206	Male	Three y/o	Treatment (Wk 1)	Treatment	Fast Day	No	07:50	07:00 - 07:59	Morning	37,8	117	169,6	169,6	18,95	605,02	174,32
2019/05/18	CH2205	Male	Three y/o	Treatment (Wk 1)	Treatment	Fast Day	No	07:55	07:00 - 07:59	Morning	37,6	108	70,4	70,4	18,75	599,39	143,64
2019/05/18	CH2206	Male	Three y/o	Treatment (Wk 1)	Treatment	Fast Day	No	07:55	07:00 - 07:59	Morning	37,8	115	138,2	138,2	18,95	603,82	167,15
2019/05/18	CH2205	Male	Three y/o	Treatment (Wk 1)	Treatment	Fast Day	No	08:00	08:00 - 08:59	Late Morning	37,7	129	124,4	124,4	18,85	611,75	163,47
2019/05/18	CH2206	Male	Three y/o	Treatment (Wk 1)	Treatment	Fast Day	No	08:00	08:00 - 08:59	Late Morning	37,8	84	233,0	233,0	18,95	581,01	185,50
2019/05/18	CH2205	Male	Three y/o	Treatment (Wk 1)	Treatment	Fast Day	No	08:05	08:00 - 08:59	Late Morning	37,6	54	88,6	88,6	18,75	545,99	151,63
2019/05/18	CH2206	Male	Three y/o	Treatment (Wk 1)	Treatment	Fast Day	No	08:05	08:00 - 08:59	Late Morning	37,8	101	110,8	110,8	18,95	594,59	159,42
2019/05/18	CH2205	Male	Three y/o	Treatment (Wk 1)	Treatment	Fast Day	No	08:10	08:00 - 08:59	Late Morning	37,6		426,0	426,0	18,75		206,85
2019/05/18	CH2206	Male	Three y/o	Treatment (Wk 1)	Treatment	Fast Day	No	08:10	08:00 - 08:59	Late Morning	37,8	108	95,6	95,6	18,95	599,39	154,28
2019/05/18	CH2205	Male	Three y/o	Treatment (Wk 1)	Treatment	Fast Day	No	08:15	08:00 - 08:59	Late Morning	37,6	53	24,0	24,0	18,75	544,43	106,55
2019/05/18	CH2206	Male	Three y/o	Treatment (Wk 1)	Treatment	Fast Day	No	08:15	08:00 - 08:59	Late Morning	37,8	129	78,0	78,0	18,95	611,75	147,20
2019/05/18	CH2205	Male	Three y/o	Treatment (Wk 1)	Treatment	Fast Day	No	08:20	08:00 - 08:59	Late Morning	37,5	72	32,6	32,6	18,65	569,20	117,05
2019/05/18	CH2206	Male	Three y/o	Treatment (Wk 1)	Treatment	Fast Day	No	08:20	08:00 - 08:59	Late Morning	37,8		83,6	83,6	18,95		149,61
2019/05/18	CH2205	Male	Three y/o	Treatment (Wk 1)	Treatment	Fast Day	No	08:25	08:00 - 08:59	Late Morning	37,4		22,8	22,8	18,55		104,79
2019/05/18	CH2206	Male	Three y/o	Treatment (Wk 1)	Treatment	Fast Day	No	08:25	08:00 - 08:59	Late Morning	37,7	103	207,2	207,2	18,85	596,00	181,36
2019/05/18	CH2205	Male	Three y/o	Treatment (Wk 1)	Treatment	Fast Day	No	08:30	08:00 - 08:59	Late Morning	37,5	64	43,4	43,4	18,65	559,88	126,90
2019/05/18	CH2206	Male	Three y/o	Treatment (Wk 1)	Treatment	Fast Day	No	08:30	08:00 - 08:59	Late Morning	37,7	121	90,4	90,4	18,85	607,35	152,33
2019/05/18	CH2205	Male	Three y/o	Treatment (Wk 1)	Treatment	Fast Day	No	08:35	08:00 - 08:59	Late Morning	37,6		99,6	99,6	18,75		155,70
2019/05/18	CH2206	Male	Three y/o	Treatment (Wk 1)	Treatment	Fast Day	No	08:35	08:00 - 08:59	Late Morning	37,8	104	149,4	149,4	18,95	596,69	169,88
2019/05/18	CH2205	Male	Three y/o	Treatment (Wk 1)	Treatment	Fast Day	No	08:40	08:00 - 08:59	Late Morning	37,7	102	79,2	79,2	18,85	595,30	147,73
2019/05/18	CH2206	Male	Three y/o	Treatment (Wk 1)	Treatment	Fast Day	No	08:40	08:00 - 08:59	Late Morning	37,7	109	72,6	72,6	18,85	600,04	144,70
2019/05/18	CH2205	Male	Three y/o	Treatment (Wk 1)	Treatment	Fast Day	No	08:45	08:00 - 08:59	Late Morning	37,6	67	37,8	37,8	18,75	563,53	122,14
2019/05/18	CH2206	Male	Three y/o	Treatment (Wk 1)	Treatment	Fast Day	No	08:45	08:00 - 08:59	Late Morning	37,7		186,6	186,6	18,85		177,68
2019/05/18	CH2205	Male	Three y/o	Treatment (Wk 1)	Treatment	Fast Day	No	08:50	08:00 - 08:59	Late Morning	37,6	97	54,6	54,6	18,75	591,66	134,83
2019/05/18	CH2206	Male	Three y/o	Treatment (Wk 1)	Treatment	Fast Day	No	08:50	08:00 - 08:59	Late Morning	37,7	124	404,8	404,8	18,85	609,04	205,04
2019/05/18	CH2205	Male	Three y/o	Treatment (Wk 1)	Treatment	Fast Day	No	08:55	08:00 - 08:59	Late Morning	37,6	62	13,4	13,4	18,75	557,33	86,68
2019/05/18	CH2206	Male	Three y/o	Treatment (Wk 1)	Treatment	Fast Day	No	08:55	08:00 - 08:59	Late Morning	37,7	103	36,2	36,2	18,85	596,00	120,65
2019/05/18	CH2205	Male	Three y/o	Treatment (Wk 1)	Treatment	Fast Day	No	09:00	09:00 - 09:59	Late Morning	37,6	60	13,2	13,2	18,75	554,67	86,17
2019/05/18	CH2206	Male	Three y/o	Treatment (Wk 1)	Treatment	Fast Day	No	09:00	09:00 - 09:59	Late Morning	37,7	112	136,0	136,0	18,85	601,96	166,59
2019/05/18	CH2205	Male	Three y/o	Treatment (Wk 1)	Treatment	Fast Day	No	09:05	09:00 - 09:59	Late Morning	37,6		17,2	17,2	18,75		95,17
2019/05/18	CH2206	Male	Three y/o	Treatment (Wk 1)	Treatment	Fast Day	No	09:05	09:00 - 09:59	Late Morning	37,6	90	153,4	153,4	18,75	586,16	170,80
2019/05/18	CH2205	Male	Three y/o	Treatment (Wk 1)	Treatment	Fast Day	No	09:10	09:00 - 09:59	Late Morning	37,6		6,0	6,0	18,75		59,53
2019/05/18	CH2206	Male	Three y/o	Treatment (Wk 1)	Treatment	Fast Day	No	09:10	09:00 - 09:59	Late Morning	37,7	115	124,6	124,6	18,85	603,82	163,52
2019/05/18	CH2205	Male	Three y/o	Treatment (Wk 1)	Treatment	Fast Day	No	09:15	09:00 - 09:59	Late Morning	37,5		9,4	9,4	18,65		74,66
2019/05/18	CH2206	Male	Three y/o	Treatment (Wk 1)	Treatment	Fast Day	No	09:15	09:00 - 09:59	Late Morning	37,6	115	140,0	140,0	18,75	603,82	167,60
2019/05/18	CH2205	Male	Three y/o	Treatment (Wk 1)	Treatment	Fast Day	No	09:20	09:00 - 09:59	Late Morning	37,6	47	8,2	8,2	18,75	534,23	70,05
2019/05/18	CH2206	Male	Three y/o	Treatment (Wk 1)	Treatment	Fast Day	No	09:20	09:00 - 09:59	Late Morning	37,5	194	20,4	20,4	18,65	638,30	100,99
2019/05/18	CH2205	Male	Three y/o	Treatment (Wk 1)	Treatment	Fast Day	No	09:25	09:00 - 09:59	Late Morning	37,6	58	46,0	46,0	18,75	551,90	128,91
2019/05/18	CH2206	Male	Three y/o	Treatment (Wk 1)	Treatment	Fast Day	No	09:25	09:00 - 09:59	Late Morning	37,5	162	121,2	121,2	18,65	626,88	162,56
2019/05/18	CH2205	Male	Three y/o	Treatment (Wk 1)	Treatment	Fast Day	No	09:30	09:00 - 09:59	Late Morning	37,6	57	6,4	6,4	18,75	550,47	61,70

2019/05/18	CH2206	Male	Three y/o	Treatment (Wk 1)	Treatment	Fast Day	No	09:30	09:00 - 09:59	Late Morning	37,7	110	24,6	24,6	18,85	600,69	107,39
2019/05/18	CH2205	Male	Three y/o	Treatment (Wk 1)	Treatment	Fast Day	No	09:35	09:00 - 09:59	Late Morning	37,6	55	6,2	6,2	18,75	547,52	60,63
2019/05/18	CH2206	Male	Three y/o	Treatment (Wk 1)	Treatment	Fast Day	No	09:35	09:00 - 09:59	Late Morning	37,7	91	77,4	77,4	18,85	586,98	146,93
2019/05/18	CH2205	Male	Three y/o	Treatment (Wk 1)	Treatment	Fast Day	No	09:40	09:00 - 09:59	Late Morning	37,6	53	5,8	5,8	18,75	544,43	58,39
2019/05/18	CH2206	Male	Three y/o	Treatment (Wk 1)	Treatment	Fast Day	No	09:40	09:00 - 09:59	Late Morning	37,8	96	78,2	78,2	18,95	590,91	147,28
2019/05/18	CH2205	Male	Three y/o	Treatment (Wk 1)	Treatment	Fast Day	No	09:45	09:00 - 09:59	Late Morning	37,6	51	3,6	3,6	18,75	541,20	42,42
2019/05/18	CH2206	Male	Three y/o	Treatment (Wk 1)	Treatment	Fast Day	No	09:45	09:00 - 09:59	Late Morning	37,8	91	4,4	4,4	18,95	586,98	49,12
2019/05/18	CH2205	Male	Three y/o	Treatment (Wk 1)	Treatment	Fast Day	No	09:50	09:00 - 09:59	Late Morning	37,6	63	2,8	2,8	18,75	558,62	34,04
2019/05/18	CH2206	Male	Three y/o	Treatment (Wk 1)	Treatment	Fast Day	No	09:50	09:00 - 09:59	Late Morning	37,7	54	19,2	19,2	18,85	545,99	98,92
2019/05/18	CH2205	Male	Three y/o	Treatment (Wk 1)	Treatment	Fast Day	No	09:55	09:00 - 09:59	Late Morning	37,6	45	3,4	3,4	18,75	530,47	40,51
2019/05/18	CH2206	Male	Three y/o	Treatment (Wk 1)	Treatment	Fast Day	No	09:55	09:00 - 09:59	Late Morning	37,7	50	40,0	40,0	18,85	539,52	124,09
2019/05/18	CH2205	Male	Three y/o	Treatment (Wk 1)	Treatment	Fast Day	No	10:00	10:00 - 10:59	Late Morning	37,5	47	5,2	5,2	18,65	534,23	54,72
2019/05/18	CH2206	Male	Three y/o	Treatment (Wk 1)	Treatment	Fast Day	No	10:00	10:00 - 10:59	Late Morning	37,5	50	35,2	35,2	18,65	539,52	119,69
2019/05/18	CH2205	Male	Three y/o	Treatment (Wk 1)	Treatment	Fast Day	No	10:05	10:00 - 10:59	Late Morning	37,5	42	13,4	13,4	18,65	524,42	86,68
2019/05/18	CH2206	Male	Three y/o	Treatment (Wk 1)	Treatment	Fast Day	No	10:05	10:00 - 10:59	Late Morning	37,5	46	28,0	28,0	18,65	532,38	111,83
2019/05/18	CH2205	Male	Three y/o	Treatment (Wk 1)	Treatment	Fast Day	No	10:10	10:00 - 10:59	Late Morning	37,5	66	5,2	5,2	18,65	562,34	54,72
2019/05/18	CH2206	Male	Three y/o	Treatment (Wk 1)	Treatment	Fast Day	No	10:10	10:00 - 10:59	Late Morning	37,5	112	103,6	103,6	18,65	601,96	157,08
2019/05/18	CH2205	Male	Three y/o	Treatment (Wk 1)	Treatment	Fast Day	No	10:15	10:00 - 10:59	Late Morning	37,5	51	41,8	41,8	18,65	541,20	125,60
2019/05/18	CH2206	Male	Three y/o	Treatment (Wk 1)	Treatment	Fast Day	No	10:15	10:00 - 10:59	Late Morning	37,5	98	209,4	209,4	18,65	592,41	181,74
2019/05/18	CH2205	Male	Three y/o	Treatment (Wk 1)	Treatment	Fast Day	No	10:20	10:00 - 10:59	Late Morning	37,5	57	9,2	9,2	18,65	550,47	73,94
2019/05/18	CH2206	Male	Three y/o	Treatment (Wk 1)	Treatment	Fast Day	No	10:20	10:00 - 10:59	Late Morning	37,5	108	90,6	90,6	18,65	599,39	152,40
2019/05/18	CH2205	Male	Three y/o	Treatment (Wk 1)	Treatment	Fast Day	No	10:25	10:00 - 10:59	Late Morning	37,5	51	4,8	4,8	18,65	541,20	52,04
2019/05/18	CH2206	Male	Three y/o	Treatment (Wk 1)	Treatment	Fast Day	No	10:25	10:00 - 10:59	Late Morning	37,5	119	147,8	147,8	18,65	606,20	169,50
2019/05/18	CH2205	Male	Three y/o	Treatment (Wk 1)	Treatment	Fast Day	No	10:30	10:00 - 10:59	Late Morning	37,4	51	14,0	14,0	18,55	541,20	88,17
2019/05/18	CH2206	Male	Three y/o	Treatment (Wk 1)	Treatment	Fast Day	No	10:30	10:00 - 10:59	Late Morning	37,7	105	211,2	211,2	18,85	597,38	182,04
2019/05/18	CH2205	Male	Three y/o	Treatment (Wk 1)	Treatment	Fast Day	No	10:35	10:00 - 10:59	Late Morning	37,4	65	9,0	9,0	18,55	561,12	73,19
2019/05/18	CH2206	Male	Three y/o	Treatment (Wk 1)	Treatment	Fast Day	No	10:35	10:00 - 10:59	Late Morning	37,5	77	21,2	21,2	18,65	574,39	102,31
2019/05/18	CH2205	Male	Three y/o	Treatment (Wk 1)	Treatment	Fast Day	No	10:40	10:00 - 10:59	Late Morning	37,4	50	3,8	3,8	18,55	539,52	44,22
2019/05/18	CH2206	Male	Three y/o	Treatment (Wk 1)	Treatment	Fast Day	No	10:40	10:00 - 10:59	Late Morning	37,5	73	31,8	31,8	18,65	570,27	116,20
2019/05/18	CH2205	Male	Three y/o	Treatment (Wk 1)	Treatment	Fast Day	No	10:45	10:00 - 10:59	Late Morning	37,4	57	5,0	5,0	18,55	550,47	53,41
2019/05/18	CH2206	Male	Three y/o	Treatment (Wk 1)	Treatment	Fast Day	No	10:45	10:00 - 10:59	Late Morning	37,5	57	35,4	35,4	18,65	550,47	119,88
2019/05/18	CH2205	Male	Three y/o	Treatment (Wk 1)	Treatment	Fast Day	No	10:50	10:00 - 10:59	Late Morning	37,4		24,8	24,8	18,55		107,67
2019/05/18	CH2206	Male	Three y/o	Treatment (Wk 1)	Treatment	Fast Day	No	10:50	10:00 - 10:59	Late Morning	37,5	53	32,0	32,0	18,65	544,43	116,41
2019/05/18	CH2205	Male	Three y/o	Treatment (Wk 1)	Treatment	Fast Day	No	10:55	10:00 - 10:59	Late Morning	37,4	43	32,8	32,8	18,55	526,50	117,26
2019/05/18	CH2206	Male	Three y/o	Treatment (Wk 1)	Treatment	Fast Day	No	10:55	10:00 - 10:59	Late Morning	37,5	82	24,0	24,0	18,65	579,19	106,55
2019/05/18	CH2205	Male	Three y/o	Treatment (Wk 1)	Treatment	Fast Day	No	11:00	11:00 - 11:59	Late Morning	37,4		14,4	14,4	18,55		89,12
2019/05/18	CH2206	Male	Three y/o	Treatment (Wk 1)	Treatment	Fast Day	No	11:00	11:00 - 11:59	Late Morning	37,7		197,4	197,4	18,85		179,66
2019/05/18	CH2205	Male	Three y/o	Treatment (Wk 1)	Treatment	Fast Day	No	11:05	11:00 - 11:59	Late Morning	37,4	32	43,6	43,6	18,55	499,63	127,06
2019/05/18	CH2206	Male	Three y/o	Treatment (Wk 1)	Treatment	Fast Day	No	11:05	11:00 - 11:59	Late Morning	37,7	111	87,8	87,8	18,85	601,33	151,31
2019/05/18	CH2205	Male	Three y/o	Treatment (Wk 1)	Treatment	Fast Day	No	11:10	11:00 - 11:59	Late Morning	37,3	46	125,2	125,2	18,46	532,38	163,69
2019/05/18	CH2206	Male	Three y/o	Treatment (Wk 1)	Treatment	Fast Day	No	11:10	11:00 - 11:59	Late Morning	37,7	103	150,0	150,0	18,85	596,00	170,02
2019/05/18	CH2205	Male	Three y/o	Treatment (Wk 1)	Treatment	Fast Day	No	11:15	11:00 - 11:59	Late Morning	37,4	77	66,2	66,2	18,55	574,39	141,50
2019/05/18	CH2206	Male	Three y/o	Treatment (Wk 1)	Treatment	Fast Day	No	11:15	11:00 - 11:59	Late Morning	37,7	97	87,4	87,4	18,85	591,66	151,15
2019/05/18	CH2205	Male	Three y/o	Treatment (Wk 1)	Treatment	Fast Day	No	11:20	11:00 - 11:59	Late Morning	37,4		34,0	34,0	18,55		118,49
2019/05/18	CH2206	Male	Three y/o	Treatment (Wk 1)	Treatment	Fast Day	No	11:20	11:00 - 11:59	Late Morning	37,5	113	223,4	223,4	18,65	602,58	184,01
2019/05/18	CH2205	Male	Three y/o	Treatment (Wk 1)	Treatment	Fast Day	No	11:25	11:00 - 11:59	Late Morning	37,6		74,6	74,6	18,75		145,65
2019/05/18	CH2206	Male	Three y/o	Treatment (Wk 1)	Treatment	Fast Day	No	11:25	11:00 - 11:59	Late Morning	37,6		174,2	174,2	18,75		175,26
2019/05/18	CH2205	Male	Three y/o	Treatment (Wk 1)	Treatment	Fast Day	No	11:30	11:00 - 11:59	Late Morning	37,9	107	100,2	100,2	19,05	598,73	155,91
2019/05/18	CH2206	Male	Three y/o	Treatment (Wk 1)	Treatment	Fast Day	No	11:30	11:00 - 11:59	Late Morning	37,6	126	153,0	153,0	18,75	610,14	170,71
2019/05/18	CH2205	Male	Three y/o	Treatment (Wk 1)	Treatment	Fast Day	No	11:35	11:00 - 11:59	Late Morning	38,0	98	63,2	63,2	19,15	592,41	139,89
2019/05/18	CH2206	Male	Three y/o	Treatment (Wk 1)	Treatment	Fast Day	No	11:35	11:00 - 11:59	Late Morning	37,7	131	80,8	80,8	18,85	612,80	148,42
2019/05/18	CH2205	Male	Three y/o	Treatment (Wk 1)	Treatment	Fast Day	No	11:40	11:00 - 11:59	Late Morning	38,0	84	30,0	30,0	19,15	581,01	114,20
2019/05/18	CH2206	Male	Three y/o	Treatment (Wk 1)	Treatment	Fast Day	No	11:40	11:00 - 11:59	Late Morning	37,7	119	124,8	124,8	18,85	606,20	163,58
2019/05/18	CH2205	Male	Three y/o	Treatment (Wk 1)	Treatment	Fast Day	No	11:45	11:00 - 11:59	Late Morning	38,0	74	23,4	23,4	19,15	571,33	105,68
2019/05/18	CH2206	Male	Three y/o	Treatment (Wk 1)	Treatment	Fast Day	No	11:45	11:00 - 11:59	Late Morning	37,7		38,8	38,8	18,85		123,04
2019/05/18	CH2205	Male	Three y/o	Treatment (Wk 1)	Treatment	Fast Day	No	11:50	11:00 - 11:59	Late Morning	37,9	56	30,4	30,4	19,05	549,01	114,65
2019/05/18	CH2206	Male	Three y/o	Treatment (Wk 1)	Treatment	Fast Day	No	11:50	11:00 - 11:59	Late Morning	37,8	51	226,6	226,6	18,95	541,20	184,52
2019/05/18	CH2205	Male	Three y/o	Treatment (Wk 1)	Treatment	Fast Day	No	11:55	11:00 - 11:59	Late Morning	38,0		91,4	91,4	19,15		152,71
2019/05/18	CH2206	Male	Three y/o	Treatment (Wk 1)	Treatment	Fast Day	No	11:55	11:00 - 11:59	Late Morning	37,9	99	43,8	43,8	19,05	593,14	127,22
2019/05/18	CH2205	Male	Three y/o	Treatment (Wk 1)	Treatment	Fast Day	No	12:00	12:00 - 12:59	Afternoon	38,0	70	15,8	15,8	19,15	566,99	92,28
2019/05/18	CH2206	Male	Three y/o	Treatment (Wk 1)	Treatment	Fast Day	No	12:00	12:00 - 12:59	Afternoon	37,9	84	117,6	117,6	19,05	581,01	161,50
2019/05/18	CH2205	Male	Three y/o	Treatment (Wk 1)	Treatment	Fast Day	No	12:05	12:00 - 12:59	Afternoon	37,9		18,2	18,2	19,05		97,10
2019/05/18	CH2206	Male	Three y/o	Treatment (Wk 1)	Treatment	Fast Day	No	12:05	12:00 - 12:59	Afternoon	37,9	109	12,2	12,2	19,05	600,04	83,49
2019/05/18	CH2205	Male	Three y/o	Treatment (Wk 1)	Treatment	Fast Day	No	12:10	12:00 - 12:59	Afternoon	37,7	59	28,0	28,0	18,85	553,30	111,83
2019/05/18	CH2206	Male	Three y/o	Treatment (Wk 1)	Treatment	Fast Day	No	12:10	12:00 - 12:59	Afternoon	37,9	59	9,4	9,4	19,05	553,30	74,66
2019/05/18	CH2205	Male	Three y/o	Treatment (Wk 1)	Treatment	Fast Day	No	12:15	12:00 - 12:59	Afternoon	37,9	131	68,2	68,2	19,05	612,80	142,53
2019/05/18	CH2206	Male	Three y/o	Treatment (Wk 1)	Treatment	Fast Day	No	12:15	12:00 - 12:59	Afternoon	37,8	65	42,4	42,4	18,95	561,12	126,10
2019/05/18	CH2205	Male	Three y/o	Treatment (Wk 1)	Treatment	Fast Day	No	12:20	12:00 - 12:59	Afternoon	38,0		190,8	190,8	19,15		178,46
2019/05/18	CH2206	Male	Three y/o	Treatment (Wk 1)	Treatment	Fast Day	No	12:20	12:00 - 12:59	Afternoon	37,8	67	41,0	41,0	18,95	563,53	124,94
2019/05/18	CH2205	Male	Three y/o	Treatment (Wk 1)	Treatment	Fast Day	No	12:25	12:00 - 12:59	Afternoon	37,9		29,4	29,4	19,05		113,50

2019/05/18	CH2206	Male	Three y/o	Treatment (Wk 1)	Treatment	Fast Day	No	12:25	12:00 - 12:59	Afternoon	37,8	52	38,4	38,4	18,95	542,83	122,68
2019/05/18	CH2205	Male	Three y/o	Treatment (Wk 1)	Treatment	Fast Day	No	12:30	12:00 - 12:59	Afternoon	37,8	48	1,8	1,8	18,95	536,04	19,38
2019/05/18	CH2206	Male	Three y/o	Treatment (Wk 1)	Treatment	Fast Day	No	12:30	12:00 - 12:59	Afternoon	37,8	68	37,2	37,2	18,95	564,71	121,59
2019/05/18	CH2205	Male	Three y/o	Treatment (Wk 1)	Treatment	Fast Day	No	12:35	12:00 - 12:59	Afternoon	37,9	65	11,0	11,0	19,05	561,12	79,98
2019/05/18	CH2206	Male	Three y/o	Treatment (Wk 1)	Treatment	Fast Day	No	12:35	12:00 - 12:59	Afternoon	37,8	46	30,4	30,4	18,95	532,38	114,65
2019/05/18	CH2205	Male	Three y/o	Treatment (Wk 1)	Treatment	Fast Day	No	12:40	12:00 - 12:59	Afternoon	37,9	92	95,2	95,2	19,05	587,78	154,13
2019/05/18	CH2206	Male	Three y/o	Treatment (Wk 1)	Treatment	Fast Day	No	12:40	12:00 - 12:59	Afternoon	37,8	48	42,6	42,6	18,95	536,04	126,26
2019/05/18	CH2205	Male	Three y/o	Treatment (Wk 1)	Treatment	Fast Day	No	12:45	12:00 - 12:59	Afternoon	37,9	60	18,4	18,4	19,05	554,67	97,47
2019/05/18	CH2206	Male	Three y/o	Treatment (Wk 1)	Treatment	Fast Day	No	12:45	12:00 - 12:59	Afternoon	37,7	40	36,2	36,2	18,85	520,09	120,65
2019/05/18	CH2205	Male	Three y/o	Treatment (Wk 1)	Treatment	Fast Day	No	12:50	12:00 - 12:59	Afternoon	37,9	55	19,2	19,2	19,05	547,52	98,92
2019/05/18	CH2206	Male	Three y/o	Treatment (Wk 1)	Treatment	Fast Day	No	12:50	12:00 - 12:59	Afternoon	37,7	54	46,2	46,2	18,85	545,99	129,06
2019/05/18	CH2205	Male	Three y/o	Treatment (Wk 1)	Treatment	Fast Day	No	12:55	12:00 - 12:59	Afternoon	37,9	58	24,2	24,2	19,05	551,90	106,83
2019/05/18	CH2206	Male	Three y/o	Treatment (Wk 1)	Treatment	Fast Day	No	12:55	12:00 - 12:59	Afternoon	37,7	50	53,4	53,4	18,85	539,52	134,06
2019/05/18	CH2205	Male	Three y/o	Treatment (Wk 1)	Treatment	Fast Day	No	13:00	13:00 - 13:59	Afternoon	37,9	52	24,4	24,4	19,05	542,83	107,11
2019/05/18	CH2206	Male	Three y/o	Treatment (Wk 1)	Treatment	Fast Day	No	13:00	13:00 - 13:59	Afternoon	37,7	60	36,8	36,8	18,85	554,67	121,22
2019/05/18	CH2205	Male	Three y/o	Treatment (Wk 1)	Treatment	Fast Day	No	13:05	13:00 - 13:59	Afternoon	37,7	51	60,6	60,6	18,85	541,20	138,44
2019/05/18	CH2206	Male	Three y/o	Treatment (Wk 1)	Treatment	Fast Day	No	13:05	13:00 - 13:59	Afternoon	37,7	44	36,6	36,6	18,85	528,51	121,03
2019/05/18	CH2205	Male	Three y/o	Treatment (Wk 1)	Treatment	Fast Day	No	13:10	13:00 - 13:59	Afternoon	37,7		115,2	115,2	18,85		160,78
2019/05/18	CH2206	Male	Three y/o	Treatment (Wk 1)	Treatment	Fast Day	No	13:10	13:00 - 13:59	Afternoon	37,5	68	34,4	34,4	18,65	564,71	118,90
2019/05/18	CH2205	Male	Three y/o	Treatment (Wk 1)	Treatment	Fast Day	No	13:15	13:00 - 13:59	Afternoon	37,6	141	169,6	169,6	18,75	617,75	174,32
2019/05/18	CH2206	Male	Three y/o	Treatment (Wk 1)	Treatment	Fast Day	No	13:15	13:00 - 13:59	Afternoon	37,5		108,8	108,8	18,65		158,79
2019/05/18	CH2205	Male	Three y/o	Treatment (Wk 1)	Treatment	Fast Day	No	13:20	13:00 - 13:59	Afternoon	37,6	106	128,6	128,6	18,75	598,06	164,63
2019/05/18	CH2206	Male	Three y/o	Treatment (Wk 1)	Treatment	Fast Day	No	13:20	13:00 - 13:59	Afternoon	37,5	97	52,8	52,8	18,65	591,66	133,67
2019/05/18	CH2205	Male	Three y/o	Treatment (Wk 1)	Treatment	Fast Day	No	13:25	13:00 - 13:59	Afternoon	37,6	88	14,0	14,0	18,75	584,49	88,17
2019/05/18	CH2206	Male	Three y/o	Treatment (Wk 1)	Treatment	Fast Day	No	13:25	13:00 - 13:59	Afternoon	37,4	68	26,6	26,6	18,55	564,71	110,07
2019/05/18	CH2205	Male	Three y/o	Treatment (Wk 1)	Treatment	Fast Day	No	13:30	13:00 - 13:59	Afternoon	37,5	62	21,0	21,0	18,65	557,33	101,98
2019/05/18	CH2206	Male	Three y/o	Treatment (Wk 1)	Treatment	Fast Day	No	13:30	13:00 - 13:59	Afternoon	37,3	51	19,4	19,4	18,46	541,20	99,28
2019/05/18	CH2205	Male	Three y/o	Treatment (Wk 1)	Treatment	Fast Day	No	13:35	13:00 - 13:59	Afternoon	37,6	108	159,0	159,0	18,75	599,39	172,06
2019/05/18	CH2206	Male	Three y/o	Treatment (Wk 1)	Treatment	Fast Day	No	13:35	13:00 - 13:59	Afternoon	37,2	54	17,6	17,6	18,36	545,99	95,95
2019/05/18	CH2205	Male	Three y/o	Treatment (Wk 1)	Treatment	Fast Day	No	13:40	13:00 - 13:59	Afternoon	37,7	93	131,0	131,0	18,85	588,58	165,27
2019/05/18	CH2206	Male	Three y/o	Treatment (Wk 1)	Treatment	Fast Day	No	13:40	13:00 - 13:59	Afternoon	37,2	47	38,8	38,8	18,36	534,23	123,04
2019/05/18	CH2205	Male	Three y/o	Treatment (Wk 1)	Treatment	Fast Day	No	13:45	13:00 - 13:59	Afternoon	37,7	110	98,0	98,0	18,85	600,69	155,14
2019/05/18	CH2206	Male	Three y/o	Treatment (Wk 1)	Treatment	Fast Day	No	13:45	13:00 - 13:59	Afternoon	37,3	96	54,2	54,2	18,46	590,91	134,57
2019/05/18	CH2205	Male	Three y/o	Treatment (Wk 1)	Treatment	Fast Day	No	13:50	13:00 - 13:59	Afternoon	37,7	96	76,6	76,6	18,85	590,91	146,57
2019/05/18	CH2206	Male	Three y/o	Treatment (Wk 1)	Treatment	Fast Day	No	13:50	13:00 - 13:59	Afternoon	37,3	108	303,6	303,6	18,46	599,39	194,84
2019/05/18	CH2205	Male	Three y/o	Treatment (Wk 1)	Treatment	Fast Day	No	13:55	13:00 - 13:59	Afternoon	37,9		60,0	60,0	19,05		138,09
2019/05/18	CH2206	Male	Three y/o	Treatment (Wk 1)	Treatment	Fast Day	No	13:55	13:00 - 13:59	Afternoon	37,4		125,8	125,8	18,55		163,86
2019/05/18	CH2205	Male	Three y/o	Treatment (Wk 1)	Treatment	Fast Day	No	14:00	14:00 - 14:59	Afternoon	37,9	99	52,2	52,2	19,05	593,14	133,27
2019/05/18	CH2206	Male	Three y/o	Treatment (Wk 1)	Treatment	Fast Day	No	14:00	14:00 - 14:59	Afternoon	37,4		80,2	80,2	18,55		148,16
2019/05/18	CH2205	Male	Three y/o	Treatment (Wk 1)	Treatment	Fast Day	No	14:05	14:00 - 14:59	Afternoon	37,9	137	587,0	587,0	19,05	615,83	218,26
2019/05/18	CH2206	Male	Three y/o	Treatment (Wk 1)	Treatment	Fast Day	No	14:05	14:00 - 14:59	Afternoon	37,4	103	70,2	70,2	18,55	596,00	143,54
2019/05/18	CH2205	Male	Three y/o	Treatment (Wk 1)	Treatment	Fast Day	No	14:10	14:00 - 14:59	Afternoon	37,9		109,6	109,6	19,05		159,04
2019/05/18	CH2206	Male	Three y/o	Treatment (Wk 1)	Treatment	Fast Day	No	14:10	14:00 - 14:59	Afternoon	37,5	115	31,8	31,8	18,65	603,82	116,20
2019/05/18	CH2205	Male	Three y/o	Treatment (Wk 1)	Treatment	Fast Day	No	14:15	14:00 - 14:59	Afternoon	37,9	73	179,2	179,2	19,05	570,27	176,26
2019/05/18	CH2206	Male	Three y/o	Treatment (Wk 1)	Treatment	Fast Day	No	14:15	14:00 - 14:59	Afternoon	37,6	95	134,8	134,8	18,75	590,14	166,28
2019/05/18	CH2205	Male	Three y/o	Treatment (Wk 1)	Treatment	Fast Day	No	14:20	14:00 - 14:59	Afternoon	38,0		70,0	70,0	19,15		143,44
2019/05/18	CH2206	Male	Three y/o	Treatment (Wk 1)	Treatment	Fast Day	No	14:20	14:00 - 14:59	Afternoon	37,6		329,8	329,8	18,75		197,77
2019/05/18	CH2205	Male	Three y/o	Treatment (Wk 1)	Treatment	Fast Day	No	14:25	14:00 - 14:59	Afternoon	38,0		139,4	139,4	19,15		167,45
2019/05/18	CH2206	Male	Three y/o	Treatment (Wk 1)	Treatment	Fast Day	No	14:25	14:00 - 14:59	Afternoon	37,7	138	105,6	105,6	18,85	616,31	157,74
2019/05/18	CH2205	Male	Three y/o	Treatment (Wk 1)	Treatment	Fast Day	No	14:30	14:00 - 14:59	Afternoon	38,0	137	120,6	120,6	19,15	615,83	162,38
2019/05/18	CH2206	Male	Three y/o	Treatment (Wk 1)	Treatment	Fast Day	No	14:30	14:00 - 14:59	Afternoon	37,8	91	107,8	107,8	18,95	586,98	158,46
2019/05/18	CH2205	Male	Three y/o	Treatment (Wk 1)	Treatment	Fast Day	No	14:35	14:00 - 14:59	Afternoon	38,1		149,6	149,6	19,26		169,92
2019/05/18	CH2206	Male	Three y/o	Treatment (Wk 1)	Treatment	Fast Day	No	14:35	14:00 - 14:59	Afternoon	37,8	56	148,2	148,2	18,95	549,01	169,59
2019/05/18	CH2205	Male	Three y/o	Treatment (Wk 1)	Treatment	Fast Day	No	14:40	14:00 - 14:59	Afternoon	38,1	58	14,4	14,4	19,26	551,90	89,12
2019/05/18	CH2206	Male	Three y/o	Treatment (Wk 1)	Treatment	Fast Day	No	14:40	14:00 - 14:59	Afternoon	37,8	61	55,2	55,2	18,95	556,01	135,21
2019/05/18	CH2205	Male	Three y/o	Treatment (Wk 1)	Treatment	Fast Day	No	14:45	14:00 - 14:59	Afternoon	38,1	98	45,6	45,6	19,26	592,41	128,61
2019/05/18	CH2206	Male	Three y/o	Treatment (Wk 1)	Treatment	Fast Day	No	14:45	14:00 - 14:59	Afternoon	37,8	46	8,8	8,8	18,95	532,38	72,43
2019/05/18	CH2205	Male	Three y/o	Treatment (Wk 1)	Treatment	Fast Day	No	14:50	14:00 - 14:59	Afternoon	38,1	113	145,0	145,0	19,26	602,58	168,83
2019/05/18	CH2206	Male	Three y/o	Treatment (Wk 1)	Treatment	Fast Day	No	14:50	14:00 - 14:59	Afternoon	37,8	50	12,6	12,6	18,95	539,52	84,59
2019/05/18	CH2205	Male	Three y/o	Treatment (Wk 1)	Treatment	Fast Day	No	14:55	14:00 - 14:59	Afternoon	38,1	148	52,6	52,6	19,26	620,97	133,54
2019/05/18	CH2206	Male	Three y/o	Treatment (Wk 1)	Treatment	Fast Day	No	14:55	14:00 - 14:59	Afternoon	37,8	57	4,2	4,2	18,95	550,47	47,57
2019/05/18	CH2205	Male	Three y/o	Treatment (Wk 1)	Treatment	Fast Day	No	15:00	15:00 - 15:59	Afternoon	38,0	40	30,4	30,4	19,15	520,09	114,65
2019/05/18	CH2206	Male	Three y/o	Treatment (Wk 1)	Treatment	Fast Day	No	15:00	15:00 - 15:59	Afternoon	37,9	47	4,6	4,6	19,05	534,23	50,61
2019/05/18	CH2205	Male	Three y/o	Treatment (Wk 1)	Treatment	Fast Day	No	15:05	15:00 - 15:59	Afternoon	38,0	68	117,8	117,8	19,15	564,71	161,56
2019/05/18	CH2206	Male	Three y/o	Treatment (Wk 1)	Treatment	Fast Day	No	15:05	15:00 - 15:59	Afternoon	37,8	43	117,8	117,8	18,95	526,50	161,56
2019/05/18	CH2205	Male	Three y/o	Treatment (Wk 1)	Treatment	Fast Day	No	15:10	15:00 - 15:59	Afternoon	38,1	99	232,0	232,0	19,26	593,14	185,35
2019/05/18	CH2206	Male	Three y/o	Treatment (Wk 1)	Treatment	Fast Day	No	15:10	15:00 - 15:59	Afternoon	37,9	121	236,6	236,6	19,05	607,35	186,04
2019/05/18	CH2205	Male	Three y/o	Treatment (Wk 1)	Treatment	Fast Day	No	15:15	15:00 - 15:59	Afternoon	38,1		530,8	530,8	19,26		214,68
2019/05/18	CH2206	Male	Three y/o	Treatment (Wk 1)	Treatment	Fast Day	No	15:15	15:00 - 15:59	Afternoon	37,9	150	267,6	267,6	19,05	621,86	190,38
2019/05/18	CH2205	Male	Three y/o	Treatment (Wk 1)	Treatment	Fast Day	No	15:20	15:00 - 15:59	Afternoon	38,3	180	16,0	16,0	19,46	633,61	92,71

2019/05/18	CH2206	Male	Three y/o	Treatment (Wk 1)	Treatment	Fast Day	No	15:20	15:00 - 15:59	Afternoon	38,0	148	32,8	32,8	19,15	620,97	117,26
2019/05/18	CH2205	Male	Three y/o	Treatment (Wk 1)	Treatment	Fast Day	No	15:25	15:00 - 15:59	Afternoon	38,3	64	12,8	12,8	19,46	559,88	85,12
2019/05/18	CH2206	Male	Three y/o	Treatment (Wk 1)	Treatment	Fast Day	No	15:25	15:00 - 15:59	Afternoon	38,1	75	56,0	56,0	19,26	572,36	135,70
2019/05/18	CH2205	Male	Three y/o	Treatment (Wk 1)	Treatment	Fast Day	No	15:30	15:00 - 15:59	Afternoon	38,3	61	12,2	12,2	19,46	556,01	83,49
2019/05/18	CH2206	Male	Three y/o	Treatment (Wk 1)	Treatment	Fast Day	No	15:30	15:00 - 15:59	Afternoon	38,0	66	106,4	106,4	19,15	562,34	158,01
2019/05/18	CH2205	Male	Three y/o	Treatment (Wk 1)	Treatment	Fast Day	No	15:35	15:00 - 15:59	Afternoon	38,4	51	21,6	21,6	19,56	541,20	102,94
2019/05/18	CH2206	Male	Three y/o	Treatment (Wk 1)	Treatment	Fast Day	No	15:35	15:00 - 15:59	Afternoon	38,0	92	61,6	61,6	19,15	587,78	139,00
2019/05/18	CH2205	Male	Three y/o	Treatment (Wk 1)	Treatment	Fast Day	No	15:40	15:00 - 15:59	Afternoon	38,3		14,2	14,2	19,46		88,65
2019/05/18	CH2206	Male	Three y/o	Treatment (Wk 1)	Treatment	Fast Day	No	15:40	15:00 - 15:59	Afternoon	38,1	96	159,4	159,4	19,26	590,91	172,15
2019/05/18	CH2205	Male	Three y/o	Treatment (Wk 1)	Treatment	Fast Day	No	15:45	15:00 - 15:59	Afternoon	38,2	106	85,0	85,0	19,36	598,06	150,18
2019/05/18	CH2206	Male	Three y/o	Treatment (Wk 1)	Treatment	Fast Day	No	15:45	15:00 - 15:59	Afternoon	38,0	114	133,8	133,8	19,15	603,20	166,01
2019/05/18	CH2205	Male	Three y/o	Treatment (Wk 1)	Treatment	Fast Day	No	15:50	15:00 - 15:59	Afternoon	38,1	85	108,8	108,8	19,26	581,90	158,79
2019/05/18	CH2206	Male	Three y/o	Treatment (Wk 1)	Treatment	Fast Day	No	15:50	15:00 - 15:59	Afternoon	38,1	125	264,6	264,6	19,26	609,60	189,98
2019/05/18	CH2205	Male	Three y/o	Treatment (Wk 1)	Treatment	Fast Day	No	15:55	15:00 - 15:59	Afternoon	38,2	94	445,8	445,8	19,36	589,37	208,46
2019/05/18	CH2206	Male	Three y/o	Treatment (Wk 1)	Treatment	Fast Day	No	15:55	15:00 - 15:59	Afternoon	38,1	98	398,2	398,2	19,26	592,41	204,45
2019/05/18	CH2205	Male	Three y/o	Treatment (Wk 1)	Treatment	Fast Day	No	16:00	16:00 - 16:59	Evening	38,1	56	15,2	15,2	19,26	549,01	90,96
2019/05/18	CH2206	Male	Three y/o	Treatment (Wk 1)	Treatment	Fast Day	No	16:00	16:00 - 16:59	Evening	38,1	105	126,6	126,6	19,26	597,38	164,08
2019/05/18	CH2205	Male	Three y/o	Treatment (Wk 1)	Treatment	Fast Day	No	16:05	16:00 - 16:59	Evening	38,1	65	13,4	13,4	19,26	561,12	86,68
2019/05/18	CH2206	Male	Three y/o	Treatment (Wk 1)	Treatment	Fast Day	No	16:05	16:00 - 16:59	Evening	38,1		253,8	253,8	19,26		188,51
2019/05/18	CH2205	Male	Three y/o	Treatment (Wk 1)	Treatment	Fast Day	No	16:10	16:00 - 16:59	Evening	38,2		11,6	11,6	19,36		81,78
2019/05/18	CH2206	Male	Three y/o	Treatment (Wk 1)	Treatment	Fast Day	No	16:10	16:00 - 16:59	Evening	38,2	109	113,6	113,6	19,36	600,04	160,29
2019/05/18	CH2205	Male	Three y/o	Treatment (Wk 1)	Treatment	Fast Day	No	16:15	16:00 - 16:59	Evening	38,2	49	8,0	8,0	19,36	537,80	69,22
2019/05/18	CH2206	Male	Three y/o	Treatment (Wk 1)	Treatment	Fast Day	No	16:15	16:00 - 16:59	Evening	38,1	111	164,6	164,6	19,26	601,33	173,27
2019/05/18	CH2205	Male	Three y/o	Treatment (Wk 1)	Treatment	Fast Day	No	16:20	16:00 - 16:59	Evening	38,2	62	2,6	2,6	19,36	557,33	31,57
2019/05/18	CH2206	Male	Three y/o	Treatment (Wk 1)	Treatment	Fast Day	No	16:20	16:00 - 16:59	Evening	38,2	94	256,2	256,2	19,36	589,37	188,84
2019/05/18	CH2205	Male	Three y/o	Treatment (Wk 1)	Treatment	Fast Day	No	16:25	16:00 - 16:59	Evening	38,1	47	4,0	4,0	19,26	534,23	45,94
2019/05/18	CH2206	Male	Three y/o	Treatment (Wk 1)	Treatment	Fast Day	No	16:25	16:00 - 16:59	Evening	38,2	94	58,2	58,2	19,36	589,37	137,04
2019/05/18	CH2205	Male	Three y/o	Treatment (Wk 1)	Treatment	Fast Day	No	16:30	16:00 - 16:59	Evening	38,1	53	6,0	6,0	19,26	544,43	59,53
2019/05/18	CH2206	Male	Three y/o	Treatment (Wk 1)	Treatment	Fast Day	No	16:30	16:00 - 16:59	Evening	38,1	97	68,8	68,8	19,26	591,66	142,84
2019/05/18	CH2205	Male	Three y/o	Treatment (Wk 1)	Treatment	Fast Day	No	16:35	16:00 - 16:59	Evening	38,1	45	1,6	1,6	19,26	530,47	15,48
2019/05/18	CH2206	Male	Three y/o	Treatment (Wk 1)	Treatment	Fast Day	No	16:35	16:00 - 16:59	Evening	38,1	54	28,0	28,0	19,26	545,99	111,83
2019/05/18	CH2205	Male	Three y/o	Treatment (Wk 1)	Treatment	Fast Day	No	16:40	16:00 - 16:59	Evening	38,0	51	10,4	10,4	19,15	541,20	78,08
2019/05/18	CH2206	Male	Three y/o	Treatment (Wk 1)	Treatment	Fast Day	No	16:40	16:00 - 16:59	Evening	38,1	52	24,6	24,6	19,26	542,83	107,39
2019/05/18	CH2205	Male	Three y/o	Treatment (Wk 1)	Treatment	Fast Day	No	16:45	16:00 - 16:59	Evening	38,1	54	6,0	6,0	19,26	545,99	59,53
2019/05/18	CH2206	Male	Three y/o	Treatment (Wk 1)	Treatment	Fast Day	No	16:45	16:00 - 16:59	Evening	38,1	60	27,8	27,8	19,26	554,67	111,58
2019/05/18	CH2205	Male	Three y/o	Treatment (Wk 1)	Treatment	Fast Day	No	16:50	16:00 - 16:59	Evening	38,0	67	12,0	12,0	19,15	563,53	82,93
2019/05/18	CH2206	Male	Three y/o	Treatment (Wk 1)	Treatment	Fast Day	No	16:50	16:00 - 16:59	Evening	38,1	48	24,2	24,2	19,26	536,04	106,83
2019/05/18	CH2205	Male	Three y/o	Treatment (Wk 1)	Treatment	Fast Day	No	16:55	16:00 - 16:59	Evening	38,0		6,2	6,2	19,15		60,63
2019/05/18	CH2206	Male	Three y/o	Treatment (Wk 1)	Treatment	Fast Day	No	16:55	16:00 - 16:59	Evening	38,0	57	254,6	254,6	19,15	550,47	188,62
2019/05/18	CH2205	Male	Three y/o	Treatment (Wk 1)	Treatment	Fast Day	No	17:00	17:00 - 17:59	Evening	37,9		188,8	188,8	19,05		178,09
2019/05/18	CH2206	Male	Three y/o	Treatment (Wk 1)	Treatment	Fast Day	No	17:00	17:00 - 17:59	Evening	38,1		188,0	188,0	19,26		177,94
2019/05/18	CH2205	Male	Three y/o	Treatment (Wk 1)	Treatment	Fast Day	No	17:05	17:00 - 17:59	Evening	37,9	45	69,4	69,4	19,05	530,47	143,14
2019/05/18	CH2206	Male	Three y/o	Treatment (Wk 1)	Treatment	Fast Day	No	17:05	17:00 - 17:59	Evening	38,1	105	422,6	422,6	19,26	597,38	206,57
2019/05/18	CH2205	Male	Three y/o	Treatment (Wk 1)	Treatment	Fast Day	No	17:10	17:00 - 17:59	Evening	37,9	63	34,8	34,8	19,05	558,62	119,29
2019/05/18	CH2206	Male	Three y/o	Treatment (Wk 1)	Treatment	Fast Day	No	17:10	17:00 - 17:59	Evening	38,1	97	56,6	56,6	19,26	591,66	136,07
2019/05/18	CH2205	Male	Three y/o	Treatment (Wk 1)	Treatment	Fast Day	No	17:15	17:00 - 17:59	Evening	37,9		51,2	51,2	19,05		132,61
2019/05/18	CH2206	Male	Three y/o	Treatment (Wk 1)	Treatment	Fast Day	No	17:15	17:00 - 17:59	Evening	38,1	123	183,2	183,2	19,26	608,49	177,03
2019/05/18	CH2205	Male	Three y/o	Treatment (Wk 1)	Treatment	Fast Day	No	17:20	17:00 - 17:59	Evening	37,9	80	141,6	141,6	19,05	577,31	168,00
2019/05/18	CH2206	Male	Three y/o	Treatment (Wk 1)	Treatment	Fast Day	No	17:20	17:00 - 17:59	Evening	38,2	118	116,8	116,8	19,36	605,61	161,26
2019/05/18	CH2205	Male	Three y/o	Treatment (Wk 1)	Treatment	Fast Day	No	17:25	17:00 - 17:59	Evening	37,9	158	72,0	72,0	19,05	625,26	144,42
2019/05/18	CH2206	Male	Three y/o	Treatment (Wk 1)	Treatment	Fast Day	No	17:25	17:00 - 17:59	Evening	38,2	103	338,2	338,2	19,36	596,00	198,66
2019/05/18	CH2205	Male	Three y/o	Treatment (Wk 1)	Treatment	Fast Day	No	17:30	17:00 - 17:59	Evening	37,9	70	26,0	26,0	19,05	566,99	109,29
2019/05/18	CH2206	Male	Three y/o	Treatment (Wk 1)	Treatment	Fast Day	No	17:30	17:00 - 17:59	Evening	38,2	132	112,2	112,2	19,36	613,32	159,86
2019/05/18	CH2205	Male	Three y/o	Treatment (Wk 1)	Treatment	Fast Day	No	17:35	17:00 - 17:59	Evening	38,0	98	73,4	73,4	19,15	592,41	145,08
2019/05/18	CH2206	Male	Three y/o	Treatment (Wk 1)	Treatment	Fast Day	No	17:35	17:00 - 17:59	Evening	38,2	101	131,4	131,4	19,36	594,59	165,38
2019/05/18	CH2205	Male	Three y/o	Treatment (Wk 1)	Treatment	Fast Day	No	17:40	17:00 - 17:59	Evening	38,0	172	925,2		19,15	630,73	
2019/05/18	CH2206	Male	Three y/o	Treatment (Wk 1)	Treatment	Fast Day	No	17:40	17:00 - 17:59	Evening	38,3	145	73,6	73,6	19,46	619,62	145,18
2019/05/18	CH2205	Male	Three y/o	Treatment (Wk 1)	Treatment	Fast Day	No	17:45	17:00 - 17:59	Evening	38,0	76	67,2	67,2	19,15	573,39	142,02
2019/05/18	CH2206	Male	Three y/o	Treatment (Wk 1)	Treatment	Fast Day	No	17:45	17:00 - 17:59	Evening	38,3	108	106,6	106,6	19,46	599,39	158,07
2019/05/18	CH2205	Male	Three y/o	Treatment (Wk 1)	Treatment	Fast Day	No	17:50	17:00 - 17:59	Evening	38,0		28,6	28,6	19,15		112,56
2019/05/18	CH2206	Male	Three y/o	Treatment (Wk 1)	Treatment	Fast Day	No	17:50	17:00 - 17:59	Evening	38,2	102	64,0	64,0	19,36	595,30	140,33
2019/05/18	CH2205	Male	Three y/o	Treatment (Wk 1)	Treatment	Fast Day	No	17:55	17:00 - 17:59	Evening	38,1		30,4	30,4	19,26		114,65
2019/05/18	CH2206	Male	Three y/o	Treatment (Wk 1)	Treatment	Fast Day	No	17:55	17:00 - 17:59	Evening	38,1	84	56,0	56,0	19,26	581,01	135,70
2019/05/18	CH2205	Male	Three y/o	Treatment (Wk 1)	Treatment	Fast Day	No	18:00	18:00 - 18:59	Evening	38,1	84	34,4	34,4	19,26	581,01	118,90
2019/05/18	CH2206	Male	Three y/o	Treatment (Wk 1)	Treatment	Fast Day	No	18:00	18:00 - 18:59	Evening	38,0	63	56,0	56,0	19,15	558,62	135,70
2019/05/18	CH2205	Male	Three y/o	Treatment (Wk 1)	Treatment	Fast Day	No	18:05	18:00 - 18:59	Evening	38,1	50	36,2	36,2	19,26	539,52	120,65
2019/05/18	CH2206	Male	Three y/o	Treatment (Wk 1)	Treatment	Fast Day	No	18:05	18:00 - 18:59	Evening	37,9	90	65,0	65,0	19,05	586,16	140,87
2019/05/18	CH2205	Male	Three y/o	Treatment (Wk 1)	Treatment	Fast Day	No	18:10	18:00 - 18:59	Evening	38,0		31,6	31,6	19,15		115,98
2019/05/18	CH2206	Male	Three y/o	Treatment (Wk 1)	Treatment	Fast Day	No	18:10	18:00 - 18:59	Evening	37,8	49	47,0	47,0	18,95	537,80	129,65
2019/05/18	CH2205	Male	Three y/o	Treatment (Wk 1)	Treatment	Fast Day	No	18:15	18:00 - 18:59	Evening	38,0		26,8	26,8	19,15		110,33

2019/05/18	CH2206	Male	Three y/o	Treatment (Wk 1)	Treatment	Fast Day	No	18:15	18:00 - 18:59	Evening	37,7	60	47,0	47,0	18,85	554,67	129,65
2019/05/18	CH2205	Male	Three y/o	Treatment (Wk 1)	Treatment	Fast Day	No	18:20	18:00 - 18:59	Evening	38,0	65	12,2	12,2	19,15	561,12	83,49
2019/05/18	CH2206	Male	Three y/o	Treatment (Wk 1)	Treatment	Fast Day	No	18:20	18:00 - 18:59	Evening	37,5	47	55,6	55,6	18,65	534,23	135,46
2019/05/18	CH2205	Male	Three y/o	Treatment (Wk 1)	Treatment	Fast Day	No	18:25	18:00 - 18:59	Evening	38,0	47	16,6	16,6	19,15	534,23	93,96
2019/05/18	CH2206	Male	Three y/o	Treatment (Wk 1)	Treatment	Fast Day	No	18:25	18:00 - 18:59	Evening	37,5	47	60,6	60,6	18,65	534,23	138,44
2019/05/18	CH2205	Male	Three y/o	Treatment (Wk 1)	Treatment	Fast Day	No	18:30	18:00 - 18:59	Evening	37,9	39	7,0	7,0	19,05	517,82	64,72
2019/05/18	CH2206	Male	Three y/o	Treatment (Wk 1)	Treatment	Fast Day	No	18:30	18:00 - 18:59	Evening	37,5	46	59,8	59,8	18,65	532,38	137,98
2019/05/18	CH2205	Male	Three y/o	Treatment (Wk 1)	Treatment	Fast Day	No	18:35	18:00 - 18:59	Evening	37,9	39	13,4	13,4	19,05	517,82	86,68
2019/05/18	CH2206	Male	Three y/o	Treatment (Wk 1)	Treatment	Fast Day	No	18:35	18:00 - 18:59	Evening	37,7	50	58,4	58,4	18,85	539,52	137,16
2019/05/18	CH2205	Male	Three y/o	Treatment (Wk 1)	Treatment	Fast Day	No	18:40	18:00 - 18:59	Evening	37,9	49	16,0	16,0	19,05	537,80	92,71
2019/05/18	CH2206	Male	Three y/o	Treatment (Wk 1)	Treatment	Fast Day	No	18:40	18:00 - 18:59	Evening	37,7	59	67,2	67,2	18,85	553,30	142,02
2019/05/18	CH2205	Male	Three y/o	Treatment (Wk 1)	Treatment	Fast Day	No	18:45	18:00 - 18:59	Evening	37,7	48	23,2	23,2	18,85	536,04	105,39
2019/05/18	CH2206	Male	Three y/o	Treatment (Wk 1)	Treatment	Fast Day	No	18:45	18:00 - 18:59	Evening	37,7	45	52,8	52,8	18,85	530,47	133,67
2019/05/18	CH2205	Male	Three y/o	Treatment (Wk 1)	Treatment	Fast Day	No	18:50	18:00 - 18:59	Evening	37,6	75	20,4	20,4	18,75	572,36	100,99
2019/05/18	CH2206	Male	Three y/o	Treatment (Wk 1)	Treatment	Fast Day	No	18:50	18:00 - 18:59	Evening	37,5	47	58,4	58,4	18,65	534,23	137,16
2019/05/18	CH2205	Male	Three y/o	Treatment (Wk 1)	Treatment	Fast Day	No	18:55	18:00 - 18:59	Evening	37,6	44	20,6	20,6	18,75	528,51	101,32
2019/05/18	CH2206	Male	Three y/o	Treatment (Wk 1)	Treatment	Fast Day	No	18:55	18:00 - 18:59	Evening	37,4	45	59,0	59,0	18,55	530,47	137,51
2019/05/18	CH2205	Male	Three y/o	Treatment (Wk 1)	Treatment	Fast Day	No	19:00	19:00 - 19:59	Evening	37,5	41	16,8	16,8	18,65	522,29	94,37
2019/05/18	CH2206	Male	Three y/o	Treatment (Wk 1)	Treatment	Fast Day	No	19:00	19:00 - 19:59	Evening	37,4	72	58,4	58,4	18,55	569,20	137,16
2019/05/18	CH2205	Male	Three y/o	Treatment (Wk 1)	Treatment	Fast Day	No	19:05	19:00 - 19:59	Evening	37,5	50	13,4	13,4	18,65	539,52	86,68
2019/05/18	CH2206	Male	Three y/o	Treatment (Wk 1)	Treatment	Fast Day	No	19:05	19:00 - 19:59	Evening	37,3	68	59,8	59,8	18,46	564,71	137,98
2019/05/18	CH2205	Male	Three y/o	Treatment (Wk 1)	Treatment	Fast Day	No	19:10	19:00 - 19:59	Evening	37,5	73	19,0	19,0	18,65	570,27	98,56
2019/05/18	CH2206	Male	Three y/o	Treatment (Wk 1)	Treatment	Fast Day	No	19:10	19:00 - 19:59	Evening	37,3	44	57,6	57,6	18,46	528,51	136,68
2019/05/18	CH2205	Male	Three y/o	Treatment (Wk 1)	Treatment	Fast Day	No	19:15	19:00 - 19:59	Evening	37,5	51	24,8	24,8	18,65	541,20	107,67
2019/05/18	CH2206	Male	Three y/o	Treatment (Wk 1)	Treatment	Fast Day	No	19:15	19:00 - 19:59	Evening	37,2	48	63,4	63,4	18,36	536,04	140,00
2019/05/18	CH2205	Male	Three y/o	Treatment (Wk 1)	Treatment	Fast Day	No	19:20	19:00 - 19:59	Evening	37,5		24,6	24,6	18,65		107,39
2019/05/18	CH2206	Male	Three y/o	Treatment (Wk 1)	Treatment	Fast Day	No	19:20	19:00 - 19:59	Evening	37,2	94	57,8	57,8	18,36	589,37	136,80
2019/05/18	CH2205	Male	Three y/o	Treatment (Wk 1)	Treatment	Fast Day	No	19:25	19:00 - 19:59	Evening	37,7		22,0	22,0	18,85		103,57
2019/05/18	CH2206	Male	Three y/o	Treatment (Wk 1)	Treatment	Fast Day	No	19:25	19:00 - 19:59	Evening	37,2	47	56,2	56,2	18,36	534,23	135,83
2019/05/18	CH2205	Male	Three y/o	Treatment (Wk 1)	Treatment	Fast Day	No	19:30	19:00 - 19:59	Evening	37,6		24,4	24,4	18,75		107,11
2019/05/18	CH2206	Male	Three y/o	Treatment (Wk 1)	Treatment	Fast Day	No	19:30	19:00 - 19:59	Evening	37,1	49	56,6	56,6	18,26	537,80	136,07
2019/05/18	CH2205	Male	Three y/o	Treatment (Wk 1)	Treatment	Fast Day	No	19:35	19:00 - 19:59	Evening	37,6		22,0	22,0	18,75		103,57
2019/05/18	CH2206	Male	Three y/o	Treatment (Wk 1)	Treatment	Fast Day	No	19:35	19:00 - 19:59	Evening	37,1	51	63,0	63,0	18,26	541,20	139,78
2019/05/18	CH2205	Male	Three y/o	Treatment (Wk 1)	Treatment	Fast Day	No	19:40	19:00 - 19:59	Evening	37,6		23,2	23,2	18,75		105,39
2019/05/18	CH2206	Male	Three y/o	Treatment (Wk 1)	Treatment	Fast Day	No	19:40	19:00 - 19:59	Evening	37,0	46	51,8	51,8	18,16	532,38	133,01
2019/05/18	CH2205	Male	Three y/o	Treatment (Wk 1)	Treatment	Fast Day	No	19:45	19:00 - 19:59	Evening	37,6		27,8	27,8	18,75		111,58
2019/05/18	CH2206	Male	Three y/o	Treatment (Wk 1)	Treatment	Fast Day	No	19:45	19:00 - 19:59	Evening	37,0	48	18,2	18,2	18,16	536,04	97,10
2019/05/18	CH2205	Male	Three y/o	Treatment (Wk 1)	Treatment	Fast Day	No	19:50	19:00 - 19:59	Evening	37,5		25,2	25,2	18,65		108,22
2019/05/18	CH2206	Male	Three y/o	Treatment (Wk 1)	Treatment	Fast Day	No	19:50	19:00 - 19:59	Evening	37,0	40	11,2	11,2	18,16	520,09	80,59
2019/05/18	CH2205	Male	Three y/o	Treatment (Wk 1)	Treatment	Fast Day	No	19:55	19:00 - 19:59	Evening	37,5		27,0	27,0	18,65		110,58
2019/05/18	CH2206	Male	Three y/o	Treatment (Wk 1)	Treatment	Fast Day	No	19:55	19:00 - 19:59	Evening	37,1	43	13,6	13,6	18,26	526,50	87,18
2019/05/18	CH2205	Male	Three y/o	Treatment (Wk 1)	Treatment	Fast Day	No	20:00	20:00 - 20:59	Night	37,5		20,4	20,4	18,65		100,99
2019/05/18	CH2206	Male	Three y/o	Treatment (Wk 1)	Treatment	Fast Day	No	20:00	20:00 - 20:59	Night	37,3	48	17,4	17,4	18,46	536,04	95,56
2019/05/18	CH2205	Male	Three y/o	Treatment (Wk 1)	Treatment	Fast Day	No	20:05	20:00 - 20:59	Night	37,5		23,8	23,8	18,65		106,26
2019/05/18	CH2206	Male	Three y/o	Treatment (Wk 1)	Treatment	Fast Day	No	20:05	20:00 - 20:59	Night	37,3	63	21,4	21,4	18,46	558,62	102,63
2019/05/18	CH2205	Male	Three y/o	Treatment (Wk 1)	Treatment	Fast Day	No	20:10	20:00 - 20:59	Night	37,4	90	28,4	28,4	18,55	586,16	112,31
2019/05/18	CH2206	Male	Three y/o	Treatment (Wk 1)	Treatment	Fast Day	No	20:10	20:00 - 20:59	Night	37,3	42	16,6	16,6	18,46	524,42	93,96
2019/05/18	CH2205	Male	Three y/o	Treatment (Wk 1)	Treatment	Fast Day	No	20:15	20:00 - 20:59	Night	37,5		60,6	60,6	18,65		138,44
2019/05/18	CH2206	Male	Three y/o	Treatment (Wk 1)	Treatment	Fast Day	No	20:15	20:00 - 20:59	Night	37,3	55	13,2	13,2	18,46	547,52	86,17
2019/05/18	CH2205	Male	Three y/o	Treatment (Wk 1)	Treatment	Fast Day	No	20:20	20:00 - 20:59	Night	37,5		15,2	15,2	18,65		90,96
2019/05/18	CH2206	Male	Three y/o	Treatment (Wk 1)	Treatment	Fast Day	No	20:20	20:00 - 20:59	Night	37,4	60	25,8	25,8	18,55	554,67	109,02
2019/05/18	CH2205	Male	Three y/o	Treatment (Wk 1)	Treatment	Fast Day	No	20:25	20:00 - 20:59	Night	37,4	83	23,0	23,0	18,55	580,11	105,09
2019/05/18	CH2206	Male	Three y/o	Treatment (Wk 1)	Treatment	Fast Day	No	20:25	20:00 - 20:59	Night	37,4	67	53,8	53,8	18,55	563,53	134,32
2019/05/18	CH2205	Male	Three y/o	Treatment (Wk 1)	Treatment	Fast Day	No	20:30	20:00 - 20:59	Night	37,2	78	20,0	20,0	18,36	575,38	100,31
2019/05/18	CH2206	Male	Three y/o	Treatment (Wk 1)	Treatment	Fast Day	No	20:30	20:00 - 20:59	Night	37,4	48	58,6	58,6	18,55	536,04	137,28
2019/05/18	CH2205	Male	Three y/o	Treatment (Wk 1)	Treatment	Fast Day	No	20:35	20:00 - 20:59	Night	37,2	53	23,4	23,4	18,36	544,43	105,68
2019/05/18	CH2206	Male	Three y/o	Treatment (Wk 1)	Treatment	Fast Day	No	20:35	20:00 - 20:59	Night	37,4	50	60,2	60,2	18,55	539,52	138,21
2019/05/18	CH2205	Male	Three y/o	Treatment (Wk 1)	Treatment	Fast Day	No	20:40	20:00 - 20:59	Night	37,2	43	22,4	22,4	18,36	526,50	104,19
2019/05/18	CH2206	Male	Three y/o	Treatment (Wk 1)	Treatment	Fast Day	No	20:40	20:00 - 20:59	Night	37,4	43	55,8	55,8	18,55	526,50	135,58
2019/05/18	CH2205	Male	Three y/o	Treatment (Wk 1)	Treatment	Fast Day	No	20:45	20:00 - 20:59	Night	37,2	35	21,4	21,4	18,36	507,97	102,63
2019/05/18	CH2206	Male	Three y/o	Treatment (Wk 1)	Treatment	Fast Day	No	20:45	20:00 - 20:59	Night	37,4	40	52,4	52,4	18,55	520,09	133,41
2019/05/18	CH2205	Male	Three y/o	Treatment (Wk 1)	Treatment	Fast Day	No	20:50	20:00 - 20:59	Night	37,2	43	21,4	21,4	18,36	526,50	102,63
2019/05/18	CH2206	Male	Three y/o	Treatment (Wk 1)	Treatment	Fast Day	No	20:50	20:00 - 20:59	Night	37,4	67	28,4	28,4	18,55	563,53	112,31
2019/05/18	CH2205	Male	Three y/o	Treatment (Wk 1)	Treatment	Fast Day	No	20:55	20:00 - 20:59	Night	37,4	49	18,6	18,6	18,55	537,80	97,84
2019/05/18	CH2206	Male	Three y/o	Treatment (Wk 1)	Treatment	Fast Day	No	20:55	20:00 - 20:59	Night	37,3	47	44,8	44,8	18,46	534,23	127,99
2019/05/18	CH2205	Male	Three y/o	Treatment (Wk 1)	Treatment	Fast Day	No	21:00	21:00 - 21:59	Night	37,4	49	20,2	20,2	18,55	537,80	100,65
2019/05/18	CH2206	Male	Three y/o	Treatment (Wk 1)	Treatment	Fast Day	No	21:00	21:00 - 21:59	Night	37,3	44	43,0	43,0	18,46	528,51	126,58
2019/05/18	CH2205	Male	Three y/o	Treatment (Wk 1)	Treatment	Fast Day	No	21:05	21:00 - 21:59	Night	37,4		27,6	27,6	18,55		111,33
2019/05/18	CH2206	Male	Three y/o	Treatment (Wk 1)	Treatment	Fast Day	No	21:05	21:00 - 21:59	Night	37,3	44	32,4	32,4	18,46	528,51	116,84
2019/05/18	CH2205	Male	Three y/o	Treatment (Wk 1)	Treatment	Fast Day	No	21:10	21:00 - 21:59	Night	37,4		19,8	19,8	18,55		99,97

	2019/05/18	CH2206	Male	Three y/o	Treatment (Wk 1)	Treatment	Fast Day	No	21:10	21:00 - 21:59	Night	37,3		34,2	34,2	18,46		118,70
	2019/05/18	CH2205	Male	Three y/o	Treatment (Wk 1)	Treatment	Fast Day	No	21:15	21:00 - 21:59	Night	37,4	42	16,0	16,0	18,55	524,42	92,71
	2019/05/18	CH2206	Male	Three y/o	Treatment (Wk 1)	Treatment	Fast Day	No	21:15	21:00 - 21:59	Night	37,3	51	23,4	23,4	18,46	541,20	105,68
	2019/05/18	CH2205	Male	Three y/o	Treatment (Wk 1)	Treatment	Fast Day	No	21:20	21:00 - 21:59	Night	37,4	79	15,4	15,4	18,55	576,36	91,41
	2019/05/18	CH2206	Male	Three y/o	Treatment (Wk 1)	Treatment	Fast Day	No	21:20	21:00 - 21:59	Night	37,3	46	8,4	8,4	18,46	532,38	70,86
	2019/05/18	CH2205	Male	Three y/o	Treatment (Wk 1)	Treatment	Fast Day	No	21:25	21:00 - 21:59	Night	37,4	44	19,8	19,8	18,55	528,51	99,97
	2019/05/18	CH2206	Male	Three y/o	Treatment (Wk 1)	Treatment	Fast Day	No	21:25	21:00 - 21:59	Night	37,3	47	7,0	7,0	18,46	534,23	64,72
	2019/05/18	CH2205	Male	Three y/o	Treatment (Wk 1)	Treatment	Fast Day	No	21:30	21:00 - 21:59	Night	37,4	55	22,0	22,0	18,55	547,52	103,57
	2019/05/18	CH2206	Male	Three y/o	Treatment (Wk 1)	Treatment	Fast Day	No	21:30	21:00 - 21:59	Night	37,3	45	20,8	20,8	18,46	530,47	101,65
	2019/05/18	CH2205	Male	Three y/o	Treatment (Wk 1)	Treatment	Fast Day	No	21:35	21:00 - 21:59	Night	37,4	46	20,0	20,0	18,55	532,38	100,31
	2019/05/18	CH2206	Male	Three y/o	Treatment (Wk 1)	Treatment	Fast Day	No	21:35	21:00 - 21:59	Night	37,3	53	21,0	21,0	18,46	544,43	101,98
	2019/05/18	CH2205	Male	Three y/o	Treatment (Wk 1)	Treatment	Fast Day	No	21:40	21:00 - 21:59	Night	37,4	38	18,6	18,6	18,55	515,48	97,84
	2019/05/18	CH2206	Male	Three y/o	Treatment (Wk 1)	Treatment	Fast Day	No	21:40	21:00 - 21:59	Night	37,2	51	19,2	19,2	18,36	541,20	98,92
	2019/05/18	CH2205	Male	Three y/o	Treatment (Wk 1)	Treatment	Fast Day	No	21:45	21:00 - 21:59	Night	37,4	60	18,8	18,8	18,55	554,67	98,20
	2019/05/18	CH2206	Male	Three y/o	Treatment (Wk 1)	Treatment	Fast Day	No	21:45	21:00 - 21:59	Night	37,2	43	18,8	18,8	18,36	526,50	98,20
	2019/05/18	CH2205	Male	Three y/o	Treatment (Wk 1)	Treatment	Fast Day	No	21:50	21:00 - 21:59	Night	37,4	83	23,4	23,4	18,55	580,11	105,68
	2019/05/18	CH2206	Male	Three y/o	Treatment (Wk 1)	Treatment	Fast Day	No	21:50	21:00 - 21:59	Night	37,2	45	12,2	12,2	18,36	530,47	83,49
	2019/05/18	CH2205	Male	Three y/o	Treatment (Wk 1)	Treatment	Fast Day	No	21:55	21:00 - 21:59	Night	37,4	44	29,0	29,0	18,55	528,51	113,03
	2019/05/18	CH2206	Male	Three y/o	Treatment (Wk 1)	Treatment	Fast Day	No	21:55	21:00 - 21:59	Night	37,2	45	11,2	11,2	18,36	530,47	80,59
	2019/05/18	CH2205	Male	Three y/o	Treatment (Wk 1)	Treatment	Fast Day	No	22:00	22:00 - 22:59	Night	37,4	56	24,8	24,8	18,55	549,01	107,67
	2019/05/18	CH2206	Male	Three y/o	Treatment (Wk 1)	Treatment	Fast Day	No	22:00	22:00 - 22:59	Night	37,2	45	7,4	7,4	18,36	530,47	66,59
	2019/05/18	CH2205	Male	Three y/o	Treatment (Wk 1)	Treatment	Fast Day	No	22:05	22:00 - 22:59	Night	37,4	44	26,0	26,0	18,55	528,51	109,29
	2019/05/18	CH2206	Male	Three y/o	Treatment (Wk 1)	Treatment	Fast Day	No	22:05	22:00 - 22:59	Night	37,2	73	14,2	14,2	18,36	570,27	88,65
	2019/05/18	CH2205	Male	Three y/o	Treatment (Wk 1)	Treatment	Fast Day	No	22:10	22:00 - 22:59	Night	37,4	57	24,6	24,6	18,55	550,47	107,39
	2019/05/18	CH2206	Male	Three y/o	Treatment (Wk 1)	Treatment	Fast Day	No	22:10	22:00 - 22:59	Night	37,2	47	14,0	14,0	18,36	534,23	88,17
	2019/05/18	CH2205	Male	Three y/o	Treatment (Wk 1)	Treatment	Fast Day	No	22:15	22:00 - 22:59	Night	37,4	55	26,8	26,8	18,55	547,52	110,33
	2019/05/18	CH2206	Male	Three y/o	Treatment (Wk 1)	Treatment	Fast Day	No	22:15	22:00 - 22:59	Night	37,2	40	9,0	9,0	18,36	520,09	73,19
	2019/05/18	CH2205	Male	Three y/o	Treatment (Wk 1)	Treatment	Fast Day	No	22:20	22:00 - 22:59	Night	37,4	43	27,6	27,6	18,55	526,50	111,33
	2019/05/18	CH2206	Male	Three y/o	Treatment (Wk 1)	Treatment	Fast Day	No	22:20	22:00 - 22:59	Night	37,3	36	5,6	5,6	18,46	510,56	57,21
	2019/05/18	CH2205	Male	Three y/o	Treatment (Wk 1)	Treatment	Fast Day	No	22:25	22:00 - 22:59	Night	37,4	45	24,6	24,6	18,55	530,47	107,39
	2019/05/18	CH2206	Male	Three y/o	Treatment (Wk 1)	Treatment	Fast Day	No	22:25	22:00 - 22:59	Night	37,3	49	7,6	7,6	18,46	537,80	67,49
	2019/05/18	CH2205	Male	Three y/o	Treatment (Wk 1)	Treatment	Fast Day	No	22:30	22:00 - 22:59	Night	37,4	50	22,8	22,8	18,55	539,52	104,79
	2019/05/18	CH2206	Male	Three y/o	Treatment (Wk 1)	Treatment	Fast Day	No	22:30	22:00 - 22:59	Night	37,3	75	34,0	34,0	18,46	572,36	118,49
	2019/05/18	CH2205	Male	Three y/o	Treatment (Wk 1)	Treatment	Fast Day	No	22:35	22:00 - 22:59	Night	37,4		18,2	18,2	18,55		97,10
	2019/05/18	CH2206	Male	Three y/o	Treatment (Wk 1)	Treatment	Fast Day	No	22:35	22:00 - 22:59	Night	37,2	54	32,6	32,6	18,36	545,99	117,05
	2019/05/18	CH2205	Male	Three y/o	Treatment (Wk 1)	Treatment	Fast Day	No	22:40	22:00 - 22:59	Night	37,3	46	18,0	18,0	18,46	532,38	96,72
	2019/05/18	CH2206	Male	Three y/o	Treatment (Wk 1)	Treatment	Fast Day	No	22:40	22:00 - 22:59	Night	37,2	44	34,6	34,6	18,36	528,51	119,10
	2019/05/18	CH2205	Male	Three y/o	Treatment (Wk 1)	Treatment	Fast Day	No	22:45	22:00 - 22:59	Night	37,4	42	14,2	14,2	18,55	524,42	88,65
	2019/05/18	CH2206	Male	Three y/o	Treatment (Wk 1)	Treatment	Fast Day	No	22:45	22:00 - 22:59	Night	37,2	44	32,6	32,6	18,36	528,51	117,05
	2019/05/18	CH2205	Male	Three y/o	Treatment (Wk 1)	Treatment	Fast Day	No	22:50	22:00 - 22:59	Night	37,4	43	13,6	13,6	18,55	526,50	87,18
	2019/05/18	CH2206	Male	Three y/o	Treatment (Wk 1)	Treatment	Fast Day	No	22:50	22:00 - 22:59	Night	37,2	40	31,4	31,4	18,36	520,09	115,76
	2019/05/18	CH2205	Male	Three y/o	Treatment (Wk 1)	Treatment	Fast Day	No	22:55	22:00 - 22:59	Night	37,4	48	18,4	18,4	18,55	536,04	97,47
	2019/05/18	CH2206	Male	Three y/o	Treatment (Wk 1)	Treatment	Fast Day	No	22:55	22:00 - 22:59	Night	37,1	38	27,8	27,8	18,26	515,48	111,58
	2019/05/18	CH2205	Male	Three y/o	Treatment (Wk 1)	Treatment	Fast Day	No	23:00	23:00 - 23:59	Night	37,4		12,0	12,0	18,55		82,93
	2019/05/18	CH2206	Male	Three y/o	Treatment (Wk 1)	Treatment	Fast Day	No	23:00	23:00 - 23:59	Night	37,1	76	32,4	32,4	18,26	573,39	116,84
	2019/05/18	CH2205	Male	Three y/o	Treatment (Wk 1)	Treatment	Fast Day	No	23:05	23:00 - 23:59	Night	37,4	57	13,2	13,2	18,55	550,47	86,17
	2019/05/18	CH2206	Male	Three y/o	Treatment (Wk 1)	Treatment	Fast Day	No	23:05	23:00 - 23:59	Night	37,1	47	34,6	34,6	18,26	534,23	119,10
	2019/05/18	CH2205	Male	Three y/o	Treatment (Wk 1)	Treatment	Fast Day	No	23:10	23:00 - 23:59	Night	37,4	34	14,2	14,2	18,55	505,29	88,65
	2019/05/18	CH2206	Male	Three y/o	Treatment (Wk 1)	Treatment	Fast Day	No	23:10	23:00 - 23:59	Night	37,1	67	37,8	37,8	18,26	563,53	122,14
	2019/05/18	CH2205	Male	Three y/o	Treatment (Wk 1)	Treatment	Fast Day	No	23:15	23:00 - 23:59	Night	37,4	88	10,6	10,6	18,55	584,49	78,73
	2019/05/18	CH2206	Male	Three y/o	Treatment (Wk 1)	Treatment	Fast Day	No	23:15	23:00 - 23:59	Night	37,1	57	37,2	37,2	18,26	550,47	121,59
	2019/05/18	CH2205	Male	Three y/o	Treatment (Wk 1)	Treatment	Fast Day	No	23:20	23:00 - 23:59	Night	37,4	88	7,6	7,6	18,55	584,49	67,49
	2019/05/18	CH2206	Male	Three y/o	Treatment (Wk 1)	Treatment	Fast Day	No	23:20	23:00 - 23:59	Night	37,1	42	33,2	33,2	18,26	524,42	117,68
	2019/05/18	CH2205	Male	Three y/o	Treatment (Wk 1)	Treatment	Fast Day	No	23:25	23:00 - 23:59	Night	37,4	53	15,8	15,8	18,55	544,43	92,28
	2019/05/18	CH2206	Male	Three y/o	Treatment (Wk 1)	Treatment	Fast Day	No	23:25	23:00 - 23:59	Night	37,1	50	39,8	39,8	18,26	539,52	123,91
	2019/05/18	CH2205	Male	Three y/o	Treatment (Wk 1)	Treatment	Fast Day	No	23:30	23:00 - 23:59	Night	37,3	45	15,0	15,0	18,46	530,47	90,51
	2019/05/18	CH2206	Male	Three y/o	Treatment (Wk 1)	Treatment	Fast Day	No	23:30	23:00 - 23:59	Night	37,1	43	40,0	40,0	18,26	526,50	124,09
	2019/05/18	CH2205	Male	Three y/o	Treatment (Wk 1)	Treatment	Fast Day	No	23:35	23:00 - 23:59	Night	37,3	43	20,8	20,8	18,46	526,50	101,65
	2019/05/18	CH2206	Male	Three y/o	Treatment (Wk 1)	Treatment	Fast Day	No	23:35	23:00 - 23:59	Night	37,0	51	34,8	34,8	18,16	541,20	119,29
	2019/05/18	CH2205	Male	Three y/o	Treatment (Wk 1)	Treatment	Fast Day	No	23:40	23:00 - 23:59	Night	37,3	46	13,2	13,2	18,46	532,38	86,17
	2019/05/18	CH2206	Male	Three y/o	Treatment (Wk 1)	Treatment	Fast Day	No	23:40	23:00 - 23:59	Night	37,0	53	24,2	24,2	18,16	544,43	106,83
	2019/05/18	CH2205	Male	Three y/o	Treatment (Wk 1)	Treatment	Fast Day	No	23:45	23:00 - 23:59	Night	37,3	41	20,6	20,6	18,46	522,29	101,32
	2019/05/18	CH2206	Male	Three y/o	Treatment (Wk 1)	Treatment	Fast Day	No	23:45	23:00 - 23:59	Night	37,0	45	30,4	30,4	18,16	530,47	114,65
	2019/05/18	CH2205	Male	Three y/o	Treatment (Wk 1)	Treatment	Fast Day	No	23:50	23:00 - 23:59	Night	37,4	128	70,2	70,2	18,55	611,22	143,54
	2019/05/18	CH2206	Male	Three y/o	Treatment (Wk 1)	Treatment	Fast Day	No	23:50	23:00 - 23:59	Night	37,1	129	43,4	43,4	18,26	611,75	126,90
	2019/05/18	CH2205	Male	Three y/o	Treatment (Wk 1)	Treatment	Fast Day	No	23:55	23:00 - 23:59	Night	37,4		21,0	21,0	18,55		101,98
	2019/05/18	CH2206	Male	Three y/o	Treatment (Wk 1)	Treatment	Fast Day	No	23:55	23:00 - 23:59	Night	37,1	172	43,2	43,2	18,26	630,73	126,74
5	2019/05/19	CH2205	Male	Three y/o	Treatment (Wk 1)	Treatment	Fast Day	No	00:00	00:00 - 00:59	Early Morning	37,3	81	13,8	13,8	18,46	578,26	87,68
	2019/05/19	CH2206	Male	Three y/o	Treatment (Wk 1)	Treatment	Fast Day	No	00:00	00:00 - 00:59	Early Morning	37,1	43	60,0	60,0	18,26	526,50	138,09
	2019/05/19	CH2205	Male	Three y/o	Treatment (Wk 1)	Treatment	Fast Day	No	00:05	00:00 - 00:59	Early Morning	37,3	54	16,0	16,0	18,46	545,99	92,71

2019/05/19	CH2206	Male	Three y/o	Treatment (Wk 1)	Treatment	Fast Day	No	00:05	00:00 - 00:59	Early Morning	37,0	44	57,8	57,8	18,16	528,51	136,80
2019/05/19	CH2205	Male	Three y/o	Treatment (Wk 1)	Treatment	Fast Day	No	00:10	00:00 - 00:59	Early Morning	37,2	87	14,4	14,4	18,36	583,64	89,12
2019/05/19	CH2206	Male	Three y/o	Treatment (Wk 1)	Treatment	Fast Day	No	00:10	00:00 - 00:59	Early Morning	36,9	51	54,0	54,0	18,06	541,20	134,45
2019/05/19	CH2205	Male	Three y/o	Treatment (Wk 1)	Treatment	Fast Day	No	00:15	00:00 - 00:59	Early Morning	37,2	43	4,6	4,6	18,36	526,50	50,61
2019/05/19	CH2206	Male	Three y/o	Treatment (Wk 1)	Treatment	Fast Day	No	00:15	00:00 - 00:59	Early Morning	36,9	53	47,2	47,2	18,06	544,43	129,80
2019/05/19	CH2205	Male	Three y/o	Treatment (Wk 1)	Treatment	Fast Day	No	00:20	00:00 - 00:59	Early Morning	37,1	67	8,2	8,2	18,26	563,53	70,05
2019/05/19	CH2206	Male	Three y/o	Treatment (Wk 1)	Treatment	Fast Day	No	00:20	00:00 - 00:59	Early Morning	37,0	44	47,6	47,6	18,16	528,51	130,09
2019/05/19	CH2205	Male	Three y/o	Treatment (Wk 1)	Treatment	Fast Day	No	00:25	00:00 - 00:59	Early Morning	37,1	57	17,0	17,0	18,26	550,47	94,77
2019/05/19	CH2206	Male	Three y/o	Treatment (Wk 1)	Treatment	Fast Day	No	00:25	00:00 - 00:59	Early Morning	37,0	129	51,6	51,6	18,16	611,75	132,87
2019/05/19	CH2205	Male	Three y/o	Treatment (Wk 1)	Treatment	Fast Day	No	00:30	00:00 - 00:59	Early Morning	37,2	87	97,6	97,6	18,36	583,64	155,00
2019/05/19	CH2206	Male	Three y/o	Treatment (Wk 1)	Treatment	Fast Day	No	00:30	00:00 - 00:59	Early Morning	37,2		70,8	70,8	18,36		143,83
2019/05/19	CH2205	Male	Three y/o	Treatment (Wk 1)	Treatment	Fast Day	No	00:35	00:00 - 00:59	Early Morning	37,4	112	62,4	62,4	18,55	601,96	139,45
2019/05/19	CH2206	Male	Three y/o	Treatment (Wk 1)	Treatment	Fast Day	No	00:35	00:00 - 00:59	Early Morning	37,1	132	138,4	138,4	18,26	613,32	167,20
2019/05/19	CH2205	Male	Three y/o	Treatment (Wk 1)	Treatment	Fast Day	No	00:40	00:00 - 00:59	Early Morning	37,4	64	87,6	87,6	18,55	559,88	151,23
2019/05/19	CH2206	Male	Three y/o	Treatment (Wk 1)	Treatment	Fast Day	No	00:40	00:00 - 00:59	Early Morning	37,2	104	162,2	162,2	18,36	596,69	172,76
2019/05/19	CH2205	Male	Three y/o	Treatment (Wk 1)	Treatment	Fast Day	No	00:45	00:00 - 00:59	Early Morning	37,4	63	225,8	225,8	18,55	558,62	184,39
2019/05/19	CH2206	Male	Three y/o	Treatment (Wk 1)	Treatment	Fast Day	No	00:45	00:00 - 00:59	Early Morning	37,4	129	275,8	275,8	18,55	611,75	191,45
2019/05/19	CH2205	Male	Three y/o	Treatment (Wk 1)	Treatment	Fast Day	No	00:50	00:00 - 00:59	Early Morning	37,5	109	178,4	178,4	18,65	600,04	176,10
2019/05/19	CH2206	Male	Three y/o	Treatment (Wk 1)	Treatment	Fast Day	No	00:50	00:00 - 00:59	Early Morning	37,4		82,6	82,6	18,55		149,19
2019/05/19	CH2205	Male	Three y/o	Treatment (Wk 1)	Treatment	Fast Day	No	00:55	00:00 - 00:59	Early Morning	37,4	119	109,4	109,4	18,55	606,20	158,98
2019/05/19	CH2206	Male	Three y/o	Treatment (Wk 1)	Treatment	Fast Day	No	00:55	00:00 - 00:59	Early Morning	37,5	119	101,2	101,2	18,65	606,20	156,26
2019/05/19	CH2205	Male	Three y/o	Treatment (Wk 1)	Treatment	Fast Day	No	01:00	01:00 - 01:59	Early Morning	37,5	158	79,4	79,4	18,65	625,26	147,81
2019/05/19	CH2206	Male	Three y/o	Treatment (Wk 1)	Treatment	Fast Day	No	01:00	01:00 - 01:59	Early Morning	37,7	109	122,6	122,6	18,85	600,04	162,96
2019/05/19	CH2205	Male	Three y/o	Treatment (Wk 1)	Treatment	Fast Day	No	01:05	01:00 - 01:59	Early Morning	37,5	74	78,6	78,6	18,65	571,33	147,46
2019/05/19	CH2206	Male	Three y/o	Treatment (Wk 1)	Treatment	Fast Day	No	01:05	01:00 - 01:59	Early Morning	37,7		56,0	56,0	18,85		135,70
2019/05/19	CH2205	Male	Three y/o	Treatment (Wk 1)	Treatment	Fast Day	No	01:10	01:00 - 01:59	Early Morning	37,4	120	34,2	34,2	18,55	606,78	118,70
2019/05/19	CH2206	Male	Three y/o	Treatment (Wk 1)	Treatment	Fast Day	No	01:10	01:00 - 01:59	Early Morning	37,7	78	38,2	38,2	18,85	575,38	122,50
2019/05/19	CH2205	Male	Three y/o	Treatment (Wk 1)	Treatment	Fast Day	No	01:15	01:00 - 01:59	Early Morning	37,2	99	38,8	38,8	18,36	593,14	123,04
2019/05/19	CH2206	Male	Three y/o	Treatment (Wk 1)	Treatment	Fast Day	No	01:15	01:00 - 01:59	Early Morning	37,3	88	51,8	51,8	18,46	584,49	133,01
2019/05/19	CH2205	Male	Three y/o	Treatment (Wk 1)	Treatment	Fast Day	No	01:20	01:00 - 01:59	Early Morning	37,2		28,6	28,6	18,36		112,56
2019/05/19	CH2206	Male	Three y/o	Treatment (Wk 1)	Treatment	Fast Day	No	01:20	01:00 - 01:59	Early Morning	37,2	45	64,2	64,2	18,36	530,47	140,44
2019/05/19	CH2205	Male	Three y/o	Treatment (Wk 1)	Treatment	Fast Day	No	01:25	01:00 - 01:59	Early Morning	37,1	109	30,2	30,2	18,26	600,04	114,42
2019/05/19	CH2206	Male	Three y/o	Treatment (Wk 1)	Treatment	Fast Day	No	01:25	01:00 - 01:59	Early Morning	37,1	71	63,0	63,0	18,26	568,10	139,78
2019/05/19	CH2205	Male	Three y/o	Treatment (Wk 1)	Treatment	Fast Day	No	01:30	01:00 - 01:59	Early Morning	37,1		34,8	34,8	18,26		119,29
2019/05/19	CH2206	Male	Three y/o	Treatment (Wk 1)	Treatment	Fast Day	No	01:30	01:00 - 01:59	Early Morning	37,1	49	51,4	51,4	18,26	537,80	132,74
2019/05/19	CH2205	Male	Three y/o	Treatment (Wk 1)	Treatment	Fast Day	No	01:35	01:00 - 01:59	Early Morning	37,1	80	25,0	25,0	18,26	577,31	107,94
2019/05/19	CH2206	Male	Three y/o	Treatment (Wk 1)	Treatment	Fast Day	No	01:35	01:00 - 01:59	Early Morning	37,1	55	49,0	49,0	18,26	547,52	131,09
2019/05/19	CH2205	Male	Three y/o	Treatment (Wk 1)	Treatment	Fast Day	No	01:40	01:00 - 01:59	Early Morning	37,2	43	18,6	18,6	18,36	526,50	97,84
2019/05/19	CH2206	Male	Three y/o	Treatment (Wk 1)	Treatment	Fast Day	No	01:40	01:00 - 01:59	Early Morning	37,3	50	50,4	50,4	18,46	539,52	132,06
2019/05/19	CH2205	Male	Three y/o	Treatment (Wk 1)	Treatment	Fast Day	No	01:45	01:00 - 01:59	Early Morning	37,3	60	20,2	20,2	18,46	554,67	100,65
2019/05/19	CH2206	Male	Three y/o	Treatment (Wk 1)	Treatment	Fast Day	No	01:45	01:00 - 01:59	Early Morning	37,4	57	56,0	56,0	18,55	550,47	135,70
2019/05/19	CH2205	Male	Three y/o	Treatment (Wk 1)	Treatment	Fast Day	No	01:50	01:00 - 01:59	Early Morning	37,4	36	17,4	17,4	18,55	510,56	95,56
2019/05/19	CH2206	Male	Three y/o	Treatment (Wk 1)	Treatment	Fast Day	No	01:50	01:00 - 01:59	Early Morning	37,4	40	49,0	49,0	18,55	520,09	131,09
2019/05/19	CH2205	Male	Three y/o	Treatment (Wk 1)	Treatment	Fast Day	No	01:55	01:00 - 01:59	Early Morning	37,4	74	13,2	13,2	18,55	571,33	86,17
2019/05/19	CH2206	Male	Three y/o	Treatment (Wk 1)	Treatment	Fast Day	No	01:55	01:00 - 01:59	Early Morning	37,4	48	49,4	49,4	18,55	536,04	131,37
2019/05/19	CH2205	Male	Three y/o	Treatment (Wk 1)	Treatment	Fast Day	No	02:00	02:00 - 02:59	Early Morning	37,5	47	16,0	16,0	18,65	534,23	92,71
2019/05/19	CH2206	Male	Three y/o	Treatment (Wk 1)	Treatment	Fast Day	No	02:00	02:00 - 02:59	Early Morning	37,4	57	58,8	58,8	18,55	550,47	137,39
2019/05/19	CH2205	Male	Three y/o	Treatment (Wk 1)	Treatment	Fast Day	No	02:05	02:00 - 02:59	Early Morning	37,5	98	18,4	18,4	18,65	592,41	97,47
2019/05/19	CH2206	Male	Three y/o	Treatment (Wk 1)	Treatment	Fast Day	No	02:05	02:00 - 02:59	Early Morning	37,4	79	66,4	66,4	18,55	576,36	141,61
2019/05/19	CH2205	Male	Three y/o	Treatment (Wk 1)	Treatment	Fast Day	No	02:10	02:00 - 02:59	Early Morning	37,5	44	18,2	18,2	18,65	528,51	97,10
2019/05/19	CH2206	Male	Three y/o	Treatment (Wk 1)	Treatment	Fast Day	No	02:10	02:00 - 02:59	Early Morning	37,4	73	58,8	58,8	18,55	570,27	137,39
2019/05/19	CH2205	Male	Three y/o	Treatment (Wk 1)	Treatment	Fast Day	No	02:15	02:00 - 02:59	Early Morning	37,5	58	24,4	24,4	18,65	551,90	107,11
2019/05/19	CH2206	Male	Three y/o	Treatment (Wk 1)	Treatment	Fast Day	No	02:15	02:00 - 02:59	Early Morning	37,3	54	48,8	48,8	18,46	545,99	130,95
2019/05/19	CH2205	Male	Three y/o	Treatment (Wk 1)	Treatment	Fast Day	No	02:20	02:00 - 02:59	Early Morning	37,3		21,2	21,2	18,46		102,31
2019/05/19	CH2206	Male	Three y/o	Treatment (Wk 1)	Treatment	Fast Day	No	02:20	02:00 - 02:59	Early Morning	37,3	41	59,4	59,4	18,46	522,29	137,75
2019/05/19	CH2205	Male	Three y/o	Treatment (Wk 1)	Treatment	Fast Day	No	02:25	02:00 - 02:59	Early Morning	37,3		26,0	26,0	18,46		109,29
2019/05/19	CH2206	Male	Three y/o	Treatment (Wk 1)	Treatment	Fast Day	No	02:25	02:00 - 02:59	Early Morning	37,3	59	60,8	60,8	18,46	553,30	138,55
2019/05/19	CH2205	Male	Three y/o	Treatment (Wk 1)	Treatment	Fast Day	No	02:30	02:00 - 02:59	Early Morning	37,3	147	26,8	26,8	18,46	620,52	110,33
2019/05/19	CH2206	Male	Three y/o	Treatment (Wk 1)	Treatment	Fast Day	No	02:30	02:00 - 02:59	Early Morning	37,3	44	63,2	63,2	18,46	528,51	139,89
2019/05/19	CH2205	Male	Three y/o	Treatment (Wk 1)	Treatment	Fast Day	No	02:35	02:00 - 02:59	Early Morning	37,3	50	23,4	23,4	18,46	539,52	105,68
2019/05/19	CH2206	Male	Three y/o	Treatment (Wk 1)	Treatment	Fast Day	No	02:35	02:00 - 02:59	Early Morning	37,3	75	51,6	51,6	18,46	572,36	132,87
2019/05/19	CH2205	Male	Three y/o	Treatment (Wk 1)	Treatment	Fast Day	No	02:40	02:00 - 02:59	Early Morning	37,3		23,6	23,6	18,46		105,97
2019/05/19	CH2206	Male	Three y/o	Treatment (Wk 1)	Treatment	Fast Day	No	02:40	02:00 - 02:59	Early Morning	37,3	71	50,2	50,2	18,46	568,10	131,92
2019/05/19	CH2205	Male	Three y/o	Treatment (Wk 1)	Treatment	Fast Day	No	02:45	02:00 - 02:59	Early Morning	37,2	41	26,6	26,6	18,36	522,29	110,07
2019/05/19	CH2206	Male	Three y/o	Treatment (Wk 1)	Treatment	Fast Day	No	02:45	02:00 - 02:59	Early Morning	37,3	43	48,2	48,2	18,46	526,50	130,52
2019/05/19	CH2205	Male	Three y/o	Treatment (Wk 1)	Treatment	Fast Day	No	02:50	02:00 - 02:59	Early Morning	37,2		25,4	25,4	18,36		108,49
2019/05/19	CH2206	Male	Three y/o	Treatment (Wk 1)	Treatment	Fast Day	No	02:50	02:00 - 02:59	Early Morning	37,3	47	48,0	48,0	18,46	534,23	130,38
2019/05/19	CH2205	Male	Three y/o	Treatment (Wk 1)	Treatment	Fast Day	No	02:55	02:00 - 02:59	Early Morning	37,2	50	17,6	17,6	18,36	539,52	95,95
2019/05/19	CH2206	Male	Three y/o	Treatment (Wk 1)	Treatment	Fast Day	No	02:55	02:00 - 02:59	Early Morning	37,3	45	44,4	44,4	18,46	530,47	127,69
2019/05/19	CH2205	Male	Three y/o	Treatment (Wk 1)	Treatment	Fast Day	No	03:00	03:00 - 03:59	Early Morning	37,2	52	18,6	18,6	18,36	542,83	97,84

2019/05/19	CH2206	Male	Three y/o	Treatment (Wk 1)	Treatment	Fast Day	No	03:00	03:00 - 03:59	Early Morning	37,3	49	41,2	41,2	18,46	537,80	125,11
2019/05/19	CH2205	Male	Three y/o	Treatment (Wk 1)	Treatment	Fast Day	No	03:05	03:00 - 03:59	Early Morning	37,2	91	19,2	19,2	18,36	586,98	98,92
2019/05/19	CH2206	Male	Three y/o	Treatment (Wk 1)	Treatment	Fast Day	No	03:05	03:00 - 03:59	Early Morning	37,3	50	41,6	41,6	18,46	539,52	125,44
2019/05/19	CH2205	Male	Three y/o	Treatment (Wk 1)	Treatment	Fast Day	No	03:10	03:00 - 03:59	Early Morning	37,2	37	22,2	22,2	18,36	513,06	103,88
2019/05/19	CH2206	Male	Three y/o	Treatment (Wk 1)	Treatment	Fast Day	No	03:10	03:00 - 03:59	Early Morning	37,3	51	47,6	47,6	18,46	541,20	130,09
2019/05/19	CH2205	Male	Three y/o	Treatment (Wk 1)	Treatment	Fast Day	No	03:15	03:00 - 03:59	Early Morning	37,2	51	22,2	22,2	18,36	541,20	103,88
2019/05/19	CH2206	Male	Three y/o	Treatment (Wk 1)	Treatment	Fast Day	No	03:15	03:00 - 03:59	Early Morning	37,3	68	43,8	43,8	18,46	564,71	127,22
2019/05/19	CH2205	Male	Three y/o	Treatment (Wk 1)	Treatment	Fast Day	No	03:20	03:00 - 03:59	Early Morning	37,2	148	5,2	5,2	18,36	620,97	54,72
2019/05/19	CH2206	Male	Three y/o	Treatment (Wk 1)	Treatment	Fast Day	No	03:20	03:00 - 03:59	Early Morning	37,3	76	17,4	17,4	18,46	573,39	95,56
2019/05/19	CH2205	Male	Three y/o	Treatment (Wk 1)	Treatment	Fast Day	No	03:25	03:00 - 03:59	Early Morning	37,2	51	3,0	3,0	18,36	541,20	36,34
2019/05/19	CH2206	Male	Three y/o	Treatment (Wk 1)	Treatment	Fast Day	No	03:25	03:00 - 03:59	Early Morning	37,3	49	31,4	31,4	18,46	537,80	115,76
2019/05/19	CH2205	Male	Three y/o	Treatment (Wk 1)	Treatment	Fast Day	No	03:30	03:00 - 03:59	Early Morning	37,3	43	3,0	3,0	18,46	526,50	36,34
2019/05/19	CH2206	Male	Three y/o	Treatment (Wk 1)	Treatment	Fast Day	No	03:30	03:00 - 03:59	Early Morning	37,3	63	34,6	34,6	18,46	558,62	119,10
2019/05/19	CH2205	Male	Three y/o	Treatment (Wk 1)	Treatment	Fast Day	No	03:35	03:00 - 03:59	Early Morning	37,3	43	3,4	3,4	18,46	526,50	40,51
2019/05/19	CH2206	Male	Three y/o	Treatment (Wk 1)	Treatment	Fast Day	No	03:35	03:00 - 03:59	Early Morning	37,2	38	24,6	24,6	18,36	515,48	107,39
2019/05/19	CH2205	Male	Three y/o	Treatment (Wk 1)	Treatment	Fast Day	No	03:40	03:00 - 03:59	Early Morning	37,3	47	4,2	4,2	18,46	534,23	47,57
2019/05/19	CH2206	Male	Three y/o	Treatment (Wk 1)	Treatment	Fast Day	No	03:40	03:00 - 03:59	Early Morning	37,2	45	17,2	17,2	18,36	530,47	95,17
2019/05/19	CH2205	Male	Three y/o	Treatment (Wk 1)	Treatment	Fast Day	No	03:45	03:00 - 03:59	Early Morning	37,3	67	39,8	39,8	18,46	563,53	123,91
2019/05/19	CH2206	Male	Three y/o	Treatment (Wk 1)	Treatment	Fast Day	No	03:45	03:00 - 03:59	Early Morning	37,1	41	37,8	37,8	18,26	522,29	122,14
2019/05/19	CH2205	Male	Three y/o	Treatment (Wk 1)	Treatment	Fast Day	No	03:50	03:00 - 03:59	Early Morning	37,3	145	35,4	35,4	18,46	619,62	119,88
2019/05/19	CH2206	Male	Three y/o	Treatment (Wk 1)	Treatment	Fast Day	No	03:50	03:00 - 03:59	Early Morning	37,0	141	29,6	29,6	18,16	617,75	113,73
2019/05/19	CH2205	Male	Three y/o	Treatment (Wk 1)	Treatment	Fast Day	No	03:55	03:00 - 03:59	Early Morning	37,3		40,6	40,6	18,46		124,60
2019/05/19	CH2206	Male	Three y/o	Treatment (Wk 1)	Treatment	Fast Day	No	03:55	03:00 - 03:59	Early Morning	37,1	128	134,0	134,0	18,26	611,22	166,07
2019/05/19	CH2205	Male	Three y/o	Treatment (Wk 1)	Treatment	Fast Day	No	04:00	04:00 - 04:59	Morning	37,3		157,6	157,6	18,46		171,75
2019/05/19	CH2206	Male	Three y/o	Treatment (Wk 1)	Treatment	Fast Day	No	04:00	04:00 - 04:59	Morning	37,2	107	55,0	55,0	18,36	598,73	135,08
2019/05/19	CH2205	Male	Three y/o	Treatment (Wk 1)	Treatment	Fast Day	No	04:05	04:00 - 04:59	Morning	37,2	49	175,0	175,0	18,36	537,80	175,43
2019/05/19	CH2206	Male	Three y/o	Treatment (Wk 1)	Treatment	Fast Day	No	04:05	04:00 - 04:59	Morning	37,2	120	194,4	194,4	18,36	606,78	179,12
2019/05/19	CH2205	Male	Three y/o	Treatment (Wk 1)	Treatment	Fast Day	No	04:10	04:00 - 04:59	Morning	37,3	60	163,2	163,2	18,46	554,67	172,97
2019/05/19	CH2206	Male	Three y/o	Treatment (Wk 1)	Treatment	Fast Day	No	04:10	04:00 - 04:59	Morning	37,3	59	179,4	179,4	18,46	553,30	176,30
2019/05/19	CH2205	Male	Three y/o	Treatment (Wk 1)	Treatment	Fast Day	No	04:15	04:00 - 04:59	Morning	37,4	113	93,8	93,8	18,55	602,58	153,61
2019/05/19	CH2206	Male	Three y/o	Treatment (Wk 1)	Treatment	Fast Day	No	04:15	04:00 - 04:59	Morning	37,4	135	103,0	103,0	18,55	614,84	156,87
2019/05/19	CH2205	Male	Three y/o	Treatment (Wk 1)	Treatment	Fast Day	No	04:20	04:00 - 04:59	Morning	37,6		23,8	23,8	18,75		106,26
2019/05/19	CH2206	Male	Three y/o	Treatment (Wk 1)	Treatment	Fast Day	No	04:20	04:00 - 04:59	Morning	37,5	129	186,2	186,2	18,65	611,75	177,60
2019/05/19	CH2205	Male	Three y/o	Treatment (Wk 1)	Treatment	Fast Day	No	04:25	04:00 - 04:59	Morning	37,7	98	275,2	275,2	18,85	592,41	191,37
2019/05/19	CH2206	Male	Three y/o	Treatment (Wk 1)	Treatment	Fast Day	No	04:25	04:00 - 04:59	Morning	37,7	114	111,0	111,0	18,85	603,20	159,48
2019/05/19	CH2205	Male	Three y/o	Treatment (Wk 1)	Treatment	Fast Day	No	04:30	04:00 - 04:59	Morning	37,7	125	18,8	18,8	18,85	609,60	98,20
2019/05/19	CH2206	Male	Three y/o	Treatment (Wk 1)	Treatment	Fast Day	No	04:30	04:00 - 04:59	Morning	37,7		130,0	130,0	18,85		165,01
2019/05/19	CH2205	Male	Three y/o	Treatment (Wk 1)	Treatment	Fast Day	No	04:35	04:00 - 04:59	Morning	38,0		34,2	34,2	19,15		118,70
2019/05/19	CH2206	Male	Three y/o	Treatment (Wk 1)	Treatment	Fast Day	No	04:35	04:00 - 04:59	Morning	37,8	123	227,0	227,0	18,95	608,49	184,58
2019/05/19	CH2205	Male	Three y/o	Treatment (Wk 1)	Treatment	Fast Day	No	04:40	04:00 - 04:59	Morning	37,6		18,0	18,0	18,75		96,72
2019/05/19	CH2206	Male	Three y/o	Treatment (Wk 1)	Treatment	Fast Day	No	04:40	04:00 - 04:59	Morning	37,8	112	29,2	29,2	18,95	601,96	113,27
2019/05/19	CH2205	Male	Three y/o	Treatment (Wk 1)	Treatment	Fast Day	No	04:45	04:00 - 04:59	Morning	37,7	99	69,0	69,0	18,85	593,14	142,94
2019/05/19	CH2206	Male	Three y/o	Treatment (Wk 1)	Treatment	Fast Day	No	04:45	04:00 - 04:59	Morning	37,7	129	151,2	151,2	18,85	611,75	170,30
2019/05/19	CH2205	Male	Three y/o	Treatment (Wk 1)	Treatment	Fast Day	No	04:50	04:00 - 04:59	Morning	37,5	117	47,6	47,6	18,65	605,02	130,09
2019/05/19	CH2206	Male	Three y/o	Treatment (Wk 1)	Treatment	Fast Day	No	04:50	04:00 - 04:59	Morning	37,8	129	76,2	76,2	18,95	611,75	146,38
2019/05/19	CH2205	Male	Three y/o	Treatment (Wk 1)	Treatment	Fast Day	No	04:55	04:00 - 04:59	Morning	37,3	64	24,2	24,2	18,46	559,88	106,83
2019/05/19	CH2206	Male	Three y/o	Treatment (Wk 1)	Treatment	Fast Day	No	04:55	04:00 - 04:59	Morning	37,7	107	18,4	18,4	18,85	598,73	97,47
2019/05/19	CH2205	Male	Three y/o	Treatment (Wk 1)	Treatment	Fast Day	No	05:00	05:00 - 05:59	Morning	37,3	56	34,0	34,0	18,46	549,01	118,49
2019/05/19	CH2206	Male	Three y/o	Treatment (Wk 1)	Treatment	Fast Day	No	05:00	05:00 - 05:59	Morning	37,2	57	22,0	22,0	18,36	550,47	103,57
2019/05/19	CH2205	Male	Three y/o	Treatment (Wk 1)	Treatment	Fast Day	No	05:05	05:00 - 05:59	Morning	37,4		25,0	25,0	18,55		107,94
2019/05/19	CH2206	Male	Three y/o	Treatment (Wk 1)	Treatment	Fast Day	No	05:05	05:00 - 05:59	Morning	37,0	50	13,4	13,4	18,16	539,52	86,68
2019/05/19	CH2205	Male	Three y/o	Treatment (Wk 1)	Treatment	Fast Day	No	05:10	05:00 - 05:59	Morning	37,4		27,8	27,8	18,55		111,58
2019/05/19	CH2206	Male	Three y/o	Treatment (Wk 1)	Treatment	Fast Day	No	05:10	05:00 - 05:59	Morning	36,8	74	27,6	27,6	17,96	571,33	111,33
2019/05/19	CH2205	Male	Three y/o	Treatment (Wk 1)	Treatment	Fast Day	No	05:15	05:00 - 05:59	Morning	37,4	52	27,2	27,2	18,55	542,83	110,83
2019/05/19	CH2206	Male	Three y/o	Treatment (Wk 1)	Treatment	Fast Day	No	05:15	05:00 - 05:59	Morning	36,8	49	29,0	29,0	17,96	537,80	113,03
2019/05/19	CH2205	Male	Three y/o	Treatment (Wk 1)	Treatment	Fast Day	No	05:20	05:00 - 05:59	Morning	37,3		31,4	31,4	18,46		115,76
2019/05/19	CH2206	Male	Three y/o	Treatment (Wk 1)	Treatment	Fast Day	No	05:20	05:00 - 05:59	Morning	36,8	45	34,4	34,4	17,96	530,47	118,90
2019/05/19	CH2205	Male	Three y/o	Treatment (Wk 1)	Treatment	Fast Day	No	05:25	05:00 - 05:59	Morning	37,3		30,8	30,8	18,46		115,10
2019/05/19	CH2206	Male	Three y/o	Treatment (Wk 1)	Treatment	Fast Day	No	05:25	05:00 - 05:59	Morning	36,9	78	33,0	33,0	18,06	575,38	117,47
2019/05/19	CH2205	Male	Three y/o	Treatment (Wk 1)	Treatment	Fast Day	No	05:30	05:00 - 05:59	Morning	37,3		26,6	26,6	18,46		110,07
2019/05/19	CH2206	Male	Three y/o	Treatment (Wk 1)	Treatment	Fast Day	No	05:30	05:00 - 05:59	Morning	36,9	44	32,8	32,8	18,06	528,51	117,26
2019/05/19	CH2205	Male	Three y/o	Treatment (Wk 1)	Treatment	Fast Day	No	05:35	05:00 - 05:59	Morning	37,3	68	28,6	28,6	18,46	564,71	112,56
2019/05/19	CH2206	Male	Three y/o	Treatment (Wk 1)	Treatment	Fast Day	No	05:35	05:00 - 05:59	Morning	37,0	65	34,6	34,6	18,16	561,12	119,10
2019/05/19	CH2205	Male	Three y/o	Treatment (Wk 1)	Treatment	Fast Day	No	05:40	05:00 - 05:59	Morning	37,3	40	26,6	26,6	18,46	520,09	110,07
2019/05/19	CH2206	Male	Three y/o	Treatment (Wk 1)	Treatment	Fast Day	No	05:40	05:00 - 05:59	Morning	37,0	45	40,2	40,2	18,16	530,47	124,26
2019/05/19	CH2205	Male	Three y/o	Treatment (Wk 1)	Treatment	Fast Day	No	05:45	05:00 - 05:59	Morning	37,3	68	28,0	28,0	18,46	564,71	111,83
2019/05/19	CH2206	Male	Three y/o	Treatment (Wk 1)	Treatment	Fast Day	No	05:45	05:00 - 05:59	Morning	37,1	48	38,0	38,0	18,26	536,04	122,32
2019/05/19	CH2205	Male	Three y/o	Treatment (Wk 1)	Treatment	Fast Day	No	05:50	05:00 - 05:59	Morning	37,3		30,0	30,0	18,46		114,20
2019/05/19	CH2206	Male	Three y/o	Treatment (Wk 1)	Treatment	Fast Day	No	05:50	05:00 - 05:59	Morning	37,1	75	39,0	39,0	18,26	572,36	123,22
2019/05/19	CH2205	Male	Three y/o	Treatment (Wk 1)	Treatment	Fast Day	No	05:55	05:00 - 05:59	Morning	37,4	112	27,4	27,4	18,55	601,96	111,09

2019/05/19	CH2206	Male	Three y/o	Treatment (Wk 1)	Treatment	Fast Day	No	05:55	05:00 - 05:59	Morning	37,1	78	26,2	26,2	18,26	575,38	109,55
2019/05/19	CH2205	Male	Three y/o	Treatment (Wk 1)	Treatment	Fast Day	No	06:00	06:00 - 06:59	Morning	37,4	46	2,2	2,2	18,55	532,38	26,02
2019/05/19	CH2206	Male	Three y/o	Treatment (Wk 1)	Treatment	Fast Day	No	06:00	06:00 - 06:59	Morning	37,1	50	30,0	30,0	18,26	539,52	114,20
2019/05/19	CH2205	Male	Three y/o	Treatment (Wk 1)	Treatment	Fast Day	No	06:05	06:00 - 06:59	Morning	37,2	66	6,2	6,2	18,36	562,34	60,63
2019/05/19	CH2206	Male	Three y/o	Treatment (Wk 1)	Treatment	Fast Day	No	06:05	06:00 - 06:59	Morning	37,1	50	22,2	22,2	18,26	539,52	103,88
2019/05/19	CH2205	Male	Three y/o	Treatment (Wk 1)	Treatment	Fast Day	No	06:10	06:00 - 06:59	Morning	37,2	50	10,8	10,8	18,36	539,52	79,36
2019/05/19	CH2206	Male	Three y/o	Treatment (Wk 1)	Treatment	Fast Day	No	06:10	06:00 - 06:59	Morning	37,1	82	41,6	41,6	18,26	579,19	125,44
2019/05/19	CH2205	Male	Three y/o	Treatment (Wk 1)	Treatment	Fast Day	No	06:15	06:00 - 06:59	Morning	37,2	55	12,6	12,6	18,36	547,52	84,59
2019/05/19	CH2206	Male	Three y/o	Treatment (Wk 1)	Treatment	Fast Day	No	06:15	06:00 - 06:59	Morning	37,2	48	44,4	44,4	18,36	536,04	127,69
2019/05/19	CH2205	Male	Three y/o	Treatment (Wk 1)	Treatment	Fast Day	No	06:20	06:00 - 06:59	Morning	37,3	56	12,2	12,2	18,46	549,01	83,49
2019/05/19	CH2206	Male	Three y/o	Treatment (Wk 1)	Treatment	Fast Day	No	06:20	06:00 - 06:59	Morning	37,2	79	39,8	39,8	18,36	576,36	123,91
2019/05/19	CH2205	Male	Three y/o	Treatment (Wk 1)	Treatment	Fast Day	No	06:25	06:00 - 06:59	Morning	37,3	86	17,0	17,0	18,46	582,77	94,77
2019/05/19	CH2206	Male	Three y/o	Treatment (Wk 1)	Treatment	Fast Day	No	06:25	06:00 - 06:59	Morning	37,2	86	33,6	33,6	18,36	582,77	118,09
2019/05/19	CH2205	Male	Three y/o	Treatment (Wk 1)	Treatment	Fast Day	No	06:30	06:00 - 06:59	Morning	37,3	113	124,2	124,2	18,46	602,58	163,41
2019/05/19	CH2206	Male	Three y/o	Treatment (Wk 1)	Treatment	Fast Day	No	06:30	06:00 - 06:59	Morning	37,3	51	108,0	108,0	18,46	541,20	158,53
2019/05/19	CH2205	Male	Three y/o	Treatment (Wk 1)	Treatment	Fast Day	No	06:35	06:00 - 06:59	Morning	37,3	182	192,2	192,2	18,46	634,31	178,72
2019/05/19	CH2206	Male	Three y/o	Treatment (Wk 1)	Treatment	Fast Day	No	06:35	06:00 - 06:59	Morning	37,4	128	148,4	148,4	18,55	611,22	169,64
2019/05/19	CH2205	Male	Three y/o	Treatment (Wk 1)	Treatment	Fast Day	No	06:40	06:00 - 06:59	Morning	37,3	101	129,6	129,6	18,46	594,59	164,90
2019/05/19	CH2206	Male	Three y/o	Treatment (Wk 1)	Treatment	Fast Day	No	06:40	06:00 - 06:59	Morning	37,4	162	396,8	396,8	18,55	626,88	204,33
2019/05/19	CH2205	Male	Three y/o	Treatment (Wk 1)	Treatment	Fast Day	No	06:45	06:00 - 06:59	Morning	37,3	109	171,2	171,2	18,46	600,04	174,65
2019/05/19	CH2206	Male	Three y/o	Treatment (Wk 1)	Treatment	Fast Day	No	06:45	06:00 - 06:59	Morning	37,5		95,4	95,4	18,65		154,20
2019/05/19	CH2205	Male	Three y/o	Treatment (Wk 1)	Treatment	Fast Day	No	06:50	06:00 - 06:59	Morning	37,4	114	145,2	145,2	18,55	603,20	168,88
2019/05/19	CH2206	Male	Three y/o	Treatment (Wk 1)	Treatment	Fast Day	No	06:50	06:00 - 06:59	Morning	37,7	118	413,4	413,4	18,85	605,61	205,78
2019/05/19	CH2205	Male	Three y/o	Treatment (Wk 1)	Treatment	Fast Day	No	06:55	06:00 - 06:59	Morning	37,4		160,2	160,2	18,55		172,32
2019/05/19	CH2206	Male	Three y/o	Treatment (Wk 1)	Treatment	Fast Day	No	06:55	06:00 - 06:59	Morning	37,8	128	295,2	295,2	18,95	611,22	193,85
2019/05/19	CH2205	Male	Three y/o	Treatment (Wk 1)	Treatment	Fast Day	No	07:00	07:00 - 07:59	Morning	37,5	103	138,2	138,2	18,65	596,00	167,15
2019/05/19	CH2206	Male	Three y/o	Treatment (Wk 1)	Treatment	Fast Day	No	07:00	07:00 - 07:59	Morning	37,9	110	237,0	237,0	19,05	600,69	186,10
2019/05/19	CH2205	Male	Three y/o	Treatment (Wk 1)	Treatment	Fast Day	No	07:05	07:00 - 07:59	Morning	37,5	98	82,8	82,8	18,65	592,41	149,27
2019/05/19	CH2206	Male	Three y/o	Treatment (Wk 1)	Treatment	Fast Day	No	07:05	07:00 - 07:59	Morning	37,9		725,4	725,4	19,05		225,83
2019/05/19	CH2205	Male	Three y/o	Treatment (Wk 1)	Treatment	Fast Day	No	07:10	07:00 - 07:59	Morning	37,7	71	165,4	165,4	18,85	568,10	173,44
2019/05/19	CH2206	Male	Three y/o	Treatment (Wk 1)	Treatment	Fast Day	No	07:10	07:00 - 07:59	Morning	38,0	134	71,4	71,4	19,15	614,33	144,12
2019/05/19	CH2205	Male	Three y/o	Treatment (Wk 1)	Treatment	Fast Day	No	07:15	07:00 - 07:59	Morning	37,9	59	225,2	225,2	19,05	553,30	184,30
2019/05/19	CH2206	Male	Three y/o	Treatment (Wk 1)	Treatment	Fast Day	No	07:15	07:00 - 07:59	Morning	38,1	117	233,6	233,6	19,26	605,02	185,59
2019/05/19	CH2205	Male	Three y/o	Treatment (Wk 1)	Treatment	Fast Day	No	07:20	07:00 - 07:59	Morning	38,0		199,8	199,8	19,15		180,08
2019/05/19	CH2206	Male	Three y/o	Treatment (Wk 1)	Treatment	Fast Day	No	07:20	07:00 - 07:59	Morning	38,1	131	164,2	164,2	19,26	612,80	173,19
2019/05/19	CH2205	Male	Three y/o	Treatment (Wk 1)	Treatment	Fast Day	No	07:25	07:00 - 07:59	Morning	38,1		118,0	118,0	19,26		161,62
2019/05/19	CH2206	Male	Three y/o	Treatment (Wk 1)	Treatment	Fast Day	No	07:25	07:00 - 07:59	Morning	38,1	126	131,4	131,4	19,26	610,14	165,38
2019/05/19	CH2205	Male	Three y/o	Treatment (Wk 1)	Treatment	Fast Day	No	07:30	07:00 - 07:59	Morning	38,1	89	41,6	41,6	19,26	585,33	125,44
2019/05/19	CH2206	Male	Three y/o	Treatment (Wk 1)	Treatment	Fast Day	No	07:30	07:00 - 07:59	Morning	38,3		133,4	133,4	19,46		165,91
2019/05/19	CH2205	Male	Three y/o	Treatment (Wk 1)	Treatment	Fast Day	No	07:35	07:00 - 07:59	Morning	37,7	160	79,0	79,0	18,85	626,07	147,64
2019/05/19	CH2206	Male	Three y/o	Treatment (Wk 1)	Treatment	Fast Day	No	07:35	07:00 - 07:59	Morning	38,2	124	146,2	146,2	19,36	609,04	169,12
2019/05/19	CH2205	Male	Three y/o	Treatment (Wk 1)	Treatment	Fast Day	No	07:40	07:00 - 07:59	Morning	37,5		10,0	10,0	18,65		76,76
2019/05/19	CH2206	Male	Three y/o	Treatment (Wk 1)	Treatment	Fast Day	No	07:40	07:00 - 07:59	Morning	38,1	104	74,2	74,2	19,26	596,69	145,46
2019/05/19	CH2205	Male	Three y/o	Treatment (Wk 1)	Treatment	Fast Day	No	07:45	07:00 - 07:59	Morning	37,3		108,0	108,0	18,46		158,53
2019/05/19	CH2206	Male	Three y/o	Treatment (Wk 1)	Treatment	Fast Day	No	07:45	07:00 - 07:59	Morning	38,1	129	125,2	125,2	19,26	611,75	163,69
2019/05/19	CH2205	Male	Three y/o	Treatment (Wk 1)	Treatment	Fast Day	No	07:50	07:00 - 07:59	Morning	37,4	97	242,6	242,6	18,55	591,66	186,92
2019/05/19	CH2206	Male	Three y/o	Treatment (Wk 1)	Treatment	Fast Day	No	07:50	07:00 - 07:59	Morning	38,1	158	263,6	263,6	19,26	625,26	189,85
2019/05/19	CH2205	Male	Three y/o	Treatment (Wk 1)	Treatment	Fast Day	No	07:55	07:00 - 07:59	Morning	37,5	108	33,4	33,4	18,65	599,39	117,88
2019/05/19	CH2206	Male	Three y/o	Treatment (Wk 1)	Treatment	Fast Day	No	07:55	07:00 - 07:59	Morning	38,0	110	465,8	465,8	19,15	600,69	210,03
2019/05/19	CH2205	Male	Three y/o	Treatment (Wk 1)	Treatment	Fast Day	No	08:00	08:00 - 08:59	Late Morning	37,6	101	320,0	320,0	18,75	594,59	196,70
2019/05/19	CH2206	Male	Three y/o	Treatment (Wk 1)	Treatment	Fast Day	No	08:00	08:00 - 08:59	Late Morning	38,0	106	128,6	128,6	19,15	598,06	164,63
2019/05/19	CH2205	Male	Three y/o	Treatment (Wk 1)	Treatment	Fast Day	No	08:05	08:00 - 08:59	Late Morning	37,6	106	127,4	127,4	18,75	598,06	164,30
2019/05/19	CH2206	Male	Three y/o	Treatment (Wk 1)	Treatment	Fast Day	No	08:05	08:00 - 08:59	Late Morning	38,0	118	206,8	206,8	19,15	605,61	181,30
2019/05/19	CH2205	Male	Three y/o	Treatment (Wk 1)	Treatment	Fast Day	No	08:10	08:00 - 08:59	Late Morning	37,6	54	106,4	106,4	18,75	545,99	158,01
2019/05/19	CH2206	Male	Three y/o	Treatment (Wk 1)	Treatment	Fast Day	No	08:10	08:00 - 08:59	Late Morning	38,0	131	134,8	134,8	19,15	612,80	166,28
2019/05/19	CH2205	Male	Three y/o	Treatment (Wk 1)	Treatment	Fast Day	No	08:15	08:00 - 08:59	Late Morning	37,7	75	99,2	99,2	18,85	572,36	155,56
2019/05/19	CH2206	Male	Three y/o	Treatment (Wk 1)	Treatment	Fast Day	No	08:15	08:00 - 08:59	Late Morning	38,0	114	130,6	130,6	19,15	603,20	165,17
2019/05/19	CH2205	Male	Three y/o	Treatment (Wk 1)	Treatment	Fast Day	No	08:20	08:00 - 08:59	Late Morning	37,7		131,8	131,8	18,85		165,49
2019/05/19	CH2206	Male	Three y/o	Treatment (Wk 1)	Treatment	Fast Day	No	08:20	08:00 - 08:59	Late Morning	37,9		176,4	176,4	19,05		175,71
2019/05/19	CH2205	Male	Three y/o	Treatment (Wk 1)	Treatment	Fast Day	No	08:25	08:00 - 08:59	Late Morning	37,7	51	120,2	120,2	18,85	541,20	162,27
2019/05/19	CH2206	Male	Three y/o	Treatment (Wk 1)	Treatment	Fast Day	No	08:25	08:00 - 08:59	Late Morning	37,9	114	154,4	154,4	19,05	603,20	171,03
2019/05/19	CH2205	Male	Three y/o	Treatment (Wk 1)	Treatment	Fast Day	No	08:30	08:00 - 08:59	Late Morning	37,7		212,4	212,4	18,85		182,24
2019/05/19	CH2206	Male	Three y/o	Treatment (Wk 1)	Treatment	Fast Day	No	08:30	08:00 - 08:59	Late Morning	37,9	115	523,8	523,8	19,05	603,82	214,20
2019/05/19	CH2205	Male	Three y/o	Treatment (Wk 1)	Treatment	Fast Day	No	08:35	08:00 - 08:59	Late Morning	37,7	83	114,0	114,0	18,85	580,11	160,42
2019/05/19	CH2206	Male	Three y/o	Treatment (Wk 1)	Treatment	Fast Day	No	08:35	08:00 - 08:59	Late Morning	37,9	111	84,4	84,4	19,05	601,33	149,94
2019/05/19	CH2205	Male	Three y/o	Treatment (Wk 1)	Treatment	Fast Day	No	08:40	08:00 - 08:59	Late Morning	37,8	66	14,4	14,4	18,95	562,34	89,12
2019/05/19	CH2206	Male	Three y/o	Treatment (Wk 1)	Treatment	Fast Day	No	08:40	08:00 - 08:59	Late Morning	37,9	112	130,2	130,2	19,05	601,96	165,06
2019/05/19	CH2205	Male	Three y/o	Treatment (Wk 1)	Treatment	Fast Day	No	08:45	08:00 - 08:59	Late Morning	37,9	108	129,0	129,0	19,05	599,39	164,74
2019/05/19	CH2206	Male	Three y/o	Treatment (Wk 1)	Treatment	Fast Day	No	08:45	08:00 - 08:59	Late Morning	37,9	110	87,2	87,2	19,05	600,69	151,07
2019/05/19	CH2205	Male	Three y/o	Treatment (Wk 1)	Treatment	Fast Day	No	08:50	08:00 - 08:59	Late Morning	37,9		63,6	63,6	19,05		140,11

2019/05/19	CH2206	Male	Three y/o	Treatment (Wk 1)	Treatment	Fast Day	No	08:50	08:00 - 08:59	Late Morning	37,9	58	155,6	155,6	19,05	551,90	171,30
2019/05/19	CH2205	Male	Three y/o	Treatment (Wk 1)	Treatment	Fast Day	No	08:55	08:00 - 08:59	Late Morning	37,7	82	470,0	470,0	18,85	579,19	210,34
2019/05/19	CH2206	Male	Three y/o	Treatment (Wk 1)	Treatment	Fast Day	No	08:55	08:00 - 08:59	Late Morning	37,9	115	655,4	655,4	19,05	603,82	222,20
2019/05/19	CH2205	Male	Three y/o	Treatment (Wk 1)	Treatment	Fast Day	No	09:00	09:00 - 09:59	Late Morning	37,7	56	185,0	185,0	18,85	549,01	177,38
2019/05/19	CH2206	Male	Three y/o	Treatment (Wk 1)	Treatment	Fast Day	No	09:00	09:00 - 09:59	Late Morning	37,9	126	290,2	290,2	19,05	610,14	193,25
2019/05/19	CH2205	Male	Three y/o	Treatment (Wk 1)	Treatment	Fast Day	No	09:05	09:00 - 09:59	Late Morning	37,7		40,4	40,4	18,85		124,43
2019/05/19	CH2206	Male	Three y/o	Treatment (Wk 1)	Treatment	Fast Day	No	09:05	09:00 - 09:59	Late Morning	37,9	117	103,2	103,2	19,05	605,02	156,94
2019/05/19	CH2205	Male	Three y/o	Treatment (Wk 1)	Treatment	Fast Day	No	09:10	09:00 - 09:59	Late Morning	37,6	100	72,6	72,6	18,75	593,87	144,70
2019/05/19	CH2206	Male	Three y/o	Treatment (Wk 1)	Treatment	Fast Day	No	09:10	09:00 - 09:59	Late Morning	37,9	115	94,2	94,2	19,05	603,82	153,76
2019/05/19	CH2205	Male	Three y/o	Treatment (Wk 1)	Treatment	Fast Day	No	09:15	09:00 - 09:59	Late Morning	37,6	80	27,0	27,0	18,75	577,31	110,58
2019/05/19	CH2206	Male	Three y/o	Treatment (Wk 1)	Treatment	Fast Day	No	09:15	09:00 - 09:59	Late Morning	37,9	129	110,4	110,4	19,05	611,75	159,30
2019/05/19	CH2205	Male	Three y/o	Treatment (Wk 1)	Treatment	Fast Day	No	09:20	09:00 - 09:59	Late Morning	37,6	120	60,4	60,4	18,75	606,78	138,32
2019/05/19	CH2206	Male	Three y/o	Treatment (Wk 1)	Treatment	Fast Day	No	09:20	09:00 - 09:59	Late Morning	37,9	121	154,2	154,2	19,05	607,35	170,99
2019/05/19	CH2205	Male	Three y/o	Treatment (Wk 1)	Treatment	Fast Day	No	09:25	09:00 - 09:59	Late Morning	37,5		267,0	267,0	18,65		190,30
2019/05/19	CH2206	Male	Three y/o	Treatment (Wk 1)	Treatment	Fast Day	No	09:25	09:00 - 09:59	Late Morning	37,9	91	218,0	218,0	19,05	586,98	183,15
2019/05/19	CH2205	Male	Three y/o	Treatment (Wk 1)	Treatment	Fast Day	No	09:30	09:00 - 09:59	Late Morning	37,6	119	345,6	345,6	18,75	606,20	199,43
2019/05/19	CH2206	Male	Three y/o	Treatment (Wk 1)	Treatment	Fast Day	No	09:30	09:00 - 09:59	Late Morning	37,9	141	186,8	186,8	19,05	617,75	177,72
2019/05/19	CH2205	Male	Three y/o	Treatment (Wk 1)	Treatment	Fast Day	No	09:35	09:00 - 09:59	Late Morning	37,7		138,4	138,4	18,85		167,20
2019/05/19	CH2206	Male	Three y/o	Treatment (Wk 1)	Treatment	Fast Day	No	09:35	09:00 - 09:59	Late Morning	37,9	118	136,6	136,6	19,05	605,61	166,74
2019/05/19	CH2205	Male	Three y/o	Treatment (Wk 1)	Treatment	Fast Day	No	09:40	09:00 - 09:59	Late Morning	37,7	106	25,8	25,8	18,85	598,06	109,02
2019/05/19	CH2206	Male	Three y/o	Treatment (Wk 1)	Treatment	Fast Day	No	09:40	09:00 - 09:59	Late Morning	37,9	180	140,0	140,0	19,05	633,61	167,60
2019/05/19	CH2205	Male	Three y/o	Treatment (Wk 1)	Treatment	Fast Day	No	09:45	09:00 - 09:59	Late Morning	37,7		242,8	242,8	18,85		186,95
2019/05/19	CH2206	Male	Three y/o	Treatment (Wk 1)	Treatment	Fast Day	No	09:45	09:00 - 09:59	Late Morning	37,9	119	127,6	127,6	19,05	606,20	164,35
2019/05/19	CH2205	Male	Three y/o	Treatment (Wk 1)	Treatment	Fast Day	No	09:50	09:00 - 09:59	Late Morning	37,7	128	50,2	50,2	18,85	611,22	131,92
2019/05/19	CH2206	Male	Three y/o	Treatment (Wk 1)	Treatment	Fast Day	No	09:50	09:00 - 09:59	Late Morning	37,8	111	130,6	130,6	18,95	601,33	165,17
2019/05/19	CH2205	Male	Three y/o	Treatment (Wk 1)	Treatment	Fast Day	No	09:55	09:00 - 09:59	Late Morning	37,8	103	76,8	76,8	18,95	596,00	146,66
2019/05/19	CH2206	Male	Three y/o	Treatment (Wk 1)	Treatment	Fast Day	No	09:55	09:00 - 09:59	Late Morning	37,9	57	60,0	60,0	19,05	550,47	138,09
2019/05/19	CH2205	Male	Three y/o	Treatment (Wk 1)	Treatment	Fast Day	No	10:00	10:00 - 10:59	Late Morning	37,7		323,2	323,2	18,85		197,06
2019/05/19	CH2206	Male	Three y/o	Treatment (Wk 1)	Treatment	Fast Day	No	10:00	10:00 - 10:59	Late Morning	37,9	49	126,8	126,8	19,05	537,80	164,13
2019/05/19	CH2205	Male	Three y/o	Treatment (Wk 1)	Treatment	Fast Day	No	10:05	10:00 - 10:59	Late Morning	37,9	97	24,4	24,4	19,05	591,66	107,11
2019/05/19	CH2206	Male	Three y/o	Treatment (Wk 1)	Treatment	Fast Day	No	10:05	10:00 - 10:59	Late Morning	38,0	54	110,0	110,0	19,15	545,99	159,17
2019/05/19	CH2205	Male	Three y/o	Treatment (Wk 1)	Treatment	Fast Day	No	10:10	10:00 - 10:59	Late Morning	37,7	113	66,2	66,2	18,85	602,58	141,50
2019/05/19	CH2206	Male	Three y/o	Treatment (Wk 1)	Treatment	Fast Day	No	10:10	10:00 - 10:59	Late Morning	37,9	118	84,0	84,0	19,05	605,61	149,77
2019/05/19	CH2205	Male	Three y/o	Treatment (Wk 1)	Treatment	Fast Day	No	10:15	10:00 - 10:59	Late Morning	37,9	109	110,2	110,2	19,05	600,04	159,23
2019/05/19	CH2206	Male	Three y/o	Treatment (Wk 1)	Treatment	Fast Day	No	10:15	10:00 - 10:59	Late Morning	37,9	143	225,4	225,4	19,05	618,69	184,33
2019/05/19	CH2205	Male	Three y/o	Treatment (Wk 1)	Treatment	Fast Day	No	10:20	10:00 - 10:59	Late Morning	37,9	54	99,0	99,0	19,05	545,99	155,49
2019/05/19	CH2206	Male	Three y/o	Treatment (Wk 1)	Treatment	Fast Day	No	10:20	10:00 - 10:59	Late Morning	38,0	126	196,2	196,2	19,15	610,14	179,44
2019/05/19	CH2205	Male	Three y/o	Treatment (Wk 1)	Treatment	Fast Day	No	10:25	10:00 - 10:59	Late Morning	37,7		36,4	36,4	18,85		120,84
2019/05/19	CH2206	Male	Three y/o	Treatment (Wk 1)	Treatment	Fast Day	No	10:25	10:00 - 10:59	Late Morning	38,0	119	33,8	33,8	19,15	606,20	118,29
2019/05/19	CH2205	Male	Three y/o	Treatment (Wk 1)	Treatment	Fast Day	No	10:30	10:00 - 10:59	Late Morning	37,6	63	36,2	36,2	18,75	558,62	120,65
2019/05/19	CH2206	Male	Three y/o	Treatment (Wk 1)	Treatment	Fast Day	No	10:30	10:00 - 10:59	Late Morning	38,0	78	27,8	27,8	19,15	575,38	111,58
2019/05/19	CH2205	Male	Three y/o	Treatment (Wk 1)	Treatment	Fast Day	No	10:35	10:00 - 10:59	Late Morning	37,6	60	35,4	35,4	18,75	554,67	119,88
2019/05/19	CH2206	Male	Three y/o	Treatment (Wk 1)	Treatment	Fast Day	No	10:35	10:00 - 10:59	Late Morning	37,7	62	30,2	30,2	18,85	557,33	114,42
2019/05/19	CH2205	Male	Three y/o	Treatment (Wk 1)	Treatment	Fast Day	No	10:40	10:00 - 10:59	Late Morning	37,5		12,6	12,6	18,65		84,59
2019/05/19	CH2206	Male	Three y/o	Treatment (Wk 1)	Treatment	Fast Day	No	10:40	10:00 - 10:59	Late Morning	37,4	48	20,2	20,2	18,55	536,04	100,65
2019/05/19	CH2205	Male	Three y/o	Treatment (Wk 1)	Treatment	Fast Day	No	10:45	10:00 - 10:59	Late Morning	37,5	55	16,0	16,0	18,65	547,52	92,71
2019/05/19	CH2206	Male	Three y/o	Treatment (Wk 1)	Treatment	Fast Day	No	10:45	10:00 - 10:59	Late Morning	37,4	48	3,4	3,4	18,55	536,04	40,51
2019/05/19	CH2205	Male	Three y/o	Treatment (Wk 1)	Treatment	Fast Day	No	10:50	10:00 - 10:59	Late Morning	37,5	50	14,8	14,8	18,65	539,52	90,05
2019/05/19	CH2206	Male	Three y/o	Treatment (Wk 1)	Treatment	Fast Day	No	10:50	10:00 - 10:59	Late Morning	37,3	95	20,6	20,6	18,46	590,14	101,32
2019/05/19	CH2205	Male	Three y/o	Treatment (Wk 1)	Treatment	Fast Day	No	10:55	10:00 - 10:59	Late Morning	37,5	51	20,0	20,0	18,65	541,20	100,31
2019/05/19	CH2206	Male	Three y/o	Treatment (Wk 1)	Treatment	Fast Day	No	10:55	10:00 - 10:59	Late Morning	37,4	47	28,2	28,2	18,55	534,23	112,07
2019/05/19	CH2205	Male	Three y/o	Treatment (Wk 1)	Treatment	Fast Day	No	11:00	11:00 - 11:59	Late Morning	37,5	73	43,0	43,0	18,65	570,27	126,58
2019/05/19	CH2206	Male	Three y/o	Treatment (Wk 1)	Treatment	Fast Day	No	11:00	11:00 - 11:59	Late Morning	37,5	115	59,8	59,8	18,65	603,82	137,98
2019/05/19	CH2205	Male	Three y/o	Treatment (Wk 1)	Treatment	Fast Day	No	11:05	11:00 - 11:59	Late Morning	37,4	104	74,2	74,2	18,55	596,69	145,46
2019/05/19	CH2206	Male	Three y/o	Treatment (Wk 1)	Treatment	Fast Day	No	11:05	11:00 - 11:59	Late Morning	37,5	114	137,6	137,6	18,65	603,20	166,99
2019/05/19	CH2205	Male	Three y/o	Treatment (Wk 1)	Treatment	Fast Day	No	11:10	11:00 - 11:59	Late Morning	37,4	46	353,8	353,8	18,55	532,38	200,26
2019/05/19	CH2206	Male	Three y/o	Treatment (Wk 1)	Treatment	Fast Day	No	11:10	11:00 - 11:59	Late Morning	37,5	114	116,0	116,0	18,65	603,20	161,02
2019/05/19	CH2205	Male	Three y/o	Treatment (Wk 1)	Treatment	Fast Day	No	11:15	11:00 - 11:59	Late Morning	37,4	103	184,2	184,2	18,55	596,00	177,23
2019/05/19	CH2206	Male	Three y/o	Treatment (Wk 1)	Treatment	Fast Day	No	11:15	11:00 - 11:59	Late Morning	37,7	107	122,6	122,6	18,85	598,73	162,96
2019/05/19	CH2205	Male	Three y/o	Treatment (Wk 1)	Treatment	Fast Day	No	11:20	11:00 - 11:59	Late Morning	37,4	98	94,8	94,8	18,55	592,41	153,98
2019/05/19	CH2206	Male	Three y/o	Treatment (Wk 1)	Treatment	Fast Day	No	11:20	11:00 - 11:59	Late Morning	37,7	105	191,4	191,4	18,85	597,38	178,57
2019/05/19	CH2205	Male	Three y/o	Treatment (Wk 1)	Treatment	Fast Day	No	11:25	11:00 - 11:59	Late Morning	37,5	109	93,4	93,4	18,65	600,04	153,46
2019/05/19	CH2206	Male	Three y/o	Treatment (Wk 1)	Treatment	Fast Day	No	11:25	11:00 - 11:59	Late Morning	37,7	101	61,4	61,4	18,85	594,59	138,89
2019/05/19	CH2205	Male	Three y/o	Treatment (Wk 1)	Treatment	Fast Day	No	11:30	11:00 - 11:59	Late Morning	37,6		51,2	51,2	18,75		132,61
2019/05/19	CH2206	Male	Three y/o	Treatment (Wk 1)	Treatment	Fast Day	No	11:30	11:00 - 11:59	Late Morning	37,7	117	120,6	120,6	18,85	605,02	162,38
2019/05/19	CH2205	Male	Three y/o	Treatment (Wk 1)	Treatment	Fast Day	No	11:35	11:00 - 11:59	Late Morning	37,5	53	783,8	783,8	18,65	544,43	228,60
2019/05/19	CH2206	Male	Three y/o	Treatment (Wk 1)	Treatment	Fast Day	No	11:35	11:00 - 11:59	Late Morning	37,8		1206,6		18,95		
2019/05/19	CH2205	Male	Three y/o	Treatment (Wk 1)	Treatment	Fast Day	No	11:40	11:00 - 11:59	Late Morning	38,0		151,6	151,6	19,15		170,39
2019/05/19	CH2206	Male	Three y/o	Treatment (Wk 1)	Treatment	Fast Day	No	11:40	11:00 - 11:59	Late Morning	37,9	119	65,4	65,4	19,05	606,20	141,08
2019/05/19	CH2205	Male	Three y/o	Treatment (Wk 1)	Treatment	Fast Day	No	11:45	11:00 - 11:59	Late Morning	38,0	73	407,8	407,8	19,15	570,27	205,30

2019/05/19	CH2206	Male	Three y/o	Treatment (Wk 1)	Treatment	Fast Day	No	11:45	11:00 - 11:59	Late Morning	37,9	150	459,8	459,8	19,05	621,86	209,56
2019/05/19	CH2205	Male	Three y/o	Treatment (Wk 1)	Treatment	Fast Day	No	11:50	11:00 - 11:59	Late Morning	38,0	89	169,8	169,8	19,15	585,33	174,37
2019/05/19	CH2206	Male	Three y/o	Treatment (Wk 1)	Treatment	Fast Day	No	11:50	11:00 - 11:59	Late Morning	37,9		79,2	79,2	19,05		147,73
2019/05/19	CH2205	Male	Three y/o	Treatment (Wk 1)	Treatment	Fast Day	No	11:55	11:00 - 11:59	Late Morning	38,0	111	86,0	86,0	19,15	601,33	150,59
2019/05/19	CH2206	Male	Three y/o	Treatment (Wk 1)	Treatment	Fast Day	No	11:55	11:00 - 11:59	Late Morning	38,0	115	439,4	439,4	19,15	603,82	207,95
2019/05/19	CH2205	Male	Three y/o	Treatment (Wk 1)	Treatment	Fast Day	No	12:00	12:00 - 12:59	Afternoon	38,0	84	254,2	254,2	19,15	581,01	188,57
2019/05/19	CH2206	Male	Three y/o	Treatment (Wk 1)	Treatment	Fast Day	No	12:00	12:00 - 12:59	Afternoon	38,0	131	152,0	152,0	19,15	612,80	170,48
2019/05/19	CH2205	Male	Three y/o	Treatment (Wk 1)	Treatment	Fast Day	No	12:05	12:00 - 12:59	Afternoon	38,0	119	145,0	145,0	19,15	606,20	168,83
2019/05/19	CH2206	Male	Three y/o	Treatment (Wk 1)	Treatment	Fast Day	No	12:05	12:00 - 12:59	Afternoon	38,0	141	74,8	74,8	19,15	617,75	145,74
2019/05/19	CH2205	Male	Three y/o	Treatment (Wk 1)	Treatment	Fast Day	No	12:10	12:00 - 12:59	Afternoon	38,0	191	137,0	137,0	19,15	637,33	166,84
2019/05/19	CH2206	Male	Three y/o	Treatment (Wk 1)	Treatment	Fast Day	No	12:10	12:00 - 12:59	Afternoon	38,0	112	112,0	112,0	19,15	601,96	159,80
2019/05/19	CH2205	Male	Three y/o	Treatment (Wk 1)	Treatment	Fast Day	No	12:15	12:00 - 12:59	Afternoon	38,0	87	142,2	142,2	19,15	583,64	168,15
2019/05/19	CH2206	Male	Three y/o	Treatment (Wk 1)	Treatment	Fast Day	No	12:15	12:00 - 12:59	Afternoon	38,0	124	329,4	329,4	19,15	609,04	197,73
2019/05/19	CH2205	Male	Three y/o	Treatment (Wk 1)	Treatment	Fast Day	No	12:20	12:00 - 12:59	Afternoon	38,0		325,4	325,4	19,15		197,30
2019/05/19	CH2206	Male	Three y/o	Treatment (Wk 1)	Treatment	Fast Day	No	12:20	12:00 - 12:59	Afternoon	38,0		69,4	69,4	19,15		143,14
2019/05/19	CH2205	Male	Three y/o	Treatment (Wk 1)	Treatment	Fast Day	No	12:25	12:00 - 12:59	Afternoon	38,3		28,0	28,0	19,46		111,83
2019/05/19	CH2206	Male	Three y/o	Treatment (Wk 1)	Treatment	Fast Day	No	12:25	12:00 - 12:59	Afternoon	38,1	117	127,4	127,4	19,26	605,02	164,30
2019/05/19	CH2205	Male	Three y/o	Treatment (Wk 1)	Treatment	Fast Day	No	12:30	12:00 - 12:59	Afternoon	38,3	82	5,2	5,2	19,46	579,19	54,72
2019/05/19	CH2206	Male	Three y/o	Treatment (Wk 1)	Treatment	Fast Day	No	12:30	12:00 - 12:59	Afternoon	38,1	106	39,6	39,6	19,26	598,06	123,74
2019/05/19	CH2205	Male	Three y/o	Treatment (Wk 1)	Treatment	Fast Day	No	12:35	12:00 - 12:59	Afternoon	38,3	63	74,6	74,6	19,46	558,62	145,65
2019/05/19	CH2206	Male	Three y/o	Treatment (Wk 1)	Treatment	Fast Day	No	12:35	12:00 - 12:59	Afternoon	38,1	73	36,0	36,0	19,26	570,27	120,46
2019/05/19	CH2205	Male	Three y/o	Treatment (Wk 1)	Treatment	Fast Day	No	12:40	12:00 - 12:59	Afternoon	38,3	101	132,4	132,4	19,46	594,59	165,65
2019/05/19	CH2206	Male	Three y/o	Treatment (Wk 1)	Treatment	Fast Day	No	12:40	12:00 - 12:59	Afternoon	38,0	112	104,2	104,2	19,15	601,96	157,28
2019/05/19	CH2205	Male	Three y/o	Treatment (Wk 1)	Treatment	Fast Day	No	12:45	12:00 - 12:59	Afternoon	38,3	101	32,4	32,4	19,46	594,59	116,84
2019/05/19	CH2206	Male	Three y/o	Treatment (Wk 1)	Treatment	Fast Day	No	12:45	12:00 - 12:59	Afternoon	38,1	126	118,6	118,6	19,26	610,14	161,80
2019/05/19	CH2205	Male	Three y/o	Treatment (Wk 1)	Treatment	Fast Day	No	12:50	12:00 - 12:59	Afternoon	38,1	138	30,2	30,2	19,26	616,31	114,42
2019/05/19	CH2206	Male	Three y/o	Treatment (Wk 1)	Treatment	Fast Day	No	12:50	12:00 - 12:59	Afternoon	38,0		84,8	84,8	19,15		150,10
2019/05/19	CH2205	Male	Three y/o	Treatment (Wk 1)	Treatment	Fast Day	No	12:55	12:00 - 12:59	Afternoon	38,1	63	20,4	20,4	19,26	558,62	100,99
2019/05/19	CH2206	Male	Three y/o	Treatment (Wk 1)	Treatment	Fast Day	No	12:55	12:00 - 12:59	Afternoon	38,0	131	293,8	293,8	19,15	612,80	193,68
2019/05/19	CH2205	Male	Three y/o	Treatment (Wk 1)	Treatment	Fast Day	No	13:00	13:00 - 13:59	Afternoon	38,1	86	50,4	50,4	19,26	582,77	132,06
2019/05/19	CH2206	Male	Three y/o	Treatment (Wk 1)	Treatment	Fast Day	No	13:00	13:00 - 13:59	Afternoon	38,0	147	447,8	447,8	19,15	620,52	208,62
2019/05/19	CH2205	Male	Three y/o	Treatment (Wk 1)	Treatment	Fast Day	No	13:05	13:00 - 13:59	Afternoon	38,1	64	9,0	9,0	19,26	559,88	73,19
2019/05/19	CH2206	Male	Three y/o	Treatment (Wk 1)	Treatment	Fast Day	No	13:05	13:00 - 13:59	Afternoon	38,0	80	37,8	37,8	19,15	577,31	122,14
2019/05/19	CH2205	Male	Three y/o	Treatment (Wk 1)	Treatment	Fast Day	No	13:10	13:00 - 13:59	Afternoon	38,1	61	11,0	11,0	19,26	556,01	79,98
2019/05/19	CH2206	Male	Three y/o	Treatment (Wk 1)	Treatment	Fast Day	No	13:10	13:00 - 13:59	Afternoon	38,0	70	25,8	25,8	19,15	566,99	109,02
2019/05/19	CH2205	Male	Three y/o	Treatment (Wk 1)	Treatment	Fast Day	No	13:15	13:00 - 13:59	Afternoon	38,1	49	6,0	6,0	19,26	537,80	59,53
2019/05/19	CH2206	Male	Three y/o	Treatment (Wk 1)	Treatment	Fast Day	No	13:15	13:00 - 13:59	Afternoon	38,0		69,6	69,6	19,15		143,24
2019/05/19	CH2205	Male	Three y/o	Treatment (Wk 1)	Treatment	Fast Day	No	13:20	13:00 - 13:59	Afternoon	38,0	71	2,8	2,8	19,15	568,10	34,04
2019/05/19	CH2206	Male	Three y/o	Treatment (Wk 1)	Treatment	Fast Day	No	13:20	13:00 - 13:59	Afternoon	38,0	74	21,2	21,2	19,15	571,33	102,31
2019/05/19	CH2205	Male	Three y/o	Treatment (Wk 1)	Treatment	Fast Day	No	13:25	13:00 - 13:59	Afternoon	38,0	50	9,8	9,8	19,15	539,52	76,07
2019/05/19	CH2206	Male	Three y/o	Treatment (Wk 1)	Treatment	Fast Day	No	13:25	13:00 - 13:59	Afternoon	38,0	61	47,0	47,0	19,15	556,01	129,65
2019/05/19	CH2205	Male	Three y/o	Treatment (Wk 1)	Treatment	Fast Day	No	13:30	13:00 - 13:59	Afternoon	38,0	104	25,4	25,4	19,15	596,69	108,49
2019/05/19	CH2206	Male	Three y/o	Treatment (Wk 1)	Treatment	Fast Day	No	13:30	13:00 - 13:59	Afternoon	38,0	83	88,2	88,2	19,15	580,11	151,47
2019/05/19	CH2205	Male	Three y/o	Treatment (Wk 1)	Treatment	Fast Day	No	13:35	13:00 - 13:59	Afternoon	38,0	56	10,0	10,0	19,15	549,01	76,76
2019/05/19	CH2206	Male	Three y/o	Treatment (Wk 1)	Treatment	Fast Day	No	13:35	13:00 - 13:59	Afternoon	38,0	69	33,6	33,6	19,15	565,86	118,09
2019/05/19	CH2205	Male	Three y/o	Treatment (Wk 1)	Treatment	Fast Day	No	13:40	13:00 - 13:59	Afternoon	37,9	46	7,8	7,8	19,05	532,38	68,36
2019/05/19	CH2206	Male	Three y/o	Treatment (Wk 1)	Treatment	Fast Day	No	13:40	13:00 - 13:59	Afternoon	38,0	52	36,6	36,6	19,15	542,83	121,03
2019/05/19	CH2205	Male	Three y/o	Treatment (Wk 1)	Treatment	Fast Day	No	13:45	13:00 - 13:59	Afternoon	37,9	56	10,4	10,4	19,05	549,01	78,08
2019/05/19	CH2206	Male	Three y/o	Treatment (Wk 1)	Treatment	Fast Day	No	13:45	13:00 - 13:59	Afternoon	37,9	47	59,8	59,8	19,05	534,23	137,98
2019/05/19	CH2205	Male	Three y/o	Treatment (Wk 1)	Treatment	Fast Day	No	13:50	13:00 - 13:59	Afternoon	37,9	52	11,8	11,8	19,05	542,83	82,36
2019/05/19	CH2206	Male	Three y/o	Treatment (Wk 1)	Treatment	Fast Day	No	13:50	13:00 - 13:59	Afternoon	37,9	59	62,8	62,8	19,05	553,30	139,67
2019/05/19	CH2205	Male	Three y/o	Treatment (Wk 1)	Treatment	Fast Day	No	13:55	13:00 - 13:59	Afternoon	37,9	53	3,4	3,4	19,05	544,43	40,51
2019/05/19	CH2206	Male	Three y/o	Treatment (Wk 1)	Treatment	Fast Day	No	13:55	13:00 - 13:59	Afternoon	38,0	67	64,8	64,8	19,15	563,53	140,76
2019/05/19	CH2205	Male	Three y/o	Treatment (Wk 1)	Treatment	Fast Day	No	14:00	14:00 - 14:59	Afternoon	37,7	48	6,2	6,2	18,85	536,04	60,63
2019/05/19	CH2206	Male	Three y/o	Treatment (Wk 1)	Treatment	Fast Day	No	14:00	14:00 - 14:59	Afternoon	38,0	64	48,2	48,2	19,15	559,88	130,52
2019/05/19	CH2205	Male	Three y/o	Treatment (Wk 1)	Treatment	Fast Day	No	14:05	14:00 - 14:59	Afternoon	37,7	69	9,2	9,2	18,85	565,86	73,94
2019/05/19	CH2206	Male	Three y/o	Treatment (Wk 1)	Treatment	Fast Day	No	14:05	14:00 - 14:59	Afternoon	38,0	72	19,6	19,6	19,15	569,20	99,63
2019/05/19	CH2205	Male	Three y/o	Treatment (Wk 1)	Treatment	Fast Day	No	14:10	14:00 - 14:59	Afternoon	37,7	67	7,4	7,4	18,85	563,53	66,59
2019/05/19	CH2206	Male	Three y/o	Treatment (Wk 1)	Treatment	Fast Day	No	14:10	14:00 - 14:59	Afternoon	38,0	52	10,6	10,6	19,15	542,83	78,73
2019/05/19	CH2205	Male	Three y/o	Treatment (Wk 1)	Treatment	Fast Day	No	14:15	14:00 - 14:59	Afternoon	37,6	50	5,2	5,2	18,75	539,52	54,72
2019/05/19	CH2206	Male	Three y/o	Treatment (Wk 1)	Treatment	Fast Day	No	14:15	14:00 - 14:59	Afternoon	38,0	46	9,0	9,0	19,15	532,38	73,19
2019/05/19	CH2205	Male	Three y/o	Treatment (Wk 1)	Treatment	Fast Day	No	14:20	14:00 - 14:59	Afternoon	37,6	44	20,2	20,2	18,75	528,51	100,65
2019/05/19	CH2206	Male	Three y/o	Treatment (Wk 1)	Treatment	Fast Day	No	14:20	14:00 - 14:59	Afternoon	38,0	63	5,0	5,0	19,15	558,62	53,41
2019/05/19	CH2205	Male	Three y/o	Treatment (Wk 1)	Treatment	Fast Day	No	14:25	14:00 - 14:59	Afternoon	37,6		27,6	27,6	18,75		111,33
2019/05/19	CH2206	Male	Three y/o	Treatment (Wk 1)	Treatment	Fast Day	No	14:25	14:00 - 14:59	Afternoon	38,0	59	18,0	18,0	19,15	553,30	96,72
2019/05/19	CH2205	Male	Three y/o	Treatment (Wk 1)	Treatment	Fast Day	No	14:30	14:00 - 14:59	Afternoon	37,5	65	18,8	18,8	18,65	561,12	98,20
2019/05/19	CH2206	Male	Three y/o	Treatment (Wk 1)	Treatment	Fast Day	No	14:30	14:00 - 14:59	Afternoon	37,9	77	26,0	26,0	19,05	574,39	109,29
2019/05/19	CH2205	Male	Three y/o	Treatment (Wk 1)	Treatment	Fast Day	No	14:35	14:00 - 14:59	Afternoon	37,4	53	10,8	10,8	18,55	544,43	79,36
2019/05/19	CH2206	Male	Three y/o	Treatment (Wk 1)	Treatment	Fast Day	No	14:35	14:00 - 14:59	Afternoon	37,9	78	26,2	26,2	19,05	575,38	109,55
2019/05/19	CH2205	Male	Three y/o	Treatment (Wk 1)	Treatment	Fast Day	No	14:40	14:00 - 14:59	Afternoon	37,4	38	7,0	7,0	18,55	515,48	64,72

2019/05/19	CH2206	Male	Three y/o	Treatment (Wk 1)	Treatment	Fast Day	No	14:40	14:00 - 14:59	Afternoon	37,9	67	26,2	26,2	19,05	563,53	109,55
2019/05/19	CH2205	Male	Three y/o	Treatment (Wk 1)	Treatment	Fast Day	No	14:45	14:00 - 14:59	Afternoon	37,4	61	20,2	20,2	18,55	556,01	100,65
2019/05/19	CH2206	Male	Three y/o	Treatment (Wk 1)	Treatment	Fast Day	No	14:45	14:00 - 14:59	Afternoon	37,9	54	9,4	9,4	19,05	545,99	74,66
2019/05/19	CH2205	Male	Three y/o	Treatment (Wk 1)	Treatment	Fast Day	No	14:50	14:00 - 14:59	Afternoon	37,4		42,6	42,6	18,55		126,26
2019/05/19	CH2206	Male	Three y/o	Treatment (Wk 1)	Treatment	Fast Day	No	14:50	14:00 - 14:59	Afternoon	37,9	72	26,2	26,2	19,05	569,20	109,55
2019/05/19	CH2205	Male	Three y/o	Treatment (Wk 1)	Treatment	Fast Day	No	14:55	14:00 - 14:59	Afternoon	37,3	79	21,8	21,8	18,46	576,36	103,26
2019/05/19	CH2206	Male	Three y/o	Treatment (Wk 1)	Treatment	Fast Day	No	14:55	14:00 - 14:59	Afternoon	37,8	51	34,0	34,0	18,95	541,20	118,49
2019/05/19	CH2205	Male	Three y/o	Treatment (Wk 1)	Treatment	Fast Day	No	15:00	15:00 - 15:59	Afternoon	37,3	43	29,2	29,2	18,46	526,50	113,27
2019/05/19	CH2206	Male	Three y/o	Treatment (Wk 1)	Treatment	Fast Day	No	15:00	15:00 - 15:59	Afternoon	37,8	62	15,0	15,0	18,95	557,33	90,51
2019/05/19	CH2205	Male	Three y/o	Treatment (Wk 1)	Treatment	Fast Day	No	15:05	15:00 - 15:59	Afternoon	37,3	47	43,4	43,4	18,46	534,23	126,90
2019/05/19	CH2206	Male	Three y/o	Treatment (Wk 1)	Treatment	Fast Day	No	15:05	15:00 - 15:59	Afternoon	37,8	50	57,2	57,2	18,95	539,52	136,44
2019/05/19	CH2205	Male	Three y/o	Treatment (Wk 1)	Treatment	Fast Day	No	15:10	15:00 - 15:59	Afternoon	37,4		27,4	27,4	18,55		111,09
2019/05/19	CH2206	Male	Three y/o	Treatment (Wk 1)	Treatment	Fast Day	No	15:10	15:00 - 15:59	Afternoon	37,8	117	20,8	20,8	18,95	605,02	101,65
2019/05/19	CH2205	Male	Three y/o	Treatment (Wk 1)	Treatment	Fast Day	No	15:15	15:00 - 15:59	Afternoon	37,4	50	57,4	57,4	18,55	539,52	136,56
2019/05/19	CH2206	Male	Three y/o	Treatment (Wk 1)	Treatment	Fast Day	No	15:15	15:00 - 15:59	Afternoon	37,8	47	48,6	48,6	18,95	534,23	130,81
2019/05/19	CH2205	Male	Three y/o	Treatment (Wk 1)	Treatment	Fast Day	No	15:20	15:00 - 15:59	Afternoon	37,4	64	50,0	50,0	18,55	559,88	131,79
2019/05/19	CH2206	Male	Three y/o	Treatment (Wk 1)	Treatment	Fast Day	No	15:20	15:00 - 15:59	Afternoon	37,8	108	215,2	215,2	18,95	599,39	182,70
2019/05/19	CH2205	Male	Three y/o	Treatment (Wk 1)	Treatment	Fast Day	No	15:25	15:00 - 15:59	Afternoon	37,5	109	313,8	313,8	18,65	600,04	196,01
2019/05/19	CH2206	Male	Three y/o	Treatment (Wk 1)	Treatment	Fast Day	No	15:25	15:00 - 15:59	Afternoon	37,8	107	325,6	325,6	18,95	598,73	197,32
2019/05/19	CH2205	Male	Three y/o	Treatment (Wk 1)	Treatment	Fast Day	No	15:30	15:00 - 15:59	Afternoon	37,6	70	324,4	324,4	18,75	566,99	197,19
2019/05/19	CH2206	Male	Three y/o	Treatment (Wk 1)	Treatment	Fast Day	No	15:30	15:00 - 15:59	Afternoon	37,8	112	257,4	257,4	18,95	601,96	189,01
2019/05/19	CH2205	Male	Three y/o	Treatment (Wk 1)	Treatment	Fast Day	No	15:35	15:00 - 15:59	Afternoon	37,7	143	21,4	21,4	18,85	618,69	102,63
2019/05/19	CH2206	Male	Three y/o	Treatment (Wk 1)	Treatment	Fast Day	No	15:35	15:00 - 15:59	Afternoon	37,8	113	81,2	81,2	18,95	602,58	148,59
2019/05/19	CH2205	Male	Three y/o	Treatment (Wk 1)	Treatment	Fast Day	No	15:40	15:00 - 15:59	Afternoon	37,9	63	24,4	24,4	19,05	558,62	107,11
2019/05/19	CH2206	Male	Three y/o	Treatment (Wk 1)	Treatment	Fast Day	No	15:40	15:00 - 15:59	Afternoon	37,8	84	216,0	216,0	18,95	581,01	182,83
2019/05/19	CH2205	Male	Three y/o	Treatment (Wk 1)	Treatment	Fast Day	No	15:45	15:00 - 15:59	Afternoon	37,9	65	16,0	16,0	19,05	561,12	92,71
2019/05/19	CH2206	Male	Three y/o	Treatment (Wk 1)	Treatment	Fast Day	No	15:45	15:00 - 15:59	Afternoon	37,9	55	72,0	72,0	19,05	547,52	144,42
2019/05/19	CH2205	Male	Three y/o	Treatment (Wk 1)	Treatment	Fast Day	No	15:50	15:00 - 15:59	Afternoon	38,0	58	31,4	31,4	19,15	551,90	115,76
2019/05/19	CH2206	Male	Three y/o	Treatment (Wk 1)	Treatment	Fast Day	No	15:50	15:00 - 15:59	Afternoon	37,9	108	55,4	55,4	19,05	599,39	135,33
2019/05/19	CH2205	Male	Three y/o	Treatment (Wk 1)	Treatment	Fast Day	No	15:55	15:00 - 15:59	Afternoon	38,0	63	25,0	25,0	19,15	558,62	107,94
2019/05/19	CH2206	Male	Three y/o	Treatment (Wk 1)	Treatment	Fast Day	No	15:55	15:00 - 15:59	Afternoon	37,9	63	64,0	64,0	19,05	558,62	140,33
2019/05/19	CH2205	Male	Three y/o	Treatment (Wk 1)	Treatment	Fast Day	No	16:00	16:00 - 16:59	Evening	38,0	49	24,0	24,0	19,15	537,80	106,55
2019/05/19	CH2206	Male	Three y/o	Treatment (Wk 1)	Treatment	Fast Day	No	16:00	16:00 - 16:59	Evening	38,0	57	66,0	66,0	19,15	550,47	141,40
2019/05/19	CH2205	Male	Three y/o	Treatment (Wk 1)	Treatment	Fast Day	No	16:05	16:00 - 16:59	Evening	38,1	48	50,2	50,2	19,26	536,04	131,92
2019/05/19	CH2206	Male	Three y/o	Treatment (Wk 1)	Treatment	Fast Day	No	16:05	16:00 - 16:59	Evening	38,0	55	48,4	48,4	19,15	547,52	130,66
2019/05/19	CH2205	Male	Three y/o	Treatment (Wk 1)	Treatment	Fast Day	No	16:10	16:00 - 16:59	Evening	38,1	96	10,6	10,6	19,26	590,91	78,73
2019/05/19	CH2206	Male	Three y/o	Treatment (Wk 1)	Treatment	Fast Day	No	16:10	16:00 - 16:59	Evening	38,0	90	79,4	79,4	19,15	586,16	147,81
2019/05/19	CH2205	Male	Three y/o	Treatment (Wk 1)	Treatment	Fast Day	No	16:15	16:00 - 16:59	Evening	38,1	90	55,0	55,0	19,26	586,16	135,08
2019/05/19	CH2206	Male	Three y/o	Treatment (Wk 1)	Treatment	Fast Day	No	16:15	16:00 - 16:59	Evening	38,0	118	166,8	166,8	19,15	605,61	173,74
2019/05/19	CH2205	Male	Three y/o	Treatment (Wk 1)	Treatment	Fast Day	No	16:20	16:00 - 16:59	Evening	38,1	93	95,0	95,0	19,26	588,58	154,06
2019/05/19	CH2206	Male	Three y/o	Treatment (Wk 1)	Treatment	Fast Day	No	16:20	16:00 - 16:59	Evening	38,0	108	35,4	35,4	19,15	599,39	119,88
2019/05/19	CH2205	Male	Three y/o	Treatment (Wk 1)	Treatment	Fast Day	No	16:25	16:00 - 16:59	Evening	38,1	90	24,6	24,6	19,26	586,16	107,39
2019/05/19	CH2206	Male	Three y/o	Treatment (Wk 1)	Treatment	Fast Day	No	16:25	16:00 - 16:59	Evening	38,0		83,2	83,2	19,15		149,44
2019/05/19	CH2205	Male	Three y/o	Treatment (Wk 1)	Treatment	Fast Day	No	16:30	16:00 - 16:59	Evening	38,1	59	11,0	11,0	19,26	553,30	79,98
2019/05/19	CH2206	Male	Three y/o	Treatment (Wk 1)	Treatment	Fast Day	No	16:30	16:00 - 16:59	Evening	37,9	94	149,6	149,6	19,05	589,37	169,92
2019/05/19	CH2205	Male	Three y/o	Treatment (Wk 1)	Treatment	Fast Day	No	16:35	16:00 - 16:59	Evening	38,0	80	40,8	40,8	19,15	577,31	124,77
2019/05/19	CH2206	Male	Three y/o	Treatment (Wk 1)	Treatment	Fast Day	No	16:35	16:00 - 16:59	Evening	38,0	121	114,6	114,6	19,15	607,35	160,60
2019/05/19	CH2205	Male	Three y/o	Treatment (Wk 1)	Treatment	Fast Day	No	16:40	16:00 - 16:59	Evening	38,1	69	42,4	42,4	19,26	565,86	126,10
2019/05/19	CH2206	Male	Three y/o	Treatment (Wk 1)	Treatment	Fast Day	No	16:40	16:00 - 16:59	Evening	38,0	126	172,0	172,0	19,15	610,14	174,82
2019/05/19	CH2205	Male	Three y/o	Treatment (Wk 1)	Treatment	Fast Day	No	16:45	16:00 - 16:59	Evening	38,0	56	37,8	37,8	19,15	549,01	122,14
2019/05/19	CH2206	Male	Three y/o	Treatment (Wk 1)	Treatment	Fast Day	No	16:45	16:00 - 16:59	Evening	38,1	118	85,2	85,2	19,26	605,61	150,27
2019/05/19	CH2205	Male	Three y/o	Treatment (Wk 1)	Treatment	Fast Day	No	16:50	16:00 - 16:59	Evening	38,0		35,2	35,2	19,15		119,69
2019/05/19	CH2206	Male	Three y/o	Treatment (Wk 1)	Treatment	Fast Day	No	16:50	16:00 - 16:59	Evening	38,1	113	92,8	92,8	19,26	602,58	153,24
2019/05/19	CH2205	Male	Three y/o	Treatment (Wk 1)	Treatment	Fast Day	No	16:55	16:00 - 16:59	Evening	38,0	72	33,2	33,2	19,15	569,20	117,68
2019/05/19	CH2206	Male	Three y/o	Treatment (Wk 1)	Treatment	Fast Day	No	16:55	16:00 - 16:59	Evening	38,2	109	214,4	214,4	19,36	600,04	182,57
2019/05/19	CH2205	Male	Three y/o	Treatment (Wk 1)	Treatment	Fast Day	No	17:00	17:00 - 17:59	Evening	37,9	54	32,6	32,6	19,05	545,99	117,05
2019/05/19	CH2206	Male	Three y/o	Treatment (Wk 1)	Treatment	Fast Day	No	17:00	17:00 - 17:59	Evening	38,2	110	93,4	93,4	19,36	600,69	153,46
2019/05/19	CH2205	Male	Three y/o	Treatment (Wk 1)	Treatment	Fast Day	No	17:05	17:00 - 17:59	Evening	37,9	54	28,8	28,8	19,05	545,99	112,79
2019/05/19	CH2206	Male	Three y/o	Treatment (Wk 1)	Treatment	Fast Day	No	17:05	17:00 - 17:59	Evening	38,2	120	188,8	188,8	19,36	606,78	178,09
2019/05/19	CH2205	Male	Three y/o	Treatment (Wk 1)	Treatment	Fast Day	No	17:10	17:00 - 17:59	Evening	37,9	42	32,0	32,0	19,05	524,42	116,41
2019/05/19	CH2206	Male	Three y/o	Treatment (Wk 1)	Treatment	Fast Day	No	17:10	17:00 - 17:59	Evening	38,3	113	157,2	157,2	19,46	602,58	171,66
2019/05/19	CH2205	Male	Three y/o	Treatment (Wk 1)	Treatment	Fast Day	No	17:15	17:00 - 17:59	Evening	38,0	125	137,6	137,6	19,15	609,60	166,99
2019/05/19	CH2206	Male	Three y/o	Treatment (Wk 1)	Treatment	Fast Day	No	17:15	17:00 - 17:59	Evening	38,3	118	55,0	55,0	19,46	605,61	135,08
2019/05/19	CH2205	Male	Three y/o	Treatment (Wk 1)	Treatment	Fast Day	No	17:20	17:00 - 17:59	Evening	38,0	98	64,0	64,0	19,15	592,41	140,33
2019/05/19	CH2206	Male	Three y/o	Treatment (Wk 1)	Treatment	Fast Day	No	17:20	17:00 - 17:59	Evening	38,3	47	41,8	41,8	19,46	534,23	125,60
2019/05/19	CH2205	Male	Three y/o	Treatment (Wk 1)	Treatment	Fast Day	No	17:25	17:00 - 17:59	Evening	38,0	78	42,8	42,8	19,15	575,38	126,42
2019/05/19	CH2206	Male	Three y/o	Treatment (Wk 1)	Treatment	Fast Day	No	17:25	17:00 - 17:59	Evening	38,2	79	42,0	42,0	19,36	576,36	125,77
2019/05/19	CH2205	Male	Three y/o	Treatment (Wk 1)	Treatment	Fast Day	No	17:30	17:00 - 17:59	Evening	38,0	63	30,2	30,2	19,15	558,62	114,42
2019/05/19	CH2206	Male	Three y/o	Treatment (Wk 1)	Treatment	Fast Day	No	17:30	17:00 - 17:59	Evening	38,1	73	48,4	48,4	19,26	570,27	130,66
2019/05/19	CH2205	Male	Three y/o	Treatment (Wk 1)	Treatment	Fast Day	No	17:35	17:00 - 17:59	Evening	38,0	43	32,0	32,0	19,15	526,50	116,41

2019/05/19	CH2206	Male	Three y/o	Treatment (Wk 1)	Treatment	Fast Day	No	17:35	17:00 - 17:59	Evening	38,0	57	51,0	51,0	19,15	550,47	132,47
2019/05/19	CH2205	Male	Three y/o	Treatment (Wk 1)	Treatment	Fast Day	No	17:40	17:00 - 17:59	Evening	38,0		36,6	36,6	19,15		121,03
2019/05/19	CH2206	Male	Three y/o	Treatment (Wk 1)	Treatment	Fast Day	No	17:40	17:00 - 17:59	Evening	37,8	54	37,8	37,8	18,95	545,99	122,14
2019/05/19	CH2205	Male	Three y/o	Treatment (Wk 1)	Treatment	Fast Day	No	17:45	17:00 - 17:59	Evening	37,9	50	31,6	31,6	19,05	539,52	115,98
2019/05/19	CH2206	Male	Three y/o	Treatment (Wk 1)	Treatment	Fast Day	No	17:45	17:00 - 17:59	Evening	37,8	67	39,8	39,8	18,95	563,53	123,91
2019/05/19	CH2205	Male	Three y/o	Treatment (Wk 1)	Treatment	Fast Day	No	17:50	17:00 - 17:59	Evening	37,9		26,4	26,4	19,05		109,81
2019/05/19	CH2206	Male	Three y/o	Treatment (Wk 1)	Treatment	Fast Day	No	17:50	17:00 - 17:59	Evening	37,8	125	29,0	29,0	18,95	609,60	113,03
2019/05/19	CH2205	Male	Three y/o	Treatment (Wk 1)	Treatment	Fast Day	No	17:55	17:00 - 17:59	Evening	37,7	59	31,8	31,8	18,85	553,30	116,20
2019/05/19	CH2206	Male	Three y/o	Treatment (Wk 1)	Treatment	Fast Day	No	17:55	17:00 - 17:59	Evening	37,9		34,8	34,8	19,05		119,29
2019/05/19	CH2205	Male	Three y/o	Treatment (Wk 1)	Treatment	Fast Day	No	18:00	18:00 - 18:59	Evening	37,7	48	24,6	24,6	18,85	536,04	107,39
2019/05/19	CH2206	Male	Three y/o	Treatment (Wk 1)	Treatment	Fast Day	No	18:00	18:00 - 18:59	Evening	37,8	57	36,4	36,4	18,95	550,47	120,84
2019/05/19	CH2205	Male	Three y/o	Treatment (Wk 1)	Treatment	Fast Day	No	18:05	18:00 - 18:59	Evening	37,7	51	16,0	16,0	18,85	541,20	92,71
2019/05/19	CH2206	Male	Three y/o	Treatment (Wk 1)	Treatment	Fast Day	No	18:05	18:00 - 18:59	Evening	37,8		28,2	28,2	18,95		112,07
2019/05/19	CH2205	Male	Three y/o	Treatment (Wk 1)	Treatment	Fast Day	No	18:10	18:00 - 18:59	Evening	37,7	41	13,6	13,6	18,85	522,29	87,18
2019/05/19	CH2206	Male	Three y/o	Treatment (Wk 1)	Treatment	Fast Day	No	18:10	18:00 - 18:59	Evening	37,8		43,2	43,2	18,95		126,74
2019/05/19	CH2205	Male	Three y/o	Treatment (Wk 1)	Treatment	Fast Day	No	18:15	18:00 - 18:59	Evening	37,7	49	9,4	9,4	18,85	537,80	74,66
2019/05/19	CH2206	Male	Three y/o	Treatment (Wk 1)	Treatment	Fast Day	No	18:15	18:00 - 18:59	Evening	37,7	47	37,8	37,8	18,85	534,23	122,14
2019/05/19	CH2205	Male	Three y/o	Treatment (Wk 1)	Treatment	Fast Day	No	18:20	18:00 - 18:59	Evening	37,7		15,4	15,4	18,85		91,41
2019/05/19	CH2206	Male	Three y/o	Treatment (Wk 1)	Treatment	Fast Day	No	18:20	18:00 - 18:59	Evening	37,7		48,4	48,4	18,85		130,66
2019/05/19	CH2205	Male	Three y/o	Treatment (Wk 1)	Treatment	Fast Day	No	18:25	18:00 - 18:59	Evening	37,7	35	13,2	13,2	18,85	507,97	86,17
2019/05/19	CH2206	Male	Three y/o	Treatment (Wk 1)	Treatment	Fast Day	No	18:25	18:00 - 18:59	Evening	37,7	68	49,2	49,2	18,85	564,71	131,23
2019/05/19	CH2205	Male	Three y/o	Treatment (Wk 1)	Treatment	Fast Day	No	18:30	18:00 - 18:59	Evening	37,7	47	7,0	7,0	18,85	534,23	64,72
2019/05/19	CH2206	Male	Three y/o	Treatment (Wk 1)	Treatment	Fast Day	No	18:30	18:00 - 18:59	Evening	37,6	43	46,0	46,0	18,75	526,50	128,91
2019/05/19	CH2205	Male	Three y/o	Treatment (Wk 1)	Treatment	Fast Day	No	18:35	18:00 - 18:59	Evening	37,7	55	10,4	10,4	18,85	547,52	78,08
2019/05/19	CH2206	Male	Three y/o	Treatment (Wk 1)	Treatment	Fast Day	No	18:35	18:00 - 18:59	Evening	37,7		47,4	47,4	18,85		129,94
2019/05/19	CH2205	Male	Three y/o	Treatment (Wk 1)	Treatment	Fast Day	No	18:40	18:00 - 18:59	Evening	37,6	56	7,6	7,6	18,75	549,01	67,49
2019/05/19	CH2206	Male	Three y/o	Treatment (Wk 1)	Treatment	Fast Day	No	18:40	18:00 - 18:59	Evening	37,7	54	31,2	31,2	18,85	545,99	115,54
2019/05/19	CH2205	Male	Three y/o	Treatment (Wk 1)	Treatment	Fast Day	No	18:45	18:00 - 18:59	Evening	37,6	38	6,0	6,0	18,75	515,48	59,53
2019/05/19	CH2206	Male	Three y/o	Treatment (Wk 1)	Treatment	Fast Day	No	18:45	18:00 - 18:59	Evening	37,8	180	29,2	29,2	18,95	633,61	113,27
2019/05/19	CH2205	Male	Three y/o	Treatment (Wk 1)	Treatment	Fast Day	No	18:50	18:00 - 18:59	Evening	37,6	43	7,4	7,4	18,75	526,50	66,59
2019/05/19	CH2206	Male	Three y/o	Treatment (Wk 1)	Treatment	Fast Day	No	18:50	18:00 - 18:59	Evening	37,8	62	27,2	27,2	18,95	557,33	110,83
2019/05/19	CH2205	Male	Three y/o	Treatment (Wk 1)	Treatment	Fast Day	No	18:55	18:00 - 18:59	Evening	37,6	41	6,0	6,0	18,75	522,29	59,53
2019/05/19	CH2206	Male	Three y/o	Treatment (Wk 1)	Treatment	Fast Day	No	18:55	18:00 - 18:59	Evening	37,7	75	27,8	27,8	18,85	572,36	111,58
2019/05/19	CH2205	Male	Three y/o	Treatment (Wk 1)	Treatment	Fast Day	No	19:00	19:00 - 19:59	Evening	37,6	44	4,0	4,0	18,75	528,51	45,94
2019/05/19	CH2206	Male	Three y/o	Treatment (Wk 1)	Treatment	Fast Day	No	19:00	19:00 - 19:59	Evening	37,7	47	26,8	26,8	18,85	534,23	110,33
2019/05/19	CH2205	Male	Three y/o	Treatment (Wk 1)	Treatment	Fast Day	No	19:05	19:00 - 19:59	Evening	37,6	60	11,0	11,0	18,75	554,67	79,98
2019/05/19	CH2206	Male	Three y/o	Treatment (Wk 1)	Treatment	Fast Day	No	19:05	19:00 - 19:59	Evening	37,7	59	31,8	31,8	18,85	553,30	116,20
2019/05/19	CH2205	Male	Three y/o	Treatment (Wk 1)	Treatment	Fast Day	No	19:10	19:00 - 19:59	Evening	37,6	108	32,2	32,2	18,75	599,39	116,63
2019/05/19	CH2206	Male	Three y/o	Treatment (Wk 1)	Treatment	Fast Day	No	19:10	19:00 - 19:59	Evening	37,7	75	18,6	18,6	18,85	572,36	97,84
2019/05/19	CH2205	Male	Three y/o	Treatment (Wk 1)	Treatment	Fast Day	No	19:15	19:00 - 19:59	Evening	37,7	46	23,2	23,2	18,85	532,38	105,39
2019/05/19	CH2206	Male	Three y/o	Treatment (Wk 1)	Treatment	Fast Day	No	19:15	19:00 - 19:59	Evening	37,7	70	17,4	17,4	18,85	566,99	95,56
2019/05/19	CH2205	Male	Three y/o	Treatment (Wk 1)	Treatment	Fast Day	No	19:20	19:00 - 19:59	Evening	37,6	40	24,6	24,6	18,75	520,09	107,39
2019/05/19	CH2206	Male	Three y/o	Treatment (Wk 1)	Treatment	Fast Day	No	19:20	19:00 - 19:59	Evening	37,6	46	15,8	15,8	18,75	532,38	92,28
2019/05/19	CH2205	Male	Three y/o	Treatment (Wk 1)	Treatment	Fast Day	No	19:25	19:00 - 19:59	Evening	37,6	62	23,0	23,0	18,75	557,33	105,09
2019/05/19	CH2206	Male	Three y/o	Treatment (Wk 1)	Treatment	Fast Day	No	19:25	19:00 - 19:59	Evening	37,5	40	12,6	12,6	18,65	520,09	84,59
2019/05/19	CH2205	Male	Three y/o	Treatment (Wk 1)	Treatment	Fast Day	No	19:30	19:00 - 19:59	Evening	37,5	44	21,4	21,4	18,65	528,51	102,63
2019/05/19	CH2206	Male	Three y/o	Treatment (Wk 1)	Treatment	Fast Day	No	19:30	19:00 - 19:59	Evening	37,5	43	10,4	10,4	18,65	526,50	78,08
2019/05/19	CH2205	Male	Three y/o	Treatment (Wk 1)	Treatment	Fast Day	No	19:35	19:00 - 19:59	Evening	37,5	45	22,4	22,4	18,65	530,47	104,19
2019/05/19	CH2206	Male	Three y/o	Treatment (Wk 1)	Treatment	Fast Day	No	19:35	19:00 - 19:59	Evening	37,5	83	4,8	4,8	18,65	580,11	52,04
2019/05/19	CH2205	Male	Three y/o	Treatment (Wk 1)	Treatment	Fast Day	No	19:40	19:00 - 19:59	Evening	37,5	48	21,4	21,4	18,65	536,04	102,63
2019/05/19	CH2206	Male	Three y/o	Treatment (Wk 1)	Treatment	Fast Day	No	19:40	19:00 - 19:59	Evening	37,4	47	9,4	9,4	18,55	534,23	74,66
2019/05/19	CH2205	Male	Three y/o	Treatment (Wk 1)	Treatment	Fast Day	No	19:45	19:00 - 19:59	Evening	37,4	41	24,2	24,2	18,55	522,29	106,83
2019/05/19	CH2206	Male	Three y/o	Treatment (Wk 1)	Treatment	Fast Day	No	19:45	19:00 - 19:59	Evening	37,4	49	12,0	12,0	18,55	537,80	82,93
2019/05/19	CH2205	Male	Three y/o	Treatment (Wk 1)	Treatment	Fast Day	No	19:50	19:00 - 19:59	Evening	37,4	55	23,8	23,8	18,55	547,52	106,26
2019/05/19	CH2206	Male	Three y/o	Treatment (Wk 1)	Treatment	Fast Day	No	19:50	19:00 - 19:59	Evening	37,3	48	14,6	14,6	18,46	536,04	89,59
2019/05/19	CH2205	Male	Three y/o	Treatment (Wk 1)	Treatment	Fast Day	No	19:55	19:00 - 19:59	Evening	37,4	75	24,4	24,4	18,55	572,36	107,11
2019/05/19	CH2206	Male	Three y/o	Treatment (Wk 1)	Treatment	Fast Day	No	19:55	19:00 - 19:59	Evening	37,3	38	13,2	13,2	18,46	515,48	86,17
2019/05/19	CH2205	Male	Three y/o	Treatment (Wk 1)	Treatment	Fast Day	No	20:00	20:00 - 20:59	Night	37,4	65	57,6	57,6	18,55	561,12	136,68
2019/05/19	CH2206	Male	Three y/o	Treatment (Wk 1)	Treatment	Fast Day	No	20:00	20:00 - 20:59	Night	37,3	61	38,4	38,4	18,46	556,01	122,68
2019/05/19	CH2205	Male	Three y/o	Treatment (Wk 1)	Treatment	Fast Day	No	20:05	20:00 - 20:59	Night	37,4	68	22,6	22,6	18,55	564,71	104,49
2019/05/19	CH2206	Male	Three y/o	Treatment (Wk 1)	Treatment	Fast Day	No	20:05	20:00 - 20:59	Night	37,3	45	60,4	60,4	18,46	530,47	138,32
2019/05/19	CH2205	Male	Three y/o	Treatment (Wk 1)	Treatment	Fast Day	No	20:10	20:00 - 20:59	Night	37,3	43	22,6	22,6	18,46	526,50	104,49
2019/05/19	CH2206	Male	Three y/o	Treatment (Wk 1)	Treatment	Fast Day	No	20:10	20:00 - 20:59	Night	37,3	44	65,0	65,0	18,46	528,51	140,87
2019/05/19	CH2205	Male	Three y/o	Treatment (Wk 1)	Treatment	Fast Day	No	20:15	20:00 - 20:59	Night	37,2	78	19,8	19,8	18,36	575,38	99,97
2019/05/19	CH2206	Male	Three y/o	Treatment (Wk 1)	Treatment	Fast Day	No	20:15	20:00 - 20:59	Night	37,3	46	62,2	62,2	18,46	532,38	139,34
2019/05/19	CH2205	Male	Three y/o	Treatment (Wk 1)	Treatment	Fast Day	No	20:20	20:00 - 20:59	Night	37,1	58	22,0	22,0	18,26	551,90	103,57
2019/05/19	CH2206	Male	Three y/o	Treatment (Wk 1)	Treatment	Fast Day	No	20:20	20:00 - 20:59	Night	37,3	76	66,8	66,8	18,46	573,39	141,81
2019/05/19	CH2205	Male	Three y/o	Treatment (Wk 1)	Treatment	Fast Day	No	20:25	20:00 - 20:59	Night	37,1	94	27,4	27,4	18,26	589,37	111,09
2019/05/19	CH2206	Male	Three y/o	Treatment (Wk 1)	Treatment	Fast Day	No	20:25	20:00 - 20:59	Night	37,3	39	65,8	65,8	18,46	517,82	141,29
2019/05/19	CH2205	Male	Three y/o	Treatment (Wk 1)	Treatment	Fast Day	No	20:30	20:00 - 20:59	Night	37,1	40	22,8	22,8	18,26	520,09	104,79

2019/05/19	CH2206	Male	Three y/o	Treatment (Wk 1)	Treatment	Fast Day	No	20:30	20:00 - 20:59	Night	37,3	83	64,6	64,6	18,46	580,11	140,65
2019/05/19	CH2205	Male	Three y/o	Treatment (Wk 1)	Treatment	Fast Day	No	20:35	20:00 - 20:59	Night	37,0	45	23,8	23,8	18,16	530,47	106,26
2019/05/19	CH2206	Male	Three y/o	Treatment (Wk 1)	Treatment	Fast Day	No	20:35	20:00 - 20:59	Night	37,3	46	63,8	63,8	18,46	532,38	140,22
2019/05/19	CH2205	Male	Three y/o	Treatment (Wk 1)	Treatment	Fast Day	No	20:40	20:00 - 20:59	Night	37,1	44	25,8	25,8	18,26	528,51	109,02
2019/05/19	CH2206	Male	Three y/o	Treatment (Wk 1)	Treatment	Fast Day	No	20:40	20:00 - 20:59	Night	37,3	44	63,8	63,8	18,46	528,51	140,22
2019/05/19	CH2205	Male	Three y/o	Treatment (Wk 1)	Treatment	Fast Day	No	20:45	20:00 - 20:59	Night	37,1	68	26,6	26,6	18,26	564,71	110,07
2019/05/19	CH2206	Male	Three y/o	Treatment (Wk 1)	Treatment	Fast Day	No	20:45	20:00 - 20:59	Night	37,3	42	65,2	65,2	18,46	524,42	140,97
2019/05/19	CH2205	Male	Three y/o	Treatment (Wk 1)	Treatment	Fast Day	No	20:50	20:00 - 20:59	Night	37,1	75	20,6	20,6	18,26	572,36	101,32
2019/05/19	CH2206	Male	Three y/o	Treatment (Wk 1)	Treatment	Fast Day	No	20:50	20:00 - 20:59	Night	37,3	76	63,2	63,2	18,46	573,39	139,89
2019/05/19	CH2205	Male	Three y/o	Treatment (Wk 1)	Treatment	Fast Day	No	20:55	20:00 - 20:59	Night	37,2	41	30,2	30,2	18,36	522,29	114,42
2019/05/19	CH2206	Male	Three y/o	Treatment (Wk 1)	Treatment	Fast Day	No	20:55	20:00 - 20:59	Night	37,3	38	56,6	56,6	18,46	515,48	136,07
2019/05/19	CH2205	Male	Three y/o	Treatment (Wk 1)	Treatment	Fast Day	No	21:00	21:00 - 21:59	Night	37,2		26,6	26,6	18,36		110,07
2019/05/19	CH2206	Male	Three y/o	Treatment (Wk 1)	Treatment	Fast Day	No	21:00	21:00 - 21:59	Night	37,3	48	62,8	62,8	18,46	536,04	139,67
2019/05/19	CH2205	Male	Three y/o	Treatment (Wk 1)	Treatment	Fast Day	No	21:05	21:00 - 21:59	Night	37,1		32,8	32,8	18,26		117,26
2019/05/19	CH2206	Male	Three y/o	Treatment (Wk 1)	Treatment	Fast Day	No	21:05	21:00 - 21:59	Night	37,3	49	60,2	60,2	18,46	537,80	138,21
2019/05/19	CH2205	Male	Three y/o	Treatment (Wk 1)	Treatment	Fast Day	No	21:10	21:00 - 21:59	Night	37,1	55	24,4	24,4	18,26	547,52	107,11
2019/05/19	CH2206	Male	Three y/o	Treatment (Wk 1)	Treatment	Fast Day	No	21:10	21:00 - 21:59	Night	37,3	80	62,2	62,2	18,46	577,31	139,34
2019/05/19	CH2205	Male	Three y/o	Treatment (Wk 1)	Treatment	Fast Day	No	21:15	21:00 - 21:59	Night	37,2		23,0	23,0	18,36		105,09
2019/05/19	CH2206	Male	Three y/o	Treatment (Wk 1)	Treatment	Fast Day	No	21:15	21:00 - 21:59	Night	37,3	50	62,6	62,6	18,46	539,52	139,56
2019/05/19	CH2205	Male	Three y/o	Treatment (Wk 1)	Treatment	Fast Day	No	21:20	21:00 - 21:59	Night	37,3	62	21,0	21,0	18,46	557,33	101,98
2019/05/19	CH2206	Male	Three y/o	Treatment (Wk 1)	Treatment	Fast Day	No	21:20	21:00 - 21:59	Night	37,3	43	64,2	64,2	18,46	526,50	140,44
2019/05/19	CH2205	Male	Three y/o	Treatment (Wk 1)	Treatment	Fast Day	No	21:25	21:00 - 21:59	Night	37,3	46	27,0	27,0	18,46	532,38	110,58
2019/05/19	CH2206	Male	Three y/o	Treatment (Wk 1)	Treatment	Fast Day	No	21:25	21:00 - 21:59	Night	37,3	40	63,8	63,8	18,46	520,09	140,22
2019/05/19	CH2205	Male	Three y/o	Treatment (Wk 1)	Treatment	Fast Day	No	21:30	21:00 - 21:59	Night	37,3		20,2	20,2	18,46		100,65
2019/05/19	CH2206	Male	Three y/o	Treatment (Wk 1)	Treatment	Fast Day	No	21:30	21:00 - 21:59	Night	37,3	44	67,6	67,6	18,46	528,51	142,23
2019/05/19	CH2205	Male	Three y/o	Treatment (Wk 1)	Treatment	Fast Day	No	21:35	21:00 - 21:59	Night	37,3	51	21,8	21,8	18,46	541,20	103,26
2019/05/19	CH2206	Male	Three y/o	Treatment (Wk 1)	Treatment	Fast Day	No	21:35	21:00 - 21:59	Night	37,4	78	64,4	64,4	18,55	575,38	140,55
2019/05/19	CH2205	Male	Three y/o	Treatment (Wk 1)	Treatment	Fast Day	No	21:40	21:00 - 21:59	Night	37,3		18,0	18,0	18,46		96,72
2019/05/19	CH2206	Male	Three y/o	Treatment (Wk 1)	Treatment	Fast Day	No	21:40	21:00 - 21:59	Night	37,4	109	64,4	64,4	18,55	600,04	140,55
2019/05/19	CH2205	Male	Three y/o	Treatment (Wk 1)	Treatment	Fast Day	No	21:45	21:00 - 21:59	Night	37,3		19,4	19,4	18,46		99,28
2019/05/19	CH2206	Male	Three y/o	Treatment (Wk 1)	Treatment	Fast Day	No	21:45	21:00 - 21:59	Night	37,4	46	62,6	62,6	18,55	532,38	139,56
2019/05/19	CH2205	Male	Three y/o	Treatment (Wk 1)	Treatment	Fast Day	No	21:50	21:00 - 21:59	Night	37,3		20,6	20,6	18,46		101,32
2019/05/19	CH2206	Male	Three y/o	Treatment (Wk 1)	Treatment	Fast Day	No	21:50	21:00 - 21:59	Night	37,4	43	65,4	65,4	18,55	526,50	141,08
2019/05/19	CH2205	Male	Three y/o	Treatment (Wk 1)	Treatment	Fast Day	No	21:55	21:00 - 21:59	Night	37,3		14,2	14,2	18,46		88,65
2019/05/19	CH2206	Male	Three y/o	Treatment (Wk 1)	Treatment	Fast Day	No	21:55	21:00 - 21:59	Night	37,3	49	63,0	63,0	18,46	537,80	139,78
2019/05/19	CH2205	Male	Three y/o	Treatment (Wk 1)	Treatment	Fast Day	No	22:00	22:00 - 22:59	Night	37,4	63	9,6	9,6	18,55	558,62	75,37
2019/05/19	CH2206	Male	Three y/o	Treatment (Wk 1)	Treatment	Fast Day	No	22:00	22:00 - 22:59	Night	37,3	51	62,8	62,8	18,46	541,20	139,67
2019/05/19	CH2205	Male	Three y/o	Treatment (Wk 1)	Treatment	Fast Day	No	22:05	22:00 - 22:59	Night	37,4	45	7,6	7,6	18,55	530,47	67,49
2019/05/19	CH2206	Male	Three y/o	Treatment (Wk 1)	Treatment	Fast Day	No	22:05	22:00 - 22:59	Night	37,3	50	59,6	59,6	18,46	539,52	137,86
2019/05/19	CH2205	Male	Three y/o	Treatment (Wk 1)	Treatment	Fast Day	No	22:10	22:00 - 22:59	Night	37,4	54	8,4	8,4	18,55	545,99	70,86
2019/05/19	CH2206	Male	Three y/o	Treatment (Wk 1)	Treatment	Fast Day	No	22:10	22:00 - 22:59	Night	37,3	50	52,8	52,8	18,46	539,52	133,67
2019/05/19	CH2205	Male	Three y/o	Treatment (Wk 1)	Treatment	Fast Day	No	22:15	22:00 - 22:59	Night	37,4	44	6,2	6,2	18,55	528,51	60,63
2019/05/19	CH2206	Male	Three y/o	Treatment (Wk 1)	Treatment	Fast Day	No	22:15	22:00 - 22:59	Night	37,3	43	50,0	50,0	18,46	526,50	131,79
2019/05/19	CH2205	Male	Three y/o	Treatment (Wk 1)	Treatment	Fast Day	No	22:20	22:00 - 22:59	Night	37,4	69	6,6	6,6	18,55	565,86	62,73
2019/05/19	CH2206	Male	Three y/o	Treatment (Wk 1)	Treatment	Fast Day	No	22:20	22:00 - 22:59	Night	37,3	55	57,2	57,2	18,46	547,52	136,44
2019/05/19	CH2205	Male	Three y/o	Treatment (Wk 1)	Treatment	Fast Day	No	22:25	22:00 - 22:59	Night	37,4	45	8,2	8,2	18,55	530,47	70,05
2019/05/19	CH2206	Male	Three y/o	Treatment (Wk 1)	Treatment	Fast Day	No	22:25	22:00 - 22:59	Night	37,3	79	61,8	61,8	18,46	576,36	139,12
2019/05/19	CH2205	Male	Three y/o	Treatment (Wk 1)	Treatment	Fast Day	No	22:30	22:00 - 22:59	Night	37,4	80	2,8	2,8	18,55	577,31	34,04
2019/05/19	CH2206	Male	Three y/o	Treatment (Wk 1)	Treatment	Fast Day	No	22:30	22:00 - 22:59	Night	37,3	40	60,0	60,0	18,46	520,09	138,09
2019/05/19	CH2205	Male	Three y/o	Treatment (Wk 1)	Treatment	Fast Day	No	22:35	22:00 - 22:59	Night	37,4	73	2,2	2,2	18,55	570,27	26,02
2019/05/19	CH2206	Male	Three y/o	Treatment (Wk 1)	Treatment	Fast Day	No	22:35	22:00 - 22:59	Night	37,3	78	54,6	54,6	18,46	575,38	134,83
2019/05/19	CH2205	Male	Three y/o	Treatment (Wk 1)	Treatment	Fast Day	No	22:40	22:00 - 22:59	Night	37,3	51	3,6	3,6	18,46	541,20	42,42
2019/05/19	CH2206	Male	Three y/o	Treatment (Wk 1)	Treatment	Fast Day	No	22:40	22:00 - 22:59	Night	37,3	47	57,0	57,0	18,46	534,23	136,32
2019/05/19	CH2205	Male	Three y/o	Treatment (Wk 1)	Treatment	Fast Day	No	22:45	22:00 - 22:59	Night	37,3	50	4,2	4,2	18,46	539,52	47,57
2019/05/19	CH2206	Male	Three y/o	Treatment (Wk 1)	Treatment	Fast Day	No	22:45	22:00 - 22:59	Night	37,3	44	56,0	56,0	18,46	528,51	135,70
2019/05/19	CH2205	Male	Three y/o	Treatment (Wk 1)	Treatment	Fast Day	No	22:50	22:00 - 22:59	Night	37,3	40	4,2	4,2	18,46	520,09	47,57
2019/05/19	CH2206	Male	Three y/o	Treatment (Wk 1)	Treatment	Fast Day	No	22:50	22:00 - 22:59	Night	37,3	46	58,0	58,0	18,46	532,38	136,92
2019/05/19	CH2205	Male	Three y/o	Treatment (Wk 1)	Treatment	Fast Day	No	22:55	22:00 - 22:59	Night	37,3	42	5,0	5,0	18,46	524,42	53,41
2019/05/19	CH2206	Male	Three y/o	Treatment (Wk 1)	Treatment	Fast Day	No	22:55	22:00 - 22:59	Night	37,3	41	53,6	53,6	18,46	522,29	134,19
2019/05/19	CH2205	Male	Three y/o	Treatment (Wk 1)	Treatment	Fast Day	No	23:00	23:00 - 23:59	Night	37,3	86	5,2	5,2	18,46	582,77	54,72
2019/05/19	CH2206	Male	Three y/o	Treatment (Wk 1)	Treatment	Fast Day	No	23:00	23:00 - 23:59	Night	37,3	38	59,8	59,8	18,46	515,48	137,98
2019/05/19	CH2205	Male	Three y/o	Treatment (Wk 1)	Treatment	Fast Day	No	23:05	23:00 - 23:59	Night	37,3	88	6,4	6,4	18,46	584,49	61,70
2019/05/19	CH2206	Male	Three y/o	Treatment (Wk 1)	Treatment	Fast Day	No	23:05	23:00 - 23:59	Night	37,3	49	67,4	67,4	18,46	537,80	142,12
2019/05/19	CH2205	Male	Three y/o	Treatment (Wk 1)	Treatment	Fast Day	No	23:10	23:00 - 23:59	Night	37,2	40	5,6	5,6	18,36	520,09	57,21
2019/05/19	CH2206	Male	Three y/o	Treatment (Wk 1)	Treatment	Fast Day	No	23:10	23:00 - 23:59	Night	37,2	48	60,2	60,2	18,36	536,04	138,21
2019/05/19	CH2205	Male	Three y/o	Treatment (Wk 1)	Treatment	Fast Day	No	23:15	23:00 - 23:59	Night	37,1	43	6,4	6,4	18,26	526,50	61,70
2019/05/19	CH2206	Male	Three y/o	Treatment (Wk 1)	Treatment	Fast Day	No	23:15	23:00 - 23:59	Night	37,2	48	68,0	68,0	18,36	536,04	142,43
2019/05/19	CH2205	Male	Three y/o	Treatment (Wk 1)	Treatment	Fast Day	No	23:20	23:00 - 23:59	Night	37,1	53	8,8	8,8	18,26	544,43	72,43
2019/05/19	CH2206	Male	Three y/o	Treatment (Wk 1)	Treatment	Fast Day	No	23:20	23:00 - 23:59	Night	37,2	41	67,6	67,6	18,36	522,29	142,23
2019/05/19	CH2205	Male	Three y/o	Treatment (Wk 1)	Treatment	Fast Day	No	23:25	23:00 - 23:59	Night	37,0	45	7,4	7,4	18,16	530,47	66,59

	2019/05/19	CH2206	Male	Three y/o	Treatment (Wk 1)	Treatment	Fast Day	No	23:25	23:00 - 23:59	Night	37,2	44	64,4	64,4	18,36	528,51	140,55
	2019/05/19	CH2205	Male	Three y/o	Treatment (Wk 1)	Treatment	Fast Day	No	23:30	23:00 - 23:59	Night	37,2	57	6,2	6,2	18,36	550,47	60,63
	2019/05/19	CH2206	Male	Three y/o	Treatment (Wk 1)	Treatment	Fast Day	No	23:30	23:00 - 23:59	Night	37,2	44	79,6	79,6	18,36	528,51	147,90
	2019/05/19	CH2205	Male	Three y/o	Treatment (Wk 1)	Treatment	Fast Day	No	23:35	23:00 - 23:59	Night	37,2	41	9,6	9,6	18,36	522,29	75,37
	2019/05/19	CH2206	Male	Three y/o	Treatment (Wk 1)	Treatment	Fast Day	No	23:35	23:00 - 23:59	Night	37,2	53	66,4	66,4	18,36	544,43	141,61
	2019/05/19	CH2205	Male	Three y/o	Treatment (Wk 1)	Treatment	Fast Day	No	23:40	23:00 - 23:59	Night	37,2	42	19,0	19,0	18,36	524,42	98,56
	2019/05/19	CH2206	Male	Three y/o	Treatment (Wk 1)	Treatment	Fast Day	No	23:40	23:00 - 23:59	Night	37,3	45	47,0	47,0	18,46	530,47	129,65
	2019/05/19	CH2205	Male	Three y/o	Treatment (Wk 1)	Treatment	Fast Day	No	23:45	23:00 - 23:59	Night	37,3	77	18,0	18,0	18,46	574,39	96,72
	2019/05/19	CH2206	Male	Three y/o	Treatment (Wk 1)	Treatment	Fast Day	No	23:45	23:00 - 23:59	Night	37,3	95	31,6	31,6	18,46	590,14	115,98
	2019/05/19	CH2205	Male	Three y/o	Treatment (Wk 1)	Treatment	Fast Day	No	23:50	23:00 - 23:59	Night	37,3	38	12,2	12,2	18,46	515,48	83,49
	2019/05/19	CH2206	Male	Three y/o	Treatment (Wk 1)	Treatment	Fast Day	No	23:50	23:00 - 23:59	Night	37,3	87	47,4	47,4	18,46	583,64	129,94
	2019/05/19	CH2205	Male	Three y/o	Treatment (Wk 1)	Treatment	Fast Day	No	23:55	23:00 - 23:59	Night	37,3	56	8,6	8,6	18,46	549,01	71,66
	2019/05/19	CH2206	Male	Three y/o	Treatment (Wk 1)	Treatment	Fast Day	No	23:55	23:00 - 23:59	Night	37,1	43	42,8	42,8	18,26	526,50	126,42
6	2019/05/20	CH2205	Male	Three y/o	Treatment (Wk 1)	Treatment	Feed Day	No	00:00	00:00 - 00:59	Early Morning	37,3	49	15,6	15,6	18,46	537,80	91,85
	2019/05/20	CH2206	Male	Three y/o	Treatment (Wk 1)	Treatment	Feed Day	No	00:00	00:00 - 00:59	Early Morning	37,3	60	42,2	42,2	18,46	554,67	125,93
	2019/05/20	CH2205	Male	Three y/o	Treatment (Wk 1)	Treatment	Feed Day	No	00:05	00:00 - 00:59	Early Morning	37,3	65	14,8	14,8	18,46	561,12	90,05
	2019/05/20	CH2206	Male	Three y/o	Treatment (Wk 1)	Treatment	Feed Day	No	00:05	00:00 - 00:59	Early Morning	37,3	57	43,2	43,2	18,46	550,47	126,74
	2019/05/20	CH2205	Male	Three y/o	Treatment (Wk 1)	Treatment	Feed Day	No	00:10	00:00 - 00:59	Early Morning	37,2	46	7,4	7,4	18,36	532,38	66,59
	2019/05/20	CH2206	Male	Three y/o	Treatment (Wk 1)	Treatment	Feed Day	No	00:10	00:00 - 00:59	Early Morning	37,2	59	1,4	1,4	18,36	553,30	11,07
	2019/05/20	CH2205	Male	Three y/o	Treatment (Wk 1)	Treatment	Feed Day	No	00:15	00:00 - 00:59	Early Morning	37,2	39	10,0	10,0	18,36	517,82	76,76
	2019/05/20	CH2206	Male	Three y/o	Treatment (Wk 1)	Treatment	Feed Day	No	00:15	00:00 - 00:59	Early Morning	37,2	79	5,8	5,8	18,36	576,36	58,39
	2019/05/20	CH2205	Male	Three y/o	Treatment (Wk 1)	Treatment	Feed Day	No	00:20	00:00 - 00:59	Early Morning	37,2	71	10,4	10,4	18,36	568,10	78,08
	2019/05/20	CH2206	Male	Three y/o	Treatment (Wk 1)	Treatment	Feed Day	No	00:20	00:00 - 00:59	Early Morning	37,2	83	6,6	6,6	18,36	580,11	62,73
	2019/05/20	CH2205	Male	Three y/o	Treatment (Wk 1)	Treatment	Feed Day	No	00:25	00:00 - 00:59	Early Morning	37,3	53	8,8	8,8	18,46	544,43	72,43
	2019/05/20	CH2206	Male	Three y/o	Treatment (Wk 1)	Treatment	Feed Day	No	00:25	00:00 - 00:59	Early Morning	37,2	42	6,4	6,4	18,36	524,42	61,70
	2019/05/20	CH2205	Male	Three y/o	Treatment (Wk 1)	Treatment	Feed Day	No	00:30	00:00 - 00:59	Early Morning	37,3	42	8,0	8,0	18,46	524,42	69,22
	2019/05/20	CH2206	Male	Three y/o	Treatment (Wk 1)	Treatment	Feed Day	No	00:30	00:00 - 00:59	Early Morning	37,2	45	8,4	8,4	18,36	530,47	70,86
	2019/05/20	CH2205	Male	Three y/o	Treatment (Wk 1)	Treatment	Feed Day	No	00:35	00:00 - 00:59	Early Morning	37,3	44	5,2	5,2	18,46	528,51	54,72
	2019/05/20	CH2206	Male	Three y/o	Treatment (Wk 1)	Treatment	Feed Day	No	00:35	00:00 - 00:59	Early Morning	37,1	48	9,8	9,8	18,26	536,04	76,07
	2019/05/20	CH2205	Male	Three y/o	Treatment (Wk 1)	Treatment	Feed Day	No	00:40	00:00 - 00:59	Early Morning	37,3	47	12,0	12,0	18,46	534,23	82,93
	2019/05/20	CH2206	Male	Three y/o	Treatment (Wk 1)	Treatment	Feed Day	No	00:40	00:00 - 00:59	Early Morning	37,1	50	5,6	5,6	18,26	539,52	57,21
	2019/05/20	CH2205	Male	Three y/o	Treatment (Wk 1)	Treatment	Feed Day	No	00:45	00:00 - 00:59	Early Morning	37,3	51	4,8	4,8	18,46	541,20	52,04
	2019/05/20	CH2206	Male	Three y/o	Treatment (Wk 1)	Treatment	Feed Day	No	00:45	00:00 - 00:59	Early Morning	37,0	44	8,2	8,2	18,16	528,51	70,05
	2019/05/20	CH2205	Male	Three y/o	Treatment (Wk 1)	Treatment	Feed Day	No	00:50	00:00 - 00:59	Early Morning	37,3	108	36,4	36,4	18,46	599,39	120,84
	2019/05/20	CH2206	Male	Three y/o	Treatment (Wk 1)	Treatment	Feed Day	No	00:50	00:00 - 00:59	Early Morning	37,0	172	75,0	75,0	18,16	630,73	145,83
	2019/05/20	CH2205	Male	Three y/o	Treatment (Wk 1)	Treatment	Feed Day	No	00:55	00:00 - 00:59	Early Morning	37,3	111	145,4	145,4	18,46	601,33	168,93
	2019/05/20	CH2206	Male	Three y/o	Treatment (Wk 1)	Treatment	Feed Day	No	00:55	00:00 - 00:59	Early Morning	37,2	121	126,0	126,0	18,36	607,35	163,91
	2019/05/20	CH2205	Male	Three y/o	Treatment (Wk 1)	Treatment	Feed Day	No	01:00	01:00 - 01:59	Early Morning	37,3	113	25,2	25,2	18,46	602,58	108,22
	2019/05/20	CH2206	Male	Three y/o	Treatment (Wk 1)	Treatment	Feed Day	No	01:00	01:00 - 01:59	Early Morning	37,3		212,0	212,0	18,46		182,17
	2019/05/20	CH2205	Male	Three y/o	Treatment (Wk 1)	Treatment	Feed Day	No	01:05	01:00 - 01:59	Early Morning	37,3	114	67,6	67,6	18,46	603,20	142,23
	2019/05/20	CH2206	Male	Three y/o	Treatment (Wk 1)	Treatment	Feed Day	No	01:05	01:00 - 01:59	Early Morning	37,4	96	64,4	64,4	18,55	590,91	140,55
	2019/05/20	CH2205	Male	Three y/o	Treatment (Wk 1)	Treatment	Feed Day	No	01:10	01:00 - 01:59	Early Morning	37,3	124	42,4	42,4	18,46	609,04	126,10
	2019/05/20	CH2206	Male	Three y/o	Treatment (Wk 1)	Treatment	Feed Day	No	01:10	01:00 - 01:59	Early Morning	37,5	123	105,8	105,8	18,65	608,49	157,81
	2019/05/20	CH2205	Male	Three y/o	Treatment (Wk 1)	Treatment	Feed Day	No	01:15	01:00 - 01:59	Early Morning	37,4	108	106,0	106,0	18,55	599,39	157,88
	2019/05/20	CH2206	Male	Three y/o	Treatment (Wk 1)	Treatment	Feed Day	No	01:15	01:00 - 01:59	Early Morning	37,7	117	79,4	79,4	18,85	605,02	147,81
	2019/05/20	CH2205	Male	Three y/o	Treatment (Wk 1)	Treatment	Feed Day	No	01:20	01:00 - 01:59	Early Morning	37,4		72,2	72,2	18,55		144,51
	2019/05/20	CH2206	Male	Three y/o	Treatment (Wk 1)	Treatment	Feed Day	No	01:20	01:00 - 01:59	Early Morning	37,7	108	172,4	172,4	18,85	599,39	174,90
	2019/05/20	CH2205	Male	Three y/o	Treatment (Wk 1)	Treatment	Feed Day	No	01:25	01:00 - 01:59	Early Morning	37,6	49	315,4	315,4	18,75	537,80	196,19
	2019/05/20	CH2206	Male	Three y/o	Treatment (Wk 1)	Treatment	Feed Day	No	01:25	01:00 - 01:59	Early Morning	37,8	100	109,0	109,0	18,95	593,87	158,85
	2019/05/20	CH2205	Male	Three y/o	Treatment (Wk 1)	Treatment	Feed Day	No	01:30	01:00 - 01:59	Early Morning	37,6		17,8	17,8	18,75		96,34
	2019/05/20	CH2206	Male	Three y/o	Treatment (Wk 1)	Treatment	Feed Day	No	01:30	01:00 - 01:59	Early Morning	37,7	90	76,2	76,2	18,85	586,16	146,38
	2019/05/20	CH2205	Male	Three y/o	Treatment (Wk 1)	Treatment	Feed Day	No	01:35	01:00 - 01:59	Early Morning	37,6	150	35,4	35,4	18,75	621,86	119,88
	2019/05/20	CH2206	Male	Three y/o	Treatment (Wk 1)	Treatment	Feed Day	No	01:35	01:00 - 01:59	Early Morning	37,7	119	139,6	139,6	18,85	606,20	167,50
	2019/05/20	CH2205	Male	Three y/o	Treatment (Wk 1)	Treatment	Feed Day	No	01:40	01:00 - 01:59	Early Morning	37,6	86	35,0	35,0	18,75	582,77	119,49
	2019/05/20	CH2206	Male	Three y/o	Treatment (Wk 1)	Treatment	Feed Day	No	01:40	01:00 - 01:59	Early Morning	37,8	121	42,6	42,6	18,95	607,35	126,26
	2019/05/20	CH2205	Male	Three y/o	Treatment (Wk 1)	Treatment	Feed Day	No	01:45	01:00 - 01:59	Early Morning	37,5		21,8	21,8	18,65		103,26
	2019/05/20	CH2206	Male	Three y/o	Treatment (Wk 1)	Treatment	Feed Day	No	01:45	01:00 - 01:59	Early Morning	37,4	73	91,8	91,8	18,55	570,27	152,86
	2019/05/20	CH2205	Male	Three y/o	Treatment (Wk 1)	Treatment	Feed Day	No	01:50	01:00 - 01:59	Early Morning	37,5	46	4,4	4,4	18,65	532,38	49,12
	2019/05/20	CH2206	Male	Three y/o	Treatment (Wk 1)	Treatment	Feed Day	No	01:50	01:00 - 01:59	Early Morning	37,4	99	57,8	57,8	18,55	593,14	136,80
	2019/05/20	CH2205	Male	Three y/o	Treatment (Wk 1)	Treatment	Feed Day	No	01:55	01:00 - 01:59	Early Morning	37,5	49	5,6	5,6	18,65	537,80	57,21
	2019/05/20	CH2206	Male	Three y/o	Treatment (Wk 1)	Treatment	Feed Day	No	01:55	01:00 - 01:59	Early Morning	37,5	119	82,6	82,6	18,65	606,20	149,19
	2019/05/20	CH2205	Male	Three y/o	Treatment (Wk 1)	Treatment	Feed Day	No	02:00	02:00 - 02:59	Early Morning	37,6	83	20,4	20,4	18,75	580,11	100,99
	2019/05/20	CH2206	Male	Three y/o	Treatment (Wk 1)	Treatment	Feed Day	No	02:00	02:00 - 02:59	Early Morning	37,5	92	55,0	55,0	18,65	587,78	135,08
	2019/05/20	CH2205	Male	Three y/o	Treatment (Wk 1)	Treatment	Feed Day	No	02:05	02:00 - 02:59	Early Morning	37,5	112	27,2	27,2	18,65	601,96	110,83
	2019/05/20	CH2206	Male	Three y/o	Treatment (Wk 1)	Treatment	Feed Day	No	02:05	02:00 - 02:59	Early Morning	37,5	101	94,0	94,0	18,65	594,59	153,69
	2019/05/20	CH2205	Male	Three y/o	Treatment (Wk 1)	Treatment	Feed Day	No	02:10	02:00 - 02:59	Early Morning	37,5		27,6	27,6	18,65		111,33
	2019/05/20	CH2206	Male	Three y/o	Treatment (Wk 1)	Treatment	Feed Day	No	02:10	02:00 - 02:59	Early Morning	37,7	105	253,6	253,6	18,85	597,38	188,48
	2019/05/20	CH2205	Male	Three y/o	Treatment (Wk 1)	Treatment	Feed Day	No	02:15	02:00 - 02:59	Early Morning	37,5		29,8	29,8	18,65		113,97
	2019/05/20	CH2206	Male	Three y/o	Treatment (Wk 1)	Treatment	Feed Day	No	02:15	02:00 - 02:59	Early Morning	37,7	51	43,2	43,2	18,85	541,20	126,74
	2019/05/20	CH2205	Male	Three y/o	Treatment (Wk 1)	Treatment	Feed Day	No	02:20	02:00 - 02:59	Early Morning	37,5	39	20,2	20,2	18,65	517,82	100,65

2019/05/20	CH2206	Male	Three y/o	Treatment (Wk 1)	Treatment	Feed Day	No	02:20	02:00 - 02:59	Early Morning	37,3	86	49,6	49,6	18,46	582,77	131,51
2019/05/20	CH2205	Male	Three y/o	Treatment (Wk 1)	Treatment	Feed Day	No	02:25	02:00 - 02:59	Early Morning	37,4	51	4,8	4,8	18,55	541,20	52,04
2019/05/20	CH2206	Male	Three y/o	Treatment (Wk 1)	Treatment	Feed Day	No	02:25	02:00 - 02:59	Early Morning	37,1	100	54,6	54,6	18,26	593,87	134,83
2019/05/20	CH2205	Male	Three y/o	Treatment (Wk 1)	Treatment	Feed Day	No	02:30	02:00 - 02:59	Early Morning	37,4	52	4,2	4,2	18,55	542,83	47,57
2019/05/20	CH2206	Male	Three y/o	Treatment (Wk 1)	Treatment	Feed Day	No	02:30	02:00 - 02:59	Early Morning	37,0	78	58,8	58,8	18,16	575,38	137,39
2019/05/20	CH2205	Male	Three y/o	Treatment (Wk 1)	Treatment	Feed Day	No	02:35	02:00 - 02:59	Early Morning	37,3	45	5,2	5,2	18,46	530,47	54,72
2019/05/20	CH2206	Male	Three y/o	Treatment (Wk 1)	Treatment	Feed Day	No	02:35	02:00 - 02:59	Early Morning	37,0	72	57,8	57,8	18,16	569,20	136,80
2019/05/20	CH2205	Male	Three y/o	Treatment (Wk 1)	Treatment	Feed Day	No	02:40	02:00 - 02:59	Early Morning	37,3	64	9,0	9,0	18,46	559,88	73,19
2019/05/20	CH2206	Male	Three y/o	Treatment (Wk 1)	Treatment	Feed Day	No	02:40	02:00 - 02:59	Early Morning	37,1	47	51,8	51,8	18,26	534,23	133,01
2019/05/20	CH2205	Male	Three y/o	Treatment (Wk 1)	Treatment	Feed Day	No	02:45	02:00 - 02:59	Early Morning	37,3	73	5,6	5,6	18,46	570,27	57,21
2019/05/20	CH2206	Male	Three y/o	Treatment (Wk 1)	Treatment	Feed Day	No	02:45	02:00 - 02:59	Early Morning	37,2	66	44,4	44,4	18,36	562,34	127,69
2019/05/20	CH2205	Male	Three y/o	Treatment (Wk 1)	Treatment	Feed Day	No	02:50	02:00 - 02:59	Early Morning	37,3	45	5,0	5,0	18,46	530,47	53,41
2019/05/20	CH2206	Male	Three y/o	Treatment (Wk 1)	Treatment	Feed Day	No	02:50	02:00 - 02:59	Early Morning	37,3	47	6,8	6,8	18,46	534,23	63,74
2019/05/20	CH2205	Male	Three y/o	Treatment (Wk 1)	Treatment	Feed Day	No	02:55	02:00 - 02:59	Early Morning	37,3	67	6,4	6,4	18,46	563,53	61,70
2019/05/20	CH2206	Male	Three y/o	Treatment (Wk 1)	Treatment	Feed Day	No	02:55	02:00 - 02:59	Early Morning	37,3	46	12,8	12,8	18,46	532,38	85,12
2019/05/20	CH2205	Male	Three y/o	Treatment (Wk 1)	Treatment	Feed Day	No	03:00	03:00 - 03:59	Early Morning	37,3	39	17,2	17,2	18,46	517,82	95,17
2019/05/20	CH2206	Male	Three y/o	Treatment (Wk 1)	Treatment	Feed Day	No	03:00	03:00 - 03:59	Early Morning	37,3	72	24,6	24,6	18,46	569,20	107,39
2019/05/20	CH2205	Male	Three y/o	Treatment (Wk 1)	Treatment	Feed Day	No	03:05	03:00 - 03:59	Early Morning	37,3		25,0	25,0	18,46		107,94
2019/05/20	CH2206	Male	Three y/o	Treatment (Wk 1)	Treatment	Feed Day	No	03:05	03:00 - 03:59	Early Morning	37,3	47	57,6	57,6	18,46	534,23	136,68
2019/05/20	CH2205	Male	Three y/o	Treatment (Wk 1)	Treatment	Feed Day	No	03:10	03:00 - 03:59	Early Morning	37,3		19,4	19,4	18,46		99,28
2019/05/20	CH2206	Male	Three y/o	Treatment (Wk 1)	Treatment	Feed Day	No	03:10	03:00 - 03:59	Early Morning	37,3	79	57,4	57,4	18,46	576,36	136,56
2019/05/20	CH2205	Male	Three y/o	Treatment (Wk 1)	Treatment	Feed Day	No	03:15	03:00 - 03:59	Early Morning	37,3	42	13,2	13,2	18,46	524,42	86,17
2019/05/20	CH2206	Male	Three y/o	Treatment (Wk 1)	Treatment	Feed Day	No	03:15	03:00 - 03:59	Early Morning	37,3	41	57,6	57,6	18,46	522,29	136,68
2019/05/20	CH2205	Male	Three y/o	Treatment (Wk 1)	Treatment	Feed Day	No	03:20	03:00 - 03:59	Early Morning	37,3	46	21,6	21,6	18,46	532,38	102,94
2019/05/20	CH2206	Male	Three y/o	Treatment (Wk 1)	Treatment	Feed Day	No	03:20	03:00 - 03:59	Early Morning	37,3	65	54,4	54,4	18,46	561,12	134,70
2019/05/20	CH2205	Male	Three y/o	Treatment (Wk 1)	Treatment	Feed Day	No	03:25	03:00 - 03:59	Early Morning	37,2	63	19,2	19,2	18,36	558,62	98,92
2019/05/20	CH2206	Male	Three y/o	Treatment (Wk 1)	Treatment	Feed Day	No	03:25	03:00 - 03:59	Early Morning	37,3	76	61,4	61,4	18,46	573,39	138,89
2019/05/20	CH2205	Male	Three y/o	Treatment (Wk 1)	Treatment	Feed Day	No	03:30	03:00 - 03:59	Early Morning	37,2	70	20,0	20,0	18,36	566,99	100,31
2019/05/20	CH2206	Male	Three y/o	Treatment (Wk 1)	Treatment	Feed Day	No	03:30	03:00 - 03:59	Early Morning	37,3	73	59,4	59,4	18,46	570,27	137,75
2019/05/20	CH2205	Male	Three y/o	Treatment (Wk 1)	Treatment	Feed Day	No	03:35	03:00 - 03:59	Early Morning	37,2	98	17,8	17,8	18,36	592,41	96,34
2019/05/20	CH2206	Male	Three y/o	Treatment (Wk 1)	Treatment	Feed Day	No	03:35	03:00 - 03:59	Early Morning	37,3	41	62,8	62,8	18,46	522,29	139,67
2019/05/20	CH2205	Male	Three y/o	Treatment (Wk 1)	Treatment	Feed Day	No	03:40	03:00 - 03:59	Early Morning	37,2	63	22,6	22,6	18,36	558,62	104,49
2019/05/20	CH2206	Male	Three y/o	Treatment (Wk 1)	Treatment	Feed Day	No	03:40	03:00 - 03:59	Early Morning	37,2	42	62,2	62,2	18,36	524,42	139,34
2019/05/20	CH2205	Male	Three y/o	Treatment (Wk 1)	Treatment	Feed Day	No	03:45	03:00 - 03:59	Early Morning	37,2	69	20,0	20,0	18,36	565,86	100,31
2019/05/20	CH2206	Male	Three y/o	Treatment (Wk 1)	Treatment	Feed Day	No	03:45	03:00 - 03:59	Early Morning	37,2	48	52,6	52,6	18,36	536,04	133,54
2019/05/20	CH2205	Male	Three y/o	Treatment (Wk 1)	Treatment	Feed Day	No	03:50	03:00 - 03:59	Early Morning	37,2	79	20,2	20,2	18,36	576,36	100,65
2019/05/20	CH2206	Male	Three y/o	Treatment (Wk 1)	Treatment	Feed Day	No	03:50	03:00 - 03:59	Early Morning	37,2	45	13,6	13,6	18,36	530,47	87,18
2019/05/20	CH2205	Male	Three y/o	Treatment (Wk 1)	Treatment	Feed Day	No	03:55	03:00 - 03:59	Early Morning	37,2	49	19,4	19,4	18,36	537,80	99,28
2019/05/20	CH2206	Male	Three y/o	Treatment (Wk 1)	Treatment	Feed Day	No	03:55	03:00 - 03:59	Early Morning	37,2	76	11,4	11,4	18,36	573,39	81,19
2019/05/20	CH2205	Male	Three y/o	Treatment (Wk 1)	Treatment	Feed Day	No	04:00	04:00 - 04:59	Morning	37,2	57	21,0	21,0	18,36	550,47	101,98
2019/05/20	CH2206	Male	Three y/o	Treatment (Wk 1)	Treatment	Feed Day	No	04:00	04:00 - 04:59	Morning	37,2	49	9,6	9,6	18,36	537,80	75,37
2019/05/20	CH2205	Male	Three y/o	Treatment (Wk 1)	Treatment	Feed Day	No	04:05	04:00 - 04:59	Morning	37,2	82	19,0	19,0	18,36	579,19	98,56
2019/05/20	CH2206	Male	Three y/o	Treatment (Wk 1)	Treatment	Feed Day	No	04:05	04:00 - 04:59	Morning	37,1	63	9,2	9,2	18,26	558,62	73,94
2019/05/20	CH2205	Male	Three y/o	Treatment (Wk 1)	Treatment	Feed Day	No	04:10	04:00 - 04:59	Morning	37,2	102	19,2	19,2	18,36	595,30	98,92
2019/05/20	CH2206	Male	Three y/o	Treatment (Wk 1)	Treatment	Feed Day	No	04:10	04:00 - 04:59	Morning	37,1	46	9,4	9,4	18,26	532,38	74,66
2019/05/20	CH2205	Male	Three y/o	Treatment (Wk 1)	Treatment	Feed Day	No	04:15	04:00 - 04:59	Morning	37,2	39	19,4	19,4	18,36	517,82	99,28
2019/05/20	CH2206	Male	Three y/o	Treatment (Wk 1)	Treatment	Feed Day	No	04:15	04:00 - 04:59	Morning	37,1	51	11,4	11,4	18,26	541,20	81,19
2019/05/20	CH2205	Male	Three y/o	Treatment (Wk 1)	Treatment	Feed Day	No	04:20	04:00 - 04:59	Morning	37,2	46	13,4	13,4	18,36	532,38	86,68
2019/05/20	CH2206	Male	Three y/o	Treatment (Wk 1)	Treatment	Feed Day	No	04:20	04:00 - 04:59	Morning	37,1	47	13,8	13,8	18,26	534,23	87,68
2019/05/20	CH2205	Male	Three y/o	Treatment (Wk 1)	Treatment	Feed Day	No	04:25	04:00 - 04:59	Morning	37,2	42	18,0	18,0	18,36	524,42	96,72
2019/05/20	CH2206	Male	Three y/o	Treatment (Wk 1)	Treatment	Feed Day	No	04:25	04:00 - 04:59	Morning	37,1	46	14,2	14,2	18,26	532,38	88,65
2019/05/20	CH2205	Male	Three y/o	Treatment (Wk 1)	Treatment	Feed Day	No	04:30	04:00 - 04:59	Morning	37,2	48	15,8	15,8	18,36	536,04	92,28
2019/05/20	CH2206	Male	Three y/o	Treatment (Wk 1)	Treatment	Feed Day	No	04:30	04:00 - 04:59	Morning	37,1	48	35,8	35,8	18,26	536,04	120,27
2019/05/20	CH2205	Male	Three y/o	Treatment (Wk 1)	Treatment	Feed Day	No	04:35	04:00 - 04:59	Morning	37,2	58	14,0	14,0	18,36	551,90	88,17
2019/05/20	CH2206	Male	Three y/o	Treatment (Wk 1)	Treatment	Feed Day	No	04:35	04:00 - 04:59	Morning	37,1	64	17,2	17,2	18,26	559,88	95,17
2019/05/20	CH2205	Male	Three y/o	Treatment (Wk 1)	Treatment	Feed Day	No	04:40	04:00 - 04:59	Morning	37,1	38	15,0	15,0	18,26	515,48	90,51
2019/05/20	CH2206	Male	Three y/o	Treatment (Wk 1)	Treatment	Feed Day	No	04:40	04:00 - 04:59	Morning	37,1	47	17,6	17,6	18,26	534,23	95,95
2019/05/20	CH2205	Male	Three y/o	Treatment (Wk 1)	Treatment	Feed Day	No	04:45	04:00 - 04:59	Morning	37,1	39	35,0	35,0	18,26	517,82	119,49
2019/05/20	CH2206	Male	Three y/o	Treatment (Wk 1)	Treatment	Feed Day	No	04:45	04:00 - 04:59	Morning	37,1	73	20,6	20,6	18,26	570,27	101,32
2019/05/20	CH2205	Male	Three y/o	Treatment (Wk 1)	Treatment	Feed Day	No	04:50	04:00 - 04:59	Morning	37,1	43	31,4	31,4	18,26	526,50	115,76
2019/05/20	CH2206	Male	Three y/o	Treatment (Wk 1)	Treatment	Feed Day	No	04:50	04:00 - 04:59	Morning	37,0	45	35,8	35,8	18,16	530,47	120,27
2019/05/20	CH2205	Male	Three y/o	Treatment (Wk 1)	Treatment	Feed Day	No	04:55	04:00 - 04:59	Morning	37,1	56	30,8	30,8	18,26	549,01	115,10
2019/05/20	CH2206	Male	Three y/o	Treatment (Wk 1)	Treatment	Feed Day	No	04:55	04:00 - 04:59	Morning	37,0	41	42,2	42,2	18,16	522,29	125,93
2019/05/20	CH2205	Male	Three y/o	Treatment (Wk 1)	Treatment	Feed Day	No	05:00	05:00 - 05:59	Morning	37,2		33,0	33,0	18,36		117,47
2019/05/20	CH2206	Male	Three y/o	Treatment (Wk 1)	Treatment	Feed Day	No	05:00	05:00 - 05:59	Morning	37,0	49	60,0	60,0	18,16	537,80	138,09
2019/05/20	CH2205	Male	Three y/o	Treatment (Wk 1)	Treatment	Feed Day	No	05:05	05:00 - 05:59	Morning	37,1		30,6	30,6	18,26		114,87
2019/05/20	CH2206	Male	Three y/o	Treatment (Wk 1)	Treatment	Feed Day	No	05:05	05:00 - 05:59	Morning	36,9	43	24,8	24,8	18,06	526,50	107,67
2019/05/20	CH2205	Male	Three y/o	Treatment (Wk 1)	Treatment	Feed Day	No	05:10	05:00 - 05:59	Morning	37,0	51	20,6	20,6	18,16	541,20	101,32
2019/05/20	CH2206	Male	Three y/o	Treatment (Wk 1)	Treatment	Feed Day	No	05:10	05:00 - 05:59	Morning	36,9	54	3,0	3,0	18,06	545,99	36,34
2019/05/20	CH2205	Male	Three y/o	Treatment (Wk 1)	Treatment	Feed Day	No	05:15	05:00 - 05:59	Morning	37,0	44	23,8	23,8	18,16	528,51	106,26

2019/05/20	CH2206	Male	Three y/o	Treatment (Wk 1)	Treatment	Feed Day	No	05:15	05:00 - 05:59	Morning	36,9	48	5,4	5,4	18,06	536,04	55,99
2019/05/20	CH2205	Male	Three y/o	Treatment (Wk 1)	Treatment	Feed Day	No	05:20	05:00 - 05:59	Morning	37,0	41	25,4	25,4	18,16	522,29	108,49
2019/05/20	CH2206	Male	Three y/o	Treatment (Wk 1)	Treatment	Feed Day	No	05:20	05:00 - 05:59	Morning	36,9	44	4,2	4,2	18,06	528,51	47,57
2019/05/20	CH2205	Male	Three y/o	Treatment (Wk 1)	Treatment	Feed Day	No	05:25	05:00 - 05:59	Morning	37,0	50	24,4	24,4	18,16	539,52	107,11
2019/05/20	CH2206	Male	Three y/o	Treatment (Wk 1)	Treatment	Feed Day	No	05:25	05:00 - 05:59	Morning	36,9	37	1,0	1,0	18,06	513,06	0,00
2019/05/20	CH2205	Male	Three y/o	Treatment (Wk 1)	Treatment	Feed Day	No	05:30	05:00 - 05:59	Morning	37,0	48	23,8	23,8	18,16	536,04	106,26
2019/05/20	CH2206	Male	Three y/o	Treatment (Wk 1)	Treatment	Feed Day	No	05:30	05:00 - 05:59	Morning	36,9	48	3,4	3,4	18,06	536,04	40,51
2019/05/20	CH2205	Male	Three y/o	Treatment (Wk 1)	Treatment	Feed Day	No	05:35	05:00 - 05:59	Morning	37,0	55	24,4	24,4	18,16	547,52	107,11
2019/05/20	CH2206	Male	Three y/o	Treatment (Wk 1)	Treatment	Feed Day	No	05:35	05:00 - 05:59	Morning	36,9	47	21,0	21,0	18,06	534,23	101,98
2019/05/20	CH2205	Male	Three y/o	Treatment (Wk 1)	Treatment	Feed Day	No	05:40	05:00 - 05:59	Morning	37,1	46	16,8	16,8	18,26	532,38	94,37
2019/05/20	CH2206	Male	Three y/o	Treatment (Wk 1)	Treatment	Feed Day	No	05:40	05:00 - 05:59	Morning	37,0	43	42,6	42,6	18,16	526,50	126,26
2019/05/20	CH2205	Male	Three y/o	Treatment (Wk 1)	Treatment	Feed Day	No	05:45	05:00 - 05:59	Morning	37,1	49	15,4	15,4	18,26	537,80	91,41
2019/05/20	CH2206	Male	Three y/o	Treatment (Wk 1)	Treatment	Feed Day	No	05:45	05:00 - 05:59	Morning	37,0	64	52,4	52,4	18,16	559,88	133,41
2019/05/20	CH2205	Male	Three y/o	Treatment (Wk 1)	Treatment	Feed Day	No	05:50	05:00 - 05:59	Morning	37,1	46	14,0	14,0	18,26	532,38	88,17
2019/05/20	CH2206	Male	Three y/o	Treatment (Wk 1)	Treatment	Feed Day	No	05:50	05:00 - 05:59	Morning	37,0	67	58,4	58,4	18,16	563,53	137,16
2019/05/20	CH2205	Male	Three y/o	Treatment (Wk 1)	Treatment	Feed Day	No	05:55	05:00 - 05:59	Morning	37,2	134	30,2	30,2	18,36	614,33	114,42
2019/05/20	CH2206	Male	Three y/o	Treatment (Wk 1)	Treatment	Feed Day	No	05:55	05:00 - 05:59	Morning	37,1	119	52,0	52,0	18,26	606,20	133,14
2019/05/20	CH2205	Male	Three y/o	Treatment (Wk 1)	Treatment	Feed Day	No	06:00	06:00 - 06:59	Morning	37,2	108	62,2	62,2	18,36	599,39	139,34
2019/05/20	CH2206	Male	Three y/o	Treatment (Wk 1)	Treatment	Feed Day	No	06:00	06:00 - 06:59	Morning	37,2	134	178,6	178,6	18,36	614,33	176,14
2019/05/20	CH2205	Male	Three y/o	Treatment (Wk 1)	Treatment	Feed Day	No	06:05	06:00 - 06:59	Morning	37,2	115	119,6	119,6	18,36	603,82	162,09
2019/05/20	CH2206	Male	Three y/o	Treatment (Wk 1)	Treatment	Feed Day	No	06:05	06:00 - 06:59	Morning	37,3		108,2	108,2	18,46		158,59
2019/05/20	CH2205	Male	Three y/o	Treatment (Wk 1)	Treatment	Feed Day	No	06:10	06:00 - 06:59	Morning	37,2	84	56,4	56,4	18,36	581,01	135,95
2019/05/20	CH2206	Male	Three y/o	Treatment (Wk 1)	Treatment	Feed Day	No	06:10	06:00 - 06:59	Morning	37,3	124	75,2	75,2	18,46	609,04	145,93
2019/05/20	CH2205	Male	Three y/o	Treatment (Wk 1)	Treatment	Feed Day	No	06:15	06:00 - 06:59	Morning	37,2	191	387,4	387,4	18,36	637,33	203,48
2019/05/20	CH2206	Male	Three y/o	Treatment (Wk 1)	Treatment	Feed Day	No	06:15	06:00 - 06:59	Morning	37,5	47	136,2	136,2	18,65	534,23	166,64
2019/05/20	CH2205	Male	Three y/o	Treatment (Wk 1)	Treatment	Feed Day	No	06:20	06:00 - 06:59	Morning	37,3	109	205,2	205,2	18,46	600,04	181,02
2019/05/20	CH2206	Male	Three y/o	Treatment (Wk 1)	Treatment	Feed Day	No	06:20	06:00 - 06:59	Morning	37,7	125	193,4	193,4	18,85	609,60	178,94
2019/05/20	CH2205	Male	Three y/o	Treatment (Wk 1)	Treatment	Feed Day	No	06:25	06:00 - 06:59	Morning	37,4	117	248,8	248,8	18,55	605,02	187,81
2019/05/20	CH2206	Male	Three y/o	Treatment (Wk 1)	Treatment	Feed Day	No	06:25	06:00 - 06:59	Morning	37,7	114	142,2	142,2	18,85	603,20	168,15
2019/05/20	CH2205	Male	Three y/o	Treatment (Wk 1)	Treatment	Feed Day	No	06:30	06:00 - 06:59	Morning	37,5	103	85,6	85,6	18,65	596,00	150,43
2019/05/20	CH2206	Male	Three y/o	Treatment (Wk 1)	Treatment	Feed Day	No	06:30	06:00 - 06:59	Morning	37,8	123	364,6	364,6	18,95	608,49	201,33
2019/05/20	CH2205	Male	Three y/o	Treatment (Wk 1)	Treatment	Feed Day	No	06:35	06:00 - 06:59	Morning	37,5	84	27,4	27,4	18,65	581,01	111,09
2019/05/20	CH2206	Male	Three y/o	Treatment (Wk 1)	Treatment	Feed Day	No	06:35	06:00 - 06:59	Morning	37,9	132	148,8	148,8	19,05	613,32	169,74
2019/05/20	CH2205	Male	Three y/o	Treatment (Wk 1)	Treatment	Feed Day	No	06:40	06:00 - 06:59	Morning	37,5	53	368,0	368,0	18,65	544,43	201,66
2019/05/20	CH2206	Male	Three y/o	Treatment (Wk 1)	Treatment	Feed Day	No	06:40	06:00 - 06:59	Morning	37,9	104	77,4	77,4	19,05	596,69	146,93
2019/05/20	CH2205	Male	Three y/o	Treatment (Wk 1)	Treatment	Feed Day	No	06:45	06:00 - 06:59	Morning	37,6	115	82,2	82,2	18,75	603,82	149,02
2019/05/20	CH2206	Male	Three y/o	Treatment (Wk 1)	Treatment	Feed Day	No	06:45	06:00 - 06:59	Morning	37,9	119	193,6	193,6	19,05	606,20	178,97
2019/05/20	CH2205	Male	Three y/o	Treatment (Wk 1)	Treatment	Feed Day	No	06:50	06:00 - 06:59	Morning	37,6	88	41,0	41,0	18,75	584,49	124,94
2019/05/20	CH2206	Male	Three y/o	Treatment (Wk 1)	Treatment	Feed Day	No	06:50	06:00 - 06:59	Morning	37,8	119	336,8	336,8	18,95	606,20	198,52
2019/05/20	CH2205	Male	Three y/o	Treatment (Wk 1)	Treatment	Feed Day	No	06:55	06:00 - 06:59	Morning	37,6		157,6	157,6	18,75		171,75
2019/05/20	CH2206	Male	Three y/o	Treatment (Wk 1)	Treatment	Feed Day	No	06:55	06:00 - 06:59	Morning	37,9	111	103,4	103,4	19,05	601,33	157,01
2019/05/20	CH2205	Male	Three y/o	Treatment (Wk 1)	Treatment	Feed Day	No	07:00	07:00 - 07:59	Morning	37,7	49	54,8	54,8	18,85	537,80	134,96
2019/05/20	CH2206	Male	Three y/o	Treatment (Wk 1)	Treatment	Feed Day	No	07:00	07:00 - 07:59	Morning	37,9	106	252,0	252,0	19,05	598,06	188,26
2019/05/20	CH2205	Male	Three y/o	Treatment (Wk 1)	Treatment	Feed Day	No	07:05	07:00 - 07:59	Morning	37,6	74	18,4	18,4	18,75	571,33	97,47
2019/05/20	CH2206	Male	Three y/o	Treatment (Wk 1)	Treatment	Feed Day	No	07:05	07:00 - 07:59	Morning	37,9	59	188,8	188,8	19,05	553,30	178,09
2019/05/20	CH2205	Male	Three y/o	Treatment (Wk 1)	Treatment	Feed Day	No	07:10	07:00 - 07:59	Morning	37,5		20,0	20,0	18,65		100,31
2019/05/20	CH2206	Male	Three y/o	Treatment (Wk 1)	Treatment	Feed Day	No	07:10	07:00 - 07:59	Morning	37,9	137	115,4	115,4	19,05	615,83	160,84
2019/05/20	CH2205	Male	Three y/o	Treatment (Wk 1)	Treatment	Feed Day	No	07:15	07:00 - 07:59	Morning	37,3	88	129,4	129,4	18,46	584,49	164,84
2019/05/20	CH2206	Male	Three y/o	Treatment (Wk 1)	Treatment	Feed Day	No	07:15	07:00 - 07:59	Morning	37,9	121	495,2	495,2	19,05	607,35	212,20
2019/05/20	CH2205	Male	Three y/o	Treatment (Wk 1)	Treatment	Feed Day	No	07:20	07:00 - 07:59	Morning	37,7	71	88,4	88,4	18,85	568,10	151,55
2019/05/20	CH2206	Male	Three y/o	Treatment (Wk 1)	Treatment	Feed Day	No	07:20	07:00 - 07:59	Morning	37,9		185,6	185,6	19,05		177,49
2019/05/20	CH2205	Male	Three y/o	Treatment (Wk 1)	Treatment	Feed Day	No	07:25	07:00 - 07:59	Morning	37,9	94	39,8	39,8	19,05	589,37	123,91
2019/05/20	CH2206	Male	Three y/o	Treatment (Wk 1)	Treatment	Feed Day	No	07:25	07:00 - 07:59	Morning	38,0	112	221,8	221,8	19,15	601,96	183,76
2019/05/20	CH2205	Male	Three y/o	Treatment (Wk 1)	Treatment	Feed Day	No	07:30	07:00 - 07:59	Morning	37,7	102	118,0	118,0	18,85	595,30	161,62
2019/05/20	CH2206	Male	Three y/o	Treatment (Wk 1)	Treatment	Feed Day	No	07:30	07:00 - 07:59	Morning	37,9	94	282,0	282,0	19,05	589,37	192,23
2019/05/20	CH2205	Male	Three y/o	Treatment (Wk 1)	Treatment	Feed Day	No	07:35	07:00 - 07:59	Morning	37,7	112	181,6	181,6	18,85	601,96	176,73
2019/05/20	CH2206	Male	Three y/o	Treatment (Wk 1)	Treatment	Feed Day	No	07:35	07:00 - 07:59	Morning	38,0	118	141,8	141,8	19,15	605,61	168,05
2019/05/20	CH2205	Male	Three y/o	Treatment (Wk 1)	Treatment	Feed Day	No	07:40	07:00 - 07:59	Morning	37,7	115	143,8	143,8	18,85	603,82	168,54
2019/05/20	CH2206	Male	Three y/o	Treatment (Wk 1)	Treatment	Feed Day	No	07:40	07:00 - 07:59	Morning	37,9	117	117,0	117,0	19,05	605,02	161,32
2019/05/20	CH2205	Male	Three y/o	Treatment (Wk 1)	Treatment	Feed Day	No	07:45	07:00 - 07:59	Morning	37,7	105	91,4	91,4	18,85	597,38	152,71
2019/05/20	CH2206	Male	Three y/o	Treatment (Wk 1)	Treatment	Feed Day	No	07:45	07:00 - 07:59	Morning	37,9	105	306,6	306,6	19,05	597,38	195,19
2019/05/20	CH2205	Male	Three y/o	Treatment (Wk 1)	Treatment	Feed Day	No	07:50	07:00 - 07:59	Morning	37,7	109	100,6	100,6	18,85	600,04	156,05
2019/05/20	CH2206	Male	Three y/o	Treatment (Wk 1)	Treatment	Feed Day	No	07:50	07:00 - 07:59	Morning	37,9	126	259,6	259,6	19,05	610,14	189,31
2019/05/20	CH2205	Male	Three y/o	Treatment (Wk 1)	Treatment	Feed Day	No	07:55	07:00 - 07:59	Morning	37,6		23,0	23,0	18,75		105,09
2019/05/20	CH2206	Male	Three y/o	Treatment (Wk 1)	Treatment	Feed Day	No	07:55	07:00 - 07:59	Morning	37,9	93	48,8	48,8	19,05	588,58	130,95
2019/05/20	CH2205	Male	Three y/o	Treatment (Wk 1)	Treatment	Feed Day	No	08:00	08:00 - 08:59	Late Morning	37,4		30,6	30,6	18,55		114,87
2019/05/20	CH2206	Male	Three y/o	Treatment (Wk 1)	Treatment	Feed Day	No	08:00	08:00 - 08:59	Late Morning	37,8	103	164,0	164,0	18,95	596,00	173,15
2019/05/20	CH2205	Male	Three y/o	Treatment (Wk 1)	Treatment	Feed Day	No	08:05	08:00 - 08:59	Late Morning	37,4		19,6	19,6	18,55		99,63
2019/05/20	CH2206	Male	Three y/o	Treatment (Wk 1)	Treatment	Feed Day	No	08:05	08:00 - 08:59	Late Morning	37,8	114	55,0	55,0	18,95	603,20	135,08
2019/05/20	CH2205	Male	Three y/o	Treatment (Wk 1)	Treatment	Feed Day	No	08:10	08:00 - 08:59	Late Morning	37,4	59	259,6	259,6	18,55	553,30	189,31

2019/05/20	CH2206	Male	Three y/o	Treatment (Wk 1)	Treatment	Feed Day	No	08:10	08:00 - 08:59	Late Morning	37,8	107	239,2	239,2	18,95	598,73	186,42
2019/05/20	CH2205	Male	Three y/o	Treatment (Wk 1)	Treatment	Feed Day	No	08:15	08:00 - 08:59	Late Morning	37,4	102	32,8	32,8	18,55	595,30	117,26
2019/05/20	CH2206	Male	Three y/o	Treatment (Wk 1)	Treatment	Feed Day	No	08:15	08:00 - 08:59	Late Morning	37,8	115	179,8	179,8	18,95	603,82	176,38
2019/05/20	CH2205	Male	Three y/o	Treatment (Wk 1)	Treatment	Feed Day	No	08:20	08:00 - 08:59	Late Morning	37,3	104	146,0	146,0	18,46	596,69	169,07
2019/05/20	CH2206	Male	Three y/o	Treatment (Wk 1)	Treatment	Feed Day	No	08:20	08:00 - 08:59	Late Morning	37,8	105	341,6	341,6	18,95	597,38	199,02
2019/05/20	CH2205	Male	Three y/o	Treatment (Wk 1)	Treatment	Feed Day	No	08:25	08:00 - 08:59	Late Morning	37,4	98	140,2	140,2	18,55	592,41	167,65
2019/05/20	CH2206	Male	Three y/o	Treatment (Wk 1)	Treatment	Feed Day	No	08:25	08:00 - 08:59	Late Morning	37,8	104	141,0	141,0	18,95	596,69	167,85
2019/05/20	CH2205	Male	Three y/o	Treatment (Wk 1)	Treatment	Feed Day	No	08:30	08:00 - 08:59	Late Morning	37,4	174	306,6	306,6	18,55	631,46	195,19
2019/05/20	CH2206	Male	Three y/o	Treatment (Wk 1)	Treatment	Feed Day	No	08:30	08:00 - 08:59	Late Morning	37,8	114	100,0	100,0	18,95	603,20	155,84
2019/05/20	CH2205	Male	Three y/o	Treatment (Wk 1)	Treatment	Feed Day	No	08:35	08:00 - 08:59	Late Morning	37,5	188	17,0	17,0	18,65	636,34	94,77
2019/05/20	CH2206	Male	Three y/o	Treatment (Wk 1)	Treatment	Feed Day	No	08:35	08:00 - 08:59	Late Morning	37,8	105	201,2	201,2	18,95	597,38	180,33
2019/05/20	CH2205	Male	Three y/o	Treatment (Wk 1)	Treatment	Feed Day	No	08:40	08:00 - 08:59	Late Morning	37,5	59	4,4	4,4	18,65	553,30	49,12
2019/05/20	CH2206	Male	Three y/o	Treatment (Wk 1)	Treatment	Feed Day	No	08:40	08:00 - 08:59	Late Morning	37,8	108	194,2	194,2	18,95	599,39	179,08
2019/05/20	CH2205	Male	Three y/o	Treatment (Wk 1)	Treatment	Feed Day	No	08:45	08:00 - 08:59	Late Morning	37,7	160	24,6	24,6	18,85	626,07	107,39
2019/05/20	CH2206	Male	Three y/o	Treatment (Wk 1)	Treatment	Feed Day	No	08:45	08:00 - 08:59	Late Morning	37,8	123	67,8	67,8	18,95	608,49	142,33
2019/05/20	CH2205	Male	Three y/o	Treatment (Wk 1)	Treatment	Feed Day	No	08:50	08:00 - 08:59	Late Morning	37,6	98	145,4	145,4	18,75	592,41	168,93
2019/05/20	CH2206	Male	Three y/o	Treatment (Wk 1)	Treatment	Feed Day	No	08:50	08:00 - 08:59	Late Morning	37,9	104	183,0	183,0	19,05	596,69	177,00
2019/05/20	CH2205	Male	Three y/o	Treatment (Wk 1)	Treatment	Feed Day	No	08:55	08:00 - 08:59	Late Morning	37,6	66	4,0	4,0	18,75	562,34	45,94
2019/05/20	CH2206	Male	Three y/o	Treatment (Wk 1)	Treatment	Feed Day	No	08:55	08:00 - 08:59	Late Morning	37,9	77	78,8	78,8	19,05	574,39	147,55
2019/05/20	CH2205	Male	Three y/o	Treatment (Wk 1)	Treatment	Feed Day	No	09:00	09:00 - 09:59	Late Morning	37,7	55	4,8	4,8	18,85	547,52	52,04
2019/05/20	CH2206	Male	Three y/o	Treatment (Wk 1)	Treatment	Feed Day	No	09:00	09:00 - 09:59	Late Morning	37,9	128	119,2	119,2	19,05	611,22	161,97
2019/05/20	CH2205	Male	Three y/o	Treatment (Wk 1)	Treatment	Feed Day	No	09:05	09:00 - 09:59	Late Morning	37,7	50	56,4	56,4	18,85	539,52	135,95
2019/05/20	CH2206	Male	Three y/o	Treatment (Wk 1)	Treatment	Feed Day	No	09:05	09:00 - 09:59	Late Morning	37,9	52	53,0	53,0	19,05	542,83	133,80
2019/05/20	CH2205	Male	Three y/o	Treatment (Wk 1)	Treatment	Feed Day	No	09:10	09:00 - 09:59	Late Morning	37,7	74	36,6	36,6	18,85	571,33	121,03
2019/05/20	CH2206	Male	Three y/o	Treatment (Wk 1)	Treatment	Feed Day	No	09:10	09:00 - 09:59	Late Morning	37,9	118	85,6	85,6	19,05	605,61	150,43
2019/05/20	CH2205	Male	Three y/o	Treatment (Wk 1)	Treatment	Feed Day	No	09:15	09:00 - 09:59	Late Morning	37,7	86	88,2	88,2	18,85	582,77	151,47
2019/05/20	CH2206	Male	Three y/o	Treatment (Wk 1)	Treatment	Feed Day	No	09:15	09:00 - 09:59	Late Morning	37,9	115	126,6	126,6	19,05	603,82	164,08
2019/05/20	CH2205	Male	Three y/o	Treatment (Wk 1)	Treatment	Feed Day	No	09:20	09:00 - 09:59	Late Morning	37,7	97	149,2	149,2	18,85	591,66	169,83
2019/05/20	CH2206	Male	Three y/o	Treatment (Wk 1)	Treatment	Feed Day	No	09:20	09:00 - 09:59	Late Morning	37,9		373,4	373,4	19,05		202,17
2019/05/20	CH2205	Male	Three y/o	Treatment (Wk 1)	Treatment	Feed Day	No	09:25	09:00 - 09:59	Late Morning	37,9	140	69,8	69,8	19,05	617,28	143,34
2019/05/20	CH2206	Male	Three y/o	Treatment (Wk 1)	Treatment	Feed Day	No	09:25	09:00 - 09:59	Late Morning	37,9		189,6	189,6	19,05		178,24
2019/05/20	CH2205	Male	Three y/o	Treatment (Wk 1)	Treatment	Feed Day	No	09:30	09:00 - 09:59	Late Morning	37,9	68	23,2	23,2	19,05	564,71	105,39
2019/05/20	CH2206	Male	Three y/o	Treatment (Wk 1)	Treatment	Feed Day	No	09:30	09:00 - 09:59	Late Morning	37,9	143	59,8	59,8	19,05	618,69	137,98
2019/05/20	CH2205	Male	Three y/o	Treatment (Wk 1)	Treatment	Feed Day	No	09:35	09:00 - 09:59	Late Morning	38,0	103	57,8	57,8	19,15	596,00	136,80
2019/05/20	CH2206	Male	Three y/o	Treatment (Wk 1)	Treatment	Feed Day	No	09:35	09:00 - 09:59	Late Morning	37,8	51	397,6	397,6	18,95	541,20	204,40
2019/05/20	CH2205	Male	Three y/o	Treatment (Wk 1)	Treatment	Feed Day	No	09:40	09:00 - 09:59	Late Morning	37,9		177,6	177,6	19,05		175,94
2019/05/20	CH2206	Male	Three y/o	Treatment (Wk 1)	Treatment	Feed Day	No	09:40	09:00 - 09:59	Late Morning	37,9	104	165,6	165,6	19,05	596,69	173,49
2019/05/20	CH2205	Male	Three y/o	Treatment (Wk 1)	Treatment	Feed Day	No	09:45	09:00 - 09:59	Late Morning	37,9	63	18,4	18,4	19,05	558,62	97,47
2019/05/20	CH2206	Male	Three y/o	Treatment (Wk 1)	Treatment	Feed Day	No	09:45	09:00 - 09:59	Late Morning	37,9	101	114,0	114,0	19,05	594,59	160,42
2019/05/20	CH2205	Male	Three y/o	Treatment (Wk 1)	Treatment	Feed Day	No	09:50	09:00 - 09:59	Late Morning	38,0	56	10,2	10,2	19,15	549,01	77,43
2019/05/20	CH2206	Male	Three y/o	Treatment (Wk 1)	Treatment	Feed Day	No	09:50	09:00 - 09:59	Late Morning	37,9	180	189,4	189,4	19,05	633,61	178,20
2019/05/20	CH2205	Male	Three y/o	Treatment (Wk 1)	Treatment	Feed Day	No	09:55	09:00 - 09:59	Late Morning	38,0	49	23,8	23,8	19,15	537,80	106,26
2019/05/20	CH2206	Male	Three y/o	Treatment (Wk 1)	Treatment	Feed Day	No	09:55	09:00 - 09:59	Late Morning	37,9	120	167,4	167,4	19,05	606,78	173,87
2019/05/20	CH2205	Male	Three y/o	Treatment (Wk 1)	Treatment	Feed Day	No	10:00	10:00 - 10:59	Late Morning	37,9		50,0	50,0	19,05		131,79
2019/05/20	CH2206	Male	Three y/o	Treatment (Wk 1)	Treatment	Feed Day	No	10:00	10:00 - 10:59	Late Morning	37,9	105	80,0	80,0	19,05	597,38	148,08
2019/05/20	CH2205	Male	Three y/o	Treatment (Wk 1)	Treatment	Feed Day	No	10:05	10:00 - 10:59	Late Morning	37,9	81	131,4	131,4	19,05	578,26	165,38
2019/05/20	CH2206	Male	Three y/o	Treatment (Wk 1)	Treatment	Feed Day	No	10:05	10:00 - 10:59	Late Morning	37,9	99	155,4	155,4	19,05	593,14	171,26
2019/05/20	CH2205	Male	Three y/o	Treatment (Wk 1)	Treatment	Feed Day	No	10:10	10:00 - 10:59	Late Morning	37,9	70	10,4	10,4	19,05	566,99	78,08
2019/05/20	CH2206	Male	Three y/o	Treatment (Wk 1)	Treatment	Feed Day	No	10:10	10:00 - 10:59	Late Morning	38,0	140	225,0	225,0	19,15	617,28	184,27
2019/05/20	CH2205	Male	Three y/o	Treatment (Wk 1)	Treatment	Feed Day	No	10:15	10:00 - 10:59	Late Morning	37,9		33,4	33,4	19,05		117,88
2019/05/20	CH2206	Male	Three y/o	Treatment (Wk 1)	Treatment	Feed Day	No	10:15	10:00 - 10:59	Late Morning	38,0	125	289,4	289,4	19,15	609,60	193,15
2019/05/20	CH2205	Male	Three y/o	Treatment (Wk 1)	Treatment	Feed Day	No	10:20	10:00 - 10:59	Late Morning	37,9		332,2	332,2	19,05		198,03
2019/05/20	CH2206	Male	Three y/o	Treatment (Wk 1)	Treatment	Feed Day	No	10:20	10:00 - 10:59	Late Morning	38,0	102	111,0	111,0	19,15	595,30	159,48
2019/05/20	CH2205	Male	Three y/o	Treatment (Wk 1)	Treatment	Feed Day	No	10:25	10:00 - 10:59	Late Morning	37,9		20,6	20,6	19,05		101,32
2019/05/20	CH2206	Male	Three y/o	Treatment (Wk 1)	Treatment	Feed Day	No	10:25	10:00 - 10:59	Late Morning	38,0	101	69,0	69,0	19,15	594,59	142,94
2019/05/20	CH2205	Male	Three y/o	Treatment (Wk 1)	Treatment	Feed Day	Yes	10:30	10:00 - 10:59	Late Morning	37,7		31,8	31,8	18,85		116,20
2019/05/20	CH2206	Male	Three y/o	Treatment (Wk 1)	Treatment	Feed Day	Yes	10:30	10:00 - 10:59	Late Morning	37,9	76	59,2	59,2	19,05	573,39	137,63
2019/05/20	CH2205	Male	Three y/o	Treatment (Wk 1)	Treatment	Feed Day	Yes	10:35	10:00 - 10:59	Late Morning	37,7	67	31,0	31,0	18,85	563,53	115,32
2019/05/20	CH2206	Male	Three y/o	Treatment (Wk 1)	Treatment	Feed Day	Yes	10:35	10:00 - 10:59	Late Morning	38,0	62	132,6	132,6	19,15	557,33	165,70
2019/05/20	CH2205	Male	Three y/o	Treatment (Wk 1)	Treatment	Feed Day	Yes	10:40	10:00 - 10:59	Late Morning	37,9		109,6	109,6	19,05		159,04
2019/05/20	CH2206	Male	Three y/o	Treatment (Wk 1)	Treatment	Feed Day	Yes	10:40	10:00 - 10:59	Late Morning	38,0		116,2	116,2	19,15		161,08
2019/05/20	CH2205	Male	Three y/o	Treatment (Wk 1)	Treatment	Feed Day	Yes	10:45	10:00 - 10:59	Late Morning	38,0		120,6	120,6	19,15		162,38
2019/05/20	CH2206	Male	Three y/o	Treatment (Wk 1)	Treatment	Feed Day	Yes	10:45	10:00 - 10:59	Late Morning	38,0	125	38,2	38,2	19,15	609,60	122,50
2019/05/20	CH2205	Male	Three y/o	Treatment (Wk 1)	Treatment	Feed Day	Yes	10:50	10:00 - 10:59	Late Morning	38,1	123	291,2	291,2	19,26	608,49	193,37
2019/05/20	CH2206	Male	Three y/o	Treatment (Wk 1)	Treatment	Feed Day	Yes	10:50	10:00 - 10:59	Late Morning	37,9	86	45,8	45,8	19,05	582,77	128,76
2019/05/20	CH2205	Male	Three y/o	Treatment (Wk 1)	Treatment	Feed Day	Yes	10:55	10:00 - 10:59	Late Morning	38,1	137	65,4	65,4	19,26	615,83	141,08
2019/05/20	CH2206	Male	Three y/o	Treatment (Wk 1)	Treatment	Feed Day	Yes	10:55	10:00 - 10:59	Late Morning	38,0	96	79,8	79,8	19,15	590,91	147,99
2019/05/20	CH2205	Male	Three y/o	Treatment (Wk 1)	Treatment	Feed Day	Yes	11:00	11:00 - 11:59	Late Morning	38,2	113	25,2	25,2	19,36	602,58	108,22
2019/05/20	CH2206	Male	Three y/o	Treatment (Wk 1)	Treatment	Feed Day	Yes	11:00	11:00 - 11:59	Late Morning	38,0	101	396,0	396,0	19,15	594,59	204,26
2019/05/20	CH2205	Male	Three y/o	Treatment (Wk 1)	Treatment	Feed Day	Yes	11:05	11:00 - 11:59	Late Morning	38,2	105	18,0	18,0	19,36	597,38	96,72

2019/05/20	CH2206	Male	Three y/o	Treatment (Wk 1)	Treatment	Feed Day	Yes	11:05	11:00 - 11:59	Late Morning	38,0	106	55,0	55,0	19,15	598,06	135,08
2019/05/20	CH2205	Male	Three y/o	Treatment (Wk 1)	Treatment	Feed Day	Yes	11:10	11:00 - 11:59	Late Morning	38,2	112	97,6	97,6	19,36	601,96	155,00
2019/05/20	CH2206	Male	Three y/o	Treatment (Wk 1)	Treatment	Feed Day	Yes	11:10	11:00 - 11:59	Late Morning	38,0	108	365,8	365,8	19,15	599,39	201,44
2019/05/20	CH2205	Male	Three y/o	Treatment (Wk 1)	Treatment	Feed Day	Yes	11:15	11:00 - 11:59	Late Morning	38,2		64,4	64,4	19,36		140,55
2019/05/20	CH2206	Male	Three y/o	Treatment (Wk 1)	Treatment	Feed Day	Yes	11:15	11:00 - 11:59	Late Morning	38,1	115	162,8	162,8	19,26	603,82	172,89
2019/05/20	CH2205	Male	Three y/o	Treatment (Wk 1)	Treatment	Feed Day	Yes	11:20	11:00 - 11:59	Late Morning	38,3	120	36,8	36,8	19,46	606,78	121,22
2019/05/20	CH2206	Male	Three y/o	Treatment (Wk 1)	Treatment	Feed Day	Yes	11:20	11:00 - 11:59	Late Morning	38,1		67,2	67,2	19,26		142,02
2019/05/20	CH2205	Male	Three y/o	Treatment (Wk 1)	Treatment	Feed Day	Yes	11:25	11:00 - 11:59	Late Morning	38,1		30,0	30,0	19,26		114,20
2019/05/20	CH2206	Male	Three y/o	Treatment (Wk 1)	Treatment	Feed Day	Yes	11:25	11:00 - 11:59	Late Morning	37,8		88,8	88,8	18,95		151,71
2019/05/20	CH2205	Male	Three y/o	Treatment (Wk 1)	Treatment	Feed Day	No	11:30	11:00 - 11:59	Late Morning	37,9	110	628,4	628,4	19,05	600,69	220,70
2019/05/20	CH2206	Male	Three y/o	Treatment (Wk 1)	Treatment	Feed Day	No	11:30	11:00 - 11:59	Late Morning	37,8	85	54,0	54,0	18,95	581,90	134,45
2019/05/20	CH2205	Male	Three y/o	Treatment (Wk 1)	Treatment	Feed Day	No	11:35	11:00 - 11:59	Late Morning	38,0	117	38,6	38,6	19,15	605,02	122,86
2019/05/20	CH2206	Male	Three y/o	Treatment (Wk 1)	Treatment	Feed Day	No	11:35	11:00 - 11:59	Late Morning	37,8	132	121,8	121,8	18,95	613,32	162,73
2019/05/20	CH2205	Male	Three y/o	Treatment (Wk 1)	Treatment	Feed Day	No	11:40	11:00 - 11:59	Late Morning	38,0	107	38,2	38,2	19,15	598,73	122,50
2019/05/20	CH2206	Male	Three y/o	Treatment (Wk 1)	Treatment	Feed Day	No	11:40	11:00 - 11:59	Late Morning	37,9	103	29,0	29,0	19,05	596,00	113,03
2019/05/20	CH2205	Male	Three y/o	Treatment (Wk 1)	Treatment	Feed Day	No	11:45	11:00 - 11:59	Late Morning	37,9		87,2	87,2	19,05		151,07
2019/05/20	CH2206	Male	Three y/o	Treatment (Wk 1)	Treatment	Feed Day	No	11:45	11:00 - 11:59	Late Morning	37,8		310,8	310,8	18,95		195,67
2019/05/20	CH2205	Male	Three y/o	Treatment (Wk 1)	Treatment	Feed Day	No	11:50	11:00 - 11:59	Late Morning	38,0		142,6	142,6	19,15		168,24
2019/05/20	CH2206	Male	Three y/o	Treatment (Wk 1)	Treatment	Feed Day	No	11:50	11:00 - 11:59	Late Morning	37,9	134	171,4	171,4	19,05	614,33	174,70
2019/05/20	CH2205	Male	Three y/o	Treatment (Wk 1)	Treatment	Feed Day	No	11:55	11:00 - 11:59	Late Morning	38,2	114	43,8	43,8	19,36	603,20	127,22
2019/05/20	CH2206	Male	Three y/o	Treatment (Wk 1)	Treatment	Feed Day	No	11:55	11:00 - 11:59	Late Morning	38,0	113	186,4	186,4	19,15	602,58	177,64
2019/05/20	CH2205	Male	Three y/o	Treatment (Wk 1)	Treatment	Feed Day	No	12:00	12:00 - 12:59	Afternoon	38,2	93	147,4	147,4	19,36	588,58	169,40
2019/05/20	CH2206	Male	Three y/o	Treatment (Wk 1)	Treatment	Feed Day	No	12:00	12:00 - 12:59	Afternoon	38,1	114	40,8	40,8	19,26	603,20	124,77
2019/05/20	CH2205	Male	Three y/o	Treatment (Wk 1)	Treatment	Feed Day	No	12:05	12:00 - 12:59	Afternoon	38,3		46,6	46,6	19,46		129,35
2019/05/20	CH2206	Male	Three y/o	Treatment (Wk 1)	Treatment	Feed Day	No	12:05	12:00 - 12:59	Afternoon	38,1	128	112,6	112,6	19,26	611,22	159,98
2019/05/20	CH2205	Male	Three y/o	Treatment (Wk 1)	Treatment	Feed Day	No	12:10	12:00 - 12:59	Afternoon	38,3		26,4	26,4	19,46		109,81
2019/05/20	CH2206	Male	Three y/o	Treatment (Wk 1)	Treatment	Feed Day	No	12:10	12:00 - 12:59	Afternoon	38,1	125	81,0	81,0	19,26	609,60	148,51
2019/05/20	CH2205	Male	Three y/o	Treatment (Wk 1)	Treatment	Feed Day	No	12:15	12:00 - 12:59	Afternoon	38,2	119	97,2	97,2	19,36	606,20	154,85
2019/05/20	CH2206	Male	Three y/o	Treatment (Wk 1)	Treatment	Feed Day	No	12:15	12:00 - 12:59	Afternoon	38,1	123	79,4	79,4	19,26	608,49	147,81
2019/05/20	CH2205	Male	Three y/o	Treatment (Wk 1)	Treatment	Feed Day	No	12:20	12:00 - 12:59	Afternoon	38,3	107	163,4	163,4	19,46	598,73	173,02
2019/05/20	CH2206	Male	Three y/o	Treatment (Wk 1)	Treatment	Feed Day	No	12:20	12:00 - 12:59	Afternoon	38,1	100	70,4	70,4	19,26	593,87	143,64
2019/05/20	CH2205	Male	Three y/o	Treatment (Wk 1)	Treatment	Feed Day	No	12:25	12:00 - 12:59	Afternoon	38,5		60,2	60,2	19,66		138,21
2019/05/20	CH2206	Male	Three y/o	Treatment (Wk 1)	Treatment	Feed Day	No	12:25	12:00 - 12:59	Afternoon	38,3		139,8	139,8	19,46		167,55
2019/05/20	CH2205	Male	Three y/o	Treatment (Wk 1)	Treatment	Feed Day	No	12:30	12:00 - 12:59	Afternoon	38,4	92	27,4	27,4	19,56	587,78	111,09
2019/05/20	CH2206	Male	Three y/o	Treatment (Wk 1)	Treatment	Feed Day	No	12:30	12:00 - 12:59	Afternoon	38,4	140	116,4	116,4	19,56	617,28	161,14
2019/05/20	CH2205	Male	Three y/o	Treatment (Wk 1)	Treatment	Feed Day	No	12:35	12:00 - 12:59	Afternoon	38,5		23,8	23,8	19,66		106,26
2019/05/20	CH2206	Male	Three y/o	Treatment (Wk 1)	Treatment	Feed Day	No	12:35	12:00 - 12:59	Afternoon	38,4	143	144,6	144,6	19,56	618,69	168,73
2019/05/20	CH2205	Male	Three y/o	Treatment (Wk 1)	Treatment	Feed Day	No	12:40	12:00 - 12:59	Afternoon	38,5	98	149,4	149,4	19,66	592,41	169,88
2019/05/20	CH2206	Male	Three y/o	Treatment (Wk 1)	Treatment	Feed Day	No	12:40	12:00 - 12:59	Afternoon	38,6	64	200,6	200,6	19,76	559,88	180,22
2019/05/20	CH2205	Male	Three y/o	Treatment (Wk 1)	Treatment	Feed Day	No	12:45	12:00 - 12:59	Afternoon	38,4	112	79,8	79,8	19,56	601,96	147,99
2019/05/20	CH2206	Male	Three y/o	Treatment (Wk 1)	Treatment	Feed Day	No	12:45	12:00 - 12:59	Afternoon	38,6	126	654,6	654,6	19,76	610,14	222,15
2019/05/20	CH2205	Male	Three y/o	Treatment (Wk 1)	Treatment	Feed Day	No	12:50	12:00 - 12:59	Afternoon	38,3	138	2,6	2,6	19,46	616,31	31,57
2019/05/20	CH2206	Male	Three y/o	Treatment (Wk 1)	Treatment	Feed Day	No	12:50	12:00 - 12:59	Afternoon	38,5	129	51,8	51,8	19,66	611,75	133,01
2019/05/20	CH2205	Male	Three y/o	Treatment (Wk 1)	Treatment	Feed Day	No	12:55	12:00 - 12:59	Afternoon	38,1		130,6	130,6	19,26		165,17
2019/05/20	CH2206	Male	Three y/o	Treatment (Wk 1)	Treatment	Feed Day	No	12:55	12:00 - 12:59	Afternoon	38,4	108	45,8	45,8	19,56	599,39	128,76
2019/05/20	CH2205	Male	Three y/o	Treatment (Wk 1)	Treatment	Feed Day	No	13:00	13:00 - 13:59	Afternoon	38,1	113	51,6	51,6	19,26	602,58	132,87
2019/05/20	CH2206	Male	Three y/o	Treatment (Wk 1)	Treatment	Feed Day	No	13:00	13:00 - 13:59	Afternoon	38,3	88	49,8	49,8	19,46	584,49	131,65
2019/05/20	CH2205	Male	Three y/o	Treatment (Wk 1)	Treatment	Feed Day	No	13:05	13:00 - 13:59	Afternoon	38,1		20,2	20,2	19,26		100,65
2019/05/20	CH2206	Male	Three y/o	Treatment (Wk 1)	Treatment	Feed Day	No	13:05	13:00 - 13:59	Afternoon	38,2	81	50,2	50,2	19,36	578,26	131,92
2019/05/20	CH2205	Male	Three y/o	Treatment (Wk 1)	Treatment	Feed Day	No	13:10	13:00 - 13:59	Afternoon	37,9	76	20,8	20,8	19,05	573,39	101,65
2019/05/20	CH2206	Male	Three y/o	Treatment (Wk 1)	Treatment	Feed Day	No	13:10	13:00 - 13:59	Afternoon	38,2	94	52,0	52,0	19,36	589,37	133,14
2019/05/20	CH2205	Male	Three y/o	Treatment (Wk 1)	Treatment	Feed Day	No	13:15	13:00 - 13:59	Afternoon	37,7	77	34,4	34,4	18,85	574,39	118,90
2019/05/20	CH2206	Male	Three y/o	Treatment (Wk 1)	Treatment	Feed Day	No	13:15	13:00 - 13:59	Afternoon	38,1	90	50,6	50,6	19,26	586,16	132,20
2019/05/20	CH2205	Male	Three y/o	Treatment (Wk 1)	Treatment	Feed Day	No	13:20	13:00 - 13:59	Afternoon	37,7	68	15,4	15,4	18,85	564,71	91,41
2019/05/20	CH2206	Male	Three y/o	Treatment (Wk 1)	Treatment	Feed Day	No	13:20	13:00 - 13:59	Afternoon	38,1	67	48,0	48,0	19,26	563,53	130,38
2019/05/20	CH2205	Male	Three y/o	Treatment (Wk 1)	Treatment	Feed Day	No	13:25	13:00 - 13:59	Afternoon	37,7	88	15,2	15,2	18,85	584,49	90,96
2019/05/20	CH2206	Male	Three y/o	Treatment (Wk 1)	Treatment	Feed Day	No	13:25	13:00 - 13:59	Afternoon	38,0	120	63,4	63,4	19,15	606,78	140,00
2019/05/20	CH2205	Male	Three y/o	Treatment (Wk 1)	Treatment	Feed Day	No	13:30	13:00 - 13:59	Afternoon	37,8	70	14,2	14,2	18,95	566,99	88,65
2019/05/20	CH2206	Male	Three y/o	Treatment (Wk 1)	Treatment	Feed Day	No	13:30	13:00 - 13:59	Afternoon	38,0	86	45,8	45,8	19,15	582,77	128,76
2019/05/20	CH2205	Male	Three y/o	Treatment (Wk 1)	Treatment	Feed Day	No	13:35	13:00 - 13:59	Afternoon	37,7	69	13,0	13,0	18,85	565,86	85,65
2019/05/20	CH2206	Male	Three y/o	Treatment (Wk 1)	Treatment	Feed Day	No	13:35	13:00 - 13:59	Afternoon	38,0	106	54,8	54,8	19,15	598,06	134,96
2019/05/20	CH2205	Male	Three y/o	Treatment (Wk 1)	Treatment	Feed Day	No	13:40	13:00 - 13:59	Afternoon	37,7	72	12,2	12,2	18,85	569,20	83,49
2019/05/20	CH2206	Male	Three y/o	Treatment (Wk 1)	Treatment	Feed Day	No	13:40	13:00 - 13:59	Afternoon	38,0	95	38,6	38,6	19,15	590,14	122,86
2019/05/20	CH2205	Male	Three y/o	Treatment (Wk 1)	Treatment	Feed Day	No	13:45	13:00 - 13:59	Afternoon	37,7	76	27,8	27,8	18,85	573,39	111,58
2019/05/20	CH2206	Male	Three y/o	Treatment (Wk 1)	Treatment	Feed Day	No	13:45	13:00 - 13:59	Afternoon	38,1		178,8	178,8	19,26		176,18
2019/05/20	CH2205	Male	Three y/o	Treatment (Wk 1)	Treatment	Feed Day	No	13:50	13:00 - 13:59	Afternoon	37,6	88	28,6	28,6	18,75	584,49	112,56
2019/05/20	CH2206	Male	Three y/o	Treatment (Wk 1)	Treatment	Feed Day	No	13:50	13:00 - 13:59	Afternoon	38,2	129	217,6	217,6	19,36	611,75	183,09
2019/05/20	CH2205	Male	Three y/o	Treatment (Wk 1)	Treatment	Feed Day	No	13:55	13:00 - 13:59	Afternoon	37,6		35,2	35,2	18,75		119,69
2019/05/20	CH2206	Male	Three y/o	Treatment (Wk 1)	Treatment	Feed Day	No	13:55	13:00 - 13:59	Afternoon	38,2		453,4	453,4	19,36		209,07
2019/05/20	CH2205	Male	Three y/o	Treatment (Wk 1)	Treatment	Feed Day	No	14:00	14:00 - 14:59	Afternoon	37,8	174	85,0	85,0	18,95	631,46	150,18

2019/05/20	CH2206	Male	Three y/o	Treatment (Wk 1)	Treatment	Feed Day	No	14:00	14:00 - 14:59	Afternoon	38,3	129	336,0	336,0	19,46	611,75	198,43
2019/05/20	CH2205	Male	Three y/o	Treatment (Wk 1)	Treatment	Feed Day	No	14:05	14:00 - 14:59	Afternoon	37,9	120	21,2	21,2	19,05	606,78	102,31
2019/05/20	CH2206	Male	Three y/o	Treatment (Wk 1)	Treatment	Feed Day	No	14:05	14:00 - 14:59	Afternoon	38,3	98	44,4	44,4	19,46	592,41	127,69
2019/05/20	CH2205	Male	Three y/o	Treatment (Wk 1)	Treatment	Feed Day	No	14:10	14:00 - 14:59	Afternoon	37,9		29,8	29,8	19,05		113,97
2019/05/20	CH2206	Male	Three y/o	Treatment (Wk 1)	Treatment	Feed Day	No	14:10	14:00 - 14:59	Afternoon	38,1	48	48,0	48,0	19,26	536,04	130,38
2019/05/20	CH2205	Male	Three y/o	Treatment (Wk 1)	Treatment	Feed Day	No	14:15	14:00 - 14:59	Afternoon	37,9	97	21,0	21,0	19,05	591,66	101,98
2019/05/20	CH2206	Male	Three y/o	Treatment (Wk 1)	Treatment	Feed Day	No	14:15	14:00 - 14:59	Afternoon	38,0	77	52,2	52,2	19,15	574,39	133,27
2019/05/20	CH2205	Male	Three y/o	Treatment (Wk 1)	Treatment	Feed Day	No	14:20	14:00 - 14:59	Afternoon	37,9	83	20,0	20,0	19,05	580,11	100,31
2019/05/20	CH2206	Male	Three y/o	Treatment (Wk 1)	Treatment	Feed Day	No	14:20	14:00 - 14:59	Afternoon	38,0	81	48,2	48,2	19,15	578,26	130,52
2019/05/20	CH2205	Male	Three y/o	Treatment (Wk 1)	Treatment	Feed Day	No	14:25	14:00 - 14:59	Afternoon	37,9		29,6	29,6	19,05		113,73
2019/05/20	CH2206	Male	Three y/o	Treatment (Wk 1)	Treatment	Feed Day	No	14:25	14:00 - 14:59	Afternoon	37,9	84	56,4	56,4	19,05	581,01	135,95
2019/05/20	CH2205	Male	Three y/o	Treatment (Wk 1)	Treatment	Feed Day	No	14:30	14:00 - 14:59	Afternoon	37,9	180	75,6	75,6	19,05	633,61	146,11
2019/05/20	CH2206	Male	Three y/o	Treatment (Wk 1)	Treatment	Feed Day	No	14:30	14:00 - 14:59	Afternoon	38,0		144,6	144,6	19,15		168,73
2019/05/20	CH2205	Male	Three y/o	Treatment (Wk 1)	Treatment	Feed Day	No	14:35	14:00 - 14:59	Afternoon	37,9		35,8	35,8	19,05		120,27
2019/05/20	CH2206	Male	Three y/o	Treatment (Wk 1)	Treatment	Feed Day	No	14:35	14:00 - 14:59	Afternoon	38,1	132	42,2	42,2	19,26	613,32	125,93
2019/05/20	CH2205	Male	Three y/o	Treatment (Wk 1)	Treatment	Feed Day	No	14:40	14:00 - 14:59	Afternoon	37,6	60	31,8	31,8	18,75	554,67	116,20
2019/05/20	CH2206	Male	Three y/o	Treatment (Wk 1)	Treatment	Feed Day	No	14:40	14:00 - 14:59	Afternoon	38,0	188	30,2	30,2	19,15	636,34	114,42
2019/05/20	CH2205	Male	Three y/o	Treatment (Wk 1)	Treatment	Feed Day	No	14:45	14:00 - 14:59	Afternoon	37,7	83	20,0	20,0	18,85	580,11	100,31
2019/05/20	CH2206	Male	Three y/o	Treatment (Wk 1)	Treatment	Feed Day	No	14:45	14:00 - 14:59	Afternoon	37,9	72	25,4	25,4	19,05	569,20	108,49
2019/05/20	CH2205	Male	Three y/o	Treatment (Wk 1)	Treatment	Feed Day	No	14:50	14:00 - 14:59	Afternoon	37,9	89	8,8	8,8	19,05	585,33	72,43
2019/05/20	CH2206	Male	Three y/o	Treatment (Wk 1)	Treatment	Feed Day	No	14:50	14:00 - 14:59	Afternoon	37,9	90	28,2	28,2	19,05	586,16	112,07
2019/05/20	CH2205	Male	Three y/o	Treatment (Wk 1)	Treatment	Feed Day	No	14:55	14:00 - 14:59	Afternoon	37,9	63	15,0	15,0	19,05	558,62	90,51
2019/05/20	CH2206	Male	Three y/o	Treatment (Wk 1)	Treatment	Feed Day	No	14:55	14:00 - 14:59	Afternoon	37,9	87	19,0	19,0	19,05	583,64	98,56
2019/05/20	CH2205	Male	Three y/o	Treatment (Wk 1)	Treatment	Feed Day	No	15:00	15:00 - 15:59	Afternoon	38,0	65	15,2	15,2	19,15	561,12	90,96
2019/05/20	CH2206	Male	Three y/o	Treatment (Wk 1)	Treatment	Feed Day	No	15:00	15:00 - 15:59	Afternoon	37,9	86	27,8	27,8	19,05	582,77	111,58
2019/05/20	CH2205	Male	Three y/o	Treatment (Wk 1)	Treatment	Feed Day	No	15:05	15:00 - 15:59	Afternoon	38,0	92	38,6	38,6	19,15	587,78	122,86
2019/05/20	CH2206	Male	Three y/o	Treatment (Wk 1)	Treatment	Feed Day	No	15:05	15:00 - 15:59	Afternoon	37,9	102	34,4	34,4	19,05	595,30	118,90
2019/05/20	CH2205	Male	Three y/o	Treatment (Wk 1)	Treatment	Feed Day	No	15:10	15:00 - 15:59	Afternoon	38,0		30,6	30,6	19,15		114,87
2019/05/20	CH2206	Male	Three y/o	Treatment (Wk 1)	Treatment	Feed Day	No	15:10	15:00 - 15:59	Afternoon	37,9	98	33,0	33,0	19,05	592,41	117,47
2019/05/20	CH2205	Male	Three y/o	Treatment (Wk 1)	Treatment	Feed Day	No	15:15	15:00 - 15:59	Afternoon	38,0		32,4	32,4	19,15		116,84
2019/05/20	CH2206	Male	Three y/o	Treatment (Wk 1)	Treatment	Feed Day	No	15:15	15:00 - 15:59	Afternoon	38,0	90	34,4	34,4	19,15	586,16	118,90
2019/05/20	CH2205	Male	Three y/o	Treatment (Wk 1)	Treatment	Feed Day	No	15:20	15:00 - 15:59	Afternoon	38,1	108	49,0	49,0	19,26	599,39	131,09
2019/05/20	CH2206	Male	Three y/o	Treatment (Wk 1)	Treatment	Feed Day	No	15:20	15:00 - 15:59	Afternoon	38,1	131	36,6	36,6	19,26	612,80	121,03
2019/05/20	CH2205	Male	Three y/o	Treatment (Wk 1)	Treatment	Feed Day	No	15:25	15:00 - 15:59	Afternoon	38,0		33,4	33,4	19,15		117,88
2019/05/20	CH2206	Male	Three y/o	Treatment (Wk 1)	Treatment	Feed Day	No	15:25	15:00 - 15:59	Afternoon	38,1	81	91,6	91,6	19,26	578,26	152,79
2019/05/20	CH2205	Male	Three y/o	Treatment (Wk 1)	Treatment	Feed Day	No	15:30	15:00 - 15:59	Afternoon	37,9		104,8	104,8	19,05		157,48
2019/05/20	CH2206	Male	Three y/o	Treatment (Wk 1)	Treatment	Feed Day	No	15:30	15:00 - 15:59	Afternoon	38,2		525,4	525,4	19,36		214,31
2019/05/20	CH2205	Male	Three y/o	Treatment (Wk 1)	Treatment	Feed Day	No	15:35	15:00 - 15:59	Afternoon	38,1	141	34,8	34,8	19,26	617,75	119,29
2019/05/20	CH2206	Male	Three y/o	Treatment (Wk 1)	Treatment	Feed Day	No	15:35	15:00 - 15:59	Afternoon	38,3		73,8	73,8	19,46		145,27
2019/05/20	CH2205	Male	Three y/o	Treatment (Wk 1)	Treatment	Feed Day	No	15:40	15:00 - 15:59	Afternoon	38,0		43,2	43,2	19,15		126,74
2019/05/20	CH2206	Male	Three y/o	Treatment (Wk 1)	Treatment	Feed Day	No	15:40	15:00 - 15:59	Afternoon	38,3	47	24,0	24,0	19,46	534,23	106,55
2019/05/20	CH2205	Male	Three y/o	Treatment (Wk 1)	Treatment	Feed Day	No	15:45	15:00 - 15:59	Afternoon	37,7	94	19,4	19,4	18,85	589,37	99,28
2019/05/20	CH2206	Male	Three y/o	Treatment (Wk 1)	Treatment	Feed Day	No	15:45	15:00 - 15:59	Afternoon	38,2	114	35,0	35,0	19,36	603,20	119,49
2019/05/20	CH2205	Male	Three y/o	Treatment (Wk 1)	Treatment	Feed Day	No	15:50	15:00 - 15:59	Afternoon	37,7	102	39,4	39,4	18,85	595,30	123,57
2019/05/20	CH2206	Male	Three y/o	Treatment (Wk 1)	Treatment	Feed Day	No	15:50	15:00 - 15:59	Afternoon	38,0	91	61,0	61,0	19,15	586,98	138,67
2019/05/20	CH2205	Male	Three y/o	Treatment (Wk 1)	Treatment	Feed Day	No	15:55	15:00 - 15:59	Afternoon	37,9	103	67,2	67,2	19,05	596,00	142,02
2019/05/20	CH2206	Male	Three y/o	Treatment (Wk 1)	Treatment	Feed Day	No	15:55	15:00 - 15:59	Afternoon	38,2		48,8	48,8	19,36		130,95
2019/05/20	CH2205	Male	Three y/o	Treatment (Wk 1)	Treatment	Feed Day	No	16:00	16:00 - 16:59	Evening	38,0	103	19,4	19,4	19,15	596,00	99,28
2019/05/20	CH2206	Male	Three y/o	Treatment (Wk 1)	Treatment	Feed Day	No	16:00	16:00 - 16:59	Evening	38,2	162	170,2	170,2	19,36	626,88	174,45
2019/05/20	CH2205	Male	Three y/o	Treatment (Wk 1)	Treatment	Feed Day	No	16:05	16:00 - 16:59	Evening	38,1	100	151,2	151,2	19,26	593,87	170,30
2019/05/20	CH2206	Male	Three y/o	Treatment (Wk 1)	Treatment	Feed Day	No	16:05	16:00 - 16:59	Evening	38,2	112	94,8	94,8	19,36	601,96	153,98
2019/05/20	CH2205	Male	Three y/o	Treatment (Wk 1)	Treatment	Feed Day	No	16:10	16:00 - 16:59	Evening	38,2		30,4	30,4	19,36		114,65
2019/05/20	CH2206	Male	Three y/o	Treatment (Wk 1)	Treatment	Feed Day	No	16:10	16:00 - 16:59	Evening	38,2	45	141,8	141,8	19,36	530,47	168,05
2019/05/20	CH2205	Male	Three y/o	Treatment (Wk 1)	Treatment	Feed Day	No	16:15	16:00 - 16:59	Evening	38,2	86	18,2	18,2	19,36	582,77	97,10
2019/05/20	CH2206	Male	Three y/o	Treatment (Wk 1)	Treatment	Feed Day	No	16:15	16:00 - 16:59	Evening	38,3	114	15,8	15,8	19,46	603,20	92,28
2019/05/20	CH2205	Male	Three y/o	Treatment (Wk 1)	Treatment	Feed Day	No	16:20	16:00 - 16:59	Evening	38,2	129	23,2	23,2	19,36	611,75	105,39
2019/05/20	CH2206	Male	Three y/o	Treatment (Wk 1)	Treatment	Feed Day	No	16:20	16:00 - 16:59	Evening	38,2	75	199,0	199,0	19,36	572,36	179,94
2019/05/20	CH2205	Male	Three y/o	Treatment (Wk 1)	Treatment	Feed Day	No	16:25	16:00 - 16:59	Evening	38,2		30,2	30,2	19,36		114,42
2019/05/20	CH2206	Male	Three y/o	Treatment (Wk 1)	Treatment	Feed Day	No	16:25	16:00 - 16:59	Evening	38,2	117	248,0	248,0	19,36	605,02	187,70
2019/05/20	CH2205	Male	Three y/o	Treatment (Wk 1)	Treatment	Feed Day	No	16:30	16:00 - 16:59	Evening	38,1	123	46,8	46,8	19,26	608,49	129,50
2019/05/20	CH2206	Male	Three y/o	Treatment (Wk 1)	Treatment	Feed Day	No	16:30	16:00 - 16:59	Evening	38,3	114	130,2	130,2	19,46	603,20	165,06
2019/05/20	CH2205	Male	Three y/o	Treatment (Wk 1)	Treatment	Feed Day	No	16:35	16:00 - 16:59	Evening	38,2	138	179,2	179,2	19,36	616,31	176,26
2019/05/20	CH2206	Male	Three y/o	Treatment (Wk 1)	Treatment	Feed Day	No	16:35	16:00 - 16:59	Evening	38,3	59	44,4	44,4	19,46	553,30	127,69
2019/05/20	CH2205	Male	Three y/o	Treatment (Wk 1)	Treatment	Feed Day	No	16:40	16:00 - 16:59	Evening	38,1	129	53,8	53,8	19,26	611,75	134,32
2019/05/20	CH2206	Male	Three y/o	Treatment (Wk 1)	Treatment	Feed Day	No	16:40	16:00 - 16:59	Evening	38,2	91	83,8	83,8	19,36	586,98	149,69
2019/05/20	CH2205	Male	Three y/o	Treatment (Wk 1)	Treatment	Feed Day	No	16:45	16:00 - 16:59	Evening	38,1	101	35,8	35,8	19,26	594,59	120,27
2019/05/20	CH2206	Male	Three y/o	Treatment (Wk 1)	Treatment	Feed Day	No	16:45	16:00 - 16:59	Evening	38,2	118	200,8	200,8	19,36	605,61	180,26
2019/05/20	CH2205	Male	Three y/o	Treatment (Wk 1)	Treatment	Feed Day	No	16:50	16:00 - 16:59	Evening	38,2		61,0	61,0	19,36		138,67
2019/05/20	CH2206	Male	Three y/o	Treatment (Wk 1)	Treatment	Feed Day	No	16:50	16:00 - 16:59	Evening	38,2	107	25,6	25,6	19,36	598,73	108,76
2019/05/20	CH2205	Male	Three y/o	Treatment (Wk 1)	Treatment	Feed Day	No	16:55	16:00 - 16:59	Evening	38,0	88	33,4	33,4	19,15	584,49	117,88

2019/05/20	CH2206	Male	Three y/o	Treatment (Wk 1)	Treatment	Feed Day	No	16:55	16:00 - 16:59	Evening	38,2	88	43,2	43,2	19,36	584,49	126,74
2019/05/20	CH2205	Male	Three y/o	Treatment (Wk 1)	Treatment	Feed Day	No	17:00	17:00 - 17:59	Evening	37,9	115	34,2	34,2	19,05	603,82	118,70
2019/05/20	CH2206	Male	Three y/o	Treatment (Wk 1)	Treatment	Feed Day	No	17:00	17:00 - 17:59	Evening	38,0	87	41,2	41,2	19,15	583,64	125,11
2019/05/20	CH2205	Male	Three y/o	Treatment (Wk 1)	Treatment	Feed Day	No	17:05	17:00 - 17:59	Evening	38,0		30,0	30,0	19,15		114,20
2019/05/20	CH2206	Male	Three y/o	Treatment (Wk 1)	Treatment	Feed Day	No	17:05	17:00 - 17:59	Evening	37,9	46	43,8	43,8	19,05	532,38	127,22
2019/05/20	CH2205	Male	Three y/o	Treatment (Wk 1)	Treatment	Feed Day	No	17:10	17:00 - 17:59	Evening	37,6	107	33,0	33,0	18,75	598,73	117,47
2019/05/20	CH2206	Male	Three y/o	Treatment (Wk 1)	Treatment	Feed Day	No	17:10	17:00 - 17:59	Evening	37,8	56	42,8	42,8	18,95	549,01	126,42
2019/05/20	CH2205	Male	Three y/o	Treatment (Wk 1)	Treatment	Feed Day	No	17:15	17:00 - 17:59	Evening	37,4		33,0	33,0	18,55		117,47
2019/05/20	CH2206	Male	Three y/o	Treatment (Wk 1)	Treatment	Feed Day	No	17:15	17:00 - 17:59	Evening	37,7	80	50,8	50,8	18,85	577,31	132,33
2019/05/20	CH2205	Male	Three y/o	Treatment (Wk 1)	Treatment	Feed Day	No	17:20	17:00 - 17:59	Evening	37,3	143	64,6	64,6	18,46	618,69	140,65
2019/05/20	CH2206	Male	Three y/o	Treatment (Wk 1)	Treatment	Feed Day	No	17:20	17:00 - 17:59	Evening	37,7	50	50,8	50,8	18,85	539,52	132,33
2019/05/20	CH2205	Male	Three y/o	Treatment (Wk 1)	Treatment	Feed Day	No	17:25	17:00 - 17:59	Evening	37,4		38,4	38,4	18,55		122,68
2019/05/20	CH2206	Male	Three y/o	Treatment (Wk 1)	Treatment	Feed Day	No	17:25	17:00 - 17:59	Evening	37,5	105	60,4	60,4	18,65	597,38	138,32
2019/05/20	CH2205	Male	Three y/o	Treatment (Wk 1)	Treatment	Feed Day	No	17:30	17:00 - 17:59	Evening	37,5		25,2	25,2	18,65		108,22
2019/05/20	CH2206	Male	Three y/o	Treatment (Wk 1)	Treatment	Feed Day	No	17:30	17:00 - 17:59	Evening	37,5	48	70,4	70,4	18,65	536,04	143,64
2019/05/20	CH2205	Male	Three y/o	Treatment (Wk 1)	Treatment	Feed Day	No	17:35	17:00 - 17:59	Evening	37,5	86	22,0	22,0	18,65	582,77	103,57
2019/05/20	CH2206	Male	Three y/o	Treatment (Wk 1)	Treatment	Feed Day	No	17:35	17:00 - 17:59	Evening	37,7	82	61,8	61,8	18,85	579,19	139,12
2019/05/20	CH2205	Male	Three y/o	Treatment (Wk 1)	Treatment	Feed Day	No	17:40	17:00 - 17:59	Evening	37,5	76	25,8	25,8	18,65	573,39	109,02
2019/05/20	CH2206	Male	Three y/o	Treatment (Wk 1)	Treatment	Feed Day	No	17:40	17:00 - 17:59	Evening	37,7	86	52,2	52,2	18,85	582,77	133,27
2019/05/20	CH2205	Male	Three y/o	Treatment (Wk 1)	Treatment	Feed Day	No	17:45	17:00 - 17:59	Evening	37,5	64	21,0	21,0	18,65	559,88	101,98
2019/05/20	CH2206	Male	Three y/o	Treatment (Wk 1)	Treatment	Feed Day	No	17:45	17:00 - 17:59	Evening	37,8		37,6	37,6	18,95		121,96
2019/05/20	CH2205	Male	Three y/o	Treatment (Wk 1)	Treatment	Feed Day	No	17:50	17:00 - 17:59	Evening	37,5	68	19,0	19,0	18,65	564,71	98,56
2019/05/20	CH2206	Male	Three y/o	Treatment (Wk 1)	Treatment	Feed Day	No	17:50	17:00 - 17:59	Evening	37,8	81	46,4	46,4	18,95	578,26	129,21
2019/05/20	CH2205	Male	Three y/o	Treatment (Wk 1)	Treatment	Feed Day	No	17:55	17:00 - 17:59	Evening	37,5	77	21,0	21,0	18,65	574,39	101,98
2019/05/20	CH2206	Male	Three y/o	Treatment (Wk 1)	Treatment	Feed Day	No	17:55	17:00 - 17:59	Evening	37,8	84	48,0	48,0	18,95	581,01	130,38
2019/05/20	CH2205	Male	Three y/o	Treatment (Wk 1)	Treatment	Feed Day	No	18:00	18:00 - 18:59	Evening	37,6	70	24,2	24,2	18,75	566,99	106,83
2019/05/20	CH2206	Male	Three y/o	Treatment (Wk 1)	Treatment	Feed Day	No	18:00	18:00 - 18:59	Evening	37,9	68	51,2	51,2	19,05	564,71	132,61
2019/05/20	CH2205	Male	Three y/o	Treatment (Wk 1)	Treatment	Feed Day	No	18:05	18:00 - 18:59	Evening	37,6	120	21,2	21,2	18,75	606,78	102,31
2019/05/20	CH2206	Male	Three y/o	Treatment (Wk 1)	Treatment	Feed Day	No	18:05	18:00 - 18:59	Evening	37,9	86	60,2	60,2	19,05	582,77	138,21
2019/05/20	CH2205	Male	Three y/o	Treatment (Wk 1)	Treatment	Feed Day	No	18:10	18:00 - 18:59	Evening	37,7	78	20,8	20,8	18,85	575,38	101,65
2019/05/20	CH2206	Male	Three y/o	Treatment (Wk 1)	Treatment	Feed Day	No	18:10	18:00 - 18:59	Evening	37,9	70	56,0	56,0	19,05	566,99	135,70
2019/05/20	CH2205	Male	Three y/o	Treatment (Wk 1)	Treatment	Feed Day	No	18:15	18:00 - 18:59	Evening	37,7	43	18,8	18,8	18,85	526,50	98,20
2019/05/20	CH2206	Male	Three y/o	Treatment (Wk 1)	Treatment	Feed Day	No	18:15	18:00 - 18:59	Evening	38,0	80	44,0	44,0	19,15	577,31	127,37
2019/05/20	CH2205	Male	Three y/o	Treatment (Wk 1)	Treatment	Feed Day	No	18:20	18:00 - 18:59	Evening	37,9	108	26,8	26,8	19,05	599,39	110,33
2019/05/20	CH2206	Male	Three y/o	Treatment (Wk 1)	Treatment	Feed Day	No	18:20	18:00 - 18:59	Evening	38,0	123	34,2	34,2	19,15	608,49	118,70
2019/05/20	CH2205	Male	Three y/o	Treatment (Wk 1)	Treatment	Feed Day	No	18:25	18:00 - 18:59	Evening	37,9	82	20,4	20,4	19,05	579,19	100,99
2019/05/20	CH2206	Male	Three y/o	Treatment (Wk 1)	Treatment	Feed Day	No	18:25	18:00 - 18:59	Evening	37,9	76	28,2	28,2	19,05	573,39	112,07
2019/05/20	CH2205	Male	Three y/o	Treatment (Wk 1)	Treatment	Feed Day	No	18:30	18:00 - 18:59	Evening	38,0		29,8	29,8	19,15		113,97
2019/05/20	CH2206	Male	Three y/o	Treatment (Wk 1)	Treatment	Feed Day	No	18:30	18:00 - 18:59	Evening	37,9	162	46,4	46,4	19,05	626,88	129,21
2019/05/20	CH2205	Male	Three y/o	Treatment (Wk 1)	Treatment	Feed Day	No	18:35	18:00 - 18:59	Evening	37,9		34,4	34,4	19,05		118,90
2019/05/20	CH2206	Male	Three y/o	Treatment (Wk 1)	Treatment	Feed Day	No	18:35	18:00 - 18:59	Evening	37,8	82	79,6	79,6	18,95	579,19	147,90
2019/05/20	CH2205	Male	Three y/o	Treatment (Wk 1)	Treatment	Feed Day	No	18:40	18:00 - 18:59	Evening	37,9		35,0	35,0	19,05		119,49
2019/05/20	CH2206	Male	Three y/o	Treatment (Wk 1)	Treatment	Feed Day	No	18:40	18:00 - 18:59	Evening	37,8	83	20,0	20,0	18,95	580,11	100,31
2019/05/20	CH2205	Male	Three y/o	Treatment (Wk 1)	Treatment	Feed Day	No	18:45	18:00 - 18:59	Evening	37,8	119	26,6	26,6	18,95	606,20	110,07
2019/05/20	CH2206	Male	Three y/o	Treatment (Wk 1)	Treatment	Feed Day	No	18:45	18:00 - 18:59	Evening	37,8	74	9,8	9,8	18,95	571,33	76,07
2019/05/20	CH2205	Male	Three y/o	Treatment (Wk 1)	Treatment	Feed Day	No	18:50	18:00 - 18:59	Evening	37,7		24,0	24,0	18,85		106,55
2019/05/20	CH2206	Male	Three y/o	Treatment (Wk 1)	Treatment	Feed Day	No	18:50	18:00 - 18:59	Evening	37,9	82	8,2	8,2	19,05	579,19	70,05
2019/05/20	CH2205	Male	Three y/o	Treatment (Wk 1)	Treatment	Feed Day	No	18:55	18:00 - 18:59	Evening	37,7	84	24,4	24,4	18,85	581,01	107,11
2019/05/20	CH2206	Male	Three y/o	Treatment (Wk 1)	Treatment	Feed Day	No	18:55	18:00 - 18:59	Evening	37,9	79	8,0	8,0	19,05	576,36	69,22
2019/05/20	CH2205	Male	Three y/o	Treatment (Wk 1)	Treatment	Feed Day	No	19:00	19:00 - 19:59	Evening	37,7	58	22,8	22,8	18,85	551,90	104,79
2019/05/20	CH2206	Male	Three y/o	Treatment (Wk 1)	Treatment	Feed Day	No	19:00	19:00 - 19:59	Evening	38,0	78	5,8	5,8	19,15	575,38	58,39
2019/05/20	CH2205	Male	Three y/o	Treatment (Wk 1)	Treatment	Feed Day	No	19:05	19:00 - 19:59	Evening	37,7	58	24,8	24,8	18,85	551,90	107,67
2019/05/20	CH2206	Male	Three y/o	Treatment (Wk 1)	Treatment	Feed Day	No	19:05	19:00 - 19:59	Evening	38,0	63	6,0	6,0	19,15	558,62	59,53
2019/05/20	CH2205	Male	Three y/o	Treatment (Wk 1)	Treatment	Feed Day	No	19:10	19:00 - 19:59	Evening	37,7	59	22,2	22,2	18,85	553,30	103,88
2019/05/20	CH2206	Male	Three y/o	Treatment (Wk 1)	Treatment	Feed Day	No	19:10	19:00 - 19:59	Evening	38,0	49	41,4	41,4	19,15	537,80	125,27
2019/05/20	CH2205	Male	Three y/o	Treatment (Wk 1)	Treatment	Feed Day	No	19:15	19:00 - 19:59	Evening	37,8	68	19,0	19,0	18,95	564,71	98,56
2019/05/20	CH2206	Male	Three y/o	Treatment (Wk 1)	Treatment	Feed Day	No	19:15	19:00 - 19:59	Evening	38,0	84	61,8	61,8	19,15	581,01	139,12
2019/05/20	CH2205	Male	Three y/o	Treatment (Wk 1)	Treatment	Feed Day	No	19:20	19:00 - 19:59	Evening	37,9	79	21,0	21,0	19,05	576,36	101,98
2019/05/20	CH2206	Male	Three y/o	Treatment (Wk 1)	Treatment	Feed Day	No	19:20	19:00 - 19:59	Evening	37,9	73	47,6	47,6	19,05	570,27	130,09
2019/05/20	CH2205	Male	Three y/o	Treatment (Wk 1)	Treatment	Feed Day	No	19:25	19:00 - 19:59	Evening	38,0	84	22,4	22,4	19,15	581,01	104,19
2019/05/20	CH2206	Male	Three y/o	Treatment (Wk 1)	Treatment	Feed Day	No	19:25	19:00 - 19:59	Evening	37,8	46	50,6	50,6	18,95	532,38	132,20
2019/05/20	CH2205	Male	Three y/o	Treatment (Wk 1)	Treatment	Feed Day	No	19:30	19:00 - 19:59	Evening	38,0	98	16,0	16,0	19,15	592,41	92,71
2019/05/20	CH2206	Male	Three y/o	Treatment (Wk 1)	Treatment	Feed Day	No	19:30	19:00 - 19:59	Evening	37,7	46	47,4	47,4	18,85	532,38	129,94
2019/05/20	CH2205	Male	Three y/o	Treatment (Wk 1)	Treatment	Feed Day	No	19:35	19:00 - 19:59	Evening	38,1	82	18,4	18,4	19,26	579,19	97,47
2019/05/20	CH2206	Male	Three y/o	Treatment (Wk 1)	Treatment	Feed Day	No	19:35	19:00 - 19:59	Evening	37,7	84	49,2	49,2	18,85	581,01	131,23
2019/05/20	CH2205	Male	Three y/o	Treatment (Wk 1)	Treatment	Feed Day	No	19:40	19:00 - 19:59	Evening	38,1	58	17,2	17,2	19,26	551,90	95,17
2019/05/20	CH2206	Male	Three y/o	Treatment (Wk 1)	Treatment	Feed Day	No	19:40	19:00 - 19:59	Evening	37,7	57	57,2	57,2	18,85	550,47	136,44
2019/05/20	CH2205	Male	Three y/o	Treatment (Wk 1)	Treatment	Feed Day	No	19:45	19:00 - 19:59	Evening	38,1	83	15,8	15,8	19,26	580,11	92,28
2019/05/20	CH2206	Male	Three y/o	Treatment (Wk 1)	Treatment	Feed Day	No	19:45	19:00 - 19:59	Evening	37,6	83	61,2	61,2	18,75	580,11	138,78
2019/05/20	CH2205	Male	Three y/o	Treatment (Wk 1)	Treatment	Feed Day	No	19:50	19:00 - 19:59	Evening	38,1	44	17,8	17,8	19,26	528,51	96,34

2019/05/20	CH2206	Male	Three y/o	Treatment (Wk 1)	Treatment	Feed Day	No	19:50	19:00 - 19:59	Evening	37,5	86	57,4	57,4	18,65	582,77	136,56
2019/05/20	CH2205	Male	Three y/o	Treatment (Wk 1)	Treatment	Feed Day	No	19:55	19:00 - 19:59	Evening	38,1	58	14,2	14,2	19,26	551,90	88,65
2019/05/20	CH2206	Male	Three y/o	Treatment (Wk 1)	Treatment	Feed Day	No	19:55	19:00 - 19:59	Evening	37,6	45	58,8	58,8	18,75	530,47	137,39
2019/05/20	CH2205	Male	Three y/o	Treatment (Wk 1)	Treatment	Feed Day	No	20:00	20:00 - 20:59	Night	38,1	86	19,4	19,4	19,26	582,77	99,28
2019/05/20	CH2206	Male	Three y/o	Treatment (Wk 1)	Treatment	Feed Day	No	20:00	20:00 - 20:59	Night	37,7	50	60,0	60,0	18,85	539,52	138,09
2019/05/20	CH2205	Male	Three y/o	Treatment (Wk 1)	Treatment	Feed Day	No	20:05	20:00 - 20:59	Night	38,0	84	15,6	15,6	19,15	581,01	91,85
2019/05/20	CH2206	Male	Three y/o	Treatment (Wk 1)	Treatment	Feed Day	No	20:05	20:00 - 20:59	Night	37,7	84	65,2	65,2	18,85	581,01	140,97
2019/05/20	CH2205	Male	Three y/o	Treatment (Wk 1)	Treatment	Feed Day	No	20:10	20:00 - 20:59	Night	38,0	58	10,0	10,0	19,15	551,90	76,76
2019/05/20	CH2206	Male	Three y/o	Treatment (Wk 1)	Treatment	Feed Day	No	20:10	20:00 - 20:59	Night	37,7	51	59,0	59,0	18,85	541,20	137,51
2019/05/20	CH2205	Male	Three y/o	Treatment (Wk 1)	Treatment	Feed Day	No	20:15	20:00 - 20:59	Night	38,0	55	12,6	12,6	19,15	547,52	84,59
2019/05/20	CH2206	Male	Three y/o	Treatment (Wk 1)	Treatment	Feed Day	No	20:15	20:00 - 20:59	Night	37,7	65	68,6	68,6	18,85	561,12	142,74
2019/05/20	CH2205	Male	Three y/o	Treatment (Wk 1)	Treatment	Feed Day	No	20:20	20:00 - 20:59	Night	38,0	80	14,0	14,0	19,15	577,31	88,17
2019/05/20	CH2206	Male	Three y/o	Treatment (Wk 1)	Treatment	Feed Day	No	20:20	20:00 - 20:59	Night	37,7	74	63,4	63,4	18,85	571,33	140,00
2019/05/20	CH2205	Male	Three y/o	Treatment (Wk 1)	Treatment	Feed Day	No	20:25	20:00 - 20:59	Night	38,0	63	11,4	11,4	19,15	558,62	81,19
2019/05/20	CH2206	Male	Three y/o	Treatment (Wk 1)	Treatment	Feed Day	No	20:25	20:00 - 20:59	Night	37,7	47	63,0	63,0	18,85	534,23	139,78
2019/05/20	CH2205	Male	Three y/o	Treatment (Wk 1)	Treatment	Feed Day	No	20:30	20:00 - 20:59	Night	38,0	85	10,4	10,4	19,15	581,90	78,08
2019/05/20	CH2206	Male	Three y/o	Treatment (Wk 1)	Treatment	Feed Day	No	20:30	20:00 - 20:59	Night	37,7	58	64,0	64,0	18,85	551,90	140,33
2019/05/20	CH2205	Male	Three y/o	Treatment (Wk 1)	Treatment	Feed Day	No	20:35	20:00 - 20:59	Night	38,0	47	19,0	19,0	19,15	534,23	98,56
2019/05/20	CH2206	Male	Three y/o	Treatment (Wk 1)	Treatment	Feed Day	No	20:35	20:00 - 20:59	Night	37,7	77	62,4	62,4	18,85	574,39	139,45
2019/05/20	CH2205	Male	Three y/o	Treatment (Wk 1)	Treatment	Feed Day	No	20:40	20:00 - 20:59	Night	38,0	68	12,0	12,0	19,15	564,71	82,93
2019/05/20	CH2206	Male	Three y/o	Treatment (Wk 1)	Treatment	Feed Day	No	20:40	20:00 - 20:59	Night	37,7	84	67,0	67,0	18,85	581,01	141,92
2019/05/20	CH2205	Male	Three y/o	Treatment (Wk 1)	Treatment	Feed Day	No	20:45	20:00 - 20:59	Night	38,0	54	12,0	12,0	19,15	545,99	82,93
2019/05/20	CH2206	Male	Three y/o	Treatment (Wk 1)	Treatment	Feed Day	No	20:45	20:00 - 20:59	Night	37,7	74	61,4	61,4	18,85	571,33	138,89
2019/05/20	CH2205	Male	Three y/o	Treatment (Wk 1)	Treatment	Feed Day	No	20:50	20:00 - 20:59	Night	38,0	94	13,6	13,6	19,15	589,37	87,18
2019/05/20	CH2206	Male	Three y/o	Treatment (Wk 1)	Treatment	Feed Day	No	20:50	20:00 - 20:59	Night	37,7	46	54,6	54,6	18,85	532,38	134,83
2019/05/20	CH2205	Male	Three y/o	Treatment (Wk 1)	Treatment	Feed Day	No	20:55	20:00 - 20:59	Night	37,9		11,2	11,2	19,05		80,59
2019/05/20	CH2206	Male	Three y/o	Treatment (Wk 1)	Treatment	Feed Day	No	20:55	20:00 - 20:59	Night	37,6	44	48,6	48,6	18,75	528,51	130,81
2019/05/20	CH2205	Male	Three y/o	Treatment (Wk 1)	Treatment	Feed Day	No	21:00	21:00 - 21:59	Night	37,9	59	8,8	8,8	19,05	553,30	72,43
2019/05/20	CH2206	Male	Three y/o	Treatment (Wk 1)	Treatment	Feed Day	No	21:00	21:00 - 21:59	Night	37,5	90	50,2	50,2	18,65	586,16	131,92
2019/05/20	CH2205	Male	Three y/o	Treatment (Wk 1)	Treatment	Feed Day	No	21:05	21:00 - 21:59	Night	37,9	61	8,2	8,2	19,05	556,01	70,05
2019/05/20	CH2206	Male	Three y/o	Treatment (Wk 1)	Treatment	Feed Day	No	21:05	21:00 - 21:59	Night	37,5	89	56,4	56,4	18,65	585,33	135,95
2019/05/20	CH2205	Male	Three y/o	Treatment (Wk 1)	Treatment	Feed Day	No	21:10	21:00 - 21:59	Night	37,9	100	5,6	5,6	19,05	593,87	57,21
2019/05/20	CH2206	Male	Three y/o	Treatment (Wk 1)	Treatment	Feed Day	No	21:10	21:00 - 21:59	Night	37,4	89	64,6	64,6	18,55	585,33	140,65
2019/05/20	CH2205	Male	Three y/o	Treatment (Wk 1)	Treatment	Feed Day	No	21:15	21:00 - 21:59	Night	37,9	92	2,6	2,6	19,05	587,78	31,57
2019/05/20	CH2206	Male	Three y/o	Treatment (Wk 1)	Treatment	Feed Day	No	21:15	21:00 - 21:59	Night	37,4	90	59,2	59,2	18,55	586,16	137,63
2019/05/20	CH2205	Male	Three y/o	Treatment (Wk 1)	Treatment	Feed Day	No	21:20	21:00 - 21:59	Night	37,9	62	3,2	3,2	19,05	557,33	38,49
2019/05/20	CH2206	Male	Three y/o	Treatment (Wk 1)	Treatment	Feed Day	No	21:20	21:00 - 21:59	Night	37,4	44	59,6	59,6	18,55	528,51	137,86
2019/05/20	CH2205	Male	Three y/o	Treatment (Wk 1)	Treatment	Feed Day	No	21:25	21:00 - 21:59	Night	37,9	58	1,2	1,2	19,05	551,90	5,99
2019/05/20	CH2206	Male	Three y/o	Treatment (Wk 1)	Treatment	Feed Day	No	21:25	21:00 - 21:59	Night	37,4	84	62,8	62,8	18,55	581,01	139,67
2019/05/20	CH2205	Male	Three y/o	Treatment (Wk 1)	Treatment	Feed Day	No	21:30	21:00 - 21:59	Night	37,9	84	4,4	4,4	19,05	581,01	49,12
2019/05/20	CH2206	Male	Three y/o	Treatment (Wk 1)	Treatment	Feed Day	No	21:30	21:00 - 21:59	Night	37,4	88	61,0	61,0	18,55	584,49	138,67
2019/05/20	CH2205	Male	Three y/o	Treatment (Wk 1)	Treatment	Feed Day	No	21:35	21:00 - 21:59	Night	37,9	56	5,0	5,0	19,05	549,01	53,41
2019/05/20	CH2206	Male	Three y/o	Treatment (Wk 1)	Treatment	Feed Day	No	21:35	21:00 - 21:59	Night	37,4	61	62,2	62,2	18,55	556,01	139,34
2019/05/20	CH2205	Male	Three y/o	Treatment (Wk 1)	Treatment	Feed Day	No	21:40	21:00 - 21:59	Night	37,9	63	5,8	5,8	19,05	558,62	58,39
2019/05/20	CH2206	Male	Three y/o	Treatment (Wk 1)	Treatment	Feed Day	No	21:40	21:00 - 21:59	Night	37,4	50	56,2	56,2	18,55	539,52	135,83
2019/05/20	CH2205	Male	Three y/o	Treatment (Wk 1)	Treatment	Feed Day	No	21:45	21:00 - 21:59	Night	37,9	60	7,2	7,2	19,05	554,67	65,66
2019/05/20	CH2206	Male	Three y/o	Treatment (Wk 1)	Treatment	Feed Day	No	21:45	21:00 - 21:59	Night	37,4	87	47,6	47,6	18,55	583,64	130,09
2019/05/20	CH2205	Male	Three y/o	Treatment (Wk 1)	Treatment	Feed Day	No	21:50	21:00 - 21:59	Night	37,9	88	5,4	5,4	19,05	584,49	55,99
2019/05/20	CH2206	Male	Three y/o	Treatment (Wk 1)	Treatment	Feed Day	No	21:50	21:00 - 21:59	Night	37,7	46	46,4	46,4	18,85	532,38	129,21
2019/05/20	CH2205	Male	Three y/o	Treatment (Wk 1)	Treatment	Feed Day	No	21:55	21:00 - 21:59	Night	37,9	54	6,4	6,4	19,05	545,99	61,70
2019/05/20	CH2206	Male	Three y/o	Treatment (Wk 1)	Treatment	Feed Day	No	21:55	21:00 - 21:59	Night	37,7	64	45,2	45,2	18,85	559,88	128,30
2019/05/20	CH2205	Male	Three y/o	Treatment (Wk 1)	Treatment	Feed Day	No	22:00	22:00 - 22:59	Night	37,9	78	3,2	3,2	19,05	575,38	38,49
2019/05/20	CH2206	Male	Three y/o	Treatment (Wk 1)	Treatment	Feed Day	No	22:00	22:00 - 22:59	Night	37,5	102	53,2	53,2	18,65	595,30	133,93
2019/05/20	CH2205	Male	Three y/o	Treatment (Wk 1)	Treatment	Feed Day	No	22:05	22:00 - 22:59	Night	37,9	78	2,2	2,2	19,05	575,38	26,02
2019/05/20	CH2206	Male	Three y/o	Treatment (Wk 1)	Treatment	Feed Day	No	22:05	22:00 - 22:59	Night	37,5	69	58,6	58,6	18,65	565,86	137,28
2019/05/20	CH2205	Male	Three y/o	Treatment (Wk 1)	Treatment	Feed Day	No	22:10	22:00 - 22:59	Night	37,9	51	28,4	28,4	19,05	541,20	112,31
2019/05/20	CH2206	Male	Three y/o	Treatment (Wk 1)	Treatment	Feed Day	No	22:10	22:00 - 22:59	Night	37,5	76	94,4	94,4	18,65	573,39	153,84
2019/05/20	CH2205	Male	Three y/o	Treatment (Wk 1)	Treatment	Feed Day	No	22:15	22:00 - 22:59	Night	37,8	62	5,6	5,6	18,95	557,33	57,21
2019/05/20	CH2206	Male	Three y/o	Treatment (Wk 1)	Treatment	Feed Day	No	22:15	22:00 - 22:59	Night	37,4	83	62,4	62,4	18,55	580,11	139,45
2019/05/20	CH2205	Male	Three y/o	Treatment (Wk 1)	Treatment	Feed Day	No	22:20	22:00 - 22:59	Night	37,6	92	2,8	2,8	18,75	587,78	34,04
2019/05/20	CH2206	Male	Three y/o	Treatment (Wk 1)	Treatment	Feed Day	No	22:20	22:00 - 22:59	Night	37,3	44	60,4	60,4	18,46	528,51	138,32
2019/05/20	CH2205	Male	Three y/o	Treatment (Wk 1)	Treatment	Feed Day	No	22:25	22:00 - 22:59	Night	37,6	94	8,8	8,8	18,75	589,37	72,43
2019/05/20	CH2206	Male	Three y/o	Treatment (Wk 1)	Treatment	Feed Day	No	22:25	22:00 - 22:59	Night	37,3	69	60,6	60,6	18,46	565,86	138,44
2019/05/20	CH2205	Male	Three y/o	Treatment (Wk 1)	Treatment	Feed Day	No	22:30	22:00 - 22:59	Night	37,6	93	19,2	19,2	18,75	588,58	98,92
2019/05/20	CH2206	Male	Three y/o	Treatment (Wk 1)	Treatment	Feed Day	No	22:30	22:00 - 22:59	Night	37,4	79	39,2	39,2	18,55	576,36	123,39
2019/05/20	CH2205	Male	Three y/o	Treatment (Wk 1)	Treatment	Feed Day	No	22:35	22:00 - 22:59	Night	37,7	65	21,0	21,0	18,85	561,12	101,98
2019/05/20	CH2206	Male	Three y/o	Treatment (Wk 1)	Treatment	Feed Day	No	22:35	22:00 - 22:59	Night	37,4	42	7,2	7,2	18,55	524,42	65,66
2019/05/20	CH2205	Male	Three y/o	Treatment (Wk 1)	Treatment	Feed Day	No	22:40	22:00 - 22:59	Night	37,7	73	19,8	19,8	18,85	570,27	99,97
2019/05/20	CH2206	Male	Three y/o	Treatment (Wk 1)	Treatment	Feed Day	No	22:40	22:00 - 22:59	Night	37,4	78	4,6	4,6	18,55	575,38	50,61
2019/05/20	CH2205	Male	Three y/o	Treatment (Wk 1)	Treatment	Feed Day	No	22:45	22:00 - 22:59	Night	37,7	63	18,6	18,6	18,85	558,62	97,84

	2019/05/20	CH2206	Male	Three y/o	Treatment (Wk 1)	Treatment	Feed Day	No	22:45	22:00 - 22:59	Night	37,4	51	7,6	7,6	18,55	541,20	67,49
	2019/05/20	CH2205	Male	Three y/o	Treatment (Wk 1)	Treatment	Feed Day	No	22:50	22:00 - 22:59	Night	37,7	55	18,4	18,4	18,85	547,52	97,47
	2019/05/20	CH2206	Male	Three y/o	Treatment (Wk 1)	Treatment	Feed Day	No	22:50	22:00 - 22:59	Night	37,4	84	6,0	6,0	18,55	581,01	59,53
	2019/05/20	CH2205	Male	Three y/o	Treatment (Wk 1)	Treatment	Feed Day	No	22:55	22:00 - 22:59	Night	37,7	73	25,0	25,0	18,85	570,27	107,94
	2019/05/20	CH2206	Male	Three y/o	Treatment (Wk 1)	Treatment	Feed Day	No	22:55	22:00 - 22:59	Night	37,4	72	10,0	10,0	18,55	569,20	76,76
	2019/05/20	CH2205	Male	Three y/o	Treatment (Wk 1)	Treatment	Feed Day	No	23:00	23:00 - 23:59	Night	37,7	60	15,4	15,4	18,85	554,67	91,41
	2019/05/20	CH2206	Male	Three y/o	Treatment (Wk 1)	Treatment	Feed Day	No	23:00	23:00 - 23:59	Night	37,4	86	3,8	3,8	18,55	582,77	44,22
	2019/05/20	CH2205	Male	Three y/o	Treatment (Wk 1)	Treatment	Feed Day	No	23:05	23:00 - 23:59	Night	37,7	93	17,4	17,4	18,85	588,58	95,56
	2019/05/20	CH2206	Male	Three y/o	Treatment (Wk 1)	Treatment	Feed Day	No	23:05	23:00 - 23:59	Night	37,4	84	10,4	10,4	18,55	581,01	78,08
	2019/05/20	CH2205	Male	Three y/o	Treatment (Wk 1)	Treatment	Feed Day	No	23:10	23:00 - 23:59	Night	37,7	73	23,8	23,8	18,85	570,27	106,26
	2019/05/20	CH2206	Male	Three y/o	Treatment (Wk 1)	Treatment	Feed Day	No	23:10	23:00 - 23:59	Night	37,4	67	8,0	8,0	18,55	563,53	69,22
	2019/05/20	CH2205	Male	Three y/o	Treatment (Wk 1)	Treatment	Feed Day	No	23:15	23:00 - 23:59	Night	37,7	56	20,2	20,2	18,85	549,01	100,65
	2019/05/20	CH2206	Male	Three y/o	Treatment (Wk 1)	Treatment	Feed Day	No	23:15	23:00 - 23:59	Night	37,3	51	4,8	4,8	18,46	541,20	52,04
	2019/05/20	CH2205	Male	Three y/o	Treatment (Wk 1)	Treatment	Feed Day	No	23:20	23:00 - 23:59	Night	37,6	41	20,4	20,4	18,75	522,29	100,99
	2019/05/20	CH2206	Male	Three y/o	Treatment (Wk 1)	Treatment	Feed Day	No	23:20	23:00 - 23:59	Night	37,3	63	4,6	4,6	18,46	558,62	50,61
	2019/05/20	CH2205	Male	Three y/o	Treatment (Wk 1)	Treatment	Feed Day	No	23:25	23:00 - 23:59	Night	37,6	46	18,2	18,2	18,75	532,38	97,10
	2019/05/20	CH2206	Male	Three y/o	Treatment (Wk 1)	Treatment	Feed Day	No	23:25	23:00 - 23:59	Night	37,3	77	5,4	5,4	18,46	574,39	55,99
	2019/05/20	CH2205	Male	Three y/o	Treatment (Wk 1)	Treatment	Feed Day	No	23:30	23:00 - 23:59	Night	37,6	41	17,0	17,0	18,75	522,29	94,77
	2019/05/20	CH2206	Male	Three y/o	Treatment (Wk 1)	Treatment	Feed Day	No	23:30	23:00 - 23:59	Night	37,3	43	6,8	6,8	18,46	526,50	63,74
	2019/05/20	CH2205	Male	Three y/o	Treatment (Wk 1)	Treatment	Feed Day	No	23:35	23:00 - 23:59	Night	37,6	50	25,2	25,2	18,75	539,52	108,22
	2019/05/20	CH2206	Male	Three y/o	Treatment (Wk 1)	Treatment	Feed Day	No	23:35	23:00 - 23:59	Night	37,3	65	11,6	11,6	18,46	561,12	81,78
	2019/05/20	CH2205	Male	Three y/o	Treatment (Wk 1)	Treatment	Feed Day	No	23:40	23:00 - 23:59	Night	37,7	57	20,2	20,2	18,85	550,47	100,65
	2019/05/20	CH2206	Male	Three y/o	Treatment (Wk 1)	Treatment	Feed Day	No	23:40	23:00 - 23:59	Night	37,3	79	28,4	28,4	18,46	576,36	112,31
	2019/05/20	CH2205	Male	Three y/o	Treatment (Wk 1)	Treatment	Feed Day	No	23:45	23:00 - 23:59	Night	37,7	50	22,4	22,4	18,85	539,52	104,19
	2019/05/20	CH2206	Male	Three y/o	Treatment (Wk 1)	Treatment	Feed Day	No	23:45	23:00 - 23:59	Night	37,4	67	17,0	17,0	18,55	563,53	94,77
	2019/05/20	CH2205	Male	Three y/o	Treatment (Wk 1)	Treatment	Feed Day	No	23:50	23:00 - 23:59	Night	37,7	92	18,6	18,6	18,85	587,78	97,84
	2019/05/20	CH2206	Male	Three y/o	Treatment (Wk 1)	Treatment	Feed Day	No	23:50	23:00 - 23:59	Night	37,5	59	19,8	19,8	18,65	553,30	99,97
	2019/05/20	CH2205	Male	Three y/o	Treatment (Wk 1)	Treatment	Feed Day	No	23:55	23:00 - 23:59	Night	37,7	95	21,8	21,8	18,85	590,14	103,26
	2019/05/20	CH2206	Male	Three y/o	Treatment (Wk 1)	Treatment	Feed Day	No	23:55	23:00 - 23:59	Night	37,5	88	13,4	13,4	18,65	584,49	86,68
7	2019/05/21	CH2205	Male	Three y/o	Treatment (Wk 1)	Treatment	Feed Day	No	00:00	00:00 - 00:59	Early Morning	37,7	90	22,4	22,4	18,85	586,16	104,19
	2019/05/21	CH2206	Male	Three y/o	Treatment (Wk 1)	Treatment	Feed Day	No	00:00	00:00 - 00:59	Early Morning	37,5	45	20,2	20,2	18,65	530,47	100,65
	2019/05/21	CH2205	Male	Three y/o	Treatment (Wk 1)	Treatment	Feed Day	No	00:05	00:00 - 00:59	Early Morning	37,7	54	23,8	23,8	18,85	545,99	106,26
	2019/05/21	CH2206	Male	Three y/o	Treatment (Wk 1)	Treatment	Feed Day	No	00:05	00:00 - 00:59	Early Morning	37,5	89	17,4	17,4	18,65	585,33	95,56
	2019/05/21	CH2205	Male	Three y/o	Treatment (Wk 1)	Treatment	Feed Day	No	00:10	00:00 - 00:59	Early Morning	37,7	75	24,4	24,4	18,85	572,36	107,11
	2019/05/21	CH2206	Male	Three y/o	Treatment (Wk 1)	Treatment	Feed Day	No	00:10	00:00 - 00:59	Early Morning	37,5	66	15,4	15,4	18,65	562,34	91,41
	2019/05/21	CH2205	Male	Three y/o	Treatment (Wk 1)	Treatment	Feed Day	No	00:15	00:00 - 00:59	Early Morning	37,7	89	17,8	17,8	18,85	585,33	96,34
	2019/05/21	CH2206	Male	Three y/o	Treatment (Wk 1)	Treatment	Feed Day	No	00:15	00:00 - 00:59	Early Morning	37,5	87	24,0	24,0	18,65	583,64	106,55
	2019/05/21	CH2205	Male	Three y/o	Treatment (Wk 1)	Treatment	Feed Day	No	00:20	00:00 - 00:59	Early Morning	37,7	55	22,0	22,0	18,85	547,52	103,57
	2019/05/21	CH2206	Male	Three y/o	Treatment (Wk 1)	Treatment	Feed Day	No	00:20	00:00 - 00:59	Early Morning	37,5	56	29,2	29,2	18,65	549,01	113,27
	2019/05/21	CH2205	Male	Three y/o	Treatment (Wk 1)	Treatment	Feed Day	No	00:25	00:00 - 00:59	Early Morning	37,7	167	21,0	21,0	18,85	628,84	101,98
	2019/05/21	CH2206	Male	Three y/o	Treatment (Wk 1)	Treatment	Feed Day	No	00:25	00:00 - 00:59	Early Morning	37,5	101	67,8	67,8	18,65	594,59	142,33
	2019/05/21	CH2205	Male	Three y/o	Treatment (Wk 1)	Treatment	Feed Day	No	00:30	00:00 - 00:59	Early Morning	37,7	115	20,6	20,6	18,85	603,82	101,32
	2019/05/21	CH2206	Male	Three y/o	Treatment (Wk 1)	Treatment	Feed Day	No	00:30	00:00 - 00:59	Early Morning	37,5	85	58,0	58,0	18,65	581,90	136,92
	2019/05/21	CH2205	Male	Three y/o	Treatment (Wk 1)	Treatment	Feed Day	No	00:35	00:00 - 00:59	Early Morning	37,6	86	20,6	20,6	18,75	582,77	101,32
	2019/05/21	CH2206	Male	Three y/o	Treatment (Wk 1)	Treatment	Feed Day	No	00:35	00:00 - 00:59	Early Morning	37,5	46	65,0	65,0	18,65	532,38	140,87
	2019/05/21	CH2205	Male	Three y/o	Treatment (Wk 1)	Treatment	Feed Day	No	00:40	00:00 - 00:59	Early Morning	37,6	51	19,0	19,0	18,75	541,20	98,56
	2019/05/21	CH2206	Male	Three y/o	Treatment (Wk 1)	Treatment	Feed Day	No	00:40	00:00 - 00:59	Early Morning	37,5	87	58,2	58,2	18,65	583,64	137,04
	2019/05/21	CH2205	Male	Three y/o	Treatment (Wk 1)	Treatment	Feed Day	No	00:45	00:00 - 00:59	Early Morning	37,7	46	17,8	17,8	18,85	532,38	96,34
	2019/05/21	CH2206	Male	Three y/o	Treatment (Wk 1)	Treatment	Feed Day	No	00:45	00:00 - 00:59	Early Morning	37,5	53	58,2	58,2	18,65	544,43	137,04
	2019/05/21	CH2205	Male	Three y/o	Treatment (Wk 1)	Treatment	Feed Day	No	00:50	00:00 - 00:59	Early Morning	37,7	57	19,0	19,0	18,85	550,47	98,56
	2019/05/21	CH2206	Male	Three y/o	Treatment (Wk 1)	Treatment	Feed Day	No	00:50	00:00 - 00:59	Early Morning	37,5	86	55,4	55,4	18,65	582,77	135,33
	2019/05/21	CH2205	Male	Three y/o	Treatment (Wk 1)	Treatment	Feed Day	No	00:55	00:00 - 00:59	Early Morning	37,6	62	13,8	13,8	18,75	557,33	87,68
	2019/05/21	CH2206	Male	Three y/o	Treatment (Wk 1)	Treatment	Feed Day	No	00:55	00:00 - 00:59	Early Morning	37,5	52	48,8	48,8	18,65	542,83	130,95
	2019/05/21	CH2205	Male	Three y/o	Treatment (Wk 1)	Treatment	Feed Day	No	01:00	01:00 - 01:59	Early Morning	37,5	45	18,6	18,6	18,65	530,47	97,84
	2019/05/21	CH2206	Male	Three y/o	Treatment (Wk 1)	Treatment	Feed Day	No	01:00	01:00 - 01:59	Early Morning	37,5	47	55,8	55,8	18,65	534,23	135,58
	2019/05/21	CH2205	Male	Three y/o	Treatment (Wk 1)	Treatment	Feed Day	No	01:05	01:00 - 01:59	Early Morning	37,4	54	11,8	11,8	18,55	545,99	82,36
	2019/05/21	CH2206	Male	Three y/o	Treatment (Wk 1)	Treatment	Feed Day	No	01:05	01:00 - 01:59	Early Morning	37,5	90	57,4	57,4	18,65	586,16	136,56
	2019/05/21	CH2205	Male	Three y/o	Treatment (Wk 1)	Treatment	Feed Day	No	01:10	01:00 - 01:59	Early Morning	37,4	55	19,8	19,8	18,55	547,52	99,97
	2019/05/21	CH2206	Male	Three y/o	Treatment (Wk 1)	Treatment	Feed Day	No	01:10	01:00 - 01:59	Early Morning	37,5		43,8	43,8	18,65		127,22
	2019/05/21	CH2205	Male	Three y/o	Treatment (Wk 1)	Treatment	Feed Day	No	01:15	01:00 - 01:59	Early Morning	37,5	63	7,6	7,6	18,65	558,62	67,49
	2019/05/21	CH2206	Male	Three y/o	Treatment (Wk 1)	Treatment	Feed Day	No	01:15	01:00 - 01:59	Early Morning	37,4	54	35,2	35,2	18,55	545,99	119,69
	2019/05/21	CH2205	Male	Three y/o	Treatment (Wk 1)	Treatment	Feed Day	No	01:20	01:00 - 01:59	Early Morning	37,6	44	3,8	3,8	18,75	528,51	44,22
	2019/05/21	CH2206	Male	Three y/o	Treatment (Wk 1)	Treatment	Feed Day	No	01:20	01:00 - 01:59	Early Morning	37,3	42	22,2	22,2	18,46	524,42	103,88
	2019/05/21	CH2205	Male	Three y/o	Treatment (Wk 1)	Treatment	Feed Day	No	01:25	01:00 - 01:59	Early Morning	37,7	51	6,6	6,6	18,85	541,20	62,73
	2019/05/21	CH2206	Male	Three y/o	Treatment (Wk 1)	Treatment	Feed Day	No	01:25	01:00 - 01:59	Early Morning	37,3	49	21,4	21,4	18,46	537,80	102,63
	2019/05/21	CH2205	Male	Three y/o	Treatment (Wk 1)	Treatment	Feed Day	No	01:30	01:00 - 01:59	Early Morning	37,7	52	5,2	5,2	18,85	542,83	54,72
	2019/05/21	CH2206	Male	Three y/o	Treatment (Wk 1)	Treatment	Feed Day	No	01:30	01:00 - 01:59	Early Morning	37,5	54	19,6	19,6	18,65	545,99	99,63
	2019/05/21	CH2205	Male	Three y/o	Treatment (Wk 1)	Treatment	Feed Day	No	01:35	01:00 - 01:59	Early Morning	37,6	83	3,6	3,6	18,75	580,11	42,42
	2019/05/21	CH2206	Male	Three y/o	Treatment (Wk 1)	Treatment	Feed Day	No	01:35	01:00 - 01:59	Early Morning	37,5	90	36,0	36,0	18,65	586,16	120,46
	2019/05/21	CH2205	Male	Three y/o	Treatment (Wk 1)	Treatment	Feed Day	No	01:40	01:00 - 01:59	Early Morning	37,6	89	6,2	6,2	18,75	585,33	60,63

2019/05/21	CH2206	Male	Three y/o	Treatment (Wk 1)	Treatment	Feed Day	No	01:40	01:00 - 01:59	Early Morning	37,5	46	64,2	64,2	18,65	532,38	140,44
2019/05/21	CH2205	Male	Three y/o	Treatment (Wk 1)	Treatment	Feed Day	No	01:45	01:00 - 01:59	Early Morning	37,6	78	11,0	11,0	18,75	575,38	79,98
2019/05/21	CH2206	Male	Three y/o	Treatment (Wk 1)	Treatment	Feed Day	No	01:45	01:00 - 01:59	Early Morning	37,4	43	61,0	61,0	18,55	526,50	138,67
2019/05/21	CH2205	Male	Three y/o	Treatment (Wk 1)	Treatment	Feed Day	No	01:50	01:00 - 01:59	Early Morning	37,6	79	9,8	9,8	18,75	576,36	76,07
2019/05/21	CH2206	Male	Three y/o	Treatment (Wk 1)	Treatment	Feed Day	No	01:50	01:00 - 01:59	Early Morning	37,4	54	63,4	63,4	18,55	545,99	140,00
2019/05/21	CH2205	Male	Three y/o	Treatment (Wk 1)	Treatment	Feed Day	No	01:55	01:00 - 01:59	Early Morning	37,6	129	30,2	30,2	18,75	611,75	114,42
2019/05/21	CH2206	Male	Three y/o	Treatment (Wk 1)	Treatment	Feed Day	No	01:55	01:00 - 01:59	Early Morning	37,4	88	64,4	64,4	18,55	584,49	140,55
2019/05/21	CH2205	Male	Three y/o	Treatment (Wk 1)	Treatment	Feed Day	No	02:00	02:00 - 02:59	Early Morning	37,5		30,6	30,6	18,65		114,87
2019/05/21	CH2206	Male	Three y/o	Treatment (Wk 1)	Treatment	Feed Day	No	02:00	02:00 - 02:59	Early Morning	37,4	42	61,8	61,8	18,55	524,42	139,12
2019/05/21	CH2205	Male	Three y/o	Treatment (Wk 1)	Treatment	Feed Day	No	02:05	02:00 - 02:59	Early Morning	37,6		29,0	29,0	18,75		113,03
2019/05/21	CH2206	Male	Three y/o	Treatment (Wk 1)	Treatment	Feed Day	No	02:05	02:00 - 02:59	Early Morning	37,4	85	62,0	62,0	18,55	581,90	139,23
2019/05/21	CH2205	Male	Three y/o	Treatment (Wk 1)	Treatment	Feed Day	No	02:10	02:00 - 02:59	Early Morning	37,6		32,0	32,0	18,75		116,41
2019/05/21	CH2206	Male	Three y/o	Treatment (Wk 1)	Treatment	Feed Day	No	02:10	02:00 - 02:59	Early Morning	37,3	57	61,4	61,4	18,46	550,47	138,89
2019/05/21	CH2205	Male	Three y/o	Treatment (Wk 1)	Treatment	Feed Day	No	02:15	02:00 - 02:59	Early Morning	37,6	57	20,6	20,6	18,75	550,47	101,32
2019/05/21	CH2206	Male	Three y/o	Treatment (Wk 1)	Treatment	Feed Day	No	02:15	02:00 - 02:59	Early Morning	37,3	84	61,6	61,6	18,46	581,01	139,00
2019/05/21	CH2205	Male	Three y/o	Treatment (Wk 1)	Treatment	Feed Day	No	02:20	02:00 - 02:59	Early Morning	37,6	43	18,0	18,0	18,75	526,50	96,72
2019/05/21	CH2206	Male	Three y/o	Treatment (Wk 1)	Treatment	Feed Day	No	02:20	02:00 - 02:59	Early Morning	37,3	45	57,2	57,2	18,46	530,47	136,44
2019/05/21	CH2205	Male	Three y/o	Treatment (Wk 1)	Treatment	Feed Day	No	02:25	02:00 - 02:59	Early Morning	37,6	88	19,0	19,0	18,75	584,49	98,56
2019/05/21	CH2206	Male	Three y/o	Treatment (Wk 1)	Treatment	Feed Day	No	02:25	02:00 - 02:59	Early Morning	37,3	57	62,2	62,2	18,46	550,47	139,34
2019/05/21	CH2205	Male	Three y/o	Treatment (Wk 1)	Treatment	Feed Day	No	02:30	02:00 - 02:59	Early Morning	37,5	49	19,2	19,2	18,65	537,80	98,92
2019/05/21	CH2206	Male	Three y/o	Treatment (Wk 1)	Treatment	Feed Day	No	02:30	02:00 - 02:59	Early Morning	37,3	84	61,2	61,2	18,46	581,01	138,78
2019/05/21	CH2205	Male	Three y/o	Treatment (Wk 1)	Treatment	Feed Day	No	02:35	02:00 - 02:59	Early Morning	37,5	57	21,0	21,0	18,65	550,47	101,98
2019/05/21	CH2206	Male	Three y/o	Treatment (Wk 1)	Treatment	Feed Day	No	02:35	02:00 - 02:59	Early Morning	37,3	42	54,8	54,8	18,46	524,42	134,96
2019/05/21	CH2205	Male	Three y/o	Treatment (Wk 1)	Treatment	Feed Day	No	02:40	02:00 - 02:59	Early Morning	37,5	59	16,6	16,6	18,65	553,30	93,96
2019/05/21	CH2206	Male	Three y/o	Treatment (Wk 1)	Treatment	Feed Day	No	02:40	02:00 - 02:59	Early Morning	37,3	40	56,8	56,8	18,46	520,09	136,20
2019/05/21	CH2205	Male	Three y/o	Treatment (Wk 1)	Treatment	Feed Day	No	02:45	02:00 - 02:59	Early Morning	37,5	88	21,0	21,0	18,65	584,49	101,98
2019/05/21	CH2206	Male	Three y/o	Treatment (Wk 1)	Treatment	Feed Day	No	02:45	02:00 - 02:59	Early Morning	37,3	86	57,6	57,6	18,46	582,77	136,68
2019/05/21	CH2205	Male	Three y/o	Treatment (Wk 1)	Treatment	Feed Day	No	02:50	02:00 - 02:59	Early Morning	37,5	86	21,0	21,0	18,65	582,77	101,98
2019/05/21	CH2206	Male	Three y/o	Treatment (Wk 1)	Treatment	Feed Day	No	02:50	02:00 - 02:59	Early Morning	37,3	57	7,8	7,8	18,46	550,47	68,36
2019/05/21	CH2205	Male	Three y/o	Treatment (Wk 1)	Treatment	Feed Day	No	02:55	02:00 - 02:59	Early Morning	37,5	71	34,6	34,6	18,65	568,10	119,10
2019/05/21	CH2206	Male	Three y/o	Treatment (Wk 1)	Treatment	Feed Day	No	02:55	02:00 - 02:59	Early Morning	37,4	45	9,4	9,4	18,55	530,47	74,66
2019/05/21	CH2205	Male	Three y/o	Treatment (Wk 1)	Treatment	Feed Day	No	03:00	03:00 - 03:59	Early Morning	37,5	55	25,8	25,8	18,65	547,52	109,02
2019/05/21	CH2206	Male	Three y/o	Treatment (Wk 1)	Treatment	Feed Day	No	03:00	03:00 - 03:59	Early Morning	37,4	64	12,4	12,4	18,55	559,88	84,04
2019/05/21	CH2205	Male	Three y/o	Treatment (Wk 1)	Treatment	Feed Day	No	03:05	03:00 - 03:59	Early Morning	37,5	92	27,0	27,0	18,65	587,78	110,58
2019/05/21	CH2206	Male	Three y/o	Treatment (Wk 1)	Treatment	Feed Day	No	03:05	03:00 - 03:59	Early Morning	37,4	88	16,4	16,4	18,55	584,49	93,55
2019/05/21	CH2205	Male	Three y/o	Treatment (Wk 1)	Treatment	Feed Day	No	03:10	03:00 - 03:59	Early Morning	37,5	51	25,2	25,2	18,65	541,20	108,22
2019/05/21	CH2206	Male	Three y/o	Treatment (Wk 1)	Treatment	Feed Day	No	03:10	03:00 - 03:59	Early Morning	37,4	41	20,0	20,0	18,55	522,29	100,31
2019/05/21	CH2205	Male	Three y/o	Treatment (Wk 1)	Treatment	Feed Day	No	03:15	03:00 - 03:59	Early Morning	37,5	50	15,0	15,0	18,65	539,52	90,51
2019/05/21	CH2206	Male	Three y/o	Treatment (Wk 1)	Treatment	Feed Day	No	03:15	03:00 - 03:59	Early Morning	37,3	46	15,4	15,4	18,46	532,38	91,41
2019/05/21	CH2205	Male	Three y/o	Treatment (Wk 1)	Treatment	Feed Day	No	03:20	03:00 - 03:59	Early Morning	37,6	82	12,6	12,6	18,75	579,19	84,59
2019/05/21	CH2206	Male	Three y/o	Treatment (Wk 1)	Treatment	Feed Day	No	03:20	03:00 - 03:59	Early Morning	37,4	75	15,0	15,0	18,55	572,36	90,51
2019/05/21	CH2205	Male	Three y/o	Treatment (Wk 1)	Treatment	Feed Day	No	03:25	03:00 - 03:59	Early Morning	37,6	61	9,8	9,8	18,75	556,01	76,07
2019/05/21	CH2206	Male	Three y/o	Treatment (Wk 1)	Treatment	Feed Day	No	03:25	03:00 - 03:59	Early Morning	37,3	88	14,6	14,6	18,46	584,49	89,59
2019/05/21	CH2205	Male	Three y/o	Treatment (Wk 1)	Treatment	Feed Day	No	03:30	03:00 - 03:59	Early Morning	37,6	67	9,2	9,2	18,75	563,53	73,94
2019/05/21	CH2206	Male	Three y/o	Treatment (Wk 1)	Treatment	Feed Day	No	03:30	03:00 - 03:59	Early Morning	37,4	86	11,2	11,2	18,55	582,77	80,59
2019/05/21	CH2205	Male	Three y/o	Treatment (Wk 1)	Treatment	Feed Day	No	03:35	03:00 - 03:59	Early Morning	37,6	53	8,2	8,2	18,75	544,43	70,05
2019/05/21	CH2206	Male	Three y/o	Treatment (Wk 1)	Treatment	Feed Day	No	03:35	03:00 - 03:59	Early Morning	37,4	47	8,2	8,2	18,55	534,23	70,05
2019/05/21	CH2205	Male	Three y/o	Treatment (Wk 1)	Treatment	Feed Day	No	03:40	03:00 - 03:59	Early Morning	37,6	82	7,2	7,2	18,75	579,19	65,66
2019/05/21	CH2206	Male	Three y/o	Treatment (Wk 1)	Treatment	Feed Day	No	03:40	03:00 - 03:59	Early Morning	37,4	55	4,2	4,2	18,55	547,52	47,57
2019/05/21	CH2205	Male	Three y/o	Treatment (Wk 1)	Treatment	Feed Day	No	03:45	03:00 - 03:59	Early Morning	37,6	46	11,0	11,0	18,75	532,38	79,98
2019/05/21	CH2206	Male	Three y/o	Treatment (Wk 1)	Treatment	Feed Day	No	03:45	03:00 - 03:59	Early Morning	37,4	60	5,4	5,4	18,55	554,67	55,99
2019/05/21	CH2205	Male	Three y/o	Treatment (Wk 1)	Treatment	Feed Day	No	03:50	03:00 - 03:59	Early Morning	37,6	54	17,2	17,2	18,75	545,99	95,17
2019/05/21	CH2206	Male	Three y/o	Treatment (Wk 1)	Treatment	Feed Day	No	03:50	03:00 - 03:59	Early Morning	37,4	97	146,8	146,8	18,55	591,66	169,26
2019/05/21	CH2205	Male	Three y/o	Treatment (Wk 1)	Treatment	Feed Day	No	03:55	03:00 - 03:59	Early Morning	37,6	88	34,6	34,6	18,75	584,49	119,10
2019/05/21	CH2206	Male	Three y/o	Treatment (Wk 1)	Treatment	Feed Day	No	03:55	03:00 - 03:59	Early Morning	37,4	123	226,6	226,6	18,55	608,49	184,52
2019/05/21	CH2205	Male	Three y/o	Treatment (Wk 1)	Treatment	Feed Day	No	04:00	04:00 - 04:59	Morning	37,5	120	67,0	67,0	18,65	606,78	141,92
2019/05/21	CH2206	Male	Three y/o	Treatment (Wk 1)	Treatment	Feed Day	No	04:00	04:00 - 04:59	Morning	37,4	114	154,4	154,4	18,55	603,20	171,03
2019/05/21	CH2205	Male	Three y/o	Treatment (Wk 1)	Treatment	Feed Day	No	04:05	04:00 - 04:59	Morning	37,5	131	49,8	49,8	18,65	612,80	131,65
2019/05/21	CH2206	Male	Three y/o	Treatment (Wk 1)	Treatment	Feed Day	No	04:05	04:00 - 04:59	Morning	37,4	138	59,8	59,8	18,55	616,31	137,98
2019/05/21	CH2205	Male	Three y/o	Treatment (Wk 1)	Treatment	Feed Day	No	04:10	04:00 - 04:59	Morning	37,4	111	76,4	76,4	18,55	601,33	146,48
2019/05/21	CH2206	Male	Three y/o	Treatment (Wk 1)	Treatment	Feed Day	No	04:10	04:00 - 04:59	Morning	37,4	125	60,2	60,2	18,55	609,60	138,21
2019/05/21	CH2205	Male	Three y/o	Treatment (Wk 1)	Treatment	Feed Day	No	04:15	04:00 - 04:59	Morning	37,5	79	208,8	208,8	18,65	576,36	181,63
2019/05/21	CH2206	Male	Three y/o	Treatment (Wk 1)	Treatment	Feed Day	No	04:15	04:00 - 04:59	Morning	37,3	131	229,4	229,4	18,46	612,80	184,95
2019/05/21	CH2205	Male	Three y/o	Treatment (Wk 1)	Treatment	Feed Day	No	04:20	04:00 - 04:59	Morning	37,5	56	167,0	167,0	18,65	549,01	173,78
2019/05/21	CH2206	Male	Three y/o	Treatment (Wk 1)	Treatment	Feed Day	No	04:20	04:00 - 04:59	Morning	37,4	145	103,4	103,4	18,55	619,62	157,01
2019/05/21	CH2205	Male	Three y/o	Treatment (Wk 1)	Treatment	Feed Day	No	04:25	04:00 - 04:59	Morning	37,5	128	82,4	82,4	18,65	611,22	149,10
2019/05/21	CH2206	Male	Three y/o	Treatment (Wk 1)	Treatment	Feed Day	No	04:25	04:00 - 04:59	Morning	37,5	135	116,8	116,8	18,65	614,84	161,26
2019/05/21	CH2205	Male	Three y/o	Treatment (Wk 1)	Treatment	Feed Day	No	04:30	04:00 - 04:59	Morning	37,5	84	147,8	147,8	18,65	581,01	169,50
2019/05/21	CH2206	Male	Three y/o	Treatment (Wk 1)	Treatment	Feed Day	No	04:30	04:00 - 04:59	Morning	37,6	114	224,8	224,8	18,75	603,20	184,23
2019/05/21	CH2205	Male	Three y/o	Treatment (Wk 1)	Treatment	Feed Day	No	04:35	04:00 - 04:59	Morning	37,6		62,6	62,6	18,75		139,56

2019/05/21	CH2206	Male	Three y/o	Treatment (Wk 1)	Treatment	Feed Day	No	04:35	04:00 - 04:59	Morning	37,7	106	61,0	61,0	18,85	598,06	138,67
2019/05/21	CH2205	Male	Three y/o	Treatment (Wk 1)	Treatment	Feed Day	No	04:40	04:00 - 04:59	Morning	37,6	60	200,6	200,6	18,75	554,67	180,22
2019/05/21	CH2206	Male	Three y/o	Treatment (Wk 1)	Treatment	Feed Day	No	04:40	04:00 - 04:59	Morning	37,7	110	347,0	347,0	18,85	600,69	199,57
2019/05/21	CH2205	Male	Three y/o	Treatment (Wk 1)	Treatment	Feed Day	No	04:45	04:00 - 04:59	Morning	37,7		72,0	72,0	18,85		144,42
2019/05/21	CH2206	Male	Three y/o	Treatment (Wk 1)	Treatment	Feed Day	No	04:45	04:00 - 04:59	Morning	37,7		98,2	98,2	18,85		155,21
2019/05/21	CH2205	Male	Three y/o	Treatment (Wk 1)	Treatment	Feed Day	No	04:50	04:00 - 04:59	Morning	37,7	141	211,6	211,6	18,85	617,75	182,10
2019/05/21	CH2206	Male	Three y/o	Treatment (Wk 1)	Treatment	Feed Day	No	04:50	04:00 - 04:59	Morning	37,7	128	174,2	174,2	18,85	611,22	175,26
2019/05/21	CH2205	Male	Three y/o	Treatment (Wk 1)	Treatment	Feed Day	No	04:55	04:00 - 04:59	Morning	37,7	104	94,8	94,8	18,85	596,69	153,98
2019/05/21	CH2206	Male	Three y/o	Treatment (Wk 1)	Treatment	Feed Day	No	04:55	04:00 - 04:59	Morning	37,7	131	219,0	219,0	18,85	612,80	183,31
2019/05/21	CH2205	Male	Three y/o	Treatment (Wk 1)	Treatment	Feed Day	No	05:00	05:00 - 05:59	Morning	37,7	111	25,0	25,0	18,85	601,33	107,94
2019/05/21	CH2206	Male	Three y/o	Treatment (Wk 1)	Treatment	Feed Day	No	05:00	05:00 - 05:59	Morning	37,7	119	9,0	9,0	18,85	606,20	73,19
2019/05/21	CH2205	Male	Three y/o	Treatment (Wk 1)	Treatment	Feed Day	No	05:05	05:00 - 05:59	Morning	37,6	35	64,4	64,4	18,75	507,97	140,55
2019/05/21	CH2206	Male	Three y/o	Treatment (Wk 1)	Treatment	Feed Day	No	05:05	05:00 - 05:59	Morning	37,7	131	339,2	339,2	18,85	612,80	198,77
2019/05/21	CH2205	Male	Three y/o	Treatment (Wk 1)	Treatment	Feed Day	No	05:10	05:00 - 05:59	Morning	37,5	96	141,4	141,4	18,65	590,91	167,95
2019/05/21	CH2206	Male	Three y/o	Treatment (Wk 1)	Treatment	Feed Day	No	05:10	05:00 - 05:59	Morning	37,7		182,6	182,6	18,85		176,92
2019/05/21	CH2205	Male	Three y/o	Treatment (Wk 1)	Treatment	Feed Day	No	05:15	05:00 - 05:59	Morning	37,6	94	27,8	27,8	18,75	589,37	111,58
2019/05/21	CH2206	Male	Three y/o	Treatment (Wk 1)	Treatment	Feed Day	No	05:15	05:00 - 05:59	Morning	37,7	124	60,8	60,8	18,85	609,04	138,55
2019/05/21	CH2205	Male	Three y/o	Treatment (Wk 1)	Treatment	Feed Day	No	05:20	05:00 - 05:59	Morning	37,5	51	210,0	210,0	18,65	541,20	181,84
2019/05/21	CH2206	Male	Three y/o	Treatment (Wk 1)	Treatment	Feed Day	No	05:20	05:00 - 05:59	Morning	37,8	126	57,4	57,4	18,95	610,14	136,56
2019/05/21	CH2205	Male	Three y/o	Treatment (Wk 1)	Treatment	Feed Day	No	05:25	05:00 - 05:59	Morning	37,6	138	326,4	326,4	18,75	616,31	197,41
2019/05/21	CH2206	Male	Three y/o	Treatment (Wk 1)	Treatment	Feed Day	No	05:25	05:00 - 05:59	Morning	37,8	174	30,0	30,0	18,95	631,46	114,20
2019/05/21	CH2205	Male	Three y/o	Treatment (Wk 1)	Treatment	Feed Day	No	05:30	05:00 - 05:59	Morning	37,6	124	69,2	69,2	18,75	609,04	143,04
2019/05/21	CH2206	Male	Three y/o	Treatment (Wk 1)	Treatment	Feed Day	No	05:30	05:00 - 05:59	Morning	37,9	141	143,2	143,2	19,05	617,75	168,39
2019/05/21	CH2205	Male	Three y/o	Treatment (Wk 1)	Treatment	Feed Day	No	05:35	05:00 - 05:59	Morning	37,4	74	10,2	10,2	18,55	571,33	77,43
2019/05/21	CH2206	Male	Three y/o	Treatment (Wk 1)	Treatment	Feed Day	No	05:35	05:00 - 05:59	Morning	37,8	107	25,0	25,0	18,95	598,73	107,94
2019/05/21	CH2205	Male	Three y/o	Treatment (Wk 1)	Treatment	Feed Day	No	05:40	05:00 - 05:59	Morning	37,4		25,6	25,6	18,55		108,76
2019/05/21	CH2206	Male	Three y/o	Treatment (Wk 1)	Treatment	Feed Day	No	05:40	05:00 - 05:59	Morning	37,4	103	70,4	70,4	18,55	596,00	143,64
2019/05/21	CH2205	Male	Three y/o	Treatment (Wk 1)	Treatment	Feed Day	No	05:45	05:00 - 05:59	Morning	37,4		36,0	36,0	18,55		120,46
2019/05/21	CH2206	Male	Three y/o	Treatment (Wk 1)	Treatment	Feed Day	No	05:45	05:00 - 05:59	Morning	37,4	98	125,6	125,6	18,55	592,41	163,80
2019/05/21	CH2205	Male	Three y/o	Treatment (Wk 1)	Treatment	Feed Day	No	05:50	05:00 - 05:59	Morning	37,5	152	248,0	248,0	18,65	622,73	187,70
2019/05/21	CH2206	Male	Three y/o	Treatment (Wk 1)	Treatment	Feed Day	No	05:50	05:00 - 05:59	Morning	37,6	128	148,8	148,8	18,75	611,22	169,74
2019/05/21	CH2205	Male	Three y/o	Treatment (Wk 1)	Treatment	Feed Day	No	05:55	05:00 - 05:59	Morning	37,7	60	87,2	87,2	18,85	554,67	151,07
2019/05/21	CH2206	Male	Three y/o	Treatment (Wk 1)	Treatment	Feed Day	No	05:55	05:00 - 05:59	Morning	37,7	124	314,6	314,6	18,85	609,04	196,10
2019/05/21	CH2205	Male	Three y/o	Treatment (Wk 1)	Treatment	Feed Day	No	06:00	06:00 - 06:59	Morning	37,6	111	101,8	101,8	18,75	601,33	156,47
2019/05/21	CH2206	Male	Three y/o	Treatment (Wk 1)	Treatment	Feed Day	No	06:00	06:00 - 06:59	Morning	37,7	115	89,0	89,0	18,85	603,82	151,78
2019/05/21	CH2205	Male	Three y/o	Treatment (Wk 1)	Treatment	Feed Day	No	06:05	06:00 - 06:59	Morning	37,6	53	90,8	90,8	18,75	544,43	152,48
2019/05/21	CH2206	Male	Three y/o	Treatment (Wk 1)	Treatment	Feed Day	No	06:05	06:00 - 06:59	Morning	37,4	118	124,0	124,0	18,55	605,61	163,35
2019/05/21	CH2205	Male	Three y/o	Treatment (Wk 1)	Treatment	Feed Day	No	06:10	06:00 - 06:59	Morning	37,5	56	243,6	243,6	18,65	549,01	187,07
2019/05/21	CH2206	Male	Three y/o	Treatment (Wk 1)	Treatment	Feed Day	No	06:10	06:00 - 06:59	Morning	37,5	118	75,0	75,0	18,65	605,61	145,83
2019/05/21	CH2205	Male	Three y/o	Treatment (Wk 1)	Treatment	Feed Day	No	06:15	06:00 - 06:59	Morning	37,5	71	43,4	43,4	18,65	568,10	126,90
2019/05/21	CH2206	Male	Three y/o	Treatment (Wk 1)	Treatment	Feed Day	No	06:15	06:00 - 06:59	Morning	37,5		151,8	151,8	18,65		170,44
2019/05/21	CH2205	Male	Three y/o	Treatment (Wk 1)	Treatment	Feed Day	No	06:20	06:00 - 06:59	Morning	37,5	115	110,6	110,6	18,65	603,82	159,36
2019/05/21	CH2206	Male	Three y/o	Treatment (Wk 1)	Treatment	Feed Day	No	06:20	06:00 - 06:59	Morning	37,6	140	61,6	61,6	18,75	617,28	139,00
2019/05/21	CH2205	Male	Three y/o	Treatment (Wk 1)	Treatment	Feed Day	No	06:25	06:00 - 06:59	Morning	37,6	106	106,2	106,2	18,75	598,06	157,94
2019/05/21	CH2206	Male	Three y/o	Treatment (Wk 1)	Treatment	Feed Day	No	06:25	06:00 - 06:59	Morning	37,7	54	215,2	215,2	18,85	545,99	182,70
2019/05/21	CH2205	Male	Three y/o	Treatment (Wk 1)	Treatment	Feed Day	No	06:30	06:00 - 06:59	Morning	37,5	103	137,6	137,6	18,65	596,00	166,99
2019/05/21	CH2206	Male	Three y/o	Treatment (Wk 1)	Treatment	Feed Day	No	06:30	06:00 - 06:59	Morning	37,7	121	80,0	80,0	18,85	607,35	148,08
2019/05/21	CH2205	Male	Three y/o	Treatment (Wk 1)	Treatment	Feed Day	No	06:35	06:00 - 06:59	Morning	37,5	52	44,6	44,6	18,65	542,83	127,84
2019/05/21	CH2206	Male	Three y/o	Treatment (Wk 1)	Treatment	Feed Day	No	06:35	06:00 - 06:59	Morning	37,7	108	289,2	289,2	18,85	599,39	193,12
2019/05/21	CH2205	Male	Three y/o	Treatment (Wk 1)	Treatment	Feed Day	No	06:40	06:00 - 06:59	Morning	37,4		30,6	30,6	18,55		114,87
2019/05/21	CH2206	Male	Three y/o	Treatment (Wk 1)	Treatment	Feed Day	No	06:40	06:00 - 06:59	Morning	37,7	113	94,6	94,6	18,85	602,58	153,91
2019/05/21	CH2205	Male	Three y/o	Treatment (Wk 1)	Treatment	Feed Day	No	06:45	06:00 - 06:59	Morning	37,1	124	27,0	27,0	18,26	609,04	110,58
2019/05/21	CH2206	Male	Three y/o	Treatment (Wk 1)	Treatment	Feed Day	No	06:45	06:00 - 06:59	Morning	37,7	111	388,6	388,6	18,85	601,33	203,59
2019/05/21	CH2205	Male	Three y/o	Treatment (Wk 1)	Treatment	Feed Day	No	06:50	06:00 - 06:59	Morning	37,0		25,6	25,6	18,16		108,76
2019/05/21	CH2206	Male	Three y/o	Treatment (Wk 1)	Treatment	Feed Day	No	06:50	06:00 - 06:59	Morning	37,7	117	93,2	93,2	18,85	605,02	153,39
2019/05/21	CH2205	Male	Three y/o	Treatment (Wk 1)	Treatment	Feed Day	No	06:55	06:00 - 06:59	Morning	37,1	61	27,2	27,2	18,26	556,01	110,83
2019/05/21	CH2206	Male	Three y/o	Treatment (Wk 1)	Treatment	Feed Day	No	06:55	06:00 - 06:59	Morning	37,7	126	201,4	201,4	18,85	610,14	180,36
2019/05/21	CH2205	Male	Three y/o	Treatment (Wk 1)	Treatment	Feed Day	No	07:00	07:00 - 07:59	Morning	37,3	54	24,4	24,4	18,46	545,99	107,11
2019/05/21	CH2206	Male	Three y/o	Treatment (Wk 1)	Treatment	Feed Day	No	07:00	07:00 - 07:59	Morning	37,7	108	100,0	100,0	18,85	599,39	155,84
2019/05/21	CH2205	Male	Three y/o	Treatment (Wk 1)	Treatment	Feed Day	No	07:05	07:00 - 07:59	Morning	37,2		20,2	20,2	18,36		100,65
2019/05/21	CH2206	Male	Three y/o	Treatment (Wk 1)	Treatment	Feed Day	No	07:05	07:00 - 07:59	Morning	37,7		170,6	170,6	18,85		174,53
2019/05/21	CH2205	Male	Three y/o	Treatment (Wk 1)	Treatment	Feed Day	No	07:10	07:00 - 07:59	Morning	37,2		77,6	77,6	18,36		147,02
2019/05/21	CH2206	Male	Three y/o	Treatment (Wk 1)	Treatment	Feed Day	No	07:10	07:00 - 07:59	Morning	37,7	131	102,8	102,8	18,85	612,80	156,81
2019/05/21	CH2205	Male	Three y/o	Treatment (Wk 1)	Treatment	Feed Day	No	07:15	07:00 - 07:59	Morning	37,3	102	37,8	37,8	18,46	595,30	122,14
2019/05/21	CH2206	Male	Three y/o	Treatment (Wk 1)	Treatment	Feed Day	No	07:15	07:00 - 07:59	Morning	37,7	134	90,0	90,0	18,85	614,33	152,17
2019/05/21	CH2205	Male	Three y/o	Treatment (Wk 1)	Treatment	Feed Day	No	07:20	07:00 - 07:59	Morning	37,3	52	92,8	92,8	18,46	542,83	153,24
2019/05/21	CH2206	Male	Three y/o	Treatment (Wk 1)	Treatment	Feed Day	No	07:20	07:00 - 07:59	Morning	37,7	112	142,2	142,2	18,85	601,96	168,15
2019/05/21	CH2205	Male	Three y/o	Treatment (Wk 1)	Treatment	Feed Day	No	07:25	07:00 - 07:59	Morning	37,3	98	148,8	148,8	18,46	592,41	169,74
2019/05/21	CH2206	Male	Three y/o	Treatment (Wk 1)	Treatment	Feed Day	No	07:25	07:00 - 07:59	Morning	37,7	121	25,8	25,8	18,85	607,35	109,02
2019/05/21	CH2205	Male	Three y/o	Treatment (Wk 1)	Treatment	Feed Day	No	07:30	07:00 - 07:59	Morning	37,4	83	157,6	157,6	18,55	580,11	171,75

2019/05/21	CH2206	Male	Three y/o	Treatment (Wk 1)	Treatment	Feed Day	No	07:30	07:00 - 07:59	Morning	37,7	106	120,2	120,2	18,85	598,06	162,27
2019/05/21	CH2205	Male	Three y/o	Treatment (Wk 1)	Treatment	Feed Day	No	07:35	07:00 - 07:59	Morning	37,5	169	108,8	108,8	18,65	629,60	158,79
2019/05/21	CH2206	Male	Three y/o	Treatment (Wk 1)	Treatment	Feed Day	No	07:35	07:00 - 07:59	Morning	37,7	124	801,8	801,8	18,85	609,04	229,41
2019/05/21	CH2205	Male	Three y/o	Treatment (Wk 1)	Treatment	Feed Day	No	07:40	07:00 - 07:59	Morning	37,9	152	63,2	63,2	19,05	622,73	139,89
2019/05/21	CH2206	Male	Three y/o	Treatment (Wk 1)	Treatment	Feed Day	No	07:40	07:00 - 07:59	Morning	37,8	140	231,8	231,8	18,95	617,28	185,31
2019/05/21	CH2205	Male	Three y/o	Treatment (Wk 1)	Treatment	Feed Day	No	07:45	07:00 - 07:59	Morning	37,7	75	73,4	73,4	18,85	572,36	145,08
2019/05/21	CH2206	Male	Three y/o	Treatment (Wk 1)	Treatment	Feed Day	No	07:45	07:00 - 07:59	Morning	37,8	102	94,6	94,6	18,95	595,30	153,91
2019/05/21	CH2205	Male	Three y/o	Treatment (Wk 1)	Treatment	Feed Day	No	07:50	07:00 - 07:59	Morning	37,6		60,8	60,8	18,75		138,55
2019/05/21	CH2206	Male	Three y/o	Treatment (Wk 1)	Treatment	Feed Day	No	07:50	07:00 - 07:59	Morning	37,8	105	108,4	108,4	18,95	597,38	158,66
2019/05/21	CH2205	Male	Three y/o	Treatment (Wk 1)	Treatment	Feed Day	No	07:55	07:00 - 07:59	Morning	37,7	91	106,0	106,0	18,85	586,98	157,88
2019/05/21	CH2206	Male	Three y/o	Treatment (Wk 1)	Treatment	Feed Day	No	07:55	07:00 - 07:59	Morning	37,8	58	145,6	145,6	18,95	551,90	168,97
2019/05/21	CH2205	Male	Three y/o	Treatment (Wk 1)	Treatment	Feed Day	No	08:00	08:00 - 08:59	Late Morning	37,6	147	78,2	78,2	18,75	620,52	147,28
2019/05/21	CH2206	Male	Three y/o	Treatment (Wk 1)	Treatment	Feed Day	No	08:00	08:00 - 08:59	Late Morning	37,7	54	246,8	246,8	18,85	545,99	187,53
2019/05/21	CH2205	Male	Three y/o	Treatment (Wk 1)	Treatment	Feed Day	No	08:05	08:00 - 08:59	Late Morning	37,5	84	111,6	111,6	18,65	581,01	159,67
2019/05/21	CH2206	Male	Three y/o	Treatment (Wk 1)	Treatment	Feed Day	No	08:05	08:00 - 08:59	Late Morning	37,4		266,8	266,8	18,55		190,28
2019/05/21	CH2205	Male	Three y/o	Treatment (Wk 1)	Treatment	Feed Day	No	08:10	08:00 - 08:59	Late Morning	37,5	99	200,4	200,4	18,65	593,14	180,19
2019/05/21	CH2206	Male	Three y/o	Treatment (Wk 1)	Treatment	Feed Day	No	08:10	08:00 - 08:59	Late Morning	37,5	160	100,6	100,6	18,65	626,07	156,05
2019/05/21	CH2205	Male	Three y/o	Treatment (Wk 1)	Treatment	Feed Day	No	08:15	08:00 - 08:59	Late Morning	37,4	84	31,4	31,4	18,55	581,01	115,76
2019/05/21	CH2206	Male	Three y/o	Treatment (Wk 1)	Treatment	Feed Day	No	08:15	08:00 - 08:59	Late Morning	37,4	58	47,6	47,6	18,55	551,90	130,09
2019/05/21	CH2205	Male	Three y/o	Treatment (Wk 1)	Treatment	Feed Day	No	08:20	08:00 - 08:59	Late Morning	37,0	185	30,2	30,2	18,16	635,34	114,42
2019/05/21	CH2206	Male	Three y/o	Treatment (Wk 1)	Treatment	Feed Day	No	08:20	08:00 - 08:59	Late Morning	37,0	69	47,6	47,6	18,16	565,86	130,09
2019/05/21	CH2205	Male	Three y/o	Treatment (Wk 1)	Treatment	Feed Day	No	08:25	08:00 - 08:59	Late Morning	36,7		34,0	34,0	17,87		118,49
2019/05/21	CH2206	Male	Three y/o	Treatment (Wk 1)	Treatment	Feed Day	No	08:25	08:00 - 08:59	Late Morning	36,8	77	41,4	41,4	17,96	574,39	125,27
2019/05/21	CH2205	Male	Three y/o	Treatment (Wk 1)	Treatment	Feed Day	No	08:30	08:00 - 08:59	Late Morning	36,8	72	29,8	29,8	17,96	569,20	113,97
2019/05/21	CH2206	Male	Three y/o	Treatment (Wk 1)	Treatment	Feed Day	No	08:30	08:00 - 08:59	Late Morning	36,6	48	30,0	30,0	17,77	536,04	114,20
2019/05/21	CH2205	Male	Three y/o	Treatment (Wk 1)	Treatment	Feed Day	No	08:35	08:00 - 08:59	Late Morning	37,0	64	29,6	29,6	18,16	559,88	113,73
2019/05/21	CH2206	Male	Three y/o	Treatment (Wk 1)	Treatment	Feed Day	No	08:35	08:00 - 08:59	Late Morning	36,8	90	23,4	23,4	17,96	586,16	105,68
2019/05/21	CH2205	Male	Three y/o	Treatment (Wk 1)	Treatment	Feed Day	No	08:40	08:00 - 08:59	Late Morning	37,1		35,0	35,0	18,26		119,49
2019/05/21	CH2206	Male	Three y/o	Treatment (Wk 1)	Treatment	Feed Day	No	08:40	08:00 - 08:59	Late Morning	36,7	85	37,4	37,4	17,87	581,90	121,77
2019/05/21	CH2205	Male	Three y/o	Treatment (Wk 1)	Treatment	Feed Day	No	08:45	08:00 - 08:59	Late Morning	37,2	96	46,8	46,8	18,36	590,91	129,50
2019/05/21	CH2206	Male	Three y/o	Treatment (Wk 1)	Treatment	Feed Day	No	08:45	08:00 - 08:59	Late Morning	36,6	113	84,8	84,8	17,77	602,58	150,10
2019/05/21	CH2205	Male	Three y/o	Treatment (Wk 1)	Treatment	Feed Day	No	08:50	08:00 - 08:59	Late Morning	37,2		109,2	109,2	18,36		158,91
2019/05/21	CH2206	Male	Three y/o	Treatment (Wk 1)	Treatment	Feed Day	No	08:50	08:00 - 08:59	Late Morning	37,0	131	83,6	83,6	18,16	612,80	149,61
2019/05/21	CH2205	Male	Three y/o	Treatment (Wk 1)	Treatment	Feed Day	No	08:55	08:00 - 08:59	Late Morning	37,4	101	175,2	175,2	18,55	594,59	175,47
2019/05/21	CH2206	Male	Three y/o	Treatment (Wk 1)	Treatment	Feed Day	No	08:55	08:00 - 08:59	Late Morning	37,2	107	221,4	221,4	18,36	598,73	183,70
2019/05/21	CH2205	Male	Three y/o	Treatment (Wk 1)	Treatment	Feed Day	No	09:00	09:00 - 09:59	Late Morning	37,4	135	100,4	100,4	18,55	614,84	155,98
2019/05/21	CH2206	Male	Three y/o	Treatment (Wk 1)	Treatment	Feed Day	No	09:00	09:00 - 09:59	Late Morning	37,3	108	77,4	77,4	18,46	599,39	146,93
2019/05/21	CH2205	Male	Three y/o	Treatment (Wk 1)	Treatment	Feed Day	No	09:05	09:00 - 09:59	Late Morning	37,4		83,0	83,0	18,55		149,36
2019/05/21	CH2206	Male	Three y/o	Treatment (Wk 1)	Treatment	Feed Day	No	09:05	09:00 - 09:59	Late Morning	37,4	110	266,8	266,8	18,55	600,69	190,28
2019/05/21	CH2205	Male	Three y/o	Treatment (Wk 1)	Treatment	Feed Day	No	09:10	09:00 - 09:59	Late Morning	37,5	57	79,8	79,8	18,65	550,47	147,99
2019/05/21	CH2206	Male	Three y/o	Treatment (Wk 1)	Treatment	Feed Day	No	09:10	09:00 - 09:59	Late Morning	37,5	99	52,0	52,0	18,65	593,14	133,14
2019/05/21	CH2205	Male	Three y/o	Treatment (Wk 1)	Treatment	Feed Day	No	09:15	09:00 - 09:59	Late Morning	37,5		164,6	164,6	18,65		173,27
2019/05/21	CH2206	Male	Three y/o	Treatment (Wk 1)	Treatment	Feed Day	No	09:15	09:00 - 09:59	Late Morning	37,7	119	326,8	326,8	18,85	606,20	197,45
2019/05/21	CH2205	Male	Three y/o	Treatment (Wk 1)	Treatment	Feed Day	No	09:20	09:00 - 09:59	Late Morning	37,5	172	28,2	28,2	18,65	630,73	112,07
2019/05/21	CH2206	Male	Three y/o	Treatment (Wk 1)	Treatment	Feed Day	No	09:20	09:00 - 09:59	Late Morning	37,7	108	304,6	304,6	18,85	599,39	194,96
2019/05/21	CH2205	Male	Three y/o	Treatment (Wk 1)	Treatment	Feed Day	No	09:25	09:00 - 09:59	Late Morning	37,6	185	14,4	14,4	18,75	635,34	89,12
2019/05/21	CH2206	Male	Three y/o	Treatment (Wk 1)	Treatment	Feed Day	No	09:25	09:00 - 09:59	Late Morning	37,7	104	131,8	131,8	18,85	596,69	165,49
2019/05/21	CH2205	Male	Three y/o	Treatment (Wk 1)	Treatment	Feed Day	No	09:30	09:00 - 09:59	Late Morning	37,7	63	56,2	56,2	18,85	558,62	135,83
2019/05/21	CH2206	Male	Three y/o	Treatment (Wk 1)	Treatment	Feed Day	No	09:30	09:00 - 09:59	Late Morning	37,8	109	85,6	85,6	18,95	600,04	150,43
2019/05/21	CH2205	Male	Three y/o	Treatment (Wk 1)	Treatment	Feed Day	No	09:35	09:00 - 09:59	Late Morning	37,5	65	24,4	24,4	18,65	561,12	107,11
2019/05/21	CH2206	Male	Three y/o	Treatment (Wk 1)	Treatment	Feed Day	No	09:35	09:00 - 09:59	Late Morning	37,7	45	43,6	43,6	18,85	530,47	127,06
2019/05/21	CH2205	Male	Three y/o	Treatment (Wk 1)	Treatment	Feed Day	No	09:40	09:00 - 09:59	Late Morning	37,3		24,0	24,0	18,46		106,55
2019/05/21	CH2206	Male	Three y/o	Treatment (Wk 1)	Treatment	Feed Day	No	09:40	09:00 - 09:59	Late Morning	37,8	72	31,8	31,8	18,95	569,20	116,20
2019/05/21	CH2205	Male	Three y/o	Treatment (Wk 1)	Treatment	Feed Day	No	09:45	09:00 - 09:59	Late Morning	37,4	87	11,6	11,6	18,55	583,64	81,78
2019/05/21	CH2206	Male	Three y/o	Treatment (Wk 1)	Treatment	Feed Day	No	09:45	09:00 - 09:59	Late Morning	37,8	45	20,4	20,4	18,95	530,47	100,99
2019/05/21	CH2205	Male	Three y/o	Treatment (Wk 1)	Treatment	Feed Day	No	09:50	09:00 - 09:59	Late Morning	37,4		7,4	7,4	18,55		66,59
2019/05/21	CH2206	Male	Three y/o	Treatment (Wk 1)	Treatment	Feed Day	No	09:50	09:00 - 09:59	Late Morning	37,9	47	34,2	34,2	19,05	534,23	118,70
2019/05/21	CH2205	Male	Three y/o	Treatment (Wk 1)	Treatment	Feed Day	No	09:55	09:00 - 09:59	Late Morning	37,5	49	5,8	5,8	18,65	537,80	58,39
2019/05/21	CH2206	Male	Three y/o	Treatment (Wk 1)	Treatment	Feed Day	No	09:55	09:00 - 09:59	Late Morning	37,9	51	37,2	37,2	19,05	541,20	121,59
2019/05/21	CH2205	Male	Three y/o	Treatment (Wk 1)	Treatment	Feed Day	No	10:00	10:00 - 10:59	Late Morning	37,5	40	16,4	16,4	18,65	520,09	93,55
2019/05/21	CH2206	Male	Three y/o	Treatment (Wk 1)	Treatment	Feed Day	No	10:00	10:00 - 10:59	Late Morning	37,8	53	35,8	35,8	18,95	544,43	120,27
2019/05/21	CH2205	Male	Three y/o	Treatment (Wk 1)	Treatment	Feed Day	No	10:05	10:00 - 10:59	Late Morning	37,6		20,8	20,8	18,75		101,65
2019/05/21	CH2206	Male	Three y/o	Treatment (Wk 1)	Treatment	Feed Day	No	10:05	10:00 - 10:59	Late Morning	37,8	48	37,0	37,0	18,95	536,04	121,40
2019/05/21	CH2205	Male	Three y/o	Treatment (Wk 1)	Treatment	Feed Day	No	10:10	10:00 - 10:59	Late Morning	37,6	82	6,6	6,6	18,75	579,19	62,73
2019/05/21	CH2206	Male	Three y/o	Treatment (Wk 1)	Treatment	Feed Day	No	10:10	10:00 - 10:59	Late Morning	37,8	49	27,2	27,2	18,95	537,80	110,83
2019/05/21	CH2205	Male	Three y/o	Treatment (Wk 1)	Treatment	Feed Day	No	10:15	10:00 - 10:59	Late Morning	37,6	73	12,4	12,4	18,75	570,27	84,04
2019/05/21	CH2206	Male	Three y/o	Treatment (Wk 1)	Treatment	Feed Day	No	10:15	10:00 - 10:59	Late Morning	37,8	50	35,2	35,2	18,95	539,52	119,69
2019/05/21	CH2205	Male	Three y/o	Treatment (Wk 1)	Treatment	Feed Day	No	10:20	10:00 - 10:59	Late Morning	37,5	49	30,0	30,0	18,65	537,80	114,20
2019/05/21	CH2206	Male	Three y/o	Treatment (Wk 1)	Treatment	Feed Day	No	10:20	10:00 - 10:59	Late Morning	37,8	48	34,4	34,4	18,95	536,04	118,90
2019/05/21	CH2205	Male	Three y/o	Treatment (Wk 1)	Treatment	Feed Day	No	10:25	10:00 - 10:59	Late Morning	37,6	64	8,6	8,6	18,75	559,88	71,66

2019/05/21	CH2206	Male	Three y/o	Treatment (Wk 1)	Treatment	Feed Day	No	10:25	10:00 - 10:59	Late Morning	37,7	57	35,8	35,8	18,85	550,47	120,27
2019/05/21	CH2205	Male	Three y/o	Treatment (Wk 1)	Treatment	Feed Day	Yes	10:30	10:00 - 10:59	Late Morning	37,6	69	20,0	20,0	18,75	565,86	100,31
2019/05/21	CH2206	Male	Three y/o	Treatment (Wk 1)	Treatment	Feed Day	Yes	10:30	10:00 - 10:59	Late Morning	37,7	77	45,8	45,8	18,85	574,39	128,76
2019/05/21	CH2205	Male	Three y/o	Treatment (Wk 1)	Treatment	Feed Day	Yes	10:35	10:00 - 10:59	Late Morning	37,6	68	12,4	12,4	18,75	564,71	84,04
2019/05/21	CH2206	Male	Three y/o	Treatment (Wk 1)	Treatment	Feed Day	Yes	10:35	10:00 - 10:59	Late Morning	37,7	47	33,8	33,8	18,85	534,23	118,29
2019/05/21	CH2205	Male	Three y/o	Treatment (Wk 1)	Treatment	Feed Day	Yes	10:40	10:00 - 10:59	Late Morning	37,6	61	7,4	7,4	18,75	556,01	66,59
2019/05/21	CH2206	Male	Three y/o	Treatment (Wk 1)	Treatment	Feed Day	Yes	10:40	10:00 - 10:59	Late Morning	37,7	46	37,6	37,6	18,85	532,38	121,96
2019/05/21	CH2205	Male	Three y/o	Treatment (Wk 1)	Treatment	Feed Day	Yes	10:45	10:00 - 10:59	Late Morning	37,5	51	54,4	54,4	18,65	541,20	134,70
2019/05/21	CH2206	Male	Three y/o	Treatment (Wk 1)	Treatment	Feed Day	Yes	10:45	10:00 - 10:59	Late Morning	37,7	53	58,4	58,4	18,85	544,43	137,16
2019/05/21	CH2205	Male	Three y/o	Treatment (Wk 1)	Treatment	Feed Day	Yes	10:50	10:00 - 10:59	Late Morning	37,5	156	157,8	157,8	18,65	624,43	171,79
2019/05/21	CH2206	Male	Three y/o	Treatment (Wk 1)	Treatment	Feed Day	Yes	10:50	10:00 - 10:59	Late Morning	37,5	98	183,6	183,6	18,65	592,41	177,11
2019/05/21	CH2205	Male	Three y/o	Treatment (Wk 1)	Treatment	Feed Day	Yes	10:55	10:00 - 10:59	Late Morning	37,5		103,6	103,6	18,65		157,08
2019/05/21	CH2206	Male	Three y/o	Treatment (Wk 1)	Treatment	Feed Day	Yes	10:55	10:00 - 10:59	Late Morning	37,5	101	149,2	149,2	18,65	594,59	169,83
2019/05/21	CH2205	Male	Three y/o	Treatment (Wk 1)	Treatment	Feed Day	Yes	11:00	11:00 - 11:59	Late Morning	37,5	104	90,2	90,2	18,65	596,69	152,25
2019/05/21	CH2206	Male	Three y/o	Treatment (Wk 1)	Treatment	Feed Day	Yes	11:00	11:00 - 11:59	Late Morning	37,5		49,2	49,2	18,65		131,23
2019/05/21	CH2205	Male	Three y/o	Treatment (Wk 1)	Treatment	Feed Day	Yes	11:05	11:00 - 11:59	Late Morning	37,6	103	71,8	71,8	18,75	596,00	144,32
2019/05/21	CH2206	Male	Three y/o	Treatment (Wk 1)	Treatment	Feed Day	Yes	11:05	11:00 - 11:59	Late Morning	37,6		191,6	191,6	18,75		178,61
2019/05/21	CH2205	Male	Three y/o	Treatment (Wk 1)	Treatment	Feed Day	Yes	11:10	11:00 - 11:59	Late Morning	37,7		309,2	309,2	18,85		195,49
2019/05/21	CH2206	Male	Three y/o	Treatment (Wk 1)	Treatment	Feed Day	Yes	11:10	11:00 - 11:59	Late Morning	37,7	107	90,6	90,6	18,85	598,73	152,40
2019/05/21	CH2205	Male	Three y/o	Treatment (Wk 1)	Treatment	Feed Day	Yes	11:15	11:00 - 11:59	Late Morning	37,7	57	37,4	37,4	18,85	550,47	121,77
2019/05/21	CH2206	Male	Three y/o	Treatment (Wk 1)	Treatment	Feed Day	Yes	11:15	11:00 - 11:59	Late Morning	37,7	111	115,6	115,6	18,85	601,33	160,90
2019/05/21	CH2205	Male	Three y/o	Treatment (Wk 1)	Treatment	Feed Day	Yes	11:20	11:00 - 11:59	Late Morning	37,9	99	36,2	36,2	19,05	593,14	120,65
2019/05/21	CH2206	Male	Three y/o	Treatment (Wk 1)	Treatment	Feed Day	Yes	11:20	11:00 - 11:59	Late Morning	37,7	106	99,2	99,2	18,85	598,06	155,56
2019/05/21	CH2205	Male	Three y/o	Treatment (Wk 1)	Treatment	Feed Day	Yes	11:25	11:00 - 11:59	Late Morning	37,7		46,6	46,6	18,85		129,35
2019/05/21	CH2206	Male	Three y/o	Treatment (Wk 1)	Treatment	Feed Day	Yes	11:25	11:00 - 11:59	Late Morning	37,7	92	30,8	30,8	18,85	587,78	115,10
2019/05/21	CH2205	Male	Three y/o	Treatment (Wk 1)	Treatment	Feed Day	No	11:30	11:00 - 11:59	Late Morning	37,7	118	131,6	131,6	18,85	605,61	165,43
2019/05/21	CH2206	Male	Three y/o	Treatment (Wk 1)	Treatment	Feed Day	No	11:30	11:00 - 11:59	Late Morning	37,7	109	50,2	50,2	18,85	600,04	131,92
2019/05/21	CH2205	Male	Three y/o	Treatment (Wk 1)	Treatment	Feed Day	No	11:35	11:00 - 11:59	Late Morning	37,9	57	39,8	39,8	19,05	550,47	123,91
2019/05/21	CH2206	Male	Three y/o	Treatment (Wk 1)	Treatment	Feed Day	No	11:35	11:00 - 11:59	Late Morning	37,5	49	34,8	34,8	18,65	537,80	119,29
2019/05/21	CH2205	Male	Three y/o	Treatment (Wk 1)	Treatment	Feed Day	No	11:40	11:00 - 11:59	Late Morning	38,0	102	187,6	187,6	19,15	595,30	177,87
2019/05/21	CH2206	Male	Three y/o	Treatment (Wk 1)	Treatment	Feed Day	No	11:40	11:00 - 11:59	Late Morning	37,7	86	96,0	96,0	18,85	582,77	154,42
2019/05/21	CH2205	Male	Three y/o	Treatment (Wk 1)	Treatment	Feed Day	No	11:45	11:00 - 11:59	Late Morning	38,0	98	72,2	72,2	19,15	592,41	144,51
2019/05/21	CH2206	Male	Three y/o	Treatment (Wk 1)	Treatment	Feed Day	No	11:45	11:00 - 11:59	Late Morning	37,8	111	43,6	43,6	18,95	601,33	127,06
2019/05/21	CH2205	Male	Three y/o	Treatment (Wk 1)	Treatment	Feed Day	No	11:50	11:00 - 11:59	Late Morning	38,0	86	32,0	32,0	19,15	582,77	116,41
2019/05/21	CH2206	Male	Three y/o	Treatment (Wk 1)	Treatment	Feed Day	No	11:50	11:00 - 11:59	Late Morning	37,8	101	43,2	43,2	18,95	594,59	126,74
2019/05/21	CH2205	Male	Three y/o	Treatment (Wk 1)	Treatment	Feed Day	No	11:55	11:00 - 11:59	Late Morning	37,9	104	168,2	168,2	19,05	596,69	174,03
2019/05/21	CH2206	Male	Three y/o	Treatment (Wk 1)	Treatment	Feed Day	No	11:55	11:00 - 11:59	Late Morning	37,8	75	236,0	236,0	18,95	572,36	185,95
2019/05/21	CH2205	Male	Three y/o	Treatment (Wk 1)	Treatment	Feed Day	No	12:00	12:00 - 12:59	Afternoon	37,9	135	17,6	17,6	19,05	614,84	95,95
2019/05/21	CH2206	Male	Three y/o	Treatment (Wk 1)	Treatment	Feed Day	No	12:00	12:00 - 12:59	Afternoon	37,8	109	116,4	116,4	18,95	600,04	161,14
2019/05/21	CH2205	Male	Three y/o	Treatment (Wk 1)	Treatment	Feed Day	No	12:05	12:00 - 12:59	Afternoon	38,0	70	17,2	17,2	19,15	566,99	95,17
2019/05/21	CH2206	Male	Three y/o	Treatment (Wk 1)	Treatment	Feed Day	No	12:05	12:00 - 12:59	Afternoon	37,9	138	135,6	135,6	19,05	616,31	166,48
2019/05/21	CH2205	Male	Three y/o	Treatment (Wk 1)	Treatment	Feed Day	No	12:10	12:00 - 12:59	Afternoon	38,0	87	10,2	10,2	19,15	583,64	77,43
2019/05/21	CH2206	Male	Three y/o	Treatment (Wk 1)	Treatment	Feed Day	No	12:10	12:00 - 12:59	Afternoon	37,9	99	18,0	18,0	19,05	593,14	96,72
2019/05/21	CH2205	Male	Three y/o	Treatment (Wk 1)	Treatment	Feed Day	No	12:15	12:00 - 12:59	Afternoon	38,0	67	8,4	8,4	19,15	563,53	70,86
2019/05/21	CH2206	Male	Three y/o	Treatment (Wk 1)	Treatment	Feed Day	No	12:15	12:00 - 12:59	Afternoon	37,8	89	24,4	24,4	18,95	585,33	107,11
2019/05/21	CH2205	Male	Three y/o	Treatment (Wk 1)	Treatment	Feed Day	No	12:20	12:00 - 12:59	Afternoon	38,0	64	34,4	34,4	19,15	559,88	118,90
2019/05/21	CH2206	Male	Three y/o	Treatment (Wk 1)	Treatment	Feed Day	No	12:20	12:00 - 12:59	Afternoon	37,8	73	44,6	44,6	18,95	570,27	127,84
2019/05/21	CH2205	Male	Three y/o	Treatment (Wk 1)	Treatment	Feed Day	No	12:25	12:00 - 12:59	Afternoon	38,0	111	95,2	95,2	19,15	601,33	154,13
2019/05/21	CH2206	Male	Three y/o	Treatment (Wk 1)	Treatment	Feed Day	No	12:25	12:00 - 12:59	Afternoon	37,8	63	33,2	33,2	18,95	558,62	117,68
2019/05/21	CH2205	Male	Three y/o	Treatment (Wk 1)	Treatment	Feed Day	No	12:30	12:00 - 12:59	Afternoon	38,0	62	14,0	14,0	19,15	557,33	88,17
2019/05/21	CH2206	Male	Three y/o	Treatment (Wk 1)	Treatment	Feed Day	No	12:30	12:00 - 12:59	Afternoon	37,9	71	26,6	26,6	19,05	568,10	110,07
2019/05/21	CH2205	Male	Three y/o	Treatment (Wk 1)	Treatment	Feed Day	No	12:35	12:00 - 12:59	Afternoon	38,0	77	5,6	5,6	19,15	574,39	57,21
2019/05/21	CH2206	Male	Three y/o	Treatment (Wk 1)	Treatment	Feed Day	No	12:35	12:00 - 12:59	Afternoon	37,9	80	28,0	28,0	19,05	577,31	111,83
2019/05/21	CH2205	Male	Three y/o	Treatment (Wk 1)	Treatment	Feed Day	No	12:40	12:00 - 12:59	Afternoon	38,0	78	5,8	5,8	19,15	575,38	58,39
2019/05/21	CH2206	Male	Three y/o	Treatment (Wk 1)	Treatment	Feed Day	No	12:40	12:00 - 12:59	Afternoon	37,9	99	32,4	32,4	19,05	593,14	116,84
2019/05/21	CH2205	Male	Three y/o	Treatment (Wk 1)	Treatment	Feed Day	No	12:45	12:00 - 12:59	Afternoon	38,0	66	3,4	3,4	19,15	562,34	40,51
2019/05/21	CH2206	Male	Three y/o	Treatment (Wk 1)	Treatment	Feed Day	No	12:45	12:00 - 12:59	Afternoon	37,9	88	21,2	21,2	19,05	584,49	102,31
2019/05/21	CH2205	Male	Three y/o	Treatment (Wk 1)	Treatment	Feed Day	No	12:50	12:00 - 12:59	Afternoon	38,0	63	5,2	5,2	19,15	558,62	54,72
2019/05/21	CH2206	Male	Three y/o	Treatment (Wk 1)	Treatment	Feed Day	No	12:50	12:00 - 12:59	Afternoon	37,9	65	26,0	26,0	19,05	561,12	109,29
2019/05/21	CH2205	Male	Three y/o	Treatment (Wk 1)	Treatment	Feed Day	No	12:55	12:00 - 12:59	Afternoon	38,1	84	2,8	2,8	19,26	581,01	34,04
2019/05/21	CH2206	Male	Three y/o	Treatment (Wk 1)	Treatment	Feed Day	No	12:55	12:00 - 12:59	Afternoon	37,9	80	24,6	24,6	19,05	577,31	107,39
2019/05/21	CH2205	Male	Three y/o	Treatment (Wk 1)	Treatment	Feed Day	No	13:00	13:00 - 13:59	Afternoon	38,0		31,2	31,2	19,15		115,54
2019/05/21	CH2206	Male	Three y/o	Treatment (Wk 1)	Treatment	Feed Day	No	13:00	13:00 - 13:59	Afternoon	37,9	68	28,0	28,0	19,05	564,71	111,83
2019/05/21	CH2205	Male	Three y/o	Treatment (Wk 1)	Treatment	Feed Day	No	13:05	13:00 - 13:59	Afternoon	38,0	64	18,4	18,4	19,15	559,88	97,47
2019/05/21	CH2206	Male	Three y/o	Treatment (Wk 1)	Treatment	Feed Day	No	13:05	13:00 - 13:59	Afternoon	37,9	79	28,2	28,2	19,05	576,36	112,07
2019/05/21	CH2205	Male	Three y/o	Treatment (Wk 1)	Treatment	Feed Day	No	13:10	13:00 - 13:59	Afternoon	38,0	93	4,2	4,2	19,15	588,58	47,57
2019/05/21	CH2206	Male	Three y/o	Treatment (Wk 1)	Treatment	Feed Day	No	13:10	13:00 - 13:59	Afternoon	37,9	92	37,6	37,6	19,05	587,78	121,96
2019/05/21	CH2205	Male	Three y/o	Treatment (Wk 1)	Treatment	Feed Day	No	13:15	13:00 - 13:59	Afternoon	38,0	90	5,6	5,6	19,15	586,16	57,21
2019/05/21	CH2206	Male	Three y/o	Treatment (Wk 1)	Treatment	Feed Day	No	13:15	13:00 - 13:59	Afternoon	37,8	86	36,8	36,8	18,95	582,77	121,22
2019/05/21	CH2205	Male	Three y/o	Treatment (Wk 1)	Treatment	Feed Day	No	13:20	13:00 - 13:59	Afternoon	38,1	60	20,0	20,0	19,26	554,67	100,31

2019/05/21	CH2206	Male	Three y/o	Treatment (Wk 1)	Treatment	Feed Day	No	13:20	13:00 - 13:59	Afternoon	37,8	82	42,6	42,6	18,95	579,19	126,26
2019/05/21	CH2205	Male	Three y/o	Treatment (Wk 1)	Treatment	Feed Day	No	13:25	13:00 - 13:59	Afternoon	38,0		95,6	95,6	19,15		154,28
2019/05/21	CH2206	Male	Three y/o	Treatment (Wk 1)	Treatment	Feed Day	No	13:25	13:00 - 13:59	Afternoon	37,8	55	54,4	54,4	18,95	547,52	134,70
2019/05/21	CH2205	Male	Three y/o	Treatment (Wk 1)	Treatment	Feed Day	No	13:30	13:00 - 13:59	Afternoon	37,9	108	63,0	63,0	19,05	599,39	139,78
2019/05/21	CH2206	Male	Three y/o	Treatment (Wk 1)	Treatment	Feed Day	No	13:30	13:00 - 13:59	Afternoon	37,8	112	47,0	47,0	18,95	601,96	129,65
2019/05/21	CH2205	Male	Three y/o	Treatment (Wk 1)	Treatment	Feed Day	No	13:35	13:00 - 13:59	Afternoon	37,7		26,6	26,6	18,85		110,07
2019/05/21	CH2206	Male	Three y/o	Treatment (Wk 1)	Treatment	Feed Day	No	13:35	13:00 - 13:59	Afternoon	37,8	76	10,0	10,0	18,95	573,39	76,76
2019/05/21	CH2205	Male	Three y/o	Treatment (Wk 1)	Treatment	Feed Day	No	13:40	13:00 - 13:59	Afternoon	37,7	63	33,0	33,0	18,85	558,62	117,47
2019/05/21	CH2206	Male	Three y/o	Treatment (Wk 1)	Treatment	Feed Day	No	13:40	13:00 - 13:59	Afternoon	37,8	97	15,0	15,0	18,95	591,66	90,51
2019/05/21	CH2205	Male	Three y/o	Treatment (Wk 1)	Treatment	Feed Day	No	13:45	13:00 - 13:59	Afternoon	37,9	124	22,8	22,8	19,05	609,04	104,79
2019/05/21	CH2206	Male	Three y/o	Treatment (Wk 1)	Treatment	Feed Day	No	13:45	13:00 - 13:59	Afternoon	37,8	97	8,8	8,8	18,95	591,66	72,43
2019/05/21	CH2205	Male	Three y/o	Treatment (Wk 1)	Treatment	Feed Day	No	13:50	13:00 - 13:59	Afternoon	37,9	84	26,8	26,8	19,05	581,01	110,33
2019/05/21	CH2206	Male	Three y/o	Treatment (Wk 1)	Treatment	Feed Day	No	13:50	13:00 - 13:59	Afternoon	37,8	88	26,8	26,8	18,95	584,49	110,33
2019/05/21	CH2205	Male	Three y/o	Treatment (Wk 1)	Treatment	Feed Day	No	13:55	13:00 - 13:59	Afternoon	37,9	43	230,6	230,6	19,05	526,50	185,13
2019/05/21	CH2206	Male	Three y/o	Treatment (Wk 1)	Treatment	Feed Day	No	13:55	13:00 - 13:59	Afternoon	37,9	93	26,4	26,4	19,05	588,58	109,81
2019/05/21	CH2205	Male	Three y/o	Treatment (Wk 1)	Treatment	Feed Day	No	14:00	14:00 - 14:59	Afternoon	38,0		49,0	49,0	19,15		131,09
2019/05/21	CH2206	Male	Three y/o	Treatment (Wk 1)	Treatment	Feed Day	No	14:00	14:00 - 14:59	Afternoon	37,9	106	145,6	145,6	19,05	598,06	168,97
2019/05/21	CH2205	Male	Three y/o	Treatment (Wk 1)	Treatment	Feed Day	No	14:05	14:00 - 14:59	Afternoon	37,9	74	21,2	21,2	19,05	571,33	102,31
2019/05/21	CH2206	Male	Three y/o	Treatment (Wk 1)	Treatment	Feed Day	No	14:05	14:00 - 14:59	Afternoon	37,9	93	100,8	100,8	19,05	588,58	156,12
2019/05/21	CH2205	Male	Three y/o	Treatment (Wk 1)	Treatment	Feed Day	No	14:10	14:00 - 14:59	Afternoon	38,0		10,8	10,8	19,15		79,36
2019/05/21	CH2206	Male	Three y/o	Treatment (Wk 1)	Treatment	Feed Day	No	14:10	14:00 - 14:59	Afternoon	37,9	91	5,2	5,2	19,05	586,98	54,72
2019/05/21	CH2205	Male	Three y/o	Treatment (Wk 1)	Treatment	Feed Day	No	14:15	14:00 - 14:59	Afternoon	38,0	83	5,0	5,0	19,15	580,11	53,41
2019/05/21	CH2206	Male	Three y/o	Treatment (Wk 1)	Treatment	Feed Day	No	14:15	14:00 - 14:59	Afternoon	37,9	77	4,6	4,6	19,05	574,39	50,61
2019/05/21	CH2205	Male	Three y/o	Treatment (Wk 1)	Treatment	Feed Day	No	14:20	14:00 - 14:59	Afternoon	38,1	64	9,0	9,0	19,26	559,88	73,19
2019/05/21	CH2206	Male	Three y/o	Treatment (Wk 1)	Treatment	Feed Day	No	14:20	14:00 - 14:59	Afternoon	37,9	74	6,2	6,2	19,05	571,33	60,63
2019/05/21	CH2205	Male	Three y/o	Treatment (Wk 1)	Treatment	Feed Day	No	14:25	14:00 - 14:59	Afternoon	38,1	66	12,8	12,8	19,26	562,34	85,12
2019/05/21	CH2206	Male	Three y/o	Treatment (Wk 1)	Treatment	Feed Day	No	14:25	14:00 - 14:59	Afternoon	37,9	86	2,4	2,4	19,05	582,77	28,91
2019/05/21	CH2205	Male	Three y/o	Treatment (Wk 1)	Treatment	Feed Day	No	14:30	14:00 - 14:59	Afternoon	38,1	55	20,6	20,6	19,26	547,52	101,32
2019/05/21	CH2206	Male	Three y/o	Treatment (Wk 1)	Treatment	Feed Day	No	14:30	14:00 - 14:59	Afternoon	38,0	84	4,4	4,4	19,15	581,01	49,12
2019/05/21	CH2205	Male	Three y/o	Treatment (Wk 1)	Treatment	Feed Day	No	14:35	14:00 - 14:59	Afternoon	38,1	86	20,6	20,6	19,26	582,77	101,32
2019/05/21	CH2206	Male	Three y/o	Treatment (Wk 1)	Treatment	Feed Day	No	14:35	14:00 - 14:59	Afternoon	38,0		40,6	40,6	19,15		124,60
2019/05/21	CH2205	Male	Three y/o	Treatment (Wk 1)	Treatment	Feed Day	No	14:40	14:00 - 14:59	Afternoon	38,1	63	10,8	10,8	19,26	558,62	79,36
2019/05/21	CH2206	Male	Three y/o	Treatment (Wk 1)	Treatment	Feed Day	No	14:40	14:00 - 14:59	Afternoon	38,0	90	54,6	54,6	19,15	586,16	134,83
2019/05/21	CH2205	Male	Three y/o	Treatment (Wk 1)	Treatment	Feed Day	No	14:45	14:00 - 14:59	Afternoon	38,1	68	4,8	4,8	19,26	564,71	52,04
2019/05/21	CH2206	Male	Three y/o	Treatment (Wk 1)	Treatment	Feed Day	No	14:45	14:00 - 14:59	Afternoon	38,0	85	57,0	57,0	19,15	581,90	136,32
2019/05/21	CH2205	Male	Three y/o	Treatment (Wk 1)	Treatment	Feed Day	No	14:50	14:00 - 14:59	Afternoon	38,2		11,2	11,2	19,36		80,59
2019/05/21	CH2206	Male	Three y/o	Treatment (Wk 1)	Treatment	Feed Day	No	14:50	14:00 - 14:59	Afternoon	38,1	88	47,4	47,4	19,26	584,49	129,94
2019/05/21	CH2205	Male	Three y/o	Treatment (Wk 1)	Treatment	Feed Day	No	14:55	14:00 - 14:59	Afternoon	38,2	69	14,0	14,0	19,36	565,86	88,17
2019/05/21	CH2206	Male	Three y/o	Treatment (Wk 1)	Treatment	Feed Day	No	14:55	14:00 - 14:59	Afternoon	38,1	72	53,4	53,4	19,26	569,20	134,06
2019/05/21	CH2205	Male	Three y/o	Treatment (Wk 1)	Treatment	Feed Day	No	15:00	15:00 - 15:59	Afternoon	38,2	74	10,2	10,2	19,36	571,33	77,43
2019/05/21	CH2206	Male	Three y/o	Treatment (Wk 1)	Treatment	Feed Day	No	15:00	15:00 - 15:59	Afternoon	38,1	76	55,6	55,6	19,26	573,39	135,46
2019/05/21	CH2205	Male	Three y/o	Treatment (Wk 1)	Treatment	Feed Day	No	15:05	15:00 - 15:59	Afternoon	38,2	72	7,2	7,2	19,36	569,20	65,66
2019/05/21	CH2206	Male	Three y/o	Treatment (Wk 1)	Treatment	Feed Day	No	15:05	15:00 - 15:59	Afternoon	38,1	84	54,6	54,6	19,26	581,01	134,83
2019/05/21	CH2205	Male	Three y/o	Treatment (Wk 1)	Treatment	Feed Day	No	15:10	15:00 - 15:59	Afternoon	38,2	63	8,8	8,8	19,36	558,62	72,43
2019/05/21	CH2206	Male	Three y/o	Treatment (Wk 1)	Treatment	Feed Day	No	15:10	15:00 - 15:59	Afternoon	38,1	74	48,2	48,2	19,26	571,33	130,52
2019/05/21	CH2205	Male	Three y/o	Treatment (Wk 1)	Treatment	Feed Day	No	15:15	15:00 - 15:59	Afternoon	38,2	68	6,2	6,2	19,36	564,71	60,63
2019/05/21	CH2206	Male	Three y/o	Treatment (Wk 1)	Treatment	Feed Day	No	15:15	15:00 - 15:59	Afternoon	38,2	61	54,4	54,4	19,36	556,01	134,70
2019/05/21	CH2205	Male	Three y/o	Treatment (Wk 1)	Treatment	Feed Day	No	15:20	15:00 - 15:59	Afternoon	38,2	90	10,2	10,2	19,36	586,16	77,43
2019/05/21	CH2206	Male	Three y/o	Treatment (Wk 1)	Treatment	Feed Day	No	15:20	15:00 - 15:59	Afternoon	38,2	101	49,8	49,8	19,36	594,59	131,65
2019/05/21	CH2205	Male	Three y/o	Treatment (Wk 1)	Treatment	Feed Day	No	15:25	15:00 - 15:59	Afternoon	38,2	76	3,6	3,6	19,36	573,39	42,42
2019/05/21	CH2206	Male	Three y/o	Treatment (Wk 1)	Treatment	Feed Day	No	15:25	15:00 - 15:59	Afternoon	38,2	97	44,2	44,2	19,36	591,66	127,53
2019/05/21	CH2205	Male	Three y/o	Treatment (Wk 1)	Treatment	Feed Day	No	15:30	15:00 - 15:59	Afternoon	38,2	86	3,8	3,8	19,36	582,77	44,22
2019/05/21	CH2206	Male	Three y/o	Treatment (Wk 1)	Treatment	Feed Day	No	15:30	15:00 - 15:59	Afternoon	38,2	68	39,2	39,2	19,36	564,71	123,39
2019/05/21	CH2205	Male	Three y/o	Treatment (Wk 1)	Treatment	Feed Day	No	15:35	15:00 - 15:59	Afternoon	38,2	77	3,2	3,2	19,36	574,39	38,49
2019/05/21	CH2206	Male	Three y/o	Treatment (Wk 1)	Treatment	Feed Day	No	15:35	15:00 - 15:59	Afternoon	38,2	56	35,2	35,2	19,36	549,01	119,69
2019/05/21	CH2205	Male	Three y/o	Treatment (Wk 1)	Treatment	Feed Day	No	15:40	15:00 - 15:59	Afternoon	38,2	49	32,8	32,8	19,36	537,80	117,26
2019/05/21	CH2206	Male	Three y/o	Treatment (Wk 1)	Treatment	Feed Day	No	15:40	15:00 - 15:59	Afternoon	38,2	94	56,2	56,2	19,36	589,37	135,83
2019/05/21	CH2205	Male	Three y/o	Treatment (Wk 1)	Treatment	Feed Day	No	15:45	15:00 - 15:59	Afternoon	38,1	124	72,0	72,0	19,26	609,04	144,42
2019/05/21	CH2206	Male	Three y/o	Treatment (Wk 1)	Treatment	Feed Day	No	15:45	15:00 - 15:59	Afternoon	38,2	106	48,8	48,8	19,36	598,06	130,95
2019/05/21	CH2205	Male	Three y/o	Treatment (Wk 1)	Treatment	Feed Day	No	15:50	15:00 - 15:59	Afternoon	38,0	75	11,8	11,8	19,15	572,36	82,36
2019/05/21	CH2206	Male	Three y/o	Treatment (Wk 1)	Treatment	Feed Day	No	15:50	15:00 - 15:59	Afternoon	38,1	115	55,4	55,4	19,26	603,82	135,33
2019/05/21	CH2205	Male	Three y/o	Treatment (Wk 1)	Treatment	Feed Day	No	15:55	15:00 - 15:59	Afternoon	38,1	63	13,4	13,4	19,26	558,62	86,68
2019/05/21	CH2206	Male	Three y/o	Treatment (Wk 1)	Treatment	Feed Day	No	15:55	15:00 - 15:59	Afternoon	38,1	98	95,2	95,2	19,26	592,41	154,13
2019/05/21	CH2205	Male	Three y/o	Treatment (Wk 1)	Treatment	Feed Day	No	16:00	16:00 - 16:59	Evening	38,0	74	10,0	10,0	19,15	571,33	76,76
2019/05/21	CH2206	Male	Three y/o	Treatment (Wk 1)	Treatment	Feed Day	No	16:00	16:00 - 16:59	Evening	38,1	93	4,4	4,4	19,26	588,58	49,12
2019/05/21	CH2205	Male	Three y/o	Treatment (Wk 1)	Treatment	Feed Day	No	16:05	16:00 - 16:59	Evening	38,1	54	6,6	6,6	19,26	545,99	62,73
2019/05/21	CH2206	Male	Three y/o	Treatment (Wk 1)	Treatment	Feed Day	No	16:05	16:00 - 16:59	Evening	38,1	90	9,4	9,4	19,26	586,16	74,66
2019/05/21	CH2205	Male	Three y/o	Treatment (Wk 1)	Treatment	Feed Day	No	16:10	16:00 - 16:59	Evening	38,1	66	8,4	8,4	19,26	562,34	70,86
2019/05/21	CH2206	Male	Three y/o	Treatment (Wk 1)	Treatment	Feed Day	No	16:10	16:00 - 16:59	Evening	38,1	90	15,6	15,6	19,26	586,16	91,85
2019/05/21	CH2205	Male	Three y/o	Treatment (Wk 1)	Treatment	Feed Day	No	16:15	16:00 - 16:59	Evening	38,1	46	10,4	10,4	19,26	532,38	78,08

2019/05/21	CH2206	Male	Three y/o	Treatment (Wk 1)	Treatment	Feed Day	No	16:15	16:00 - 16:59	Evening	38,1	82	28,4	28,4	19,26	579,19	112,31
2019/05/21	CH2205	Male	Three y/o	Treatment (Wk 1)	Treatment	Feed Day	No	16:20	16:00 - 16:59	Evening	38,2	78	12,0	12,0	19,36	575,38	82,93
2019/05/21	CH2206	Male	Three y/o	Treatment (Wk 1)	Treatment	Feed Day	No	16:20	16:00 - 16:59	Evening	38,1	45	39,4	39,4	19,26	530,47	123,57
2019/05/21	CH2205	Male	Three y/o	Treatment (Wk 1)	Treatment	Feed Day	No	16:25	16:00 - 16:59	Evening	38,2	57	13,6	13,6	19,36	550,47	87,18
2019/05/21	CH2206	Male	Three y/o	Treatment (Wk 1)	Treatment	Feed Day	No	16:25	16:00 - 16:59	Evening	38,0	78	27,8	27,8	19,15	575,38	111,58
2019/05/21	CH2205	Male	Three y/o	Treatment (Wk 1)	Treatment	Feed Day	No	16:30	16:00 - 16:59	Evening	38,2	68	9,2	9,2	19,36	564,71	73,94
2019/05/21	CH2206	Male	Three y/o	Treatment (Wk 1)	Treatment	Feed Day	No	16:30	16:00 - 16:59	Evening	37,9	82	31,6	31,6	19,05	579,19	115,98
2019/05/21	CH2205	Male	Three y/o	Treatment (Wk 1)	Treatment	Feed Day	No	16:35	16:00 - 16:59	Evening	38,2	61	6,2	6,2	19,36	556,01	60,63
2019/05/21	CH2206	Male	Three y/o	Treatment (Wk 1)	Treatment	Feed Day	No	16:35	16:00 - 16:59	Evening	37,9	76	45,6	45,6	19,05	573,39	128,61
2019/05/21	CH2205	Male	Three y/o	Treatment (Wk 1)	Treatment	Feed Day	No	16:40	16:00 - 16:59	Evening	38,2	60	12,2	12,2	19,36	554,67	83,49
2019/05/21	CH2206	Male	Three y/o	Treatment (Wk 1)	Treatment	Feed Day	No	16:40	16:00 - 16:59	Evening	37,9	80	49,0	49,0	19,05	577,31	131,09
2019/05/21	CH2205	Male	Three y/o	Treatment (Wk 1)	Treatment	Feed Day	No	16:45	16:00 - 16:59	Evening	38,2	75	11,6	11,6	19,36	572,36	81,78
2019/05/21	CH2206	Male	Three y/o	Treatment (Wk 1)	Treatment	Feed Day	No	16:45	16:00 - 16:59	Evening	37,9	87	42,2	42,2	19,05	583,64	125,93
2019/05/21	CH2205	Male	Three y/o	Treatment (Wk 1)	Treatment	Feed Day	No	16:50	16:00 - 16:59	Evening	38,2	56	8,8	8,8	19,36	549,01	72,43
2019/05/21	CH2206	Male	Three y/o	Treatment (Wk 1)	Treatment	Feed Day	No	16:50	16:00 - 16:59	Evening	37,8	76	23,8	23,8	18,95	573,39	106,26
2019/05/21	CH2205	Male	Three y/o	Treatment (Wk 1)	Treatment	Feed Day	No	16:55	16:00 - 16:59	Evening	38,2	89	12,4	12,4	19,36	585,33	84,04
2019/05/21	CH2206	Male	Three y/o	Treatment (Wk 1)	Treatment	Feed Day	No	16:55	16:00 - 16:59	Evening	37,9	77	8,0	8,0	19,05	574,39	69,22
2019/05/21	CH2205	Male	Three y/o	Treatment (Wk 1)	Treatment	Feed Day	No	17:00	17:00 - 17:59	Evening	38,1	80	9,0	9,0	19,26	577,31	73,19
2019/05/21	CH2206	Male	Three y/o	Treatment (Wk 1)	Treatment	Feed Day	No	17:00	17:00 - 17:59	Evening	37,9	78	28,6	28,6	19,05	575,38	112,56
2019/05/21	CH2205	Male	Three y/o	Treatment (Wk 1)	Treatment	Feed Day	No	17:05	17:00 - 17:59	Evening	38,1	131	19,0	19,0	19,26	612,80	98,56
2019/05/21	CH2206	Male	Three y/o	Treatment (Wk 1)	Treatment	Feed Day	No	17:05	17:00 - 17:59	Evening	37,9	44	41,2	41,2	19,05	528,51	125,11
2019/05/21	CH2205	Male	Three y/o	Treatment (Wk 1)	Treatment	Feed Day	No	17:10	17:00 - 17:59	Evening	38,0	56	155,4	155,4	19,15	549,01	171,26
2019/05/21	CH2206	Male	Three y/o	Treatment (Wk 1)	Treatment	Feed Day	No	17:10	17:00 - 17:59	Evening	37,9	87	32,4	32,4	19,05	583,64	116,84
2019/05/21	CH2205	Male	Three y/o	Treatment (Wk 1)	Treatment	Feed Day	No	17:15	17:00 - 17:59	Evening	38,0	172	92,0	92,0	19,15	630,73	152,94
2019/05/21	CH2206	Male	Three y/o	Treatment (Wk 1)	Treatment	Feed Day	No	17:15	17:00 - 17:59	Evening	37,8	62	15,4	15,4	18,95	557,33	91,41
2019/05/21	CH2205	Male	Three y/o	Treatment (Wk 1)	Treatment	Feed Day	No	17:20	17:00 - 17:59	Evening	37,9	93	44,4	44,4	19,05	588,58	127,69
2019/05/21	CH2206	Male	Three y/o	Treatment (Wk 1)	Treatment	Feed Day	No	17:20	17:00 - 17:59	Evening	37,7	43	26,4	26,4	18,85	526,50	109,81
2019/05/21	CH2205	Male	Three y/o	Treatment (Wk 1)	Treatment	Feed Day	No	17:25	17:00 - 17:59	Evening	37,9		16,8	16,8	19,05		94,37
2019/05/21	CH2206	Male	Three y/o	Treatment (Wk 1)	Treatment	Feed Day	No	17:25	17:00 - 17:59	Evening	37,7	73	45,2	45,2	18,85	570,27	128,30
2019/05/21	CH2205	Male	Three y/o	Treatment (Wk 1)	Treatment	Feed Day	No	17:30	17:00 - 17:59	Evening	37,9		13,2	13,2	19,05		86,17
2019/05/21	CH2206	Male	Three y/o	Treatment (Wk 1)	Treatment	Feed Day	No	17:30	17:00 - 17:59	Evening	37,7	76	36,2	36,2	18,85	573,39	120,65
2019/05/21	CH2205	Male	Three y/o	Treatment (Wk 1)	Treatment	Feed Day	No	17:35	17:00 - 17:59	Evening	37,9	95	14,6	14,6	19,05	590,14	89,59
2019/05/21	CH2206	Male	Three y/o	Treatment (Wk 1)	Treatment	Feed Day	No	17:35	17:00 - 17:59	Evening	37,8	82	30,6	30,6	18,95	579,19	114,87
2019/05/21	CH2205	Male	Three y/o	Treatment (Wk 1)	Treatment	Feed Day	No	17:40	17:00 - 17:59	Evening	38,0	67	11,0	11,0	19,15	563,53	79,98
2019/05/21	CH2206	Male	Three y/o	Treatment (Wk 1)	Treatment	Feed Day	No	17:40	17:00 - 17:59	Evening	37,9	80	38,2	38,2	19,05	577,31	122,50
2019/05/21	CH2205	Male	Three y/o	Treatment (Wk 1)	Treatment	Feed Day	No	17:45	17:00 - 17:59	Evening	38,0	77	11,0	11,0	19,15	574,39	79,98
2019/05/21	CH2206	Male	Three y/o	Treatment (Wk 1)	Treatment	Feed Day	No	17:45	17:00 - 17:59	Evening	37,9	61	36,2	36,2	19,05	556,01	120,65
2019/05/21	CH2205	Male	Three y/o	Treatment (Wk 1)	Treatment	Feed Day	No	17:50	17:00 - 17:59	Evening	38,0	56	17,6	17,6	19,15	549,01	95,95
2019/05/21	CH2206	Male	Three y/o	Treatment (Wk 1)	Treatment	Feed Day	No	17:50	17:00 - 17:59	Evening	37,9	76	46,8	46,8	19,05	573,39	129,50
2019/05/21	CH2205	Male	Three y/o	Treatment (Wk 1)	Treatment	Feed Day	No	17:55	17:00 - 17:59	Evening	38,0	80	15,4	15,4	19,15	577,31	91,41
2019/05/21	CH2206	Male	Three y/o	Treatment (Wk 1)	Treatment	Feed Day	No	17:55	17:00 - 17:59	Evening	37,9	91	28,8	28,8	19,05	586,98	112,79
2019/05/21	CH2205	Male	Three y/o	Treatment (Wk 1)	Treatment	Feed Day	No	18:00	18:00 - 18:59	Evening	38,0	87	13,8	13,8	19,15	583,64	87,68
2019/05/21	CH2206	Male	Three y/o	Treatment (Wk 1)	Treatment	Feed Day	No	18:00	18:00 - 18:59	Evening	37,9	81	44,6	44,6	19,05	578,26	127,84
2019/05/21	CH2205	Male	Three y/o	Treatment (Wk 1)	Treatment	Feed Day	No	18:05	18:00 - 18:59	Evening	37,9	95	23,2	23,2	19,05	590,14	105,39
2019/05/21	CH2206	Male	Three y/o	Treatment (Wk 1)	Treatment	Feed Day	No	18:05	18:00 - 18:59	Evening	37,9	49	131,8	131,8	19,05	537,80	165,49
2019/05/21	CH2205	Male	Three y/o	Treatment (Wk 1)	Treatment	Feed Day	No	18:10	18:00 - 18:59	Evening	37,9	98	23,2	23,2	19,05	592,41	105,39
2019/05/21	CH2206	Male	Three y/o	Treatment (Wk 1)	Treatment	Feed Day	No	18:10	18:00 - 18:59	Evening	37,9	129	149,4	149,4	19,05	611,75	169,88
2019/05/21	CH2205	Male	Three y/o	Treatment (Wk 1)	Treatment	Feed Day	No	18:15	18:00 - 18:59	Evening	38,0	97	21,6	21,6	19,15	591,66	102,94
2019/05/21	CH2206	Male	Three y/o	Treatment (Wk 1)	Treatment	Feed Day	No	18:15	18:00 - 18:59	Evening	37,8	51	55,2	55,2	18,95	541,20	135,21
2019/05/21	CH2205	Male	Three y/o	Treatment (Wk 1)	Treatment	Feed Day	No	18:20	18:00 - 18:59	Evening	38,0	152	6,0	6,0	19,15	622,73	59,53
2019/05/21	CH2206	Male	Three y/o	Treatment (Wk 1)	Treatment	Feed Day	No	18:20	18:00 - 18:59	Evening	37,7	81	54,6	54,6	18,85	578,26	134,83
2019/05/21	CH2205	Male	Three y/o	Treatment (Wk 1)	Treatment	Feed Day	No	18:25	18:00 - 18:59	Evening	37,7	64	3,0	3,0	18,85	559,88	36,34
2019/05/21	CH2206	Male	Three y/o	Treatment (Wk 1)	Treatment	Feed Day	No	18:25	18:00 - 18:59	Evening	37,7	46	53,2	53,2	18,85	532,38	133,93
2019/05/21	CH2205	Male	Three y/o	Treatment (Wk 1)	Treatment	Feed Day	No	18:30	18:00 - 18:59	Evening	37,5	64	7,4	7,4	18,65	559,88	66,59
2019/05/21	CH2206	Male	Three y/o	Treatment (Wk 1)	Treatment	Feed Day	No	18:30	18:00 - 18:59	Evening	37,8	81	62,6	62,6	18,95	578,26	139,56
2019/05/21	CH2205	Male	Three y/o	Treatment (Wk 1)	Treatment	Feed Day	No	18:35	18:00 - 18:59	Evening	37,3	62	3,4	3,4	18,46	557,33	40,51
2019/05/21	CH2206	Male	Three y/o	Treatment (Wk 1)	Treatment	Feed Day	No	18:35	18:00 - 18:59	Evening	37,8	66	61,8	61,8	18,95	562,34	139,12
2019/05/21	CH2205	Male	Three y/o	Treatment (Wk 1)	Treatment	Feed Day	No	18:40	18:00 - 18:59	Evening	37,3	73	3,0	3,0	18,46	570,27	36,34
2019/05/21	CH2206	Male	Three y/o	Treatment (Wk 1)	Treatment	Feed Day	No	18:40	18:00 - 18:59	Evening	37,8	85	49,2	49,2	18,95	581,90	131,23
2019/05/21	CH2205	Male	Three y/o	Treatment (Wk 1)	Treatment	Feed Day	No	18:45	18:00 - 18:59	Evening	37,2		3,6	3,6	18,36		42,42
2019/05/21	CH2206	Male	Three y/o	Treatment (Wk 1)	Treatment	Feed Day	No	18:45	18:00 - 18:59	Evening	37,7	77	51,4	51,4	18,85	574,39	132,74
2019/05/21	CH2205	Male	Three y/o	Treatment (Wk 1)	Treatment	Feed Day	No	18:50	18:00 - 18:59	Evening	37,1	88	5,2	5,2	18,26	584,49	54,72
2019/05/21	CH2206	Male	Three y/o	Treatment (Wk 1)	Treatment	Feed Day	No	18:50	18:00 - 18:59	Evening	37,8	67	61,6	61,6	18,95	563,53	139,00
2019/05/21	CH2205	Male	Three y/o	Treatment (Wk 1)	Treatment	Feed Day	No	18:55	18:00 - 18:59	Evening	37,1	61	4,2	4,2	18,26	556,01	47,57
2019/05/21	CH2206	Male	Three y/o	Treatment (Wk 1)	Treatment	Feed Day	No	18:55	18:00 - 18:59	Evening	37,5	48	57,6	57,6	18,65	536,04	136,68
2019/05/21	CH2205	Male	Three y/o	Treatment (Wk 1)	Treatment	Feed Day	No	19:00	19:00 - 19:59	Evening	37,0	57	4,0	4,0	18,16	550,47	45,94
2019/05/21	CH2206	Male	Three y/o	Treatment (Wk 1)	Treatment	Feed Day	No	19:00	19:00 - 19:59	Evening	37,7	86	55,6	55,6	18,85	582,77	135,46
2019/05/21	CH2205	Male	Three y/o	Treatment (Wk 1)	Treatment	Feed Day	No	19:05	19:00 - 19:59	Evening	37,0	94	4,6	4,6	18,16	589,37	50,61
2019/05/21	CH2206	Male	Three y/o	Treatment (Wk 1)	Treatment	Feed Day	No	19:05	19:00 - 19:59	Evening	37,8	60	54,0	54,0	18,95	554,67	134,45
2019/05/21	CH2205	Male	Three y/o	Treatment (Wk 1)	Treatment	Feed Day	No	19:10	19:00 - 19:59	Evening	37,0	93	7,6	7,6	18,16	588,58	67,49

2019/05/21	CH2206	Male	Three y/o	Treatment (Wk 1)	Treatment	Feed Day	No	19:10	19:00 - 19:59	Evening	37,8	80	55,0	55,0	18,95	577,31	135,08
2019/05/21	CH2205	Male	Three y/o	Treatment (Wk 1)	Treatment	Feed Day	No	19:15	19:00 - 19:59	Evening	37,1	83	6,4	6,4	18,26	580,11	61,70
2019/05/21	CH2206	Male	Three y/o	Treatment (Wk 1)	Treatment	Feed Day	No	19:15	19:00 - 19:59	Evening	37,8	90	56,0	56,0	18,95	586,16	135,70
2019/05/21	CH2205	Male	Three y/o	Treatment (Wk 1)	Treatment	Feed Day	No	19:20	19:00 - 19:59	Evening	37,0	94	1,8	1,8	18,16	589,37	19,38
2019/05/21	CH2206	Male	Three y/o	Treatment (Wk 1)	Treatment	Feed Day	No	19:20	19:00 - 19:59	Evening	37,9	46	49,4	49,4	19,05	532,38	131,37
2019/05/21	CH2205	Male	Three y/o	Treatment (Wk 1)	Treatment	Feed Day	No	19:25	19:00 - 19:59	Evening	37,0	83	3,4	3,4	18,16	580,11	40,51
2019/05/21	CH2206	Male	Three y/o	Treatment (Wk 1)	Treatment	Feed Day	No	19:25	19:00 - 19:59	Evening	37,8	77	42,8	42,8	18,95	574,39	126,42
2019/05/21	CH2205	Male	Three y/o	Treatment (Wk 1)	Treatment	Feed Day	No	19:30	19:00 - 19:59	Evening	36,8	90	8,8	8,8	17,96	586,16	72,43
2019/05/21	CH2206	Male	Three y/o	Treatment (Wk 1)	Treatment	Feed Day	No	19:30	19:00 - 19:59	Evening	37,8	47	43,8	43,8	18,95	534,23	127,22
2019/05/21	CH2205	Male	Three y/o	Treatment (Wk 1)	Treatment	Feed Day	No	19:35	19:00 - 19:59	Evening	37,0	97	9,4	9,4	18,16	591,66	74,66
2019/05/21	CH2206	Male	Three y/o	Treatment (Wk 1)	Treatment	Feed Day	No	19:35	19:00 - 19:59	Evening	37,8	63	42,6	42,6	18,95	558,62	126,26
2019/05/21	CH2205	Male	Three y/o	Treatment (Wk 1)	Treatment	Feed Day	No	19:40	19:00 - 19:59	Evening	37,1	84	11,0	11,0	18,26	581,01	79,98
2019/05/21	CH2206	Male	Three y/o	Treatment (Wk 1)	Treatment	Feed Day	No	19:40	19:00 - 19:59	Evening	37,9	94	46,0	46,0	19,05	589,37	128,91
2019/05/21	CH2205	Male	Three y/o	Treatment (Wk 1)	Treatment	Feed Day	No	19:45	19:00 - 19:59	Evening	37,2	100	8,0	8,0	18,36	593,87	69,22
2019/05/21	CH2206	Male	Three y/o	Treatment (Wk 1)	Treatment	Feed Day	No	19:45	19:00 - 19:59	Evening	37,9	94	56,0	56,0	19,05	589,37	135,70
2019/05/21	CH2205	Male	Three y/o	Treatment (Wk 1)	Treatment	Feed Day	No	19:50	19:00 - 19:59	Evening	37,3	94	3,8	3,8	18,46	589,37	44,22
2019/05/21	CH2206	Male	Three y/o	Treatment (Wk 1)	Treatment	Feed Day	No	19:50	19:00 - 19:59	Evening	37,8	49	54,0	54,0	18,95	537,80	134,45
2019/05/21	CH2205	Male	Three y/o	Treatment (Wk 1)	Treatment	Feed Day	No	19:55	19:00 - 19:59	Evening	37,3	58	9,4	9,4	18,46	551,90	74,66
2019/05/21	CH2206	Male	Three y/o	Treatment (Wk 1)	Treatment	Feed Day	No	19:55	19:00 - 19:59	Evening	37,8	44	60,4	60,4	18,95	528,51	138,32
2019/05/21	CH2205	Male	Three y/o	Treatment (Wk 1)	Treatment	Feed Day	No	20:00	20:00 - 20:59	Night	37,4	57	44,4	44,4	18,55	550,47	127,69
2019/05/21	CH2206	Male	Three y/o	Treatment (Wk 1)	Treatment	Feed Day	No	20:00	20:00 - 20:59	Night	37,8	67	47,0	47,0	18,95	563,53	129,65
2019/05/21	CH2205	Male	Three y/o	Treatment (Wk 1)	Treatment	Feed Day	No	20:05	20:00 - 20:59	Night	37,5	90	3,4	3,4	18,65	586,16	40,51
2019/05/21	CH2206	Male	Three y/o	Treatment (Wk 1)	Treatment	Feed Day	No	20:05	20:00 - 20:59	Night	37,8	76	36,4	36,4	18,95	573,39	120,84
2019/05/21	CH2205	Male	Three y/o	Treatment (Wk 1)	Treatment	Feed Day	No	20:10	20:00 - 20:59	Night	37,4	70	4,2	4,2	18,55	566,99	47,57
2019/05/21	CH2206	Male	Three y/o	Treatment (Wk 1)	Treatment	Feed Day	No	20:10	20:00 - 20:59	Night	37,8	55	35,4	35,4	18,95	547,52	119,88
2019/05/21	CH2205	Male	Three y/o	Treatment (Wk 1)	Treatment	Feed Day	No	20:15	20:00 - 20:59	Night	37,2	109	4,4	4,4	18,36	600,04	49,12
2019/05/21	CH2206	Male	Three y/o	Treatment (Wk 1)	Treatment	Feed Day	No	20:15	20:00 - 20:59	Night	37,9	96	41,4	41,4	19,05	590,91	125,27
2019/05/21	CH2205	Male	Three y/o	Treatment (Wk 1)	Treatment	Feed Day	No	20:20	20:00 - 20:59	Night	37,0	65	5,6	5,6	18,16	561,12	57,21
2019/05/21	CH2206	Male	Three y/o	Treatment (Wk 1)	Treatment	Feed Day	No	20:20	20:00 - 20:59	Night	37,8	51	44,4	44,4	18,95	541,20	127,69
2019/05/21	CH2205	Male	Three y/o	Treatment (Wk 1)	Treatment	Feed Day	No	20:25	20:00 - 20:59	Night	36,8	57	5,0	5,0	17,96	550,47	53,41
2019/05/21	CH2206	Male	Three y/o	Treatment (Wk 1)	Treatment	Feed Day	No	20:25	20:00 - 20:59	Night	37,8	45	49,8	49,8	18,95	530,47	131,65
2019/05/21	CH2205	Male	Three y/o	Treatment (Wk 1)	Treatment	Feed Day	No	20:30	20:00 - 20:59	Night	36,8	57	6,6	6,6	17,96	550,47	62,73
2019/05/21	CH2206	Male	Three y/o	Treatment (Wk 1)	Treatment	Feed Day	No	20:30	20:00 - 20:59	Night	37,8	87	55,4	55,4	18,95	583,64	135,33
2019/05/21	CH2205	Male	Three y/o	Treatment (Wk 1)	Treatment	Feed Day	No	20:35	20:00 - 20:59	Night	36,8	93	11,6	11,6	17,96	588,58	81,78
2019/05/21	CH2206	Male	Three y/o	Treatment (Wk 1)	Treatment	Feed Day	No	20:35	20:00 - 20:59	Night	37,7	66	65,8	65,8	18,85	562,34	141,29
2019/05/21	CH2205	Male	Three y/o	Treatment (Wk 1)	Treatment	Feed Day	No	20:40	20:00 - 20:59	Night	36,8	118	3,4	3,4	17,96	605,61	40,51
2019/05/21	CH2206	Male	Three y/o	Treatment (Wk 1)	Treatment	Feed Day	No	20:40	20:00 - 20:59	Night	37,5	64	63,6	63,6	18,65	559,88	140,11
2019/05/21	CH2205	Male	Three y/o	Treatment (Wk 1)	Treatment	Feed Day	No	20:45	20:00 - 20:59	Night	36,8	55	7,6	7,6	17,96	547,52	67,49
2019/05/21	CH2206	Male	Three y/o	Treatment (Wk 1)	Treatment	Feed Day	No	20:45	20:00 - 20:59	Night	37,4	47	61,0	61,0	18,55	534,23	138,67
2019/05/21	CH2205	Male	Three y/o	Treatment (Wk 1)	Treatment	Feed Day	No	20:50	20:00 - 20:59	Night	36,7	119	9,4	9,4	17,87	606,20	74,66
2019/05/21	CH2206	Male	Three y/o	Treatment (Wk 1)	Treatment	Feed Day	No	20:50	20:00 - 20:59	Night	37,3	81	56,6	56,6	18,46	578,26	136,07
2019/05/21	CH2205	Male	Three y/o	Treatment (Wk 1)	Treatment	Feed Day	No	20:55	20:00 - 20:59	Night	37,0	112	30,6	30,6	18,16	601,96	114,87
2019/05/21	CH2206	Male	Three y/o	Treatment (Wk 1)	Treatment	Feed Day	No	20:55	20:00 - 20:59	Night	37,3	140	45,6	45,6	18,46	617,28	128,61
2019/05/21	CH2205	Male	Three y/o	Treatment (Wk 1)	Treatment	Feed Day	No	21:00	21:00 - 21:59	Night	37,0	93	20,6	20,6	18,16	588,58	101,32
2019/05/21	CH2206	Male	Three y/o	Treatment (Wk 1)	Treatment	Feed Day	No	21:00	21:00 - 21:59	Night	37,2	71	46,4	46,4	18,36	568,10	129,21
2019/05/21	CH2205	Male	Three y/o	Treatment (Wk 1)	Treatment	Feed Day	No	21:05	21:00 - 21:59	Night	37,1	91	21,6	21,6	18,26	586,98	102,94
2019/05/21	CH2206	Male	Three y/o	Treatment (Wk 1)	Treatment	Feed Day	No	21:05	21:00 - 21:59	Night	37,1	90	47,6	47,6	18,26	586,16	130,09
2019/05/21	CH2205	Male	Three y/o	Treatment (Wk 1)	Treatment	Feed Day	No	21:10	21:00 - 21:59	Night	37,1	60	23,4	23,4	18,26	554,67	105,68
2019/05/21	CH2206	Male	Three y/o	Treatment (Wk 1)	Treatment	Feed Day	No	21:10	21:00 - 21:59	Night	37,0	83	50,4	50,4	18,16	580,11	132,06
2019/05/21	CH2205	Male	Three y/o	Treatment (Wk 1)	Treatment	Feed Day	No	21:15	21:00 - 21:59	Night	37,1	59	22,6	22,6	18,26	553,30	104,49
2019/05/21	CH2206	Male	Three y/o	Treatment (Wk 1)	Treatment	Feed Day	No	21:15	21:00 - 21:59	Night	37,0	60	42,6	42,6	18,16	554,67	126,26
2019/05/21	CH2205	Male	Three y/o	Treatment (Wk 1)	Treatment	Feed Day	No	21:20	21:00 - 21:59	Night	37,2	62	22,6	22,6	18,36	557,33	104,49
2019/05/21	CH2206	Male	Three y/o	Treatment (Wk 1)	Treatment	Feed Day	No	21:20	21:00 - 21:59	Night	37,0	87	26,8	26,8	18,16	583,64	110,33
2019/05/21	CH2205	Male	Three y/o	Treatment (Wk 1)	Treatment	Feed Day	No	21:25	21:00 - 21:59	Night	37,2	54	24,2	24,2	18,36	545,99	106,83
2019/05/21	CH2206	Male	Three y/o	Treatment (Wk 1)	Treatment	Feed Day	No	21:25	21:00 - 21:59	Night	37,2	81	30,6	30,6	18,36	578,26	114,87
2019/05/21	CH2205	Male	Three y/o	Treatment (Wk 1)	Treatment	Feed Day	No	21:30	21:00 - 21:59	Night	37,2	51	26,4	26,4	18,36	541,20	109,81
2019/05/21	CH2206	Male	Three y/o	Treatment (Wk 1)	Treatment	Feed Day	No	21:30	21:00 - 21:59	Night	37,3	80	33,0	33,0	18,46	577,31	117,47
2019/05/21	CH2205	Male	Three y/o	Treatment (Wk 1)	Treatment	Feed Day	No	21:35	21:00 - 21:59	Night	37,2	63	33,6	33,6	18,36	558,62	118,09
2019/05/21	CH2206	Male	Three y/o	Treatment (Wk 1)	Treatment	Feed Day	No	21:35	21:00 - 21:59	Night	37,4	93	34,6	34,6	18,55	588,58	119,10
2019/05/21	CH2205	Male	Three y/o	Treatment (Wk 1)	Treatment	Feed Day	No	21:40	21:00 - 21:59	Night	37,3	83	48,4	48,4	18,46	580,11	130,66
2019/05/21	CH2206	Male	Three y/o	Treatment (Wk 1)	Treatment	Feed Day	No	21:40	21:00 - 21:59	Night	37,4		31,8	31,8	18,55		116,20
2019/05/21	CH2205	Male	Three y/o	Treatment (Wk 1)	Treatment	Feed Day	No	21:45	21:00 - 21:59	Night	37,4		21,8	21,8	18,55		103,26
2019/05/21	CH2206	Male	Three y/o	Treatment (Wk 1)	Treatment	Feed Day	No	21:45	21:00 - 21:59	Night	37,5	45	58,4	58,4	18,65	530,47	137,16
2019/05/21	CH2205	Male	Three y/o	Treatment (Wk 1)	Treatment	Feed Day	No	21:50	21:00 - 21:59	Night	37,4		30,8	30,8	18,55		115,10
2019/05/21	CH2206	Male	Three y/o	Treatment (Wk 1)	Treatment	Feed Day	No	21:50	21:00 - 21:59	Night	37,5	90	63,4	63,4	18,65	586,16	140,00
2019/05/21	CH2205	Male	Three y/o	Treatment (Wk 1)	Treatment	Feed Day	No	21:55	21:00 - 21:59	Night	37,5		28,2	28,2	18,65		112,07
2019/05/21	CH2206	Male	Three y/o	Treatment (Wk 1)	Treatment	Feed Day	No	21:55	21:00 - 21:59	Night	37,5	44	70,8	70,8	18,65	528,51	143,83
2019/05/21	CH2205	Male	Three y/o	Treatment (Wk 1)	Treatment	Feed Day	No	22:00	22:00 - 22:59	Night	37,6	95	17,6	17,6	18,75	590,14	95,95
2019/05/21	CH2206	Male	Three y/o	Treatment (Wk 1)	Treatment	Feed Day	No	22:00	22:00 - 22:59	Night	37,5	92	27,4	27,4	18,65	587,78	111,09
2019/05/21	CH2205	Male	Three y/o	Treatment (Wk 1)	Treatment	Feed Day	No	22:05	22:00 - 22:59	Night	37,7		11,6	11,6	18,85		81,78

	2019/05/21	CH2206	Male	Three y/o	Treatment (Wk 1)	Treatment	Feed Day	No	22:05	22:00 - 22:59	Night	37,5	63	25,8	25,8	18,65	558,62	109,02
	2019/05/21	CH2205	Male	Three y/o	Treatment (Wk 1)	Treatment	Feed Day	No	22:10	22:00 - 22:59	Night	37,9	49	20,4	20,4	19,05	537,80	100,99
	2019/05/21	CH2206	Male	Three y/o	Treatment (Wk 1)	Treatment	Feed Day	No	22:10	22:00 - 22:59	Night	37,5	62	24,2	24,2	18,65	557,33	106,83
	2019/05/21	CH2205	Male	Three y/o	Treatment (Wk 1)	Treatment	Feed Day	No	22:15	22:00 - 22:59	Night	37,9	44	13,2	13,2	19,05	528,51	86,17
	2019/05/21	CH2206	Male	Three y/o	Treatment (Wk 1)	Treatment	Feed Day	No	22:15	22:00 - 22:59	Night	37,6	80	27,6	27,6	18,75	577,31	111,33
	2019/05/21	CH2205	Male	Three y/o	Treatment (Wk 1)	Treatment	Feed Day	No	22:20	22:00 - 22:59	Night	37,9	51	13,2	13,2	19,05	541,20	86,17
	2019/05/21	CH2206	Male	Three y/o	Treatment (Wk 1)	Treatment	Feed Day	No	22:20	22:00 - 22:59	Night	37,7	81	26,2	26,2	18,85	578,26	109,55
	2019/05/21	CH2205	Male	Three y/o	Treatment (Wk 1)	Treatment	Feed Day	No	22:25	22:00 - 22:59	Night	37,9	47	25,8	25,8	19,05	534,23	109,02
	2019/05/21	CH2206	Male	Three y/o	Treatment (Wk 1)	Treatment	Feed Day	No	22:25	22:00 - 22:59	Night	37,7	45	20,6	20,6	18,85	530,47	101,32
	2019/05/21	CH2205	Male	Three y/o	Treatment (Wk 1)	Treatment	Feed Day	No	22:30	22:00 - 22:59	Night	37,9	67	15,8	15,8	19,05	563,53	92,28
	2019/05/21	CH2206	Male	Three y/o	Treatment (Wk 1)	Treatment	Feed Day	No	22:30	22:00 - 22:59	Night	37,7	82	24,6	24,6	18,85	579,19	107,39
	2019/05/21	CH2205	Male	Three y/o	Treatment (Wk 1)	Treatment	Feed Day	No	22:35	22:00 - 22:59	Night	37,9	104	15,8	15,8	19,05	596,69	92,28
	2019/05/21	CH2206	Male	Three y/o	Treatment (Wk 1)	Treatment	Feed Day	No	22:35	22:00 - 22:59	Night	37,7	56	26,6	26,6	18,85	549,01	110,07
	2019/05/21	CH2205	Male	Three y/o	Treatment (Wk 1)	Treatment	Feed Day	No	22:40	22:00 - 22:59	Night	37,9	79	18,4	18,4	19,05	576,36	97,47
	2019/05/21	CH2206	Male	Three y/o	Treatment (Wk 1)	Treatment	Feed Day	No	22:40	22:00 - 22:59	Night	37,7	45	31,2	31,2	18,85	530,47	115,54
	2019/05/21	CH2205	Male	Three y/o	Treatment (Wk 1)	Treatment	Feed Day	No	22:45	22:00 - 22:59	Night	37,9	47	18,8	18,8	19,05	534,23	98,20
	2019/05/21	CH2206	Male	Three y/o	Treatment (Wk 1)	Treatment	Feed Day	No	22:45	22:00 - 22:59	Night	37,7	86	28,6	28,6	18,85	582,77	112,56
	2019/05/21	CH2205	Male	Three y/o	Treatment (Wk 1)	Treatment	Feed Day	No	22:50	22:00 - 22:59	Night	37,9	61	20,6	20,6	19,05	556,01	101,32
	2019/05/21	CH2206	Male	Three y/o	Treatment (Wk 1)	Treatment	Feed Day	No	22:50	22:00 - 22:59	Night	37,8	82	17,8	17,8	18,95	579,19	96,34
	2019/05/21	CH2205	Male	Three y/o	Treatment (Wk 1)	Treatment	Feed Day	No	22:55	22:00 - 22:59	Night	37,9	93	19,2	19,2	19,05	588,58	98,92
	2019/05/21	CH2206	Male	Three y/o	Treatment (Wk 1)	Treatment	Feed Day	No	22:55	22:00 - 22:59	Night	37,8	42	17,8	17,8	18,95	524,42	96,34
	2019/05/21	CH2205	Male	Three y/o	Treatment (Wk 1)	Treatment	Feed Day	No	23:00	23:00 - 23:59	Night	37,9	64	19,6	19,6	19,05	559,88	99,63
	2019/05/21	CH2206	Male	Three y/o	Treatment (Wk 1)	Treatment	Feed Day	No	23:00	23:00 - 23:59	Night	37,8	65	17,4	17,4	18,95	561,12	95,56
	2019/05/21	CH2205	Male	Three y/o	Treatment (Wk 1)	Treatment	Feed Day	No	23:05	23:00 - 23:59	Night	37,9	59	20,0	20,0	19,05	553,30	100,31
	2019/05/21	CH2206	Male	Three y/o	Treatment (Wk 1)	Treatment	Feed Day	No	23:05	23:00 - 23:59	Night	37,8	80	18,4	18,4	18,95	577,31	97,47
	2019/05/21	CH2205	Male	Three y/o	Treatment (Wk 1)	Treatment	Feed Day	No	23:10	23:00 - 23:59	Night	37,9	86	20,8	20,8	19,05	582,77	101,65
	2019/05/21	CH2206	Male	Three y/o	Treatment (Wk 1)	Treatment	Feed Day	No	23:10	23:00 - 23:59	Night	37,8	50	19,0	19,0	18,95	539,52	98,56
	2019/05/21	CH2205	Male	Three y/o	Treatment (Wk 1)	Treatment	Feed Day	No	23:15	23:00 - 23:59	Night	37,9	47	23,8	23,8	19,05	534,23	106,26
	2019/05/21	CH2206	Male	Three y/o	Treatment (Wk 1)	Treatment	Feed Day	No	23:15	23:00 - 23:59	Night	37,8	79	14,2	14,2	18,95	576,36	88,65
	2019/05/21	CH2205	Male	Three y/o	Treatment (Wk 1)	Treatment	Feed Day	No	23:20	23:00 - 23:59	Night	37,9	59	37,0	37,0	19,05	553,30	121,40
	2019/05/21	CH2206	Male	Three y/o	Treatment (Wk 1)	Treatment	Feed Day	No	23:20	23:00 - 23:59	Night	37,8	106	67,8	67,8	18,95	598,06	142,33
	2019/05/21	CH2205	Male	Three y/o	Treatment (Wk 1)	Treatment	Feed Day	No	23:25	23:00 - 23:59	Night	37,7	94	15,2	15,2	18,85	589,37	90,96
	2019/05/21	CH2206	Male	Three y/o	Treatment (Wk 1)	Treatment	Feed Day	No	23:25	23:00 - 23:59	Night	37,7	47	39,0	39,0	18,85	534,23	123,22
	2019/05/21	CH2205	Male	Three y/o	Treatment (Wk 1)	Treatment	Feed Day	No	23:30	23:00 - 23:59	Night	37,7	58	9,0	9,0	18,85	551,90	73,19
	2019/05/21	CH2206	Male	Three y/o	Treatment (Wk 1)	Treatment	Feed Day	No	23:30	23:00 - 23:59	Night	37,7	86	20,0	20,0	18,85	582,77	100,31
	2019/05/21	CH2205	Male	Three y/o	Treatment (Wk 1)	Treatment	Feed Day	No	23:35	23:00 - 23:59	Night	37,7	77	11,0	11,0	18,85	574,39	79,98
	2019/05/21	CH2206	Male	Three y/o	Treatment (Wk 1)	Treatment	Feed Day	No	23:35	23:00 - 23:59	Night	37,7	46	19,6	19,6	18,85	532,38	99,63
	2019/05/21	CH2205	Male	Three y/o	Treatment (Wk 1)	Treatment	Feed Day	No	23:40	23:00 - 23:59	Night	37,7	48	11,6	11,6	18,85	536,04	81,78
	2019/05/21	CH2206	Male	Three y/o	Treatment (Wk 1)	Treatment	Feed Day	No	23:40	23:00 - 23:59	Night	37,8	82	14,4	14,4	18,95	579,19	89,12
	2019/05/21	CH2205	Male	Three y/o	Treatment (Wk 1)	Treatment	Feed Day	No	23:45	23:00 - 23:59	Night	37,7	62	10,0	10,0	18,85	557,33	76,76
	2019/05/21	CH2206	Male	Three y/o	Treatment (Wk 1)	Treatment	Feed Day	No	23:45	23:00 - 23:59	Night	37,8	68	2,6	2,6	18,95	564,71	31,57
	2019/05/21	CH2205	Male	Three y/o	Treatment (Wk 1)	Treatment	Feed Day	No	23:50	23:00 - 23:59	Night	37,7		30,2	30,2	18,85		114,42
	2019/05/21	CH2206	Male	Three y/o	Treatment (Wk 1)	Treatment	Feed Day	No	23:50	23:00 - 23:59	Night	37,8	88	10,4	10,4	18,95	584,49	78,08
	2019/05/21	CH2205	Male	Three y/o	Treatment (Wk 1)	Treatment	Feed Day	No	23:55	23:00 - 23:59	Night	37,9	63	7,6	7,6	19,05	558,62	67,49
	2019/05/21	CH2206	Male	Three y/o	Treatment (Wk 1)	Treatment	Feed Day	No	23:55	23:00 - 23:59	Night	37,8	46	13,8	13,8	18,95	532,38	87,68
8	2019/05/22	CH2205	Male	Three y/o	Treatment (Wk 2)	Treatment	Fast Day	No	00:00	00:00 - 00:59	Early Morning	37,9	58	7,4	7,4	19,05	551,90	66,59
	2019/05/22	CH2206	Male	Three y/o	Treatment (Wk 2)	Treatment	Fast Day	No	00:00	00:00 - 00:59	Early Morning	37,8	86	12,4	12,4	18,95	582,77	84,04
	2019/05/22	CH2205	Male	Three y/o	Treatment (Wk 2)	Treatment	Fast Day	No	00:05	00:00 - 00:59	Early Morning	37,8	52	7,0	7,0	18,95	542,83	64,72
	2019/05/22	CH2206	Male	Three y/o	Treatment (Wk 2)	Treatment	Fast Day	No	00:05	00:00 - 00:59	Early Morning	37,8	90	10,2	10,2	18,95	586,16	77,43
	2019/05/22	CH2205	Male	Three y/o	Treatment (Wk 2)	Treatment	Fast Day	No	00:10	00:00 - 00:59	Early Morning	37,7	92	7,4	7,4	18,85	587,78	66,59
	2019/05/22	CH2206	Male	Three y/o	Treatment (Wk 2)	Treatment	Fast Day	No	00:10	00:00 - 00:59	Early Morning	37,8	99	14,8	14,8	18,95	593,14	90,05
	2019/05/22	CH2205	Male	Three y/o	Treatment (Wk 2)	Treatment	Fast Day	No	00:15	00:00 - 00:59	Early Morning	37,7	45	8,2	8,2	18,85	530,47	70,05
	2019/05/22	CH2206	Male	Three y/o	Treatment (Wk 2)	Treatment	Fast Day	No	00:15	00:00 - 00:59	Early Morning	37,8	82	10,0	10,0	18,95	579,19	76,76
	2019/05/22	CH2205	Male	Three y/o	Treatment (Wk 2)	Treatment	Fast Day	No	00:20	00:00 - 00:59	Early Morning	37,7	59	8,2	8,2	18,85	553,30	70,05
	2019/05/22	CH2206	Male	Three y/o	Treatment (Wk 2)	Treatment	Fast Day	No	00:20	00:00 - 00:59	Early Morning	37,8	55	11,8	11,8	18,95	547,52	82,36
	2019/05/22	CH2205	Male	Three y/o	Treatment (Wk 2)	Treatment	Fast Day	No	00:25	00:00 - 00:59	Early Morning	37,7		4,0	4,0	18,85		45,94
	2019/05/22	CH2206	Male	Three y/o	Treatment (Wk 2)	Treatment	Fast Day	No	00:25	00:00 - 00:59	Early Morning	37,8	100	4,2	4,2	18,95	593,87	47,57
	2019/05/22	CH2205	Male	Three y/o	Treatment (Wk 2)	Treatment	Fast Day	No	00:30	00:00 - 00:59	Early Morning	37,7	103	7,0	7,0	18,85	596,00	64,72
	2019/05/22	CH2206	Male	Three y/o	Treatment (Wk 2)	Treatment	Fast Day	No	00:30	00:00 - 00:59	Early Morning	37,8	104	3,6	3,6	18,95	596,69	42,42
	2019/05/22	CH2205	Male	Three y/o	Treatment (Wk 2)	Treatment	Fast Day	No	00:35	00:00 - 00:59	Early Morning	37,7	86	8,4	8,4	18,85	582,77	70,86
	2019/05/22	CH2206	Male	Three y/o	Treatment (Wk 2)	Treatment	Fast Day	No	00:35	00:00 - 00:59	Early Morning	37,8	54	5,4	5,4	18,95	545,99	55,99
	2019/05/22	CH2205	Male	Three y/o	Treatment (Wk 2)	Treatment	Fast Day	No	00:40	00:00 - 00:59	Early Morning	37,7	55	9,2	9,2	18,85	547,52	73,94
	2019/05/22	CH2206	Male	Three y/o	Treatment (Wk 2)	Treatment	Fast Day	No	00:40	00:00 - 00:59	Early Morning	37,8	43	4,4	4,4	18,95	526,50	49,12
	2019/05/22	CH2205	Male	Three y/o	Treatment (Wk 2)	Treatment	Fast Day	No	00:45	00:00 - 00:59	Early Morning	37,7	61	5,6	5,6	18,85	556,01	57,21
	2019/05/22	CH2206	Male	Three y/o	Treatment (Wk 2)	Treatment	Fast Day	No	00:45	00:00 - 00:59	Early Morning	37,8	84	7,8	7,8	18,95	581,01	68,36
	2019/05/22	CH2205	Male	Three y/o	Treatment (Wk 2)	Treatment	Fast Day	No	00:50	00:00 - 00:59	Early Morning	37,7	99	2,4	2,4	18,85	593,14	28,91
	2019/05/22	CH2206	Male	Three y/o	Treatment (Wk 2)	Treatment	Fast Day	No	00:50	00:00 - 00:59	Early Morning	37,8	83	4,8	4,8	18,95	580,11	52,04
	2019/05/22	CH2205	Male	Three y/o	Treatment (Wk 2)	Treatment	Fast Day	No	00:55	00:00 - 00:59	Early Morning	37,7	55	7,4	7,4	18,85	547,52	66,59
	2019/05/22	CH2206	Male	Three y/o	Treatment (Wk 2)	Treatment	Fast Day	No	00:55	00:00 - 00:59	Early Morning	37,8	84	4,8	4,8	18,95	581,01	52,04
	2019/05/22	CH2205	Male	Three y/o	Treatment (Wk 2)	Treatment	Fast Day	No	01:00	01:00 - 01:59	Early Morning	37,7	58	14,2	14,2	18,85	551,90	88,65

2019/05/22	CH2206	Male	Three y/o	Treatment (Wk 2)	Treatment	Fast Day	No	01:00	01:00 - 01:59	Early Morning	37,8	79	3,6	3,6	18,95	576,36	42,42
2019/05/22	CH2205	Male	Three y/o	Treatment (Wk 2)	Treatment	Fast Day	No	01:05	01:00 - 01:59	Early Morning	37,7	99	11,4	11,4	18,85	593,14	81,19
2019/05/22	CH2206	Male	Three y/o	Treatment (Wk 2)	Treatment	Fast Day	No	01:05	01:00 - 01:59	Early Morning	37,8	56	1,2	1,2	18,95	549,01	5,99
2019/05/22	CH2205	Male	Three y/o	Treatment (Wk 2)	Treatment	Fast Day	No	01:10	01:00 - 01:59	Early Morning	37,7	90	10,4	10,4	18,85	586,16	78,08
2019/05/22	CH2206	Male	Three y/o	Treatment (Wk 2)	Treatment	Fast Day	No	01:10	01:00 - 01:59	Early Morning	37,8	76	1,2	1,2	18,95	573,39	5,99
2019/05/22	CH2205	Male	Three y/o	Treatment (Wk 2)	Treatment	Fast Day	No	01:15	01:00 - 01:59	Early Morning	37,7	61	13,0	13,0	18,85	556,01	85,65
2019/05/22	CH2206	Male	Three y/o	Treatment (Wk 2)	Treatment	Fast Day	No	01:15	01:00 - 01:59	Early Morning	37,8	91	2,6	2,6	18,95	586,98	31,57
2019/05/22	CH2205	Male	Three y/o	Treatment (Wk 2)	Treatment	Fast Day	No	01:20	01:00 - 01:59	Early Morning	37,7	57	8,2	8,2	18,85	550,47	70,05
2019/05/22	CH2206	Male	Three y/o	Treatment (Wk 2)	Treatment	Fast Day	No	01:20	01:00 - 01:59	Early Morning	37,8	79	7,2	7,2	18,95	576,36	65,66
2019/05/22	CH2205	Male	Three y/o	Treatment (Wk 2)	Treatment	Fast Day	No	01:25	01:00 - 01:59	Early Morning	37,7	45	13,0	13,0	18,85	530,47	85,65
2019/05/22	CH2206	Male	Three y/o	Treatment (Wk 2)	Treatment	Fast Day	No	01:25	01:00 - 01:59	Early Morning	37,8	44	13,4	13,4	18,95	528,51	86,68
2019/05/22	CH2205	Male	Three y/o	Treatment (Wk 2)	Treatment	Fast Day	No	01:30	01:00 - 01:59	Early Morning	37,7	51	9,8	9,8	18,85	541,20	76,07
2019/05/22	CH2206	Male	Three y/o	Treatment (Wk 2)	Treatment	Fast Day	No	01:30	01:00 - 01:59	Early Morning	37,8	86	11,2	11,2	18,95	582,77	80,59
2019/05/22	CH2205	Male	Three y/o	Treatment (Wk 2)	Treatment	Fast Day	No	01:35	01:00 - 01:59	Early Morning	37,7	90	9,6	9,6	18,85	586,16	75,37
2019/05/22	CH2206	Male	Three y/o	Treatment (Wk 2)	Treatment	Fast Day	No	01:35	01:00 - 01:59	Early Morning	37,8	94	10,6	10,6	18,95	589,37	78,73
2019/05/22	CH2205	Male	Three y/o	Treatment (Wk 2)	Treatment	Fast Day	No	01:40	01:00 - 01:59	Early Morning	37,7	48	11,4	11,4	18,85	536,04	81,19
2019/05/22	CH2206	Male	Three y/o	Treatment (Wk 2)	Treatment	Fast Day	No	01:40	01:00 - 01:59	Early Morning	37,8	93	10,2	10,2	18,95	588,58	77,43
2019/05/22	CH2205	Male	Three y/o	Treatment (Wk 2)	Treatment	Fast Day	No	01:45	01:00 - 01:59	Early Morning	37,7	75	13,2	13,2	18,85	572,36	86,17
2019/05/22	CH2206	Male	Three y/o	Treatment (Wk 2)	Treatment	Fast Day	No	01:45	01:00 - 01:59	Early Morning	37,8	76	13,2	13,2	18,95	573,39	86,17
2019/05/22	CH2205	Male	Three y/o	Treatment (Wk 2)	Treatment	Fast Day	No	01:50	01:00 - 01:59	Early Morning	37,7	48	9,4	9,4	18,85	536,04	74,66
2019/05/22	CH2206	Male	Three y/o	Treatment (Wk 2)	Treatment	Fast Day	No	01:50	01:00 - 01:59	Early Morning	37,8	50	14,6	14,6	18,95	539,52	89,59
2019/05/22	CH2205	Male	Three y/o	Treatment (Wk 2)	Treatment	Fast Day	No	01:55	01:00 - 01:59	Early Morning	37,7	55	13,2	13,2	18,85	547,52	86,17
2019/05/22	CH2206	Male	Three y/o	Treatment (Wk 2)	Treatment	Fast Day	No	01:55	01:00 - 01:59	Early Morning	37,8	79	12,6	12,6	18,95	576,36	84,59
2019/05/22	CH2205	Male	Three y/o	Treatment (Wk 2)	Treatment	Fast Day	No	02:00	02:00 - 02:59	Early Morning	37,7	94	15,4	15,4	18,85	589,37	91,41
2019/05/22	CH2206	Male	Three y/o	Treatment (Wk 2)	Treatment	Fast Day	No	02:00	02:00 - 02:59	Early Morning	37,8	62	8,6	8,6	18,95	557,33	71,66
2019/05/22	CH2205	Male	Three y/o	Treatment (Wk 2)	Treatment	Fast Day	No	02:05	02:00 - 02:59	Early Morning	37,7	46	16,2	16,2	18,85	532,38	93,13
2019/05/22	CH2206	Male	Three y/o	Treatment (Wk 2)	Treatment	Fast Day	No	02:05	02:00 - 02:59	Early Morning	37,8	45	11,0	11,0	18,95	530,47	79,98
2019/05/22	CH2205	Male	Three y/o	Treatment (Wk 2)	Treatment	Fast Day	No	02:10	02:00 - 02:59	Early Morning	37,7	57	31,2	31,2	18,85	550,47	115,54
2019/05/22	CH2206	Male	Three y/o	Treatment (Wk 2)	Treatment	Fast Day	No	02:10	02:00 - 02:59	Early Morning	37,8		59,0	59,0	18,95		137,51
2019/05/22	CH2205	Male	Three y/o	Treatment (Wk 2)	Treatment	Fast Day	No	02:15	02:00 - 02:59	Early Morning	37,7	88	49,6	49,6	18,85	584,49	131,51
2019/05/22	CH2206	Male	Three y/o	Treatment (Wk 2)	Treatment	Fast Day	No	02:15	02:00 - 02:59	Early Morning	37,8	91	58,8	58,8	18,95	586,98	137,39
2019/05/22	CH2205	Male	Three y/o	Treatment (Wk 2)	Treatment	Fast Day	No	02:20	02:00 - 02:59	Early Morning	37,6	92	28,8	28,8	18,75	587,78	112,79
2019/05/22	CH2206	Male	Three y/o	Treatment (Wk 2)	Treatment	Fast Day	No	02:20	02:00 - 02:59	Early Morning	37,8	84	39,4	39,4	18,95	581,01	123,57
2019/05/22	CH2205	Male	Three y/o	Treatment (Wk 2)	Treatment	Fast Day	No	02:25	02:00 - 02:59	Early Morning	37,5	50	22,6	22,6	18,65	539,52	104,49
2019/05/22	CH2206	Male	Three y/o	Treatment (Wk 2)	Treatment	Fast Day	No	02:25	02:00 - 02:59	Early Morning	37,7	51	33,4	33,4	18,85	541,20	117,88
2019/05/22	CH2205	Male	Three y/o	Treatment (Wk 2)	Treatment	Fast Day	No	02:30	02:00 - 02:59	Early Morning	37,5	85	5,6	5,6	18,65	581,90	57,21
2019/05/22	CH2206	Male	Three y/o	Treatment (Wk 2)	Treatment	Fast Day	No	02:30	02:00 - 02:59	Early Morning	37,5	61	12,0	12,0	18,65	556,01	82,93
2019/05/22	CH2205	Male	Three y/o	Treatment (Wk 2)	Treatment	Fast Day	No	02:35	02:00 - 02:59	Early Morning	37,5	84	3,8	3,8	18,65	581,01	44,22
2019/05/22	CH2206	Male	Three y/o	Treatment (Wk 2)	Treatment	Fast Day	No	02:35	02:00 - 02:59	Early Morning	37,5	48	24,2	24,2	18,65	536,04	106,83
2019/05/22	CH2205	Male	Three y/o	Treatment (Wk 2)	Treatment	Fast Day	No	02:40	02:00 - 02:59	Early Morning	37,4	55	5,4	5,4	18,55	547,52	55,99
2019/05/22	CH2206	Male	Three y/o	Treatment (Wk 2)	Treatment	Fast Day	No	02:40	02:00 - 02:59	Early Morning	37,5	83	13,8	13,8	18,65	580,11	87,68
2019/05/22	CH2205	Male	Three y/o	Treatment (Wk 2)	Treatment	Fast Day	No	02:45	02:00 - 02:59	Early Morning	37,5	62	14,2	14,2	18,65	557,33	88,65
2019/05/22	CH2206	Male	Three y/o	Treatment (Wk 2)	Treatment	Fast Day	No	02:45	02:00 - 02:59	Early Morning	37,6	74	7,8	7,8	18,75	571,33	68,36
2019/05/22	CH2205	Male	Three y/o	Treatment (Wk 2)	Treatment	Fast Day	No	02:50	02:00 - 02:59	Early Morning	37,5	83	9,2	9,2	18,65	580,11	73,94
2019/05/22	CH2206	Male	Three y/o	Treatment (Wk 2)	Treatment	Fast Day	No	02:50	02:00 - 02:59	Early Morning	37,5	76	13,2	13,2	18,65	573,39	86,17
2019/05/22	CH2205	Male	Three y/o	Treatment (Wk 2)	Treatment	Fast Day	No	02:55	02:00 - 02:59	Early Morning	37,5	91	18,4	18,4	18,65	586,98	97,47
2019/05/22	CH2206	Male	Three y/o	Treatment (Wk 2)	Treatment	Fast Day	No	02:55	02:00 - 02:59	Early Morning	37,5	60	10,2	10,2	18,65	554,67	77,43
2019/05/22	CH2205	Male	Three y/o	Treatment (Wk 2)	Treatment	Fast Day	No	03:00	03:00 - 03:59	Early Morning	37,5	68	16,4	16,4	18,65	564,71	93,55
2019/05/22	CH2206	Male	Three y/o	Treatment (Wk 2)	Treatment	Fast Day	No	03:00	03:00 - 03:59	Early Morning	37,5	61	10,0	10,0	18,65	556,01	76,76
2019/05/22	CH2205	Male	Three y/o	Treatment (Wk 2)	Treatment	Fast Day	No	03:05	03:00 - 03:59	Early Morning	37,5	75	15,0	15,0	18,65	572,36	90,51
2019/05/22	CH2206	Male	Three y/o	Treatment (Wk 2)	Treatment	Fast Day	No	03:05	03:00 - 03:59	Early Morning	37,5	58	11,6	11,6	18,65	551,90	81,78
2019/05/22	CH2205	Male	Three y/o	Treatment (Wk 2)	Treatment	Fast Day	No	03:10	03:00 - 03:59	Early Morning	37,6	52	19,2	19,2	18,75	542,83	98,92
2019/05/22	CH2206	Male	Three y/o	Treatment (Wk 2)	Treatment	Fast Day	No	03:10	03:00 - 03:59	Early Morning	37,5	59	17,0	17,0	18,65	553,30	94,77
2019/05/22	CH2205	Male	Three y/o	Treatment (Wk 2)	Treatment	Fast Day	No	03:15	03:00 - 03:59	Early Morning	37,6	83	16,2	16,2	18,75	580,11	93,13
2019/05/22	CH2206	Male	Three y/o	Treatment (Wk 2)	Treatment	Fast Day	No	03:15	03:00 - 03:59	Early Morning	37,5	50	9,6	9,6	18,65	539,52	75,37
2019/05/22	CH2205	Male	Three y/o	Treatment (Wk 2)	Treatment	Fast Day	No	03:20	03:00 - 03:59	Early Morning	37,6	58	10,2	10,2	18,75	551,90	77,43
2019/05/22	CH2206	Male	Three y/o	Treatment (Wk 2)	Treatment	Fast Day	No	03:20	03:00 - 03:59	Early Morning	37,5	81	9,8	9,8	18,65	578,26	76,07
2019/05/22	CH2205	Male	Three y/o	Treatment (Wk 2)	Treatment	Fast Day	No	03:25	03:00 - 03:59	Early Morning	37,6	70	14,0	14,0	18,75	566,99	88,17
2019/05/22	CH2206	Male	Three y/o	Treatment (Wk 2)	Treatment	Fast Day	No	03:25	03:00 - 03:59	Early Morning	37,4	92	13,0	13,0	18,55	587,78	85,65
2019/05/22	CH2205	Male	Three y/o	Treatment (Wk 2)	Treatment	Fast Day	No	03:30	03:00 - 03:59	Early Morning	37,6	78	2,6	2,6	18,75	575,38	31,57
2019/05/22	CH2206	Male	Three y/o	Treatment (Wk 2)	Treatment	Fast Day	No	03:30	03:00 - 03:59	Early Morning	37,4	48	4,6	4,6	18,55	536,04	50,61
2019/05/22	CH2205	Male	Three y/o	Treatment (Wk 2)	Treatment	Fast Day	No	03:35	03:00 - 03:59	Early Morning	37,5	59	14,0	14,0	18,65	553,30	88,17
2019/05/22	CH2206	Male	Three y/o	Treatment (Wk 2)	Treatment	Fast Day	No	03:35	03:00 - 03:59	Early Morning	37,5	57	6,6	6,6	18,65	550,47	62,73
2019/05/22	CH2205	Male	Three y/o	Treatment (Wk 2)	Treatment	Fast Day	No	03:40	03:00 - 03:59	Early Morning	37,5	69	10,6	10,6	18,65	565,86	78,73
2019/05/22	CH2206	Male	Three y/o	Treatment (Wk 2)	Treatment	Fast Day	No	03:40	03:00 - 03:59	Early Morning	37,4	80	5,6	5,6	18,55	577,31	57,21
2019/05/22	CH2205	Male	Three y/o	Treatment (Wk 2)	Treatment	Fast Day	No	03:45	03:00 - 03:59	Early Morning	37,5	82	7,0	7,0	18,65	579,19	64,72
2019/05/22	CH2206	Male	Three y/o	Treatment (Wk 2)	Treatment	Fast Day	No	03:45	03:00 - 03:59	Early Morning	37,4	45	3,6	3,6	18,55	530,47	42,42
2019/05/22	CH2205	Male	Three y/o	Treatment (Wk 2)	Treatment	Fast Day	No	03:50	03:00 - 03:59	Early Morning	37,5	55	6,4	6,4	18,65	547,52	61,70
2019/05/22	CH2206	Male	Three y/o	Treatment (Wk 2)	Treatment	Fast Day	No	03:50	03:00 - 03:59	Early Morning	37,3	105	18,4	18,4	18,46	597,38	97,47
2019/05/22	CH2205	Male	Three y/o	Treatment (Wk 2)	Treatment	Fast Day	No	03:55	03:00 - 03:59	Early Morning	37,4	49	10,2	10,2	18,55	537,80	77,43

2019/05/22	CH2206	Male	Three y/o	Treatment (Wk 2)	Treatment	Fast Day	No	03:55	03:00 - 03:59	Early Morning	37,3	90	8,4	8,4	18,46	586,16	70,86
2019/05/22	CH2205	Male	Three y/o	Treatment (Wk 2)	Treatment	Fast Day	No	04:00	04:00 - 04:59	Morning	37,4	49	15,4	15,4	18,55	537,80	91,41
2019/05/22	CH2206	Male	Three y/o	Treatment (Wk 2)	Treatment	Fast Day	No	04:00	04:00 - 04:59	Morning	37,3	71	7,4	7,4	18,46	568,10	66,59
2019/05/22	CH2205	Male	Three y/o	Treatment (Wk 2)	Treatment	Fast Day	No	04:05	04:00 - 04:59	Morning	37,4	65	12,2	12,2	18,55	561,12	83,49
2019/05/22	CH2206	Male	Three y/o	Treatment (Wk 2)	Treatment	Fast Day	No	04:05	04:00 - 04:59	Morning	37,2	79	5,2	5,2	18,36	576,36	54,72
2019/05/22	CH2205	Male	Three y/o	Treatment (Wk 2)	Treatment	Fast Day	No	04:10	04:00 - 04:59	Morning	37,4	51	19,2	19,2	18,55	541,20	98,92
2019/05/22	CH2206	Male	Three y/o	Treatment (Wk 2)	Treatment	Fast Day	No	04:10	04:00 - 04:59	Morning	37,2	80	9,8	9,8	18,36	577,31	76,07
2019/05/22	CH2205	Male	Three y/o	Treatment (Wk 2)	Treatment	Fast Day	No	04:15	04:00 - 04:59	Morning	37,4	56	14,0	14,0	18,55	549,01	88,17
2019/05/22	CH2206	Male	Three y/o	Treatment (Wk 2)	Treatment	Fast Day	No	04:15	04:00 - 04:59	Morning	37,3	86	15,2	15,2	18,46	582,77	90,96
2019/05/22	CH2205	Male	Three y/o	Treatment (Wk 2)	Treatment	Fast Day	No	04:20	04:00 - 04:59	Morning	37,4	90	22,0	22,0	18,55	586,16	103,57
2019/05/22	CH2206	Male	Three y/o	Treatment (Wk 2)	Treatment	Fast Day	No	04:20	04:00 - 04:59	Morning	37,3	50	20,2	20,2	18,46	539,52	100,65
2019/05/22	CH2205	Male	Three y/o	Treatment (Wk 2)	Treatment	Fast Day	No	04:25	04:00 - 04:59	Morning	37,5	110	196,0	196,0	18,65	600,69	179,41
2019/05/22	CH2206	Male	Three y/o	Treatment (Wk 2)	Treatment	Fast Day	No	04:25	04:00 - 04:59	Morning	37,3	134	54,4	54,4	18,46	614,33	134,70
2019/05/22	CH2205	Male	Three y/o	Treatment (Wk 2)	Treatment	Fast Day	No	04:30	04:00 - 04:59	Morning	37,4		111,0	111,0	18,55		159,48
2019/05/22	CH2206	Male	Three y/o	Treatment (Wk 2)	Treatment	Fast Day	No	04:30	04:00 - 04:59	Morning	37,2	169	85,4	85,4	18,36	629,60	150,35
2019/05/22	CH2205	Male	Three y/o	Treatment (Wk 2)	Treatment	Fast Day	No	04:35	04:00 - 04:59	Morning	37,3	120	307,4	307,4	18,46	606,78	195,28
2019/05/22	CH2206	Male	Three y/o	Treatment (Wk 2)	Treatment	Fast Day	No	04:35	04:00 - 04:59	Morning	37,3	126	135,8	135,8	18,46	610,14	166,53
2019/05/22	CH2205	Male	Three y/o	Treatment (Wk 2)	Treatment	Fast Day	No	04:40	04:00 - 04:59	Morning	37,3	103	185,2	185,2	18,46	596,00	177,42
2019/05/22	CH2206	Male	Three y/o	Treatment (Wk 2)	Treatment	Fast Day	No	04:40	04:00 - 04:59	Morning	37,3	131	48,6	48,6	18,46	612,80	130,81
2019/05/22	CH2205	Male	Three y/o	Treatment (Wk 2)	Treatment	Fast Day	No	04:45	04:00 - 04:59	Morning	37,3	40	184,0	184,0	18,46	520,09	177,19
2019/05/22	CH2206	Male	Three y/o	Treatment (Wk 2)	Treatment	Fast Day	No	04:45	04:00 - 04:59	Morning	37,2	134	85,6	85,6	18,36	614,33	150,43
2019/05/22	CH2205	Male	Three y/o	Treatment (Wk 2)	Treatment	Fast Day	No	04:50	04:00 - 04:59	Morning	37,4	76	82,6	82,6	18,55	573,39	149,19
2019/05/22	CH2206	Male	Three y/o	Treatment (Wk 2)	Treatment	Fast Day	No	04:50	04:00 - 04:59	Morning	36,9	90	58,6	58,6	18,06	586,16	137,28
2019/05/22	CH2205	Male	Three y/o	Treatment (Wk 2)	Treatment	Fast Day	No	04:55	04:00 - 04:59	Morning	37,4	99	107,0	107,0	18,55	593,14	158,20
2019/05/22	CH2206	Male	Three y/o	Treatment (Wk 2)	Treatment	Fast Day	No	04:55	04:00 - 04:59	Morning	36,8	88	61,0	61,0	17,96	584,49	138,67
2019/05/22	CH2205	Male	Three y/o	Treatment (Wk 2)	Treatment	Fast Day	No	05:00	05:00 - 05:59	Morning	37,5	150	19,0	19,0	18,65	621,86	98,56
2019/05/22	CH2206	Male	Three y/o	Treatment (Wk 2)	Treatment	Fast Day	No	05:00	05:00 - 05:59	Morning	36,8	68	63,0	63,0	17,96	564,71	139,78
2019/05/22	CH2205	Male	Three y/o	Treatment (Wk 2)	Treatment	Fast Day	No	05:05	05:00 - 05:59	Morning	37,5	188	162,4	162,4	18,65	636,34	172,80
2019/05/22	CH2206	Male	Three y/o	Treatment (Wk 2)	Treatment	Fast Day	No	05:05	05:00 - 05:59	Morning	36,9	57	62,4	62,4	18,06	550,47	139,45
2019/05/22	CH2205	Male	Three y/o	Treatment (Wk 2)	Treatment	Fast Day	No	05:10	05:00 - 05:59	Morning	37,6		288,6	288,6	18,75		193,05
2019/05/22	CH2206	Male	Three y/o	Treatment (Wk 2)	Treatment	Fast Day	No	05:10	05:00 - 05:59	Morning	36,9	46	58,0	58,0	18,06	532,38	136,92
2019/05/22	CH2205	Male	Three y/o	Treatment (Wk 2)	Treatment	Fast Day	No	05:15	05:00 - 05:59	Morning	37,5	70	27,6	27,6	18,65	566,99	111,33
2019/05/22	CH2206	Male	Three y/o	Treatment (Wk 2)	Treatment	Fast Day	No	05:15	05:00 - 05:59	Morning	36,9	59	53,6	53,6	18,06	553,30	134,19
2019/05/22	CH2205	Male	Three y/o	Treatment (Wk 2)	Treatment	Fast Day	No	05:20	05:00 - 05:59	Morning	37,3		33,4	33,4	18,46		117,88
2019/05/22	CH2206	Male	Three y/o	Treatment (Wk 2)	Treatment	Fast Day	No	05:20	05:00 - 05:59	Morning	37,0	84	39,2	39,2	18,16	581,01	123,39
2019/05/22	CH2205	Male	Three y/o	Treatment (Wk 2)	Treatment	Fast Day	No	05:25	05:00 - 05:59	Morning	37,1	68	5,8	5,8	18,26	564,71	58,39
2019/05/22	CH2206	Male	Three y/o	Treatment (Wk 2)	Treatment	Fast Day	No	05:25	05:00 - 05:59	Morning	37,0	94	56,0	56,0	18,16	589,37	135,70
2019/05/22	CH2205	Male	Three y/o	Treatment (Wk 2)	Treatment	Fast Day	No	05:30	05:00 - 05:59	Morning	36,8	56	10,4	10,4	17,96	549,01	78,08
2019/05/22	CH2206	Male	Three y/o	Treatment (Wk 2)	Treatment	Fast Day	No	05:30	05:00 - 05:59	Morning	36,8	90	48,8	48,8	17,96	586,16	130,95
2019/05/22	CH2205	Male	Three y/o	Treatment (Wk 2)	Treatment	Fast Day	No	05:35	05:00 - 05:59	Morning	36,8	48	10,6	10,6	17,96	536,04	78,73
2019/05/22	CH2206	Male	Three y/o	Treatment (Wk 2)	Treatment	Fast Day	No	05:35	05:00 - 05:59	Morning	36,6	62	42,8	42,8	17,77	557,33	126,42
2019/05/22	CH2205	Male	Three y/o	Treatment (Wk 2)	Treatment	Fast Day	No	05:40	05:00 - 05:59	Morning	37,1	48	3,6	3,6	18,26	536,04	42,42
2019/05/22	CH2206	Male	Three y/o	Treatment (Wk 2)	Treatment	Fast Day	No	05:40	05:00 - 05:59	Morning	36,6	47	42,6	42,6	17,77	534,23	126,26
2019/05/22	CH2205	Male	Three y/o	Treatment (Wk 2)	Treatment	Fast Day	No	05:45	05:00 - 05:59	Morning	37,3	71	3,4	3,4	18,46	568,10	40,51
2019/05/22	CH2206	Male	Three y/o	Treatment (Wk 2)	Treatment	Fast Day	No	05:45	05:00 - 05:59	Morning	36,6	51	51,2	51,2	17,77	541,20	132,61
2019/05/22	CH2205	Male	Three y/o	Treatment (Wk 2)	Treatment	Fast Day	No	05:50	05:00 - 05:59	Morning	37,3	154	15,2	15,2	18,46	623,58	90,96
2019/05/22	CH2206	Male	Three y/o	Treatment (Wk 2)	Treatment	Fast Day	No	05:50	05:00 - 05:59	Morning	36,8	131	17,6	17,6	17,96	612,80	95,95
2019/05/22	CH2205	Male	Three y/o	Treatment (Wk 2)	Treatment	Fast Day	No	05:55	05:00 - 05:59	Morning	37,3	74	17,4	17,4	18,46	571,33	95,56
2019/05/22	CH2206	Male	Three y/o	Treatment (Wk 2)	Treatment	Fast Day	No	05:55	05:00 - 05:59	Morning	37,1	59	35,2	35,2	18,26	553,30	119,69
2019/05/22	CH2205	Male	Three y/o	Treatment (Wk 2)	Treatment	Fast Day	No	06:00	06:00 - 06:59	Morning	37,3	42	13,8	13,8	18,46	524,42	87,68
2019/05/22	CH2206	Male	Three y/o	Treatment (Wk 2)	Treatment	Fast Day	No	06:00	06:00 - 06:59	Morning	37,1	51	41,2	41,2	18,26	541,20	125,11
2019/05/22	CH2205	Male	Three y/o	Treatment (Wk 2)	Treatment	Fast Day	No	06:05	06:00 - 06:59	Morning	37,3	56	13,0	13,0	18,46	549,01	85,65
2019/05/22	CH2206	Male	Three y/o	Treatment (Wk 2)	Treatment	Fast Day	No	06:05	06:00 - 06:59	Morning	37,2	45	39,8	39,8	18,36	530,47	123,91
2019/05/22	CH2205	Male	Three y/o	Treatment (Wk 2)	Treatment	Fast Day	No	06:10	06:00 - 06:59	Morning	37,3	46	9,0	9,0	18,46	532,38	73,19
2019/05/22	CH2206	Male	Three y/o	Treatment (Wk 2)	Treatment	Fast Day	No	06:10	06:00 - 06:59	Morning	37,2	42	46,4	46,4	18,36	524,42	129,21
2019/05/22	CH2205	Male	Three y/o	Treatment (Wk 2)	Treatment	Fast Day	No	06:15	06:00 - 06:59	Morning	37,2	46	9,2	9,2	18,36	532,38	73,94
2019/05/22	CH2206	Male	Three y/o	Treatment (Wk 2)	Treatment	Fast Day	No	06:15	06:00 - 06:59	Morning	37,2	83	53,8	53,8	18,36	580,11	134,32
2019/05/22	CH2205	Male	Three y/o	Treatment (Wk 2)	Treatment	Fast Day	No	06:20	06:00 - 06:59	Morning	37,2	49	7,8	7,8	18,36	537,80	68,36
2019/05/22	CH2206	Male	Three y/o	Treatment (Wk 2)	Treatment	Fast Day	No	06:20	06:00 - 06:59	Morning	37,2	56	50,4	50,4	18,36	549,01	132,06
2019/05/22	CH2205	Male	Three y/o	Treatment (Wk 2)	Treatment	Fast Day	No	06:25	06:00 - 06:59	Morning	37,2	44	18,4	18,4	18,36	528,51	97,47
2019/05/22	CH2206	Male	Three y/o	Treatment (Wk 2)	Treatment	Fast Day	No	06:25	06:00 - 06:59	Morning	37,2	49	44,8	44,8	18,36	537,80	127,99
2019/05/22	CH2205	Male	Three y/o	Treatment (Wk 2)	Treatment	Fast Day	No	06:30	06:00 - 06:59	Morning	37,2	140	53,8	53,8	18,36	617,28	134,32
2019/05/22	CH2206	Male	Three y/o	Treatment (Wk 2)	Treatment	Fast Day	No	06:30	06:00 - 06:59	Morning	37,3	141	67,8	67,8	18,46	617,75	142,33
2019/05/22	CH2205	Male	Three y/o	Treatment (Wk 2)	Treatment	Fast Day	No	06:35	06:00 - 06:59	Morning	37,2	77	49,2	49,2	18,36	574,39	131,23
2019/05/22	CH2206	Male	Three y/o	Treatment (Wk 2)	Treatment	Fast Day	No	06:35	06:00 - 06:59	Morning	37,3	147	106,0	106,0	18,46	620,52	157,88
2019/05/22	CH2205	Male	Three y/o	Treatment (Wk 2)	Treatment	Fast Day	No	06:40	06:00 - 06:59	Morning	37,3	132	40,6	40,6	18,46	613,32	124,60
2019/05/22	CH2206	Male	Three y/o	Treatment (Wk 2)	Treatment	Fast Day	No	06:40	06:00 - 06:59	Morning	37,4	141	123,2	123,2	18,55	617,75	163,13
2019/05/22	CH2205	Male	Three y/o	Treatment (Wk 2)	Treatment	Fast Day	No	06:45	06:00 - 06:59	Morning	37,3	129	103,6	103,6	18,46	611,75	157,08
2019/05/22	CH2206	Male	Three y/o	Treatment (Wk 2)	Treatment	Fast Day	No	06:45	06:00 - 06:59	Morning	37,4	120	579,4	579,4	18,55	606,78	217,80
2019/05/22	CH2205	Male	Three y/o	Treatment (Wk 2)	Treatment	Fast Day	No	06:50	06:00 - 06:59	Morning	37,3	103	221,8	221,8	18,46	596,00	183,76

2019/05/22	CH2206	Male	Three y/o	Treatment (Wk 2)	Treatment	Fast Day	No	06:50	06:00 - 06:59	Morning	37,5	119	52,0	52,0	18,65	606,20	133,14
2019/05/22	CH2205	Male	Three y/o	Treatment (Wk 2)	Treatment	Fast Day	No	06:55	06:00 - 06:59	Morning	37,4	66	346,8	346,8	18,55	562,34	199,55
2019/05/22	CH2206	Male	Three y/o	Treatment (Wk 2)	Treatment	Fast Day	No	06:55	06:00 - 06:59	Morning	37,5	124	290,4	290,4	18,65	609,04	193,27
2019/05/22	CH2205	Male	Three y/o	Treatment (Wk 2)	Treatment	Fast Day	No	07:00	07:00 - 07:59	Morning	37,5	107	135,0	135,0	18,65	598,73	166,33
2019/05/22	CH2206	Male	Three y/o	Treatment (Wk 2)	Treatment	Fast Day	No	07:00	07:00 - 07:59	Morning	37,6	126	284,0	284,0	18,75	610,14	192,48
2019/05/22	CH2205	Male	Three y/o	Treatment (Wk 2)	Treatment	Fast Day	No	07:05	07:00 - 07:59	Morning	37,4	55	117,0	117,0	18,55	547,52	161,32
2019/05/22	CH2206	Male	Three y/o	Treatment (Wk 2)	Treatment	Fast Day	No	07:05	07:00 - 07:59	Morning	37,5	126	91,0	91,0	18,65	610,14	152,56
2019/05/22	CH2205	Male	Three y/o	Treatment (Wk 2)	Treatment	Fast Day	No	07:10	07:00 - 07:59	Morning	37,6	115	87,0	87,0	18,75	603,82	150,99
2019/05/22	CH2206	Male	Three y/o	Treatment (Wk 2)	Treatment	Fast Day	No	07:10	07:00 - 07:59	Morning	37,9	140	300,6	300,6	19,05	617,28	194,49
2019/05/22	CH2205	Male	Three y/o	Treatment (Wk 2)	Treatment	Fast Day	No	07:15	07:00 - 07:59	Morning	37,5		336,4	336,4	18,65		198,47
2019/05/22	CH2206	Male	Three y/o	Treatment (Wk 2)	Treatment	Fast Day	No	07:15	07:00 - 07:59	Morning	37,8	117	215,4	215,4	18,95	605,02	182,73
2019/05/22	CH2205	Male	Three y/o	Treatment (Wk 2)	Treatment	Fast Day	No	07:20	07:00 - 07:59	Morning	37,5	103	131,0	131,0	18,65	596,00	165,27
2019/05/22	CH2206	Male	Three y/o	Treatment (Wk 2)	Treatment	Fast Day	No	07:20	07:00 - 07:59	Morning	37,8		345,4	345,4	18,95		199,41
2019/05/22	CH2205	Male	Three y/o	Treatment (Wk 2)	Treatment	Fast Day	No	07:25	07:00 - 07:59	Morning	37,7		157,0	157,0	18,85		171,62
2019/05/22	CH2206	Male	Three y/o	Treatment (Wk 2)	Treatment	Fast Day	No	07:25	07:00 - 07:59	Morning	37,8	105	137,2	137,2	18,95	597,38	166,89
2019/05/22	CH2205	Male	Three y/o	Treatment (Wk 2)	Treatment	Fast Day	No	07:30	07:00 - 07:59	Morning	37,6	59	56,2	56,2	18,75	553,30	135,83
2019/05/22	CH2206	Male	Three y/o	Treatment (Wk 2)	Treatment	Fast Day	No	07:30	07:00 - 07:59	Morning	37,8	125	175,8	175,8	18,95	609,60	175,59
2019/05/22	CH2205	Male	Three y/o	Treatment (Wk 2)	Treatment	Fast Day	No	07:35	07:00 - 07:59	Morning	37,5	101	74,4	74,4	18,65	594,59	145,55
2019/05/22	CH2206	Male	Three y/o	Treatment (Wk 2)	Treatment	Fast Day	No	07:35	07:00 - 07:59	Morning	37,9	135	191,6	191,6	19,05	614,84	178,61
2019/05/22	CH2205	Male	Three y/o	Treatment (Wk 2)	Treatment	Fast Day	No	07:40	07:00 - 07:59	Morning	37,5	68	44,0	44,0	18,65	564,71	127,37
2019/05/22	CH2206	Male	Three y/o	Treatment (Wk 2)	Treatment	Fast Day	No	07:40	07:00 - 07:59	Morning	37,8	110	138,2	138,2	18,95	600,69	167,15
2019/05/22	CH2205	Male	Three y/o	Treatment (Wk 2)	Treatment	Fast Day	No	07:45	07:00 - 07:59	Morning	37,6	106	33,6	33,6	18,75	598,06	118,09
2019/05/22	CH2206	Male	Three y/o	Treatment (Wk 2)	Treatment	Fast Day	No	07:45	07:00 - 07:59	Morning	37,8	64	174,6	174,6	18,95	559,88	175,34
2019/05/22	CH2205	Male	Three y/o	Treatment (Wk 2)	Treatment	Fast Day	No	07:50	07:00 - 07:59	Morning	37,5	50	268,4	268,4	18,65	539,52	190,49
2019/05/22	CH2206	Male	Three y/o	Treatment (Wk 2)	Treatment	Fast Day	No	07:50	07:00 - 07:59	Morning	37,7	134	179,4	179,4	18,85	614,33	176,30
2019/05/22	CH2205	Male	Three y/o	Treatment (Wk 2)	Treatment	Fast Day	No	07:55	07:00 - 07:59	Morning	37,5	117	118,6	118,6	18,65	605,02	161,80
2019/05/22	CH2206	Male	Three y/o	Treatment (Wk 2)	Treatment	Fast Day	No	07:55	07:00 - 07:59	Morning	37,7	118	146,2	146,2	18,85	605,61	169,12
2019/05/22	CH2205	Male	Three y/o	Treatment (Wk 2)	Treatment	Fast Day	No	08:00	08:00 - 08:59	Late Morning	37,5		67,8	67,8	18,65		142,33
2019/05/22	CH2206	Male	Three y/o	Treatment (Wk 2)	Treatment	Fast Day	No	08:00	08:00 - 08:59	Late Morning	37,7	114	139,8	139,8	18,85	603,20	167,55
2019/05/22	CH2205	Male	Three y/o	Treatment (Wk 2)	Treatment	Fast Day	No	08:05	08:00 - 08:59	Late Morning	37,4	68	23,6	23,6	18,55	564,71	105,97
2019/05/22	CH2206	Male	Three y/o	Treatment (Wk 2)	Treatment	Fast Day	No	08:05	08:00 - 08:59	Late Morning	37,8	129	152,0	152,0	18,95	611,75	170,48
2019/05/22	CH2205	Male	Three y/o	Treatment (Wk 2)	Treatment	Fast Day	No	08:10	08:00 - 08:59	Late Morning	37,4	94	37,0	37,0	18,55	589,37	121,40
2019/05/22	CH2206	Male	Three y/o	Treatment (Wk 2)	Treatment	Fast Day	No	08:10	08:00 - 08:59	Late Morning	37,7	132	225,6	225,6	18,85	613,32	184,36
2019/05/22	CH2205	Male	Three y/o	Treatment (Wk 2)	Treatment	Fast Day	No	08:15	08:00 - 08:59	Late Morning	37,5	128	97,4	97,4	18,65	611,22	154,93
2019/05/22	CH2206	Male	Three y/o	Treatment (Wk 2)	Treatment	Fast Day	No	08:15	08:00 - 08:59	Late Morning	37,5	84	129,2	129,2	18,65	581,01	164,79
2019/05/22	CH2205	Male	Three y/o	Treatment (Wk 2)	Treatment	Fast Day	No	08:20	08:00 - 08:59	Late Morning	37,5	140	28,2	28,2	18,65	617,28	112,07
2019/05/22	CH2206	Male	Three y/o	Treatment (Wk 2)	Treatment	Fast Day	No	08:20	08:00 - 08:59	Late Morning	37,4	128	105,0	105,0	18,55	611,22	157,55
2019/05/22	CH2205	Male	Three y/o	Treatment (Wk 2)	Treatment	Fast Day	No	08:25	08:00 - 08:59	Late Morning	37,4	72	24,2	24,2	18,55	569,20	106,83
2019/05/22	CH2206	Male	Three y/o	Treatment (Wk 2)	Treatment	Fast Day	No	08:25	08:00 - 08:59	Late Morning	37,5	58	58,6	58,6	18,65	551,90	137,28
2019/05/22	CH2205	Male	Three y/o	Treatment (Wk 2)	Treatment	Fast Day	No	08:30	08:00 - 08:59	Late Morning	37,3	97	78,0	78,0	18,46	591,66	147,20
2019/05/22	CH2206	Male	Three y/o	Treatment (Wk 2)	Treatment	Fast Day	No	08:30	08:00 - 08:59	Late Morning	37,5		57,4	57,4	18,65		136,56
2019/05/22	CH2205	Male	Three y/o	Treatment (Wk 2)	Treatment	Fast Day	No	08:35	08:00 - 08:59	Late Morning	37,5	125	172,4	172,4	18,65	609,60	174,90
2019/05/22	CH2206	Male	Three y/o	Treatment (Wk 2)	Treatment	Fast Day	No	08:35	08:00 - 08:59	Late Morning	37,7	128	107,8	107,8	18,85	611,22	158,46
2019/05/22	CH2205	Male	Three y/o	Treatment (Wk 2)	Treatment	Fast Day	No	08:40	08:00 - 08:59	Late Morning	37,6		273,6	273,6	18,75		191,16
2019/05/22	CH2206	Male	Three y/o	Treatment (Wk 2)	Treatment	Fast Day	No	08:40	08:00 - 08:59	Late Morning	37,7	120	181,0	181,0	18,85	606,78	176,61
2019/05/22	CH2205	Male	Three y/o	Treatment (Wk 2)	Treatment	Fast Day	No	08:45	08:00 - 08:59	Late Morning	37,6	123	194,2	194,2	18,75	608,49	179,08
2019/05/22	CH2206	Male	Three y/o	Treatment (Wk 2)	Treatment	Fast Day	No	08:45	08:00 - 08:59	Late Morning	37,7	114	167,8	167,8	18,85	603,20	173,95
2019/05/22	CH2205	Male	Three y/o	Treatment (Wk 2)	Treatment	Fast Day	No	08:50	08:00 - 08:59	Late Morning	37,7	63	90,0	90,0	18,85	558,62	152,17
2019/05/22	CH2206	Male	Three y/o	Treatment (Wk 2)	Treatment	Fast Day	No	08:50	08:00 - 08:59	Late Morning	37,7	180	96,0	96,0	18,85	633,61	154,42
2019/05/22	CH2205	Male	Three y/o	Treatment (Wk 2)	Treatment	Fast Day	No	08:55	08:00 - 08:59	Late Morning	37,7	134	32,8	32,8	18,85	614,33	117,26
2019/05/22	CH2206	Male	Three y/o	Treatment (Wk 2)	Treatment	Fast Day	No	08:55	08:00 - 08:59	Late Morning	37,7	109	114,2	114,2	18,85	600,04	160,48
2019/05/22	CH2205	Male	Three y/o	Treatment (Wk 2)	Treatment	Fast Day	No	09:00	09:00 - 09:59	Late Morning	37,8	113	29,2	29,2	18,95	602,58	113,27
2019/05/22	CH2206	Male	Three y/o	Treatment (Wk 2)	Treatment	Fast Day	No	09:00	09:00 - 09:59	Late Morning	37,7	109	17,6	17,6	18,85	600,04	95,95
2019/05/22	CH2205	Male	Three y/o	Treatment (Wk 2)	Treatment	Fast Day	No	09:05	09:00 - 09:59	Late Morning	37,7	84	17,2	17,2	18,85	581,01	95,17
2019/05/22	CH2206	Male	Three y/o	Treatment (Wk 2)	Treatment	Fast Day	No	09:05	09:00 - 09:59	Late Morning	37,7	72	38,0	38,0	18,85	569,20	122,32
2019/05/22	CH2205	Male	Three y/o	Treatment (Wk 2)	Treatment	Fast Day	No	09:10	09:00 - 09:59	Late Morning	37,9		235,6	235,6	19,05		185,89
2019/05/22	CH2206	Male	Three y/o	Treatment (Wk 2)	Treatment	Fast Day	No	09:10	09:00 - 09:59	Late Morning	37,9		263,4	263,4	19,05		189,82
2019/05/22	CH2205	Male	Three y/o	Treatment (Wk 2)	Treatment	Fast Day	No	09:15	09:00 - 09:59	Late Morning	38,0	89	81,0	81,0	19,15	585,33	148,51
2019/05/22	CH2206	Male	Three y/o	Treatment (Wk 2)	Treatment	Fast Day	No	09:15	09:00 - 09:59	Late Morning	37,9	113	270,4	270,4	19,05	602,58	190,75
2019/05/22	CH2205	Male	Three y/o	Treatment (Wk 2)	Treatment	Fast Day	No	09:20	09:00 - 09:59	Late Morning	37,9	125	11,4	11,4	19,05	609,60	81,19
2019/05/22	CH2206	Male	Three y/o	Treatment (Wk 2)	Treatment	Fast Day	No	09:20	09:00 - 09:59	Late Morning	37,9	120	310,4	310,4	19,05	606,78	195,63
2019/05/22	CH2205	Male	Three y/o	Treatment (Wk 2)	Treatment	Fast Day	No	09:25	09:00 - 09:59	Late Morning	37,9	64	20,4	20,4	19,05	559,88	100,99
2019/05/22	CH2206	Male	Three y/o	Treatment (Wk 2)	Treatment	Fast Day	No	09:25	09:00 - 09:59	Late Morning	37,9	51	188,6	188,6	19,05	541,20	178,05
2019/05/22	CH2205	Male	Three y/o	Treatment (Wk 2)	Treatment	Fast Day	No	09:30	09:00 - 09:59	Late Morning	38,0	83	12,4	12,4	19,15	580,11	84,04
2019/05/22	CH2206	Male	Three y/o	Treatment (Wk 2)	Treatment	Fast Day	No	09:30	09:00 - 09:59	Late Morning	37,9	113	320,2	320,2	19,05	602,58	196,73
2019/05/22	CH2205	Male	Three y/o	Treatment (Wk 2)	Treatment	Fast Day	No	09:35	09:00 - 09:59	Late Morning	38,0	61	11,6	11,6	19,15	556,01	81,78
2019/05/22	CH2206	Male	Three y/o	Treatment (Wk 2)	Treatment	Fast Day	No	09:35	09:00 - 09:59	Late Morning	37,9	128	235,8	235,8	19,05	611,22	185,92
2019/05/22	CH2205	Male	Three y/o	Treatment (Wk 2)	Treatment	Fast Day	No	09:40	09:00 - 09:59	Late Morning	38,0		12,6	12,6	19,15		84,59
2019/05/22	CH2206	Male	Three y/o	Treatment (Wk 2)	Treatment	Fast Day	No	09:40	09:00 - 09:59	Late Morning	37,8	111	380,6	380,6	18,95	601,33	202,85
2019/05/22	CH2205	Male	Three y/o	Treatment (Wk 2)	Treatment	Fast Day	No	09:45	09:00 - 09:59	Late Morning	38,0	92	48,8	48,8	19,15	587,78	130,95

2019/05/22	CH2206	Male	Three y/o	Treatment (Wk 2)	Treatment	Fast Day	No	09:45	09:00 - 09:59	Late Morning	37,8	99	186,4	186,4	18,95	593,14	177,64
2019/05/22	CH2205	Male	Three y/o	Treatment (Wk 2)	Treatment	Fast Day	No	09:50	09:00 - 09:59	Late Morning	38,0		71,8	71,8	19,15		144,32
2019/05/22	CH2206	Male	Three y/o	Treatment (Wk 2)	Treatment	Fast Day	No	09:50	09:00 - 09:59	Late Morning	37,8	119	95,4	95,4	18,95	606,20	154,20
2019/05/22	CH2205	Male	Three y/o	Treatment (Wk 2)	Treatment	Fast Day	No	09:55	09:00 - 09:59	Late Morning	37,9	64	11,4	11,4	19,05	559,88	81,19
2019/05/22	CH2206	Male	Three y/o	Treatment (Wk 2)	Treatment	Fast Day	No	09:55	09:00 - 09:59	Late Morning	37,7	109	69,8	69,8	18,85	600,04	143,34
2019/05/22	CH2205	Male	Three y/o	Treatment (Wk 2)	Treatment	Fast Day	No	10:00	10:00 - 10:59	Late Morning	37,7	54	16,4	16,4	18,85	545,99	93,55
2019/05/22	CH2206	Male	Three y/o	Treatment (Wk 2)	Treatment	Fast Day	No	10:00	10:00 - 10:59	Late Morning	37,8	96	187,2	187,2	18,95	590,91	177,79
2019/05/22	CH2205	Male	Three y/o	Treatment (Wk 2)	Treatment	Fast Day	No	10:05	10:00 - 10:59	Late Morning	37,7		22,2	22,2	18,85		103,88
2019/05/22	CH2206	Male	Three y/o	Treatment (Wk 2)	Treatment	Fast Day	No	10:05	10:00 - 10:59	Late Morning	37,8		70,8	70,8	18,95		143,83
2019/05/22	CH2205	Male	Three y/o	Treatment (Wk 2)	Treatment	Fast Day	No	10:10	10:00 - 10:59	Late Morning	37,7	62	17,2	17,2	18,85	557,33	95,17
2019/05/22	CH2206	Male	Three y/o	Treatment (Wk 2)	Treatment	Fast Day	No	10:10	10:00 - 10:59	Late Morning	37,8	78	96,4	96,4	18,95	575,38	154,57
2019/05/22	CH2205	Male	Three y/o	Treatment (Wk 2)	Treatment	Fast Day	No	10:15	10:00 - 10:59	Late Morning	37,6	57	20,4	20,4	18,75	550,47	100,99
2019/05/22	CH2206	Male	Three y/o	Treatment (Wk 2)	Treatment	Fast Day	No	10:15	10:00 - 10:59	Late Morning	37,8	56	156,0	156,0	18,95	549,01	171,39
2019/05/22	CH2205	Male	Three y/o	Treatment (Wk 2)	Treatment	Fast Day	No	10:20	10:00 - 10:59	Late Morning	37,6	88	31,6	31,6	18,75	584,49	115,98
2019/05/22	CH2206	Male	Three y/o	Treatment (Wk 2)	Treatment	Fast Day	No	10:20	10:00 - 10:59	Late Morning	37,8	106	103,8	103,8	18,95	598,06	157,14
2019/05/22	CH2205	Male	Three y/o	Treatment (Wk 2)	Treatment	Fast Day	No	10:25	10:00 - 10:59	Late Morning	37,6	60	30,2	30,2	18,75	554,67	114,42
2019/05/22	CH2206	Male	Three y/o	Treatment (Wk 2)	Treatment	Fast Day	No	10:25	10:00 - 10:59	Late Morning	37,8	94	29,8	29,8	18,95	589,37	113,97
2019/05/22	CH2205	Male	Three y/o	Treatment (Wk 2)	Treatment	Fast Day	No	10:30	10:00 - 10:59	Late Morning	37,6		29,2	29,2	18,75		113,27
2019/05/22	CH2206	Male	Three y/o	Treatment (Wk 2)	Treatment	Fast Day	No	10:30	10:00 - 10:59	Late Morning	37,7	110	21,8	21,8	18,85	600,69	103,26
2019/05/22	CH2205	Male	Three y/o	Treatment (Wk 2)	Treatment	Fast Day	No	10:35	10:00 - 10:59	Late Morning	37,5	53	2,6	2,6	18,65	544,43	31,57
2019/05/22	CH2206	Male	Three y/o	Treatment (Wk 2)	Treatment	Fast Day	No	10:35	10:00 - 10:59	Late Morning	37,7	63	4,2	4,2	18,85	558,62	47,57
2019/05/22	CH2205	Male	Three y/o	Treatment (Wk 2)	Treatment	Fast Day	No	10:40	10:00 - 10:59	Late Morning	37,4	49	7,2	7,2	18,55	537,80	65,66
2019/05/22	CH2206	Male	Three y/o	Treatment (Wk 2)	Treatment	Fast Day	No	10:40	10:00 - 10:59	Late Morning	37,7	49	9,2	9,2	18,85	537,80	73,94
2019/05/22	CH2205	Male	Three y/o	Treatment (Wk 2)	Treatment	Fast Day	No	10:45	10:00 - 10:59	Late Morning	37,4	48	8,4	8,4	18,55	536,04	70,86
2019/05/22	CH2206	Male	Three y/o	Treatment (Wk 2)	Treatment	Fast Day	No	10:45	10:00 - 10:59	Late Morning	37,7	59	31,6	31,6	18,85	553,30	115,98
2019/05/22	CH2205	Male	Three y/o	Treatment (Wk 2)	Treatment	Fast Day	No	10:50	10:00 - 10:59	Late Morning	37,4	54	6,6	6,6	18,55	545,99	62,73
2019/05/22	CH2206	Male	Three y/o	Treatment (Wk 2)	Treatment	Fast Day	No	10:50	10:00 - 10:59	Late Morning	37,7	62	39,2	39,2	18,85	557,33	123,39
2019/05/22	CH2205	Male	Three y/o	Treatment (Wk 2)	Treatment	Fast Day	No	10:55	10:00 - 10:59	Late Morning	37,3	52	7,8	7,8	18,46	542,83	68,36
2019/05/22	CH2206	Male	Three y/o	Treatment (Wk 2)	Treatment	Fast Day	No	10:55	10:00 - 10:59	Late Morning	37,7	45	24,6	24,6	18,85	530,47	107,39
2019/05/22	CH2205	Male	Three y/o	Treatment (Wk 2)	Treatment	Fast Day	No	11:00	11:00 - 11:59	Late Morning	37,3	51	11,0	11,0	18,46	541,20	79,98
2019/05/22	CH2206	Male	Three y/o	Treatment (Wk 2)	Treatment	Fast Day	No	11:00	11:00 - 11:59	Late Morning	37,6	47	20,0	20,0	18,75	534,23	100,31
2019/05/22	CH2205	Male	Three y/o	Treatment (Wk 2)	Treatment	Fast Day	No	11:05	11:00 - 11:59	Late Morning	37,3	52	11,0	11,0	18,46	542,83	79,98
2019/05/22	CH2206	Male	Three y/o	Treatment (Wk 2)	Treatment	Fast Day	No	11:05	11:00 - 11:59	Late Morning	37,5	50	8,4	8,4	18,65	539,52	70,86
2019/05/22	CH2205	Male	Three y/o	Treatment (Wk 2)	Treatment	Fast Day	No	11:10	11:00 - 11:59	Late Morning	37,4	54	4,6	4,6	18,55	545,99	50,61
2019/05/22	CH2206	Male	Three y/o	Treatment (Wk 2)	Treatment	Fast Day	No	11:10	11:00 - 11:59	Late Morning	37,5	44	19,6	19,6	18,65	528,51	99,63
2019/05/22	CH2205	Male	Three y/o	Treatment (Wk 2)	Treatment	Fast Day	No	11:15	11:00 - 11:59	Late Morning	37,5		248,8	248,8	18,65		187,81
2019/05/22	CH2206	Male	Three y/o	Treatment (Wk 2)	Treatment	Fast Day	No	11:15	11:00 - 11:59	Late Morning	37,5	90	44,2	44,2	18,65	586,16	127,53
2019/05/22	CH2205	Male	Three y/o	Treatment (Wk 2)	Treatment	Fast Day	No	11:20	11:00 - 11:59	Late Morning	37,5	158	50,0	50,0	18,65	625,26	131,79
2019/05/22	CH2206	Male	Three y/o	Treatment (Wk 2)	Treatment	Fast Day	No	11:20	11:00 - 11:59	Late Morning	37,5	112	104,2	104,2	18,65	601,96	157,28
2019/05/22	CH2205	Male	Three y/o	Treatment (Wk 2)	Treatment	Fast Day	No	11:25	11:00 - 11:59	Late Morning	37,5		35,2	35,2	18,65		119,69
2019/05/22	CH2206	Male	Three y/o	Treatment (Wk 2)	Treatment	Fast Day	No	11:25	11:00 - 11:59	Late Morning	37,4	103	105,8	105,8	18,55	596,00	157,81
2019/05/22	CH2205	Male	Three y/o	Treatment (Wk 2)	Treatment	Fast Day	No	11:30	11:00 - 11:59	Late Morning	37,3	108	83,4	83,4	18,46	599,39	149,52
2019/05/22	CH2206	Male	Three y/o	Treatment (Wk 2)	Treatment	Fast Day	No	11:30	11:00 - 11:59	Late Morning	37,3	95	66,4	66,4	18,46	590,14	141,61
2019/05/22	CH2205	Male	Three y/o	Treatment (Wk 2)	Treatment	Fast Day	No	11:35	11:00 - 11:59	Late Morning	37,4	107	15,6	15,6	18,55	598,73	91,85
2019/05/22	CH2206	Male	Three y/o	Treatment (Wk 2)	Treatment	Fast Day	No	11:35	11:00 - 11:59	Late Morning	37,4	88	112,0	112,0	18,55	584,49	159,80
2019/05/22	CH2205	Male	Three y/o	Treatment (Wk 2)	Treatment	Fast Day	No	11:40	11:00 - 11:59	Late Morning	37,4	71	10,0	10,0	18,55	568,10	76,76
2019/05/22	CH2206	Male	Three y/o	Treatment (Wk 2)	Treatment	Fast Day	No	11:40	11:00 - 11:59	Late Morning	37,4	51	104,4	104,4	18,55	541,20	157,35
2019/05/22	CH2205	Male	Three y/o	Treatment (Wk 2)	Treatment	Fast Day	No	11:45	11:00 - 11:59	Late Morning	37,5	63	6,4	6,4	18,65	558,62	61,70
2019/05/22	CH2206	Male	Three y/o	Treatment (Wk 2)	Treatment	Fast Day	No	11:45	11:00 - 11:59	Late Morning	37,4	45	67,6	67,6	18,55	530,47	142,23
2019/05/22	CH2205	Male	Three y/o	Treatment (Wk 2)	Treatment	Fast Day	No	11:50	11:00 - 11:59	Late Morning	37,5	62	55,2	55,2	18,65	557,33	135,21
2019/05/22	CH2206	Male	Three y/o	Treatment (Wk 2)	Treatment	Fast Day	No	11:50	11:00 - 11:59	Late Morning	37,5	98	31,4	31,4	18,65	592,41	115,76
2019/05/22	CH2205	Male	Three y/o	Treatment (Wk 2)	Treatment	Fast Day	No	11:55	11:00 - 11:59	Late Morning	37,7	77	39,4	39,4	18,85	574,39	123,57
2019/05/22	CH2206	Male	Three y/o	Treatment (Wk 2)	Treatment	Fast Day	No	11:55	11:00 - 11:59	Late Morning	37,4	45	28,6	28,6	18,55	530,47	112,56
2019/05/22	CH2205	Male	Three y/o	Treatment (Wk 2)	Treatment	Fast Day	No	12:00	12:00 - 12:59	Afternoon	37,7	51	199,6	199,6	18,85	541,20	180,05
2019/05/22	CH2206	Male	Three y/o	Treatment (Wk 2)	Treatment	Fast Day	No	12:00	12:00 - 12:59	Afternoon	37,4	94	107,6	107,6	18,55	589,37	158,40
2019/05/22	CH2205	Male	Three y/o	Treatment (Wk 2)	Treatment	Fast Day	No	12:05	12:00 - 12:59	Afternoon	37,7	83	24,6	24,6	18,85	580,11	107,39
2019/05/22	CH2206	Male	Three y/o	Treatment (Wk 2)	Treatment	Fast Day	No	12:05	12:00 - 12:59	Afternoon	37,5	115	130,2	130,2	18,65	603,82	165,06
2019/05/22	CH2205	Male	Three y/o	Treatment (Wk 2)	Treatment	Fast Day	No	12:10	12:00 - 12:59	Afternoon	37,7	76	15,6	15,6	18,85	573,39	91,85
2019/05/22	CH2206	Male	Three y/o	Treatment (Wk 2)	Treatment	Fast Day	No	12:10	12:00 - 12:59	Afternoon	37,7	112	153,2	153,2	18,85	601,96	170,76
2019/05/22	CH2205	Male	Three y/o	Treatment (Wk 2)	Treatment	Fast Day	No	12:15	12:00 - 12:59	Afternoon	37,7	46	17,6	17,6	18,85	532,38	95,95
2019/05/22	CH2206	Male	Three y/o	Treatment (Wk 2)	Treatment	Fast Day	No	12:15	12:00 - 12:59	Afternoon	37,7	102	170,2	170,2	18,85	595,30	174,45
2019/05/22	CH2205	Male	Three y/o	Treatment (Wk 2)	Treatment	Fast Day	No	12:20	12:00 - 12:59	Afternoon	37,9	72	82,0	82,0	19,05	569,20	148,93
2019/05/22	CH2206	Male	Three y/o	Treatment (Wk 2)	Treatment	Fast Day	No	12:20	12:00 - 12:59	Afternoon	37,7		134,6	134,6	18,85		166,22
2019/05/22	CH2205	Male	Three y/o	Treatment (Wk 2)	Treatment	Fast Day	No	12:25	12:00 - 12:59	Afternoon	37,9	62	38,4	38,4	19,05	557,33	122,68
2019/05/22	CH2206	Male	Three y/o	Treatment (Wk 2)	Treatment	Fast Day	No	12:25	12:00 - 12:59	Afternoon	37,7	112	85,6	85,6	18,85	601,96	150,43
2019/05/22	CH2205	Male	Three y/o	Treatment (Wk 2)	Treatment	Fast Day	No	12:30	12:00 - 12:59	Afternoon	37,7	74	30,6	30,6	18,85	571,33	114,87
2019/05/22	CH2206	Male	Three y/o	Treatment (Wk 2)	Treatment	Fast Day	No	12:30	12:00 - 12:59	Afternoon	37,8		43,4	43,4	18,95		126,90
2019/05/22	CH2205	Male	Three y/o	Treatment (Wk 2)	Treatment	Fast Day	No	12:35	12:00 - 12:59	Afternoon	37,7	55	35,4	35,4	18,85	547,52	119,88
2019/05/22	CH2206	Male	Three y/o	Treatment (Wk 2)	Treatment	Fast Day	No	12:35	12:00 - 12:59	Afternoon	37,8	97	12,4	12,4	18,95	591,66	84,04
2019/05/22	CH2205	Male	Three y/o	Treatment (Wk 2)	Treatment	Fast Day	No	12:40	12:00 - 12:59	Afternoon	37,7	41	35,6	35,6	18,85	522,29	120,08

2019/05/22	CH2206	Male	Three y/o	Treatment (Wk 2)	Treatment	Fast Day	No	12:40	12:00 - 12:59	Afternoon	37,7	55	7,4	7,4	18,85	547,52	66,59
2019/05/22	CH2205	Male	Three y/o	Treatment (Wk 2)	Treatment	Fast Day	No	12:45	12:00 - 12:59	Afternoon	37,7	58	22,4	22,4	18,85	551,90	104,19
2019/05/22	CH2206	Male	Three y/o	Treatment (Wk 2)	Treatment	Fast Day	No	12:45	12:00 - 12:59	Afternoon	37,7	51	8,4	8,4	18,85	541,20	70,86
2019/05/22	CH2205	Male	Three y/o	Treatment (Wk 2)	Treatment	Fast Day	No	12:50	12:00 - 12:59	Afternoon	37,7	47	28,4	28,4	18,85	534,23	112,31
2019/05/22	CH2206	Male	Three y/o	Treatment (Wk 2)	Treatment	Fast Day	No	12:50	12:00 - 12:59	Afternoon	37,8	51	7,0	7,0	18,95	541,20	64,72
2019/05/22	CH2205	Male	Three y/o	Treatment (Wk 2)	Treatment	Fast Day	No	12:55	12:00 - 12:59	Afternoon	37,7	65	28,6	28,6	18,85	561,12	112,56
2019/05/22	CH2206	Male	Three y/o	Treatment (Wk 2)	Treatment	Fast Day	No	12:55	12:00 - 12:59	Afternoon	37,8	49	4,2	4,2	18,95	537,80	47,57
2019/05/22	CH2205	Male	Three y/o	Treatment (Wk 2)	Treatment	Fast Day	No	13:00	13:00 - 13:59	Afternoon	37,7	60	26,4	26,4	18,85	554,67	109,81
2019/05/22	CH2206	Male	Three y/o	Treatment (Wk 2)	Treatment	Fast Day	No	13:00	13:00 - 13:59	Afternoon	37,8	41	7,6	7,6	18,95	522,29	67,49
2019/05/22	CH2205	Male	Three y/o	Treatment (Wk 2)	Treatment	Fast Day	No	13:05	13:00 - 13:59	Afternoon	37,7	50	26,2	26,2	18,85	539,52	109,55
2019/05/22	CH2206	Male	Three y/o	Treatment (Wk 2)	Treatment	Fast Day	No	13:05	13:00 - 13:59	Afternoon	37,8	76	15,6	15,6	18,95	573,39	91,85
2019/05/22	CH2205	Male	Three y/o	Treatment (Wk 2)	Treatment	Fast Day	No	13:10	13:00 - 13:59	Afternoon	37,7	54	12,4	12,4	18,85	545,99	84,04
2019/05/22	CH2206	Male	Three y/o	Treatment (Wk 2)	Treatment	Fast Day	No	13:10	13:00 - 13:59	Afternoon	37,8	64	58,4	58,4	18,95	559,88	137,16
2019/05/22	CH2205	Male	Three y/o	Treatment (Wk 2)	Treatment	Fast Day	No	13:15	13:00 - 13:59	Afternoon	37,9	60	74,8	74,8	19,05	554,67	145,74
2019/05/22	CH2206	Male	Three y/o	Treatment (Wk 2)	Treatment	Fast Day	No	13:15	13:00 - 13:59	Afternoon	37,8	46	53,6	53,6	18,95	532,38	134,19
2019/05/22	CH2205	Male	Three y/o	Treatment (Wk 2)	Treatment	Fast Day	No	13:20	13:00 - 13:59	Afternoon	37,7	141	88,8	88,8	18,85	617,75	151,71
2019/05/22	CH2206	Male	Three y/o	Treatment (Wk 2)	Treatment	Fast Day	No	13:20	13:00 - 13:59	Afternoon	37,8	52	55,4	55,4	18,95	542,83	135,33
2019/05/22	CH2205	Male	Three y/o	Treatment (Wk 2)	Treatment	Fast Day	No	13:25	13:00 - 13:59	Afternoon	37,7		43,0	43,0	18,85		126,58
2019/05/22	CH2206	Male	Three y/o	Treatment (Wk 2)	Treatment	Fast Day	No	13:25	13:00 - 13:59	Afternoon	37,7	101	94,4	94,4	18,85	594,59	153,84
2019/05/22	CH2205	Male	Three y/o	Treatment (Wk 2)	Treatment	Fast Day	No	13:30	13:00 - 13:59	Afternoon	37,7	57	17,4	17,4	18,85	550,47	95,56
2019/05/22	CH2206	Male	Three y/o	Treatment (Wk 2)	Treatment	Fast Day	No	13:30	13:00 - 13:59	Afternoon	37,7	104	112,6	112,6	18,85	596,69	159,98
2019/05/22	CH2205	Male	Three y/o	Treatment (Wk 2)	Treatment	Fast Day	No	13:35	13:00 - 13:59	Afternoon	37,7		95,6	95,6	18,85		154,28
2019/05/22	CH2206	Male	Three y/o	Treatment (Wk 2)	Treatment	Fast Day	No	13:35	13:00 - 13:59	Afternoon	37,7	103	275,8	275,8	18,85	596,00	191,45
2019/05/22	CH2205	Male	Three y/o	Treatment (Wk 2)	Treatment	Fast Day	No	13:40	13:00 - 13:59	Afternoon	37,7		26,4	26,4	18,85		109,81
2019/05/22	CH2206	Male	Three y/o	Treatment (Wk 2)	Treatment	Fast Day	No	13:40	13:00 - 13:59	Afternoon	37,7	121	111,0	111,0	18,85	607,35	159,48
2019/05/22	CH2205	Male	Three y/o	Treatment (Wk 2)	Treatment	Fast Day	No	13:45	13:00 - 13:59	Afternoon	37,9		80,8	80,8	19,05		148,42
2019/05/22	CH2206	Male	Three y/o	Treatment (Wk 2)	Treatment	Fast Day	No	13:45	13:00 - 13:59	Afternoon	37,8		191,0	191,0	18,95		178,50
2019/05/22	CH2205	Male	Three y/o	Treatment (Wk 2)	Treatment	Fast Day	No	13:50	13:00 - 13:59	Afternoon	37,7		53,2	53,2	18,85		133,93
2019/05/22	CH2206	Male	Three y/o	Treatment (Wk 2)	Treatment	Fast Day	No	13:50	13:00 - 13:59	Afternoon	37,8	112	128,8	128,8	18,95	601,96	164,68
2019/05/22	CH2205	Male	Three y/o	Treatment (Wk 2)	Treatment	Fast Day	No	13:55	13:00 - 13:59	Afternoon	37,7	182	56,6	56,6	18,85	634,31	136,07
2019/05/22	CH2206	Male	Three y/o	Treatment (Wk 2)	Treatment	Fast Day	No	13:55	13:00 - 13:59	Afternoon	37,8	77	33,0	33,0	18,95	574,39	117,47
2019/05/22	CH2205	Male	Three y/o	Treatment (Wk 2)	Treatment	Fast Day	No	14:00	14:00 - 14:59	Afternoon	37,7	154	75,2	75,2	18,85	623,58	145,93
2019/05/22	CH2206	Male	Three y/o	Treatment (Wk 2)	Treatment	Fast Day	No	14:00	14:00 - 14:59	Afternoon	37,8		42,8	42,8	18,95		126,42
2019/05/22	CH2205	Male	Three y/o	Treatment (Wk 2)	Treatment	Fast Day	No	14:05	14:00 - 14:59	Afternoon	37,7	72	103,0	103,0	18,85	569,20	156,87
2019/05/22	CH2206	Male	Three y/o	Treatment (Wk 2)	Treatment	Fast Day	No	14:05	14:00 - 14:59	Afternoon	37,7		49,0	49,0	18,85		131,09
2019/05/22	CH2205	Male	Three y/o	Treatment (Wk 2)	Treatment	Fast Day	No	14:10	14:00 - 14:59	Afternoon	37,7	194	95,4	95,4	18,85	638,30	154,20
2019/05/22	CH2206	Male	Three y/o	Treatment (Wk 2)	Treatment	Fast Day	No	14:10	14:00 - 14:59	Afternoon	37,7	51	38,6	38,6	18,85	541,20	122,86
2019/05/22	CH2205	Male	Three y/o	Treatment (Wk 2)	Treatment	Fast Day	No	14:15	14:00 - 14:59	Afternoon	37,9	80	19,2	19,2	19,05	577,31	98,92
2019/05/22	CH2206	Male	Three y/o	Treatment (Wk 2)	Treatment	Fast Day	No	14:15	14:00 - 14:59	Afternoon	37,7	125	233,4	233,4	18,85	609,60	185,56
2019/05/22	CH2205	Male	Three y/o	Treatment (Wk 2)	Treatment	Fast Day	No	14:20	14:00 - 14:59	Afternoon	37,9		37,0	37,0	19,05		121,40
2019/05/22	CH2206	Male	Three y/o	Treatment (Wk 2)	Treatment	Fast Day	No	14:20	14:00 - 14:59	Afternoon	37,8	90	278,0	278,0	18,95	586,16	191,73
2019/05/22	CH2205	Male	Three y/o	Treatment (Wk 2)	Treatment	Fast Day	No	14:25	14:00 - 14:59	Afternoon	37,9	62	8,2	8,2	19,05	557,33	70,05
2019/05/22	CH2206	Male	Three y/o	Treatment (Wk 2)	Treatment	Fast Day	No	14:25	14:00 - 14:59	Afternoon	37,8		111,8	111,8	18,95		159,74
2019/05/22	CH2205	Male	Three y/o	Treatment (Wk 2)	Treatment	Fast Day	No	14:30	14:00 - 14:59	Afternoon	37,9		57,0	57,0	19,05		136,32
2019/05/22	CH2206	Male	Three y/o	Treatment (Wk 2)	Treatment	Fast Day	No	14:30	14:00 - 14:59	Afternoon	37,8	49	154,6	154,6	18,95	537,80	171,08
2019/05/22	CH2205	Male	Three y/o	Treatment (Wk 2)	Treatment	Fast Day	No	14:35	14:00 - 14:59	Afternoon	38,0	118	70,4	70,4	19,15	605,61	143,64
2019/05/22	CH2206	Male	Three y/o	Treatment (Wk 2)	Treatment	Fast Day	No	14:35	14:00 - 14:59	Afternoon	37,8	105	54,0	54,0	18,95	597,38	134,45
2019/05/22	CH2205	Male	Three y/o	Treatment (Wk 2)	Treatment	Fast Day	No	14:40	14:00 - 14:59	Afternoon	38,1		82,2	82,2	19,26		149,02
2019/05/22	CH2206	Male	Three y/o	Treatment (Wk 2)	Treatment	Fast Day	No	14:40	14:00 - 14:59	Afternoon	37,8	111	133,0	133,0	18,95	601,33	165,80
2019/05/22	CH2205	Male	Three y/o	Treatment (Wk 2)	Treatment	Fast Day	No	14:45	14:00 - 14:59	Afternoon	38,1	90	264,8	264,8	19,26	586,16	190,01
2019/05/22	CH2206	Male	Three y/o	Treatment (Wk 2)	Treatment	Fast Day	No	14:45	14:00 - 14:59	Afternoon	37,9	118	161,2	161,2	19,05	605,61	172,54
2019/05/22	CH2205	Male	Three y/o	Treatment (Wk 2)	Treatment	Fast Day	No	14:50	14:00 - 14:59	Afternoon	38,1	120	86,4	86,4	19,26	606,78	150,75
2019/05/22	CH2206	Male	Three y/o	Treatment (Wk 2)	Treatment	Fast Day	No	14:50	14:00 - 14:59	Afternoon	37,9	113	43,2	43,2	19,05	602,58	126,74
2019/05/22	CH2205	Male	Three y/o	Treatment (Wk 2)	Treatment	Fast Day	No	14:55	14:00 - 14:59	Afternoon	38,1	65	100,2	100,2	19,26	561,12	155,91
2019/05/22	CH2206	Male	Three y/o	Treatment (Wk 2)	Treatment	Fast Day	No	14:55	14:00 - 14:59	Afternoon	37,9	119	92,8	92,8	19,05	606,20	153,24
2019/05/22	CH2205	Male	Three y/o	Treatment (Wk 2)	Treatment	Fast Day	No	15:00	15:00 - 15:59	Afternoon	38,1	58	142,2	142,2	19,26	551,90	168,15
2019/05/22	CH2206	Male	Three y/o	Treatment (Wk 2)	Treatment	Fast Day	No	15:00	15:00 - 15:59	Afternoon	37,9	69	156,8	156,8	19,05	565,86	171,57
2019/05/22	CH2205	Male	Three y/o	Treatment (Wk 2)	Treatment	Fast Day	No	15:05	15:00 - 15:59	Afternoon	38,1	99	31,0	31,0	19,26	593,14	115,32
2019/05/22	CH2206	Male	Three y/o	Treatment (Wk 2)	Treatment	Fast Day	No	15:05	15:00 - 15:59	Afternoon	38,0	96	232,2	232,2	19,15	590,91	185,38
2019/05/22	CH2205	Male	Three y/o	Treatment (Wk 2)	Treatment	Fast Day	No	15:10	15:00 - 15:59	Afternoon	38,1		141,2	141,2	19,26		167,90
2019/05/22	CH2206	Male	Three y/o	Treatment (Wk 2)	Treatment	Fast Day	No	15:10	15:00 - 15:59	Afternoon	38,0	105	185,0	185,0	19,15	597,38	177,38
2019/05/22	CH2205	Male	Three y/o	Treatment (Wk 2)	Treatment	Fast Day	No	15:15	15:00 - 15:59	Afternoon	38,1	103	204,2	204,2	19,26	596,00	180,85
2019/05/22	CH2206	Male	Three y/o	Treatment (Wk 2)	Treatment	Fast Day	No	15:15	15:00 - 15:59	Afternoon	38,0	103	65,6	65,6	19,15	596,00	141,19
2019/05/22	CH2205	Male	Three y/o	Treatment (Wk 2)	Treatment	Fast Day	No	15:20	15:00 - 15:59	Afternoon	38,1	109	74,0	74,0	19,26	600,04	145,37
2019/05/22	CH2206	Male	Three y/o	Treatment (Wk 2)	Treatment	Fast Day	No	15:20	15:00 - 15:59	Afternoon	38,0		184,4	184,4	19,15		177,26
2019/05/22	CH2205	Male	Three y/o	Treatment (Wk 2)	Treatment	Fast Day	No	15:25	15:00 - 15:59	Afternoon	38,1	59	46,6	46,6	19,26	553,30	129,35
2019/05/22	CH2206	Male	Three y/o	Treatment (Wk 2)	Treatment	Fast Day	No	15:25	15:00 - 15:59	Afternoon	38,0	113	208,0	208,0	19,15	602,58	181,50
2019/05/22	CH2205	Male	Three y/o	Treatment (Wk 2)	Treatment	Fast Day	No	15:30	15:00 - 15:59	Afternoon	38,1	77	99,6	99,6	19,26	574,39	155,70
2019/05/22	CH2206	Male	Three y/o	Treatment (Wk 2)	Treatment	Fast Day	No	15:30	15:00 - 15:59	Afternoon	38,1	123	283,6	283,6	19,26	608,49	192,43
2019/05/22	CH2205	Male	Three y/o	Treatment (Wk 2)	Treatment	Fast Day	No	15:35	15:00 - 15:59	Afternoon	38,1	154	106,0	106,0	19,26	623,58	157,88

2019/05/22	CH2206	Male	Three y/o	Treatment (Wk 2)	Treatment	Fast Day	No	15:35	15:00 - 15:59	Afternoon	38,1	106	150,2	150,2	19,26	598,06	170,06
2019/05/22	CH2205	Male	Three y/o	Treatment (Wk 2)	Treatment	Fast Day	No	15:40	15:00 - 15:59	Afternoon	38,1	162	34,6	34,6	19,26	626,88	119,10
2019/05/22	CH2206	Male	Three y/o	Treatment (Wk 2)	Treatment	Fast Day	No	15:40	15:00 - 15:59	Afternoon	38,1	109	281,8	281,8	19,26	600,04	192,21
2019/05/22	CH2205	Male	Three y/o	Treatment (Wk 2)	Treatment	Fast Day	No	15:45	15:00 - 15:59	Afternoon	38,1	61	21,6	21,6	19,26	556,01	102,94
2019/05/22	CH2206	Male	Three y/o	Treatment (Wk 2)	Treatment	Fast Day	No	15:45	15:00 - 15:59	Afternoon	38,1	99	164,6	164,6	19,26	593,14	173,27
2019/05/22	CH2205	Male	Three y/o	Treatment (Wk 2)	Treatment	Fast Day	No	15:50	15:00 - 15:59	Afternoon	38,1	55	17,4	17,4	19,26	547,52	95,56
2019/05/22	CH2206	Male	Three y/o	Treatment (Wk 2)	Treatment	Fast Day	No	15:50	15:00 - 15:59	Afternoon	38,1	126	98,6	98,6	19,26	610,14	155,35
2019/05/22	CH2205	Male	Three y/o	Treatment (Wk 2)	Treatment	Fast Day	No	15:55	15:00 - 15:59	Afternoon	38,2		55,6	55,6	19,36		135,46
2019/05/22	CH2206	Male	Three y/o	Treatment (Wk 2)	Treatment	Fast Day	No	15:55	15:00 - 15:59	Afternoon	38,2		226,6	226,6	19,36		184,52
2019/05/22	CH2205	Male	Three y/o	Treatment (Wk 2)	Treatment	Fast Day	No	16:00	16:00 - 16:59	Evening	38,2	43	133,8	133,8	19,36	526,50	166,01
2019/05/22	CH2206	Male	Three y/o	Treatment (Wk 2)	Treatment	Fast Day	No	16:00	16:00 - 16:59	Evening	38,2	60	163,0	163,0	19,36	554,67	172,93
2019/05/22	CH2205	Male	Three y/o	Treatment (Wk 2)	Treatment	Fast Day	No	16:05	16:00 - 16:59	Evening	38,2	73	131,8	131,8	19,36	570,27	165,49
2019/05/22	CH2206	Male	Three y/o	Treatment (Wk 2)	Treatment	Fast Day	No	16:05	16:00 - 16:59	Evening	38,3	138	213,8	213,8	19,46	616,31	182,47
2019/05/22	CH2205	Male	Three y/o	Treatment (Wk 2)	Treatment	Fast Day	No	16:10	16:00 - 16:59	Evening	38,1	71	139,2	139,2	19,26	568,10	167,40
2019/05/22	CH2206	Male	Three y/o	Treatment (Wk 2)	Treatment	Fast Day	No	16:10	16:00 - 16:59	Evening	38,3	145	69,4	69,4	19,46	619,62	143,14
2019/05/22	CH2205	Male	Three y/o	Treatment (Wk 2)	Treatment	Fast Day	No	16:15	16:00 - 16:59	Evening	38,3	111	57,4	57,4	19,46	601,33	136,56
2019/05/22	CH2206	Male	Three y/o	Treatment (Wk 2)	Treatment	Fast Day	No	16:15	16:00 - 16:59	Evening	38,3	71	85,0	85,0	19,46	568,10	150,18
2019/05/22	CH2205	Male	Three y/o	Treatment (Wk 2)	Treatment	Fast Day	No	16:20	16:00 - 16:59	Evening	38,2	185	29,2	29,2	19,36	635,34	113,27
2019/05/22	CH2206	Male	Three y/o	Treatment (Wk 2)	Treatment	Fast Day	No	16:20	16:00 - 16:59	Evening	38,2	80	32,0	32,0	19,36	577,31	116,41
2019/05/22	CH2205	Male	Three y/o	Treatment (Wk 2)	Treatment	Fast Day	No	16:25	16:00 - 16:59	Evening	38,1	56	28,8	28,8	19,26	549,01	112,79
2019/05/22	CH2206	Male	Three y/o	Treatment (Wk 2)	Treatment	Fast Day	No	16:25	16:00 - 16:59	Evening	38,2		319,0	319,0	19,36		196,59
2019/05/22	CH2205	Male	Three y/o	Treatment (Wk 2)	Treatment	Fast Day	No	16:30	16:00 - 16:59	Evening	38,1	102	288,4	288,4	19,26	595,30	193,03
2019/05/22	CH2206	Male	Three y/o	Treatment (Wk 2)	Treatment	Fast Day	No	16:30	16:00 - 16:59	Evening	38,3	124	49,0	49,0	19,46	609,04	131,09
2019/05/22	CH2205	Male	Three y/o	Treatment (Wk 2)	Treatment	Fast Day	No	16:35	16:00 - 16:59	Evening	38,2	167	86,6	86,6	19,36	628,84	150,83
2019/05/22	CH2206	Male	Three y/o	Treatment (Wk 2)	Treatment	Fast Day	No	16:35	16:00 - 16:59	Evening	38,3	58	41,4	41,4	19,46	551,90	125,27
2019/05/22	CH2205	Male	Three y/o	Treatment (Wk 2)	Treatment	Fast Day	No	16:40	16:00 - 16:59	Evening	38,1		32,6	32,6	19,26		117,05
2019/05/22	CH2206	Male	Three y/o	Treatment (Wk 2)	Treatment	Fast Day	No	16:40	16:00 - 16:59	Evening	38,2	55	57,2	57,2	19,36	547,52	136,44
2019/05/22	CH2205	Male	Three y/o	Treatment (Wk 2)	Treatment	Fast Day	No	16:45	16:00 - 16:59	Evening	38,1		23,2	23,2	19,26		105,39
2019/05/22	CH2206	Male	Three y/o	Treatment (Wk 2)	Treatment	Fast Day	No	16:45	16:00 - 16:59	Evening	38,0	46	53,2	53,2	19,15	532,38	133,93
2019/05/22	CH2205	Male	Three y/o	Treatment (Wk 2)	Treatment	Fast Day	No	16:50	16:00 - 16:59	Evening	38,1	91	113,6	113,6	19,26	586,98	160,29
2019/05/22	CH2206	Male	Three y/o	Treatment (Wk 2)	Treatment	Fast Day	No	16:50	16:00 - 16:59	Evening	37,9	125	161,2	161,2	19,05	609,60	172,54
2019/05/22	CH2205	Male	Three y/o	Treatment (Wk 2)	Treatment	Fast Day	No	16:55	16:00 - 16:59	Evening	38,0		28,6	28,6	19,15		112,56
2019/05/22	CH2206	Male	Three y/o	Treatment (Wk 2)	Treatment	Fast Day	No	16:55	16:00 - 16:59	Evening	38,1	109	97,0	97,0	19,26	600,04	154,78
2019/05/22	CH2205	Male	Three y/o	Treatment (Wk 2)	Treatment	Fast Day	No	17:00	17:00 - 17:59	Evening	37,9	47	22,4	22,4	19,05	534,23	104,19
2019/05/22	CH2206	Male	Three y/o	Treatment (Wk 2)	Treatment	Fast Day	No	17:00	17:00 - 17:59	Evening	38,1	98	64,2	64,2	19,26	592,41	140,44
2019/05/22	CH2205	Male	Three y/o	Treatment (Wk 2)	Treatment	Fast Day	No	17:05	17:00 - 17:59	Evening	37,9	60	9,4	9,4	19,05	554,67	74,66
2019/05/22	CH2206	Male	Three y/o	Treatment (Wk 2)	Treatment	Fast Day	No	17:05	17:00 - 17:59	Evening	38,1	99	133,2	133,2	19,26	593,14	165,86
2019/05/22	CH2205	Male	Three y/o	Treatment (Wk 2)	Treatment	Fast Day	No	17:10	17:00 - 17:59	Evening	38,0		30,8	30,8	19,15		115,10
2019/05/22	CH2206	Male	Three y/o	Treatment (Wk 2)	Treatment	Fast Day	No	17:10	17:00 - 17:59	Evening	38,1	107	207,2	207,2	19,26	598,73	181,36
2019/05/22	CH2205	Male	Three y/o	Treatment (Wk 2)	Treatment	Fast Day	No	17:15	17:00 - 17:59	Evening	38,0	117	32,6	32,6	19,15	605,02	117,05
2019/05/22	CH2206	Male	Three y/o	Treatment (Wk 2)	Treatment	Fast Day	No	17:15	17:00 - 17:59	Evening	38,2	154	30,8	30,8	19,36	623,58	115,10
2019/05/22	CH2205	Male	Three y/o	Treatment (Wk 2)	Treatment	Fast Day	No	17:20	17:00 - 17:59	Evening	38,0		26,0	26,0	19,15		109,29
2019/05/22	CH2206	Male	Three y/o	Treatment (Wk 2)	Treatment	Fast Day	No	17:20	17:00 - 17:59	Evening	38,2	88	49,0	49,0	19,36	584,49	131,09
2019/05/22	CH2205	Male	Three y/o	Treatment (Wk 2)	Treatment	Fast Day	No	17:25	17:00 - 17:59	Evening	38,0	43	21,0	21,0	19,15	526,50	101,98
2019/05/22	CH2206	Male	Three y/o	Treatment (Wk 2)	Treatment	Fast Day	No	17:25	17:00 - 17:59	Evening	38,1	52	48,8	48,8	19,26	542,83	130,95
2019/05/22	CH2205	Male	Three y/o	Treatment (Wk 2)	Treatment	Fast Day	No	17:30	17:00 - 17:59	Evening	38,0	38	6,4	6,4	19,15	515,48	61,70
2019/05/22	CH2206	Male	Three y/o	Treatment (Wk 2)	Treatment	Fast Day	No	17:30	17:00 - 17:59	Evening	37,9	51	41,4	41,4	19,05	541,20	125,27
2019/05/22	CH2205	Male	Three y/o	Treatment (Wk 2)	Treatment	Fast Day	No	17:35	17:00 - 17:59	Evening	38,0	41	8,0	8,0	19,15	522,29	69,22
2019/05/22	CH2206	Male	Three y/o	Treatment (Wk 2)	Treatment	Fast Day	No	17:35	17:00 - 17:59	Evening	37,9	49	54,2	54,2	19,05	537,80	134,57
2019/05/22	CH2205	Male	Three y/o	Treatment (Wk 2)	Treatment	Fast Day	No	17:40	17:00 - 17:59	Evening	37,9	59	61,4	61,4	19,05	553,30	138,89
2019/05/22	CH2206	Male	Three y/o	Treatment (Wk 2)	Treatment	Fast Day	No	17:40	17:00 - 17:59	Evening	37,9	42	62,8	62,8	19,05	524,42	139,67
2019/05/22	CH2205	Male	Three y/o	Treatment (Wk 2)	Treatment	Fast Day	No	17:45	17:00 - 17:59	Evening	37,9		20,0	20,0	19,05		100,31
2019/05/22	CH2206	Male	Three y/o	Treatment (Wk 2)	Treatment	Fast Day	No	17:45	17:00 - 17:59	Evening	37,8		50,4	50,4	18,95		132,06
2019/05/22	CH2205	Male	Three y/o	Treatment (Wk 2)	Treatment	Fast Day	No	17:50	17:00 - 17:59	Evening	37,9	85	11,2	11,2	19,05	581,90	80,59
2019/05/22	CH2206	Male	Three y/o	Treatment (Wk 2)	Treatment	Fast Day	No	17:50	17:00 - 17:59	Evening	37,7	79	13,4	13,4	18,85	576,36	86,68
2019/05/22	CH2205	Male	Three y/o	Treatment (Wk 2)	Treatment	Fast Day	No	17:55	17:00 - 17:59	Evening	37,7	42	3,2	3,2	18,85	524,42	38,49
2019/05/22	CH2206	Male	Three y/o	Treatment (Wk 2)	Treatment	Fast Day	No	17:55	17:00 - 17:59	Evening	37,5	50	4,8	4,8	18,65	539,52	52,04
2019/05/22	CH2205	Male	Three y/o	Treatment (Wk 2)	Treatment	Fast Day	No	18:00	18:00 - 18:59	Evening	37,7	43	6,8	6,8	18,85	526,50	63,74
2019/05/22	CH2206	Male	Three y/o	Treatment (Wk 2)	Treatment	Fast Day	No	18:00	18:00 - 18:59	Evening	37,7	47	5,0	5,0	18,85	534,23	53,41
2019/05/22	CH2205	Male	Three y/o	Treatment (Wk 2)	Treatment	Fast Day	No	18:05	18:00 - 18:59	Evening	37,7	51	8,8	8,8	18,85	541,20	72,43
2019/05/22	CH2206	Male	Three y/o	Treatment (Wk 2)	Treatment	Fast Day	No	18:05	18:00 - 18:59	Evening	37,8	41	4,6	4,6	18,95	522,29	50,61
2019/05/22	CH2205	Male	Three y/o	Treatment (Wk 2)	Treatment	Fast Day	No	18:10	18:00 - 18:59	Evening	37,6	52	5,4	5,4	18,75	542,83	55,99
2019/05/22	CH2206	Male	Three y/o	Treatment (Wk 2)	Treatment	Fast Day	No	18:10	18:00 - 18:59	Evening	37,8	46	5,0	5,0	18,95	532,38	53,41
2019/05/22	CH2205	Male	Three y/o	Treatment (Wk 2)	Treatment	Fast Day	No	18:15	18:00 - 18:59	Evening	37,5	47	8,0	8,0	18,65	534,23	69,22
2019/05/22	CH2206	Male	Three y/o	Treatment (Wk 2)	Treatment	Fast Day	No	18:15	18:00 - 18:59	Evening	37,8	82	12,4	12,4	18,95	579,19	84,04
2019/05/22	CH2205	Male	Three y/o	Treatment (Wk 2)	Treatment	Fast Day	No	18:20	18:00 - 18:59	Evening	37,5	62	10,8	10,8	18,65	557,33	79,36
2019/05/22	CH2206	Male	Three y/o	Treatment (Wk 2)	Treatment	Fast Day	No	18:20	18:00 - 18:59	Evening	37,8	53	12,8	12,8	18,95	544,43	85,12
2019/05/22	CH2205	Male	Three y/o	Treatment (Wk 2)	Treatment	Fast Day	No	18:25	18:00 - 18:59	Evening	37,5	110	13,0	13,0	18,65	600,69	85,65
2019/05/22	CH2206	Male	Three y/o	Treatment (Wk 2)	Treatment	Fast Day	No	18:25	18:00 - 18:59	Evening	37,8	50	28,2	28,2	18,95	539,52	112,07
2019/05/22	CH2205	Male	Three y/o	Treatment (Wk 2)	Treatment	Fast Day	No	18:30	18:00 - 18:59	Evening	37,5	51	22,8	22,8	18,65	541,20	104,79

2019/05/22	CH2206	Male	Three y/o	Treatment (Wk 2)	Treatment	Fast Day	No	18:30	18:00 - 18:59	Evening	37,8	45	41,2	41,2	18,95	530,47	125,11
2019/05/22	CH2205	Male	Three y/o	Treatment (Wk 2)	Treatment	Fast Day	No	18:35	18:00 - 18:59	Evening	37,5	44	24,0	24,0	18,65	528,51	106,55
2019/05/22	CH2206	Male	Three y/o	Treatment (Wk 2)	Treatment	Fast Day	No	18:35	18:00 - 18:59	Evening	37,8	43	44,6	44,6	18,95	526,50	127,84
2019/05/22	CH2205	Male	Three y/o	Treatment (Wk 2)	Treatment	Fast Day	No	18:40	18:00 - 18:59	Evening	37,5	59	28,4	28,4	18,65	553,30	112,31
2019/05/22	CH2206	Male	Three y/o	Treatment (Wk 2)	Treatment	Fast Day	No	18:40	18:00 - 18:59	Evening	37,8	80	64,0	64,0	18,95	577,31	140,33
2019/05/22	CH2205	Male	Three y/o	Treatment (Wk 2)	Treatment	Fast Day	No	18:45	18:00 - 18:59	Evening	37,5	44	13,4	13,4	18,65	528,51	86,68
2019/05/22	CH2206	Male	Three y/o	Treatment (Wk 2)	Treatment	Fast Day	No	18:45	18:00 - 18:59	Evening	37,7	54	37,0	37,0	18,85	545,99	121,40
2019/05/22	CH2205	Male	Three y/o	Treatment (Wk 2)	Treatment	Fast Day	No	18:50	18:00 - 18:59	Evening	37,4	79	28,4	28,4	18,55	576,36	112,31
2019/05/22	CH2206	Male	Three y/o	Treatment (Wk 2)	Treatment	Fast Day	No	18:50	18:00 - 18:59	Evening	37,6	78	48,2	48,2	18,75	575,38	130,52
2019/05/22	CH2205	Male	Three y/o	Treatment (Wk 2)	Treatment	Fast Day	No	18:55	18:00 - 18:59	Evening	37,4	54	28,8	28,8	18,55	545,99	112,79
2019/05/22	CH2206	Male	Three y/o	Treatment (Wk 2)	Treatment	Fast Day	No	18:55	18:00 - 18:59	Evening	37,7	46	64,0	64,0	18,85	532,38	140,33
2019/05/22	CH2205	Male	Three y/o	Treatment (Wk 2)	Treatment	Fast Day	No	19:00	19:00 - 19:59	Evening	37,3	44	24,4	24,4	18,46	528,51	107,11
2019/05/22	CH2206	Male	Three y/o	Treatment (Wk 2)	Treatment	Fast Day	No	19:00	19:00 - 19:59	Evening	37,7	43	71,0	71,0	18,85	526,50	143,93
2019/05/22	CH2205	Male	Three y/o	Treatment (Wk 2)	Treatment	Fast Day	No	19:05	19:00 - 19:59	Evening	37,5		18,8	18,8	18,65		98,20
2019/05/22	CH2206	Male	Three y/o	Treatment (Wk 2)	Treatment	Fast Day	No	19:05	19:00 - 19:59	Evening	37,5	47	62,6	62,6	18,65	534,23	139,56
2019/05/22	CH2205	Male	Three y/o	Treatment (Wk 2)	Treatment	Fast Day	No	19:10	19:00 - 19:59	Evening	37,5		20,6	20,6	18,65		101,32
2019/05/22	CH2206	Male	Three y/o	Treatment (Wk 2)	Treatment	Fast Day	No	19:10	19:00 - 19:59	Evening	37,4	49	62,0	62,0	18,55	537,80	139,23
2019/05/22	CH2205	Male	Three y/o	Treatment (Wk 2)	Treatment	Fast Day	No	19:15	19:00 - 19:59	Evening	37,4		25,0	25,0	18,55		107,94
2019/05/22	CH2206	Male	Three y/o	Treatment (Wk 2)	Treatment	Fast Day	No	19:15	19:00 - 19:59	Evening	37,4	64	60,6	60,6	18,55	559,88	138,44
2019/05/22	CH2205	Male	Three y/o	Treatment (Wk 2)	Treatment	Fast Day	No	19:20	19:00 - 19:59	Evening	37,4	58	26,4	26,4	18,55	551,90	109,81
2019/05/22	CH2206	Male	Three y/o	Treatment (Wk 2)	Treatment	Fast Day	No	19:20	19:00 - 19:59	Evening	37,4	52	60,6	60,6	18,55	542,83	138,44
2019/05/22	CH2205	Male	Three y/o	Treatment (Wk 2)	Treatment	Fast Day	No	19:25	19:00 - 19:59	Evening	37,4		22,2	22,2	18,55		103,88
2019/05/22	CH2206	Male	Three y/o	Treatment (Wk 2)	Treatment	Fast Day	No	19:25	19:00 - 19:59	Evening	37,4	43	63,4	63,4	18,55	526,50	140,00
2019/05/22	CH2205	Male	Three y/o	Treatment (Wk 2)	Treatment	Fast Day	No	19:30	19:00 - 19:59	Evening	37,4	39	22,4	22,4	18,55	517,82	104,19
2019/05/22	CH2206	Male	Three y/o	Treatment (Wk 2)	Treatment	Fast Day	No	19:30	19:00 - 19:59	Evening	37,4	55	55,4	55,4	18,55	547,52	135,33
2019/05/22	CH2205	Male	Three y/o	Treatment (Wk 2)	Treatment	Fast Day	No	19:35	19:00 - 19:59	Evening	37,4		21,6	21,6	18,55		102,94
2019/05/22	CH2206	Male	Three y/o	Treatment (Wk 2)	Treatment	Fast Day	No	19:35	19:00 - 19:59	Evening	37,4	41	58,2	58,2	18,55	522,29	137,04
2019/05/22	CH2205	Male	Three y/o	Treatment (Wk 2)	Treatment	Fast Day	No	19:40	19:00 - 19:59	Evening	37,4		23,8	23,8	18,55		106,26
2019/05/22	CH2206	Male	Three y/o	Treatment (Wk 2)	Treatment	Fast Day	No	19:40	19:00 - 19:59	Evening	37,4	40	58,8	58,8	18,55	520,09	137,39
2019/05/22	CH2205	Male	Three y/o	Treatment (Wk 2)	Treatment	Fast Day	No	19:45	19:00 - 19:59	Evening	37,4	48	23,8	23,8	18,55	536,04	106,26
2019/05/22	CH2206	Male	Three y/o	Treatment (Wk 2)	Treatment	Fast Day	No	19:45	19:00 - 19:59	Evening	37,4	40	58,8	58,8	18,55	520,09	137,39
2019/05/22	CH2205	Male	Three y/o	Treatment (Wk 2)	Treatment	Fast Day	No	19:50	19:00 - 19:59	Evening	37,4		24,6	24,6	18,55		107,39
2019/05/22	CH2206	Male	Three y/o	Treatment (Wk 2)	Treatment	Fast Day	No	19:50	19:00 - 19:59	Evening	37,4	41	46,4	46,4	18,55	522,29	129,21
2019/05/22	CH2205	Male	Three y/o	Treatment (Wk 2)	Treatment	Fast Day	No	19:55	19:00 - 19:59	Evening	37,4		27,0	27,0	18,55		110,58
2019/05/22	CH2206	Male	Three y/o	Treatment (Wk 2)	Treatment	Fast Day	No	19:55	19:00 - 19:59	Evening	37,4	46	55,4	55,4	18,55	532,38	135,33
2019/05/22	CH2205	Male	Three y/o	Treatment (Wk 2)	Treatment	Fast Day	No	20:00	20:00 - 20:59	Night	37,5	140	17,6	17,6	18,65	617,28	95,95
2019/05/22	CH2206	Male	Three y/o	Treatment (Wk 2)	Treatment	Fast Day	No	20:00	20:00 - 20:59	Night	37,4	75	62,4	62,4	18,55	572,36	139,45
2019/05/22	CH2205	Male	Three y/o	Treatment (Wk 2)	Treatment	Fast Day	No	20:05	20:00 - 20:59	Night	37,5	39	16,0	16,0	18,65	517,82	92,71
2019/05/22	CH2206	Male	Three y/o	Treatment (Wk 2)	Treatment	Fast Day	No	20:05	20:00 - 20:59	Night	37,3	43	64,6	64,6	18,46	526,50	140,65
2019/05/22	CH2205	Male	Three y/o	Treatment (Wk 2)	Treatment	Fast Day	No	20:10	20:00 - 20:59	Night	37,5	38	17,2	17,2	18,65	515,48	95,17
2019/05/22	CH2206	Male	Three y/o	Treatment (Wk 2)	Treatment	Fast Day	No	20:10	20:00 - 20:59	Night	37,3	45	62,2	62,2	18,46	530,47	139,34
2019/05/22	CH2205	Male	Three y/o	Treatment (Wk 2)	Treatment	Fast Day	No	20:15	20:00 - 20:59	Night	37,5	43	21,2	21,2	18,65	526,50	102,31
2019/05/22	CH2206	Male	Three y/o	Treatment (Wk 2)	Treatment	Fast Day	No	20:15	20:00 - 20:59	Night	37,3	43	61,2	61,2	18,46	526,50	138,78
2019/05/22	CH2205	Male	Three y/o	Treatment (Wk 2)	Treatment	Fast Day	No	20:20	20:00 - 20:59	Night	37,5	123	13,6	13,6	18,65	608,49	87,18
2019/05/22	CH2206	Male	Three y/o	Treatment (Wk 2)	Treatment	Fast Day	No	20:20	20:00 - 20:59	Night	37,4	50	66,8	66,8	18,55	539,52	141,81
2019/05/22	CH2205	Male	Three y/o	Treatment (Wk 2)	Treatment	Fast Day	No	20:25	20:00 - 20:59	Night	37,6	44	21,4	21,4	18,75	528,51	102,63
2019/05/22	CH2206	Male	Three y/o	Treatment (Wk 2)	Treatment	Fast Day	No	20:25	20:00 - 20:59	Night	37,4	46	63,6	63,6	18,55	532,38	140,11
2019/05/22	CH2205	Male	Three y/o	Treatment (Wk 2)	Treatment	Fast Day	No	20:30	20:00 - 20:59	Night	37,6	81	18,4	18,4	18,75	578,26	97,47
2019/05/22	CH2206	Male	Three y/o	Treatment (Wk 2)	Treatment	Fast Day	No	20:30	20:00 - 20:59	Night	37,4	43	65,4	65,4	18,55	526,50	141,08
2019/05/22	CH2205	Male	Three y/o	Treatment (Wk 2)	Treatment	Fast Day	No	20:35	20:00 - 20:59	Night	37,5	54	20,8	20,8	18,65	545,99	101,65
2019/05/22	CH2206	Male	Three y/o	Treatment (Wk 2)	Treatment	Fast Day	No	20:35	20:00 - 20:59	Night	37,4	45	69,2	69,2	18,55	530,47	143,04
2019/05/22	CH2205	Male	Three y/o	Treatment (Wk 2)	Treatment	Fast Day	No	20:40	20:00 - 20:59	Night	37,5	52	25,4	25,4	18,65	542,83	108,49
2019/05/22	CH2206	Male	Three y/o	Treatment (Wk 2)	Treatment	Fast Day	No	20:40	20:00 - 20:59	Night	37,4	41	61,4	61,4	18,55	522,29	138,89
2019/05/22	CH2205	Male	Three y/o	Treatment (Wk 2)	Treatment	Fast Day	No	20:45	20:00 - 20:59	Night	37,5	42	27,8	27,8	18,65	524,42	111,58
2019/05/22	CH2206	Male	Three y/o	Treatment (Wk 2)	Treatment	Fast Day	No	20:45	20:00 - 20:59	Night	37,4	57	67,0	67,0	18,55	550,47	141,92
2019/05/22	CH2205	Male	Three y/o	Treatment (Wk 2)	Treatment	Fast Day	No	20:50	20:00 - 20:59	Night	37,5	52	25,6	25,6	18,65	542,83	108,76
2019/05/22	CH2206	Male	Three y/o	Treatment (Wk 2)	Treatment	Fast Day	No	20:50	20:00 - 20:59	Night	37,4	44	67,2	67,2	18,55	528,51	142,02
2019/05/22	CH2205	Male	Three y/o	Treatment (Wk 2)	Treatment	Fast Day	No	20:55	20:00 - 20:59	Night	37,5	42	22,2	22,2	18,65	524,42	103,88
2019/05/22	CH2206	Male	Three y/o	Treatment (Wk 2)	Treatment	Fast Day	No	20:55	20:00 - 20:59	Night	37,4	48	65,4	65,4	18,55	536,04	141,08
2019/05/22	CH2205	Male	Three y/o	Treatment (Wk 2)	Treatment	Fast Day	No	21:00	21:00 - 21:59	Night	37,5	46	24,8	24,8	18,65	532,38	107,67
2019/05/22	CH2206	Male	Three y/o	Treatment (Wk 2)	Treatment	Fast Day	No	21:00	21:00 - 21:59	Night	37,4	44	58,0	58,0	18,55	528,51	136,92
2019/05/22	CH2205	Male	Three y/o	Treatment (Wk 2)	Treatment	Fast Day	No	21:05	21:00 - 21:59	Night	37,5	77	19,6	19,6	18,65	574,39	99,63
2019/05/22	CH2206	Male	Three y/o	Treatment (Wk 2)	Treatment	Fast Day	No	21:05	21:00 - 21:59	Night	37,4	50	52,6	52,6	18,55	539,52	133,54
2019/05/22	CH2205	Male	Three y/o	Treatment (Wk 2)	Treatment	Fast Day	No	21:10	21:00 - 21:59	Night	37,5	93	26,6	26,6	18,65	588,58	110,07
2019/05/22	CH2206	Male	Three y/o	Treatment (Wk 2)	Treatment	Fast Day	No	21:10	21:00 - 21:59	Night	37,4	82	44,8	44,8	18,55	579,19	127,99
2019/05/22	CH2205	Male	Three y/o	Treatment (Wk 2)	Treatment	Fast Day	No	21:15	21:00 - 21:59	Night	37,5	42	24,6	24,6	18,65	524,42	107,39
2019/05/22	CH2206	Male	Three y/o	Treatment (Wk 2)	Treatment	Fast Day	No	21:15	21:00 - 21:59	Night	37,3	93	40,6	40,6	18,46	588,58	124,60
2019/05/22	CH2205	Male	Three y/o	Treatment (Wk 2)	Treatment	Fast Day	No	21:20	21:00 - 21:59	Night	37,4	37	30,6	30,6	18,55	513,06	114,87
2019/05/22	CH2206	Male	Three y/o	Treatment (Wk 2)	Treatment	Fast Day	No	21:20	21:00 - 21:59	Night	37,3	48	26,0	26,0	18,46	536,04	109,29
2019/05/22	CH2205	Male	Three y/o	Treatment (Wk 2)	Treatment	Fast Day	No	21:25	21:00 - 21:59	Night	37,4	52	24,4	24,4	18,55	542,83	107,11

	2019/05/22	CH2206	Male	Three y/o	Treatment (Wk 2)	Treatment	Fast Day	No	21:25	21:00 - 21:59	Night	37,3	44	20,4	20,4	18,46	528,51	100,99
	2019/05/22	CH2205	Male	Three y/o	Treatment (Wk 2)	Treatment	Fast Day	No	21:30	21:00 - 21:59	Night	37,4	57	26,8	26,8	18,55	550,47	110,33
	2019/05/22	CH2206	Male	Three y/o	Treatment (Wk 2)	Treatment	Fast Day	No	21:30	21:00 - 21:59	Night	37,3	72	16,6	16,6	18,46	569,20	93,96
	2019/05/22	CH2205	Male	Three y/o	Treatment (Wk 2)	Treatment	Fast Day	No	21:35	21:00 - 21:59	Night	37,4	79	26,4	26,4	18,55	576,36	109,81
	2019/05/22	CH2206	Male	Three y/o	Treatment (Wk 2)	Treatment	Fast Day	No	21:35	21:00 - 21:59	Night	37,3	45	17,8	17,8	18,46	530,47	96,34
	2019/05/22	CH2205	Male	Three y/o	Treatment (Wk 2)	Treatment	Fast Day	No	21:40	21:00 - 21:59	Night	37,3	34	28,4	28,4	18,46	505,29	112,31
	2019/05/22	CH2206	Male	Three y/o	Treatment (Wk 2)	Treatment	Fast Day	No	21:40	21:00 - 21:59	Night	37,3	52	13,6	13,6	18,46	542,83	87,18
	2019/05/22	CH2205	Male	Three y/o	Treatment (Wk 2)	Treatment	Fast Day	No	21:45	21:00 - 21:59	Night	37,3	42	28,8	28,8	18,46	524,42	112,79
	2019/05/22	CH2206	Male	Three y/o	Treatment (Wk 2)	Treatment	Fast Day	No	21:45	21:00 - 21:59	Night	37,3	78	4,2	4,2	18,46	575,38	47,57
	2019/05/22	CH2205	Male	Three y/o	Treatment (Wk 2)	Treatment	Fast Day	No	21:50	21:00 - 21:59	Night	37,3	37	24,6	24,6	18,46	513,06	107,39
	2019/05/22	CH2206	Male	Three y/o	Treatment (Wk 2)	Treatment	Fast Day	No	21:50	21:00 - 21:59	Night	37,3	47	14,6	14,6	18,46	534,23	89,59
	2019/05/22	CH2205	Male	Three y/o	Treatment (Wk 2)	Treatment	Fast Day	No	21:55	21:00 - 21:59	Night	37,3	41	26,2	26,2	18,46	522,29	109,55
	2019/05/22	CH2206	Male	Three y/o	Treatment (Wk 2)	Treatment	Fast Day	No	21:55	21:00 - 21:59	Night	37,3	43	23,2	23,2	18,46	526,50	105,39
	2019/05/22	CH2205	Male	Three y/o	Treatment (Wk 2)	Treatment	Fast Day	No	22:00	22:00 - 22:59	Night	37,3	36	26,0	26,0	18,46	510,56	109,29
	2019/05/22	CH2206	Male	Three y/o	Treatment (Wk 2)	Treatment	Fast Day	No	22:00	22:00 - 22:59	Night	37,3	55	27,8	27,8	18,46	547,52	111,58
	2019/05/22	CH2205	Male	Three y/o	Treatment (Wk 2)	Treatment	Fast Day	No	22:05	22:00 - 22:59	Night	37,3	79	55,4	55,4	18,46	576,36	135,33
	2019/05/22	CH2206	Male	Three y/o	Treatment (Wk 2)	Treatment	Fast Day	No	22:05	22:00 - 22:59	Night	37,3	46	28,8	28,8	18,46	532,38	112,79
	2019/05/22	CH2205	Male	Three y/o	Treatment (Wk 2)	Treatment	Fast Day	No	22:10	22:00 - 22:59	Night	37,3	44	5,4	5,4	18,46	528,51	55,99
	2019/05/22	CH2206	Male	Three y/o	Treatment (Wk 2)	Treatment	Fast Day	No	22:10	22:00 - 22:59	Night	37,4	71	50,2	50,2	18,55	568,10	131,92
	2019/05/22	CH2205	Male	Three y/o	Treatment (Wk 2)	Treatment	Fast Day	No	22:15	22:00 - 22:59	Night	37,3	43	7,4	7,4	18,46	526,50	66,59
	2019/05/22	CH2206	Male	Three y/o	Treatment (Wk 2)	Treatment	Fast Day	No	22:15	22:00 - 22:59	Night	37,4	45	47,8	47,8	18,55	530,47	130,23
	2019/05/22	CH2205	Male	Three y/o	Treatment (Wk 2)	Treatment	Fast Day	No	22:20	22:00 - 22:59	Night	37,3	45	9,4	9,4	18,46	530,47	74,66
	2019/05/22	CH2206	Male	Three y/o	Treatment (Wk 2)	Treatment	Fast Day	No	22:20	22:00 - 22:59	Night	37,4	43	50,0	50,0	18,55	526,50	131,79
	2019/05/22	CH2205	Male	Three y/o	Treatment (Wk 2)	Treatment	Fast Day	No	22:25	22:00 - 22:59	Night	37,3	85	6,2	6,2	18,46	581,90	60,63
	2019/05/22	CH2206	Male	Three y/o	Treatment (Wk 2)	Treatment	Fast Day	No	22:25	22:00 - 22:59	Night	37,4	42	55,6	55,6	18,55	524,42	135,46
	2019/05/22	CH2205	Male	Three y/o	Treatment (Wk 2)	Treatment	Fast Day	No	22:30	22:00 - 22:59	Night	37,3	38	8,4	8,4	18,46	515,48	70,86
	2019/05/22	CH2206	Male	Three y/o	Treatment (Wk 2)	Treatment	Fast Day	No	22:30	22:00 - 22:59	Night	37,4	44	51,4	51,4	18,55	528,51	132,74
	2019/05/22	CH2205	Male	Three y/o	Treatment (Wk 2)	Treatment	Fast Day	No	22:35	22:00 - 22:59	Night	37,3	49	12,6	12,6	18,46	537,80	84,59
	2019/05/22	CH2206	Male	Three y/o	Treatment (Wk 2)	Treatment	Fast Day	No	22:35	22:00 - 22:59	Night	37,4	71	46,8	46,8	18,55	568,10	129,50
	2019/05/22	CH2205	Male	Three y/o	Treatment (Wk 2)	Treatment	Fast Day	No	22:40	22:00 - 22:59	Night	37,3	36	15,6	15,6	18,46	510,56	91,85
	2019/05/22	CH2206	Male	Three y/o	Treatment (Wk 2)	Treatment	Fast Day	No	22:40	22:00 - 22:59	Night	37,4	48	44,2	44,2	18,55	536,04	127,53
	2019/05/22	CH2205	Male	Three y/o	Treatment (Wk 2)	Treatment	Fast Day	No	22:45	22:00 - 22:59	Night	37,3	51	11,4	11,4	18,46	541,20	81,19
	2019/05/22	CH2206	Male	Three y/o	Treatment (Wk 2)	Treatment	Fast Day	No	22:45	22:00 - 22:59	Night	37,4	51	41,8	41,8	18,55	541,20	125,60
	2019/05/22	CH2205	Male	Three y/o	Treatment (Wk 2)	Treatment	Fast Day	No	22:50	22:00 - 22:59	Night	37,3	54	11,4	11,4	18,46	545,99	81,19
	2019/05/22	CH2206	Male	Three y/o	Treatment (Wk 2)	Treatment	Fast Day	No	22:50	22:00 - 22:59	Night	37,4	50	43,8	43,8	18,55	539,52	127,22
	2019/05/22	CH2205	Male	Three y/o	Treatment (Wk 2)	Treatment	Fast Day	No	22:55	22:00 - 22:59	Night	37,3	46	15,6	15,6	18,46	532,38	91,85
	2019/05/22	CH2206	Male	Three y/o	Treatment (Wk 2)	Treatment	Fast Day	No	22:55	22:00 - 22:59	Night	37,4	45	46,2	46,2	18,55	530,47	129,06
	2019/05/22	CH2205	Male	Three y/o	Treatment (Wk 2)	Treatment	Fast Day	No	23:00	23:00 - 23:59	Night	37,3	39	17,2	17,2	18,46	517,82	95,17
	2019/05/22	CH2206	Male	Three y/o	Treatment (Wk 2)	Treatment	Fast Day	No	23:00	23:00 - 23:59	Night	37,4	43	46,2	46,2	18,55	526,50	129,06
	2019/05/22	CH2205	Male	Three y/o	Treatment (Wk 2)	Treatment	Fast Day	No	23:05	23:00 - 23:59	Night	37,3	41	15,0	15,0	18,46	522,29	90,51
	2019/05/22	CH2206	Male	Three y/o	Treatment (Wk 2)	Treatment	Fast Day	No	23:05	23:00 - 23:59	Night	37,4	38	46,2	46,2	18,55	515,48	129,06
	2019/05/22	CH2205	Male	Three y/o	Treatment (Wk 2)	Treatment	Fast Day	No	23:10	23:00 - 23:59	Night	37,3	45	14,0	14,0	18,46	530,47	88,17
	2019/05/22	CH2206	Male	Three y/o	Treatment (Wk 2)	Treatment	Fast Day	No	23:10	23:00 - 23:59	Night	37,4	95	43,2	43,2	18,55	590,14	126,74
	2019/05/22	CH2205	Male	Three y/o	Treatment (Wk 2)	Treatment	Fast Day	No	23:15	23:00 - 23:59	Night	37,3	46	16,2	16,2	18,46	532,38	93,13
	2019/05/22	CH2206	Male	Three y/o	Treatment (Wk 2)	Treatment	Fast Day	No	23:15	23:00 - 23:59	Night	37,4	48	38,6	38,6	18,55	536,04	122,86
	2019/05/22	CH2205	Male	Three y/o	Treatment (Wk 2)	Treatment	Fast Day	No	23:20	23:00 - 23:59	Night	37,4	98	31,0	31,0	18,55	592,41	115,32
	2019/05/22	CH2206	Male	Three y/o	Treatment (Wk 2)	Treatment	Fast Day	No	23:20	23:00 - 23:59	Night	37,4	113	32,2	32,2	18,55	602,58	116,63
	2019/05/22	CH2205	Male	Three y/o	Treatment (Wk 2)	Treatment	Fast Day	No	23:25	23:00 - 23:59	Night	37,3		41,0	41,0	18,46		124,94
	2019/05/22	CH2206	Male	Three y/o	Treatment (Wk 2)	Treatment	Fast Day	No	23:25	23:00 - 23:59	Night	37,4	115	57,2	57,2	18,55	603,82	136,44
	2019/05/22	CH2205	Male	Three y/o	Treatment (Wk 2)	Treatment	Fast Day	No	23:30	23:00 - 23:59	Night	37,4	103	30,8	30,8	18,55	596,00	115,10
	2019/05/22	CH2206	Male	Three y/o	Treatment (Wk 2)	Treatment	Fast Day	No	23:30	23:00 - 23:59	Night	37,3	129	44,6	44,6	18,46	611,75	127,84
	2019/05/22	CH2205	Male	Three y/o	Treatment (Wk 2)	Treatment	Fast Day	No	23:35	23:00 - 23:59	Night	37,4	45	30,2	30,2	18,55	530,47	114,42
	2019/05/22	CH2206	Male	Three y/o	Treatment (Wk 2)	Treatment	Fast Day	No	23:35	23:00 - 23:59	Night	37,3	47	64,6	64,6	18,46	534,23	140,65
	2019/05/22	CH2205	Male	Three y/o	Treatment (Wk 2)	Treatment	Fast Day	No	23:40	23:00 - 23:59	Night	37,4	42	27,8	27,8	18,55	524,42	111,58
	2019/05/22	CH2206	Male	Three y/o	Treatment (Wk 2)	Treatment	Fast Day	No	23:40	23:00 - 23:59	Night	36,9	49	63,4	63,4	18,06	537,80	140,00
	2019/05/22	CH2205	Male	Three y/o	Treatment (Wk 2)	Treatment	Fast Day	No	23:45	23:00 - 23:59	Night	37,4	35	22,8	22,8	18,55	507,97	104,79
	2019/05/22	CH2206	Male	Three y/o	Treatment (Wk 2)	Treatment	Fast Day	No	23:45	23:00 - 23:59	Night	36,9	44	59,8	59,8	18,06	528,51	137,98
	2019/05/22	CH2205	Male	Three y/o	Treatment (Wk 2)	Treatment	Fast Day	No	23:50	23:00 - 23:59	Night	37,4	50	14,4	14,4	18,55	539,52	89,12
	2019/05/22	CH2206	Male	Three y/o	Treatment (Wk 2)	Treatment	Fast Day	No	23:50	23:00 - 23:59	Night	37,2	47	62,2	62,2	18,36	534,23	139,34
	2019/05/22	CH2205	Male	Three y/o	Treatment (Wk 2)	Treatment	Fast Day	No	23:55	23:00 - 23:59	Night	37,3	47	19,2	19,2	18,46	534,23	98,92
	2019/05/22	CH2206	Male	Three y/o	Treatment (Wk 2)	Treatment	Fast Day	No	23:55	23:00 - 23:59	Night	37,2	48	51,8	51,8	18,36	536,04	133,01
9	2019/05/23	CH2205	Male	Three y/o	Treatment (Wk 2)	Treatment	Feed Day	No	00:00	00:00 - 00:59	Early Morning	37,4	58	26,0	26,0	18,55	551,90	109,29
	2019/05/23	CH2206	Male	Three y/o	Treatment (Wk 2)	Treatment	Feed Day	No	00:00	00:00 - 00:59	Early Morning	37,2	152	60,2	60,2	18,36	622,73	138,21
	2019/05/23	CH2205	Male	Three y/o	Treatment (Wk 2)	Treatment	Feed Day	No	00:05	00:00 - 00:59	Early Morning	37,2	58	25,0	25,0	18,36	551,90	107,94
	2019/05/23	CH2206	Male	Three y/o	Treatment (Wk 2)	Treatment	Feed Day	No	00:05	00:00 - 00:59	Early Morning	37,2	46	51,4	51,4	18,36	532,38	132,74
	2019/05/23	CH2205	Male	Three y/o	Treatment (Wk 2)	Treatment	Feed Day	No	00:10	00:00 - 00:59	Early Morning	37,2	42	23,8	23,8	18,36	524,42	106,26
	2019/05/23	CH2206	Male	Three y/o	Treatment (Wk 2)	Treatment	Feed Day	No	00:10	00:00 - 00:59	Early Morning	37,2	49	59,4	59,4	18,36	537,80	137,75
	2019/05/23	CH2205	Male	Three y/o	Treatment (Wk 2)	Treatment	Feed Day	No	00:15	00:00 - 00:59	Early Morning	37,1	88	25,0	25,0	18,26	584,49	107,94
	2019/05/23	CH2206	Male	Three y/o	Treatment (Wk 2)	Treatment	Feed Day	No	00:15	00:00 - 00:59	Early Morning	37,2	83	58,4	58,4	18,36	580,11	137,16
	2019/05/23	CH2205	Male	Three y/o	Treatment (Wk 2)	Treatment	Feed Day	No	00:20	00:00 - 00:59	Early Morning	37,1	56	24,2	24,2	18,26	549,01	106,83

2019/05/23	CH2206	Male	Three y/o	Treatment (Wk 2)	Treatment	Feed Day	No	00:20	00:00 - 00:59	Early Morning	37,2	51	61,8	61,8	18,36	541,20	139,12
2019/05/23	CH2205	Male	Three y/o	Treatment (Wk 2)	Treatment	Feed Day	No	00:25	00:00 - 00:59	Early Morning	37,1	43	23,6	23,6	18,26	526,50	105,97
2019/05/23	CH2206	Male	Three y/o	Treatment (Wk 2)	Treatment	Feed Day	No	00:25	00:00 - 00:59	Early Morning	37,2	46	48,6	48,6	18,36	532,38	130,81
2019/05/23	CH2205	Male	Three y/o	Treatment (Wk 2)	Treatment	Feed Day	No	00:30	00:00 - 00:59	Early Morning	37,1	64	25,2	25,2	18,26	559,88	108,22
2019/05/23	CH2206	Male	Three y/o	Treatment (Wk 2)	Treatment	Feed Day	No	00:30	00:00 - 00:59	Early Morning	37,2	48	48,4	48,4	18,36	536,04	130,66
2019/05/23	CH2205	Male	Three y/o	Treatment (Wk 2)	Treatment	Feed Day	No	00:35	00:00 - 00:59	Early Morning	37,2		29,0	29,0	18,36		113,03
2019/05/23	CH2206	Male	Three y/o	Treatment (Wk 2)	Treatment	Feed Day	No	00:35	00:00 - 00:59	Early Morning	37,1	51	57,2	57,2	18,26	541,20	136,44
2019/05/23	CH2205	Male	Three y/o	Treatment (Wk 2)	Treatment	Feed Day	No	00:40	00:00 - 00:59	Early Morning	37,2	35	24,2	24,2	18,36	507,97	106,83
2019/05/23	CH2206	Male	Three y/o	Treatment (Wk 2)	Treatment	Feed Day	No	00:40	00:00 - 00:59	Early Morning	37,1	50	59,4	59,4	18,26	539,52	137,75
2019/05/23	CH2205	Male	Three y/o	Treatment (Wk 2)	Treatment	Feed Day	No	00:45	00:00 - 00:59	Early Morning	37,2	71	29,4	29,4	18,36	568,10	113,50
2019/05/23	CH2206	Male	Three y/o	Treatment (Wk 2)	Treatment	Feed Day	No	00:45	00:00 - 00:59	Early Morning	37,1	56	53,4	53,4	18,26	549,01	134,06
2019/05/23	CH2205	Male	Three y/o	Treatment (Wk 2)	Treatment	Feed Day	No	00:50	00:00 - 00:59	Early Morning	37,2	36	24,2	24,2	18,36	510,56	106,83
2019/05/23	CH2206	Male	Three y/o	Treatment (Wk 2)	Treatment	Feed Day	No	00:50	00:00 - 00:59	Early Morning	37,1	82	51,6	51,6	18,26	579,19	132,87
2019/05/23	CH2205	Male	Three y/o	Treatment (Wk 2)	Treatment	Feed Day	No	00:55	00:00 - 00:59	Early Morning	37,2	43	26,2	26,2	18,36	526,50	109,55
2019/05/23	CH2206	Male	Three y/o	Treatment (Wk 2)	Treatment	Feed Day	No	00:55	00:00 - 00:59	Early Morning	37,1	53	56,8	56,8	18,26	544,43	136,20
2019/05/23	CH2205	Male	Three y/o	Treatment (Wk 2)	Treatment	Feed Day	No	01:00	01:00 - 01:59	Early Morning	37,2	44	26,6	26,6	18,36	528,51	110,07
2019/05/23	CH2206	Male	Three y/o	Treatment (Wk 2)	Treatment	Feed Day	No	01:00	01:00 - 01:59	Early Morning	37,1	86	21,0	21,0	18,26	582,77	101,98
2019/05/23	CH2205	Male	Three y/o	Treatment (Wk 2)	Treatment	Feed Day	No	01:05	01:00 - 01:59	Early Morning	37,2	63	26,8	26,8	18,36	558,62	110,33
2019/05/23	CH2206	Male	Three y/o	Treatment (Wk 2)	Treatment	Feed Day	No	01:05	01:00 - 01:59	Early Morning	37,1	73	7,4	7,4	18,26	570,27	66,59
2019/05/23	CH2205	Male	Three y/o	Treatment (Wk 2)	Treatment	Feed Day	No	01:10	01:00 - 01:59	Early Morning	37,2	58	27,6	27,6	18,36	551,90	111,33
2019/05/23	CH2206	Male	Three y/o	Treatment (Wk 2)	Treatment	Feed Day	No	01:10	01:00 - 01:59	Early Morning	37,2	42	15,4	15,4	18,36	524,42	91,41
2019/05/23	CH2205	Male	Three y/o	Treatment (Wk 2)	Treatment	Feed Day	No	01:15	01:00 - 01:59	Early Morning	37,2	55	25,8	25,8	18,36	547,52	109,02
2019/05/23	CH2206	Male	Three y/o	Treatment (Wk 2)	Treatment	Feed Day	No	01:15	01:00 - 01:59	Early Morning	37,2	46	16,2	16,2	18,36	532,38	93,13
2019/05/23	CH2205	Male	Three y/o	Treatment (Wk 2)	Treatment	Feed Day	No	01:20	01:00 - 01:59	Early Morning	37,3	57	28,0	28,0	18,46	550,47	111,83
2019/05/23	CH2206	Male	Three y/o	Treatment (Wk 2)	Treatment	Feed Day	No	01:20	01:00 - 01:59	Early Morning	37,2	50	12,4	12,4	18,36	539,52	84,04
2019/05/23	CH2205	Male	Three y/o	Treatment (Wk 2)	Treatment	Feed Day	No	01:25	01:00 - 01:59	Early Morning	37,3	54	28,0	28,0	18,46	545,99	111,83
2019/05/23	CH2206	Male	Three y/o	Treatment (Wk 2)	Treatment	Feed Day	No	01:25	01:00 - 01:59	Early Morning	37,2	90	6,2	6,2	18,36	586,16	60,63
2019/05/23	CH2205	Male	Three y/o	Treatment (Wk 2)	Treatment	Feed Day	No	01:30	01:00 - 01:59	Early Morning	37,3		27,6	27,6	18,46		111,33
2019/05/23	CH2206	Male	Three y/o	Treatment (Wk 2)	Treatment	Feed Day	No	01:30	01:00 - 01:59	Early Morning	37,2	45	6,2	6,2	18,36	530,47	60,63
2019/05/23	CH2205	Male	Three y/o	Treatment (Wk 2)	Treatment	Feed Day	No	01:35	01:00 - 01:59	Early Morning	37,4		21,8	21,8	18,55		103,26
2019/05/23	CH2206	Male	Three y/o	Treatment (Wk 2)	Treatment	Feed Day	No	01:35	01:00 - 01:59	Early Morning	37,2	78	11,6	11,6	18,36	575,38	81,78
2019/05/23	CH2205	Male	Three y/o	Treatment (Wk 2)	Treatment	Feed Day	No	01:40	01:00 - 01:59	Early Morning	37,4	39	15,4	15,4	18,55	517,82	91,41
2019/05/23	CH2206	Male	Three y/o	Treatment (Wk 2)	Treatment	Feed Day	No	01:40	01:00 - 01:59	Early Morning	37,2	86	25,6	25,6	18,36	582,77	108,76
2019/05/23	CH2205	Male	Three y/o	Treatment (Wk 2)	Treatment	Feed Day	No	01:45	01:00 - 01:59	Early Morning	37,4	44	7,8	7,8	18,55	528,51	68,36
2019/05/23	CH2206	Male	Three y/o	Treatment (Wk 2)	Treatment	Feed Day	No	01:45	01:00 - 01:59	Early Morning	37,2	84	30,2	30,2	18,36	581,01	114,42
2019/05/23	CH2205	Male	Three y/o	Treatment (Wk 2)	Treatment	Feed Day	No	01:50	01:00 - 01:59	Early Morning	37,3	67	10,6	10,6	18,46	563,53	78,73
2019/05/23	CH2206	Male	Three y/o	Treatment (Wk 2)	Treatment	Feed Day	No	01:50	01:00 - 01:59	Early Morning	37,2	42	30,6	30,6	18,36	524,42	114,87
2019/05/23	CH2205	Male	Three y/o	Treatment (Wk 2)	Treatment	Feed Day	No	01:55	01:00 - 01:59	Early Morning	37,3	40	8,0	8,0	18,46	520,09	69,22
2019/05/23	CH2206	Male	Three y/o	Treatment (Wk 2)	Treatment	Feed Day	No	01:55	01:00 - 01:59	Early Morning	37,2	42	36,0	36,0	18,36	524,42	120,46
2019/05/23	CH2205	Male	Three y/o	Treatment (Wk 2)	Treatment	Feed Day	No	02:00	02:00 - 02:59	Early Morning	37,3		8,8	8,8	18,46		72,43
2019/05/23	CH2206	Male	Three y/o	Treatment (Wk 2)	Treatment	Feed Day	No	02:00	02:00 - 02:59	Early Morning	37,2	46	40,0	40,0	18,36	532,38	124,09
2019/05/23	CH2205	Male	Three y/o	Treatment (Wk 2)	Treatment	Feed Day	No	02:05	02:00 - 02:59	Early Morning	37,3		5,6	5,6	18,46		57,21
2019/05/23	CH2206	Male	Three y/o	Treatment (Wk 2)	Treatment	Feed Day	No	02:05	02:00 - 02:59	Early Morning	37,2	49	39,8	39,8	18,36	537,80	123,91
2019/05/23	CH2205	Male	Three y/o	Treatment (Wk 2)	Treatment	Feed Day	No	02:10	02:00 - 02:59	Early Morning	37,3		7,2	7,2	18,46		65,66
2019/05/23	CH2206	Male	Three y/o	Treatment (Wk 2)	Treatment	Feed Day	No	02:10	02:00 - 02:59	Early Morning	37,2	47	34,8	34,8	18,36	534,23	119,29
2019/05/23	CH2205	Male	Three y/o	Treatment (Wk 2)	Treatment	Feed Day	No	02:15	02:00 - 02:59	Early Morning	37,3	83	4,8	4,8	18,46	580,11	52,04
2019/05/23	CH2206	Male	Three y/o	Treatment (Wk 2)	Treatment	Feed Day	No	02:15	02:00 - 02:59	Early Morning	37,2	128	59,4	59,4	18,36	611,22	137,75
2019/05/23	CH2205	Male	Three y/o	Treatment (Wk 2)	Treatment	Feed Day	No	02:20	02:00 - 02:59	Early Morning	37,3	68	20,2	20,2	18,46	564,71	100,65
2019/05/23	CH2206	Male	Three y/o	Treatment (Wk 2)	Treatment	Feed Day	No	02:20	02:00 - 02:59	Early Morning	37,1	88	61,6	61,6	18,26	584,49	139,00
2019/05/23	CH2205	Male	Three y/o	Treatment (Wk 2)	Treatment	Feed Day	No	02:25	02:00 - 02:59	Early Morning	37,4	147	39,0	39,0	18,55	620,52	123,22
2019/05/23	CH2206	Male	Three y/o	Treatment (Wk 2)	Treatment	Feed Day	No	02:25	02:00 - 02:59	Early Morning	37,2	108	97,4	97,4	18,36	599,39	154,93
2019/05/23	CH2205	Male	Three y/o	Treatment (Wk 2)	Treatment	Feed Day	No	02:30	02:00 - 02:59	Early Morning	37,4		26,4	26,4	18,55		109,81
2019/05/23	CH2206	Male	Three y/o	Treatment (Wk 2)	Treatment	Feed Day	No	02:30	02:00 - 02:59	Early Morning	37,2		123,6	123,6	18,36		163,24
2019/05/23	CH2205	Male	Three y/o	Treatment (Wk 2)	Treatment	Feed Day	No	02:35	02:00 - 02:59	Early Morning	37,3	123	73,0	73,0	18,46	608,49	144,89
2019/05/23	CH2206	Male	Three y/o	Treatment (Wk 2)	Treatment	Feed Day	No	02:35	02:00 - 02:59	Early Morning	37,2	114	253,2	253,2	18,36	603,20	188,43
2019/05/23	CH2205	Male	Three y/o	Treatment (Wk 2)	Treatment	Feed Day	No	02:40	02:00 - 02:59	Early Morning	37,4	51	95,8	95,8	18,55	541,20	154,35
2019/05/23	CH2206	Male	Three y/o	Treatment (Wk 2)	Treatment	Feed Day	No	02:40	02:00 - 02:59	Early Morning	37,3	121	78,6	78,6	18,46	607,35	147,46
2019/05/23	CH2205	Male	Three y/o	Treatment (Wk 2)	Treatment	Feed Day	No	02:45	02:00 - 02:59	Early Morning	37,4	52	15,0	15,0	18,55	542,83	90,51
2019/05/23	CH2206	Male	Three y/o	Treatment (Wk 2)	Treatment	Feed Day	No	02:45	02:00 - 02:59	Early Morning	37,3	112	115,4	115,4	18,46	601,96	160,84
2019/05/23	CH2205	Male	Three y/o	Treatment (Wk 2)	Treatment	Feed Day	No	02:50	02:00 - 02:59	Early Morning	37,3	100	48,8	48,8	18,46	593,87	130,95
2019/05/23	CH2206	Male	Three y/o	Treatment (Wk 2)	Treatment	Feed Day	No	02:50	02:00 - 02:59	Early Morning	37,4	101	209,0	209,0	18,55	594,59	181,67
2019/05/23	CH2205	Male	Three y/o	Treatment (Wk 2)	Treatment	Feed Day	No	02:55	02:00 - 02:59	Early Morning	37,4	118	109,8	109,8	18,55	605,61	159,11
2019/05/23	CH2206	Male	Three y/o	Treatment (Wk 2)	Treatment	Feed Day	No	02:55	02:00 - 02:59	Early Morning	37,5	126	145,6	145,6	18,65	610,14	168,97
2019/05/23	CH2205	Male	Three y/o	Treatment (Wk 2)	Treatment	Feed Day	No	03:00	03:00 - 03:59	Early Morning	37,4	114	67,0	67,0	18,55	603,20	141,92
2019/05/23	CH2206	Male	Three y/o	Treatment (Wk 2)	Treatment	Feed Day	No	03:00	03:00 - 03:59	Early Morning	37,8	124	26,6	26,6	18,95	609,04	110,07
2019/05/23	CH2205	Male	Three y/o	Treatment (Wk 2)	Treatment	Feed Day	No	03:05	03:00 - 03:59	Early Morning	37,5	61	97,8	97,8	18,65	556,01	155,07
2019/05/23	CH2206	Male	Three y/o	Treatment (Wk 2)	Treatment	Feed Day	No	03:05	03:00 - 03:59	Early Morning	37,8	94	127,4	127,4	18,95	589,37	164,30
2019/05/23	CH2205	Male	Three y/o	Treatment (Wk 2)	Treatment	Feed Day	No	03:10	03:00 - 03:59	Early Morning	37,5	63	18,4	18,4	18,65	558,62	97,47
2019/05/23	CH2206	Male	Three y/o	Treatment (Wk 2)	Treatment	Feed Day	No	03:10	03:00 - 03:59	Early Morning	37,8	128	85,8	85,8	18,95	611,22	150,51
2019/05/23	CH2205	Male	Three y/o	Treatment (Wk 2)	Treatment	Feed Day	No	03:15	03:00 - 03:59	Early Morning	37,5	86	148,8	148,8	18,65	582,77	169,74

2019/05/23	CH2206	Male	Three y/o	Treatment (Wk 2)	Treatment	Feed Day	No	03:15	03:00 - 03:59	Early Morning	37,4	108	171,8	171,8	18,55	599,39	174,78
2019/05/23	CH2205	Male	Three y/o	Treatment (Wk 2)	Treatment	Feed Day	No	03:20	03:00 - 03:59	Early Morning	37,5	108	1170,8		18,65	599,39	
2019/05/23	CH2206	Male	Three y/o	Treatment (Wk 2)	Treatment	Feed Day	No	03:20	03:00 - 03:59	Early Morning	37,4		108,6	108,6	18,55		158,72
2019/05/23	CH2205	Male	Three y/o	Treatment (Wk 2)	Treatment	Feed Day	No	03:25	03:00 - 03:59	Early Morning	37,7	55	324,4	324,4	18,85	547,52	197,19
2019/05/23	CH2206	Male	Three y/o	Treatment (Wk 2)	Treatment	Feed Day	No	03:25	03:00 - 03:59	Early Morning	37,5	105	199,0	199,0	18,65	597,38	179,94
2019/05/23	CH2205	Male	Three y/o	Treatment (Wk 2)	Treatment	Feed Day	No	03:30	03:00 - 03:59	Early Morning	37,7	83	144,2	144,2	18,85	580,11	168,64
2019/05/23	CH2206	Male	Three y/o	Treatment (Wk 2)	Treatment	Feed Day	No	03:30	03:00 - 03:59	Early Morning	37,7	128	113,2	113,2	18,85	611,22	160,17
2019/05/23	CH2205	Male	Three y/o	Treatment (Wk 2)	Treatment	Feed Day	No	03:35	03:00 - 03:59	Early Morning	37,4	124	51,2	51,2	18,55	609,04	132,61
2019/05/23	CH2206	Male	Three y/o	Treatment (Wk 2)	Treatment	Feed Day	No	03:35	03:00 - 03:59	Early Morning	37,5	145	113,6	113,6	18,65	619,62	160,29
2019/05/23	CH2205	Male	Three y/o	Treatment (Wk 2)	Treatment	Feed Day	No	03:40	03:00 - 03:59	Early Morning	37,5	94	256,8	256,8	18,65	589,37	188,93
2019/05/23	CH2206	Male	Three y/o	Treatment (Wk 2)	Treatment	Feed Day	No	03:40	03:00 - 03:59	Early Morning	37,8	98	297,6	297,6	18,95	592,41	194,14
2019/05/23	CH2205	Male	Three y/o	Treatment (Wk 2)	Treatment	Feed Day	No	03:45	03:00 - 03:59	Early Morning	37,5		94,2	94,2	18,65		153,76
2019/05/23	CH2206	Male	Three y/o	Treatment (Wk 2)	Treatment	Feed Day	No	03:45	03:00 - 03:59	Early Morning	37,8	106	31,8	31,8	18,95	598,06	116,20
2019/05/23	CH2205	Male	Three y/o	Treatment (Wk 2)	Treatment	Feed Day	No	03:50	03:00 - 03:59	Early Morning	37,4	44	33,4	33,4	18,55	528,51	117,88
2019/05/23	CH2206	Male	Three y/o	Treatment (Wk 2)	Treatment	Feed Day	No	03:50	03:00 - 03:59	Early Morning	37,5	52	53,0	53,0	18,65	542,83	133,80
2019/05/23	CH2205	Male	Three y/o	Treatment (Wk 2)	Treatment	Feed Day	No	03:55	03:00 - 03:59	Early Morning	37,3	64	35,2	35,2	18,46	559,88	119,69
2019/05/23	CH2206	Male	Three y/o	Treatment (Wk 2)	Treatment	Feed Day	No	03:55	03:00 - 03:59	Early Morning	37,1	71	55,2	55,2	18,26	568,10	135,21
2019/05/23	CH2205	Male	Three y/o	Treatment (Wk 2)	Treatment	Feed Day	No	04:00	04:00 - 04:59	Morning	37,4		26,8	26,8	18,55		110,33
2019/05/23	CH2206	Male	Three y/o	Treatment (Wk 2)	Treatment	Feed Day	No	04:00	04:00 - 04:59	Morning	37,0	56	40,4	40,4	18,16	549,01	124,43
2019/05/23	CH2205	Male	Three y/o	Treatment (Wk 2)	Treatment	Feed Day	No	04:05	04:00 - 04:59	Morning	37,4	70	23,4	23,4	18,55	566,99	105,68
2019/05/23	CH2206	Male	Three y/o	Treatment (Wk 2)	Treatment	Feed Day	No	04:05	04:00 - 04:59	Morning	37,1	50	43,2	43,2	18,26	539,52	126,74
2019/05/23	CH2205	Male	Three y/o	Treatment (Wk 2)	Treatment	Feed Day	No	04:10	04:00 - 04:59	Morning	37,5	66	27,2	27,2	18,65	562,34	110,83
2019/05/23	CH2206	Male	Three y/o	Treatment (Wk 2)	Treatment	Feed Day	No	04:10	04:00 - 04:59	Morning	37,1	46	43,2	43,2	18,26	532,38	126,74
2019/05/23	CH2205	Male	Three y/o	Treatment (Wk 2)	Treatment	Feed Day	No	04:15	04:00 - 04:59	Morning	37,5	43	19,2	19,2	18,65	526,50	98,92
2019/05/23	CH2206	Male	Three y/o	Treatment (Wk 2)	Treatment	Feed Day	No	04:15	04:00 - 04:59	Morning	37,2	70	45,8	45,8	18,36	566,99	128,76
2019/05/23	CH2205	Male	Three y/o	Treatment (Wk 2)	Treatment	Feed Day	No	04:20	04:00 - 04:59	Morning	37,5	49	20,0	20,0	18,65	537,80	100,31
2019/05/23	CH2206	Male	Three y/o	Treatment (Wk 2)	Treatment	Feed Day	No	04:20	04:00 - 04:59	Morning	37,2	44	41,0	41,0	18,36	528,51	124,94
2019/05/23	CH2205	Male	Three y/o	Treatment (Wk 2)	Treatment	Feed Day	No	04:25	04:00 - 04:59	Morning	37,4	91	20,8	20,8	18,55	586,98	101,65
2019/05/23	CH2206	Male	Three y/o	Treatment (Wk 2)	Treatment	Feed Day	No	04:25	04:00 - 04:59	Morning	37,2	48	38,8	38,8	18,36	536,04	123,04
2019/05/23	CH2205	Male	Three y/o	Treatment (Wk 2)	Treatment	Feed Day	No	04:30	04:00 - 04:59	Morning	37,4	48	23,6	23,6	18,55	536,04	105,97
2019/05/23	CH2206	Male	Three y/o	Treatment (Wk 2)	Treatment	Feed Day	No	04:30	04:00 - 04:59	Morning	37,2	60	39,8	39,8	18,36	554,67	123,91
2019/05/23	CH2205	Male	Three y/o	Treatment (Wk 2)	Treatment	Feed Day	No	04:35	04:00 - 04:59	Morning	37,4		12,8	12,8	18,55		85,12
2019/05/23	CH2206	Male	Three y/o	Treatment (Wk 2)	Treatment	Feed Day	No	04:35	04:00 - 04:59	Morning	37,1	82	17,2	17,2	18,26	579,19	95,17
2019/05/23	CH2205	Male	Three y/o	Treatment (Wk 2)	Treatment	Feed Day	No	04:40	04:00 - 04:59	Morning	37,4	47	9,0	9,0	18,55	534,23	73,19
2019/05/23	CH2206	Male	Three y/o	Treatment (Wk 2)	Treatment	Feed Day	No	04:40	04:00 - 04:59	Morning	37,0	79	13,6	13,6	18,16	576,36	87,18
2019/05/23	CH2205	Male	Three y/o	Treatment (Wk 2)	Treatment	Feed Day	No	04:45	04:00 - 04:59	Morning	37,4	68	8,0	8,0	18,55	564,71	69,22
2019/05/23	CH2206	Male	Three y/o	Treatment (Wk 2)	Treatment	Feed Day	No	04:45	04:00 - 04:59	Morning	37,1	62	16,2	16,2	18,26	557,33	93,13
2019/05/23	CH2205	Male	Three y/o	Treatment (Wk 2)	Treatment	Feed Day	No	04:50	04:00 - 04:59	Morning	37,3	62	4,8	4,8	18,46	557,33	52,04
2019/05/23	CH2206	Male	Three y/o	Treatment (Wk 2)	Treatment	Feed Day	No	04:50	04:00 - 04:59	Morning	37,1	80	17,4	17,4	18,26	577,31	95,56
2019/05/23	CH2205	Male	Three y/o	Treatment (Wk 2)	Treatment	Feed Day	No	04:55	04:00 - 04:59	Morning	37,2	61	10,0	10,0	18,36	556,01	76,76
2019/05/23	CH2206	Male	Three y/o	Treatment (Wk 2)	Treatment	Feed Day	No	04:55	04:00 - 04:59	Morning	37,1	82	18,0	18,0	18,26	579,19	96,72
2019/05/23	CH2205	Male	Three y/o	Treatment (Wk 2)	Treatment	Feed Day	No	05:00	05:00 - 05:59	Morning	37,1	51	10,0	10,0	18,26	541,20	76,76
2019/05/23	CH2206	Male	Three y/o	Treatment (Wk 2)	Treatment	Feed Day	No	05:00	05:00 - 05:59	Morning	37,1	65	33,0	33,0	18,26	561,12	117,47
2019/05/23	CH2205	Male	Three y/o	Treatment (Wk 2)	Treatment	Feed Day	No	05:05	05:00 - 05:59	Morning	37,0	52	5,8	5,8	18,16	542,83	58,39
2019/05/23	CH2206	Male	Three y/o	Treatment (Wk 2)	Treatment	Feed Day	No	05:05	05:00 - 05:59	Morning	37,1	42	5,8	5,8	18,26	524,42	58,39
2019/05/23	CH2205	Male	Three y/o	Treatment (Wk 2)	Treatment	Feed Day	No	05:10	05:00 - 05:59	Morning	37,1	41	5,4	5,4	18,26	522,29	55,99
2019/05/23	CH2206	Male	Three y/o	Treatment (Wk 2)	Treatment	Feed Day	No	05:10	05:00 - 05:59	Morning	37,1	45	21,8	21,8	18,26	530,47	103,26
2019/05/23	CH2205	Male	Three y/o	Treatment (Wk 2)	Treatment	Feed Day	No	05:15	05:00 - 05:59	Morning	37,0	80	6,4	6,4	18,16	577,31	61,70
2019/05/23	CH2206	Male	Three y/o	Treatment (Wk 2)	Treatment	Feed Day	No	05:15	05:00 - 05:59	Morning	37,1	43	24,8	24,8	18,26	526,50	107,67
2019/05/23	CH2205	Male	Three y/o	Treatment (Wk 2)	Treatment	Feed Day	No	05:20	05:00 - 05:59	Morning	36,8	50	5,4	5,4	17,96	539,52	55,99
2019/05/23	CH2206	Male	Three y/o	Treatment (Wk 2)	Treatment	Feed Day	No	05:20	05:00 - 05:59	Morning	37,1	51	28,2	28,2	18,26	541,20	112,07
2019/05/23	CH2205	Male	Three y/o	Treatment (Wk 2)	Treatment	Feed Day	No	05:25	05:00 - 05:59	Morning	36,7	63	9,2	9,2	17,87	558,62	73,94
2019/05/23	CH2206	Male	Three y/o	Treatment (Wk 2)	Treatment	Feed Day	No	05:25	05:00 - 05:59	Morning	37,1	40	35,6	35,6	18,26	520,09	120,08
2019/05/23	CH2205	Male	Three y/o	Treatment (Wk 2)	Treatment	Feed Day	No	05:30	05:00 - 05:59	Morning	36,8	56	27,6	27,6	17,96	549,01	111,33
2019/05/23	CH2206	Male	Three y/o	Treatment (Wk 2)	Treatment	Feed Day	No	05:30	05:00 - 05:59	Morning	37,1	54	62,8	62,8	18,26	545,99	139,67
2019/05/23	CH2205	Male	Three y/o	Treatment (Wk 2)	Treatment	Feed Day	No	05:35	05:00 - 05:59	Morning	37,1	84	24,8	24,8	18,26	581,01	107,67
2019/05/23	CH2206	Male	Three y/o	Treatment (Wk 2)	Treatment	Feed Day	No	05:35	05:00 - 05:59	Morning	37,0	42	62,0	62,0	18,16	524,42	139,23
2019/05/23	CH2205	Male	Three y/o	Treatment (Wk 2)	Treatment	Feed Day	No	05:40	05:00 - 05:59	Morning	37,1	72	26,2	26,2	18,26	569,20	109,55
2019/05/23	CH2206	Male	Three y/o	Treatment (Wk 2)	Treatment	Feed Day	No	05:40	05:00 - 05:59	Morning	37,0	45	58,2	58,2	18,16	530,47	137,04
2019/05/23	CH2205	Male	Three y/o	Treatment (Wk 2)	Treatment	Feed Day	No	05:45	05:00 - 05:59	Morning	37,1	84	23,6	23,6	18,26	581,01	105,97
2019/05/23	CH2206	Male	Three y/o	Treatment (Wk 2)	Treatment	Feed Day	No	05:45	05:00 - 05:59	Morning	37,1	60	63,2	63,2	18,26	554,67	139,89
2019/05/23	CH2205	Male	Three y/o	Treatment (Wk 2)	Treatment	Feed Day	No	05:50	05:00 - 05:59	Morning	37,1	78	24,0	24,0	18,26	575,38	106,55
2019/05/23	CH2206	Male	Three y/o	Treatment (Wk 2)	Treatment	Feed Day	No	05:50	05:00 - 05:59	Morning	37,1	41	62,8	62,8	18,26	522,29	139,67
2019/05/23	CH2205	Male	Three y/o	Treatment (Wk 2)	Treatment	Feed Day	No	05:55	05:00 - 05:59	Morning	37,1	72	21,8	21,8	18,26	569,20	103,26
2019/05/23	CH2206	Male	Three y/o	Treatment (Wk 2)	Treatment	Feed Day	No	05:55	05:00 - 05:59	Morning	37,1	67	63,8	63,8	18,26	563,53	140,22
2019/05/23	CH2205	Male	Three y/o	Treatment (Wk 2)	Treatment	Feed Day	No	06:00	06:00 - 06:59	Morning	37,1		23,0	23,0	18,26		105,09
2019/05/23	CH2206	Male	Three y/o	Treatment (Wk 2)	Treatment	Feed Day	No	06:00	06:00 - 06:59	Morning	37,1	73	64,0	64,0	18,26	570,27	140,33
2019/05/23	CH2205	Male	Three y/o	Treatment (Wk 2)	Treatment	Feed Day	No	06:05	06:00 - 06:59	Morning	37,1	82	28,0	28,0	18,26	579,19	111,83
2019/05/23	CH2206	Male	Three y/o	Treatment (Wk 2)	Treatment	Feed Day	No	06:05	06:00 - 06:59	Morning	37,2	54	66,4	66,4	18,36	545,99	141,61
2019/05/23	CH2205	Male	Three y/o	Treatment (Wk 2)	Treatment	Feed Day	No	06:10	06:00 - 06:59	Morning	37,1		25,2	25,2	18,26		108,22

2019/05/23	CH2206	Male	Three y/o	Treatment (Wk 2)	Treatment	Feed Day	No	06:10	06:00 - 06:59	Morning	37,2	85	57,2	57,2	18,36	581,90	136,44
2019/05/23	CH2205	Male	Three y/o	Treatment (Wk 2)	Treatment	Feed Day	No	06:15	06:00 - 06:59	Morning	37,2		32,2	32,2	18,36		116,63
2019/05/23	CH2206	Male	Three y/o	Treatment (Wk 2)	Treatment	Feed Day	No	06:15	06:00 - 06:59	Morning	37,3		112,0	112,0	18,46		159,80
2019/05/23	CH2205	Male	Three y/o	Treatment (Wk 2)	Treatment	Feed Day	No	06:20	06:00 - 06:59	Morning	37,2	132	38,6	38,6	18,36	613,32	122,86
2019/05/23	CH2206	Male	Three y/o	Treatment (Wk 2)	Treatment	Feed Day	No	06:20	06:00 - 06:59	Morning	37,3	115	106,2	106,2	18,46	603,82	157,94
2019/05/23	CH2205	Male	Three y/o	Treatment (Wk 2)	Treatment	Feed Day	No	06:25	06:00 - 06:59	Morning	37,2	91	135,6	135,6	18,36	586,98	166,48
2019/05/23	CH2206	Male	Three y/o	Treatment (Wk 2)	Treatment	Feed Day	No	06:25	06:00 - 06:59	Morning	37,4	128	80,0	80,0	18,55	611,22	148,08
2019/05/23	CH2205	Male	Three y/o	Treatment (Wk 2)	Treatment	Feed Day	No	06:30	06:00 - 06:59	Morning	37,3	124	53,0	53,0	18,46	609,04	133,80
2019/05/23	CH2206	Male	Three y/o	Treatment (Wk 2)	Treatment	Feed Day	No	06:30	06:00 - 06:59	Morning	37,5		226,0	226,0	18,65		184,42
2019/05/23	CH2205	Male	Three y/o	Treatment (Wk 2)	Treatment	Feed Day	No	06:35	06:00 - 06:59	Morning	37,3	103	59,2	59,2	18,46	596,00	137,63
2019/05/23	CH2206	Male	Three y/o	Treatment (Wk 2)	Treatment	Feed Day	No	06:35	06:00 - 06:59	Morning	37,5	119	80,2	80,2	18,65	606,20	148,16
2019/05/23	CH2205	Male	Three y/o	Treatment (Wk 2)	Treatment	Feed Day	No	06:40	06:00 - 06:59	Morning	37,4		67,6	67,6	18,55		142,23
2019/05/23	CH2206	Male	Three y/o	Treatment (Wk 2)	Treatment	Feed Day	No	06:40	06:00 - 06:59	Morning	37,7	99	79,8	79,8	18,85	593,14	147,99
2019/05/23	CH2205	Male	Three y/o	Treatment (Wk 2)	Treatment	Feed Day	No	06:45	06:00 - 06:59	Morning	37,4	111	93,4	93,4	18,55	601,33	153,46
2019/05/23	CH2206	Male	Three y/o	Treatment (Wk 2)	Treatment	Feed Day	No	06:45	06:00 - 06:59	Morning	37,5	50	70,4	70,4	18,65	539,52	143,64
2019/05/23	CH2205	Male	Three y/o	Treatment (Wk 2)	Treatment	Feed Day	No	06:50	06:00 - 06:59	Morning	37,4	50	38,4	38,4	18,55	539,52	122,68
2019/05/23	CH2206	Male	Three y/o	Treatment (Wk 2)	Treatment	Feed Day	No	06:50	06:00 - 06:59	Morning	37,5	128	123,6	123,6	18,65	611,22	163,24
2019/05/23	CH2205	Male	Three y/o	Treatment (Wk 2)	Treatment	Feed Day	No	06:55	06:00 - 06:59	Morning	37,5	40	253,8	253,8	18,65	520,09	188,51
2019/05/23	CH2206	Male	Three y/o	Treatment (Wk 2)	Treatment	Feed Day	No	06:55	06:00 - 06:59	Morning	37,7	138	142,0	142,0	18,85	616,31	168,10
2019/05/23	CH2205	Male	Three y/o	Treatment (Wk 2)	Treatment	Feed Day	No	07:00	07:00 - 07:59	Morning	37,5	124	47,2	47,2	18,65	609,04	129,80
2019/05/23	CH2206	Male	Three y/o	Treatment (Wk 2)	Treatment	Feed Day	No	07:00	07:00 - 07:59	Morning	37,8	140	79,6	79,6	18,95	617,28	147,90
2019/05/23	CH2205	Male	Three y/o	Treatment (Wk 2)	Treatment	Feed Day	No	07:05	07:00 - 07:59	Morning	37,6		304,8	304,8	18,75		194,98
2019/05/23	CH2206	Male	Three y/o	Treatment (Wk 2)	Treatment	Feed Day	No	07:05	07:00 - 07:59	Morning	37,8	118	196,0	196,0	18,95	605,61	179,41
2019/05/23	CH2205	Male	Three y/o	Treatment (Wk 2)	Treatment	Feed Day	No	07:10	07:00 - 07:59	Morning	37,6	119	84,0	84,0	18,75	606,20	149,77
2019/05/23	CH2206	Male	Three y/o	Treatment (Wk 2)	Treatment	Feed Day	No	07:10	07:00 - 07:59	Morning	37,8	134	151,2	151,2	18,95	614,33	170,30
2019/05/23	CH2205	Male	Three y/o	Treatment (Wk 2)	Treatment	Feed Day	No	07:15	07:00 - 07:59	Morning	37,6	98	55,0	55,0	18,75	592,41	135,08
2019/05/23	CH2206	Male	Three y/o	Treatment (Wk 2)	Treatment	Feed Day	No	07:15	07:00 - 07:59	Morning	37,8	119	223,8	223,8	18,95	606,20	184,08
2019/05/23	CH2205	Male	Three y/o	Treatment (Wk 2)	Treatment	Feed Day	No	07:20	07:00 - 07:59	Morning	37,6	99	117,8	117,8	18,75	593,14	161,56
2019/05/23	CH2206	Male	Three y/o	Treatment (Wk 2)	Treatment	Feed Day	No	07:20	07:00 - 07:59	Morning	37,8	124	100,6	100,6	18,95	609,04	156,05
2019/05/23	CH2205	Male	Three y/o	Treatment (Wk 2)	Treatment	Feed Day	No	07:25	07:00 - 07:59	Morning	37,7	99	120,2	120,2	18,85	593,14	162,27
2019/05/23	CH2206	Male	Three y/o	Treatment (Wk 2)	Treatment	Feed Day	No	07:25	07:00 - 07:59	Morning	37,8	135	140,0	140,0	18,95	614,84	167,60
2019/05/23	CH2205	Male	Three y/o	Treatment (Wk 2)	Treatment	Feed Day	No	07:30	07:00 - 07:59	Morning	37,7	103	130,0	130,0	18,85	596,00	165,01
2019/05/23	CH2206	Male	Three y/o	Treatment (Wk 2)	Treatment	Feed Day	No	07:30	07:00 - 07:59	Morning	37,8	118	75,2	75,2	18,95	605,61	145,93
2019/05/23	CH2205	Male	Three y/o	Treatment (Wk 2)	Treatment	Feed Day	No	07:35	07:00 - 07:59	Morning	37,7	106	96,0	96,0	18,85	598,06	154,42
2019/05/23	CH2206	Male	Three y/o	Treatment (Wk 2)	Treatment	Feed Day	No	07:35	07:00 - 07:59	Morning	37,9		80,0	80,0	19,05		148,08
2019/05/23	CH2205	Male	Three y/o	Treatment (Wk 2)	Treatment	Feed Day	No	07:40	07:00 - 07:59	Morning	37,9	79	54,0	54,0	19,05	576,36	134,45
2019/05/23	CH2206	Male	Three y/o	Treatment (Wk 2)	Treatment	Feed Day	No	07:40	07:00 - 07:59	Morning	38,1		336,2	336,2	19,26		198,45
2019/05/23	CH2205	Male	Three y/o	Treatment (Wk 2)	Treatment	Feed Day	No	07:45	07:00 - 07:59	Morning	37,9		52,8	52,8	19,05		133,67
2019/05/23	CH2206	Male	Three y/o	Treatment (Wk 2)	Treatment	Feed Day	No	07:45	07:00 - 07:59	Morning	38,0	126	141,4	141,4	19,15	610,14	167,95
2019/05/23	CH2205	Male	Three y/o	Treatment (Wk 2)	Treatment	Feed Day	No	07:50	07:00 - 07:59	Morning	37,7	108	153,8	153,8	18,85	599,39	170,89
2019/05/23	CH2206	Male	Three y/o	Treatment (Wk 2)	Treatment	Feed Day	No	07:50	07:00 - 07:59	Morning	37,9	131	250,8	250,8	19,05	612,80	188,09
2019/05/23	CH2205	Male	Three y/o	Treatment (Wk 2)	Treatment	Feed Day	No	07:55	07:00 - 07:59	Morning	37,6	110	57,0	57,0	18,75	600,69	136,32
2019/05/23	CH2206	Male	Three y/o	Treatment (Wk 2)	Treatment	Feed Day	No	07:55	07:00 - 07:59	Morning	37,9		281,8	281,8	19,05		192,21
2019/05/23	CH2205	Male	Three y/o	Treatment (Wk 2)	Treatment	Feed Day	No	08:00	08:00 - 08:59	Late Morning	37,6		114,2	114,2	18,75		160,48
2019/05/23	CH2206	Male	Three y/o	Treatment (Wk 2)	Treatment	Feed Day	No	08:00	08:00 - 08:59	Late Morning	38,0	111	104,2	104,2	19,15	601,33	157,28
2019/05/23	CH2205	Male	Three y/o	Treatment (Wk 2)	Treatment	Feed Day	No	08:05	08:00 - 08:59	Late Morning	37,6	56	167,8	167,8	18,75	549,01	173,95
2019/05/23	CH2206	Male	Three y/o	Treatment (Wk 2)	Treatment	Feed Day	No	08:05	08:00 - 08:59	Late Morning	37,9	114	110,2	110,2	19,05	603,20	159,23
2019/05/23	CH2205	Male	Three y/o	Treatment (Wk 2)	Treatment	Feed Day	No	08:10	08:00 - 08:59	Late Morning	37,6	53	93,8	93,8	18,75	544,43	153,61
2019/05/23	CH2206	Male	Three y/o	Treatment (Wk 2)	Treatment	Feed Day	No	08:10	08:00 - 08:59	Late Morning	37,9	58	61,6	61,6	19,05	551,90	139,00
2019/05/23	CH2205	Male	Three y/o	Treatment (Wk 2)	Treatment	Feed Day	No	08:15	08:00 - 08:59	Late Morning	37,6	100	20,8	20,8	18,75	593,87	101,65
2019/05/23	CH2206	Male	Three y/o	Treatment (Wk 2)	Treatment	Feed Day	No	08:15	08:00 - 08:59	Late Morning	37,9	132	112,4	112,4	19,05	613,32	159,92
2019/05/23	CH2205	Male	Three y/o	Treatment (Wk 2)	Treatment	Feed Day	No	08:20	08:00 - 08:59	Late Morning	37,4		5,0	5,0	18,55		53,41
2019/05/23	CH2206	Male	Three y/o	Treatment (Wk 2)	Treatment	Feed Day	No	08:20	08:00 - 08:59	Late Morning	37,8	182	252,8	252,8	18,95	634,31	188,37
2019/05/23	CH2205	Male	Three y/o	Treatment (Wk 2)	Treatment	Feed Day	No	08:25	08:00 - 08:59	Late Morning	37,4	92	123,0	123,0	18,55	587,78	163,07
2019/05/23	CH2206	Male	Three y/o	Treatment (Wk 2)	Treatment	Feed Day	No	08:25	08:00 - 08:59	Late Morning	37,8	119	123,0	123,0	18,95	606,20	163,07
2019/05/23	CH2205	Male	Three y/o	Treatment (Wk 2)	Treatment	Feed Day	No	08:30	08:00 - 08:59	Late Morning	37,4	147	130,2	130,2	18,55	620,52	165,06
2019/05/23	CH2206	Male	Three y/o	Treatment (Wk 2)	Treatment	Feed Day	No	08:30	08:00 - 08:59	Late Morning	37,8	125	112,6	112,6	18,95	609,60	159,98
2019/05/23	CH2205	Male	Three y/o	Treatment (Wk 2)	Treatment	Feed Day	No	08:35	08:00 - 08:59	Late Morning	37,4	54	103,4	103,4	18,55	545,99	157,01
2019/05/23	CH2206	Male	Three y/o	Treatment (Wk 2)	Treatment	Feed Day	No	08:35	08:00 - 08:59	Late Morning	37,8	137	190,6	190,6	18,95	615,83	178,43
2019/05/23	CH2205	Male	Three y/o	Treatment (Wk 2)	Treatment	Feed Day	No	08:40	08:00 - 08:59	Late Morning	37,4		27,4	27,4	18,55		111,09
2019/05/23	CH2206	Male	Three y/o	Treatment (Wk 2)	Treatment	Feed Day	No	08:40	08:00 - 08:59	Late Morning	37,8		163,2	163,2	18,95		172,97
2019/05/23	CH2205	Male	Three y/o	Treatment (Wk 2)	Treatment	Feed Day	No	08:45	08:00 - 08:59	Late Morning	37,5	76	22,8	22,8	18,65	573,39	104,79
2019/05/23	CH2206	Male	Three y/o	Treatment (Wk 2)	Treatment	Feed Day	No	08:45	08:00 - 08:59	Late Morning	37,8	123	196,4	196,4	18,95	608,49	179,48
2019/05/23	CH2205	Male	Three y/o	Treatment (Wk 2)	Treatment	Feed Day	No	08:50	08:00 - 08:59	Late Morning	37,7	66	31,4	31,4	18,85	562,34	115,76
2019/05/23	CH2206	Male	Three y/o	Treatment (Wk 2)	Treatment	Feed Day	No	08:50	08:00 - 08:59	Late Morning	37,8	109	220,8	220,8	18,95	600,04	183,60
2019/05/23	CH2205	Male	Three y/o	Treatment (Wk 2)	Treatment	Feed Day	No	08:55	08:00 - 08:59	Late Morning	37,6	73	30,8	30,8	18,75	570,27	115,10
2019/05/23	CH2206	Male	Three y/o	Treatment (Wk 2)	Treatment	Feed Day	No	08:55	08:00 - 08:59	Late Morning	37,8	105	174,2	174,2	18,95	597,38	175,26
2019/05/23	CH2205	Male	Three y/o	Treatment (Wk 2)	Treatment	Feed Day	No	09:00	09:00 - 09:59	Late Morning	37,5		83,0	83,0	18,65		149,36
2019/05/23	CH2206	Male	Three y/o	Treatment (Wk 2)	Treatment	Feed Day	No	09:00	09:00 - 09:59	Late Morning	37,8	100	234,4	234,4	18,95	593,87	185,71
2019/05/23	CH2205	Male	Three y/o	Treatment (Wk 2)	Treatment	Feed Day	No	09:05	09:00 - 09:59	Late Morning	37,5		69,8	69,8	18,65		143,34

2019/05/23	CH2206	Male	Three y/o	Treatment (Wk 2)	Treatment	Feed Day	No	09:05	09:00 - 09:59	Late Morning	37,8	118	180,4	180,4	18,95	605,61	176,49
2019/05/23	CH2205	Male	Three y/o	Treatment (Wk 2)	Treatment	Feed Day	No	09:10	09:00 - 09:59	Late Morning	37,5	128	114,8	114,8	18,65	611,22	160,66
2019/05/23	CH2206	Male	Three y/o	Treatment (Wk 2)	Treatment	Feed Day	No	09:10	09:00 - 09:59	Late Morning	37,8	86	62,2	62,2	18,95	582,77	139,34
2019/05/23	CH2205	Male	Three y/o	Treatment (Wk 2)	Treatment	Feed Day	No	09:15	09:00 - 09:59	Late Morning	37,5	129	115,6	115,6	18,65	611,75	160,90
2019/05/23	CH2206	Male	Three y/o	Treatment (Wk 2)	Treatment	Feed Day	No	09:15	09:00 - 09:59	Late Morning	37,5	94	53,6	53,6	18,65	589,37	134,19
2019/05/23	CH2205	Male	Three y/o	Treatment (Wk 2)	Treatment	Feed Day	No	09:20	09:00 - 09:59	Late Morning	37,5	70	6,2	6,2	18,65	566,99	60,63
2019/05/23	CH2206	Male	Three y/o	Treatment (Wk 2)	Treatment	Feed Day	No	09:20	09:00 - 09:59	Late Morning	37,7	88	36,0	36,0	18,85	584,49	120,46
2019/05/23	CH2205	Male	Three y/o	Treatment (Wk 2)	Treatment	Feed Day	No	09:25	09:00 - 09:59	Late Morning	37,6	55	10,6	10,6	18,75	547,52	78,73
2019/05/23	CH2206	Male	Three y/o	Treatment (Wk 2)	Treatment	Feed Day	No	09:25	09:00 - 09:59	Late Morning	37,5	68	45,4	45,4	18,65	564,71	128,45
2019/05/23	CH2205	Male	Three y/o	Treatment (Wk 2)	Treatment	Feed Day	No	09:30	09:00 - 09:59	Late Morning	37,7	49	12,2	12,2	18,85	537,80	83,49
2019/05/23	CH2206	Male	Three y/o	Treatment (Wk 2)	Treatment	Feed Day	No	09:30	09:00 - 09:59	Late Morning	37,4	52	100,0	100,0	18,55	542,83	155,84
2019/05/23	CH2205	Male	Three y/o	Treatment (Wk 2)	Treatment	Feed Day	No	09:35	09:00 - 09:59	Late Morning	37,9	102	12,0	12,0	19,05	595,30	82,93
2019/05/23	CH2206	Male	Three y/o	Treatment (Wk 2)	Treatment	Feed Day	No	09:35	09:00 - 09:59	Late Morning	37,5	104	72,0	72,0	18,65	596,69	144,42
2019/05/23	CH2205	Male	Three y/o	Treatment (Wk 2)	Treatment	Feed Day	No	09:40	09:00 - 09:59	Late Morning	37,9	92	25,0	25,0	19,05	587,78	107,94
2019/05/23	CH2206	Male	Three y/o	Treatment (Wk 2)	Treatment	Feed Day	No	09:40	09:00 - 09:59	Late Morning	37,5	74	30,4	30,4	18,65	571,33	114,65
2019/05/23	CH2205	Male	Three y/o	Treatment (Wk 2)	Treatment	Feed Day	No	09:45	09:00 - 09:59	Late Morning	37,7	65	19,2	19,2	18,85	561,12	98,92
2019/05/23	CH2206	Male	Three y/o	Treatment (Wk 2)	Treatment	Feed Day	No	09:45	09:00 - 09:59	Late Morning	37,5	58	27,8	27,8	18,65	551,90	111,58
2019/05/23	CH2205	Male	Three y/o	Treatment (Wk 2)	Treatment	Feed Day	No	09:50	09:00 - 09:59	Late Morning	37,7	67	49,8	49,8	18,85	563,53	131,65
2019/05/23	CH2206	Male	Three y/o	Treatment (Wk 2)	Treatment	Feed Day	No	09:50	09:00 - 09:59	Late Morning	37,7	48	24,4	24,4	18,85	536,04	107,11
2019/05/23	CH2205	Male	Three y/o	Treatment (Wk 2)	Treatment	Feed Day	No	09:55	09:00 - 09:59	Late Morning	37,7		15,8	15,8	18,85		92,28
2019/05/23	CH2206	Male	Three y/o	Treatment (Wk 2)	Treatment	Feed Day	No	09:55	09:00 - 09:59	Late Morning	37,5	70	21,6	21,6	18,65	566,99	102,94
2019/05/23	CH2205	Male	Three y/o	Treatment (Wk 2)	Treatment	Feed Day	No	10:00	10:00 - 10:59	Late Morning	37,6		17,6	17,6	18,75		95,95
2019/05/23	CH2206	Male	Three y/o	Treatment (Wk 2)	Treatment	Feed Day	No	10:00	10:00 - 10:59	Late Morning	37,6	63	21,2	21,2	18,75	558,62	102,31
2019/05/23	CH2205	Male	Three y/o	Treatment (Wk 2)	Treatment	Feed Day	No	10:05	10:00 - 10:59	Late Morning	37,5		15,4	15,4	18,65		91,41
2019/05/23	CH2206	Male	Three y/o	Treatment (Wk 2)	Treatment	Feed Day	No	10:05	10:00 - 10:59	Late Morning	37,5	44	32,4	32,4	18,65	528,51	116,84
2019/05/23	CH2205	Male	Three y/o	Treatment (Wk 2)	Treatment	Feed Day	No	10:10	10:00 - 10:59	Late Morning	37,5		19,4	19,4	18,65		99,28
2019/05/23	CH2206	Male	Three y/o	Treatment (Wk 2)	Treatment	Feed Day	No	10:10	10:00 - 10:59	Late Morning	37,5	54	38,2	38,2	18,65	545,99	122,50
2019/05/23	CH2205	Male	Three y/o	Treatment (Wk 2)	Treatment	Feed Day	No	10:15	10:00 - 10:59	Late Morning	37,3	52	31,6	31,6	18,46	542,83	115,98
2019/05/23	CH2206	Male	Three y/o	Treatment (Wk 2)	Treatment	Feed Day	No	10:15	10:00 - 10:59	Late Morning	37,5	110	50,6	50,6	18,65	600,69	132,20
2019/05/23	CH2205	Male	Three y/o	Treatment (Wk 2)	Treatment	Feed Day	No	10:20	10:00 - 10:59	Late Morning	37,4	44	29,2	29,2	18,55	528,51	113,27
2019/05/23	CH2206	Male	Three y/o	Treatment (Wk 2)	Treatment	Feed Day	No	10:20	10:00 - 10:59	Late Morning	37,3	42	35,6	35,6	18,46	524,42	120,08
2019/05/23	CH2205	Male	Three y/o	Treatment (Wk 2)	Treatment	Feed Day	No	10:25	10:00 - 10:59	Late Morning	37,5	38	23,0	23,0	18,65	515,48	105,09
2019/05/23	CH2206	Male	Three y/o	Treatment (Wk 2)	Treatment	Feed Day	No	10:25	10:00 - 10:59	Late Morning	37,0	47	40,0	40,0	18,16	534,23	124,09
2019/05/23	CH2205	Male	Three y/o	Treatment (Wk 2)	Treatment	Feed Day	Yes	10:30	10:00 - 10:59	Late Morning	37,5	49	19,6	19,6	18,65	537,80	99,63
2019/05/23	CH2206	Male	Three y/o	Treatment (Wk 2)	Treatment	Feed Day	Yes	10:30	10:00 - 10:59	Late Morning	37,0	88	57,6	57,6	18,16	584,49	136,68
2019/05/23	CH2205	Male	Three y/o	Treatment (Wk 2)	Treatment	Feed Day	Yes	10:35	10:00 - 10:59	Late Morning	37,6	50	41,8	41,8	18,75	539,52	125,60
2019/05/23	CH2206	Male	Three y/o	Treatment (Wk 2)	Treatment	Feed Day	Yes	10:35	10:00 - 10:59	Late Morning	37,0	80	53,4	53,4	18,16	577,31	134,06
2019/05/23	CH2205	Male	Three y/o	Treatment (Wk 2)	Treatment	Feed Day	Yes	10:40	10:00 - 10:59	Late Morning	37,6	38	54,0	54,0	18,75	515,48	134,45
2019/05/23	CH2206	Male	Three y/o	Treatment (Wk 2)	Treatment	Feed Day	Yes	10:40	10:00 - 10:59	Late Morning	37,0	90	81,6	81,6	18,16	586,16	148,76
2019/05/23	CH2205	Male	Three y/o	Treatment (Wk 2)	Treatment	Feed Day	Yes	10:45	10:00 - 10:59	Late Morning	37,6	94	18,4	18,4	18,75	589,37	97,47
2019/05/23	CH2206	Male	Three y/o	Treatment (Wk 2)	Treatment	Feed Day	Yes	10:45	10:00 - 10:59	Late Morning	37,1	89	43,0	43,0	18,26	585,33	126,58
2019/05/23	CH2205	Male	Three y/o	Treatment (Wk 2)	Treatment	Feed Day	Yes	10:50	10:00 - 10:59	Late Morning	37,6	79	68,2	68,2	18,75	576,36	142,53
2019/05/23	CH2206	Male	Three y/o	Treatment (Wk 2)	Treatment	Feed Day	Yes	10:50	10:00 - 10:59	Late Morning	37,2	108	133,8	133,8	18,36	599,39	166,01
2019/05/23	CH2205	Male	Three y/o	Treatment (Wk 2)	Treatment	Feed Day	Yes	10:55	10:00 - 10:59	Late Morning	37,7	87	167,6	167,6	18,85	583,64	173,91
2019/05/23	CH2206	Male	Three y/o	Treatment (Wk 2)	Treatment	Feed Day	Yes	10:55	10:00 - 10:59	Late Morning	37,4		126,6	126,6	18,55		164,08
2019/05/23	CH2205	Male	Three y/o	Treatment (Wk 2)	Treatment	Feed Day	Yes	11:00	11:00 - 11:59	Late Morning	37,9		122,0	122,0	19,05		162,79
2019/05/23	CH2206	Male	Three y/o	Treatment (Wk 2)	Treatment	Feed Day	Yes	11:00	11:00 - 11:59	Late Morning	37,5	97	295,6	295,6	18,65	591,66	193,90
2019/05/23	CH2205	Male	Three y/o	Treatment (Wk 2)	Treatment	Feed Day	Yes	11:05	11:00 - 11:59	Late Morning	38,0	113	234,6	234,6	19,15	602,58	185,74
2019/05/23	CH2206	Male	Three y/o	Treatment (Wk 2)	Treatment	Feed Day	Yes	11:05	11:00 - 11:59	Late Morning	37,7	118	302,8	302,8	18,85	605,61	194,75
2019/05/23	CH2205	Male	Three y/o	Treatment (Wk 2)	Treatment	Feed Day	Yes	11:10	11:00 - 11:59	Late Morning	38,0	148	154,0	154,0	19,15	620,97	170,94
2019/05/23	CH2206	Male	Three y/o	Treatment (Wk 2)	Treatment	Feed Day	Yes	11:10	11:00 - 11:59	Late Morning	37,7	40	82,6	82,6	18,85	520,09	149,19
2019/05/23	CH2205	Male	Three y/o	Treatment (Wk 2)	Treatment	Feed Day	Yes	11:15	11:00 - 11:59	Late Morning	38,0		247,8	247,8	19,15		187,67
2019/05/23	CH2206	Male	Three y/o	Treatment (Wk 2)	Treatment	Feed Day	Yes	11:15	11:00 - 11:59	Late Morning	37,8	150	179,0	179,0	18,95	621,86	176,22
2019/05/23	CH2205	Male	Three y/o	Treatment (Wk 2)	Treatment	Feed Day	Yes	11:20	11:00 - 11:59	Late Morning	38,0	96	22,0	22,0	19,15	590,91	103,57
2019/05/23	CH2206	Male	Three y/o	Treatment (Wk 2)	Treatment	Feed Day	Yes	11:20	11:00 - 11:59	Late Morning	37,9	99	35,2	35,2	19,05	593,14	119,69
2019/05/23	CH2205	Male	Three y/o	Treatment (Wk 2)	Treatment	Feed Day	Yes	11:25	11:00 - 11:59	Late Morning	37,7	80	14,8	14,8	18,85	577,31	90,05
2019/05/23	CH2206	Male	Three y/o	Treatment (Wk 2)	Treatment	Feed Day	Yes	11:25	11:00 - 11:59	Late Morning	37,8	83	25,6	25,6	18,95	580,11	108,76
2019/05/23	CH2205	Male	Three y/o	Treatment (Wk 2)	Treatment	Feed Day	No	11:30	11:00 - 11:59	Late Morning	37,5		33,6	33,6	18,65		118,09
2019/05/23	CH2206	Male	Three y/o	Treatment (Wk 2)	Treatment	Feed Day	No	11:30	11:00 - 11:59	Late Morning	37,7	109	49,8	49,8	18,85	600,04	131,65
2019/05/23	CH2205	Male	Three y/o	Treatment (Wk 2)	Treatment	Feed Day	No	11:35	11:00 - 11:59	Late Morning	37,7	103	166,4	166,4	18,85	596,00	173,66
2019/05/23	CH2206	Male	Three y/o	Treatment (Wk 2)	Treatment	Feed Day	No	11:35	11:00 - 11:59	Late Morning	37,7	94	21,4	21,4	18,85	589,37	102,63
2019/05/23	CH2205	Male	Three y/o	Treatment (Wk 2)	Treatment	Feed Day	No	11:40	11:00 - 11:59	Late Morning	37,9	177	43,0	43,0	19,05	632,55	126,58
2019/05/23	CH2206	Male	Three y/o	Treatment (Wk 2)	Treatment	Feed Day	No	11:40	11:00 - 11:59	Late Morning	37,7	78	29,6	29,6	18,85	575,38	113,73
2019/05/23	CH2205	Male	Three y/o	Treatment (Wk 2)	Treatment	Feed Day	No	11:45	11:00 - 11:59	Late Morning	37,9	98	59,2	59,2	19,05	592,41	137,63
2019/05/23	CH2206	Male	Three y/o	Treatment (Wk 2)	Treatment	Feed Day	No	11:45	11:00 - 11:59	Late Morning	37,7	46	33,8	33,8	18,85	532,38	118,29
2019/05/23	CH2205	Male	Three y/o	Treatment (Wk 2)	Treatment	Feed Day	No	11:50	11:00 - 11:59	Late Morning	38,0	121	56,8	56,8	19,15	607,35	136,20
2019/05/23	CH2206	Male	Three y/o	Treatment (Wk 2)	Treatment	Feed Day	No	11:50	11:00 - 11:59	Late Morning	37,8	88	30,6	30,6	18,95	584,49	114,87
2019/05/23	CH2205	Male	Three y/o	Treatment (Wk 2)	Treatment	Feed Day	No	11:55	11:00 - 11:59	Late Morning	38,1	98	68,2	68,2	19,26	592,41	142,53
2019/05/23	CH2206	Male	Three y/o	Treatment (Wk 2)	Treatment	Feed Day	No	11:55	11:00 - 11:59	Late Morning	37,8	51	15,0	15,0	18,95	541,20	90,51
2019/05/23	CH2205	Male	Three y/o	Treatment (Wk 2)	Treatment	Feed Day	No	12:00	12:00 - 12:59	Afternoon	38,1	75	14,2	14,2	19,26	572,36	88,65

2019/05/23	CH2206	Male	Three y/o	Treatment (Wk 2)	Treatment	Feed Day	No	12:00	12:00 - 12:59	Afternoon	37,8	54	5,0	5,0	18,95	545,99	53,41
2019/05/23	CH2205	Male	Three y/o	Treatment (Wk 2)	Treatment	Feed Day	No	12:05	12:00 - 12:59	Afternoon	37,7		22,4	22,4	18,85		104,19
2019/05/23	CH2206	Male	Three y/o	Treatment (Wk 2)	Treatment	Feed Day	No	12:05	12:00 - 12:59	Afternoon	37,9	63	14,6	14,6	19,05	558,62	89,59
2019/05/23	CH2205	Male	Three y/o	Treatment (Wk 2)	Treatment	Feed Day	No	12:10	12:00 - 12:59	Afternoon	37,7	62	10,0	10,0	18,85	557,33	76,76
2019/05/23	CH2206	Male	Three y/o	Treatment (Wk 2)	Treatment	Feed Day	No	12:10	12:00 - 12:59	Afternoon	37,9	80	7,8	7,8	19,05	577,31	68,36
2019/05/23	CH2205	Male	Three y/o	Treatment (Wk 2)	Treatment	Feed Day	No	12:15	12:00 - 12:59	Afternoon	37,9	134	21,0	21,0	19,05	614,33	101,98
2019/05/23	CH2206	Male	Three y/o	Treatment (Wk 2)	Treatment	Feed Day	No	12:15	12:00 - 12:59	Afternoon	37,9	76	37,0	37,0	19,05	573,39	121,40
2019/05/23	CH2205	Male	Three y/o	Treatment (Wk 2)	Treatment	Feed Day	No	12:20	12:00 - 12:59	Afternoon	37,9	53	16,6	16,6	19,05	544,43	93,96
2019/05/23	CH2206	Male	Three y/o	Treatment (Wk 2)	Treatment	Feed Day	No	12:20	12:00 - 12:59	Afternoon	37,9	71	35,0	35,0	19,05	568,10	119,49
2019/05/23	CH2205	Male	Three y/o	Treatment (Wk 2)	Treatment	Feed Day	No	12:25	12:00 - 12:59	Afternoon	37,9	65	6,0	6,0	19,05	561,12	59,53
2019/05/23	CH2206	Male	Three y/o	Treatment (Wk 2)	Treatment	Feed Day	No	12:25	12:00 - 12:59	Afternoon	38,0	65	38,0	38,0	19,15	561,12	122,32
2019/05/23	CH2205	Male	Three y/o	Treatment (Wk 2)	Treatment	Feed Day	No	12:30	12:00 - 12:59	Afternoon	37,9	65	14,8	14,8	19,05	561,12	90,05
2019/05/23	CH2206	Male	Three y/o	Treatment (Wk 2)	Treatment	Feed Day	No	12:30	12:00 - 12:59	Afternoon	38,0	63	28,4	28,4	19,15	558,62	112,31
2019/05/23	CH2205	Male	Three y/o	Treatment (Wk 2)	Treatment	Feed Day	No	12:35	12:00 - 12:59	Afternoon	37,9	57	22,8	22,8	19,05	550,47	104,79
2019/05/23	CH2206	Male	Three y/o	Treatment (Wk 2)	Treatment	Feed Day	No	12:35	12:00 - 12:59	Afternoon	38,0	46	19,8	19,8	19,15	532,38	99,97
2019/05/23	CH2205	Male	Three y/o	Treatment (Wk 2)	Treatment	Feed Day	No	12:40	12:00 - 12:59	Afternoon	37,9	59	25,0	25,0	19,05	553,30	107,94
2019/05/23	CH2206	Male	Three y/o	Treatment (Wk 2)	Treatment	Feed Day	No	12:40	12:00 - 12:59	Afternoon	38,0	64	20,4	20,4	19,15	559,88	100,99
2019/05/23	CH2205	Male	Three y/o	Treatment (Wk 2)	Treatment	Feed Day	No	12:45	12:00 - 12:59	Afternoon	37,9	68	22,6	22,6	19,05	564,71	104,49
2019/05/23	CH2206	Male	Three y/o	Treatment (Wk 2)	Treatment	Feed Day	No	12:45	12:00 - 12:59	Afternoon	38,0	90	26,6	26,6	19,15	586,16	110,07
2019/05/23	CH2205	Male	Three y/o	Treatment (Wk 2)	Treatment	Feed Day	No	12:50	12:00 - 12:59	Afternoon	37,9	62	17,0	17,0	19,05	557,33	94,77
2019/05/23	CH2206	Male	Three y/o	Treatment (Wk 2)	Treatment	Feed Day	No	12:50	12:00 - 12:59	Afternoon	38,1	89	85,4	85,4	19,26	585,33	150,35
2019/05/23	CH2205	Male	Three y/o	Treatment (Wk 2)	Treatment	Feed Day	No	12:55	12:00 - 12:59	Afternoon	37,7		23,0	23,0	18,85		105,09
2019/05/23	CH2206	Male	Three y/o	Treatment (Wk 2)	Treatment	Feed Day	No	12:55	12:00 - 12:59	Afternoon	38,1	69	45,4	45,4	19,26	565,86	128,45
2019/05/23	CH2205	Male	Three y/o	Treatment (Wk 2)	Treatment	Feed Day	No	13:00	13:00 - 13:59	Afternoon	37,7	50	22,0	22,0	18,85	539,52	103,57
2019/05/23	CH2206	Male	Three y/o	Treatment (Wk 2)	Treatment	Feed Day	No	13:00	13:00 - 13:59	Afternoon	38,1	48	50,6	50,6	19,26	536,04	132,20
2019/05/23	CH2205	Male	Three y/o	Treatment (Wk 2)	Treatment	Feed Day	No	13:05	13:00 - 13:59	Afternoon	37,9	64	17,8	17,8	19,05	559,88	96,34
2019/05/23	CH2206	Male	Three y/o	Treatment (Wk 2)	Treatment	Feed Day	No	13:05	13:00 - 13:59	Afternoon	38,1	91	27,0	27,0	19,26	586,98	110,58
2019/05/23	CH2205	Male	Three y/o	Treatment (Wk 2)	Treatment	Feed Day	No	13:10	13:00 - 13:59	Afternoon	37,9	60	4,6	4,6	19,05	554,67	50,61
2019/05/23	CH2206	Male	Three y/o	Treatment (Wk 2)	Treatment	Feed Day	No	13:10	13:00 - 13:59	Afternoon	38,1	76	34,6	34,6	19,26	573,39	119,10
2019/05/23	CH2205	Male	Three y/o	Treatment (Wk 2)	Treatment	Feed Day	No	13:15	13:00 - 13:59	Afternoon	37,9	67	14,6	14,6	19,05	563,53	89,59
2019/05/23	CH2206	Male	Three y/o	Treatment (Wk 2)	Treatment	Feed Day	No	13:15	13:00 - 13:59	Afternoon	38,1	88	33,0	33,0	19,26	584,49	117,47
2019/05/23	CH2205	Male	Three y/o	Treatment (Wk 2)	Treatment	Feed Day	No	13:20	13:00 - 13:59	Afternoon	37,9	84	19,2	19,2	19,05	581,01	98,92
2019/05/23	CH2206	Male	Three y/o	Treatment (Wk 2)	Treatment	Feed Day	No	13:20	13:00 - 13:59	Afternoon	38,1	84	31,0	31,0	19,26	581,01	115,32
2019/05/23	CH2205	Male	Three y/o	Treatment (Wk 2)	Treatment	Feed Day	No	13:25	13:00 - 13:59	Afternoon	37,9	83	22,6	22,6	19,05	580,11	104,49
2019/05/23	CH2206	Male	Three y/o	Treatment (Wk 2)	Treatment	Feed Day	No	13:25	13:00 - 13:59	Afternoon	38,1	47	31,8	31,8	19,26	534,23	116,20
2019/05/23	CH2205	Male	Three y/o	Treatment (Wk 2)	Treatment	Feed Day	No	13:30	13:00 - 13:59	Afternoon	38,0	70	26,8	26,8	19,15	566,99	110,33
2019/05/23	CH2206	Male	Three y/o	Treatment (Wk 2)	Treatment	Feed Day	No	13:30	13:00 - 13:59	Afternoon	38,0	83	34,6	34,6	19,15	580,11	119,10
2019/05/23	CH2205	Male	Three y/o	Treatment (Wk 2)	Treatment	Feed Day	No	13:35	13:00 - 13:59	Afternoon	38,0	60	27,8	27,8	19,15	554,67	111,58
2019/05/23	CH2206	Male	Three y/o	Treatment (Wk 2)	Treatment	Feed Day	No	13:35	13:00 - 13:59	Afternoon	38,0	47	29,0	29,0	19,15	534,23	113,03
2019/05/23	CH2205	Male	Three y/o	Treatment (Wk 2)	Treatment	Feed Day	No	13:40	13:00 - 13:59	Afternoon	38,0	59	27,4	27,4	19,15	553,30	111,09
2019/05/23	CH2206	Male	Three y/o	Treatment (Wk 2)	Treatment	Feed Day	No	13:40	13:00 - 13:59	Afternoon	38,0	57	29,2	29,2	19,15	550,47	113,27
2019/05/23	CH2205	Male	Three y/o	Treatment (Wk 2)	Treatment	Feed Day	No	13:45	13:00 - 13:59	Afternoon	38,0	64	27,2	27,2	19,15	559,88	110,83
2019/05/23	CH2206	Male	Three y/o	Treatment (Wk 2)	Treatment	Feed Day	No	13:45	13:00 - 13:59	Afternoon	38,0	82	39,8	39,8	19,15	579,19	123,91
2019/05/23	CH2205	Male	Three y/o	Treatment (Wk 2)	Treatment	Feed Day	No	13:50	13:00 - 13:59	Afternoon	38,0	57	24,6	24,6	19,15	550,47	107,39
2019/05/23	CH2206	Male	Three y/o	Treatment (Wk 2)	Treatment	Feed Day	No	13:50	13:00 - 13:59	Afternoon	37,9	82	36,6	36,6	19,05	579,19	121,03
2019/05/23	CH2205	Male	Three y/o	Treatment (Wk 2)	Treatment	Feed Day	No	13:55	13:00 - 13:59	Afternoon	38,0	70	25,4	25,4	19,15	566,99	108,49
2019/05/23	CH2206	Male	Three y/o	Treatment (Wk 2)	Treatment	Feed Day	No	13:55	13:00 - 13:59	Afternoon	37,9	50	37,8	37,8	19,05	539,52	122,14
2019/05/23	CH2205	Male	Three y/o	Treatment (Wk 2)	Treatment	Feed Day	No	14:00	14:00 - 14:59	Afternoon	38,0	57	21,8	21,8	19,15	550,47	103,26
2019/05/23	CH2206	Male	Three y/o	Treatment (Wk 2)	Treatment	Feed Day	No	14:00	14:00 - 14:59	Afternoon	37,9	52	34,4	34,4	19,05	542,83	118,90
2019/05/23	CH2205	Male	Three y/o	Treatment (Wk 2)	Treatment	Feed Day	No	14:05	14:00 - 14:59	Afternoon	38,1		13,4	13,4	19,26		86,68
2019/05/23	CH2206	Male	Three y/o	Treatment (Wk 2)	Treatment	Feed Day	No	14:05	14:00 - 14:59	Afternoon	37,9	113	57,4	57,4	19,05	602,58	136,56
2019/05/23	CH2205	Male	Three y/o	Treatment (Wk 2)	Treatment	Feed Day	No	14:10	14:00 - 14:59	Afternoon	38,1	60	5,0	5,0	19,26	554,67	53,41
2019/05/23	CH2206	Male	Three y/o	Treatment (Wk 2)	Treatment	Feed Day	No	14:10	14:00 - 14:59	Afternoon	37,9	71	54,6	54,6	19,05	568,10	134,83
2019/05/23	CH2205	Male	Three y/o	Treatment (Wk 2)	Treatment	Feed Day	No	14:15	14:00 - 14:59	Afternoon	38,1	57	10,6	10,6	19,26	550,47	78,73
2019/05/23	CH2206	Male	Three y/o	Treatment (Wk 2)	Treatment	Feed Day	No	14:15	14:00 - 14:59	Afternoon	37,9	80	41,2	41,2	19,05	577,31	125,11
2019/05/23	CH2205	Male	Three y/o	Treatment (Wk 2)	Treatment	Feed Day	No	14:20	14:00 - 14:59	Afternoon	38,1	57	17,0	17,0	19,26	550,47	94,77
2019/05/23	CH2206	Male	Three y/o	Treatment (Wk 2)	Treatment	Feed Day	No	14:20	14:00 - 14:59	Afternoon	37,9	74	47,6	47,6	19,05	571,33	130,09
2019/05/23	CH2205	Male	Three y/o	Treatment (Wk 2)	Treatment	Feed Day	No	14:25	14:00 - 14:59	Afternoon	38,0	50	4,4	4,4	19,15	539,52	49,12
2019/05/23	CH2206	Male	Three y/o	Treatment (Wk 2)	Treatment	Feed Day	No	14:25	14:00 - 14:59	Afternoon	37,9	86	68,6	68,6	19,05	582,77	142,74
2019/05/23	CH2205	Male	Three y/o	Treatment (Wk 2)	Treatment	Feed Day	No	14:30	14:00 - 14:59	Afternoon	38,0	82	1,4	1,4	19,15	579,19	11,07
2019/05/23	CH2206	Male	Three y/o	Treatment (Wk 2)	Treatment	Feed Day	No	14:30	14:00 - 14:59	Afternoon	37,9	84	37,8	37,8	19,05	581,01	122,14
2019/05/23	CH2205	Male	Three y/o	Treatment (Wk 2)	Treatment	Feed Day	No	14:35	14:00 - 14:59	Afternoon	38,0	99	3,6	3,6	19,15	593,14	42,42
2019/05/23	CH2206	Male	Three y/o	Treatment (Wk 2)	Treatment	Feed Day	No	14:35	14:00 - 14:59	Afternoon	37,9	70	14,0	14,0	19,05	566,99	88,17
2019/05/23	CH2205	Male	Three y/o	Treatment (Wk 2)	Treatment	Feed Day	No	14:40	14:00 - 14:59	Afternoon	38,0	56	3,4	3,4	19,15	549,01	40,51
2019/05/23	CH2206	Male	Three y/o	Treatment (Wk 2)	Treatment	Feed Day	No	14:40	14:00 - 14:59	Afternoon	37,9	72	8,6	8,6	19,05	569,20	71,66
2019/05/23	CH2205	Male	Three y/o	Treatment (Wk 2)	Treatment	Feed Day	No	14:45	14:00 - 14:59	Afternoon	38,0	65	35,0	35,0	19,15	561,12	119,49
2019/05/23	CH2206	Male	Three y/o	Treatment (Wk 2)	Treatment	Feed Day	No	14:45	14:00 - 14:59	Afternoon	37,9	78	7,8	7,8	19,05	575,38	68,36
2019/05/23	CH2205	Male	Three y/o	Treatment (Wk 2)	Treatment	Feed Day	No	14:50	14:00 - 14:59	Afternoon	37,9	197	17,2	17,2	19,05	639,25	95,17
2019/05/23	CH2206	Male	Three y/o	Treatment (Wk 2)	Treatment	Feed Day	No	14:50	14:00 - 14:59	Afternoon	37,9	90	20,6	20,6	19,05	586,16	101,32
2019/05/23	CH2205	Male	Three y/o	Treatment (Wk 2)	Treatment	Feed Day	No	14:55	14:00 - 14:59	Afternoon	37,9	72	24,8	24,8	19,05	569,20	107,67

2019/05/23	CH2206	Male	Three y/o	Treatment (Wk 2)	Treatment	Feed Day	No	14:55	14:00 - 14:59	Afternoon	38,0	46	8,2	8,2	19,15	532,38	70,05
2019/05/23	CH2205	Male	Three y/o	Treatment (Wk 2)	Treatment	Feed Day	No	15:00	15:00 - 15:59	Afternoon	37,9	60	21,6	21,6	19,05	554,67	102,94
2019/05/23	CH2206	Male	Three y/o	Treatment (Wk 2)	Treatment	Feed Day	No	15:00	15:00 - 15:59	Afternoon	38,0	81	3,8	3,8	19,15	578,26	44,22
2019/05/23	CH2205	Male	Three y/o	Treatment (Wk 2)	Treatment	Feed Day	No	15:05	15:00 - 15:59	Afternoon	38,0	62	23,2	23,2	19,15	557,33	105,39
2019/05/23	CH2206	Male	Three y/o	Treatment (Wk 2)	Treatment	Feed Day	No	15:05	15:00 - 15:59	Afternoon	38,0	65	3,4	3,4	19,15	561,12	40,51
2019/05/23	CH2205	Male	Three y/o	Treatment (Wk 2)	Treatment	Feed Day	No	15:10	15:00 - 15:59	Afternoon	38,0	83	23,8	23,8	19,15	580,11	106,26
2019/05/23	CH2206	Male	Three y/o	Treatment (Wk 2)	Treatment	Feed Day	No	15:10	15:00 - 15:59	Afternoon	38,0	46	15,8	15,8	19,15	532,38	92,28
2019/05/23	CH2205	Male	Three y/o	Treatment (Wk 2)	Treatment	Feed Day	No	15:15	15:00 - 15:59	Afternoon	38,0	61	23,6	23,6	19,15	556,01	105,97
2019/05/23	CH2206	Male	Three y/o	Treatment (Wk 2)	Treatment	Feed Day	No	15:15	15:00 - 15:59	Afternoon	38,1	86	32,6	32,6	19,26	582,77	117,05
2019/05/23	CH2205	Male	Three y/o	Treatment (Wk 2)	Treatment	Feed Day	No	15:20	15:00 - 15:59	Afternoon	38,0	63	26,0	26,0	19,15	558,62	109,29
2019/05/23	CH2206	Male	Three y/o	Treatment (Wk 2)	Treatment	Feed Day	No	15:20	15:00 - 15:59	Afternoon	38,1	89	26,6	26,6	19,26	585,33	110,07
2019/05/23	CH2205	Male	Three y/o	Treatment (Wk 2)	Treatment	Feed Day	No	15:25	15:00 - 15:59	Afternoon	38,0	63	42,2	42,2	19,15	558,62	125,93
2019/05/23	CH2206	Male	Three y/o	Treatment (Wk 2)	Treatment	Feed Day	No	15:25	15:00 - 15:59	Afternoon	38,1	78	23,8	23,8	19,26	575,38	106,26
2019/05/23	CH2205	Male	Three y/o	Treatment (Wk 2)	Treatment	Feed Day	No	15:30	15:00 - 15:59	Afternoon	38,0	73	18,0	18,0	19,15	570,27	96,72
2019/05/23	CH2206	Male	Three y/o	Treatment (Wk 2)	Treatment	Feed Day	No	15:30	15:00 - 15:59	Afternoon	38,1	86	15,8	15,8	19,26	582,77	92,28
2019/05/23	CH2205	Male	Three y/o	Treatment (Wk 2)	Treatment	Feed Day	No	15:35	15:00 - 15:59	Afternoon	38,0		15,0	15,0	19,15		90,51
2019/05/23	CH2206	Male	Three y/o	Treatment (Wk 2)	Treatment	Feed Day	No	15:35	15:00 - 15:59	Afternoon	38,1	64	16,0	16,0	19,26	559,88	92,71
2019/05/23	CH2205	Male	Three y/o	Treatment (Wk 2)	Treatment	Feed Day	No	15:40	15:00 - 15:59	Afternoon	38,0	61	3,6	3,6	19,15	556,01	42,42
2019/05/23	CH2206	Male	Three y/o	Treatment (Wk 2)	Treatment	Feed Day	No	15:40	15:00 - 15:59	Afternoon	38,1	55	35,2	35,2	19,26	547,52	119,69
2019/05/23	CH2205	Male	Three y/o	Treatment (Wk 2)	Treatment	Feed Day	No	15:45	15:00 - 15:59	Afternoon	38,0	92	5,6	5,6	19,15	587,78	57,21
2019/05/23	CH2206	Male	Three y/o	Treatment (Wk 2)	Treatment	Feed Day	No	15:45	15:00 - 15:59	Afternoon	38,0	82	37,8	37,8	19,15	579,19	122,14
2019/05/23	CH2205	Male	Three y/o	Treatment (Wk 2)	Treatment	Feed Day	No	15:50	15:00 - 15:59	Afternoon	38,0	64	90,4	90,4	19,15	559,88	152,33
2019/05/23	CH2206	Male	Three y/o	Treatment (Wk 2)	Treatment	Feed Day	No	15:50	15:00 - 15:59	Afternoon	38,0	82	48,0	48,0	19,15	579,19	130,38
2019/05/23	CH2205	Male	Three y/o	Treatment (Wk 2)	Treatment	Feed Day	No	15:55	15:00 - 15:59	Afternoon	38,0	102	29,8	29,8	19,15	595,30	113,97
2019/05/23	CH2206	Male	Three y/o	Treatment (Wk 2)	Treatment	Feed Day	No	15:55	15:00 - 15:59	Afternoon	38,0	103	65,8	65,8	19,15	596,00	141,29
2019/05/23	CH2205	Male	Three y/o	Treatment (Wk 2)	Treatment	Feed Day	No	16:00	16:00 - 16:59	Evening	38,0		17,4	17,4	19,15		95,56
2019/05/23	CH2206	Male	Three y/o	Treatment (Wk 2)	Treatment	Feed Day	No	16:00	16:00 - 16:59	Evening	38,0	105	44,2	44,2	19,15	597,38	127,53
2019/05/23	CH2205	Male	Three y/o	Treatment (Wk 2)	Treatment	Feed Day	No	16:05	16:00 - 16:59	Evening	37,7	91	17,4	17,4	18,85	586,98	95,56
2019/05/23	CH2206	Male	Three y/o	Treatment (Wk 2)	Treatment	Feed Day	No	16:05	16:00 - 16:59	Evening	38,0	111	55,0	55,0	19,15	601,33	135,08
2019/05/23	CH2205	Male	Three y/o	Treatment (Wk 2)	Treatment	Feed Day	No	16:10	16:00 - 16:59	Evening	37,7	93	40,4	40,4	18,85	588,58	124,43
2019/05/23	CH2206	Male	Three y/o	Treatment (Wk 2)	Treatment	Feed Day	No	16:10	16:00 - 16:59	Evening	37,9	103	64,6	64,6	19,05	596,00	140,65
2019/05/23	CH2205	Male	Three y/o	Treatment (Wk 2)	Treatment	Feed Day	No	16:15	16:00 - 16:59	Evening	37,7	79	17,6	17,6	18,85	576,36	95,95
2019/05/23	CH2206	Male	Three y/o	Treatment (Wk 2)	Treatment	Feed Day	No	16:15	16:00 - 16:59	Evening	37,9	126	53,2	53,2	19,05	610,14	133,93
2019/05/23	CH2205	Male	Three y/o	Treatment (Wk 2)	Treatment	Feed Day	No	16:20	16:00 - 16:59	Evening	37,9	94	78,2	78,2	19,05	589,37	147,28
2019/05/23	CH2206	Male	Three y/o	Treatment (Wk 2)	Treatment	Feed Day	No	16:20	16:00 - 16:59	Evening	37,9	47	8,6	8,6	19,05	534,23	71,66
2019/05/23	CH2205	Male	Three y/o	Treatment (Wk 2)	Treatment	Feed Day	No	16:25	16:00 - 16:59	Evening	38,0	98	49,6	49,6	19,15	592,41	131,51
2019/05/23	CH2206	Male	Three y/o	Treatment (Wk 2)	Treatment	Feed Day	No	16:25	16:00 - 16:59	Evening	37,9	80	14,8	14,8	19,05	577,31	90,05
2019/05/23	CH2205	Male	Three y/o	Treatment (Wk 2)	Treatment	Feed Day	No	16:30	16:00 - 16:59	Evening	37,9	167	30,0	30,0	19,05	628,84	114,20
2019/05/23	CH2206	Male	Three y/o	Treatment (Wk 2)	Treatment	Feed Day	No	16:30	16:00 - 16:59	Evening	37,9	92	95,8	95,8	19,05	587,78	154,35
2019/05/23	CH2205	Male	Three y/o	Treatment (Wk 2)	Treatment	Feed Day	No	16:35	16:00 - 16:59	Evening	38,0	91	25,6	25,6	19,15	586,98	108,76
2019/05/23	CH2206	Male	Three y/o	Treatment (Wk 2)	Treatment	Feed Day	No	16:35	16:00 - 16:59	Evening	37,9	84	30,8	30,8	19,05	581,01	115,10
2019/05/23	CH2205	Male	Three y/o	Treatment (Wk 2)	Treatment	Feed Day	No	16:40	16:00 - 16:59	Evening	38,0	77	18,0	18,0	19,15	574,39	96,72
2019/05/23	CH2206	Male	Three y/o	Treatment (Wk 2)	Treatment	Feed Day	No	16:40	16:00 - 16:59	Evening	37,8	79	34,0	34,0	18,95	576,36	118,49
2019/05/23	CH2205	Male	Three y/o	Treatment (Wk 2)	Treatment	Feed Day	No	16:45	16:00 - 16:59	Evening	38,1	60	21,0	21,0	19,26	554,67	101,98
2019/05/23	CH2206	Male	Three y/o	Treatment (Wk 2)	Treatment	Feed Day	No	16:45	16:00 - 16:59	Evening	37,8	90	30,8	30,8	18,95	586,16	115,10
2019/05/23	CH2205	Male	Three y/o	Treatment (Wk 2)	Treatment	Feed Day	No	16:50	16:00 - 16:59	Evening	38,2	56	18,6	18,6	19,36	549,01	97,84
2019/05/23	CH2206	Male	Three y/o	Treatment (Wk 2)	Treatment	Feed Day	No	16:50	16:00 - 16:59	Evening	37,7	73	36,0	36,0	18,85	570,27	120,46
2019/05/23	CH2205	Male	Three y/o	Treatment (Wk 2)	Treatment	Feed Day	No	16:55	16:00 - 16:59	Evening	38,2	65	20,0	20,0	19,36	561,12	100,31
2019/05/23	CH2206	Male	Three y/o	Treatment (Wk 2)	Treatment	Feed Day	No	16:55	16:00 - 16:59	Evening	37,8	53	38,0	38,0	18,95	544,43	122,32
2019/05/23	CH2205	Male	Three y/o	Treatment (Wk 2)	Treatment	Feed Day	No	17:00	17:00 - 17:59	Evening	38,2	47	19,2	19,2	19,36	534,23	98,92
2019/05/23	CH2206	Male	Three y/o	Treatment (Wk 2)	Treatment	Feed Day	No	17:00	17:00 - 17:59	Evening	37,8	50	46,0	46,0	18,95	539,52	128,91
2019/05/23	CH2205	Male	Three y/o	Treatment (Wk 2)	Treatment	Feed Day	No	17:05	17:00 - 17:59	Evening	38,2	49	16,8	16,8	19,36	537,80	94,37
2019/05/23	CH2206	Male	Three y/o	Treatment (Wk 2)	Treatment	Feed Day	No	17:05	17:00 - 17:59	Evening	37,8	84	40,2	40,2	18,95	581,01	124,26
2019/05/23	CH2205	Male	Three y/o	Treatment (Wk 2)	Treatment	Feed Day	No	17:10	17:00 - 17:59	Evening	38,2	71	21,8	21,8	19,36	568,10	103,26
2019/05/23	CH2206	Male	Three y/o	Treatment (Wk 2)	Treatment	Feed Day	No	17:10	17:00 - 17:59	Evening	37,7	88	36,0	36,0	18,85	584,49	120,46
2019/05/23	CH2205	Male	Three y/o	Treatment (Wk 2)	Treatment	Feed Day	No	17:15	17:00 - 17:59	Evening	38,1	62	16,0	16,0	19,26	557,33	92,71
2019/05/23	CH2206	Male	Three y/o	Treatment (Wk 2)	Treatment	Feed Day	No	17:15	17:00 - 17:59	Evening	37,5	49	38,8	38,8	18,65	537,80	123,04
2019/05/23	CH2205	Male	Three y/o	Treatment (Wk 2)	Treatment	Feed Day	No	17:20	17:00 - 17:59	Evening	38,1	77	15,2	15,2	19,26	574,39	90,96
2019/05/23	CH2206	Male	Three y/o	Treatment (Wk 2)	Treatment	Feed Day	No	17:20	17:00 - 17:59	Evening	37,4	83	46,8	46,8	18,55	580,11	129,50
2019/05/23	CH2205	Male	Three y/o	Treatment (Wk 2)	Treatment	Feed Day	No	17:25	17:00 - 17:59	Evening	38,1	100	29,0	29,0	19,26	593,87	113,03
2019/05/23	CH2206	Male	Three y/o	Treatment (Wk 2)	Treatment	Feed Day	No	17:25	17:00 - 17:59	Evening	37,4	90	27,2	27,2	18,55	586,16	110,83
2019/05/23	CH2205	Male	Three y/o	Treatment (Wk 2)	Treatment	Feed Day	No	17:30	17:00 - 17:59	Evening	38,1		28,6	28,6	19,26		112,56
2019/05/23	CH2206	Male	Three y/o	Treatment (Wk 2)	Treatment	Feed Day	No	17:30	17:00 - 17:59	Evening	37,3	92	44,2	44,2	18,46	587,78	127,53
2019/05/23	CH2205	Male	Three y/o	Treatment (Wk 2)	Treatment	Feed Day	No	17:35	17:00 - 17:59	Evening	38,0		47,4	47,4	19,15		129,94
2019/05/23	CH2206	Male	Three y/o	Treatment (Wk 2)	Treatment	Feed Day	No	17:35	17:00 - 17:59	Evening	37,3	86	43,6	43,6	18,46	582,77	127,06
2019/05/23	CH2205	Male	Three y/o	Treatment (Wk 2)	Treatment	Feed Day	No	17:40	17:00 - 17:59	Evening	37,9		205,0	205,0	19,05		180,99
2019/05/23	CH2206	Male	Three y/o	Treatment (Wk 2)	Treatment	Feed Day	No	17:40	17:00 - 17:59	Evening	37,4	93	49,4	49,4	18,55	588,58	131,37
2019/05/23	CH2205	Male	Three y/o	Treatment (Wk 2)	Treatment	Feed Day	No	17:45	17:00 - 17:59	Evening	37,9	103	190,4	190,4	19,05	596,00	178,39
2019/05/23	CH2206	Male	Three y/o	Treatment (Wk 2)	Treatment	Feed Day	No	17:45	17:00 - 17:59	Evening	37,5	128	135,0	135,0	18,65	611,22	166,33
2019/05/23	CH2205	Male	Three y/o	Treatment (Wk 2)	Treatment	Feed Day	No	17:50	17:00 - 17:59	Evening	37,7		41,0	41,0	18,85		124,94

2019/05/23	CH2206	Male	Three y/o	Treatment (Wk 2)	Treatment	Feed Day	No	17:50	17:00 - 17:59	Evening	37,5	43	40,4	40,4	18,65	526,50	124,43
2019/05/23	CH2205	Male	Three y/o	Treatment (Wk 2)	Treatment	Feed Day	No	17:55	17:00 - 17:59	Evening	37,7	46	27,4	27,4	18,85	532,38	111,09
2019/05/23	CH2206	Male	Three y/o	Treatment (Wk 2)	Treatment	Feed Day	No	17:55	17:00 - 17:59	Evening	37,3	85	63,0	63,0	18,46	581,90	139,78
2019/05/23	CH2205	Male	Three y/o	Treatment (Wk 2)	Treatment	Feed Day	No	18:00	18:00 - 18:59	Evening	37,7	94	35,4	35,4	18,85	589,37	119,88
2019/05/23	CH2206	Male	Three y/o	Treatment (Wk 2)	Treatment	Feed Day	No	18:00	18:00 - 18:59	Evening	37,4	125	205,4	205,4	18,55	609,60	181,06
2019/05/23	CH2205	Male	Three y/o	Treatment (Wk 2)	Treatment	Feed Day	No	18:05	18:00 - 18:59	Evening	37,7		34,4	34,4	18,85		118,90
2019/05/23	CH2206	Male	Three y/o	Treatment (Wk 2)	Treatment	Feed Day	No	18:05	18:00 - 18:59	Evening	37,4	118	93,2	93,2	18,55	605,61	153,39
2019/05/23	CH2205	Male	Three y/o	Treatment (Wk 2)	Treatment	Feed Day	No	18:10	18:00 - 18:59	Evening	37,7	45	28,6	28,6	18,85	530,47	112,56
2019/05/23	CH2206	Male	Three y/o	Treatment (Wk 2)	Treatment	Feed Day	No	18:10	18:00 - 18:59	Evening	37,4	94	98,4	98,4	18,55	589,37	155,28
2019/05/23	CH2205	Male	Three y/o	Treatment (Wk 2)	Treatment	Feed Day	No	18:15	18:00 - 18:59	Evening	37,6	81	34,8	34,8	18,75	578,26	119,29
2019/05/23	CH2206	Male	Three y/o	Treatment (Wk 2)	Treatment	Feed Day	No	18:15	18:00 - 18:59	Evening	37,5	113	71,0	71,0	18,65	602,58	143,93
2019/05/23	CH2205	Male	Three y/o	Treatment (Wk 2)	Treatment	Feed Day	No	18:20	18:00 - 18:59	Evening	37,5	46	20,2	20,2	18,65	532,38	100,65
2019/05/23	CH2206	Male	Three y/o	Treatment (Wk 2)	Treatment	Feed Day	No	18:20	18:00 - 18:59	Evening	37,1	68	53,6	53,6	18,26	564,71	134,19
2019/05/23	CH2205	Male	Three y/o	Treatment (Wk 2)	Treatment	Feed Day	No	18:25	18:00 - 18:59	Evening	37,4	65	25,4	25,4	18,55	561,12	108,49
2019/05/23	CH2206	Male	Three y/o	Treatment (Wk 2)	Treatment	Feed Day	No	18:25	18:00 - 18:59	Evening	36,5	87	60,4	60,4	17,67	583,64	138,32
2019/05/23	CH2205	Male	Three y/o	Treatment (Wk 2)	Treatment	Feed Day	No	18:30	18:00 - 18:59	Evening	37,3	67	30,6	30,6	18,46	563,53	114,87
2019/05/23	CH2206	Male	Three y/o	Treatment (Wk 2)	Treatment	Feed Day	No	18:30	18:00 - 18:59	Evening	36,6	79	51,2	51,2	17,77	576,36	132,61
2019/05/23	CH2205	Male	Three y/o	Treatment (Wk 2)	Treatment	Feed Day	No	18:35	18:00 - 18:59	Evening	37,2	73	34,2	34,2	18,36	570,27	118,70
2019/05/23	CH2206	Male	Three y/o	Treatment (Wk 2)	Treatment	Feed Day	No	18:35	18:00 - 18:59	Evening	37,1	63	44,8	44,8	18,26	558,62	127,99
2019/05/23	CH2205	Male	Three y/o	Treatment (Wk 2)	Treatment	Feed Day	No	18:40	18:00 - 18:59	Evening	37,1	49	31,0	31,0	18,26	537,80	115,32
2019/05/23	CH2206	Male	Three y/o	Treatment (Wk 2)	Treatment	Feed Day	No	18:40	18:00 - 18:59	Evening	37,3	55	47,4	47,4	18,46	547,52	129,94
2019/05/23	CH2205	Male	Three y/o	Treatment (Wk 2)	Treatment	Feed Day	No	18:45	18:00 - 18:59	Evening	37,1	101	21,4	21,4	18,26	594,59	102,63
2019/05/23	CH2206	Male	Three y/o	Treatment (Wk 2)	Treatment	Feed Day	No	18:45	18:00 - 18:59	Evening	37,5	86	52,6	52,6	18,65	582,77	133,54
2019/05/23	CH2205	Male	Three y/o	Treatment (Wk 2)	Treatment	Feed Day	No	18:50	18:00 - 18:59	Evening	37,5	87	11,0	11,0	18,65	583,64	79,98
2019/05/23	CH2206	Male	Three y/o	Treatment (Wk 2)	Treatment	Feed Day	No	18:50	18:00 - 18:59	Evening	37,5	89	59,0	59,0	18,65	585,33	137,51
2019/05/23	CH2205	Male	Three y/o	Treatment (Wk 2)	Treatment	Feed Day	No	18:55	18:00 - 18:59	Evening	37,5	58	7,6	7,6	18,65	551,90	67,49
2019/05/23	CH2206	Male	Three y/o	Treatment (Wk 2)	Treatment	Feed Day	No	18:55	18:00 - 18:59	Evening	37,7	45	64,4	64,4	18,85	530,47	140,55
2019/05/23	CH2205	Male	Three y/o	Treatment (Wk 2)	Treatment	Feed Day	No	19:00	19:00 - 19:59	Evening	37,5	54	7,2	7,2	18,65	545,99	65,66
2019/05/23	CH2206	Male	Three y/o	Treatment (Wk 2)	Treatment	Feed Day	No	19:00	19:00 - 19:59	Evening	37,7	86	50,8	50,8	18,85	582,77	132,33
2019/05/23	CH2205	Male	Three y/o	Treatment (Wk 2)	Treatment	Feed Day	No	19:05	19:00 - 19:59	Evening	37,3	92	46,0	46,0	18,46	587,78	128,91
2019/05/23	CH2206	Male	Three y/o	Treatment (Wk 2)	Treatment	Feed Day	No	19:05	19:00 - 19:59	Evening	37,7	86	60,8	60,8	18,85	582,77	138,55
2019/05/23	CH2205	Male	Three y/o	Treatment (Wk 2)	Treatment	Feed Day	No	19:10	19:00 - 19:59	Evening	37,9	90	24,6	24,6	19,05	586,16	107,39
2019/05/23	CH2206	Male	Three y/o	Treatment (Wk 2)	Treatment	Feed Day	No	19:10	19:00 - 19:59	Evening	37,5	92	63,6	63,6	18,65	587,78	140,11
2019/05/23	CH2205	Male	Three y/o	Treatment (Wk 2)	Treatment	Feed Day	No	19:15	19:00 - 19:59	Evening	37,9		30,0	30,0	19,05		114,20
2019/05/23	CH2206	Male	Three y/o	Treatment (Wk 2)	Treatment	Feed Day	No	19:15	19:00 - 19:59	Evening	37,3	91	59,4	59,4	18,46	586,98	137,75
2019/05/23	CH2205	Male	Three y/o	Treatment (Wk 2)	Treatment	Feed Day	No	19:20	19:00 - 19:59	Evening	37,7		28,4	28,4	18,85		112,31
2019/05/23	CH2206	Male	Three y/o	Treatment (Wk 2)	Treatment	Feed Day	No	19:20	19:00 - 19:59	Evening	37,0	83	63,8	63,8	18,16	580,11	140,22
2019/05/23	CH2205	Male	Three y/o	Treatment (Wk 2)	Treatment	Feed Day	No	19:25	19:00 - 19:59	Evening	37,7	68	26,4	26,4	18,85	564,71	109,81
2019/05/23	CH2206	Male	Three y/o	Treatment (Wk 2)	Treatment	Feed Day	No	19:25	19:00 - 19:59	Evening	36,6	44	57,8	57,8	17,77	528,51	136,80
2019/05/23	CH2205	Male	Three y/o	Treatment (Wk 2)	Treatment	Feed Day	No	19:30	19:00 - 19:59	Evening	37,7	84	23,4	23,4	18,85	581,01	105,68
2019/05/23	CH2206	Male	Three y/o	Treatment (Wk 2)	Treatment	Feed Day	No	19:30	19:00 - 19:59	Evening	36,3	84	61,4	61,4	17,48	581,01	138,89
2019/05/23	CH2205	Male	Three y/o	Treatment (Wk 2)	Treatment	Feed Day	No	19:35	19:00 - 19:59	Evening	37,6	47	22,4	22,4	18,75	534,23	104,19
2019/05/23	CH2206	Male	Three y/o	Treatment (Wk 2)	Treatment	Feed Day	No	19:35	19:00 - 19:59	Evening	36,1		54,0	54,0	17,29		134,45
2019/05/23	CH2205	Male	Three y/o	Treatment (Wk 2)	Treatment	Feed Day	No	19:40	19:00 - 19:59	Evening	37,6	71	26,2	26,2	18,75	568,10	109,55
2019/05/23	CH2206	Male	Three y/o	Treatment (Wk 2)	Treatment	Feed Day	No	19:40	19:00 - 19:59	Evening	35,9	46	63,0	63,0	17,09	532,38	139,78
2019/05/23	CH2205	Male	Three y/o	Treatment (Wk 2)	Treatment	Feed Day	No	19:45	19:00 - 19:59	Evening	37,6	45	22,4	22,4	18,75	530,47	104,19
2019/05/23	CH2206	Male	Three y/o	Treatment (Wk 2)	Treatment	Feed Day	No	19:45	19:00 - 19:59	Evening	35,6	84	60,0	60,0	16,81	581,01	138,09
2019/05/23	CH2205	Male	Three y/o	Treatment (Wk 2)	Treatment	Feed Day	No	19:50	19:00 - 19:59	Evening	37,7	72	20,8	20,8	18,85	569,20	101,65
2019/05/23	CH2206	Male	Three y/o	Treatment (Wk 2)	Treatment	Feed Day	No	19:50	19:00 - 19:59	Evening	35,4	83	49,6	49,6	16,62	580,11	131,51
2019/05/23	CH2205	Male	Three y/o	Treatment (Wk 2)	Treatment	Feed Day	No	19:55	19:00 - 19:59	Evening	37,6	84	30,6	30,6	18,75	581,01	114,87
2019/05/23	CH2206	Male	Three y/o	Treatment (Wk 2)	Treatment	Feed Day	No	19:55	19:00 - 19:59	Evening	35,3	45	46,2	46,2	16,53	530,47	129,06
2019/05/23	CH2205	Male	Three y/o	Treatment (Wk 2)	Treatment	Feed Day	No	20:00	20:00 - 20:59	Night	37,6	85	25,8	25,8	18,75	581,90	109,02
2019/05/23	CH2206	Male	Three y/o	Treatment (Wk 2)	Treatment	Feed Day	No	20:00	20:00 - 20:59	Night	35,4	88	51,4	51,4	16,62	584,49	132,74
2019/05/23	CH2205	Male	Three y/o	Treatment (Wk 2)	Treatment	Feed Day	No	20:05	20:00 - 20:59	Night	37,5	90	26,2	26,2	18,65	586,16	109,55
2019/05/23	CH2206	Male	Three y/o	Treatment (Wk 2)	Treatment	Feed Day	No	20:05	20:00 - 20:59	Night	35,4	84	49,8	49,8	16,62	581,01	131,65
2019/05/23	CH2205	Male	Three y/o	Treatment (Wk 2)	Treatment	Feed Day	No	20:10	20:00 - 20:59	Night	37,5	49	22,0	22,0	18,65	537,80	103,57
2019/05/23	CH2206	Male	Three y/o	Treatment (Wk 2)	Treatment	Feed Day	No	20:10	20:00 - 20:59	Night	35,5	90	49,0	49,0	16,72	586,16	131,09
2019/05/23	CH2205	Male	Three y/o	Treatment (Wk 2)	Treatment	Feed Day	No	20:15	20:00 - 20:59	Night	37,5	60	20,6	20,6	18,65	554,67	101,32
2019/05/23	CH2206	Male	Three y/o	Treatment (Wk 2)	Treatment	Feed Day	No	20:15	20:00 - 20:59	Night	35,6	92	103,8	103,8	16,81	587,78	157,14
2019/05/23	CH2205	Male	Three y/o	Treatment (Wk 2)	Treatment	Feed Day	No	20:20	20:00 - 20:59	Night	37,6	102	73,2	73,2	18,75	595,30	144,99
2019/05/23	CH2206	Male	Three y/o	Treatment (Wk 2)	Treatment	Feed Day	No	20:20	20:00 - 20:59	Night	36,3	65	48,4	48,4	17,48	561,12	130,66
2019/05/23	CH2205	Male	Three y/o	Treatment (Wk 2)	Treatment	Feed Day	No	20:25	20:00 - 20:59	Night	37,5	150	20,6	20,6	18,65	621,86	101,32
2019/05/23	CH2206	Male	Three y/o	Treatment (Wk 2)	Treatment	Feed Day	No	20:25	20:00 - 20:59	Night	36,3	47	51,0	51,0	17,48	534,23	132,47
2019/05/23	CH2205	Male	Three y/o	Treatment (Wk 2)	Treatment	Feed Day	No	20:30	20:00 - 20:59	Night	37,6		15,6	15,6	18,75		91,85
2019/05/23	CH2206	Male	Three y/o	Treatment (Wk 2)	Treatment	Feed Day	No	20:30	20:00 - 20:59	Night	36,5	44	47,4	47,4	17,67	528,51	129,94
2019/05/23	CH2205	Male	Three y/o	Treatment (Wk 2)	Treatment	Feed Day	No	20:35	20:00 - 20:59	Night	37,6	94	6,6	6,6	18,75	589,37	62,73
2019/05/23	CH2206	Male	Three y/o	Treatment (Wk 2)	Treatment	Feed Day	No	20:35	20:00 - 20:59	Night	36,8	90	45,6	45,6	17,96	586,16	128,61
2019/05/23	CH2205	Male	Three y/o	Treatment (Wk 2)	Treatment	Feed Day	No	20:40	20:00 - 20:59	Night	37,5	84	2,2	2,2	18,65	581,01	26,02
2019/05/23	CH2206	Male	Three y/o	Treatment (Wk 2)	Treatment	Feed Day	No	20:40	20:00 - 20:59	Night	36,9	83	41,0	41,0	18,06	580,11	124,94
2019/05/23	CH2205	Male	Three y/o	Treatment (Wk 2)	Treatment	Feed Day	No	20:45	20:00 - 20:59	Night	37,4	82	4,2	4,2	18,55	579,19	47,57

2019/05/23	CH2206	Male	Three y/o	Treatment (Wk 2)	Treatment	Feed Day	No	20:45	20:00 - 20:59	Night	37,0	94	37,8	37,8	18,16	589,37	122,14
2019/05/23	CH2205	Male	Three y/o	Treatment (Wk 2)	Treatment	Feed Day	No	20:50	20:00 - 20:59	Night	37,4	55	5,2	5,2	18,55	547,52	54,72
2019/05/23	CH2206	Male	Three y/o	Treatment (Wk 2)	Treatment	Feed Day	No	20:50	20:00 - 20:59	Night	37,1	63	34,8	34,8	18,26	558,62	119,29
2019/05/23	CH2205	Male	Three y/o	Treatment (Wk 2)	Treatment	Feed Day	No	20:55	20:00 - 20:59	Night	37,5	60	5,0	5,0	18,65	554,67	53,41
2019/05/23	CH2206	Male	Three y/o	Treatment (Wk 2)	Treatment	Feed Day	No	20:55	20:00 - 20:59	Night	37,2	48	39,8	39,8	18,36	536,04	123,91
2019/05/23	CH2205	Male	Three y/o	Treatment (Wk 2)	Treatment	Feed Day	No	21:00	21:00 - 21:59	Night	37,5	62	6,4	6,4	18,65	557,33	61,70
2019/05/23	CH2206	Male	Three y/o	Treatment (Wk 2)	Treatment	Feed Day	No	21:00	21:00 - 21:59	Night	37,2	92	38,8	38,8	18,36	587,78	123,04
2019/05/23	CH2205	Male	Three y/o	Treatment (Wk 2)	Treatment	Feed Day	No	21:05	21:00 - 21:59	Night	37,6	92	9,0	9,0	18,75	587,78	73,19
2019/05/23	CH2206	Male	Three y/o	Treatment (Wk 2)	Treatment	Feed Day	No	21:05	21:00 - 21:59	Night	37,3	43	25,4	25,4	18,46	526,50	108,49
2019/05/23	CH2205	Male	Three y/o	Treatment (Wk 2)	Treatment	Feed Day	No	21:10	21:00 - 21:59	Night	37,6	55	4,4	4,4	18,75	547,52	49,12
2019/05/23	CH2206	Male	Three y/o	Treatment (Wk 2)	Treatment	Feed Day	No	21:10	21:00 - 21:59	Night	37,4	82	31,4	31,4	18,55	579,19	115,76
2019/05/23	CH2205	Male	Three y/o	Treatment (Wk 2)	Treatment	Feed Day	No	21:15	21:00 - 21:59	Night	37,6	79	5,2	5,2	18,75	576,36	54,72
2019/05/23	CH2206	Male	Three y/o	Treatment (Wk 2)	Treatment	Feed Day	No	21:15	21:00 - 21:59	Night	37,7	86	42,4	42,4	18,85	582,77	126,10
2019/05/23	CH2205	Male	Three y/o	Treatment (Wk 2)	Treatment	Feed Day	No	21:20	21:00 - 21:59	Night	37,7	48	4,8	4,8	18,85	536,04	52,04
2019/05/23	CH2206	Male	Three y/o	Treatment (Wk 2)	Treatment	Feed Day	No	21:20	21:00 - 21:59	Night	37,7	79	37,0	37,0	18,85	576,36	121,40
2019/05/23	CH2205	Male	Three y/o	Treatment (Wk 2)	Treatment	Feed Day	No	21:25	21:00 - 21:59	Night	37,7	83	7,2	7,2	18,85	580,11	65,66
2019/05/23	CH2206	Male	Three y/o	Treatment (Wk 2)	Treatment	Feed Day	No	21:25	21:00 - 21:59	Night	37,8	76	35,2	35,2	18,95	573,39	119,69
2019/05/23	CH2205	Male	Three y/o	Treatment (Wk 2)	Treatment	Feed Day	No	21:30	21:00 - 21:59	Night	37,7	55	8,8	8,8	18,85	547,52	72,43
2019/05/23	CH2206	Male	Three y/o	Treatment (Wk 2)	Treatment	Feed Day	No	21:30	21:00 - 21:59	Night	37,8	44	40,6	40,6	18,95	528,51	124,60
2019/05/23	CH2205	Male	Three y/o	Treatment (Wk 2)	Treatment	Feed Day	No	21:35	21:00 - 21:59	Night	37,7		6,0	6,0	18,85		59,53
2019/05/23	CH2206	Male	Three y/o	Treatment (Wk 2)	Treatment	Feed Day	No	21:35	21:00 - 21:59	Night	37,8	67	37,8	37,8	18,95	563,53	122,14
2019/05/23	CH2205	Male	Three y/o	Treatment (Wk 2)	Treatment	Feed Day	No	21:40	21:00 - 21:59	Night	37,7	50	2,4	2,4	18,85	539,52	28,91
2019/05/23	CH2206	Male	Three y/o	Treatment (Wk 2)	Treatment	Feed Day	No	21:40	21:00 - 21:59	Night	37,8	90	41,6	41,6	18,95	586,16	125,44
2019/05/23	CH2205	Male	Three y/o	Treatment (Wk 2)	Treatment	Feed Day	No	21:45	21:00 - 21:59	Night	37,7	95	6,8	6,8	18,85	590,14	63,74
2019/05/23	CH2206	Male	Three y/o	Treatment (Wk 2)	Treatment	Feed Day	No	21:45	21:00 - 21:59	Night	37,9	71	40,2	40,2	19,05	568,10	124,26
2019/05/23	CH2205	Male	Three y/o	Treatment (Wk 2)	Treatment	Feed Day	No	21:50	21:00 - 21:59	Night	37,7	59	5,6	5,6	18,85	553,30	57,21
2019/05/23	CH2206	Male	Three y/o	Treatment (Wk 2)	Treatment	Feed Day	No	21:50	21:00 - 21:59	Night	37,9	75	39,2	39,2	19,05	572,36	123,39
2019/05/23	CH2205	Male	Three y/o	Treatment (Wk 2)	Treatment	Feed Day	No	21:55	21:00 - 21:59	Night	37,7	83	7,8	7,8	18,85	580,11	68,36
2019/05/23	CH2206	Male	Three y/o	Treatment (Wk 2)	Treatment	Feed Day	No	21:55	21:00 - 21:59	Night	37,9	73	27,0	27,0	19,05	570,27	110,58
2019/05/23	CH2205	Male	Three y/o	Treatment (Wk 2)	Treatment	Feed Day	No	22:00	22:00 - 22:59	Night	37,7	51	2,4	2,4	18,85	541,20	28,91
2019/05/23	CH2206	Male	Three y/o	Treatment (Wk 2)	Treatment	Feed Day	No	22:00	22:00 - 22:59	Night	37,9	126	1,6	1,6	19,05	610,14	15,48
2019/05/23	CH2205	Male	Three y/o	Treatment (Wk 2)	Treatment	Feed Day	No	22:05	22:00 - 22:59	Night	37,9	83	7,2	7,2	19,05	580,11	65,66
2019/05/23	CH2206	Male	Three y/o	Treatment (Wk 2)	Treatment	Feed Day	No	22:05	22:00 - 22:59	Night	37,8	66	5,4	5,4	18,95	562,34	55,99
2019/05/23	CH2205	Male	Three y/o	Treatment (Wk 2)	Treatment	Feed Day	No	22:10	22:00 - 22:59	Night	38,0	54	10,0	10,0	19,15	545,99	76,76
2019/05/23	CH2206	Male	Three y/o	Treatment (Wk 2)	Treatment	Feed Day	No	22:10	22:00 - 22:59	Night	37,8	93	17,2	17,2	18,95	588,58	95,17
2019/05/23	CH2205	Male	Three y/o	Treatment (Wk 2)	Treatment	Feed Day	No	22:15	22:00 - 22:59	Night	38,0	84	25,6	25,6	19,15	581,01	108,76
2019/05/23	CH2206	Male	Three y/o	Treatment (Wk 2)	Treatment	Feed Day	No	22:15	22:00 - 22:59	Night	37,8	86	47,0	47,0	18,95	582,77	129,65
2019/05/23	CH2205	Male	Three y/o	Treatment (Wk 2)	Treatment	Feed Day	No	22:20	22:00 - 22:59	Night	38,0	50	22,6	22,6	19,15	539,52	104,49
2019/05/23	CH2206	Male	Three y/o	Treatment (Wk 2)	Treatment	Feed Day	No	22:20	22:00 - 22:59	Night	37,8	56	46,2	46,2	18,95	549,01	129,06
2019/05/23	CH2205	Male	Three y/o	Treatment (Wk 2)	Treatment	Feed Day	No	22:25	22:00 - 22:59	Night	38,0	56	19,4	19,4	19,15	549,01	99,28
2019/05/23	CH2206	Male	Three y/o	Treatment (Wk 2)	Treatment	Feed Day	No	22:25	22:00 - 22:59	Night	37,8	55	39,8	39,8	18,95	547,52	123,91
2019/05/23	CH2205	Male	Three y/o	Treatment (Wk 2)	Treatment	Feed Day	No	22:30	22:00 - 22:59	Night	38,0	48	25,6	25,6	19,15	536,04	108,76
2019/05/23	CH2206	Male	Three y/o	Treatment (Wk 2)	Treatment	Feed Day	No	22:30	22:00 - 22:59	Night	37,8	40	40,4	40,4	18,95	520,09	124,43
2019/05/23	CH2205	Male	Three y/o	Treatment (Wk 2)	Treatment	Feed Day	No	22:35	22:00 - 22:59	Night	38,0	54	28,4	28,4	19,15	545,99	112,31
2019/05/23	CH2206	Male	Three y/o	Treatment (Wk 2)	Treatment	Feed Day	No	22:35	22:00 - 22:59	Night	37,8	65	40,8	40,8	18,95	561,12	124,77
2019/05/23	CH2205	Male	Three y/o	Treatment (Wk 2)	Treatment	Feed Day	No	22:40	22:00 - 22:59	Night	37,9		26,4	26,4	19,05		109,81
2019/05/23	CH2206	Male	Three y/o	Treatment (Wk 2)	Treatment	Feed Day	No	22:40	22:00 - 22:59	Night	37,8	49	29,6	29,6	18,95	537,80	113,73
2019/05/23	CH2205	Male	Three y/o	Treatment (Wk 2)	Treatment	Feed Day	No	22:45	22:00 - 22:59	Night	37,9	87	20,0	20,0	19,05	583,64	100,31
2019/05/23	CH2206	Male	Three y/o	Treatment (Wk 2)	Treatment	Feed Day	No	22:45	22:00 - 22:59	Night	37,9	40	9,2	9,2	19,05	520,09	73,94
2019/05/23	CH2205	Male	Three y/o	Treatment (Wk 2)	Treatment	Feed Day	No	22:50	22:00 - 22:59	Night	37,7	94	20,4	20,4	18,85	589,37	100,99
2019/05/23	CH2206	Male	Three y/o	Treatment (Wk 2)	Treatment	Feed Day	No	22:50	22:00 - 22:59	Night	37,9	58	11,6	11,6	19,05	551,90	81,78
2019/05/23	CH2205	Male	Three y/o	Treatment (Wk 2)	Treatment	Feed Day	No	22:55	22:00 - 22:59	Night	37,7	52	24,8	24,8	18,85	542,83	107,67
2019/05/23	CH2206	Male	Three y/o	Treatment (Wk 2)	Treatment	Feed Day	No	22:55	22:00 - 22:59	Night	38,0	58	13,4	13,4	19,15	551,90	86,68
2019/05/23	CH2205	Male	Three y/o	Treatment (Wk 2)	Treatment	Feed Day	No	23:00	23:00 - 23:59	Night	37,7	86	21,4	21,4	18,85	582,77	102,63
2019/05/23	CH2206	Male	Three y/o	Treatment (Wk 2)	Treatment	Feed Day	No	23:00	23:00 - 23:59	Night	38,0	99	14,8	14,8	19,15	593,14	90,05
2019/05/23	CH2205	Male	Three y/o	Treatment (Wk 2)	Treatment	Feed Day	No	23:05	23:00 - 23:59	Night	37,6	59	21,2	21,2	18,75	553,30	102,31
2019/05/23	CH2206	Male	Three y/o	Treatment (Wk 2)	Treatment	Feed Day	No	23:05	23:00 - 23:59	Night	38,0	46	17,6	17,6	19,15	532,38	95,95
2019/05/23	CH2205	Male	Three y/o	Treatment (Wk 2)	Treatment	Feed Day	No	23:10	23:00 - 23:59	Night	37,7	47	21,4	21,4	18,85	534,23	102,63
2019/05/23	CH2206	Male	Three y/o	Treatment (Wk 2)	Treatment	Feed Day	No	23:10	23:00 - 23:59	Night	38,0	52	19,4	19,4	19,15	542,83	99,28
2019/05/23	CH2205	Male	Three y/o	Treatment (Wk 2)	Treatment	Feed Day	No	23:15	23:00 - 23:59	Night	37,7	92	26,4	26,4	18,85	587,78	109,81
2019/05/23	CH2206	Male	Three y/o	Treatment (Wk 2)	Treatment	Feed Day	No	23:15	23:00 - 23:59	Night	38,0	43	21,6	21,6	19,15	526,50	102,94
2019/05/23	CH2205	Male	Three y/o	Treatment (Wk 2)	Treatment	Feed Day	No	23:20	23:00 - 23:59	Night	37,7	55	24,0	24,0	18,85	547,52	106,55
2019/05/23	CH2206	Male	Three y/o	Treatment (Wk 2)	Treatment	Feed Day	No	23:20	23:00 - 23:59	Night	38,0	53	13,6	13,6	19,15	544,43	87,18
2019/05/23	CH2205	Male	Three y/o	Treatment (Wk 2)	Treatment	Feed Day	No	23:25	23:00 - 23:59	Night	37,7	55	28,8	28,8	18,85	547,52	112,79
2019/05/23	CH2206	Male	Three y/o	Treatment (Wk 2)	Treatment	Feed Day	No	23:25	23:00 - 23:59	Night	38,0		6,2	6,2	19,15		60,63
2019/05/23	CH2205	Male	Three y/o	Treatment (Wk 2)	Treatment	Feed Day	No	23:30	23:00 - 23:59	Night	37,7	85	27,0	27,0	18,85	581,90	110,58
2019/05/23	CH2206	Male	Three y/o	Treatment (Wk 2)	Treatment	Feed Day	No	23:30	23:00 - 23:59	Night	38,0	65	6,0	6,0	19,15	561,12	59,53
2019/05/23	CH2205	Male	Three y/o	Treatment (Wk 2)	Treatment	Feed Day	No	23:35	23:00 - 23:59	Night	37,8	60	26,4	26,4	18,95	554,67	109,81
2019/05/23	CH2206	Male	Three y/o	Treatment (Wk 2)	Treatment	Feed Day	No	23:35	23:00 - 23:59	Night	38,0	73	5,2	5,2	19,15	570,27	54,72
2019/05/23	CH2205	Male	Three y/o	Treatment (Wk 2)	Treatment	Feed Day	No	23:40	23:00 - 23:59	Night	37,8	79	29,0	29,0	18,95	576,36	113,03

	2019/05/23	CH2206	Male	Three y/o	Treatment (Wk 2)	Treatment	Feed Day	No	23:40	23:00 - 23:59	Night	38,0	60	4,8	4,8	19,15	554,67	52,04
	2019/05/23	CH2205	Male	Three y/o	Treatment (Wk 2)	Treatment	Feed Day	No	23:45	23:00 - 23:59	Night	37,9	77	28,2	28,2	19,05	574,39	112,07
	2019/05/23	CH2206	Male	Three y/o	Treatment (Wk 2)	Treatment	Feed Day	No	23:45	23:00 - 23:59	Night	38,0	69	4,4	4,4	19,15	565,86	49,12
	2019/05/23	CH2205	Male	Three y/o	Treatment (Wk 2)	Treatment	Feed Day	No	23:50	23:00 - 23:59	Night	37,8	59	22,2	22,2	18,95	553,30	103,88
	2019/05/23	CH2206	Male	Three y/o	Treatment (Wk 2)	Treatment	Feed Day	No	23:50	23:00 - 23:59	Night	38,0	75	1,8	1,8	19,15	572,36	19,38
	2019/05/23	CH2205	Male	Three y/o	Treatment (Wk 2)	Treatment	Feed Day	No	23:55	23:00 - 23:59	Night	37,7	96	12,0	12,0	18,85	590,91	82,93
	2019/05/23	CH2206	Male	Three y/o	Treatment (Wk 2)	Treatment	Feed Day	No	23:55	23:00 - 23:59	Night	38,0	59	10,2	10,2	19,15	553,30	77,43
10	2019/05/24	CH2205	Male	Three y/o	Treatment (Wk 2)	Treatment	Feed Day	No	00:00	00:00 - 00:59	Early Morning	37,7	85	7,2	7,2	18,85	581,90	65,66
	2019/05/24	CH2206	Male	Three y/o	Treatment (Wk 2)	Treatment	Feed Day	No	00:00	00:00 - 00:59	Early Morning	37,9	74	7,6	7,6	19,05	571,33	67,49
	2019/05/24	CH2205	Male	Three y/o	Treatment (Wk 2)	Treatment	Feed Day	No	00:05	00:00 - 00:59	Early Morning	37,6	94	6,0	6,0	18,75	589,37	59,53
	2019/05/24	CH2206	Male	Three y/o	Treatment (Wk 2)	Treatment	Feed Day	No	00:05	00:00 - 00:59	Early Morning	37,9	51	11,2	11,2	19,05	541,20	80,59
	2019/05/24	CH2205	Male	Three y/o	Treatment (Wk 2)	Treatment	Feed Day	No	00:10	00:00 - 00:59	Early Morning	37,6	82	11,2	11,2	18,75	579,19	80,59
	2019/05/24	CH2206	Male	Three y/o	Treatment (Wk 2)	Treatment	Feed Day	No	00:10	00:00 - 00:59	Early Morning	37,9	56	43,4	43,4	19,05	549,01	126,90
	2019/05/24	CH2205	Male	Three y/o	Treatment (Wk 2)	Treatment	Feed Day	No	00:15	00:00 - 00:59	Early Morning	37,6	53	8,0	8,0	18,75	544,43	69,22
	2019/05/24	CH2206	Male	Three y/o	Treatment (Wk 2)	Treatment	Feed Day	No	00:15	00:00 - 00:59	Early Morning	37,9	82	53,8	53,8	19,05	579,19	134,32
	2019/05/24	CH2205	Male	Three y/o	Treatment (Wk 2)	Treatment	Feed Day	No	00:20	00:00 - 00:59	Early Morning	37,6	49	13,0	13,0	18,75	537,80	85,65
	2019/05/24	CH2206	Male	Three y/o	Treatment (Wk 2)	Treatment	Feed Day	No	00:20	00:00 - 00:59	Early Morning	37,8	54	59,6	59,6	18,95	545,99	137,86
	2019/05/24	CH2205	Male	Three y/o	Treatment (Wk 2)	Treatment	Feed Day	No	00:25	00:00 - 00:59	Early Morning	37,6	37	14,2	14,2	18,75	513,06	88,65
	2019/05/24	CH2206	Male	Three y/o	Treatment (Wk 2)	Treatment	Feed Day	No	00:25	00:00 - 00:59	Early Morning	37,8	56	57,0	57,0	18,95	549,01	136,32
	2019/05/24	CH2205	Male	Three y/o	Treatment (Wk 2)	Treatment	Feed Day	No	00:30	00:00 - 00:59	Early Morning	37,6	90	15,8	15,8	18,75	586,16	92,28
	2019/05/24	CH2206	Male	Three y/o	Treatment (Wk 2)	Treatment	Feed Day	No	00:30	00:00 - 00:59	Early Morning	37,8	47	58,2	58,2	18,95	534,23	137,04
	2019/05/24	CH2205	Male	Three y/o	Treatment (Wk 2)	Treatment	Feed Day	No	00:35	00:00 - 00:59	Early Morning	37,6	107	10,8	10,8	18,75	598,73	79,36
	2019/05/24	CH2206	Male	Three y/o	Treatment (Wk 2)	Treatment	Feed Day	No	00:35	00:00 - 00:59	Early Morning	37,8	101	56,0	56,0	18,95	594,59	135,70
	2019/05/24	CH2205	Male	Three y/o	Treatment (Wk 2)	Treatment	Feed Day	No	00:40	00:00 - 00:59	Early Morning	37,6	88	14,4	14,4	18,75	584,49	89,12
	2019/05/24	CH2206	Male	Three y/o	Treatment (Wk 2)	Treatment	Feed Day	No	00:40	00:00 - 00:59	Early Morning	37,8	64	56,8	56,8	18,95	559,88	136,20
	2019/05/24	CH2205	Male	Three y/o	Treatment (Wk 2)	Treatment	Feed Day	No	00:45	00:00 - 00:59	Early Morning	37,6	52	14,8	14,8	18,75	542,83	90,05
	2019/05/24	CH2206	Male	Three y/o	Treatment (Wk 2)	Treatment	Feed Day	No	00:45	00:00 - 00:59	Early Morning	37,8	84	57,4	57,4	18,95	581,01	136,56
	2019/05/24	CH2205	Male	Three y/o	Treatment (Wk 2)	Treatment	Feed Day	No	00:50	00:00 - 00:59	Early Morning	37,6	79	11,6	11,6	18,75	576,36	81,78
	2019/05/24	CH2206	Male	Three y/o	Treatment (Wk 2)	Treatment	Feed Day	No	00:50	00:00 - 00:59	Early Morning	37,8	76	56,8	56,8	18,95	573,39	136,20
	2019/05/24	CH2205	Male	Three y/o	Treatment (Wk 2)	Treatment	Feed Day	No	00:55	00:00 - 00:59	Early Morning	37,6	78	12,6	12,6	18,75	575,38	84,59
	2019/05/24	CH2206	Male	Three y/o	Treatment (Wk 2)	Treatment	Feed Day	No	00:55	00:00 - 00:59	Early Morning	37,8	79	50,4	50,4	18,95	576,36	132,06
	2019/05/24	CH2205	Male	Three y/o	Treatment (Wk 2)	Treatment	Feed Day	No	01:00	01:00 - 01:59	Early Morning	37,6	49	10,6	10,6	18,75	537,80	78,73
	2019/05/24	CH2206	Male	Three y/o	Treatment (Wk 2)	Treatment	Feed Day	No	01:00	01:00 - 01:59	Early Morning	37,7	63	51,6	51,6	18,85	558,62	132,87
	2019/05/24	CH2205	Male	Three y/o	Treatment (Wk 2)	Treatment	Feed Day	No	01:05	01:00 - 01:59	Early Morning	37,6	43	11,6	11,6	18,75	526,50	81,78
	2019/05/24	CH2206	Male	Three y/o	Treatment (Wk 2)	Treatment	Feed Day	No	01:05	01:00 - 01:59	Early Morning	37,7	86	55,2	55,2	18,85	582,77	135,21
	2019/05/24	CH2205	Male	Three y/o	Treatment (Wk 2)	Treatment	Feed Day	No	01:10	01:00 - 01:59	Early Morning	37,6	88	10,2	10,2	18,75	584,49	77,43
	2019/05/24	CH2206	Male	Three y/o	Treatment (Wk 2)	Treatment	Feed Day	No	01:10	01:00 - 01:59	Early Morning	37,7	85	54,0	54,0	18,85	581,90	134,45
	2019/05/24	CH2205	Male	Three y/o	Treatment (Wk 2)	Treatment	Feed Day	No	01:15	01:00 - 01:59	Early Morning	37,6	91	11,0	11,0	18,75	586,98	79,98
	2019/05/24	CH2206	Male	Three y/o	Treatment (Wk 2)	Treatment	Feed Day	No	01:15	01:00 - 01:59	Early Morning	37,7	43	51,8	51,8	18,85	526,50	133,01
	2019/05/24	CH2205	Male	Three y/o	Treatment (Wk 2)	Treatment	Feed Day	No	01:20	01:00 - 01:59	Early Morning	37,6	74	19,4	19,4	18,75	571,33	99,28
	2019/05/24	CH2206	Male	Three y/o	Treatment (Wk 2)	Treatment	Feed Day	No	01:20	01:00 - 01:59	Early Morning	37,7	87	32,2	32,2	18,85	583,64	116,63
	2019/05/24	CH2205	Male	Three y/o	Treatment (Wk 2)	Treatment	Feed Day	No	01:25	01:00 - 01:59	Early Morning	37,6	120	4,8	4,8	18,75	606,78	52,04
	2019/05/24	CH2206	Male	Three y/o	Treatment (Wk 2)	Treatment	Feed Day	No	01:25	01:00 - 01:59	Early Morning	37,7	93	11,0	11,0	18,85	588,58	79,98
	2019/05/24	CH2205	Male	Three y/o	Treatment (Wk 2)	Treatment	Feed Day	No	01:30	01:00 - 01:59	Early Morning	37,5	70	3,2	3,2	18,65	566,99	38,49
	2019/05/24	CH2206	Male	Three y/o	Treatment (Wk 2)	Treatment	Feed Day	No	01:30	01:00 - 01:59	Early Morning	37,5	84	6,6	6,6	18,65	581,01	62,73
	2019/05/24	CH2205	Male	Three y/o	Treatment (Wk 2)	Treatment	Feed Day	No	01:35	01:00 - 01:59	Early Morning	37,5		3,0	3,0	18,65		36,34
	2019/05/24	CH2206	Male	Three y/o	Treatment (Wk 2)	Treatment	Feed Day	No	01:35	01:00 - 01:59	Early Morning	37,4	82	10,0	10,0	18,55	579,19	76,76
	2019/05/24	CH2205	Male	Three y/o	Treatment (Wk 2)	Treatment	Feed Day	No	01:40	01:00 - 01:59	Early Morning	37,5		3,0	3,0	18,65		36,34
	2019/05/24	CH2206	Male	Three y/o	Treatment (Wk 2)	Treatment	Feed Day	No	01:40	01:00 - 01:59	Early Morning	37,3	53	5,6	5,6	18,46	544,43	57,21
	2019/05/24	CH2205	Male	Three y/o	Treatment (Wk 2)	Treatment	Feed Day	No	01:45	01:00 - 01:59	Early Morning	37,5		15,6	15,6	18,65		91,85
	2019/05/24	CH2206	Male	Three y/o	Treatment (Wk 2)	Treatment	Feed Day	No	01:45	01:00 - 01:59	Early Morning	37,3	44	74,0	74,0	18,46	528,51	145,37
	2019/05/24	CH2205	Male	Three y/o	Treatment (Wk 2)	Treatment	Feed Day	No	01:50	01:00 - 01:59	Early Morning	37,5	88	35,4	35,4	18,65	584,49	119,88
	2019/05/24	CH2206	Male	Three y/o	Treatment (Wk 2)	Treatment	Feed Day	No	01:50	01:00 - 01:59	Early Morning	37,4	134	111,2	111,2	18,55	614,33	159,55
	2019/05/24	CH2205	Male	Three y/o	Treatment (Wk 2)	Treatment	Feed Day	No	01:55	01:00 - 01:59	Early Morning	37,5	56	28,6	28,6	18,65	549,01	112,56
	2019/05/24	CH2206	Male	Three y/o	Treatment (Wk 2)	Treatment	Feed Day	No	01:55	01:00 - 01:59	Early Morning	37,4	46	226,0	226,0	18,55	532,38	184,42
	2019/05/24	CH2205	Male	Three y/o	Treatment (Wk 2)	Treatment	Feed Day	No	02:00	02:00 - 02:59	Early Morning	37,4	54	26,8	26,8	18,55	545,99	110,33
	2019/05/24	CH2206	Male	Three y/o	Treatment (Wk 2)	Treatment	Feed Day	No	02:00	02:00 - 02:59	Early Morning	37,4	134	49,8	49,8	18,55	614,33	131,65
	2019/05/24	CH2205	Male	Three y/o	Treatment (Wk 2)	Treatment	Feed Day	No	02:05	02:00 - 02:59	Early Morning	37,4	86	23,8	23,8	18,55	582,77	106,26
	2019/05/24	CH2206	Male	Three y/o	Treatment (Wk 2)	Treatment	Feed Day	No	02:05	02:00 - 02:59	Early Morning	37,4	107	62,2	62,2	18,55	598,73	139,34
	2019/05/24	CH2205	Male	Three y/o	Treatment (Wk 2)	Treatment	Feed Day	No	02:10	02:00 - 02:59	Early Morning	37,5	54	26,2	26,2	18,65	545,99	109,55
	2019/05/24	CH2206	Male	Three y/o	Treatment (Wk 2)	Treatment	Feed Day	No	02:10	02:00 - 02:59	Early Morning	37,1	44	65,0	65,0	18,26	528,51	140,87
	2019/05/24	CH2205	Male	Three y/o	Treatment (Wk 2)	Treatment	Feed Day	No	02:15	02:00 - 02:59	Early Morning	37,5	73	21,4	21,4	18,65	570,27	102,63
	2019/05/24	CH2206	Male	Three y/o	Treatment (Wk 2)	Treatment	Feed Day	No	02:15	02:00 - 02:59	Early Morning	36,9	84	60,2	60,2	18,06	581,01	138,21
	2019/05/24	CH2205	Male	Three y/o	Treatment (Wk 2)	Treatment	Feed Day	No	02:20	02:00 - 02:59	Early Morning	37,5	58	24,0	24,0	18,65	551,90	106,55
	2019/05/24	CH2206	Male	Three y/o	Treatment (Wk 2)	Treatment	Feed Day	No	02:20	02:00 - 02:59	Early Morning	36,9	56	47,8	47,8	18,06	549,01	130,23
	2019/05/24	CH2205	Male	Three y/o	Treatment (Wk 2)	Treatment	Feed Day	No	02:25	02:00 - 02:59	Early Morning	37,5	55	19,6	19,6	18,65	547,52	99,63
	2019/05/24	CH2206	Male	Three y/o	Treatment (Wk 2)	Treatment	Feed Day	No	02:25	02:00 - 02:59	Early Morning	36,9	48	33,4	33,4	18,06	536,04	117,88
	2019/05/24	CH2205	Male	Three y/o	Treatment (Wk 2)	Treatment	Feed Day	No	02:30	02:00 - 02:59	Early Morning	37,5	42	25,2	25,2	18,65	524,42	108,22
	2019/05/24	CH2206	Male	Three y/o	Treatment (Wk 2)	Treatment	Feed Day	No	02:30	02:00 - 02:59	Early Morning	37,0	83	34,6	34,6	18,16	580,11	119,10
	2019/05/24	CH2205	Male	Three y/o	Treatment (Wk 2)	Treatment	Feed Day	No	02:35	02:00 - 02:59	Early Morning	37,5	55	22,4	22,4	18,65	547,52	104,19

2019/05/24	CH2206	Male	Three y/o	Treatment (Wk 2)	Treatment	Feed Day	No	02:35	02:00 - 02:59	Early Morning	37,1	45	29,4	29,4	18,26	530,47	113,50
2019/05/24	CH2205	Male	Three y/o	Treatment (Wk 2)	Treatment	Feed Day	No	02:40	02:00 - 02:59	Early Morning	37,5	54	26,8	26,8	18,65	545,99	110,33
2019/05/24	CH2206	Male	Three y/o	Treatment (Wk 2)	Treatment	Feed Day	No	02:40	02:00 - 02:59	Early Morning	37,2	79	26,8	26,8	18,36	576,36	110,33
2019/05/24	CH2205	Male	Three y/o	Treatment (Wk 2)	Treatment	Feed Day	No	02:45	02:00 - 02:59	Early Morning	37,5	52	15,0	15,0	18,65	542,83	90,51
2019/05/24	CH2206	Male	Three y/o	Treatment (Wk 2)	Treatment	Feed Day	No	02:45	02:00 - 02:59	Early Morning	37,3	78	9,0	9,0	18,46	575,38	73,19
2019/05/24	CH2205	Male	Three y/o	Treatment (Wk 2)	Treatment	Feed Day	No	02:50	02:00 - 02:59	Early Morning	37,5	59	17,8	17,8	18,65	553,30	96,34
2019/05/24	CH2206	Male	Three y/o	Treatment (Wk 2)	Treatment	Feed Day	No	02:50	02:00 - 02:59	Early Morning	37,3	83	2,0	2,0	18,46	580,11	22,86
2019/05/24	CH2205	Male	Three y/o	Treatment (Wk 2)	Treatment	Feed Day	No	02:55	02:00 - 02:59	Early Morning	37,5	83	18,2	18,2	18,65	580,11	97,10
2019/05/24	CH2206	Male	Three y/o	Treatment (Wk 2)	Treatment	Feed Day	No	02:55	02:00 - 02:59	Early Morning	37,3	59	3,4	3,4	18,46	553,30	40,51
2019/05/24	CH2205	Male	Three y/o	Treatment (Wk 2)	Treatment	Feed Day	No	03:00	03:00 - 03:59	Early Morning	37,5	60	13,8	13,8	18,65	554,67	87,68
2019/05/24	CH2206	Male	Three y/o	Treatment (Wk 2)	Treatment	Feed Day	No	03:00	03:00 - 03:59	Early Morning	37,3	42	4,2	4,2	18,46	524,42	47,57
2019/05/24	CH2205	Male	Three y/o	Treatment (Wk 2)	Treatment	Feed Day	No	03:05	03:00 - 03:59	Early Morning	37,5	98	15,4	15,4	18,65	592,41	91,41
2019/05/24	CH2206	Male	Three y/o	Treatment (Wk 2)	Treatment	Feed Day	No	03:05	03:00 - 03:59	Early Morning	37,3	100	6,0	6,0	18,46	593,87	59,53
2019/05/24	CH2205	Male	Three y/o	Treatment (Wk 2)	Treatment	Feed Day	No	03:10	03:00 - 03:59	Early Morning	37,5	84	11,2	11,2	18,65	581,01	80,59
2019/05/24	CH2206	Male	Three y/o	Treatment (Wk 2)	Treatment	Feed Day	No	03:10	03:00 - 03:59	Early Morning	37,3	66	4,2	4,2	18,46	562,34	47,57
2019/05/24	CH2205	Male	Three y/o	Treatment (Wk 2)	Treatment	Feed Day	No	03:15	03:00 - 03:59	Early Morning	37,5	49	9,4	9,4	18,65	537,80	74,66
2019/05/24	CH2206	Male	Three y/o	Treatment (Wk 2)	Treatment	Feed Day	No	03:15	03:00 - 03:59	Early Morning	37,3	48	30,4	30,4	18,46	536,04	114,65
2019/05/24	CH2205	Male	Three y/o	Treatment (Wk 2)	Treatment	Feed Day	No	03:20	03:00 - 03:59	Early Morning	37,6	86	6,6	6,6	18,75	582,77	62,73
2019/05/24	CH2206	Male	Three y/o	Treatment (Wk 2)	Treatment	Feed Day	No	03:20	03:00 - 03:59	Early Morning	37,3	92	66,8	66,8	18,46	587,78	141,81
2019/05/24	CH2205	Male	Three y/o	Treatment (Wk 2)	Treatment	Feed Day	No	03:25	03:00 - 03:59	Early Morning	37,5	84	9,4	9,4	18,65	581,01	74,66
2019/05/24	CH2206	Male	Three y/o	Treatment (Wk 2)	Treatment	Feed Day	No	03:25	03:00 - 03:59	Early Morning	37,3	71	60,8	60,8	18,46	568,10	138,55
2019/05/24	CH2205	Male	Three y/o	Treatment (Wk 2)	Treatment	Feed Day	No	03:30	03:00 - 03:59	Early Morning	37,5	83	10,0	10,0	18,65	580,11	76,76
2019/05/24	CH2206	Male	Three y/o	Treatment (Wk 2)	Treatment	Feed Day	No	03:30	03:00 - 03:59	Early Morning	37,3	86	66,4	66,4	18,46	582,77	141,61
2019/05/24	CH2205	Male	Three y/o	Treatment (Wk 2)	Treatment	Feed Day	No	03:35	03:00 - 03:59	Early Morning	37,5	83	8,4	8,4	18,65	580,11	70,86
2019/05/24	CH2206	Male	Three y/o	Treatment (Wk 2)	Treatment	Feed Day	No	03:35	03:00 - 03:59	Early Morning	37,3	65	61,0	61,0	18,46	561,12	138,67
2019/05/24	CH2205	Male	Three y/o	Treatment (Wk 2)	Treatment	Feed Day	No	03:40	03:00 - 03:59	Early Morning	37,5	40	10,4	10,4	18,65	520,09	78,08
2019/05/24	CH2206	Male	Three y/o	Treatment (Wk 2)	Treatment	Feed Day	No	03:40	03:00 - 03:59	Early Morning	37,4	44	62,6	62,6	18,55	528,51	139,56
2019/05/24	CH2205	Male	Three y/o	Treatment (Wk 2)	Treatment	Feed Day	No	03:45	03:00 - 03:59	Early Morning	37,5	53	7,4	7,4	18,65	544,43	66,59
2019/05/24	CH2206	Male	Three y/o	Treatment (Wk 2)	Treatment	Feed Day	No	03:45	03:00 - 03:59	Early Morning	37,4	86	63,6	63,6	18,55	582,77	140,11
2019/05/24	CH2205	Male	Three y/o	Treatment (Wk 2)	Treatment	Feed Day	No	03:50	03:00 - 03:59	Early Morning	37,5	90	6,6	6,6	18,65	586,16	62,73
2019/05/24	CH2206	Male	Three y/o	Treatment (Wk 2)	Treatment	Feed Day	No	03:50	03:00 - 03:59	Early Morning	37,4	52	64,6	64,6	18,55	542,83	140,65
2019/05/24	CH2205	Male	Three y/o	Treatment (Wk 2)	Treatment	Feed Day	No	03:55	03:00 - 03:59	Early Morning	37,5	97	7,6	7,6	18,65	591,66	67,49
2019/05/24	CH2206	Male	Three y/o	Treatment (Wk 2)	Treatment	Feed Day	No	03:55	03:00 - 03:59	Early Morning	37,4	81	62,8	62,8	18,55	578,26	139,67
2019/05/24	CH2205	Male	Three y/o	Treatment (Wk 2)	Treatment	Feed Day	No	04:00	04:00 - 04:59	Morning	37,6	52	9,6	9,6	18,75	542,83	75,37
2019/05/24	CH2206	Male	Three y/o	Treatment (Wk 2)	Treatment	Feed Day	No	04:00	04:00 - 04:59	Morning	37,4	56	65,8	65,8	18,55	549,01	141,29
2019/05/24	CH2205	Male	Three y/o	Treatment (Wk 2)	Treatment	Feed Day	No	04:05	04:00 - 04:59	Morning	37,6	58	14,0	14,0	18,75	551,90	88,17
2019/05/24	CH2206	Male	Three y/o	Treatment (Wk 2)	Treatment	Feed Day	No	04:05	04:00 - 04:59	Morning	37,4	92	63,4	63,4	18,55	587,78	140,00
2019/05/24	CH2205	Male	Three y/o	Treatment (Wk 2)	Treatment	Feed Day	No	04:10	04:00 - 04:59	Morning	37,6	86	15,4	15,4	18,75	582,77	91,41
2019/05/24	CH2206	Male	Three y/o	Treatment (Wk 2)	Treatment	Feed Day	No	04:10	04:00 - 04:59	Morning	37,5	82	60,8	60,8	18,65	579,19	138,55
2019/05/24	CH2205	Male	Three y/o	Treatment (Wk 2)	Treatment	Feed Day	No	04:15	04:00 - 04:59	Morning	37,6	57	13,4	13,4	18,75	550,47	86,68
2019/05/24	CH2206	Male	Three y/o	Treatment (Wk 2)	Treatment	Feed Day	No	04:15	04:00 - 04:59	Morning	37,5	52	61,2	61,2	18,65	542,83	138,78
2019/05/24	CH2205	Male	Three y/o	Treatment (Wk 2)	Treatment	Feed Day	No	04:20	04:00 - 04:59	Morning	37,7	63	8,6	8,6	18,85	558,62	71,66
2019/05/24	CH2206	Male	Three y/o	Treatment (Wk 2)	Treatment	Feed Day	No	04:20	04:00 - 04:59	Morning	37,7	44	62,6	62,6	18,85	528,51	139,56
2019/05/24	CH2205	Male	Three y/o	Treatment (Wk 2)	Treatment	Feed Day	No	04:25	04:00 - 04:59	Morning	37,7	52	10,8	10,8	18,85	542,83	79,36
2019/05/24	CH2206	Male	Three y/o	Treatment (Wk 2)	Treatment	Feed Day	No	04:25	04:00 - 04:59	Morning	37,7	76	65,0	65,0	18,85	573,39	140,87
2019/05/24	CH2205	Male	Three y/o	Treatment (Wk 2)	Treatment	Feed Day	No	04:30	04:00 - 04:59	Morning	37,7	48	8,0	8,0	18,85	536,04	69,22
2019/05/24	CH2206	Male	Three y/o	Treatment (Wk 2)	Treatment	Feed Day	No	04:30	04:00 - 04:59	Morning	37,7	52	63,8	63,8	18,85	542,83	140,22
2019/05/24	CH2205	Male	Three y/o	Treatment (Wk 2)	Treatment	Feed Day	No	04:35	04:00 - 04:59	Morning	37,7	50	29,6	29,6	18,85	539,52	113,73
2019/05/24	CH2206	Male	Three y/o	Treatment (Wk 2)	Treatment	Feed Day	No	04:35	04:00 - 04:59	Morning	37,5	86	29,4	29,4	18,65	582,77	113,50
2019/05/24	CH2205	Male	Three y/o	Treatment (Wk 2)	Treatment	Feed Day	No	04:40	04:00 - 04:59	Morning	37,7	156	160,6	160,6	18,85	624,43	172,41
2019/05/24	CH2206	Male	Three y/o	Treatment (Wk 2)	Treatment	Feed Day	No	04:40	04:00 - 04:59	Morning	37,5	46	6,2	6,2	18,65	532,38	60,63
2019/05/24	CH2205	Male	Three y/o	Treatment (Wk 2)	Treatment	Feed Day	No	04:45	04:00 - 04:59	Morning	37,6	67	141,6	141,6	18,75	563,53	168,00
2019/05/24	CH2206	Male	Three y/o	Treatment (Wk 2)	Treatment	Feed Day	No	04:45	04:00 - 04:59	Morning	37,4	68	23,4	23,4	18,55	564,71	105,68
2019/05/24	CH2205	Male	Three y/o	Treatment (Wk 2)	Treatment	Feed Day	No	04:50	04:00 - 04:59	Morning	37,5	132	148,4	148,4	18,65	613,32	169,64
2019/05/24	CH2206	Male	Three y/o	Treatment (Wk 2)	Treatment	Feed Day	No	04:50	04:00 - 04:59	Morning	37,4	76	110,8	110,8	18,55	573,39	159,42
2019/05/24	CH2205	Male	Three y/o	Treatment (Wk 2)	Treatment	Feed Day	No	04:55	04:00 - 04:59	Morning	37,5		37,0	37,0	18,65		121,40
2019/05/24	CH2206	Male	Three y/o	Treatment (Wk 2)	Treatment	Feed Day	No	04:55	04:00 - 04:59	Morning	37,4	110	30,2	30,2	18,55	600,69	114,42
2019/05/24	CH2205	Male	Three y/o	Treatment (Wk 2)	Treatment	Feed Day	No	05:00	05:00 - 05:59	Morning	37,5		124,0	124,0	18,65		163,35
2019/05/24	CH2206	Male	Three y/o	Treatment (Wk 2)	Treatment	Feed Day	No	05:00	05:00 - 05:59	Morning	37,5	108	308,0	308,0	18,65	599,39	195,35
2019/05/24	CH2205	Male	Three y/o	Treatment (Wk 2)	Treatment	Feed Day	No	05:05	05:00 - 05:59	Morning	37,6	141	88,2	88,2	18,75	617,75	151,47
2019/05/24	CH2206	Male	Three y/o	Treatment (Wk 2)	Treatment	Feed Day	No	05:05	05:00 - 05:59	Morning	37,5	125	87,8	87,8	18,65	609,60	151,31
2019/05/24	CH2205	Male	Three y/o	Treatment (Wk 2)	Treatment	Feed Day	No	05:10	05:00 - 05:59	Morning	37,5	81	31,8	31,8	18,65	578,26	116,20
2019/05/24	CH2206	Male	Three y/o	Treatment (Wk 2)	Treatment	Feed Day	No	05:10	05:00 - 05:59	Morning	37,4	141	182,8	182,8	18,55	617,75	176,96
2019/05/24	CH2205	Male	Three y/o	Treatment (Wk 2)	Treatment	Feed Day	No	05:15	05:00 - 05:59	Morning	37,4	99	102,2	102,2	18,55	593,14	156,60
2019/05/24	CH2206	Male	Three y/o	Treatment (Wk 2)	Treatment	Feed Day	No	05:15	05:00 - 05:59	Morning	37,4	134	83,2	83,2	18,55	614,33	149,44
2019/05/24	CH2205	Male	Three y/o	Treatment (Wk 2)	Treatment	Feed Day	No	05:20	05:00 - 05:59	Morning	37,5	96	34,8	34,8	18,65	590,91	119,29
2019/05/24	CH2206	Male	Three y/o	Treatment (Wk 2)	Treatment	Feed Day	No	05:20	05:00 - 05:59	Morning	37,4		91,4	91,4	18,55		152,71
2019/05/24	CH2205	Male	Three y/o	Treatment (Wk 2)	Treatment	Feed Day	No	05:25	05:00 - 05:59	Morning	37,5	108	34,4	34,4	18,65	599,39	118,90
2019/05/24	CH2206	Male	Three y/o	Treatment (Wk 2)	Treatment	Feed Day	No	05:25	05:00 - 05:59	Morning	37,4	119	45,2	45,2	18,55	606,20	128,30
2019/05/24	CH2205	Male	Three y/o	Treatment (Wk 2)	Treatment	Feed Day	No	05:30	05:00 - 05:59	Morning	37,2	55	28,8	28,8	18,36	547,52	112,79

2019/05/24	CH2206	Male	Three y/o	Treatment (Wk 2)	Treatment	Feed Day	No	05:30	05:00 - 05:59	Morning	37,3	98	52,4	52,4	18,46	592,41	133,41
2019/05/24	CH2205	Male	Three y/o	Treatment (Wk 2)	Treatment	Feed Day	No	05:35	05:00 - 05:59	Morning	36,8	41	30,2	30,2	17,96	522,29	114,42
2019/05/24	CH2206	Male	Three y/o	Treatment (Wk 2)	Treatment	Feed Day	No	05:35	05:00 - 05:59	Morning	36,9	67	53,2	53,2	18,06	563,53	133,93
2019/05/24	CH2205	Male	Three y/o	Treatment (Wk 2)	Treatment	Feed Day	No	05:40	05:00 - 05:59	Morning	36,8		24,8	24,8	17,96		107,67
2019/05/24	CH2206	Male	Three y/o	Treatment (Wk 2)	Treatment	Feed Day	No	05:40	05:00 - 05:59	Morning	36,7	90	60,6	60,6	17,87	586,16	138,44
2019/05/24	CH2205	Male	Three y/o	Treatment (Wk 2)	Treatment	Feed Day	No	05:45	05:00 - 05:59	Morning	36,8	86	24,6	24,6	17,96	582,77	107,39
2019/05/24	CH2206	Male	Three y/o	Treatment (Wk 2)	Treatment	Feed Day	No	05:45	05:00 - 05:59	Morning	36,6	44	59,8	59,8	17,77	528,51	137,98
2019/05/24	CH2205	Male	Three y/o	Treatment (Wk 2)	Treatment	Feed Day	No	05:50	05:00 - 05:59	Morning	37,0	54	13,6	13,6	18,16	545,99	87,18
2019/05/24	CH2206	Male	Three y/o	Treatment (Wk 2)	Treatment	Feed Day	No	05:50	05:00 - 05:59	Morning	36,8	49	57,8	57,8	17,96	537,80	136,80
2019/05/24	CH2205	Male	Three y/o	Treatment (Wk 2)	Treatment	Feed Day	No	05:55	05:00 - 05:59	Morning	37,0	53	17,2	17,2	18,16	544,43	95,17
2019/05/24	CH2206	Male	Three y/o	Treatment (Wk 2)	Treatment	Feed Day	No	05:55	05:00 - 05:59	Morning	37,0	84	54,8	54,8	18,16	581,01	134,96
2019/05/24	CH2205	Male	Three y/o	Treatment (Wk 2)	Treatment	Feed Day	No	06:00	06:00 - 06:59	Morning	37,1	57	16,6	16,6	18,26	550,47	93,96
2019/05/24	CH2206	Male	Three y/o	Treatment (Wk 2)	Treatment	Feed Day	No	06:00	06:00 - 06:59	Morning	37,3	54	54,4	54,4	18,46	545,99	134,70
2019/05/24	CH2205	Male	Three y/o	Treatment (Wk 2)	Treatment	Feed Day	No	06:05	06:00 - 06:59	Morning	37,2	61	4,2	4,2	18,36	556,01	47,57
2019/05/24	CH2206	Male	Three y/o	Treatment (Wk 2)	Treatment	Feed Day	No	06:05	06:00 - 06:59	Morning	37,4	69	54,8	54,8	18,55	565,86	134,96
2019/05/24	CH2205	Male	Three y/o	Treatment (Wk 2)	Treatment	Feed Day	No	06:10	06:00 - 06:59	Morning	37,3	50	4,8	4,8	18,46	539,52	52,04
2019/05/24	CH2206	Male	Three y/o	Treatment (Wk 2)	Treatment	Feed Day	No	06:10	06:00 - 06:59	Morning	37,3	59	65,2	65,2	18,46	553,30	140,97
2019/05/24	CH2205	Male	Three y/o	Treatment (Wk 2)	Treatment	Feed Day	No	06:15	06:00 - 06:59	Morning	37,3	80	5,4	5,4	18,46	577,31	55,99
2019/05/24	CH2206	Male	Three y/o	Treatment (Wk 2)	Treatment	Feed Day	No	06:15	06:00 - 06:59	Morning	37,3	83	52,4	52,4	18,46	580,11	133,41
2019/05/24	CH2205	Male	Three y/o	Treatment (Wk 2)	Treatment	Feed Day	No	06:20	06:00 - 06:59	Morning	37,3	60	23,8	23,8	18,46	554,67	106,26
2019/05/24	CH2206	Male	Three y/o	Treatment (Wk 2)	Treatment	Feed Day	No	06:20	06:00 - 06:59	Morning	37,3	71	59,2	59,2	18,46	568,10	137,63
2019/05/24	CH2205	Male	Three y/o	Treatment (Wk 2)	Treatment	Feed Day	No	06:25	06:00 - 06:59	Morning	37,4	68	28,2	28,2	18,55	564,71	112,07
2019/05/24	CH2206	Male	Three y/o	Treatment (Wk 2)	Treatment	Feed Day	No	06:25	06:00 - 06:59	Morning	37,3	81	57,8	57,8	18,46	578,26	136,80
2019/05/24	CH2205	Male	Three y/o	Treatment (Wk 2)	Treatment	Feed Day	No	06:30	06:00 - 06:59	Morning	37,3		21,8	21,8	18,46		103,26
2019/05/24	CH2206	Male	Three y/o	Treatment (Wk 2)	Treatment	Feed Day	No	06:30	06:00 - 06:59	Morning	37,4	109	131,0	131,0	18,55	600,04	165,27
2019/05/24	CH2205	Male	Three y/o	Treatment (Wk 2)	Treatment	Feed Day	No	06:35	06:00 - 06:59	Morning	37,3	62	32,8	32,8	18,46	557,33	117,26
2019/05/24	CH2206	Male	Three y/o	Treatment (Wk 2)	Treatment	Feed Day	No	06:35	06:00 - 06:59	Morning	37,5	132	116,8	116,8	18,65	613,32	161,26
2019/05/24	CH2205	Male	Three y/o	Treatment (Wk 2)	Treatment	Feed Day	No	06:40	06:00 - 06:59	Morning	37,3	141	38,0	38,0	18,46	617,75	122,32
2019/05/24	CH2206	Male	Three y/o	Treatment (Wk 2)	Treatment	Feed Day	No	06:40	06:00 - 06:59	Morning	37,5	118	105,0	105,0	18,65	605,61	157,55
2019/05/24	CH2205	Male	Three y/o	Treatment (Wk 2)	Treatment	Feed Day	No	06:45	06:00 - 06:59	Morning	37,3	108	52,4	52,4	18,46	599,39	133,41
2019/05/24	CH2206	Male	Three y/o	Treatment (Wk 2)	Treatment	Feed Day	No	06:45	06:00 - 06:59	Morning	37,5	129	179,0	179,0	18,65	611,75	176,22
2019/05/24	CH2205	Male	Three y/o	Treatment (Wk 2)	Treatment	Feed Day	No	06:50	06:00 - 06:59	Morning	37,2	92	312,0	312,0	18,36	587,78	195,81
2019/05/24	CH2206	Male	Three y/o	Treatment (Wk 2)	Treatment	Feed Day	No	06:50	06:00 - 06:59	Morning	37,5	169	597,0	597,0	18,65	629,60	218,87
2019/05/24	CH2205	Male	Three y/o	Treatment (Wk 2)	Treatment	Feed Day	No	06:55	06:00 - 06:59	Morning	37,3	100	134,4	134,4	18,46	593,87	166,17
2019/05/24	CH2206	Male	Three y/o	Treatment (Wk 2)	Treatment	Feed Day	No	06:55	06:00 - 06:59	Morning	37,5	111	189,0	189,0	18,65	601,33	178,13
2019/05/24	CH2205	Male	Three y/o	Treatment (Wk 2)	Treatment	Feed Day	No	07:00	07:00 - 07:59	Morning	37,4	46	48,0	48,0	18,55	532,38	130,38
2019/05/24	CH2206	Male	Three y/o	Treatment (Wk 2)	Treatment	Feed Day	No	07:00	07:00 - 07:59	Morning	37,7	88	52,6	52,6	18,85	584,49	133,54
2019/05/24	CH2205	Male	Three y/o	Treatment (Wk 2)	Treatment	Feed Day	No	07:05	07:00 - 07:59	Morning	37,4	82	54,6	54,6	18,55	579,19	134,83
2019/05/24	CH2206	Male	Three y/o	Treatment (Wk 2)	Treatment	Feed Day	No	07:05	07:00 - 07:59	Morning	37,7	124	244,0	244,0	18,85	609,04	187,12
2019/05/24	CH2205	Male	Three y/o	Treatment (Wk 2)	Treatment	Feed Day	No	07:10	07:00 - 07:59	Morning	37,5	124	71,2	71,2	18,65	609,04	144,03
2019/05/24	CH2206	Male	Three y/o	Treatment (Wk 2)	Treatment	Feed Day	No	07:10	07:00 - 07:59	Morning	37,7	113	97,2	97,2	18,85	602,58	154,85
2019/05/24	CH2205	Male	Three y/o	Treatment (Wk 2)	Treatment	Feed Day	No	07:15	07:00 - 07:59	Morning	37,5		54,2	54,2	18,65		134,57
2019/05/24	CH2206	Male	Three y/o	Treatment (Wk 2)	Treatment	Feed Day	No	07:15	07:00 - 07:59	Morning	37,8	126	52,6	52,6	18,95	610,14	133,54
2019/05/24	CH2205	Male	Three y/o	Treatment (Wk 2)	Treatment	Feed Day	No	07:20	07:00 - 07:59	Morning	37,5	103	29,0	29,0	18,65	596,00	113,03
2019/05/24	CH2206	Male	Three y/o	Treatment (Wk 2)	Treatment	Feed Day	No	07:20	07:00 - 07:59	Morning	37,7	108	105,4	105,4	18,85	599,39	157,68
2019/05/24	CH2205	Male	Three y/o	Treatment (Wk 2)	Treatment	Feed Day	No	07:25	07:00 - 07:59	Morning	37,4		73,0	73,0	18,55		144,89
2019/05/24	CH2206	Male	Three y/o	Treatment (Wk 2)	Treatment	Feed Day	No	07:25	07:00 - 07:59	Morning	37,7	120	51,6	51,6	18,85	606,78	132,87
2019/05/24	CH2205	Male	Three y/o	Treatment (Wk 2)	Treatment	Feed Day	No	07:30	07:00 - 07:59	Morning	37,5		127,6	127,6	18,65		164,35
2019/05/24	CH2206	Male	Three y/o	Treatment (Wk 2)	Treatment	Feed Day	No	07:30	07:00 - 07:59	Morning	37,7	107	65,0	65,0	18,85	598,73	140,87
2019/05/24	CH2205	Male	Three y/o	Treatment (Wk 2)	Treatment	Feed Day	No	07:35	07:00 - 07:59	Morning	37,7	62	154,6	154,6	18,85	557,33	171,08
2019/05/24	CH2206	Male	Three y/o	Treatment (Wk 2)	Treatment	Feed Day	No	07:35	07:00 - 07:59	Morning	37,6	108	125,8	125,8	18,75	599,39	163,86
2019/05/24	CH2205	Male	Three y/o	Treatment (Wk 2)	Treatment	Feed Day	No	07:40	07:00 - 07:59	Morning	37,7	119	32,0	32,0	18,85	606,20	116,41
2019/05/24	CH2206	Male	Three y/o	Treatment (Wk 2)	Treatment	Feed Day	No	07:40	07:00 - 07:59	Morning	37,7	154	208,8	208,8	18,85	623,58	181,63
2019/05/24	CH2205	Male	Three y/o	Treatment (Wk 2)	Treatment	Feed Day	No	07:45	07:00 - 07:59	Morning	37,7	194	123,6	123,6	18,85	638,30	163,24
2019/05/24	CH2206	Male	Three y/o	Treatment (Wk 2)	Treatment	Feed Day	No	07:45	07:00 - 07:59	Morning	37,8	118	159,4	159,4	18,95	605,61	172,15
2019/05/24	CH2205	Male	Three y/o	Treatment (Wk 2)	Treatment	Feed Day	No	07:50	07:00 - 07:59	Morning	37,7	109	115,8	115,8	18,85	600,04	160,96
2019/05/24	CH2206	Male	Three y/o	Treatment (Wk 2)	Treatment	Feed Day	No	07:50	07:00 - 07:59	Morning	37,8	118	114,6	114,6	18,95	605,61	160,60
2019/05/24	CH2205	Male	Three y/o	Treatment (Wk 2)	Treatment	Feed Day	No	07:55	07:00 - 07:59	Morning	37,6	46	90,0	90,0	18,75	532,38	152,17
2019/05/24	CH2206	Male	Three y/o	Treatment (Wk 2)	Treatment	Feed Day	No	07:55	07:00 - 07:59	Morning	37,7	64	148,4	148,4	18,85	559,88	169,64
2019/05/24	CH2205	Male	Three y/o	Treatment (Wk 2)	Treatment	Feed Day	No	08:00	08:00 - 08:59	Late Morning	37,6	100	40,8	40,8	18,75	593,87	124,77
2019/05/24	CH2206	Male	Three y/o	Treatment (Wk 2)	Treatment	Feed Day	No	08:00	08:00 - 08:59	Late Morning	37,7		127,6	127,6	18,85		164,35
2019/05/24	CH2205	Male	Three y/o	Treatment (Wk 2)	Treatment	Feed Day	No	08:05	08:00 - 08:59	Late Morning	37,7		45,2	45,2	18,85		128,30
2019/05/24	CH2206	Male	Three y/o	Treatment (Wk 2)	Treatment	Feed Day	No	08:05	08:00 - 08:59	Late Morning	37,7	106	398,6	398,6	18,85	598,06	204,49
2019/05/24	CH2205	Male	Three y/o	Treatment (Wk 2)	Treatment	Feed Day	No	08:10	08:00 - 08:59	Late Morning	37,6	95	100,4	100,4	18,75	590,14	155,98
2019/05/24	CH2206	Male	Three y/o	Treatment (Wk 2)	Treatment	Feed Day	No	08:10	08:00 - 08:59	Late Morning	37,7	113	258,4	258,4	18,85	602,58	189,15
2019/05/24	CH2205	Male	Three y/o	Treatment (Wk 2)	Treatment	Feed Day	No	08:15	08:00 - 08:59	Late Morning	37,6		126,6	126,6	18,75		164,08
2019/05/24	CH2206	Male	Three y/o	Treatment (Wk 2)	Treatment	Feed Day	No	08:15	08:00 - 08:59	Late Morning	37,7	123	118,0	118,0	18,85	608,49	161,62
2019/05/24	CH2205	Male	Three y/o	Treatment (Wk 2)	Treatment	Feed Day	No	08:20	08:00 - 08:59	Late Morning	37,6	110	23,4	23,4	18,75	600,69	105,68
2019/05/24	CH2206	Male	Three y/o	Treatment (Wk 2)	Treatment	Feed Day	No	08:20	08:00 - 08:59	Late Morning	37,7	123	193,6	193,6	18,85	608,49	178,97
2019/05/24	CH2205	Male	Three y/o	Treatment (Wk 2)	Treatment	Feed Day	No	08:25	08:00 - 08:59	Late Morning	37,5	73	28,2	28,2	18,65	570,27	112,07

2019/05/24	CH2206	Male	Three y/o	Treatment (Wk 2)	Treatment	Feed Day	No	08:25	08:00 - 08:59	Late Morning	37,7	117	197,8	197,8	18,85	605,02	179,73
2019/05/24	CH2205	Male	Three y/o	Treatment (Wk 2)	Treatment	Feed Day	No	08:30	08:00 - 08:59	Late Morning	37,7	89	19,0	19,0	18,85	585,33	98,56
2019/05/24	CH2206	Male	Three y/o	Treatment (Wk 2)	Treatment	Feed Day	No	08:30	08:00 - 08:59	Late Morning	37,7	131	211,4	211,4	18,85	612,80	182,07
2019/05/24	CH2205	Male	Three y/o	Treatment (Wk 2)	Treatment	Feed Day	No	08:35	08:00 - 08:59	Late Morning	37,9	66	26,6	26,6	19,05	562,34	110,07
2019/05/24	CH2206	Male	Three y/o	Treatment (Wk 2)	Treatment	Feed Day	No	08:35	08:00 - 08:59	Late Morning	37,7	61	103,6	103,6	18,85	556,01	157,08
2019/05/24	CH2205	Male	Three y/o	Treatment (Wk 2)	Treatment	Feed Day	No	08:40	08:00 - 08:59	Late Morning	37,9	66	52,4	52,4	19,05	562,34	133,41
2019/05/24	CH2206	Male	Three y/o	Treatment (Wk 2)	Treatment	Feed Day	No	08:40	08:00 - 08:59	Late Morning	37,7	112	76,2	76,2	18,85	601,96	146,38
2019/05/24	CH2205	Male	Three y/o	Treatment (Wk 2)	Treatment	Feed Day	No	08:45	08:00 - 08:59	Late Morning	37,7	123	67,4	67,4	18,85	608,49	142,12
2019/05/24	CH2206	Male	Three y/o	Treatment (Wk 2)	Treatment	Feed Day	No	08:45	08:00 - 08:59	Late Morning	37,7	124	338,6	338,6	18,85	609,04	198,70
2019/05/24	CH2205	Male	Three y/o	Treatment (Wk 2)	Treatment	Feed Day	No	08:50	08:00 - 08:59	Late Morning	37,7	76	39,0	39,0	18,85	573,39	123,22
2019/05/24	CH2206	Male	Three y/o	Treatment (Wk 2)	Treatment	Feed Day	No	08:50	08:00 - 08:59	Late Morning	37,7	98	140,2	140,2	18,85	592,41	167,65
2019/05/24	CH2205	Male	Three y/o	Treatment (Wk 2)	Treatment	Feed Day	No	08:55	08:00 - 08:59	Late Morning	37,5	74	33,4	33,4	18,65	571,33	117,88
2019/05/24	CH2206	Male	Three y/o	Treatment (Wk 2)	Treatment	Feed Day	No	08:55	08:00 - 08:59	Late Morning	37,7	106	141,6	141,6	18,85	598,06	168,00
2019/05/24	CH2205	Male	Three y/o	Treatment (Wk 2)	Treatment	Feed Day	No	09:00	09:00 - 09:59	Late Morning	37,2	84	32,8	32,8	18,36	581,01	117,26
2019/05/24	CH2206	Male	Three y/o	Treatment (Wk 2)	Treatment	Feed Day	No	09:00	09:00 - 09:59	Late Morning	37,7	115	136,6	136,6	18,85	603,82	166,74
2019/05/24	CH2205	Male	Three y/o	Treatment (Wk 2)	Treatment	Feed Day	No	09:05	09:00 - 09:59	Late Morning	36,8	135	25,2	25,2	17,96	614,84	108,22
2019/05/24	CH2206	Male	Three y/o	Treatment (Wk 2)	Treatment	Feed Day	No	09:05	09:00 - 09:59	Late Morning	37,7	119	81,2	81,2	18,85	606,20	148,59
2019/05/24	CH2205	Male	Three y/o	Treatment (Wk 2)	Treatment	Feed Day	No	09:10	09:00 - 09:59	Late Morning	37,1	102	35,0	35,0	18,26	595,30	119,49
2019/05/24	CH2206	Male	Three y/o	Treatment (Wk 2)	Treatment	Feed Day	No	09:10	09:00 - 09:59	Late Morning	37,9	156	129,0	129,0	19,05	624,43	164,74
2019/05/24	CH2205	Male	Three y/o	Treatment (Wk 2)	Treatment	Feed Day	No	09:15	09:00 - 09:59	Late Morning	37,1	165	41,0	41,0	18,26	628,06	124,94
2019/05/24	CH2206	Male	Three y/o	Treatment (Wk 2)	Treatment	Feed Day	No	09:15	09:00 - 09:59	Late Morning	38,0	115	26,8	26,8	19,15	603,82	110,33
2019/05/24	CH2205	Male	Three y/o	Treatment (Wk 2)	Treatment	Feed Day	No	09:20	09:00 - 09:59	Late Morning	37,3	188	35,0	35,0	18,46	636,34	119,49
2019/05/24	CH2206	Male	Three y/o	Treatment (Wk 2)	Treatment	Feed Day	No	09:20	09:00 - 09:59	Late Morning	37,9	117	175,2	175,2	19,05	605,02	175,47
2019/05/24	CH2205	Male	Three y/o	Treatment (Wk 2)	Treatment	Feed Day	No	09:25	09:00 - 09:59	Late Morning	37,3	94	34,6	34,6	18,46	589,37	119,10
2019/05/24	CH2206	Male	Three y/o	Treatment (Wk 2)	Treatment	Feed Day	No	09:25	09:00 - 09:59	Late Morning	37,9	114	200,4	200,4	19,05	603,20	180,19
2019/05/24	CH2205	Male	Three y/o	Treatment (Wk 2)	Treatment	Feed Day	No	09:30	09:00 - 09:59	Late Morning	37,2		21,2	21,2	18,36		102,31
2019/05/24	CH2206	Male	Three y/o	Treatment (Wk 2)	Treatment	Feed Day	No	09:30	09:00 - 09:59	Late Morning	37,8	106	19,2	19,2	18,95	598,06	98,92
2019/05/24	CH2205	Male	Three y/o	Treatment (Wk 2)	Treatment	Feed Day	No	09:35	09:00 - 09:59	Late Morning	37,0	88	11,4	11,4	18,16	584,49	81,19
2019/05/24	CH2206	Male	Three y/o	Treatment (Wk 2)	Treatment	Feed Day	No	09:35	09:00 - 09:59	Late Morning	37,9	106	206,6	206,6	19,05	598,06	181,26
2019/05/24	CH2205	Male	Three y/o	Treatment (Wk 2)	Treatment	Feed Day	No	09:40	09:00 - 09:59	Late Morning	37,1	61	22,0	22,0	18,26	556,01	103,57
2019/05/24	CH2206	Male	Three y/o	Treatment (Wk 2)	Treatment	Feed Day	No	09:40	09:00 - 09:59	Late Morning	37,9	106	48,6	48,6	19,05	598,06	130,81
2019/05/24	CH2205	Male	Three y/o	Treatment (Wk 2)	Treatment	Feed Day	No	09:45	09:00 - 09:59	Late Morning	37,1	197	16,6	16,6	18,26	639,25	93,96
2019/05/24	CH2206	Male	Three y/o	Treatment (Wk 2)	Treatment	Feed Day	No	09:45	09:00 - 09:59	Late Morning	37,9	121	109,8	109,8	19,05	607,35	159,11
2019/05/24	CH2205	Male	Three y/o	Treatment (Wk 2)	Treatment	Feed Day	No	09:50	09:00 - 09:59	Late Morning	37,2	148	15,6	15,6	18,36	620,97	91,85
2019/05/24	CH2206	Male	Three y/o	Treatment (Wk 2)	Treatment	Feed Day	No	09:50	09:00 - 09:59	Late Morning	37,8	124	84,4	84,4	18,95	609,04	149,94
2019/05/24	CH2205	Male	Three y/o	Treatment (Wk 2)	Treatment	Feed Day	No	09:55	09:00 - 09:59	Late Morning	37,3	148	8,8	8,8	18,46	620,97	72,43
2019/05/24	CH2206	Male	Three y/o	Treatment (Wk 2)	Treatment	Feed Day	No	09:55	09:00 - 09:59	Late Morning	37,8	73	30,0	30,0	18,95	570,27	114,20
2019/05/24	CH2205	Male	Three y/o	Treatment (Wk 2)	Treatment	Feed Day	No	10:00	10:00 - 10:59	Late Morning	37,5	41	10,4	10,4	18,65	522,29	78,08
2019/05/24	CH2206	Male	Three y/o	Treatment (Wk 2)	Treatment	Feed Day	No	10:00	10:00 - 10:59	Late Morning	37,7	65	33,2	33,2	18,85	561,12	117,68
2019/05/24	CH2205	Male	Three y/o	Treatment (Wk 2)	Treatment	Feed Day	No	10:05	10:00 - 10:59	Late Morning	37,4	61	9,6	9,6	18,55	556,01	75,37
2019/05/24	CH2206	Male	Three y/o	Treatment (Wk 2)	Treatment	Feed Day	No	10:05	10:00 - 10:59	Late Morning	37,5	45	31,2	31,2	18,65	530,47	115,54
2019/05/24	CH2205	Male	Three y/o	Treatment (Wk 2)	Treatment	Feed Day	No	10:10	10:00 - 10:59	Late Morning	37,3	67	5,6	5,6	18,46	563,53	57,21
2019/05/24	CH2206	Male	Three y/o	Treatment (Wk 2)	Treatment	Feed Day	No	10:10	10:00 - 10:59	Late Morning	37,6	59	54,2	54,2	18,75	553,30	134,57
2019/05/24	CH2205	Male	Three y/o	Treatment (Wk 2)	Treatment	Feed Day	No	10:15	10:00 - 10:59	Late Morning	37,3	58	3,6	3,6	18,46	551,90	42,42
2019/05/24	CH2206	Male	Three y/o	Treatment (Wk 2)	Treatment	Feed Day	No	10:15	10:00 - 10:59	Late Morning	37,7	58	31,6	31,6	18,85	551,90	115,98
2019/05/24	CH2205	Male	Three y/o	Treatment (Wk 2)	Treatment	Feed Day	No	10:20	10:00 - 10:59	Late Morning	37,3	67	9,4	9,4	18,46	563,53	74,66
2019/05/24	CH2206	Male	Three y/o	Treatment (Wk 2)	Treatment	Feed Day	No	10:20	10:00 - 10:59	Late Morning	37,7	74	38,0	38,0	18,85	571,33	122,32
2019/05/24	CH2205	Male	Three y/o	Treatment (Wk 2)	Treatment	Feed Day	No	10:25	10:00 - 10:59	Late Morning	37,3	87	2,6	2,6	18,46	583,64	31,57
2019/05/24	CH2206	Male	Three y/o	Treatment (Wk 2)	Treatment	Feed Day	No	10:25	10:00 - 10:59	Late Morning	37,5	61	41,8	41,8	18,65	556,01	125,60
2019/05/24	CH2205	Male	Three y/o	Treatment (Wk 2)	Treatment	Feed Day	Yes	10:30	10:00 - 10:59	Late Morning	37,3	51	5,4	5,4	18,46	541,20	55,99
2019/05/24	CH2206	Male	Three y/o	Treatment (Wk 2)	Treatment	Feed Day	Yes	10:30	10:00 - 10:59	Late Morning	37,5	69	10,4	10,4	18,65	565,86	78,08
2019/05/24	CH2205	Male	Three y/o	Treatment (Wk 2)	Treatment	Feed Day	Yes	10:35	10:00 - 10:59	Late Morning	37,3	55	6,8	6,8	18,46	547,52	63,74
2019/05/24	CH2206	Male	Three y/o	Treatment (Wk 2)	Treatment	Feed Day	Yes	10:35	10:00 - 10:59	Late Morning	37,5	65	16,8	16,8	18,65	561,12	94,37
2019/05/24	CH2205	Male	Three y/o	Treatment (Wk 2)	Treatment	Feed Day	Yes	10:40	10:00 - 10:59	Late Morning	37,3	64	7,0	7,0	18,46	559,88	64,72
2019/05/24	CH2206	Male	Three y/o	Treatment (Wk 2)	Treatment	Feed Day	Yes	10:40	10:00 - 10:59	Late Morning	37,5	55	25,4	25,4	18,65	547,52	108,49
2019/05/24	CH2205	Male	Three y/o	Treatment (Wk 2)	Treatment	Feed Day	Yes	10:45	10:00 - 10:59	Late Morning	37,3	65	16,0	16,0	18,46	561,12	92,71
2019/05/24	CH2206	Male	Three y/o	Treatment (Wk 2)	Treatment	Feed Day	Yes	10:45	10:00 - 10:59	Late Morning	37,5	128	38,2	38,2	18,65	611,22	122,50
2019/05/24	CH2205	Male	Three y/o	Treatment (Wk 2)	Treatment	Feed Day	Yes	10:50	10:00 - 10:59	Late Morning	37,4		96,8	96,8	18,55		154,71
2019/05/24	CH2206	Male	Three y/o	Treatment (Wk 2)	Treatment	Feed Day	Yes	10:50	10:00 - 10:59	Late Morning	37,6	126	93,6	93,6	18,75	610,14	153,54
2019/05/24	CH2205	Male	Three y/o	Treatment (Wk 2)	Treatment	Feed Day	Yes	10:55	10:00 - 10:59	Late Morning	37,4	141	441,0	441,0	18,55	617,75	208,08
2019/05/24	CH2206	Male	Three y/o	Treatment (Wk 2)	Treatment	Feed Day	Yes	10:55	10:00 - 10:59	Late Morning	37,5	102	83,2	83,2	18,65	595,30	149,44
2019/05/24	CH2205	Male	Three y/o	Treatment (Wk 2)	Treatment	Feed Day	Yes	11:00	11:00 - 11:59	Late Morning	37,7	49	35,8	35,8	18,85	537,80	120,27
2019/05/24	CH2206	Male	Three y/o	Treatment (Wk 2)	Treatment	Feed Day	Yes	11:00	11:00 - 11:59	Late Morning	37,5	102	155,2	155,2	18,65	595,30	171,21
2019/05/24	CH2205	Male	Three y/o	Treatment (Wk 2)	Treatment	Feed Day	Yes	11:05	11:00 - 11:59	Late Morning	37,7		19,0	19,0	18,85		98,56
2019/05/24	CH2206	Male	Three y/o	Treatment (Wk 2)	Treatment	Feed Day	Yes	11:05	11:00 - 11:59	Late Morning	37,5	121	300,4	300,4	18,65	607,35	194,47
2019/05/24	CH2205	Male	Three y/o	Treatment (Wk 2)	Treatment	Feed Day	Yes	11:10	11:00 - 11:59	Late Morning	37,9	78	59,4	59,4	19,05	575,38	137,75
2019/05/24	CH2206	Male	Three y/o	Treatment (Wk 2)	Treatment	Feed Day	Yes	11:10	11:00 - 11:59	Late Morning	37,7	132	249,8	249,8	18,85	613,32	187,95
2019/05/24	CH2205	Male	Three y/o	Treatment (Wk 2)	Treatment	Feed Day	Yes	11:15	11:00 - 11:59	Late Morning	37,7	110	113,8	113,8	18,85	600,69	160,35
2019/05/24	CH2206	Male	Three y/o	Treatment (Wk 2)	Treatment	Feed Day	Yes	11:15	11:00 - 11:59	Late Morning	37,7	104	192,8	192,8	18,85	596,69	178,83
2019/05/24	CH2205	Male	Three y/o	Treatment (Wk 2)	Treatment	Feed Day	Yes	11:20	11:00 - 11:59	Late Morning	37,7	96	202,2	202,2	18,85	590,91	180,50

2019/05/24	CH2206	Male	Three y/o	Treatment (Wk 2)	Treatment	Feed Day	Yes	11:20	11:00 - 11:59	Late Morning	37,7	115	163,2	163,2	18,85	603,82	172,97
2019/05/24	CH2205	Male	Three y/o	Treatment (Wk 2)	Treatment	Feed Day	Yes	11:25	11:00 - 11:59	Late Morning	37,7	109	53,8	53,8	18,85	600,04	134,32
2019/05/24	CH2206	Male	Three y/o	Treatment (Wk 2)	Treatment	Feed Day	Yes	11:25	11:00 - 11:59	Late Morning	37,7	114	271,4	271,4	18,85	603,20	190,88
2019/05/24	CH2205	Male	Three y/o	Treatment (Wk 2)	Treatment	Feed Day	No	11:30	11:00 - 11:59	Late Morning	37,7	112	44,0	44,0	18,85	601,96	127,37
2019/05/24	CH2206	Male	Three y/o	Treatment (Wk 2)	Treatment	Feed Day	No	11:30	11:00 - 11:59	Late Morning	37,8	111	65,2	65,2	18,95	601,33	140,97
2019/05/24	CH2205	Male	Three y/o	Treatment (Wk 2)	Treatment	Feed Day	No	11:35	11:00 - 11:59	Late Morning	37,6	76	24,8	24,8	18,75	573,39	107,67
2019/05/24	CH2206	Male	Three y/o	Treatment (Wk 2)	Treatment	Feed Day	No	11:35	11:00 - 11:59	Late Morning	37,8	162	64,8	64,8	18,95	626,88	140,76
2019/05/24	CH2205	Male	Three y/o	Treatment (Wk 2)	Treatment	Feed Day	No	11:40	11:00 - 11:59	Late Morning	37,5	73	28,8	28,8	18,65	570,27	112,79
2019/05/24	CH2206	Male	Three y/o	Treatment (Wk 2)	Treatment	Feed Day	No	11:40	11:00 - 11:59	Late Morning	37,7		52,0	52,0	18,85		133,14
2019/05/24	CH2205	Male	Three y/o	Treatment (Wk 2)	Treatment	Feed Day	No	11:45	11:00 - 11:59	Late Morning	37,5	90	22,2	22,2	18,65	586,16	103,88
2019/05/24	CH2206	Male	Three y/o	Treatment (Wk 2)	Treatment	Feed Day	No	11:45	11:00 - 11:59	Late Morning	37,7	102	79,6	79,6	18,85	595,30	147,90
2019/05/24	CH2205	Male	Three y/o	Treatment (Wk 2)	Treatment	Feed Day	No	11:50	11:00 - 11:59	Late Morning	37,6		32,4	32,4	18,75		116,84
2019/05/24	CH2206	Male	Three y/o	Treatment (Wk 2)	Treatment	Feed Day	No	11:50	11:00 - 11:59	Late Morning	37,8	120	57,0	57,0	18,95	606,78	136,32
2019/05/24	CH2205	Male	Three y/o	Treatment (Wk 2)	Treatment	Feed Day	No	11:55	11:00 - 11:59	Late Morning	37,9	84	32,8	32,8	19,05	581,01	117,26
2019/05/24	CH2206	Male	Three y/o	Treatment (Wk 2)	Treatment	Feed Day	No	11:55	11:00 - 11:59	Late Morning	37,8	131	86,2	86,2	18,95	612,80	150,67
2019/05/24	CH2205	Male	Three y/o	Treatment (Wk 2)	Treatment	Feed Day	No	12:00	12:00 - 12:59	Afternoon	38,0	100	23,2	23,2	19,15	593,87	105,39
2019/05/24	CH2206	Male	Three y/o	Treatment (Wk 2)	Treatment	Feed Day	No	12:00	12:00 - 12:59	Afternoon	37,9	105	31,2	31,2	19,05	597,38	115,54
2019/05/24	CH2205	Male	Three y/o	Treatment (Wk 2)	Treatment	Feed Day	No	12:05	12:00 - 12:59	Afternoon	38,0	90	43,0	43,0	19,15	586,16	126,58
2019/05/24	CH2206	Male	Three y/o	Treatment (Wk 2)	Treatment	Feed Day	No	12:05	12:00 - 12:59	Afternoon	37,9	177	43,2	43,2	19,05	632,55	126,74
2019/05/24	CH2205	Male	Three y/o	Treatment (Wk 2)	Treatment	Feed Day	No	12:10	12:00 - 12:59	Afternoon	38,0	72	15,4	15,4	19,15	569,20	91,41
2019/05/24	CH2206	Male	Three y/o	Treatment (Wk 2)	Treatment	Feed Day	No	12:10	12:00 - 12:59	Afternoon	37,9	120	42,2	42,2	19,05	606,78	125,93
2019/05/24	CH2205	Male	Three y/o	Treatment (Wk 2)	Treatment	Feed Day	No	12:15	12:00 - 12:59	Afternoon	38,1	67	17,2	17,2	19,26	563,53	95,17
2019/05/24	CH2206	Male	Three y/o	Treatment (Wk 2)	Treatment	Feed Day	No	12:15	12:00 - 12:59	Afternoon	38,0	147	53,2	53,2	19,15	620,52	133,93
2019/05/24	CH2205	Male	Three y/o	Treatment (Wk 2)	Treatment	Feed Day	No	12:20	12:00 - 12:59	Afternoon	38,1	99	284,6	284,6	19,26	593,14	192,56
2019/05/24	CH2206	Male	Three y/o	Treatment (Wk 2)	Treatment	Feed Day	No	12:20	12:00 - 12:59	Afternoon	38,0	167	194,6	194,6	19,15	628,84	179,16
2019/05/24	CH2205	Male	Three y/o	Treatment (Wk 2)	Treatment	Feed Day	No	12:25	12:00 - 12:59	Afternoon	38,1	79	88,4	88,4	19,26	576,36	151,55
2019/05/24	CH2206	Male	Three y/o	Treatment (Wk 2)	Treatment	Feed Day	No	12:25	12:00 - 12:59	Afternoon	38,1	124	159,8	159,8	19,26	609,04	172,24
2019/05/24	CH2205	Male	Three y/o	Treatment (Wk 2)	Treatment	Feed Day	No	12:30	12:00 - 12:59	Afternoon	38,0	99	56,8	56,8	19,15	593,14	136,20
2019/05/24	CH2206	Male	Three y/o	Treatment (Wk 2)	Treatment	Feed Day	No	12:30	12:00 - 12:59	Afternoon	38,2		82,0	82,0	19,36		148,93
2019/05/24	CH2205	Male	Three y/o	Treatment (Wk 2)	Treatment	Feed Day	No	12:35	12:00 - 12:59	Afternoon	38,0	50	13,0	13,0	19,15	539,52	85,65
2019/05/24	CH2206	Male	Three y/o	Treatment (Wk 2)	Treatment	Feed Day	No	12:35	12:00 - 12:59	Afternoon	38,2	112	51,0	51,0	19,36	601,96	132,47
2019/05/24	CH2205	Male	Three y/o	Treatment (Wk 2)	Treatment	Feed Day	No	12:40	12:00 - 12:59	Afternoon	38,0	46	16,2	16,2	19,15	532,38	93,13
2019/05/24	CH2206	Male	Three y/o	Treatment (Wk 2)	Treatment	Feed Day	No	12:40	12:00 - 12:59	Afternoon	38,3	111	14,8	14,8	19,46	601,33	90,05
2019/05/24	CH2205	Male	Three y/o	Treatment (Wk 2)	Treatment	Feed Day	No	12:45	12:00 - 12:59	Afternoon	38,0	53	12,0	12,0	19,15	544,43	82,93
2019/05/24	CH2206	Male	Three y/o	Treatment (Wk 2)	Treatment	Feed Day	No	12:45	12:00 - 12:59	Afternoon	38,3	150	75,8	75,8	19,46	621,86	146,20
2019/05/24	CH2205	Male	Three y/o	Treatment (Wk 2)	Treatment	Feed Day	No	12:50	12:00 - 12:59	Afternoon	38,0	58	16,2	16,2	19,15	551,90	93,13
2019/05/24	CH2206	Male	Three y/o	Treatment (Wk 2)	Treatment	Feed Day	No	12:50	12:00 - 12:59	Afternoon	38,3	87	29,2	29,2	19,46	583,64	113,27
2019/05/24	CH2205	Male	Three y/o	Treatment (Wk 2)	Treatment	Feed Day	No	12:55	12:00 - 12:59	Afternoon	38,0	43	14,8	14,8	19,15	526,50	90,05
2019/05/24	CH2206	Male	Three y/o	Treatment (Wk 2)	Treatment	Feed Day	No	12:55	12:00 - 12:59	Afternoon	38,3	112	16,8	16,8	19,46	601,96	94,37
2019/05/24	CH2205	Male	Three y/o	Treatment (Wk 2)	Treatment	Feed Day	No	13:00	13:00 - 13:59	Afternoon	38,0	59	12,8	12,8	19,15	553,30	85,12
2019/05/24	CH2206	Male	Three y/o	Treatment (Wk 2)	Treatment	Feed Day	No	13:00	13:00 - 13:59	Afternoon	38,3	88	11,0	11,0	19,46	584,49	79,98
2019/05/24	CH2205	Male	Three y/o	Treatment (Wk 2)	Treatment	Feed Day	No	13:05	13:00 - 13:59	Afternoon	38,0	64	12,6	12,6	19,15	559,88	84,59
2019/05/24	CH2206	Male	Three y/o	Treatment (Wk 2)	Treatment	Feed Day	No	13:05	13:00 - 13:59	Afternoon	38,3	75	16,6	16,6	19,46	572,36	93,96
2019/05/24	CH2205	Male	Three y/o	Treatment (Wk 2)	Treatment	Feed Day	No	13:10	13:00 - 13:59	Afternoon	37,9	50	11,8	11,8	19,05	539,52	82,36
2019/05/24	CH2206	Male	Three y/o	Treatment (Wk 2)	Treatment	Feed Day	No	13:10	13:00 - 13:59	Afternoon	38,3	70	14,6	14,6	19,46	566,99	89,59
2019/05/24	CH2205	Male	Three y/o	Treatment (Wk 2)	Treatment	Feed Day	No	13:15	13:00 - 13:59	Afternoon	37,9	43	17,0	17,0	19,05	526,50	94,77
2019/05/24	CH2206	Male	Three y/o	Treatment (Wk 2)	Treatment	Feed Day	No	13:15	13:00 - 13:59	Afternoon	38,3	67	12,0	12,0	19,46	563,53	82,93
2019/05/24	CH2205	Male	Three y/o	Treatment (Wk 2)	Treatment	Feed Day	No	13:20	13:00 - 13:59	Afternoon	37,9	86	28,0	28,0	19,05	582,77	111,83
2019/05/24	CH2206	Male	Three y/o	Treatment (Wk 2)	Treatment	Feed Day	No	13:20	13:00 - 13:59	Afternoon	38,3	152	39,8	39,8	19,46	622,73	123,91
2019/05/24	CH2205	Male	Three y/o	Treatment (Wk 2)	Treatment	Feed Day	No	13:25	13:00 - 13:59	Afternoon	37,7	43	21,2	21,2	18,85	526,50	102,31
2019/05/24	CH2206	Male	Three y/o	Treatment (Wk 2)	Treatment	Feed Day	No	13:25	13:00 - 13:59	Afternoon	38,2	46	30,0	30,0	19,36	532,38	114,20
2019/05/24	CH2205	Male	Three y/o	Treatment (Wk 2)	Treatment	Feed Day	No	13:30	13:00 - 13:59	Afternoon	37,7	49	50,2	50,2	18,85	537,80	131,92
2019/05/24	CH2206	Male	Three y/o	Treatment (Wk 2)	Treatment	Feed Day	No	13:30	13:00 - 13:59	Afternoon	38,1	60	9,0	9,0	19,26	554,67	73,19
2019/05/24	CH2205	Male	Three y/o	Treatment (Wk 2)	Treatment	Feed Day	No	13:35	13:00 - 13:59	Afternoon	37,7	77	255,4	255,4	18,85	574,39	188,73
2019/05/24	CH2206	Male	Three y/o	Treatment (Wk 2)	Treatment	Feed Day	No	13:35	13:00 - 13:59	Afternoon	38,1	61	84,8	84,8	19,26	556,01	150,10
2019/05/24	CH2205	Male	Three y/o	Treatment (Wk 2)	Treatment	Feed Day	No	13:40	13:00 - 13:59	Afternoon	37,7	182	40,8	40,8	18,85	634,31	124,77
2019/05/24	CH2206	Male	Three y/o	Treatment (Wk 2)	Treatment	Feed Day	No	13:40	13:00 - 13:59	Afternoon	38,1	123	33,2	33,2	19,26	608,49	117,68
2019/05/24	CH2205	Male	Three y/o	Treatment (Wk 2)	Treatment	Feed Day	No	13:45	13:00 - 13:59	Afternoon	37,7		56,6	56,6	18,85		136,07
2019/05/24	CH2206	Male	Three y/o	Treatment (Wk 2)	Treatment	Feed Day	No	13:45	13:00 - 13:59	Afternoon	38,1	90	95,2	95,2	19,26	586,16	154,13
2019/05/24	CH2205	Male	Three y/o	Treatment (Wk 2)	Treatment	Feed Day	No	13:50	13:00 - 13:59	Afternoon	37,9	121	135,8	135,8	19,05	607,35	166,53
2019/05/24	CH2206	Male	Three y/o	Treatment (Wk 2)	Treatment	Feed Day	No	13:50	13:00 - 13:59	Afternoon	38,0	112	54,8	54,8	19,15	601,96	134,96
2019/05/24	CH2205	Male	Three y/o	Treatment (Wk 2)	Treatment	Feed Day	No	13:55	13:00 - 13:59	Afternoon	37,9	93	73,8	73,8	19,05	588,58	145,27
2019/05/24	CH2206	Male	Three y/o	Treatment (Wk 2)	Treatment	Feed Day	No	13:55	13:00 - 13:59	Afternoon	38,0	118	64,4	64,4	19,15	605,61	140,55
2019/05/24	CH2205	Male	Three y/o	Treatment (Wk 2)	Treatment	Feed Day	No	14:00	14:00 - 14:59	Afternoon	37,9	162	716,0	716,0	19,05	626,88	225,36
2019/05/24	CH2206	Male	Three y/o	Treatment (Wk 2)	Treatment	Feed Day	No	14:00	14:00 - 14:59	Afternoon	38,0	134	60,4	60,4	19,15	614,33	138,32
2019/05/24	CH2205	Male	Three y/o	Treatment (Wk 2)	Treatment	Feed Day	No	14:05	14:00 - 14:59	Afternoon	38,6	101	62,4	62,4	19,76	594,59	139,45
2019/05/24	CH2206	Male	Three y/o	Treatment (Wk 2)	Treatment	Feed Day	No	14:05	14:00 - 14:59	Afternoon	38,0		132,2	132,2	19,15		165,59
2019/05/24	CH2205	Male	Three y/o	Treatment (Wk 2)	Treatment	Feed Day	No	14:10	14:00 - 14:59	Afternoon	38,5		37,2	37,2	19,66		121,59
2019/05/24	CH2206	Male	Three y/o	Treatment (Wk 2)	Treatment	Feed Day	No	14:10	14:00 - 14:59	Afternoon	38,1	185	238,4	238,4	19,26	635,34	186,30
2019/05/24	CH2205	Male	Three y/o	Treatment (Wk 2)	Treatment	Feed Day	No	14:15	14:00 - 14:59	Afternoon	38,4		31,2	31,2	19,56		115,54

2019/05/24	CH2206	Male	Three y/o	Treatment (Wk 2)	Treatment	Feed Day	No	14:15	14:00 - 14:59	Afternoon	38,1	103	195,0	195,0	19,26	596,00	179,23
2019/05/24	CH2205	Male	Three y/o	Treatment (Wk 2)	Treatment	Feed Day	No	14:20	14:00 - 14:59	Afternoon	38,3	90	62,4	62,4	19,46	586,16	139,45
2019/05/24	CH2206	Male	Three y/o	Treatment (Wk 2)	Treatment	Feed Day	No	14:20	14:00 - 14:59	Afternoon	38,1	134	131,8	131,8	19,26	614,33	165,49
2019/05/24	CH2205	Male	Three y/o	Treatment (Wk 2)	Treatment	Feed Day	No	14:25	14:00 - 14:59	Afternoon	38,4	65	56,4	56,4	19,56	561,12	135,95
2019/05/24	CH2206	Male	Three y/o	Treatment (Wk 2)	Treatment	Feed Day	No	14:25	14:00 - 14:59	Afternoon	38,1	131	71,4	71,4	19,26	612,80	144,12
2019/05/24	CH2205	Male	Three y/o	Treatment (Wk 2)	Treatment	Feed Day	No	14:30	14:00 - 14:59	Afternoon	38,3		30,4	30,4	19,46		114,65
2019/05/24	CH2206	Male	Three y/o	Treatment (Wk 2)	Treatment	Feed Day	No	14:30	14:00 - 14:59	Afternoon	38,1	71	58,6	58,6	19,26	568,10	137,28
2019/05/24	CH2205	Male	Three y/o	Treatment (Wk 2)	Treatment	Feed Day	No	14:35	14:00 - 14:59	Afternoon	38,3	95	39,6	39,6	19,46	590,14	123,74
2019/05/24	CH2206	Male	Three y/o	Treatment (Wk 2)	Treatment	Feed Day	No	14:35	14:00 - 14:59	Afternoon	38,1	70	33,4	33,4	19,26	566,99	117,88
2019/05/24	CH2205	Male	Three y/o	Treatment (Wk 2)	Treatment	Feed Day	No	14:40	14:00 - 14:59	Afternoon	38,3	64	26,8	26,8	19,46	559,88	110,33
2019/05/24	CH2206	Male	Three y/o	Treatment (Wk 2)	Treatment	Feed Day	No	14:40	14:00 - 14:59	Afternoon	38,1	67	56,4	56,4	19,26	563,53	135,95
2019/05/24	CH2205	Male	Three y/o	Treatment (Wk 2)	Treatment	Feed Day	No	14:45	14:00 - 14:59	Afternoon	38,3	81	11,8	11,8	19,46	578,26	82,36
2019/05/24	CH2206	Male	Three y/o	Treatment (Wk 2)	Treatment	Feed Day	No	14:45	14:00 - 14:59	Afternoon	38,1	52	37,8	37,8	19,26	542,83	122,14
2019/05/24	CH2205	Male	Three y/o	Treatment (Wk 2)	Treatment	Feed Day	No	14:50	14:00 - 14:59	Afternoon	38,3	65	2,2	2,2	19,46	561,12	26,02
2019/05/24	CH2206	Male	Three y/o	Treatment (Wk 2)	Treatment	Feed Day	No	14:50	14:00 - 14:59	Afternoon	38,1	45	40,0	40,0	19,26	530,47	124,09
2019/05/24	CH2205	Male	Three y/o	Treatment (Wk 2)	Treatment	Feed Day	No	14:55	14:00 - 14:59	Afternoon	38,3	49	11,6	11,6	19,46	537,80	81,78
2019/05/24	CH2206	Male	Three y/o	Treatment (Wk 2)	Treatment	Feed Day	No	14:55	14:00 - 14:59	Afternoon	38,1	71	39,4	39,4	19,26	568,10	123,57
2019/05/24	CH2205	Male	Three y/o	Treatment (Wk 2)	Treatment	Feed Day	No	15:00	15:00 - 15:59	Afternoon	38,3	68	14,8	14,8	19,46	564,71	90,05
2019/05/24	CH2206	Male	Three y/o	Treatment (Wk 2)	Treatment	Feed Day	No	15:00	15:00 - 15:59	Afternoon	38,1	75	40,6	40,6	19,26	572,36	124,60
2019/05/24	CH2205	Male	Three y/o	Treatment (Wk 2)	Treatment	Feed Day	No	15:05	15:00 - 15:59	Afternoon	38,3	55	28,0	28,0	19,46	547,52	111,83
2019/05/24	CH2206	Male	Three y/o	Treatment (Wk 2)	Treatment	Feed Day	No	15:05	15:00 - 15:59	Afternoon	38,0	51	41,6	41,6	19,15	541,20	125,44
2019/05/24	CH2205	Male	Three y/o	Treatment (Wk 2)	Treatment	Feed Day	No	15:10	15:00 - 15:59	Afternoon	38,3	41	26,2	26,2	19,46	522,29	109,55
2019/05/24	CH2206	Male	Three y/o	Treatment (Wk 2)	Treatment	Feed Day	No	15:10	15:00 - 15:59	Afternoon	38,0	45	42,0	42,0	19,15	530,47	125,77
2019/05/24	CH2205	Male	Three y/o	Treatment (Wk 2)	Treatment	Feed Day	No	15:15	15:00 - 15:59	Afternoon	38,2	52	24,6	24,6	19,36	542,83	107,39
2019/05/24	CH2206	Male	Three y/o	Treatment (Wk 2)	Treatment	Feed Day	No	15:15	15:00 - 15:59	Afternoon	38,0	74	32,2	32,2	19,15	571,33	116,63
2019/05/24	CH2205	Male	Three y/o	Treatment (Wk 2)	Treatment	Feed Day	No	15:20	15:00 - 15:59	Afternoon	38,2	46	26,6	26,6	19,36	532,38	110,07
2019/05/24	CH2206	Male	Three y/o	Treatment (Wk 2)	Treatment	Feed Day	No	15:20	15:00 - 15:59	Afternoon	38,0	56	31,2	31,2	19,15	549,01	115,54
2019/05/24	CH2205	Male	Three y/o	Treatment (Wk 2)	Treatment	Feed Day	No	15:25	15:00 - 15:59	Afternoon	38,2	61	71,8	71,8	19,36	556,01	144,32
2019/05/24	CH2206	Male	Three y/o	Treatment (Wk 2)	Treatment	Feed Day	No	15:25	15:00 - 15:59	Afternoon	37,9	48	26,6	26,6	19,05	536,04	110,07
2019/05/24	CH2205	Male	Three y/o	Treatment (Wk 2)	Treatment	Feed Day	No	15:30	15:00 - 15:59	Afternoon	38,1	90	107,0	107,0	19,26	586,16	158,20
2019/05/24	CH2206	Male	Three y/o	Treatment (Wk 2)	Treatment	Feed Day	No	15:30	15:00 - 15:59	Afternoon	37,9	80	17,8	17,8	19,05	577,31	96,34
2019/05/24	CH2205	Male	Three y/o	Treatment (Wk 2)	Treatment	Feed Day	No	15:35	15:00 - 15:59	Afternoon	38,1	74	49,0	49,0	19,26	571,33	131,09
2019/05/24	CH2206	Male	Three y/o	Treatment (Wk 2)	Treatment	Feed Day	No	15:35	15:00 - 15:59	Afternoon	37,9	53	38,4	38,4	19,05	544,43	122,68
2019/05/24	CH2205	Male	Three y/o	Treatment (Wk 2)	Treatment	Feed Day	No	15:40	15:00 - 15:59	Afternoon	38,1		63,0	63,0	19,26		139,78
2019/05/24	CH2206	Male	Three y/o	Treatment (Wk 2)	Treatment	Feed Day	No	15:40	15:00 - 15:59	Afternoon	37,8	114	177,4	177,4	18,95	603,20	175,90
2019/05/24	CH2205	Male	Three y/o	Treatment (Wk 2)	Treatment	Feed Day	No	15:45	15:00 - 15:59	Afternoon	38,1	98	75,6	75,6	19,26	592,41	146,11
2019/05/24	CH2206	Male	Three y/o	Treatment (Wk 2)	Treatment	Feed Day	No	15:45	15:00 - 15:59	Afternoon	37,8	47	86,8	86,8	18,95	534,23	150,91
2019/05/24	CH2205	Male	Three y/o	Treatment (Wk 2)	Treatment	Feed Day	No	15:50	15:00 - 15:59	Afternoon	38,1	78	34,4	34,4	19,26	575,38	118,90
2019/05/24	CH2206	Male	Three y/o	Treatment (Wk 2)	Treatment	Feed Day	No	15:50	15:00 - 15:59	Afternoon	37,9	61	125,6	125,6	19,05	556,01	163,80
2019/05/24	CH2205	Male	Three y/o	Treatment (Wk 2)	Treatment	Feed Day	No	15:55	15:00 - 15:59	Afternoon	38,1		32,6	32,6	19,26		117,05
2019/05/24	CH2206	Male	Three y/o	Treatment (Wk 2)	Treatment	Feed Day	No	15:55	15:00 - 15:59	Afternoon	38,0	73	38,8	38,8	19,15	570,27	123,04
2019/05/24	CH2205	Male	Three y/o	Treatment (Wk 2)	Treatment	Feed Day	No	16:00	16:00 - 16:59	Evening	37,7	115	37,0	37,0	18,85	603,82	121,40
2019/05/24	CH2206	Male	Three y/o	Treatment (Wk 2)	Treatment	Feed Day	No	16:00	16:00 - 16:59	Evening	38,0	76	170,6	170,6	19,15	573,39	174,53
2019/05/24	CH2205	Male	Three y/o	Treatment (Wk 2)	Treatment	Feed Day	No	16:05	16:00 - 16:59	Evening	37,9	78	105,4	105,4	19,05	575,38	157,68
2019/05/24	CH2206	Male	Three y/o	Treatment (Wk 2)	Treatment	Feed Day	No	16:05	16:00 - 16:59	Evening	38,1	110	26,0	26,0	19,26	600,69	109,29
2019/05/24	CH2205	Male	Three y/o	Treatment (Wk 2)	Treatment	Feed Day	No	16:10	16:00 - 16:59	Evening	38,0		111,0	111,0	19,15		159,48
2019/05/24	CH2206	Male	Three y/o	Treatment (Wk 2)	Treatment	Feed Day	No	16:10	16:00 - 16:59	Evening	38,1	61	23,0	23,0	19,26	556,01	105,09
2019/05/24	CH2205	Male	Three y/o	Treatment (Wk 2)	Treatment	Feed Day	No	16:15	16:00 - 16:59	Evening	38,0	137	136,6	136,6	19,15	615,83	166,74
2019/05/24	CH2206	Male	Three y/o	Treatment (Wk 2)	Treatment	Feed Day	No	16:15	16:00 - 16:59	Evening	38,1	60	27,0	27,0	19,26	554,67	110,58
2019/05/24	CH2205	Male	Three y/o	Treatment (Wk 2)	Treatment	Feed Day	No	16:20	16:00 - 16:59	Evening	38,0	180	121,4	121,4	19,15	633,61	162,61
2019/05/24	CH2206	Male	Three y/o	Treatment (Wk 2)	Treatment	Feed Day	No	16:20	16:00 - 16:59	Evening	38,1	62	21,0	21,0	19,26	557,33	101,98
2019/05/24	CH2205	Male	Three y/o	Treatment (Wk 2)	Treatment	Feed Day	No	16:25	16:00 - 16:59	Evening	38,1	108	198,2	198,2	19,26	599,39	179,80
2019/05/24	CH2206	Male	Three y/o	Treatment (Wk 2)	Treatment	Feed Day	No	16:25	16:00 - 16:59	Evening	38,1	59	22,6	22,6	19,26	553,30	104,49
2019/05/24	CH2205	Male	Three y/o	Treatment (Wk 2)	Treatment	Feed Day	No	16:30	16:00 - 16:59	Evening	38,1	185	146,4	146,4	19,26	635,34	169,17
2019/05/24	CH2206	Male	Three y/o	Treatment (Wk 2)	Treatment	Feed Day	No	16:30	16:00 - 16:59	Evening	38,1	48	41,6	41,6	19,26	536,04	125,44
2019/05/24	CH2205	Male	Three y/o	Treatment (Wk 2)	Treatment	Feed Day	No	16:35	16:00 - 16:59	Evening	38,1	74	22,2	22,2	19,26	571,33	103,88
2019/05/24	CH2206	Male	Three y/o	Treatment (Wk 2)	Treatment	Feed Day	No	16:35	16:00 - 16:59	Evening	38,0	48	26,6	26,6	19,15	536,04	110,07
2019/05/24	CH2205	Male	Three y/o	Treatment (Wk 2)	Treatment	Feed Day	No	16:40	16:00 - 16:59	Evening	38,1		18,2	18,2	19,26		97,10
2019/05/24	CH2206	Male	Three y/o	Treatment (Wk 2)	Treatment	Feed Day	No	16:40	16:00 - 16:59	Evening	38,0	54	40,8	40,8	19,15	545,99	124,77
2019/05/24	CH2205	Male	Three y/o	Treatment (Wk 2)	Treatment	Feed Day	No	16:45	16:00 - 16:59	Evening	37,9		30,4	30,4	19,05		114,65
2019/05/24	CH2206	Male	Three y/o	Treatment (Wk 2)	Treatment	Feed Day	No	16:45	16:00 - 16:59	Evening	37,9	70	53,4	53,4	19,05	566,99	134,06
2019/05/24	CH2205	Male	Three y/o	Treatment (Wk 2)	Treatment	Feed Day	No	16:50	16:00 - 16:59	Evening	37,9		32,2	32,2	19,05		116,63
2019/05/24	CH2206	Male	Three y/o	Treatment (Wk 2)	Treatment	Feed Day	No	16:50	16:00 - 16:59	Evening	37,9	40	43,6	43,6	19,05	520,09	127,06
2019/05/24	CH2205	Male	Three y/o	Treatment (Wk 2)	Treatment	Feed Day	No	16:55	16:00 - 16:59	Evening	37,9	68	25,0	25,0	19,05	564,71	107,94
2019/05/24	CH2206	Male	Three y/o	Treatment (Wk 2)	Treatment	Feed Day	No	16:55	16:00 - 16:59	Evening	37,8	75	41,0	41,0	18,95	572,36	124,94
2019/05/24	CH2205	Male	Three y/o	Treatment (Wk 2)	Treatment	Feed Day	No	17:00	17:00 - 17:59	Evening	37,9		37,0	37,0	19,05		121,40
2019/05/24	CH2206	Male	Three y/o	Treatment (Wk 2)	Treatment	Feed Day	No	17:00	17:00 - 17:59	Evening	37,8	44	104,0	104,0	18,95	528,51	157,21
2019/05/24	CH2205	Male	Three y/o	Treatment (Wk 2)	Treatment	Feed Day	No	17:05	17:00 - 17:59	Evening	37,7	104	27,2	27,2	18,85	596,69	110,83
2019/05/24	CH2206	Male	Three y/o	Treatment (Wk 2)	Treatment	Feed Day	No	17:05	17:00 - 17:59	Evening	37,8	103	58,4	58,4	18,95	596,00	137,16
2019/05/24	CH2205	Male	Three y/o	Treatment (Wk 2)	Treatment	Feed Day	No	17:10	17:00 - 17:59	Evening	37,7	90	31,6	31,6	18,85	586,16	115,98

2019/05/24	CH2206	Male	Three y/o	Treatment (Wk 2)	Treatment	Feed Day	No	17:10	17:00 - 17:59	Evening	37,8	48	54,6	54,6	18,95	536,04	134,83
2019/05/24	CH2205	Male	Three y/o	Treatment (Wk 2)	Treatment	Feed Day	No	17:15	17:00 - 17:59	Evening	37,9		26,6	26,6	19,05		110,07
2019/05/24	CH2206	Male	Three y/o	Treatment (Wk 2)	Treatment	Feed Day	No	17:15	17:00 - 17:59	Evening	37,7	47	48,8	48,8	18,85	534,23	130,95
2019/05/24	CH2205	Male	Three y/o	Treatment (Wk 2)	Treatment	Feed Day	No	17:20	17:00 - 17:59	Evening	37,9	61	7,8	7,8	19,05	556,01	68,36
2019/05/24	CH2206	Male	Three y/o	Treatment (Wk 2)	Treatment	Feed Day	No	17:20	17:00 - 17:59	Evening	37,7	47	64,6	64,6	18,85	534,23	140,65
2019/05/24	CH2205	Male	Three y/o	Treatment (Wk 2)	Treatment	Feed Day	No	17:25	17:00 - 17:59	Evening	37,9	68	9,0	9,0	19,05	564,71	73,19
2019/05/24	CH2206	Male	Three y/o	Treatment (Wk 2)	Treatment	Feed Day	No	17:25	17:00 - 17:59	Evening	37,5	88	63,8	63,8	18,65	584,49	140,22
2019/05/24	CH2205	Male	Three y/o	Treatment (Wk 2)	Treatment	Feed Day	No	17:30	17:00 - 17:59	Evening	37,9	61	8,0	8,0	19,05	556,01	69,22
2019/05/24	CH2206	Male	Three y/o	Treatment (Wk 2)	Treatment	Feed Day	No	17:30	17:00 - 17:59	Evening	37,7	42	63,0	63,0	18,85	524,42	139,78
2019/05/24	CH2205	Male	Three y/o	Treatment (Wk 2)	Treatment	Feed Day	No	17:35	17:00 - 17:59	Evening	37,9	56	6,0	6,0	19,05	549,01	59,53
2019/05/24	CH2206	Male	Three y/o	Treatment (Wk 2)	Treatment	Feed Day	No	17:35	17:00 - 17:59	Evening	37,5	41	58,0	58,0	18,65	522,29	136,92
2019/05/24	CH2205	Male	Three y/o	Treatment (Wk 2)	Treatment	Feed Day	No	17:40	17:00 - 17:59	Evening	37,9	66	13,4	13,4	19,05	562,34	86,68
2019/05/24	CH2206	Male	Three y/o	Treatment (Wk 2)	Treatment	Feed Day	No	17:40	17:00 - 17:59	Evening	37,4	46	58,2	58,2	18,55	532,38	137,04
2019/05/24	CH2205	Male	Three y/o	Treatment (Wk 2)	Treatment	Feed Day	No	17:45	17:00 - 17:59	Evening	37,9		29,4	29,4	19,05		113,50
2019/05/24	CH2206	Male	Three y/o	Treatment (Wk 2)	Treatment	Feed Day	No	17:45	17:00 - 17:59	Evening	37,5	102	156,8	156,8	18,65	595,30	171,57
2019/05/24	CH2205	Male	Three y/o	Treatment (Wk 2)	Treatment	Feed Day	No	17:50	17:00 - 17:59	Evening	37,9	87	29,0	29,0	19,05	583,64	113,03
2019/05/24	CH2206	Male	Three y/o	Treatment (Wk 2)	Treatment	Feed Day	No	17:50	17:00 - 17:59	Evening	37,7	119	99,4	99,4	18,85	606,20	155,63
2019/05/24	CH2205	Male	Three y/o	Treatment (Wk 2)	Treatment	Feed Day	No	17:55	17:00 - 17:59	Evening	37,9		34,0	34,0	19,05		118,49
2019/05/24	CH2206	Male	Three y/o	Treatment (Wk 2)	Treatment	Feed Day	No	17:55	17:00 - 17:59	Evening	37,8	112	110,0	110,0	18,95	601,96	159,17
2019/05/24	CH2205	Male	Three y/o	Treatment (Wk 2)	Treatment	Feed Day	No	18:00	18:00 - 18:59	Evening	37,9		27,8	27,8	19,05		111,58
2019/05/24	CH2206	Male	Three y/o	Treatment (Wk 2)	Treatment	Feed Day	No	18:00	18:00 - 18:59	Evening	37,9	108	59,6	59,6	19,05	599,39	137,86
2019/05/24	CH2205	Male	Three y/o	Treatment (Wk 2)	Treatment	Feed Day	No	18:05	18:00 - 18:59	Evening	37,6	188	36,2	36,2	18,75	636,34	120,65
2019/05/24	CH2206	Male	Three y/o	Treatment (Wk 2)	Treatment	Feed Day	No	18:05	18:00 - 18:59	Evening	37,9	125	55,2	55,2	19,05	609,60	135,21
2019/05/24	CH2205	Male	Three y/o	Treatment (Wk 2)	Treatment	Feed Day	No	18:10	18:00 - 18:59	Evening	37,5		27,4	27,4	18,65		111,09
2019/05/24	CH2206	Male	Three y/o	Treatment (Wk 2)	Treatment	Feed Day	No	18:10	18:00 - 18:59	Evening	37,8	49	59,6	59,6	18,95	537,80	137,86
2019/05/24	CH2205	Male	Three y/o	Treatment (Wk 2)	Treatment	Feed Day	No	18:15	18:00 - 18:59	Evening	37,5		27,6	27,6	18,65		111,33
2019/05/24	CH2206	Male	Three y/o	Treatment (Wk 2)	Treatment	Feed Day	No	18:15	18:00 - 18:59	Evening	37,5	46	57,8	57,8	18,65	532,38	136,80
2019/05/24	CH2205	Male	Three y/o	Treatment (Wk 2)	Treatment	Feed Day	No	18:20	18:00 - 18:59	Evening	37,7		23,0	23,0	18,85		105,09
2019/05/24	CH2206	Male	Three y/o	Treatment (Wk 2)	Treatment	Feed Day	No	18:20	18:00 - 18:59	Evening	37,7	49	52,4	52,4	18,85	537,80	133,41
2019/05/24	CH2205	Male	Three y/o	Treatment (Wk 2)	Treatment	Feed Day	No	18:25	18:00 - 18:59	Evening	37,7		32,6	32,6	18,85		117,05
2019/05/24	CH2206	Male	Three y/o	Treatment (Wk 2)	Treatment	Feed Day	No	18:25	18:00 - 18:59	Evening	37,7	49	40,6	40,6	18,85	537,80	124,60
2019/05/24	CH2205	Male	Three y/o	Treatment (Wk 2)	Treatment	Feed Day	No	18:30	18:00 - 18:59	Evening	37,7	81	38,2	38,2	18,85	578,26	122,50
2019/05/24	CH2206	Male	Three y/o	Treatment (Wk 2)	Treatment	Feed Day	No	18:30	18:00 - 18:59	Evening	37,8	46	42,6	42,6	18,95	532,38	126,26
2019/05/24	CH2205	Male	Three y/o	Treatment (Wk 2)	Treatment	Feed Day	No	18:35	18:00 - 18:59	Evening	37,6	131	34,0	34,0	18,75	612,80	118,49
2019/05/24	CH2206	Male	Three y/o	Treatment (Wk 2)	Treatment	Feed Day	No	18:35	18:00 - 18:59	Evening	37,8	44	43,6	43,6	18,95	528,51	127,06
2019/05/24	CH2205	Male	Three y/o	Treatment (Wk 2)	Treatment	Feed Day	No	18:40	18:00 - 18:59	Evening	37,6	65	30,0	30,0	18,75	561,12	114,20
2019/05/24	CH2206	Male	Three y/o	Treatment (Wk 2)	Treatment	Feed Day	No	18:40	18:00 - 18:59	Evening	37,8	51	45,4	45,4	18,95	541,20	128,45
2019/05/24	CH2205	Male	Three y/o	Treatment (Wk 2)	Treatment	Feed Day	No	18:45	18:00 - 18:59	Evening	37,6		26,2	26,2	18,75		109,55
2019/05/24	CH2206	Male	Three y/o	Treatment (Wk 2)	Treatment	Feed Day	No	18:45	18:00 - 18:59	Evening	37,7	46	42,0	42,0	18,85	532,38	125,77
2019/05/24	CH2205	Male	Three y/o	Treatment (Wk 2)	Treatment	Feed Day	No	18:50	18:00 - 18:59	Evening	37,4		24,2	24,2	18,55		106,83
2019/05/24	CH2206	Male	Three y/o	Treatment (Wk 2)	Treatment	Feed Day	No	18:50	18:00 - 18:59	Evening	37,7	43	37,0	37,0	18,85	526,50	121,40
2019/05/24	CH2205	Male	Three y/o	Treatment (Wk 2)	Treatment	Feed Day	No	18:55	18:00 - 18:59	Evening	37,6		21,2	21,2	18,75		102,31
2019/05/24	CH2206	Male	Three y/o	Treatment (Wk 2)	Treatment	Feed Day	No	18:55	18:00 - 18:59	Evening	37,7	45	22,8	22,8	18,85	530,47	104,79
2019/05/24	CH2205	Male	Three y/o	Treatment (Wk 2)	Treatment	Feed Day	No	19:00	19:00 - 19:59	Evening	37,6		20,4	20,4	18,75		100,99
2019/05/24	CH2206	Male	Three y/o	Treatment (Wk 2)	Treatment	Feed Day	No	19:00	19:00 - 19:59	Evening	37,7	46	36,4	36,4	18,85	532,38	120,84
2019/05/24	CH2205	Male	Three y/o	Treatment (Wk 2)	Treatment	Feed Day	No	19:05	19:00 - 19:59	Evening	37,6		21,4	21,4	18,75		102,63
2019/05/24	CH2206	Male	Three y/o	Treatment (Wk 2)	Treatment	Feed Day	No	19:05	19:00 - 19:59	Evening	37,7	62	37,2	37,2	18,85	557,33	121,59
2019/05/24	CH2205	Male	Three y/o	Treatment (Wk 2)	Treatment	Feed Day	No	19:10	19:00 - 19:59	Evening	37,6		24,6	24,6	18,75		107,39
2019/05/24	CH2206	Male	Three y/o	Treatment (Wk 2)	Treatment	Feed Day	No	19:10	19:00 - 19:59	Evening	37,5	49	38,0	38,0	18,65	537,80	122,32
2019/05/24	CH2205	Male	Three y/o	Treatment (Wk 2)	Treatment	Feed Day	No	19:15	19:00 - 19:59	Evening	37,7		25,0	25,0	18,85		107,94
2019/05/24	CH2206	Male	Three y/o	Treatment (Wk 2)	Treatment	Feed Day	No	19:15	19:00 - 19:59	Evening	37,5	75	50,2	50,2	18,65	572,36	131,92
2019/05/24	CH2205	Male	Three y/o	Treatment (Wk 2)	Treatment	Feed Day	No	19:20	19:00 - 19:59	Evening	37,7	94	20,4	20,4	18,85	589,37	100,99
2019/05/24	CH2206	Male	Three y/o	Treatment (Wk 2)	Treatment	Feed Day	No	19:20	19:00 - 19:59	Evening	37,7	48	43,0	43,0	18,85	536,04	126,58
2019/05/24	CH2205	Male	Three y/o	Treatment (Wk 2)	Treatment	Feed Day	No	19:25	19:00 - 19:59	Evening	37,7	89	12,8	12,8	18,85	585,33	85,12
2019/05/24	CH2206	Male	Three y/o	Treatment (Wk 2)	Treatment	Feed Day	No	19:25	19:00 - 19:59	Evening	37,7	48	49,0	49,0	18,85	536,04	131,09
2019/05/24	CH2205	Male	Three y/o	Treatment (Wk 2)	Treatment	Feed Day	No	19:30	19:00 - 19:59	Evening	37,7	61	20,2	20,2	18,85	556,01	100,65
2019/05/24	CH2206	Male	Three y/o	Treatment (Wk 2)	Treatment	Feed Day	No	19:30	19:00 - 19:59	Evening	37,7	50	44,4	44,4	18,85	539,52	127,69
2019/05/24	CH2205	Male	Three y/o	Treatment (Wk 2)	Treatment	Feed Day	No	19:35	19:00 - 19:59	Evening	37,7	60	20,8	20,8	18,85	554,67	101,65
2019/05/24	CH2206	Male	Three y/o	Treatment (Wk 2)	Treatment	Feed Day	No	19:35	19:00 - 19:59	Evening	37,7	42	44,8	44,8	18,85	524,42	127,99
2019/05/24	CH2205	Male	Three y/o	Treatment (Wk 2)	Treatment	Feed Day	No	19:40	19:00 - 19:59	Evening	37,7	86	15,6	15,6	18,85	582,77	91,85
2019/05/24	CH2206	Male	Three y/o	Treatment (Wk 2)	Treatment	Feed Day	No	19:40	19:00 - 19:59	Evening	37,7	45	44,2	44,2	18,85	530,47	127,53
2019/05/24	CH2205	Male	Three y/o	Treatment (Wk 2)	Treatment	Feed Day	No	19:45	19:00 - 19:59	Evening	37,7	82	16,0	16,0	18,85	579,19	92,71
2019/05/24	CH2206	Male	Three y/o	Treatment (Wk 2)	Treatment	Feed Day	No	19:45	19:00 - 19:59	Evening	37,7	70	44,4	44,4	18,85	566,99	127,69
2019/05/24	CH2205	Male	Three y/o	Treatment (Wk 2)	Treatment	Feed Day	No	19:50	19:00 - 19:59	Evening	37,7		31,8	31,8	18,85		116,20
2019/05/24	CH2206	Male	Three y/o	Treatment (Wk 2)	Treatment	Feed Day	No	19:50	19:00 - 19:59	Evening	37,7	54	49,0	49,0	18,85	545,99	131,09
2019/05/24	CH2205	Male	Three y/o	Treatment (Wk 2)	Treatment	Feed Day	No	19:55	19:00 - 19:59	Evening	37,7		26,8	26,8	18,85		110,33
2019/05/24	CH2206	Male	Three y/o	Treatment (Wk 2)	Treatment	Feed Day	No	19:55	19:00 - 19:59	Evening	37,7	69	43,8	43,8	18,85	565,86	127,22
2019/05/24	CH2205	Male	Three y/o	Treatment (Wk 2)	Treatment	Feed Day	No	20:00	20:00 - 20:59	Night	37,7		23,2	23,2	18,85		105,39
2019/05/24	CH2206	Male	Three y/o	Treatment (Wk 2)	Treatment	Feed Day	No	20:00	20:00 - 20:59	Night	37,7	43	33,4	33,4	18,85	526,50	117,88
2019/05/24	CH2205	Male	Three y/o	Treatment (Wk 2)	Treatment	Feed Day	No	20:05	20:00 - 20:59	Night	37,8	92	21,8	21,8	18,95	587,78	103,26

2019/05/24	CH2206	Male	Three y/o	Treatment (Wk 2)	Treatment	Feed Day	No	20:05	20:00 - 20:59	Night	37,7	44	26,4	26,4	18,85	528,51	109,81
2019/05/24	CH2205	Male	Three y/o	Treatment (Wk 2)	Treatment	Feed Day	No	20:10	20:00 - 20:59	Night	37,7	57	23,2	23,2	18,85	550,47	105,39
2019/05/24	CH2206	Male	Three y/o	Treatment (Wk 2)	Treatment	Feed Day	No	20:10	20:00 - 20:59	Night	37,7	43	22,0	22,0	18,85	526,50	103,57
2019/05/24	CH2205	Male	Three y/o	Treatment (Wk 2)	Treatment	Feed Day	No	20:15	20:00 - 20:59	Night	37,7		22,2	22,2	18,85		103,88
2019/05/24	CH2206	Male	Three y/o	Treatment (Wk 2)	Treatment	Feed Day	No	20:15	20:00 - 20:59	Night	37,7	50	8,8	8,8	18,85	539,52	72,43
2019/05/24	CH2205	Male	Three y/o	Treatment (Wk 2)	Treatment	Feed Day	No	20:20	20:00 - 20:59	Night	37,6	94	21,4	21,4	18,75	589,37	102,63
2019/05/24	CH2206	Male	Three y/o	Treatment (Wk 2)	Treatment	Feed Day	No	20:20	20:00 - 20:59	Night	37,7	43	14,2	14,2	18,85	526,50	88,65
2019/05/24	CH2205	Male	Three y/o	Treatment (Wk 2)	Treatment	Feed Day	No	20:25	20:00 - 20:59	Night	37,6		22,4	22,4	18,75		104,19
2019/05/24	CH2206	Male	Three y/o	Treatment (Wk 2)	Treatment	Feed Day	No	20:25	20:00 - 20:59	Night	37,6	41	13,8	13,8	18,75	522,29	87,68
2019/05/24	CH2205	Male	Three y/o	Treatment (Wk 2)	Treatment	Feed Day	No	20:30	20:00 - 20:59	Night	37,6	90	20,0	20,0	18,75	586,16	100,31
2019/05/24	CH2206	Male	Three y/o	Treatment (Wk 2)	Treatment	Feed Day	No	20:30	20:00 - 20:59	Night	37,5	65	16,6	16,6	18,65	561,12	93,96
2019/05/24	CH2205	Male	Three y/o	Treatment (Wk 2)	Treatment	Feed Day	No	20:35	20:00 - 20:59	Night	37,6	62	24,0	24,0	18,75	557,33	106,55
2019/05/24	CH2206	Male	Three y/o	Treatment (Wk 2)	Treatment	Feed Day	No	20:35	20:00 - 20:59	Night	37,5	44	18,0	18,0	18,65	528,51	96,72
2019/05/24	CH2205	Male	Three y/o	Treatment (Wk 2)	Treatment	Feed Day	No	20:40	20:00 - 20:59	Night	37,5	88	23,0	23,0	18,65	584,49	105,09
2019/05/24	CH2206	Male	Three y/o	Treatment (Wk 2)	Treatment	Feed Day	No	20:40	20:00 - 20:59	Night	37,5	43	17,8	17,8	18,65	526,50	96,34
2019/05/24	CH2205	Male	Three y/o	Treatment (Wk 2)	Treatment	Feed Day	No	20:45	20:00 - 20:59	Night	37,5	82	25,2	25,2	18,65	579,19	108,22
2019/05/24	CH2206	Male	Three y/o	Treatment (Wk 2)	Treatment	Feed Day	No	20:45	20:00 - 20:59	Night	37,5	52	19,4	19,4	18,65	542,83	99,28
2019/05/24	CH2205	Male	Three y/o	Treatment (Wk 2)	Treatment	Feed Day	No	20:50	20:00 - 20:59	Night	37,5	63	19,2	19,2	18,65	558,62	98,92
2019/05/24	CH2206	Male	Three y/o	Treatment (Wk 2)	Treatment	Feed Day	No	20:50	20:00 - 20:59	Night	37,5	49	18,6	18,6	18,65	537,80	97,84
2019/05/24	CH2205	Male	Three y/o	Treatment (Wk 2)	Treatment	Feed Day	No	20:55	20:00 - 20:59	Night	37,5	71	18,8	18,8	18,65	568,10	98,20
2019/05/24	CH2206	Male	Three y/o	Treatment (Wk 2)	Treatment	Feed Day	No	20:55	20:00 - 20:59	Night	37,5	39	16,4	16,4	18,65	517,82	93,55
2019/05/24	CH2205	Male	Three y/o	Treatment (Wk 2)	Treatment	Feed Day	No	21:00	21:00 - 21:59	Night	37,7	69	18,6	18,6	18,85	565,86	97,84
2019/05/24	CH2206	Male	Three y/o	Treatment (Wk 2)	Treatment	Feed Day	No	21:00	21:00 - 21:59	Night	37,5	40	19,6	19,6	18,65	520,09	99,63
2019/05/24	CH2205	Male	Three y/o	Treatment (Wk 2)	Treatment	Feed Day	No	21:05	21:00 - 21:59	Night	37,7	72	15,8	15,8	18,85	569,20	92,28
2019/05/24	CH2206	Male	Three y/o	Treatment (Wk 2)	Treatment	Feed Day	No	21:05	21:00 - 21:59	Night	37,5	56	21,6	21,6	18,65	549,01	102,94
2019/05/24	CH2205	Male	Three y/o	Treatment (Wk 2)	Treatment	Feed Day	No	21:10	21:00 - 21:59	Night	37,7	51	19,2	19,2	18,85	541,20	98,92
2019/05/24	CH2206	Male	Three y/o	Treatment (Wk 2)	Treatment	Feed Day	No	21:10	21:00 - 21:59	Night	37,5	42	25,8	25,8	18,65	524,42	109,02
2019/05/24	CH2205	Male	Three y/o	Treatment (Wk 2)	Treatment	Feed Day	No	21:15	21:00 - 21:59	Night	37,9	97	59,8	59,8	19,05	591,66	137,98
2019/05/24	CH2206	Male	Three y/o	Treatment (Wk 2)	Treatment	Feed Day	No	21:15	21:00 - 21:59	Night	37,5	48	49,8	49,8	18,65	536,04	131,65
2019/05/24	CH2205	Male	Three y/o	Treatment (Wk 2)	Treatment	Feed Day	No	21:20	21:00 - 21:59	Night	37,8	104	18,8	18,8	18,95	596,69	98,20
2019/05/24	CH2206	Male	Three y/o	Treatment (Wk 2)	Treatment	Feed Day	No	21:20	21:00 - 21:59	Night	37,5	43	59,0	59,0	18,65	526,50	137,51
2019/05/24	CH2205	Male	Three y/o	Treatment (Wk 2)	Treatment	Feed Day	No	21:25	21:00 - 21:59	Night	37,7	64	23,8	23,8	18,85	559,88	106,26
2019/05/24	CH2206	Male	Three y/o	Treatment (Wk 2)	Treatment	Feed Day	No	21:25	21:00 - 21:59	Night	37,4	60	60,8	60,8	18,55	554,67	138,55
2019/05/24	CH2205	Male	Three y/o	Treatment (Wk 2)	Treatment	Feed Day	No	21:30	21:00 - 21:59	Night	37,6	98	20,4	20,4	18,75	592,41	100,99
2019/05/24	CH2206	Male	Three y/o	Treatment (Wk 2)	Treatment	Feed Day	No	21:30	21:00 - 21:59	Night	37,5		63,8	63,8	18,65		140,22
2019/05/24	CH2205	Male	Three y/o	Treatment (Wk 2)	Treatment	Feed Day	No	21:35	21:00 - 21:59	Night	37,6	51	24,2	24,2	18,75	541,20	106,83
2019/05/24	CH2206	Male	Three y/o	Treatment (Wk 2)	Treatment	Feed Day	No	21:35	21:00 - 21:59	Night	37,5	65	60,2	60,2	18,65	561,12	138,21
2019/05/24	CH2205	Male	Three y/o	Treatment (Wk 2)	Treatment	Feed Day	No	21:40	21:00 - 21:59	Night	37,6	86	22,8	22,8	18,75	582,77	104,79
2019/05/24	CH2206	Male	Three y/o	Treatment (Wk 2)	Treatment	Feed Day	No	21:40	21:00 - 21:59	Night	37,5	39	60,0	60,0	18,65	517,82	138,09
2019/05/24	CH2205	Male	Three y/o	Treatment (Wk 2)	Treatment	Feed Day	No	21:45	21:00 - 21:59	Night	37,5	60	21,6	21,6	18,65	554,67	102,94
2019/05/24	CH2206	Male	Three y/o	Treatment (Wk 2)	Treatment	Feed Day	No	21:45	21:00 - 21:59	Night	37,5	40	67,6	67,6	18,65	520,09	142,23
2019/05/24	CH2205	Male	Three y/o	Treatment (Wk 2)	Treatment	Feed Day	No	21:50	21:00 - 21:59	Night	37,5	91	5,4	5,4	18,65	586,98	55,99
2019/05/24	CH2206	Male	Three y/o	Treatment (Wk 2)	Treatment	Feed Day	No	21:50	21:00 - 21:59	Night	37,5	63	55,0	55,0	18,65	558,62	135,08
2019/05/24	CH2205	Male	Three y/o	Treatment (Wk 2)	Treatment	Feed Day	No	21:55	21:00 - 21:59	Night	37,6	57	3,2	3,2	18,75	550,47	38,49
2019/05/24	CH2206	Male	Three y/o	Treatment (Wk 2)	Treatment	Feed Day	No	21:55	21:00 - 21:59	Night	37,5	43	65,4	65,4	18,65	526,50	141,08
2019/05/24	CH2205	Male	Three y/o	Treatment (Wk 2)	Treatment	Feed Day	No	22:00	22:00 - 22:59	Night	37,6	80	9,6	9,6	18,75	577,31	75,37
2019/05/24	CH2206	Male	Three y/o	Treatment (Wk 2)	Treatment	Feed Day	No	22:00	22:00 - 22:59	Night	37,5	46	64,8	64,8	18,65	532,38	140,76
2019/05/24	CH2205	Male	Three y/o	Treatment (Wk 2)	Treatment	Feed Day	No	22:05	22:00 - 22:59	Night	37,7		5,0	5,0	18,85		53,41
2019/05/24	CH2206	Male	Three y/o	Treatment (Wk 2)	Treatment	Feed Day	No	22:05	22:00 - 22:59	Night	37,5	42	66,6	66,6	18,65	524,42	141,71
2019/05/24	CH2205	Male	Three y/o	Treatment (Wk 2)	Treatment	Feed Day	No	22:10	22:00 - 22:59	Night	37,7	96	6,0	6,0	18,85	590,91	59,53
2019/05/24	CH2206	Male	Three y/o	Treatment (Wk 2)	Treatment	Feed Day	No	22:10	22:00 - 22:59	Night	37,5	44	63,2	63,2	18,65	528,51	139,89
2019/05/24	CH2205	Male	Three y/o	Treatment (Wk 2)	Treatment	Feed Day	No	22:15	22:00 - 22:59	Night	37,9	92	4,2	4,2	19,05	587,78	47,57
2019/05/24	CH2206	Male	Three y/o	Treatment (Wk 2)	Treatment	Feed Day	No	22:15	22:00 - 22:59	Night	37,4	40	50,8	50,8	18,55	520,09	132,33
2019/05/24	CH2205	Male	Three y/o	Treatment (Wk 2)	Treatment	Feed Day	No	22:20	22:00 - 22:59	Night	37,9	98	7,8	7,8	19,05	592,41	68,36
2019/05/24	CH2206	Male	Three y/o	Treatment (Wk 2)	Treatment	Feed Day	No	22:20	22:00 - 22:59	Night	37,4	60	36,8	36,8	18,55	554,67	121,22
2019/05/24	CH2205	Male	Three y/o	Treatment (Wk 2)	Treatment	Feed Day	No	22:25	22:00 - 22:59	Night	37,7	84	4,4	4,4	18,85	581,01	49,12
2019/05/24	CH2206	Male	Three y/o	Treatment (Wk 2)	Treatment	Feed Day	No	22:25	22:00 - 22:59	Night	37,4	45	29,2	29,2	18,55	530,47	113,27
2019/05/24	CH2205	Male	Three y/o	Treatment (Wk 2)	Treatment	Feed Day	No	22:30	22:00 - 22:59	Night	37,7	82	12,2	12,2	18,85	579,19	83,49
2019/05/24	CH2206	Male	Three y/o	Treatment (Wk 2)	Treatment	Feed Day	No	22:30	22:00 - 22:59	Night	37,3	47	28,4	28,4	18,46	534,23	112,31
2019/05/24	CH2205	Male	Three y/o	Treatment (Wk 2)	Treatment	Feed Day	No	22:35	22:00 - 22:59	Night	37,7	62	10,2	10,2	18,85	557,33	77,43
2019/05/24	CH2206	Male	Three y/o	Treatment (Wk 2)	Treatment	Feed Day	No	22:35	22:00 - 22:59	Night	37,2	47	33,8	33,8	18,36	534,23	118,29
2019/05/24	CH2205	Male	Three y/o	Treatment (Wk 2)	Treatment	Feed Day	No	22:40	22:00 - 22:59	Night	37,9	70	4,8	4,8	19,05	566,99	52,04
2019/05/24	CH2206	Male	Three y/o	Treatment (Wk 2)	Treatment	Feed Day	No	22:40	22:00 - 22:59	Night	37,2	90	26,4	26,4	18,36	586,16	109,81
2019/05/24	CH2205	Male	Three y/o	Treatment (Wk 2)	Treatment	Feed Day	No	22:45	22:00 - 22:59	Night	37,9	71	4,4	4,4	19,05	568,10	49,12
2019/05/24	CH2206	Male	Three y/o	Treatment (Wk 2)	Treatment	Feed Day	No	22:45	22:00 - 22:59	Night	37,1	49	43,4	43,4	18,26	537,80	126,90
2019/05/24	CH2205	Male	Three y/o	Treatment (Wk 2)	Treatment	Feed Day	No	22:50	22:00 - 22:59	Night	37,9	83	7,4	7,4	19,05	580,11	66,59
2019/05/24	CH2206	Male	Three y/o	Treatment (Wk 2)	Treatment	Feed Day	No	22:50	22:00 - 22:59	Night	37,1	92	46,0	46,0	18,26	587,78	128,91
2019/05/24	CH2205	Male	Three y/o	Treatment (Wk 2)	Treatment	Feed Day	No	22:55	22:00 - 22:59	Night	38,0	76	9,6	9,6	19,15	573,39	75,37
2019/05/24	CH2206	Male	Three y/o	Treatment (Wk 2)	Treatment	Feed Day	No	22:55	22:00 - 22:59	Night	37,1	45	37,0	37,0	18,26	530,47	121,40
2019/05/24	CH2205	Male	Three y/o	Treatment (Wk 2)	Treatment	Feed Day	No	23:00	23:00 - 23:59	Night	38,0	66	10,4	10,4	19,15	562,34	78,08

	2019/05/24	CH2206	Male	Three y/o	Treatment (Wk 2)	Treatment	Feed Day	No	23:00	23:00 - 23:59	Night	37,2	48	28,4	28,4	18,36	536,04	112,31
	2019/05/24	CH2205	Male	Three y/o	Treatment (Wk 2)	Treatment	Feed Day	No	23:05	23:00 - 23:59	Night	38,0	87	16,0	16,0	19,15	583,64	92,71
	2019/05/24	CH2206	Male	Three y/o	Treatment (Wk 2)	Treatment	Feed Day	No	23:05	23:00 - 23:59	Night	37,1	55	46,0	46,0	18,26	547,52	128,91
	2019/05/24	CH2205	Male	Three y/o	Treatment (Wk 2)	Treatment	Feed Day	No	23:10	23:00 - 23:59	Night	37,9	70	13,2	13,2	19,05	566,99	86,17
	2019/05/24	CH2206	Male	Three y/o	Treatment (Wk 2)	Treatment	Feed Day	No	23:10	23:00 - 23:59	Night	37,1	48	37,6	37,6	18,26	536,04	121,96
	2019/05/24	CH2205	Male	Three y/o	Treatment (Wk 2)	Treatment	Feed Day	No	23:15	23:00 - 23:59	Night	37,9	78	14,2	14,2	19,05	575,38	88,65
	2019/05/24	CH2206	Male	Three y/o	Treatment (Wk 2)	Treatment	Feed Day	No	23:15	23:00 - 23:59	Night	37,1	45	24,2	24,2	18,26	530,47	106,83
	2019/05/24	CH2205	Male	Three y/o	Treatment (Wk 2)	Treatment	Feed Day	No	23:20	23:00 - 23:59	Night	37,7	67	17,0	17,0	18,85	563,53	94,77
	2019/05/24	CH2206	Male	Three y/o	Treatment (Wk 2)	Treatment	Feed Day	No	23:20	23:00 - 23:59	Night	37,1		19,2	19,2	18,26		98,92
	2019/05/24	CH2205	Male	Three y/o	Treatment (Wk 2)	Treatment	Feed Day	No	23:25	23:00 - 23:59	Night	37,7	87	11,0	11,0	18,85	583,64	79,98
	2019/05/24	CH2206	Male	Three y/o	Treatment (Wk 2)	Treatment	Feed Day	No	23:25	23:00 - 23:59	Night	37,0	73	22,2	22,2	18,16	570,27	103,88
	2019/05/24	CH2205	Male	Three y/o	Treatment (Wk 2)	Treatment	Feed Day	No	23:30	23:00 - 23:59	Night	37,7	63	13,8	13,8	18,85	558,62	87,68
	2019/05/24	CH2206	Male	Three y/o	Treatment (Wk 2)	Treatment	Feed Day	No	23:30	23:00 - 23:59	Night	37,0	44	21,2	21,2	18,16	528,51	102,31
	2019/05/24	CH2205	Male	Three y/o	Treatment (Wk 2)	Treatment	Feed Day	No	23:35	23:00 - 23:59	Night	37,7	65	13,2	13,2	18,85	561,12	86,17
	2019/05/24	CH2206	Male	Three y/o	Treatment (Wk 2)	Treatment	Feed Day	No	23:35	23:00 - 23:59	Night	37,0	53	19,0	19,0	18,16	544,43	98,56
	2019/05/24	CH2205	Male	Three y/o	Treatment (Wk 2)	Treatment	Feed Day	No	23:40	23:00 - 23:59	Night	37,7	64	14,4	14,4	18,85	559,88	89,12
	2019/05/24	CH2206	Male	Three y/o	Treatment (Wk 2)	Treatment	Feed Day	No	23:40	23:00 - 23:59	Night	36,9	81	19,2	19,2	18,06	578,26	98,92
	2019/05/24	CH2205	Male	Three y/o	Treatment (Wk 2)	Treatment	Feed Day	No	23:45	23:00 - 23:59	Night	37,7	80	13,8	13,8	18,85	577,31	87,68
	2019/05/24	CH2206	Male	Three y/o	Treatment (Wk 2)	Treatment	Feed Day	No	23:45	23:00 - 23:59	Night	36,9	50	19,4	19,4	18,06	539,52	99,28
	2019/05/24	CH2205	Male	Three y/o	Treatment (Wk 2)	Treatment	Feed Day	No	23:50	23:00 - 23:59	Night	37,7	77	15,6	15,6	18,85	574,39	91,85
	2019/05/24	CH2206	Male	Three y/o	Treatment (Wk 2)	Treatment	Feed Day	No	23:50	23:00 - 23:59	Night	36,9	50	17,0	17,0	18,06	539,52	94,77
	2019/05/24	CH2205	Male	Three y/o	Treatment (Wk 2)	Treatment	Feed Day	No	23:55	23:00 - 23:59	Night	37,7	69	13,6	13,6	18,85	565,86	87,18
	2019/05/24	CH2206	Male	Three y/o	Treatment (Wk 2)	Treatment	Feed Day	No	23:55	23:00 - 23:59	Night	36,9	48	16,8	16,8	18,06	536,04	94,37
11	2019/05/25	CH2205	Male	Three y/o	Treatment (Wk 2)	Treatment	Fast Day	No	00:00	00:00 - 00:59	Early Morning	37,7	63	14,2	14,2	18,85	558,62	88,65
	2019/05/25	CH2206	Male	Three y/o	Treatment (Wk 2)	Treatment	Fast Day	No	00:00	00:00 - 00:59	Early Morning	36,9	43	21,0	21,0	18,06	526,50	101,98
	2019/05/25	CH2205	Male	Three y/o	Treatment (Wk 2)	Treatment	Fast Day	No	00:05	00:00 - 00:59	Early Morning	37,7	67	17,4	17,4	18,85	563,53	95,56
	2019/05/25	CH2206	Male	Three y/o	Treatment (Wk 2)	Treatment	Fast Day	No	00:05	00:00 - 00:59	Early Morning	37,0	145	11,0	11,0	18,16	619,62	79,98
	2019/05/25	CH2205	Male	Three y/o	Treatment (Wk 2)	Treatment	Fast Day	No	00:10	00:00 - 00:59	Early Morning	37,7	69	20,8	20,8	18,85	565,86	101,65
	2019/05/25	CH2206	Male	Three y/o	Treatment (Wk 2)	Treatment	Fast Day	No	00:10	00:00 - 00:59	Early Morning	36,9	49	5,4	5,4	18,06	537,80	55,99
	2019/05/25	CH2205	Male	Three y/o	Treatment (Wk 2)	Treatment	Fast Day	No	00:15	00:00 - 00:59	Early Morning	37,7	72	21,0	21,0	18,85	569,20	101,98
	2019/05/25	CH2206	Male	Three y/o	Treatment (Wk 2)	Treatment	Fast Day	No	00:15	00:00 - 00:59	Early Morning	36,9	46	13,2	13,2	18,06	532,38	86,17
	2019/05/25	CH2205	Male	Three y/o	Treatment (Wk 2)	Treatment	Fast Day	No	00:20	00:00 - 00:59	Early Morning	37,7	70	17,2	17,2	18,85	566,99	95,17
	2019/05/25	CH2206	Male	Three y/o	Treatment (Wk 2)	Treatment	Fast Day	No	00:20	00:00 - 00:59	Early Morning	36,9	42	22,2	22,2	18,06	524,42	103,88
	2019/05/25	CH2205	Male	Three y/o	Treatment (Wk 2)	Treatment	Fast Day	No	00:25	00:00 - 00:59	Early Morning	37,7	61	17,6	17,6	18,85	556,01	95,95
	2019/05/25	CH2206	Male	Three y/o	Treatment (Wk 2)	Treatment	Fast Day	No	00:25	00:00 - 00:59	Early Morning	36,9	55	28,6	28,6	18,06	547,52	112,56
	2019/05/25	CH2205	Male	Three y/o	Treatment (Wk 2)	Treatment	Fast Day	No	00:30	00:00 - 00:59	Early Morning	37,7	65	16,0	16,0	18,85	561,12	92,71
	2019/05/25	CH2206	Male	Three y/o	Treatment (Wk 2)	Treatment	Fast Day	No	00:30	00:00 - 00:59	Early Morning	37,0	40	36,8	36,8	18,16	520,09	121,22
	2019/05/25	CH2205	Male	Three y/o	Treatment (Wk 2)	Treatment	Fast Day	No	00:35	00:00 - 00:59	Early Morning	37,8	71	18,4	18,4	18,95	568,10	97,47
	2019/05/25	CH2206	Male	Three y/o	Treatment (Wk 2)	Treatment	Fast Day	No	00:35	00:00 - 00:59	Early Morning	37,0	44	39,6	39,6	18,16	528,51	123,74
	2019/05/25	CH2205	Male	Three y/o	Treatment (Wk 2)	Treatment	Fast Day	No	00:40	00:00 - 00:59	Early Morning	37,9	88	22,4	22,4	19,05	584,49	104,19
	2019/05/25	CH2206	Male	Three y/o	Treatment (Wk 2)	Treatment	Fast Day	No	00:40	00:00 - 00:59	Early Morning	37,0	55	32,8	32,8	18,16	547,52	117,26
	2019/05/25	CH2205	Male	Three y/o	Treatment (Wk 2)	Treatment	Fast Day	No	00:45	00:00 - 00:59	Early Morning	37,9	67	21,6	21,6	19,05	563,53	102,94
	2019/05/25	CH2206	Male	Three y/o	Treatment (Wk 2)	Treatment	Fast Day	No	00:45	00:00 - 00:59	Early Morning	37,0	63	36,4	36,4	18,16	558,62	120,84
	2019/05/25	CH2205	Male	Three y/o	Treatment (Wk 2)	Treatment	Fast Day	No	00:50	00:00 - 00:59	Early Morning	37,9	61	19,6	19,6	19,05	556,01	99,63
	2019/05/25	CH2206	Male	Three y/o	Treatment (Wk 2)	Treatment	Fast Day	No	00:50	00:00 - 00:59	Early Morning	37,1	43	23,2	23,2	18,26	526,50	105,39
	2019/05/25	CH2205	Male	Three y/o	Treatment (Wk 2)	Treatment	Fast Day	No	00:55	00:00 - 00:59	Early Morning	37,9	60	15,6	15,6	19,05	554,67	91,85
	2019/05/25	CH2206	Male	Three y/o	Treatment (Wk 2)	Treatment	Fast Day	No	00:55	00:00 - 00:59	Early Morning	37,1	47	11,0	11,0	18,26	534,23	79,98
	2019/05/25	CH2205	Male	Three y/o	Treatment (Wk 2)	Treatment	Fast Day	No	01:00	01:00 - 01:59	Early Morning	37,9	63	15,6	15,6	19,05	558,62	91,85
	2019/05/25	CH2206	Male	Three y/o	Treatment (Wk 2)	Treatment	Fast Day	No	01:00	01:00 - 01:59	Early Morning	37,0	47	10,6	10,6	18,16	534,23	78,73
	2019/05/25	CH2205	Male	Three y/o	Treatment (Wk 2)	Treatment	Fast Day	No	01:05	01:00 - 01:59	Early Morning	37,9	89	35,8	35,8	19,05	585,33	120,27
	2019/05/25	CH2206	Male	Three y/o	Treatment (Wk 2)	Treatment	Fast Day	No	01:05	01:00 - 01:59	Early Morning	36,9	71	20,8	20,8	18,06	568,10	101,65
	2019/05/25	CH2205	Male	Three y/o	Treatment (Wk 2)	Treatment	Fast Day	No	01:10	01:00 - 01:59	Early Morning	37,9	97	21,0	21,0	19,05	591,66	101,98
	2019/05/25	CH2206	Male	Three y/o	Treatment (Wk 2)	Treatment	Fast Day	No	01:10	01:00 - 01:59	Early Morning	36,9	88	47,4	47,4	18,06	584,49	129,94
	2019/05/25	CH2205	Male	Three y/o	Treatment (Wk 2)	Treatment	Fast Day	No	01:15	01:00 - 01:59	Early Morning	37,7		24,8	24,8	18,85		107,67
	2019/05/25	CH2206	Male	Three y/o	Treatment (Wk 2)	Treatment	Fast Day	No	01:15	01:00 - 01:59	Early Morning	36,9	80	58,4	58,4	18,06	577,31	137,16
	2019/05/25	CH2205	Male	Three y/o	Treatment (Wk 2)	Treatment	Fast Day	No	01:20	01:00 - 01:59	Early Morning	37,6	75	22,8	22,8	18,75	572,36	104,79
	2019/05/25	CH2206	Male	Three y/o	Treatment (Wk 2)	Treatment	Fast Day	No	01:20	01:00 - 01:59	Early Morning	36,9	43	57,8	57,8	18,06	526,50	136,80
	2019/05/25	CH2205	Male	Three y/o	Treatment (Wk 2)	Treatment	Fast Day	No	01:25	01:00 - 01:59	Early Morning	37,5		21,8	21,8	18,65		103,26
	2019/05/25	CH2206	Male	Three y/o	Treatment (Wk 2)	Treatment	Fast Day	No	01:25	01:00 - 01:59	Early Morning	37,0	70	62,4	62,4	18,16	566,99	139,45
	2019/05/25	CH2205	Male	Three y/o	Treatment (Wk 2)	Treatment	Fast Day	No	01:30	01:00 - 01:59	Early Morning	37,5	95	21,4	21,4	18,65	590,14	102,63
	2019/05/25	CH2206	Male	Three y/o	Treatment (Wk 2)	Treatment	Fast Day	No	01:30	01:00 - 01:59	Early Morning	37,2	73	65,4	65,4	18,36	570,27	141,08
	2019/05/25	CH2205	Male	Three y/o	Treatment (Wk 2)	Treatment	Fast Day	No	01:35	01:00 - 01:59	Early Morning	37,5	55	22,0	22,0	18,65	547,52	103,57
	2019/05/25	CH2206	Male	Three y/o	Treatment (Wk 2)	Treatment	Fast Day	No	01:35	01:00 - 01:59	Early Morning	37,2	60	61,6	61,6	18,36	554,67	139,00
	2019/05/25	CH2205	Male	Three y/o	Treatment (Wk 2)	Treatment	Fast Day	No	01:40	01:00 - 01:59	Early Morning	37,4	93	18,4	18,4	18,55	588,58	97,47
	2019/05/25	CH2206	Male	Three y/o	Treatment (Wk 2)	Treatment	Fast Day	No	01:40	01:00 - 01:59	Early Morning	37,3	41	58,6	58,6	18,46	522,29	137,28
	2019/05/25	CH2205	Male	Three y/o	Treatment (Wk 2)	Treatment	Fast Day	No	01:45	01:00 - 01:59	Early Morning	37,4	84	29,4	29,4	18,55	581,01	113,50
	2019/05/25	CH2206	Male	Three y/o	Treatment (Wk 2)	Treatment	Fast Day	No	01:45	01:00 - 01:59	Early Morning	37,3	90	52,6	52,6	18,46	586,16	133,54
	2019/05/25	CH2205	Male	Three y/o	Treatment (Wk 2)	Treatment	Fast Day	No	01:50	01:00 - 01:59	Early Morning	37,4	111	24,2	24,2	18,55	601,33	106,83
	2019/05/25	CH2206	Male	Three y/o	Treatment (Wk 2)	Treatment	Fast Day	No	01:50	01:00 - 01:59	Early Morning	37,3	48	57,2	57,2	18,46	536,04	136,44
	2019/05/25	CH2205	Male	Three y/o	Treatment (Wk 2)	Treatment	Fast Day	No	01:55	01:00 - 01:59	Early Morning	37,4		24,4	24,4	18,55		107,11

2019/05/25	CH2206	Male	Three y/o	Treatment (Wk 2)	Treatment	Fast Day	No	01:55	01:00 - 01:59	Early Morning	37,3	68	54,2	54,2	18,46	564,71	134,57
2019/05/25	CH2205	Male	Three y/o	Treatment (Wk 2)	Treatment	Fast Day	No	02:00	02:00 - 02:59	Early Morning	37,4	88	24,6	24,6	18,55	584,49	107,39
2019/05/25	CH2206	Male	Three y/o	Treatment (Wk 2)	Treatment	Fast Day	No	02:00	02:00 - 02:59	Early Morning	37,3	123	39,4	39,4	18,46	608,49	123,57
2019/05/25	CH2205	Male	Three y/o	Treatment (Wk 2)	Treatment	Fast Day	No	02:05	02:00 - 02:59	Early Morning	37,4		17,2	17,2	18,55		95,17
2019/05/25	CH2206	Male	Three y/o	Treatment (Wk 2)	Treatment	Fast Day	No	02:05	02:00 - 02:59	Early Morning	37,4	78	40,0	40,0	18,55	575,38	124,09
2019/05/25	CH2205	Male	Three y/o	Treatment (Wk 2)	Treatment	Fast Day	No	02:10	02:00 - 02:59	Early Morning	37,4	89	19,0	19,0	18,55	585,33	98,56
2019/05/25	CH2206	Male	Three y/o	Treatment (Wk 2)	Treatment	Fast Day	No	02:10	02:00 - 02:59	Early Morning	37,4	66	41,2	41,2	18,55	562,34	125,11
2019/05/25	CH2205	Male	Three y/o	Treatment (Wk 2)	Treatment	Fast Day	No	02:15	02:00 - 02:59	Early Morning	37,3		21,4	21,4	18,46		102,63
2019/05/25	CH2206	Male	Three y/o	Treatment (Wk 2)	Treatment	Fast Day	No	02:15	02:00 - 02:59	Early Morning	37,4	44	42,4	42,4	18,55	528,51	126,10
2019/05/25	CH2205	Male	Three y/o	Treatment (Wk 2)	Treatment	Fast Day	No	02:20	02:00 - 02:59	Early Morning	37,3		21,6	21,6	18,46		102,94
2019/05/25	CH2206	Male	Three y/o	Treatment (Wk 2)	Treatment	Fast Day	No	02:20	02:00 - 02:59	Early Morning	37,4	53	60,0	60,0	18,55	544,43	138,09
2019/05/25	CH2205	Male	Three y/o	Treatment (Wk 2)	Treatment	Fast Day	No	02:25	02:00 - 02:59	Early Morning	37,3	124	41,0	41,0	18,46	609,04	124,94
2019/05/25	CH2206	Male	Three y/o	Treatment (Wk 2)	Treatment	Fast Day	No	02:25	02:00 - 02:59	Early Morning	37,4	47	58,6	58,6	18,55	534,23	137,28
2019/05/25	CH2205	Male	Three y/o	Treatment (Wk 2)	Treatment	Fast Day	No	02:30	02:00 - 02:59	Early Morning	37,1		15,0	15,0	18,26		90,51
2019/05/25	CH2206	Male	Three y/o	Treatment (Wk 2)	Treatment	Fast Day	No	02:30	02:00 - 02:59	Early Morning	37,4		32,8	32,8	18,55		117,26
2019/05/25	CH2205	Male	Three y/o	Treatment (Wk 2)	Treatment	Fast Day	No	02:35	02:00 - 02:59	Early Morning	37,0	75	26,2	26,2	18,16	572,36	109,55
2019/05/25	CH2206	Male	Three y/o	Treatment (Wk 2)	Treatment	Fast Day	No	02:35	02:00 - 02:59	Early Morning	37,4	103	55,4	55,4	18,55	596,00	135,33
2019/05/25	CH2205	Male	Three y/o	Treatment (Wk 2)	Treatment	Fast Day	No	02:40	02:00 - 02:59	Early Morning	37,1	49	27,0	27,0	18,26	537,80	110,58
2019/05/25	CH2206	Male	Three y/o	Treatment (Wk 2)	Treatment	Fast Day	No	02:40	02:00 - 02:59	Early Morning	37,1		50,8	50,8	18,26		132,33
2019/05/25	CH2205	Male	Three y/o	Treatment (Wk 2)	Treatment	Fast Day	No	02:45	02:00 - 02:59	Early Morning	37,3	60	34,0	34,0	18,46	554,67	118,49
2019/05/25	CH2206	Male	Three y/o	Treatment (Wk 2)	Treatment	Fast Day	No	02:45	02:00 - 02:59	Early Morning	37,0	49	54,8	54,8	18,16	537,80	134,96
2019/05/25	CH2205	Male	Three y/o	Treatment (Wk 2)	Treatment	Fast Day	No	02:50	02:00 - 02:59	Early Morning	37,3	78	32,2	32,2	18,46	575,38	116,63
2019/05/25	CH2206	Male	Three y/o	Treatment (Wk 2)	Treatment	Fast Day	No	02:50	02:00 - 02:59	Early Morning	37,3	49	54,8	54,8	18,46	537,80	134,96
2019/05/25	CH2205	Male	Three y/o	Treatment (Wk 2)	Treatment	Fast Day	No	02:55	02:00 - 02:59	Early Morning	37,3	57	30,8	30,8	18,46	550,47	115,10
2019/05/25	CH2206	Male	Three y/o	Treatment (Wk 2)	Treatment	Fast Day	No	02:55	02:00 - 02:59	Early Morning	37,2	42	55,6	55,6	18,36	524,42	135,46
2019/05/25	CH2205	Male	Three y/o	Treatment (Wk 2)	Treatment	Fast Day	No	03:00	03:00 - 03:59	Early Morning	37,3	80	30,6	30,6	18,46	577,31	114,87
2019/05/25	CH2206	Male	Three y/o	Treatment (Wk 2)	Treatment	Fast Day	No	03:00	03:00 - 03:59	Early Morning	37,2	46	59,6	59,6	18,36	532,38	137,86
2019/05/25	CH2205	Male	Three y/o	Treatment (Wk 2)	Treatment	Fast Day	No	03:05	03:00 - 03:59	Early Morning	37,3	98	24,4	24,4	18,46	592,41	107,11
2019/05/25	CH2206	Male	Three y/o	Treatment (Wk 2)	Treatment	Fast Day	No	03:05	03:00 - 03:59	Early Morning	37,2	90	69,8	69,8	18,36	586,16	143,34
2019/05/25	CH2205	Male	Three y/o	Treatment (Wk 2)	Treatment	Fast Day	No	03:10	03:00 - 03:59	Early Morning	37,3	59	23,0	23,0	18,46	553,30	105,09
2019/05/25	CH2206	Male	Three y/o	Treatment (Wk 2)	Treatment	Fast Day	No	03:10	03:00 - 03:59	Early Morning	37,2	86	61,8	61,8	18,36	582,77	139,12
2019/05/25	CH2205	Male	Three y/o	Treatment (Wk 2)	Treatment	Fast Day	No	03:15	03:00 - 03:59	Early Morning	37,4	62	21,6	21,6	18,55	557,33	102,94
2019/05/25	CH2206	Male	Three y/o	Treatment (Wk 2)	Treatment	Fast Day	No	03:15	03:00 - 03:59	Early Morning	37,1	143	45,8	45,8	18,26	618,69	128,76
2019/05/25	CH2205	Male	Three y/o	Treatment (Wk 2)	Treatment	Fast Day	No	03:20	03:00 - 03:59	Early Morning	37,4	66	21,2	21,2	18,55	562,34	102,31
2019/05/25	CH2206	Male	Three y/o	Treatment (Wk 2)	Treatment	Fast Day	No	03:20	03:00 - 03:59	Early Morning	37,3	51	55,6	55,6	18,46	541,20	135,46
2019/05/25	CH2205	Male	Three y/o	Treatment (Wk 2)	Treatment	Fast Day	No	03:25	03:00 - 03:59	Early Morning	37,5	55	22,6	22,6	18,65	547,52	104,49
2019/05/25	CH2206	Male	Three y/o	Treatment (Wk 2)	Treatment	Fast Day	No	03:25	03:00 - 03:59	Early Morning	37,3	43	55,0	55,0	18,46	526,50	135,08
2019/05/25	CH2205	Male	Three y/o	Treatment (Wk 2)	Treatment	Fast Day	No	03:30	03:00 - 03:59	Early Morning	37,5	65	18,4	18,4	18,65	561,12	97,47
2019/05/25	CH2206	Male	Three y/o	Treatment (Wk 2)	Treatment	Fast Day	No	03:30	03:00 - 03:59	Early Morning	37,2	125	62,4	62,4	18,36	609,60	139,45
2019/05/25	CH2205	Male	Three y/o	Treatment (Wk 2)	Treatment	Fast Day	No	03:35	03:00 - 03:59	Early Morning	37,6	110	126,0	126,0	18,75	600,69	163,91
2019/05/25	CH2206	Male	Three y/o	Treatment (Wk 2)	Treatment	Fast Day	No	03:35	03:00 - 03:59	Early Morning	37,3	53	130,6	130,6	18,46	544,43	165,17
2019/05/25	CH2205	Male	Three y/o	Treatment (Wk 2)	Treatment	Fast Day	No	03:40	03:00 - 03:59	Early Morning	37,4		35,8	35,8	18,55		120,27
2019/05/25	CH2206	Male	Three y/o	Treatment (Wk 2)	Treatment	Fast Day	No	03:40	03:00 - 03:59	Early Morning	37,2	169	83,8	83,8	18,36	629,60	149,69
2019/05/25	CH2205	Male	Three y/o	Treatment (Wk 2)	Treatment	Fast Day	No	03:45	03:00 - 03:59	Early Morning	37,4		30,6	30,6	18,55		114,87
2019/05/25	CH2206	Male	Three y/o	Treatment (Wk 2)	Treatment	Fast Day	No	03:45	03:00 - 03:59	Early Morning	37,3	132	141,6	141,6	18,46	613,32	168,00
2019/05/25	CH2205	Male	Three y/o	Treatment (Wk 2)	Treatment	Fast Day	No	03:50	03:00 - 03:59	Early Morning	37,4		83,4	83,4	18,55		149,52
2019/05/25	CH2206	Male	Three y/o	Treatment (Wk 2)	Treatment	Fast Day	No	03:50	03:00 - 03:59	Early Morning	37,4	129	434,6	434,6	18,55	611,75	207,56
2019/05/25	CH2205	Male	Three y/o	Treatment (Wk 2)	Treatment	Fast Day	No	03:55	03:00 - 03:59	Early Morning	37,3	59	219,4	219,4	18,46	553,30	183,38
2019/05/25	CH2206	Male	Three y/o	Treatment (Wk 2)	Treatment	Fast Day	No	03:55	03:00 - 03:59	Early Morning	37,4	141	102,0	102,0	18,55	617,75	156,53
2019/05/25	CH2205	Male	Three y/o	Treatment (Wk 2)	Treatment	Fast Day	No	04:00	04:00 - 04:59	Morning	37,4	117	229,0	229,0	18,55	605,02	184,89
2019/05/25	CH2206	Male	Three y/o	Treatment (Wk 2)	Treatment	Fast Day	No	04:00	04:00 - 04:59	Morning	37,5	121	91,0	91,0	18,65	607,35	152,56
2019/05/25	CH2205	Male	Three y/o	Treatment (Wk 2)	Treatment	Fast Day	No	04:05	04:00 - 04:59	Morning	37,5	78	79,4	79,4	18,65	575,38	147,81
2019/05/25	CH2206	Male	Three y/o	Treatment (Wk 2)	Treatment	Fast Day	No	04:05	04:00 - 04:59	Morning	37,5	109	119,2	119,2	18,65	600,04	161,97
2019/05/25	CH2205	Male	Three y/o	Treatment (Wk 2)	Treatment	Fast Day	No	04:10	04:00 - 04:59	Morning	37,5	65	18,0	18,0	18,65	561,12	96,72
2019/05/25	CH2206	Male	Three y/o	Treatment (Wk 2)	Treatment	Fast Day	No	04:10	04:00 - 04:59	Morning	37,7	99	49,6	49,6	18,85	593,14	131,51
2019/05/25	CH2205	Male	Three y/o	Treatment (Wk 2)	Treatment	Fast Day	No	04:15	04:00 - 04:59	Morning	37,3	80	12,8	12,8	18,46	577,31	85,12
2019/05/25	CH2206	Male	Three y/o	Treatment (Wk 2)	Treatment	Fast Day	No	04:15	04:00 - 04:59	Morning	37,4	87	36,2	36,2	18,55	583,64	120,65
2019/05/25	CH2205	Male	Three y/o	Treatment (Wk 2)	Treatment	Fast Day	No	04:20	04:00 - 04:59	Morning	37,1	59	11,0	11,0	18,26	553,30	79,98
2019/05/25	CH2206	Male	Three y/o	Treatment (Wk 2)	Treatment	Fast Day	No	04:20	04:00 - 04:59	Morning	37,3	48	36,4	36,4	18,46	536,04	120,84
2019/05/25	CH2205	Male	Three y/o	Treatment (Wk 2)	Treatment	Fast Day	No	04:25	04:00 - 04:59	Morning	36,8	75	9,2	9,2	17,96	572,36	73,94
2019/05/25	CH2206	Male	Three y/o	Treatment (Wk 2)	Treatment	Fast Day	No	04:25	04:00 - 04:59	Morning	37,1	69	32,6	32,6	18,26	565,86	117,05
2019/05/25	CH2205	Male	Three y/o	Treatment (Wk 2)	Treatment	Fast Day	No	04:30	04:00 - 04:59	Morning	36,8		6,6	6,6	17,96		62,73
2019/05/25	CH2206	Male	Three y/o	Treatment (Wk 2)	Treatment	Fast Day	No	04:30	04:00 - 04:59	Morning	37,0	48	28,6	28,6	18,16	536,04	112,56
2019/05/25	CH2205	Male	Three y/o	Treatment (Wk 2)	Treatment	Fast Day	No	04:35	04:00 - 04:59	Morning	36,8		9,2	9,2	17,96		73,94
2019/05/25	CH2206	Male	Three y/o	Treatment (Wk 2)	Treatment	Fast Day	No	04:35	04:00 - 04:59	Morning	37,0	55	64,2	64,2	18,16	547,52	140,44
2019/05/25	CH2205	Male	Three y/o	Treatment (Wk 2)	Treatment	Fast Day	No	04:40	04:00 - 04:59	Morning	37,1		6,2	6,2	18,26		60,63
2019/05/25	CH2206	Male	Three y/o	Treatment (Wk 2)	Treatment	Fast Day	No	04:40	04:00 - 04:59	Morning	37,2	51	65,8	65,8	18,36	541,20	141,29
2019/05/25	CH2205	Male	Three y/o	Treatment (Wk 2)	Treatment	Fast Day	No	04:45	04:00 - 04:59	Morning	37,2	87	6,4	6,4	18,36	583,64	61,70
2019/05/25	CH2206	Male	Three y/o	Treatment (Wk 2)	Treatment	Fast Day	No	04:45	04:00 - 04:59	Morning	37,3	54	65,6	65,6	18,46	545,99	141,19
2019/05/25	CH2205	Male	Three y/o	Treatment (Wk 2)	Treatment	Fast Day	No	04:50	04:00 - 04:59	Morning	37,4		2,4	2,4	18,55		28,91

2019/05/25	CH2206	Male	Three y/o	Treatment (Wk 2)	Treatment	Fast Day	No	04:50	04:00 - 04:59	Morning	37,3	74	60,2	60,2	18,46	571,33	138,21
2019/05/25	CH2205	Male	Three y/o	Treatment (Wk 2)	Treatment	Fast Day	No	04:55	04:00 - 04:59	Morning	37,5		7,6	7,6	18,65		67,49
2019/05/25	CH2206	Male	Three y/o	Treatment (Wk 2)	Treatment	Fast Day	No	04:55	04:00 - 04:59	Morning	37,3	49	57,2	57,2	18,46	537,80	136,44
2019/05/25	CH2205	Male	Three y/o	Treatment (Wk 2)	Treatment	Fast Day	No	05:00	05:00 - 05:59	Morning	37,5	64	2,0	2,0	18,65	559,88	22,86
2019/05/25	CH2206	Male	Three y/o	Treatment (Wk 2)	Treatment	Fast Day	No	05:00	05:00 - 05:59	Morning	37,2	59	55,0	55,0	18,36	553,30	135,08
2019/05/25	CH2205	Male	Three y/o	Treatment (Wk 2)	Treatment	Fast Day	No	05:05	05:00 - 05:59	Morning	37,5	93	5,2	5,2	18,65	588,58	54,72
2019/05/25	CH2206	Male	Three y/o	Treatment (Wk 2)	Treatment	Fast Day	No	05:05	05:00 - 05:59	Morning	37,2	75	66,4	66,4	18,36	572,36	141,61
2019/05/25	CH2205	Male	Three y/o	Treatment (Wk 2)	Treatment	Fast Day	No	05:10	05:00 - 05:59	Morning	37,6	62	4,6	4,6	18,75	557,33	50,61
2019/05/25	CH2206	Male	Three y/o	Treatment (Wk 2)	Treatment	Fast Day	No	05:10	05:00 - 05:59	Morning	37,2	43	64,6	64,6	18,36	526,50	140,65
2019/05/25	CH2205	Male	Three y/o	Treatment (Wk 2)	Treatment	Fast Day	No	05:15	05:00 - 05:59	Morning	37,6	41	6,4	6,4	18,75	522,29	61,70
2019/05/25	CH2206	Male	Three y/o	Treatment (Wk 2)	Treatment	Fast Day	No	05:15	05:00 - 05:59	Morning	37,2	41	62,4	62,4	18,36	522,29	139,45
2019/05/25	CH2205	Male	Three y/o	Treatment (Wk 2)	Treatment	Fast Day	No	05:20	05:00 - 05:59	Morning	37,6	91	8,0	8,0	18,75	586,98	69,22
2019/05/25	CH2206	Male	Three y/o	Treatment (Wk 2)	Treatment	Fast Day	No	05:20	05:00 - 05:59	Morning	37,3	59	66,2	66,2	18,46	553,30	141,50
2019/05/25	CH2205	Male	Three y/o	Treatment (Wk 2)	Treatment	Fast Day	No	05:25	05:00 - 05:59	Morning	37,6	82	5,0	5,0	18,75	579,19	53,41
2019/05/25	CH2206	Male	Three y/o	Treatment (Wk 2)	Treatment	Fast Day	No	05:25	05:00 - 05:59	Morning	37,3	56	60,2	60,2	18,46	549,01	138,21
2019/05/25	CH2205	Male	Three y/o	Treatment (Wk 2)	Treatment	Fast Day	No	05:30	05:00 - 05:59	Morning	37,6	70	4,4	4,4	18,75	566,99	49,12
2019/05/25	CH2206	Male	Three y/o	Treatment (Wk 2)	Treatment	Fast Day	No	05:30	05:00 - 05:59	Morning	37,3	42	61,0	61,0	18,46	524,42	138,67
2019/05/25	CH2205	Male	Three y/o	Treatment (Wk 2)	Treatment	Fast Day	No	05:35	05:00 - 05:59	Morning	37,6	88	7,6	7,6	18,75	584,49	67,49
2019/05/25	CH2206	Male	Three y/o	Treatment (Wk 2)	Treatment	Fast Day	No	05:35	05:00 - 05:59	Morning	37,3	42	62,8	62,8	18,46	524,42	139,67
2019/05/25	CH2205	Male	Three y/o	Treatment (Wk 2)	Treatment	Fast Day	No	05:40	05:00 - 05:59	Morning	37,6	66	4,2	4,2	18,75	562,34	47,57
2019/05/25	CH2206	Male	Three y/o	Treatment (Wk 2)	Treatment	Fast Day	No	05:40	05:00 - 05:59	Morning	37,3	95	68,0	68,0	18,46	590,14	142,43
2019/05/25	CH2205	Male	Three y/o	Treatment (Wk 2)	Treatment	Fast Day	No	05:45	05:00 - 05:59	Morning	37,6	59	2,2	2,2	18,75	553,30	26,02
2019/05/25	CH2206	Male	Three y/o	Treatment (Wk 2)	Treatment	Fast Day	No	05:45	05:00 - 05:59	Morning	37,3	90	52,4	52,4	18,46	586,16	133,41
2019/05/25	CH2205	Male	Three y/o	Treatment (Wk 2)	Treatment	Fast Day	No	05:50	05:00 - 05:59	Morning	37,6	56	4,8	4,8	18,75	549,01	52,04
2019/05/25	CH2206	Male	Three y/o	Treatment (Wk 2)	Treatment	Fast Day	No	05:50	05:00 - 05:59	Morning	37,3	47	58,4	58,4	18,46	534,23	137,16
2019/05/25	CH2205	Male	Three y/o	Treatment (Wk 2)	Treatment	Fast Day	No	05:55	05:00 - 05:59	Morning	37,7	59	3,4	3,4	18,85	553,30	40,51
2019/05/25	CH2206	Male	Three y/o	Treatment (Wk 2)	Treatment	Fast Day	No	05:55	05:00 - 05:59	Morning	37,2	52	58,6	58,6	18,36	542,83	137,28
2019/05/25	CH2205	Male	Three y/o	Treatment (Wk 2)	Treatment	Fast Day	No	06:00	06:00 - 06:59	Morning	37,7	90	40,2	40,2	18,85	586,16	124,26
2019/05/25	CH2206	Male	Three y/o	Treatment (Wk 2)	Treatment	Fast Day	No	06:00	06:00 - 06:59	Morning	37,2	38	57,6	57,6	18,36	515,48	136,68
2019/05/25	CH2205	Male	Three y/o	Treatment (Wk 2)	Treatment	Fast Day	No	06:05	06:00 - 06:59	Morning	37,7	56	5,8	5,8	18,85	549,01	58,39
2019/05/25	CH2206	Male	Three y/o	Treatment (Wk 2)	Treatment	Fast Day	No	06:05	06:00 - 06:59	Morning	37,1	45	62,0	62,0	18,26	530,47	139,23
2019/05/25	CH2205	Male	Three y/o	Treatment (Wk 2)	Treatment	Fast Day	No	06:10	06:00 - 06:59	Morning	37,7	65	1,6	1,6	18,85	561,12	15,48
2019/05/25	CH2206	Male	Three y/o	Treatment (Wk 2)	Treatment	Fast Day	No	06:10	06:00 - 06:59	Morning	37,0	41	62,8	62,8	18,16	522,29	139,67
2019/05/25	CH2205	Male	Three y/o	Treatment (Wk 2)	Treatment	Fast Day	No	06:15	06:00 - 06:59	Morning	37,7	93	45,8	45,8	18,85	588,58	128,76
2019/05/25	CH2206	Male	Three y/o	Treatment (Wk 2)	Treatment	Fast Day	No	06:15	06:00 - 06:59	Morning	36,9	63	129,2	129,2	18,06	558,62	164,79
2019/05/25	CH2205	Male	Three y/o	Treatment (Wk 2)	Treatment	Fast Day	No	06:20	06:00 - 06:59	Morning	37,7	123	117,6	117,6	18,85	608,49	161,50
2019/05/25	CH2206	Male	Three y/o	Treatment (Wk 2)	Treatment	Fast Day	No	06:20	06:00 - 06:59	Morning	37,0	123	169,6	169,6	18,16	608,49	174,32
2019/05/25	CH2205	Male	Three y/o	Treatment (Wk 2)	Treatment	Fast Day	No	06:25	06:00 - 06:59	Morning	37,6	60	135,2	135,2	18,75	554,67	166,38
2019/05/25	CH2206	Male	Three y/o	Treatment (Wk 2)	Treatment	Fast Day	No	06:25	06:00 - 06:59	Morning	37,1	125	115,8	115,8	18,26	609,60	160,96
2019/05/25	CH2205	Male	Three y/o	Treatment (Wk 2)	Treatment	Fast Day	No	06:30	06:00 - 06:59	Morning	37,4		26,4	26,4	18,55		109,81
2019/05/25	CH2206	Male	Three y/o	Treatment (Wk 2)	Treatment	Fast Day	No	06:30	06:00 - 06:59	Morning	37,2	141	138,2	138,2	18,36	617,75	167,15
2019/05/25	CH2205	Male	Three y/o	Treatment (Wk 2)	Treatment	Fast Day	No	06:35	06:00 - 06:59	Morning	37,3	73	29,2	29,2	18,46	570,27	113,27
2019/05/25	CH2206	Male	Three y/o	Treatment (Wk 2)	Treatment	Fast Day	No	06:35	06:00 - 06:59	Morning	37,3	182	169,2	169,2	18,46	634,31	174,24
2019/05/25	CH2205	Male	Three y/o	Treatment (Wk 2)	Treatment	Fast Day	No	06:40	06:00 - 06:59	Morning	37,2		32,6	32,6	18,36		117,05
2019/05/25	CH2206	Male	Three y/o	Treatment (Wk 2)	Treatment	Fast Day	No	06:40	06:00 - 06:59	Morning	37,4	188	237,2	237,2	18,55	636,34	186,13
2019/05/25	CH2205	Male	Three y/o	Treatment (Wk 2)	Treatment	Fast Day	No	06:45	06:00 - 06:59	Morning	37,3	121	82,0	82,0	18,46	607,35	148,93
2019/05/25	CH2206	Male	Three y/o	Treatment (Wk 2)	Treatment	Fast Day	No	06:45	06:00 - 06:59	Morning	37,6	125	115,0	115,0	18,75	609,60	160,72
2019/05/25	CH2205	Male	Three y/o	Treatment (Wk 2)	Treatment	Fast Day	No	06:50	06:00 - 06:59	Morning	37,4	81	70,4	70,4	18,55	578,26	143,64
2019/05/25	CH2206	Male	Three y/o	Treatment (Wk 2)	Treatment	Fast Day	No	06:50	06:00 - 06:59	Morning	37,8	140	265,6	265,6	18,95	617,28	190,12
2019/05/25	CH2205	Male	Three y/o	Treatment (Wk 2)	Treatment	Fast Day	No	06:55	06:00 - 06:59	Morning	37,3	90	86,6	86,6	18,46	586,16	150,83
2019/05/25	CH2206	Male	Three y/o	Treatment (Wk 2)	Treatment	Fast Day	No	06:55	06:00 - 06:59	Morning	37,8	135	144,6	144,6	18,95	614,84	168,73
2019/05/25	CH2205	Male	Three y/o	Treatment (Wk 2)	Treatment	Fast Day	No	07:00	07:00 - 07:59	Morning	37,4	141	29,6	29,6	18,55	617,75	113,73
2019/05/25	CH2206	Male	Three y/o	Treatment (Wk 2)	Treatment	Fast Day	No	07:00	07:00 - 07:59	Morning	37,8	119	334,4	334,4	18,95	606,20	198,26
2019/05/25	CH2205	Male	Three y/o	Treatment (Wk 2)	Treatment	Fast Day	No	07:05	07:00 - 07:59	Morning	37,4	108	86,6	86,6	18,55	599,39	150,83
2019/05/25	CH2206	Male	Three y/o	Treatment (Wk 2)	Treatment	Fast Day	No	07:05	07:00 - 07:59	Morning	37,8	71	108,2	108,2	18,95	568,10	158,59
2019/05/25	CH2205	Male	Three y/o	Treatment (Wk 2)	Treatment	Fast Day	No	07:10	07:00 - 07:59	Morning	37,4		97,2	97,2	18,55		154,85
2019/05/25	CH2206	Male	Three y/o	Treatment (Wk 2)	Treatment	Fast Day	No	07:10	07:00 - 07:59	Morning	37,8	145	163,4	163,4	18,95	619,62	173,02
2019/05/25	CH2205	Male	Three y/o	Treatment (Wk 2)	Treatment	Fast Day	No	07:15	07:00 - 07:59	Morning	37,5	83	56,4	56,4	18,65	580,11	135,95
2019/05/25	CH2206	Male	Three y/o	Treatment (Wk 2)	Treatment	Fast Day	No	07:15	07:00 - 07:59	Morning	37,8	135	80,6	80,6	18,95	614,84	148,34
2019/05/25	CH2205	Male	Three y/o	Treatment (Wk 2)	Treatment	Fast Day	No	07:20	07:00 - 07:59	Morning	37,5	106	26,8	26,8	18,65	598,06	110,33
2019/05/25	CH2206	Male	Three y/o	Treatment (Wk 2)	Treatment	Fast Day	No	07:20	07:00 - 07:59	Morning	37,8	121	148,2	148,2	18,95	607,35	169,59
2019/05/25	CH2205	Male	Three y/o	Treatment (Wk 2)	Treatment	Fast Day	No	07:25	07:00 - 07:59	Morning	37,4	108	33,2	33,2	18,55	599,39	117,68
2019/05/25	CH2206	Male	Three y/o	Treatment (Wk 2)	Treatment	Fast Day	No	07:25	07:00 - 07:59	Morning	37,8	120	195,8	195,8	18,95	606,78	179,37
2019/05/25	CH2205	Male	Three y/o	Treatment (Wk 2)	Treatment	Fast Day	No	07:30	07:00 - 07:59	Morning	37,3	108	37,4	37,4	18,46	599,39	121,77
2019/05/25	CH2206	Male	Three y/o	Treatment (Wk 2)	Treatment	Fast Day	No	07:30	07:00 - 07:59	Morning	37,8	63	196,4	196,4	18,95	558,62	179,48
2019/05/25	CH2205	Male	Three y/o	Treatment (Wk 2)	Treatment	Fast Day	No	07:35	07:00 - 07:59	Morning	37,4	125	77,2	77,2	18,55	609,60	146,84
2019/05/25	CH2206	Male	Three y/o	Treatment (Wk 2)	Treatment	Fast Day	No	07:35	07:00 - 07:59	Morning	37,9	115	134,0	134,0	19,05	603,82	166,07
2019/05/25	CH2205	Male	Three y/o	Treatment (Wk 2)	Treatment	Fast Day	No	07:40	07:00 - 07:59	Morning	37,4	101	226,0	226,0	18,55	594,59	184,42
2019/05/25	CH2206	Male	Three y/o	Treatment (Wk 2)	Treatment	Fast Day	No	07:40	07:00 - 07:59	Morning	37,9	60	38,6	38,6	19,05	554,67	122,86
2019/05/25	CH2205	Male	Three y/o	Treatment (Wk 2)	Treatment	Fast Day	No	07:45	07:00 - 07:59	Morning	37,5	114	64,0	64,0	18,65	603,20	140,33

2019/05/25	CH2206	Male	Three y/o	Treatment (Wk 2)	Treatment	Fast Day	No	07:45	07:00 - 07:59	Morning	37,9	126	172,6	172,6	19,05	610,14	174,94
2019/05/25	CH2205	Male	Three y/o	Treatment (Wk 2)	Treatment	Fast Day	No	07:50	07:00 - 07:59	Morning	37,4	108	105,6	105,6	18,55	599,39	157,74
2019/05/25	CH2206	Male	Three y/o	Treatment (Wk 2)	Treatment	Fast Day	No	07:50	07:00 - 07:59	Morning	37,9	125	180,2	180,2	19,05	609,60	176,45
2019/05/25	CH2205	Male	Three y/o	Treatment (Wk 2)	Treatment	Fast Day	No	07:55	07:00 - 07:59	Morning	37,5	68	122,6	122,6	18,65	564,71	162,96
2019/05/25	CH2206	Male	Three y/o	Treatment (Wk 2)	Treatment	Fast Day	No	07:55	07:00 - 07:59	Morning	37,9	109	73,8	73,8	19,05	600,04	145,27
2019/05/25	CH2205	Male	Three y/o	Treatment (Wk 2)	Treatment	Fast Day	No	08:00	08:00 - 08:59	Late Morning	37,5		79,6	79,6	18,65		147,90
2019/05/25	CH2206	Male	Three y/o	Treatment (Wk 2)	Treatment	Fast Day	No	08:00	08:00 - 08:59	Late Morning	37,9	115	121,2	121,2	19,05	603,82	162,56
2019/05/25	CH2205	Male	Three y/o	Treatment (Wk 2)	Treatment	Fast Day	No	08:05	08:00 - 08:59	Late Morning	37,5	74	18,6	18,6	18,65	571,33	97,84
2019/05/25	CH2206	Male	Three y/o	Treatment (Wk 2)	Treatment	Fast Day	No	08:05	08:00 - 08:59	Late Morning	37,9	128	151,0	151,0	19,05	611,22	170,25
2019/05/25	CH2205	Male	Three y/o	Treatment (Wk 2)	Treatment	Fast Day	No	08:10	08:00 - 08:59	Late Morning	37,4	71	17,6	17,6	18,55	568,10	95,95
2019/05/25	CH2206	Male	Three y/o	Treatment (Wk 2)	Treatment	Fast Day	No	08:10	08:00 - 08:59	Late Morning	37,9	126	101,8	101,8	19,05	610,14	156,47
2019/05/25	CH2205	Male	Three y/o	Treatment (Wk 2)	Treatment	Fast Day	No	08:15	08:00 - 08:59	Late Morning	37,2	62	8,4	8,4	18,36	557,33	70,86
2019/05/25	CH2206	Male	Three y/o	Treatment (Wk 2)	Treatment	Fast Day	No	08:15	08:00 - 08:59	Late Morning	37,9	110	133,6	133,6	19,05	600,69	165,96
2019/05/25	CH2205	Male	Three y/o	Treatment (Wk 2)	Treatment	Fast Day	No	08:20	08:00 - 08:59	Late Morning	37,2	63	8,0	8,0	18,36	558,62	69,22
2019/05/25	CH2206	Male	Three y/o	Treatment (Wk 2)	Treatment	Fast Day	No	08:20	08:00 - 08:59	Late Morning	37,9	129	235,4	235,4	19,05	611,75	185,86
2019/05/25	CH2205	Male	Three y/o	Treatment (Wk 2)	Treatment	Fast Day	No	08:25	08:00 - 08:59	Late Morning	37,3	69	6,6	6,6	18,46	565,86	62,73
2019/05/25	CH2206	Male	Three y/o	Treatment (Wk 2)	Treatment	Fast Day	No	08:25	08:00 - 08:59	Late Morning	37,9	121	240,2	240,2	19,05	607,35	186,57
2019/05/25	CH2205	Male	Three y/o	Treatment (Wk 2)	Treatment	Fast Day	No	08:30	08:00 - 08:59	Late Morning	37,3	61	60,8	60,8	18,46	556,01	138,55
2019/05/25	CH2206	Male	Three y/o	Treatment (Wk 2)	Treatment	Fast Day	No	08:30	08:00 - 08:59	Late Morning	37,9	129	180,8	180,8	19,05	611,75	176,57
2019/05/25	CH2205	Male	Three y/o	Treatment (Wk 2)	Treatment	Fast Day	No	08:35	08:00 - 08:59	Late Morning	37,3	98	30,8	30,8	18,46	592,41	115,10
2019/05/25	CH2206	Male	Three y/o	Treatment (Wk 2)	Treatment	Fast Day	No	08:35	08:00 - 08:59	Late Morning	37,9	118	166,6	166,6	19,05	605,61	173,70
2019/05/25	CH2205	Male	Three y/o	Treatment (Wk 2)	Treatment	Fast Day	No	08:40	08:00 - 08:59	Late Morning	37,3	143	49,8	49,8	18,46	618,69	131,65
2019/05/25	CH2206	Male	Three y/o	Treatment (Wk 2)	Treatment	Fast Day	No	08:40	08:00 - 08:59	Late Morning	37,9	160	55,2	55,2	19,05	626,07	135,21
2019/05/25	CH2205	Male	Three y/o	Treatment (Wk 2)	Treatment	Fast Day	No	08:45	08:00 - 08:59	Late Morning	37,4	113	31,4	31,4	18,55	602,58	115,76
2019/05/25	CH2206	Male	Three y/o	Treatment (Wk 2)	Treatment	Fast Day	No	08:45	08:00 - 08:59	Late Morning	38,0	97	131,8	131,8	19,15	591,66	165,49
2019/05/25	CH2205	Male	Three y/o	Treatment (Wk 2)	Treatment	Fast Day	No	08:50	08:00 - 08:59	Late Morning	37,2	51	9,8	9,8	18,36	541,20	76,07
2019/05/25	CH2206	Male	Three y/o	Treatment (Wk 2)	Treatment	Fast Day	No	08:50	08:00 - 08:59	Late Morning	37,9	107	283,0	283,0	19,05	598,73	192,36
2019/05/25	CH2205	Male	Three y/o	Treatment (Wk 2)	Treatment	Fast Day	No	08:55	08:00 - 08:59	Late Morning	37,4	79	115,8	115,8	18,55	576,36	160,96
2019/05/25	CH2206	Male	Three y/o	Treatment (Wk 2)	Treatment	Fast Day	No	08:55	08:00 - 08:59	Late Morning	37,9	108	117,6	117,6	19,05	599,39	161,50
2019/05/25	CH2205	Male	Three y/o	Treatment (Wk 2)	Treatment	Fast Day	No	09:00	09:00 - 09:59	Late Morning	37,4	101	56,6	56,6	18,55	594,59	136,07
2019/05/25	CH2206	Male	Three y/o	Treatment (Wk 2)	Treatment	Fast Day	No	09:00	09:00 - 09:59	Late Morning	37,9	103	40,0	40,0	19,05	596,00	124,09
2019/05/25	CH2205	Male	Three y/o	Treatment (Wk 2)	Treatment	Fast Day	No	09:05	09:00 - 09:59	Late Morning	37,4	114	34,8	34,8	18,55	603,20	119,29
2019/05/25	CH2206	Male	Three y/o	Treatment (Wk 2)	Treatment	Fast Day	No	09:05	09:00 - 09:59	Late Morning	37,9	53	226,0	226,0	19,05	544,43	184,42
2019/05/25	CH2205	Male	Three y/o	Treatment (Wk 2)	Treatment	Fast Day	No	09:10	09:00 - 09:59	Late Morning	37,4	68	14,4	14,4	18,55	564,71	89,12
2019/05/25	CH2206	Male	Three y/o	Treatment (Wk 2)	Treatment	Fast Day	No	09:10	09:00 - 09:59	Late Morning	37,9	108	5,2	5,2	19,05	599,39	54,72
2019/05/25	CH2205	Male	Three y/o	Treatment (Wk 2)	Treatment	Fast Day	No	09:15	09:00 - 09:59	Late Morning	37,5	73	15,2	15,2	18,65	570,27	90,96
2019/05/25	CH2206	Male	Three y/o	Treatment (Wk 2)	Treatment	Fast Day	No	09:15	09:00 - 09:59	Late Morning	37,9	59	6,2	6,2	19,05	553,30	60,63
2019/05/25	CH2205	Male	Three y/o	Treatment (Wk 2)	Treatment	Fast Day	No	09:20	09:00 - 09:59	Late Morning	37,5	109	14,4	14,4	18,65	600,04	89,12
2019/05/25	CH2206	Male	Three y/o	Treatment (Wk 2)	Treatment	Fast Day	No	09:20	09:00 - 09:59	Late Morning	37,9	49	6,0	6,0	19,05	537,80	59,53
2019/05/25	CH2205	Male	Three y/o	Treatment (Wk 2)	Treatment	Fast Day	No	09:25	09:00 - 09:59	Late Morning	37,6	55	16,6	16,6	18,75	547,52	93,96
2019/05/25	CH2206	Male	Three y/o	Treatment (Wk 2)	Treatment	Fast Day	No	09:25	09:00 - 09:59	Late Morning	37,8	50	234,2	234,2	18,95	539,52	185,68
2019/05/25	CH2205	Male	Three y/o	Treatment (Wk 2)	Treatment	Fast Day	No	09:30	09:00 - 09:59	Late Morning	37,6	59	30,2	30,2	18,75	553,30	114,42
2019/05/25	CH2206	Male	Three y/o	Treatment (Wk 2)	Treatment	Fast Day	No	09:30	09:00 - 09:59	Late Morning	37,8		79,4	79,4	18,95		147,81
2019/05/25	CH2205	Male	Three y/o	Treatment (Wk 2)	Treatment	Fast Day	No	09:35	09:00 - 09:59	Late Morning	37,5	69	27,4	27,4	18,65	565,86	111,09
2019/05/25	CH2206	Male	Three y/o	Treatment (Wk 2)	Treatment	Fast Day	No	09:35	09:00 - 09:59	Late Morning	37,8		833,4		18,95		
2019/05/25	CH2205	Male	Three y/o	Treatment (Wk 2)	Treatment	Fast Day	No	09:40	09:00 - 09:59	Late Morning	37,5	45	19,4	19,4	18,65	530,47	99,28
2019/05/25	CH2206	Male	Three y/o	Treatment (Wk 2)	Treatment	Fast Day	No	09:40	09:00 - 09:59	Late Morning	37,9	107	91,0	91,0	19,05	598,73	152,56
2019/05/25	CH2205	Male	Three y/o	Treatment (Wk 2)	Treatment	Fast Day	No	09:45	09:00 - 09:59	Late Morning	37,5	188	17,2	17,2	18,65	636,34	95,17
2019/05/25	CH2206	Male	Three y/o	Treatment (Wk 2)	Treatment	Fast Day	No	09:45	09:00 - 09:59	Late Morning	37,9	121	92,8	92,8	19,05	607,35	153,24
2019/05/25	CH2205	Male	Three y/o	Treatment (Wk 2)	Treatment	Fast Day	No	09:50	09:00 - 09:59	Late Morning	37,6	50	4,0	4,0	18,75	539,52	45,94
2019/05/25	CH2206	Male	Three y/o	Treatment (Wk 2)	Treatment	Fast Day	No	09:50	09:00 - 09:59	Late Morning	37,9	110	71,6	71,6	19,05	600,69	144,22
2019/05/25	CH2205	Male	Three y/o	Treatment (Wk 2)	Treatment	Fast Day	No	09:55	09:00 - 09:59	Late Morning	37,6	55	9,8	9,8	18,75	547,52	76,07
2019/05/25	CH2206	Male	Three y/o	Treatment (Wk 2)	Treatment	Fast Day	No	09:55	09:00 - 09:59	Late Morning	37,9		127,0	127,0	19,05		164,19
2019/05/25	CH2205	Male	Three y/o	Treatment (Wk 2)	Treatment	Fast Day	No	10:00	10:00 - 10:59	Late Morning	37,7	98	14,6	14,6	18,85	592,41	89,59
2019/05/25	CH2206	Male	Three y/o	Treatment (Wk 2)	Treatment	Fast Day	No	10:00	10:00 - 10:59	Late Morning	37,9	86	6,2	6,2	19,05	582,77	60,63
2019/05/25	CH2205	Male	Three y/o	Treatment (Wk 2)	Treatment	Fast Day	No	10:05	10:00 - 10:59	Late Morning	37,7	56	13,8	13,8	18,85	549,01	87,68
2019/05/25	CH2206	Male	Three y/o	Treatment (Wk 2)	Treatment	Fast Day	No	10:05	10:00 - 10:59	Late Morning	37,9	67	5,8	5,8	19,05	563,53	58,39
2019/05/25	CH2205	Male	Three y/o	Treatment (Wk 2)	Treatment	Fast Day	No	10:10	10:00 - 10:59	Late Morning	37,7		22,6	22,6	18,85		104,49
2019/05/25	CH2206	Male	Three y/o	Treatment (Wk 2)	Treatment	Fast Day	No	10:10	10:00 - 10:59	Late Morning	37,9	66	5,6	5,6	19,05	562,34	57,21
2019/05/25	CH2205	Male	Three y/o	Treatment (Wk 2)	Treatment	Fast Day	No	10:15	10:00 - 10:59	Late Morning	37,6	92	3,8	3,8	18,75	587,78	44,22
2019/05/25	CH2206	Male	Three y/o	Treatment (Wk 2)	Treatment	Fast Day	No	10:15	10:00 - 10:59	Late Morning	37,9	68	28,0	28,0	19,05	564,71	111,83
2019/05/25	CH2205	Male	Three y/o	Treatment (Wk 2)	Treatment	Fast Day	No	10:20	10:00 - 10:59	Late Morning	37,6	52	5,8	5,8	18,75	542,83	58,39
2019/05/25	CH2206	Male	Three y/o	Treatment (Wk 2)	Treatment	Fast Day	No	10:20	10:00 - 10:59	Late Morning	37,8	45	53,0	53,0	18,95	530,47	133,80
2019/05/25	CH2205	Male	Three y/o	Treatment (Wk 2)	Treatment	Fast Day	No	10:25	10:00 - 10:59	Late Morning	37,6	72	3,0	3,0	18,75	569,20	36,34
2019/05/25	CH2206	Male	Three y/o	Treatment (Wk 2)	Treatment	Fast Day	No	10:25	10:00 - 10:59	Late Morning	37,8	50	40,4	40,4	18,95	539,52	124,43
2019/05/25	CH2205	Male	Three y/o	Treatment (Wk 2)	Treatment	Fast Day	No	10:30	10:00 - 10:59	Late Morning	37,6	46	4,2	4,2	18,75	532,38	47,57
2019/05/25	CH2206	Male	Three y/o	Treatment (Wk 2)	Treatment	Fast Day	No	10:30	10:00 - 10:59	Late Morning	37,8	76	21,6	21,6	18,95	573,39	102,94
2019/05/25	CH2205	Male	Three y/o	Treatment (Wk 2)	Treatment	Fast Day	No	10:35	10:00 - 10:59	Late Morning	37,6	81	8,4	8,4	18,75	578,26	70,86
2019/05/25	CH2206	Male	Three y/o	Treatment (Wk 2)	Treatment	Fast Day	No	10:35	10:00 - 10:59	Late Morning	37,8	47	23,4	23,4	18,95	534,23	105,68
2019/05/25	CH2205	Male	Three y/o	Treatment (Wk 2)	Treatment	Fast Day	No	10:40	10:00 - 10:59	Late Morning	37,5		25,0	25,0	18,65		107,94

2019/05/25	CH2206	Male	Three y/o	Treatment (Wk 2)	Treatment	Fast Day	No	10:40	10:00 - 10:59	Late Morning	37,8	55	48,6	48,6	18,95	547,52	130,81
2019/05/25	CH2205	Male	Three y/o	Treatment (Wk 2)	Treatment	Fast Day	No	10:45	10:00 - 10:59	Late Morning	37,5	50	15,2	15,2	18,65	539,52	90,96
2019/05/25	CH2206	Male	Three y/o	Treatment (Wk 2)	Treatment	Fast Day	No	10:45	10:00 - 10:59	Late Morning	37,8	48	40,4	40,4	18,95	536,04	124,43
2019/05/25	CH2205	Male	Three y/o	Treatment (Wk 2)	Treatment	Fast Day	No	10:50	10:00 - 10:59	Late Morning	37,5		82,2	82,2	18,65		149,02
2019/05/25	CH2206	Male	Three y/o	Treatment (Wk 2)	Treatment	Fast Day	No	10:50	10:00 - 10:59	Late Morning	37,8	154	124,6	124,6	18,95	623,58	163,52
2019/05/25	CH2205	Male	Three y/o	Treatment (Wk 2)	Treatment	Fast Day	No	10:55	10:00 - 10:59	Late Morning	37,5		48,2	48,2	18,65		130,52
2019/05/25	CH2206	Male	Three y/o	Treatment (Wk 2)	Treatment	Fast Day	No	10:55	10:00 - 10:59	Late Morning	37,8		157,6	157,6	18,95		171,75
2019/05/25	CH2205	Male	Three y/o	Treatment (Wk 2)	Treatment	Fast Day	No	11:00	11:00 - 11:59	Late Morning	37,4	150	67,0	67,0	18,55	621,86	141,92
2019/05/25	CH2206	Male	Three y/o	Treatment (Wk 2)	Treatment	Fast Day	No	11:00	11:00 - 11:59	Late Morning	37,7	102	201,2	201,2	18,85	595,30	180,33
2019/05/25	CH2205	Male	Three y/o	Treatment (Wk 2)	Treatment	Fast Day	No	11:05	11:00 - 11:59	Late Morning	37,4	37	58,2	58,2	18,55	513,06	137,04
2019/05/25	CH2206	Male	Three y/o	Treatment (Wk 2)	Treatment	Fast Day	No	11:05	11:00 - 11:59	Late Morning	37,8	103	112,8	112,8	18,95	596,00	160,05
2019/05/25	CH2205	Male	Three y/o	Treatment (Wk 2)	Treatment	Fast Day	No	11:10	11:00 - 11:59	Late Morning	37,6	98	72,8	72,8	18,75	592,41	144,80
2019/05/25	CH2206	Male	Three y/o	Treatment (Wk 2)	Treatment	Fast Day	No	11:10	11:00 - 11:59	Late Morning	37,8	120	91,6	91,6	18,95	606,78	152,79
2019/05/25	CH2205	Male	Three y/o	Treatment (Wk 2)	Treatment	Fast Day	No	11:15	11:00 - 11:59	Late Morning	37,6		30,4	30,4	18,75		114,65
2019/05/25	CH2206	Male	Three y/o	Treatment (Wk 2)	Treatment	Fast Day	No	11:15	11:00 - 11:59	Late Morning	37,8	103	27,4	27,4	18,95	596,00	111,09
2019/05/25	CH2205	Male	Three y/o	Treatment (Wk 2)	Treatment	Fast Day	No	11:20	11:00 - 11:59	Late Morning	37,5	106	90,4	90,4	18,65	598,06	152,33
2019/05/25	CH2206	Male	Three y/o	Treatment (Wk 2)	Treatment	Fast Day	No	11:20	11:00 - 11:59	Late Morning	37,8	71	35,2	35,2	18,95	568,10	119,69
2019/05/25	CH2205	Male	Three y/o	Treatment (Wk 2)	Treatment	Fast Day	No	11:25	11:00 - 11:59	Late Morning	37,6		37,0	37,0	18,75		121,40
2019/05/25	CH2206	Male	Three y/o	Treatment (Wk 2)	Treatment	Fast Day	No	11:25	11:00 - 11:59	Late Morning	37,8	78	57,0	57,0	18,95	575,38	136,32
2019/05/25	CH2205	Male	Three y/o	Treatment (Wk 2)	Treatment	Fast Day	No	11:30	11:00 - 11:59	Late Morning	37,6		126,8	126,8	18,75		164,13
2019/05/25	CH2206	Male	Three y/o	Treatment (Wk 2)	Treatment	Fast Day	No	11:30	11:00 - 11:59	Late Morning	37,9	98	38,6	38,6	19,05	592,41	122,86
2019/05/25	CH2205	Male	Three y/o	Treatment (Wk 2)	Treatment	Fast Day	No	11:35	11:00 - 11:59	Late Morning	37,7	80	75,4	75,4	18,85	577,31	146,02
2019/05/25	CH2206	Male	Three y/o	Treatment (Wk 2)	Treatment	Fast Day	No	11:35	11:00 - 11:59	Late Morning	37,9	88	110,8	110,8	19,05	584,49	159,42
2019/05/25	CH2205	Male	Three y/o	Treatment (Wk 2)	Treatment	Fast Day	No	11:40	11:00 - 11:59	Late Morning	37,7	59	20,8	20,8	18,85	553,30	101,65
2019/05/25	CH2206	Male	Three y/o	Treatment (Wk 2)	Treatment	Fast Day	No	11:40	11:00 - 11:59	Late Morning	37,9	106	131,8	131,8	19,05	598,06	165,49
2019/05/25	CH2205	Male	Three y/o	Treatment (Wk 2)	Treatment	Fast Day	No	11:45	11:00 - 11:59	Late Morning	37,7	43	18,4	18,4	18,85	526,50	97,47
2019/05/25	CH2206	Male	Three y/o	Treatment (Wk 2)	Treatment	Fast Day	No	11:45	11:00 - 11:59	Late Morning	37,9	94	613,2	613,2	19,05	589,37	219,82
2019/05/25	CH2205	Male	Three y/o	Treatment (Wk 2)	Treatment	Fast Day	No	11:50	11:00 - 11:59	Late Morning	37,9	138	297,8	297,8	19,05	616,31	194,16
2019/05/25	CH2206	Male	Three y/o	Treatment (Wk 2)	Treatment	Fast Day	No	11:50	11:00 - 11:59	Late Morning	38,0		28,8	28,8	19,15		112,79
2019/05/25	CH2205	Male	Three y/o	Treatment (Wk 2)	Treatment	Fast Day	No	11:55	11:00 - 11:59	Late Morning	37,9	57	10,2	10,2	19,05	550,47	77,43
2019/05/25	CH2206	Male	Three y/o	Treatment (Wk 2)	Treatment	Fast Day	No	11:55	11:00 - 11:59	Late Morning	38,0	59	34,0	34,0	19,15	553,30	118,49
2019/05/25	CH2205	Male	Three y/o	Treatment (Wk 2)	Treatment	Fast Day	No	12:00	12:00 - 12:59	Afternoon	37,9	48	4,4	4,4	19,05	536,04	49,12
2019/05/25	CH2206	Male	Three y/o	Treatment (Wk 2)	Treatment	Fast Day	No	12:00	12:00 - 12:59	Afternoon	38,0	81	48,4	48,4	19,15	578,26	130,66
2019/05/25	CH2205	Male	Three y/o	Treatment (Wk 2)	Treatment	Fast Day	No	12:05	12:00 - 12:59	Afternoon	37,9	63	8,8	8,8	19,05	558,62	72,43
2019/05/25	CH2206	Male	Three y/o	Treatment (Wk 2)	Treatment	Fast Day	No	12:05	12:00 - 12:59	Afternoon	38,0	50	60,0	60,0	19,15	539,52	138,09
2019/05/25	CH2205	Male	Three y/o	Treatment (Wk 2)	Treatment	Fast Day	No	12:10	12:00 - 12:59	Afternoon	37,8	42	7,4	7,4	18,95	524,42	66,59
2019/05/25	CH2206	Male	Three y/o	Treatment (Wk 2)	Treatment	Fast Day	No	12:10	12:00 - 12:59	Afternoon	38,0	68	60,0	60,0	19,15	564,71	138,09
2019/05/25	CH2205	Male	Three y/o	Treatment (Wk 2)	Treatment	Fast Day	No	12:15	12:00 - 12:59	Afternoon	37,7	46	6,4	6,4	18,85	532,38	61,70
2019/05/25	CH2206	Male	Three y/o	Treatment (Wk 2)	Treatment	Fast Day	No	12:15	12:00 - 12:59	Afternoon	38,0	47	57,8	57,8	19,15	534,23	136,80
2019/05/25	CH2205	Male	Three y/o	Treatment (Wk 2)	Treatment	Fast Day	No	12:20	12:00 - 12:59	Afternoon	37,7	79	14,2	14,2	18,85	576,36	88,65
2019/05/25	CH2206	Male	Three y/o	Treatment (Wk 2)	Treatment	Fast Day	No	12:20	12:00 - 12:59	Afternoon	38,0	79	67,0	67,0	19,15	576,36	141,92
2019/05/25	CH2205	Male	Three y/o	Treatment (Wk 2)	Treatment	Fast Day	No	12:25	12:00 - 12:59	Afternoon	37,7	45	10,2	10,2	18,85	530,47	77,43
2019/05/25	CH2206	Male	Three y/o	Treatment (Wk 2)	Treatment	Fast Day	No	12:25	12:00 - 12:59	Afternoon	38,0	50	67,2	67,2	19,15	539,52	142,02
2019/05/25	CH2205	Male	Three y/o	Treatment (Wk 2)	Treatment	Fast Day	No	12:30	12:00 - 12:59	Afternoon	37,7	49	2,0	2,0	18,85	537,80	22,86
2019/05/25	CH2206	Male	Three y/o	Treatment (Wk 2)	Treatment	Fast Day	No	12:30	12:00 - 12:59	Afternoon	38,0	46	59,4	59,4	19,15	532,38	137,75
2019/05/25	CH2205	Male	Three y/o	Treatment (Wk 2)	Treatment	Fast Day	No	12:35	12:00 - 12:59	Afternoon	37,7	38	66,4	66,4	18,85	515,48	141,61
2019/05/25	CH2206	Male	Three y/o	Treatment (Wk 2)	Treatment	Fast Day	No	12:35	12:00 - 12:59	Afternoon	37,9	45	64,6	64,6	19,05	530,47	140,65
2019/05/25	CH2205	Male	Three y/o	Treatment (Wk 2)	Treatment	Fast Day	No	12:40	12:00 - 12:59	Afternoon	37,7	44	19,2	19,2	18,85	528,51	98,92
2019/05/25	CH2206	Male	Three y/o	Treatment (Wk 2)	Treatment	Fast Day	No	12:40	12:00 - 12:59	Afternoon	37,9	39	67,2	67,2	19,05	517,82	142,02
2019/05/25	CH2205	Male	Three y/o	Treatment (Wk 2)	Treatment	Fast Day	No	12:45	12:00 - 12:59	Afternoon	37,7	53	15,8	15,8	18,85	544,43	92,28
2019/05/25	CH2206	Male	Three y/o	Treatment (Wk 2)	Treatment	Fast Day	No	12:45	12:00 - 12:59	Afternoon	37,9	46	65,0	65,0	19,05	532,38	140,87
2019/05/25	CH2205	Male	Three y/o	Treatment (Wk 2)	Treatment	Fast Day	No	12:50	12:00 - 12:59	Afternoon	37,7	55	22,4	22,4	18,85	547,52	104,19
2019/05/25	CH2206	Male	Three y/o	Treatment (Wk 2)	Treatment	Fast Day	No	12:50	12:00 - 12:59	Afternoon	37,9	42	61,4	61,4	19,05	524,42	138,89
2019/05/25	CH2205	Male	Three y/o	Treatment (Wk 2)	Treatment	Fast Day	No	12:55	12:00 - 12:59	Afternoon	37,6	46	27,6	27,6	18,75	532,38	111,33
2019/05/25	CH2206	Male	Three y/o	Treatment (Wk 2)	Treatment	Fast Day	No	12:55	12:00 - 12:59	Afternoon	37,8	43	70,2	70,2	18,95	526,50	143,54
2019/05/25	CH2205	Male	Three y/o	Treatment (Wk 2)	Treatment	Fast Day	No	13:00	13:00 - 13:59	Afternoon	37,6	46	28,0	28,0	18,75	532,38	111,83
2019/05/25	CH2206	Male	Three y/o	Treatment (Wk 2)	Treatment	Fast Day	No	13:00	13:00 - 13:59	Afternoon	37,8	40	65,8	65,8	18,95	520,09	141,29
2019/05/25	CH2205	Male	Three y/o	Treatment (Wk 2)	Treatment	Fast Day	No	13:05	13:00 - 13:59	Afternoon	37,6	46	26,0	26,0	18,75	532,38	109,29
2019/05/25	CH2206	Male	Three y/o	Treatment (Wk 2)	Treatment	Fast Day	No	13:05	13:00 - 13:59	Afternoon	37,7	42	66,6	66,6	18,85	524,42	141,71
2019/05/25	CH2205	Male	Three y/o	Treatment (Wk 2)	Treatment	Fast Day	No	13:10	13:00 - 13:59	Afternoon	37,6	45	11,2	11,2	18,75	530,47	80,59
2019/05/25	CH2206	Male	Three y/o	Treatment (Wk 2)	Treatment	Fast Day	No	13:10	13:00 - 13:59	Afternoon	37,7	44	54,6	54,6	18,85	528,51	134,83
2019/05/25	CH2205	Male	Three y/o	Treatment (Wk 2)	Treatment	Fast Day	No	13:15	13:00 - 13:59	Afternoon	37,5	51	6,2	6,2	18,65	541,20	60,63
2019/05/25	CH2206	Male	Three y/o	Treatment (Wk 2)	Treatment	Fast Day	No	13:15	13:00 - 13:59	Afternoon	37,7	53	49,0	49,0	18,85	544,43	131,09
2019/05/25	CH2205	Male	Three y/o	Treatment (Wk 2)	Treatment	Fast Day	No	13:20	13:00 - 13:59	Afternoon	37,5	41	4,8	4,8	18,65	522,29	52,04
2019/05/25	CH2206	Male	Three y/o	Treatment (Wk 2)	Treatment	Fast Day	No	13:20	13:00 - 13:59	Afternoon	37,7	53	47,2	47,2	18,85	544,43	129,80
2019/05/25	CH2205	Male	Three y/o	Treatment (Wk 2)	Treatment	Fast Day	No	13:25	13:00 - 13:59	Afternoon	37,5	58	44,6	44,6	18,65	551,90	127,84
2019/05/25	CH2206	Male	Three y/o	Treatment (Wk 2)	Treatment	Fast Day	No	13:25	13:00 - 13:59	Afternoon	37,7		217,4	217,4	18,85		183,06
2019/05/25	CH2205	Male	Three y/o	Treatment (Wk 2)	Treatment	Fast Day	No	13:30	13:00 - 13:59	Afternoon	37,5		37,4	37,4	18,65		121,77
2019/05/25	CH2206	Male	Three y/o	Treatment (Wk 2)	Treatment	Fast Day	No	13:30	13:00 - 13:59	Afternoon	37,7	95	30,4	30,4	18,85	590,14	114,65
2019/05/25	CH2205	Male	Three y/o	Treatment (Wk 2)	Treatment	Fast Day	No	13:35	13:00 - 13:59	Afternoon	37,5		177,0	177,0	18,65		175,82

2019/05/25	CH2206	Male	Three y/o	Treatment (Wk 2)	Treatment	Fast Day	No	13:35	13:00 - 13:59	Afternoon	37,7	70	39,0	39,0	18,85	566,99	123,22
2019/05/25	CH2205	Male	Three y/o	Treatment (Wk 2)	Treatment	Fast Day	No	13:40	13:00 - 13:59	Afternoon	37,5	92	178,4	178,4	18,65	587,78	176,10
2019/05/25	CH2206	Male	Three y/o	Treatment (Wk 2)	Treatment	Fast Day	No	13:40	13:00 - 13:59	Afternoon	37,7	87	121,0	121,0	18,85	583,64	162,50
2019/05/25	CH2205	Male	Three y/o	Treatment (Wk 2)	Treatment	Fast Day	No	13:45	13:00 - 13:59	Afternoon	37,5	69	77,6	77,6	18,65	565,86	147,02
2019/05/25	CH2206	Male	Three y/o	Treatment (Wk 2)	Treatment	Fast Day	No	13:45	13:00 - 13:59	Afternoon	37,7	79	81,8	81,8	18,85	576,36	148,85
2019/05/25	CH2205	Male	Three y/o	Treatment (Wk 2)	Treatment	Fast Day	No	13:50	13:00 - 13:59	Afternoon	37,6		44,2	44,2	18,75		127,53
2019/05/25	CH2206	Male	Three y/o	Treatment (Wk 2)	Treatment	Fast Day	No	13:50	13:00 - 13:59	Afternoon	37,7	104	51,4	51,4	18,85	596,69	132,74
2019/05/25	CH2205	Male	Three y/o	Treatment (Wk 2)	Treatment	Fast Day	No	13:55	13:00 - 13:59	Afternoon	37,6	135	38,2	38,2	18,75	614,84	122,50
2019/05/25	CH2206	Male	Three y/o	Treatment (Wk 2)	Treatment	Fast Day	No	13:55	13:00 - 13:59	Afternoon	37,7	73	32,0	32,0	18,85	570,27	116,41
2019/05/25	CH2205	Male	Three y/o	Treatment (Wk 2)	Treatment	Fast Day	No	14:00	14:00 - 14:59	Afternoon	37,6		31,2	31,2	18,75		115,54
2019/05/25	CH2206	Male	Three y/o	Treatment (Wk 2)	Treatment	Fast Day	No	14:00	14:00 - 14:59	Afternoon	37,7	43	38,6	38,6	18,85	526,50	122,86
2019/05/25	CH2205	Male	Three y/o	Treatment (Wk 2)	Treatment	Fast Day	No	14:05	14:00 - 14:59	Afternoon	37,7		122,8	122,8	18,85		163,01
2019/05/25	CH2206	Male	Three y/o	Treatment (Wk 2)	Treatment	Fast Day	No	14:05	14:00 - 14:59	Afternoon	37,7		98,2	98,2	18,85		155,21
2019/05/25	CH2205	Male	Three y/o	Treatment (Wk 2)	Treatment	Fast Day	No	14:10	14:00 - 14:59	Afternoon	37,9	131	38,2	38,2	19,05	612,80	122,50
2019/05/25	CH2206	Male	Three y/o	Treatment (Wk 2)	Treatment	Fast Day	No	14:10	14:00 - 14:59	Afternoon	37,8	95	119,2	119,2	18,95	590,14	161,97
2019/05/25	CH2205	Male	Three y/o	Treatment (Wk 2)	Treatment	Fast Day	No	14:15	14:00 - 14:59	Afternoon	37,9	70	60,8	60,8	19,05	566,99	138,55
2019/05/25	CH2206	Male	Three y/o	Treatment (Wk 2)	Treatment	Fast Day	No	14:15	14:00 - 14:59	Afternoon	37,8	117	163,2	163,2	18,95	605,02	172,97
2019/05/25	CH2205	Male	Three y/o	Treatment (Wk 2)	Treatment	Fast Day	No	14:20	14:00 - 14:59	Afternoon	38,0	64	13,8	13,8	19,15	559,88	87,68
2019/05/25	CH2206	Male	Three y/o	Treatment (Wk 2)	Treatment	Fast Day	No	14:20	14:00 - 14:59	Afternoon	37,9	92	221,4	221,4	19,05	587,78	183,70
2019/05/25	CH2205	Male	Three y/o	Treatment (Wk 2)	Treatment	Fast Day	No	14:25	14:00 - 14:59	Afternoon	38,0	69	23,8	23,8	19,15	565,86	106,26
2019/05/25	CH2206	Male	Three y/o	Treatment (Wk 2)	Treatment	Fast Day	No	14:25	14:00 - 14:59	Afternoon	37,9	104	62,0	62,0	19,05	596,69	139,23
2019/05/25	CH2205	Male	Three y/o	Treatment (Wk 2)	Treatment	Fast Day	No	14:30	14:00 - 14:59	Afternoon	37,7	94	8,4	8,4	18,85	589,37	70,86
2019/05/25	CH2206	Male	Three y/o	Treatment (Wk 2)	Treatment	Fast Day	No	14:30	14:00 - 14:59	Afternoon	37,8	59	63,0	63,0	18,95	553,30	139,78
2019/05/25	CH2205	Male	Three y/o	Treatment (Wk 2)	Treatment	Fast Day	No	14:35	14:00 - 14:59	Afternoon	37,9	48	9,6	9,6	19,05	536,04	75,37
2019/05/25	CH2206	Male	Three y/o	Treatment (Wk 2)	Treatment	Fast Day	No	14:35	14:00 - 14:59	Afternoon	37,9	55	61,0	61,0	19,05	547,52	138,67
2019/05/25	CH2205	Male	Three y/o	Treatment (Wk 2)	Treatment	Fast Day	No	14:40	14:00 - 14:59	Afternoon	37,9	42	9,4	9,4	19,05	524,42	74,66
2019/05/25	CH2206	Male	Three y/o	Treatment (Wk 2)	Treatment	Fast Day	No	14:40	14:00 - 14:59	Afternoon	37,9	53	60,6	60,6	19,05	544,43	138,44
2019/05/25	CH2205	Male	Three y/o	Treatment (Wk 2)	Treatment	Fast Day	No	14:45	14:00 - 14:59	Afternoon	37,9		12,0	12,0	19,05		82,93
2019/05/25	CH2206	Male	Three y/o	Treatment (Wk 2)	Treatment	Fast Day	No	14:45	14:00 - 14:59	Afternoon	37,9	45	55,2	55,2	19,05	530,47	135,21
2019/05/25	CH2205	Male	Three y/o	Treatment (Wk 2)	Treatment	Fast Day	No	14:50	14:00 - 14:59	Afternoon	37,9		36,6	36,6	19,05		121,03
2019/05/25	CH2206	Male	Three y/o	Treatment (Wk 2)	Treatment	Fast Day	No	14:50	14:00 - 14:59	Afternoon	37,9	67	53,2	53,2	19,05	563,53	133,93
2019/05/25	CH2205	Male	Three y/o	Treatment (Wk 2)	Treatment	Fast Day	No	14:55	14:00 - 14:59	Afternoon	37,9	47	30,8	30,8	19,05	534,23	115,10
2019/05/25	CH2206	Male	Three y/o	Treatment (Wk 2)	Treatment	Fast Day	No	14:55	14:00 - 14:59	Afternoon	37,9	83	48,8	48,8	19,05	580,11	130,95
2019/05/25	CH2205	Male	Three y/o	Treatment (Wk 2)	Treatment	Fast Day	No	15:00	15:00 - 15:59	Afternoon	37,9	49	35,8	35,8	19,05	537,80	120,27
2019/05/25	CH2206	Male	Three y/o	Treatment (Wk 2)	Treatment	Fast Day	No	15:00	15:00 - 15:59	Afternoon	37,9	44	51,0	51,0	19,05	528,51	132,47
2019/05/25	CH2205	Male	Three y/o	Treatment (Wk 2)	Treatment	Fast Day	No	15:05	15:00 - 15:59	Afternoon	37,8	53	27,8	27,8	18,95	544,43	111,58
2019/05/25	CH2206	Male	Three y/o	Treatment (Wk 2)	Treatment	Fast Day	No	15:05	15:00 - 15:59	Afternoon	37,8	49	47,6	47,6	18,95	537,80	130,09
2019/05/25	CH2205	Male	Three y/o	Treatment (Wk 2)	Treatment	Fast Day	No	15:10	15:00 - 15:59	Afternoon	37,8		37,6	37,6	18,95		121,96
2019/05/25	CH2206	Male	Three y/o	Treatment (Wk 2)	Treatment	Fast Day	No	15:10	15:00 - 15:59	Afternoon	37,8	197	198,0	198,0	18,95	639,25	179,77
2019/05/25	CH2205	Male	Three y/o	Treatment (Wk 2)	Treatment	Fast Day	No	15:15	15:00 - 15:59	Afternoon	37,7	81	15,6	15,6	18,85	578,26	91,85
2019/05/25	CH2206	Male	Three y/o	Treatment (Wk 2)	Treatment	Fast Day	No	15:15	15:00 - 15:59	Afternoon	37,8	96	137,2	137,2	18,95	590,91	166,89
2019/05/25	CH2205	Male	Three y/o	Treatment (Wk 2)	Treatment	Fast Day	No	15:20	15:00 - 15:59	Afternoon	37,7	51	14,8	14,8	18,85	541,20	90,05
2019/05/25	CH2206	Male	Three y/o	Treatment (Wk 2)	Treatment	Fast Day	No	15:20	15:00 - 15:59	Afternoon	37,8		84,0	84,0	18,95		149,77
2019/05/25	CH2205	Male	Three y/o	Treatment (Wk 2)	Treatment	Fast Day	No	15:25	15:00 - 15:59	Afternoon	37,7	88	171,0	171,0	18,85	584,49	174,61
2019/05/25	CH2206	Male	Three y/o	Treatment (Wk 2)	Treatment	Fast Day	No	15:25	15:00 - 15:59	Afternoon	37,9	106	94,0	94,0	19,05	598,06	153,69
2019/05/25	CH2205	Male	Three y/o	Treatment (Wk 2)	Treatment	Fast Day	No	15:30	15:00 - 15:59	Afternoon	37,7	108	64,4	64,4	18,85	599,39	140,55
2019/05/25	CH2206	Male	Three y/o	Treatment (Wk 2)	Treatment	Fast Day	No	15:30	15:00 - 15:59	Afternoon	37,9	118	76,8	76,8	19,05	605,61	146,66
2019/05/25	CH2205	Male	Three y/o	Treatment (Wk 2)	Treatment	Fast Day	No	15:35	15:00 - 15:59	Afternoon	37,6		31,0	31,0	18,75		115,32
2019/05/25	CH2206	Male	Three y/o	Treatment (Wk 2)	Treatment	Fast Day	No	15:35	15:00 - 15:59	Afternoon	37,9	92	95,2	95,2	19,05	587,78	154,13
2019/05/25	CH2205	Male	Three y/o	Treatment (Wk 2)	Treatment	Fast Day	No	15:40	15:00 - 15:59	Afternoon	37,6	104	399,4	399,4	18,75	596,69	204,56
2019/05/25	CH2206	Male	Three y/o	Treatment (Wk 2)	Treatment	Fast Day	No	15:40	15:00 - 15:59	Afternoon	38,0	104	64,0	64,0	19,15	596,69	140,33
2019/05/25	CH2205	Male	Three y/o	Treatment (Wk 2)	Treatment	Fast Day	No	15:45	15:00 - 15:59	Afternoon	37,7	89	6,4	6,4	18,85	585,33	61,70
2019/05/25	CH2206	Male	Three y/o	Treatment (Wk 2)	Treatment	Fast Day	No	15:45	15:00 - 15:59	Afternoon	38,0	65	63,0	63,0	19,15	561,12	139,78
2019/05/25	CH2205	Male	Three y/o	Treatment (Wk 2)	Treatment	Fast Day	No	15:50	15:00 - 15:59	Afternoon	37,7		27,4	27,4	18,85		111,09
2019/05/25	CH2206	Male	Three y/o	Treatment (Wk 2)	Treatment	Fast Day	No	15:50	15:00 - 15:59	Afternoon	38,0	99	40,8	40,8	19,15	593,14	124,77
2019/05/25	CH2205	Male	Three y/o	Treatment (Wk 2)	Treatment	Fast Day	No	15:55	15:00 - 15:59	Afternoon	37,9	61	2,4	2,4	19,05	556,01	28,91
2019/05/25	CH2206	Male	Three y/o	Treatment (Wk 2)	Treatment	Fast Day	No	15:55	15:00 - 15:59	Afternoon	38,0	68	53,8	53,8	19,15	564,71	134,32
2019/05/25	CH2205	Male	Three y/o	Treatment (Wk 2)	Treatment	Fast Day	No	16:00	16:00 - 16:59	Evening	37,9	53	5,0	5,0	19,05	544,43	53,41
2019/05/25	CH2206	Male	Three y/o	Treatment (Wk 2)	Treatment	Fast Day	No	16:00	16:00 - 16:59	Evening	38,0	43	51,8	51,8	19,15	526,50	133,01
2019/05/25	CH2205	Male	Three y/o	Treatment (Wk 2)	Treatment	Fast Day	No	16:05	16:00 - 16:59	Evening	38,0	56	3,6	3,6	19,15	549,01	42,42
2019/05/25	CH2206	Male	Three y/o	Treatment (Wk 2)	Treatment	Fast Day	No	16:05	16:00 - 16:59	Evening	38,0	57	49,0	49,0	19,15	550,47	131,09
2019/05/25	CH2205	Male	Three y/o	Treatment (Wk 2)	Treatment	Fast Day	No	16:10	16:00 - 16:59	Evening	38,0	46	10,2	10,2	19,15	532,38	77,43
2019/05/25	CH2206	Male	Three y/o	Treatment (Wk 2)	Treatment	Fast Day	No	16:10	16:00 - 16:59	Evening	38,0	46	43,0	43,0	19,15	532,38	126,58
2019/05/25	CH2205	Male	Three y/o	Treatment (Wk 2)	Treatment	Fast Day	No	16:15	16:00 - 16:59	Evening	38,0	46	2,4	2,4	19,15	532,38	28,91
2019/05/25	CH2206	Male	Three y/o	Treatment (Wk 2)	Treatment	Fast Day	No	16:15	16:00 - 16:59	Evening	38,0	51	41,8	41,8	19,15	541,20	125,60
2019/05/25	CH2205	Male	Three y/o	Treatment (Wk 2)	Treatment	Fast Day	No	16:20	16:00 - 16:59	Evening	38,0	68	8,4	8,4	19,15	564,71	70,86
2019/05/25	CH2206	Male	Three y/o	Treatment (Wk 2)	Treatment	Fast Day	No	16:20	16:00 - 16:59	Evening	37,9	41	37,2	37,2	19,05	522,29	121,59
2019/05/25	CH2205	Male	Three y/o	Treatment (Wk 2)	Treatment	Fast Day	No	16:25	16:00 - 16:59	Evening	38,1	48	8,2	8,2	19,26	536,04	70,05
2019/05/25	CH2206	Male	Three y/o	Treatment (Wk 2)	Treatment	Fast Day	No	16:25	16:00 - 16:59	Evening	37,8	56	43,8	43,8	18,95	549,01	127,22
2019/05/25	CH2205	Male	Three y/o	Treatment (Wk 2)	Treatment	Fast Day	No	16:30	16:00 - 16:59	Evening	38,1	45	9,0	9,0	19,26	530,47	73,19

2019/05/25	CH2206	Male	Three y/o	Treatment (Wk 2)	Treatment	Fast Day	No	16:30	16:00 - 16:59	Evening	37,9	39	32,8	32,8	19,05	517,82	117,26
2019/05/25	CH2205	Male	Three y/o	Treatment (Wk 2)	Treatment	Fast Day	No	16:35	16:00 - 16:59	Evening	38,1	51	3,4	3,4	19,26	541,20	40,51
2019/05/25	CH2206	Male	Three y/o	Treatment (Wk 2)	Treatment	Fast Day	No	16:35	16:00 - 16:59	Evening	37,9	45	35,8	35,8	19,05	530,47	120,27
2019/05/25	CH2205	Male	Three y/o	Treatment (Wk 2)	Treatment	Fast Day	No	16:40	16:00 - 16:59	Evening	38,1	38	6,6	6,6	19,26	515,48	62,73
2019/05/25	CH2206	Male	Three y/o	Treatment (Wk 2)	Treatment	Fast Day	No	16:40	16:00 - 16:59	Evening	37,8	46	23,8	23,8	18,95	532,38	106,26
2019/05/25	CH2205	Male	Three y/o	Treatment (Wk 2)	Treatment	Fast Day	No	16:45	16:00 - 16:59	Evening	38,0	68	4,8	4,8	19,15	564,71	52,04
2019/05/25	CH2206	Male	Three y/o	Treatment (Wk 2)	Treatment	Fast Day	No	16:45	16:00 - 16:59	Evening	37,7	49	10,0	10,0	18,85	537,80	76,76
2019/05/25	CH2205	Male	Three y/o	Treatment (Wk 2)	Treatment	Fast Day	No	16:50	16:00 - 16:59	Evening	38,0	70	4,6	4,6	19,15	566,99	50,61
2019/05/25	CH2206	Male	Three y/o	Treatment (Wk 2)	Treatment	Fast Day	No	16:50	16:00 - 16:59	Evening	37,7	39	23,4	23,4	18,85	517,82	105,68
2019/05/25	CH2205	Male	Three y/o	Treatment (Wk 2)	Treatment	Fast Day	No	16:55	16:00 - 16:59	Evening	38,0	63	17,0	17,0	19,15	558,62	94,77
2019/05/25	CH2206	Male	Three y/o	Treatment (Wk 2)	Treatment	Fast Day	No	16:55	16:00 - 16:59	Evening	37,7	44	29,4	29,4	18,85	528,51	113,50
2019/05/25	CH2205	Male	Three y/o	Treatment (Wk 2)	Treatment	Fast Day	No	17:00	17:00 - 17:59	Evening	38,0		26,2	26,2	19,15		109,55
2019/05/25	CH2206	Male	Three y/o	Treatment (Wk 2)	Treatment	Fast Day	No	17:00	17:00 - 17:59	Evening	37,7	43	29,8	29,8	18,85	526,50	113,97
2019/05/25	CH2205	Male	Three y/o	Treatment (Wk 2)	Treatment	Fast Day	No	17:05	17:00 - 17:59	Evening	37,9		24,8	24,8	19,05		107,67
2019/05/25	CH2206	Male	Three y/o	Treatment (Wk 2)	Treatment	Fast Day	No	17:05	17:00 - 17:59	Evening	37,5	41	68,6	68,6	18,65	522,29	142,74
2019/05/25	CH2205	Male	Three y/o	Treatment (Wk 2)	Treatment	Fast Day	No	17:10	17:00 - 17:59	Evening	37,9	89	55,2	55,2	19,05	585,33	135,21
2019/05/25	CH2206	Male	Three y/o	Treatment (Wk 2)	Treatment	Fast Day	No	17:10	17:00 - 17:59	Evening	37,5	99	116,0	116,0	18,65	593,14	161,02
2019/05/25	CH2205	Male	Three y/o	Treatment (Wk 2)	Treatment	Fast Day	No	17:15	17:00 - 17:59	Evening	37,8		65,4	65,4	18,95		141,08
2019/05/25	CH2206	Male	Three y/o	Treatment (Wk 2)	Treatment	Fast Day	No	17:15	17:00 - 17:59	Evening	37,7	115	85,4	85,4	18,85	603,82	150,35
2019/05/25	CH2205	Male	Three y/o	Treatment (Wk 2)	Treatment	Fast Day	No	17:20	17:00 - 17:59	Evening	37,7	66	45,4	45,4	18,85	562,34	128,45
2019/05/25	CH2206	Male	Three y/o	Treatment (Wk 2)	Treatment	Fast Day	No	17:20	17:00 - 17:59	Evening	37,8	162	53,8	53,8	18,95	626,88	134,32
2019/05/25	CH2205	Male	Three y/o	Treatment (Wk 2)	Treatment	Fast Day	No	17:25	17:00 - 17:59	Evening	37,6	78	44,6	44,6	18,75	575,38	127,84
2019/05/25	CH2206	Male	Three y/o	Treatment (Wk 2)	Treatment	Fast Day	No	17:25	17:00 - 17:59	Evening	37,9		82,0	82,0	19,05		148,93
2019/05/25	CH2205	Male	Three y/o	Treatment (Wk 2)	Treatment	Fast Day	No	17:30	17:00 - 17:59	Evening	37,6		24,0	24,0	18,75		106,55
2019/05/25	CH2206	Male	Three y/o	Treatment (Wk 2)	Treatment	Fast Day	No	17:30	17:00 - 17:59	Evening	37,9	180	364,6	364,6	19,05	633,61	201,33
2019/05/25	CH2205	Male	Three y/o	Treatment (Wk 2)	Treatment	Fast Day	No	17:35	17:00 - 17:59	Evening	37,5	150	102,4	102,4	18,65	621,86	156,67
2019/05/25	CH2206	Male	Three y/o	Treatment (Wk 2)	Treatment	Fast Day	No	17:35	17:00 - 17:59	Evening	38,1	95	41,2	41,2	19,26	590,14	125,11
2019/05/25	CH2205	Male	Three y/o	Treatment (Wk 2)	Treatment	Fast Day	No	17:40	17:00 - 17:59	Evening	37,7		301,6	301,6	18,85		194,61
2019/05/25	CH2206	Male	Three y/o	Treatment (Wk 2)	Treatment	Fast Day	No	17:40	17:00 - 17:59	Evening	38,1	61	24,8	24,8	19,26	556,01	107,67
2019/05/25	CH2205	Male	Three y/o	Treatment (Wk 2)	Treatment	Fast Day	No	17:45	17:00 - 17:59	Evening	37,9	147	72,0	72,0	19,05	620,52	144,42
2019/05/25	CH2206	Male	Three y/o	Treatment (Wk 2)	Treatment	Fast Day	No	17:45	17:00 - 17:59	Evening	38,1	79	297,0	297,0	19,26	576,36	194,07
2019/05/25	CH2205	Male	Three y/o	Treatment (Wk 2)	Treatment	Fast Day	No	17:50	17:00 - 17:59	Evening	38,0	97	44,8	44,8	19,15	591,66	127,99
2019/05/25	CH2206	Male	Three y/o	Treatment (Wk 2)	Treatment	Fast Day	No	17:50	17:00 - 17:59	Evening	38,1	96	55,2	55,2	19,26	590,91	135,21
2019/05/25	CH2205	Male	Three y/o	Treatment (Wk 2)	Treatment	Fast Day	No	17:55	17:00 - 17:59	Evening	38,1	104	62,4	62,4	19,26	596,69	139,45
2019/05/25	CH2206	Male	Three y/o	Treatment (Wk 2)	Treatment	Fast Day	No	17:55	17:00 - 17:59	Evening	38,1	90	56,8	56,8	19,26	586,16	136,20
2019/05/25	CH2205	Male	Three y/o	Treatment (Wk 2)	Treatment	Fast Day	No	18:00	18:00 - 18:59	Evening	38,0		25,2	25,2	19,15		108,22
2019/05/25	CH2206	Male	Three y/o	Treatment (Wk 2)	Treatment	Fast Day	No	18:00	18:00 - 18:59	Evening	38,0	45	85,2	85,2	19,15	530,47	150,27
2019/05/25	CH2205	Male	Three y/o	Treatment (Wk 2)	Treatment	Fast Day	No	18:05	18:00 - 18:59	Evening	37,9	48	41,8	41,8	19,05	536,04	125,60
2019/05/25	CH2206	Male	Three y/o	Treatment (Wk 2)	Treatment	Fast Day	No	18:05	18:00 - 18:59	Evening	37,9	95	65,2	65,2	19,05	590,14	140,97
2019/05/25	CH2205	Male	Three y/o	Treatment (Wk 2)	Treatment	Fast Day	No	18:10	18:00 - 18:59	Evening	37,9	67	26,6	26,6	19,05	563,53	110,07
2019/05/25	CH2206	Male	Three y/o	Treatment (Wk 2)	Treatment	Fast Day	No	18:10	18:00 - 18:59	Evening	37,8	41	63,6	63,6	18,95	522,29	140,11
2019/05/25	CH2205	Male	Three y/o	Treatment (Wk 2)	Treatment	Fast Day	No	18:15	18:00 - 18:59	Evening	38,0	70	76,6	76,6	19,15	566,99	146,57
2019/05/25	CH2206	Male	Three y/o	Treatment (Wk 2)	Treatment	Fast Day	No	18:15	18:00 - 18:59	Evening	37,7	54	63,4	63,4	18,85	545,99	140,00
2019/05/25	CH2205	Male	Three y/o	Treatment (Wk 2)	Treatment	Fast Day	No	18:20	18:00 - 18:59	Evening	37,9	48	35,2	35,2	19,05	536,04	119,69
2019/05/25	CH2206	Male	Three y/o	Treatment (Wk 2)	Treatment	Fast Day	No	18:20	18:00 - 18:59	Evening	37,7	49	57,4	57,4	18,85	537,80	136,56
2019/05/25	CH2205	Male	Three y/o	Treatment (Wk 2)	Treatment	Fast Day	No	18:25	18:00 - 18:59	Evening	38,0	46	27,2	27,2	19,15	532,38	110,83
2019/05/25	CH2206	Male	Three y/o	Treatment (Wk 2)	Treatment	Fast Day	No	18:25	18:00 - 18:59	Evening	37,8	45	60,4	60,4	18,95	530,47	138,32
2019/05/25	CH2205	Male	Three y/o	Treatment (Wk 2)	Treatment	Fast Day	No	18:30	18:00 - 18:59	Evening	38,0	58	17,8	17,8	19,15	551,90	96,34
2019/05/25	CH2206	Male	Three y/o	Treatment (Wk 2)	Treatment	Fast Day	No	18:30	18:00 - 18:59	Evening	37,8	48	57,6	57,6	18,95	536,04	136,68
2019/05/25	CH2205	Male	Three y/o	Treatment (Wk 2)	Treatment	Fast Day	No	18:35	18:00 - 18:59	Evening	38,0	51	18,4	18,4	19,15	541,20	97,47
2019/05/25	CH2206	Male	Three y/o	Treatment (Wk 2)	Treatment	Fast Day	No	18:35	18:00 - 18:59	Evening	37,9	46	59,6	59,6	19,05	532,38	137,86
2019/05/25	CH2205	Male	Three y/o	Treatment (Wk 2)	Treatment	Fast Day	No	18:40	18:00 - 18:59	Evening	38,0	46	12,4	12,4	19,15	532,38	84,04
2019/05/25	CH2206	Male	Three y/o	Treatment (Wk 2)	Treatment	Fast Day	No	18:40	18:00 - 18:59	Evening	37,9	49	59,8	59,8	19,05	537,80	137,98
2019/05/25	CH2205	Male	Three y/o	Treatment (Wk 2)	Treatment	Fast Day	No	18:45	18:00 - 18:59	Evening	38,0	43	12,0	12,0	19,15	526,50	82,93
2019/05/25	CH2206	Male	Three y/o	Treatment (Wk 2)	Treatment	Fast Day	No	18:45	18:00 - 18:59	Evening	37,9	49	61,6	61,6	19,05	537,80	139,00
2019/05/25	CH2205	Male	Three y/o	Treatment (Wk 2)	Treatment	Fast Day	No	18:50	18:00 - 18:59	Evening	38,0	40	16,0	16,0	19,15	520,09	92,71
2019/05/25	CH2206	Male	Three y/o	Treatment (Wk 2)	Treatment	Fast Day	No	18:50	18:00 - 18:59	Evening	37,9	47	62,0	62,0	19,05	534,23	139,23
2019/05/25	CH2205	Male	Three y/o	Treatment (Wk 2)	Treatment	Fast Day	No	18:55	18:00 - 18:59	Evening	38,0	66	8,4	8,4	19,15	562,34	70,86
2019/05/25	CH2206	Male	Three y/o	Treatment (Wk 2)	Treatment	Fast Day	No	18:55	18:00 - 18:59	Evening	37,8	60	60,6	60,6	18,95	554,67	138,44
2019/05/25	CH2205	Male	Three y/o	Treatment (Wk 2)	Treatment	Fast Day	No	19:00	19:00 - 19:59	Evening	38,0	60	15,0	15,0	19,15	554,67	90,51
2019/05/25	CH2206	Male	Three y/o	Treatment (Wk 2)	Treatment	Fast Day	No	19:00	19:00 - 19:59	Evening	37,8	76	62,0	62,0	18,95	573,39	139,23
2019/05/25	CH2205	Male	Three y/o	Treatment (Wk 2)	Treatment	Fast Day	No	19:05	19:00 - 19:59	Evening	38,0	76	16,2	16,2	19,15	573,39	93,13
2019/05/25	CH2206	Male	Three y/o	Treatment (Wk 2)	Treatment	Fast Day	No	19:05	19:00 - 19:59	Evening	37,8	42	61,2	61,2	18,95	524,42	138,78
2019/05/25	CH2205	Male	Three y/o	Treatment (Wk 2)	Treatment	Fast Day	No	19:10	19:00 - 19:59	Evening	38,0	35	13,0	13,0	19,15	507,97	85,65
2019/05/25	CH2206	Male	Three y/o	Treatment (Wk 2)	Treatment	Fast Day	No	19:10	19:00 - 19:59	Evening	37,8	51	56,0	56,0	18,95	541,20	135,70
2019/05/25	CH2205	Male	Three y/o	Treatment (Wk 2)	Treatment	Fast Day	No	19:15	19:00 - 19:59	Evening	37,9	60	17,8	17,8	19,05	554,67	96,34
2019/05/25	CH2206	Male	Three y/o	Treatment (Wk 2)	Treatment	Fast Day	No	19:15	19:00 - 19:59	Evening	37,7	44	62,8	62,8	18,85	528,51	139,67
2019/05/25	CH2205	Male	Three y/o	Treatment (Wk 2)	Treatment	Fast Day	No	19:20	19:00 - 19:59	Evening	37,9	48	16,0	16,0	19,05	536,04	92,71
2019/05/25	CH2206	Male	Three y/o	Treatment (Wk 2)	Treatment	Fast Day	No	19:20	19:00 - 19:59	Evening	37,4	45	58,8	58,8	18,55	530,47	137,39
2019/05/25	CH2205	Male	Three y/o	Treatment (Wk 2)	Treatment	Fast Day	No	19:25	19:00 - 19:59	Evening	37,9	44	13,6	13,6	19,05	528,51	87,18

2019/05/25	CH2206	Male	Three y/o	Treatment (Wk 2)	Treatment	Fast Day	No	19:25	19:00 - 19:59	Evening	37,4	41	60,0	60,0	18,55	522,29	138,09
2019/05/25	CH2205	Male	Three y/o	Treatment (Wk 2)	Treatment	Fast Day	No	19:30	19:00 - 19:59	Evening	37,9	42	14,0	14,0	19,05	524,42	88,17
2019/05/25	CH2206	Male	Three y/o	Treatment (Wk 2)	Treatment	Fast Day	No	19:30	19:00 - 19:59	Evening	37,4	45	59,8	59,8	18,55	530,47	137,98
2019/05/25	CH2205	Male	Three y/o	Treatment (Wk 2)	Treatment	Fast Day	No	19:35	19:00 - 19:59	Evening	37,9	46	20,0	20,0	19,05	532,38	100,31
2019/05/25	CH2206	Male	Three y/o	Treatment (Wk 2)	Treatment	Fast Day	No	19:35	19:00 - 19:59	Evening	37,5	39	58,0	58,0	18,65	517,82	136,92
2019/05/25	CH2205	Male	Three y/o	Treatment (Wk 2)	Treatment	Fast Day	No	19:40	19:00 - 19:59	Evening	37,8	47	26,8	26,8	18,95	534,23	110,33
2019/05/25	CH2206	Male	Three y/o	Treatment (Wk 2)	Treatment	Fast Day	No	19:40	19:00 - 19:59	Evening	37,5		58,6	58,6	18,65		137,28
2019/05/25	CH2205	Male	Three y/o	Treatment (Wk 2)	Treatment	Fast Day	No	19:45	19:00 - 19:59	Evening	37,7	50	27,2	27,2	18,85	539,52	110,83
2019/05/25	CH2206	Male	Three y/o	Treatment (Wk 2)	Treatment	Fast Day	No	19:45	19:00 - 19:59	Evening	37,7	44	59,0	59,0	18,85	528,51	137,51
2019/05/25	CH2205	Male	Three y/o	Treatment (Wk 2)	Treatment	Fast Day	No	19:50	19:00 - 19:59	Evening	37,7	50	22,6	22,6	18,85	539,52	104,49
2019/05/25	CH2206	Male	Three y/o	Treatment (Wk 2)	Treatment	Fast Day	No	19:50	19:00 - 19:59	Evening	37,7	39	54,0	54,0	18,85	517,82	134,45
2019/05/25	CH2205	Male	Three y/o	Treatment (Wk 2)	Treatment	Fast Day	No	19:55	19:00 - 19:59	Evening	37,7	68	23,2	23,2	18,85	564,71	105,39
2019/05/25	CH2206	Male	Three y/o	Treatment (Wk 2)	Treatment	Fast Day	No	19:55	19:00 - 19:59	Evening	37,7	94	46,8	46,8	18,85	589,37	129,50
2019/05/25	CH2205	Male	Three y/o	Treatment (Wk 2)	Treatment	Fast Day	No	20:00	20:00 - 20:59	Night	37,7	36	22,2	22,2	18,85	510,56	103,88
2019/05/25	CH2206	Male	Three y/o	Treatment (Wk 2)	Treatment	Fast Day	No	20:00	20:00 - 20:59	Night	37,7	41	54,8	54,8	18,85	522,29	134,96
2019/05/25	CH2205	Male	Three y/o	Treatment (Wk 2)	Treatment	Fast Day	No	20:05	20:00 - 20:59	Night	37,7	43	20,8	20,8	18,85	526,50	101,65
2019/05/25	CH2206	Male	Three y/o	Treatment (Wk 2)	Treatment	Fast Day	No	20:05	20:00 - 20:59	Night	37,7	48	55,8	55,8	18,85	536,04	135,58
2019/05/25	CH2205	Male	Three y/o	Treatment (Wk 2)	Treatment	Fast Day	No	20:10	20:00 - 20:59	Night	37,7	44	23,2	23,2	18,85	528,51	105,39
2019/05/25	CH2206	Male	Three y/o	Treatment (Wk 2)	Treatment	Fast Day	No	20:10	20:00 - 20:59	Night	37,7	39	47,6	47,6	18,85	517,82	130,09
2019/05/25	CH2205	Male	Three y/o	Treatment (Wk 2)	Treatment	Fast Day	No	20:15	20:00 - 20:59	Night	37,6	43	18,2	18,2	18,75	526,50	97,10
2019/05/25	CH2206	Male	Three y/o	Treatment (Wk 2)	Treatment	Fast Day	No	20:15	20:00 - 20:59	Night	37,7	47	46,4	46,4	18,85	534,23	129,21
2019/05/25	CH2205	Male	Three y/o	Treatment (Wk 2)	Treatment	Fast Day	No	20:20	20:00 - 20:59	Night	37,6	51	20,0	20,0	18,75	541,20	100,31
2019/05/25	CH2206	Male	Three y/o	Treatment (Wk 2)	Treatment	Fast Day	No	20:20	20:00 - 20:59	Night	37,7	49	45,2	45,2	18,85	537,80	128,30
2019/05/25	CH2205	Male	Three y/o	Treatment (Wk 2)	Treatment	Fast Day	No	20:25	20:00 - 20:59	Night	37,6	64	18,8	18,8	18,75	559,88	98,20
2019/05/25	CH2206	Male	Three y/o	Treatment (Wk 2)	Treatment	Fast Day	No	20:25	20:00 - 20:59	Night	37,7	42	50,0	50,0	18,85	524,42	131,79
2019/05/25	CH2205	Male	Three y/o	Treatment (Wk 2)	Treatment	Fast Day	No	20:30	20:00 - 20:59	Night	37,6	41	17,6	17,6	18,75	522,29	95,95
2019/05/25	CH2206	Male	Three y/o	Treatment (Wk 2)	Treatment	Fast Day	No	20:30	20:00 - 20:59	Night	37,7	63	44,8	44,8	18,85	558,62	127,99
2019/05/25	CH2205	Male	Three y/o	Treatment (Wk 2)	Treatment	Fast Day	No	20:35	20:00 - 20:59	Night	37,6	49	22,2	22,2	18,75	537,80	103,88
2019/05/25	CH2206	Male	Three y/o	Treatment (Wk 2)	Treatment	Fast Day	No	20:35	20:00 - 20:59	Night	37,5	45	40,8	40,8	18,65	530,47	124,77
2019/05/25	CH2205	Male	Three y/o	Treatment (Wk 2)	Treatment	Fast Day	No	20:40	20:00 - 20:59	Night	37,6	61	15,4	15,4	18,75	556,01	91,41
2019/05/25	CH2206	Male	Three y/o	Treatment (Wk 2)	Treatment	Fast Day	No	20:40	20:00 - 20:59	Night	37,5	42	42,4	42,4	18,65	524,42	126,10
2019/05/25	CH2205	Male	Three y/o	Treatment (Wk 2)	Treatment	Fast Day	No	20:45	20:00 - 20:59	Night	37,5	50	14,4	14,4	18,65	539,52	89,12
2019/05/25	CH2206	Male	Three y/o	Treatment (Wk 2)	Treatment	Fast Day	No	20:45	20:00 - 20:59	Night	37,5	36	46,0	46,0	18,65	510,56	128,91
2019/05/25	CH2205	Male	Three y/o	Treatment (Wk 2)	Treatment	Fast Day	No	20:50	20:00 - 20:59	Night	37,5	49	17,0	17,0	18,65	537,80	94,77
2019/05/25	CH2206	Male	Three y/o	Treatment (Wk 2)	Treatment	Fast Day	No	20:50	20:00 - 20:59	Night	37,5	48	40,2	40,2	18,65	536,04	124,26
2019/05/25	CH2205	Male	Three y/o	Treatment (Wk 2)	Treatment	Fast Day	No	20:55	20:00 - 20:59	Night	37,5	47	25,2	25,2	18,65	534,23	108,22
2019/05/25	CH2206	Male	Three y/o	Treatment (Wk 2)	Treatment	Fast Day	No	20:55	20:00 - 20:59	Night	37,4		44,0	44,0	18,55		127,37
2019/05/25	CH2205	Male	Three y/o	Treatment (Wk 2)	Treatment	Fast Day	No	21:00	21:00 - 21:59	Night	37,6	87	40,0	40,0	18,75	583,64	124,09
2019/05/25	CH2206	Male	Three y/o	Treatment (Wk 2)	Treatment	Fast Day	No	21:00	21:00 - 21:59	Night	37,4	42	55,8	55,8	18,55	524,42	135,58
2019/05/25	CH2205	Male	Three y/o	Treatment (Wk 2)	Treatment	Fast Day	No	21:05	21:00 - 21:59	Night	37,5		29,0	29,0	18,65		113,03
2019/05/25	CH2206	Male	Three y/o	Treatment (Wk 2)	Treatment	Fast Day	No	21:05	21:00 - 21:59	Night	37,4	63	64,2	64,2	18,55	558,62	140,44
2019/05/25	CH2205	Male	Three y/o	Treatment (Wk 2)	Treatment	Fast Day	No	21:10	21:00 - 21:59	Night	37,4		31,0	31,0	18,55		115,32
2019/05/25	CH2206	Male	Three y/o	Treatment (Wk 2)	Treatment	Fast Day	No	21:10	21:00 - 21:59	Night	37,4	49	61,2	61,2	18,55	537,80	138,78
2019/05/25	CH2205	Male	Three y/o	Treatment (Wk 2)	Treatment	Fast Day	No	21:15	21:00 - 21:59	Night	37,4		32,2	32,2	18,55		116,63
2019/05/25	CH2206	Male	Three y/o	Treatment (Wk 2)	Treatment	Fast Day	No	21:15	21:00 - 21:59	Night	37,4	42	40,6	40,6	18,55	524,42	124,60
2019/05/25	CH2205	Male	Three y/o	Treatment (Wk 2)	Treatment	Fast Day	No	21:20	21:00 - 21:59	Night	37,4	55	29,2	29,2	18,55	547,52	113,27
2019/05/25	CH2206	Male	Three y/o	Treatment (Wk 2)	Treatment	Fast Day	No	21:20	21:00 - 21:59	Night	37,4	42	34,2	34,2	18,55	524,42	118,70
2019/05/25	CH2205	Male	Three y/o	Treatment (Wk 2)	Treatment	Fast Day	No	21:25	21:00 - 21:59	Night	37,4		27,0	27,0	18,55		110,58
2019/05/25	CH2206	Male	Three y/o	Treatment (Wk 2)	Treatment	Fast Day	No	21:25	21:00 - 21:59	Night	37,4	38	40,0	40,0	18,55	515,48	124,09
2019/05/25	CH2205	Male	Three y/o	Treatment (Wk 2)	Treatment	Fast Day	No	21:30	21:00 - 21:59	Night	37,4		30,8	30,8	18,55		115,10
2019/05/25	CH2206	Male	Three y/o	Treatment (Wk 2)	Treatment	Fast Day	No	21:30	21:00 - 21:59	Night	37,4	43	37,6	37,6	18,55	526,50	121,96
2019/05/25	CH2205	Male	Three y/o	Treatment (Wk 2)	Treatment	Fast Day	No	21:35	21:00 - 21:59	Night	37,4		30,0	30,0	18,55		114,20
2019/05/25	CH2206	Male	Three y/o	Treatment (Wk 2)	Treatment	Fast Day	No	21:35	21:00 - 21:59	Night	37,4	47	37,4	37,4	18,55	534,23	121,77
2019/05/25	CH2205	Male	Three y/o	Treatment (Wk 2)	Treatment	Fast Day	No	21:40	21:00 - 21:59	Night	37,3	47	24,8	24,8	18,46	534,23	107,67
2019/05/25	CH2206	Male	Three y/o	Treatment (Wk 2)	Treatment	Fast Day	No	21:40	21:00 - 21:59	Night	37,4	57	40,4	40,4	18,55	550,47	124,43
2019/05/25	CH2205	Male	Three y/o	Treatment (Wk 2)	Treatment	Fast Day	No	21:45	21:00 - 21:59	Night	37,3	48	23,4	23,4	18,46	536,04	105,68
2019/05/25	CH2206	Male	Three y/o	Treatment (Wk 2)	Treatment	Fast Day	No	21:45	21:00 - 21:59	Night	37,3	45	34,4	34,4	18,46	530,47	118,90
2019/05/25	CH2205	Male	Three y/o	Treatment (Wk 2)	Treatment	Fast Day	No	21:50	21:00 - 21:59	Night	37,3	44	26,2	26,2	18,46	528,51	109,55
2019/05/25	CH2206	Male	Three y/o	Treatment (Wk 2)	Treatment	Fast Day	No	21:50	21:00 - 21:59	Night	37,4	43	36,4	36,4	18,55	526,50	120,84
2019/05/25	CH2205	Male	Three y/o	Treatment (Wk 2)	Treatment	Fast Day	No	21:55	21:00 - 21:59	Night	37,3	80	35,2	35,2	18,46	577,31	119,69
2019/05/25	CH2206	Male	Three y/o	Treatment (Wk 2)	Treatment	Fast Day	No	21:55	21:00 - 21:59	Night	37,4	55	37,4	37,4	18,55	547,52	121,77
2019/05/25	CH2205	Male	Three y/o	Treatment (Wk 2)	Treatment	Fast Day	No	22:00	22:00 - 22:59	Night	37,3	50	31,4	31,4	18,46	539,52	115,76
2019/05/25	CH2206	Male	Three y/o	Treatment (Wk 2)	Treatment	Fast Day	No	22:00	22:00 - 22:59	Night	37,4	46	36,0	36,0	18,55	532,38	120,46
2019/05/25	CH2205	Male	Three y/o	Treatment (Wk 2)	Treatment	Fast Day	No	22:05	22:00 - 22:59	Night	37,4	41	25,2	25,2	18,55	522,29	108,22
2019/05/25	CH2206	Male	Three y/o	Treatment (Wk 2)	Treatment	Fast Day	No	22:05	22:00 - 22:59	Night	37,4	44	32,0	32,0	18,55	528,51	116,41
2019/05/25	CH2205	Male	Three y/o	Treatment (Wk 2)	Treatment	Fast Day	No	22:10	22:00 - 22:59	Night	37,3	79	30,6	30,6	18,46	576,36	114,87
2019/05/25	CH2206	Male	Three y/o	Treatment (Wk 2)	Treatment	Fast Day	No	22:10	22:00 - 22:59	Night	37,4	54	33,2	33,2	18,55	545,99	117,68
2019/05/25	CH2205	Male	Three y/o	Treatment (Wk 2)	Treatment	Fast Day	No	22:15	22:00 - 22:59	Night	37,3	47	27,8	27,8	18,46	534,23	111,58
2019/05/25	CH2206	Male	Three y/o	Treatment (Wk 2)	Treatment	Fast Day	No	22:15	22:00 - 22:59	Night	37,4	59	35,2	35,2	18,55	553,30	119,69
2019/05/25	CH2205	Male	Three y/o	Treatment (Wk 2)	Treatment	Fast Day	No	22:20	22:00 - 22:59	Night	37,3	48	26,8	26,8	18,46	536,04	110,33

	2019/05/25	CH2206	Male	Three y/o	Treatment (Wk 2)	Treatment	Fast Day	No	22:20	22:00 - 22:59	Night	37,4	49	27,4	27,4	18,55	537,80	111,09
	2019/05/25	CH2205	Male	Three y/o	Treatment (Wk 2)	Treatment	Fast Day	No	22:25	22:00 - 22:59	Night	37,3	48	32,8	32,8	18,46	536,04	117,26
	2019/05/25	CH2206	Male	Three y/o	Treatment (Wk 2)	Treatment	Fast Day	No	22:25	22:00 - 22:59	Night	37,4	48	18,8	18,8	18,55	536,04	98,20
	2019/05/25	CH2205	Male	Three y/o	Treatment (Wk 2)	Treatment	Fast Day	No	22:30	22:00 - 22:59	Night	37,3		34,8	34,8	18,46		119,29
	2019/05/25	CH2206	Male	Three y/o	Treatment (Wk 2)	Treatment	Fast Day	No	22:30	22:00 - 22:59	Night	37,4	43	30,6	30,6	18,55	526,50	114,87
	2019/05/25	CH2205	Male	Three y/o	Treatment (Wk 2)	Treatment	Fast Day	No	22:35	22:00 - 22:59	Night	37,2		30,8	30,8	18,36		115,10
	2019/05/25	CH2206	Male	Three y/o	Treatment (Wk 2)	Treatment	Fast Day	No	22:35	22:00 - 22:59	Night	37,4	48	27,0	27,0	18,55	536,04	110,58
	2019/05/25	CH2205	Male	Three y/o	Treatment (Wk 2)	Treatment	Fast Day	No	22:40	22:00 - 22:59	Night	37,1		26,6	26,6	18,26		110,07
	2019/05/25	CH2206	Male	Three y/o	Treatment (Wk 2)	Treatment	Fast Day	No	22:40	22:00 - 22:59	Night	37,4	45	21,6	21,6	18,55	530,47	102,94
	2019/05/25	CH2205	Male	Three y/o	Treatment (Wk 2)	Treatment	Fast Day	No	22:45	22:00 - 22:59	Night	37,2		25,4	25,4	18,36		108,49
	2019/05/25	CH2206	Male	Three y/o	Treatment (Wk 2)	Treatment	Fast Day	No	22:45	22:00 - 22:59	Night	37,4	68	24,8	24,8	18,55	564,71	107,67
	2019/05/25	CH2205	Male	Three y/o	Treatment (Wk 2)	Treatment	Fast Day	No	22:50	22:00 - 22:59	Night	37,3		24,4	24,4	18,46		107,11
	2019/05/25	CH2206	Male	Three y/o	Treatment (Wk 2)	Treatment	Fast Day	No	22:50	22:00 - 22:59	Night	37,4	49	22,2	22,2	18,55	537,80	103,88
	2019/05/25	CH2205	Male	Three y/o	Treatment (Wk 2)	Treatment	Fast Day	No	22:55	22:00 - 22:59	Night	37,4	51	10,6	10,6	18,55	541,20	78,73
	2019/05/25	CH2206	Male	Three y/o	Treatment (Wk 2)	Treatment	Fast Day	No	22:55	22:00 - 22:59	Night	37,4	45	33,4	33,4	18,55	530,47	117,88
	2019/05/25	CH2205	Male	Three y/o	Treatment (Wk 2)	Treatment	Fast Day	No	23:00	23:00 - 23:59	Night	37,4	50	11,8	11,8	18,55	539,52	82,36
	2019/05/25	CH2206	Male	Three y/o	Treatment (Wk 2)	Treatment	Fast Day	No	23:00	23:00 - 23:59	Night	37,4	40	31,4	31,4	18,55	520,09	115,76
	2019/05/25	CH2205	Male	Three y/o	Treatment (Wk 2)	Treatment	Fast Day	No	23:05	23:00 - 23:59	Night	37,4	46	10,2	10,2	18,55	532,38	77,43
	2019/05/25	CH2206	Male	Three y/o	Treatment (Wk 2)	Treatment	Fast Day	No	23:05	23:00 - 23:59	Night	37,4	46	18,0	18,0	18,55	532,38	96,72
	2019/05/25	CH2205	Male	Three y/o	Treatment (Wk 2)	Treatment	Fast Day	No	23:10	23:00 - 23:59	Night	37,4	48	15,0	15,0	18,55	536,04	90,51
	2019/05/25	CH2206	Male	Three y/o	Treatment (Wk 2)	Treatment	Fast Day	No	23:10	23:00 - 23:59	Night	37,4	44	14,6	14,6	18,55	528,51	89,59
	2019/05/25	CH2205	Male	Three y/o	Treatment (Wk 2)	Treatment	Fast Day	No	23:15	23:00 - 23:59	Night	37,4	50	16,0	16,0	18,55	539,52	92,71
	2019/05/25	CH2206	Male	Three y/o	Treatment (Wk 2)	Treatment	Fast Day	No	23:15	23:00 - 23:59	Night	37,4	42	19,0	19,0	18,55	524,42	98,56
	2019/05/25	CH2205	Male	Three y/o	Treatment (Wk 2)	Treatment	Fast Day	No	23:20	23:00 - 23:59	Night	37,4	39	12,6	12,6	18,55	517,82	84,59
	2019/05/25	CH2206	Male	Three y/o	Treatment (Wk 2)	Treatment	Fast Day	No	23:20	23:00 - 23:59	Night	37,4	44	18,4	18,4	18,55	528,51	97,47
	2019/05/25	CH2205	Male	Three y/o	Treatment (Wk 2)	Treatment	Fast Day	No	23:25	23:00 - 23:59	Night	37,4	55	5,0	5,0	18,55	547,52	53,41
	2019/05/25	CH2206	Male	Three y/o	Treatment (Wk 2)	Treatment	Fast Day	No	23:25	23:00 - 23:59	Night	37,4	43	20,2	20,2	18,55	526,50	100,65
	2019/05/25	CH2205	Male	Three y/o	Treatment (Wk 2)	Treatment	Fast Day	No	23:30	23:00 - 23:59	Night	37,4	56	8,0	8,0	18,55	549,01	69,22
	2019/05/25	CH2206	Male	Three y/o	Treatment (Wk 2)	Treatment	Fast Day	No	23:30	23:00 - 23:59	Night	37,4	37	18,2	18,2	18,55	513,06	97,10
	2019/05/25	CH2205	Male	Three y/o	Treatment (Wk 2)	Treatment	Fast Day	No	23:35	23:00 - 23:59	Night	37,4	80	8,0	8,0	18,55	577,31	69,22
	2019/05/25	CH2206	Male	Three y/o	Treatment (Wk 2)	Treatment	Fast Day	No	23:35	23:00 - 23:59	Night	37,4	63	19,6	19,6	18,55	558,62	99,63
	2019/05/25	CH2205	Male	Three y/o	Treatment (Wk 2)	Treatment	Fast Day	No	23:40	23:00 - 23:59	Night	37,4	46	12,6	12,6	18,55	532,38	84,59
	2019/05/25	CH2206	Male	Three y/o	Treatment (Wk 2)	Treatment	Fast Day	No	23:40	23:00 - 23:59	Night	37,4	44	18,6	18,6	18,55	528,51	97,84
	2019/05/25	CH2205	Male	Three y/o	Treatment (Wk 2)	Treatment	Fast Day	No	23:45	23:00 - 23:59	Night	37,4	65	6,4	6,4	18,55	561,12	61,70
	2019/05/25	CH2206	Male	Three y/o	Treatment (Wk 2)	Treatment	Fast Day	No	23:45	23:00 - 23:59	Night	37,4	51	20,6	20,6	18,55	541,20	101,32
	2019/05/25	CH2205	Male	Three y/o	Treatment (Wk 2)	Treatment	Fast Day	No	23:50	23:00 - 23:59	Night	37,4	62	11,0	11,0	18,55	557,33	79,98
	2019/05/25	CH2206	Male	Three y/o	Treatment (Wk 2)	Treatment	Fast Day	No	23:50	23:00 - 23:59	Night	37,4	45	21,0	21,0	18,55	530,47	101,98
	2019/05/25	CH2205	Male	Three y/o	Treatment (Wk 2)	Treatment	Fast Day	No	23:55	23:00 - 23:59	Night	37,4	40	10,4	10,4	18,55	520,09	78,08
	2019/05/25	CH2206	Male	Three y/o	Treatment (Wk 2)	Treatment	Fast Day	No	23:55	23:00 - 23:59	Night	37,4	49	9,4	9,4	18,55	537,80	74,66
12	2019/05/26	CH2205	Male	Three y/o	Treatment (Wk 2)	Treatment	Fast Day	No	00:00	00:00 - 00:59	Early Morning	37,4	64	9,2	9,2	18,55	559,88	73,94
	2019/05/26	CH2206	Male	Three y/o	Treatment (Wk 2)	Treatment	Fast Day	No	00:00	00:00 - 00:59	Early Morning	37,4	42	20,4	20,4	18,55	524,42	100,99
	2019/05/26	CH2205	Male	Three y/o	Treatment (Wk 2)	Treatment	Fast Day	No	00:05	00:00 - 00:59	Early Morning	37,3	43	11,2	11,2	18,46	526,50	80,59
	2019/05/26	CH2206	Male	Three y/o	Treatment (Wk 2)	Treatment	Fast Day	No	00:05	00:00 - 00:59	Early Morning	37,4	55	18,4	18,4	18,55	547,52	97,47
	2019/05/26	CH2205	Male	Three y/o	Treatment (Wk 2)	Treatment	Fast Day	No	00:10	00:00 - 00:59	Early Morning	37,3	41	9,4	9,4	18,46	522,29	74,66
	2019/05/26	CH2206	Male	Three y/o	Treatment (Wk 2)	Treatment	Fast Day	No	00:10	00:00 - 00:59	Early Morning	37,3	46	17,2	17,2	18,46	532,38	95,17
	2019/05/26	CH2205	Male	Three y/o	Treatment (Wk 2)	Treatment	Fast Day	No	00:15	00:00 - 00:59	Early Morning	37,4	38	16,4	16,4	18,55	515,48	93,55
	2019/05/26	CH2206	Male	Three y/o	Treatment (Wk 2)	Treatment	Fast Day	No	00:15	00:00 - 00:59	Early Morning	37,2	48	18,6	18,6	18,36	536,04	97,84
	2019/05/26	CH2205	Male	Three y/o	Treatment (Wk 2)	Treatment	Fast Day	No	00:20	00:00 - 00:59	Early Morning	37,5		12,4	12,4	18,65		84,04
	2019/05/26	CH2206	Male	Three y/o	Treatment (Wk 2)	Treatment	Fast Day	No	00:20	00:00 - 00:59	Early Morning	37,1	43	24,0	24,0	18,26	526,50	106,55
	2019/05/26	CH2205	Male	Three y/o	Treatment (Wk 2)	Treatment	Fast Day	No	00:25	00:00 - 00:59	Early Morning	37,5	63	17,2	17,2	18,65	558,62	95,17
	2019/05/26	CH2206	Male	Three y/o	Treatment (Wk 2)	Treatment	Fast Day	No	00:25	00:00 - 00:59	Early Morning	37,1	100	21,4	21,4	18,26	593,87	102,63
	2019/05/26	CH2205	Male	Three y/o	Treatment (Wk 2)	Treatment	Fast Day	No	00:30	00:00 - 00:59	Early Morning	37,5	50	15,2	15,2	18,65	539,52	90,96
	2019/05/26	CH2206	Male	Three y/o	Treatment (Wk 2)	Treatment	Fast Day	No	00:30	00:00 - 00:59	Early Morning	37,1	51	15,8	15,8	18,26	541,20	92,28
	2019/05/26	CH2205	Male	Three y/o	Treatment (Wk 2)	Treatment	Fast Day	No	00:35	00:00 - 00:59	Early Morning	37,5	46	15,0	15,0	18,65	532,38	90,51
	2019/05/26	CH2206	Male	Three y/o	Treatment (Wk 2)	Treatment	Fast Day	No	00:35	00:00 - 00:59	Early Morning	37,1	67	12,6	12,6	18,26	563,53	84,59
	2019/05/26	CH2205	Male	Three y/o	Treatment (Wk 2)	Treatment	Fast Day	No	00:40	00:00 - 00:59	Early Morning	37,5	36	10,2	10,2	18,65	510,56	77,43
	2019/05/26	CH2206	Male	Three y/o	Treatment (Wk 2)	Treatment	Fast Day	No	00:40	00:00 - 00:59	Early Morning	37,2	78	11,8	11,8	18,36	575,38	82,36
	2019/05/26	CH2205	Male	Three y/o	Treatment (Wk 2)	Treatment	Fast Day	No	00:45	00:00 - 00:59	Early Morning	37,5	63	13,0	13,0	18,65	558,62	85,65
	2019/05/26	CH2206	Male	Three y/o	Treatment (Wk 2)	Treatment	Fast Day	No	00:45	00:00 - 00:59	Early Morning	37,2	55	7,4	7,4	18,36	547,52	66,59
	2019/05/26	CH2205	Male	Three y/o	Treatment (Wk 2)	Treatment	Fast Day	No	00:50	00:00 - 00:59	Early Morning	37,4	39	13,0	13,0	18,55	517,82	85,65
	2019/05/26	CH2206	Male	Three y/o	Treatment (Wk 2)	Treatment	Fast Day	No	00:50	00:00 - 00:59	Early Morning	37,2	80	14,4	14,4	18,36	577,31	89,12
	2019/05/26	CH2205	Male	Three y/o	Treatment (Wk 2)	Treatment	Fast Day	No	00:55	00:00 - 00:59	Early Morning	37,4	82	11,6	11,6	18,55	579,19	81,78
	2019/05/26	CH2206	Male	Three y/o	Treatment (Wk 2)	Treatment	Fast Day	No	00:55	00:00 - 00:59	Early Morning	37,2	57	7,8	7,8	18,36	550,47	68,36
	2019/05/26	CH2205	Male	Three y/o	Treatment (Wk 2)	Treatment	Fast Day	No	01:00	01:00 - 01:59	Early Morning	37,4		13,0	13,0	18,55		85,65
	2019/05/26	CH2206	Male	Three y/o	Treatment (Wk 2)	Treatment	Fast Day	No	01:00	01:00 - 01:59	Early Morning	37,1	79	42,2	42,2	18,26	576,36	125,93
	2019/05/26	CH2205	Male	Three y/o	Treatment (Wk 2)	Treatment	Fast Day	No	01:05	01:00 - 01:59	Early Morning	37,3	101	21,4	21,4	18,46	594,59	102,63
	2019/05/26	CH2206	Male	Three y/o	Treatment (Wk 2)	Treatment	Fast Day	No	01:05	01:00 - 01:59	Early Morning	37,2	82	63,4	63,4	18,36	579,19	140,00
	2019/05/26	CH2205	Male	Three y/o	Treatment (Wk 2)	Treatment	Fast Day	No	01:10	01:00 - 01:59	Early Morning	37,3	81	24,8	24,8	18,46	578,26	107,67
	2019/05/26	CH2206	Male	Three y/o	Treatment (Wk 2)	Treatment	Fast Day	No	01:10	01:00 - 01:59	Early Morning	37,2	39	62,0	62,0	18,36	517,82	139,23
	2019/05/26	CH2205	Male	Three y/o	Treatment (Wk 2)	Treatment	Fast Day	No	01:15	01:00 - 01:59	Early Morning	37,3		16,6	16,6	18,46		93,96

2019/05/26	CH2206	Male	Three y/o	Treatment (Wk 2)	Treatment	Fast Day	No	01:15	01:00 - 01:59	Early Morning	37,2	40	65,8	65,8	18,36	520,09	141,29
2019/05/26	CH2205	Male	Three y/o	Treatment (Wk 2)	Treatment	Fast Day	No	01:20	01:00 - 01:59	Early Morning	37,3		10,0	10,0	18,46		76,76
2019/05/26	CH2206	Male	Three y/o	Treatment (Wk 2)	Treatment	Fast Day	No	01:20	01:00 - 01:59	Early Morning	37,3	79	62,6	62,6	18,46	576,36	139,56
2019/05/26	CH2205	Male	Three y/o	Treatment (Wk 2)	Treatment	Fast Day	No	01:25	01:00 - 01:59	Early Morning	37,3		15,2	15,2	18,46		90,96
2019/05/26	CH2206	Male	Three y/o	Treatment (Wk 2)	Treatment	Fast Day	No	01:25	01:00 - 01:59	Early Morning	37,3	128	64,2	64,2	18,46	611,22	140,44
2019/05/26	CH2205	Male	Three y/o	Treatment (Wk 2)	Treatment	Fast Day	No	01:30	01:00 - 01:59	Early Morning	37,3		8,0	8,0	18,46		69,22
2019/05/26	CH2206	Male	Three y/o	Treatment (Wk 2)	Treatment	Fast Day	No	01:30	01:00 - 01:59	Early Morning	37,4	68	3,4	3,4	18,55	564,71	40,51
2019/05/26	CH2205	Male	Three y/o	Treatment (Wk 2)	Treatment	Fast Day	No	01:35	01:00 - 01:59	Early Morning	37,3	76	8,8	8,8	18,46	573,39	72,43
2019/05/26	CH2206	Male	Three y/o	Treatment (Wk 2)	Treatment	Fast Day	No	01:35	01:00 - 01:59	Early Morning	37,3	48	8,8	8,8	18,46	536,04	72,43
2019/05/26	CH2205	Male	Three y/o	Treatment (Wk 2)	Treatment	Fast Day	No	01:40	01:00 - 01:59	Early Morning	37,3		10,0	10,0	18,46		76,76
2019/05/26	CH2206	Male	Three y/o	Treatment (Wk 2)	Treatment	Fast Day	No	01:40	01:00 - 01:59	Early Morning	37,3	54	7,2	7,2	18,46	545,99	65,66
2019/05/26	CH2205	Male	Three y/o	Treatment (Wk 2)	Treatment	Fast Day	No	01:45	01:00 - 01:59	Early Morning	37,3	32	9,8	9,8	18,46	499,63	76,07
2019/05/26	CH2206	Male	Three y/o	Treatment (Wk 2)	Treatment	Fast Day	No	01:45	01:00 - 01:59	Early Morning	37,3	44	4,8	4,8	18,46	528,51	52,04
2019/05/26	CH2205	Male	Three y/o	Treatment (Wk 2)	Treatment	Fast Day	No	01:50	01:00 - 01:59	Early Morning	37,3	65	4,0	4,0	18,46	561,12	45,94
2019/05/26	CH2206	Male	Three y/o	Treatment (Wk 2)	Treatment	Fast Day	No	01:50	01:00 - 01:59	Early Morning	37,3	46	8,6	8,6	18,46	532,38	71,66
2019/05/26	CH2205	Male	Three y/o	Treatment (Wk 2)	Treatment	Fast Day	No	01:55	01:00 - 01:59	Early Morning	37,3	51	6,0	6,0	18,46	541,20	59,53
2019/05/26	CH2206	Male	Three y/o	Treatment (Wk 2)	Treatment	Fast Day	No	01:55	01:00 - 01:59	Early Morning	37,3	33	23,8	23,8	18,46	502,51	106,26
2019/05/26	CH2205	Male	Three y/o	Treatment (Wk 2)	Treatment	Fast Day	No	02:00	02:00 - 02:59	Early Morning	37,3		12,4	12,4	18,46		84,04
2019/05/26	CH2206	Male	Three y/o	Treatment (Wk 2)	Treatment	Fast Day	No	02:00	02:00 - 02:59	Early Morning	37,3	53	24,2	24,2	18,46	544,43	106,83
2019/05/26	CH2205	Male	Three y/o	Treatment (Wk 2)	Treatment	Fast Day	No	02:05	02:00 - 02:59	Early Morning	37,3		7,6	7,6	18,46		67,49
2019/05/26	CH2206	Male	Three y/o	Treatment (Wk 2)	Treatment	Fast Day	No	02:05	02:00 - 02:59	Early Morning	37,3	41	25,8	25,8	18,46	522,29	109,02
2019/05/26	CH2205	Male	Three y/o	Treatment (Wk 2)	Treatment	Fast Day	No	02:10	02:00 - 02:59	Early Morning	37,3		7,6	7,6	18,46		67,49
2019/05/26	CH2206	Male	Three y/o	Treatment (Wk 2)	Treatment	Fast Day	No	02:10	02:00 - 02:59	Early Morning	37,3	39	27,6	27,6	18,46	517,82	111,33
2019/05/26	CH2205	Male	Three y/o	Treatment (Wk 2)	Treatment	Fast Day	No	02:15	02:00 - 02:59	Early Morning	37,3	58	8,4	8,4	18,46	551,90	70,86
2019/05/26	CH2206	Male	Three y/o	Treatment (Wk 2)	Treatment	Fast Day	No	02:15	02:00 - 02:59	Early Morning	37,3	76	39,8	39,8	18,46	573,39	123,91
2019/05/26	CH2205	Male	Three y/o	Treatment (Wk 2)	Treatment	Fast Day	No	02:20	02:00 - 02:59	Early Morning	37,3	62	10,8	10,8	18,46	557,33	79,36
2019/05/26	CH2206	Male	Three y/o	Treatment (Wk 2)	Treatment	Fast Day	No	02:20	02:00 - 02:59	Early Morning	37,3	49	51,0	51,0	18,46	537,80	132,47
2019/05/26	CH2205	Male	Three y/o	Treatment (Wk 2)	Treatment	Fast Day	No	02:25	02:00 - 02:59	Early Morning	37,3	53	62,0	62,0	18,46	544,43	139,23
2019/05/26	CH2206	Male	Three y/o	Treatment (Wk 2)	Treatment	Fast Day	No	02:25	02:00 - 02:59	Early Morning	37,4	79	59,2	59,2	18,55	576,36	137,63
2019/05/26	CH2205	Male	Three y/o	Treatment (Wk 2)	Treatment	Fast Day	No	02:30	02:00 - 02:59	Early Morning	37,4	117	92,8	92,8	18,55	605,02	153,24
2019/05/26	CH2206	Male	Three y/o	Treatment (Wk 2)	Treatment	Fast Day	No	02:30	02:00 - 02:59	Early Morning	37,3	128	68,8	68,8	18,46	611,22	142,84
2019/05/26	CH2205	Male	Three y/o	Treatment (Wk 2)	Treatment	Fast Day	No	02:35	02:00 - 02:59	Early Morning	37,3	75	164,8	164,8	18,46	572,36	173,32
2019/05/26	CH2206	Male	Three y/o	Treatment (Wk 2)	Treatment	Fast Day	No	02:35	02:00 - 02:59	Early Morning	37,3	105	92,8	92,8	18,46	597,38	153,24
2019/05/26	CH2205	Male	Three y/o	Treatment (Wk 2)	Treatment	Fast Day	No	02:40	02:00 - 02:59	Early Morning	37,3	120	79,2	79,2	18,46	606,78	147,73
2019/05/26	CH2206	Male	Three y/o	Treatment (Wk 2)	Treatment	Fast Day	No	02:40	02:00 - 02:59	Early Morning	37,4	148	253,2	253,2	18,55	620,97	188,43
2019/05/26	CH2205	Male	Three y/o	Treatment (Wk 2)	Treatment	Fast Day	No	02:45	02:00 - 02:59	Early Morning	37,4		178,8	178,8	18,55		176,18
2019/05/26	CH2206	Male	Three y/o	Treatment (Wk 2)	Treatment	Fast Day	No	02:45	02:00 - 02:59	Early Morning	37,4	110	152,2	152,2	18,55	600,69	170,53
2019/05/26	CH2205	Male	Three y/o	Treatment (Wk 2)	Treatment	Fast Day	No	02:50	02:00 - 02:59	Early Morning	37,4		210,0	210,0	18,55		181,84
2019/05/26	CH2206	Male	Three y/o	Treatment (Wk 2)	Treatment	Fast Day	No	02:50	02:00 - 02:59	Early Morning	37,5	160	56,2	56,2	18,65	626,07	135,83
2019/05/26	CH2205	Male	Three y/o	Treatment (Wk 2)	Treatment	Fast Day	No	02:55	02:00 - 02:59	Early Morning	37,5	86	28,2	28,2	18,65	582,77	112,07
2019/05/26	CH2206	Male	Three y/o	Treatment (Wk 2)	Treatment	Fast Day	No	02:55	02:00 - 02:59	Early Morning	37,4	96	54,2	54,2	18,55	590,91	134,57
2019/05/26	CH2205	Male	Three y/o	Treatment (Wk 2)	Treatment	Fast Day	No	03:00	03:00 - 03:59	Early Morning	37,4		67,6	67,6	18,55		142,23
2019/05/26	CH2206	Male	Three y/o	Treatment (Wk 2)	Treatment	Fast Day	No	03:00	03:00 - 03:59	Early Morning	37,4	69	67,8	67,8	18,55	565,86	142,33
2019/05/26	CH2205	Male	Three y/o	Treatment (Wk 2)	Treatment	Fast Day	No	03:05	03:00 - 03:59	Early Morning	37,6	95	25,6	25,6	18,75	590,14	108,76
2019/05/26	CH2206	Male	Three y/o	Treatment (Wk 2)	Treatment	Fast Day	No	03:05	03:00 - 03:59	Early Morning	37,2	71	61,6	61,6	18,36	568,10	139,00
2019/05/26	CH2205	Male	Three y/o	Treatment (Wk 2)	Treatment	Fast Day	No	03:10	03:00 - 03:59	Early Morning	37,2	143	26,8	26,8	18,36	618,69	110,33
2019/05/26	CH2206	Male	Three y/o	Treatment (Wk 2)	Treatment	Fast Day	No	03:10	03:00 - 03:59	Early Morning	37,1	60	52,6	52,6	18,26	554,67	133,54
2019/05/26	CH2205	Male	Three y/o	Treatment (Wk 2)	Treatment	Fast Day	No	03:15	03:00 - 03:59	Early Morning	37,1	42	27,6	27,6	18,26	524,42	111,33
2019/05/26	CH2206	Male	Three y/o	Treatment (Wk 2)	Treatment	Fast Day	No	03:15	03:00 - 03:59	Early Morning	37,1	68	41,0	41,0	18,26	564,71	124,94
2019/05/26	CH2205	Male	Three y/o	Treatment (Wk 2)	Treatment	Fast Day	No	03:20	03:00 - 03:59	Early Morning	37,2		6,0	6,0	18,36		59,53
2019/05/26	CH2206	Male	Three y/o	Treatment (Wk 2)	Treatment	Fast Day	No	03:20	03:00 - 03:59	Early Morning	37,1	70	52,2	52,2	18,26	566,99	133,27
2019/05/26	CH2205	Male	Three y/o	Treatment (Wk 2)	Treatment	Fast Day	No	03:25	03:00 - 03:59	Early Morning	37,1	50	8,4	8,4	18,26	539,52	70,86
2019/05/26	CH2206	Male	Three y/o	Treatment (Wk 2)	Treatment	Fast Day	No	03:25	03:00 - 03:59	Early Morning	37,0	46	54,6	54,6	18,16	532,38	134,83
2019/05/26	CH2205	Male	Three y/o	Treatment (Wk 2)	Treatment	Fast Day	No	03:30	03:00 - 03:59	Early Morning	37,1	43	7,4	7,4	18,26	526,50	66,59
2019/05/26	CH2206	Male	Three y/o	Treatment (Wk 2)	Treatment	Fast Day	No	03:30	03:00 - 03:59	Early Morning	37,0	51	56,8	56,8	18,16	541,20	136,20
2019/05/26	CH2205	Male	Three y/o	Treatment (Wk 2)	Treatment	Fast Day	No	03:35	03:00 - 03:59	Early Morning	37,1	60	7,0	7,0	18,26	554,67	64,72
2019/05/26	CH2206	Male	Three y/o	Treatment (Wk 2)	Treatment	Fast Day	No	03:35	03:00 - 03:59	Early Morning	37,0	77	41,8	41,8	18,16	574,39	125,60
2019/05/26	CH2205	Male	Three y/o	Treatment (Wk 2)	Treatment	Fast Day	No	03:40	03:00 - 03:59	Early Morning	37,2	64	4,4	4,4	18,36	559,88	49,12
2019/05/26	CH2206	Male	Three y/o	Treatment (Wk 2)	Treatment	Fast Day	No	03:40	03:00 - 03:59	Early Morning	37,0	43	43,4	43,4	18,16	526,50	126,90
2019/05/26	CH2205	Male	Three y/o	Treatment (Wk 2)	Treatment	Fast Day	No	03:45	03:00 - 03:59	Early Morning	37,2	50	3,6	3,6	18,36	539,52	42,42
2019/05/26	CH2206	Male	Three y/o	Treatment (Wk 2)	Treatment	Fast Day	No	03:45	03:00 - 03:59	Early Morning	37,0	42	47,0	47,0	18,16	524,42	129,65
2019/05/26	CH2205	Male	Three y/o	Treatment (Wk 2)	Treatment	Fast Day	No	03:50	03:00 - 03:59	Early Morning	37,2	45	1,2	1,2	18,36	530,47	5,99
2019/05/26	CH2206	Male	Three y/o	Treatment (Wk 2)	Treatment	Fast Day	No	03:50	03:00 - 03:59	Early Morning	37,0	57	43,0	43,0	18,16	550,47	126,58
2019/05/26	CH2205	Male	Three y/o	Treatment (Wk 2)	Treatment	Fast Day	No	03:55	03:00 - 03:59	Early Morning	37,2	43	3,0	3,0	18,36	526,50	36,34
2019/05/26	CH2206	Male	Three y/o	Treatment (Wk 2)	Treatment	Fast Day	No	03:55	03:00 - 03:59	Early Morning	37,1	47	43,2	43,2	18,26	534,23	126,74
2019/05/26	CH2205	Male	Three y/o	Treatment (Wk 2)	Treatment	Fast Day	No	04:00	04:00 - 04:59	Morning	37,2	42	3,8	3,8	18,36	524,42	44,22
2019/05/26	CH2206	Male	Three y/o	Treatment (Wk 2)	Treatment	Fast Day	No	04:00	04:00 - 04:59	Morning	37,1	49	38,0	38,0	18,26	537,80	122,32
2019/05/26	CH2205	Male	Three y/o	Treatment (Wk 2)	Treatment	Fast Day	No	04:05	04:00 - 04:59	Morning	37,2	47	3,4	3,4	18,36	534,23	40,51
2019/05/26	CH2206	Male	Three y/o	Treatment (Wk 2)	Treatment	Fast Day	No	04:05	04:00 - 04:59	Morning	37,1	49	11,6	11,6	18,26	537,80	81,78
2019/05/26	CH2205	Male	Three y/o	Treatment (Wk 2)	Treatment	Fast Day	No	04:10	04:00 - 04:59	Morning	37,2	46	3,8	3,8	18,36	532,38	44,22

2019/05/26	CH2206	Male	Three y/o	Treatment (Wk 2)	Treatment	Fast Day	No	04:10	04:00 - 04:59	Morning	37,1	47	7,6	7,6	18,26	534,23	67,49
2019/05/26	CH2205	Male	Three y/o	Treatment (Wk 2)	Treatment	Fast Day	No	04:15	04:00 - 04:59	Morning	37,2	72	1,2	1,2	18,36	569,20	5,99
2019/05/26	CH2206	Male	Three y/o	Treatment (Wk 2)	Treatment	Fast Day	No	04:15	04:00 - 04:59	Morning	37,1	51	7,8	7,8	18,26	541,20	68,36
2019/05/26	CH2205	Male	Three y/o	Treatment (Wk 2)	Treatment	Fast Day	No	04:20	04:00 - 04:59	Morning	37,2	45	4,2	4,2	18,36	530,47	47,57
2019/05/26	CH2206	Male	Three y/o	Treatment (Wk 2)	Treatment	Fast Day	No	04:20	04:00 - 04:59	Morning	37,1	75	7,6	7,6	18,26	572,36	67,49
2019/05/26	CH2205	Male	Three y/o	Treatment (Wk 2)	Treatment	Fast Day	No	04:25	04:00 - 04:59	Morning	37,2	45	4,0	4,0	18,36	530,47	45,94
2019/05/26	CH2206	Male	Three y/o	Treatment (Wk 2)	Treatment	Fast Day	No	04:25	04:00 - 04:59	Morning	37,1	77	1,6	1,6	18,26	574,39	15,48
2019/05/26	CH2205	Male	Three y/o	Treatment (Wk 2)	Treatment	Fast Day	No	04:30	04:00 - 04:59	Morning	37,2	35	2,4	2,4	18,36	507,97	28,91
2019/05/26	CH2206	Male	Three y/o	Treatment (Wk 2)	Treatment	Fast Day	No	04:30	04:00 - 04:59	Morning	37,1	45	21,0	21,0	18,26	530,47	101,98
2019/05/26	CH2205	Male	Three y/o	Treatment (Wk 2)	Treatment	Fast Day	No	04:35	04:00 - 04:59	Morning	37,2	42	4,2	4,2	18,36	524,42	47,57
2019/05/26	CH2206	Male	Three y/o	Treatment (Wk 2)	Treatment	Fast Day	No	04:35	04:00 - 04:59	Morning	37,1	68	16,2	16,2	18,26	564,71	93,13
2019/05/26	CH2205	Male	Three y/o	Treatment (Wk 2)	Treatment	Fast Day	No	04:40	04:00 - 04:59	Morning	37,2	37	3,8	3,8	18,36	513,06	44,22
2019/05/26	CH2206	Male	Three y/o	Treatment (Wk 2)	Treatment	Fast Day	No	04:40	04:00 - 04:59	Morning	37,1	42	17,6	17,6	18,26	524,42	95,95
2019/05/26	CH2205	Male	Three y/o	Treatment (Wk 2)	Treatment	Fast Day	No	04:45	04:00 - 04:59	Morning	37,2	42	8,6	8,6	18,36	524,42	71,66
2019/05/26	CH2206	Male	Three y/o	Treatment (Wk 2)	Treatment	Fast Day	No	04:45	04:00 - 04:59	Morning	37,1	43	45,0	45,0	18,26	526,50	128,15
2019/05/26	CH2205	Male	Three y/o	Treatment (Wk 2)	Treatment	Fast Day	No	04:50	04:00 - 04:59	Morning	37,2	45	12,4	12,4	18,36	530,47	84,04
2019/05/26	CH2206	Male	Three y/o	Treatment (Wk 2)	Treatment	Fast Day	No	04:50	04:00 - 04:59	Morning	37,2	73	63,6	63,6	18,36	570,27	140,11
2019/05/26	CH2205	Male	Three y/o	Treatment (Wk 2)	Treatment	Fast Day	No	04:55	04:00 - 04:59	Morning	37,3	43	10,2	10,2	18,46	526,50	77,43
2019/05/26	CH2206	Male	Three y/o	Treatment (Wk 2)	Treatment	Fast Day	No	04:55	04:00 - 04:59	Morning	37,2	48	63,6	63,6	18,36	536,04	140,11
2019/05/26	CH2205	Male	Three y/o	Treatment (Wk 2)	Treatment	Fast Day	No	05:00	05:00 - 05:59	Morning	37,3	57	10,0	10,0	18,46	550,47	76,76
2019/05/26	CH2206	Male	Three y/o	Treatment (Wk 2)	Treatment	Fast Day	No	05:00	05:00 - 05:59	Morning	37,2	48	70,0	70,0	18,36	536,04	143,44
2019/05/26	CH2205	Male	Three y/o	Treatment (Wk 2)	Treatment	Fast Day	No	05:05	05:00 - 05:59	Morning	37,2	41	3,8	3,8	18,36	522,29	44,22
2019/05/26	CH2206	Male	Three y/o	Treatment (Wk 2)	Treatment	Fast Day	No	05:05	05:00 - 05:59	Morning	37,2	47	71,4	71,4	18,36	534,23	144,12
2019/05/26	CH2205	Male	Three y/o	Treatment (Wk 2)	Treatment	Fast Day	No	05:10	05:00 - 05:59	Morning	37,2	50	7,2	7,2	18,36	539,52	65,66
2019/05/26	CH2206	Male	Three y/o	Treatment (Wk 2)	Treatment	Fast Day	No	05:10	05:00 - 05:59	Morning	37,2	43	64,4	64,4	18,36	526,50	140,55
2019/05/26	CH2205	Male	Three y/o	Treatment (Wk 2)	Treatment	Fast Day	No	05:15	05:00 - 05:59	Morning	37,1	37	8,6	8,6	18,26	513,06	71,66
2019/05/26	CH2206	Male	Three y/o	Treatment (Wk 2)	Treatment	Fast Day	No	05:15	05:00 - 05:59	Morning	37,2	92	56,2	56,2	18,36	587,78	135,83
2019/05/26	CH2205	Male	Three y/o	Treatment (Wk 2)	Treatment	Fast Day	No	05:20	05:00 - 05:59	Morning	37,1	71	15,2	15,2	18,26	568,10	90,96
2019/05/26	CH2206	Male	Three y/o	Treatment (Wk 2)	Treatment	Fast Day	No	05:20	05:00 - 05:59	Morning	37,2		51,0	51,0	18,36		132,47
2019/05/26	CH2205	Male	Three y/o	Treatment (Wk 2)	Treatment	Fast Day	No	05:25	05:00 - 05:59	Morning	37,2	73	28,0	28,0	18,36	570,27	111,83
2019/05/26	CH2206	Male	Three y/o	Treatment (Wk 2)	Treatment	Fast Day	No	05:25	05:00 - 05:59	Morning	37,1	77	60,2	60,2	18,26	574,39	138,21
2019/05/26	CH2205	Male	Three y/o	Treatment (Wk 2)	Treatment	Fast Day	No	05:30	05:00 - 05:59	Morning	37,2	67	23,4	23,4	18,36	563,53	105,68
2019/05/26	CH2206	Male	Three y/o	Treatment (Wk 2)	Treatment	Fast Day	No	05:30	05:00 - 05:59	Morning	37,1	70	49,6	49,6	18,26	566,99	131,51
2019/05/26	CH2205	Male	Three y/o	Treatment (Wk 2)	Treatment	Fast Day	No	05:35	05:00 - 05:59	Morning	37,2		58,8	58,8	18,36		137,39
2019/05/26	CH2206	Male	Three y/o	Treatment (Wk 2)	Treatment	Fast Day	No	05:35	05:00 - 05:59	Morning	37,2	120	138,0	138,0	18,36	606,78	167,10
2019/05/26	CH2205	Male	Three y/o	Treatment (Wk 2)	Treatment	Fast Day	No	05:40	05:00 - 05:59	Morning	37,2	107	63,8	63,8	18,36	598,73	140,22
2019/05/26	CH2206	Male	Three y/o	Treatment (Wk 2)	Treatment	Fast Day	No	05:40	05:00 - 05:59	Morning	37,2	134	135,8	135,8	18,36	614,33	166,53
2019/05/26	CH2205	Male	Three y/o	Treatment (Wk 2)	Treatment	Fast Day	No	05:45	05:00 - 05:59	Morning	37,2	128	295,0	295,0	18,36	611,22	193,83
2019/05/26	CH2206	Male	Three y/o	Treatment (Wk 2)	Treatment	Fast Day	No	05:45	05:00 - 05:59	Morning	37,3	119	508,2	508,2	18,46	606,20	213,13
2019/05/26	CH2205	Male	Three y/o	Treatment (Wk 2)	Treatment	Fast Day	No	05:50	05:00 - 05:59	Morning	37,2		105,8	105,8	18,36		157,81
2019/05/26	CH2206	Male	Three y/o	Treatment (Wk 2)	Treatment	Fast Day	No	05:50	05:00 - 05:59	Morning	37,4	174	457,2	457,2	18,55	631,46	209,36
2019/05/26	CH2205	Male	Three y/o	Treatment (Wk 2)	Treatment	Fast Day	No	05:55	05:00 - 05:59	Morning	37,3	101	48,0	48,0	18,46	594,59	130,38
2019/05/26	CH2206	Male	Three y/o	Treatment (Wk 2)	Treatment	Fast Day	No	05:55	05:00 - 05:59	Morning	37,7	113	91,0	91,0	18,85	602,58	152,56
2019/05/26	CH2205	Male	Three y/o	Treatment (Wk 2)	Treatment	Fast Day	No	06:00	06:00 - 06:59	Morning	37,4		186,8	186,8	18,55		177,72
2019/05/26	CH2206	Male	Three y/o	Treatment (Wk 2)	Treatment	Fast Day	No	06:00	06:00 - 06:59	Morning	37,8	131	231,2	231,2	18,95	612,80	185,22
2019/05/26	CH2205	Male	Three y/o	Treatment (Wk 2)	Treatment	Fast Day	No	06:05	06:00 - 06:59	Morning	37,7		21,2	21,2	18,85		102,31
2019/05/26	CH2206	Male	Three y/o	Treatment (Wk 2)	Treatment	Fast Day	No	06:05	06:00 - 06:59	Morning	37,8		46,2	46,2	18,95		129,06
2019/05/26	CH2205	Male	Three y/o	Treatment (Wk 2)	Treatment	Fast Day	No	06:10	06:00 - 06:59	Morning	37,4		34,8	34,8	18,55		119,29
2019/05/26	CH2206	Male	Three y/o	Treatment (Wk 2)	Treatment	Fast Day	No	06:10	06:00 - 06:59	Morning	37,5	53	56,2	56,2	18,65	544,43	135,83
2019/05/26	CH2205	Male	Three y/o	Treatment (Wk 2)	Treatment	Fast Day	No	06:15	06:00 - 06:59	Morning	37,4		31,6	31,6	18,55		115,98
2019/05/26	CH2206	Male	Three y/o	Treatment (Wk 2)	Treatment	Fast Day	No	06:15	06:00 - 06:59	Morning	37,3	123	32,8	32,8	18,46	608,49	117,26
2019/05/26	CH2205	Male	Three y/o	Treatment (Wk 2)	Treatment	Fast Day	No	06:20	06:00 - 06:59	Morning	37,3	102	29,6	29,6	18,46	595,30	113,73
2019/05/26	CH2206	Male	Three y/o	Treatment (Wk 2)	Treatment	Fast Day	No	06:20	06:00 - 06:59	Morning	37,5	118	158,2	158,2	18,65	605,61	171,88
2019/05/26	CH2205	Male	Three y/o	Treatment (Wk 2)	Treatment	Fast Day	No	06:25	06:00 - 06:59	Morning	37,2	90	37,0	37,0	18,36	586,16	121,40
2019/05/26	CH2206	Male	Three y/o	Treatment (Wk 2)	Treatment	Fast Day	No	06:25	06:00 - 06:59	Morning	37,7	128	77,2	77,2	18,85	611,22	146,84
2019/05/26	CH2205	Male	Three y/o	Treatment (Wk 2)	Treatment	Fast Day	No	06:30	06:00 - 06:59	Morning	37,4	169	26,2	26,2	18,55	629,60	109,55
2019/05/26	CH2206	Male	Three y/o	Treatment (Wk 2)	Treatment	Fast Day	No	06:30	06:00 - 06:59	Morning	37,7	112	167,8	167,8	18,85	601,96	173,95
2019/05/26	CH2205	Male	Three y/o	Treatment (Wk 2)	Treatment	Fast Day	No	06:35	06:00 - 06:59	Morning	37,3	75	47,4	47,4	18,46	572,36	129,94
2019/05/26	CH2206	Male	Three y/o	Treatment (Wk 2)	Treatment	Fast Day	No	06:35	06:00 - 06:59	Morning	37,8	121	41,4	41,4	18,95	607,35	125,27
2019/05/26	CH2205	Male	Three y/o	Treatment (Wk 2)	Treatment	Fast Day	No	06:40	06:00 - 06:59	Morning	37,2	84	63,4	63,4	18,36	581,01	140,00
2019/05/26	CH2206	Male	Three y/o	Treatment (Wk 2)	Treatment	Fast Day	No	06:40	06:00 - 06:59	Morning	37,8	117	81,4	81,4	18,95	605,02	148,68
2019/05/26	CH2205	Male	Three y/o	Treatment (Wk 2)	Treatment	Fast Day	No	06:45	06:00 - 06:59	Morning	37,3	90	1064,0		18,46	586,16	
2019/05/26	CH2206	Male	Three y/o	Treatment (Wk 2)	Treatment	Fast Day	No	06:45	06:00 - 06:59	Morning	37,8	131	107,2	107,2	18,95	612,80	158,27
2019/05/26	CH2205	Male	Three y/o	Treatment (Wk 2)	Treatment	Fast Day	No	06:50	06:00 - 06:59	Morning	37,5	121	279,0	279,0	18,65	607,35	191,86
2019/05/26	CH2206	Male	Three y/o	Treatment (Wk 2)	Treatment	Fast Day	No	06:50	06:00 - 06:59	Morning	37,8	123	226,2	226,2	18,95	608,49	184,45
2019/05/26	CH2205	Male	Three y/o	Treatment (Wk 2)	Treatment	Fast Day	No	06:55	06:00 - 06:59	Morning	37,5	120	112,2	112,2	18,65	606,78	159,86
2019/05/26	CH2206	Male	Three y/o	Treatment (Wk 2)	Treatment	Fast Day	No	06:55	06:00 - 06:59	Morning	37,8	95	341,8	341,8	18,95	590,14	199,04
2019/05/26	CH2205	Male	Three y/o	Treatment (Wk 2)	Treatment	Fast Day	No	07:00	07:00 - 07:59	Morning	37,5	99	78,8	78,8	18,65	593,14	147,55
2019/05/26	CH2206	Male	Three y/o	Treatment (Wk 2)	Treatment	Fast Day	No	07:00	07:00 - 07:59	Morning	37,9	103	112,8	112,8	19,05	596,00	160,05
2019/05/26	CH2205	Male	Three y/o	Treatment (Wk 2)	Treatment	Fast Day	No	07:05	07:00 - 07:59	Morning	37,5	113	153,2	153,2	18,65	602,58	170,76

2019/05/26	CH2206	Male	Three y/o	Treatment (Wk 2)	Treatment	Fast Day	No	07:05	07:00 - 07:59	Morning	37,9	108	157,0	157,0	19,05	599,39	171,62
2019/05/26	CH2205	Male	Three y/o	Treatment (Wk 2)	Treatment	Fast Day	No	07:10	07:00 - 07:59	Morning	37,6	119	154,8	154,8	18,75	606,20	171,12
2019/05/26	CH2206	Male	Three y/o	Treatment (Wk 2)	Treatment	Fast Day	No	07:10	07:00 - 07:59	Morning	37,9	112	38,2	38,2	19,05	601,96	122,50
2019/05/26	CH2205	Male	Three y/o	Treatment (Wk 2)	Treatment	Fast Day	No	07:15	07:00 - 07:59	Morning	37,6	99	44,4	44,4	18,75	593,14	127,69
2019/05/26	CH2206	Male	Three y/o	Treatment (Wk 2)	Treatment	Fast Day	No	07:15	07:00 - 07:59	Morning	37,9	60	81,6	81,6	19,05	554,67	148,76
2019/05/26	CH2205	Male	Three y/o	Treatment (Wk 2)	Treatment	Fast Day	No	07:20	07:00 - 07:59	Morning	37,6		20,8	20,8	18,75		101,65
2019/05/26	CH2206	Male	Three y/o	Treatment (Wk 2)	Treatment	Fast Day	No	07:20	07:00 - 07:59	Morning	37,9	129	184,6	184,6	19,05	611,75	177,30
2019/05/26	CH2205	Male	Three y/o	Treatment (Wk 2)	Treatment	Fast Day	No	07:25	07:00 - 07:59	Morning	37,5	68	66,2	66,2	18,65	564,71	141,50
2019/05/26	CH2206	Male	Three y/o	Treatment (Wk 2)	Treatment	Fast Day	No	07:25	07:00 - 07:59	Morning	37,9	107	102,6	102,6	19,05	598,73	156,74
2019/05/26	CH2205	Male	Three y/o	Treatment (Wk 2)	Treatment	Fast Day	No	07:30	07:00 - 07:59	Morning	37,6		121,6	121,6	18,75		162,67
2019/05/26	CH2206	Male	Three y/o	Treatment (Wk 2)	Treatment	Fast Day	No	07:30	07:00 - 07:59	Morning	37,9	104	84,2	84,2	19,05	596,69	149,85
2019/05/26	CH2205	Male	Three y/o	Treatment (Wk 2)	Treatment	Fast Day	No	07:35	07:00 - 07:59	Morning	37,6	123	130,8	130,8	18,75	608,49	165,22
2019/05/26	CH2206	Male	Three y/o	Treatment (Wk 2)	Treatment	Fast Day	No	07:35	07:00 - 07:59	Morning	37,8	115	66,8	66,8	18,95	603,82	141,81
2019/05/26	CH2205	Male	Three y/o	Treatment (Wk 2)	Treatment	Fast Day	No	07:40	07:00 - 07:59	Morning	37,7	73	31,0	31,0	18,85	570,27	115,32
2019/05/26	CH2206	Male	Three y/o	Treatment (Wk 2)	Treatment	Fast Day	No	07:40	07:00 - 07:59	Morning	37,9	97	123,0	123,0	19,05	591,66	163,07
2019/05/26	CH2205	Male	Three y/o	Treatment (Wk 2)	Treatment	Fast Day	No	07:45	07:00 - 07:59	Morning	37,7	106	214,6	214,6	18,85	598,06	182,60
2019/05/26	CH2206	Male	Three y/o	Treatment (Wk 2)	Treatment	Fast Day	No	07:45	07:00 - 07:59	Morning	37,9	129	110,0	110,0	19,05	611,75	159,17
2019/05/26	CH2205	Male	Three y/o	Treatment (Wk 2)	Treatment	Fast Day	No	07:50	07:00 - 07:59	Morning	37,7		91,2	91,2	18,85		152,63
2019/05/26	CH2206	Male	Three y/o	Treatment (Wk 2)	Treatment	Fast Day	No	07:50	07:00 - 07:59	Morning	37,9		160,0	160,0	19,05		172,28
2019/05/26	CH2205	Male	Three y/o	Treatment (Wk 2)	Treatment	Fast Day	No	07:55	07:00 - 07:59	Morning	37,7	106	34,0	34,0	18,85	598,06	118,49
2019/05/26	CH2206	Male	Three y/o	Treatment (Wk 2)	Treatment	Fast Day	No	07:55	07:00 - 07:59	Morning	37,9		135,6	135,6	19,05		166,48
2019/05/26	CH2205	Male	Three y/o	Treatment (Wk 2)	Treatment	Fast Day	No	08:00	08:00 - 08:59	Late Morning	37,6	49	23,4	23,4	18,75	537,80	105,68
2019/05/26	CH2206	Male	Three y/o	Treatment (Wk 2)	Treatment	Fast Day	No	08:00	08:00 - 08:59	Late Morning	37,9	128	152,6	152,6	19,05	611,22	170,62
2019/05/26	CH2205	Male	Three y/o	Treatment (Wk 2)	Treatment	Fast Day	No	08:05	08:00 - 08:59	Late Morning	37,5		51,6	51,6	18,65		132,87
2019/05/26	CH2206	Male	Three y/o	Treatment (Wk 2)	Treatment	Fast Day	No	08:05	08:00 - 08:59	Late Morning	37,9	118	216,8	216,8	19,05	605,61	182,96
2019/05/26	CH2205	Male	Three y/o	Treatment (Wk 2)	Treatment	Fast Day	No	08:10	08:00 - 08:59	Late Morning	37,5	97	157,2	157,2	18,65	591,66	171,66
2019/05/26	CH2206	Male	Three y/o	Treatment (Wk 2)	Treatment	Fast Day	No	08:10	08:00 - 08:59	Late Morning	37,9	112	68,2	68,2	19,05	601,96	142,53
2019/05/26	CH2205	Male	Three y/o	Treatment (Wk 2)	Treatment	Fast Day	No	08:15	08:00 - 08:59	Late Morning	37,5	114	159,6	159,6	18,65	603,20	172,19
2019/05/26	CH2206	Male	Three y/o	Treatment (Wk 2)	Treatment	Fast Day	No	08:15	08:00 - 08:59	Late Morning	37,9	105	121,2	121,2	19,05	597,38	162,56
2019/05/26	CH2205	Male	Three y/o	Treatment (Wk 2)	Treatment	Fast Day	No	08:20	08:00 - 08:59	Late Morning	37,5	44	200,2	200,2	18,65	528,51	180,15
2019/05/26	CH2206	Male	Three y/o	Treatment (Wk 2)	Treatment	Fast Day	No	08:20	08:00 - 08:59	Late Morning	37,8	165	204,2	204,2	18,95	628,06	180,85
2019/05/26	CH2205	Male	Three y/o	Treatment (Wk 2)	Treatment	Fast Day	No	08:25	08:00 - 08:59	Late Morning	37,5	112	76,2	76,2	18,65	601,96	146,38
2019/05/26	CH2206	Male	Three y/o	Treatment (Wk 2)	Treatment	Fast Day	No	08:25	08:00 - 08:59	Late Morning	37,8	56	166,8	166,8	18,95	549,01	173,74
2019/05/26	CH2205	Male	Three y/o	Treatment (Wk 2)	Treatment	Fast Day	No	08:30	08:00 - 08:59	Late Morning	37,6	86	66,0	66,0	18,75	582,77	141,40
2019/05/26	CH2206	Male	Three y/o	Treatment (Wk 2)	Treatment	Fast Day	No	08:30	08:00 - 08:59	Late Morning	37,8		107,4	107,4	18,95		158,33
2019/05/26	CH2205	Male	Three y/o	Treatment (Wk 2)	Treatment	Fast Day	No	08:35	08:00 - 08:59	Late Morning	37,6	105	84,8	84,8	18,75	597,38	150,10
2019/05/26	CH2206	Male	Three y/o	Treatment (Wk 2)	Treatment	Fast Day	No	08:35	08:00 - 08:59	Late Morning	37,9	93	171,6	171,6	19,05	588,58	174,74
2019/05/26	CH2205	Male	Three y/o	Treatment (Wk 2)	Treatment	Fast Day	No	08:40	08:00 - 08:59	Late Morning	37,5	76	28,6	28,6	18,65	573,39	112,56
2019/05/26	CH2206	Male	Three y/o	Treatment (Wk 2)	Treatment	Fast Day	No	08:40	08:00 - 08:59	Late Morning	37,9	162	70,6	70,6	19,05	626,88	143,73
2019/05/26	CH2205	Male	Three y/o	Treatment (Wk 2)	Treatment	Fast Day	No	08:45	08:00 - 08:59	Late Morning	37,5		26,6	26,6	18,65		110,07
2019/05/26	CH2206	Male	Three y/o	Treatment (Wk 2)	Treatment	Fast Day	No	08:45	08:00 - 08:59	Late Morning	37,8	120	146,6	146,6	18,95	606,78	169,21
2019/05/26	CH2205	Male	Three y/o	Treatment (Wk 2)	Treatment	Fast Day	No	08:50	08:00 - 08:59	Late Morning	37,4		28,8	28,8	18,55		112,79
2019/05/26	CH2206	Male	Three y/o	Treatment (Wk 2)	Treatment	Fast Day	No	08:50	08:00 - 08:59	Late Morning	37,8	96	104,0	104,0	18,95	590,91	157,21
2019/05/26	CH2205	Male	Three y/o	Treatment (Wk 2)	Treatment	Fast Day	No	08:55	08:00 - 08:59	Late Morning	37,3		285,8	285,8	18,46		192,71
2019/05/26	CH2206	Male	Three y/o	Treatment (Wk 2)	Treatment	Fast Day	No	08:55	08:00 - 08:59	Late Morning	37,8	102	69,2	69,2	18,95	595,30	143,04
2019/05/26	CH2205	Male	Three y/o	Treatment (Wk 2)	Treatment	Fast Day	No	09:00	09:00 - 09:59	Late Morning	37,4		35,2	35,2	18,55		119,69
2019/05/26	CH2206	Male	Three y/o	Treatment (Wk 2)	Treatment	Fast Day	No	09:00	09:00 - 09:59	Late Morning	37,9	105	75,2	75,2	19,05	597,38	145,93
2019/05/26	CH2205	Male	Three y/o	Treatment (Wk 2)	Treatment	Fast Day	No	09:05	09:00 - 09:59	Late Morning	37,6	154	32,6	32,6	18,75	623,58	117,05
2019/05/26	CH2206	Male	Three y/o	Treatment (Wk 2)	Treatment	Fast Day	No	09:05	09:00 - 09:59	Late Morning	37,9	111	127,0	127,0	19,05	601,33	164,19
2019/05/26	CH2205	Male	Three y/o	Treatment (Wk 2)	Treatment	Fast Day	No	09:10	09:00 - 09:59	Late Morning	37,6	88	58,4	58,4	18,75	584,49	137,16
2019/05/26	CH2206	Male	Three y/o	Treatment (Wk 2)	Treatment	Fast Day	No	09:10	09:00 - 09:59	Late Morning	37,9	110	151,4	151,4	19,05	600,69	170,34
2019/05/26	CH2205	Male	Three y/o	Treatment (Wk 2)	Treatment	Fast Day	No	09:15	09:00 - 09:59	Late Morning	37,4	108	226,0	226,0	18,55	599,39	184,42
2019/05/26	CH2206	Male	Three y/o	Treatment (Wk 2)	Treatment	Fast Day	No	09:15	09:00 - 09:59	Late Morning	37,8	92	250,4	250,4	18,95	587,78	188,04
2019/05/26	CH2205	Male	Three y/o	Treatment (Wk 2)	Treatment	Fast Day	No	09:20	09:00 - 09:59	Late Morning	37,4		104,0	104,0	18,55		157,21
2019/05/26	CH2206	Male	Three y/o	Treatment (Wk 2)	Treatment	Fast Day	No	09:20	09:00 - 09:59	Late Morning	37,8	124	300,2	300,2	18,95	609,04	194,44
2019/05/26	CH2205	Male	Three y/o	Treatment (Wk 2)	Treatment	Fast Day	No	09:25	09:00 - 09:59	Late Morning	37,5		79,4	79,4	18,65		147,81
2019/05/26	CH2206	Male	Three y/o	Treatment (Wk 2)	Treatment	Fast Day	No	09:25	09:00 - 09:59	Late Morning	37,8	104	139,0	139,0	18,95	596,69	167,35
2019/05/26	CH2205	Male	Three y/o	Treatment (Wk 2)	Treatment	Fast Day	No	09:30	09:00 - 09:59	Late Morning	37,4	68	21,2	21,2	18,55	564,71	102,31
2019/05/26	CH2206	Male	Three y/o	Treatment (Wk 2)	Treatment	Fast Day	No	09:30	09:00 - 09:59	Late Morning	37,9	110	154,6	154,6	19,05	600,69	171,08
2019/05/26	CH2205	Male	Three y/o	Treatment (Wk 2)	Treatment	Fast Day	No	09:35	09:00 - 09:59	Late Morning	37,5	124	199,2	199,2	18,65	609,04	179,98
2019/05/26	CH2206	Male	Three y/o	Treatment (Wk 2)	Treatment	Fast Day	No	09:35	09:00 - 09:59	Late Morning	37,9	118	154,0	154,0	19,05	605,61	170,94
2019/05/26	CH2205	Male	Three y/o	Treatment (Wk 2)	Treatment	Fast Day	No	09:40	09:00 - 09:59	Late Morning	37,5	147	10,0	10,0	18,65	620,52	76,76
2019/05/26	CH2206	Male	Three y/o	Treatment (Wk 2)	Treatment	Fast Day	No	09:40	09:00 - 09:59	Late Morning	37,9	84	35,0	35,0	19,05	581,01	119,49
2019/05/26	CH2205	Male	Three y/o	Treatment (Wk 2)	Treatment	Fast Day	No	09:45	09:00 - 09:59	Late Morning	37,6	63	18,6	18,6	18,75	558,62	97,84
2019/05/26	CH2206	Male	Three y/o	Treatment (Wk 2)	Treatment	Fast Day	No	09:45	09:00 - 09:59	Late Morning	37,8	50	37,0	37,0	18,95	539,52	121,40
2019/05/26	CH2205	Male	Three y/o	Treatment (Wk 2)	Treatment	Fast Day	No	09:50	09:00 - 09:59	Late Morning	37,6	66	23,4	23,4	18,75	562,34	105,68
2019/05/26	CH2206	Male	Three y/o	Treatment (Wk 2)	Treatment	Fast Day	No	09:50	09:00 - 09:59	Late Morning	37,8	90	18,4	18,4	18,95	586,16	97,47
2019/05/26	CH2205	Male	Three y/o	Treatment (Wk 2)	Treatment	Fast Day	No	09:55	09:00 - 09:59	Late Morning	37,6	49	21,4	21,4	18,75	537,80	102,63
2019/05/26	CH2206	Male	Three y/o	Treatment (Wk 2)	Treatment	Fast Day	No	09:55	09:00 - 09:59	Late Morning	37,8	60	17,0	17,0	18,95	554,67	94,77
2019/05/26	CH2205	Male	Three y/o	Treatment (Wk 2)	Treatment	Fast Day	No	10:00	10:00 - 10:59	Late Morning	37,6	49	23,6	23,6	18,75	537,80	105,97

2019/05/26	CH2206	Male	Three y/o	Treatment (Wk 2)	Treatment	Fast Day	No	10:00	10:00 - 10:59	Late Morning	37,8	47	24,0	24,0	18,95	534,23	106,55
2019/05/26	CH2205	Male	Three y/o	Treatment (Wk 2)	Treatment	Fast Day	No	10:05	10:00 - 10:59	Late Morning	37,6	63	22,2	22,2	18,75	558,62	103,88
2019/05/26	CH2206	Male	Three y/o	Treatment (Wk 2)	Treatment	Fast Day	No	10:05	10:00 - 10:59	Late Morning	37,9	57	15,4	15,4	19,05	550,47	91,41
2019/05/26	CH2205	Male	Three y/o	Treatment (Wk 2)	Treatment	Fast Day	No	10:10	10:00 - 10:59	Late Morning	37,6	61	24,0	24,0	18,75	556,01	106,55
2019/05/26	CH2206	Male	Three y/o	Treatment (Wk 2)	Treatment	Fast Day	No	10:10	10:00 - 10:59	Late Morning	37,9	63	10,6	10,6	19,05	558,62	78,73
2019/05/26	CH2205	Male	Three y/o	Treatment (Wk 2)	Treatment	Fast Day	No	10:15	10:00 - 10:59	Late Morning	37,6	42	24,0	24,0	18,75	524,42	106,55
2019/05/26	CH2206	Male	Three y/o	Treatment (Wk 2)	Treatment	Fast Day	No	10:15	10:00 - 10:59	Late Morning	37,8	47	12,0	12,0	18,95	534,23	82,93
2019/05/26	CH2205	Male	Three y/o	Treatment (Wk 2)	Treatment	Fast Day	No	10:20	10:00 - 10:59	Late Morning	37,6	65	17,0	17,0	18,75	561,12	94,77
2019/05/26	CH2206	Male	Three y/o	Treatment (Wk 2)	Treatment	Fast Day	No	10:20	10:00 - 10:59	Late Morning	37,8	43	15,0	15,0	18,95	526,50	90,51
2019/05/26	CH2205	Male	Three y/o	Treatment (Wk 2)	Treatment	Fast Day	No	10:25	10:00 - 10:59	Late Morning	37,5	48	12,8	12,8	18,65	536,04	85,12
2019/05/26	CH2206	Male	Three y/o	Treatment (Wk 2)	Treatment	Fast Day	No	10:25	10:00 - 10:59	Late Morning	37,8	58	25,4	25,4	18,95	551,90	108,49
2019/05/26	CH2205	Male	Three y/o	Treatment (Wk 2)	Treatment	Fast Day	No	10:30	10:00 - 10:59	Late Morning	37,4	53	20,6	20,6	18,55	544,43	101,32
2019/05/26	CH2206	Male	Three y/o	Treatment (Wk 2)	Treatment	Fast Day	No	10:30	10:00 - 10:59	Late Morning	37,8	83	75,2	75,2	18,95	580,11	145,93
2019/05/26	CH2205	Male	Three y/o	Treatment (Wk 2)	Treatment	Fast Day	No	10:35	10:00 - 10:59	Late Morning	37,4		36,8	36,8	18,55		121,22
2019/05/26	CH2206	Male	Three y/o	Treatment (Wk 2)	Treatment	Fast Day	No	10:35	10:00 - 10:59	Late Morning	37,8	100	87,4	87,4	18,95	593,87	151,15
2019/05/26	CH2205	Male	Three y/o	Treatment (Wk 2)	Treatment	Fast Day	No	10:40	10:00 - 10:59	Late Morning	37,4		9,2	9,2	18,55		73,94
2019/05/26	CH2206	Male	Three y/o	Treatment (Wk 2)	Treatment	Fast Day	No	10:40	10:00 - 10:59	Late Morning	37,8	113	42,4	42,4	18,95	602,58	126,10
2019/05/26	CH2205	Male	Three y/o	Treatment (Wk 2)	Treatment	Fast Day	No	10:45	10:00 - 10:59	Late Morning	37,3	77	3,4	3,4	18,46	574,39	40,51
2019/05/26	CH2206	Male	Three y/o	Treatment (Wk 2)	Treatment	Fast Day	No	10:45	10:00 - 10:59	Late Morning	37,7	141	177,2	177,2	18,85	617,75	175,86
2019/05/26	CH2205	Male	Three y/o	Treatment (Wk 2)	Treatment	Fast Day	No	10:50	10:00 - 10:59	Late Morning	37,3	65	12,0	12,0	18,46	561,12	82,93
2019/05/26	CH2206	Male	Three y/o	Treatment (Wk 2)	Treatment	Fast Day	No	10:50	10:00 - 10:59	Late Morning	37,7	90	107,8	107,8	18,85	586,16	158,46
2019/05/26	CH2205	Male	Three y/o	Treatment (Wk 2)	Treatment	Fast Day	No	10:55	10:00 - 10:59	Late Morning	37,4	42	3,2	3,2	18,55	524,42	38,49
2019/05/26	CH2206	Male	Three y/o	Treatment (Wk 2)	Treatment	Fast Day	No	10:55	10:00 - 10:59	Late Morning	37,7	120	77,2	77,2	18,85	606,78	146,84
2019/05/26	CH2205	Male	Three y/o	Treatment (Wk 2)	Treatment	Fast Day	No	11:00	11:00 - 11:59	Late Morning	37,4	52	37,0	37,0	18,55	542,83	121,40
2019/05/26	CH2206	Male	Three y/o	Treatment (Wk 2)	Treatment	Fast Day	No	11:00	11:00 - 11:59	Late Morning	37,8		413,8	413,8	18,95		205,82
2019/05/26	CH2205	Male	Three y/o	Treatment (Wk 2)	Treatment	Fast Day	No	11:05	11:00 - 11:59	Late Morning	37,4	78	137,4	137,4	18,55	575,38	166,94
2019/05/26	CH2206	Male	Three y/o	Treatment (Wk 2)	Treatment	Fast Day	No	11:05	11:00 - 11:59	Late Morning	37,9	118	408,8	408,8	19,05	605,61	205,39
2019/05/26	CH2205	Male	Three y/o	Treatment (Wk 2)	Treatment	Fast Day	No	11:10	11:00 - 11:59	Late Morning	37,5	172	378,8	378,8	18,65	630,73	202,68
2019/05/26	CH2206	Male	Three y/o	Treatment (Wk 2)	Treatment	Fast Day	No	11:10	11:00 - 11:59	Late Morning	38,0		32,0	32,0	19,15		116,41
2019/05/26	CH2205	Male	Three y/o	Treatment (Wk 2)	Treatment	Fast Day	No	11:15	11:00 - 11:59	Late Morning	37,6	75	18,0	18,0	18,75	572,36	96,72
2019/05/26	CH2206	Male	Three y/o	Treatment (Wk 2)	Treatment	Fast Day	No	11:15	11:00 - 11:59	Late Morning	38,0	84	36,6	36,6	19,15	581,01	121,03
2019/05/26	CH2205	Male	Three y/o	Treatment (Wk 2)	Treatment	Fast Day	No	11:20	11:00 - 11:59	Late Morning	37,7		34,4	34,4	18,85		118,90
2019/05/26	CH2206	Male	Three y/o	Treatment (Wk 2)	Treatment	Fast Day	No	11:20	11:00 - 11:59	Late Morning	37,9	93	25,6	25,6	19,05	588,58	108,76
2019/05/26	CH2205	Male	Three y/o	Treatment (Wk 2)	Treatment	Fast Day	No	11:25	11:00 - 11:59	Late Morning	37,7		35,2	35,2	18,85		119,69
2019/05/26	CH2206	Male	Three y/o	Treatment (Wk 2)	Treatment	Fast Day	No	11:25	11:00 - 11:59	Late Morning	37,9	138	85,4	85,4	19,05	616,31	150,35
2019/05/26	CH2205	Male	Three y/o	Treatment (Wk 2)	Treatment	Fast Day	No	11:30	11:00 - 11:59	Late Morning	37,7		119,2	119,2	18,85		161,97
2019/05/26	CH2206	Male	Three y/o	Treatment (Wk 2)	Treatment	Fast Day	No	11:30	11:00 - 11:59	Late Morning	37,9	80	123,0	123,0	19,05	577,31	163,07
2019/05/26	CH2205	Male	Three y/o	Treatment (Wk 2)	Treatment	Fast Day	No	11:35	11:00 - 11:59	Late Morning	37,7		158,6	158,6	18,85		171,97
2019/05/26	CH2206	Male	Three y/o	Treatment (Wk 2)	Treatment	Fast Day	No	11:35	11:00 - 11:59	Late Morning	38,0	92	51,8	51,8	19,15	587,78	133,01
2019/05/26	CH2205	Male	Three y/o	Treatment (Wk 2)	Treatment	Fast Day	No	11:40	11:00 - 11:59	Late Morning	38,0	80	53,2	53,2	19,15	577,31	133,93
2019/05/26	CH2206	Male	Three y/o	Treatment (Wk 2)	Treatment	Fast Day	No	11:40	11:00 - 11:59	Late Morning	38,0		15,6	15,6	19,15		91,85
2019/05/26	CH2205	Male	Three y/o	Treatment (Wk 2)	Treatment	Fast Day	No	11:45	11:00 - 11:59	Late Morning	38,0	76	12,4	12,4	19,15	573,39	84,04
2019/05/26	CH2206	Male	Three y/o	Treatment (Wk 2)	Treatment	Fast Day	No	11:45	11:00 - 11:59	Late Morning	38,0	105	36,2	36,2	19,15	597,38	120,65
2019/05/26	CH2205	Male	Three y/o	Treatment (Wk 2)	Treatment	Fast Day	No	11:50	11:00 - 11:59	Late Morning	38,1		20,8	20,8	19,26		101,65
2019/05/26	CH2206	Male	Three y/o	Treatment (Wk 2)	Treatment	Fast Day	No	11:50	11:00 - 11:59	Late Morning	37,9	96	181,0	181,0	19,05	590,91	176,61
2019/05/26	CH2205	Male	Three y/o	Treatment (Wk 2)	Treatment	Fast Day	No	11:55	11:00 - 11:59	Late Morning	38,1	60	5,2	5,2	19,26	554,67	54,72
2019/05/26	CH2206	Male	Three y/o	Treatment (Wk 2)	Treatment	Fast Day	No	11:55	11:00 - 11:59	Late Morning	37,9	56	62,8	62,8	19,05	549,01	139,67
2019/05/26	CH2205	Male	Three y/o	Treatment (Wk 2)	Treatment	Fast Day	No	12:00	12:00 - 12:59	Afternoon	38,1	54	24,6	24,6	19,26	545,99	107,39
2019/05/26	CH2206	Male	Three y/o	Treatment (Wk 2)	Treatment	Fast Day	No	12:00	12:00 - 12:59	Afternoon	37,9	62	54,0	54,0	19,05	557,33	134,45
2019/05/26	CH2205	Male	Three y/o	Treatment (Wk 2)	Treatment	Fast Day	No	12:05	12:00 - 12:59	Afternoon	38,0	55	8,6	8,6	19,15	547,52	71,66
2019/05/26	CH2206	Male	Three y/o	Treatment (Wk 2)	Treatment	Fast Day	No	12:05	12:00 - 12:59	Afternoon	37,9	52	63,2	63,2	19,05	542,83	139,89
2019/05/26	CH2205	Male	Three y/o	Treatment (Wk 2)	Treatment	Fast Day	No	12:10	12:00 - 12:59	Afternoon	38,0	57	4,4	4,4	19,15	550,47	49,12
2019/05/26	CH2206	Male	Three y/o	Treatment (Wk 2)	Treatment	Fast Day	No	12:10	12:00 - 12:59	Afternoon	37,9	62	74,0	74,0	19,05	557,33	145,37
2019/05/26	CH2205	Male	Three y/o	Treatment (Wk 2)	Treatment	Fast Day	No	12:15	12:00 - 12:59	Afternoon	38,0	47	7,4	7,4	19,15	534,23	66,59
2019/05/26	CH2206	Male	Three y/o	Treatment (Wk 2)	Treatment	Fast Day	No	12:15	12:00 - 12:59	Afternoon	37,9	46	66,4	66,4	19,05	532,38	141,61
2019/05/26	CH2205	Male	Three y/o	Treatment (Wk 2)	Treatment	Fast Day	No	12:20	12:00 - 12:59	Afternoon	38,0	47	36,0	36,0	19,15	534,23	120,46
2019/05/26	CH2206	Male	Three y/o	Treatment (Wk 2)	Treatment	Fast Day	No	12:20	12:00 - 12:59	Afternoon	37,9	48	66,2	66,2	19,05	536,04	141,50
2019/05/26	CH2205	Male	Three y/o	Treatment (Wk 2)	Treatment	Fast Day	No	12:25	12:00 - 12:59	Afternoon	37,9	46	31,0	31,0	19,05	532,38	115,32
2019/05/26	CH2206	Male	Three y/o	Treatment (Wk 2)	Treatment	Fast Day	No	12:25	12:00 - 12:59	Afternoon	37,9	47	65,6	65,6	19,05	534,23	141,19
2019/05/26	CH2205	Male	Three y/o	Treatment (Wk 2)	Treatment	Fast Day	No	12:30	12:00 - 12:59	Afternoon	37,9	51	26,4	26,4	19,05	541,20	109,81
2019/05/26	CH2206	Male	Three y/o	Treatment (Wk 2)	Treatment	Fast Day	No	12:30	12:00 - 12:59	Afternoon	37,8	46	59,4	59,4	18,95	532,38	137,75
2019/05/26	CH2205	Male	Three y/o	Treatment (Wk 2)	Treatment	Fast Day	No	12:35	12:00 - 12:59	Afternoon	37,9		29,0	29,0	19,05		113,03
2019/05/26	CH2206	Male	Three y/o	Treatment (Wk 2)	Treatment	Fast Day	No	12:35	12:00 - 12:59	Afternoon	37,8	49	57,6	57,6	18,95	537,80	136,68
2019/05/26	CH2205	Male	Three y/o	Treatment (Wk 2)	Treatment	Fast Day	No	12:40	12:00 - 12:59	Afternoon	37,7	37	28,0	28,0	18,85	513,06	111,83
2019/05/26	CH2206	Male	Three y/o	Treatment (Wk 2)	Treatment	Fast Day	No	12:40	12:00 - 12:59	Afternoon	37,8	47	62,2	62,2	18,95	534,23	139,34
2019/05/26	CH2205	Male	Three y/o	Treatment (Wk 2)	Treatment	Fast Day	No	12:45	12:00 - 12:59	Afternoon	37,7	47	29,2	29,2	18,85	534,23	113,27
2019/05/26	CH2206	Male	Three y/o	Treatment (Wk 2)	Treatment	Fast Day	No	12:45	12:00 - 12:59	Afternoon	37,8	43	61,2	61,2	18,95	526,50	138,78
2019/05/26	CH2205	Male	Three y/o	Treatment (Wk 2)	Treatment	Fast Day	No	12:50	12:00 - 12:59	Afternoon	37,6	44	47,2	47,2	18,75	528,51	129,80
2019/05/26	CH2206	Male	Three y/o	Treatment (Wk 2)	Treatment	Fast Day	No	12:50	12:00 - 12:59	Afternoon	37,8	48	57,2	57,2	18,95	536,04	136,44
2019/05/26	CH2205	Male	Three y/o	Treatment (Wk 2)	Treatment	Fast Day	No	12:55	12:00 - 12:59	Afternoon	37,5	49	17,0	17,0	18,65	537,80	94,77

2019/05/26	CH2206	Male	Three y/o	Treatment (Wk 2)	Treatment	Fast Day	No	12:55	12:00 - 12:59	Afternoon	37,8	47	45,2	45,2	18,95	534,23	128,30
2019/05/26	CH2205	Male	Three y/o	Treatment (Wk 2)	Treatment	Fast Day	No	13:00	13:00 - 13:59	Afternoon	37,6	61	23,6	23,6	18,75	556,01	105,97
2019/05/26	CH2206	Male	Three y/o	Treatment (Wk 2)	Treatment	Fast Day	No	13:00	13:00 - 13:59	Afternoon	37,7	79	97,6	97,6	18,85	576,36	155,00
2019/05/26	CH2205	Male	Three y/o	Treatment (Wk 2)	Treatment	Fast Day	No	13:05	13:00 - 13:59	Afternoon	37,6		27,0	27,0	18,75		110,58
2019/05/26	CH2206	Male	Three y/o	Treatment (Wk 2)	Treatment	Fast Day	No	13:05	13:00 - 13:59	Afternoon	37,8	103	196,4	196,4	18,95	596,00	179,48
2019/05/26	CH2205	Male	Three y/o	Treatment (Wk 2)	Treatment	Fast Day	No	13:10	13:00 - 13:59	Afternoon	37,6	64	31,8	31,8	18,75	559,88	116,20
2019/05/26	CH2206	Male	Three y/o	Treatment (Wk 2)	Treatment	Fast Day	No	13:10	13:00 - 13:59	Afternoon	37,8		79,2	79,2	18,95		147,73
2019/05/26	CH2205	Male	Three y/o	Treatment (Wk 2)	Treatment	Fast Day	No	13:15	13:00 - 13:59	Afternoon	37,6		42,8	42,8	18,75		126,42
2019/05/26	CH2206	Male	Three y/o	Treatment (Wk 2)	Treatment	Fast Day	No	13:15	13:00 - 13:59	Afternoon	37,7	94	19,6	19,6	18,85	589,37	99,63
2019/05/26	CH2205	Male	Three y/o	Treatment (Wk 2)	Treatment	Fast Day	No	13:20	13:00 - 13:59	Afternoon	37,6		13,8	13,8	18,75		87,68
2019/05/26	CH2206	Male	Three y/o	Treatment (Wk 2)	Treatment	Fast Day	No	13:20	13:00 - 13:59	Afternoon	37,7	47	14,4	14,4	18,85	534,23	89,12
2019/05/26	CH2205	Male	Three y/o	Treatment (Wk 2)	Treatment	Fast Day	No	13:25	13:00 - 13:59	Afternoon	37,5	42	10,4	10,4	18,65	524,42	78,08
2019/05/26	CH2206	Male	Three y/o	Treatment (Wk 2)	Treatment	Fast Day	No	13:25	13:00 - 13:59	Afternoon	37,7	46	30,6	30,6	18,85	532,38	114,87
2019/05/26	CH2205	Male	Three y/o	Treatment (Wk 2)	Treatment	Fast Day	No	13:30	13:00 - 13:59	Afternoon	37,5	55	83,8	83,8	18,65	547,52	149,69
2019/05/26	CH2206	Male	Three y/o	Treatment (Wk 2)	Treatment	Fast Day	No	13:30	13:00 - 13:59	Afternoon	37,7	70	44,0	44,0	18,85	566,99	127,37
2019/05/26	CH2205	Male	Three y/o	Treatment (Wk 2)	Treatment	Fast Day	No	13:35	13:00 - 13:59	Afternoon	37,6	72	62,2	62,2	18,75	569,20	139,34
2019/05/26	CH2206	Male	Three y/o	Treatment (Wk 2)	Treatment	Fast Day	No	13:35	13:00 - 13:59	Afternoon	37,7	98	270,8	270,8	18,85	592,41	190,80
2019/05/26	CH2205	Male	Three y/o	Treatment (Wk 2)	Treatment	Fast Day	No	13:40	13:00 - 13:59	Afternoon	37,6	44	26,6	26,6	18,75	528,51	110,07
2019/05/26	CH2206	Male	Three y/o	Treatment (Wk 2)	Treatment	Fast Day	No	13:40	13:00 - 13:59	Afternoon	37,8	104	139,8	139,8	18,95	596,69	167,55
2019/05/26	CH2205	Male	Three y/o	Treatment (Wk 2)	Treatment	Fast Day	No	13:45	13:00 - 13:59	Afternoon	37,6		15,0	15,0	18,75		90,51
2019/05/26	CH2206	Male	Three y/o	Treatment (Wk 2)	Treatment	Fast Day	No	13:45	13:00 - 13:59	Afternoon	37,7	72	51,0	51,0	18,85	569,20	132,47
2019/05/26	CH2205	Male	Three y/o	Treatment (Wk 2)	Treatment	Fast Day	No	13:50	13:00 - 13:59	Afternoon	37,6	65	13,8	13,8	18,75	561,12	87,68
2019/05/26	CH2206	Male	Three y/o	Treatment (Wk 2)	Treatment	Fast Day	No	13:50	13:00 - 13:59	Afternoon	37,8	52	42,8	42,8	18,95	542,83	126,42
2019/05/26	CH2205	Male	Three y/o	Treatment (Wk 2)	Treatment	Fast Day	No	13:55	13:00 - 13:59	Afternoon	37,6		16,0	16,0	18,75		92,71
2019/05/26	CH2206	Male	Three y/o	Treatment (Wk 2)	Treatment	Fast Day	No	13:55	13:00 - 13:59	Afternoon	37,7	49	51,6	51,6	18,85	537,80	132,87
2019/05/26	CH2205	Male	Three y/o	Treatment (Wk 2)	Treatment	Fast Day	No	14:00	14:00 - 14:59	Afternoon	37,7	58	41,4	41,4	18,85	551,90	125,27
2019/05/26	CH2206	Male	Three y/o	Treatment (Wk 2)	Treatment	Fast Day	No	14:00	14:00 - 14:59	Afternoon	37,8	48	24,2	24,2	18,95	536,04	106,83
2019/05/26	CH2205	Male	Three y/o	Treatment (Wk 2)	Treatment	Fast Day	No	14:05	14:00 - 14:59	Afternoon	37,7	79	149,2	149,2	18,85	576,36	169,83
2019/05/26	CH2206	Male	Three y/o	Treatment (Wk 2)	Treatment	Fast Day	No	14:05	14:00 - 14:59	Afternoon	37,7	52	67,6	67,6	18,85	542,83	142,23
2019/05/26	CH2205	Male	Three y/o	Treatment (Wk 2)	Treatment	Fast Day	No	14:10	14:00 - 14:59	Afternoon	37,7	64	6,8	6,8	18,85	559,88	63,74
2019/05/26	CH2206	Male	Three y/o	Treatment (Wk 2)	Treatment	Fast Day	No	14:10	14:00 - 14:59	Afternoon	37,8	65	60,8	60,8	18,95	561,12	138,55
2019/05/26	CH2205	Male	Three y/o	Treatment (Wk 2)	Treatment	Fast Day	No	14:15	14:00 - 14:59	Afternoon	37,7	62	8,6	8,6	18,85	557,33	71,66
2019/05/26	CH2206	Male	Three y/o	Treatment (Wk 2)	Treatment	Fast Day	No	14:15	14:00 - 14:59	Afternoon	37,8	109	176,8	176,8	18,95	600,04	175,78
2019/05/26	CH2205	Male	Three y/o	Treatment (Wk 2)	Treatment	Fast Day	No	14:20	14:00 - 14:59	Afternoon	37,7		37,8	37,8	18,85		122,14
2019/05/26	CH2206	Male	Three y/o	Treatment (Wk 2)	Treatment	Fast Day	No	14:20	14:00 - 14:59	Afternoon	37,8		59,4	59,4	18,95		137,75
2019/05/26	CH2205	Male	Three y/o	Treatment (Wk 2)	Treatment	Fast Day	No	14:25	14:00 - 14:59	Afternoon	37,9		22,4	22,4	19,05		104,19
2019/05/26	CH2206	Male	Three y/o	Treatment (Wk 2)	Treatment	Fast Day	No	14:25	14:00 - 14:59	Afternoon	37,9	100	170,4	170,4	19,05	593,87	174,49
2019/05/26	CH2205	Male	Three y/o	Treatment (Wk 2)	Treatment	Fast Day	No	14:30	14:00 - 14:59	Afternoon	37,9	68	61,8	61,8	19,05	564,71	139,12
2019/05/26	CH2206	Male	Three y/o	Treatment (Wk 2)	Treatment	Fast Day	No	14:30	14:00 - 14:59	Afternoon	37,9	95	100,6	100,6	19,05	590,14	156,05
2019/05/26	CH2205	Male	Three y/o	Treatment (Wk 2)	Treatment	Fast Day	No	14:35	14:00 - 14:59	Afternoon	37,9	65	67,2	67,2	19,05	561,12	142,02
2019/05/26	CH2206	Male	Three y/o	Treatment (Wk 2)	Treatment	Fast Day	No	14:35	14:00 - 14:59	Afternoon	37,9	76	35,8	35,8	19,05	573,39	120,27
2019/05/26	CH2205	Male	Three y/o	Treatment (Wk 2)	Treatment	Fast Day	No	14:40	14:00 - 14:59	Afternoon	37,9	93	29,0	29,0	19,05	588,58	113,03
2019/05/26	CH2206	Male	Three y/o	Treatment (Wk 2)	Treatment	Fast Day	No	14:40	14:00 - 14:59	Afternoon	37,9		180,2	180,2	19,05		176,45
2019/05/26	CH2205	Male	Three y/o	Treatment (Wk 2)	Treatment	Fast Day	No	14:45	14:00 - 14:59	Afternoon	38,0	63	31,8	31,8	19,15	558,62	116,20
2019/05/26	CH2206	Male	Three y/o	Treatment (Wk 2)	Treatment	Fast Day	No	14:45	14:00 - 14:59	Afternoon	38,0	93	95,2	95,2	19,15	588,58	154,13
2019/05/26	CH2205	Male	Three y/o	Treatment (Wk 2)	Treatment	Fast Day	No	14:50	14:00 - 14:59	Afternoon	38,0	56	10,0	10,0	19,15	549,01	76,76
2019/05/26	CH2206	Male	Three y/o	Treatment (Wk 2)	Treatment	Fast Day	No	14:50	14:00 - 14:59	Afternoon	38,0	128	50,2	50,2	19,15	611,22	131,92
2019/05/26	CH2205	Male	Three y/o	Treatment (Wk 2)	Treatment	Fast Day	No	14:55	14:00 - 14:59	Afternoon	38,0	86	221,4	221,4	19,15	582,77	183,70
2019/05/26	CH2206	Male	Three y/o	Treatment (Wk 2)	Treatment	Fast Day	No	14:55	14:00 - 14:59	Afternoon	38,0	80	199,2	199,2	19,15	577,31	179,98
2019/05/26	CH2205	Male	Three y/o	Treatment (Wk 2)	Treatment	Fast Day	No	15:00	15:00 - 15:59	Afternoon	38,1	72	6,2	6,2	19,26	569,20	60,63
2019/05/26	CH2206	Male	Three y/o	Treatment (Wk 2)	Treatment	Fast Day	No	15:00	15:00 - 15:59	Afternoon	38,1	96	132,2	132,2	19,26	590,91	165,59
2019/05/26	CH2205	Male	Three y/o	Treatment (Wk 2)	Treatment	Fast Day	No	15:05	15:00 - 15:59	Afternoon	38,1	58	202,2	202,2	19,26	551,90	180,50
2019/05/26	CH2206	Male	Three y/o	Treatment (Wk 2)	Treatment	Fast Day	No	15:05	15:00 - 15:59	Afternoon	38,1	100	190,4	190,4	19,26	593,87	178,39
2019/05/26	CH2205	Male	Three y/o	Treatment (Wk 2)	Treatment	Fast Day	No	15:10	15:00 - 15:59	Afternoon	38,1	73	31,8	31,8	19,26	570,27	116,20
2019/05/26	CH2206	Male	Three y/o	Treatment (Wk 2)	Treatment	Fast Day	No	15:10	15:00 - 15:59	Afternoon	38,1	89	62,6	62,6	19,26	585,33	139,56
2019/05/26	CH2205	Male	Three y/o	Treatment (Wk 2)	Treatment	Fast Day	No	15:15	15:00 - 15:59	Afternoon	38,1	59	41,4	41,4	19,26	553,30	125,27
2019/05/26	CH2206	Male	Three y/o	Treatment (Wk 2)	Treatment	Fast Day	No	15:15	15:00 - 15:59	Afternoon	38,2	110	113,2	113,2	19,36	600,69	160,17
2019/05/26	CH2205	Male	Three y/o	Treatment (Wk 2)	Treatment	Fast Day	No	15:20	15:00 - 15:59	Afternoon	38,1	88	198,8	198,8	19,26	584,49	179,91
2019/05/26	CH2206	Male	Three y/o	Treatment (Wk 2)	Treatment	Fast Day	No	15:20	15:00 - 15:59	Afternoon	38,2	103	222,8	222,8	19,36	596,00	183,92
2019/05/26	CH2205	Male	Three y/o	Treatment (Wk 2)	Treatment	Fast Day	No	15:25	15:00 - 15:59	Afternoon	38,2	115	211,4	211,4	19,36	603,82	182,07
2019/05/26	CH2206	Male	Three y/o	Treatment (Wk 2)	Treatment	Fast Day	No	15:25	15:00 - 15:59	Afternoon	38,3	156	217,4	217,4	19,46	624,43	183,06
2019/05/26	CH2205	Male	Three y/o	Treatment (Wk 2)	Treatment	Fast Day	No	15:30	15:00 - 15:59	Afternoon	38,2		67,4	67,4	19,36		142,12
2019/05/26	CH2206	Male	Three y/o	Treatment (Wk 2)	Treatment	Fast Day	No	15:30	15:00 - 15:59	Afternoon	38,4	92	107,0	107,0	19,56	587,78	158,20
2019/05/26	CH2205	Male	Three y/o	Treatment (Wk 2)	Treatment	Fast Day	No	15:35	15:00 - 15:59	Afternoon	38,3	126	258,0	258,0	19,46	610,14	189,09
2019/05/26	CH2206	Male	Three y/o	Treatment (Wk 2)	Treatment	Fast Day	No	15:35	15:00 - 15:59	Afternoon	38,4	174	181,8	181,8	19,56	631,46	176,76
2019/05/26	CH2205	Male	Three y/o	Treatment (Wk 2)	Treatment	Fast Day	No	15:40	15:00 - 15:59	Afternoon	38,5	72	20,2	20,2	19,66	569,20	100,65
2019/05/26	CH2206	Male	Three y/o	Treatment (Wk 2)	Treatment	Fast Day	No	15:40	15:00 - 15:59	Afternoon	38,6	119	117,4	117,4	19,76	606,20	161,44
2019/05/26	CH2205	Male	Three y/o	Treatment (Wk 2)	Treatment	Fast Day	No	15:45	15:00 - 15:59	Afternoon	38,5	56	26,8	26,8	19,66	549,01	110,33
2019/05/26	CH2206	Male	Three y/o	Treatment (Wk 2)	Treatment	Fast Day	No	15:45	15:00 - 15:59	Afternoon	38,6	91	343,2	343,2	19,76	586,98	199,18
2019/05/26	CH2205	Male	Three y/o	Treatment (Wk 2)	Treatment	Fast Day	No	15:50	15:00 - 15:59	Afternoon	38,5	53	31,8	31,8	19,66	544,43	116,20

2019/05/26	CH2206	Male	Three y/o	Treatment (Wk 2)	Treatment	Fast Day	No	15:50	15:00 - 15:59	Afternoon	38,6	103	78,2	78,2	19,76	596,00	147,28
2019/05/26	CH2205	Male	Three y/o	Treatment (Wk 2)	Treatment	Fast Day	No	15:55	15:00 - 15:59	Afternoon	38,5	50	58,6	58,6	19,66	539,52	137,28
2019/05/26	CH2206	Male	Three y/o	Treatment (Wk 2)	Treatment	Fast Day	No	15:55	15:00 - 15:59	Afternoon	38,6	66	241,2	241,2	19,76	562,34	186,72
2019/05/26	CH2205	Male	Three y/o	Treatment (Wk 2)	Treatment	Fast Day	No	16:00	16:00 - 16:59	Evening	38,5	64	24,2	24,2	19,66	559,88	106,83
2019/05/26	CH2206	Male	Three y/o	Treatment (Wk 2)	Treatment	Fast Day	No	16:00	16:00 - 16:59	Evening	38,6	97	178,2	178,2	19,76	591,66	176,06
2019/05/26	CH2205	Male	Three y/o	Treatment (Wk 2)	Treatment	Fast Day	No	16:05	16:00 - 16:59	Evening	38,5	70	20,2	20,2	19,66	566,99	100,65
2019/05/26	CH2206	Male	Three y/o	Treatment (Wk 2)	Treatment	Fast Day	No	16:05	16:00 - 16:59	Evening	38,6	92	38,0	38,0	19,76	587,78	122,32
2019/05/26	CH2205	Male	Three y/o	Treatment (Wk 2)	Treatment	Fast Day	No	16:10	16:00 - 16:59	Evening	38,5	61	23,2	23,2	19,66	556,01	105,39
2019/05/26	CH2206	Male	Three y/o	Treatment (Wk 2)	Treatment	Fast Day	No	16:10	16:00 - 16:59	Evening	38,4	67	126,2	126,2	19,56	563,53	163,97
2019/05/26	CH2205	Male	Three y/o	Treatment (Wk 2)	Treatment	Fast Day	No	16:15	16:00 - 16:59	Evening	38,5	54	24,2	24,2	19,66	545,99	106,83
2019/05/26	CH2206	Male	Three y/o	Treatment (Wk 2)	Treatment	Fast Day	No	16:15	16:00 - 16:59	Evening	38,4	85	29,2	29,2	19,56	581,90	113,27
2019/05/26	CH2205	Male	Three y/o	Treatment (Wk 2)	Treatment	Fast Day	No	16:20	16:00 - 16:59	Evening	38,5	45	25,6	25,6	19,66	530,47	108,76
2019/05/26	CH2206	Male	Three y/o	Treatment (Wk 2)	Treatment	Fast Day	No	16:20	16:00 - 16:59	Evening	38,4	48	36,6	36,6	19,56	536,04	121,03
2019/05/26	CH2205	Male	Three y/o	Treatment (Wk 2)	Treatment	Fast Day	No	16:25	16:00 - 16:59	Evening	38,4	55	28,2	28,2	19,56	547,52	112,07
2019/05/26	CH2206	Male	Three y/o	Treatment (Wk 2)	Treatment	Fast Day	No	16:25	16:00 - 16:59	Evening	38,4	45	32,0	32,0	19,56	530,47	116,41
2019/05/26	CH2205	Male	Three y/o	Treatment (Wk 2)	Treatment	Fast Day	No	16:30	16:00 - 16:59	Evening	38,4	45	25,0	25,0	19,56	530,47	107,94
2019/05/26	CH2206	Male	Three y/o	Treatment (Wk 2)	Treatment	Fast Day	No	16:30	16:00 - 16:59	Evening	38,3	46	34,2	34,2	19,46	532,38	118,70
2019/05/26	CH2205	Male	Three y/o	Treatment (Wk 2)	Treatment	Fast Day	No	16:35	16:00 - 16:59	Evening	38,3	45	25,8	25,8	19,46	530,47	109,02
2019/05/26	CH2206	Male	Three y/o	Treatment (Wk 2)	Treatment	Fast Day	No	16:35	16:00 - 16:59	Evening	38,3	51	44,6	44,6	19,46	541,20	127,84
2019/05/26	CH2205	Male	Three y/o	Treatment (Wk 2)	Treatment	Fast Day	No	16:40	16:00 - 16:59	Evening	38,3	82	34,0	34,0	19,46	579,19	118,49
2019/05/26	CH2206	Male	Three y/o	Treatment (Wk 2)	Treatment	Fast Day	No	16:40	16:00 - 16:59	Evening	38,3	86	161,2	161,2	19,46	582,77	172,54
2019/05/26	CH2205	Male	Three y/o	Treatment (Wk 2)	Treatment	Fast Day	No	16:45	16:00 - 16:59	Evening	38,3	99	251,0	251,0	19,46	593,14	188,12
2019/05/26	CH2206	Male	Three y/o	Treatment (Wk 2)	Treatment	Fast Day	No	16:45	16:00 - 16:59	Evening	38,3	80	56,6	56,6	19,46	577,31	136,07
2019/05/26	CH2205	Male	Three y/o	Treatment (Wk 2)	Treatment	Fast Day	No	16:50	16:00 - 16:59	Evening	38,3	84	25,4	25,4	19,46	581,01	108,49
2019/05/26	CH2206	Male	Three y/o	Treatment (Wk 2)	Treatment	Fast Day	No	16:50	16:00 - 16:59	Evening	38,3	45	70,4	70,4	19,46	530,47	143,64
2019/05/26	CH2205	Male	Three y/o	Treatment (Wk 2)	Treatment	Fast Day	No	16:55	16:00 - 16:59	Evening	38,2	125	53,8	53,8	19,36	609,60	134,32
2019/05/26	CH2206	Male	Three y/o	Treatment (Wk 2)	Treatment	Fast Day	No	16:55	16:00 - 16:59	Evening	38,3	64	29,6	29,6	19,46	559,88	113,73
2019/05/26	CH2205	Male	Three y/o	Treatment (Wk 2)	Treatment	Fast Day	No	17:00	17:00 - 17:59	Evening	38,2	53	50,2	50,2	19,36	544,43	131,92
2019/05/26	CH2206	Male	Three y/o	Treatment (Wk 2)	Treatment	Fast Day	No	17:00	17:00 - 17:59	Evening	38,3	46	23,4	23,4	19,46	532,38	105,68
2019/05/26	CH2205	Male	Three y/o	Treatment (Wk 2)	Treatment	Fast Day	No	17:05	17:00 - 17:59	Evening	38,2	39	23,6	23,6	19,36	517,82	105,97
2019/05/26	CH2206	Male	Three y/o	Treatment (Wk 2)	Treatment	Fast Day	No	17:05	17:00 - 17:59	Evening	38,2	45	26,4	26,4	19,36	530,47	109,81
2019/05/26	CH2205	Male	Three y/o	Treatment (Wk 2)	Treatment	Fast Day	No	17:10	17:00 - 17:59	Evening	38,1	41	20,6	20,6	19,26	522,29	101,32
2019/05/26	CH2206	Male	Three y/o	Treatment (Wk 2)	Treatment	Fast Day	No	17:10	17:00 - 17:59	Evening	38,2	72	26,4	26,4	19,36	569,20	109,81
2019/05/26	CH2205	Male	Three y/o	Treatment (Wk 2)	Treatment	Fast Day	No	17:15	17:00 - 17:59	Evening	38,0	54	22,8	22,8	19,15	545,99	104,79
2019/05/26	CH2206	Male	Three y/o	Treatment (Wk 2)	Treatment	Fast Day	No	17:15	17:00 - 17:59	Evening	38,2	49	33,2	33,2	19,36	537,80	117,68
2019/05/26	CH2205	Male	Three y/o	Treatment (Wk 2)	Treatment	Fast Day	No	17:20	17:00 - 17:59	Evening	38,0	56	24,2	24,2	19,15	549,01	106,83
2019/05/26	CH2206	Male	Three y/o	Treatment (Wk 2)	Treatment	Fast Day	No	17:20	17:00 - 17:59	Evening	38,1	67	22,6	22,6	19,26	563,53	104,49
2019/05/26	CH2205	Male	Three y/o	Treatment (Wk 2)	Treatment	Fast Day	No	17:25	17:00 - 17:59	Evening	38,0	65	4,8	4,8	19,15	561,12	52,04
2019/05/26	CH2206	Male	Three y/o	Treatment (Wk 2)	Treatment	Fast Day	No	17:25	17:00 - 17:59	Evening	38,1	45	13,2	13,2	19,26	530,47	86,17
2019/05/26	CH2205	Male	Three y/o	Treatment (Wk 2)	Treatment	Fast Day	No	17:30	17:00 - 17:59	Evening	38,1	47	4,8	4,8	19,26	534,23	52,04
2019/05/26	CH2206	Male	Three y/o	Treatment (Wk 2)	Treatment	Fast Day	No	17:30	17:00 - 17:59	Evening	37,9	50	29,0	29,0	19,05	539,52	113,03
2019/05/26	CH2205	Male	Three y/o	Treatment (Wk 2)	Treatment	Fast Day	No	17:35	17:00 - 17:59	Evening	38,0	54	14,0	14,0	19,15	545,99	88,17
2019/05/26	CH2206	Male	Three y/o	Treatment (Wk 2)	Treatment	Fast Day	No	17:35	17:00 - 17:59	Evening	37,8	42	36,2	36,2	18,95	524,42	120,65
2019/05/26	CH2205	Male	Three y/o	Treatment (Wk 2)	Treatment	Fast Day	No	17:40	17:00 - 17:59	Evening	38,0	47	29,6	29,6	19,15	534,23	113,73
2019/05/26	CH2206	Male	Three y/o	Treatment (Wk 2)	Treatment	Fast Day	No	17:40	17:00 - 17:59	Evening	37,7	44	31,8	31,8	18,85	528,51	116,20
2019/05/26	CH2205	Male	Three y/o	Treatment (Wk 2)	Treatment	Fast Day	No	17:45	17:00 - 17:59	Evening	38,0	45	28,6	28,6	19,15	530,47	112,56
2019/05/26	CH2206	Male	Three y/o	Treatment (Wk 2)	Treatment	Fast Day	No	17:45	17:00 - 17:59	Evening	37,7	42	25,8	25,8	18,85	524,42	109,02
2019/05/26	CH2205	Male	Three y/o	Treatment (Wk 2)	Treatment	Fast Day	No	17:50	17:00 - 17:59	Evening	38,0	49	20,6	20,6	19,15	537,80	101,32
2019/05/26	CH2206	Male	Three y/o	Treatment (Wk 2)	Treatment	Fast Day	No	17:50	17:00 - 17:59	Evening	37,7	47	36,2	36,2	18,85	534,23	120,65
2019/05/26	CH2205	Male	Three y/o	Treatment (Wk 2)	Treatment	Fast Day	No	17:55	17:00 - 17:59	Evening	37,9	169	6,6	6,6	19,05	629,60	62,73
2019/05/26	CH2206	Male	Three y/o	Treatment (Wk 2)	Treatment	Fast Day	No	17:55	17:00 - 17:59	Evening	37,8	39	38,8	38,8	18,95	517,82	123,04
2019/05/26	CH2205	Male	Three y/o	Treatment (Wk 2)	Treatment	Fast Day	No	18:00	18:00 - 18:59	Evening	37,9		8,8	8,8	19,05		72,43
2019/05/26	CH2206	Male	Three y/o	Treatment (Wk 2)	Treatment	Fast Day	No	18:00	18:00 - 18:59	Evening	37,8	42	41,4	41,4	18,95	524,42	125,27
2019/05/26	CH2205	Male	Three y/o	Treatment (Wk 2)	Treatment	Fast Day	No	18:05	18:00 - 18:59	Evening	37,9	39	17,0	17,0	19,05	517,82	94,77
2019/05/26	CH2206	Male	Three y/o	Treatment (Wk 2)	Treatment	Fast Day	No	18:05	18:00 - 18:59	Evening	37,8	44	36,4	36,4	18,95	528,51	120,84
2019/05/26	CH2205	Male	Three y/o	Treatment (Wk 2)	Treatment	Fast Day	No	18:10	18:00 - 18:59	Evening	37,7	43	10,6	10,6	18,85	526,50	78,73
2019/05/26	CH2206	Male	Three y/o	Treatment (Wk 2)	Treatment	Fast Day	No	18:10	18:00 - 18:59	Evening	37,8	46	36,0	36,0	18,95	532,38	120,46
2019/05/26	CH2205	Male	Three y/o	Treatment (Wk 2)	Treatment	Fast Day	No	18:15	18:00 - 18:59	Evening	37,6	47	13,8	13,8	18,75	534,23	87,68
2019/05/26	CH2206	Male	Three y/o	Treatment (Wk 2)	Treatment	Fast Day	No	18:15	18:00 - 18:59	Evening	37,8	48	49,0	49,0	18,95	536,04	131,09
2019/05/26	CH2205	Male	Three y/o	Treatment (Wk 2)	Treatment	Fast Day	No	18:20	18:00 - 18:59	Evening	37,6	41	12,4	12,4	18,75	522,29	84,04
2019/05/26	CH2206	Male	Three y/o	Treatment (Wk 2)	Treatment	Fast Day	No	18:20	18:00 - 18:59	Evening	37,8	45	41,0	41,0	18,95	530,47	124,94
2019/05/26	CH2205	Male	Three y/o	Treatment (Wk 2)	Treatment	Fast Day	No	18:25	18:00 - 18:59	Evening	37,5	69	11,4	11,4	18,65	565,86	81,19
2019/05/26	CH2206	Male	Three y/o	Treatment (Wk 2)	Treatment	Fast Day	No	18:25	18:00 - 18:59	Evening	37,7	50	46,2	46,2	18,85	539,52	129,06
2019/05/26	CH2205	Male	Three y/o	Treatment (Wk 2)	Treatment	Fast Day	No	18:30	18:00 - 18:59	Evening	37,5	41	9,8	9,8	18,65	522,29	76,07
2019/05/26	CH2206	Male	Three y/o	Treatment (Wk 2)	Treatment	Fast Day	No	18:30	18:00 - 18:59	Evening	37,7	40	54,0	54,0	18,85	520,09	134,45
2019/05/26	CH2205	Male	Three y/o	Treatment (Wk 2)	Treatment	Fast Day	No	18:35	18:00 - 18:59	Evening	37,4	52	14,8	14,8	18,55	542,83	90,05
2019/05/26	CH2206	Male	Three y/o	Treatment (Wk 2)	Treatment	Fast Day	No	18:35	18:00 - 18:59	Evening	37,7	33	58,0	58,0	18,85	502,51	136,92
2019/05/26	CH2205	Male	Three y/o	Treatment (Wk 2)	Treatment	Fast Day	No	18:40	18:00 - 18:59	Evening	37,4	32	11,2	11,2	18,55	499,63	80,59
2019/05/26	CH2206	Male	Three y/o	Treatment (Wk 2)	Treatment	Fast Day	No	18:40	18:00 - 18:59	Evening	37,5	44	59,4	59,4	18,65	528,51	137,75
2019/05/26	CH2205	Male	Three y/o	Treatment (Wk 2)	Treatment	Fast Day	No	18:45	18:00 - 18:59	Evening	37,3		11,2	11,2	18,46		80,59

Dataset S1: LOGGER

2019/05/26	CH2206	Male	Three y/o	Treatment (Wk 2)	Treatment	Fast Day	No	18:45	18:00 - 18:59	Evening	37,5	45	56,8	56,8	18,65	530,47	136,20
2019/05/26	CH2205	Male	Three y/o	Treatment (Wk 2)	Treatment	Fast Day	No	18:50	18:00 - 18:59	Evening	37,4	43	5,8	5,8	18,55	526,50	58,39
2019/05/26	CH2206	Male	Three y/o	Treatment (Wk 2)	Treatment	Fast Day	No	18:50	18:00 - 18:59	Evening	37,7	39	55,8	55,8	18,85	517,82	135,58
2019/05/26	CH2205	Male	Three y/o	Treatment (Wk 2)	Treatment	Fast Day	No	18:55	18:00 - 18:59	Evening	37,5		10,6	10,6	18,65		78,73
2019/05/26	CH2206	Male	Three y/o	Treatment (Wk 2)	Treatment	Fast Day	No	18:55	18:00 - 18:59	Evening	37,6	49	59,4	59,4	18,75	537,80	137,75
2019/05/26	CH2205	Male	Three y/o	Treatment (Wk 2)	Treatment	Fast Day	No	19:00	19:00 - 19:59	Evening	37,5	65	6,8	6,8	18,65	561,12	63,74
2019/05/26	CH2206	Male	Three y/o	Treatment (Wk 2)	Treatment	Fast Day	No	19:00	19:00 - 19:59	Evening	37,7	78	61,0	61,0	18,85	575,38	138,67
2019/05/26	CH2205	Male	Three y/o	Treatment (Wk 2)	Treatment	Fast Day	No	19:05	19:00 - 19:59	Evening	37,5	60	10,6	10,6	18,65	554,67	78,73
2019/05/26	CH2206	Male	Three y/o	Treatment (Wk 2)	Treatment	Fast Day	No	19:05	19:00 - 19:59	Evening	37,7	39	65,6	65,6	18,85	517,82	141,19
2019/05/26	CH2205	Male	Three y/o	Treatment (Wk 2)	Treatment	Fast Day	No	19:10	19:00 - 19:59	Evening	37,5		14,2	14,2	18,65		88,65
2019/05/26	CH2206	Male	Three y/o	Treatment (Wk 2)	Treatment	Fast Day	No	19:10	19:00 - 19:59	Evening	37,7	48	66,6	66,6	18,85	536,04	141,71
2019/05/26	CH2205	Male	Three y/o	Treatment (Wk 2)	Treatment	Fast Day	No	19:15	19:00 - 19:59	Evening	37,5	50	11,2	11,2	18,65	539,52	80,59
2019/05/26	CH2206	Male	Three y/o	Treatment (Wk 2)	Treatment	Fast Day	No	19:15	19:00 - 19:59	Evening	37,7	75	64,8	64,8	18,85	572,36	140,76
2019/05/26	CH2205	Male	Three y/o	Treatment (Wk 2)	Treatment	Fast Day	No	19:20	19:00 - 19:59	Evening	37,5		11,0	11,0	18,65		79,98
2019/05/26	CH2206	Male	Three y/o	Treatment (Wk 2)	Treatment	Fast Day	No	19:20	19:00 - 19:59	Evening	37,7	49	62,8	62,8	18,85	537,80	139,67
2019/05/26	CH2205	Male	Three y/o	Treatment (Wk 2)	Treatment	Fast Day	No	19:25	19:00 - 19:59	Evening	37,5	39	6,8	6,8	18,65	517,82	63,74
2019/05/26	CH2206	Male	Three y/o	Treatment (Wk 2)	Treatment	Fast Day	No	19:25	19:00 - 19:59	Evening	37,7	41	63,4	63,4	18,85	522,29	140,00
2019/05/26	CH2205	Male	Three y/o	Treatment (Wk 2)	Treatment	Fast Day	No	19:30	19:00 - 19:59	Evening	37,5	40	6,8	6,8	18,65	520,09	63,74
2019/05/26	CH2206	Male	Three y/o	Treatment (Wk 2)	Treatment	Fast Day	No	19:30	19:00 - 19:59	Evening	37,7	48	69,4	69,4	18,85	536,04	143,14
2019/05/26	CH2205	Male	Three y/o	Treatment (Wk 2)	Treatment	Fast Day	No	19:35	19:00 - 19:59	Evening	37,5	57	10,6	10,6	18,65	550,47	78,73
2019/05/26	CH2206	Male	Three y/o	Treatment (Wk 2)	Treatment	Fast Day	No	19:35	19:00 - 19:59	Evening	37,7	48	63,6	63,6	18,85	536,04	140,11
2019/05/26	CH2205	Male	Three y/o	Treatment (Wk 2)	Treatment	Fast Day	No	19:40	19:00 - 19:59	Evening	37,4	42	9,8	9,8	18,55	524,42	76,07
2019/05/26	CH2206	Male	Three y/o	Treatment (Wk 2)	Treatment	Fast Day	No	19:40	19:00 - 19:59	Evening	37,7	41	64,4	64,4	18,85	522,29	140,55
2019/05/26	CH2205	Male	Three y/o	Treatment (Wk 2)	Treatment	Fast Day	No	19:45	19:00 - 19:59	Evening	37,4	45	7,4	7,4	18,55	530,47	66,59
2019/05/26	CH2206	Male	Three y/o	Treatment (Wk 2)	Treatment	Fast Day	No	19:45	19:00 - 19:59	Evening	37,7	51	64,6	64,6	18,85	541,20	140,65
2019/05/26	CH2205	Male	Three y/o	Treatment (Wk 2)	Treatment	Fast Day	No	19:50	19:00 - 19:59	Evening	37,4		17,2	17,2	18,55		95,17
2019/05/26	CH2206	Male	Three y/o	Treatment (Wk 2)	Treatment	Fast Day	No	19:50	19:00 - 19:59	Evening	37,7	48	63,0	63,0	18,85	536,04	139,78
2019/05/26	CH2205	Male	Three y/o	Treatment (Wk 2)	Treatment	Fast Day	No	19:55	19:00 - 19:59	Evening	37,4		21,0	21,0	18,55		101,98
2019/05/26	CH2206	Male	Three y/o	Treatment (Wk 2)	Treatment	Fast Day	No	19:55	19:00 - 19:59	Evening	37,7	42	65,2	65,2	18,85	524,42	140,97
2019/05/26	CH2205	Male	Three y/o	Treatment (Wk 2)	Treatment	Fast Day	No	20:00	20:00 - 20:59	Night	37,4		23,4	23,4	18,55		105,68
2019/05/26	CH2206	Male	Three y/o	Treatment (Wk 2)	Treatment	Fast Day	No	20:00	20:00 - 20:59	Night	37,7	41	56,6	56,6	18,85	522,29	136,07
2019/05/26	CH2205	Male	Three y/o	Treatment (Wk 2)	Treatment	Fast Day	No	20:05	20:00 - 20:59	Night	37,4	43	25,4	25,4	18,55	526,50	108,49
2019/05/26	CH2206	Male	Three y/o	Treatment (Wk 2)	Treatment	Fast Day	No	20:05	20:00 - 20:59	Night	37,7	83	34,0	34,0	18,85	580,11	118,49
2019/05/26	CH2205	Male	Three y/o	Treatment (Wk 2)	Treatment	Fast Day	No	20:10	20:00 - 20:59	Night	37,5	42	26,6	26,6	18,65	524,42	110,07
2019/05/26	CH2206	Male	Three y/o	Treatment (Wk 2)	Treatment	Fast Day	No	20:10	20:00 - 20:59	Night	37,5	43	29,6	29,6	18,65	526,50	113,73
2019/05/26	CH2205	Male	Three y/o	Treatment (Wk 2)	Treatment	Fast Day	No	20:15	20:00 - 20:59	Night	37,5	44	25,4	25,4	18,65	528,51	108,49
2019/05/26	CH2206	Male	Three y/o	Treatment (Wk 2)	Treatment	Fast Day	No	20:15	20:00 - 20:59	Night	37,2	44	43,4	43,4	18,36	528,51	126,90
2019/05/26	CH2205	Male	Three y/o	Treatment (Wk 2)	Treatment	Fast Day	No	20:20	20:00 - 20:59	Night	37,5	43	29,6	29,6	18,65	526,50	113,73
2019/05/26	CH2206	Male	Three y/o	Treatment (Wk 2)	Treatment	Fast Day	No	20:20	20:00 - 20:59	Night	37,0	48	52,0	52,0	18,16	536,04	133,14
2019/05/26	CH2205	Male	Three y/o	Treatment (Wk 2)	Treatment	Fast Day	No	20:25	20:00 - 20:59	Night	37,6	47	28,8	28,8	18,75	534,23	112,79
2019/05/26	CH2206	Male	Three y/o	Treatment (Wk 2)	Treatment	Fast Day	No	20:25	20:00 - 20:59	Night	37,0	43	48,0	48,0	18,16	526,50	130,38
2019/05/26	CH2205	Male	Three y/o	Treatment (Wk 2)	Treatment	Fast Day	No	20:30	20:00 - 20:59	Night	37,6	39	27,8	27,8	18,75	517,82	111,58
2019/05/26	CH2206	Male	Three y/o	Treatment (Wk 2)	Treatment	Fast Day	No	20:30	20:00 - 20:59	Night	37,1	44	44,8	44,8	18,26	528,51	127,99
2019/05/26	CH2205	Male	Three y/o	Treatment (Wk 2)	Treatment	Fast Day	No	20:35	20:00 - 20:59	Night	37,6	50	21,2	21,2	18,75	539,52	102,31
2019/05/26	CH2206	Male	Three y/o	Treatment (Wk 2)	Treatment	Fast Day	No	20:35	20:00 - 20:59	Night	37,1	43	40,2	40,2	18,26	526,50	124,26
2019/05/26	CH2205	Male	Three y/o	Treatment (Wk 2)	Treatment	Fast Day	No	20:40	20:00 - 20:59	Night	37,6	43	17,0	17,0	18,75	526,50	94,77
2019/05/26	CH2206	Male	Three y/o	Treatment (Wk 2)	Treatment	Fast Day	No	20:40	20:00 - 20:59	Night	37,2	49	29,4	29,4	18,36	537,80	113,50
2019/05/26	CH2205	Male	Three y/o	Treatment (Wk 2)	Treatment	Fast Day	No	20:45	20:00 - 20:59	Night	37,6	61	20,8	20,8	18,75	556,01	101,65
2019/05/26	CH2206	Male	Three y/o	Treatment (Wk 2)	Treatment	Fast Day	No	20:45	20:00 - 20:59	Night	37,3	49	11,0	11,0	18,46	537,80	79,98
2019/05/26	CH2205	Male	Three y/o	Treatment (Wk 2)	Treatment	Fast Day	No	20:50	20:00 - 20:59	Night	37,7	46	15,6	15,6	18,85	532,38	91,85
2019/05/26	CH2206	Male	Three y/o	Treatment (Wk 2)	Treatment	Fast Day	No	20:50	20:00 - 20:59	Night	37,4	39	13,6	13,6	18,55	517,82	87,18
2019/05/26	CH2205	Male	Three y/o	Treatment (Wk 2)	Treatment	Fast Day	No	20:55	20:00 - 20:59	Night	37,7	68	19,8	19,8	18,85	564,71	99,97
2019/05/26	CH2206	Male	Three y/o	Treatment (Wk 2)	Treatment	Fast Day	No	20:55	20:00 - 20:59	Night	37,4	81	8,8	8,8	18,55	578,26	72,43
2019/05/26	CH2205	Male	Three y/o	Treatment (Wk 2)	Treatment	Fast Day	No	21:00	21:00 - 21:59	Night	37,7	47	19,4	19,4	18,85	534,23	99,28
2019/05/26	CH2206	Male	Three y/o	Treatment (Wk 2)	Treatment	Fast Day	No	21:00	21:00 - 21:59	Night	37,5	84	6,4	6,4	18,65	581,01	61,70
2019/05/26	CH2205	Male	Three y/o	Treatment (Wk 2)	Treatment	Fast Day	No	21:05	21:00 - 21:59	Night	37,7	44	21,0	21,0	18,85	528,51	101,98
2019/05/26	CH2206	Male	Three y/o	Treatment (Wk 2)	Treatment	Fast Day	No	21:05	21:00 - 21:59	Night	37,5	41	6,0	6,0	18,65	522,29	59,53
2019/05/26	CH2205	Male	Three y/o	Treatment (Wk 2)	Treatment	Fast Day	No	21:10	21:00 - 21:59	Night	37,7	65	22,0	22,0	18,85	561,12	103,57
2019/05/26	CH2206	Male	Three y/o	Treatment (Wk 2)	Treatment	Fast Day	No	21:10	21:00 - 21:59	Night	37,7	94	9,6	9,6	18,85	589,37	75,37
2019/05/26	CH2205	Male	Three y/o	Treatment (Wk 2)	Treatment	Fast Day	No	21:15	21:00 - 21:59	Night	37,7	40	28,0	28,0	18,85	520,09	111,83
2019/05/26	CH2206	Male	Three y/o	Treatment (Wk 2)	Treatment	Fast Day	No	21:15	21:00 - 21:59	Night	37,7	47	14,2	14,2	18,85	534,23	88,65
2019/05/26	CH2205	Male	Three y/o	Treatment (Wk 2)	Treatment	Fast Day	No	21:20	21:00 - 21:59	Night	37,7	47	29,8	29,8	18,85	534,23	113,97
2019/05/26	CH2206	Male	Three y/o	Treatment (Wk 2)	Treatment	Fast Day	No	21:20	21:00 - 21:59	Night	37,7	43	9,2	9,2	18,85	526,50	73,94
2019/05/26	CH2205	Male	Three y/o	Treatment (Wk 2)	Treatment	Fast Day	No	21:25	21:00 - 21:59	Night	37,7		32,6	32,6	18,85		117,05
2019/05/26	CH2206	Male	Three y/o	Treatment (Wk 2)	Treatment	Fast Day	No	21:25	21:00 - 21:59	Night	37,7	48	17,2	17,2	18,85	536,04	95,17
2019/05/26	CH2205	Male	Three y/o	Treatment (Wk 2)	Treatment	Fast Day	No	21:30	21:00 - 21:59	Night	37,7	46	26,8	26,8	18,85	532,38	110,33
2019/05/26	CH2206	Male	Three y/o	Treatment (Wk 2)	Treatment	Fast Day	No	21:30	21:00 - 21:59	Night	37,7	53	22,8	22,8	18,85	544,43	104,79
2019/05/26	CH2205	Male	Three y/o	Treatment (Wk 2)	Treatment	Fast Day	No	21:35	21:00 - 21:59	Night	37,7	46	25,6	25,6	18,85	532,38	108,76
2019/05/26	CH2206	Male	Three y/o	Treatment (Wk 2)	Treatment	Fast Day	No	21:35	21:00 - 21:59	Night	37,7	41	9,8	9,8	18,85	522,29	76,07
2019/05/26	CH2205	Male	Three y/o	Treatment (Wk 2)	Treatment	Fast Day	No	21:40	21:00 - 21:59	Night	37,7		27,0	27,0	18,85		110,58

	2019/05/26	CH2206	Male	Three y/o	Treatment (Wk 2)	Treatment	Fast Day	No	21:40	21:00 - 21:59	Night	37,7	59	23,6	23,6	18,85	553,30	105,97
	2019/05/26	CH2205	Male	Three y/o	Treatment (Wk 2)	Treatment	Fast Day	No	21:45	21:00 - 21:59	Night	37,6	44	28,6	28,6	18,75	528,51	112,56
	2019/05/26	CH2206	Male	Three y/o	Treatment (Wk 2)	Treatment	Fast Day	No	21:45	21:00 - 21:59	Night	37,7	47	25,8	25,8	18,85	534,23	109,02
	2019/05/26	CH2205	Male	Three y/o	Treatment (Wk 2)	Treatment	Fast Day	No	21:50	21:00 - 21:59	Night	37,6	41	23,8	23,8	18,75	522,29	106,26
	2019/05/26	CH2206	Male	Three y/o	Treatment (Wk 2)	Treatment	Fast Day	No	21:50	21:00 - 21:59	Night	37,7	76	26,6	26,6	18,85	573,39	110,07
	2019/05/26	CH2205	Male	Three y/o	Treatment (Wk 2)	Treatment	Fast Day	No	21:55	21:00 - 21:59	Night	37,7	42	20,2	20,2	18,85	524,42	100,65
	2019/05/26	CH2206	Male	Three y/o	Treatment (Wk 2)	Treatment	Fast Day	No	21:55	21:00 - 21:59	Night	37,7	47	26,0	26,0	18,85	534,23	109,29
	2019/05/26	CH2205	Male	Three y/o	Treatment (Wk 2)	Treatment	Fast Day	No	22:00	22:00 - 22:59	Night	37,7	62	24,4	24,4	18,85	557,33	107,11
	2019/05/26	CH2206	Male	Three y/o	Treatment (Wk 2)	Treatment	Fast Day	No	22:00	22:00 - 22:59	Night	37,7	84	25,6	25,6	18,85	581,01	108,76
	2019/05/26	CH2205	Male	Three y/o	Treatment (Wk 2)	Treatment	Fast Day	No	22:05	22:00 - 22:59	Night	37,7	51	24,8	24,8	18,85	541,20	107,67
	2019/05/26	CH2206	Male	Three y/o	Treatment (Wk 2)	Treatment	Fast Day	No	22:05	22:00 - 22:59	Night	37,7	49	21,8	21,8	18,85	537,80	103,26
	2019/05/26	CH2205	Male	Three y/o	Treatment (Wk 2)	Treatment	Fast Day	No	22:10	22:00 - 22:59	Night	37,7	64	23,6	23,6	18,85	559,88	105,97
	2019/05/26	CH2206	Male	Three y/o	Treatment (Wk 2)	Treatment	Fast Day	No	22:10	22:00 - 22:59	Night	37,7	56	22,6	22,6	18,85	549,01	104,49
	2019/05/26	CH2205	Male	Three y/o	Treatment (Wk 2)	Treatment	Fast Day	No	22:15	22:00 - 22:59	Night	37,7	61	12,4	12,4	18,85	556,01	84,04
	2019/05/26	CH2206	Male	Three y/o	Treatment (Wk 2)	Treatment	Fast Day	No	22:15	22:00 - 22:59	Night	37,7	68	41,2	41,2	18,85	564,71	125,11
	2019/05/26	CH2205	Male	Three y/o	Treatment (Wk 2)	Treatment	Fast Day	No	22:20	22:00 - 22:59	Night	37,7	40	2,4	2,4	18,85	520,09	28,91
	2019/05/26	CH2206	Male	Three y/o	Treatment (Wk 2)	Treatment	Fast Day	No	22:20	22:00 - 22:59	Night	37,7	69	45,8	45,8	18,85	565,86	128,76
	2019/05/26	CH2205	Male	Three y/o	Treatment (Wk 2)	Treatment	Fast Day	No	22:25	22:00 - 22:59	Night	37,7	45	6,6	6,6	18,85	530,47	62,73
	2019/05/26	CH2206	Male	Three y/o	Treatment (Wk 2)	Treatment	Fast Day	No	22:25	22:00 - 22:59	Night	37,5	57	39,6	39,6	18,65	550,47	123,74
	2019/05/26	CH2205	Male	Three y/o	Treatment (Wk 2)	Treatment	Fast Day	No	22:30	22:00 - 22:59	Night	37,7	57	5,4	5,4	18,85	550,47	55,99
	2019/05/26	CH2206	Male	Three y/o	Treatment (Wk 2)	Treatment	Fast Day	No	22:30	22:00 - 22:59	Night	37,5	49	43,0	43,0	18,65	537,80	126,58
	2019/05/26	CH2205	Male	Three y/o	Treatment (Wk 2)	Treatment	Fast Day	No	22:35	22:00 - 22:59	Night	37,7	39	3,0	3,0	18,85	517,82	36,34
	2019/05/26	CH2206	Male	Three y/o	Treatment (Wk 2)	Treatment	Fast Day	No	22:35	22:00 - 22:59	Night	37,5	47	47,2	47,2	18,65	534,23	129,80
	2019/05/26	CH2205	Male	Three y/o	Treatment (Wk 2)	Treatment	Fast Day	No	22:40	22:00 - 22:59	Night	37,7	45	25,4	25,4	18,85	530,47	108,49
	2019/05/26	CH2206	Male	Three y/o	Treatment (Wk 2)	Treatment	Fast Day	No	22:40	22:00 - 22:59	Night	37,5	46	42,6	42,6	18,65	532,38	126,26
	2019/05/26	CH2205	Male	Three y/o	Treatment (Wk 2)	Treatment	Fast Day	No	22:45	22:00 - 22:59	Night	37,6	45	63,8	63,8	18,75	530,47	140,22
	2019/05/26	CH2206	Male	Three y/o	Treatment (Wk 2)	Treatment	Fast Day	No	22:45	22:00 - 22:59	Night	37,5	77	54,2	54,2	18,65	574,39	134,57
	2019/05/26	CH2205	Male	Three y/o	Treatment (Wk 2)	Treatment	Fast Day	No	22:50	22:00 - 22:59	Night	37,6	53	34,6	34,6	18,75	544,43	119,10
	2019/05/26	CH2206	Male	Three y/o	Treatment (Wk 2)	Treatment	Fast Day	No	22:50	22:00 - 22:59	Night	37,5	80	59,8	59,8	18,65	577,31	137,98
	2019/05/26	CH2205	Male	Three y/o	Treatment (Wk 2)	Treatment	Fast Day	No	22:55	22:00 - 22:59	Night	37,5		33,2	33,2	18,65		117,68
	2019/05/26	CH2206	Male	Three y/o	Treatment (Wk 2)	Treatment	Fast Day	No	22:55	22:00 - 22:59	Night	37,5	45	58,8	58,8	18,65	530,47	137,39
	2019/05/26	CH2205	Male	Three y/o	Treatment (Wk 2)	Treatment	Fast Day	No	23:00	23:00 - 23:59	Night	37,4		35,0	35,0	18,55		119,49
	2019/05/26	CH2206	Male	Three y/o	Treatment (Wk 2)	Treatment	Fast Day	No	23:00	23:00 - 23:59	Night	37,5	44	56,6	56,6	18,65	528,51	136,07
	2019/05/26	CH2205	Male	Three y/o	Treatment (Wk 2)	Treatment	Fast Day	No	23:05	23:00 - 23:59	Night	37,4		33,2	33,2	18,55		117,68
	2019/05/26	CH2206	Male	Three y/o	Treatment (Wk 2)	Treatment	Fast Day	No	23:05	23:00 - 23:59	Night	37,5	46	59,8	59,8	18,65	532,38	137,98
	2019/05/26	CH2205	Male	Three y/o	Treatment (Wk 2)	Treatment	Fast Day	No	23:10	23:00 - 23:59	Night	37,4		37,0	37,0	18,55		121,40
	2019/05/26	CH2206	Male	Three y/o	Treatment (Wk 2)	Treatment	Fast Day	No	23:10	23:00 - 23:59	Night	37,5	37	57,4	57,4	18,65	513,06	136,56
	2019/05/26	CH2205	Male	Three y/o	Treatment (Wk 2)	Treatment	Fast Day	No	23:15	23:00 - 23:59	Night	37,4		34,6	34,6	18,55		119,10
	2019/05/26	CH2206	Male	Three y/o	Treatment (Wk 2)	Treatment	Fast Day	No	23:15	23:00 - 23:59	Night	37,4	46	55,2	55,2	18,55	532,38	135,21
	2019/05/26	CH2205	Male	Three y/o	Treatment (Wk 2)	Treatment	Fast Day	No	23:20	23:00 - 23:59	Night	37,4		30,2	30,2	18,55		114,42
	2019/05/26	CH2206	Male	Three y/o	Treatment (Wk 2)	Treatment	Fast Day	No	23:20	23:00 - 23:59	Night	37,4	51	56,6	56,6	18,55	541,20	136,07
	2019/05/26	CH2205	Male	Three y/o	Treatment (Wk 2)	Treatment	Fast Day	No	23:25	23:00 - 23:59	Night	37,4		30,4	30,4	18,55		114,65
	2019/05/26	CH2206	Male	Three y/o	Treatment (Wk 2)	Treatment	Fast Day	No	23:25	23:00 - 23:59	Night	37,4	48	61,4	61,4	18,55	536,04	138,89
	2019/05/26	CH2205	Male	Three y/o	Treatment (Wk 2)	Treatment	Fast Day	No	23:30	23:00 - 23:59	Night	37,4	53	19,4	19,4	18,55	544,43	99,28
	2019/05/26	CH2206	Male	Three y/o	Treatment (Wk 2)	Treatment	Fast Day	No	23:30	23:00 - 23:59	Night	37,4	48	58,0	58,0	18,55	536,04	136,92
	2019/05/26	CH2205	Male	Three y/o	Treatment (Wk 2)	Treatment	Fast Day	No	23:35	23:00 - 23:59	Night	37,4	49	16,6	16,6	18,55	537,80	93,96
	2019/05/26	CH2206	Male	Three y/o	Treatment (Wk 2)	Treatment	Fast Day	No	23:35	23:00 - 23:59	Night	37,4	44	57,6	57,6	18,55	528,51	136,68
	2019/05/26	CH2205	Male	Three y/o	Treatment (Wk 2)	Treatment	Fast Day	No	23:40	23:00 - 23:59	Night	37,4	50	20,8	20,8	18,55	539,52	101,65
	2019/05/26	CH2206	Male	Three y/o	Treatment (Wk 2)	Treatment	Fast Day	No	23:40	23:00 - 23:59	Night	37,4	40	50,2	50,2	18,55	520,09	131,92
	2019/05/26	CH2205	Male	Three y/o	Treatment (Wk 2)	Treatment	Fast Day	No	23:45	23:00 - 23:59	Night	37,4	45	20,0	20,0	18,55	530,47	100,31
	2019/05/26	CH2206	Male	Three y/o	Treatment (Wk 2)	Treatment	Fast Day	No	23:45	23:00 - 23:59	Night	37,4	75	50,6	50,6	18,55	572,36	132,20
	2019/05/26	CH2205	Male	Three y/o	Treatment (Wk 2)	Treatment	Fast Day	No	23:50	23:00 - 23:59	Night	37,4	44	22,0	22,0	18,55	528,51	103,57
	2019/05/26	CH2206	Male	Three y/o	Treatment (Wk 2)	Treatment	Fast Day	No	23:50	23:00 - 23:59	Night	37,4	46	51,8	51,8	18,55	532,38	133,01
	2019/05/26	CH2205	Male	Three y/o	Treatment (Wk 2)	Treatment	Fast Day	No	23:55	23:00 - 23:59	Night	37,4	48	22,6	22,6	18,55	536,04	104,49
	2019/05/26	CH2206	Male	Three y/o	Treatment (Wk 2)	Treatment	Fast Day	No	23:55	23:00 - 23:59	Night	37,4	51	59,2	59,2	18,55	541,20	137,63
13	2019/05/27	CH2205	Male	Three y/o	Treatment (Wk 2)	Treatment	Feed Day	No	00:00	00:00 - 00:59	Early Morning	37,4	53	24,2	24,2	18,55	544,43	106,83
	2019/05/27	CH2206	Male	Three y/o	Treatment (Wk 2)	Treatment	Feed Day	No	00:00	00:00 - 00:59	Early Morning	37,4	63	62,6	62,6	18,55	558,62	139,56
	2019/05/27	CH2205	Male	Three y/o	Treatment (Wk 2)	Treatment	Feed Day	No	00:05	00:00 - 00:59	Early Morning	37,4	46	19,8	19,8	18,55	532,38	99,97
	2019/05/27	CH2206	Male	Three y/o	Treatment (Wk 2)	Treatment	Feed Day	No	00:05	00:00 - 00:59	Early Morning	37,4	51	45,2	45,2	18,55	541,20	128,30
	2019/05/27	CH2205	Male	Three y/o	Treatment (Wk 2)	Treatment	Feed Day	No	00:10	00:00 - 00:59	Early Morning	37,5	67	20,6	20,6	18,65	563,53	101,32
	2019/05/27	CH2206	Male	Three y/o	Treatment (Wk 2)	Treatment	Feed Day	No	00:10	00:00 - 00:59	Early Morning	37,4	41	41,4	41,4	18,55	522,29	125,27
	2019/05/27	CH2205	Male	Three y/o	Treatment (Wk 2)	Treatment	Feed Day	No	00:15	00:00 - 00:59	Early Morning	37,5	45	22,2	22,2	18,65	530,47	103,88
	2019/05/27	CH2206	Male	Three y/o	Treatment (Wk 2)	Treatment	Feed Day	No	00:15	00:00 - 00:59	Early Morning	37,4	89	34,6	34,6	18,55	585,33	119,10
	2019/05/27	CH2205	Male	Three y/o	Treatment (Wk 2)	Treatment	Feed Day	No	00:20	00:00 - 00:59	Early Morning	37,5	45	19,8	19,8	18,65	530,47	99,97
	2019/05/27	CH2206	Male	Three y/o	Treatment (Wk 2)	Treatment	Feed Day	No	00:20	00:00 - 00:59	Early Morning	37,4	43	37,4	37,4	18,55	526,50	121,77
	2019/05/27	CH2205	Male	Three y/o	Treatment (Wk 2)	Treatment	Feed Day	No	00:25	00:00 - 00:59	Early Morning	37,5	46	31,6	31,6	18,65	532,38	115,98
	2019/05/27	CH2206	Male	Three y/o	Treatment (Wk 2)	Treatment	Feed Day	No	00:25	00:00 - 00:59	Early Morning	37,4	44	70,2	70,2	18,55	528,51	143,54
	2019/05/27	CH2205	Male	Three y/o	Treatment (Wk 2)	Treatment	Feed Day	No	00:30	00:00 - 00:59	Early Morning	37,5	123	59,0	59,0	18,65	608,49	137,51
	2019/05/27	CH2206	Male	Three y/o	Treatment (Wk 2)	Treatment	Feed Day	No	00:30	00:00 - 00:59	Early Morning	37,5	135	79,8	79,8	18,65	614,84	147,99
	2019/05/27	CH2205	Male	Three y/o	Treatment (Wk 2)	Treatment	Feed Day	No	00:35	00:00 - 00:59	Early Morning	37,5	54	121,6	121,6	18,65	545,99	162,67

2019/05/27	CH2206	Male	Three y/o	Treatment (Wk 2)	Treatment	Feed Day	No	00:35	00:00 - 00:59	Early Morning	37,5	129	71,6	71,6	18,65	611,75	144,22
2019/05/27	CH2205	Male	Three y/o	Treatment (Wk 2)	Treatment	Feed Day	No	00:40	00:00 - 00:59	Early Morning	37,5	72	45,6	45,6	18,65	569,20	128,61
2019/05/27	CH2206	Male	Three y/o	Treatment (Wk 2)	Treatment	Feed Day	No	00:40	00:00 - 00:59	Early Morning	37,5	97	45,4	45,4	18,65	591,66	128,45
2019/05/27	CH2205	Male	Three y/o	Treatment (Wk 2)	Treatment	Feed Day	No	00:45	00:00 - 00:59	Early Morning	37,4	73	24,2	24,2	18,55	570,27	106,83
2019/05/27	CH2206	Male	Three y/o	Treatment (Wk 2)	Treatment	Feed Day	No	00:45	00:00 - 00:59	Early Morning	37,5	112	28,2	28,2	18,65	601,96	112,07
2019/05/27	CH2205	Male	Three y/o	Treatment (Wk 2)	Treatment	Feed Day	No	00:50	00:00 - 00:59	Early Morning	37,4		11,8	11,8	18,55		82,36
2019/05/27	CH2206	Male	Three y/o	Treatment (Wk 2)	Treatment	Feed Day	No	00:50	00:00 - 00:59	Early Morning	37,4	45	59,8	59,8	18,55	530,47	137,98
2019/05/27	CH2205	Male	Three y/o	Treatment (Wk 2)	Treatment	Feed Day	No	00:55	00:00 - 00:59	Early Morning	37,4	160	22,8	22,8	18,55	626,07	104,79
2019/05/27	CH2206	Male	Three y/o	Treatment (Wk 2)	Treatment	Feed Day	No	00:55	00:00 - 00:59	Early Morning	37,3	60	60,2	60,2	18,46	554,67	138,21
2019/05/27	CH2205	Male	Three y/o	Treatment (Wk 2)	Treatment	Feed Day	No	01:00	01:00 - 01:59	Early Morning	37,5		25,2	25,2	18,65		108,22
2019/05/27	CH2206	Male	Three y/o	Treatment (Wk 2)	Treatment	Feed Day	No	01:00	01:00 - 01:59	Early Morning	37,2	55	62,6	62,6	18,36	547,52	139,56
2019/05/27	CH2205	Male	Three y/o	Treatment (Wk 2)	Treatment	Feed Day	No	01:05	01:00 - 01:59	Early Morning	37,3	101	17,8	17,8	18,46	594,59	96,34
2019/05/27	CH2206	Male	Three y/o	Treatment (Wk 2)	Treatment	Feed Day	No	01:05	01:00 - 01:59	Early Morning	37,2		80,2	80,2	18,36		148,16
2019/05/27	CH2205	Male	Three y/o	Treatment (Wk 2)	Treatment	Feed Day	No	01:10	01:00 - 01:59	Early Morning	37,3	45	21,2	21,2	18,46	530,47	102,31
2019/05/27	CH2206	Male	Three y/o	Treatment (Wk 2)	Treatment	Feed Day	No	01:10	01:00 - 01:59	Early Morning	37,3	79	56,2	56,2	18,46	576,36	135,83
2019/05/27	CH2205	Male	Three y/o	Treatment (Wk 2)	Treatment	Feed Day	No	01:15	01:00 - 01:59	Early Morning	37,3	45	16,0	16,0	18,46	530,47	92,71
2019/05/27	CH2206	Male	Three y/o	Treatment (Wk 2)	Treatment	Feed Day	No	01:15	01:00 - 01:59	Early Morning	37,2	47	59,0	59,0	18,36	534,23	137,51
2019/05/27	CH2205	Male	Three y/o	Treatment (Wk 2)	Treatment	Feed Day	No	01:20	01:00 - 01:59	Early Morning	37,4	41	11,6	11,6	18,55	522,29	81,78
2019/05/27	CH2206	Male	Three y/o	Treatment (Wk 2)	Treatment	Feed Day	No	01:20	01:00 - 01:59	Early Morning	37,3	51	46,4	46,4	18,46	541,20	129,21
2019/05/27	CH2205	Male	Three y/o	Treatment (Wk 2)	Treatment	Feed Day	No	01:25	01:00 - 01:59	Early Morning	37,4		140,0	140,0	18,55		167,60
2019/05/27	CH2206	Male	Three y/o	Treatment (Wk 2)	Treatment	Feed Day	No	01:25	01:00 - 01:59	Early Morning	37,3	107	112,4	112,4	18,46	598,73	159,92
2019/05/27	CH2205	Male	Three y/o	Treatment (Wk 2)	Treatment	Feed Day	No	01:30	01:00 - 01:59	Early Morning	37,5		191,8	191,8	18,65		178,65
2019/05/27	CH2206	Male	Three y/o	Treatment (Wk 2)	Treatment	Feed Day	No	01:30	01:00 - 01:59	Early Morning	37,4	98	170,6	170,6	18,55	592,41	174,53
2019/05/27	CH2205	Male	Three y/o	Treatment (Wk 2)	Treatment	Feed Day	No	01:35	01:00 - 01:59	Early Morning	37,4	177	78,4	78,4	18,55	632,55	147,37
2019/05/27	CH2206	Male	Three y/o	Treatment (Wk 2)	Treatment	Feed Day	No	01:35	01:00 - 01:59	Early Morning	37,4	111	49,0	49,0	18,55	601,33	131,09
2019/05/27	CH2205	Male	Three y/o	Treatment (Wk 2)	Treatment	Feed Day	No	01:40	01:00 - 01:59	Early Morning	37,5	104	24,2	24,2	18,65	596,69	106,83
2019/05/27	CH2206	Male	Three y/o	Treatment (Wk 2)	Treatment	Feed Day	No	01:40	01:00 - 01:59	Early Morning	37,3	49	44,2	44,2	18,46	537,80	127,53
2019/05/27	CH2205	Male	Three y/o	Treatment (Wk 2)	Treatment	Feed Day	No	01:45	01:00 - 01:59	Early Morning	37,3	72	39,4	39,4	18,46	569,20	123,57
2019/05/27	CH2206	Male	Three y/o	Treatment (Wk 2)	Treatment	Feed Day	No	01:45	01:00 - 01:59	Early Morning	37,1	104	113,8	113,8	18,26	596,69	160,35
2019/05/27	CH2205	Male	Three y/o	Treatment (Wk 2)	Treatment	Feed Day	No	01:50	01:00 - 01:59	Early Morning	37,5	54	111,6	111,6	18,65	545,99	159,67
2019/05/27	CH2206	Male	Three y/o	Treatment (Wk 2)	Treatment	Feed Day	No	01:50	01:00 - 01:59	Early Morning	37,1	50	104,2	104,2	18,26	539,52	157,28
2019/05/27	CH2205	Male	Three y/o	Treatment (Wk 2)	Treatment	Feed Day	No	01:55	01:00 - 01:59	Early Morning	37,6	103	49,8	49,8	18,75	596,00	131,65
2019/05/27	CH2206	Male	Three y/o	Treatment (Wk 2)	Treatment	Feed Day	No	01:55	01:00 - 01:59	Early Morning	37,3	124	285,2	285,2	18,46	609,04	192,63
2019/05/27	CH2205	Male	Three y/o	Treatment (Wk 2)	Treatment	Feed Day	No	02:00	02:00 - 02:59	Early Morning	37,7		102,6	102,6	18,85		156,74
2019/05/27	CH2206	Male	Three y/o	Treatment (Wk 2)	Treatment	Feed Day	No	02:00	02:00 - 02:59	Early Morning	37,5	109	222,6	222,6	18,65	600,04	183,89
2019/05/27	CH2205	Male	Three y/o	Treatment (Wk 2)	Treatment	Feed Day	No	02:05	02:00 - 02:59	Early Morning	37,7	64	20,4	20,4	18,85	559,88	100,99
2019/05/27	CH2206	Male	Three y/o	Treatment (Wk 2)	Treatment	Feed Day	No	02:05	02:00 - 02:59	Early Morning	37,7	83	43,2	43,2	18,85	580,11	126,74
2019/05/27	CH2205	Male	Three y/o	Treatment (Wk 2)	Treatment	Feed Day	No	02:10	02:00 - 02:59	Early Morning	37,6	57	31,2	31,2	18,75	550,47	115,54
2019/05/27	CH2206	Male	Three y/o	Treatment (Wk 2)	Treatment	Feed Day	No	02:10	02:00 - 02:59	Early Morning	37,5	55	56,2	56,2	18,65	547,52	135,83
2019/05/27	CH2205	Male	Three y/o	Treatment (Wk 2)	Treatment	Feed Day	No	02:15	02:00 - 02:59	Early Morning	37,6	45	20,0	20,0	18,75	530,47	100,31
2019/05/27	CH2206	Male	Three y/o	Treatment (Wk 2)	Treatment	Feed Day	No	02:15	02:00 - 02:59	Early Morning	37,3	43	57,8	57,8	18,46	526,50	136,80
2019/05/27	CH2205	Male	Three y/o	Treatment (Wk 2)	Treatment	Feed Day	No	02:20	02:00 - 02:59	Early Morning	37,6	42	34,8	34,8	18,75	524,42	119,29
2019/05/27	CH2206	Male	Three y/o	Treatment (Wk 2)	Treatment	Feed Day	No	02:20	02:00 - 02:59	Early Morning	37,5	50	60,4	60,4	18,65	539,52	138,32
2019/05/27	CH2205	Male	Three y/o	Treatment (Wk 2)	Treatment	Feed Day	No	02:25	02:00 - 02:59	Early Morning	37,7	51	32,4	32,4	18,85	541,20	116,84
2019/05/27	CH2206	Male	Three y/o	Treatment (Wk 2)	Treatment	Feed Day	No	02:25	02:00 - 02:59	Early Morning	37,5	43	50,6	50,6	18,65	526,50	132,20
2019/05/27	CH2205	Male	Three y/o	Treatment (Wk 2)	Treatment	Feed Day	No	02:30	02:00 - 02:59	Early Morning	37,7		23,8	23,8	18,85		106,26
2019/05/27	CH2206	Male	Three y/o	Treatment (Wk 2)	Treatment	Feed Day	No	02:30	02:00 - 02:59	Early Morning	37,3	44	54,0	54,0	18,46	528,51	134,45
2019/05/27	CH2205	Male	Three y/o	Treatment (Wk 2)	Treatment	Feed Day	No	02:35	02:00 - 02:59	Early Morning	37,7		143,0	143,0	18,85		168,34
2019/05/27	CH2206	Male	Three y/o	Treatment (Wk 2)	Treatment	Feed Day	No	02:35	02:00 - 02:59	Early Morning	37,4	111	36,4	36,4	18,55	601,33	120,84
2019/05/27	CH2205	Male	Three y/o	Treatment (Wk 2)	Treatment	Feed Day	No	02:40	02:00 - 02:59	Early Morning	37,7		80,0	80,0	18,85		148,08
2019/05/27	CH2206	Male	Three y/o	Treatment (Wk 2)	Treatment	Feed Day	No	02:40	02:00 - 02:59	Early Morning	37,4	56	55,0	55,0	18,55	549,01	135,08
2019/05/27	CH2205	Male	Three y/o	Treatment (Wk 2)	Treatment	Feed Day	No	02:45	02:00 - 02:59	Early Morning	37,5		19,4	19,4	18,65		99,28
2019/05/27	CH2206	Male	Three y/o	Treatment (Wk 2)	Treatment	Feed Day	No	02:45	02:00 - 02:59	Early Morning	37,3	51	57,6	57,6	18,46	541,20	136,68
2019/05/27	CH2205	Male	Three y/o	Treatment (Wk 2)	Treatment	Feed Day	No	02:50	02:00 - 02:59	Early Morning	37,4	83	17,8	17,8	18,55	580,11	96,34
2019/05/27	CH2206	Male	Three y/o	Treatment (Wk 2)	Treatment	Feed Day	No	02:50	02:00 - 02:59	Early Morning	37,2	68	53,0	53,0	18,36	564,71	133,80
2019/05/27	CH2205	Male	Three y/o	Treatment (Wk 2)	Treatment	Feed Day	No	02:55	02:00 - 02:59	Early Morning	37,4	41	23,8	23,8	18,55	522,29	106,26
2019/05/27	CH2206	Male	Three y/o	Treatment (Wk 2)	Treatment	Feed Day	No	02:55	02:00 - 02:59	Early Morning	37,3	48	54,8	54,8	18,46	536,04	134,96
2019/05/27	CH2205	Male	Three y/o	Treatment (Wk 2)	Treatment	Feed Day	No	03:00	03:00 - 03:59	Early Morning	37,4	44	24,0	24,0	18,55	528,51	106,55
2019/05/27	CH2206	Male	Three y/o	Treatment (Wk 2)	Treatment	Feed Day	No	03:00	03:00 - 03:59	Early Morning	37,3	47	48,8	48,8	18,46	534,23	130,95
2019/05/27	CH2205	Male	Three y/o	Treatment (Wk 2)	Treatment	Feed Day	No	03:05	03:00 - 03:59	Early Morning	37,5	53	24,2	24,2	18,65	544,43	106,83
2019/05/27	CH2206	Male	Three y/o	Treatment (Wk 2)	Treatment	Feed Day	No	03:05	03:00 - 03:59	Early Morning	37,3	45	44,0	44,0	18,46	530,47	127,37
2019/05/27	CH2205	Male	Three y/o	Treatment (Wk 2)	Treatment	Feed Day	No	03:10	03:00 - 03:59	Early Morning	37,5	42	24,8	24,8	18,65	524,42	107,67
2019/05/27	CH2206	Male	Three y/o	Treatment (Wk 2)	Treatment	Feed Day	No	03:10	03:00 - 03:59	Early Morning	37,4	69	41,0	41,0	18,55	565,86	124,94
2019/05/27	CH2205	Male	Three y/o	Treatment (Wk 2)	Treatment	Feed Day	No	03:15	03:00 - 03:59	Early Morning	37,6	53	25,2	25,2	18,75	544,43	108,22
2019/05/27	CH2206	Male	Three y/o	Treatment (Wk 2)	Treatment	Feed Day	No	03:15	03:00 - 03:59	Early Morning	37,4	45	48,2	48,2	18,55	530,47	130,52
2019/05/27	CH2205	Male	Three y/o	Treatment (Wk 2)	Treatment	Feed Day	No	03:20	03:00 - 03:59	Early Morning	37,6	42	15,8	15,8	18,75	524,42	92,28
2019/05/27	CH2206	Male	Three y/o	Treatment (Wk 2)	Treatment	Feed Day	No	03:20	03:00 - 03:59	Early Morning	37,4	68	53,4	53,4	18,55	564,71	134,06
2019/05/27	CH2205	Male	Three y/o	Treatment (Wk 2)	Treatment	Feed Day	No	03:25	03:00 - 03:59	Early Morning	37,6	68	14,8	14,8	18,75	564,71	90,05
2019/05/27	CH2206	Male	Three y/o	Treatment (Wk 2)	Treatment	Feed Day	No	03:25	03:00 - 03:59	Early Morning	37,4	50	54,6	54,6	18,55	539,52	134,83
2019/05/27	CH2205	Male	Three y/o	Treatment (Wk 2)	Treatment	Feed Day	No	03:30	03:00 - 03:59	Early Morning	37,6	42	17,6	17,6	18,75	524,42	95,95

2019/05/27	CH2206	Male	Three y/o	Treatment (Wk 2)	Treatment	Feed Day	No	03:30	03:00 - 03:59	Early Morning	37,5	43	54,8	54,8	18,65	526,50	134,96
2019/05/27	CH2205	Male	Three y/o	Treatment (Wk 2)	Treatment	Feed Day	No	03:35	03:00 - 03:59	Early Morning	37,6	53	16,4	16,4	18,75	544,43	93,55
2019/05/27	CH2206	Male	Three y/o	Treatment (Wk 2)	Treatment	Feed Day	No	03:35	03:00 - 03:59	Early Morning	37,5	43	49,2	49,2	18,65	526,50	131,23
2019/05/27	CH2205	Male	Three y/o	Treatment (Wk 2)	Treatment	Feed Day	No	03:40	03:00 - 03:59	Early Morning	37,6	38	16,2	16,2	18,75	515,48	93,13
2019/05/27	CH2206	Male	Three y/o	Treatment (Wk 2)	Treatment	Feed Day	No	03:40	03:00 - 03:59	Early Morning	37,5		12,2	12,2	18,65		83,49
2019/05/27	CH2205	Male	Three y/o	Treatment (Wk 2)	Treatment	Feed Day	No	03:45	03:00 - 03:59	Early Morning	37,6	41	18,4	18,4	18,75	522,29	97,47
2019/05/27	CH2206	Male	Three y/o	Treatment (Wk 2)	Treatment	Feed Day	No	03:45	03:00 - 03:59	Early Morning	37,5	55	34,8	34,8	18,65	547,52	119,29
2019/05/27	CH2205	Male	Three y/o	Treatment (Wk 2)	Treatment	Feed Day	No	03:50	03:00 - 03:59	Early Morning	37,5	114	18,2	18,2	18,65	603,20	97,10
2019/05/27	CH2206	Male	Three y/o	Treatment (Wk 2)	Treatment	Feed Day	No	03:50	03:00 - 03:59	Early Morning	37,5	43	47,0	47,0	18,65	526,50	129,65
2019/05/27	CH2205	Male	Three y/o	Treatment (Wk 2)	Treatment	Feed Day	No	03:55	03:00 - 03:59	Early Morning	37,5		16,0	16,0	18,65		92,71
2019/05/27	CH2206	Male	Three y/o	Treatment (Wk 2)	Treatment	Feed Day	No	03:55	03:00 - 03:59	Early Morning	37,5	43	47,0	47,0	18,65	526,50	129,65
2019/05/27	CH2205	Male	Three y/o	Treatment (Wk 2)	Treatment	Feed Day	No	04:00	04:00 - 04:59	Morning	37,4		21,2	21,2	18,55		102,31
2019/05/27	CH2206	Male	Three y/o	Treatment (Wk 2)	Treatment	Feed Day	No	04:00	04:00 - 04:59	Morning	37,5	43	11,4	11,4	18,65	526,50	81,19
2019/05/27	CH2205	Male	Three y/o	Treatment (Wk 2)	Treatment	Feed Day	No	04:05	04:00 - 04:59	Morning	37,5	86	22,4	22,4	18,65	582,77	104,19
2019/05/27	CH2206	Male	Three y/o	Treatment (Wk 2)	Treatment	Feed Day	No	04:05	04:00 - 04:59	Morning	37,5	47	6,6	6,6	18,65	534,23	62,73
2019/05/27	CH2205	Male	Three y/o	Treatment (Wk 2)	Treatment	Feed Day	No	04:10	04:00 - 04:59	Morning	37,4	49	24,0	24,0	18,55	537,80	106,55
2019/05/27	CH2206	Male	Three y/o	Treatment (Wk 2)	Treatment	Feed Day	No	04:10	04:00 - 04:59	Morning	37,5	42	14,0	14,0	18,65	524,42	88,17
2019/05/27	CH2205	Male	Three y/o	Treatment (Wk 2)	Treatment	Feed Day	No	04:15	04:00 - 04:59	Morning	37,4	152	16,2	16,2	18,55	622,73	93,13
2019/05/27	CH2206	Male	Three y/o	Treatment (Wk 2)	Treatment	Feed Day	No	04:15	04:00 - 04:59	Morning	37,5	111	63,0	63,0	18,65	601,33	139,78
2019/05/27	CH2205	Male	Three y/o	Treatment (Wk 2)	Treatment	Feed Day	No	04:20	04:00 - 04:59	Morning	37,5	80	10,0	10,0	18,65	577,31	76,76
2019/05/27	CH2206	Male	Three y/o	Treatment (Wk 2)	Treatment	Feed Day	No	04:20	04:00 - 04:59	Morning	37,4	55	66,4	66,4	18,55	547,52	141,61
2019/05/27	CH2205	Male	Three y/o	Treatment (Wk 2)	Treatment	Feed Day	No	04:25	04:00 - 04:59	Morning	37,5	48	10,6	10,6	18,65	536,04	78,73
2019/05/27	CH2206	Male	Three y/o	Treatment (Wk 2)	Treatment	Feed Day	No	04:25	04:00 - 04:59	Morning	37,3	47	53,4	53,4	18,46	534,23	134,06
2019/05/27	CH2205	Male	Three y/o	Treatment (Wk 2)	Treatment	Feed Day	No	04:30	04:00 - 04:59	Morning	37,5	51	7,2	7,2	18,65	541,20	65,66
2019/05/27	CH2206	Male	Three y/o	Treatment (Wk 2)	Treatment	Feed Day	No	04:30	04:00 - 04:59	Morning	37,4	40	48,0	48,0	18,55	520,09	130,38
2019/05/27	CH2205	Male	Three y/o	Treatment (Wk 2)	Treatment	Feed Day	No	04:35	04:00 - 04:59	Morning	37,5	59	8,4	8,4	18,65	553,30	70,86
2019/05/27	CH2206	Male	Three y/o	Treatment (Wk 2)	Treatment	Feed Day	No	04:35	04:00 - 04:59	Morning	37,4	39	48,6	48,6	18,55	517,82	130,81
2019/05/27	CH2205	Male	Three y/o	Treatment (Wk 2)	Treatment	Feed Day	No	04:40	04:00 - 04:59	Morning	37,5	61	16,4	16,4	18,65	556,01	93,55
2019/05/27	CH2206	Male	Three y/o	Treatment (Wk 2)	Treatment	Feed Day	No	04:40	04:00 - 04:59	Morning	37,4	58	54,0	54,0	18,55	551,90	134,45
2019/05/27	CH2205	Male	Three y/o	Treatment (Wk 2)	Treatment	Feed Day	No	04:45	04:00 - 04:59	Morning	37,5	56	19,4	19,4	18,65	549,01	99,28
2019/05/27	CH2206	Male	Three y/o	Treatment (Wk 2)	Treatment	Feed Day	No	04:45	04:00 - 04:59	Morning	37,4	41	59,8	59,8	18,55	522,29	137,98
2019/05/27	CH2205	Male	Three y/o	Treatment (Wk 2)	Treatment	Feed Day	No	04:50	04:00 - 04:59	Morning	37,5	48	15,2	15,2	18,65	536,04	90,96
2019/05/27	CH2206	Male	Three y/o	Treatment (Wk 2)	Treatment	Feed Day	No	04:50	04:00 - 04:59	Morning	37,4	48	65,0	65,0	18,55	536,04	140,87
2019/05/27	CH2205	Male	Three y/o	Treatment (Wk 2)	Treatment	Feed Day	No	04:55	04:00 - 04:59	Morning	37,4	46	14,6	14,6	18,55	532,38	89,59
2019/05/27	CH2206	Male	Three y/o	Treatment (Wk 2)	Treatment	Feed Day	No	04:55	04:00 - 04:59	Morning	37,3	46	61,8	61,8	18,46	532,38	139,12
2019/05/27	CH2205	Male	Three y/o	Treatment (Wk 2)	Treatment	Feed Day	No	05:00	05:00 - 05:59	Morning	37,4	41	14,0	14,0	18,55	522,29	88,17
2019/05/27	CH2206	Male	Three y/o	Treatment (Wk 2)	Treatment	Feed Day	No	05:00	05:00 - 05:59	Morning	37,3	40	64,0	64,0	18,46	520,09	140,33
2019/05/27	CH2205	Male	Three y/o	Treatment (Wk 2)	Treatment	Feed Day	No	05:05	05:00 - 05:59	Morning	37,4	42	8,4	8,4	18,55	524,42	70,86
2019/05/27	CH2206	Male	Three y/o	Treatment (Wk 2)	Treatment	Feed Day	No	05:05	05:00 - 05:59	Morning	37,3	36	62,2	62,2	18,46	510,56	139,34
2019/05/27	CH2205	Male	Three y/o	Treatment (Wk 2)	Treatment	Feed Day	No	05:10	05:00 - 05:59	Morning	37,4	45	9,6	9,6	18,55	530,47	75,37
2019/05/27	CH2206	Male	Three y/o	Treatment (Wk 2)	Treatment	Feed Day	No	05:10	05:00 - 05:59	Morning	37,2	78	52,4	52,4	18,36	575,38	133,41
2019/05/27	CH2205	Male	Three y/o	Treatment (Wk 2)	Treatment	Feed Day	No	05:15	05:00 - 05:59	Morning	37,4	41	12,2	12,2	18,55	522,29	83,49
2019/05/27	CH2206	Male	Three y/o	Treatment (Wk 2)	Treatment	Feed Day	No	05:15	05:00 - 05:59	Morning	37,2	46	58,0	58,0	18,36	532,38	136,92
2019/05/27	CH2205	Male	Three y/o	Treatment (Wk 2)	Treatment	Feed Day	No	05:20	05:00 - 05:59	Morning	37,4	47	10,6	10,6	18,55	534,23	78,73
2019/05/27	CH2206	Male	Three y/o	Treatment (Wk 2)	Treatment	Feed Day	No	05:20	05:00 - 05:59	Morning	37,2	94	60,8	60,8	18,36	589,37	138,55
2019/05/27	CH2205	Male	Three y/o	Treatment (Wk 2)	Treatment	Feed Day	No	05:25	05:00 - 05:59	Morning	37,3	44	11,2	11,2	18,46	528,51	80,59
2019/05/27	CH2206	Male	Three y/o	Treatment (Wk 2)	Treatment	Feed Day	No	05:25	05:00 - 05:59	Morning	37,2	56	62,4	62,4	18,36	549,01	139,45
2019/05/27	CH2205	Male	Three y/o	Treatment (Wk 2)	Treatment	Feed Day	No	05:30	05:00 - 05:59	Morning	37,3	69	9,8	9,8	18,46	565,86	76,07
2019/05/27	CH2206	Male	Three y/o	Treatment (Wk 2)	Treatment	Feed Day	No	05:30	05:00 - 05:59	Morning	37,2	44	66,0	66,0	18,36	528,51	141,40
2019/05/27	CH2205	Male	Three y/o	Treatment (Wk 2)	Treatment	Feed Day	No	05:35	05:00 - 05:59	Morning	37,3	39	16,6	16,6	18,46	517,82	93,96
2019/05/27	CH2206	Male	Three y/o	Treatment (Wk 2)	Treatment	Feed Day	No	05:35	05:00 - 05:59	Morning	37,2	41	59,6	59,6	18,36	522,29	137,86
2019/05/27	CH2205	Male	Three y/o	Treatment (Wk 2)	Treatment	Feed Day	No	05:40	05:00 - 05:59	Morning	37,3	41	10,8	10,8	18,46	522,29	79,36
2019/05/27	CH2206	Male	Three y/o	Treatment (Wk 2)	Treatment	Feed Day	No	05:40	05:00 - 05:59	Morning	37,2	95	67,8	67,8	18,36	590,14	142,33
2019/05/27	CH2205	Male	Three y/o	Treatment (Wk 2)	Treatment	Feed Day	No	05:45	05:00 - 05:59	Morning	37,3	101	18,2	18,2	18,46	594,59	97,10
2019/05/27	CH2206	Male	Three y/o	Treatment (Wk 2)	Treatment	Feed Day	No	05:45	05:00 - 05:59	Morning	37,2	45	46,2	46,2	18,36	530,47	129,06
2019/05/27	CH2205	Male	Three y/o	Treatment (Wk 2)	Treatment	Feed Day	No	05:50	05:00 - 05:59	Morning	37,3	87	23,6	23,6	18,46	583,64	105,97
2019/05/27	CH2206	Male	Three y/o	Treatment (Wk 2)	Treatment	Feed Day	No	05:50	05:00 - 05:59	Morning	37,1	40	45,6	45,6	18,26	520,09	128,61
2019/05/27	CH2205	Male	Three y/o	Treatment (Wk 2)	Treatment	Feed Day	No	05:55	05:00 - 05:59	Morning	37,2	112	33,4	33,4	18,36	601,96	117,88
2019/05/27	CH2206	Male	Three y/o	Treatment (Wk 2)	Treatment	Feed Day	No	05:55	05:00 - 05:59	Morning	37,1	138	36,4	36,4	18,26	616,31	120,84
2019/05/27	CH2205	Male	Three y/o	Treatment (Wk 2)	Treatment	Feed Day	No	06:00	06:00 - 06:59	Morning	37,2	47	163,0	163,0	18,36	534,23	172,93
2019/05/27	CH2206	Male	Three y/o	Treatment (Wk 2)	Treatment	Feed Day	No	06:00	06:00 - 06:59	Morning	37,2	125	140,8	140,8	18,36	609,60	167,80
2019/05/27	CH2205	Male	Three y/o	Treatment (Wk 2)	Treatment	Feed Day	No	06:05	06:00 - 06:59	Morning	37,2	85	69,4	69,4	18,36	581,90	143,14
2019/05/27	CH2206	Male	Three y/o	Treatment (Wk 2)	Treatment	Feed Day	No	06:05	06:00 - 06:59	Morning	37,3	194	251,8	251,8	18,46	638,30	188,23
2019/05/27	CH2205	Male	Three y/o	Treatment (Wk 2)	Treatment	Feed Day	No	06:10	06:00 - 06:59	Morning	37,2	98	25,0	25,0	18,36	592,41	107,94
2019/05/27	CH2206	Male	Three y/o	Treatment (Wk 2)	Treatment	Feed Day	No	06:10	06:00 - 06:59	Morning	37,4		88,4	88,4	18,55		151,55
2019/05/27	CH2205	Male	Three y/o	Treatment (Wk 2)	Treatment	Feed Day	No	06:15	06:00 - 06:59	Morning	37,2		13,8	13,8	18,36		87,68
2019/05/27	CH2206	Male	Three y/o	Treatment (Wk 2)	Treatment	Feed Day	No	06:15	06:00 - 06:59	Morning	37,5		273,2	273,2	18,65		191,11
2019/05/27	CH2205	Male	Three y/o	Treatment (Wk 2)	Treatment	Feed Day	No	06:20	06:00 - 06:59	Morning	37,1	65	19,6	19,6	18,26	561,12	99,63
2019/05/27	CH2206	Male	Three y/o	Treatment (Wk 2)	Treatment	Feed Day	No	06:20	06:00 - 06:59	Morning	37,7	126	160,8	160,8	18,85	610,14	172,46
2019/05/27	CH2205	Male	Three y/o	Treatment (Wk 2)	Treatment	Feed Day	No	06:25	06:00 - 06:59	Morning	37,0	52	36,6	36,6	18,16	542,83	121,03

2019/05/27	CH2206	Male	Three y/o	Treatment (Wk 2)	Treatment	Feed Day	No	06:25	06:00 - 06:59	Morning	37,8	67	68,8	68,8	18,95	563,53	142,84
2019/05/27	CH2205	Male	Three y/o	Treatment (Wk 2)	Treatment	Feed Day	No	06:30	06:00 - 06:59	Morning	37,2		185,2	185,2	18,36		177,42
2019/05/27	CH2206	Male	Three y/o	Treatment (Wk 2)	Treatment	Feed Day	No	06:30	06:00 - 06:59	Morning	37,9	115	201,8	201,8	19,05	603,82	180,43
2019/05/27	CH2205	Male	Three y/o	Treatment (Wk 2)	Treatment	Feed Day	No	06:35	06:00 - 06:59	Morning	37,3	109	157,8	157,8	18,46	600,04	171,79
2019/05/27	CH2206	Male	Three y/o	Treatment (Wk 2)	Treatment	Feed Day	No	06:35	06:00 - 06:59	Morning	37,9	66	163,4	163,4	19,05	562,34	173,02
2019/05/27	CH2205	Male	Three y/o	Treatment (Wk 2)	Treatment	Feed Day	No	06:40	06:00 - 06:59	Morning	37,3		198,0	198,0	18,46		179,77
2019/05/27	CH2206	Male	Three y/o	Treatment (Wk 2)	Treatment	Feed Day	No	06:40	06:00 - 06:59	Morning	37,9		342,2	342,2	19,05		199,08
2019/05/27	CH2205	Male	Three y/o	Treatment (Wk 2)	Treatment	Feed Day	No	06:45	06:00 - 06:59	Morning	37,5	90	121,6	121,6	18,65	586,16	162,67
2019/05/27	CH2206	Male	Three y/o	Treatment (Wk 2)	Treatment	Feed Day	No	06:45	06:00 - 06:59	Morning	38,0	112	55,0	55,0	19,15	601,96	135,08
2019/05/27	CH2205	Male	Three y/o	Treatment (Wk 2)	Treatment	Feed Day	No	06:50	06:00 - 06:59	Morning	37,4	101	54,2	54,2	18,55	594,59	134,57
2019/05/27	CH2206	Male	Three y/o	Treatment (Wk 2)	Treatment	Feed Day	No	06:50	06:00 - 06:59	Morning	38,0	138	131,8	131,8	19,15	616,31	165,49
2019/05/27	CH2205	Male	Three y/o	Treatment (Wk 2)	Treatment	Feed Day	No	06:55	06:00 - 06:59	Morning	37,6	115	48,0	48,0	18,75	603,82	130,38
2019/05/27	CH2206	Male	Three y/o	Treatment (Wk 2)	Treatment	Feed Day	No	06:55	06:00 - 06:59	Morning	38,0	113	82,4	82,4	19,15	602,58	149,10
2019/05/27	CH2205	Male	Three y/o	Treatment (Wk 2)	Treatment	Feed Day	No	07:00	07:00 - 07:59	Morning	37,6	79	224,2	224,2	18,75	576,36	184,14
2019/05/27	CH2206	Male	Three y/o	Treatment (Wk 2)	Treatment	Feed Day	No	07:00	07:00 - 07:59	Morning	37,9	93	68,4	68,4	19,05	588,58	142,64
2019/05/27	CH2205	Male	Three y/o	Treatment (Wk 2)	Treatment	Feed Day	No	07:05	07:00 - 07:59	Morning	37,7	172	166,6	166,6	18,85	630,73	173,70
2019/05/27	CH2206	Male	Three y/o	Treatment (Wk 2)	Treatment	Feed Day	No	07:05	07:00 - 07:59	Morning	38,0	95	124,0	124,0	19,15	590,14	163,35
2019/05/27	CH2205	Male	Three y/o	Treatment (Wk 2)	Treatment	Feed Day	No	07:10	07:00 - 07:59	Morning	37,9	80	16,0	16,0	19,05	577,31	92,71
2019/05/27	CH2206	Male	Three y/o	Treatment (Wk 2)	Treatment	Feed Day	No	07:10	07:00 - 07:59	Morning	37,9	126	129,6	129,6	19,05	610,14	164,90
2019/05/27	CH2205	Male	Three y/o	Treatment (Wk 2)	Treatment	Feed Day	No	07:15	07:00 - 07:59	Morning	38,0	47	21,4	21,4	19,15	534,23	102,63
2019/05/27	CH2206	Male	Three y/o	Treatment (Wk 2)	Treatment	Feed Day	No	07:15	07:00 - 07:59	Morning	38,0	113	131,4	131,4	19,15	602,58	165,38
2019/05/27	CH2205	Male	Three y/o	Treatment (Wk 2)	Treatment	Feed Day	No	07:20	07:00 - 07:59	Morning	37,9	46	14,0	14,0	19,05	532,38	88,17
2019/05/27	CH2206	Male	Three y/o	Treatment (Wk 2)	Treatment	Feed Day	No	07:20	07:00 - 07:59	Morning	37,8	79	28,4	28,4	18,95	576,36	112,31
2019/05/27	CH2205	Male	Three y/o	Treatment (Wk 2)	Treatment	Feed Day	No	07:25	07:00 - 07:59	Morning	37,9	57	55,4	55,4	19,05	550,47	135,33
2019/05/27	CH2206	Male	Three y/o	Treatment (Wk 2)	Treatment	Feed Day	No	07:25	07:00 - 07:59	Morning	37,8	55	255,4	255,4	18,95	547,52	188,73
2019/05/27	CH2205	Male	Three y/o	Treatment (Wk 2)	Treatment	Feed Day	No	07:30	07:00 - 07:59	Morning	37,7	131	451,2	451,2	18,85	612,80	208,89
2019/05/27	CH2206	Male	Three y/o	Treatment (Wk 2)	Treatment	Feed Day	No	07:30	07:00 - 07:59	Morning	37,9	131	200,6	200,6	19,05	612,80	180,22
2019/05/27	CH2205	Male	Three y/o	Treatment (Wk 2)	Treatment	Feed Day	No	07:35	07:00 - 07:59	Morning	38,1	104	131,4	131,4	19,26	596,69	165,38
2019/05/27	CH2206	Male	Three y/o	Treatment (Wk 2)	Treatment	Feed Day	No	07:35	07:00 - 07:59	Morning	37,9	79	101,2	101,2	19,05	576,36	156,26
2019/05/27	CH2205	Male	Three y/o	Treatment (Wk 2)	Treatment	Feed Day	No	07:40	07:00 - 07:59	Morning	38,1	86	32,2	32,2	19,26	582,77	116,63
2019/05/27	CH2206	Male	Three y/o	Treatment (Wk 2)	Treatment	Feed Day	No	07:40	07:00 - 07:59	Morning	38,0		127,2	127,2	19,15		164,24
2019/05/27	CH2205	Male	Three y/o	Treatment (Wk 2)	Treatment	Feed Day	No	07:45	07:00 - 07:59	Morning	38,0	85	314,4	314,4	19,15	581,90	196,08
2019/05/27	CH2206	Male	Three y/o	Treatment (Wk 2)	Treatment	Feed Day	No	07:45	07:00 - 07:59	Morning	38,0		160,4	160,4	19,15		172,37
2019/05/27	CH2205	Male	Three y/o	Treatment (Wk 2)	Treatment	Feed Day	No	07:50	07:00 - 07:59	Morning	38,0	98	90,8	90,8	19,15	592,41	152,48
2019/05/27	CH2206	Male	Three y/o	Treatment (Wk 2)	Treatment	Feed Day	No	07:50	07:00 - 07:59	Morning	38,0	109	82,8	82,8	19,15	600,04	149,27
2019/05/27	CH2205	Male	Three y/o	Treatment (Wk 2)	Treatment	Feed Day	No	07:55	07:00 - 07:59	Morning	38,0	70	23,0	23,0	19,15	566,99	105,09
2019/05/27	CH2206	Male	Three y/o	Treatment (Wk 2)	Treatment	Feed Day	No	07:55	07:00 - 07:59	Morning	38,0	114	209,6	209,6	19,15	603,20	181,77
2019/05/27	CH2205	Male	Three y/o	Treatment (Wk 2)	Treatment	Feed Day	No	08:00	08:00 - 08:59	Late Morning	38,0	60	87,0	87,0	19,15	554,67	150,99
2019/05/27	CH2206	Male	Three y/o	Treatment (Wk 2)	Treatment	Feed Day	No	08:00	08:00 - 08:59	Late Morning	38,0	104	266,2	266,2	19,15	596,69	190,20
2019/05/27	CH2205	Male	Three y/o	Treatment (Wk 2)	Treatment	Feed Day	No	08:05	08:00 - 08:59	Late Morning	38,0		81,0	81,0	19,15		148,51
2019/05/27	CH2206	Male	Three y/o	Treatment (Wk 2)	Treatment	Feed Day	No	08:05	08:00 - 08:59	Late Morning	38,0	109	204,6	204,6	19,15	600,04	180,92
2019/05/27	CH2205	Male	Three y/o	Treatment (Wk 2)	Treatment	Feed Day	No	08:10	08:00 - 08:59	Late Morning	38,0	172	212,2	212,2	19,15	630,73	182,20
2019/05/27	CH2206	Male	Three y/o	Treatment (Wk 2)	Treatment	Feed Day	No	08:10	08:00 - 08:59	Late Morning	38,0	126	474,8	474,8	19,15	610,14	210,71
2019/05/27	CH2205	Male	Three y/o	Treatment (Wk 2)	Treatment	Feed Day	No	08:15	08:00 - 08:59	Late Morning	38,0		130,2	130,2	19,15		165,06
2019/05/27	CH2206	Male	Three y/o	Treatment (Wk 2)	Treatment	Feed Day	No	08:15	08:00 - 08:59	Late Morning	38,0	110	149,0	149,0	19,15	600,69	169,78
2019/05/27	CH2205	Male	Three y/o	Treatment (Wk 2)	Treatment	Feed Day	No	08:20	08:00 - 08:59	Late Morning	38,0	123	252,0	252,0	19,15	608,49	188,26
2019/05/27	CH2206	Male	Three y/o	Treatment (Wk 2)	Treatment	Feed Day	No	08:20	08:00 - 08:59	Late Morning	38,0	126	459,0	459,0	19,15	610,14	209,50
2019/05/27	CH2205	Male	Three y/o	Treatment (Wk 2)	Treatment	Feed Day	No	08:25	08:00 - 08:59	Late Morning	38,0	30	86,8	86,8	19,15	493,51	150,91
2019/05/27	CH2206	Male	Three y/o	Treatment (Wk 2)	Treatment	Feed Day	No	08:25	08:00 - 08:59	Late Morning	38,0	114	324,2	324,2	19,15	603,20	197,17
2019/05/27	CH2205	Male	Three y/o	Treatment (Wk 2)	Treatment	Feed Day	Yes	08:30	08:00 - 08:59	Late Morning	38,0	102	517,8	517,8	19,15	595,30	213,79
2019/05/27	CH2206	Male	Three y/o	Treatment (Wk 2)	Treatment	Feed Day	Yes	08:30	08:00 - 08:59	Late Morning	38,0		167,0	167,0	19,15		173,78
2019/05/27	CH2205	Male	Three y/o	Treatment (Wk 2)	Treatment	Feed Day	Yes	08:35	08:00 - 08:59	Late Morning	38,0	118	231,8	231,8	19,15	605,61	185,31
2019/05/27	CH2206	Male	Three y/o	Treatment (Wk 2)	Treatment	Feed Day	Yes	08:35	08:00 - 08:59	Late Morning	38,1	141	125,4	125,4	19,26	617,75	163,75
2019/05/27	CH2205	Male	Three y/o	Treatment (Wk 2)	Treatment	Feed Day	Yes	08:40	08:00 - 08:59	Late Morning	38,1		263,8	263,8	19,26		189,88
2019/05/27	CH2206	Male	Three y/o	Treatment (Wk 2)	Treatment	Feed Day	Yes	08:40	08:00 - 08:59	Late Morning	38,2	124	211,8	211,8	19,36	609,04	182,14
2019/05/27	CH2205	Male	Three y/o	Treatment (Wk 2)	Treatment	Feed Day	Yes	08:45	08:00 - 08:59	Late Morning	38,1	105	225,4	225,4	19,26	597,38	184,33
2019/05/27	CH2206	Male	Three y/o	Treatment (Wk 2)	Treatment	Feed Day	Yes	08:45	08:00 - 08:59	Late Morning	38,2	121	126,8	126,8	19,36	607,35	164,13
2019/05/27	CH2205	Male	Three y/o	Treatment (Wk 2)	Treatment	Feed Day	Yes	08:50	08:00 - 08:59	Late Morning	38,1	110	94,4	94,4	19,26	600,69	153,84
2019/05/27	CH2206	Male	Three y/o	Treatment (Wk 2)	Treatment	Feed Day	Yes	08:50	08:00 - 08:59	Late Morning	38,2	188	262,8	262,8	19,36	636,34	189,74
2019/05/27	CH2205	Male	Three y/o	Treatment (Wk 2)	Treatment	Feed Day	Yes	08:55	08:00 - 08:59	Late Morning	38,2		246,6	246,6	19,36		187,50
2019/05/27	CH2206	Male	Three y/o	Treatment (Wk 2)	Treatment	Feed Day	Yes	08:55	08:00 - 08:59	Late Morning	38,2	185	291,6	291,6	19,36	635,34	193,42
2019/05/27	CH2205	Male	Three y/o	Treatment (Wk 2)	Treatment	Feed Day	Yes	09:00	09:00 - 09:59	Late Morning	38,2	106	155,8	155,8	19,36	598,06	171,35
2019/05/27	CH2206	Male	Three y/o	Treatment (Wk 2)	Treatment	Feed Day	Yes	09:00	09:00 - 09:59	Late Morning	38,3		160,2	160,2	19,46		172,32
2019/05/27	CH2205	Male	Three y/o	Treatment (Wk 2)	Treatment	Feed Day	Yes	09:05	09:00 - 09:59	Late Morning	38,3		59,4	59,4	19,46		137,75
2019/05/27	CH2206	Male	Three y/o	Treatment (Wk 2)	Treatment	Feed Day	Yes	09:05	09:00 - 09:59	Late Morning	38,3	165	35,8	35,8	19,46	628,06	120,27
2019/05/27	CH2205	Male	Three y/o	Treatment (Wk 2)	Treatment	Feed Day	Yes	09:10	09:00 - 09:59	Late Morning	38,0	81	25,8	25,8	19,15	578,26	109,02
2019/05/27	CH2206	Male	Three y/o	Treatment (Wk 2)	Treatment	Feed Day	Yes	09:10	09:00 - 09:59	Late Morning	38,0	156	61,2	61,2	19,15	624,43	138,78
2019/05/27	CH2205	Male	Three y/o	Treatment (Wk 2)	Treatment	Feed Day	Yes	09:15	09:00 - 09:59	Late Morning	37,7		16,2	16,2	18,85		93,13
2019/05/27	CH2206	Male	Three y/o	Treatment (Wk 2)	Treatment	Feed Day	Yes	09:15	09:00 - 09:59	Late Morning	37,9		33,4	33,4	19,05		117,88
2019/05/27	CH2205	Male	Three y/o	Treatment (Wk 2)	Treatment	Feed Day	Yes	09:20	09:00 - 09:59	Late Morning	37,4		17,2	17,2	18,55		95,17

Dataset S1: LOGGER

2019/05/27	CH2206	Male	Three y/o	Treatment (Wk 2)	Treatment	Feed Day	Yes	09:20	09:00 - 09:59	Late Morning	37,8	150	188,8	188,8	18,95	621,86	178,09
2019/05/27	CH2205	Male	Three y/o	Treatment (Wk 2)	Treatment	Feed Day	Yes	09:25	09:00 - 09:59	Late Morning	37,6	51	33,8	33,8	18,75	541,20	118,29
2019/05/27	CH2206	Male	Three y/o	Treatment (Wk 2)	Treatment	Feed Day	Yes	09:25	09:00 - 09:59	Late Morning	38,0		110,2	110,2	19,15		159,23
2019/05/27	CH2205	Male	Three y/o	Treatment (Wk 2)	Treatment	Feed Day	No	09:30	09:00 - 09:59	Late Morning	37,7	105	69,2	69,2	18,85	597,38	143,04
2019/05/27	CH2206	Male	Three y/o	Treatment (Wk 2)	Treatment	Feed Day	No	09:30	09:00 - 09:59	Late Morning	38,1	118	85,4	85,4	19,26	605,61	150,35
2019/05/27	CH2205	Male	Three y/o	Treatment (Wk 2)	Treatment	Feed Day	No	09:35	09:00 - 09:59	Late Morning	37,9		87,0	87,0	19,05		150,99
2019/05/27	CH2206	Male	Three y/o	Treatment (Wk 2)	Treatment	Feed Day	No	09:35	09:00 - 09:59	Late Morning	38,1		72,6	72,6	19,26		144,70
2019/05/27	CH2205	Male	Three y/o	Treatment (Wk 2)	Treatment	Feed Day	No	09:40	09:00 - 09:59	Late Morning	37,9	107	82,6	82,6	19,05	598,73	149,19
2019/05/27	CH2206	Male	Three y/o	Treatment (Wk 2)	Treatment	Feed Day	No	09:40	09:00 - 09:59	Late Morning	38,1	108	126,4	126,4	19,26	599,39	164,02
2019/05/27	CH2205	Male	Three y/o	Treatment (Wk 2)	Treatment	Feed Day	No	09:45	09:00 - 09:59	Late Morning	37,9	103	84,0	84,0	19,05	596,00	149,77
2019/05/27	CH2206	Male	Three y/o	Treatment (Wk 2)	Treatment	Feed Day	No	09:45	09:00 - 09:59	Late Morning	38,1	124	171,2	171,2	19,26	609,04	174,65
2019/05/27	CH2205	Male	Three y/o	Treatment (Wk 2)	Treatment	Feed Day	No	09:50	09:00 - 09:59	Late Morning	37,9		22,2	22,2	19,05		103,88
2019/05/27	CH2206	Male	Three y/o	Treatment (Wk 2)	Treatment	Feed Day	No	09:50	09:00 - 09:59	Late Morning	38,2	123	227,8	227,8	19,36	608,49	184,70
2019/05/27	CH2205	Male	Three y/o	Treatment (Wk 2)	Treatment	Feed Day	No	09:55	09:00 - 09:59	Late Morning	37,9	121	31,4	31,4	19,05	607,35	115,76
2019/05/27	CH2206	Male	Three y/o	Treatment (Wk 2)	Treatment	Feed Day	No	09:55	09:00 - 09:59	Late Morning	38,2	103	35,0	35,0	19,36	596,00	119,49
2019/05/27	CH2205	Male	Three y/o	Treatment (Wk 2)	Treatment	Feed Day	No	10:00	10:00 - 10:59	Late Morning	37,9	96	118,0	118,0	19,05	590,91	161,62
2019/05/27	CH2206	Male	Three y/o	Treatment (Wk 2)	Treatment	Feed Day	No	10:00	10:00 - 10:59	Late Morning	38,2	131	202,8	202,8	19,36	612,80	180,61
2019/05/27	CH2205	Male	Three y/o	Treatment (Wk 2)	Treatment	Feed Day	No	10:05	10:00 - 10:59	Late Morning	37,9		54,2	54,2	19,05		134,57
2019/05/27	CH2206	Male	Three y/o	Treatment (Wk 2)	Treatment	Feed Day	No	10:05	10:00 - 10:59	Late Morning	38,2	150	359,6	359,6	19,36	621,86	200,84
2019/05/27	CH2205	Male	Three y/o	Treatment (Wk 2)	Treatment	Feed Day	No	10:10	10:00 - 10:59	Late Morning	37,7	66	83,2	83,2	18,85	562,34	149,44
2019/05/27	CH2206	Male	Three y/o	Treatment (Wk 2)	Treatment	Feed Day	No	10:10	10:00 - 10:59	Late Morning	38,1	132	134,6	134,6	19,26	613,32	166,22
2019/05/27	CH2205	Male	Three y/o	Treatment (Wk 2)	Treatment	Feed Day	No	10:15	10:00 - 10:59	Late Morning	37,9	71	28,4	28,4	19,05	568,10	112,31
2019/05/27	CH2206	Male	Three y/o	Treatment (Wk 2)	Treatment	Feed Day	No	10:15	10:00 - 10:59	Late Morning	38,1	135	136,4	136,4	19,26	614,84	166,69
2019/05/27	CH2205	Male	Three y/o	Treatment (Wk 2)	Treatment	Feed Day	No	10:20	10:00 - 10:59	Late Morning	37,9	90	29,6	29,6	19,05	586,16	113,73
2019/05/27	CH2206	Male	Three y/o	Treatment (Wk 2)	Treatment	Feed Day	No	10:20	10:00 - 10:59	Late Morning	38,1	125	105,2	105,2	19,26	609,60	157,61
2019/05/27	CH2205	Male	Three y/o	Treatment (Wk 2)	Treatment	Feed Day	No	10:25	10:00 - 10:59	Late Morning	37,5	87	28,6	28,6	18,65	583,64	112,56
2019/05/27	CH2206	Male	Three y/o	Treatment (Wk 2)	Treatment	Feed Day	No	10:25	10:00 - 10:59	Late Morning	38,1	96	48,6	48,6	19,26	590,91	130,81
2019/05/27	CH2205	Male	Three y/o	Treatment (Wk 2)	Treatment	Feed Day	No	10:30	10:00 - 10:59	Late Morning	37,3	117	37,6	37,6	18,46	605,02	121,96
2019/05/27	CH2206	Male	Three y/o	Treatment (Wk 2)	Treatment	Feed Day	No	10:30	10:00 - 10:59	Late Morning	38,1	90	27,2	27,2	19,26	586,16	110,83
2019/05/27	CH2205	Male	Three y/o	Treatment (Wk 2)	Treatment	Feed Day	No	10:35	10:00 - 10:59	Late Morning	37,3		45,6	45,6	18,46		128,61
2019/05/27	CH2206	Male	Three y/o	Treatment (Wk 2)	Treatment	Feed Day	No	10:35	10:00 - 10:59	Late Morning	37,9	103	156,2	156,2	19,05	596,00	171,44
2019/05/27	CH2205	Male	Three y/o	Treatment (Wk 2)	Treatment	Feed Day	No	10:40	10:00 - 10:59	Late Morning	37,2		27,2	27,2	18,36		110,83
2019/05/27	CH2206	Male	Three y/o	Treatment (Wk 2)	Treatment	Feed Day	No	10:40	10:00 - 10:59	Late Morning	37,9	123	61,6	61,6	19,05	608,49	139,00
2019/05/27	CH2205	Male	Three y/o	Treatment (Wk 2)	Treatment	Feed Day	No	10:45	10:00 - 10:59	Late Morning	37,3		27,8	27,8	18,46		111,58
2019/05/27	CH2206	Male	Three y/o	Treatment (Wk 2)	Treatment	Feed Day	No	10:45	10:00 - 10:59	Late Morning	38,0	89	19,0	19,0	19,15	585,33	98,56
2019/05/27	CH2205	Male	Three y/o	Treatment (Wk 2)	Treatment	Feed Day	No	10:50	10:00 - 10:59	Late Morning	37,3		27,6	27,6	18,46		111,33
2019/05/27	CH2206	Male	Three y/o	Treatment (Wk 2)	Treatment	Feed Day	No	10:50	10:00 - 10:59	Late Morning	38,0	76	18,2	18,2	19,15	573,39	97,10
2019/05/27	CH2205	Male	Three y/o	Treatment (Wk 2)	Treatment	Feed Day	No	10:55	10:00 - 10:59	Late Morning	37,4	69	14,6	14,6	18,55	565,86	89,59
2019/05/27	CH2206	Male	Three y/o	Treatment (Wk 2)	Treatment	Feed Day	No	10:55	10:00 - 10:59	Late Morning	38,0	86	2,8	2,8	19,15	582,77	34,04
2019/05/27	CH2205	Male	Three y/o	Treatment (Wk 2)	Treatment	Feed Day	No	11:00	11:00 - 11:59	Late Morning	37,5	77	10,2	10,2	18,65	574,39	77,43
2019/05/27	CH2206	Male	Three y/o	Treatment (Wk 2)	Treatment	Feed Day	No	11:00	11:00 - 11:59	Late Morning	38,0	95	6,8	6,8	19,15	590,14	63,74
2019/05/27	CH2205	Male	Three y/o	Treatment (Wk 2)	Treatment	Feed Day	No	11:05	11:00 - 11:59	Late Morning	37,5	69	11,2	11,2	18,65	565,86	80,59
2019/05/27	CH2206	Male	Three y/o	Treatment (Wk 2)	Treatment	Feed Day	No	11:05	11:00 - 11:59	Late Morning	38,0	63	4,4	4,4	19,15	558,62	49,12
2019/05/27	CH2205	Male	Three y/o	Treatment (Wk 2)	Treatment	Feed Day	No	11:10	11:00 - 11:59	Late Morning	37,6	62	11,2	11,2	18,75	557,33	80,59
2019/05/27	CH2206	Male	Three y/o	Treatment (Wk 2)	Treatment	Feed Day	No	11:10	11:00 - 11:59	Late Morning	38,0	92	5,0	5,0	19,15	587,78	53,41
2019/05/27	CH2205	Male	Three y/o	Treatment (Wk 2)	Treatment	Feed Day	No	11:15	11:00 - 11:59	Late Morning	37,6	64	16,0	16,0	18,75	559,88	92,71
2019/05/27	CH2206	Male	Three y/o	Treatment (Wk 2)	Treatment	Feed Day	No	11:15	11:00 - 11:59	Late Morning	38,0	84	4,2	4,2	19,15	581,01	47,57
2019/05/27	CH2205	Male	Three y/o	Treatment (Wk 2)	Treatment	Feed Day	No	11:20	11:00 - 11:59	Late Morning	37,6	68	27,0	27,0	18,75	564,71	110,58
2019/05/27	CH2206	Male	Three y/o	Treatment (Wk 2)	Treatment	Feed Day	No	11:20	11:00 - 11:59	Late Morning	38,0	74	23,8	23,8	19,15	571,33	106,26
2019/05/27	CH2205	Male	Three y/o	Treatment (Wk 2)	Treatment	Feed Day	No	11:25	11:00 - 11:59	Late Morning	37,6		31,2	31,2	18,75		115,54
2019/05/27	CH2206	Male	Three y/o	Treatment (Wk 2)	Treatment	Feed Day	No	11:25	11:00 - 11:59	Late Morning	38,0	83	49,0	49,0	19,15	580,11	131,09
2019/05/27	CH2205	Male	Three y/o	Treatment (Wk 2)	Treatment	Feed Day	No	11:30	11:00 - 11:59	Late Morning	37,6	145	92,0	92,0	18,75	619,62	152,94
2019/05/27	CH2206	Male	Three y/o	Treatment (Wk 2)	Treatment	Feed Day	No	11:30	11:00 - 11:59	Late Morning	37,9		64,0	64,0	19,05		140,33
2019/05/27	CH2205	Male	Three y/o	Treatment (Wk 2)	Treatment	Feed Day	No	11:35	11:00 - 11:59	Late Morning	37,5	92	43,8	43,8	18,65	587,78	127,22
2019/05/27	CH2206	Male	Three y/o	Treatment (Wk 2)	Treatment	Feed Day	No	11:35	11:00 - 11:59	Late Morning	37,9	92	44,2	44,2	19,05	587,78	127,53
2019/05/27	CH2205	Male	Three y/o	Treatment (Wk 2)	Treatment	Feed Day	No	11:40	11:00 - 11:59	Late Morning	37,6	120	59,6	59,6	18,75	606,78	137,86
2019/05/27	CH2206	Male	Three y/o	Treatment (Wk 2)	Treatment	Feed Day	No	11:40	11:00 - 11:59	Late Morning	37,8	84	47,2	47,2	18,95	581,01	129,80
2019/05/27	CH2205	Male	Three y/o	Treatment (Wk 2)	Treatment	Feed Day	No	11:45	11:00 - 11:59	Late Morning	37,6	62	31,4	31,4	18,75	557,33	115,76
2019/05/27	CH2206	Male	Three y/o	Treatment (Wk 2)	Treatment	Feed Day	No	11:45	11:00 - 11:59	Late Morning	37,7	79	46,8	46,8	18,85	576,36	129,50
2019/05/27	CH2205	Male	Three y/o	Treatment (Wk 2)	Treatment	Feed Day	No	11:50	11:00 - 11:59	Late Morning	37,6		34,0	34,0	18,75		118,49
2019/05/27	CH2206	Male	Three y/o	Treatment (Wk 2)	Treatment	Feed Day	No	11:50	11:00 - 11:59	Late Morning	37,7	48	47,8	47,8	18,85	536,04	130,23
2019/05/27	CH2205	Male	Three y/o	Treatment (Wk 2)	Treatment	Feed Day	No	11:55	11:00 - 11:59	Late Morning	37,5		28,0	28,0	18,65		111,83
2019/05/27	CH2206	Male	Three y/o	Treatment (Wk 2)	Treatment	Feed Day	No	11:55	11:00 - 11:59	Late Morning	37,6	97	39,2	39,2	18,75	591,66	123,39
2019/05/27	CH2205	Male	Three y/o	Treatment (Wk 2)	Treatment	Feed Day	No	12:00	12:00 - 12:59	Afternoon	37,3	65	24,8	24,8	18,46	561,12	107,67
2019/05/27	CH2206	Male	Three y/o	Treatment (Wk 2)	Treatment	Feed Day	No	12:00	12:00 - 12:59	Afternoon	37,7	47	64,2	64,2	18,85	534,23	140,44
2019/05/27	CH2205	Male	Three y/o	Treatment (Wk 2)	Treatment	Feed Day	No	12:05	12:00 - 12:59	Afternoon	37,3	172	33,8	33,8	18,46	630,73	118,29
2019/05/27	CH2206	Male	Three y/o	Treatment (Wk 2)	Treatment	Feed Day	No	12:05	12:00 - 12:59	Afternoon	37,7	50	60,8	60,8	18,85	539,52	138,55
2019/05/27	CH2205	Male	Three y/o	Treatment (Wk 2)	Treatment	Feed Day	No	12:10	12:00 - 12:59	Afternoon	37,2		24,6	24,6	18,36		107,39
2019/05/27	CH2206	Male	Three y/o	Treatment (Wk 2)	Treatment	Feed Day	No	12:10	12:00 - 12:59	Afternoon	37,5	82	62,8	62,8	18,65	579,19	139,67
2019/05/27	CH2205	Male	Three y/o	Treatment (Wk 2)	Treatment	Feed Day	No	12:15	12:00 - 12:59	Afternoon	37,2	61	20,8	20,8	18,36	556,01	101,65

2019/05/27	CH2206	Male	Three y/o	Treatment (Wk 2)	Treatment	Feed Day	No	12:15	12:00 - 12:59	Afternoon	37,5	88	58,8	58,8	18,65	584,49	137,39
2019/05/27	CH2205	Male	Three y/o	Treatment (Wk 2)	Treatment	Feed Day	No	12:20	12:00 - 12:59	Afternoon	37,3	93	22,4	22,4	18,46	588,58	104,19
2019/05/27	CH2206	Male	Three y/o	Treatment (Wk 2)	Treatment	Feed Day	No	12:20	12:00 - 12:59	Afternoon	37,4	66	55,6	55,6	18,55	562,34	135,46
2019/05/27	CH2205	Male	Three y/o	Treatment (Wk 2)	Treatment	Feed Day	No	12:25	12:00 - 12:59	Afternoon	37,4	92	17,6	17,6	18,55	587,78	95,95
2019/05/27	CH2206	Male	Three y/o	Treatment (Wk 2)	Treatment	Feed Day	No	12:25	12:00 - 12:59	Afternoon	37,3	84	3,0	3,0	18,46	581,01	36,34
2019/05/27	CH2205	Male	Three y/o	Treatment (Wk 2)	Treatment	Feed Day	No	12:30	12:00 - 12:59	Afternoon	37,5	50	24,8	24,8	18,65	539,52	107,67
2019/05/27	CH2206	Male	Three y/o	Treatment (Wk 2)	Treatment	Feed Day	No	12:30	12:00 - 12:59	Afternoon	37,4	76	6,2	6,2	18,55	573,39	60,63
2019/05/27	CH2205	Male	Three y/o	Treatment (Wk 2)	Treatment	Feed Day	No	12:35	12:00 - 12:59	Afternoon	37,5	55	20,2	20,2	18,65	547,52	100,65
2019/05/27	CH2206	Male	Three y/o	Treatment (Wk 2)	Treatment	Feed Day	No	12:35	12:00 - 12:59	Afternoon	37,5	90	33,0	33,0	18,65	586,16	117,47
2019/05/27	CH2205	Male	Three y/o	Treatment (Wk 2)	Treatment	Feed Day	No	12:40	12:00 - 12:59	Afternoon	37,5	60	21,4	21,4	18,65	554,67	102,63
2019/05/27	CH2206	Male	Three y/o	Treatment (Wk 2)	Treatment	Feed Day	No	12:40	12:00 - 12:59	Afternoon	37,7	92	36,8	36,8	18,85	587,78	121,22
2019/05/27	CH2205	Male	Three y/o	Treatment (Wk 2)	Treatment	Feed Day	No	12:45	12:00 - 12:59	Afternoon	37,6	88	9,0	9,0	18,75	584,49	73,19
2019/05/27	CH2206	Male	Three y/o	Treatment (Wk 2)	Treatment	Feed Day	No	12:45	12:00 - 12:59	Afternoon	37,8	84	30,2	30,2	18,95	581,01	114,42
2019/05/27	CH2205	Male	Three y/o	Treatment (Wk 2)	Treatment	Feed Day	No	12:50	12:00 - 12:59	Afternoon	37,7	60	11,2	11,2	18,85	554,67	80,59
2019/05/27	CH2206	Male	Three y/o	Treatment (Wk 2)	Treatment	Feed Day	No	12:50	12:00 - 12:59	Afternoon	37,8	105	33,0	33,0	18,95	597,38	117,47
2019/05/27	CH2205	Male	Three y/o	Treatment (Wk 2)	Treatment	Feed Day	No	12:55	12:00 - 12:59	Afternoon	37,7	95	11,6	11,6	18,85	590,14	81,78
2019/05/27	CH2206	Male	Three y/o	Treatment (Wk 2)	Treatment	Feed Day	No	12:55	12:00 - 12:59	Afternoon	37,8		70,4	70,4	18,95		143,64
2019/05/27	CH2205	Male	Three y/o	Treatment (Wk 2)	Treatment	Feed Day	No	13:00	13:00 - 13:59	Afternoon	37,7	70	4,8	4,8	18,85	566,99	52,04
2019/05/27	CH2206	Male	Three y/o	Treatment (Wk 2)	Treatment	Feed Day	No	13:00	13:00 - 13:59	Afternoon	37,8	84	22,6	22,6	18,95	581,01	104,49
2019/05/27	CH2205	Male	Three y/o	Treatment (Wk 2)	Treatment	Feed Day	No	13:05	13:00 - 13:59	Afternoon	37,8	68	4,0	4,0	18,95	564,71	45,94
2019/05/27	CH2206	Male	Three y/o	Treatment (Wk 2)	Treatment	Feed Day	No	13:05	13:00 - 13:59	Afternoon	37,8	83	21,8	21,8	18,95	580,11	103,26
2019/05/27	CH2205	Male	Three y/o	Treatment (Wk 2)	Treatment	Feed Day	No	13:10	13:00 - 13:59	Afternoon	37,9	95	16,4	16,4	19,05	590,14	93,55
2019/05/27	CH2206	Male	Three y/o	Treatment (Wk 2)	Treatment	Feed Day	No	13:10	13:00 - 13:59	Afternoon	37,5	47	22,0	22,0	18,65	534,23	103,57
2019/05/27	CH2205	Male	Three y/o	Treatment (Wk 2)	Treatment	Feed Day	No	13:15	13:00 - 13:59	Afternoon	37,7	177	34,0	34,0	18,85	632,55	118,49
2019/05/27	CH2206	Male	Three y/o	Treatment (Wk 2)	Treatment	Feed Day	No	13:15	13:00 - 13:59	Afternoon	37,5	98	14,8	14,8	18,65	592,41	90,05
2019/05/27	CH2205	Male	Three y/o	Treatment (Wk 2)	Treatment	Feed Day	No	13:20	13:00 - 13:59	Afternoon	37,7	67	19,8	19,8	18,85	563,53	99,97
2019/05/27	CH2206	Male	Three y/o	Treatment (Wk 2)	Treatment	Feed Day	No	13:20	13:00 - 13:59	Afternoon	37,5	81	30,2	30,2	18,65	578,26	114,42
2019/05/27	CH2205	Male	Three y/o	Treatment (Wk 2)	Treatment	Feed Day	No	13:25	13:00 - 13:59	Afternoon	37,6	85	12,8	12,8	18,75	581,90	85,12
2019/05/27	CH2206	Male	Three y/o	Treatment (Wk 2)	Treatment	Feed Day	No	13:25	13:00 - 13:59	Afternoon	37,7	73	34,4	34,4	18,85	570,27	118,90
2019/05/27	CH2205	Male	Three y/o	Treatment (Wk 2)	Treatment	Feed Day	No	13:30	13:00 - 13:59	Afternoon	37,6	68	12,0	12,0	18,75	564,71	82,93
2019/05/27	CH2206	Male	Three y/o	Treatment (Wk 2)	Treatment	Feed Day	No	13:30	13:00 - 13:59	Afternoon	37,8	86	33,6	33,6	18,95	582,77	118,09
2019/05/27	CH2205	Male	Three y/o	Treatment (Wk 2)	Treatment	Feed Day	No	13:35	13:00 - 13:59	Afternoon	37,6	66	13,2	13,2	18,75	562,34	86,17
2019/05/27	CH2206	Male	Three y/o	Treatment (Wk 2)	Treatment	Feed Day	No	13:35	13:00 - 13:59	Afternoon	37,9	93	31,2	31,2	19,05	588,58	115,54
2019/05/27	CH2205	Male	Three y/o	Treatment (Wk 2)	Treatment	Feed Day	No	13:40	13:00 - 13:59	Afternoon	37,6	70	11,4	11,4	18,75	566,99	81,19
2019/05/27	CH2206	Male	Three y/o	Treatment (Wk 2)	Treatment	Feed Day	No	13:40	13:00 - 13:59	Afternoon	37,9	76	27,2	27,2	19,05	573,39	110,83
2019/05/27	CH2205	Male	Three y/o	Treatment (Wk 2)	Treatment	Feed Day	No	13:45	13:00 - 13:59	Afternoon	37,6	77	6,8	6,8	18,75	574,39	63,74
2019/05/27	CH2206	Male	Three y/o	Treatment (Wk 2)	Treatment	Feed Day	No	13:45	13:00 - 13:59	Afternoon	37,9	80	9,4	9,4	19,05	577,31	74,66
2019/05/27	CH2205	Male	Three y/o	Treatment (Wk 2)	Treatment	Feed Day	No	13:50	13:00 - 13:59	Afternoon	37,7	63	3,2	3,2	18,85	558,62	38,49
2019/05/27	CH2206	Male	Three y/o	Treatment (Wk 2)	Treatment	Feed Day	No	13:50	13:00 - 13:59	Afternoon	37,9	47	11,8	11,8	19,05	534,23	82,36
2019/05/27	CH2205	Male	Three y/o	Treatment (Wk 2)	Treatment	Feed Day	No	13:55	13:00 - 13:59	Afternoon	37,7	88	5,2	5,2	18,85	584,49	54,72
2019/05/27	CH2206	Male	Three y/o	Treatment (Wk 2)	Treatment	Feed Day	No	13:55	13:00 - 13:59	Afternoon	37,9	94	18,4	18,4	19,05	589,37	97,47
2019/05/27	CH2205	Male	Three y/o	Treatment (Wk 2)	Treatment	Feed Day	No	14:00	14:00 - 14:59	Afternoon	37,7	113	23,6	23,6	18,85	602,58	105,97
2019/05/27	CH2206	Male	Three y/o	Treatment (Wk 2)	Treatment	Feed Day	No	14:00	14:00 - 14:59	Afternoon	37,9	51	52,6	52,6	19,05	541,20	133,54
2019/05/27	CH2205	Male	Three y/o	Treatment (Wk 2)	Treatment	Feed Day	No	14:05	14:00 - 14:59	Afternoon	37,6	53	21,6	21,6	18,75	544,43	102,94
2019/05/27	CH2206	Male	Three y/o	Treatment (Wk 2)	Treatment	Feed Day	No	14:05	14:00 - 14:59	Afternoon	38,0		75,0	75,0	19,15		145,83
2019/05/27	CH2205	Male	Three y/o	Treatment (Wk 2)	Treatment	Feed Day	No	14:10	14:00 - 14:59	Afternoon	37,6	172	121,4	121,4	18,75	630,73	162,61
2019/05/27	CH2206	Male	Three y/o	Treatment (Wk 2)	Treatment	Feed Day	No	14:10	14:00 - 14:59	Afternoon	38,0		117,8	117,8	19,15		161,56
2019/05/27	CH2205	Male	Three y/o	Treatment (Wk 2)	Treatment	Feed Day	No	14:15	14:00 - 14:59	Afternoon	37,6	104	42,4	42,4	18,75	596,69	126,10
2019/05/27	CH2206	Male	Three y/o	Treatment (Wk 2)	Treatment	Feed Day	No	14:15	14:00 - 14:59	Afternoon	38,0	103	17,2	17,2	19,15	596,00	95,17
2019/05/27	CH2205	Male	Three y/o	Treatment (Wk 2)	Treatment	Feed Day	No	14:20	14:00 - 14:59	Afternoon	37,6	70	32,2	32,2	18,75	566,99	116,63
2019/05/27	CH2206	Male	Three y/o	Treatment (Wk 2)	Treatment	Feed Day	No	14:20	14:00 - 14:59	Afternoon	38,0	91	23,4	23,4	19,15	586,98	105,68
2019/05/27	CH2205	Male	Three y/o	Treatment (Wk 2)	Treatment	Feed Day	No	14:25	14:00 - 14:59	Afternoon	37,7	63	13,2	13,2	18,85	558,62	86,17
2019/05/27	CH2206	Male	Three y/o	Treatment (Wk 2)	Treatment	Feed Day	No	14:25	14:00 - 14:59	Afternoon	38,0	90	14,4	14,4	19,15	586,16	89,12
2019/05/27	CH2205	Male	Three y/o	Treatment (Wk 2)	Treatment	Feed Day	No	14:30	14:00 - 14:59	Afternoon	37,9		3,6	3,6	19,05		42,42
2019/05/27	CH2206	Male	Three y/o	Treatment (Wk 2)	Treatment	Feed Day	No	14:30	14:00 - 14:59	Afternoon	38,0	107	4,0	4,0	19,15	598,73	45,94
2019/05/27	CH2205	Male	Three y/o	Treatment (Wk 2)	Treatment	Feed Day	No	14:35	14:00 - 14:59	Afternoon	37,9	95	79,4	79,4	19,05	590,14	147,81
2019/05/27	CH2206	Male	Three y/o	Treatment (Wk 2)	Treatment	Feed Day	No	14:35	14:00 - 14:59	Afternoon	38,1	96	3,4	3,4	19,26	590,91	40,51
2019/05/27	CH2205	Male	Three y/o	Treatment (Wk 2)	Treatment	Feed Day	No	14:40	14:00 - 14:59	Afternoon	37,9	65	5,0	5,0	19,05	561,12	53,41
2019/05/27	CH2206	Male	Three y/o	Treatment (Wk 2)	Treatment	Feed Day	No	14:40	14:00 - 14:59	Afternoon	38,1	98	5,8	5,8	19,26	592,41	58,39
2019/05/27	CH2205	Male	Three y/o	Treatment (Wk 2)	Treatment	Feed Day	No	14:45	14:00 - 14:59	Afternoon	37,9	72	6,8	6,8	19,05	569,20	63,74
2019/05/27	CH2206	Male	Three y/o	Treatment (Wk 2)	Treatment	Feed Day	No	14:45	14:00 - 14:59	Afternoon	38,1	109	35,2	35,2	19,26	600,04	119,69
2019/05/27	CH2205	Male	Three y/o	Treatment (Wk 2)	Treatment	Feed Day	No	14:50	14:00 - 14:59	Afternoon	38,0	90	4,0	4,0	19,15	586,16	45,94
2019/05/27	CH2206	Male	Three y/o	Treatment (Wk 2)	Treatment	Feed Day	No	14:50	14:00 - 14:59	Afternoon	38,1	71	40,0	40,0	19,26	568,10	124,09
2019/05/27	CH2205	Male	Three y/o	Treatment (Wk 2)	Treatment	Feed Day	No	14:55	14:00 - 14:59	Afternoon	38,0	53	5,4	5,4	19,15	544,43	55,99
2019/05/27	CH2206	Male	Three y/o	Treatment (Wk 2)	Treatment	Feed Day	No	14:55	14:00 - 14:59	Afternoon	38,1	80	45,0	45,0	19,26	577,31	128,15
2019/05/27	CH2205	Male	Three y/o	Treatment (Wk 2)	Treatment	Feed Day	No	15:00	15:00 - 15:59	Afternoon	38,0	49	5,2	5,2	19,15	537,80	54,72
2019/05/27	CH2206	Male	Three y/o	Treatment (Wk 2)	Treatment	Feed Day	No	15:00	15:00 - 15:59	Afternoon	38,1	76	53,2	53,2	19,26	573,39	133,93
2019/05/27	CH2205	Male	Three y/o	Treatment (Wk 2)	Treatment	Feed Day	No	15:05	15:00 - 15:59	Afternoon	38,0	66	7,8	7,8	19,15	562,34	68,36
2019/05/27	CH2206	Male	Three y/o	Treatment (Wk 2)	Treatment	Feed Day	No	15:05	15:00 - 15:59	Afternoon	38,1	63	49,8	49,8	19,26	558,62	131,65
2019/05/27	CH2205	Male	Three y/o	Treatment (Wk 2)	Treatment	Feed Day	No	15:10	15:00 - 15:59	Afternoon	38,0	69	4,0	4,0	19,15	565,86	45,94

2019/05/27	CH2206	Male	Three y/o	Treatment (Wk 2)	Treatment	Feed Day	No	15:10	15:00 - 15:59	Afternoon	38,2	77	48,6	48,6	19,36	574,39	130,81
2019/05/27	CH2205	Male	Three y/o	Treatment (Wk 2)	Treatment	Feed Day	No	15:15	15:00 - 15:59	Afternoon	38,0	72	4,4	4,4	19,15	569,20	49,12
2019/05/27	CH2206	Male	Three y/o	Treatment (Wk 2)	Treatment	Feed Day	No	15:15	15:00 - 15:59	Afternoon	38,2	92	36,6	36,6	19,36	587,78	121,03
2019/05/27	CH2205	Male	Three y/o	Treatment (Wk 2)	Treatment	Feed Day	No	15:20	15:00 - 15:59	Afternoon	38,0	62	4,2	4,2	19,15	557,33	47,57
2019/05/27	CH2206	Male	Three y/o	Treatment (Wk 2)	Treatment	Feed Day	No	15:20	15:00 - 15:59	Afternoon	38,2	97	42,6	42,6	19,36	591,66	126,26
2019/05/27	CH2205	Male	Three y/o	Treatment (Wk 2)	Treatment	Feed Day	No	15:25	15:00 - 15:59	Afternoon	38,0	56	2,4	2,4	19,15	549,01	28,91
2019/05/27	CH2206	Male	Three y/o	Treatment (Wk 2)	Treatment	Feed Day	No	15:25	15:00 - 15:59	Afternoon	38,2	94	48,2	48,2	19,36	589,37	130,52
2019/05/27	CH2205	Male	Three y/o	Treatment (Wk 2)	Treatment	Feed Day	No	15:30	15:00 - 15:59	Afternoon	38,0	83	6,2	6,2	19,15	580,11	60,63
2019/05/27	CH2206	Male	Three y/o	Treatment (Wk 2)	Treatment	Feed Day	No	15:30	15:00 - 15:59	Afternoon	38,2		55,0	55,0	19,36		135,08
2019/05/27	CH2205	Male	Three y/o	Treatment (Wk 2)	Treatment	Feed Day	No	15:35	15:00 - 15:59	Afternoon	38,0	60	2,2	2,2	19,15	554,67	26,02
2019/05/27	CH2206	Male	Three y/o	Treatment (Wk 2)	Treatment	Feed Day	No	15:35	15:00 - 15:59	Afternoon	38,2	86	54,8	54,8	19,36	582,77	134,96
2019/05/27	CH2205	Male	Three y/o	Treatment (Wk 2)	Treatment	Feed Day	No	15:40	15:00 - 15:59	Afternoon	38,0	82	108,6	108,6	19,15	579,19	158,72
2019/05/27	CH2206	Male	Three y/o	Treatment (Wk 2)	Treatment	Feed Day	No	15:40	15:00 - 15:59	Afternoon	38,2	104	138,6	138,6	19,36	596,69	167,25
2019/05/27	CH2205	Male	Three y/o	Treatment (Wk 2)	Treatment	Feed Day	No	15:45	15:00 - 15:59	Afternoon	38,0	91	27,2	27,2	19,15	586,98	110,83
2019/05/27	CH2206	Male	Three y/o	Treatment (Wk 2)	Treatment	Feed Day	No	15:45	15:00 - 15:59	Afternoon	38,2	174	78,2	78,2	19,36	631,46	147,28
2019/05/27	CH2205	Male	Three y/o	Treatment (Wk 2)	Treatment	Feed Day	No	15:50	15:00 - 15:59	Afternoon	37,9	91	35,2	35,2	19,05	586,98	119,69
2019/05/27	CH2206	Male	Three y/o	Treatment (Wk 2)	Treatment	Feed Day	No	15:50	15:00 - 15:59	Afternoon	38,2	113	37,4	37,4	19,36	602,58	121,77
2019/05/27	CH2205	Male	Three y/o	Treatment (Wk 2)	Treatment	Feed Day	No	15:55	15:00 - 15:59	Afternoon	37,9		46,8	46,8	19,05		129,50
2019/05/27	CH2206	Male	Three y/o	Treatment (Wk 2)	Treatment	Feed Day	No	15:55	15:00 - 15:59	Afternoon	38,2	90	44,0	44,0	19,36	586,16	127,37
2019/05/27	CH2205	Male	Three y/o	Treatment (Wk 2)	Treatment	Feed Day	No	16:00	16:00 - 16:59	Evening	37,8	110	3,6	3,6	18,95	600,69	42,42
2019/05/27	CH2206	Male	Three y/o	Treatment (Wk 2)	Treatment	Feed Day	No	16:00	16:00 - 16:59	Evening	38,1	84	46,6	46,6	19,26	581,01	129,35
2019/05/27	CH2205	Male	Three y/o	Treatment (Wk 2)	Treatment	Feed Day	No	16:05	16:00 - 16:59	Evening	37,9	62	13,6	13,6	19,05	557,33	87,18
2019/05/27	CH2206	Male	Three y/o	Treatment (Wk 2)	Treatment	Feed Day	No	16:05	16:00 - 16:59	Evening	38,1	50	41,6	41,6	19,26	539,52	125,44
2019/05/27	CH2205	Male	Three y/o	Treatment (Wk 2)	Treatment	Feed Day	No	16:10	16:00 - 16:59	Evening	37,8	100	94,4	94,4	18,95	593,87	153,84
2019/05/27	CH2206	Male	Three y/o	Treatment (Wk 2)	Treatment	Feed Day	No	16:10	16:00 - 16:59	Evening	38,1	63	59,0	59,0	19,26	558,62	137,51
2019/05/27	CH2205	Male	Three y/o	Treatment (Wk 2)	Treatment	Feed Day	No	16:15	16:00 - 16:59	Evening	37,9	72	20,6	20,6	19,05	569,20	101,32
2019/05/27	CH2206	Male	Three y/o	Treatment (Wk 2)	Treatment	Feed Day	No	16:15	16:00 - 16:59	Evening	38,1	90	7,2	7,2	19,26	586,16	65,66
2019/05/27	CH2205	Male	Three y/o	Treatment (Wk 2)	Treatment	Feed Day	No	16:20	16:00 - 16:59	Evening	37,9		19,4	19,4	19,05		99,28
2019/05/27	CH2206	Male	Three y/o	Treatment (Wk 2)	Treatment	Feed Day	No	16:20	16:00 - 16:59	Evening	38,0	74	15,2	15,2	19,15	571,33	90,96
2019/05/27	CH2205	Male	Three y/o	Treatment (Wk 2)	Treatment	Feed Day	No	16:25	16:00 - 16:59	Evening	37,8		40,2	40,2	18,95		124,26
2019/05/27	CH2206	Male	Three y/o	Treatment (Wk 2)	Treatment	Feed Day	No	16:25	16:00 - 16:59	Evening	38,0	87	29,2	29,2	19,15	583,64	113,27
2019/05/27	CH2205	Male	Three y/o	Treatment (Wk 2)	Treatment	Feed Day	No	16:30	16:00 - 16:59	Evening	37,7	60	62,8	62,8	18,85	554,67	139,67
2019/05/27	CH2206	Male	Three y/o	Treatment (Wk 2)	Treatment	Feed Day	No	16:30	16:00 - 16:59	Evening	38,0	147	264,4	264,4	19,15	620,52	189,96
2019/05/27	CH2205	Male	Three y/o	Treatment (Wk 2)	Treatment	Feed Day	No	16:35	16:00 - 16:59	Evening	37,7	83	26,0	26,0	18,85	580,11	109,29
2019/05/27	CH2206	Male	Three y/o	Treatment (Wk 2)	Treatment	Feed Day	No	16:35	16:00 - 16:59	Evening	38,1	104	49,4	49,4	19,26	596,69	131,37
2019/05/27	CH2205	Male	Three y/o	Treatment (Wk 2)	Treatment	Feed Day	No	16:40	16:00 - 16:59	Evening	37,7		190,8	190,8	18,85		178,46
2019/05/27	CH2206	Male	Three y/o	Treatment (Wk 2)	Treatment	Feed Day	No	16:40	16:00 - 16:59	Evening	38,1	123	144,8	144,8	19,26	608,49	168,78
2019/05/27	CH2205	Male	Three y/o	Treatment (Wk 2)	Treatment	Feed Day	No	16:45	16:00 - 16:59	Evening	37,9		14,2	14,2	19,05		88,65
2019/05/27	CH2206	Male	Three y/o	Treatment (Wk 2)	Treatment	Feed Day	No	16:45	16:00 - 16:59	Evening	38,0		53,2	53,2	19,15		133,93
2019/05/27	CH2205	Male	Three y/o	Treatment (Wk 2)	Treatment	Feed Day	No	16:50	16:00 - 16:59	Evening	37,9	148	40,8	40,8	19,05	620,97	124,77
2019/05/27	CH2206	Male	Three y/o	Treatment (Wk 2)	Treatment	Feed Day	No	16:50	16:00 - 16:59	Evening	38,0		184,2	184,2	19,15		177,23
2019/05/27	CH2205	Male	Three y/o	Treatment (Wk 2)	Treatment	Feed Day	No	16:55	16:00 - 16:59	Evening	38,0	65	153,6	153,6	19,15	561,12	170,85
2019/05/27	CH2206	Male	Three y/o	Treatment (Wk 2)	Treatment	Feed Day	No	16:55	16:00 - 16:59	Evening	38,1	92	122,0	122,0	19,26	587,78	162,79
2019/05/27	CH2205	Male	Three y/o	Treatment (Wk 2)	Treatment	Feed Day	No	17:00	17:00 - 17:59	Evening	38,2	44	70,2	70,2	19,36	528,51	143,54
2019/05/27	CH2206	Male	Three y/o	Treatment (Wk 2)	Treatment	Feed Day	No	17:00	17:00 - 17:59	Evening	38,3	64	157,2	157,2	19,46	559,88	171,66
2019/05/27	CH2205	Male	Three y/o	Treatment (Wk 2)	Treatment	Feed Day	No	17:05	17:00 - 17:59	Evening	38,4	101	58,2	58,2	19,56	594,59	137,04
2019/05/27	CH2206	Male	Three y/o	Treatment (Wk 2)	Treatment	Feed Day	No	17:05	17:00 - 17:59	Evening	38,2	97	10,0	10,0	19,36	591,66	76,76
2019/05/27	CH2205	Male	Three y/o	Treatment (Wk 2)	Treatment	Feed Day	No	17:10	17:00 - 17:59	Evening	38,3	119	123,8	123,8	19,46	606,20	163,30
2019/05/27	CH2206	Male	Three y/o	Treatment (Wk 2)	Treatment	Feed Day	No	17:10	17:00 - 17:59	Evening	38,2	95	154,2	154,2	19,36	590,14	170,99
2019/05/27	CH2205	Male	Three y/o	Treatment (Wk 2)	Treatment	Feed Day	No	17:15	17:00 - 17:59	Evening	38,4		185,2	185,2	19,56		177,42
2019/05/27	CH2206	Male	Three y/o	Treatment (Wk 2)	Treatment	Feed Day	No	17:15	17:00 - 17:59	Evening	38,2		99,8	99,8	19,36		155,77
2019/05/27	CH2205	Male	Three y/o	Treatment (Wk 2)	Treatment	Feed Day	No	17:20	17:00 - 17:59	Evening	38,4	85	27,8	27,8	19,56	581,90	111,58
2019/05/27	CH2206	Male	Three y/o	Treatment (Wk 2)	Treatment	Feed Day	No	17:20	17:00 - 17:59	Evening	38,4	129	110,4	110,4	19,56	611,75	159,30
2019/05/27	CH2205	Male	Three y/o	Treatment (Wk 2)	Treatment	Feed Day	No	17:25	17:00 - 17:59	Evening	38,4		37,2	37,2	19,56		121,59
2019/05/27	CH2206	Male	Three y/o	Treatment (Wk 2)	Treatment	Feed Day	No	17:25	17:00 - 17:59	Evening	38,3	92	7,6	7,6	19,46	587,78	67,49
2019/05/27	CH2205	Male	Three y/o	Treatment (Wk 2)	Treatment	Feed Day	No	17:30	17:00 - 17:59	Evening	38,2	103	27,8	27,8	19,36	596,00	111,58
2019/05/27	CH2206	Male	Three y/o	Treatment (Wk 2)	Treatment	Feed Day	No	17:30	17:00 - 17:59	Evening	38,0	93	39,8	39,8	19,15	588,58	123,91
2019/05/27	CH2205	Male	Three y/o	Treatment (Wk 2)	Treatment	Feed Day	No	17:35	17:00 - 17:59	Evening	38,2		24,0	24,0	19,36		106,55
2019/05/27	CH2206	Male	Three y/o	Treatment (Wk 2)	Treatment	Feed Day	No	17:35	17:00 - 17:59	Evening	38,0	98	7,2	7,2	19,15	592,41	65,66
2019/05/27	CH2205	Male	Three y/o	Treatment (Wk 2)	Treatment	Feed Day	No	17:40	17:00 - 17:59	Evening	38,2	119	52,6	52,6	19,36	606,20	133,54
2019/05/27	CH2206	Male	Three y/o	Treatment (Wk 2)	Treatment	Feed Day	No	17:40	17:00 - 17:59	Evening	38,1	53	57,8	57,8	19,26	544,43	136,80
2019/05/27	CH2205	Male	Three y/o	Treatment (Wk 2)	Treatment	Feed Day	No	17:45	17:00 - 17:59	Evening	38,2	99	12,2	12,2	19,36	593,14	83,49
2019/05/27	CH2206	Male	Three y/o	Treatment (Wk 2)	Treatment	Feed Day	No	17:45	17:00 - 17:59	Evening	38,1	92	36,2	36,2	19,26	587,78	120,65
2019/05/27	CH2205	Male	Three y/o	Treatment (Wk 2)	Treatment	Feed Day	No	17:50	17:00 - 17:59	Evening	38,0		25,6	25,6	19,15		108,76
2019/05/27	CH2206	Male	Three y/o	Treatment (Wk 2)	Treatment	Feed Day	No	17:50	17:00 - 17:59	Evening	38,0	83	42,0	42,0	19,15	580,11	125,77
2019/05/27	CH2205	Male	Three y/o	Treatment (Wk 2)	Treatment	Feed Day	No	17:55	17:00 - 17:59	Evening	37,9		21,8	21,8	19,05		103,26
2019/05/27	CH2206	Male	Three y/o	Treatment (Wk 2)	Treatment	Feed Day	No	17:55	17:00 - 17:59	Evening	37,9	69	27,0	27,0	19,05	565,86	110,58
2019/05/27	CH2205	Male	Three y/o	Treatment (Wk 2)	Treatment	Feed Day	No	18:00	18:00 - 18:59	Evening	37,9	140	27,6	27,6	19,05	617,28	111,33
2019/05/27	CH2206	Male	Three y/o	Treatment (Wk 2)	Treatment	Feed Day	No	18:00	18:00 - 18:59	Evening	37,8	94	29,0	29,0	18,95	589,37	113,03
2019/05/27	CH2205	Male	Three y/o	Treatment (Wk 2)	Treatment	Feed Day	No	18:05	18:00 - 18:59	Evening	37,7	80	26,2	26,2	18,85	577,31	109,55

2019/05/27	CH2206	Male	Three y/o	Treatment (Wk 2)	Treatment	Feed Day	No	18:05	18:00 - 18:59	Evening	37,8	99	21,4	21,4	18,95	593,14	102,63
2019/05/27	CH2205	Male	Three y/o	Treatment (Wk 2)	Treatment	Feed Day	No	18:10	18:00 - 18:59	Evening	37,8	86	21,0	21,0	18,95	582,77	101,98
2019/05/27	CH2206	Male	Three y/o	Treatment (Wk 2)	Treatment	Feed Day	No	18:10	18:00 - 18:59	Evening	37,7	99	18,4	18,4	18,85	593,14	97,47
2019/05/27	CH2205	Male	Three y/o	Treatment (Wk 2)	Treatment	Feed Day	No	18:15	18:00 - 18:59	Evening	38,1	74	22,4	22,4	19,26	571,33	104,19
2019/05/27	CH2206	Male	Three y/o	Treatment (Wk 2)	Treatment	Feed Day	No	18:15	18:00 - 18:59	Evening	37,5	94	15,0	15,0	18,65	589,37	90,51
2019/05/27	CH2205	Male	Three y/o	Treatment (Wk 2)	Treatment	Feed Day	No	18:20	18:00 - 18:59	Evening	38,2	87	22,4	22,4	19,36	583,64	104,19
2019/05/27	CH2206	Male	Three y/o	Treatment (Wk 2)	Treatment	Feed Day	No	18:20	18:00 - 18:59	Evening	37,5	97	24,2	24,2	18,65	591,66	106,83
2019/05/27	CH2205	Male	Three y/o	Treatment (Wk 2)	Treatment	Feed Day	No	18:25	18:00 - 18:59	Evening	38,2	73	22,8	22,8	19,36	570,27	104,79
2019/05/27	CH2206	Male	Three y/o	Treatment (Wk 2)	Treatment	Feed Day	No	18:25	18:00 - 18:59	Evening	37,5	89	28,6	28,6	18,65	585,33	112,56
2019/05/27	CH2205	Male	Three y/o	Treatment (Wk 2)	Treatment	Feed Day	No	18:30	18:00 - 18:59	Evening	38,2	72	20,6	20,6	19,36	569,20	101,32
2019/05/27	CH2206	Male	Three y/o	Treatment (Wk 2)	Treatment	Feed Day	No	18:30	18:00 - 18:59	Evening	37,7	128	107,4	107,4	18,85	611,22	158,33
2019/05/27	CH2205	Male	Three y/o	Treatment (Wk 2)	Treatment	Feed Day	No	18:35	18:00 - 18:59	Evening	38,3	90	18,2	18,2	19,46	586,16	97,10
2019/05/27	CH2206	Male	Three y/o	Treatment (Wk 2)	Treatment	Feed Day	No	18:35	18:00 - 18:59	Evening	37,9	84	17,2	17,2	19,05	581,01	95,17
2019/05/27	CH2205	Male	Three y/o	Treatment (Wk 2)	Treatment	Feed Day	No	18:40	18:00 - 18:59	Evening	38,3	68	23,6	23,6	19,46	564,71	105,97
2019/05/27	CH2206	Male	Three y/o	Treatment (Wk 2)	Treatment	Feed Day	No	18:40	18:00 - 18:59	Evening	37,9	80	19,6	19,6	19,05	577,31	99,63
2019/05/27	CH2205	Male	Three y/o	Treatment (Wk 2)	Treatment	Feed Day	No	18:45	18:00 - 18:59	Evening	38,3	68	16,8	16,8	19,46	564,71	94,37
2019/05/27	CH2206	Male	Three y/o	Treatment (Wk 2)	Treatment	Feed Day	No	18:45	18:00 - 18:59	Evening	37,9	77	16,2	16,2	19,05	574,39	93,13
2019/05/27	CH2205	Male	Three y/o	Treatment (Wk 2)	Treatment	Feed Day	No	18:50	18:00 - 18:59	Evening	38,3	73	16,2	16,2	19,46	570,27	93,13
2019/05/27	CH2206	Male	Three y/o	Treatment (Wk 2)	Treatment	Feed Day	No	18:50	18:00 - 18:59	Evening	38,0	67	23,4	23,4	19,15	563,53	105,68
2019/05/27	CH2205	Male	Three y/o	Treatment (Wk 2)	Treatment	Feed Day	No	18:55	18:00 - 18:59	Evening	38,3	66	19,6	19,6	19,46	562,34	99,63
2019/05/27	CH2206	Male	Three y/o	Treatment (Wk 2)	Treatment	Feed Day	No	18:55	18:00 - 18:59	Evening	38,0	77	28,2	28,2	19,15	574,39	112,07
2019/05/27	CH2205	Male	Three y/o	Treatment (Wk 2)	Treatment	Feed Day	No	19:00	19:00 - 19:59	Evening	38,3	77	18,8	18,8	19,46	574,39	98,20
2019/05/27	CH2206	Male	Three y/o	Treatment (Wk 2)	Treatment	Feed Day	No	19:00	19:00 - 19:59	Evening	38,0	83	33,0	33,0	19,15	580,11	117,47
2019/05/27	CH2205	Male	Three y/o	Treatment (Wk 2)	Treatment	Feed Day	No	19:05	19:00 - 19:59	Evening	38,3	63	19,4	19,4	19,46	558,62	99,28
2019/05/27	CH2206	Male	Three y/o	Treatment (Wk 2)	Treatment	Feed Day	No	19:05	19:00 - 19:59	Evening	38,1	79	27,8	27,8	19,26	576,36	111,58
2019/05/27	CH2205	Male	Three y/o	Treatment (Wk 2)	Treatment	Feed Day	No	19:10	19:00 - 19:59	Evening	38,3	54	20,4	20,4	19,46	545,99	100,99
2019/05/27	CH2206	Male	Three y/o	Treatment (Wk 2)	Treatment	Feed Day	No	19:10	19:00 - 19:59	Evening	38,1	67	26,0	26,0	19,26	563,53	109,29
2019/05/27	CH2205	Male	Three y/o	Treatment (Wk 2)	Treatment	Feed Day	No	19:15	19:00 - 19:59	Evening	38,3	83	20,0	20,0	19,46	580,11	100,31
2019/05/27	CH2206	Male	Three y/o	Treatment (Wk 2)	Treatment	Feed Day	No	19:15	19:00 - 19:59	Evening	38,1	78	24,8	24,8	19,26	575,38	107,67
2019/05/27	CH2205	Male	Three y/o	Treatment (Wk 2)	Treatment	Feed Day	No	19:20	19:00 - 19:59	Evening	38,2	78	16,4	16,4	19,36	575,38	93,55
2019/05/27	CH2206	Male	Three y/o	Treatment (Wk 2)	Treatment	Feed Day	No	19:20	19:00 - 19:59	Evening	38,1	94	12,6	12,6	19,26	589,37	84,59
2019/05/27	CH2205	Male	Three y/o	Treatment (Wk 2)	Treatment	Feed Day	No	19:25	19:00 - 19:59	Evening	38,2	62	16,4	16,4	19,36	557,33	93,55
2019/05/27	CH2206	Male	Three y/o	Treatment (Wk 2)	Treatment	Feed Day	No	19:25	19:00 - 19:59	Evening	38,1	148	13,0	13,0	19,26	620,97	85,65
2019/05/27	CH2205	Male	Three y/o	Treatment (Wk 2)	Treatment	Feed Day	No	19:30	19:00 - 19:59	Evening	38,2	75	20,0	20,0	19,36	572,36	100,31
2019/05/27	CH2206	Male	Three y/o	Treatment (Wk 2)	Treatment	Feed Day	No	19:30	19:00 - 19:59	Evening	38,1	80	6,8	6,8	19,26	577,31	63,74
2019/05/27	CH2205	Male	Three y/o	Treatment (Wk 2)	Treatment	Feed Day	No	19:35	19:00 - 19:59	Evening	38,2	40	24,0	24,0	19,36	520,09	106,55
2019/05/27	CH2206	Male	Three y/o	Treatment (Wk 2)	Treatment	Feed Day	No	19:35	19:00 - 19:59	Evening	38,1	78	4,6	4,6	19,26	575,38	50,61
2019/05/27	CH2205	Male	Three y/o	Treatment (Wk 2)	Treatment	Feed Day	No	19:40	19:00 - 19:59	Evening	38,2		24,0	24,0	19,36		106,55
2019/05/27	CH2206	Male	Three y/o	Treatment (Wk 2)	Treatment	Feed Day	No	19:40	19:00 - 19:59	Evening	38,1	64	3,6	3,6	19,26	559,88	42,42
2019/05/27	CH2205	Male	Three y/o	Treatment (Wk 2)	Treatment	Feed Day	No	19:45	19:00 - 19:59	Evening	38,2	57	20,8	20,8	19,36	550,47	101,65
2019/05/27	CH2206	Male	Three y/o	Treatment (Wk 2)	Treatment	Feed Day	No	19:45	19:00 - 19:59	Evening	38,1	78	8,0	8,0	19,26	575,38	69,22
2019/05/27	CH2205	Male	Three y/o	Treatment (Wk 2)	Treatment	Feed Day	No	19:50	19:00 - 19:59	Evening	38,1	51	19,6	19,6	19,26	541,20	99,63
2019/05/27	CH2206	Male	Three y/o	Treatment (Wk 2)	Treatment	Feed Day	No	19:50	19:00 - 19:59	Evening	38,1	69	7,2	7,2	19,26	565,86	65,66
2019/05/27	CH2205	Male	Three y/o	Treatment (Wk 2)	Treatment	Feed Day	No	19:55	19:00 - 19:59	Evening	38,1	93	20,4	20,4	19,26	588,58	100,99
2019/05/27	CH2206	Male	Three y/o	Treatment (Wk 2)	Treatment	Feed Day	No	19:55	19:00 - 19:59	Evening	38,1	75	3,8	3,8	19,26	572,36	44,22
2019/05/27	CH2205	Male	Three y/o	Treatment (Wk 2)	Treatment	Feed Day	No	20:00	20:00 - 20:59	Night	38,1		6,4	6,4	19,26		61,70
2019/05/27	CH2206	Male	Three y/o	Treatment (Wk 2)	Treatment	Feed Day	No	20:00	20:00 - 20:59	Night	38,1	86	3,6	3,6	19,26	582,77	42,42
2019/05/27	CH2205	Male	Three y/o	Treatment (Wk 2)	Treatment	Feed Day	No	20:05	20:00 - 20:59	Night	38,1	61	3,2	3,2	19,26	556,01	38,49
2019/05/27	CH2206	Male	Three y/o	Treatment (Wk 2)	Treatment	Feed Day	No	20:05	20:00 - 20:59	Night	38,0	75	14,0	14,0	19,15	572,36	88,17
2019/05/27	CH2205	Male	Three y/o	Treatment (Wk 2)	Treatment	Feed Day	No	20:10	20:00 - 20:59	Night	38,1	46	5,2	5,2	19,26	532,38	54,72
2019/05/27	CH2206	Male	Three y/o	Treatment (Wk 2)	Treatment	Feed Day	No	20:10	20:00 - 20:59	Night	38,0	74	15,4	15,4	19,15	571,33	91,41
2019/05/27	CH2205	Male	Three y/o	Treatment (Wk 2)	Treatment	Feed Day	No	20:15	20:00 - 20:59	Night	38,1	92	7,0	7,0	19,26	587,78	64,72
2019/05/27	CH2206	Male	Three y/o	Treatment (Wk 2)	Treatment	Feed Day	No	20:15	20:00 - 20:59	Night	38,0	86	10,2	10,2	19,15	582,77	77,43
2019/05/27	CH2205	Male	Three y/o	Treatment (Wk 2)	Treatment	Feed Day	No	20:20	20:00 - 20:59	Night	38,1	85	2,8	2,8	19,26	581,90	34,04
2019/05/27	CH2206	Male	Three y/o	Treatment (Wk 2)	Treatment	Feed Day	No	20:20	20:00 - 20:59	Night	38,0	86	10,2	10,2	19,15	582,77	77,43
2019/05/27	CH2205	Male	Three y/o	Treatment (Wk 2)	Treatment	Feed Day	No	20:25	20:00 - 20:59	Night	38,1	65	5,2	5,2	19,26	561,12	54,72
2019/05/27	CH2206	Male	Three y/o	Treatment (Wk 2)	Treatment	Feed Day	No	20:25	20:00 - 20:59	Night	38,0	48	8,0	8,0	19,15	536,04	69,22
2019/05/27	CH2205	Male	Three y/o	Treatment (Wk 2)	Treatment	Feed Day	No	20:30	20:00 - 20:59	Night	38,1	41	5,0	5,0	19,26	522,29	53,41
2019/05/27	CH2206	Male	Three y/o	Treatment (Wk 2)	Treatment	Feed Day	No	20:30	20:00 - 20:59	Night	38,0	75	11,8	11,8	19,15	572,36	82,36
2019/05/27	CH2205	Male	Three y/o	Treatment (Wk 2)	Treatment	Feed Day	No	20:35	20:00 - 20:59	Night	38,1	39	3,6	3,6	19,26	517,82	42,42
2019/05/27	CH2206	Male	Three y/o	Treatment (Wk 2)	Treatment	Feed Day	No	20:35	20:00 - 20:59	Night	38,0	85	10,0	10,0	19,15	581,90	76,76
2019/05/27	CH2205	Male	Three y/o	Treatment (Wk 2)	Treatment	Feed Day	No	20:40	20:00 - 20:59	Night	38,1	44	3,2	3,2	19,26	528,51	38,49
2019/05/27	CH2206	Male	Three y/o	Treatment (Wk 2)	Treatment	Feed Day	No	20:40	20:00 - 20:59	Night	38,0	75	8,6	8,6	19,15	572,36	71,66
2019/05/27	CH2205	Male	Three y/o	Treatment (Wk 2)	Treatment	Feed Day	No	20:45	20:00 - 20:59	Night	38,1	54	4,2	4,2	19,26	545,99	47,57
2019/05/27	CH2206	Male	Three y/o	Treatment (Wk 2)	Treatment	Feed Day	No	20:45	20:00 - 20:59	Night	38,0	84	4,2	4,2	19,15	581,01	47,57
2019/05/27	CH2205	Male	Three y/o	Treatment (Wk 2)	Treatment	Feed Day	No	20:50	20:00 - 20:59	Night	38,1	62	5,6	5,6	19,26	557,33	57,21
2019/05/27	CH2206	Male	Three y/o	Treatment (Wk 2)	Treatment	Feed Day	No	20:50	20:00 - 20:59	Night	38,0	59	7,2	7,2	19,15	553,30	65,66
2019/05/27	CH2205	Male	Three y/o	Treatment (Wk 2)	Treatment	Feed Day	No	20:55	20:00 - 20:59	Night	38,1	41	6,0	6,0	19,26	522,29	59,53
2019/05/27	CH2206	Male	Three y/o	Treatment (Wk 2)	Treatment	Feed Day	No	20:55	20:00 - 20:59	Night	37,9	85	8,8	8,8	19,05	581,90	72,43
2019/05/27	CH2205	Male	Three y/o	Treatment (Wk 2)	Treatment	Feed Day	No	21:00	21:00 - 21:59	Night	38,1	73	6,0	6,0	19,26	570,27	59,53

2019/05/27	CH2206	Male	Three y/o	Treatment (Wk 2)	Treatment	Feed Day	No	21:00	21:00 - 21:59	Night	37,9	68	6,0	6,0	19,05	564,71	59,53
2019/05/27	CH2205	Male	Three y/o	Treatment (Wk 2)	Treatment	Feed Day	No	21:05	21:00 - 21:59	Night	38,1	48	4,4	4,4	19,26	536,04	49,12
2019/05/27	CH2206	Male	Three y/o	Treatment (Wk 2)	Treatment	Feed Day	No	21:05	21:00 - 21:59	Night	37,9	59	3,4	3,4	19,05	553,30	40,51
2019/05/27	CH2205	Male	Three y/o	Treatment (Wk 2)	Treatment	Feed Day	No	21:10	21:00 - 21:59	Night	38,1	74	24,2	24,2	19,26	571,33	106,83
2019/05/27	CH2206	Male	Three y/o	Treatment (Wk 2)	Treatment	Feed Day	No	21:10	21:00 - 21:59	Night	37,9	59	15,6	15,6	19,05	553,30	91,85
2019/05/27	CH2205	Male	Three y/o	Treatment (Wk 2)	Treatment	Feed Day	No	21:15	21:00 - 21:59	Night	38,1		30,2	30,2	19,26		114,42
2019/05/27	CH2206	Male	Three y/o	Treatment (Wk 2)	Treatment	Feed Day	No	21:15	21:00 - 21:59	Night	38,0	100	63,4	63,4	19,15	593,87	140,00
2019/05/27	CH2205	Male	Three y/o	Treatment (Wk 2)	Treatment	Feed Day	No	21:20	21:00 - 21:59	Night	38,0	145	19,2	19,2	19,15	619,62	98,92
2019/05/27	CH2206	Male	Three y/o	Treatment (Wk 2)	Treatment	Feed Day	No	21:20	21:00 - 21:59	Night	37,8	105	55,0	55,0	18,95	597,38	135,08
2019/05/27	CH2205	Male	Three y/o	Treatment (Wk 2)	Treatment	Feed Day	No	21:25	21:00 - 21:59	Night	37,9	94	14,8	14,8	19,05	589,37	90,05
2019/05/27	CH2206	Male	Three y/o	Treatment (Wk 2)	Treatment	Feed Day	No	21:25	21:00 - 21:59	Night	37,8	92	25,8	25,8	18,95	587,78	109,02
2019/05/27	CH2205	Male	Three y/o	Treatment (Wk 2)	Treatment	Feed Day	No	21:30	21:00 - 21:59	Night	37,7	45	16,4	16,4	18,85	530,47	93,55
2019/05/27	CH2206	Male	Three y/o	Treatment (Wk 2)	Treatment	Feed Day	No	21:30	21:00 - 21:59	Night	37,8	71	25,6	25,6	18,95	568,10	108,76
2019/05/27	CH2205	Male	Three y/o	Treatment (Wk 2)	Treatment	Feed Day	No	21:35	21:00 - 21:59	Night	37,6	59	17,6	17,6	18,75	553,30	95,95
2019/05/27	CH2206	Male	Three y/o	Treatment (Wk 2)	Treatment	Feed Day	No	21:35	21:00 - 21:59	Night	37,8	66	30,4	30,4	18,95	562,34	114,65
2019/05/27	CH2205	Male	Three y/o	Treatment (Wk 2)	Treatment	Feed Day	No	21:40	21:00 - 21:59	Night	37,6	58	21,4	21,4	18,75	551,90	102,63
2019/05/27	CH2206	Male	Three y/o	Treatment (Wk 2)	Treatment	Feed Day	No	21:40	21:00 - 21:59	Night	37,8	66	24,4	24,4	18,95	562,34	107,11
2019/05/27	CH2205	Male	Three y/o	Treatment (Wk 2)	Treatment	Feed Day	No	21:45	21:00 - 21:59	Night	37,6	65	17,8	17,8	18,75	561,12	96,34
2019/05/27	CH2206	Male	Three y/o	Treatment (Wk 2)	Treatment	Feed Day	No	21:45	21:00 - 21:59	Night	37,8	83	12,0	12,0	18,95	580,11	82,93
2019/05/27	CH2205	Male	Three y/o	Treatment (Wk 2)	Treatment	Feed Day	No	21:50	21:00 - 21:59	Night	37,6	84	26,0	26,0	18,75	581,01	109,29
2019/05/27	CH2206	Male	Three y/o	Treatment (Wk 2)	Treatment	Feed Day	No	21:50	21:00 - 21:59	Night	37,8	103	10,0	10,0	18,95	596,00	76,76
2019/05/27	CH2205	Male	Three y/o	Treatment (Wk 2)	Treatment	Feed Day	No	21:55	21:00 - 21:59	Night	37,5	74	27,0	27,0	18,65	571,33	110,58
2019/05/27	CH2206	Male	Three y/o	Treatment (Wk 2)	Treatment	Feed Day	No	21:55	21:00 - 21:59	Night	37,8	89	20,6	20,6	18,95	585,33	101,32
2019/05/27	CH2205	Male	Three y/o	Treatment (Wk 2)	Treatment	Feed Day	No	22:00	22:00 - 22:59	Night	37,4	72	22,4	22,4	18,55	569,20	104,19
2019/05/27	CH2206	Male	Three y/o	Treatment (Wk 2)	Treatment	Feed Day	No	22:00	22:00 - 22:59	Night	37,8	90	20,6	20,6	18,95	586,16	101,32
2019/05/27	CH2205	Male	Three y/o	Treatment (Wk 2)	Treatment	Feed Day	No	22:05	22:00 - 22:59	Night	37,3	54	17,2	17,2	18,46	545,99	95,17
2019/05/27	CH2206	Male	Three y/o	Treatment (Wk 2)	Treatment	Feed Day	No	22:05	22:00 - 22:59	Night	37,8	97	18,8	18,8	18,95	591,66	98,20
2019/05/27	CH2205	Male	Three y/o	Treatment (Wk 2)	Treatment	Feed Day	No	22:10	22:00 - 22:59	Night	37,3	109	30,4	30,4	18,46	600,04	114,65
2019/05/27	CH2206	Male	Three y/o	Treatment (Wk 2)	Treatment	Feed Day	No	22:10	22:00 - 22:59	Night	37,8	86	14,8	14,8	18,95	582,77	90,05
2019/05/27	CH2205	Male	Three y/o	Treatment (Wk 2)	Treatment	Feed Day	No	22:15	22:00 - 22:59	Night	37,4		31,2	31,2	18,55		115,54
2019/05/27	CH2206	Male	Three y/o	Treatment (Wk 2)	Treatment	Feed Day	No	22:15	22:00 - 22:59	Night	37,8	81	7,4	7,4	18,95	578,26	66,59
2019/05/27	CH2205	Male	Three y/o	Treatment (Wk 2)	Treatment	Feed Day	No	22:20	22:00 - 22:59	Night	37,3	154	33,4	33,4	18,46	623,58	117,88
2019/05/27	CH2206	Male	Three y/o	Treatment (Wk 2)	Treatment	Feed Day	No	22:20	22:00 - 22:59	Night	37,8	52	7,0	7,0	18,95	542,83	64,72
2019/05/27	CH2205	Male	Three y/o	Treatment (Wk 2)	Treatment	Feed Day	No	22:25	22:00 - 22:59	Night	37,4		33,6	33,6	18,55		118,09
2019/05/27	CH2206	Male	Three y/o	Treatment (Wk 2)	Treatment	Feed Day	No	22:25	22:00 - 22:59	Night	37,8	76	8,2	8,2	18,95	573,39	70,05
2019/05/27	CH2205	Male	Three y/o	Treatment (Wk 2)	Treatment	Feed Day	No	22:30	22:00 - 22:59	Night	37,5	77	27,4	27,4	18,65	574,39	111,09
2019/05/27	CH2206	Male	Three y/o	Treatment (Wk 2)	Treatment	Feed Day	No	22:30	22:00 - 22:59	Night	37,7	82	7,4	7,4	18,85	579,19	66,59
2019/05/27	CH2205	Male	Three y/o	Treatment (Wk 2)	Treatment	Feed Day	No	22:35	22:00 - 22:59	Night	37,7	92	30,0	30,0	18,85	587,78	114,20
2019/05/27	CH2206	Male	Three y/o	Treatment (Wk 2)	Treatment	Feed Day	No	22:35	22:00 - 22:59	Night	37,7	46	15,0	15,0	18,85	532,38	90,51
2019/05/27	CH2205	Male	Three y/o	Treatment (Wk 2)	Treatment	Feed Day	No	22:40	22:00 - 22:59	Night	37,7	54	28,0	28,0	18,85	545,99	111,83
2019/05/27	CH2206	Male	Three y/o	Treatment (Wk 2)	Treatment	Feed Day	No	22:40	22:00 - 22:59	Night	37,5	88	13,0	13,0	18,65	584,49	85,65
2019/05/27	CH2205	Male	Three y/o	Treatment (Wk 2)	Treatment	Feed Day	No	22:45	22:00 - 22:59	Night	37,9	58	24,6	24,6	19,05	551,90	107,39
2019/05/27	CH2206	Male	Three y/o	Treatment (Wk 2)	Treatment	Feed Day	No	22:45	22:00 - 22:59	Night	37,7	90	7,4	7,4	18,85	586,16	66,59
2019/05/27	CH2205	Male	Three y/o	Treatment (Wk 2)	Treatment	Feed Day	No	22:50	22:00 - 22:59	Night	37,9	92	28,6	28,6	19,05	587,78	112,56
2019/05/27	CH2206	Male	Three y/o	Treatment (Wk 2)	Treatment	Feed Day	No	22:50	22:00 - 22:59	Night	37,7	80	6,8	6,8	18,85	577,31	63,74
2019/05/27	CH2205	Male	Three y/o	Treatment (Wk 2)	Treatment	Feed Day	No	22:55	22:00 - 22:59	Night	37,9	60	22,8	22,8	19,05	554,67	104,79
2019/05/27	CH2206	Male	Three y/o	Treatment (Wk 2)	Treatment	Feed Day	No	22:55	22:00 - 22:59	Night	37,7	79	8,2	8,2	18,85	576,36	70,05
2019/05/27	CH2205	Male	Three y/o	Treatment (Wk 2)	Treatment	Feed Day	No	23:00	23:00 - 23:59	Night	38,0	62	25,4	25,4	19,15	557,33	108,49
2019/05/27	CH2206	Male	Three y/o	Treatment (Wk 2)	Treatment	Feed Day	No	23:00	23:00 - 23:59	Night	37,7	46	2,8	2,8	18,85	532,38	34,04
2019/05/27	CH2205	Male	Three y/o	Treatment (Wk 2)	Treatment	Feed Day	No	23:05	23:00 - 23:59	Night	38,0	54	27,4	27,4	19,15	545,99	111,09
2019/05/27	CH2206	Male	Three y/o	Treatment (Wk 2)	Treatment	Feed Day	No	23:05	23:00 - 23:59	Night	37,7	92	5,4	5,4	18,85	587,78	55,99
2019/05/27	CH2205	Male	Three y/o	Treatment (Wk 2)	Treatment	Feed Day	No	23:10	23:00 - 23:59	Night	38,0	60	24,6	24,6	19,15	554,67	107,39
2019/05/27	CH2206	Male	Three y/o	Treatment (Wk 2)	Treatment	Feed Day	No	23:10	23:00 - 23:59	Night	37,7	69	4,4	4,4	18,85	565,86	49,12
2019/05/27	CH2205	Male	Three y/o	Treatment (Wk 2)	Treatment	Feed Day	No	23:15	23:00 - 23:59	Night	38,0	50	29,8	29,8	19,15	539,52	113,97
2019/05/27	CH2206	Male	Three y/o	Treatment (Wk 2)	Treatment	Feed Day	No	23:15	23:00 - 23:59	Night	37,7	66	7,2	7,2	18,85	562,34	65,66
2019/05/27	CH2205	Male	Three y/o	Treatment (Wk 2)	Treatment	Feed Day	No	23:20	23:00 - 23:59	Night	38,0	61	24,6	24,6	19,15	556,01	107,39
2019/05/27	CH2206	Male	Three y/o	Treatment (Wk 2)	Treatment	Feed Day	No	23:20	23:00 - 23:59	Night	37,7	44	18,8	18,8	18,85	528,51	98,20
2019/05/27	CH2205	Male	Three y/o	Treatment (Wk 2)	Treatment	Feed Day	No	23:25	23:00 - 23:59	Night	38,0	81	24,4	24,4	19,15	578,26	107,11
2019/05/27	CH2206	Male	Three y/o	Treatment (Wk 2)	Treatment	Feed Day	No	23:25	23:00 - 23:59	Night	37,7	104	25,2	25,2	18,85	596,69	108,22
2019/05/27	CH2205	Male	Three y/o	Treatment (Wk 2)	Treatment	Feed Day	No	23:30	23:00 - 23:59	Night	38,0	84	23,2	23,2	19,15	581,01	105,39
2019/05/27	CH2206	Male	Three y/o	Treatment (Wk 2)	Treatment	Feed Day	No	23:30	23:00 - 23:59	Night	37,7	62	22,0	22,0	18,85	557,33	103,57
2019/05/27	CH2205	Male	Three y/o	Treatment (Wk 2)	Treatment	Feed Day	No	23:35	23:00 - 23:59	Night	38,0	53	23,2	23,2	19,15	544,43	105,39
2019/05/27	CH2206	Male	Three y/o	Treatment (Wk 2)	Treatment	Feed Day	No	23:35	23:00 - 23:59	Night	37,7	98	10,0	10,0	18,85	592,41	76,76
2019/05/27	CH2205	Male	Three y/o	Treatment (Wk 2)	Treatment	Feed Day	No	23:40	23:00 - 23:59	Night	38,1	69	22,6	22,6	19,26	565,86	104,49
2019/05/27	CH2206	Male	Three y/o	Treatment (Wk 2)	Treatment	Feed Day	No	23:40	23:00 - 23:59	Night	37,7		18,8	18,8	18,85		98,20
2019/05/27	CH2205	Male	Three y/o	Treatment (Wk 2)	Treatment	Feed Day	No	23:45	23:00 - 23:59	Night	38,1	64	27,2	27,2	19,26	559,88	110,83
2019/05/27	CH2206	Male	Three y/o	Treatment (Wk 2)	Treatment	Feed Day	No	23:45	23:00 - 23:59	Night	37,7	78	30,2	30,2	18,85	575,38	114,42
2019/05/27	CH2205	Male	Three y/o	Treatment (Wk 2)	Treatment	Feed Day	No	23:50	23:00 - 23:59	Night	38,1	84	22,0	22,0	19,26	581,01	103,57
2019/05/27	CH2206	Male	Three y/o	Treatment (Wk 2)	Treatment	Feed Day	No	23:50	23:00 - 23:59	Night	37,5	89	18,4	18,4	18,65	585,33	97,47
2019/05/27	CH2205	Male	Three y/o	Treatment (Wk 2)	Treatment	Feed Day	No	23:55	23:00 - 23:59	Night	38,1	80	27,6	27,6	19,26	577,31	111,33

	2019/05/27	CH2206	Male	Three y/o	Treatment (Wk 2)	Treatment	Feed Day	No	23:55	23:00 - 23:59	Night	37,5	84	14,8	14,8	18,65	581,01	90,05
14	2019/05/28	CH2205	Male	Three y/o	Treatment (Wk 2)	Treatment	Feed Day	No	00:00	00:00 - 00:59	Early Morning	38,1	70	22,8	22,8	19,26	566,99	104,79
	2019/05/28	CH2206	Male	Three y/o	Treatment (Wk 2)	Treatment	Feed Day	No	00:00	00:00 - 00:59	Early Morning	37,5	90	15,0	15,0	18,65	586,16	90,51
	2019/05/28	CH2205	Male	Three y/o	Treatment (Wk 2)	Treatment	Feed Day	No	00:05	00:00 - 00:59	Early Morning	38,1	57	22,6	22,6	19,26	550,47	104,49
	2019/05/28	CH2206	Male	Three y/o	Treatment (Wk 2)	Treatment	Feed Day	No	00:05	00:00 - 00:59	Early Morning	37,5	91	6,0	6,0	18,65	586,98	59,53
	2019/05/28	CH2205	Male	Three y/o	Treatment (Wk 2)	Treatment	Feed Day	No	00:10	00:00 - 00:59	Early Morning	38,0	83	20,2	20,2	19,15	580,11	100,65
	2019/05/28	CH2206	Male	Three y/o	Treatment (Wk 2)	Treatment	Feed Day	No	00:10	00:00 - 00:59	Early Morning	37,5	113	7,4	7,4	18,65	602,58	66,59
	2019/05/28	CH2205	Male	Three y/o	Treatment (Wk 2)	Treatment	Feed Day	No	00:15	00:00 - 00:59	Early Morning	38,0	69	18,6	18,6	19,15	565,86	97,84
	2019/05/28	CH2206	Male	Three y/o	Treatment (Wk 2)	Treatment	Feed Day	No	00:15	00:00 - 00:59	Early Morning	37,6	43	8,2	8,2	18,75	526,50	70,05
	2019/05/28	CH2205	Male	Three y/o	Treatment (Wk 2)	Treatment	Feed Day	No	00:20	00:00 - 00:59	Early Morning	38,0	78	15,4	15,4	19,15	575,38	91,41
	2019/05/28	CH2206	Male	Three y/o	Treatment (Wk 2)	Treatment	Feed Day	No	00:20	00:00 - 00:59	Early Morning	37,6	61	7,4	7,4	18,75	556,01	66,59
	2019/05/28	CH2205	Male	Three y/o	Treatment (Wk 2)	Treatment	Feed Day	No	00:25	00:00 - 00:59	Early Morning	38,0	80	13,6	13,6	19,15	577,31	87,18
	2019/05/28	CH2206	Male	Three y/o	Treatment (Wk 2)	Treatment	Feed Day	No	00:25	00:00 - 00:59	Early Morning	37,6	92	12,6	12,6	18,75	587,78	84,59
	2019/05/28	CH2205	Male	Three y/o	Treatment (Wk 2)	Treatment	Feed Day	No	00:30	00:00 - 00:59	Early Morning	38,0	56	11,8	11,8	19,15	549,01	82,36
	2019/05/28	CH2206	Male	Three y/o	Treatment (Wk 2)	Treatment	Feed Day	No	00:30	00:00 - 00:59	Early Morning	37,7	81	12,8	12,8	18,85	578,26	85,12
	2019/05/28	CH2205	Male	Three y/o	Treatment (Wk 2)	Treatment	Feed Day	No	00:35	00:00 - 00:59	Early Morning	38,0	66	16,4	16,4	19,15	562,34	93,55
	2019/05/28	CH2206	Male	Three y/o	Treatment (Wk 2)	Treatment	Feed Day	No	00:35	00:00 - 00:59	Early Morning	37,7	76	14,4	14,4	18,85	573,39	89,12
	2019/05/28	CH2205	Male	Three y/o	Treatment (Wk 2)	Treatment	Feed Day	No	00:40	00:00 - 00:59	Early Morning	38,0	51	10,4	10,4	19,15	541,20	78,08
	2019/05/28	CH2206	Male	Three y/o	Treatment (Wk 2)	Treatment	Feed Day	No	00:40	00:00 - 00:59	Early Morning	37,7	79	24,0	24,0	18,85	576,36	106,55
	2019/05/28	CH2205	Male	Three y/o	Treatment (Wk 2)	Treatment	Feed Day	No	00:45	00:00 - 00:59	Early Morning	38,0	47	15,4	15,4	19,15	534,23	91,41
	2019/05/28	CH2206	Male	Three y/o	Treatment (Wk 2)	Treatment	Feed Day	No	00:45	00:00 - 00:59	Early Morning	37,5	86	31,8	31,8	18,65	582,77	116,20
	2019/05/28	CH2205	Male	Three y/o	Treatment (Wk 2)	Treatment	Feed Day	No	00:50	00:00 - 00:59	Early Morning	38,0	67	10,2	10,2	19,15	563,53	77,43
	2019/05/28	CH2206	Male	Three y/o	Treatment (Wk 2)	Treatment	Feed Day	No	00:50	00:00 - 00:59	Early Morning	37,5	49	15,8	15,8	18,65	537,80	92,28
	2019/05/28	CH2205	Male	Three y/o	Treatment (Wk 2)	Treatment	Feed Day	No	00:55	00:00 - 00:59	Early Morning	38,1	61	7,0	7,0	19,26	556,01	64,72
	2019/05/28	CH2206	Male	Three y/o	Treatment (Wk 2)	Treatment	Feed Day	No	00:55	00:00 - 00:59	Early Morning	37,7	99	13,0	13,0	18,85	593,14	85,65
	2019/05/28	CH2205	Male	Three y/o	Treatment (Wk 2)	Treatment	Feed Day	No	01:00	01:00 - 01:59	Early Morning	38,1	64	9,0	9,0	19,26	559,88	73,19
	2019/05/28	CH2206	Male	Three y/o	Treatment (Wk 2)	Treatment	Feed Day	No	01:00	01:00 - 01:59	Early Morning	37,7	55	7,8	7,8	18,85	547,52	68,36
	2019/05/28	CH2205	Male	Three y/o	Treatment (Wk 2)	Treatment	Feed Day	No	01:05	01:00 - 01:59	Early Morning	38,0	81	10,6	10,6	19,15	578,26	78,73
	2019/05/28	CH2206	Male	Three y/o	Treatment (Wk 2)	Treatment	Feed Day	No	01:05	01:00 - 01:59	Early Morning	37,7	60	6,8	6,8	18,85	554,67	63,74
	2019/05/28	CH2205	Male	Three y/o	Treatment (Wk 2)	Treatment	Feed Day	No	01:10	01:00 - 01:59	Early Morning	38,0	82	29,4	29,4	19,15	579,19	113,50
	2019/05/28	CH2206	Male	Three y/o	Treatment (Wk 2)	Treatment	Feed Day	No	01:10	01:00 - 01:59	Early Morning	37,7	71	30,2	30,2	18,85	568,10	114,42
	2019/05/28	CH2205	Male	Three y/o	Treatment (Wk 2)	Treatment	Feed Day	No	01:15	01:00 - 01:59	Early Morning	38,0	60	16,6	16,6	19,15	554,67	93,96
	2019/05/28	CH2206	Male	Three y/o	Treatment (Wk 2)	Treatment	Feed Day	No	01:15	01:00 - 01:59	Early Morning	37,5	60	11,6	11,6	18,65	554,67	81,78
	2019/05/28	CH2205	Male	Three y/o	Treatment (Wk 2)	Treatment	Feed Day	No	01:20	01:00 - 01:59	Early Morning	37,9	54	16,0	16,0	19,05	545,99	92,71
	2019/05/28	CH2206	Male	Three y/o	Treatment (Wk 2)	Treatment	Feed Day	No	01:20	01:00 - 01:59	Early Morning	37,5	47	12,8	12,8	18,65	534,23	85,12
	2019/05/28	CH2205	Male	Three y/o	Treatment (Wk 2)	Treatment	Feed Day	No	01:25	01:00 - 01:59	Early Morning	37,9	60	19,2	19,2	19,05	554,67	98,92
	2019/05/28	CH2206	Male	Three y/o	Treatment (Wk 2)	Treatment	Feed Day	No	01:25	01:00 - 01:59	Early Morning	37,4	68	17,8	17,8	18,55	564,71	96,34
	2019/05/28	CH2205	Male	Three y/o	Treatment (Wk 2)	Treatment	Feed Day	No	01:30	01:00 - 01:59	Early Morning	38,0	74	17,8	17,8	19,15	571,33	96,34
	2019/05/28	CH2206	Male	Three y/o	Treatment (Wk 2)	Treatment	Feed Day	No	01:30	01:00 - 01:59	Early Morning	37,4	60	16,2	16,2	18,55	554,67	93,13
	2019/05/28	CH2205	Male	Three y/o	Treatment (Wk 2)	Treatment	Feed Day	No	01:35	01:00 - 01:59	Early Morning	38,0	63	21,4	21,4	19,15	558,62	102,63
	2019/05/28	CH2206	Male	Three y/o	Treatment (Wk 2)	Treatment	Feed Day	No	01:35	01:00 - 01:59	Early Morning	37,5	67	6,6	6,6	18,65	563,53	62,73
	2019/05/28	CH2205	Male	Three y/o	Treatment (Wk 2)	Treatment	Feed Day	No	01:40	01:00 - 01:59	Early Morning	38,0	55	27,0	27,0	19,15	547,52	110,58
	2019/05/28	CH2206	Male	Three y/o	Treatment (Wk 2)	Treatment	Feed Day	No	01:40	01:00 - 01:59	Early Morning	37,5	46	3,4	3,4	18,65	532,38	40,51
	2019/05/28	CH2205	Male	Three y/o	Treatment (Wk 2)	Treatment	Feed Day	No	01:45	01:00 - 01:59	Early Morning	38,0	49	24,6	24,6	19,15	537,80	107,39
	2019/05/28	CH2206	Male	Three y/o	Treatment (Wk 2)	Treatment	Feed Day	No	01:45	01:00 - 01:59	Early Morning	37,5	44	45,6	45,6	18,65	528,51	128,61
	2019/05/28	CH2205	Male	Three y/o	Treatment (Wk 2)	Treatment	Feed Day	No	01:50	01:00 - 01:59	Early Morning	37,9	89	18,0	18,0	19,05	585,33	96,72
	2019/05/28	CH2206	Male	Three y/o	Treatment (Wk 2)	Treatment	Feed Day	No	01:50	01:00 - 01:59	Early Morning	37,4	56	4,4	4,4	18,55	549,01	49,12
	2019/05/28	CH2205	Male	Three y/o	Treatment (Wk 2)	Treatment	Feed Day	No	01:55	01:00 - 01:59	Early Morning	37,8	42	16,6	16,6	18,95	524,42	93,96
	2019/05/28	CH2206	Male	Three y/o	Treatment (Wk 2)	Treatment	Feed Day	No	01:55	01:00 - 01:59	Early Morning	37,3	62	35,2	35,2	18,46	557,33	119,69
	2019/05/28	CH2205	Male	Three y/o	Treatment (Wk 2)	Treatment	Feed Day	No	02:00	02:00 - 02:59	Early Morning	37,7	53	15,4	15,4	18,85	544,43	91,41
	2019/05/28	CH2206	Male	Three y/o	Treatment (Wk 2)	Treatment	Feed Day	No	02:00	02:00 - 02:59	Early Morning	37,4	45	33,8	33,8	18,55	530,47	118,29
	2019/05/28	CH2205	Male	Three y/o	Treatment (Wk 2)	Treatment	Feed Day	No	02:05	02:00 - 02:59	Early Morning	37,7	84	19,0	19,0	18,85	581,01	98,56
	2019/05/28	CH2206	Male	Three y/o	Treatment (Wk 2)	Treatment	Feed Day	No	02:05	02:00 - 02:59	Early Morning	37,4	57	51,6	51,6	18,55	550,47	132,87
	2019/05/28	CH2205	Male	Three y/o	Treatment (Wk 2)	Treatment	Feed Day	No	02:10	02:00 - 02:59	Early Morning	37,7	46	25,8	25,8	18,85	532,38	109,02
	2019/05/28	CH2206	Male	Three y/o	Treatment (Wk 2)	Treatment	Feed Day	No	02:10	02:00 - 02:59	Early Morning	37,4	45	58,0	58,0	18,55	530,47	136,92
	2019/05/28	CH2205	Male	Three y/o	Treatment (Wk 2)	Treatment	Feed Day	No	02:15	02:00 - 02:59	Early Morning	37,5	42	26,6	26,6	18,65	524,42	110,07
	2019/05/28	CH2206	Male	Three y/o	Treatment (Wk 2)	Treatment	Feed Day	No	02:15	02:00 - 02:59	Early Morning	37,4	83	57,8	57,8	18,55	580,11	136,80
	2019/05/28	CH2205	Male	Three y/o	Treatment (Wk 2)	Treatment	Feed Day	No	02:20	02:00 - 02:59	Early Morning	37,5	49	22,0	22,0	18,65	537,80	103,57
	2019/05/28	CH2206	Male	Three y/o	Treatment (Wk 2)	Treatment	Feed Day	No	02:20	02:00 - 02:59	Early Morning	37,5	50	56,4	56,4	18,65	539,52	135,95
	2019/05/28	CH2205	Male	Three y/o	Treatment (Wk 2)	Treatment	Feed Day	No	02:25	02:00 - 02:59	Early Morning	37,4	44	28,0	28,0	18,55	528,51	111,83
	2019/05/28	CH2206	Male	Three y/o	Treatment (Wk 2)	Treatment	Feed Day	No	02:25	02:00 - 02:59	Early Morning	37,5	47	55,2	55,2	18,65	534,23	135,21
	2019/05/28	CH2205	Male	Three y/o	Treatment (Wk 2)	Treatment	Feed Day	No	02:30	02:00 - 02:59	Early Morning	37,4	58	4,2	4,2	18,55	551,90	47,57
	2019/05/28	CH2206	Male	Three y/o	Treatment (Wk 2)	Treatment	Feed Day	No	02:30	02:00 - 02:59	Early Morning	37,5	42	57,6	57,6	18,65	524,42	136,68
	2019/05/28	CH2205	Male	Three y/o	Treatment (Wk 2)	Treatment	Feed Day	No	02:35	02:00 - 02:59	Early Morning	37,4	54	9,4	9,4	18,55	545,99	74,66
	2019/05/28	CH2206	Male	Three y/o	Treatment (Wk 2)	Treatment	Feed Day	No	02:35	02:00 - 02:59	Early Morning	37,5	70	56,0	56,0	18,65	566,99	135,70
	2019/05/28	CH2205	Male	Three y/o	Treatment (Wk 2)	Treatment	Feed Day	No	02:40	02:00 - 02:59	Early Morning	37,5	51	13,6	13,6	18,65	541,20	87,18
	2019/05/28	CH2206	Male	Three y/o	Treatment (Wk 2)	Treatment	Feed Day	No	02:40	02:00 - 02:59	Early Morning	37,5	70	55,4	55,4	18,65	566,99	135,33
	2019/05/28	CH2205	Male	Three y/o	Treatment (Wk 2)	Treatment	Feed Day	No	02:45	02:00 - 02:59	Early Morning	37,5	61	11,4	11,4	18,65	556,01	81,19
	2019/05/28	CH2206	Male	Three y/o	Treatment (Wk 2)	Treatment	Feed Day	No	02:45	02:00 - 02:59	Early Morning	37,6	52	54,2	54,2	18,75	542,83	134,57
	2019/05/28	CH2205	Male	Three y/o	Treatment (Wk 2)	Treatment	Feed Day	No	02:50	02:00 - 02:59	Early Morning	37,6	108	10,8	10,8	18,75	599,39	79,36

2019/05/28	CH2206	Male	Three y/o	Treatment (Wk 2)	Treatment	Feed Day	No	02:50	02:00 - 02:59	Early Morning	37,6	50	58,2	58,2	18,75	539,52	137,04
2019/05/28	CH2205	Male	Three y/o	Treatment (Wk 2)	Treatment	Feed Day	No	02:55	02:00 - 02:59	Early Morning	37,6	86	14,0	14,0	18,75	582,77	88,17
2019/05/28	CH2206	Male	Three y/o	Treatment (Wk 2)	Treatment	Feed Day	No	02:55	02:00 - 02:59	Early Morning	37,6	51	57,2	57,2	18,75	541,20	136,44
2019/05/28	CH2205	Male	Three y/o	Treatment (Wk 2)	Treatment	Feed Day	No	03:00	03:00 - 03:59	Early Morning	37,6	95	4,6	4,6	18,75	590,14	50,61
2019/05/28	CH2206	Male	Three y/o	Treatment (Wk 2)	Treatment	Feed Day	No	03:00	03:00 - 03:59	Early Morning	37,5	47	55,4	55,4	18,65	534,23	135,33
2019/05/28	CH2205	Male	Three y/o	Treatment (Wk 2)	Treatment	Feed Day	No	03:05	03:00 - 03:59	Early Morning	37,6	154	16,2	16,2	18,75	623,58	93,13
2019/05/28	CH2206	Male	Three y/o	Treatment (Wk 2)	Treatment	Feed Day	No	03:05	03:00 - 03:59	Early Morning	37,5	92	54,4	54,4	18,65	587,78	134,70
2019/05/28	CH2205	Male	Three y/o	Treatment (Wk 2)	Treatment	Feed Day	No	03:10	03:00 - 03:59	Early Morning	37,6	54	15,0	15,0	18,75	545,99	90,51
2019/05/28	CH2206	Male	Three y/o	Treatment (Wk 2)	Treatment	Feed Day	No	03:10	03:00 - 03:59	Early Morning	37,5	61	52,4	52,4	18,65	556,01	133,41
2019/05/28	CH2205	Male	Three y/o	Treatment (Wk 2)	Treatment	Feed Day	No	03:15	03:00 - 03:59	Early Morning	37,6	48	16,6	16,6	18,75	536,04	93,96
2019/05/28	CH2206	Male	Three y/o	Treatment (Wk 2)	Treatment	Feed Day	No	03:15	03:00 - 03:59	Early Morning	37,5	41	54,6	54,6	18,65	522,29	134,83
2019/05/28	CH2205	Male	Three y/o	Treatment (Wk 2)	Treatment	Feed Day	No	03:20	03:00 - 03:59	Early Morning	37,6	98	18,8	18,8	18,75	592,41	98,20
2019/05/28	CH2206	Male	Three y/o	Treatment (Wk 2)	Treatment	Feed Day	No	03:20	03:00 - 03:59	Early Morning	37,5	50	53,6	53,6	18,65	539,52	134,19
2019/05/28	CH2205	Male	Three y/o	Treatment (Wk 2)	Treatment	Feed Day	No	03:25	03:00 - 03:59	Early Morning	37,7	52	14,8	14,8	18,85	542,83	90,05
2019/05/28	CH2206	Male	Three y/o	Treatment (Wk 2)	Treatment	Feed Day	No	03:25	03:00 - 03:59	Early Morning	37,5	44	51,6	51,6	18,65	528,51	132,87
2019/05/28	CH2205	Male	Three y/o	Treatment (Wk 2)	Treatment	Feed Day	No	03:30	03:00 - 03:59	Early Morning	37,6	84	12,6	12,6	18,75	581,01	84,59
2019/05/28	CH2206	Male	Three y/o	Treatment (Wk 2)	Treatment	Feed Day	No	03:30	03:00 - 03:59	Early Morning	37,4	46	49,6	49,6	18,55	532,38	131,51
2019/05/28	CH2205	Male	Three y/o	Treatment (Wk 2)	Treatment	Feed Day	No	03:35	03:00 - 03:59	Early Morning	37,6	44	11,8	11,8	18,75	528,51	82,36
2019/05/28	CH2206	Male	Three y/o	Treatment (Wk 2)	Treatment	Feed Day	No	03:35	03:00 - 03:59	Early Morning	37,4	82	49,8	49,8	18,55	579,19	131,65
2019/05/28	CH2205	Male	Three y/o	Treatment (Wk 2)	Treatment	Feed Day	No	03:40	03:00 - 03:59	Early Morning	37,7	71	6,2	6,2	18,85	568,10	60,63
2019/05/28	CH2206	Male	Three y/o	Treatment (Wk 2)	Treatment	Feed Day	No	03:40	03:00 - 03:59	Early Morning	37,4	41	51,8	51,8	18,55	522,29	133,01
2019/05/28	CH2205	Male	Three y/o	Treatment (Wk 2)	Treatment	Feed Day	No	03:45	03:00 - 03:59	Early Morning	37,7	45	6,0	6,0	18,85	530,47	59,53
2019/05/28	CH2206	Male	Three y/o	Treatment (Wk 2)	Treatment	Feed Day	No	03:45	03:00 - 03:59	Early Morning	37,4	52	52,8	52,8	18,55	542,83	133,67
2019/05/28	CH2205	Male	Three y/o	Treatment (Wk 2)	Treatment	Feed Day	No	03:50	03:00 - 03:59	Early Morning	37,6	45	7,8	7,8	18,75	530,47	68,36
2019/05/28	CH2206	Male	Three y/o	Treatment (Wk 2)	Treatment	Feed Day	No	03:50	03:00 - 03:59	Early Morning	37,4	42	54,4	54,4	18,55	524,42	134,70
2019/05/28	CH2205	Male	Three y/o	Treatment (Wk 2)	Treatment	Feed Day	No	03:55	03:00 - 03:59	Early Morning	37,6	79	12,8	12,8	18,75	576,36	85,12
2019/05/28	CH2206	Male	Three y/o	Treatment (Wk 2)	Treatment	Feed Day	No	03:55	03:00 - 03:59	Early Morning	37,4	56	57,2	57,2	18,55	549,01	136,44
2019/05/28	CH2205	Male	Three y/o	Treatment (Wk 2)	Treatment	Feed Day	No	04:00	04:00 - 04:59	Morning	37,6	54	13,2	13,2	18,75	545,99	86,17
2019/05/28	CH2206	Male	Three y/o	Treatment (Wk 2)	Treatment	Feed Day	No	04:00	04:00 - 04:59	Morning	37,3	42	58,2	58,2	18,46	524,42	137,04
2019/05/28	CH2205	Male	Three y/o	Treatment (Wk 2)	Treatment	Feed Day	No	04:05	04:00 - 04:59	Morning	37,6	68	10,4	10,4	18,75	564,71	78,08
2019/05/28	CH2206	Male	Three y/o	Treatment (Wk 2)	Treatment	Feed Day	No	04:05	04:00 - 04:59	Morning	37,3	45	59,8	59,8	18,46	530,47	137,98
2019/05/28	CH2205	Male	Three y/o	Treatment (Wk 2)	Treatment	Feed Day	No	04:10	04:00 - 04:59	Morning	37,6	55	46,0	46,0	18,75	547,52	128,91
2019/05/28	CH2206	Male	Three y/o	Treatment (Wk 2)	Treatment	Feed Day	No	04:10	04:00 - 04:59	Morning	37,2	45	62,2	62,2	18,36	530,47	139,34
2019/05/28	CH2205	Male	Three y/o	Treatment (Wk 2)	Treatment	Feed Day	No	04:15	04:00 - 04:59	Morning	37,6	78	6,2	6,2	18,75	575,38	60,63
2019/05/28	CH2206	Male	Three y/o	Treatment (Wk 2)	Treatment	Feed Day	No	04:15	04:00 - 04:59	Morning	37,1	49	63,0	63,0	18,26	537,80	139,78
2019/05/28	CH2205	Male	Three y/o	Treatment (Wk 2)	Treatment	Feed Day	No	04:20	04:00 - 04:59	Morning	37,6	48	13,4	13,4	18,75	536,04	86,68
2019/05/28	CH2206	Male	Three y/o	Treatment (Wk 2)	Treatment	Feed Day	No	04:20	04:00 - 04:59	Morning	37,0	49	55,8	55,8	18,16	537,80	135,58
2019/05/28	CH2205	Male	Three y/o	Treatment (Wk 2)	Treatment	Feed Day	No	04:25	04:00 - 04:59	Morning	37,6	49	15,0	15,0	18,75	537,80	90,51
2019/05/28	CH2206	Male	Three y/o	Treatment (Wk 2)	Treatment	Feed Day	No	04:25	04:00 - 04:59	Morning	36,9	50	59,0	59,0	18,06	539,52	137,51
2019/05/28	CH2205	Male	Three y/o	Treatment (Wk 2)	Treatment	Feed Day	No	04:30	04:00 - 04:59	Morning	37,6	86	12,6	12,6	18,75	582,77	84,59
2019/05/28	CH2206	Male	Three y/o	Treatment (Wk 2)	Treatment	Feed Day	No	04:30	04:00 - 04:59	Morning	36,8	46	61,4	61,4	17,96	532,38	138,89
2019/05/28	CH2205	Male	Three y/o	Treatment (Wk 2)	Treatment	Feed Day	No	04:35	04:00 - 04:59	Morning	37,6		15,0	15,0	18,75		90,51
2019/05/28	CH2206	Male	Three y/o	Treatment (Wk 2)	Treatment	Feed Day	No	04:35	04:00 - 04:59	Morning	36,8	78	41,6	41,6	17,96	575,38	125,44
2019/05/28	CH2205	Male	Three y/o	Treatment (Wk 2)	Treatment	Feed Day	No	04:40	04:00 - 04:59	Morning	37,6	83	10,4	10,4	18,75	580,11	78,08
2019/05/28	CH2206	Male	Three y/o	Treatment (Wk 2)	Treatment	Feed Day	No	04:40	04:00 - 04:59	Morning	37,0	83	10,6	10,6	18,16	580,11	78,73
2019/05/28	CH2205	Male	Three y/o	Treatment (Wk 2)	Treatment	Feed Day	No	04:45	04:00 - 04:59	Morning	37,5	43	6,0	6,0	18,65	526,50	59,53
2019/05/28	CH2206	Male	Three y/o	Treatment (Wk 2)	Treatment	Feed Day	No	04:45	04:00 - 04:59	Morning	36,9	45	10,0	10,0	18,06	530,47	76,76
2019/05/28	CH2205	Male	Three y/o	Treatment (Wk 2)	Treatment	Feed Day	No	04:50	04:00 - 04:59	Morning	37,5	43	2,8	2,8	18,65	526,50	34,04
2019/05/28	CH2206	Male	Three y/o	Treatment (Wk 2)	Treatment	Feed Day	No	04:50	04:00 - 04:59	Morning	37,0	42	18,6	18,6	18,16	524,42	97,84
2019/05/28	CH2205	Male	Three y/o	Treatment (Wk 2)	Treatment	Feed Day	No	04:55	04:00 - 04:59	Morning	37,5	41	6,6	6,6	18,65	522,29	62,73
2019/05/28	CH2206	Male	Three y/o	Treatment (Wk 2)	Treatment	Feed Day	No	04:55	04:00 - 04:59	Morning	37,1		10,6	10,6	18,26		78,73
2019/05/28	CH2205	Male	Three y/o	Treatment (Wk 2)	Treatment	Feed Day	No	05:00	05:00 - 05:59	Morning	37,5	80	6,0	6,0	18,65	577,31	59,53
2019/05/28	CH2206	Male	Three y/o	Treatment (Wk 2)	Treatment	Feed Day	No	05:00	05:00 - 05:59	Morning	37,1	45	3,0	3,0	18,26	530,47	36,34
2019/05/28	CH2205	Male	Three y/o	Treatment (Wk 2)	Treatment	Feed Day	No	05:05	05:00 - 05:59	Morning	37,5		6,6	6,6	18,65		62,73
2019/05/28	CH2206	Male	Three y/o	Treatment (Wk 2)	Treatment	Feed Day	No	05:05	05:00 - 05:59	Morning	37,1	62	16,8	16,8	18,26	557,33	94,37
2019/05/28	CH2205	Male	Three y/o	Treatment (Wk 2)	Treatment	Feed Day	No	05:10	05:00 - 05:59	Morning	37,5	50	40,6	40,6	18,65	539,52	124,60
2019/05/28	CH2206	Male	Three y/o	Treatment (Wk 2)	Treatment	Feed Day	No	05:10	05:00 - 05:59	Morning	37,1	39	42,2	42,2	18,26	517,82	125,93
2019/05/28	CH2205	Male	Three y/o	Treatment (Wk 2)	Treatment	Feed Day	No	05:15	05:00 - 05:59	Morning	37,5	118	75,0	75,0	18,65	605,61	145,83
2019/05/28	CH2206	Male	Three y/o	Treatment (Wk 2)	Treatment	Feed Day	No	05:15	05:00 - 05:59	Morning	37,2	134	72,8	72,8	18,36	614,33	144,80
2019/05/28	CH2205	Male	Three y/o	Treatment (Wk 2)	Treatment	Feed Day	No	05:20	05:00 - 05:59	Morning	37,4	98	62,2	62,2	18,55	592,41	139,34
2019/05/28	CH2206	Male	Three y/o	Treatment (Wk 2)	Treatment	Feed Day	No	05:20	05:00 - 05:59	Morning	37,2		99,2	99,2	18,36		155,56
2019/05/28	CH2205	Male	Three y/o	Treatment (Wk 2)	Treatment	Feed Day	No	05:25	05:00 - 05:59	Morning	37,3	86	72,8	72,8	18,46	582,77	144,80
2019/05/28	CH2206	Male	Three y/o	Treatment (Wk 2)	Treatment	Feed Day	No	05:25	05:00 - 05:59	Morning	37,2	126	125,0	125,0	18,36	610,14	163,63
2019/05/28	CH2205	Male	Three y/o	Treatment (Wk 2)	Treatment	Feed Day	No	05:30	05:00 - 05:59	Morning	37,3	94	35,4	35,4	18,46	589,37	119,88
2019/05/28	CH2206	Male	Three y/o	Treatment (Wk 2)	Treatment	Feed Day	No	05:30	05:00 - 05:59	Morning	37,2	126	270,0	270,0	18,36	610,14	190,70
2019/05/28	CH2205	Male	Three y/o	Treatment (Wk 2)	Treatment	Feed Day	No	05:35	05:00 - 05:59	Morning	37,3	82	136,6	136,6	18,46	579,19	166,74
2019/05/28	CH2206	Male	Three y/o	Treatment (Wk 2)	Treatment	Feed Day	No	05:35	05:00 - 05:59	Morning	37,2	63	105,2	105,2	18,36	558,62	157,61
2019/05/28	CH2205	Male	Three y/o	Treatment (Wk 2)	Treatment	Feed Day	No	05:40	05:00 - 05:59	Morning	37,3	108	136,6	136,6	18,46	599,39	166,74
2019/05/28	CH2206	Male	Three y/o	Treatment (Wk 2)	Treatment	Feed Day	No	05:40	05:00 - 05:59	Morning	37,4		115,0	115,0	18,55		160,72
2019/05/28	CH2205	Male	Three y/o	Treatment (Wk 2)	Treatment	Feed Day	No	05:45	05:00 - 05:59	Morning	37,3	51	48,4	48,4	18,46	541,20	130,66

2019/05/28	CH2206	Male	Three y/o	Treatment (Wk 2)	Treatment	Feed Day	No	05:45	05:00 - 05:59	Morning	37,4		154,2	154,2	18,55		170,99
2019/05/28	CH2205	Male	Three y/o	Treatment (Wk 2)	Treatment	Feed Day	No	05:50	05:00 - 05:59	Morning	37,5	102	34,8	34,8	18,65	595,30	119,29
2019/05/28	CH2206	Male	Three y/o	Treatment (Wk 2)	Treatment	Feed Day	No	05:50	05:00 - 05:59	Morning	37,5	125	163,2	163,2	18,65	609,60	172,97
2019/05/28	CH2205	Male	Three y/o	Treatment (Wk 2)	Treatment	Feed Day	No	05:55	05:00 - 05:59	Morning	37,4	76	9,0	9,0	18,55	573,39	73,19
2019/05/28	CH2206	Male	Three y/o	Treatment (Wk 2)	Treatment	Feed Day	No	05:55	05:00 - 05:59	Morning	37,5	152	54,4	54,4	18,65	622,73	134,70
2019/05/28	CH2205	Male	Three y/o	Treatment (Wk 2)	Treatment	Feed Day	No	06:00	06:00 - 06:59	Morning	37,2	67	8,6	8,6	18,36	563,53	71,66
2019/05/28	CH2206	Male	Three y/o	Treatment (Wk 2)	Treatment	Feed Day	No	06:00	06:00 - 06:59	Morning	37,4	188	67,8	67,8	18,55	636,34	142,33
2019/05/28	CH2205	Male	Three y/o	Treatment (Wk 2)	Treatment	Feed Day	No	06:05	06:00 - 06:59	Morning	37,2	47	4,6	4,6	18,36	534,23	50,61
2019/05/28	CH2206	Male	Three y/o	Treatment (Wk 2)	Treatment	Feed Day	No	06:05	06:00 - 06:59	Morning	37,2	82	66,0	66,0	18,36	579,19	141,40
2019/05/28	CH2205	Male	Three y/o	Treatment (Wk 2)	Treatment	Feed Day	No	06:10	06:00 - 06:59	Morning	37,1	73	6,4	6,4	18,26	570,27	61,70
2019/05/28	CH2206	Male	Three y/o	Treatment (Wk 2)	Treatment	Feed Day	No	06:10	06:00 - 06:59	Morning	37,0	114	137,6	137,6	18,16	603,20	166,99
2019/05/28	CH2205	Male	Three y/o	Treatment (Wk 2)	Treatment	Feed Day	No	06:15	06:00 - 06:59	Morning	37,1	61	8,2	8,2	18,26	556,01	70,05
2019/05/28	CH2206	Male	Three y/o	Treatment (Wk 2)	Treatment	Feed Day	No	06:15	06:00 - 06:59	Morning	37,3	177	228,2	228,2	18,46	632,55	184,76
2019/05/28	CH2205	Male	Three y/o	Treatment (Wk 2)	Treatment	Feed Day	No	06:20	06:00 - 06:59	Morning	37,2	82	18,4	18,4	18,36	579,19	97,47
2019/05/28	CH2206	Male	Three y/o	Treatment (Wk 2)	Treatment	Feed Day	No	06:20	06:00 - 06:59	Morning	37,4	141	146,4	146,4	18,55	617,75	169,17
2019/05/28	CH2205	Male	Three y/o	Treatment (Wk 2)	Treatment	Feed Day	No	06:25	06:00 - 06:59	Morning	37,1	69	23,8	23,8	18,26	565,86	106,26
2019/05/28	CH2206	Male	Three y/o	Treatment (Wk 2)	Treatment	Feed Day	No	06:25	06:00 - 06:59	Morning	37,4	121	216,4	216,4	18,55	607,35	182,89
2019/05/28	CH2205	Male	Three y/o	Treatment (Wk 2)	Treatment	Feed Day	No	06:30	06:00 - 06:59	Morning	37,1	72	10,0	10,0	18,26	569,20	76,76
2019/05/28	CH2206	Male	Three y/o	Treatment (Wk 2)	Treatment	Feed Day	No	06:30	06:00 - 06:59	Morning	37,4	85	62,4	62,4	18,55	581,90	139,45
2019/05/28	CH2205	Male	Three y/o	Treatment (Wk 2)	Treatment	Feed Day	No	06:35	06:00 - 06:59	Morning	37,1	70	12,6	12,6	18,26	566,99	84,59
2019/05/28	CH2206	Male	Three y/o	Treatment (Wk 2)	Treatment	Feed Day	No	06:35	06:00 - 06:59	Morning	37,1	137	38,6	38,6	18,26	615,83	122,86
2019/05/28	CH2205	Male	Three y/o	Treatment (Wk 2)	Treatment	Feed Day	No	06:40	06:00 - 06:59	Morning	37,1	58	16,8	16,8	18,26	551,90	94,37
2019/05/28	CH2206	Male	Three y/o	Treatment (Wk 2)	Treatment	Feed Day	No	06:40	06:00 - 06:59	Morning	37,0	126	150,2	150,2	18,16	610,14	170,06
2019/05/28	CH2205	Male	Three y/o	Treatment (Wk 2)	Treatment	Feed Day	No	06:45	06:00 - 06:59	Morning	37,0		76,0	76,0	18,16		146,29
2019/05/28	CH2206	Male	Three y/o	Treatment (Wk 2)	Treatment	Feed Day	No	06:45	06:00 - 06:59	Morning	37,2	131	423,6	423,6	18,36	612,80	206,65
2019/05/28	CH2205	Male	Three y/o	Treatment (Wk 2)	Treatment	Feed Day	No	06:50	06:00 - 06:59	Morning	37,2	112	58,6	58,6	18,36	601,96	137,28
2019/05/28	CH2206	Male	Three y/o	Treatment (Wk 2)	Treatment	Feed Day	No	06:50	06:00 - 06:59	Morning	37,3	118	154,8	154,8	18,46	605,61	171,12
2019/05/28	CH2205	Male	Three y/o	Treatment (Wk 2)	Treatment	Feed Day	No	06:55	06:00 - 06:59	Morning	37,2	145	89,6	89,6	18,36	619,62	152,02
2019/05/28	CH2206	Male	Three y/o	Treatment (Wk 2)	Treatment	Feed Day	No	06:55	06:00 - 06:59	Morning	37,5	126	48,0	48,0	18,65	610,14	130,38
2019/05/28	CH2205	Male	Three y/o	Treatment (Wk 2)	Treatment	Feed Day	No	07:00	07:00 - 07:59	Morning	37,3		249,2	249,2	18,46		187,87
2019/05/28	CH2206	Male	Three y/o	Treatment (Wk 2)	Treatment	Feed Day	No	07:00	07:00 - 07:59	Morning	37,5	148	256,2	256,2	18,65	620,97	188,84
2019/05/28	CH2205	Male	Three y/o	Treatment (Wk 2)	Treatment	Feed Day	No	07:05	07:00 - 07:59	Morning	37,3		207,8	207,8	18,46		181,47
2019/05/28	CH2206	Male	Three y/o	Treatment (Wk 2)	Treatment	Feed Day	No	07:05	07:00 - 07:59	Morning	37,7	128	113,6	113,6	18,85	611,22	160,29
2019/05/28	CH2205	Male	Three y/o	Treatment (Wk 2)	Treatment	Feed Day	No	07:10	07:00 - 07:59	Morning	37,4	148	184,4	184,4	18,55	620,97	177,26
2019/05/28	CH2206	Male	Three y/o	Treatment (Wk 2)	Treatment	Feed Day	No	07:10	07:00 - 07:59	Morning	37,7	119	194,6	194,6	18,85	606,20	179,16
2019/05/28	CH2205	Male	Three y/o	Treatment (Wk 2)	Treatment	Feed Day	No	07:15	07:00 - 07:59	Morning	37,5	98	314,2	314,2	18,65	592,41	196,06
2019/05/28	CH2206	Male	Three y/o	Treatment (Wk 2)	Treatment	Feed Day	No	07:15	07:00 - 07:59	Morning	37,8	107	256,0	256,0	18,95	598,73	188,82
2019/05/28	CH2205	Male	Three y/o	Treatment (Wk 2)	Treatment	Feed Day	No	07:20	07:00 - 07:59	Morning	37,6	49	50,6	50,6	18,75	537,80	132,20
2019/05/28	CH2206	Male	Three y/o	Treatment (Wk 2)	Treatment	Feed Day	No	07:20	07:00 - 07:59	Morning	37,8	119	88,0	88,0	18,95	606,20	151,39
2019/05/28	CH2205	Male	Three y/o	Treatment (Wk 2)	Treatment	Feed Day	No	07:25	07:00 - 07:59	Morning	37,6		74,6	74,6	18,75		145,65
2019/05/28	CH2206	Male	Three y/o	Treatment (Wk 2)	Treatment	Feed Day	No	07:25	07:00 - 07:59	Morning	37,9	105	105,0	105,0	19,05	597,38	157,55
2019/05/28	CH2205	Male	Three y/o	Treatment (Wk 2)	Treatment	Feed Day	No	07:30	07:00 - 07:59	Morning	37,5	90	45,6	45,6	18,65	586,16	128,61
2019/05/28	CH2206	Male	Three y/o	Treatment (Wk 2)	Treatment	Feed Day	No	07:30	07:00 - 07:59	Morning	37,9	53	85,8	85,8	19,05	544,43	150,51
2019/05/28	CH2205	Male	Three y/o	Treatment (Wk 2)	Treatment	Feed Day	No	07:35	07:00 - 07:59	Morning	37,5	102	185,4	185,4	18,65	595,30	177,45
2019/05/28	CH2206	Male	Three y/o	Treatment (Wk 2)	Treatment	Feed Day	No	07:35	07:00 - 07:59	Morning	37,9	111	71,2	71,2	19,05	601,33	144,03
2019/05/28	CH2205	Male	Three y/o	Treatment (Wk 2)	Treatment	Feed Day	No	07:40	07:00 - 07:59	Morning	37,5		57,2	57,2	18,65		136,44
2019/05/28	CH2206	Male	Three y/o	Treatment (Wk 2)	Treatment	Feed Day	No	07:40	07:00 - 07:59	Morning	37,9		368,6	368,6	19,05		201,71
2019/05/28	CH2205	Male	Three y/o	Treatment (Wk 2)	Treatment	Feed Day	No	07:45	07:00 - 07:59	Morning	37,6	111	256,6	256,6	18,75	601,33	188,90
2019/05/28	CH2206	Male	Three y/o	Treatment (Wk 2)	Treatment	Feed Day	No	07:45	07:00 - 07:59	Morning	37,9	103	186,2	186,2	19,05	596,00	177,60
2019/05/28	CH2205	Male	Three y/o	Treatment (Wk 2)	Treatment	Feed Day	No	07:50	07:00 - 07:59	Morning	37,7	87	15,0	15,0	18,85	583,64	90,51
2019/05/28	CH2206	Male	Three y/o	Treatment (Wk 2)	Treatment	Feed Day	No	07:50	07:00 - 07:59	Morning	37,9	94	71,0	71,0	19,05	589,37	143,93
2019/05/28	CH2205	Male	Three y/o	Treatment (Wk 2)	Treatment	Feed Day	No	07:55	07:00 - 07:59	Morning	37,7	56	82,6	82,6	18,85	549,01	149,19
2019/05/28	CH2206	Male	Three y/o	Treatment (Wk 2)	Treatment	Feed Day	No	07:55	07:00 - 07:59	Morning	37,9	128	165,2	165,2	19,05	611,22	173,40
2019/05/28	CH2205	Male	Three y/o	Treatment (Wk 2)	Treatment	Feed Day	No	08:00	08:00 - 08:59	Late Morning	37,7		82,2	82,2	18,85		149,02
2019/05/28	CH2206	Male	Three y/o	Treatment (Wk 2)	Treatment	Feed Day	No	08:00	08:00 - 08:59	Late Morning	37,9	98	90,2	90,2	19,05	592,41	152,25
2019/05/28	CH2205	Male	Three y/o	Treatment (Wk 2)	Treatment	Feed Day	No	08:05	08:00 - 08:59	Late Morning	37,7	51	142,6	142,6	18,85	541,20	168,24
2019/05/28	CH2206	Male	Three y/o	Treatment (Wk 2)	Treatment	Feed Day	No	08:05	08:00 - 08:59	Late Morning	37,9	124	192,4	192,4	19,05	609,04	178,76
2019/05/28	CH2205	Male	Three y/o	Treatment (Wk 2)	Treatment	Feed Day	No	08:10	08:00 - 08:59	Late Morning	37,7	121	456,8	456,8	18,85	607,35	209,33
2019/05/28	CH2206	Male	Three y/o	Treatment (Wk 2)	Treatment	Feed Day	No	08:10	08:00 - 08:59	Late Morning	37,9	165	114,4	114,4	19,05	628,06	160,54
2019/05/28	CH2205	Male	Three y/o	Treatment (Wk 2)	Treatment	Feed Day	No	08:15	08:00 - 08:59	Late Morning	37,6	106	140,2	140,2	18,75	598,06	167,65
2019/05/28	CH2206	Male	Three y/o	Treatment (Wk 2)	Treatment	Feed Day	No	08:15	08:00 - 08:59	Late Morning	37,9	104	100,8	100,8	19,05	596,69	156,12
2019/05/28	CH2205	Male	Three y/o	Treatment (Wk 2)	Treatment	Feed Day	No	08:20	08:00 - 08:59	Late Morning	37,7	71	143,2	143,2	18,85	568,10	168,39
2019/05/28	CH2206	Male	Three y/o	Treatment (Wk 2)	Treatment	Feed Day	No	08:20	08:00 - 08:59	Late Morning	37,9	112	102,6	102,6	19,05	601,96	156,74
2019/05/28	CH2205	Male	Three y/o	Treatment (Wk 2)	Treatment	Feed Day	No	08:25	08:00 - 08:59	Late Morning	37,7	143	516,2	516,2	18,85	618,69	213,68
2019/05/28	CH2206	Male	Three y/o	Treatment (Wk 2)	Treatment	Feed Day	No	08:25	08:00 - 08:59	Late Morning	37,8	110	328,6	328,6	18,95	600,69	197,64
2019/05/28	CH2205	Male	Three y/o	Treatment (Wk 2)	Treatment	Feed Day	No	08:30	08:00 - 08:59	Late Morning	38,5	103	428,6	428,6	19,66	596,00	207,07
2019/05/28	CH2206	Male	Three y/o	Treatment (Wk 2)	Treatment	Feed Day	No	08:30	08:00 - 08:59	Late Morning	38,4	129	59,0	59,0	19,56	611,75	137,51
2019/05/28	CH2205	Male	Three y/o	Treatment (Wk 2)	Treatment	Feed Day	No	08:35	08:00 - 08:59	Late Morning	39,0		223,6	223,6	20,18		184,05
2019/05/28	CH2206	Male	Three y/o	Treatment (Wk 2)	Treatment	Feed Day	No	08:35	08:00 - 08:59	Late Morning	38,3	71	110,0	110,0	19,46	568,10	159,17
2019/05/28	CH2205	Male	Three y/o	Treatment (Wk 2)	Treatment	Feed Day	No	08:40	08:00 - 08:59	Late Morning	39,0		84,6	84,6	20,18		150,02

2019/05/28	CH2206	Male	Three y/o	Treatment (Wk 2)	Treatment	Feed Day	No	08:40	08:00 - 08:59	Late Morning	38,4		56,4	56,4	19,56		135,95
2019/05/28	CH2205	Male	Three y/o	Treatment (Wk 2)	Treatment	Feed Day	No	08:45	08:00 - 08:59	Late Morning	39,0	134	90,2	90,2	20,18	614,33	152,25
2019/05/28	CH2206	Male	Three y/o	Treatment (Wk 2)	Treatment	Feed Day	No	08:45	08:00 - 08:59	Late Morning	38,4	57	151,0	151,0	19,56	550,47	170,25
2019/05/28	CH2205	Male	Three y/o	Treatment (Wk 2)	Treatment	Feed Day	No	08:50	08:00 - 08:59	Late Morning	39,0	141	413,0	413,0	20,18	617,75	205,75
2019/05/28	CH2206	Male	Three y/o	Treatment (Wk 2)	Treatment	Feed Day	No	08:50	08:00 - 08:59	Late Morning	38,3	125	61,6	61,6	19,46	609,60	139,00
2019/05/28	CH2205	Male	Three y/o	Treatment (Wk 2)	Treatment	Feed Day	No	08:55	08:00 - 08:59	Late Morning	38,9	123	139,8	139,8	20,07	608,49	167,55
2019/05/28	CH2206	Male	Three y/o	Treatment (Wk 2)	Treatment	Feed Day	No	08:55	08:00 - 08:59	Late Morning	38,2		35,4	35,4	19,36		119,88
2019/05/28	CH2205	Male	Three y/o	Treatment (Wk 2)	Treatment	Feed Day	No	09:00	09:00 - 09:59	Late Morning	38,8	104	427,2	427,2	19,97	596,69	206,95
2019/05/28	CH2206	Male	Three y/o	Treatment (Wk 2)	Treatment	Feed Day	No	09:00	09:00 - 09:59	Late Morning	38,1	46	69,0	69,0	19,26	532,38	142,94
2019/05/28	CH2205	Male	Three y/o	Treatment (Wk 2)	Treatment	Feed Day	No	09:05	09:00 - 09:59	Late Morning	38,7		288,2	288,2	19,87		193,00
2019/05/28	CH2206	Male	Three y/o	Treatment (Wk 2)	Treatment	Feed Day	No	09:05	09:00 - 09:59	Late Morning	38,0		156,0	156,0	19,15		171,39
2019/05/28	CH2205	Male	Three y/o	Treatment (Wk 2)	Treatment	Feed Day	No	09:10	09:00 - 09:59	Late Morning	38,5	129	119,8	119,8	19,66	611,75	162,15
2019/05/28	CH2206	Male	Three y/o	Treatment (Wk 2)	Treatment	Feed Day	No	09:10	09:00 - 09:59	Late Morning	38,0	104	48,0	48,0	19,15	596,69	130,38
2019/05/28	CH2205	Male	Three y/o	Treatment (Wk 2)	Treatment	Feed Day	No	09:15	09:00 - 09:59	Late Morning	38,5		201,4	201,4	19,66		180,36
2019/05/28	CH2206	Male	Three y/o	Treatment (Wk 2)	Treatment	Feed Day	No	09:15	09:00 - 09:59	Late Morning	37,9	124	44,6	44,6	19,05	609,04	127,84
2019/05/28	CH2205	Male	Three y/o	Treatment (Wk 2)	Treatment	Feed Day	No	09:20	09:00 - 09:59	Late Morning	38,5	134	471,6	471,6	19,66	614,33	210,47
2019/05/28	CH2206	Male	Three y/o	Treatment (Wk 2)	Treatment	Feed Day	No	09:20	09:00 - 09:59	Late Morning	37,7	129	231,8	231,8	18,85	611,75	185,31
2019/05/28	CH2205	Male	Three y/o	Treatment (Wk 2)	Treatment	Feed Day	No	09:25	09:00 - 09:59	Late Morning	38,9	115	17,4	17,4	20,07	603,82	95,56
2019/05/28	CH2206	Male	Three y/o	Treatment (Wk 2)	Treatment	Feed Day	No	09:25	09:00 - 09:59	Late Morning	38,4	104	145,2	145,2	19,56	596,69	168,88
2019/05/28	CH2205	Male	Three y/o	Treatment (Wk 2)	Treatment	Feed Day	No	09:30	09:00 - 09:59	Late Morning	38,8	79	4,4	4,4	19,97	576,36	49,12
2019/05/28	CH2206	Male	Three y/o	Treatment (Wk 2)	Treatment	Feed Day	No	09:30	09:00 - 09:59	Late Morning	38,4		379,4	379,4	19,56		202,74
2019/05/28	CH2205	Male	Three y/o	Treatment (Wk 2)	Treatment	Feed Day	No	09:35	09:00 - 09:59	Late Morning	38,7	86	18,2	18,2	19,87	582,77	97,10
2019/05/28	CH2206	Male	Three y/o	Treatment (Wk 2)	Treatment	Feed Day	No	09:35	09:00 - 09:59	Late Morning	38,3	93	23,6	23,6	19,46	588,58	105,97
2019/05/28	CH2205	Male	Three y/o	Treatment (Wk 2)	Treatment	Feed Day	No	09:40	09:00 - 09:59	Late Morning	38,5	98	27,8	27,8	19,66	592,41	111,58
2019/05/28	CH2206	Male	Three y/o	Treatment (Wk 2)	Treatment	Feed Day	No	09:40	09:00 - 09:59	Late Morning	38,3	82	29,2	29,2	19,46	579,19	113,27
2019/05/28	CH2205	Male	Three y/o	Treatment (Wk 2)	Treatment	Feed Day	No	09:45	09:00 - 09:59	Late Morning	38,5		23,2	23,2	19,66		105,39
2019/05/28	CH2206	Male	Three y/o	Treatment (Wk 2)	Treatment	Feed Day	No	09:45	09:00 - 09:59	Late Morning	38,2	115	37,2	37,2	19,36	603,82	121,59
2019/05/28	CH2205	Male	Three y/o	Treatment (Wk 2)	Treatment	Feed Day	No	09:50	09:00 - 09:59	Late Morning	38,4	67	31,2	31,2	19,56	563,53	115,54
2019/05/28	CH2206	Male	Three y/o	Treatment (Wk 2)	Treatment	Feed Day	No	09:50	09:00 - 09:59	Late Morning	38,1	118	50,0	50,0	19,26	605,61	131,79
2019/05/28	CH2205	Male	Three y/o	Treatment (Wk 2)	Treatment	Feed Day	No	09:55	09:00 - 09:59	Late Morning	38,1		27,2	27,2	19,26		110,83
2019/05/28	CH2206	Male	Three y/o	Treatment (Wk 2)	Treatment	Feed Day	No	09:55	09:00 - 09:59	Late Morning	37,9	48	50,4	50,4	19,05	536,04	132,06
2019/05/28	CH2205	Male	Three y/o	Treatment (Wk 2)	Treatment	Feed Day	No	10:00	10:00 - 10:59	Late Morning	38,0	91	64,2	64,2	19,15	586,98	140,44
2019/05/28	CH2206	Male	Three y/o	Treatment (Wk 2)	Treatment	Feed Day	No	10:00	10:00 - 10:59	Late Morning	37,7	83	58,6	58,6	18,85	580,11	137,28
2019/05/28	CH2205	Male	Three y/o	Treatment (Wk 2)	Treatment	Feed Day	No	10:05	10:00 - 10:59	Late Morning	37,9	107	88,2	88,2	19,05	598,73	151,47
2019/05/28	CH2206	Male	Three y/o	Treatment (Wk 2)	Treatment	Feed Day	No	10:05	10:00 - 10:59	Late Morning	37,7	103	90,4	90,4	18,85	596,00	152,33
2019/05/28	CH2205	Male	Three y/o	Treatment (Wk 2)	Treatment	Feed Day	No	10:10	10:00 - 10:59	Late Morning	37,9	61	61,8	61,8	19,05	556,01	139,12
2019/05/28	CH2206	Male	Three y/o	Treatment (Wk 2)	Treatment	Feed Day	No	10:10	10:00 - 10:59	Late Morning	37,8	106	128,2	128,2	18,95	598,06	164,52
2019/05/28	CH2205	Male	Three y/o	Treatment (Wk 2)	Treatment	Feed Day	No	10:15	10:00 - 10:59	Late Morning	37,9	147	135,6	135,6	19,05	620,52	166,48
2019/05/28	CH2206	Male	Three y/o	Treatment (Wk 2)	Treatment	Feed Day	No	10:15	10:00 - 10:59	Late Morning	37,8	104	108,0	108,0	18,95	596,69	158,53
2019/05/28	CH2205	Male	Three y/o	Treatment (Wk 2)	Treatment	Feed Day	No	10:20	10:00 - 10:59	Late Morning	37,9		67,8	67,8	19,05		142,33
2019/05/28	CH2206	Male	Three y/o	Treatment (Wk 2)	Treatment	Feed Day	No	10:20	10:00 - 10:59	Late Morning	37,8	191	292,8	292,8	18,95	637,33	193,56
2019/05/28	CH2205	Male	Three y/o	Treatment (Wk 2)	Treatment	Feed Day	No	10:25	10:00 - 10:59	Late Morning	37,9	147	87,6	87,6	19,05	620,52	151,23
2019/05/28	CH2206	Male	Three y/o	Treatment (Wk 2)	Treatment	Feed Day	No	10:25	10:00 - 10:59	Late Morning	37,8	123	351,6	351,6	18,95	608,49	200,04
2019/05/28	CH2205	Male	Three y/o	Treatment (Wk 2)	Treatment	Feed Day	Yes	10:30	10:00 - 10:59	Late Morning	37,7	77	190,8	190,8	18,85	574,39	178,46
2019/05/28	CH2206	Male	Three y/o	Treatment (Wk 2)	Treatment	Feed Day	Yes	10:30	10:00 - 10:59	Late Morning	37,9	140	51,0	51,0	19,05	617,28	132,47
2019/05/28	CH2205	Male	Three y/o	Treatment (Wk 2)	Treatment	Feed Day	Yes	10:35	10:00 - 10:59	Late Morning	37,7	89	19,2	19,2	18,85	585,33	98,92
2019/05/28	CH2206	Male	Three y/o	Treatment (Wk 2)	Treatment	Feed Day	Yes	10:35	10:00 - 10:59	Late Morning	37,9	83	46,4	46,4	19,05	580,11	129,21
2019/05/28	CH2205	Male	Three y/o	Treatment (Wk 2)	Treatment	Feed Day	Yes	10:40	10:00 - 10:59	Late Morning	37,7		47,6	47,6	18,85		130,09
2019/05/28	CH2206	Male	Three y/o	Treatment (Wk 2)	Treatment	Feed Day	Yes	10:40	10:00 - 10:59	Late Morning	37,8	92	139,6	139,6	18,95	587,78	167,50
2019/05/28	CH2205	Male	Three y/o	Treatment (Wk 2)	Treatment	Feed Day	Yes	10:45	10:00 - 10:59	Late Morning	37,7		25,2	25,2	18,85		108,22
2019/05/28	CH2206	Male	Three y/o	Treatment (Wk 2)	Treatment	Feed Day	Yes	10:45	10:00 - 10:59	Late Morning	37,9	111	157,4	157,4	19,05	601,33	171,71
2019/05/28	CH2205	Male	Three y/o	Treatment (Wk 2)	Treatment	Feed Day	Yes	10:50	10:00 - 10:59	Late Morning	37,7	112	51,4	51,4	18,85	601,96	132,74
2019/05/28	CH2206	Male	Three y/o	Treatment (Wk 2)	Treatment	Feed Day	Yes	10:50	10:00 - 10:59	Late Morning	37,9	107	49,0	49,0	19,05	598,73	131,09
2019/05/28	CH2205	Male	Three y/o	Treatment (Wk 2)	Treatment	Feed Day	Yes	10:55	10:00 - 10:59	Late Morning	37,7	94	84,6	84,6	18,85	589,37	150,02
2019/05/28	CH2206	Male	Three y/o	Treatment (Wk 2)	Treatment	Feed Day	Yes	10:55	10:00 - 10:59	Late Morning	37,9	115	378,4	378,4	19,05	603,82	202,64
2019/05/28	CH2205	Male	Three y/o	Treatment (Wk 2)	Treatment	Feed Day	Yes	11:00	11:00 - 11:59	Late Morning	37,9	150	110,6	110,6	19,05	621,86	159,36
2019/05/28	CH2206	Male	Three y/o	Treatment (Wk 2)	Treatment	Feed Day	Yes	11:00	11:00 - 11:59	Late Morning	37,9	112	157,0	157,0	19,05	601,96	171,62
2019/05/28	CH2205	Male	Three y/o	Treatment (Wk 2)	Treatment	Feed Day	Yes	11:05	11:00 - 11:59	Late Morning	38,0	80	23,4	23,4	19,15	577,31	105,68
2019/05/28	CH2206	Male	Three y/o	Treatment (Wk 2)	Treatment	Feed Day	Yes	11:05	11:00 - 11:59	Late Morning	38,0	124	118,4	118,4	19,15	609,04	161,74
2019/05/28	CH2205	Male	Three y/o	Treatment (Wk 2)	Treatment	Feed Day	Yes	11:10	11:00 - 11:59	Late Morning	38,0	45	20,0	20,0	19,15	530,47	100,31
2019/05/28	CH2206	Male	Three y/o	Treatment (Wk 2)	Treatment	Feed Day	Yes	11:10	11:00 - 11:59	Late Morning	38,0	118	202,8	202,8	19,15	605,61	180,61
2019/05/28	CH2205	Male	Three y/o	Treatment (Wk 2)	Treatment	Feed Day	Yes	11:15	11:00 - 11:59	Late Morning	37,7	138	100,4	100,4	18,85	616,31	155,98
2019/05/28	CH2206	Male	Three y/o	Treatment (Wk 2)	Treatment	Feed Day	Yes	11:15	11:00 - 11:59	Late Morning	38,0	145	191,6	191,6	19,15	619,62	178,61
2019/05/28	CH2205	Male	Three y/o	Treatment (Wk 2)	Treatment	Feed Day	Yes	11:20	11:00 - 11:59	Late Morning	37,9	85	13,8	13,8	19,05	581,90	87,68
2019/05/28	CH2206	Male	Three y/o	Treatment (Wk 2)	Treatment	Feed Day	Yes	11:20	11:00 - 11:59	Late Morning	38,1	124	64,2	64,2	19,26	609,04	140,44
2019/05/28	CH2205	Male	Three y/o	Treatment (Wk 2)	Treatment	Feed Day	Yes	11:25	11:00 - 11:59	Late Morning	37,7	88	18,4	18,4	18,85	584,49	97,47
2019/05/28	CH2206	Male	Three y/o	Treatment (Wk 2)	Treatment	Feed Day	Yes	11:25	11:00 - 11:59	Late Morning	38,0	60	40,0	40,0	19,15	554,67	124,09
2019/05/28	CH2205	Male	Three y/o	Treatment (Wk 2)	Treatment	Feed Day	No	11:30	11:00 - 11:59	Late Morning	37,5		14,0	14,0	18,65		88,17
2019/05/28	CH2206	Male	Three y/o	Treatment (Wk 2)	Treatment	Feed Day	No	11:30	11:00 - 11:59	Late Morning	38,0	70	27,6	27,6	19,15	566,99	111,33
2019/05/28	CH2205	Male	Three y/o	Treatment (Wk 2)	Treatment	Feed Day	No	11:35	11:00 - 11:59	Late Morning	37,6	57	13,8	13,8	18,75	550,47	87,68

2019/05/28	CH2206	Male	Three y/o	Treatment (Wk 2)	Treatment	Feed Day	No	11:35	11:00 - 11:59	Late Morning	37,9	51	29,4	29,4	19,05	541,20	113,50
2019/05/28	CH2205	Male	Three y/o	Treatment (Wk 2)	Treatment	Feed Day	No	11:40	11:00 - 11:59	Late Morning	37,7	60	13,2	13,2	18,85	554,67	86,17
2019/05/28	CH2206	Male	Three y/o	Treatment (Wk 2)	Treatment	Feed Day	No	11:40	11:00 - 11:59	Late Morning	37,9	46	46,2	46,2	19,05	532,38	129,06
2019/05/28	CH2205	Male	Three y/o	Treatment (Wk 2)	Treatment	Feed Day	No	11:45	11:00 - 11:59	Late Morning	37,7		85,6	85,6	18,85		150,43
2019/05/28	CH2206	Male	Three y/o	Treatment (Wk 2)	Treatment	Feed Day	No	11:45	11:00 - 11:59	Late Morning	37,9	48	242,4	242,4	19,05	536,04	186,89
2019/05/28	CH2205	Male	Three y/o	Treatment (Wk 2)	Treatment	Feed Day	No	11:50	11:00 - 11:59	Late Morning	37,8	119	190,0	190,0	18,95	606,20	178,31
2019/05/28	CH2206	Male	Three y/o	Treatment (Wk 2)	Treatment	Feed Day	No	11:50	11:00 - 11:59	Late Morning	37,9		74,8	74,8	19,05		145,74
2019/05/28	CH2205	Male	Three y/o	Treatment (Wk 2)	Treatment	Feed Day	No	11:55	11:00 - 11:59	Late Morning	37,7	100	57,4	57,4	18,85	593,87	136,56
2019/05/28	CH2206	Male	Three y/o	Treatment (Wk 2)	Treatment	Feed Day	No	11:55	11:00 - 11:59	Late Morning	37,9	111	263,6	263,6	19,05	601,33	189,85
2019/05/28	CH2205	Male	Three y/o	Treatment (Wk 2)	Treatment	Feed Day	No	12:00	12:00 - 12:59	Afternoon	37,9		124,2	124,2	19,05		163,41
2019/05/28	CH2206	Male	Three y/o	Treatment (Wk 2)	Treatment	Feed Day	No	12:00	12:00 - 12:59	Afternoon	37,9	125	121,8	121,8	19,05	609,60	162,73
2019/05/28	CH2205	Male	Three y/o	Treatment (Wk 2)	Treatment	Feed Day	No	12:05	12:00 - 12:59	Afternoon	37,9	110	42,6	42,6	19,05	600,69	126,26
2019/05/28	CH2206	Male	Three y/o	Treatment (Wk 2)	Treatment	Feed Day	No	12:05	12:00 - 12:59	Afternoon	38,0	162	85,6	85,6	19,15	626,88	150,43
2019/05/28	CH2205	Male	Three y/o	Treatment (Wk 2)	Treatment	Feed Day	No	12:10	12:00 - 12:59	Afternoon	37,5		23,6	23,6	18,65		105,97
2019/05/28	CH2206	Male	Three y/o	Treatment (Wk 2)	Treatment	Feed Day	No	12:10	12:00 - 12:59	Afternoon	37,8	119	57,0	57,0	18,95	606,20	136,32
2019/05/28	CH2205	Male	Three y/o	Treatment (Wk 2)	Treatment	Feed Day	No	12:15	12:00 - 12:59	Afternoon	37,4	90	43,4	43,4	18,55	586,16	126,90
2019/05/28	CH2206	Male	Three y/o	Treatment (Wk 2)	Treatment	Feed Day	No	12:15	12:00 - 12:59	Afternoon	37,7	124	85,4	85,4	18,85	609,04	150,35
2019/05/28	CH2205	Male	Three y/o	Treatment (Wk 2)	Treatment	Feed Day	No	12:20	12:00 - 12:59	Afternoon	37,5	44	177,0	177,0	18,65	528,51	175,82
2019/05/28	CH2206	Male	Three y/o	Treatment (Wk 2)	Treatment	Feed Day	No	12:20	12:00 - 12:59	Afternoon	37,5	180	97,4	97,4	18,65	633,61	154,93
2019/05/28	CH2205	Male	Three y/o	Treatment (Wk 2)	Treatment	Feed Day	No	12:25	12:00 - 12:59	Afternoon	37,6	132	165,6	165,6	18,75	613,32	173,49
2019/05/28	CH2206	Male	Three y/o	Treatment (Wk 2)	Treatment	Feed Day	No	12:25	12:00 - 12:59	Afternoon	37,7	126	142,4	142,4	18,85	610,14	168,20
2019/05/28	CH2205	Male	Three y/o	Treatment (Wk 2)	Treatment	Feed Day	No	12:30	12:00 - 12:59	Afternoon	37,6	119	221,6	221,6	18,75	606,20	183,73
2019/05/28	CH2206	Male	Three y/o	Treatment (Wk 2)	Treatment	Feed Day	No	12:30	12:00 - 12:59	Afternoon	37,7	158	83,6	83,6	18,85	625,26	149,61
2019/05/28	CH2205	Male	Three y/o	Treatment (Wk 2)	Treatment	Feed Day	No	12:35	12:00 - 12:59	Afternoon	37,7	58	393,2	393,2	18,85	551,90	204,01
2019/05/28	CH2206	Male	Three y/o	Treatment (Wk 2)	Treatment	Feed Day	No	12:35	12:00 - 12:59	Afternoon	37,8	141	118,2	118,2	18,95	617,75	161,68
2019/05/28	CH2205	Male	Three y/o	Treatment (Wk 2)	Treatment	Feed Day	No	12:40	12:00 - 12:59	Afternoon	37,7	94	39,6	39,6	18,85	589,37	123,74
2019/05/28	CH2206	Male	Three y/o	Treatment (Wk 2)	Treatment	Feed Day	No	12:40	12:00 - 12:59	Afternoon	37,9	123	190,6	190,6	19,05	608,49	178,43
2019/05/28	CH2205	Male	Three y/o	Treatment (Wk 2)	Treatment	Feed Day	No	12:45	12:00 - 12:59	Afternoon	37,9	115	34,0	34,0	19,05	603,82	118,49
2019/05/28	CH2206	Male	Three y/o	Treatment (Wk 2)	Treatment	Feed Day	No	12:45	12:00 - 12:59	Afternoon	37,9	63	258,2	258,2	19,05	558,62	189,12
2019/05/28	CH2205	Male	Three y/o	Treatment (Wk 2)	Treatment	Feed Day	No	12:50	12:00 - 12:59	Afternoon	37,9	93	199,2	199,2	19,05	588,58	179,98
2019/05/28	CH2206	Male	Three y/o	Treatment (Wk 2)	Treatment	Feed Day	No	12:50	12:00 - 12:59	Afternoon	37,9	119	211,0	211,0	19,05	606,20	182,00
2019/05/28	CH2205	Male	Three y/o	Treatment (Wk 2)	Treatment	Feed Day	No	12:55	12:00 - 12:59	Afternoon	37,9	106	110,0	110,0	19,05	598,06	159,17
2019/05/28	CH2206	Male	Three y/o	Treatment (Wk 2)	Treatment	Feed Day	No	12:55	12:00 - 12:59	Afternoon	37,9	132	295,2	295,2	19,05	613,32	193,85
2019/05/28	CH2205	Male	Three y/o	Treatment (Wk 2)	Treatment	Feed Day	No	13:00	13:00 - 13:59	Afternoon	37,9	89	28,4	28,4	19,05	585,33	112,31
2019/05/28	CH2206	Male	Three y/o	Treatment (Wk 2)	Treatment	Feed Day	No	13:00	13:00 - 13:59	Afternoon	37,9	131	27,6	27,6	19,05	612,80	111,33
2019/05/28	CH2205	Male	Three y/o	Treatment (Wk 2)	Treatment	Feed Day	No	13:05	13:00 - 13:59	Afternoon	38,0	80	60,0	60,0	19,15	577,31	138,09
2019/05/28	CH2206	Male	Three y/o	Treatment (Wk 2)	Treatment	Feed Day	No	13:05	13:00 - 13:59	Afternoon	38,0	110	22,0	22,0	19,15	600,69	103,57
2019/05/28	CH2205	Male	Three y/o	Treatment (Wk 2)	Treatment	Feed Day	No	13:10	13:00 - 13:59	Afternoon	38,0	85	108,8	108,8	19,15	581,90	158,79
2019/05/28	CH2206	Male	Three y/o	Treatment (Wk 2)	Treatment	Feed Day	No	13:10	13:00 - 13:59	Afternoon	37,9	108	32,2	32,2	19,05	599,39	116,63
2019/05/28	CH2205	Male	Three y/o	Treatment (Wk 2)	Treatment	Feed Day	No	13:15	13:00 - 13:59	Afternoon	37,9	145	21,2	21,2	19,05	619,62	102,31
2019/05/28	CH2206	Male	Three y/o	Treatment (Wk 2)	Treatment	Feed Day	No	13:15	13:00 - 13:59	Afternoon	37,9	102	53,2	53,2	19,05	595,30	133,93
2019/05/28	CH2205	Male	Three y/o	Treatment (Wk 2)	Treatment	Feed Day	No	13:20	13:00 - 13:59	Afternoon	37,9	80	7,0	7,0	19,05	577,31	64,72
2019/05/28	CH2206	Male	Three y/o	Treatment (Wk 2)	Treatment	Feed Day	No	13:20	13:00 - 13:59	Afternoon	37,9	98	24,8	24,8	19,05	592,41	107,67
2019/05/28	CH2205	Male	Three y/o	Treatment (Wk 2)	Treatment	Feed Day	No	13:25	13:00 - 13:59	Afternoon	37,9		8,6	8,6	19,05		71,66
2019/05/28	CH2206	Male	Three y/o	Treatment (Wk 2)	Treatment	Feed Day	No	13:25	13:00 - 13:59	Afternoon	37,9	109	14,0	14,0	19,05	600,04	88,17
2019/05/28	CH2205	Male	Three y/o	Treatment (Wk 2)	Treatment	Feed Day	No	13:30	13:00 - 13:59	Afternoon	37,9		17,0	17,0	19,05		94,77
2019/05/28	CH2206	Male	Three y/o	Treatment (Wk 2)	Treatment	Feed Day	No	13:30	13:00 - 13:59	Afternoon	37,9	89	24,4	24,4	19,05	585,33	107,11
2019/05/28	CH2205	Male	Three y/o	Treatment (Wk 2)	Treatment	Feed Day	No	13:35	13:00 - 13:59	Afternoon	37,9		49,2	49,2	19,05		131,23
2019/05/28	CH2206	Male	Three y/o	Treatment (Wk 2)	Treatment	Feed Day	No	13:35	13:00 - 13:59	Afternoon	37,9	86	49,6	49,6	19,05	582,77	131,51
2019/05/28	CH2205	Male	Three y/o	Treatment (Wk 2)	Treatment	Feed Day	No	13:40	13:00 - 13:59	Afternoon	37,9	53	46,8	46,8	19,05	544,43	129,50
2019/05/28	CH2206	Male	Three y/o	Treatment (Wk 2)	Treatment	Feed Day	No	13:40	13:00 - 13:59	Afternoon	38,0	115	70,0	70,0	19,15	603,82	143,44
2019/05/28	CH2205	Male	Three y/o	Treatment (Wk 2)	Treatment	Feed Day	No	13:45	13:00 - 13:59	Afternoon	37,9		44,6	44,6	19,05		127,84
2019/05/28	CH2206	Male	Three y/o	Treatment (Wk 2)	Treatment	Feed Day	No	13:45	13:00 - 13:59	Afternoon	38,0	123	43,6	43,6	19,15	608,49	127,06
2019/05/28	CH2205	Male	Three y/o	Treatment (Wk 2)	Treatment	Feed Day	No	13:50	13:00 - 13:59	Afternoon	37,8	56	23,8	23,8	18,95	549,01	106,26
2019/05/28	CH2206	Male	Three y/o	Treatment (Wk 2)	Treatment	Feed Day	No	13:50	13:00 - 13:59	Afternoon	37,9	112	113,4	113,4	19,05	601,96	160,23
2019/05/28	CH2205	Male	Three y/o	Treatment (Wk 2)	Treatment	Feed Day	No	13:55	13:00 - 13:59	Afternoon	37,7		13,6	13,6	18,85		87,18
2019/05/28	CH2206	Male	Three y/o	Treatment (Wk 2)	Treatment	Feed Day	No	13:55	13:00 - 13:59	Afternoon	37,9		49,4	49,4	19,05		131,37
2019/05/28	CH2205	Male	Three y/o	Treatment (Wk 2)	Treatment	Feed Day	No	14:00	14:00 - 14:59	Afternoon	37,9	80	313,2	313,2	19,05	577,31	195,94
2019/05/28	CH2206	Male	Three y/o	Treatment (Wk 2)	Treatment	Feed Day	No	14:00	14:00 - 14:59	Afternoon	37,9	177	21,8	21,8	19,05	632,55	103,26
2019/05/28	CH2205	Male	Three y/o	Treatment (Wk 2)	Treatment	Feed Day	No	14:05	14:00 - 14:59	Afternoon	38,0	143	8,8	8,8	19,15	618,69	72,43
2019/05/28	CH2206	Male	Three y/o	Treatment (Wk 2)	Treatment	Feed Day	No	14:05	14:00 - 14:59	Afternoon	37,9	119	21,2	21,2	19,05	606,20	102,31
2019/05/28	CH2205	Male	Three y/o	Treatment (Wk 2)	Treatment	Feed Day	No	14:10	14:00 - 14:59	Afternoon	38,0	59	3,2	3,2	19,15	553,30	38,49
2019/05/28	CH2206	Male	Three y/o	Treatment (Wk 2)	Treatment	Feed Day	No	14:10	14:00 - 14:59	Afternoon	37,9	95	13,0	13,0	19,05	590,14	85,65
2019/05/28	CH2205	Male	Three y/o	Treatment (Wk 2)	Treatment	Feed Day	No	14:15	14:00 - 14:59	Afternoon	38,1	84	4,0	4,0	19,26	581,01	45,94
2019/05/28	CH2206	Male	Three y/o	Treatment (Wk 2)	Treatment	Feed Day	No	14:15	14:00 - 14:59	Afternoon	37,9	110	5,4	5,4	19,05	600,69	55,99
2019/05/28	CH2205	Male	Three y/o	Treatment (Wk 2)	Treatment	Feed Day	No	14:20	14:00 - 14:59	Afternoon	38,1	78	7,0	7,0	19,26	575,38	64,72
2019/05/28	CH2206	Male	Three y/o	Treatment (Wk 2)	Treatment	Feed Day	No	14:20	14:00 - 14:59	Afternoon	37,9	84	4,0	4,0	19,05	581,01	45,94
2019/05/28	CH2205	Male	Three y/o	Treatment (Wk 2)	Treatment	Feed Day	No	14:25	14:00 - 14:59	Afternoon	38,1	80	13,8	13,8	19,26	577,31	87,68
2019/05/28	CH2206	Male	Three y/o	Treatment (Wk 2)	Treatment	Feed Day	No	14:25	14:00 - 14:59	Afternoon	38,0	49	10,0	10,0	19,15	537,80	76,76
2019/05/28	CH2205	Male	Three y/o	Treatment (Wk 2)	Treatment	Feed Day	No	14:30	14:00 - 14:59	Afternoon	38,1	63	17,2	17,2	19,26	558,62	95,17

2019/05/28	CH2206	Male	Three y/o	Treatment (Wk 2)	Treatment	Feed Day	No	14:30	14:00 - 14:59	Afternoon	38,0	88	18,4	18,4	19,15	584,49	97,47
2019/05/28	CH2205	Male	Three y/o	Treatment (Wk 2)	Treatment	Feed Day	No	14:35	14:00 - 14:59	Afternoon	38,1	64	17,8	17,8	19,26	559,88	96,34
2019/05/28	CH2206	Male	Three y/o	Treatment (Wk 2)	Treatment	Feed Day	No	14:35	14:00 - 14:59	Afternoon	38,0	74	11,2	11,2	19,15	571,33	80,59
2019/05/28	CH2205	Male	Three y/o	Treatment (Wk 2)	Treatment	Feed Day	No	14:40	14:00 - 14:59	Afternoon	38,1	114	7,8	7,8	19,26	603,20	68,36
2019/05/28	CH2206	Male	Three y/o	Treatment (Wk 2)	Treatment	Feed Day	No	14:40	14:00 - 14:59	Afternoon	38,0	105	7,0	7,0	19,15	597,38	64,72
2019/05/28	CH2205	Male	Three y/o	Treatment (Wk 2)	Treatment	Feed Day	No	14:45	14:00 - 14:59	Afternoon	38,1	66	2,8	2,8	19,26	562,34	34,04
2019/05/28	CH2206	Male	Three y/o	Treatment (Wk 2)	Treatment	Feed Day	No	14:45	14:00 - 14:59	Afternoon	38,0	108	7,2	7,2	19,15	599,39	65,66
2019/05/28	CH2205	Male	Three y/o	Treatment (Wk 2)	Treatment	Feed Day	No	14:50	14:00 - 14:59	Afternoon	38,1	67	6,2	6,2	19,26	563,53	60,63
2019/05/28	CH2206	Male	Three y/o	Treatment (Wk 2)	Treatment	Feed Day	No	14:50	14:00 - 14:59	Afternoon	38,0	106	16,8	16,8	19,15	598,06	94,37
2019/05/28	CH2205	Male	Three y/o	Treatment (Wk 2)	Treatment	Feed Day	No	14:55	14:00 - 14:59	Afternoon	38,1	84	2,0	2,0	19,26	581,01	22,86
2019/05/28	CH2206	Male	Three y/o	Treatment (Wk 2)	Treatment	Feed Day	No	14:55	14:00 - 14:59	Afternoon	38,0	106	42,4	42,4	19,15	598,06	126,10
2019/05/28	CH2205	Male	Three y/o	Treatment (Wk 2)	Treatment	Feed Day	No	15:00	15:00 - 15:59	Afternoon	38,1	83	6,2	6,2	19,26	580,11	60,63
2019/05/28	CH2206	Male	Three y/o	Treatment (Wk 2)	Treatment	Feed Day	No	15:00	15:00 - 15:59	Afternoon	38,0	76	48,6	48,6	19,15	573,39	130,81
2019/05/28	CH2205	Male	Three y/o	Treatment (Wk 2)	Treatment	Feed Day	No	15:05	15:00 - 15:59	Afternoon	38,1	48	2,6	2,6	19,26	536,04	31,57
2019/05/28	CH2206	Male	Three y/o	Treatment (Wk 2)	Treatment	Feed Day	No	15:05	15:00 - 15:59	Afternoon	37,9	99	35,8	35,8	19,05	593,14	120,27
2019/05/28	CH2205	Male	Three y/o	Treatment (Wk 2)	Treatment	Feed Day	No	15:10	15:00 - 15:59	Afternoon	38,1		25,0	25,0	19,26		107,94
2019/05/28	CH2206	Male	Three y/o	Treatment (Wk 2)	Treatment	Feed Day	No	15:10	15:00 - 15:59	Afternoon	37,9	137	27,2	27,2	19,05	615,83	110,83
2019/05/28	CH2205	Male	Three y/o	Treatment (Wk 2)	Treatment	Feed Day	No	15:15	15:00 - 15:59	Afternoon	38,1	108	38,6	38,6	19,26	599,39	122,86
2019/05/28	CH2206	Male	Three y/o	Treatment (Wk 2)	Treatment	Feed Day	No	15:15	15:00 - 15:59	Afternoon	37,9	132	50,0	50,0	19,05	613,32	131,79
2019/05/28	CH2205	Male	Three y/o	Treatment (Wk 2)	Treatment	Feed Day	No	15:20	15:00 - 15:59	Afternoon	38,0	114	26,4	26,4	19,15	603,20	109,81
2019/05/28	CH2206	Male	Three y/o	Treatment (Wk 2)	Treatment	Feed Day	No	15:20	15:00 - 15:59	Afternoon	37,9	121	115,2	115,2	19,05	607,35	160,78
2019/05/28	CH2205	Male	Three y/o	Treatment (Wk 2)	Treatment	Feed Day	No	15:25	15:00 - 15:59	Afternoon	37,9	76	22,4	22,4	19,05	573,39	104,19
2019/05/28	CH2206	Male	Three y/o	Treatment (Wk 2)	Treatment	Feed Day	No	15:25	15:00 - 15:59	Afternoon	37,9	124	189,6	189,6	19,05	609,04	178,24
2019/05/28	CH2205	Male	Three y/o	Treatment (Wk 2)	Treatment	Feed Day	No	15:30	15:00 - 15:59	Afternoon	37,9		34,2	34,2	19,05		118,70
2019/05/28	CH2206	Male	Three y/o	Treatment (Wk 2)	Treatment	Feed Day	No	15:30	15:00 - 15:59	Afternoon	37,9	120	72,8	72,8	19,05	606,78	144,80
2019/05/28	CH2205	Male	Three y/o	Treatment (Wk 2)	Treatment	Feed Day	No	15:35	15:00 - 15:59	Afternoon	37,7		29,4	29,4	18,85		113,50
2019/05/28	CH2206	Male	Three y/o	Treatment (Wk 2)	Treatment	Feed Day	No	15:35	15:00 - 15:59	Afternoon	37,9	131	111,0	111,0	19,05	612,80	159,48
2019/05/28	CH2205	Male	Three y/o	Treatment (Wk 2)	Treatment	Feed Day	No	15:40	15:00 - 15:59	Afternoon	37,6	83	22,2	22,2	18,75	580,11	103,88
2019/05/28	CH2206	Male	Three y/o	Treatment (Wk 2)	Treatment	Feed Day	No	15:40	15:00 - 15:59	Afternoon	38,0	124	158,2	158,2	19,15	609,04	171,88
2019/05/28	CH2205	Male	Three y/o	Treatment (Wk 2)	Treatment	Feed Day	No	15:45	15:00 - 15:59	Afternoon	37,6	93	991,2		18,75	588,58	
2019/05/28	CH2206	Male	Three y/o	Treatment (Wk 2)	Treatment	Feed Day	No	15:45	15:00 - 15:59	Afternoon	38,0	143	119,6	119,6	19,15	618,69	162,09
2019/05/28	CH2205	Male	Three y/o	Treatment (Wk 2)	Treatment	Feed Day	No	15:50	15:00 - 15:59	Afternoon	37,9	88	10,6	10,6	19,05	584,49	78,73
2019/05/28	CH2206	Male	Three y/o	Treatment (Wk 2)	Treatment	Feed Day	No	15:50	15:00 - 15:59	Afternoon	38,2	119	28,6	28,6	19,36	606,20	112,56
2019/05/28	CH2205	Male	Three y/o	Treatment (Wk 2)	Treatment	Feed Day	No	15:55	15:00 - 15:59	Afternoon	38,0		23,2	23,2	19,15		105,39
2019/05/28	CH2206	Male	Three y/o	Treatment (Wk 2)	Treatment	Feed Day	No	15:55	15:00 - 15:59	Afternoon	38,2		57,4	57,4	19,36		136,56
2019/05/28	CH2205	Male	Three y/o	Treatment (Wk 2)	Treatment	Feed Day	No	16:00	16:00 - 16:59	Evening	37,7		26,0	26,0	18,85		109,29
2019/05/28	CH2206	Male	Three y/o	Treatment (Wk 2)	Treatment	Feed Day	No	16:00	16:00 - 16:59	Evening	38,2	140	90,6	90,6	19,36	617,28	152,40
2019/05/28	CH2205	Male	Three y/o	Treatment (Wk 2)	Treatment	Feed Day	No	16:05	16:00 - 16:59	Evening	37,6	77	15,4	15,4	18,75	574,39	91,41
2019/05/28	CH2206	Male	Three y/o	Treatment (Wk 2)	Treatment	Feed Day	No	16:05	16:00 - 16:59	Evening	38,2	101	12,6	12,6	19,36	594,59	84,59
2019/05/28	CH2205	Male	Three y/o	Treatment (Wk 2)	Treatment	Feed Day	No	16:10	16:00 - 16:59	Evening	37,7	97	88,8	88,8	18,85	591,66	151,71
2019/05/28	CH2206	Male	Three y/o	Treatment (Wk 2)	Treatment	Feed Day	No	16:10	16:00 - 16:59	Evening	38,2		88,2	88,2	19,36		151,47
2019/05/28	CH2205	Male	Three y/o	Treatment (Wk 2)	Treatment	Feed Day	No	16:15	16:00 - 16:59	Evening	37,9	103	25,2	25,2	19,05	596,00	108,22
2019/05/28	CH2206	Male	Three y/o	Treatment (Wk 2)	Treatment	Feed Day	No	16:15	16:00 - 16:59	Evening	38,2	165	43,8	43,8	19,36	628,06	127,22
2019/05/28	CH2205	Male	Three y/o	Treatment (Wk 2)	Treatment	Feed Day	No	16:20	16:00 - 16:59	Evening	37,9	88	21,0	21,0	19,05	584,49	101,98
2019/05/28	CH2206	Male	Three y/o	Treatment (Wk 2)	Treatment	Feed Day	No	16:20	16:00 - 16:59	Evening	38,1	129	136,0	136,0	19,26	611,75	166,59
2019/05/28	CH2205	Male	Three y/o	Treatment (Wk 2)	Treatment	Feed Day	No	16:25	16:00 - 16:59	Evening	37,9	79	11,6	11,6	19,05	576,36	81,78
2019/05/28	CH2206	Male	Three y/o	Treatment (Wk 2)	Treatment	Feed Day	No	16:25	16:00 - 16:59	Evening	38,2	59	152,6	152,6	19,36	553,30	170,62
2019/05/28	CH2205	Male	Three y/o	Treatment (Wk 2)	Treatment	Feed Day	No	16:30	16:00 - 16:59	Evening	38,0	67	20,4	20,4	19,15	563,53	100,99
2019/05/28	CH2206	Male	Three y/o	Treatment (Wk 2)	Treatment	Feed Day	No	16:30	16:00 - 16:59	Evening	38,1	112	51,4	51,4	19,26	601,96	132,74
2019/05/28	CH2205	Male	Three y/o	Treatment (Wk 2)	Treatment	Feed Day	No	16:35	16:00 - 16:59	Evening	38,0		28,2	28,2	19,15		112,07
2019/05/28	CH2206	Male	Three y/o	Treatment (Wk 2)	Treatment	Feed Day	No	16:35	16:00 - 16:59	Evening	38,1	72	27,0	27,0	19,26	569,20	110,58
2019/05/28	CH2205	Male	Three y/o	Treatment (Wk 2)	Treatment	Feed Day	No	16:40	16:00 - 16:59	Evening	37,9	111	24,4	24,4	19,05	601,33	107,11
2019/05/28	CH2206	Male	Three y/o	Treatment (Wk 2)	Treatment	Feed Day	No	16:40	16:00 - 16:59	Evening	38,2	94	25,2	25,2	19,36	589,37	108,22
2019/05/28	CH2205	Male	Three y/o	Treatment (Wk 2)	Treatment	Feed Day	No	16:45	16:00 - 16:59	Evening	37,9	74	15,0	15,0	19,05	571,33	90,51
2019/05/28	CH2206	Male	Three y/o	Treatment (Wk 2)	Treatment	Feed Day	No	16:45	16:00 - 16:59	Evening	38,2	112	27,0	27,0	19,36	601,96	110,58
2019/05/28	CH2205	Male	Three y/o	Treatment (Wk 2)	Treatment	Feed Day	No	16:50	16:00 - 16:59	Evening	38,0	82	18,4	18,4	19,15	579,19	97,47
2019/05/28	CH2206	Male	Three y/o	Treatment (Wk 2)	Treatment	Feed Day	No	16:50	16:00 - 16:59	Evening	38,2	111	41,6	41,6	19,36	601,33	125,44
2019/05/28	CH2205	Male	Three y/o	Treatment (Wk 2)	Treatment	Feed Day	No	16:55	16:00 - 16:59	Evening	38,0	94	19,2	19,2	19,15	589,37	98,92
2019/05/28	CH2206	Male	Three y/o	Treatment (Wk 2)	Treatment	Feed Day	No	16:55	16:00 - 16:59	Evening	38,2	108	47,0	47,0	19,36	599,39	129,65
2019/05/28	CH2205	Male	Three y/o	Treatment (Wk 2)	Treatment	Feed Day	No	17:00	17:00 - 17:59	Evening	38,1	92	18,4	18,4	19,26	587,78	97,47
2019/05/28	CH2206	Male	Three y/o	Treatment (Wk 2)	Treatment	Feed Day	No	17:00	17:00 - 17:59	Evening	38,1	101	43,6	43,6	19,26	594,59	127,06
2019/05/28	CH2205	Male	Three y/o	Treatment (Wk 2)	Treatment	Feed Day	No	17:05	17:00 - 17:59	Evening	38,1	78	17,4	17,4	19,26	575,38	95,56
2019/05/28	CH2206	Male	Three y/o	Treatment (Wk 2)	Treatment	Feed Day	No	17:05	17:00 - 17:59	Evening	38,0	119	11,6	11,6	19,15	606,20	81,78
2019/05/28	CH2205	Male	Three y/o	Treatment (Wk 2)	Treatment	Feed Day	No	17:10	17:00 - 17:59	Evening	38,1	76	17,8	17,8	19,26	573,39	96,34
2019/05/28	CH2206	Male	Three y/o	Treatment (Wk 2)	Treatment	Feed Day	No	17:10	17:00 - 17:59	Evening	38,0	115	22,6	22,6	19,15	603,82	104,49
2019/05/28	CH2205	Male	Three y/o	Treatment (Wk 2)	Treatment	Feed Day	No	17:15	17:00 - 17:59	Evening	38,2	56	29,2	29,2	19,36	549,01	113,27
2019/05/28	CH2206	Male	Three y/o	Treatment (Wk 2)	Treatment	Feed Day	No	17:15	17:00 - 17:59	Evening	38,0	98	44,8	44,8	19,15	592,41	127,99
2019/05/28	CH2205	Male	Three y/o	Treatment (Wk 2)	Treatment	Feed Day	No	17:20	17:00 - 17:59	Evening	38,2	89	22,8	22,8	19,36	585,33	104,79
2019/05/28	CH2206	Male	Three y/o	Treatment (Wk 2)	Treatment	Feed Day	No	17:20	17:00 - 17:59	Evening	37,9	96	56,6	56,6	19,05	590,91	136,07
2019/05/28	CH2205	Male	Three y/o	Treatment (Wk 2)	Treatment	Feed Day	No	17:25	17:00 - 17:59	Evening	38,2	101	26,2	26,2	19,36	594,59	109,55

2019/05/28	CH2206	Male	Three y/o	Treatment (Wk 2)	Treatment	Feed Day	No	17:25	17:00 - 17:59	Evening	37,8	98	51,4	51,4	18,95	592,41	132,74
2019/05/28	CH2205	Male	Three y/o	Treatment (Wk 2)	Treatment	Feed Day	No	17:30	17:00 - 17:59	Evening	38,2	120	57,2	57,2	19,36	606,78	136,44
2019/05/28	CH2206	Male	Three y/o	Treatment (Wk 2)	Treatment	Feed Day	No	17:30	17:00 - 17:59	Evening	37,7		21,0	21,0	18,85		101,98
2019/05/28	CH2205	Male	Three y/o	Treatment (Wk 2)	Treatment	Feed Day	No	17:35	17:00 - 17:59	Evening	38,0	129	51,6	51,6	19,15	611,75	132,87
2019/05/28	CH2206	Male	Three y/o	Treatment (Wk 2)	Treatment	Feed Day	No	17:35	17:00 - 17:59	Evening	37,8	113	26,6	26,6	18,95	602,58	110,07
2019/05/28	CH2205	Male	Three y/o	Treatment (Wk 2)	Treatment	Feed Day	No	17:40	17:00 - 17:59	Evening	38,0	105	46,0	46,0	19,15	597,38	128,91
2019/05/28	CH2206	Male	Three y/o	Treatment (Wk 2)	Treatment	Feed Day	No	17:40	17:00 - 17:59	Evening	37,8		131,4	131,4	18,95		165,38
2019/05/28	CH2205	Male	Three y/o	Treatment (Wk 2)	Treatment	Feed Day	No	17:45	17:00 - 17:59	Evening	38,1	128	263,2	263,2	19,26	611,22	189,80
2019/05/28	CH2206	Male	Three y/o	Treatment (Wk 2)	Treatment	Feed Day	No	17:45	17:00 - 17:59	Evening	37,8	158	48,2	48,2	18,95	625,26	130,52
2019/05/28	CH2205	Male	Three y/o	Treatment (Wk 2)	Treatment	Feed Day	No	17:50	17:00 - 17:59	Evening	38,3	109	80,2	80,2	19,46	600,04	148,16
2019/05/28	CH2206	Male	Three y/o	Treatment (Wk 2)	Treatment	Feed Day	No	17:50	17:00 - 17:59	Evening	37,8	84	44,4	44,4	18,95	581,01	127,69
2019/05/28	CH2205	Male	Three y/o	Treatment (Wk 2)	Treatment	Feed Day	No	17:55	17:00 - 17:59	Evening	38,4		87,8	87,8	19,56		151,31
2019/05/28	CH2206	Male	Three y/o	Treatment (Wk 2)	Treatment	Feed Day	No	17:55	17:00 - 17:59	Evening	37,8	120	46,6	46,6	18,95	606,78	129,35
2019/05/28	CH2205	Male	Three y/o	Treatment (Wk 2)	Treatment	Feed Day	No	18:00	18:00 - 18:59	Evening	38,3	135	39,8	39,8	19,46	614,84	123,91
2019/05/28	CH2206	Male	Three y/o	Treatment (Wk 2)	Treatment	Feed Day	No	18:00	18:00 - 18:59	Evening	37,7	94	49,4	49,4	18,85	589,37	131,37
2019/05/28	CH2205	Male	Three y/o	Treatment (Wk 2)	Treatment	Feed Day	No	18:05	18:00 - 18:59	Evening	38,3	135	54,6	54,6	19,46	614,84	134,83
2019/05/28	CH2206	Male	Three y/o	Treatment (Wk 2)	Treatment	Feed Day	No	18:05	18:00 - 18:59	Evening	37,5	67	51,0	51,0	18,65	563,53	132,47
2019/05/28	CH2205	Male	Three y/o	Treatment (Wk 2)	Treatment	Feed Day	No	18:10	18:00 - 18:59	Evening	38,3	129	107,8	107,8	19,46	611,75	158,46
2019/05/28	CH2206	Male	Three y/o	Treatment (Wk 2)	Treatment	Feed Day	No	18:10	18:00 - 18:59	Evening	37,5	104	55,8	55,8	18,65	596,69	135,58
2019/05/28	CH2205	Male	Three y/o	Treatment (Wk 2)	Treatment	Feed Day	No	18:15	18:00 - 18:59	Evening	38,2	124	29,8	29,8	19,36	609,04	113,97
2019/05/28	CH2206	Male	Three y/o	Treatment (Wk 2)	Treatment	Feed Day	No	18:15	18:00 - 18:59	Evening	37,4	82	50,2	50,2	18,55	579,19	131,92
2019/05/28	CH2205	Male	Three y/o	Treatment (Wk 2)	Treatment	Feed Day	No	18:20	18:00 - 18:59	Evening	38,2		150,8	150,8	19,36		170,20
2019/05/28	CH2206	Male	Three y/o	Treatment (Wk 2)	Treatment	Feed Day	No	18:20	18:00 - 18:59	Evening	37,4	94	42,0	42,0	18,55	589,37	125,77
2019/05/28	CH2205	Male	Three y/o	Treatment (Wk 2)	Treatment	Feed Day	No	18:25	18:00 - 18:59	Evening	38,3	84	256,4	256,4	19,46	581,01	188,87
2019/05/28	CH2206	Male	Three y/o	Treatment (Wk 2)	Treatment	Feed Day	No	18:25	18:00 - 18:59	Evening	37,5		46,2	46,2	18,65		129,06
2019/05/28	CH2205	Male	Three y/o	Treatment (Wk 2)	Treatment	Feed Day	No	18:30	18:00 - 18:59	Evening	38,3	68	125,0	125,0	19,46	564,71	163,63
2019/05/28	CH2206	Male	Three y/o	Treatment (Wk 2)	Treatment	Feed Day	No	18:30	18:00 - 18:59	Evening	37,4	165	49,0	49,0	18,55	628,06	131,09
2019/05/28	CH2205	Male	Three y/o	Treatment (Wk 2)	Treatment	Feed Day	No	18:35	18:00 - 18:59	Evening	38,2	172	170,6	170,6	19,36	630,73	174,53
2019/05/28	CH2206	Male	Three y/o	Treatment (Wk 2)	Treatment	Feed Day	No	18:35	18:00 - 18:59	Evening	37,5	98	46,6	46,6	18,65	592,41	129,35
2019/05/28	CH2205	Male	Three y/o	Treatment (Wk 2)	Treatment	Feed Day	No	18:40	18:00 - 18:59	Evening	38,2	44	30,6	30,6	19,36	528,51	114,87
2019/05/28	CH2206	Male	Three y/o	Treatment (Wk 2)	Treatment	Feed Day	No	18:40	18:00 - 18:59	Evening	37,4	90	44,8	44,8	18,55	586,16	127,99
2019/05/28	CH2205	Male	Three y/o	Treatment (Wk 2)	Treatment	Feed Day	No	18:45	18:00 - 18:59	Evening	38,1	123	22,0	22,0	19,26	608,49	103,57
2019/05/28	CH2206	Male	Three y/o	Treatment (Wk 2)	Treatment	Feed Day	No	18:45	18:00 - 18:59	Evening	37,4	90	53,0	53,0	18,55	586,16	133,80
2019/05/28	CH2205	Male	Three y/o	Treatment (Wk 2)	Treatment	Feed Day	No	18:50	18:00 - 18:59	Evening	37,9	77	20,6	20,6	19,05	574,39	101,32
2019/05/28	CH2206	Male	Three y/o	Treatment (Wk 2)	Treatment	Feed Day	No	18:50	18:00 - 18:59	Evening	37,3	65	58,6	58,6	18,46	561,12	137,28
2019/05/28	CH2205	Male	Three y/o	Treatment (Wk 2)	Treatment	Feed Day	No	18:55	18:00 - 18:59	Evening	37,7		19,6	19,6	18,85		99,63
2019/05/28	CH2206	Male	Three y/o	Treatment (Wk 2)	Treatment	Feed Day	No	18:55	18:00 - 18:59	Evening	37,5	106	56,0	56,0	18,65	598,06	135,70
2019/05/28	CH2205	Male	Three y/o	Treatment (Wk 2)	Treatment	Feed Day	No	19:00	19:00 - 19:59	Evening	37,6	84	17,0	17,0	18,75	581,01	94,77
2019/05/28	CH2206	Male	Three y/o	Treatment (Wk 2)	Treatment	Feed Day	No	19:00	19:00 - 19:59	Evening	37,4	80	40,4	40,4	18,55	577,31	124,43
2019/05/28	CH2205	Male	Three y/o	Treatment (Wk 2)	Treatment	Feed Day	No	19:05	19:00 - 19:59	Evening	37,7	82	17,0	17,0	18,85	579,19	94,77
2019/05/28	CH2206	Male	Three y/o	Treatment (Wk 2)	Treatment	Feed Day	No	19:05	19:00 - 19:59	Evening	37,4	89	46,6	46,6	18,55	585,33	129,35
2019/05/28	CH2205	Male	Three y/o	Treatment (Wk 2)	Treatment	Feed Day	No	19:10	19:00 - 19:59	Evening	38,0		37,4	37,4	19,15		121,77
2019/05/28	CH2206	Male	Three y/o	Treatment (Wk 2)	Treatment	Feed Day	No	19:10	19:00 - 19:59	Evening	37,2	92	52,8	52,8	18,36	587,78	133,67
2019/05/28	CH2205	Male	Three y/o	Treatment (Wk 2)	Treatment	Feed Day	No	19:15	19:00 - 19:59	Evening	38,0		30,8	30,8	19,15		115,10
2019/05/28	CH2206	Male	Three y/o	Treatment (Wk 2)	Treatment	Feed Day	No	19:15	19:00 - 19:59	Evening	37,1	96	44,2	44,2	18,26	590,91	127,53
2019/05/28	CH2205	Male	Three y/o	Treatment (Wk 2)	Treatment	Feed Day	No	19:20	19:00 - 19:59	Evening	38,0		34,2	34,2	19,15		118,70
2019/05/28	CH2206	Male	Three y/o	Treatment (Wk 2)	Treatment	Feed Day	No	19:20	19:00 - 19:59	Evening	37,2	75	38,4	38,4	18,36	572,36	122,68
2019/05/28	CH2205	Male	Three y/o	Treatment (Wk 2)	Treatment	Feed Day	No	19:25	19:00 - 19:59	Evening	38,0		36,0	36,0	19,15		120,46
2019/05/28	CH2206	Male	Three y/o	Treatment (Wk 2)	Treatment	Feed Day	No	19:25	19:00 - 19:59	Evening	37,2	94	25,2	25,2	18,36	589,37	108,22
2019/05/28	CH2205	Male	Three y/o	Treatment (Wk 2)	Treatment	Feed Day	No	19:30	19:00 - 19:59	Evening	37,7		25,6	25,6	18,85		108,76
2019/05/28	CH2206	Male	Three y/o	Treatment (Wk 2)	Treatment	Feed Day	No	19:30	19:00 - 19:59	Evening	37,0	89	10,0	10,0	18,16	585,33	76,76
2019/05/28	CH2205	Male	Three y/o	Treatment (Wk 2)	Treatment	Feed Day	No	19:35	19:00 - 19:59	Evening	37,7		27,6	27,6	18,85		111,33
2019/05/28	CH2206	Male	Three y/o	Treatment (Wk 2)	Treatment	Feed Day	No	19:35	19:00 - 19:59	Evening	37,0	66	9,8	9,8	18,16	562,34	76,07
2019/05/28	CH2205	Male	Three y/o	Treatment (Wk 2)	Treatment	Feed Day	No	19:40	19:00 - 19:59	Evening	37,7	82	24,2	24,2	18,85	579,19	106,83
2019/05/28	CH2206	Male	Three y/o	Treatment (Wk 2)	Treatment	Feed Day	No	19:40	19:00 - 19:59	Evening	37,2	72	15,8	15,8	18,36	569,20	92,28
2019/05/28	CH2205	Male	Three y/o	Treatment (Wk 2)	Treatment	Feed Day	No	19:45	19:00 - 19:59	Evening	37,9	64	17,4	17,4	19,05	559,88	95,56
2019/05/28	CH2206	Male	Three y/o	Treatment (Wk 2)	Treatment	Feed Day	No	19:45	19:00 - 19:59	Evening	37,3	74	12,8	12,8	18,46	571,33	85,12
2019/05/28	CH2205	Male	Three y/o	Treatment (Wk 2)	Treatment	Feed Day	No	19:50	19:00 - 19:59	Evening	38,0	75	23,6	23,6	19,15	572,36	105,97
2019/05/28	CH2206	Male	Three y/o	Treatment (Wk 2)	Treatment	Feed Day	No	19:50	19:00 - 19:59	Evening	37,5	177	19,4	19,4	18,65	632,55	99,28
2019/05/28	CH2205	Male	Three y/o	Treatment (Wk 2)	Treatment	Feed Day	No	19:55	19:00 - 19:59	Evening	38,0	86	21,6	21,6	19,15	582,77	102,94
2019/05/28	CH2206	Male	Three y/o	Treatment (Wk 2)	Treatment	Feed Day	No	19:55	19:00 - 19:59	Evening	37,5	94	22,6	22,6	18,65	589,37	104,49
2019/05/28	CH2205	Male	Three y/o	Treatment (Wk 2)	Treatment	Feed Day	No	20:00	20:00 - 20:59	Night	38,0	100	31,2	31,2	19,15	593,87	115,54
2019/05/28	CH2206	Male	Three y/o	Treatment (Wk 2)	Treatment	Feed Day	No	20:00	20:00 - 20:59	Night	37,5	85	25,8	25,8	18,65	581,90	109,02
2019/05/28	CH2205	Male	Three y/o	Treatment (Wk 2)	Treatment	Feed Day	No	20:05	20:00 - 20:59	Night	38,0	182	33,4	33,4	19,15	634,31	117,88
2019/05/28	CH2206	Male	Three y/o	Treatment (Wk 2)	Treatment	Feed Day	No	20:05	20:00 - 20:59	Night	37,4	45	6,4	6,4	18,55	530,47	61,70
2019/05/28	CH2205	Male	Three y/o	Treatment (Wk 2)	Treatment	Feed Day	No	20:10	20:00 - 20:59	Night	37,9		30,4	30,4	19,05		114,65
2019/05/28	CH2206	Male	Three y/o	Treatment (Wk 2)	Treatment	Feed Day	No	20:10	20:00 - 20:59	Night	36,9	85	9,6	9,6	18,06	581,90	75,37
2019/05/28	CH2205	Male	Three y/o	Treatment (Wk 2)	Treatment	Feed Day	No	20:15	20:00 - 20:59	Night	37,6		25,6	25,6	18,75		108,76
2019/05/28	CH2206	Male	Three y/o	Treatment (Wk 2)	Treatment	Feed Day	No	20:15	20:00 - 20:59	Night	36,2	62	14,0	14,0	17,38	557,33	88,17
2019/05/28	CH2205	Male	Three y/o	Treatment (Wk 2)	Treatment	Feed Day	No	20:20	20:00 - 20:59	Night	37,8	111	33,4	33,4	18,95	601,33	117,88

2019/05/28	CH2206	Male	Three y/o	Treatment (Wk 2)	Treatment	Feed Day	No	20:20	20:00 - 20:59	Night	35,6	95	14,2	14,2	16,81	590,14	88,65
2019/05/28	CH2205	Male	Three y/o	Treatment (Wk 2)	Treatment	Feed Day	No	20:25	20:00 - 20:59	Night	37,9		20,6	20,6	19,05		101,32
2019/05/28	CH2206	Male	Three y/o	Treatment (Wk 2)	Treatment	Feed Day	No	20:25	20:00 - 20:59	Night	35,7	99	39,0	39,0	16,90	593,14	123,22
2019/05/28	CH2205	Male	Three y/o	Treatment (Wk 2)	Treatment	Feed Day	No	20:30	20:00 - 20:59	Night	37,9	65	4,6	4,6	19,05	561,12	50,61
2019/05/28	CH2206	Male	Three y/o	Treatment (Wk 2)	Treatment	Feed Day	No	20:30	20:00 - 20:59	Night	36,1	74	31,8	31,8	17,29	571,33	116,20
2019/05/28	CH2205	Male	Three y/o	Treatment (Wk 2)	Treatment	Feed Day	No	20:35	20:00 - 20:59	Night	37,7	86	22,8	22,8	18,85	582,77	104,79
2019/05/28	CH2206	Male	Three y/o	Treatment (Wk 2)	Treatment	Feed Day	No	20:35	20:00 - 20:59	Night	36,3	62	32,2	32,2	17,48	557,33	116,63
2019/05/28	CH2205	Male	Three y/o	Treatment (Wk 2)	Treatment	Feed Day	No	20:40	20:00 - 20:59	Night	37,7	68	5,6	5,6	18,85	564,71	57,21
2019/05/28	CH2206	Male	Three y/o	Treatment (Wk 2)	Treatment	Feed Day	No	20:40	20:00 - 20:59	Night	36,5	58	46,6	46,6	17,67	551,90	129,35
2019/05/28	CH2205	Male	Three y/o	Treatment (Wk 2)	Treatment	Feed Day	No	20:45	20:00 - 20:59	Night	37,7	100	6,4	6,4	18,85	593,87	61,70
2019/05/28	CH2206	Male	Three y/o	Treatment (Wk 2)	Treatment	Feed Day	No	20:45	20:00 - 20:59	Night	36,6	124	37,0	37,0	17,77	609,04	121,40
2019/05/28	CH2205	Male	Three y/o	Treatment (Wk 2)	Treatment	Feed Day	No	20:50	20:00 - 20:59	Night	37,6	67	6,8	6,8	18,75	563,53	63,74
2019/05/28	CH2206	Male	Three y/o	Treatment (Wk 2)	Treatment	Feed Day	No	20:50	20:00 - 20:59	Night	36,9	54	48,2	48,2	18,06	545,99	130,52
2019/05/28	CH2205	Male	Three y/o	Treatment (Wk 2)	Treatment	Feed Day	No	20:55	20:00 - 20:59	Night	37,6	94	10,0	10,0	18,75	589,37	76,76
2019/05/28	CH2206	Male	Three y/o	Treatment (Wk 2)	Treatment	Feed Day	No	20:55	20:00 - 20:59	Night	37,0	61	45,4	45,4	18,16	556,01	128,45
2019/05/28	CH2205	Male	Three y/o	Treatment (Wk 2)	Treatment	Feed Day	No	21:00	21:00 - 21:59	Night	37,5	64	5,6	5,6	18,65	559,88	57,21
2019/05/28	CH2206	Male	Three y/o	Treatment (Wk 2)	Treatment	Feed Day	No	21:00	21:00 - 21:59	Night	37,0	50	39,0	39,0	18,16	539,52	123,22
2019/05/28	CH2205	Male	Three y/o	Treatment (Wk 2)	Treatment	Feed Day	No	21:05	21:00 - 21:59	Night	37,4	106	19,2	19,2	18,55	598,06	98,92
2019/05/28	CH2206	Male	Three y/o	Treatment (Wk 2)	Treatment	Feed Day	No	21:05	21:00 - 21:59	Night	37,2	97	27,6	27,6	18,36	591,66	111,33
2019/05/28	CH2205	Male	Three y/o	Treatment (Wk 2)	Treatment	Feed Day	No	21:10	21:00 - 21:59	Night	37,3	114	13,0	13,0	18,46	603,20	85,65
2019/05/28	CH2206	Male	Three y/o	Treatment (Wk 2)	Treatment	Feed Day	No	21:10	21:00 - 21:59	Night	37,3	92	47,2	47,2	18,46	587,78	129,80
2019/05/28	CH2205	Male	Three y/o	Treatment (Wk 2)	Treatment	Feed Day	No	21:15	21:00 - 21:59	Night	37,4	66	3,8	3,8	18,55	562,34	44,22
2019/05/28	CH2206	Male	Three y/o	Treatment (Wk 2)	Treatment	Feed Day	No	21:15	21:00 - 21:59	Night	37,3	73	46,6	46,6	18,46	570,27	129,35
2019/05/28	CH2205	Male	Three y/o	Treatment (Wk 2)	Treatment	Feed Day	No	21:20	21:00 - 21:59	Night	37,5	96	5,2	5,2	18,65	590,91	54,72
2019/05/28	CH2206	Male	Three y/o	Treatment (Wk 2)	Treatment	Feed Day	No	21:20	21:00 - 21:59	Night	37,3	101	40,8	40,8	18,46	594,59	124,77
2019/05/28	CH2205	Male	Three y/o	Treatment (Wk 2)	Treatment	Feed Day	No	21:25	21:00 - 21:59	Night	37,7	45	9,2	9,2	18,85	530,47	73,94
2019/05/28	CH2206	Male	Three y/o	Treatment (Wk 2)	Treatment	Feed Day	No	21:25	21:00 - 21:59	Night	37,3	44	27,4	27,4	18,46	528,51	111,09
2019/05/28	CH2205	Male	Three y/o	Treatment (Wk 2)	Treatment	Feed Day	No	21:30	21:00 - 21:59	Night	37,7	62	5,2	5,2	18,85	557,33	54,72
2019/05/28	CH2206	Male	Three y/o	Treatment (Wk 2)	Treatment	Feed Day	No	21:30	21:00 - 21:59	Night	37,1	100	12,6	12,6	18,26	593,87	84,59
2019/05/28	CH2205	Male	Three y/o	Treatment (Wk 2)	Treatment	Feed Day	No	21:35	21:00 - 21:59	Night	37,6	51	11,4	11,4	18,75	541,20	81,19
2019/05/28	CH2206	Male	Three y/o	Treatment (Wk 2)	Treatment	Feed Day	No	21:35	21:00 - 21:59	Night	37,1	90	20,0	20,0	18,26	586,16	100,31
2019/05/28	CH2205	Male	Three y/o	Treatment (Wk 2)	Treatment	Feed Day	No	21:40	21:00 - 21:59	Night	37,6	98	30,8	30,8	18,75	592,41	115,10
2019/05/28	CH2206	Male	Three y/o	Treatment (Wk 2)	Treatment	Feed Day	No	21:40	21:00 - 21:59	Night	37,2	143	21,4	21,4	18,36	618,69	102,63
2019/05/28	CH2205	Male	Three y/o	Treatment (Wk 2)	Treatment	Feed Day	No	21:45	21:00 - 21:59	Night	37,6	107	35,6	35,6	18,75	598,73	120,08
2019/05/28	CH2206	Male	Three y/o	Treatment (Wk 2)	Treatment	Feed Day	No	21:45	21:00 - 21:59	Night	37,3	62	30,6	30,6	18,46	557,33	114,87
2019/05/28	CH2205	Male	Three y/o	Treatment (Wk 2)	Treatment	Feed Day	No	21:50	21:00 - 21:59	Night	37,7		23,0	23,0	18,85		105,09
2019/05/28	CH2206	Male	Three y/o	Treatment (Wk 2)	Treatment	Feed Day	No	21:50	21:00 - 21:59	Night	37,5	46	56,2	56,2	18,65	532,38	135,83
2019/05/28	CH2205	Male	Three y/o	Treatment (Wk 2)	Treatment	Feed Day	No	21:55	21:00 - 21:59	Night	37,7		26,4	26,4	18,85		109,81
2019/05/28	CH2206	Male	Three y/o	Treatment (Wk 2)	Treatment	Feed Day	No	21:55	21:00 - 21:59	Night	37,7	58	55,8	55,8	18,85	551,90	135,58
2019/05/28	CH2205	Male	Three y/o	Treatment (Wk 2)	Treatment	Feed Day	No	22:00	22:00 - 22:59	Night	37,7		24,4	24,4	18,85		107,11
2019/05/28	CH2206	Male	Three y/o	Treatment (Wk 2)	Treatment	Feed Day	No	22:00	22:00 - 22:59	Night	37,7	62	56,2	56,2	18,85	557,33	135,83
2019/05/28	CH2205	Male	Three y/o	Treatment (Wk 2)	Treatment	Feed Day	No	22:05	22:00 - 22:59	Night	37,7	129	21,0	21,0	18,85	611,75	101,98
2019/05/28	CH2206	Male	Three y/o	Treatment (Wk 2)	Treatment	Feed Day	No	22:05	22:00 - 22:59	Night	37,8	88	50,4	50,4	18,95	584,49	132,06
2019/05/28	CH2205	Male	Three y/o	Treatment (Wk 2)	Treatment	Feed Day	No	22:10	22:00 - 22:59	Night	37,6	80	25,6	25,6	18,75	577,31	108,76
2019/05/28	CH2206	Male	Three y/o	Treatment (Wk 2)	Treatment	Feed Day	No	22:10	22:00 - 22:59	Night	37,7	185	49,6	49,6	18,85	635,34	131,51
2019/05/28	CH2205	Male	Three y/o	Treatment (Wk 2)	Treatment	Feed Day	No	22:15	22:00 - 22:59	Night	37,6		22,4	22,4	18,75		104,19
2019/05/28	CH2206	Male	Three y/o	Treatment (Wk 2)	Treatment	Feed Day	No	22:15	22:00 - 22:59	Night	37,8	95	51,0	51,0	18,95	590,14	132,47
2019/05/28	CH2205	Male	Three y/o	Treatment (Wk 2)	Treatment	Feed Day	No	22:20	22:00 - 22:59	Night	37,5		20,4	20,4	18,65		100,99
2019/05/28	CH2206	Male	Three y/o	Treatment (Wk 2)	Treatment	Feed Day	No	22:20	22:00 - 22:59	Night	37,8	56	54,0	54,0	18,95	549,01	134,45
2019/05/28	CH2205	Male	Three y/o	Treatment (Wk 2)	Treatment	Feed Day	No	22:25	22:00 - 22:59	Night	37,5	150	22,0	22,0	18,65	621,86	103,57
2019/05/28	CH2206	Male	Three y/o	Treatment (Wk 2)	Treatment	Feed Day	No	22:25	22:00 - 22:59	Night	37,8	68	52,6	52,6	18,95	564,71	133,54
2019/05/28	CH2205	Male	Three y/o	Treatment (Wk 2)	Treatment	Feed Day	No	22:30	22:00 - 22:59	Night	37,5	118	20,0	20,0	18,65	605,61	100,31
2019/05/28	CH2206	Male	Three y/o	Treatment (Wk 2)	Treatment	Feed Day	No	22:30	22:00 - 22:59	Night	37,8	93	55,6	55,6	18,95	588,58	135,46
2019/05/28	CH2205	Male	Three y/o	Treatment (Wk 2)	Treatment	Feed Day	No	22:35	22:00 - 22:59	Night	37,4		21,0	21,0	18,55		101,98
2019/05/28	CH2206	Male	Three y/o	Treatment (Wk 2)	Treatment	Feed Day	No	22:35	22:00 - 22:59	Night	37,8	64	52,4	52,4	18,95	559,88	133,41
2019/05/28	CH2205	Male	Three y/o	Treatment (Wk 2)	Treatment	Feed Day	No	22:40	22:00 - 22:59	Night	37,4		20,0	20,0	18,55		100,31
2019/05/28	CH2206	Male	Three y/o	Treatment (Wk 2)	Treatment	Feed Day	No	22:40	22:00 - 22:59	Night	37,8	86	49,6	49,6	18,95	582,77	131,51
2019/05/28	CH2205	Male	Three y/o	Treatment (Wk 2)	Treatment	Feed Day	No	22:45	22:00 - 22:59	Night	37,4		56,6	56,6	18,55		136,07
2019/05/28	CH2206	Male	Three y/o	Treatment (Wk 2)	Treatment	Feed Day	No	22:45	22:00 - 22:59	Night	37,8	92	43,2	43,2	18,95	587,78	126,74
2019/05/28	CH2205	Male	Three y/o	Treatment (Wk 2)	Treatment	Feed Day	No	22:50	22:00 - 22:59	Night	37,3	46	28,8	28,8	18,46	532,38	112,79
2019/05/28	CH2206	Male	Three y/o	Treatment (Wk 2)	Treatment	Feed Day	No	22:50	22:00 - 22:59	Night	37,8	89	32,6	32,6	18,95	585,33	117,05
2019/05/28	CH2205	Male	Three y/o	Treatment (Wk 2)	Treatment	Feed Day	No	22:55	22:00 - 22:59	Night	37,1		27,4	27,4	18,26		111,09
2019/05/28	CH2206	Male	Three y/o	Treatment (Wk 2)	Treatment	Feed Day	No	22:55	22:00 - 22:59	Night	37,6	94	34,2	34,2	18,75	589,37	118,70
2019/05/28	CH2205	Male	Three y/o	Treatment (Wk 2)	Treatment	Feed Day	No	23:00	23:00 - 23:59	Night	37,1	93	5,4	5,4	18,26	588,58	55,99
2019/05/28	CH2206	Male	Three y/o	Treatment (Wk 2)	Treatment	Feed Day	No	23:00	23:00 - 23:59	Night	37,5	46	32,4	32,4	18,65	532,38	116,84
2019/05/28	CH2205	Male	Three y/o	Treatment (Wk 2)	Treatment	Feed Day	No	23:05	23:00 - 23:59	Night	37,3		4,8	4,8	18,46		52,04
2019/05/28	CH2206	Male	Three y/o	Treatment (Wk 2)	Treatment	Feed Day	No	23:05	23:00 - 23:59	Night	37,4	93	38,2	38,2	18,55	588,58	122,50
2019/05/28	CH2205	Male	Three y/o	Treatment (Wk 2)	Treatment	Feed Day	No	23:10	23:00 - 23:59	Night	37,4	63	4,2	4,2	18,55	558,62	47,57
2019/05/28	CH2206	Male	Three y/o	Treatment (Wk 2)	Treatment	Feed Day	No	23:10	23:00 - 23:59	Night	37,4	76	33,6	33,6	18,55	573,39	118,09
2019/05/28	CH2205	Male	Three y/o	Treatment (Wk 2)	Treatment	Feed Day	No	23:15	23:00 - 23:59	Night	37,5	45	5,2	5,2	18,65	530,47	54,72

	2019/05/28	CH2206	Male	Three y/o	Treatment (Wk 2)	Treatment	Feed Day	No	23:15	23:00 - 23:59	Night	37,4	100	36,4	36,4	18,55	593,87	120,84
	2019/05/28	CH2205	Male	Three y/o	Treatment (Wk 2)	Treatment	Feed Day	No	23:20	23:00 - 23:59	Night	37,6	60	10,8	10,8	18,75	554,67	79,36
	2019/05/28	CH2206	Male	Three y/o	Treatment (Wk 2)	Treatment	Feed Day	No	23:20	23:00 - 23:59	Night	37,4	47	31,8	31,8	18,55	534,23	116,20
	2019/05/28	CH2205	Male	Three y/o	Treatment (Wk 2)	Treatment	Feed Day	No	23:25	23:00 - 23:59	Night	37,6		24,2	24,2	18,75		106,83
	2019/05/28	CH2206	Male	Three y/o	Treatment (Wk 2)	Treatment	Feed Day	No	23:25	23:00 - 23:59	Night	37,5	95	2,4	2,4	18,65	590,14	28,91
	2019/05/28	CH2205	Male	Three y/o	Treatment (Wk 2)	Treatment	Feed Day	No	23:30	23:00 - 23:59	Night	37,4	45	19,2	19,2	18,55	530,47	98,92
	2019/05/28	CH2206	Male	Three y/o	Treatment (Wk 2)	Treatment	Feed Day	No	23:30	23:00 - 23:59	Night	37,5	109	5,8	5,8	18,65	600,04	58,39
	2019/05/28	CH2205	Male	Three y/o	Treatment (Wk 2)	Treatment	Feed Day	No	23:35	23:00 - 23:59	Night	37,4	65	25,0	25,0	18,55	561,12	107,94
	2019/05/28	CH2206	Male	Three y/o	Treatment (Wk 2)	Treatment	Feed Day	No	23:35	23:00 - 23:59	Night	37,4	86	11,4	11,4	18,55	582,77	81,19
	2019/05/28	CH2205	Male	Three y/o	Treatment (Wk 2)	Treatment	Feed Day	No	23:40	23:00 - 23:59	Night	37,6	59	24,8	24,8	18,75	553,30	107,67
	2019/05/28	CH2206	Male	Three y/o	Treatment (Wk 2)	Treatment	Feed Day	No	23:40	23:00 - 23:59	Night	37,5	84	19,2	19,2	18,65	581,01	98,92
	2019/05/28	CH2205	Male	Three y/o	Treatment (Wk 2)	Treatment	Feed Day	No	23:45	23:00 - 23:59	Night	37,6	69	22,8	22,8	18,75	565,86	104,79
	2019/05/28	CH2206	Male	Three y/o	Treatment (Wk 2)	Treatment	Feed Day	No	23:45	23:00 - 23:59	Night	37,7	64	24,2	24,2	18,85	559,88	106,83
	2019/05/28	CH2205	Male	Three y/o	Treatment (Wk 2)	Treatment	Feed Day	No	23:50	23:00 - 23:59	Night	37,6	56	26,8	26,8	18,75	549,01	110,33
	2019/05/28	CH2206	Male	Three y/o	Treatment (Wk 2)	Treatment	Feed Day	No	23:50	23:00 - 23:59	Night	37,7	79	43,2	43,2	18,85	576,36	126,74
	2019/05/28	CH2205	Male	Three y/o	Treatment (Wk 2)	Treatment	Feed Day	No	23:55	23:00 - 23:59	Night	37,6	55	24,8	24,8	18,75	547,52	107,67
	2019/05/28	CH2206	Male	Three y/o	Treatment (Wk 2)	Treatment	Feed Day	No	23:55	23:00 - 23:59	Night	37,8	97	38,4	38,4	18,95	591,66	122,68
15	2019/05/29	CH2205	Male	Three y/o	Treatment (Wk 3)	Treatment	Fast Day	No	00:00	00:00 - 00:59	Early Morning	37,6	48	21,4	21,4	18,75	536,04	102,63
	2019/05/29	CH2206	Male	Three y/o	Treatment (Wk 3)	Treatment	Fast Day	No	00:00	00:00 - 00:59	Early Morning	37,8	73	40,8	40,8	18,95	570,27	124,77
	2019/05/29	CH2205	Male	Three y/o	Treatment (Wk 3)	Treatment	Fast Day	No	00:05	00:00 - 00:59	Early Morning	37,6	71	23,0	23,0	18,75	568,10	105,09
	2019/05/29	CH2206	Male	Three y/o	Treatment (Wk 3)	Treatment	Fast Day	No	00:05	00:00 - 00:59	Early Morning	37,8	71	58,6	58,6	18,95	568,10	137,28
	2019/05/29	CH2205	Male	Three y/o	Treatment (Wk 3)	Treatment	Fast Day	No	00:10	00:00 - 00:59	Early Morning	37,6	65	26,6	26,6	18,75	561,12	110,07
	2019/05/29	CH2206	Male	Three y/o	Treatment (Wk 3)	Treatment	Fast Day	No	00:10	00:00 - 00:59	Early Morning	37,8	47	57,4	57,4	18,95	534,23	136,56
	2019/05/29	CH2205	Male	Three y/o	Treatment (Wk 3)	Treatment	Fast Day	No	00:15	00:00 - 00:59	Early Morning	37,7	78	20,8	20,8	18,85	575,38	101,65
	2019/05/29	CH2206	Male	Three y/o	Treatment (Wk 3)	Treatment	Fast Day	No	00:15	00:00 - 00:59	Early Morning	37,8	53	57,8	57,8	18,95	544,43	136,80
	2019/05/29	CH2205	Male	Three y/o	Treatment (Wk 3)	Treatment	Fast Day	No	00:20	00:00 - 00:59	Early Morning	37,7	54	27,4	27,4	18,85	545,99	111,09
	2019/05/29	CH2206	Male	Three y/o	Treatment (Wk 3)	Treatment	Fast Day	No	00:20	00:00 - 00:59	Early Morning	37,8	86	61,0	61,0	18,95	582,77	138,67
	2019/05/29	CH2205	Male	Three y/o	Treatment (Wk 3)	Treatment	Fast Day	No	00:25	00:00 - 00:59	Early Morning	37,7	44	25,0	25,0	18,85	528,51	107,94
	2019/05/29	CH2206	Male	Three y/o	Treatment (Wk 3)	Treatment	Fast Day	No	00:25	00:00 - 00:59	Early Morning	37,8	76	59,8	59,8	18,95	573,39	137,98
	2019/05/29	CH2205	Male	Three y/o	Treatment (Wk 3)	Treatment	Fast Day	No	00:30	00:00 - 00:59	Early Morning	37,7	51	23,8	23,8	18,85	541,20	106,26
	2019/05/29	CH2206	Male	Three y/o	Treatment (Wk 3)	Treatment	Fast Day	No	00:30	00:00 - 00:59	Early Morning	37,9	59	54,0	54,0	19,05	553,30	134,45
	2019/05/29	CH2205	Male	Three y/o	Treatment (Wk 3)	Treatment	Fast Day	No	00:35	00:00 - 00:59	Early Morning	37,7	44	24,0	24,0	18,85	528,51	106,55
	2019/05/29	CH2206	Male	Three y/o	Treatment (Wk 3)	Treatment	Fast Day	No	00:35	00:00 - 00:59	Early Morning	37,9	50	60,0	60,0	19,05	539,52	138,09
	2019/05/29	CH2205	Male	Three y/o	Treatment (Wk 3)	Treatment	Fast Day	No	00:40	00:00 - 00:59	Early Morning	37,7	57	26,8	26,8	18,85	550,47	110,33
	2019/05/29	CH2206	Male	Three y/o	Treatment (Wk 3)	Treatment	Fast Day	No	00:40	00:00 - 00:59	Early Morning	37,8	86	50,2	50,2	18,95	582,77	131,92
	2019/05/29	CH2205	Male	Three y/o	Treatment (Wk 3)	Treatment	Fast Day	No	00:45	00:00 - 00:59	Early Morning	37,9		17,0	17,0	19,05		94,77
	2019/05/29	CH2206	Male	Three y/o	Treatment (Wk 3)	Treatment	Fast Day	No	00:45	00:00 - 00:59	Early Morning	37,8		23,2	23,2	18,95		105,39
	2019/05/29	CH2205	Male	Three y/o	Treatment (Wk 3)	Treatment	Fast Day	No	00:50	00:00 - 00:59	Early Morning	37,9	59	5,4	5,4	19,05	553,30	55,99
	2019/05/29	CH2206	Male	Three y/o	Treatment (Wk 3)	Treatment	Fast Day	No	00:50	00:00 - 00:59	Early Morning	37,8	90	20,4	20,4	18,95	586,16	100,99
	2019/05/29	CH2205	Male	Three y/o	Treatment (Wk 3)	Treatment	Fast Day	No	00:55	00:00 - 00:59	Early Morning	37,9	62	6,0	6,0	19,05	557,33	59,53
	2019/05/29	CH2206	Male	Three y/o	Treatment (Wk 3)	Treatment	Fast Day	No	00:55	00:00 - 00:59	Early Morning	37,8	86	21,0	21,0	18,95	582,77	101,98
	2019/05/29	CH2205	Male	Three y/o	Treatment (Wk 3)	Treatment	Fast Day	No	01:00	01:00 - 01:59	Early Morning	37,9	57	10,6	10,6	19,05	550,47	78,73
	2019/05/29	CH2206	Male	Three y/o	Treatment (Wk 3)	Treatment	Fast Day	No	01:00	01:00 - 01:59	Early Morning	37,8	45	6,8	6,8	18,95	530,47	63,74
	2019/05/29	CH2205	Male	Three y/o	Treatment (Wk 3)	Treatment	Fast Day	No	01:05	01:00 - 01:59	Early Morning	37,9	90	15,2	15,2	19,05	586,16	90,96
	2019/05/29	CH2206	Male	Three y/o	Treatment (Wk 3)	Treatment	Fast Day	No	01:05	01:00 - 01:59	Early Morning	37,8	85	11,4	11,4	18,95	581,90	81,19
	2019/05/29	CH2205	Male	Three y/o	Treatment (Wk 3)	Treatment	Fast Day	No	01:10	01:00 - 01:59	Early Morning	37,9	46	12,8	12,8	19,05	532,38	85,12
	2019/05/29	CH2206	Male	Three y/o	Treatment (Wk 3)	Treatment	Fast Day	No	01:10	01:00 - 01:59	Early Morning	37,8	62	12,0	12,0	18,95	557,33	82,93
	2019/05/29	CH2205	Male	Three y/o	Treatment (Wk 3)	Treatment	Fast Day	No	01:15	01:00 - 01:59	Early Morning	37,9	72	22,8	22,8	19,05	569,20	104,79
	2019/05/29	CH2206	Male	Three y/o	Treatment (Wk 3)	Treatment	Fast Day	No	01:15	01:00 - 01:59	Early Morning	37,8	56	9,6	9,6	18,95	549,01	75,37
	2019/05/29	CH2205	Male	Three y/o	Treatment (Wk 3)	Treatment	Fast Day	No	01:20	01:00 - 01:59	Early Morning	37,7	55	18,8	18,8	18,85	547,52	98,20
	2019/05/29	CH2206	Male	Three y/o	Treatment (Wk 3)	Treatment	Fast Day	No	01:20	01:00 - 01:59	Early Morning	37,8	42	11,0	11,0	18,95	524,42	79,98
	2019/05/29	CH2205	Male	Three y/o	Treatment (Wk 3)	Treatment	Fast Day	No	01:25	01:00 - 01:59	Early Morning	37,7	55	20,2	20,2	18,85	547,52	100,65
	2019/05/29	CH2206	Male	Three y/o	Treatment (Wk 3)	Treatment	Fast Day	No	01:25	01:00 - 01:59	Early Morning	37,8	61	6,4	6,4	18,95	556,01	61,70
	2019/05/29	CH2205	Male	Three y/o	Treatment (Wk 3)	Treatment	Fast Day	No	01:30	01:00 - 01:59	Early Morning	37,6	51	20,8	20,8	18,75	541,20	101,65
	2019/05/29	CH2206	Male	Three y/o	Treatment (Wk 3)	Treatment	Fast Day	No	01:30	01:00 - 01:59	Early Morning	37,8	78	9,6	9,6	18,95	575,38	75,37
	2019/05/29	CH2205	Male	Three y/o	Treatment (Wk 3)	Treatment	Fast Day	No	01:35	01:00 - 01:59	Early Morning	37,6	82	18,0	18,0	18,75	579,19	96,72
	2019/05/29	CH2206	Male	Three y/o	Treatment (Wk 3)	Treatment	Fast Day	No	01:35	01:00 - 01:59	Early Morning	37,8	45	3,8	3,8	18,95	530,47	44,22
	2019/05/29	CH2205	Male	Three y/o	Treatment (Wk 3)	Treatment	Fast Day	No	01:40	01:00 - 01:59	Early Morning	37,6	53	15,8	15,8	18,75	544,43	92,28
	2019/05/29	CH2206	Male	Three y/o	Treatment (Wk 3)	Treatment	Fast Day	No	01:40	01:00 - 01:59	Early Morning	37,8	50	5,4	5,4	18,95	539,52	55,99
	2019/05/29	CH2205	Male	Three y/o	Treatment (Wk 3)	Treatment	Fast Day	No	01:45	01:00 - 01:59	Early Morning	37,7	62	11,6	11,6	18,85	557,33	81,78
	2019/05/29	CH2206	Male	Three y/o	Treatment (Wk 3)	Treatment	Fast Day	No	01:45	01:00 - 01:59	Early Morning	37,8	64	7,2	7,2	18,95	559,88	65,66
	2019/05/29	CH2205	Male	Three y/o	Treatment (Wk 3)	Treatment	Fast Day	No	01:50	01:00 - 01:59	Early Morning	37,7	94	13,8	13,8	18,85	589,37	87,68
	2019/05/29	CH2206	Male	Three y/o	Treatment (Wk 3)	Treatment	Fast Day	No	01:50	01:00 - 01:59	Early Morning	37,8	82	11,8	11,8	18,95	579,19	82,36
	2019/05/29	CH2205	Male	Three y/o	Treatment (Wk 3)	Treatment	Fast Day	No	01:55	01:00 - 01:59	Early Morning	37,7	52	18,0	18,0	18,85	542,83	96,72
	2019/05/29	CH2206	Male	Three y/o	Treatment (Wk 3)	Treatment	Fast Day	No	01:55	01:00 - 01:59	Early Morning	37,9	73	16,4	16,4	19,05	570,27	93,55
	2019/05/29	CH2205	Male	Three y/o	Treatment (Wk 3)	Treatment	Fast Day	No	02:00	02:00 - 02:59	Early Morning	37,7	61	14,4	14,4	18,85	556,01	89,12
	2019/05/29	CH2206	Male	Three y/o	Treatment (Wk 3)	Treatment	Fast Day	No	02:00	02:00 - 02:59	Early Morning	37,9	72	15,2	15,2	19,05	569,20	90,96
	2019/05/29	CH2205	Male	Three y/o	Treatment (Wk 3)	Treatment	Fast Day	No	02:05	02:00 - 02:59	Early Morning	37,7	53	13,2	13,2	18,85	544,43	86,17
	2019/05/29	CH2206	Male	Three y/o	Treatment (Wk 3)	Treatment	Fast Day	No	02:05	02:00 - 02:59	Early Morning	37,9	62	17,4	17,4	19,05	557,33	95,56
	2019/05/29	CH2205	Male	Three y/o	Treatment (Wk 3)	Treatment	Fast Day	No	02:10	02:00 - 02:59	Early Morning	37,7	60	7,0	7,0	18,85	554,67	64,72

2019/05/29	CH2206	Male	Three y/o	Treatment (Wk 3)	Treatment	Fast Day	No	02:10	02:00 - 02:59	Early Morning	37,9	44	15,4	15,4	19,05	528,51	91,41
2019/05/29	CH2205	Male	Three y/o	Treatment (Wk 3)	Treatment	Fast Day	No	02:15	02:00 - 02:59	Early Morning	37,7	96	7,0	7,0	18,85	590,91	64,72
2019/05/29	CH2206	Male	Three y/o	Treatment (Wk 3)	Treatment	Fast Day	No	02:15	02:00 - 02:59	Early Morning	37,9	42	19,8	19,8	19,05	524,42	99,97
2019/05/29	CH2205	Male	Three y/o	Treatment (Wk 3)	Treatment	Fast Day	No	02:20	02:00 - 02:59	Early Morning	37,7	99	14,0	14,0	18,85	593,14	88,17
2019/05/29	CH2206	Male	Three y/o	Treatment (Wk 3)	Treatment	Fast Day	No	02:20	02:00 - 02:59	Early Morning	37,9	86	26,2	26,2	19,05	582,77	109,55
2019/05/29	CH2205	Male	Three y/o	Treatment (Wk 3)	Treatment	Fast Day	No	02:25	02:00 - 02:59	Early Morning	37,8	53	16,8	16,8	18,95	544,43	94,37
2019/05/29	CH2206	Male	Three y/o	Treatment (Wk 3)	Treatment	Fast Day	No	02:25	02:00 - 02:59	Early Morning	37,9	89	21,8	21,8	19,05	585,33	103,26
2019/05/29	CH2205	Male	Three y/o	Treatment (Wk 3)	Treatment	Fast Day	No	02:30	02:00 - 02:59	Early Morning	37,7	48	18,4	18,4	18,85	536,04	97,47
2019/05/29	CH2206	Male	Three y/o	Treatment (Wk 3)	Treatment	Fast Day	No	02:30	02:00 - 02:59	Early Morning	37,9	108	18,6	18,6	19,05	599,39	97,84
2019/05/29	CH2205	Male	Three y/o	Treatment (Wk 3)	Treatment	Fast Day	No	02:35	02:00 - 02:59	Early Morning	37,7	60	18,0	18,0	18,85	554,67	96,72
2019/05/29	CH2206	Male	Three y/o	Treatment (Wk 3)	Treatment	Fast Day	No	02:35	02:00 - 02:59	Early Morning	37,9	52	16,4	16,4	19,05	542,83	93,55
2019/05/29	CH2205	Male	Three y/o	Treatment (Wk 3)	Treatment	Fast Day	No	02:40	02:00 - 02:59	Early Morning	37,7	80	14,0	14,0	18,85	577,31	88,17
2019/05/29	CH2206	Male	Three y/o	Treatment (Wk 3)	Treatment	Fast Day	No	02:40	02:00 - 02:59	Early Morning	37,9		65,2	65,2	19,05		140,97
2019/05/29	CH2205	Male	Three y/o	Treatment (Wk 3)	Treatment	Fast Day	No	02:45	02:00 - 02:59	Early Morning	37,7	97	12,6	12,6	18,85	591,66	84,59
2019/05/29	CH2206	Male	Three y/o	Treatment (Wk 3)	Treatment	Fast Day	No	02:45	02:00 - 02:59	Early Morning	37,8	49	68,0	68,0	18,95	537,80	142,43
2019/05/29	CH2205	Male	Three y/o	Treatment (Wk 3)	Treatment	Fast Day	No	02:50	02:00 - 02:59	Early Morning	37,6	88	15,6	15,6	18,75	584,49	91,85
2019/05/29	CH2206	Male	Three y/o	Treatment (Wk 3)	Treatment	Fast Day	No	02:50	02:00 - 02:59	Early Morning	37,8	80	67,2	67,2	18,95	577,31	142,02
2019/05/29	CH2205	Male	Three y/o	Treatment (Wk 3)	Treatment	Fast Day	No	02:55	02:00 - 02:59	Early Morning	37,6	65	10,0	10,0	18,75	561,12	76,76
2019/05/29	CH2206	Male	Three y/o	Treatment (Wk 3)	Treatment	Fast Day	No	02:55	02:00 - 02:59	Early Morning	37,8	50	66,0	66,0	18,95	539,52	141,40
2019/05/29	CH2205	Male	Three y/o	Treatment (Wk 3)	Treatment	Fast Day	No	03:00	03:00 - 03:59	Early Morning	37,6	63	17,6	17,6	18,75	558,62	95,95
2019/05/29	CH2206	Male	Three y/o	Treatment (Wk 3)	Treatment	Fast Day	No	03:00	03:00 - 03:59	Early Morning	37,8	51	65,4	65,4	18,95	541,20	141,08
2019/05/29	CH2205	Male	Three y/o	Treatment (Wk 3)	Treatment	Fast Day	No	03:05	03:00 - 03:59	Early Morning	37,7	42	14,2	14,2	18,85	524,42	88,65
2019/05/29	CH2206	Male	Three y/o	Treatment (Wk 3)	Treatment	Fast Day	No	03:05	03:00 - 03:59	Early Morning	37,8	86	68,2	68,2	18,95	582,77	142,53
2019/05/29	CH2205	Male	Three y/o	Treatment (Wk 3)	Treatment	Fast Day	No	03:10	03:00 - 03:59	Early Morning	37,6	63	14,0	14,0	18,75	558,62	88,17
2019/05/29	CH2206	Male	Three y/o	Treatment (Wk 3)	Treatment	Fast Day	No	03:10	03:00 - 03:59	Early Morning	37,8	47	65,2	65,2	18,95	534,23	140,97
2019/05/29	CH2205	Male	Three y/o	Treatment (Wk 3)	Treatment	Fast Day	No	03:15	03:00 - 03:59	Early Morning	37,6		28,6	28,6	18,75		112,56
2019/05/29	CH2206	Male	Three y/o	Treatment (Wk 3)	Treatment	Fast Day	No	03:15	03:00 - 03:59	Early Morning	37,8	129	19,8	19,8	18,95	611,75	99,97
2019/05/29	CH2205	Male	Three y/o	Treatment (Wk 3)	Treatment	Fast Day	No	03:20	03:00 - 03:59	Early Morning	37,5	115	27,8	27,8	18,65	603,82	111,58
2019/05/29	CH2206	Male	Three y/o	Treatment (Wk 3)	Treatment	Fast Day	No	03:20	03:00 - 03:59	Early Morning	37,7	89	11,6	11,6	18,85	585,33	81,78
2019/05/29	CH2205	Male	Three y/o	Treatment (Wk 3)	Treatment	Fast Day	No	03:25	03:00 - 03:59	Early Morning	37,5	72	24,0	24,0	18,65	569,20	106,55
2019/05/29	CH2206	Male	Three y/o	Treatment (Wk 3)	Treatment	Fast Day	No	03:25	03:00 - 03:59	Early Morning	37,7	49	11,2	11,2	18,85	537,80	80,59
2019/05/29	CH2205	Male	Three y/o	Treatment (Wk 3)	Treatment	Fast Day	No	03:30	03:00 - 03:59	Early Morning	37,6	60	51,4	51,4	18,75	554,67	132,74
2019/05/29	CH2206	Male	Three y/o	Treatment (Wk 3)	Treatment	Fast Day	No	03:30	03:00 - 03:59	Early Morning	37,7	82	17,8	17,8	18,85	579,19	96,34
2019/05/29	CH2205	Male	Three y/o	Treatment (Wk 3)	Treatment	Fast Day	No	03:35	03:00 - 03:59	Early Morning	37,5	49	31,0	31,0	18,65	537,80	115,32
2019/05/29	CH2206	Male	Three y/o	Treatment (Wk 3)	Treatment	Fast Day	No	03:35	03:00 - 03:59	Early Morning	37,5	63	28,6	28,6	18,65	558,62	112,56
2019/05/29	CH2205	Male	Three y/o	Treatment (Wk 3)	Treatment	Fast Day	No	03:40	03:00 - 03:59	Early Morning	37,5	59	28,6	28,6	18,65	553,30	112,56
2019/05/29	CH2206	Male	Three y/o	Treatment (Wk 3)	Treatment	Fast Day	No	03:40	03:00 - 03:59	Early Morning	37,5	51	30,2	30,2	18,65	541,20	114,42
2019/05/29	CH2205	Male	Three y/o	Treatment (Wk 3)	Treatment	Fast Day	No	03:45	03:00 - 03:59	Early Morning	37,6	82	22,2	22,2	18,75	579,19	103,88
2019/05/29	CH2206	Male	Three y/o	Treatment (Wk 3)	Treatment	Fast Day	No	03:45	03:00 - 03:59	Early Morning	37,4	54	14,4	14,4	18,55	545,99	89,12
2019/05/29	CH2205	Male	Three y/o	Treatment (Wk 3)	Treatment	Fast Day	No	03:50	03:00 - 03:59	Early Morning	37,6	79	22,2	22,2	18,75	576,36	103,88
2019/05/29	CH2206	Male	Three y/o	Treatment (Wk 3)	Treatment	Fast Day	No	03:50	03:00 - 03:59	Early Morning	37,4	80	11,0	11,0	18,55	577,31	79,98
2019/05/29	CH2205	Male	Three y/o	Treatment (Wk 3)	Treatment	Fast Day	No	03:55	03:00 - 03:59	Early Morning	37,6	44	17,2	17,2	18,75	528,51	95,17
2019/05/29	CH2206	Male	Three y/o	Treatment (Wk 3)	Treatment	Fast Day	No	03:55	03:00 - 03:59	Early Morning	37,4	56	15,4	15,4	18,55	549,01	91,41
2019/05/29	CH2205	Male	Three y/o	Treatment (Wk 3)	Treatment	Fast Day	No	04:00	04:00 - 04:59	Morning	37,6	50	18,2	18,2	18,75	539,52	97,10
2019/05/29	CH2206	Male	Three y/o	Treatment (Wk 3)	Treatment	Fast Day	No	04:00	04:00 - 04:59	Morning	37,4	88	11,8	11,8	18,55	584,49	82,36
2019/05/29	CH2205	Male	Three y/o	Treatment (Wk 3)	Treatment	Fast Day	No	04:05	04:00 - 04:59	Morning	37,6	83	18,8	18,8	18,75	580,11	98,20
2019/05/29	CH2206	Male	Three y/o	Treatment (Wk 3)	Treatment	Fast Day	No	04:05	04:00 - 04:59	Morning	37,4	50	23,8	23,8	18,55	539,52	106,26
2019/05/29	CH2205	Male	Three y/o	Treatment (Wk 3)	Treatment	Fast Day	No	04:10	04:00 - 04:59	Morning	37,6	41	17,6	17,6	18,75	522,29	95,95
2019/05/29	CH2206	Male	Three y/o	Treatment (Wk 3)	Treatment	Fast Day	No	04:10	04:00 - 04:59	Morning	37,5	54	20,4	20,4	18,65	545,99	100,99
2019/05/29	CH2205	Male	Three y/o	Treatment (Wk 3)	Treatment	Fast Day	No	04:15	04:00 - 04:59	Morning	37,6	41	19,8	19,8	18,75	522,29	99,97
2019/05/29	CH2206	Male	Three y/o	Treatment (Wk 3)	Treatment	Fast Day	No	04:15	04:00 - 04:59	Morning	37,4	44	23,4	23,4	18,55	528,51	105,68
2019/05/29	CH2205	Male	Three y/o	Treatment (Wk 3)	Treatment	Fast Day	No	04:20	04:00 - 04:59	Morning	37,6	53	18,0	18,0	18,75	544,43	96,72
2019/05/29	CH2206	Male	Three y/o	Treatment (Wk 3)	Treatment	Fast Day	No	04:20	04:00 - 04:59	Morning	37,4	52	26,6	26,6	18,55	542,83	110,07
2019/05/29	CH2205	Male	Three y/o	Treatment (Wk 3)	Treatment	Fast Day	No	04:25	04:00 - 04:59	Morning	37,6	46	21,8	21,8	18,75	532,38	103,26
2019/05/29	CH2206	Male	Three y/o	Treatment (Wk 3)	Treatment	Fast Day	No	04:25	04:00 - 04:59	Morning	37,4	45	14,8	14,8	18,55	530,47	90,05
2019/05/29	CH2205	Male	Three y/o	Treatment (Wk 3)	Treatment	Fast Day	No	04:30	04:00 - 04:59	Morning	37,6	48	19,4	19,4	18,75	536,04	99,28
2019/05/29	CH2206	Male	Three y/o	Treatment (Wk 3)	Treatment	Fast Day	No	04:30	04:00 - 04:59	Morning	37,5	119	59,0	59,0	18,65	606,20	137,51
2019/05/29	CH2205	Male	Three y/o	Treatment (Wk 3)	Treatment	Fast Day	No	04:35	04:00 - 04:59	Morning	37,6	135	21,4	21,4	18,75	614,84	102,63
2019/05/29	CH2206	Male	Three y/o	Treatment (Wk 3)	Treatment	Fast Day	No	04:35	04:00 - 04:59	Morning	37,5	143	52,0	52,0	18,65	618,69	133,14
2019/05/29	CH2205	Male	Three y/o	Treatment (Wk 3)	Treatment	Fast Day	No	04:40	04:00 - 04:59	Morning	37,4	44	36,0	36,0	18,55	528,51	120,46
2019/05/29	CH2206	Male	Three y/o	Treatment (Wk 3)	Treatment	Fast Day	No	04:40	04:00 - 04:59	Morning	37,5	138	40,8	40,8	18,65	616,31	124,77
2019/05/29	CH2205	Male	Three y/o	Treatment (Wk 3)	Treatment	Fast Day	No	04:45	04:00 - 04:59	Morning	37,4	138	42,4	42,4	18,55	616,31	126,10
2019/05/29	CH2206	Male	Three y/o	Treatment (Wk 3)	Treatment	Fast Day	No	04:45	04:00 - 04:59	Morning	37,3	103	57,6	57,6	18,46	596,00	136,68
2019/05/29	CH2205	Male	Three y/o	Treatment (Wk 3)	Treatment	Fast Day	No	04:50	04:00 - 04:59	Morning	37,3	104	23,2	23,2	18,46	596,69	105,39
2019/05/29	CH2206	Male	Three y/o	Treatment (Wk 3)	Treatment	Fast Day	No	04:50	04:00 - 04:59	Morning	37,3	156	58,4	58,4	18,46	624,43	137,16
2019/05/29	CH2205	Male	Three y/o	Treatment (Wk 3)	Treatment	Fast Day	No	04:55	04:00 - 04:59	Morning	37,4	135	69,2	69,2	18,55	614,84	143,04
2019/05/29	CH2206	Male	Three y/o	Treatment (Wk 3)	Treatment	Fast Day	No	04:55	04:00 - 04:59	Morning	37,3	135	90,2	90,2	18,46	614,84	152,25
2019/05/29	CH2205	Male	Three y/o	Treatment (Wk 3)	Treatment	Fast Day	No	05:00	05:00 - 05:59	Morning	37,4	61	32,0	32,0	18,55	556,01	116,41
2019/05/29	CH2206	Male	Three y/o	Treatment (Wk 3)	Treatment	Fast Day	No	05:00	05:00 - 05:59	Morning	37,3	128	35,0	35,0	18,46	611,22	119,49
2019/05/29	CH2205	Male	Three y/o	Treatment (Wk 3)	Treatment	Fast Day	No	05:05	05:00 - 05:59	Morning	37,2	63	189,8	189,8	18,36	558,62	178,28

2019/05/29	CH2206	Male	Three y/o	Treatment (Wk 3)	Treatment	Fast Day	No	05:05	05:00 - 05:59	Morning	37,3		93,4	93,4	18,46		153,46
2019/05/29	CH2205	Male	Three y/o	Treatment (Wk 3)	Treatment	Fast Day	No	05:10	05:00 - 05:59	Morning	37,3	147	70,6	70,6	18,46	620,52	143,73
2019/05/29	CH2206	Male	Three y/o	Treatment (Wk 3)	Treatment	Fast Day	No	05:10	05:00 - 05:59	Morning	37,2	131	68,8	68,8	18,36	612,80	142,84
2019/05/29	CH2205	Male	Three y/o	Treatment (Wk 3)	Treatment	Fast Day	No	05:15	05:00 - 05:59	Morning	37,3	188	14,4	14,4	18,46	636,34	89,12
2019/05/29	CH2206	Male	Three y/o	Treatment (Wk 3)	Treatment	Fast Day	No	05:15	05:00 - 05:59	Morning	37,0	84	59,4	59,4	18,16	581,01	137,75
2019/05/29	CH2205	Male	Three y/o	Treatment (Wk 3)	Treatment	Fast Day	No	05:20	05:00 - 05:59	Morning	37,2	77	25,8	25,8	18,36	574,39	109,02
2019/05/29	CH2206	Male	Three y/o	Treatment (Wk 3)	Treatment	Fast Day	No	05:20	05:00 - 05:59	Morning	36,6	50	59,0	59,0	17,77	539,52	137,51
2019/05/29	CH2205	Male	Three y/o	Treatment (Wk 3)	Treatment	Fast Day	No	05:25	05:00 - 05:59	Morning	36,8	63	20,6	20,6	17,96	558,62	101,32
2019/05/29	CH2206	Male	Three y/o	Treatment (Wk 3)	Treatment	Fast Day	No	05:25	05:00 - 05:59	Morning	36,5	87	55,0	55,0	17,67	583,64	135,08
2019/05/29	CH2205	Male	Three y/o	Treatment (Wk 3)	Treatment	Fast Day	No	05:30	05:00 - 05:59	Morning	36,6	44	21,4	21,4	17,77	528,51	102,63
2019/05/29	CH2206	Male	Three y/o	Treatment (Wk 3)	Treatment	Fast Day	No	05:30	05:00 - 05:59	Morning	36,5	45	50,8	50,8	17,67	530,47	132,33
2019/05/29	CH2205	Male	Three y/o	Treatment (Wk 3)	Treatment	Fast Day	No	05:35	05:00 - 05:59	Morning	36,5	46	18,0	18,0	17,67	532,38	96,72
2019/05/29	CH2206	Male	Three y/o	Treatment (Wk 3)	Treatment	Fast Day	No	05:35	05:00 - 05:59	Morning	36,8	63	48,8	48,8	17,96	558,62	130,95
2019/05/29	CH2205	Male	Three y/o	Treatment (Wk 3)	Treatment	Fast Day	No	05:40	05:00 - 05:59	Morning	36,5	46	17,0	17,0	17,67	532,38	94,77
2019/05/29	CH2206	Male	Three y/o	Treatment (Wk 3)	Treatment	Fast Day	No	05:40	05:00 - 05:59	Morning	37,0	47	50,4	50,4	18,16	534,23	132,06
2019/05/29	CH2205	Male	Three y/o	Treatment (Wk 3)	Treatment	Fast Day	No	05:45	05:00 - 05:59	Morning	36,5	58	17,4	17,4	17,67	551,90	95,56
2019/05/29	CH2206	Male	Three y/o	Treatment (Wk 3)	Treatment	Fast Day	No	05:45	05:00 - 05:59	Morning	37,0	74	65,8	65,8	18,16	571,33	141,29
2019/05/29	CH2205	Male	Three y/o	Treatment (Wk 3)	Treatment	Fast Day	No	05:50	05:00 - 05:59	Morning	36,5	78	20,4	20,4	17,67	575,38	100,99
2019/05/29	CH2206	Male	Three y/o	Treatment (Wk 3)	Treatment	Fast Day	No	05:50	05:00 - 05:59	Morning	36,9	82	65,6	65,6	18,06	579,19	141,19
2019/05/29	CH2205	Male	Three y/o	Treatment (Wk 3)	Treatment	Fast Day	No	05:55	05:00 - 05:59	Morning	36,5	49	16,2	16,2	17,67	537,80	93,13
2019/05/29	CH2206	Male	Three y/o	Treatment (Wk 3)	Treatment	Fast Day	No	05:55	05:00 - 05:59	Morning	36,8	94	62,6	62,6	17,96	589,37	139,56
2019/05/29	CH2205	Male	Three y/o	Treatment (Wk 3)	Treatment	Fast Day	No	06:00	06:00 - 06:59	Morning	36,5	44	15,0	15,0	17,67	528,51	90,51
2019/05/29	CH2206	Male	Three y/o	Treatment (Wk 3)	Treatment	Fast Day	No	06:00	06:00 - 06:59	Morning	36,6	83	65,0	65,0	17,77	580,11	140,87
2019/05/29	CH2205	Male	Three y/o	Treatment (Wk 3)	Treatment	Fast Day	No	06:05	06:00 - 06:59	Morning	36,5	46	16,4	16,4	17,67	532,38	93,55
2019/05/29	CH2206	Male	Three y/o	Treatment (Wk 3)	Treatment	Fast Day	No	06:05	06:00 - 06:59	Morning	36,6	49	57,6	57,6	17,77	537,80	136,68
2019/05/29	CH2205	Male	Three y/o	Treatment (Wk 3)	Treatment	Fast Day	No	06:10	06:00 - 06:59	Morning	36,5	58	12,2	12,2	17,67	551,90	83,49
2019/05/29	CH2206	Male	Three y/o	Treatment (Wk 3)	Treatment	Fast Day	No	06:10	06:00 - 06:59	Morning	36,6	55	64,4	64,4	17,77	547,52	140,55
2019/05/29	CH2205	Male	Three y/o	Treatment (Wk 3)	Treatment	Fast Day	No	06:15	06:00 - 06:59	Morning	36,5	39	11,0	11,0	17,67	517,82	79,98
2019/05/29	CH2206	Male	Three y/o	Treatment (Wk 3)	Treatment	Fast Day	No	06:15	06:00 - 06:59	Morning	36,6	85	62,6	62,6	17,77	581,90	139,56
2019/05/29	CH2205	Male	Three y/o	Treatment (Wk 3)	Treatment	Fast Day	No	06:20	06:00 - 06:59	Morning	36,6	51	16,0	16,0	17,77	541,20	92,71
2019/05/29	CH2206	Male	Three y/o	Treatment (Wk 3)	Treatment	Fast Day	No	06:20	06:00 - 06:59	Morning	36,6	38	65,4	65,4	17,77	515,48	141,08
2019/05/29	CH2205	Male	Three y/o	Treatment (Wk 3)	Treatment	Fast Day	No	06:25	06:00 - 06:59	Morning	36,6	44	13,4	13,4	17,77	528,51	86,68
2019/05/29	CH2206	Male	Three y/o	Treatment (Wk 3)	Treatment	Fast Day	No	06:25	06:00 - 06:59	Morning	36,6	67	67,0	67,0	17,77	563,53	141,92
2019/05/29	CH2205	Male	Three y/o	Treatment (Wk 3)	Treatment	Fast Day	No	06:30	06:00 - 06:59	Morning	36,7	80	16,8	16,8	17,87	577,31	94,37
2019/05/29	CH2206	Male	Three y/o	Treatment (Wk 3)	Treatment	Fast Day	No	06:30	06:00 - 06:59	Morning	36,6	82	54,6	54,6	17,77	579,19	134,83
2019/05/29	CH2205	Male	Three y/o	Treatment (Wk 3)	Treatment	Fast Day	No	06:35	06:00 - 06:59	Morning	36,7	47	16,6	16,6	17,87	534,23	93,96
2019/05/29	CH2206	Male	Three y/o	Treatment (Wk 3)	Treatment	Fast Day	No	06:35	06:00 - 06:59	Morning	36,8	52	53,6	53,6	17,96	542,83	134,19
2019/05/29	CH2205	Male	Three y/o	Treatment (Wk 3)	Treatment	Fast Day	No	06:40	06:00 - 06:59	Morning	36,6	44	16,2	16,2	17,77	528,51	93,13
2019/05/29	CH2206	Male	Three y/o	Treatment (Wk 3)	Treatment	Fast Day	No	06:40	06:00 - 06:59	Morning	36,8	42	60,6	60,6	17,96	524,42	138,44
2019/05/29	CH2205	Male	Three y/o	Treatment (Wk 3)	Treatment	Fast Day	No	06:45	06:00 - 06:59	Morning	36,5	50	34,4	34,4	17,67	539,52	118,90
2019/05/29	CH2206	Male	Three y/o	Treatment (Wk 3)	Treatment	Fast Day	No	06:45	06:00 - 06:59	Morning	36,7	50	62,4	62,4	17,87	539,52	139,45
2019/05/29	CH2205	Male	Three y/o	Treatment (Wk 3)	Treatment	Fast Day	No	06:50	06:00 - 06:59	Morning	36,8	129	151,2	151,2	17,96	611,75	170,30
2019/05/29	CH2206	Male	Three y/o	Treatment (Wk 3)	Treatment	Fast Day	No	06:50	06:00 - 06:59	Morning	36,9		76,4	76,4	18,06		146,48
2019/05/29	CH2205	Male	Three y/o	Treatment (Wk 3)	Treatment	Fast Day	No	06:55	06:00 - 06:59	Morning	37,1	132	193,8	193,8	18,26	613,32	179,01
2019/05/29	CH2206	Male	Three y/o	Treatment (Wk 3)	Treatment	Fast Day	No	06:55	06:00 - 06:59	Morning	37,0	123	99,0	99,0	18,16	608,49	155,49
2019/05/29	CH2205	Male	Three y/o	Treatment (Wk 3)	Treatment	Fast Day	No	07:00	07:00 - 07:59	Morning	37,1	135	60,6	60,6	18,26	614,84	138,44
2019/05/29	CH2206	Male	Three y/o	Treatment (Wk 3)	Treatment	Fast Day	No	07:00	07:00 - 07:59	Morning	37,1		112,0	112,0	18,26		159,80
2019/05/29	CH2205	Male	Three y/o	Treatment (Wk 3)	Treatment	Fast Day	No	07:05	07:00 - 07:59	Morning	37,1	104	101,4	101,4	18,26	596,69	156,33
2019/05/29	CH2206	Male	Three y/o	Treatment (Wk 3)	Treatment	Fast Day	No	07:05	07:00 - 07:59	Morning	37,2		202,6	202,6	18,36		180,57
2019/05/29	CH2205	Male	Three y/o	Treatment (Wk 3)	Treatment	Fast Day	No	07:10	07:00 - 07:59	Morning	37,2		272,2	272,2	18,36		190,98
2019/05/29	CH2206	Male	Three y/o	Treatment (Wk 3)	Treatment	Fast Day	No	07:10	07:00 - 07:59	Morning	37,3	114	94,2	94,2	18,46	603,20	153,76
2019/05/29	CH2205	Male	Three y/o	Treatment (Wk 3)	Treatment	Fast Day	No	07:15	07:00 - 07:59	Morning	37,3	57	172,2	172,2	18,46	550,47	174,86
2019/05/29	CH2206	Male	Three y/o	Treatment (Wk 3)	Treatment	Fast Day	No	07:15	07:00 - 07:59	Morning	37,3	154	133,2	133,2	18,46	623,58	165,86
2019/05/29	CH2205	Male	Three y/o	Treatment (Wk 3)	Treatment	Fast Day	No	07:20	07:00 - 07:59	Morning	37,4		121,6	121,6	18,55		162,67
2019/05/29	CH2206	Male	Three y/o	Treatment (Wk 3)	Treatment	Fast Day	No	07:20	07:00 - 07:59	Morning	37,5	60	223,8	223,8	18,65	554,67	184,08
2019/05/29	CH2205	Male	Three y/o	Treatment (Wk 3)	Treatment	Fast Day	No	07:25	07:00 - 07:59	Morning	37,4	111	73,4	73,4	18,55	601,33	145,08
2019/05/29	CH2206	Male	Three y/o	Treatment (Wk 3)	Treatment	Fast Day	No	07:25	07:00 - 07:59	Morning	37,5	141	98,2	98,2	18,65	617,75	155,21
2019/05/29	CH2205	Male	Three y/o	Treatment (Wk 3)	Treatment	Fast Day	No	07:30	07:00 - 07:59	Morning	37,4	83	225,2	225,2	18,55	580,11	184,30
2019/05/29	CH2206	Male	Three y/o	Treatment (Wk 3)	Treatment	Fast Day	No	07:30	07:00 - 07:59	Morning	37,5	78	104,0	104,0	18,65	575,38	157,21
2019/05/29	CH2205	Male	Three y/o	Treatment (Wk 3)	Treatment	Fast Day	No	07:35	07:00 - 07:59	Morning	37,4		63,4	63,4	18,55		140,00
2019/05/29	CH2206	Male	Three y/o	Treatment (Wk 3)	Treatment	Fast Day	No	07:35	07:00 - 07:59	Morning	37,7	128	95,8	95,8	18,85	611,22	154,35
2019/05/29	CH2205	Male	Three y/o	Treatment (Wk 3)	Treatment	Fast Day	No	07:40	07:00 - 07:59	Morning	37,5		61,4	61,4	18,65		138,89
2019/05/29	CH2206	Male	Three y/o	Treatment (Wk 3)	Treatment	Fast Day	No	07:40	07:00 - 07:59	Morning	37,7	137	89,4	89,4	18,85	615,83	151,94
2019/05/29	CH2205	Male	Three y/o	Treatment (Wk 3)	Treatment	Fast Day	No	07:45	07:00 - 07:59	Morning	37,4	86	34,0	34,0	18,55	582,77	118,49
2019/05/29	CH2206	Male	Three y/o	Treatment (Wk 3)	Treatment	Fast Day	No	07:45	07:00 - 07:59	Morning	37,5	128	61,8	61,8	18,65	611,22	139,12
2019/05/29	CH2205	Male	Three y/o	Treatment (Wk 3)	Treatment	Fast Day	No	07:50	07:00 - 07:59	Morning	37,4	141	149,6	149,6	18,55	617,75	169,92
2019/05/29	CH2206	Male	Three y/o	Treatment (Wk 3)	Treatment	Fast Day	No	07:50	07:00 - 07:59	Morning	37,7	107	63,4	63,4	18,85	598,73	140,00
2019/05/29	CH2205	Male	Three y/o	Treatment (Wk 3)	Treatment	Fast Day	No	07:55	07:00 - 07:59	Morning	37,4	106	59,8	59,8	18,55	598,06	137,98
2019/05/29	CH2206	Male	Three y/o	Treatment (Wk 3)	Treatment	Fast Day	No	07:55	07:00 - 07:59	Morning	37,7	129	59,6	59,6	18,85	611,75	137,86
2019/05/29	CH2205	Male	Three y/o	Treatment (Wk 3)	Treatment	Fast Day	No	08:00	08:00 - 08:59	Late Morning	37,5	36	52,0	52,0	18,65	510,56	133,14

2019/05/29	CH2206	Male	Three y/o	Treatment (Wk 3)	Treatment	Fast Day	No	08:00	08:00 - 08:59	Late Morning	37,7	106	137,0	137,0	18,85	598,06	166,84
2019/05/29	CH2205	Male	Three y/o	Treatment (Wk 3)	Treatment	Fast Day	No	08:05	08:00 - 08:59	Late Morning	37,5	117	82,6	82,6	18,65	605,02	149,19
2019/05/29	CH2206	Male	Three y/o	Treatment (Wk 3)	Treatment	Fast Day	No	08:05	08:00 - 08:59	Late Morning	37,7	123	227,6	227,6	18,85	608,49	184,67
2019/05/29	CH2205	Male	Three y/o	Treatment (Wk 3)	Treatment	Fast Day	No	08:10	08:00 - 08:59	Late Morning	37,5	188	128,6	128,6	18,65	636,34	164,63
2019/05/29	CH2206	Male	Three y/o	Treatment (Wk 3)	Treatment	Fast Day	No	08:10	08:00 - 08:59	Late Morning	37,7	108	148,4	148,4	18,85	599,39	169,64
2019/05/29	CH2205	Male	Three y/o	Treatment (Wk 3)	Treatment	Fast Day	No	08:15	08:00 - 08:59	Late Morning	37,5	52	285,8	285,8	18,65	542,83	192,71
2019/05/29	CH2206	Male	Three y/o	Treatment (Wk 3)	Treatment	Fast Day	No	08:15	08:00 - 08:59	Late Morning	37,7	113	147,2	147,2	18,85	602,58	169,36
2019/05/29	CH2205	Male	Three y/o	Treatment (Wk 3)	Treatment	Fast Day	No	08:20	08:00 - 08:59	Late Morning	37,5		165,0	165,0	18,65		173,36
2019/05/29	CH2206	Male	Three y/o	Treatment (Wk 3)	Treatment	Fast Day	No	08:20	08:00 - 08:59	Late Morning	37,7	98	235,4	235,4	18,85	592,41	185,86
2019/05/29	CH2205	Male	Three y/o	Treatment (Wk 3)	Treatment	Fast Day	No	08:25	08:00 - 08:59	Late Morning	37,6	182	64,8	64,8	18,75	634,31	140,76
2019/05/29	CH2206	Male	Three y/o	Treatment (Wk 3)	Treatment	Fast Day	No	08:25	08:00 - 08:59	Late Morning	37,7	109	102,6	102,6	18,85	600,04	156,74
2019/05/29	CH2205	Male	Three y/o	Treatment (Wk 3)	Treatment	Fast Day	No	08:30	08:00 - 08:59	Late Morning	37,6	120	46,8	46,8	18,75	606,78	129,50
2019/05/29	CH2206	Male	Three y/o	Treatment (Wk 3)	Treatment	Fast Day	No	08:30	08:00 - 08:59	Late Morning	37,7	182	51,0	51,0	18,85	634,31	132,47
2019/05/29	CH2205	Male	Three y/o	Treatment (Wk 3)	Treatment	Fast Day	No	08:35	08:00 - 08:59	Late Morning	37,5	85	165,2	165,2	18,65	581,90	173,40
2019/05/29	CH2206	Male	Three y/o	Treatment (Wk 3)	Treatment	Fast Day	No	08:35	08:00 - 08:59	Late Morning	37,7	156	141,6	141,6	18,85	624,43	168,00
2019/05/29	CH2205	Male	Three y/o	Treatment (Wk 3)	Treatment	Fast Day	No	08:40	08:00 - 08:59	Late Morning	37,7	82	362,0	362,0	18,85	579,19	201,07
2019/05/29	CH2206	Male	Three y/o	Treatment (Wk 3)	Treatment	Fast Day	No	08:40	08:00 - 08:59	Late Morning	37,7	126	356,4	356,4	18,85	610,14	200,52
2019/05/29	CH2205	Male	Three y/o	Treatment (Wk 3)	Treatment	Fast Day	No	08:45	08:00 - 08:59	Late Morning	37,6		166,2	166,2	18,75		173,61
2019/05/29	CH2206	Male	Three y/o	Treatment (Wk 3)	Treatment	Fast Day	No	08:45	08:00 - 08:59	Late Morning	37,7	143	102,2	102,2	18,85	618,69	156,60
2019/05/29	CH2205	Male	Three y/o	Treatment (Wk 3)	Treatment	Fast Day	No	08:50	08:00 - 08:59	Late Morning	37,6	89	171,6	171,6	18,75	585,33	174,74
2019/05/29	CH2206	Male	Three y/o	Treatment (Wk 3)	Treatment	Fast Day	No	08:50	08:00 - 08:59	Late Morning	37,8	128	101,4	101,4	18,95	611,22	156,33
2019/05/29	CH2205	Male	Three y/o	Treatment (Wk 3)	Treatment	Fast Day	No	08:55	08:00 - 08:59	Late Morning	37,6	172	45,4	45,4	18,75	630,73	128,45
2019/05/29	CH2206	Male	Three y/o	Treatment (Wk 3)	Treatment	Fast Day	No	08:55	08:00 - 08:59	Late Morning	37,8	109	124,8	124,8	18,95	600,04	163,58
2019/05/29	CH2205	Male	Three y/o	Treatment (Wk 3)	Treatment	Fast Day	No	09:00	09:00 - 09:59	Late Morning	37,6	180	34,2	34,2	18,75	633,61	118,70
2019/05/29	CH2206	Male	Three y/o	Treatment (Wk 3)	Treatment	Fast Day	No	09:00	09:00 - 09:59	Late Morning	37,8		282,0	282,0	18,95		192,23
2019/05/29	CH2205	Male	Three y/o	Treatment (Wk 3)	Treatment	Fast Day	No	09:05	09:00 - 09:59	Late Morning	37,6	76	111,6	111,6	18,75	573,39	159,67
2019/05/29	CH2206	Male	Three y/o	Treatment (Wk 3)	Treatment	Fast Day	No	09:05	09:00 - 09:59	Late Morning	37,7	134	178,8	178,8	18,85	614,33	176,18
2019/05/29	CH2205	Male	Three y/o	Treatment (Wk 3)	Treatment	Fast Day	No	09:10	09:00 - 09:59	Late Morning	37,7	102	99,0	99,0	18,85	595,30	155,49
2019/05/29	CH2206	Male	Three y/o	Treatment (Wk 3)	Treatment	Fast Day	No	09:10	09:00 - 09:59	Late Morning	37,8	90	157,4	157,4	18,95	586,16	171,71
2019/05/29	CH2205	Male	Three y/o	Treatment (Wk 3)	Treatment	Fast Day	No	09:15	09:00 - 09:59	Late Morning	37,7	117	75,2	75,2	18,85	605,02	145,93
2019/05/29	CH2206	Male	Three y/o	Treatment (Wk 3)	Treatment	Fast Day	No	09:15	09:00 - 09:59	Late Morning	37,7	129	84,4	84,4	18,85	611,75	149,94
2019/05/29	CH2205	Male	Three y/o	Treatment (Wk 3)	Treatment	Fast Day	No	09:20	09:00 - 09:59	Late Morning	37,6	92	115,4	115,4	18,75	587,78	160,84
2019/05/29	CH2206	Male	Three y/o	Treatment (Wk 3)	Treatment	Fast Day	No	09:20	09:00 - 09:59	Late Morning	37,8		152,0	152,0	18,95		170,48
2019/05/29	CH2205	Male	Three y/o	Treatment (Wk 3)	Treatment	Fast Day	No	09:25	09:00 - 09:59	Late Morning	37,7	63	137,0	137,0	18,85	558,62	166,84
2019/05/29	CH2206	Male	Three y/o	Treatment (Wk 3)	Treatment	Fast Day	No	09:25	09:00 - 09:59	Late Morning	38,0		145,4	145,4	19,15		168,93
2019/05/29	CH2205	Male	Three y/o	Treatment (Wk 3)	Treatment	Fast Day	No	09:30	09:00 - 09:59	Late Morning	38,1	160	39,2	39,2	19,26	626,07	123,39
2019/05/29	CH2206	Male	Three y/o	Treatment (Wk 3)	Treatment	Fast Day	No	09:30	09:00 - 09:59	Late Morning	38,0	49	36,0	36,0	19,15	537,80	120,46
2019/05/29	CH2205	Male	Three y/o	Treatment (Wk 3)	Treatment	Fast Day	No	09:35	09:00 - 09:59	Late Morning	38,1	55	63,0	63,0	19,26	547,52	139,78
2019/05/29	CH2206	Male	Three y/o	Treatment (Wk 3)	Treatment	Fast Day	No	09:35	09:00 - 09:59	Late Morning	37,9	40	165,8	165,8	19,05	520,09	173,53
2019/05/29	CH2205	Male	Three y/o	Treatment (Wk 3)	Treatment	Fast Day	No	09:40	09:00 - 09:59	Late Morning	38,0	73	15,4	15,4	19,15	570,27	91,41
2019/05/29	CH2206	Male	Three y/o	Treatment (Wk 3)	Treatment	Fast Day	No	09:40	09:00 - 09:59	Late Morning	37,9	121	251,6	251,6	19,05	607,35	188,21
2019/05/29	CH2205	Male	Three y/o	Treatment (Wk 3)	Treatment	Fast Day	No	09:45	09:00 - 09:59	Late Morning	38,2	110	15,6	15,6	19,36	600,69	91,85
2019/05/29	CH2206	Male	Three y/o	Treatment (Wk 3)	Treatment	Fast Day	No	09:45	09:00 - 09:59	Late Morning	37,9	117	33,4	33,4	19,05	605,02	117,88
2019/05/29	CH2205	Male	Three y/o	Treatment (Wk 3)	Treatment	Fast Day	No	09:50	09:00 - 09:59	Late Morning	38,1		91,0	91,0	19,26		152,56
2019/05/29	CH2206	Male	Three y/o	Treatment (Wk 3)	Treatment	Fast Day	No	09:50	09:00 - 09:59	Late Morning	37,8	158	107,2	107,2	18,95	625,26	158,27
2019/05/29	CH2205	Male	Three y/o	Treatment (Wk 3)	Treatment	Fast Day	No	09:55	09:00 - 09:59	Late Morning	38,1	70	145,4	145,4	19,26	566,99	168,93
2019/05/29	CH2206	Male	Three y/o	Treatment (Wk 3)	Treatment	Fast Day	No	09:55	09:00 - 09:59	Late Morning	37,9	117	191,4	191,4	19,05	605,02	178,57
2019/05/29	CH2205	Male	Three y/o	Treatment (Wk 3)	Treatment	Fast Day	No	10:00	10:00 - 10:59	Late Morning	38,1	94	50,8	50,8	19,26	589,37	132,33
2019/05/29	CH2206	Male	Three y/o	Treatment (Wk 3)	Treatment	Fast Day	No	10:00	10:00 - 10:59	Late Morning	37,9	118	96,8	96,8	19,05	605,61	154,71
2019/05/29	CH2205	Male	Three y/o	Treatment (Wk 3)	Treatment	Fast Day	No	10:05	10:00 - 10:59	Late Morning	38,0	72	17,0	17,0	19,15	569,20	94,77
2019/05/29	CH2206	Male	Three y/o	Treatment (Wk 3)	Treatment	Fast Day	No	10:05	10:00 - 10:59	Late Morning	37,9	120	81,2	81,2	19,05	606,78	148,59
2019/05/29	CH2205	Male	Three y/o	Treatment (Wk 3)	Treatment	Fast Day	No	10:10	10:00 - 10:59	Late Morning	38,0	60	66,8	66,8	19,15	554,67	141,81
2019/05/29	CH2206	Male	Three y/o	Treatment (Wk 3)	Treatment	Fast Day	No	10:10	10:00 - 10:59	Late Morning	37,9	140	151,0	151,0	19,05	617,28	170,25
2019/05/29	CH2205	Male	Three y/o	Treatment (Wk 3)	Treatment	Fast Day	No	10:15	10:00 - 10:59	Late Morning	38,1	48	257,0	257,0	19,26	536,04	188,95
2019/05/29	CH2206	Male	Three y/o	Treatment (Wk 3)	Treatment	Fast Day	No	10:15	10:00 - 10:59	Late Morning	37,9	138	37,4	37,4	19,05	616,31	121,77
2019/05/29	CH2205	Male	Three y/o	Treatment (Wk 3)	Treatment	Fast Day	No	10:20	10:00 - 10:59	Late Morning	38,0	88	72,4	72,4	19,15	584,49	144,61
2019/05/29	CH2206	Male	Three y/o	Treatment (Wk 3)	Treatment	Fast Day	No	10:20	10:00 - 10:59	Late Morning	37,9	89	25,8	25,8	19,05	585,33	109,02
2019/05/29	CH2205	Male	Three y/o	Treatment (Wk 3)	Treatment	Fast Day	No	10:25	10:00 - 10:59	Late Morning	37,9	67	32,8	32,8	19,05	563,53	117,26
2019/05/29	CH2206	Male	Three y/o	Treatment (Wk 3)	Treatment	Fast Day	No	10:25	10:00 - 10:59	Late Morning	37,9	65	19,0	19,0	19,05	561,12	98,56
2019/05/29	CH2205	Male	Three y/o	Treatment (Wk 3)	Treatment	Fast Day	No	10:30	10:00 - 10:59	Late Morning	38,0	60	76,6	76,6	19,15	554,67	146,57
2019/05/29	CH2206	Male	Three y/o	Treatment (Wk 3)	Treatment	Fast Day	No	10:30	10:00 - 10:59	Late Morning	37,9	69	19,0	19,0	19,05	565,86	98,56
2019/05/29	CH2205	Male	Three y/o	Treatment (Wk 3)	Treatment	Fast Day	No	10:35	10:00 - 10:59	Late Morning	38,0	99	52,0	52,0	19,15	593,14	133,14
2019/05/29	CH2206	Male	Three y/o	Treatment (Wk 3)	Treatment	Fast Day	No	10:35	10:00 - 10:59	Late Morning	37,8	73	41,0	41,0	18,95	570,27	124,94
2019/05/29	CH2205	Male	Three y/o	Treatment (Wk 3)	Treatment	Fast Day	No	10:40	10:00 - 10:59	Late Morning	38,0	60	3,6	3,6	19,15	554,67	42,42
2019/05/29	CH2206	Male	Three y/o	Treatment (Wk 3)	Treatment	Fast Day	No	10:40	10:00 - 10:59	Late Morning	37,9	51	22,8	22,8	19,05	541,20	104,79
2019/05/29	CH2205	Male	Three y/o	Treatment (Wk 3)	Treatment	Fast Day	No	10:45	10:00 - 10:59	Late Morning	38,0	68	3,4	3,4	19,15	564,71	40,51
2019/05/29	CH2206	Male	Three y/o	Treatment (Wk 3)	Treatment	Fast Day	No	10:45	10:00 - 10:59	Late Morning	37,8	48	31,4	31,4	18,95	536,04	115,76
2019/05/29	CH2205	Male	Three y/o	Treatment (Wk 3)	Treatment	Fast Day	No	10:50	10:00 - 10:59	Late Morning	38,0	58	12,2	12,2	19,15	551,90	83,49
2019/05/29	CH2206	Male	Three y/o	Treatment (Wk 3)	Treatment	Fast Day	No	10:50	10:00 - 10:59	Late Morning	37,8	42	21,4	21,4	18,95	524,42	102,63
2019/05/29	CH2205	Male	Three y/o	Treatment (Wk 3)	Treatment	Fast Day	No	10:55	10:00 - 10:59	Late Morning	37,9	53	3,8	3,8	19,05	544,43	44,22

2019/05/29	CH2206	Male	Three y/o	Treatment (Wk 3)	Treatment	Fast Day	No	10:55	10:00 - 10:59	Late Morning	37,8	43	25,2	25,2	18,95	526,50	108,22
2019/05/29	CH2205	Male	Three y/o	Treatment (Wk 3)	Treatment	Fast Day	No	11:00	11:00 - 11:59	Late Morning	37,9		24,4	24,4	19,05		107,11
2019/05/29	CH2206	Male	Three y/o	Treatment (Wk 3)	Treatment	Fast Day	No	11:00	11:00 - 11:59	Late Morning	37,8	42	45,0	45,0	18,95	524,42	128,15
2019/05/29	CH2205	Male	Three y/o	Treatment (Wk 3)	Treatment	Fast Day	No	11:05	11:00 - 11:59	Late Morning	38,0	73	5,0	5,0	19,15	570,27	53,41
2019/05/29	CH2206	Male	Three y/o	Treatment (Wk 3)	Treatment	Fast Day	No	11:05	11:00 - 11:59	Late Morning	37,8	84	38,6	38,6	18,95	581,01	122,86
2019/05/29	CH2205	Male	Three y/o	Treatment (Wk 3)	Treatment	Fast Day	No	11:10	11:00 - 11:59	Late Morning	37,9	63	5,4	5,4	19,05	558,62	55,99
2019/05/29	CH2206	Male	Three y/o	Treatment (Wk 3)	Treatment	Fast Day	No	11:10	11:00 - 11:59	Late Morning	37,7	42	28,6	28,6	18,85	524,42	112,56
2019/05/29	CH2205	Male	Three y/o	Treatment (Wk 3)	Treatment	Fast Day	No	11:15	11:00 - 11:59	Late Morning	37,9	66	9,0	9,0	19,05	562,34	73,19
2019/05/29	CH2206	Male	Three y/o	Treatment (Wk 3)	Treatment	Fast Day	No	11:15	11:00 - 11:59	Late Morning	37,7	88	31,6	31,6	18,85	584,49	115,98
2019/05/29	CH2205	Male	Three y/o	Treatment (Wk 3)	Treatment	Fast Day	No	11:20	11:00 - 11:59	Late Morning	37,9	46	7,0	7,0	19,05	532,38	64,72
2019/05/29	CH2206	Male	Three y/o	Treatment (Wk 3)	Treatment	Fast Day	No	11:20	11:00 - 11:59	Late Morning	37,7	47	32,4	32,4	18,85	534,23	116,84
2019/05/29	CH2205	Male	Three y/o	Treatment (Wk 3)	Treatment	Fast Day	No	11:25	11:00 - 11:59	Late Morning	37,8	66	14,2	14,2	18,95	562,34	88,65
2019/05/29	CH2206	Male	Three y/o	Treatment (Wk 3)	Treatment	Fast Day	No	11:25	11:00 - 11:59	Late Morning	37,7	83	29,0	29,0	18,85	580,11	113,03
2019/05/29	CH2205	Male	Three y/o	Treatment (Wk 3)	Treatment	Fast Day	No	11:30	11:00 - 11:59	Late Morning	37,7	49	9,8	9,8	18,85	537,80	76,07
2019/05/29	CH2206	Male	Three y/o	Treatment (Wk 3)	Treatment	Fast Day	No	11:30	11:00 - 11:59	Late Morning	37,6	94	31,2	31,2	18,75	589,37	115,54
2019/05/29	CH2205	Male	Three y/o	Treatment (Wk 3)	Treatment	Fast Day	No	11:35	11:00 - 11:59	Late Morning	37,7	50	6,8	6,8	18,85	539,52	63,74
2019/05/29	CH2206	Male	Three y/o	Treatment (Wk 3)	Treatment	Fast Day	No	11:35	11:00 - 11:59	Late Morning	37,7	72	43,6	43,6	18,85	569,20	127,06
2019/05/29	CH2205	Male	Three y/o	Treatment (Wk 3)	Treatment	Fast Day	No	11:40	11:00 - 11:59	Late Morning	37,7	53	6,6	6,6	18,85	544,43	62,73
2019/05/29	CH2206	Male	Three y/o	Treatment (Wk 3)	Treatment	Fast Day	No	11:40	11:00 - 11:59	Late Morning	37,7	70	26,6	26,6	18,85	566,99	110,07
2019/05/29	CH2205	Male	Three y/o	Treatment (Wk 3)	Treatment	Fast Day	No	11:45	11:00 - 11:59	Late Morning	37,7	83	16,0	16,0	18,85	580,11	92,71
2019/05/29	CH2206	Male	Three y/o	Treatment (Wk 3)	Treatment	Fast Day	No	11:45	11:00 - 11:59	Late Morning	37,7	78	19,0	19,0	18,85	575,38	98,56
2019/05/29	CH2205	Male	Three y/o	Treatment (Wk 3)	Treatment	Fast Day	No	11:50	11:00 - 11:59	Late Morning	37,7	53	18,4	18,4	18,85	544,43	97,47
2019/05/29	CH2206	Male	Three y/o	Treatment (Wk 3)	Treatment	Fast Day	No	11:50	11:00 - 11:59	Late Morning	37,5	46	21,0	21,0	18,65	532,38	101,98
2019/05/29	CH2205	Male	Three y/o	Treatment (Wk 3)	Treatment	Fast Day	No	11:55	11:00 - 11:59	Late Morning	37,7	64	23,8	23,8	18,85	559,88	106,26
2019/05/29	CH2206	Male	Three y/o	Treatment (Wk 3)	Treatment	Fast Day	No	11:55	11:00 - 11:59	Late Morning	37,5	45	20,2	20,2	18,65	530,47	100,65
2019/05/29	CH2205	Male	Three y/o	Treatment (Wk 3)	Treatment	Fast Day	No	12:00	12:00 - 12:59	Afternoon	37,7	52	23,6	23,6	18,85	542,83	105,97
2019/05/29	CH2206	Male	Three y/o	Treatment (Wk 3)	Treatment	Fast Day	No	12:00	12:00 - 12:59	Afternoon	37,5	49	38,8	38,8	18,65	537,80	123,04
2019/05/29	CH2205	Male	Three y/o	Treatment (Wk 3)	Treatment	Fast Day	No	12:05	12:00 - 12:59	Afternoon	37,7	53	23,6	23,6	18,85	544,43	105,97
2019/05/29	CH2206	Male	Three y/o	Treatment (Wk 3)	Treatment	Fast Day	No	12:05	12:00 - 12:59	Afternoon	37,5	94	28,2	28,2	18,65	589,37	112,07
2019/05/29	CH2205	Male	Three y/o	Treatment (Wk 3)	Treatment	Fast Day	No	12:10	12:00 - 12:59	Afternoon	37,7	61	25,4	25,4	18,85	556,01	108,49
2019/05/29	CH2206	Male	Three y/o	Treatment (Wk 3)	Treatment	Fast Day	No	12:10	12:00 - 12:59	Afternoon	37,5	45	29,6	29,6	18,65	530,47	113,73
2019/05/29	CH2205	Male	Three y/o	Treatment (Wk 3)	Treatment	Fast Day	No	12:15	12:00 - 12:59	Afternoon	37,7	53	21,4	21,4	18,85	544,43	102,63
2019/05/29	CH2206	Male	Three y/o	Treatment (Wk 3)	Treatment	Fast Day	No	12:15	12:00 - 12:59	Afternoon	37,5	48	16,8	16,8	18,65	536,04	94,37
2019/05/29	CH2205	Male	Three y/o	Treatment (Wk 3)	Treatment	Fast Day	No	12:20	12:00 - 12:59	Afternoon	37,7	69	5,6	5,6	18,85	565,86	57,21
2019/05/29	CH2206	Male	Three y/o	Treatment (Wk 3)	Treatment	Fast Day	No	12:20	12:00 - 12:59	Afternoon	37,5	79	6,8	6,8	18,65	576,36	63,74
2019/05/29	CH2205	Male	Three y/o	Treatment (Wk 3)	Treatment	Fast Day	No	12:25	12:00 - 12:59	Afternoon	37,7	47	6,4	6,4	18,85	534,23	61,70
2019/05/29	CH2206	Male	Three y/o	Treatment (Wk 3)	Treatment	Fast Day	No	12:25	12:00 - 12:59	Afternoon	37,5	41	20,2	20,2	18,65	522,29	100,65
2019/05/29	CH2205	Male	Three y/o	Treatment (Wk 3)	Treatment	Fast Day	No	12:30	12:00 - 12:59	Afternoon	37,7	42	8,2	8,2	18,85	524,42	70,05
2019/05/29	CH2206	Male	Three y/o	Treatment (Wk 3)	Treatment	Fast Day	No	12:30	12:00 - 12:59	Afternoon	37,5	87	19,8	19,8	18,65	583,64	99,97
2019/05/29	CH2205	Male	Three y/o	Treatment (Wk 3)	Treatment	Fast Day	No	12:35	12:00 - 12:59	Afternoon	37,7	51	6,4	6,4	18,85	541,20	61,70
2019/05/29	CH2206	Male	Three y/o	Treatment (Wk 3)	Treatment	Fast Day	No	12:35	12:00 - 12:59	Afternoon	37,5	67	25,4	25,4	18,65	563,53	108,49
2019/05/29	CH2205	Male	Three y/o	Treatment (Wk 3)	Treatment	Fast Day	No	12:40	12:00 - 12:59	Afternoon	37,7	42	12,0	12,0	18,85	524,42	82,93
2019/05/29	CH2206	Male	Three y/o	Treatment (Wk 3)	Treatment	Fast Day	No	12:40	12:00 - 12:59	Afternoon	37,5	68	20,8	20,8	18,65	564,71	101,65
2019/05/29	CH2205	Male	Three y/o	Treatment (Wk 3)	Treatment	Fast Day	No	12:45	12:00 - 12:59	Afternoon	37,7	162	27,2	27,2	18,85	626,88	110,83
2019/05/29	CH2206	Male	Three y/o	Treatment (Wk 3)	Treatment	Fast Day	No	12:45	12:00 - 12:59	Afternoon	37,5	60	18,0	18,0	18,65	554,67	96,72
2019/05/29	CH2205	Male	Three y/o	Treatment (Wk 3)	Treatment	Fast Day	No	12:50	12:00 - 12:59	Afternoon	37,7	49	12,8	12,8	18,85	537,80	85,12
2019/05/29	CH2206	Male	Three y/o	Treatment (Wk 3)	Treatment	Fast Day	No	12:50	12:00 - 12:59	Afternoon	37,5	54	28,8	28,8	18,65	545,99	112,79
2019/05/29	CH2205	Male	Three y/o	Treatment (Wk 3)	Treatment	Fast Day	No	12:55	12:00 - 12:59	Afternoon	37,6	78	87,4	87,4	18,75	575,38	151,15
2019/05/29	CH2206	Male	Three y/o	Treatment (Wk 3)	Treatment	Fast Day	No	12:55	12:00 - 12:59	Afternoon	37,5	148	125,8	125,8	18,65	620,97	163,86
2019/05/29	CH2205	Male	Three y/o	Treatment (Wk 3)	Treatment	Fast Day	No	13:00	13:00 - 13:59	Afternoon	37,6	90	210,4	210,4	18,75	586,16	181,90
2019/05/29	CH2206	Male	Three y/o	Treatment (Wk 3)	Treatment	Fast Day	No	13:00	13:00 - 13:59	Afternoon	37,5	118	109,2	109,2	18,65	605,61	158,91
2019/05/29	CH2205	Male	Three y/o	Treatment (Wk 3)	Treatment	Fast Day	No	13:05	13:00 - 13:59	Afternoon	37,7		147,4	147,4	18,85		169,40
2019/05/29	CH2206	Male	Three y/o	Treatment (Wk 3)	Treatment	Fast Day	No	13:05	13:00 - 13:59	Afternoon	37,7		112,8	112,8	18,85		160,05
2019/05/29	CH2205	Male	Three y/o	Treatment (Wk 3)	Treatment	Fast Day	No	13:10	13:00 - 13:59	Afternoon	37,7	70	80,6	80,6	18,85	566,99	148,34
2019/05/29	CH2206	Male	Three y/o	Treatment (Wk 3)	Treatment	Fast Day	No	13:10	13:00 - 13:59	Afternoon	37,7	126	170,4	170,4	18,85	610,14	174,49
2019/05/29	CH2205	Male	Three y/o	Treatment (Wk 3)	Treatment	Fast Day	No	13:15	13:00 - 13:59	Afternoon	37,7	165	202,8	202,8	18,85	628,06	180,61
2019/05/29	CH2206	Male	Three y/o	Treatment (Wk 3)	Treatment	Fast Day	No	13:15	13:00 - 13:59	Afternoon	37,7	110	133,6	133,6	18,85	600,69	165,96
2019/05/29	CH2205	Male	Three y/o	Treatment (Wk 3)	Treatment	Fast Day	No	13:20	13:00 - 13:59	Afternoon	37,9	49	311,6	311,6	19,05	537,80	195,76
2019/05/29	CH2206	Male	Three y/o	Treatment (Wk 3)	Treatment	Fast Day	No	13:20	13:00 - 13:59	Afternoon	37,8	135	29,2	29,2	18,95	614,84	113,27
2019/05/29	CH2205	Male	Three y/o	Treatment (Wk 3)	Treatment	Fast Day	No	13:25	13:00 - 13:59	Afternoon	37,9	91	118,2	118,2	19,05	586,98	161,68
2019/05/29	CH2206	Male	Three y/o	Treatment (Wk 3)	Treatment	Fast Day	No	13:25	13:00 - 13:59	Afternoon	37,8	106	243,0	243,0	18,95	598,06	186,98
2019/05/29	CH2205	Male	Three y/o	Treatment (Wk 3)	Treatment	Fast Day	No	13:30	13:00 - 13:59	Afternoon	38,0	129	297,6	297,6	19,15	611,75	194,14
2019/05/29	CH2206	Male	Three y/o	Treatment (Wk 3)	Treatment	Fast Day	No	13:30	13:00 - 13:59	Afternoon	37,8	110	191,8	191,8	18,95	600,69	178,65
2019/05/29	CH2205	Male	Three y/o	Treatment (Wk 3)	Treatment	Fast Day	No	13:35	13:00 - 13:59	Afternoon	38,1		123,0	123,0	19,26		163,07
2019/05/29	CH2206	Male	Three y/o	Treatment (Wk 3)	Treatment	Fast Day	No	13:35	13:00 - 13:59	Afternoon	37,9	154	354,2	354,2	19,05	623,58	200,30
2019/05/29	CH2205	Male	Three y/o	Treatment (Wk 3)	Treatment	Fast Day	No	13:40	13:00 - 13:59	Afternoon	38,1	47	259,8	259,8	19,26	534,23	189,34
2019/05/29	CH2206	Male	Three y/o	Treatment (Wk 3)	Treatment	Fast Day	No	13:40	13:00 - 13:59	Afternoon	37,9	123	119,0	119,0	19,05	608,49	161,92
2019/05/29	CH2205	Male	Three y/o	Treatment (Wk 3)	Treatment	Fast Day	No	13:45	13:00 - 13:59	Afternoon	38,1	82	88,8	88,8	19,26	579,19	151,71
2019/05/29	CH2206	Male	Three y/o	Treatment (Wk 3)	Treatment	Fast Day	No	13:45	13:00 - 13:59	Afternoon	37,9		52,0	52,0	19,05		133,14
2019/05/29	CH2205	Male	Three y/o	Treatment (Wk 3)	Treatment	Fast Day	No	13:50	13:00 - 13:59	Afternoon	38,1	99	164,4	164,4	19,26	593,14	173,23

2019/05/29	CH2206	Male	Three y/o	Treatment (Wk 3)	Treatment	Fast Day	No	13:50	13:00 - 13:59	Afternoon	37,9		104,6	104,6	19,05		157,41
2019/05/29	CH2205	Male	Three y/o	Treatment (Wk 3)	Treatment	Fast Day	No	13:55	13:00 - 13:59	Afternoon	38,2	165	127,0	127,0	19,36	628,06	164,19
2019/05/29	CH2206	Male	Three y/o	Treatment (Wk 3)	Treatment	Fast Day	No	13:55	13:00 - 13:59	Afternoon	38,0	121	138,6	138,6	19,15	607,35	167,25
2019/05/29	CH2205	Male	Three y/o	Treatment (Wk 3)	Treatment	Fast Day	No	14:00	14:00 - 14:59	Afternoon	38,2	107	186,6	186,6	19,36	598,73	177,68
2019/05/29	CH2206	Male	Three y/o	Treatment (Wk 3)	Treatment	Fast Day	No	14:00	14:00 - 14:59	Afternoon	38,0	126	77,4	77,4	19,15	610,14	146,93
2019/05/29	CH2205	Male	Three y/o	Treatment (Wk 3)	Treatment	Fast Day	No	14:05	14:00 - 14:59	Afternoon	38,2	131	84,0	84,0	19,36	612,80	149,77
2019/05/29	CH2206	Male	Three y/o	Treatment (Wk 3)	Treatment	Fast Day	No	14:05	14:00 - 14:59	Afternoon	38,0	102	311,8	311,8	19,15	595,30	195,79
2019/05/29	CH2205	Male	Three y/o	Treatment (Wk 3)	Treatment	Fast Day	No	14:10	14:00 - 14:59	Afternoon	38,2	132	77,6	77,6	19,36	613,32	147,02
2019/05/29	CH2206	Male	Three y/o	Treatment (Wk 3)	Treatment	Fast Day	No	14:10	14:00 - 14:59	Afternoon	38,0		166,0	166,0	19,15		173,57
2019/05/29	CH2205	Male	Three y/o	Treatment (Wk 3)	Treatment	Fast Day	No	14:15	14:00 - 14:59	Afternoon	38,2	52	230,0	230,0	19,36	542,83	185,04
2019/05/29	CH2206	Male	Three y/o	Treatment (Wk 3)	Treatment	Fast Day	No	14:15	14:00 - 14:59	Afternoon	38,1	131	103,8	103,8	19,26	612,80	157,14
2019/05/29	CH2205	Male	Three y/o	Treatment (Wk 3)	Treatment	Fast Day	No	14:20	14:00 - 14:59	Afternoon	38,2	73	76,2	76,2	19,36	570,27	146,38
2019/05/29	CH2206	Male	Three y/o	Treatment (Wk 3)	Treatment	Fast Day	No	14:20	14:00 - 14:59	Afternoon	38,1		122,0	122,0	19,26		162,79
2019/05/29	CH2205	Male	Three y/o	Treatment (Wk 3)	Treatment	Fast Day	No	14:25	14:00 - 14:59	Afternoon	38,2	97	141,0	141,0	19,36	591,66	167,85
2019/05/29	CH2206	Male	Three y/o	Treatment (Wk 3)	Treatment	Fast Day	No	14:25	14:00 - 14:59	Afternoon	38,1	108	145,2	145,2	19,26	599,39	168,88
2019/05/29	CH2205	Male	Three y/o	Treatment (Wk 3)	Treatment	Fast Day	No	14:30	14:00 - 14:59	Afternoon	38,2	124	52,8	52,8	19,36	609,04	133,67
2019/05/29	CH2206	Male	Three y/o	Treatment (Wk 3)	Treatment	Fast Day	No	14:30	14:00 - 14:59	Afternoon	38,1	114	142,6	142,6	19,26	603,20	168,24
2019/05/29	CH2205	Male	Three y/o	Treatment (Wk 3)	Treatment	Fast Day	No	14:35	14:00 - 14:59	Afternoon	38,2	41	400,6	400,6	19,36	522,29	204,67
2019/05/29	CH2206	Male	Three y/o	Treatment (Wk 3)	Treatment	Fast Day	No	14:35	14:00 - 14:59	Afternoon	38,2	105	309,2	309,2	19,36	597,38	195,49
2019/05/29	CH2205	Male	Three y/o	Treatment (Wk 3)	Treatment	Fast Day	No	14:40	14:00 - 14:59	Afternoon	38,7	137	112,2	112,2	19,87	615,83	159,86
2019/05/29	CH2206	Male	Three y/o	Treatment (Wk 3)	Treatment	Fast Day	No	14:40	14:00 - 14:59	Afternoon	38,2	114	82,6	82,6	19,36	603,20	149,19
2019/05/29	CH2205	Male	Three y/o	Treatment (Wk 3)	Treatment	Fast Day	No	14:45	14:00 - 14:59	Afternoon	38,5	160	25,4	25,4	19,66	626,07	108,49
2019/05/29	CH2206	Male	Three y/o	Treatment (Wk 3)	Treatment	Fast Day	No	14:45	14:00 - 14:59	Afternoon	38,3	114	90,6	90,6	19,46	603,20	152,40
2019/05/29	CH2205	Male	Three y/o	Treatment (Wk 3)	Treatment	Fast Day	No	14:50	14:00 - 14:59	Afternoon	38,8	115	39,6	39,6	19,97	603,82	123,74
2019/05/29	CH2206	Male	Three y/o	Treatment (Wk 3)	Treatment	Fast Day	No	14:50	14:00 - 14:59	Afternoon	38,3		196,6	196,6	19,46		179,52
2019/05/29	CH2205	Male	Three y/o	Treatment (Wk 3)	Treatment	Fast Day	No	14:55	14:00 - 14:59	Afternoon	38,8	156	2,2	2,2	19,97	624,43	26,02
2019/05/29	CH2206	Male	Three y/o	Treatment (Wk 3)	Treatment	Fast Day	No	14:55	14:00 - 14:59	Afternoon	38,3	58	175,8	175,8	19,46	551,90	175,59
2019/05/29	CH2205	Male	Three y/o	Treatment (Wk 3)	Treatment	Fast Day	No	15:00	15:00 - 15:59	Afternoon	38,8		352,4	352,4	19,97		200,12
2019/05/29	CH2206	Male	Three y/o	Treatment (Wk 3)	Treatment	Fast Day	No	15:00	15:00 - 15:59	Afternoon	38,3	113	167,8	167,8	19,46	602,58	173,95
2019/05/29	CH2205	Male	Three y/o	Treatment (Wk 3)	Treatment	Fast Day	No	15:05	15:00 - 15:59	Afternoon	39,0		135,2	135,2	20,18		166,38
2019/05/29	CH2206	Male	Three y/o	Treatment (Wk 3)	Treatment	Fast Day	No	15:05	15:00 - 15:59	Afternoon	38,3	119	132,0	132,0	19,46	606,20	165,54
2019/05/29	CH2205	Male	Three y/o	Treatment (Wk 3)	Treatment	Fast Day	No	15:10	15:00 - 15:59	Afternoon	38,9		128,2	128,2	20,07		164,52
2019/05/29	CH2206	Male	Three y/o	Treatment (Wk 3)	Treatment	Fast Day	No	15:10	15:00 - 15:59	Afternoon	38,3	117	151,0	151,0	19,46	605,02	170,25
2019/05/29	CH2205	Male	Three y/o	Treatment (Wk 3)	Treatment	Fast Day	No	15:15	15:00 - 15:59	Afternoon	39,0		65,0	65,0	20,18		140,87
2019/05/29	CH2206	Male	Three y/o	Treatment (Wk 3)	Treatment	Fast Day	No	15:15	15:00 - 15:59	Afternoon	38,3	135	118,2	118,2	19,46	614,84	161,68
2019/05/29	CH2205	Male	Three y/o	Treatment (Wk 3)	Treatment	Fast Day	No	15:20	15:00 - 15:59	Afternoon	38,9	96	32,0	32,0	20,07	590,91	116,41
2019/05/29	CH2206	Male	Three y/o	Treatment (Wk 3)	Treatment	Fast Day	No	15:20	15:00 - 15:59	Afternoon	38,4	86	31,4	31,4	19,56	582,77	115,76
2019/05/29	CH2205	Male	Three y/o	Treatment (Wk 3)	Treatment	Fast Day	No	15:25	15:00 - 15:59	Afternoon	38,9	48	29,4	29,4	20,07	536,04	113,50
2019/05/29	CH2206	Male	Three y/o	Treatment (Wk 3)	Treatment	Fast Day	No	15:25	15:00 - 15:59	Afternoon	38,3	73	9,2	9,2	19,46	570,27	73,94
2019/05/29	CH2205	Male	Three y/o	Treatment (Wk 3)	Treatment	Fast Day	No	15:30	15:00 - 15:59	Afternoon	38,9	77	29,0	29,0	20,07	574,39	113,03
2019/05/29	CH2206	Male	Three y/o	Treatment (Wk 3)	Treatment	Fast Day	No	15:30	15:00 - 15:59	Afternoon	38,3	60	18,4	18,4	19,46	554,67	97,47
2019/05/29	CH2205	Male	Three y/o	Treatment (Wk 3)	Treatment	Fast Day	No	15:35	15:00 - 15:59	Afternoon	38,8	73	30,6	30,6	19,97	570,27	114,87
2019/05/29	CH2206	Male	Three y/o	Treatment (Wk 3)	Treatment	Fast Day	No	15:35	15:00 - 15:59	Afternoon	38,3	53	16,4	16,4	19,46	544,43	93,55
2019/05/29	CH2205	Male	Three y/o	Treatment (Wk 3)	Treatment	Fast Day	No	15:40	15:00 - 15:59	Afternoon	38,8	57	32,0	32,0	19,97	550,47	116,41
2019/05/29	CH2206	Male	Three y/o	Treatment (Wk 3)	Treatment	Fast Day	No	15:40	15:00 - 15:59	Afternoon	38,3	52	43,6	43,6	19,46	542,83	127,06
2019/05/29	CH2205	Male	Three y/o	Treatment (Wk 3)	Treatment	Fast Day	No	15:45	15:00 - 15:59	Afternoon	38,7		77,8	77,8	19,87		147,11
2019/05/29	CH2206	Male	Three y/o	Treatment (Wk 3)	Treatment	Fast Day	No	15:45	15:00 - 15:59	Afternoon	38,3	49	178,6	178,6	19,46	537,80	176,14
2019/05/29	CH2205	Male	Three y/o	Treatment (Wk 3)	Treatment	Fast Day	No	15:50	15:00 - 15:59	Afternoon	38,4		20,6	20,6	19,56		101,32
2019/05/29	CH2206	Male	Three y/o	Treatment (Wk 3)	Treatment	Fast Day	No	15:50	15:00 - 15:59	Afternoon	38,3	62	244,4	244,4	19,46	557,33	187,18
2019/05/29	CH2205	Male	Three y/o	Treatment (Wk 3)	Treatment	Fast Day	No	15:55	15:00 - 15:59	Afternoon	38,3	74	48,2	48,2	19,46	571,33	130,52
2019/05/29	CH2206	Male	Three y/o	Treatment (Wk 3)	Treatment	Fast Day	No	15:55	15:00 - 15:59	Afternoon	38,2	180	299,2	299,2	19,36	633,61	194,33
2019/05/29	CH2205	Male	Three y/o	Treatment (Wk 3)	Treatment	Fast Day	No	16:00	16:00 - 16:59	Evening	38,4	46	108,8	108,8	19,56	532,38	158,79
2019/05/29	CH2206	Male	Three y/o	Treatment (Wk 3)	Treatment	Fast Day	No	16:00	16:00 - 16:59	Evening	38,3	128	62,4	62,4	19,46	611,22	139,45
2019/05/29	CH2205	Male	Three y/o	Treatment (Wk 3)	Treatment	Fast Day	No	16:05	16:00 - 16:59	Evening	38,4		324,6	324,6	19,56		197,21
2019/05/29	CH2206	Male	Three y/o	Treatment (Wk 3)	Treatment	Fast Day	No	16:05	16:00 - 16:59	Evening	38,3	154	112,8	112,8	19,46	623,58	160,05
2019/05/29	CH2205	Male	Three y/o	Treatment (Wk 3)	Treatment	Fast Day	No	16:10	16:00 - 16:59	Evening	38,7	105	34,6	34,6	19,87	597,38	119,10
2019/05/29	CH2206	Male	Three y/o	Treatment (Wk 3)	Treatment	Fast Day	No	16:10	16:00 - 16:59	Evening	38,4	118	146,6	146,6	19,56	605,61	169,21
2019/05/29	CH2205	Male	Three y/o	Treatment (Wk 3)	Treatment	Fast Day	No	16:15	16:00 - 16:59	Evening	38,6		103,2	103,2	19,76		156,94
2019/05/29	CH2206	Male	Three y/o	Treatment (Wk 3)	Treatment	Fast Day	No	16:15	16:00 - 16:59	Evening	38,4		202,2	202,2	19,56		180,50
2019/05/29	CH2205	Male	Three y/o	Treatment (Wk 3)	Treatment	Fast Day	No	16:20	16:00 - 16:59	Evening	38,5	67	24,0	24,0	19,66	563,53	106,55
2019/05/29	CH2206	Male	Three y/o	Treatment (Wk 3)	Treatment	Fast Day	No	16:20	16:00 - 16:59	Evening	38,4	114	78,6	78,6	19,56	603,20	147,46
2019/05/29	CH2205	Male	Three y/o	Treatment (Wk 3)	Treatment	Fast Day	No	16:25	16:00 - 16:59	Evening	38,5	154	38,2	38,2	19,66	623,58	122,50
2019/05/29	CH2206	Male	Three y/o	Treatment (Wk 3)	Treatment	Fast Day	No	16:25	16:00 - 16:59	Evening	38,3	51	46,2	46,2	19,46	541,20	129,06
2019/05/29	CH2205	Male	Three y/o	Treatment (Wk 3)	Treatment	Fast Day	No	16:30	16:00 - 16:59	Evening	38,3		36,2	36,2	19,46		120,65
2019/05/29	CH2206	Male	Three y/o	Treatment (Wk 3)	Treatment	Fast Day	No	16:30	16:00 - 16:59	Evening	38,2	99	45,8	45,8	19,36	593,14	128,76
2019/05/29	CH2205	Male	Three y/o	Treatment (Wk 3)	Treatment	Fast Day	No	16:35	16:00 - 16:59	Evening	38,3		31,0	31,0	19,46		115,32
2019/05/29	CH2206	Male	Three y/o	Treatment (Wk 3)	Treatment	Fast Day	No	16:35	16:00 - 16:59	Evening	38,1	78	47,2	47,2	19,26	575,38	129,80
2019/05/29	CH2205	Male	Three y/o	Treatment (Wk 3)	Treatment	Fast Day	No	16:40	16:00 - 16:59	Evening	38,2	60	29,8	29,8	19,36	554,67	113,97
2019/05/29	CH2206	Male	Three y/o	Treatment (Wk 3)	Treatment	Fast Day	No	16:40	16:00 - 16:59	Evening	38,0	44	39,6	39,6	19,15	528,51	123,74
2019/05/29	CH2205	Male	Three y/o	Treatment (Wk 3)	Treatment	Fast Day	No	16:45	16:00 - 16:59	Evening	38,2		33,0	33,0	19,36		117,47

2019/05/29	CH2206	Male	Three y/o	Treatment (Wk 3)	Treatment	Fast Day	No	16:45	16:00 - 16:59	Evening	37,9	52	34,6	34,6	19,05	542,83	119,10
2019/05/29	CH2205	Male	Three y/o	Treatment (Wk 3)	Treatment	Fast Day	No	16:50	16:00 - 16:59	Evening	38,1		26,2	26,2	19,26		109,55
2019/05/29	CH2206	Male	Three y/o	Treatment (Wk 3)	Treatment	Fast Day	No	16:50	16:00 - 16:59	Evening	37,8	94	63,4	63,4	18,95	589,37	140,00
2019/05/29	CH2205	Male	Three y/o	Treatment (Wk 3)	Treatment	Fast Day	No	16:55	16:00 - 16:59	Evening	38,0	75	141,2	141,2	19,15	572,36	167,90
2019/05/29	CH2206	Male	Three y/o	Treatment (Wk 3)	Treatment	Fast Day	No	16:55	16:00 - 16:59	Evening	38,0	121	103,2	103,2	19,15	607,35	156,94
2019/05/29	CH2205	Male	Three y/o	Treatment (Wk 3)	Treatment	Fast Day	No	17:00	17:00 - 17:59	Evening	37,9		215,6	215,6	19,05		182,76
2019/05/29	CH2206	Male	Three y/o	Treatment (Wk 3)	Treatment	Fast Day	No	17:00	17:00 - 17:59	Evening	38,1	98	175,0	175,0	19,26	592,41	175,43
2019/05/29	CH2205	Male	Three y/o	Treatment (Wk 3)	Treatment	Fast Day	No	17:05	17:00 - 17:59	Evening	37,9	74	36,0	36,0	19,05	571,33	120,46
2019/05/29	CH2206	Male	Three y/o	Treatment (Wk 3)	Treatment	Fast Day	No	17:05	17:00 - 17:59	Evening	38,1		106,2	106,2	19,26		157,94
2019/05/29	CH2205	Male	Three y/o	Treatment (Wk 3)	Treatment	Fast Day	No	17:10	17:00 - 17:59	Evening	37,9		51,2	51,2	19,05		132,61
2019/05/29	CH2206	Male	Three y/o	Treatment (Wk 3)	Treatment	Fast Day	No	17:10	17:00 - 17:59	Evening	38,2		108,2	108,2	19,36		158,59
2019/05/29	CH2205	Male	Three y/o	Treatment (Wk 3)	Treatment	Fast Day	No	17:15	17:00 - 17:59	Evening	38,0	98	128,4	128,4	19,15	592,41	164,57
2019/05/29	CH2206	Male	Three y/o	Treatment (Wk 3)	Treatment	Fast Day	No	17:15	17:00 - 17:59	Evening	38,2	128	76,4	76,4	19,36	611,22	146,48
2019/05/29	CH2205	Male	Three y/o	Treatment (Wk 3)	Treatment	Fast Day	No	17:20	17:00 - 17:59	Evening	38,0		31,4	31,4	19,15		115,76
2019/05/29	CH2206	Male	Three y/o	Treatment (Wk 3)	Treatment	Fast Day	No	17:20	17:00 - 17:59	Evening	38,2	50	54,2	54,2	19,36	539,52	134,57
2019/05/29	CH2205	Male	Three y/o	Treatment (Wk 3)	Treatment	Fast Day	No	17:25	17:00 - 17:59	Evening	37,9		31,6	31,6	19,05		115,98
2019/05/29	CH2206	Male	Three y/o	Treatment (Wk 3)	Treatment	Fast Day	No	17:25	17:00 - 17:59	Evening	38,0	87	100,8	100,8	19,15	583,64	156,12
2019/05/29	CH2205	Male	Three y/o	Treatment (Wk 3)	Treatment	Fast Day	No	17:30	17:00 - 17:59	Evening	38,0	78	26,8	26,8	19,15	575,38	110,33
2019/05/29	CH2206	Male	Three y/o	Treatment (Wk 3)	Treatment	Fast Day	No	17:30	17:00 - 17:59	Evening	38,1		59,0	59,0	19,26		137,51
2019/05/29	CH2205	Male	Three y/o	Treatment (Wk 3)	Treatment	Fast Day	No	17:35	17:00 - 17:59	Evening	38,0	46	13,2	13,2	19,15	532,38	86,17
2019/05/29	CH2206	Male	Three y/o	Treatment (Wk 3)	Treatment	Fast Day	No	17:35	17:00 - 17:59	Evening	38,1	46	63,4	63,4	19,26	532,38	140,00
2019/05/29	CH2205	Male	Three y/o	Treatment (Wk 3)	Treatment	Fast Day	No	17:40	17:00 - 17:59	Evening	37,9		2,0	2,0	19,05		22,86
2019/05/29	CH2206	Male	Three y/o	Treatment (Wk 3)	Treatment	Fast Day	No	17:40	17:00 - 17:59	Evening	38,0	80	60,0	60,0	19,15	577,31	138,09
2019/05/29	CH2205	Male	Three y/o	Treatment (Wk 3)	Treatment	Fast Day	No	17:45	17:00 - 17:59	Evening	38,0	59	5,4	5,4	19,15	553,30	55,99
2019/05/29	CH2206	Male	Three y/o	Treatment (Wk 3)	Treatment	Fast Day	No	17:45	17:00 - 17:59	Evening	37,9	152	63,6	63,6	19,05	622,73	140,11
2019/05/29	CH2205	Male	Three y/o	Treatment (Wk 3)	Treatment	Fast Day	No	17:50	17:00 - 17:59	Evening	38,1		6,2	6,2	19,26		60,63
2019/05/29	CH2206	Male	Three y/o	Treatment (Wk 3)	Treatment	Fast Day	No	17:50	17:00 - 17:59	Evening	37,7	51	53,0	53,0	18,85	541,20	133,80
2019/05/29	CH2205	Male	Three y/o	Treatment (Wk 3)	Treatment	Fast Day	No	17:55	17:00 - 17:59	Evening	38,1	44	3,6	3,6	19,26	528,51	42,42
2019/05/29	CH2206	Male	Three y/o	Treatment (Wk 3)	Treatment	Fast Day	No	17:55	17:00 - 17:59	Evening	37,5	45	57,6	57,6	18,65	530,47	136,68
2019/05/29	CH2205	Male	Three y/o	Treatment (Wk 3)	Treatment	Fast Day	No	18:00	18:00 - 18:59	Evening	38,1	42	2,2	2,2	19,26	524,42	26,02
2019/05/29	CH2206	Male	Three y/o	Treatment (Wk 3)	Treatment	Fast Day	No	18:00	18:00 - 18:59	Evening	37,4	148	49,8	49,8	18,55	620,97	131,65
2019/05/29	CH2205	Male	Three y/o	Treatment (Wk 3)	Treatment	Fast Day	No	18:05	18:00 - 18:59	Evening	38,1	61	2,2	2,2	19,26	556,01	26,02
2019/05/29	CH2206	Male	Three y/o	Treatment (Wk 3)	Treatment	Fast Day	No	18:05	18:00 - 18:59	Evening	37,4	92	54,0	54,0	18,55	587,78	134,45
2019/05/29	CH2205	Male	Three y/o	Treatment (Wk 3)	Treatment	Fast Day	No	18:10	18:00 - 18:59	Evening	38,1	38	5,6	5,6	19,26	515,48	57,21
2019/05/29	CH2206	Male	Three y/o	Treatment (Wk 3)	Treatment	Fast Day	No	18:10	18:00 - 18:59	Evening	37,4	46	46,2	46,2	18,55	532,38	129,06
2019/05/29	CH2205	Male	Three y/o	Treatment (Wk 3)	Treatment	Fast Day	No	18:15	18:00 - 18:59	Evening	38,1	169	16,0	16,0	19,26	629,60	92,71
2019/05/29	CH2206	Male	Three y/o	Treatment (Wk 3)	Treatment	Fast Day	No	18:15	18:00 - 18:59	Evening	37,3	47	36,0	36,0	18,46	534,23	120,46
2019/05/29	CH2205	Male	Three y/o	Treatment (Wk 3)	Treatment	Fast Day	No	18:20	18:00 - 18:59	Evening	37,9		23,4	23,4	19,05		105,68
2019/05/29	CH2206	Male	Three y/o	Treatment (Wk 3)	Treatment	Fast Day	No	18:20	18:00 - 18:59	Evening	37,3	50	45,8	45,8	18,46	539,52	128,76
2019/05/29	CH2205	Male	Three y/o	Treatment (Wk 3)	Treatment	Fast Day	No	18:25	18:00 - 18:59	Evening	37,9	41	21,6	21,6	19,05	522,29	102,94
2019/05/29	CH2206	Male	Three y/o	Treatment (Wk 3)	Treatment	Fast Day	No	18:25	18:00 - 18:59	Evening	37,2	39	43,0	43,0	18,36	517,82	126,58
2019/05/29	CH2205	Male	Three y/o	Treatment (Wk 3)	Treatment	Fast Day	No	18:30	18:00 - 18:59	Evening	37,9	41	27,0	27,0	19,05	522,29	110,58
2019/05/29	CH2206	Male	Three y/o	Treatment (Wk 3)	Treatment	Fast Day	No	18:30	18:00 - 18:59	Evening	37,2	50	37,0	37,0	18,36	539,52	121,40
2019/05/29	CH2205	Male	Three y/o	Treatment (Wk 3)	Treatment	Fast Day	No	18:35	18:00 - 18:59	Evening	37,9	40	25,6	25,6	19,05	520,09	108,76
2019/05/29	CH2206	Male	Three y/o	Treatment (Wk 3)	Treatment	Fast Day	No	18:35	18:00 - 18:59	Evening	37,3	42	39,2	39,2	18,46	524,42	123,39
2019/05/29	CH2205	Male	Three y/o	Treatment (Wk 3)	Treatment	Fast Day	No	18:40	18:00 - 18:59	Evening	37,9	45	20,4	20,4	19,05	530,47	100,99
2019/05/29	CH2206	Male	Three y/o	Treatment (Wk 3)	Treatment	Fast Day	No	18:40	18:00 - 18:59	Evening	37,4	53	37,0	37,0	18,55	544,43	121,40
2019/05/29	CH2205	Male	Three y/o	Treatment (Wk 3)	Treatment	Fast Day	No	18:45	18:00 - 18:59	Evening	37,9	50	22,8	22,8	19,05	539,52	104,79
2019/05/29	CH2206	Male	Three y/o	Treatment (Wk 3)	Treatment	Fast Day	No	18:45	18:00 - 18:59	Evening	37,5	48	43,8	43,8	18,65	536,04	127,22
2019/05/29	CH2205	Male	Three y/o	Treatment (Wk 3)	Treatment	Fast Day	No	18:50	18:00 - 18:59	Evening	37,7	44	14,8	14,8	18,85	528,51	90,05
2019/05/29	CH2206	Male	Three y/o	Treatment (Wk 3)	Treatment	Fast Day	No	18:50	18:00 - 18:59	Evening	37,5	80	38,8	38,8	18,65	577,31	123,04
2019/05/29	CH2205	Male	Three y/o	Treatment (Wk 3)	Treatment	Fast Day	No	18:55	18:00 - 18:59	Evening	37,6	47	14,2	14,2	18,75	534,23	88,65
2019/05/29	CH2206	Male	Three y/o	Treatment (Wk 3)	Treatment	Fast Day	No	18:55	18:00 - 18:59	Evening	37,5	52	40,8	40,8	18,65	542,83	124,77
2019/05/29	CH2205	Male	Three y/o	Treatment (Wk 3)	Treatment	Fast Day	No	19:00	19:00 - 19:59	Evening	37,6	39	14,0	14,0	18,75	517,82	88,17
2019/05/29	CH2206	Male	Three y/o	Treatment (Wk 3)	Treatment	Fast Day	No	19:00	19:00 - 19:59	Evening	37,4	40	40,4	40,4	18,55	520,09	124,43
2019/05/29	CH2205	Male	Three y/o	Treatment (Wk 3)	Treatment	Fast Day	No	19:05	19:00 - 19:59	Evening	37,6	41	14,8	14,8	18,75	522,29	90,05
2019/05/29	CH2206	Male	Three y/o	Treatment (Wk 3)	Treatment	Fast Day	No	19:05	19:00 - 19:59	Evening	37,4	47	39,2	39,2	18,55	534,23	123,39
2019/05/29	CH2205	Male	Three y/o	Treatment (Wk 3)	Treatment	Fast Day	No	19:10	19:00 - 19:59	Evening	37,5	42	18,4	18,4	18,65	524,42	97,47
2019/05/29	CH2206	Male	Three y/o	Treatment (Wk 3)	Treatment	Fast Day	No	19:10	19:00 - 19:59	Evening	37,4	49	38,2	38,2	18,55	537,80	122,50
2019/05/29	CH2205	Male	Three y/o	Treatment (Wk 3)	Treatment	Fast Day	No	19:15	19:00 - 19:59	Evening	37,5	49	14,8	14,8	18,65	537,80	90,05
2019/05/29	CH2206	Male	Three y/o	Treatment (Wk 3)	Treatment	Fast Day	No	19:15	19:00 - 19:59	Evening	37,4	42	45,0	45,0	18,55	524,42	128,15
2019/05/29	CH2205	Male	Three y/o	Treatment (Wk 3)	Treatment	Fast Day	No	19:20	19:00 - 19:59	Evening	37,5	47	9,2	9,2	18,65	534,23	73,94
2019/05/29	CH2206	Male	Three y/o	Treatment (Wk 3)	Treatment	Fast Day	No	19:20	19:00 - 19:59	Evening	37,4	41	50,4	50,4	18,55	522,29	132,06
2019/05/29	CH2205	Male	Three y/o	Treatment (Wk 3)	Treatment	Fast Day	No	19:25	19:00 - 19:59	Evening	37,5	60	21,0	21,0	18,65	554,67	101,98
2019/05/29	CH2206	Male	Three y/o	Treatment (Wk 3)	Treatment	Fast Day	No	19:25	19:00 - 19:59	Evening	37,4	50	48,2	48,2	18,55	539,52	130,52
2019/05/29	CH2205	Male	Three y/o	Treatment (Wk 3)	Treatment	Fast Day	No	19:30	19:00 - 19:59	Evening	37,6	42	18,6	18,6	18,75	524,42	97,84
2019/05/29	CH2206	Male	Three y/o	Treatment (Wk 3)	Treatment	Fast Day	No	19:30	19:00 - 19:59	Evening	37,4	43	47,2	47,2	18,55	526,50	129,80
2019/05/29	CH2205	Male	Three y/o	Treatment (Wk 3)	Treatment	Fast Day	No	19:35	19:00 - 19:59	Evening	37,6	50	22,8	22,8	18,75	539,52	104,79
2019/05/29	CH2206	Male	Three y/o	Treatment (Wk 3)	Treatment	Fast Day	No	19:35	19:00 - 19:59	Evening	37,4	52	45,4	45,4	18,55	542,83	128,45
2019/05/29	CH2205	Male	Three y/o	Treatment (Wk 3)	Treatment	Fast Day	No	19:40	19:00 - 19:59	Evening	37,7	52	22,4	22,4	18,85	542,83	104,19

2019/05/29	CH2206	Male	Three y/o	Treatment (Wk 3)	Treatment	Fast Day	No	19:40	19:00 - 19:59	Evening	37,4	38	52,0	52,0	18,55	515,48	133,14
2019/05/29	CH2205	Male	Three y/o	Treatment (Wk 3)	Treatment	Fast Day	No	19:45	19:00 - 19:59	Evening	37,6	47	18,2	18,2	18,75	534,23	97,10
2019/05/29	CH2206	Male	Three y/o	Treatment (Wk 3)	Treatment	Fast Day	No	19:45	19:00 - 19:59	Evening	37,4	43	48,8	48,8	18,55	526,50	130,95
2019/05/29	CH2205	Male	Three y/o	Treatment (Wk 3)	Treatment	Fast Day	No	19:50	19:00 - 19:59	Evening	37,6	41	16,8	16,8	18,75	522,29	94,37
2019/05/29	CH2206	Male	Three y/o	Treatment (Wk 3)	Treatment	Fast Day	No	19:50	19:00 - 19:59	Evening	37,3	68	45,6	45,6	18,46	564,71	128,61
2019/05/29	CH2205	Male	Three y/o	Treatment (Wk 3)	Treatment	Fast Day	No	19:55	19:00 - 19:59	Evening	37,6	69	18,2	18,2	18,75	565,86	97,10
2019/05/29	CH2206	Male	Three y/o	Treatment (Wk 3)	Treatment	Fast Day	No	19:55	19:00 - 19:59	Evening	37,4	50	46,8	46,8	18,55	539,52	129,50
2019/05/29	CH2205	Male	Three y/o	Treatment (Wk 3)	Treatment	Fast Day	No	20:00	20:00 - 20:59	Night	37,6	60	19,6	19,6	18,75	554,67	99,63
2019/05/29	CH2206	Male	Three y/o	Treatment (Wk 3)	Treatment	Fast Day	No	20:00	20:00 - 20:59	Night	37,3	41	47,0	47,0	18,46	522,29	129,65
2019/05/29	CH2205	Male	Three y/o	Treatment (Wk 3)	Treatment	Fast Day	No	20:05	20:00 - 20:59	Night	37,6	42	21,8	21,8	18,75	524,42	103,26
2019/05/29	CH2206	Male	Three y/o	Treatment (Wk 3)	Treatment	Fast Day	No	20:05	20:00 - 20:59	Night	37,3	42	44,8	44,8	18,46	524,42	127,99
2019/05/29	CH2205	Male	Three y/o	Treatment (Wk 3)	Treatment	Fast Day	No	20:10	20:00 - 20:59	Night	37,6	53	16,8	16,8	18,75	544,43	94,37
2019/05/29	CH2206	Male	Three y/o	Treatment (Wk 3)	Treatment	Fast Day	No	20:10	20:00 - 20:59	Night	37,3	44	45,8	45,8	18,46	528,51	128,76
2019/05/29	CH2205	Male	Three y/o	Treatment (Wk 3)	Treatment	Fast Day	No	20:15	20:00 - 20:59	Night	37,5	74	23,6	23,6	18,65	571,33	105,97
2019/05/29	CH2206	Male	Three y/o	Treatment (Wk 3)	Treatment	Fast Day	No	20:15	20:00 - 20:59	Night	37,3	45	47,8	47,8	18,46	530,47	130,23
2019/05/29	CH2205	Male	Three y/o	Treatment (Wk 3)	Treatment	Fast Day	No	20:20	20:00 - 20:59	Night	37,5	38	17,6	17,6	18,65	515,48	95,95
2019/05/29	CH2206	Male	Three y/o	Treatment (Wk 3)	Treatment	Fast Day	No	20:20	20:00 - 20:59	Night	37,3	35	47,4	47,4	18,46	507,97	129,94
2019/05/29	CH2205	Male	Three y/o	Treatment (Wk 3)	Treatment	Fast Day	No	20:25	20:00 - 20:59	Night	37,5	56	20,4	20,4	18,65	549,01	100,99
2019/05/29	CH2206	Male	Three y/o	Treatment (Wk 3)	Treatment	Fast Day	No	20:25	20:00 - 20:59	Night	37,3	46	48,2	48,2	18,46	532,38	130,52
2019/05/29	CH2205	Male	Three y/o	Treatment (Wk 3)	Treatment	Fast Day	No	20:30	20:00 - 20:59	Night	37,5	40	17,2	17,2	18,65	520,09	95,17
2019/05/29	CH2206	Male	Three y/o	Treatment (Wk 3)	Treatment	Fast Day	No	20:30	20:00 - 20:59	Night	37,2	46	46,4	46,4	18,36	532,38	129,21
2019/05/29	CH2205	Male	Three y/o	Treatment (Wk 3)	Treatment	Fast Day	No	20:35	20:00 - 20:59	Night	37,5	55	22,2	22,2	18,65	547,52	103,88
2019/05/29	CH2206	Male	Three y/o	Treatment (Wk 3)	Treatment	Fast Day	No	20:35	20:00 - 20:59	Night	37,2	39	48,2	48,2	18,36	517,82	130,52
2019/05/29	CH2205	Male	Three y/o	Treatment (Wk 3)	Treatment	Fast Day	No	20:40	20:00 - 20:59	Night	37,5	39	22,6	22,6	18,65	517,82	104,49
2019/05/29	CH2206	Male	Three y/o	Treatment (Wk 3)	Treatment	Fast Day	No	20:40	20:00 - 20:59	Night	37,2	46	43,0	43,0	18,36	532,38	126,58
2019/05/29	CH2205	Male	Three y/o	Treatment (Wk 3)	Treatment	Fast Day	No	20:45	20:00 - 20:59	Night	37,5	40	24,0	24,0	18,65	520,09	106,55
2019/05/29	CH2206	Male	Three y/o	Treatment (Wk 3)	Treatment	Fast Day	No	20:45	20:00 - 20:59	Night	37,2	37	44,2	44,2	18,36	513,06	127,53
2019/05/29	CH2205	Male	Three y/o	Treatment (Wk 3)	Treatment	Fast Day	No	20:50	20:00 - 20:59	Night	37,5	43	24,8	24,8	18,65	526,50	107,67
2019/05/29	CH2206	Male	Three y/o	Treatment (Wk 3)	Treatment	Fast Day	No	20:50	20:00 - 20:59	Night	37,1	45	36,6	36,6	18,26	530,47	121,03
2019/05/29	CH2205	Male	Three y/o	Treatment (Wk 3)	Treatment	Fast Day	No	20:55	20:00 - 20:59	Night	37,4	56	12,8	12,8	18,55	549,01	85,12
2019/05/29	CH2206	Male	Three y/o	Treatment (Wk 3)	Treatment	Fast Day	No	20:55	20:00 - 20:59	Night	37,1	46	41,6	41,6	18,26	532,38	125,44
2019/05/29	CH2205	Male	Three y/o	Treatment (Wk 3)	Treatment	Fast Day	No	21:00	21:00 - 21:59	Night	37,4		6,0	6,0	18,55		59,53
2019/05/29	CH2206	Male	Three y/o	Treatment (Wk 3)	Treatment	Fast Day	No	21:00	21:00 - 21:59	Night	37,1	43	39,4	39,4	18,26	526,50	123,57
2019/05/29	CH2205	Male	Three y/o	Treatment (Wk 3)	Treatment	Fast Day	No	21:05	21:00 - 21:59	Night	37,4		7,2	7,2	18,55		65,66
2019/05/29	CH2206	Male	Three y/o	Treatment (Wk 3)	Treatment	Fast Day	No	21:05	21:00 - 21:59	Night	37,2	43	67,0	67,0	18,36	526,50	141,92
2019/05/29	CH2205	Male	Three y/o	Treatment (Wk 3)	Treatment	Fast Day	No	21:10	21:00 - 21:59	Night	37,3		8,2	8,2	18,46		70,05
2019/05/29	CH2206	Male	Three y/o	Treatment (Wk 3)	Treatment	Fast Day	No	21:10	21:00 - 21:59	Night	37,2	39	66,0	66,0	18,36	517,82	141,40
2019/05/29	CH2205	Male	Three y/o	Treatment (Wk 3)	Treatment	Fast Day	No	21:15	21:00 - 21:59	Night	37,3		8,2	8,2	18,46		70,05
2019/05/29	CH2206	Male	Three y/o	Treatment (Wk 3)	Treatment	Fast Day	No	21:15	21:00 - 21:59	Night	37,2	46	63,0	63,0	18,36	532,38	139,78
2019/05/29	CH2205	Male	Three y/o	Treatment (Wk 3)	Treatment	Fast Day	No	21:20	21:00 - 21:59	Night	37,3	62	7,0	7,0	18,46	557,33	64,72
2019/05/29	CH2206	Male	Three y/o	Treatment (Wk 3)	Treatment	Fast Day	No	21:20	21:00 - 21:59	Night	37,2	47	67,8	67,8	18,36	534,23	142,33
2019/05/29	CH2205	Male	Three y/o	Treatment (Wk 3)	Treatment	Fast Day	No	21:25	21:00 - 21:59	Night	37,3		9,0	9,0	18,46		73,19
2019/05/29	CH2206	Male	Three y/o	Treatment (Wk 3)	Treatment	Fast Day	No	21:25	21:00 - 21:59	Night	37,3	41	67,8	67,8	18,46	522,29	142,33
2019/05/29	CH2205	Male	Three y/o	Treatment (Wk 3)	Treatment	Fast Day	No	21:30	21:00 - 21:59	Night	37,3		7,0	7,0	18,46		64,72
2019/05/29	CH2206	Male	Three y/o	Treatment (Wk 3)	Treatment	Fast Day	No	21:30	21:00 - 21:59	Night	37,3	67	64,4	64,4	18,46	563,53	140,55
2019/05/29	CH2205	Male	Three y/o	Treatment (Wk 3)	Treatment	Fast Day	No	21:35	21:00 - 21:59	Night	37,3		10,6	10,6	18,46		78,73
2019/05/29	CH2206	Male	Three y/o	Treatment (Wk 3)	Treatment	Fast Day	No	21:35	21:00 - 21:59	Night	37,3	39	61,2	61,2	18,46	517,82	138,78
2019/05/29	CH2205	Male	Three y/o	Treatment (Wk 3)	Treatment	Fast Day	No	21:40	21:00 - 21:59	Night	37,3		11,4	11,4	18,46		81,19
2019/05/29	CH2206	Male	Three y/o	Treatment (Wk 3)	Treatment	Fast Day	No	21:40	21:00 - 21:59	Night	37,3	48	64,6	64,6	18,46	536,04	140,65
2019/05/29	CH2205	Male	Three y/o	Treatment (Wk 3)	Treatment	Fast Day	No	21:45	21:00 - 21:59	Night	37,3		11,4	11,4	18,46		81,19
2019/05/29	CH2206	Male	Three y/o	Treatment (Wk 3)	Treatment	Fast Day	No	21:45	21:00 - 21:59	Night	37,3	44	56,8	56,8	18,46	528,51	136,20
2019/05/29	CH2205	Male	Three y/o	Treatment (Wk 3)	Treatment	Fast Day	No	21:50	21:00 - 21:59	Night	37,3		10,4	10,4	18,46		78,08
2019/05/29	CH2206	Male	Three y/o	Treatment (Wk 3)	Treatment	Fast Day	No	21:50	21:00 - 21:59	Night	37,3	44	66,4	66,4	18,46	528,51	141,61
2019/05/29	CH2205	Male	Three y/o	Treatment (Wk 3)	Treatment	Fast Day	No	21:55	21:00 - 21:59	Night	37,3		12,6	12,6	18,46		84,59
2019/05/29	CH2206	Male	Three y/o	Treatment (Wk 3)	Treatment	Fast Day	No	21:55	21:00 - 21:59	Night	37,4	41	64,6	64,6	18,55	522,29	140,65
2019/05/29	CH2205	Male	Three y/o	Treatment (Wk 3)	Treatment	Fast Day	No	22:00	22:00 - 22:59	Night	37,3	40	8,8	8,8	18,46	520,09	72,43
2019/05/29	CH2206	Male	Three y/o	Treatment (Wk 3)	Treatment	Fast Day	No	22:00	22:00 - 22:59	Night	37,4	51	61,4	61,4	18,55	541,20	138,89
2019/05/29	CH2205	Male	Three y/o	Treatment (Wk 3)	Treatment	Fast Day	No	22:05	22:00 - 22:59	Night	37,3		5,8	5,8	18,46		58,39
2019/05/29	CH2206	Male	Three y/o	Treatment (Wk 3)	Treatment	Fast Day	No	22:05	22:00 - 22:59	Night	37,4	49	63,4	63,4	18,55	537,80	140,00
2019/05/29	CH2205	Male	Three y/o	Treatment (Wk 3)	Treatment	Fast Day	No	22:10	22:00 - 22:59	Night	37,3	75	3,8	3,8	18,46	572,36	44,22
2019/05/29	CH2206	Male	Three y/o	Treatment (Wk 3)	Treatment	Fast Day	No	22:10	22:00 - 22:59	Night	37,4	35	69,0	69,0	18,55	507,97	142,94
2019/05/29	CH2205	Male	Three y/o	Treatment (Wk 3)	Treatment	Fast Day	No	22:15	22:00 - 22:59	Night	37,3	45	5,8	5,8	18,46	530,47	58,39
2019/05/29	CH2206	Male	Three y/o	Treatment (Wk 3)	Treatment	Fast Day	No	22:15	22:00 - 22:59	Night	37,4	47	68,2	68,2	18,55	534,23	142,53
2019/05/29	CH2205	Male	Three y/o	Treatment (Wk 3)	Treatment	Fast Day	No	22:20	22:00 - 22:59	Night	37,2	76	5,4	5,4	18,36	573,39	55,99
2019/05/29	CH2206	Male	Three y/o	Treatment (Wk 3)	Treatment	Fast Day	No	22:20	22:00 - 22:59	Night	37,3	46	66,6	66,6	18,46	532,38	141,71
2019/05/29	CH2205	Male	Three y/o	Treatment (Wk 3)	Treatment	Fast Day	No	22:25	22:00 - 22:59	Night	37,3		5,4	5,4	18,46		55,99
2019/05/29	CH2206	Male	Three y/o	Treatment (Wk 3)	Treatment	Fast Day	No	22:25	22:00 - 22:59	Night	37,3	41	49,0	49,0	18,46	522,29	131,09
2019/05/29	CH2205	Male	Three y/o	Treatment (Wk 3)	Treatment	Fast Day	No	22:30	22:00 - 22:59	Night	37,3	51	11,2	11,2	18,46	541,20	80,59
2019/05/29	CH2206	Male	Three y/o	Treatment (Wk 3)	Treatment	Fast Day	No	22:30	22:00 - 22:59	Night	37,3	41	57,6	57,6	18,46	522,29	136,68
2019/05/29	CH2205	Male	Three y/o	Treatment (Wk 3)	Treatment	Fast Day	No	22:35	22:00 - 22:59	Night	37,3	45	5,4	5,4	18,46	530,47	55,99

	2019/05/29	CH2206	Male	Three y/o	Treatment (Wk 3)	Treatment	Fast Day	No	22:35	22:00 - 22:59	Night	37,2	47	63,6	63,6	18,36	534,23	140,11
	2019/05/29	CH2205	Male	Three y/o	Treatment (Wk 3)	Treatment	Fast Day	No	22:40	22:00 - 22:59	Night	37,3	38	7,8	7,8	18,46	515,48	68,36
	2019/05/29	CH2206	Male	Three y/o	Treatment (Wk 3)	Treatment	Fast Day	No	22:40	22:00 - 22:59	Night	37,1	39	61,4	61,4	18,26	517,82	138,89
	2019/05/29	CH2205	Male	Three y/o	Treatment (Wk 3)	Treatment	Fast Day	No	22:45	22:00 - 22:59	Night	37,2	41	8,2	8,2	18,36	522,29	70,05
	2019/05/29	CH2206	Male	Three y/o	Treatment (Wk 3)	Treatment	Fast Day	No	22:45	22:00 - 22:59	Night	37,0	47	58,2	58,2	18,16	534,23	137,04
	2019/05/29	CH2205	Male	Three y/o	Treatment (Wk 3)	Treatment	Fast Day	No	22:50	22:00 - 22:59	Night	37,2	44	18,4	18,4	18,36	528,51	97,47
	2019/05/29	CH2206	Male	Three y/o	Treatment (Wk 3)	Treatment	Fast Day	No	22:50	22:00 - 22:59	Night	36,9	36	58,4	58,4	18,06	510,56	137,16
	2019/05/29	CH2205	Male	Three y/o	Treatment (Wk 3)	Treatment	Fast Day	No	22:55	22:00 - 22:59	Night	37,3	42	21,6	21,6	18,46	524,42	102,94
	2019/05/29	CH2206	Male	Three y/o	Treatment (Wk 3)	Treatment	Fast Day	No	22:55	22:00 - 22:59	Night	36,9	44	58,8	58,8	18,06	528,51	137,39
	2019/05/29	CH2205	Male	Three y/o	Treatment (Wk 3)	Treatment	Fast Day	No	23:00	23:00 - 23:59	Night	37,2	76	23,0	23,0	18,36	573,39	105,09
	2019/05/29	CH2206	Male	Three y/o	Treatment (Wk 3)	Treatment	Fast Day	No	23:00	23:00 - 23:59	Night	36,8	48	61,8	61,8	17,96	536,04	139,12
	2019/05/29	CH2205	Male	Three y/o	Treatment (Wk 3)	Treatment	Fast Day	No	23:05	23:00 - 23:59	Night	37,3	38	22,8	22,8	18,46	515,48	104,79
	2019/05/29	CH2206	Male	Three y/o	Treatment (Wk 3)	Treatment	Fast Day	No	23:05	23:00 - 23:59	Night	36,8	45	57,4	57,4	17,96	530,47	136,56
	2019/05/29	CH2205	Male	Three y/o	Treatment (Wk 3)	Treatment	Fast Day	No	23:10	23:00 - 23:59	Night	37,3	42	21,0	21,0	18,46	524,42	101,98
	2019/05/29	CH2206	Male	Three y/o	Treatment (Wk 3)	Treatment	Fast Day	No	23:10	23:00 - 23:59	Night	36,9	44	59,6	59,6	18,06	528,51	137,86
	2019/05/29	CH2205	Male	Three y/o	Treatment (Wk 3)	Treatment	Fast Day	No	23:15	23:00 - 23:59	Night	37,3	71	19,4	19,4	18,46	568,10	99,28
	2019/05/29	CH2206	Male	Three y/o	Treatment (Wk 3)	Treatment	Fast Day	No	23:15	23:00 - 23:59	Night	37,0	38	59,4	59,4	18,16	515,48	137,75
	2019/05/29	CH2205	Male	Three y/o	Treatment (Wk 3)	Treatment	Fast Day	No	23:20	23:00 - 23:59	Night	37,3	46	20,0	20,0	18,46	532,38	100,31
	2019/05/29	CH2206	Male	Three y/o	Treatment (Wk 3)	Treatment	Fast Day	No	23:20	23:00 - 23:59	Night	36,9	44	58,8	58,8	18,06	528,51	137,39
	2019/05/29	CH2205	Male	Three y/o	Treatment (Wk 3)	Treatment	Fast Day	No	23:25	23:00 - 23:59	Night	37,4	65	18,2	18,2	18,55	561,12	97,10
	2019/05/29	CH2206	Male	Three y/o	Treatment (Wk 3)	Treatment	Fast Day	No	23:25	23:00 - 23:59	Night	36,9	50	64,8	64,8	18,06	539,52	140,76
	2019/05/29	CH2205	Male	Three y/o	Treatment (Wk 3)	Treatment	Fast Day	No	23:30	23:00 - 23:59	Night	37,4	50	14,0	14,0	18,55	539,52	88,17
	2019/05/29	CH2206	Male	Three y/o	Treatment (Wk 3)	Treatment	Fast Day	No	23:30	23:00 - 23:59	Night	36,8	41	57,0	57,0	17,96	522,29	136,32
	2019/05/29	CH2205	Male	Three y/o	Treatment (Wk 3)	Treatment	Fast Day	No	23:35	23:00 - 23:59	Night	37,4	41	15,8	15,8	18,55	522,29	92,28
	2019/05/29	CH2206	Male	Three y/o	Treatment (Wk 3)	Treatment	Fast Day	No	23:35	23:00 - 23:59	Night	36,8	40	56,2	56,2	17,96	520,09	135,83
	2019/05/29	CH2205	Male	Three y/o	Treatment (Wk 3)	Treatment	Fast Day	No	23:40	23:00 - 23:59	Night	37,3	39	13,6	13,6	18,46	517,82	87,18
	2019/05/29	CH2206	Male	Three y/o	Treatment (Wk 3)	Treatment	Fast Day	No	23:40	23:00 - 23:59	Night	36,6	43	65,6	65,6	17,77	526,50	141,19
	2019/05/29	CH2205	Male	Three y/o	Treatment (Wk 3)	Treatment	Fast Day	No	23:45	23:00 - 23:59	Night	37,3	51	11,2	11,2	18,46	541,20	80,59
	2019/05/29	CH2206	Male	Three y/o	Treatment (Wk 3)	Treatment	Fast Day	No	23:45	23:00 - 23:59	Night	36,6	102	18,2	18,2	17,77	595,30	97,10
	2019/05/29	CH2205	Male	Three y/o	Treatment (Wk 3)	Treatment	Fast Day	No	23:50	23:00 - 23:59	Night	37,3	41	21,4	21,4	18,46	522,29	102,63
	2019/05/29	CH2206	Male	Three y/o	Treatment (Wk 3)	Treatment	Fast Day	No	23:50	23:00 - 23:59	Night	36,8	40	9,6	9,6	17,96	520,09	75,37
	2019/05/29	CH2205	Male	Three y/o	Treatment (Wk 3)	Treatment	Fast Day	No	23:55	23:00 - 23:59	Night	37,3	40	18,8	18,8	18,46	520,09	98,20
	2019/05/29	CH2206	Male	Three y/o	Treatment (Wk 3)	Treatment	Fast Day	No	23:55	23:00 - 23:59	Night	36,9	42	12,8	12,8	18,06	524,42	85,12
16	2019/05/30	CH2205	Male	Three y/o	Treatment (Wk 3)	Treatment	Feed Day	No	00:00	00:00 - 00:59	Early Morning	37,3	53	20,2	20,2	18,46	544,43	100,65
	2019/05/30	CH2206	Male	Three y/o	Treatment (Wk 3)	Treatment	Feed Day	No	00:00	00:00 - 00:59	Early Morning	37,0	61	25,2	25,2	18,16	556,01	108,22
	2019/05/30	CH2205	Male	Three y/o	Treatment (Wk 3)	Treatment	Feed Day	No	00:05	00:00 - 00:59	Early Morning	37,3	44	15,2	15,2	18,46	528,51	90,96
	2019/05/30	CH2206	Male	Three y/o	Treatment (Wk 3)	Treatment	Feed Day	No	00:05	00:00 - 00:59	Early Morning	37,0	76	25,6	25,6	18,16	573,39	108,76
	2019/05/30	CH2205	Male	Three y/o	Treatment (Wk 3)	Treatment	Feed Day	No	00:10	00:00 - 00:59	Early Morning	37,3	51	11,6	11,6	18,46	541,20	81,78
	2019/05/30	CH2206	Male	Three y/o	Treatment (Wk 3)	Treatment	Feed Day	No	00:10	00:00 - 00:59	Early Morning	37,1	70	16,0	16,0	18,26	566,99	92,71
	2019/05/30	CH2205	Male	Three y/o	Treatment (Wk 3)	Treatment	Feed Day	No	00:15	00:00 - 00:59	Early Morning	37,3	51	13,2	13,2	18,46	541,20	86,17
	2019/05/30	CH2206	Male	Three y/o	Treatment (Wk 3)	Treatment	Feed Day	No	00:15	00:00 - 00:59	Early Morning	37,1	45	5,6	5,6	18,26	530,47	57,21
	2019/05/30	CH2205	Male	Three y/o	Treatment (Wk 3)	Treatment	Feed Day	No	00:20	00:00 - 00:59	Early Morning	37,2	44	8,8	8,8	18,36	528,51	72,43
	2019/05/30	CH2206	Male	Three y/o	Treatment (Wk 3)	Treatment	Feed Day	No	00:20	00:00 - 00:59	Early Morning	37,3	44	3,6	3,6	18,46	528,51	42,42
	2019/05/30	CH2205	Male	Three y/o	Treatment (Wk 3)	Treatment	Feed Day	No	00:25	00:00 - 00:59	Early Morning	37,2	137	40,6	40,6	18,36	615,83	124,60
	2019/05/30	CH2206	Male	Three y/o	Treatment (Wk 3)	Treatment	Feed Day	No	00:25	00:00 - 00:59	Early Morning	37,3	169	15,0	15,0	18,46	629,60	90,51
	2019/05/30	CH2205	Male	Three y/o	Treatment (Wk 3)	Treatment	Feed Day	No	00:30	00:00 - 00:59	Early Morning	37,3		22,2	22,2	18,46		103,88
	2019/05/30	CH2206	Male	Three y/o	Treatment (Wk 3)	Treatment	Feed Day	No	00:30	00:00 - 00:59	Early Morning	37,3	48	26,2	26,2	18,46	536,04	109,55
	2019/05/30	CH2205	Male	Three y/o	Treatment (Wk 3)	Treatment	Feed Day	No	00:35	00:00 - 00:59	Early Morning	37,3		18,0	18,0	18,46		96,72
	2019/05/30	CH2206	Male	Three y/o	Treatment (Wk 3)	Treatment	Feed Day	No	00:35	00:00 - 00:59	Early Morning	37,3	49	28,6	28,6	18,46	537,80	112,56
	2019/05/30	CH2205	Male	Three y/o	Treatment (Wk 3)	Treatment	Feed Day	No	00:40	00:00 - 00:59	Early Morning	37,3		27,6	27,6	18,46		111,33
	2019/05/30	CH2206	Male	Three y/o	Treatment (Wk 3)	Treatment	Feed Day	No	00:40	00:00 - 00:59	Early Morning	37,3	57	30,6	30,6	18,46	550,47	114,87
	2019/05/30	CH2205	Male	Three y/o	Treatment (Wk 3)	Treatment	Feed Day	No	00:45	00:00 - 00:59	Early Morning	37,2		25,2	25,2	18,36		108,22
	2019/05/30	CH2206	Male	Three y/o	Treatment (Wk 3)	Treatment	Feed Day	No	00:45	00:00 - 00:59	Early Morning	37,3	46	27,0	27,0	18,46	532,38	110,58
	2019/05/30	CH2205	Male	Three y/o	Treatment (Wk 3)	Treatment	Feed Day	No	00:50	00:00 - 00:59	Early Morning	37,2		21,6	21,6	18,36		102,94
	2019/05/30	CH2206	Male	Three y/o	Treatment (Wk 3)	Treatment	Feed Day	No	00:50	00:00 - 00:59	Early Morning	37,3	39	19,4	19,4	18,46	517,82	99,28
	2019/05/30	CH2205	Male	Three y/o	Treatment (Wk 3)	Treatment	Feed Day	No	00:55	00:00 - 00:59	Early Morning	37,2		20,0	20,0	18,36		100,31
	2019/05/30	CH2206	Male	Three y/o	Treatment (Wk 3)	Treatment	Feed Day	No	00:55	00:00 - 00:59	Early Morning	37,3	47	9,0	9,0	18,46	534,23	73,19
	2019/05/30	CH2205	Male	Three y/o	Treatment (Wk 3)	Treatment	Feed Day	No	01:00	01:00 - 01:59	Early Morning	37,2	50	22,4	22,4	18,36	539,52	104,19
	2019/05/30	CH2206	Male	Three y/o	Treatment (Wk 3)	Treatment	Feed Day	No	01:00	01:00 - 01:59	Early Morning	37,3	62	4,8	4,8	18,46	557,33	52,04
	2019/05/30	CH2205	Male	Three y/o	Treatment (Wk 3)	Treatment	Feed Day	No	01:05	01:00 - 01:59	Early Morning	37,2		22,8	22,8	18,36		104,79
	2019/05/30	CH2206	Male	Three y/o	Treatment (Wk 3)	Treatment	Feed Day	No	01:05	01:00 - 01:59	Early Morning	37,3	76	7,2	7,2	18,46	573,39	65,66
	2019/05/30	CH2205	Male	Three y/o	Treatment (Wk 3)	Treatment	Feed Day	No	01:10	01:00 - 01:59	Early Morning	37,2	117	24,6	24,6	18,36	605,02	107,39
	2019/05/30	CH2206	Male	Three y/o	Treatment (Wk 3)	Treatment	Feed Day	No	01:10	01:00 - 01:59	Early Morning	37,3	45	4,2	4,2	18,46	530,47	47,57
	2019/05/30	CH2205	Male	Three y/o	Treatment (Wk 3)	Treatment	Feed Day	No	01:15	01:00 - 01:59	Early Morning	37,2		22,8	22,8	18,36		104,79
	2019/05/30	CH2206	Male	Three y/o	Treatment (Wk 3)	Treatment	Feed Day	No	01:15	01:00 - 01:59	Early Morning	37,3	50	3,8	3,8	18,46	539,52	44,22
	2019/05/30	CH2205	Male	Three y/o	Treatment (Wk 3)	Treatment	Feed Day	No	01:20	01:00 - 01:59	Early Morning	37,2		20,4	20,4	18,36		100,99
	2019/05/30	CH2206	Male	Three y/o	Treatment (Wk 3)	Treatment	Feed Day	No	01:20	01:00 - 01:59	Early Morning	37,3	101	60,8	60,8	18,46	594,59	138,55
	2019/05/30	CH2205	Male	Three y/o	Treatment (Wk 3)	Treatment	Feed Day	No	01:25	01:00 - 01:59	Early Morning	37,2		17,8	17,8	18,36		96,34
	2019/05/30	CH2206	Male	Three y/o	Treatment (Wk 3)	Treatment	Feed Day	No	01:25	01:00 - 01:59	Early Morning	37,3	47	65,0	65,0	18,46	534,23	140,87
	2019/05/30	CH2205	Male	Three y/o	Treatment (Wk 3)	Treatment	Feed Day	No	01:30	01:00 - 01:59	Early Morning	37,2	48	21,0	21,0	18,36	536,04	101,98

2019/05/30	CH2206	Male	Three y/o	Treatment (Wk 3)	Treatment	Feed Day	No	01:30	01:00 - 01:59	Early Morning	37,3	49	65,2	65,2	18,46	537,80	140,97
2019/05/30	CH2205	Male	Three y/o	Treatment (Wk 3)	Treatment	Feed Day	No	01:35	01:00 - 01:59	Early Morning	37,2		15,0	15,0	18,36		90,51
2019/05/30	CH2206	Male	Three y/o	Treatment (Wk 3)	Treatment	Feed Day	No	01:35	01:00 - 01:59	Early Morning	37,3	48	65,2	65,2	18,46	536,04	140,97
2019/05/30	CH2205	Male	Three y/o	Treatment (Wk 3)	Treatment	Feed Day	No	01:40	01:00 - 01:59	Early Morning	37,3	48	3,8	3,8	18,46	536,04	44,22
2019/05/30	CH2206	Male	Three y/o	Treatment (Wk 3)	Treatment	Feed Day	No	01:40	01:00 - 01:59	Early Morning	37,3	42	61,8	61,8	18,46	524,42	139,12
2019/05/30	CH2205	Male	Three y/o	Treatment (Wk 3)	Treatment	Feed Day	No	01:45	01:00 - 01:59	Early Morning	37,3	57	5,0	5,0	18,46	550,47	53,41
2019/05/30	CH2206	Male	Three y/o	Treatment (Wk 3)	Treatment	Feed Day	No	01:45	01:00 - 01:59	Early Morning	37,3	40	52,0	52,0	18,46	520,09	133,14
2019/05/30	CH2205	Male	Three y/o	Treatment (Wk 3)	Treatment	Feed Day	No	01:50	01:00 - 01:59	Early Morning	37,2	51	6,8	6,8	18,36	541,20	63,74
2019/05/30	CH2206	Male	Three y/o	Treatment (Wk 3)	Treatment	Feed Day	No	01:50	01:00 - 01:59	Early Morning	37,3	57	59,4	59,4	18,46	550,47	137,75
2019/05/30	CH2205	Male	Three y/o	Treatment (Wk 3)	Treatment	Feed Day	No	01:55	01:00 - 01:59	Early Morning	37,2		6,2	6,2	18,36		60,63
2019/05/30	CH2206	Male	Three y/o	Treatment (Wk 3)	Treatment	Feed Day	No	01:55	01:00 - 01:59	Early Morning	37,3	64	58,8	58,8	18,46	559,88	137,39
2019/05/30	CH2205	Male	Three y/o	Treatment (Wk 3)	Treatment	Feed Day	No	02:00	02:00 - 02:59	Early Morning	37,2	50	4,8	4,8	18,36	539,52	52,04
2019/05/30	CH2206	Male	Three y/o	Treatment (Wk 3)	Treatment	Feed Day	No	02:00	02:00 - 02:59	Early Morning	37,3	40	57,6	57,6	18,46	520,09	136,68
2019/05/30	CH2205	Male	Three y/o	Treatment (Wk 3)	Treatment	Feed Day	No	02:05	02:00 - 02:59	Early Morning	37,2	55	88,6	88,6	18,36	547,52	151,63
2019/05/30	CH2206	Male	Three y/o	Treatment (Wk 3)	Treatment	Feed Day	No	02:05	02:00 - 02:59	Early Morning	37,2	51	57,0	57,0	18,36	541,20	136,32
2019/05/30	CH2205	Male	Three y/o	Treatment (Wk 3)	Treatment	Feed Day	No	02:10	02:00 - 02:59	Early Morning	37,2	42	8,6	8,6	18,36	524,42	71,66
2019/05/30	CH2206	Male	Three y/o	Treatment (Wk 3)	Treatment	Feed Day	No	02:10	02:00 - 02:59	Early Morning	37,2	63	67,4	67,4	18,36	558,62	142,12
2019/05/30	CH2205	Male	Three y/o	Treatment (Wk 3)	Treatment	Feed Day	No	02:15	02:00 - 02:59	Early Morning	37,2	44	10,0	10,0	18,36	528,51	76,76
2019/05/30	CH2206	Male	Three y/o	Treatment (Wk 3)	Treatment	Feed Day	No	02:15	02:00 - 02:59	Early Morning	37,3	48	60,6	60,6	18,46	536,04	138,44
2019/05/30	CH2205	Male	Three y/o	Treatment (Wk 3)	Treatment	Feed Day	No	02:20	02:00 - 02:59	Early Morning	37,2	43	15,8	15,8	18,36	526,50	92,28
2019/05/30	CH2206	Male	Three y/o	Treatment (Wk 3)	Treatment	Feed Day	No	02:20	02:00 - 02:59	Early Morning	37,3	36	66,4	66,4	18,46	510,56	141,61
2019/05/30	CH2205	Male	Three y/o	Treatment (Wk 3)	Treatment	Feed Day	No	02:25	02:00 - 02:59	Early Morning	37,2	38	12,0	12,0	18,36	515,48	82,93
2019/05/30	CH2206	Male	Three y/o	Treatment (Wk 3)	Treatment	Feed Day	No	02:25	02:00 - 02:59	Early Morning	37,3	38	65,2	65,2	18,46	515,48	140,97
2019/05/30	CH2205	Male	Three y/o	Treatment (Wk 3)	Treatment	Feed Day	No	02:30	02:00 - 02:59	Early Morning	37,2	52	10,8	10,8	18,36	542,83	79,36
2019/05/30	CH2206	Male	Three y/o	Treatment (Wk 3)	Treatment	Feed Day	No	02:30	02:00 - 02:59	Early Morning	37,3	48	64,0	64,0	18,46	536,04	140,33
2019/05/30	CH2205	Male	Three y/o	Treatment (Wk 3)	Treatment	Feed Day	No	02:35	02:00 - 02:59	Early Morning	37,2	45	12,0	12,0	18,36	530,47	82,93
2019/05/30	CH2206	Male	Three y/o	Treatment (Wk 3)	Treatment	Feed Day	No	02:35	02:00 - 02:59	Early Morning	37,3	77	64,8	64,8	18,46	574,39	140,76
2019/05/30	CH2205	Male	Three y/o	Treatment (Wk 3)	Treatment	Feed Day	No	02:40	02:00 - 02:59	Early Morning	37,2	51	15,0	15,0	18,36	541,20	90,51
2019/05/30	CH2206	Male	Three y/o	Treatment (Wk 3)	Treatment	Feed Day	No	02:40	02:00 - 02:59	Early Morning	37,3	52	59,2	59,2	18,46	542,83	137,63
2019/05/30	CH2205	Male	Three y/o	Treatment (Wk 3)	Treatment	Feed Day	No	02:45	02:00 - 02:59	Early Morning	37,2	47	15,0	15,0	18,36	534,23	90,51
2019/05/30	CH2206	Male	Three y/o	Treatment (Wk 3)	Treatment	Feed Day	No	02:45	02:00 - 02:59	Early Morning	37,2	44	55,2	55,2	18,36	528,51	135,21
2019/05/30	CH2205	Male	Three y/o	Treatment (Wk 3)	Treatment	Feed Day	No	02:50	02:00 - 02:59	Early Morning	37,2	44	18,2	18,2	18,36	528,51	97,10
2019/05/30	CH2206	Male	Three y/o	Treatment (Wk 3)	Treatment	Feed Day	No	02:50	02:00 - 02:59	Early Morning	37,2	48	52,6	52,6	18,36	536,04	133,54
2019/05/30	CH2205	Male	Three y/o	Treatment (Wk 3)	Treatment	Feed Day	No	02:55	02:00 - 02:59	Early Morning	37,2	63	21,0	21,0	18,36	558,62	101,98
2019/05/30	CH2206	Male	Three y/o	Treatment (Wk 3)	Treatment	Feed Day	No	02:55	02:00 - 02:59	Early Morning	37,2	44	51,0	51,0	18,36	528,51	132,47
2019/05/30	CH2205	Male	Three y/o	Treatment (Wk 3)	Treatment	Feed Day	No	03:00	03:00 - 03:59	Early Morning	37,2	54	16,0	16,0	18,36	545,99	92,71
2019/05/30	CH2206	Male	Three y/o	Treatment (Wk 3)	Treatment	Feed Day	No	03:00	03:00 - 03:59	Early Morning	37,1	51	54,4	54,4	18,26	541,20	134,70
2019/05/30	CH2205	Male	Three y/o	Treatment (Wk 3)	Treatment	Feed Day	No	03:05	03:00 - 03:59	Early Morning	37,1	48	21,8	21,8	18,26	536,04	103,26
2019/05/30	CH2206	Male	Three y/o	Treatment (Wk 3)	Treatment	Feed Day	No	03:05	03:00 - 03:59	Early Morning	37,0	46	27,4	27,4	18,16	532,38	111,09
2019/05/30	CH2205	Male	Three y/o	Treatment (Wk 3)	Treatment	Feed Day	No	03:10	03:00 - 03:59	Early Morning	37,2	76	16,0	16,0	18,36	573,39	92,71
2019/05/30	CH2206	Male	Three y/o	Treatment (Wk 3)	Treatment	Feed Day	No	03:10	03:00 - 03:59	Early Morning	37,0	46	28,4	28,4	18,16	532,38	112,31
2019/05/30	CH2205	Male	Three y/o	Treatment (Wk 3)	Treatment	Feed Day	No	03:15	03:00 - 03:59	Early Morning	37,2	41	18,8	18,8	18,36	522,29	98,20
2019/05/30	CH2206	Male	Three y/o	Treatment (Wk 3)	Treatment	Feed Day	No	03:15	03:00 - 03:59	Early Morning	37,0	44	27,4	27,4	18,16	528,51	111,09
2019/05/30	CH2205	Male	Three y/o	Treatment (Wk 3)	Treatment	Feed Day	No	03:20	03:00 - 03:59	Early Morning	37,1	78	22,6	22,6	18,26	575,38	104,49
2019/05/30	CH2206	Male	Three y/o	Treatment (Wk 3)	Treatment	Feed Day	No	03:20	03:00 - 03:59	Early Morning	37,1	131	91,4	91,4	18,26	612,80	152,71
2019/05/30	CH2205	Male	Three y/o	Treatment (Wk 3)	Treatment	Feed Day	No	03:25	03:00 - 03:59	Early Morning	37,2	62	31,8	31,8	18,36	557,33	116,20
2019/05/30	CH2206	Male	Three y/o	Treatment (Wk 3)	Treatment	Feed Day	No	03:25	03:00 - 03:59	Early Morning	37,2	121	92,6	92,6	18,36	607,35	153,16
2019/05/30	CH2205	Male	Three y/o	Treatment (Wk 3)	Treatment	Feed Day	No	03:30	03:00 - 03:59	Early Morning	37,2		54,8	54,8	18,36		134,96
2019/05/30	CH2206	Male	Three y/o	Treatment (Wk 3)	Treatment	Feed Day	No	03:30	03:00 - 03:59	Early Morning	37,1	104	108,2	108,2	18,26	596,69	158,59
2019/05/30	CH2205	Male	Three y/o	Treatment (Wk 3)	Treatment	Feed Day	No	03:35	03:00 - 03:59	Early Morning	37,2	129	109,8	109,8	18,36	611,75	159,11
2019/05/30	CH2206	Male	Three y/o	Treatment (Wk 3)	Treatment	Feed Day	No	03:35	03:00 - 03:59	Early Morning	37,1	126	129,4	129,4	18,26	610,14	164,84
2019/05/30	CH2205	Male	Three y/o	Treatment (Wk 3)	Treatment	Feed Day	No	03:40	03:00 - 03:59	Early Morning	37,2	60	102,2	102,2	18,36	554,67	156,60
2019/05/30	CH2206	Male	Three y/o	Treatment (Wk 3)	Treatment	Feed Day	No	03:40	03:00 - 03:59	Early Morning	37,3	125	53,2	53,2	18,46	609,60	133,93
2019/05/30	CH2205	Male	Three y/o	Treatment (Wk 3)	Treatment	Feed Day	No	03:45	03:00 - 03:59	Early Morning	37,3	107	123,6	123,6	18,46	598,73	163,24
2019/05/30	CH2206	Male	Three y/o	Treatment (Wk 3)	Treatment	Feed Day	No	03:45	03:00 - 03:59	Early Morning	37,3	118	32,4	32,4	18,46	605,61	116,84
2019/05/30	CH2205	Male	Three y/o	Treatment (Wk 3)	Treatment	Feed Day	No	03:50	03:00 - 03:59	Early Morning	37,1	81	96,2	96,2	18,26	578,26	154,49
2019/05/30	CH2206	Male	Three y/o	Treatment (Wk 3)	Treatment	Feed Day	No	03:50	03:00 - 03:59	Early Morning	37,3	115	91,2	91,2	18,46	603,82	152,63
2019/05/30	CH2205	Male	Three y/o	Treatment (Wk 3)	Treatment	Feed Day	No	03:55	03:00 - 03:59	Early Morning	37,2	90	90,0	90,0	18,36	586,16	152,17
2019/05/30	CH2206	Male	Three y/o	Treatment (Wk 3)	Treatment	Feed Day	No	03:55	03:00 - 03:59	Early Morning	37,4	119	62,8	62,8	18,55	606,20	139,67
2019/05/30	CH2205	Male	Three y/o	Treatment (Wk 3)	Treatment	Feed Day	No	04:00	04:00 - 04:59	Morning	37,3	110	129,8	129,8	18,46	600,69	164,95
2019/05/30	CH2206	Male	Three y/o	Treatment (Wk 3)	Treatment	Feed Day	No	04:00	04:00 - 04:59	Morning	37,4	135	111,6	111,6	18,55	614,84	159,67
2019/05/30	CH2205	Male	Three y/o	Treatment (Wk 3)	Treatment	Feed Day	No	04:05	04:00 - 04:59	Morning	37,4	76	19,6	19,6	18,55	573,39	99,63
2019/05/30	CH2206	Male	Three y/o	Treatment (Wk 3)	Treatment	Feed Day	No	04:05	04:00 - 04:59	Morning	37,7	112	101,2	101,2	18,85	601,96	156,26
2019/05/30	CH2205	Male	Three y/o	Treatment (Wk 3)	Treatment	Feed Day	No	04:10	04:00 - 04:59	Morning	37,4	119	225,2	225,2	18,55	606,20	184,30
2019/05/30	CH2206	Male	Three y/o	Treatment (Wk 3)	Treatment	Feed Day	No	04:10	04:00 - 04:59	Morning	37,7	118	54,6	54,6	18,85	605,61	134,83
2019/05/30	CH2205	Male	Three y/o	Treatment (Wk 3)	Treatment	Feed Day	No	04:15	04:00 - 04:59	Morning	37,4	52	36,2	36,2	18,55	542,83	120,65
2019/05/30	CH2206	Male	Three y/o	Treatment (Wk 3)	Treatment	Feed Day	No	04:15	04:00 - 04:59	Morning	37,7	98	146,6	146,6	18,85	592,41	169,21
2019/05/30	CH2205	Male	Three y/o	Treatment (Wk 3)	Treatment	Feed Day	No	04:20	04:00 - 04:59	Morning	37,3	73	13,2	13,2	18,46	570,27	86,17
2019/05/30	CH2206	Male	Three y/o	Treatment (Wk 3)	Treatment	Feed Day	No	04:20	04:00 - 04:59	Morning	37,5	92	73,8	73,8	18,65	587,78	145,27
2019/05/30	CH2205	Male	Three y/o	Treatment (Wk 3)	Treatment	Feed Day	No	04:25	04:00 - 04:59	Morning	37,1	47	10,8	10,8	18,26	534,23	79,36

2019/05/30	CH2206	Male	Three y/o	Treatment (Wk 3)	Treatment	Feed Day	No	04:25	04:00 - 04:59	Morning	37,2	82	67,4	67,4	18,36	579,19	142,12
2019/05/30	CH2205	Male	Three y/o	Treatment (Wk 3)	Treatment	Feed Day	No	04:30	04:00 - 04:59	Morning	37,1	48	8,8	8,8	18,26	536,04	72,43
2019/05/30	CH2206	Male	Three y/o	Treatment (Wk 3)	Treatment	Feed Day	No	04:30	04:00 - 04:59	Morning	37,1	53	45,8	45,8	18,26	544,43	128,76
2019/05/30	CH2205	Male	Three y/o	Treatment (Wk 3)	Treatment	Feed Day	No	04:35	04:00 - 04:59	Morning	37,1	49	11,6	11,6	18,26	537,80	81,78
2019/05/30	CH2206	Male	Three y/o	Treatment (Wk 3)	Treatment	Feed Day	No	04:35	04:00 - 04:59	Morning	37,3	47	54,8	54,8	18,46	534,23	134,96
2019/05/30	CH2205	Male	Three y/o	Treatment (Wk 3)	Treatment	Feed Day	No	04:40	04:00 - 04:59	Morning	37,2	51	12,8	12,8	18,36	541,20	85,12
2019/05/30	CH2206	Male	Three y/o	Treatment (Wk 3)	Treatment	Feed Day	No	04:40	04:00 - 04:59	Morning	37,2	46	14,8	14,8	18,36	532,38	90,05
2019/05/30	CH2205	Male	Three y/o	Treatment (Wk 3)	Treatment	Feed Day	No	04:45	04:00 - 04:59	Morning	37,2	40	11,2	11,2	18,36	520,09	80,59
2019/05/30	CH2206	Male	Three y/o	Treatment (Wk 3)	Treatment	Feed Day	No	04:45	04:00 - 04:59	Morning	37,2	46	8,6	8,6	18,36	532,38	71,66
2019/05/30	CH2205	Male	Three y/o	Treatment (Wk 3)	Treatment	Feed Day	No	04:50	04:00 - 04:59	Morning	37,2	44	8,4	8,4	18,36	528,51	70,86
2019/05/30	CH2206	Male	Three y/o	Treatment (Wk 3)	Treatment	Feed Day	No	04:50	04:00 - 04:59	Morning	37,3	46	12,8	12,8	18,46	532,38	85,12
2019/05/30	CH2205	Male	Three y/o	Treatment (Wk 3)	Treatment	Feed Day	No	04:55	04:00 - 04:59	Morning	37,3	38	10,0	10,0	18,46	515,48	76,76
2019/05/30	CH2206	Male	Three y/o	Treatment (Wk 3)	Treatment	Feed Day	No	04:55	04:00 - 04:59	Morning	37,3	46	13,6	13,6	18,46	532,38	87,18
2019/05/30	CH2205	Male	Three y/o	Treatment (Wk 3)	Treatment	Feed Day	No	05:00	05:00 - 05:59	Morning	37,3	51	8,0	8,0	18,46	541,20	69,22
2019/05/30	CH2206	Male	Three y/o	Treatment (Wk 3)	Treatment	Feed Day	No	05:00	05:00 - 05:59	Morning	37,3	52	15,0	15,0	18,46	542,83	90,51
2019/05/30	CH2205	Male	Three y/o	Treatment (Wk 3)	Treatment	Feed Day	No	05:05	05:00 - 05:59	Morning	37,3	48	11,2	11,2	18,46	536,04	80,59
2019/05/30	CH2206	Male	Three y/o	Treatment (Wk 3)	Treatment	Feed Day	No	05:05	05:00 - 05:59	Morning	37,3	43	14,4	14,4	18,46	526,50	89,12
2019/05/30	CH2205	Male	Three y/o	Treatment (Wk 3)	Treatment	Feed Day	No	05:10	05:00 - 05:59	Morning	37,3	147	9,8	9,8	18,46	620,52	76,07
2019/05/30	CH2206	Male	Three y/o	Treatment (Wk 3)	Treatment	Feed Day	No	05:10	05:00 - 05:59	Morning	37,3	50	19,0	19,0	18,46	539,52	98,56
2019/05/30	CH2205	Male	Three y/o	Treatment (Wk 3)	Treatment	Feed Day	No	05:15	05:00 - 05:59	Morning	37,2	36	7,8	7,8	18,36	510,56	68,36
2019/05/30	CH2206	Male	Three y/o	Treatment (Wk 3)	Treatment	Feed Day	No	05:15	05:00 - 05:59	Morning	37,3	55	16,2	16,2	18,46	547,52	93,13
2019/05/30	CH2205	Male	Three y/o	Treatment (Wk 3)	Treatment	Feed Day	No	05:20	05:00 - 05:59	Morning	37,2	46	9,0	9,0	18,36	532,38	73,19
2019/05/30	CH2206	Male	Three y/o	Treatment (Wk 3)	Treatment	Feed Day	No	05:20	05:00 - 05:59	Morning	37,3	46	17,2	17,2	18,46	532,38	95,17
2019/05/30	CH2205	Male	Three y/o	Treatment (Wk 3)	Treatment	Feed Day	No	05:25	05:00 - 05:59	Morning	37,2	67	8,0	8,0	18,36	563,53	69,22
2019/05/30	CH2206	Male	Three y/o	Treatment (Wk 3)	Treatment	Feed Day	No	05:25	05:00 - 05:59	Morning	37,3	67	23,4	23,4	18,46	563,53	105,68
2019/05/30	CH2205	Male	Three y/o	Treatment (Wk 3)	Treatment	Feed Day	No	05:30	05:00 - 05:59	Morning	37,1	79	7,6	7,6	18,26	576,36	67,49
2019/05/30	CH2206	Male	Three y/o	Treatment (Wk 3)	Treatment	Feed Day	No	05:30	05:00 - 05:59	Morning	37,2	34	25,6	25,6	18,36	505,29	108,76
2019/05/30	CH2205	Male	Three y/o	Treatment (Wk 3)	Treatment	Feed Day	No	05:35	05:00 - 05:59	Morning	37,2	43	7,2	7,2	18,36	526,50	65,66
2019/05/30	CH2206	Male	Three y/o	Treatment (Wk 3)	Treatment	Feed Day	No	05:35	05:00 - 05:59	Morning	37,2	52	30,8	30,8	18,36	542,83	115,10
2019/05/30	CH2205	Male	Three y/o	Treatment (Wk 3)	Treatment	Feed Day	No	05:40	05:00 - 05:59	Morning	37,2	57	5,0	5,0	18,36	550,47	53,41
2019/05/30	CH2206	Male	Three y/o	Treatment (Wk 3)	Treatment	Feed Day	No	05:40	05:00 - 05:59	Morning	37,1	88	12,6	12,6	18,26	584,49	84,59
2019/05/30	CH2205	Male	Three y/o	Treatment (Wk 3)	Treatment	Feed Day	No	05:45	05:00 - 05:59	Morning	37,2	112	28,8	28,8	18,36	601,96	112,79
2019/05/30	CH2206	Male	Three y/o	Treatment (Wk 3)	Treatment	Feed Day	No	05:45	05:00 - 05:59	Morning	37,0	94	67,2	67,2	18,16	589,37	142,02
2019/05/30	CH2205	Male	Three y/o	Treatment (Wk 3)	Treatment	Feed Day	No	05:50	05:00 - 05:59	Morning	37,1	43	15,2	15,2	18,26	526,50	90,96
2019/05/30	CH2206	Male	Three y/o	Treatment (Wk 3)	Treatment	Feed Day	No	05:50	05:00 - 05:59	Morning	37,0	50	63,8	63,8	18,16	539,52	140,22
2019/05/30	CH2205	Male	Three y/o	Treatment (Wk 3)	Treatment	Feed Day	No	05:55	05:00 - 05:59	Morning	37,0	47	16,4	16,4	18,16	534,23	93,55
2019/05/30	CH2206	Male	Three y/o	Treatment (Wk 3)	Treatment	Feed Day	No	05:55	05:00 - 05:59	Morning	37,1	73	66,0	66,0	18,26	570,27	141,40
2019/05/30	CH2205	Male	Three y/o	Treatment (Wk 3)	Treatment	Feed Day	No	06:00	06:00 - 06:59	Morning	37,0	41	21,4	21,4	18,16	522,29	102,63
2019/05/30	CH2206	Male	Three y/o	Treatment (Wk 3)	Treatment	Feed Day	No	06:00	06:00 - 06:59	Morning	37,1	48	65,2	65,2	18,26	536,04	140,97
2019/05/30	CH2205	Male	Three y/o	Treatment (Wk 3)	Treatment	Feed Day	No	06:05	06:00 - 06:59	Morning	37,0		28,2	28,2	18,16		112,07
2019/05/30	CH2206	Male	Three y/o	Treatment (Wk 3)	Treatment	Feed Day	No	06:05	06:00 - 06:59	Morning	37,1	128	46,8	46,8	18,26	611,22	129,50
2019/05/30	CH2205	Male	Three y/o	Treatment (Wk 3)	Treatment	Feed Day	No	06:10	06:00 - 06:59	Morning	37,0		107,8	107,8	18,16		158,46
2019/05/30	CH2206	Male	Three y/o	Treatment (Wk 3)	Treatment	Feed Day	No	06:10	06:00 - 06:59	Morning	37,2	97	109,4	109,4	18,36	591,66	158,98
2019/05/30	CH2205	Male	Three y/o	Treatment (Wk 3)	Treatment	Feed Day	No	06:15	06:00 - 06:59	Morning	37,0	123	79,8	79,8	18,16	608,49	147,99
2019/05/30	CH2206	Male	Three y/o	Treatment (Wk 3)	Treatment	Feed Day	No	06:15	06:00 - 06:59	Morning	37,2	117	73,8	73,8	18,36	605,02	145,27
2019/05/30	CH2205	Male	Three y/o	Treatment (Wk 3)	Treatment	Feed Day	No	06:20	06:00 - 06:59	Morning	37,1	55	94,8	94,8	18,26	547,52	153,98
2019/05/30	CH2206	Male	Three y/o	Treatment (Wk 3)	Treatment	Feed Day	No	06:20	06:00 - 06:59	Morning	37,3	107	142,8	142,8	18,46	598,73	168,29
2019/05/30	CH2205	Male	Three y/o	Treatment (Wk 3)	Treatment	Feed Day	No	06:25	06:00 - 06:59	Morning	37,1		153,2	153,2	18,26		170,76
2019/05/30	CH2206	Male	Three y/o	Treatment (Wk 3)	Treatment	Feed Day	No	06:25	06:00 - 06:59	Morning	37,4	61	109,4	109,4	18,55	556,01	158,98
2019/05/30	CH2205	Male	Three y/o	Treatment (Wk 3)	Treatment	Feed Day	No	06:30	06:00 - 06:59	Morning	37,2	98	192,6	192,6	18,36	592,41	178,79
2019/05/30	CH2206	Male	Three y/o	Treatment (Wk 3)	Treatment	Feed Day	No	06:30	06:00 - 06:59	Morning	37,5	108	77,0	77,0	18,65	599,39	146,75
2019/05/30	CH2205	Male	Three y/o	Treatment (Wk 3)	Treatment	Feed Day	No	06:35	06:00 - 06:59	Morning	37,4	101	57,8	57,8	18,55	594,59	136,80
2019/05/30	CH2206	Male	Three y/o	Treatment (Wk 3)	Treatment	Feed Day	No	06:35	06:00 - 06:59	Morning	37,8	120	16,6	16,6	18,95	606,78	93,96
2019/05/30	CH2205	Male	Three y/o	Treatment (Wk 3)	Treatment	Feed Day	No	06:40	06:00 - 06:59	Morning	37,4	99	110,6	110,6	18,55	593,14	159,36
2019/05/30	CH2206	Male	Three y/o	Treatment (Wk 3)	Treatment	Feed Day	No	06:40	06:00 - 06:59	Morning	37,7	124	112,6	112,6	18,85	609,04	159,98
2019/05/30	CH2205	Male	Three y/o	Treatment (Wk 3)	Treatment	Feed Day	No	06:45	06:00 - 06:59	Morning	37,5	124	470,0	470,0	18,65	609,04	210,34
2019/05/30	CH2206	Male	Three y/o	Treatment (Wk 3)	Treatment	Feed Day	No	06:45	06:00 - 06:59	Morning	37,7	128	74,0	74,0	18,85	611,22	145,37
2019/05/30	CH2205	Male	Three y/o	Treatment (Wk 3)	Treatment	Feed Day	No	06:50	06:00 - 06:59	Morning	37,6		107,4	107,4	18,75		158,33
2019/05/30	CH2206	Male	Three y/o	Treatment (Wk 3)	Treatment	Feed Day	No	06:50	06:00 - 06:59	Morning	37,9	137	289,0	289,0	19,05	615,83	193,10
2019/05/30	CH2205	Male	Three y/o	Treatment (Wk 3)	Treatment	Feed Day	No	06:55	06:00 - 06:59	Morning	37,9	92	20,4	20,4	19,05	587,78	100,99
2019/05/30	CH2206	Male	Three y/o	Treatment (Wk 3)	Treatment	Feed Day	No	06:55	06:00 - 06:59	Morning	37,9	119	359,6	359,6	19,05	606,20	200,84
2019/05/30	CH2205	Male	Three y/o	Treatment (Wk 3)	Treatment	Feed Day	No	07:00	07:00 - 07:59	Morning	37,9	83	167,6	167,6	19,05	580,11	173,91
2019/05/30	CH2206	Male	Three y/o	Treatment (Wk 3)	Treatment	Feed Day	No	07:00	07:00 - 07:59	Morning	37,9	117	121,2	121,2	19,05	605,02	162,56
2019/05/30	CH2205	Male	Three y/o	Treatment (Wk 3)	Treatment	Feed Day	No	07:05	07:00 - 07:59	Morning	37,7	40	220,6	220,6	18,85	520,09	183,57
2019/05/30	CH2206	Male	Three y/o	Treatment (Wk 3)	Treatment	Feed Day	No	07:05	07:00 - 07:59	Morning	37,9	121	170,0	170,0	19,05	607,35	174,41
2019/05/30	CH2205	Male	Three y/o	Treatment (Wk 3)	Treatment	Feed Day	No	07:10	07:00 - 07:59	Morning	37,7	135	138,2	138,2	18,85	614,84	167,15
2019/05/30	CH2206	Male	Three y/o	Treatment (Wk 3)	Treatment	Feed Day	No	07:10	07:00 - 07:59	Morning	37,9	107	176,0	176,0	19,05	598,73	175,63
2019/05/30	CH2205	Male	Three y/o	Treatment (Wk 3)	Treatment	Feed Day	No	07:15	07:00 - 07:59	Morning	37,7	119	256,2	256,2	18,85	606,20	188,84
2019/05/30	CH2206	Male	Three y/o	Treatment (Wk 3)	Treatment	Feed Day	No	07:15	07:00 - 07:59	Morning	37,9	143	119,4	119,4	19,05	618,69	162,03
2019/05/30	CH2205	Male	Three y/o	Treatment (Wk 3)	Treatment	Feed Day	No	07:20	07:00 - 07:59	Morning	37,7		328,0	328,0	18,85		197,58

2019/05/30	CH2206	Male	Three y/o	Treatment (Wk 3)	Treatment	Feed Day	No	07:20	07:00 - 07:59	Morning	37,8	107	182,2	182,2	18,95	598,73	176,84
2019/05/30	CH2205	Male	Three y/o	Treatment (Wk 3)	Treatment	Feed Day	No	07:25	07:00 - 07:59	Morning	37,7	125	392,0	392,0	18,85	609,60	203,90
2019/05/30	CH2206	Male	Three y/o	Treatment (Wk 3)	Treatment	Feed Day	No	07:25	07:00 - 07:59	Morning	37,8	141	132,0	132,0	18,95	617,75	165,54
2019/05/30	CH2205	Male	Three y/o	Treatment (Wk 3)	Treatment	Feed Day	No	07:30	07:00 - 07:59	Morning	37,9		153,8	153,8	19,05		170,89
2019/05/30	CH2206	Male	Three y/o	Treatment (Wk 3)	Treatment	Feed Day	No	07:30	07:00 - 07:59	Morning	37,9	118	122,4	122,4	19,05	605,61	162,90
2019/05/30	CH2205	Male	Three y/o	Treatment (Wk 3)	Treatment	Feed Day	No	07:35	07:00 - 07:59	Morning	37,9	78	34,8	34,8	19,05	575,38	119,29
2019/05/30	CH2206	Male	Three y/o	Treatment (Wk 3)	Treatment	Feed Day	No	07:35	07:00 - 07:59	Morning	37,9	126	138,6	138,6	19,05	610,14	167,25
2019/05/30	CH2205	Male	Three y/o	Treatment (Wk 3)	Treatment	Feed Day	No	07:40	07:00 - 07:59	Morning	37,9		244,6	244,6	19,05		187,21
2019/05/30	CH2206	Male	Three y/o	Treatment (Wk 3)	Treatment	Feed Day	No	07:40	07:00 - 07:59	Morning	37,9	99	168,8	168,8	19,05	593,14	174,16
2019/05/30	CH2205	Male	Three y/o	Treatment (Wk 3)	Treatment	Feed Day	No	07:45	07:00 - 07:59	Morning	37,9	150	193,0	193,0	19,05	621,86	178,87
2019/05/30	CH2206	Male	Three y/o	Treatment (Wk 3)	Treatment	Feed Day	No	07:45	07:00 - 07:59	Morning	37,9	57	119,0	119,0	19,05	550,47	161,92
2019/05/30	CH2205	Male	Three y/o	Treatment (Wk 3)	Treatment	Feed Day	No	07:50	07:00 - 07:59	Morning	37,9	64	38,6	38,6	19,05	559,88	122,86
2019/05/30	CH2206	Male	Three y/o	Treatment (Wk 3)	Treatment	Feed Day	No	07:50	07:00 - 07:59	Morning	38,0	169	75,2	75,2	19,15	629,60	145,93
2019/05/30	CH2205	Male	Three y/o	Treatment (Wk 3)	Treatment	Feed Day	No	07:55	07:00 - 07:59	Morning	37,9	61	123,6	123,6	19,05	556,01	163,24
2019/05/30	CH2206	Male	Three y/o	Treatment (Wk 3)	Treatment	Feed Day	No	07:55	07:00 - 07:59	Morning	38,0	106	187,2	187,2	19,15	598,06	177,79
2019/05/30	CH2205	Male	Three y/o	Treatment (Wk 3)	Treatment	Feed Day	No	08:00	08:00 - 08:59	Late Morning	37,9	138	76,0	76,0	19,05	616,31	146,29
2019/05/30	CH2206	Male	Three y/o	Treatment (Wk 3)	Treatment	Feed Day	No	08:00	08:00 - 08:59	Late Morning	37,9	123	272,6	272,6	19,05	608,49	191,04
2019/05/30	CH2205	Male	Three y/o	Treatment (Wk 3)	Treatment	Feed Day	No	08:05	08:00 - 08:59	Late Morning	37,9		35,4	35,4	19,05		119,88
2019/05/30	CH2206	Male	Three y/o	Treatment (Wk 3)	Treatment	Feed Day	No	08:05	08:00 - 08:59	Late Morning	37,9	115	104,6	104,6	19,05	603,82	157,41
2019/05/30	CH2205	Male	Three y/o	Treatment (Wk 3)	Treatment	Feed Day	No	08:10	08:00 - 08:59	Late Morning	37,6		85,0	85,0	18,75		150,18
2019/05/30	CH2206	Male	Three y/o	Treatment (Wk 3)	Treatment	Feed Day	No	08:10	08:00 - 08:59	Late Morning	37,9		158,0	158,0	19,05		171,84
2019/05/30	CH2205	Male	Three y/o	Treatment (Wk 3)	Treatment	Feed Day	No	08:15	08:00 - 08:59	Late Morning	37,4	119	37,4	37,4	18,55	606,20	121,77
2019/05/30	CH2206	Male	Three y/o	Treatment (Wk 3)	Treatment	Feed Day	No	08:15	08:00 - 08:59	Late Morning	37,8	126	68,2	68,2	18,95	610,14	142,53
2019/05/30	CH2205	Male	Three y/o	Treatment (Wk 3)	Treatment	Feed Day	No	08:20	08:00 - 08:59	Late Morning	37,5		105,2	105,2	18,65		157,61
2019/05/30	CH2206	Male	Three y/o	Treatment (Wk 3)	Treatment	Feed Day	No	08:20	08:00 - 08:59	Late Morning	37,8	112	102,2	102,2	18,95	601,96	156,60
2019/05/30	CH2205	Male	Three y/o	Treatment (Wk 3)	Treatment	Feed Day	No	08:25	08:00 - 08:59	Late Morning	37,5	137	181,0	181,0	18,65	615,83	176,61
2019/05/30	CH2206	Male	Three y/o	Treatment (Wk 3)	Treatment	Feed Day	No	08:25	08:00 - 08:59	Late Morning	37,8		247,0	247,0	18,95		187,55
2019/05/30	CH2205	Male	Three y/o	Treatment (Wk 3)	Treatment	Feed Day	No	08:30	08:00 - 08:59	Late Morning	37,7	131	165,6	165,6	18,85	612,80	173,49
2019/05/30	CH2206	Male	Three y/o	Treatment (Wk 3)	Treatment	Feed Day	No	08:30	08:00 - 08:59	Late Morning	37,9	115	196,0	196,0	19,05	603,82	179,41
2019/05/30	CH2205	Male	Three y/o	Treatment (Wk 3)	Treatment	Feed Day	No	08:35	08:00 - 08:59	Late Morning	37,8	197	179,0	179,0	18,95	639,25	176,22
2019/05/30	CH2206	Male	Three y/o	Treatment (Wk 3)	Treatment	Feed Day	No	08:35	08:00 - 08:59	Late Morning	37,9	112	178,4	178,4	19,05	601,96	176,10
2019/05/30	CH2205	Male	Three y/o	Treatment (Wk 3)	Treatment	Feed Day	No	08:40	08:00 - 08:59	Late Morning	37,9		225,2	225,2	19,05		184,30
2019/05/30	CH2206	Male	Three y/o	Treatment (Wk 3)	Treatment	Feed Day	No	08:40	08:00 - 08:59	Late Morning	37,9	135	334,6	334,6	19,05	614,84	198,28
2019/05/30	CH2205	Male	Three y/o	Treatment (Wk 3)	Treatment	Feed Day	No	08:45	08:00 - 08:59	Late Morning	38,0	67	92,6	92,6	19,15	563,53	153,16
2019/05/30	CH2206	Male	Three y/o	Treatment (Wk 3)	Treatment	Feed Day	No	08:45	08:00 - 08:59	Late Morning	38,0		102,6	102,6	19,15		156,74
2019/05/30	CH2205	Male	Three y/o	Treatment (Wk 3)	Treatment	Feed Day	No	08:50	08:00 - 08:59	Late Morning	37,9	72	21,4	21,4	19,05	569,20	102,63
2019/05/30	CH2206	Male	Three y/o	Treatment (Wk 3)	Treatment	Feed Day	No	08:50	08:00 - 08:59	Late Morning	38,0	99	355,4	355,4	19,15	593,14	200,42
2019/05/30	CH2205	Male	Three y/o	Treatment (Wk 3)	Treatment	Feed Day	No	08:55	08:00 - 08:59	Late Morning	37,9	74	39,6	39,6	19,05	571,33	123,74
2019/05/30	CH2206	Male	Three y/o	Treatment (Wk 3)	Treatment	Feed Day	No	08:55	08:00 - 08:59	Late Morning	37,9	156	46,2	46,2	19,05	624,43	129,06
2019/05/30	CH2205	Male	Three y/o	Treatment (Wk 3)	Treatment	Feed Day	No	09:00	09:00 - 09:59	Late Morning	37,9	49	68,4	68,4	19,05	537,80	142,64
2019/05/30	CH2206	Male	Three y/o	Treatment (Wk 3)	Treatment	Feed Day	No	09:00	09:00 - 09:59	Late Morning	37,9	106	102,8	102,8	19,05	598,06	156,81
2019/05/30	CH2205	Male	Three y/o	Treatment (Wk 3)	Treatment	Feed Day	No	09:05	09:00 - 09:59	Late Morning	37,7	77	12,6	12,6	18,85	574,39	84,59
2019/05/30	CH2206	Male	Three y/o	Treatment (Wk 3)	Treatment	Feed Day	No	09:05	09:00 - 09:59	Late Morning	37,8		242,6	242,6	18,95		186,92
2019/05/30	CH2205	Male	Three y/o	Treatment (Wk 3)	Treatment	Feed Day	No	09:10	09:00 - 09:59	Late Morning	37,7	160	73,0	73,0	18,85	626,07	144,89
2019/05/30	CH2206	Male	Three y/o	Treatment (Wk 3)	Treatment	Feed Day	No	09:10	09:00 - 09:59	Late Morning	37,8	57	68,2	68,2	18,95	550,47	142,53
2019/05/30	CH2205	Male	Three y/o	Treatment (Wk 3)	Treatment	Feed Day	No	09:15	09:00 - 09:59	Late Morning	37,7	160	42,4	42,4	18,85	626,07	126,10
2019/05/30	CH2206	Male	Three y/o	Treatment (Wk 3)	Treatment	Feed Day	No	09:15	09:00 - 09:59	Late Morning	37,9	93	167,4	167,4	19,05	588,58	173,87
2019/05/30	CH2205	Male	Three y/o	Treatment (Wk 3)	Treatment	Feed Day	No	09:20	09:00 - 09:59	Late Morning	37,9		92,8	92,8	19,05		153,24
2019/05/30	CH2206	Male	Three y/o	Treatment (Wk 3)	Treatment	Feed Day	No	09:20	09:00 - 09:59	Late Morning	37,9	112	198,4	198,4	19,05	601,96	179,84
2019/05/30	CH2205	Male	Three y/o	Treatment (Wk 3)	Treatment	Feed Day	No	09:25	09:00 - 09:59	Late Morning	38,0	72	98,4	98,4	19,15	569,20	155,28
2019/05/30	CH2206	Male	Three y/o	Treatment (Wk 3)	Treatment	Feed Day	No	09:25	09:00 - 09:59	Late Morning	37,9	117	48,6	48,6	19,05	605,02	130,81
2019/05/30	CH2205	Male	Three y/o	Treatment (Wk 3)	Treatment	Feed Day	No	09:30	09:00 - 09:59	Late Morning	38,0		23,8	23,8	19,15		106,26
2019/05/30	CH2206	Male	Three y/o	Treatment (Wk 3)	Treatment	Feed Day	No	09:30	09:00 - 09:59	Late Morning	38,0	110	99,8	99,8	19,15	600,69	155,77
2019/05/30	CH2205	Male	Three y/o	Treatment (Wk 3)	Treatment	Feed Day	No	09:35	09:00 - 09:59	Late Morning	38,0	110	71,0	71,0	19,15	600,69	143,93
2019/05/30	CH2206	Male	Three y/o	Treatment (Wk 3)	Treatment	Feed Day	No	09:35	09:00 - 09:59	Late Morning	38,0	61	114,6	114,6	19,15	556,01	160,60
2019/05/30	CH2205	Male	Three y/o	Treatment (Wk 3)	Treatment	Feed Day	No	09:40	09:00 - 09:59	Late Morning	38,0	107	26,6	26,6	19,15	598,73	110,07
2019/05/30	CH2206	Male	Three y/o	Treatment (Wk 3)	Treatment	Feed Day	No	09:40	09:00 - 09:59	Late Morning	38,0	197	54,8	54,8	19,15	639,25	134,96
2019/05/30	CH2205	Male	Three y/o	Treatment (Wk 3)	Treatment	Feed Day	No	09:45	09:00 - 09:59	Late Morning	37,9	68	16,6	16,6	19,05	564,71	93,96
2019/05/30	CH2206	Male	Three y/o	Treatment (Wk 3)	Treatment	Feed Day	No	09:45	09:00 - 09:59	Late Morning	37,9		117,6	117,6	19,05		161,50
2019/05/30	CH2205	Male	Three y/o	Treatment (Wk 3)	Treatment	Feed Day	No	09:50	09:00 - 09:59	Late Morning	37,9	65	73,6	73,6	19,05	561,12	145,18
2019/05/30	CH2206	Male	Three y/o	Treatment (Wk 3)	Treatment	Feed Day	No	09:50	09:00 - 09:59	Late Morning	37,9	112	103,2	103,2	19,05	601,96	156,94
2019/05/30	CH2205	Male	Three y/o	Treatment (Wk 3)	Treatment	Feed Day	No	09:55	09:00 - 09:59	Late Morning	37,9	67	7,8	7,8	19,05	563,53	68,36
2019/05/30	CH2206	Male	Three y/o	Treatment (Wk 3)	Treatment	Feed Day	No	09:55	09:00 - 09:59	Late Morning	37,9	92	96,8	96,8	19,05	587,78	154,71
2019/05/30	CH2205	Male	Three y/o	Treatment (Wk 3)	Treatment	Feed Day	No	10:00	10:00 - 10:59	Late Morning	37,9	51	16,4	16,4	19,05	541,20	93,55
2019/05/30	CH2206	Male	Three y/o	Treatment (Wk 3)	Treatment	Feed Day	No	10:00	10:00 - 10:59	Late Morning	37,9	141	30,4	30,4	19,05	617,75	114,65
2019/05/30	CH2205	Male	Three y/o	Treatment (Wk 3)	Treatment	Feed Day	No	10:05	10:00 - 10:59	Late Morning	37,9	54	11,4	11,4	19,05	545,99	81,19
2019/05/30	CH2206	Male	Three y/o	Treatment (Wk 3)	Treatment	Feed Day	No	10:05	10:00 - 10:59	Late Morning	37,8	67	35,0	35,0	18,95	563,53	119,49
2019/05/30	CH2205	Male	Three y/o	Treatment (Wk 3)	Treatment	Feed Day	No	10:10	10:00 - 10:59	Late Morning	37,9	64	12,2	12,2	19,05	559,88	83,49
2019/05/30	CH2206	Male	Three y/o	Treatment (Wk 3)	Treatment	Feed Day	No	10:10	10:00 - 10:59	Late Morning	37,8	73	106,6	106,6	18,95	570,27	158,07
2019/05/30	CH2205	Male	Three y/o	Treatment (Wk 3)	Treatment	Feed Day	No	10:15	10:00 - 10:59	Late Morning	37,9	62	43,2	43,2	19,05	557,33	126,74

2019/05/30	CH2206	Male	Three y/o	Treatment (Wk 3)	Treatment	Feed Day	No	10:15	10:00 - 10:59	Late Morning	37,8	110	207,8	207,8	18,95	600,69	181,47
2019/05/30	CH2205	Male	Three y/o	Treatment (Wk 3)	Treatment	Feed Day	No	10:20	10:00 - 10:59	Late Morning	37,9	61	19,6	19,6	19,05	556,01	99,63
2019/05/30	CH2206	Male	Three y/o	Treatment (Wk 3)	Treatment	Feed Day	No	10:20	10:00 - 10:59	Late Morning	37,8	55	31,4	31,4	18,95	547,52	115,76
2019/05/30	CH2205	Male	Three y/o	Treatment (Wk 3)	Treatment	Feed Day	No	10:25	10:00 - 10:59	Late Morning	37,7	51	11,8	11,8	18,85	541,20	82,36
2019/05/30	CH2206	Male	Three y/o	Treatment (Wk 3)	Treatment	Feed Day	No	10:25	10:00 - 10:59	Late Morning	37,9	64	27,0	27,0	19,05	559,88	110,58
2019/05/30	CH2205	Male	Three y/o	Treatment (Wk 3)	Treatment	Feed Day	No	10:30	10:00 - 10:59	Late Morning	37,7	50	19,4	19,4	18,85	539,52	99,28
2019/05/30	CH2206	Male	Three y/o	Treatment (Wk 3)	Treatment	Feed Day	No	10:30	10:00 - 10:59	Late Morning	37,9	59	32,8	32,8	19,05	553,30	117,26
2019/05/30	CH2205	Male	Three y/o	Treatment (Wk 3)	Treatment	Feed Day	No	10:35	10:00 - 10:59	Late Morning	37,9		70,6	70,6	19,05		143,73
2019/05/30	CH2206	Male	Three y/o	Treatment (Wk 3)	Treatment	Feed Day	No	10:35	10:00 - 10:59	Late Morning	37,9	59	34,4	34,4	19,05	553,30	118,90
2019/05/30	CH2205	Male	Three y/o	Treatment (Wk 3)	Treatment	Feed Day	No	10:40	10:00 - 10:59	Late Morning	37,9	56	25,8	25,8	19,05	549,01	109,02
2019/05/30	CH2206	Male	Three y/o	Treatment (Wk 3)	Treatment	Feed Day	No	10:40	10:00 - 10:59	Late Morning	37,9	115	139,2	139,2	19,05	603,82	167,40
2019/05/30	CH2205	Male	Three y/o	Treatment (Wk 3)	Treatment	Feed Day	No	10:45	10:00 - 10:59	Late Morning	37,7	97	60,8	60,8	18,85	591,66	138,55
2019/05/30	CH2206	Male	Three y/o	Treatment (Wk 3)	Treatment	Feed Day	No	10:45	10:00 - 10:59	Late Morning	38,0		110,6	110,6	19,15		159,36
2019/05/30	CH2205	Male	Three y/o	Treatment (Wk 3)	Treatment	Feed Day	No	10:50	10:00 - 10:59	Late Morning	37,9	169	29,6	29,6	19,05	629,60	113,73
2019/05/30	CH2206	Male	Three y/o	Treatment (Wk 3)	Treatment	Feed Day	No	10:50	10:00 - 10:59	Late Morning	37,9	70	49,8	49,8	19,05	566,99	131,65
2019/05/30	CH2205	Male	Three y/o	Treatment (Wk 3)	Treatment	Feed Day	No	10:55	10:00 - 10:59	Late Morning	37,9	63	6,4	6,4	19,05	558,62	61,70
2019/05/30	CH2206	Male	Three y/o	Treatment (Wk 3)	Treatment	Feed Day	No	10:55	10:00 - 10:59	Late Morning	37,9	115	55,4	55,4	19,05	603,82	135,33
2019/05/30	CH2205	Male	Three y/o	Treatment (Wk 3)	Treatment	Feed Day	No	11:00	11:00 - 11:59	Late Morning	37,9	80	17,4	17,4	19,05	577,31	95,56
2019/05/30	CH2206	Male	Three y/o	Treatment (Wk 3)	Treatment	Feed Day	No	11:00	11:00 - 11:59	Late Morning	37,9	113	273,2	273,2	19,05	602,58	191,11
2019/05/30	CH2205	Male	Three y/o	Treatment (Wk 3)	Treatment	Feed Day	No	11:05	11:00 - 11:59	Late Morning	37,8	61	39,6	39,6	18,95	556,01	123,74
2019/05/30	CH2206	Male	Three y/o	Treatment (Wk 3)	Treatment	Feed Day	No	11:05	11:00 - 11:59	Late Morning	37,9	53	97,0	97,0	19,05	544,43	154,78
2019/05/30	CH2205	Male	Three y/o	Treatment (Wk 3)	Treatment	Feed Day	No	11:10	11:00 - 11:59	Late Morning	37,7	88	51,2	51,2	18,85	584,49	132,61
2019/05/30	CH2206	Male	Three y/o	Treatment (Wk 3)	Treatment	Feed Day	No	11:10	11:00 - 11:59	Late Morning	37,9	31	104,8	104,8	19,05	496,63	157,48
2019/05/30	CH2205	Male	Three y/o	Treatment (Wk 3)	Treatment	Feed Day	No	11:15	11:00 - 11:59	Late Morning	37,6		322,8	322,8	18,75		197,01
2019/05/30	CH2206	Male	Three y/o	Treatment (Wk 3)	Treatment	Feed Day	No	11:15	11:00 - 11:59	Late Morning	37,9	119	99,8	99,8	19,05	606,20	155,77
2019/05/30	CH2205	Male	Three y/o	Treatment (Wk 3)	Treatment	Feed Day	No	11:20	11:00 - 11:59	Late Morning	37,7	109	11,6	11,6	18,85	600,04	81,78
2019/05/30	CH2206	Male	Three y/o	Treatment (Wk 3)	Treatment	Feed Day	No	11:20	11:00 - 11:59	Late Morning	38,0	99	64,6	64,6	19,15	593,14	140,65
2019/05/30	CH2205	Male	Three y/o	Treatment (Wk 3)	Treatment	Feed Day	No	11:25	11:00 - 11:59	Late Morning	37,6	60	23,4	23,4	18,75	554,67	105,68
2019/05/30	CH2206	Male	Three y/o	Treatment (Wk 3)	Treatment	Feed Day	No	11:25	11:00 - 11:59	Late Morning	37,9	70	5,2	5,2	19,05	566,99	54,72
2019/05/30	CH2205	Male	Three y/o	Treatment (Wk 3)	Treatment	Feed Day	No	11:30	11:00 - 11:59	Late Morning	37,6	57	9,2	9,2	18,75	550,47	73,94
2019/05/30	CH2206	Male	Three y/o	Treatment (Wk 3)	Treatment	Feed Day	No	11:30	11:00 - 11:59	Late Morning	37,9	64	7,4	7,4	19,05	559,88	66,59
2019/05/30	CH2205	Male	Three y/o	Treatment (Wk 3)	Treatment	Feed Day	No	11:35	11:00 - 11:59	Late Morning	37,7	83	23,2	23,2	18,85	580,11	105,39
2019/05/30	CH2206	Male	Three y/o	Treatment (Wk 3)	Treatment	Feed Day	No	11:35	11:00 - 11:59	Late Morning	37,9	121	37,2	37,2	19,05	607,35	121,59
2019/05/30	CH2205	Male	Three y/o	Treatment (Wk 3)	Treatment	Feed Day	No	11:40	11:00 - 11:59	Late Morning	37,8	58	7,0	7,0	18,95	551,90	64,72
2019/05/30	CH2206	Male	Three y/o	Treatment (Wk 3)	Treatment	Feed Day	No	11:40	11:00 - 11:59	Late Morning	38,1	64	51,6	51,6	19,26	559,88	132,87
2019/05/30	CH2205	Male	Three y/o	Treatment (Wk 3)	Treatment	Feed Day	No	11:45	11:00 - 11:59	Late Morning	37,9	55	7,0	7,0	19,05	547,52	64,72
2019/05/30	CH2206	Male	Three y/o	Treatment (Wk 3)	Treatment	Feed Day	No	11:45	11:00 - 11:59	Late Morning	38,0	52	48,8	48,8	19,15	542,83	130,95
2019/05/30	CH2205	Male	Three y/o	Treatment (Wk 3)	Treatment	Feed Day	No	11:50	11:00 - 11:59	Late Morning	37,9	46	6,0	6,0	19,05	532,38	59,53
2019/05/30	CH2206	Male	Three y/o	Treatment (Wk 3)	Treatment	Feed Day	No	11:50	11:00 - 11:59	Late Morning	37,9	54	55,2	55,2	19,05	545,99	135,21
2019/05/30	CH2205	Male	Three y/o	Treatment (Wk 3)	Treatment	Feed Day	No	11:55	11:00 - 11:59	Late Morning	37,9	55	5,0	5,0	19,05	547,52	53,41
2019/05/30	CH2206	Male	Three y/o	Treatment (Wk 3)	Treatment	Feed Day	No	11:55	11:00 - 11:59	Late Morning	37,9	66	43,8	43,8	19,05	562,34	127,22
2019/05/30	CH2205	Male	Three y/o	Treatment (Wk 3)	Treatment	Feed Day	No	12:00	12:00 - 12:59	Afternoon	37,9	56	2,8	2,8	19,05	549,01	34,04
2019/05/30	CH2206	Male	Three y/o	Treatment (Wk 3)	Treatment	Feed Day	No	12:00	12:00 - 12:59	Afternoon	37,9	63	49,2	49,2	19,05	558,62	131,23
2019/05/30	CH2205	Male	Three y/o	Treatment (Wk 3)	Treatment	Feed Day	No	12:05	12:00 - 12:59	Afternoon	37,7	55	3,6	3,6	18,85	547,52	42,42
2019/05/30	CH2206	Male	Three y/o	Treatment (Wk 3)	Treatment	Feed Day	No	12:05	12:00 - 12:59	Afternoon	37,8		91,4	91,4	18,95		152,71
2019/05/30	CH2205	Male	Three y/o	Treatment (Wk 3)	Treatment	Feed Day	No	12:10	12:00 - 12:59	Afternoon	37,7	48	4,0	4,0	18,85	536,04	45,94
2019/05/30	CH2206	Male	Three y/o	Treatment (Wk 3)	Treatment	Feed Day	No	12:10	12:00 - 12:59	Afternoon	37,8	49	45,0	45,0	18,95	537,80	128,15
2019/05/30	CH2205	Male	Three y/o	Treatment (Wk 3)	Treatment	Feed Day	No	12:15	12:00 - 12:59	Afternoon	37,7	51	4,6	4,6	18,85	541,20	50,61
2019/05/30	CH2206	Male	Three y/o	Treatment (Wk 3)	Treatment	Feed Day	No	12:15	12:00 - 12:59	Afternoon	37,8	50	54,6	54,6	18,95	539,52	134,83
2019/05/30	CH2205	Male	Three y/o	Treatment (Wk 3)	Treatment	Feed Day	No	12:20	12:00 - 12:59	Afternoon	37,6	63	1,6	1,6	18,75	558,62	15,48
2019/05/30	CH2206	Male	Three y/o	Treatment (Wk 3)	Treatment	Feed Day	No	12:20	12:00 - 12:59	Afternoon	37,8	79	44,2	44,2	18,95	576,36	127,53
2019/05/30	CH2205	Male	Three y/o	Treatment (Wk 3)	Treatment	Feed Day	No	12:25	12:00 - 12:59	Afternoon	37,6	55	7,6	7,6	18,75	547,52	67,49
2019/05/30	CH2206	Male	Three y/o	Treatment (Wk 3)	Treatment	Feed Day	No	12:25	12:00 - 12:59	Afternoon	37,7	53	47,2	47,2	18,85	544,43	129,80
2019/05/30	CH2205	Male	Three y/o	Treatment (Wk 3)	Treatment	Feed Day	No	12:30	12:00 - 12:59	Afternoon	37,6	52	4,8	4,8	18,75	542,83	52,04
2019/05/30	CH2206	Male	Three y/o	Treatment (Wk 3)	Treatment	Feed Day	No	12:30	12:00 - 12:59	Afternoon	37,7	53	46,2	46,2	18,85	544,43	129,06
2019/05/30	CH2205	Male	Three y/o	Treatment (Wk 3)	Treatment	Feed Day	No	12:35	12:00 - 12:59	Afternoon	37,6	78	123,8	123,8	18,75	575,38	163,30
2019/05/30	CH2206	Male	Three y/o	Treatment (Wk 3)	Treatment	Feed Day	No	12:35	12:00 - 12:59	Afternoon	37,7	52	42,8	42,8	18,85	542,83	126,42
2019/05/30	CH2205	Male	Three y/o	Treatment (Wk 3)	Treatment	Feed Day	No	12:40	12:00 - 12:59	Afternoon	37,6		41,4	41,4	18,75		125,27
2019/05/30	CH2206	Male	Three y/o	Treatment (Wk 3)	Treatment	Feed Day	No	12:40	12:00 - 12:59	Afternoon	37,7	90	40,2	40,2	18,85	586,16	124,26
2019/05/30	CH2205	Male	Three y/o	Treatment (Wk 3)	Treatment	Feed Day	No	12:45	12:00 - 12:59	Afternoon	37,6	68	13,4	13,4	18,75	564,71	86,68
2019/05/30	CH2206	Male	Three y/o	Treatment (Wk 3)	Treatment	Feed Day	No	12:45	12:00 - 12:59	Afternoon	37,7	71	53,4	53,4	18,85	568,10	134,06
2019/05/30	CH2205	Male	Three y/o	Treatment (Wk 3)	Treatment	Feed Day	No	12:50	12:00 - 12:59	Afternoon	37,6	51	5,2	5,2	18,75	541,20	54,72
2019/05/30	CH2206	Male	Three y/o	Treatment (Wk 3)	Treatment	Feed Day	No	12:50	12:00 - 12:59	Afternoon	37,5	44	59,0	59,0	18,65	528,51	137,51
2019/05/30	CH2205	Male	Three y/o	Treatment (Wk 3)	Treatment	Feed Day	No	12:55	12:00 - 12:59	Afternoon	37,6	47	4,6	4,6	18,75	534,23	50,61
2019/05/30	CH2206	Male	Three y/o	Treatment (Wk 3)	Treatment	Feed Day	No	12:55	12:00 - 12:59	Afternoon	37,4	49	51,6	51,6	18,55	537,80	132,87
2019/05/30	CH2205	Male	Three y/o	Treatment (Wk 3)	Treatment	Feed Day	No	13:00	13:00 - 13:59	Afternoon	37,5	46	4,2	4,2	18,65	532,38	47,57
2019/05/30	CH2206	Male	Three y/o	Treatment (Wk 3)	Treatment	Feed Day	No	13:00	13:00 - 13:59	Afternoon	37,4	54	59,0	59,0	18,55	545,99	137,51
2019/05/30	CH2205	Male	Three y/o	Treatment (Wk 3)	Treatment	Feed Day	No	13:05	13:00 - 13:59	Afternoon	37,5	62	3,0	3,0	18,65	557,33	36,34
2019/05/30	CH2206	Male	Three y/o	Treatment (Wk 3)	Treatment	Feed Day	No	13:05	13:00 - 13:59	Afternoon	37,4	39	70,6	70,6	18,55	517,82	143,73
2019/05/30	CH2205	Male	Three y/o	Treatment (Wk 3)	Treatment	Feed Day	No	13:10	13:00 - 13:59	Afternoon	37,5	63	17,2	17,2	18,65	558,62	95,17

2019/05/30	CH2206	Male	Three y/o	Treatment (Wk 3)	Treatment	Feed Day	No	13:10	13:00 - 13:59	Afternoon	37,4	40	53,2	53,2	18,55	520,09	133,93
2019/05/30	CH2205	Male	Three y/o	Treatment (Wk 3)	Treatment	Feed Day	No	13:15	13:00 - 13:59	Afternoon	37,5		11,4	11,4	18,65		81,19
2019/05/30	CH2206	Male	Three y/o	Treatment (Wk 3)	Treatment	Feed Day	No	13:15	13:00 - 13:59	Afternoon	37,4	96	45,2	45,2	18,55	590,91	128,30
2019/05/30	CH2205	Male	Three y/o	Treatment (Wk 3)	Treatment	Feed Day	No	13:20	13:00 - 13:59	Afternoon	37,5	42	107,6	107,6	18,65	524,42	158,40
2019/05/30	CH2206	Male	Three y/o	Treatment (Wk 3)	Treatment	Feed Day	No	13:20	13:00 - 13:59	Afternoon	37,5	141	46,8	46,8	18,65	617,75	129,50
2019/05/30	CH2205	Male	Three y/o	Treatment (Wk 3)	Treatment	Feed Day	No	13:25	13:00 - 13:59	Afternoon	37,5	104	195,2	195,2	18,65	596,69	179,26
2019/05/30	CH2206	Male	Three y/o	Treatment (Wk 3)	Treatment	Feed Day	No	13:25	13:00 - 13:59	Afternoon	37,4	102	119,0	119,0	18,55	595,30	161,92
2019/05/30	CH2205	Male	Three y/o	Treatment (Wk 3)	Treatment	Feed Day	No	13:30	13:00 - 13:59	Afternoon	37,5		70,6	70,6	18,65		143,73
2019/05/30	CH2206	Male	Three y/o	Treatment (Wk 3)	Treatment	Feed Day	No	13:30	13:00 - 13:59	Afternoon	37,4	106	164,0	164,0	18,55	598,06	173,15
2019/05/30	CH2205	Male	Three y/o	Treatment (Wk 3)	Treatment	Feed Day	No	13:35	13:00 - 13:59	Afternoon	37,5	98	173,4	173,4	18,65	592,41	175,10
2019/05/30	CH2206	Male	Three y/o	Treatment (Wk 3)	Treatment	Feed Day	No	13:35	13:00 - 13:59	Afternoon	37,5	111	225,6	225,6	18,65	601,33	184,36
2019/05/30	CH2205	Male	Three y/o	Treatment (Wk 3)	Treatment	Feed Day	No	13:40	13:00 - 13:59	Afternoon	37,5	51	26,4	26,4	18,65	541,20	109,81
2019/05/30	CH2206	Male	Three y/o	Treatment (Wk 3)	Treatment	Feed Day	No	13:40	13:00 - 13:59	Afternoon	37,5	117	120,2	120,2	18,65	605,02	162,27
2019/05/30	CH2205	Male	Three y/o	Treatment (Wk 3)	Treatment	Feed Day	No	13:45	13:00 - 13:59	Afternoon	37,6	105	45,6	45,6	18,75	597,38	128,61
2019/05/30	CH2206	Male	Three y/o	Treatment (Wk 3)	Treatment	Feed Day	No	13:45	13:00 - 13:59	Afternoon	37,5		82,0	82,0	18,65		148,93
2019/05/30	CH2205	Male	Three y/o	Treatment (Wk 3)	Treatment	Feed Day	No	13:50	13:00 - 13:59	Afternoon	37,6	100	106,8	106,8	18,75	593,87	158,14
2019/05/30	CH2206	Male	Three y/o	Treatment (Wk 3)	Treatment	Feed Day	No	13:50	13:00 - 13:59	Afternoon	37,7	114	106,8	106,8	18,85	603,20	158,14
2019/05/30	CH2205	Male	Three y/o	Treatment (Wk 3)	Treatment	Feed Day	No	13:55	13:00 - 13:59	Afternoon	37,6	93	67,0	67,0	18,75	588,58	141,92
2019/05/30	CH2206	Male	Three y/o	Treatment (Wk 3)	Treatment	Feed Day	No	13:55	13:00 - 13:59	Afternoon	37,7	145	193,4	193,4	18,85	619,62	178,94
2019/05/30	CH2205	Male	Three y/o	Treatment (Wk 3)	Treatment	Feed Day	No	14:00	14:00 - 14:59	Afternoon	37,6	80	151,6	151,6	18,75	577,31	170,39
2019/05/30	CH2206	Male	Three y/o	Treatment (Wk 3)	Treatment	Feed Day	No	14:00	14:00 - 14:59	Afternoon	37,7	45	21,4	21,4	18,85	530,47	102,63
2019/05/30	CH2205	Male	Three y/o	Treatment (Wk 3)	Treatment	Feed Day	No	14:05	14:00 - 14:59	Afternoon	37,7	41	284,4	284,4	18,85	522,29	192,53
2019/05/30	CH2206	Male	Three y/o	Treatment (Wk 3)	Treatment	Feed Day	No	14:05	14:00 - 14:59	Afternoon	37,8	92	164,0	164,0	18,95	587,78	173,15
2019/05/30	CH2205	Male	Three y/o	Treatment (Wk 3)	Treatment	Feed Day	No	14:10	14:00 - 14:59	Afternoon	37,7		38,2	38,2	18,85		122,50
2019/05/30	CH2206	Male	Three y/o	Treatment (Wk 3)	Treatment	Feed Day	No	14:10	14:00 - 14:59	Afternoon	37,8	100	14,2	14,2	18,95	593,87	88,65
2019/05/30	CH2205	Male	Three y/o	Treatment (Wk 3)	Treatment	Feed Day	No	14:15	14:00 - 14:59	Afternoon	37,7	52	23,4	23,4	18,85	542,83	105,68
2019/05/30	CH2206	Male	Three y/o	Treatment (Wk 3)	Treatment	Feed Day	No	14:15	14:00 - 14:59	Afternoon	37,8	50	22,2	22,2	18,95	539,52	103,88
2019/05/30	CH2205	Male	Three y/o	Treatment (Wk 3)	Treatment	Feed Day	No	14:20	14:00 - 14:59	Afternoon	37,8	45	25,4	25,4	18,95	530,47	108,49
2019/05/30	CH2206	Male	Three y/o	Treatment (Wk 3)	Treatment	Feed Day	No	14:20	14:00 - 14:59	Afternoon	37,8	48	27,0	27,0	18,95	536,04	110,58
2019/05/30	CH2205	Male	Three y/o	Treatment (Wk 3)	Treatment	Feed Day	No	14:25	14:00 - 14:59	Afternoon	37,9	52	26,4	26,4	19,05	542,83	109,81
2019/05/30	CH2206	Male	Three y/o	Treatment (Wk 3)	Treatment	Feed Day	No	14:25	14:00 - 14:59	Afternoon	37,8	67	24,8	24,8	18,95	563,53	107,67
2019/05/30	CH2205	Male	Three y/o	Treatment (Wk 3)	Treatment	Feed Day	No	14:30	14:00 - 14:59	Afternoon	37,9	45	17,2	17,2	19,05	530,47	95,17
2019/05/30	CH2206	Male	Three y/o	Treatment (Wk 3)	Treatment	Feed Day	No	14:30	14:00 - 14:59	Afternoon	37,8	48	24,8	24,8	18,95	536,04	107,67
2019/05/30	CH2205	Male	Three y/o	Treatment (Wk 3)	Treatment	Feed Day	No	14:35	14:00 - 14:59	Afternoon	37,9	49	125,8	125,8	19,05	537,80	163,86
2019/05/30	CH2206	Male	Three y/o	Treatment (Wk 3)	Treatment	Feed Day	No	14:35	14:00 - 14:59	Afternoon	37,8	83	91,6	91,6	18,95	580,11	152,79
2019/05/30	CH2205	Male	Three y/o	Treatment (Wk 3)	Treatment	Feed Day	No	14:40	14:00 - 14:59	Afternoon	37,9	99	54,2	54,2	19,05	593,14	134,57
2019/05/30	CH2206	Male	Three y/o	Treatment (Wk 3)	Treatment	Feed Day	No	14:40	14:00 - 14:59	Afternoon	37,8	119	178,0	178,0	18,95	606,20	176,02
2019/05/30	CH2205	Male	Three y/o	Treatment (Wk 3)	Treatment	Feed Day	No	14:45	14:00 - 14:59	Afternoon	37,7	160	122,6	122,6	18,85	626,07	162,96
2019/05/30	CH2206	Male	Three y/o	Treatment (Wk 3)	Treatment	Feed Day	No	14:45	14:00 - 14:59	Afternoon	37,8	113	140,4	140,4	18,95	602,58	167,70
2019/05/30	CH2205	Male	Three y/o	Treatment (Wk 3)	Treatment	Feed Day	No	14:50	14:00 - 14:59	Afternoon	37,7	51	252,8	252,8	18,85	541,20	188,37
2019/05/30	CH2206	Male	Three y/o	Treatment (Wk 3)	Treatment	Feed Day	No	14:50	14:00 - 14:59	Afternoon	37,8		104,2	104,2	18,95		157,28
2019/05/30	CH2205	Male	Three y/o	Treatment (Wk 3)	Treatment	Feed Day	No	14:55	14:00 - 14:59	Afternoon	37,7	105	96,0	96,0	18,85	597,38	154,42
2019/05/30	CH2206	Male	Three y/o	Treatment (Wk 3)	Treatment	Feed Day	No	14:55	14:00 - 14:59	Afternoon	37,8	125	104,4	104,4	18,95	609,60	157,35
2019/05/30	CH2205	Male	Three y/o	Treatment (Wk 3)	Treatment	Feed Day	No	15:00	15:00 - 15:59	Afternoon	37,9	119	138,8	138,8	19,05	606,20	167,30
2019/05/30	CH2206	Male	Three y/o	Treatment (Wk 3)	Treatment	Feed Day	No	15:00	15:00 - 15:59	Afternoon	37,9	140	117,2	117,2	19,05	617,28	161,38
2019/05/30	CH2205	Male	Three y/o	Treatment (Wk 3)	Treatment	Feed Day	No	15:05	15:00 - 15:59	Afternoon	37,9	61	48,4	48,4	19,05	556,01	130,66
2019/05/30	CH2206	Male	Three y/o	Treatment (Wk 3)	Treatment	Feed Day	No	15:05	15:00 - 15:59	Afternoon	37,9		134,6	134,6	19,05		166,22
2019/05/30	CH2205	Male	Three y/o	Treatment (Wk 3)	Treatment	Feed Day	No	15:10	15:00 - 15:59	Afternoon	37,9	98	34,0	34,0	19,05	592,41	118,49
2019/05/30	CH2206	Male	Three y/o	Treatment (Wk 3)	Treatment	Feed Day	No	15:10	15:00 - 15:59	Afternoon	37,9	165	113,4	113,4	19,05	628,06	160,23
2019/05/30	CH2205	Male	Three y/o	Treatment (Wk 3)	Treatment	Feed Day	No	15:15	15:00 - 15:59	Afternoon	37,9	50	6,4	6,4	19,05	539,52	61,70
2019/05/30	CH2206	Male	Three y/o	Treatment (Wk 3)	Treatment	Feed Day	No	15:15	15:00 - 15:59	Afternoon	38,0	113	238,0	238,0	19,15	602,58	186,25
2019/05/30	CH2205	Male	Three y/o	Treatment (Wk 3)	Treatment	Feed Day	No	15:20	15:00 - 15:59	Afternoon	37,9	48	7,0	7,0	19,05	536,04	64,72
2019/05/30	CH2206	Male	Three y/o	Treatment (Wk 3)	Treatment	Feed Day	No	15:20	15:00 - 15:59	Afternoon	38,0	115	126,6	126,6	19,15	603,82	164,08
2019/05/30	CH2205	Male	Three y/o	Treatment (Wk 3)	Treatment	Feed Day	No	15:25	15:00 - 15:59	Afternoon	38,0	60	25,8	25,8	19,15	554,67	109,02
2019/05/30	CH2206	Male	Three y/o	Treatment (Wk 3)	Treatment	Feed Day	No	15:25	15:00 - 15:59	Afternoon	38,0	96	155,0	155,0	19,15	590,91	171,17
2019/05/30	CH2205	Male	Three y/o	Treatment (Wk 3)	Treatment	Feed Day	Yes	15:30	15:00 - 15:59	Afternoon	38,0	51	21,4	21,4	19,15	541,20	102,63
2019/05/30	CH2206	Male	Three y/o	Treatment (Wk 3)	Treatment	Feed Day	Yes	15:30	15:00 - 15:59	Afternoon	38,0	92	104,6	104,6	19,15	587,78	157,41
2019/05/30	CH2205	Male	Three y/o	Treatment (Wk 3)	Treatment	Feed Day	Yes	15:35	15:00 - 15:59	Afternoon	38,0	63	16,6	16,6	19,15	558,62	93,96
2019/05/30	CH2206	Male	Three y/o	Treatment (Wk 3)	Treatment	Feed Day	Yes	15:35	15:00 - 15:59	Afternoon	38,0	96	124,6	124,6	19,15	590,91	163,52
2019/05/30	CH2205	Male	Three y/o	Treatment (Wk 3)	Treatment	Feed Day	Yes	15:40	15:00 - 15:59	Afternoon	38,0	52	24,0	24,0	19,15	542,83	106,55
2019/05/30	CH2206	Male	Three y/o	Treatment (Wk 3)	Treatment	Feed Day	Yes	15:40	15:00 - 15:59	Afternoon	38,0	55	17,8	17,8	19,15	547,52	96,34
2019/05/30	CH2205	Male	Three y/o	Treatment (Wk 3)	Treatment	Feed Day	Yes	15:45	15:00 - 15:59	Afternoon	38,0		47,6	47,6	19,15		130,09
2019/05/30	CH2206	Male	Three y/o	Treatment (Wk 3)	Treatment	Feed Day	Yes	15:45	15:00 - 15:59	Afternoon	38,0	103	278,8	278,8	19,15	596,00	191,83
2019/05/30	CH2205	Male	Three y/o	Treatment (Wk 3)	Treatment	Feed Day	Yes	15:50	15:00 - 15:59	Afternoon	38,0	98	60,4	60,4	19,15	592,41	138,32
2019/05/30	CH2206	Male	Three y/o	Treatment (Wk 3)	Treatment	Feed Day	Yes	15:50	15:00 - 15:59	Afternoon	38,1	110	154,0	154,0	19,26	600,69	170,94
2019/05/30	CH2205	Male	Three y/o	Treatment (Wk 3)	Treatment	Feed Day	Yes	15:55	15:00 - 15:59	Afternoon	37,9	68	52,6	52,6	19,05	564,71	133,54
2019/05/30	CH2206	Male	Three y/o	Treatment (Wk 3)	Treatment	Feed Day	Yes	15:55	15:00 - 15:59	Afternoon	38,1	117	109,8	109,8	19,26	605,02	159,11
2019/05/30	CH2205	Male	Three y/o	Treatment (Wk 3)	Treatment	Feed Day	Yes	16:00	16:00 - 16:59	Evening	38,1	48	33,8	33,8	19,26	536,04	118,29
2019/05/30	CH2206	Male	Three y/o	Treatment (Wk 3)	Treatment	Feed Day	Yes	16:00	16:00 - 16:59	Evening	38,2	117	102,0	102,0	19,36	605,02	156,53
2019/05/30	CH2205	Male	Three y/o	Treatment (Wk 3)	Treatment	Feed Day	Yes	16:05	16:00 - 16:59	Evening	37,9	113	80,2	80,2	19,05	602,58	148,16

2019/05/30	CH2206	Male	Three y/o	Treatment (Wk 3)	Treatment	Feed Day	Yes	16:05	16:00 - 16:59	Evening	38,2		40,6	40,6	19,36		124,60
2019/05/30	CH2205	Male	Three y/o	Treatment (Wk 3)	Treatment	Feed Day	Yes	16:10	16:00 - 16:59	Evening	37,7	70	25,2	25,2	18,85	566,99	108,22
2019/05/30	CH2206	Male	Three y/o	Treatment (Wk 3)	Treatment	Feed Day	Yes	16:10	16:00 - 16:59	Evening	38,1	99	116,6	116,6	19,26	593,14	161,20
2019/05/30	CH2205	Male	Three y/o	Treatment (Wk 3)	Treatment	Feed Day	Yes	16:15	16:00 - 16:59	Evening	37,7		57,6	57,6	18,85		136,68
2019/05/30	CH2206	Male	Three y/o	Treatment (Wk 3)	Treatment	Feed Day	Yes	16:15	16:00 - 16:59	Evening	38,1	56	99,2	99,2	19,26	549,01	155,56
2019/05/30	CH2205	Male	Three y/o	Treatment (Wk 3)	Treatment	Feed Day	Yes	16:20	16:00 - 16:59	Evening	37,9	91	36,0	36,0	19,05	586,98	120,46
2019/05/30	CH2206	Male	Three y/o	Treatment (Wk 3)	Treatment	Feed Day	Yes	16:20	16:00 - 16:59	Evening	38,1		54,0	54,0	19,26		134,45
2019/05/30	CH2205	Male	Three y/o	Treatment (Wk 3)	Treatment	Feed Day	Yes	16:25	16:00 - 16:59	Evening	37,9		27,8	27,8	19,05		111,58
2019/05/30	CH2206	Male	Three y/o	Treatment (Wk 3)	Treatment	Feed Day	Yes	16:25	16:00 - 16:59	Evening	38,1	88	39,4	39,4	19,26	584,49	123,57
2019/05/30	CH2205	Male	Three y/o	Treatment (Wk 3)	Treatment	Feed Day	No	16:30	16:00 - 16:59	Evening	37,9	158	25,2	25,2	19,05	625,26	108,22
2019/05/30	CH2206	Male	Three y/o	Treatment (Wk 3)	Treatment	Feed Day	No	16:30	16:00 - 16:59	Evening	38,0	106	53,6	53,6	19,15	598,06	134,19
2019/05/30	CH2205	Male	Three y/o	Treatment (Wk 3)	Treatment	Feed Day	No	16:35	16:00 - 16:59	Evening	38,0		25,0	25,0	19,15		107,94
2019/05/30	CH2206	Male	Three y/o	Treatment (Wk 3)	Treatment	Feed Day	No	16:35	16:00 - 16:59	Evening	38,1	77	44,2	44,2	19,26	574,39	127,53
2019/05/30	CH2205	Male	Three y/o	Treatment (Wk 3)	Treatment	Feed Day	No	16:40	16:00 - 16:59	Evening	37,9	68	11,4	11,4	19,05	564,71	81,19
2019/05/30	CH2206	Male	Three y/o	Treatment (Wk 3)	Treatment	Feed Day	No	16:40	16:00 - 16:59	Evening	38,0	88	58,0	58,0	19,15	584,49	136,92
2019/05/30	CH2205	Male	Three y/o	Treatment (Wk 3)	Treatment	Feed Day	No	16:45	16:00 - 16:59	Evening	37,7	63	21,2	21,2	18,85	558,62	102,31
2019/05/30	CH2206	Male	Three y/o	Treatment (Wk 3)	Treatment	Feed Day	No	16:45	16:00 - 16:59	Evening	38,0	75	28,0	28,0	19,15	572,36	111,83
2019/05/30	CH2205	Male	Three y/o	Treatment (Wk 3)	Treatment	Feed Day	No	16:50	16:00 - 16:59	Evening	37,7	58	20,8	20,8	18,85	551,90	101,65
2019/05/30	CH2206	Male	Three y/o	Treatment (Wk 3)	Treatment	Feed Day	No	16:50	16:00 - 16:59	Evening	38,1	79	47,6	47,6	19,26	576,36	130,09
2019/05/30	CH2205	Male	Three y/o	Treatment (Wk 3)	Treatment	Feed Day	No	16:55	16:00 - 16:59	Evening	37,7	72	19,2	19,2	18,85	569,20	98,92
2019/05/30	CH2206	Male	Three y/o	Treatment (Wk 3)	Treatment	Feed Day	No	16:55	16:00 - 16:59	Evening	38,0	94	25,6	25,6	19,15	589,37	108,76
2019/05/30	CH2205	Male	Three y/o	Treatment (Wk 3)	Treatment	Feed Day	No	17:00	17:00 - 17:59	Evening	37,7		18,6	18,6	18,85		97,84
2019/05/30	CH2206	Male	Three y/o	Treatment (Wk 3)	Treatment	Feed Day	No	17:00	17:00 - 17:59	Evening	37,9	46	18,4	18,4	19,05	532,38	97,47
2019/05/30	CH2205	Male	Three y/o	Treatment (Wk 3)	Treatment	Feed Day	No	17:05	17:00 - 17:59	Evening	37,8	68	16,0	16,0	18,95	564,71	92,71
2019/05/30	CH2206	Male	Three y/o	Treatment (Wk 3)	Treatment	Feed Day	No	17:05	17:00 - 17:59	Evening	37,8	91	11,4	11,4	18,95	586,98	81,19
2019/05/30	CH2205	Male	Three y/o	Treatment (Wk 3)	Treatment	Feed Day	No	17:10	17:00 - 17:59	Evening	37,9	73	14,4	14,4	19,05	570,27	89,12
2019/05/30	CH2206	Male	Three y/o	Treatment (Wk 3)	Treatment	Feed Day	No	17:10	17:00 - 17:59	Evening	37,8	117	32,0	32,0	18,95	605,02	116,41
2019/05/30	CH2205	Male	Three y/o	Treatment (Wk 3)	Treatment	Feed Day	No	17:15	17:00 - 17:59	Evening	37,9	172	19,2	19,2	19,05	630,73	98,92
2019/05/30	CH2206	Male	Three y/o	Treatment (Wk 3)	Treatment	Feed Day	No	17:15	17:00 - 17:59	Evening	37,8	65	27,0	27,0	18,95	561,12	110,58
2019/05/30	CH2205	Male	Three y/o	Treatment (Wk 3)	Treatment	Feed Day	No	17:20	17:00 - 17:59	Evening	37,9	124	19,2	19,2	19,05	609,04	98,92
2019/05/30	CH2206	Male	Three y/o	Treatment (Wk 3)	Treatment	Feed Day	No	17:20	17:00 - 17:59	Evening	37,7	102	13,0	13,0	18,85	595,30	85,65
2019/05/30	CH2205	Male	Three y/o	Treatment (Wk 3)	Treatment	Feed Day	No	17:25	17:00 - 17:59	Evening	37,9		18,0	18,0	19,05		96,72
2019/05/30	CH2206	Male	Three y/o	Treatment (Wk 3)	Treatment	Feed Day	No	17:25	17:00 - 17:59	Evening	37,8	49	13,6	13,6	18,95	537,80	87,18
2019/05/30	CH2205	Male	Three y/o	Treatment (Wk 3)	Treatment	Feed Day	No	17:30	17:00 - 17:59	Evening	37,9	66	25,4	25,4	19,05	562,34	108,49
2019/05/30	CH2206	Male	Three y/o	Treatment (Wk 3)	Treatment	Feed Day	No	17:30	17:00 - 17:59	Evening	37,8	47	28,6	28,6	18,95	534,23	112,56
2019/05/30	CH2205	Male	Three y/o	Treatment (Wk 3)	Treatment	Feed Day	No	17:35	17:00 - 17:59	Evening	37,9		55,2	55,2	19,05		135,21
2019/05/30	CH2206	Male	Three y/o	Treatment (Wk 3)	Treatment	Feed Day	No	17:35	17:00 - 17:59	Evening	37,8	94	40,6	40,6	18,95	589,37	124,60
2019/05/30	CH2205	Male	Three y/o	Treatment (Wk 3)	Treatment	Feed Day	No	17:40	17:00 - 17:59	Evening	37,9	86	73,2	73,2	19,05	582,77	144,99
2019/05/30	CH2206	Male	Three y/o	Treatment (Wk 3)	Treatment	Feed Day	No	17:40	17:00 - 17:59	Evening	37,8	104	94,6	94,6	18,95	596,69	153,91
2019/05/30	CH2205	Male	Three y/o	Treatment (Wk 3)	Treatment	Feed Day	No	17:45	17:00 - 17:59	Evening	37,9	143	134,0	134,0	19,05	618,69	166,07
2019/05/30	CH2206	Male	Three y/o	Treatment (Wk 3)	Treatment	Feed Day	No	17:45	17:00 - 17:59	Evening	37,9	53	282,0	282,0	19,05	544,43	192,23
2019/05/30	CH2205	Male	Three y/o	Treatment (Wk 3)	Treatment	Feed Day	No	17:50	17:00 - 17:59	Evening	37,9	119	384,0	384,0	19,05	606,20	203,17
2019/05/30	CH2206	Male	Three y/o	Treatment (Wk 3)	Treatment	Feed Day	No	17:50	17:00 - 17:59	Evening	38,0		381,6	381,6	19,15		202,94
2019/05/30	CH2205	Male	Three y/o	Treatment (Wk 3)	Treatment	Feed Day	No	17:55	17:00 - 17:59	Evening	37,9	36	425,4	425,4	19,05	510,56	206,80
2019/05/30	CH2206	Male	Three y/o	Treatment (Wk 3)	Treatment	Feed Day	No	17:55	17:00 - 17:59	Evening	38,0	119	86,4	86,4	19,15	606,20	150,75
2019/05/30	CH2205	Male	Three y/o	Treatment (Wk 3)	Treatment	Feed Day	No	18:00	18:00 - 18:59	Evening	38,0	124	39,8	39,8	19,15	609,04	123,91
2019/05/30	CH2206	Male	Three y/o	Treatment (Wk 3)	Treatment	Feed Day	No	18:00	18:00 - 18:59	Evening	38,3	167	48,4	48,4	19,46	628,84	130,66
2019/05/30	CH2205	Male	Three y/o	Treatment (Wk 3)	Treatment	Feed Day	No	18:05	18:00 - 18:59	Evening	37,9	65	30,6	30,6	19,05	561,12	114,87
2019/05/30	CH2206	Male	Three y/o	Treatment (Wk 3)	Treatment	Feed Day	No	18:05	18:00 - 18:59	Evening	38,3	90	31,8	31,8	19,46	586,16	116,20
2019/05/30	CH2205	Male	Three y/o	Treatment (Wk 3)	Treatment	Feed Day	No	18:10	18:00 - 18:59	Evening	37,7	74	31,0	31,0	18,85	571,33	115,32
2019/05/30	CH2206	Male	Three y/o	Treatment (Wk 3)	Treatment	Feed Day	No	18:10	18:00 - 18:59	Evening	38,3	119	48,6	48,6	19,46	606,20	130,81
2019/05/30	CH2205	Male	Three y/o	Treatment (Wk 3)	Treatment	Feed Day	No	18:15	18:00 - 18:59	Evening	38,0	53	31,2	31,2	19,15	544,43	115,54
2019/05/30	CH2206	Male	Three y/o	Treatment (Wk 3)	Treatment	Feed Day	No	18:15	18:00 - 18:59	Evening	38,1	88	48,8	48,8	19,26	584,49	130,95
2019/05/30	CH2205	Male	Three y/o	Treatment (Wk 3)	Treatment	Feed Day	No	18:20	18:00 - 18:59	Evening	38,0	85	34,4	34,4	19,15	581,90	118,90
2019/05/30	CH2206	Male	Three y/o	Treatment (Wk 3)	Treatment	Feed Day	No	18:20	18:00 - 18:59	Evening	38,2	90	43,0	43,0	19,36	586,16	126,58
2019/05/30	CH2205	Male	Three y/o	Treatment (Wk 3)	Treatment	Feed Day	No	18:25	18:00 - 18:59	Evening	38,1	71	30,8	30,8	19,26	568,10	115,10
2019/05/30	CH2206	Male	Three y/o	Treatment (Wk 3)	Treatment	Feed Day	No	18:25	18:00 - 18:59	Evening	38,2	88	42,4	42,4	19,36	584,49	126,10
2019/05/30	CH2205	Male	Three y/o	Treatment (Wk 3)	Treatment	Feed Day	No	18:30	18:00 - 18:59	Evening	38,1	68	30,0	30,0	19,26	564,71	114,20
2019/05/30	CH2206	Male	Three y/o	Treatment (Wk 3)	Treatment	Feed Day	No	18:30	18:00 - 18:59	Evening	38,2	65	43,6	43,6	19,36	561,12	127,06
2019/05/30	CH2205	Male	Three y/o	Treatment (Wk 3)	Treatment	Feed Day	No	18:35	18:00 - 18:59	Evening	38,2	86	33,8	33,8	19,36	582,77	118,29
2019/05/30	CH2206	Male	Three y/o	Treatment (Wk 3)	Treatment	Feed Day	No	18:35	18:00 - 18:59	Evening	38,3	79	48,2	48,2	19,46	576,36	130,52
2019/05/30	CH2205	Male	Three y/o	Treatment (Wk 3)	Treatment	Feed Day	No	18:40	18:00 - 18:59	Evening	38,2	64	24,6	24,6	19,36	559,88	107,39
2019/05/30	CH2206	Male	Three y/o	Treatment (Wk 3)	Treatment	Feed Day	No	18:40	18:00 - 18:59	Evening	38,3	76	46,6	46,6	19,46	573,39	129,35
2019/05/30	CH2205	Male	Three y/o	Treatment (Wk 3)	Treatment	Feed Day	No	18:45	18:00 - 18:59	Evening	38,2	91	23,8	23,8	19,36	586,98	106,26
2019/05/30	CH2206	Male	Three y/o	Treatment (Wk 3)	Treatment	Feed Day	No	18:45	18:00 - 18:59	Evening	38,3	68	50,0	50,0	19,46	564,71	131,79
2019/05/30	CH2205	Male	Three y/o	Treatment (Wk 3)	Treatment	Feed Day	No	18:50	18:00 - 18:59	Evening	38,2	68	31,2	31,2	19,36	564,71	115,54
2019/05/30	CH2206	Male	Three y/o	Treatment (Wk 3)	Treatment	Feed Day	No	18:50	18:00 - 18:59	Evening	38,3	77	47,6	47,6	19,46	574,39	130,09
2019/05/30	CH2205	Male	Three y/o	Treatment (Wk 3)	Treatment	Feed Day	No	18:55	18:00 - 18:59	Evening	38,2	89	31,2	31,2	19,36	585,33	115,54
2019/05/30	CH2206	Male	Three y/o	Treatment (Wk 3)	Treatment	Feed Day	No	18:55	18:00 - 18:59	Evening	38,3	85	47,2	47,2	19,46	581,90	129,80
2019/05/30	CH2205	Male	Three y/o	Treatment (Wk 3)	Treatment	Feed Day	No	19:00	19:00 - 19:59	Evening	38,2	62	31,4	31,4	19,36	557,33	115,76

2019/05/30	CH2206	Male	Three y/o	Treatment (Wk 3)	Treatment	Feed Day	No	19:00	19:00 - 19:59	Evening	38,3	72	46,4	46,4	19,46	569,20	129,21
2019/05/30	CH2205	Male	Three y/o	Treatment (Wk 3)	Treatment	Feed Day	No	19:05	19:00 - 19:59	Evening	38,2	87	31,2	31,2	19,36	583,64	115,54
2019/05/30	CH2206	Male	Three y/o	Treatment (Wk 3)	Treatment	Feed Day	No	19:05	19:00 - 19:59	Evening	38,3	85	52,4	52,4	19,46	581,90	133,41
2019/05/30	CH2205	Male	Three y/o	Treatment (Wk 3)	Treatment	Feed Day	No	19:10	19:00 - 19:59	Evening	38,2		29,2	29,2	19,36		113,27
2019/05/30	CH2206	Male	Three y/o	Treatment (Wk 3)	Treatment	Feed Day	No	19:10	19:00 - 19:59	Evening	38,3	50	43,6	43,6	19,46	539,52	127,06
2019/05/30	CH2205	Male	Three y/o	Treatment (Wk 3)	Treatment	Feed Day	No	19:15	19:00 - 19:59	Evening	38,1	97	26,8	26,8	19,26	591,66	110,33
2019/05/30	CH2206	Male	Three y/o	Treatment (Wk 3)	Treatment	Feed Day	No	19:15	19:00 - 19:59	Evening	38,2	82	51,0	51,0	19,36	579,19	132,47
2019/05/30	CH2205	Male	Three y/o	Treatment (Wk 3)	Treatment	Feed Day	No	19:20	19:00 - 19:59	Evening	38,1	62	27,8	27,8	19,26	557,33	111,58
2019/05/30	CH2206	Male	Three y/o	Treatment (Wk 3)	Treatment	Feed Day	No	19:20	19:00 - 19:59	Evening	38,2	74	26,8	26,8	19,36	571,33	110,33
2019/05/30	CH2205	Male	Three y/o	Treatment (Wk 3)	Treatment	Feed Day	No	19:25	19:00 - 19:59	Evening	38,0	120	18,2	18,2	19,15	606,78	97,10
2019/05/30	CH2206	Male	Three y/o	Treatment (Wk 3)	Treatment	Feed Day	No	19:25	19:00 - 19:59	Evening	38,2	46	43,4	43,4	19,36	532,38	126,90
2019/05/30	CH2205	Male	Three y/o	Treatment (Wk 3)	Treatment	Feed Day	No	19:30	19:00 - 19:59	Evening	38,0	50	5,8	5,8	19,15	539,52	58,39
2019/05/30	CH2206	Male	Three y/o	Treatment (Wk 3)	Treatment	Feed Day	No	19:30	19:00 - 19:59	Evening	38,2	73	39,8	39,8	19,36	570,27	123,91
2019/05/30	CH2205	Male	Three y/o	Treatment (Wk 3)	Treatment	Feed Day	No	19:35	19:00 - 19:59	Evening	37,9	62	7,2	7,2	19,05	557,33	65,66
2019/05/30	CH2206	Male	Three y/o	Treatment (Wk 3)	Treatment	Feed Day	No	19:35	19:00 - 19:59	Evening	38,2	91	25,0	25,0	19,36	586,98	107,94
2019/05/30	CH2205	Male	Three y/o	Treatment (Wk 3)	Treatment	Feed Day	No	19:40	19:00 - 19:59	Evening	37,7	68	6,4	6,4	18,85	564,71	61,70
2019/05/30	CH2206	Male	Three y/o	Treatment (Wk 3)	Treatment	Feed Day	No	19:40	19:00 - 19:59	Evening	38,2	73	37,0	37,0	19,36	570,27	121,40
2019/05/30	CH2205	Male	Three y/o	Treatment (Wk 3)	Treatment	Feed Day	No	19:45	19:00 - 19:59	Evening	37,6	65	5,6	5,6	18,75	561,12	57,21
2019/05/30	CH2206	Male	Three y/o	Treatment (Wk 3)	Treatment	Feed Day	No	19:45	19:00 - 19:59	Evening	38,1	76	26,4	26,4	19,26	573,39	109,81
2019/05/30	CH2205	Male	Three y/o	Treatment (Wk 3)	Treatment	Feed Day	No	19:50	19:00 - 19:59	Evening	37,6	57	1,8	1,8	18,75	550,47	19,38
2019/05/30	CH2206	Male	Three y/o	Treatment (Wk 3)	Treatment	Feed Day	No	19:50	19:00 - 19:59	Evening	38,1	96	27,2	27,2	19,26	590,91	110,83
2019/05/30	CH2205	Male	Three y/o	Treatment (Wk 3)	Treatment	Feed Day	No	19:55	19:00 - 19:59	Evening	37,6	47	6,8	6,8	18,75	534,23	63,74
2019/05/30	CH2206	Male	Three y/o	Treatment (Wk 3)	Treatment	Feed Day	No	19:55	19:00 - 19:59	Evening	38,1	69	26,4	26,4	19,26	565,86	109,81
2019/05/30	CH2205	Male	Three y/o	Treatment (Wk 3)	Treatment	Feed Day	No	20:00	20:00 - 20:59	Night	37,6	62	6,8	6,8	18,75	557,33	63,74
2019/05/30	CH2206	Male	Three y/o	Treatment (Wk 3)	Treatment	Feed Day	No	20:00	20:00 - 20:59	Night	38,1	48	25,6	25,6	19,26	536,04	108,76
2019/05/30	CH2205	Male	Three y/o	Treatment (Wk 3)	Treatment	Feed Day	No	20:05	20:00 - 20:59	Night	37,5	57	4,8	4,8	18,65	550,47	52,04
2019/05/30	CH2206	Male	Three y/o	Treatment (Wk 3)	Treatment	Feed Day	No	20:05	20:00 - 20:59	Night	38,2	77	24,4	24,4	19,36	574,39	107,11
2019/05/30	CH2205	Male	Three y/o	Treatment (Wk 3)	Treatment	Feed Day	No	20:10	20:00 - 20:59	Night	37,6	48	4,2	4,2	18,75	536,04	47,57
2019/05/30	CH2206	Male	Three y/o	Treatment (Wk 3)	Treatment	Feed Day	No	20:10	20:00 - 20:59	Night	38,2	80	24,8	24,8	19,36	577,31	107,67
2019/05/30	CH2205	Male	Three y/o	Treatment (Wk 3)	Treatment	Feed Day	No	20:15	20:00 - 20:59	Night	37,6	110	5,0	5,0	18,75	600,69	53,41
2019/05/30	CH2206	Male	Three y/o	Treatment (Wk 3)	Treatment	Feed Day	No	20:15	20:00 - 20:59	Night	38,3	73	25,2	25,2	19,46	570,27	108,22
2019/05/30	CH2205	Male	Three y/o	Treatment (Wk 3)	Treatment	Feed Day	No	20:20	20:00 - 20:59	Night	37,9	95	12,8	12,8	19,05	590,14	85,12
2019/05/30	CH2206	Male	Three y/o	Treatment (Wk 3)	Treatment	Feed Day	No	20:20	20:00 - 20:59	Night	38,2	69	22,8	22,8	19,36	565,86	104,79
2019/05/30	CH2205	Male	Three y/o	Treatment (Wk 3)	Treatment	Feed Day	No	20:25	20:00 - 20:59	Night	38,1	86	9,4	9,4	19,26	582,77	74,66
2019/05/30	CH2206	Male	Three y/o	Treatment (Wk 3)	Treatment	Feed Day	No	20:25	20:00 - 20:59	Night	38,2	92	22,4	22,4	19,36	587,78	104,19
2019/05/30	CH2205	Male	Three y/o	Treatment (Wk 3)	Treatment	Feed Day	No	20:30	20:00 - 20:59	Night	38,1	69	4,6	4,6	19,26	565,86	50,61
2019/05/30	CH2206	Male	Three y/o	Treatment (Wk 3)	Treatment	Feed Day	No	20:30	20:00 - 20:59	Night	38,2	46	23,6	23,6	19,36	532,38	105,97
2019/05/30	CH2205	Male	Three y/o	Treatment (Wk 3)	Treatment	Feed Day	No	20:35	20:00 - 20:59	Night	38,2	81	6,2	6,2	19,36	578,26	60,63
2019/05/30	CH2206	Male	Three y/o	Treatment (Wk 3)	Treatment	Feed Day	No	20:35	20:00 - 20:59	Night	38,2	73	27,8	27,8	19,36	570,27	111,58
2019/05/30	CH2205	Male	Three y/o	Treatment (Wk 3)	Treatment	Feed Day	No	20:40	20:00 - 20:59	Night	38,2	59	6,6	6,6	19,36	553,30	62,73
2019/05/30	CH2206	Male	Three y/o	Treatment (Wk 3)	Treatment	Feed Day	No	20:40	20:00 - 20:59	Night	38,2	59	19,8	19,8	19,36	553,30	99,97
2019/05/30	CH2205	Male	Three y/o	Treatment (Wk 3)	Treatment	Feed Day	No	20:45	20:00 - 20:59	Night	38,2	73	10,6	10,6	19,36	570,27	78,73
2019/05/30	CH2206	Male	Three y/o	Treatment (Wk 3)	Treatment	Feed Day	No	20:45	20:00 - 20:59	Night	38,1	110	28,4	28,4	19,26	600,69	112,31
2019/05/30	CH2205	Male	Three y/o	Treatment (Wk 3)	Treatment	Feed Day	No	20:50	20:00 - 20:59	Night	38,3	103	21,0	21,0	19,46	596,00	101,98
2019/05/30	CH2206	Male	Three y/o	Treatment (Wk 3)	Treatment	Feed Day	No	20:50	20:00 - 20:59	Night	38,1	98	19,2	19,2	19,26	592,41	98,92
2019/05/30	CH2205	Male	Three y/o	Treatment (Wk 3)	Treatment	Feed Day	No	20:55	20:00 - 20:59	Night	38,2	97	22,8	22,8	19,36	591,66	104,79
2019/05/30	CH2206	Male	Three y/o	Treatment (Wk 3)	Treatment	Feed Day	No	20:55	20:00 - 20:59	Night	38,0	69	44,6	44,6	19,15	565,86	127,84
2019/05/30	CH2205	Male	Three y/o	Treatment (Wk 3)	Treatment	Feed Day	No	21:00	21:00 - 21:59	Night	38,2	101	27,4	27,4	19,36	594,59	111,09
2019/05/30	CH2206	Male	Three y/o	Treatment (Wk 3)	Treatment	Feed Day	No	21:00	21:00 - 21:59	Night	38,0	107	13,2	13,2	19,15	598,73	86,17
2019/05/30	CH2205	Male	Three y/o	Treatment (Wk 3)	Treatment	Feed Day	No	21:05	21:00 - 21:59	Night	38,2	47	17,0	17,0	19,36	534,23	94,77
2019/05/30	CH2206	Male	Three y/o	Treatment (Wk 3)	Treatment	Feed Day	No	21:05	21:00 - 21:59	Night	38,0	89	37,2	37,2	19,15	585,33	121,59
2019/05/30	CH2205	Male	Three y/o	Treatment (Wk 3)	Treatment	Feed Day	No	21:10	21:00 - 21:59	Night	38,1	92	20,2	20,2	19,26	587,78	100,65
2019/05/30	CH2206	Male	Three y/o	Treatment (Wk 3)	Treatment	Feed Day	No	21:10	21:00 - 21:59	Night	37,9	92	40,4	40,4	19,05	587,78	124,43
2019/05/30	CH2205	Male	Three y/o	Treatment (Wk 3)	Treatment	Feed Day	No	21:15	21:00 - 21:59	Night	38,1	105	22,6	22,6	19,26	597,38	104,49
2019/05/30	CH2206	Male	Three y/o	Treatment (Wk 3)	Treatment	Feed Day	No	21:15	21:00 - 21:59	Night	38,0	46	37,6	37,6	19,15	532,38	121,96
2019/05/30	CH2205	Male	Three y/o	Treatment (Wk 3)	Treatment	Feed Day	No	21:20	21:00 - 21:59	Night	38,1	63	21,0	21,0	19,26	558,62	101,98
2019/05/30	CH2206	Male	Three y/o	Treatment (Wk 3)	Treatment	Feed Day	No	21:20	21:00 - 21:59	Night	38,0	93	32,2	32,2	19,15	588,58	116,63
2019/05/30	CH2205	Male	Three y/o	Treatment (Wk 3)	Treatment	Feed Day	No	21:25	21:00 - 21:59	Night	38,1	51	20,0	20,0	19,26	541,20	100,31
2019/05/30	CH2206	Male	Three y/o	Treatment (Wk 3)	Treatment	Feed Day	No	21:25	21:00 - 21:59	Night	38,0	65	35,6	35,6	19,15	561,12	120,08
2019/05/30	CH2205	Male	Three y/o	Treatment (Wk 3)	Treatment	Feed Day	No	21:30	21:00 - 21:59	Night	38,0	66	16,8	16,8	19,15	562,34	94,37
2019/05/30	CH2206	Male	Three y/o	Treatment (Wk 3)	Treatment	Feed Day	No	21:30	21:00 - 21:59	Night	38,0	46	34,6	34,6	19,15	532,38	119,10
2019/05/30	CH2205	Male	Three y/o	Treatment (Wk 3)	Treatment	Feed Day	No	21:35	21:00 - 21:59	Night	38,0	110	23,4	23,4	19,15	600,69	105,68
2019/05/30	CH2206	Male	Three y/o	Treatment (Wk 3)	Treatment	Feed Day	No	21:35	21:00 - 21:59	Night	38,1	46	24,8	24,8	19,26	532,38	107,67
2019/05/30	CH2205	Male	Three y/o	Treatment (Wk 3)	Treatment	Feed Day	No	21:40	21:00 - 21:59	Night	38,0	95	26,4	26,4	19,15	590,14	109,81
2019/05/30	CH2206	Male	Three y/o	Treatment (Wk 3)	Treatment	Feed Day	No	21:40	21:00 - 21:59	Night	38,1	92	27,6	27,6	19,26	587,78	111,33
2019/05/30	CH2205	Male	Three y/o	Treatment (Wk 3)	Treatment	Feed Day	No	21:45	21:00 - 21:59	Night	38,0	70	28,0	28,0	19,15	566,99	111,83
2019/05/30	CH2206	Male	Three y/o	Treatment (Wk 3)	Treatment	Feed Day	No	21:45	21:00 - 21:59	Night	38,1	52	23,4	23,4	19,26	542,83	105,68
2019/05/30	CH2205	Male	Three y/o	Treatment (Wk 3)	Treatment	Feed Day	No	21:50	21:00 - 21:59	Night	38,0	108	12,2	12,2	19,15	599,39	83,49
2019/05/30	CH2206	Male	Three y/o	Treatment (Wk 3)	Treatment	Feed Day	No	21:50	21:00 - 21:59	Night	38,1	71	28,8	28,8	19,26	568,10	112,79
2019/05/30	CH2205	Male	Three y/o	Treatment (Wk 3)	Treatment	Feed Day	No	21:55	21:00 - 21:59	Night	38,0	47	25,4	25,4	19,15	534,23	108,49

	2019/05/30	CH2206	Male	Three y/o	Treatment (Wk 3)	Treatment	Feed Day	No	21:55	21:00 - 21:59	Night	38,1	73	41,6	41,6	19,26	570,27	125,44
	2019/05/30	CH2205	Male	Three y/o	Treatment (Wk 3)	Treatment	Feed Day	No	22:00	22:00 - 22:59	Night	37,9	53	25,2	25,2	19,05	544,43	108,22
	2019/05/30	CH2206	Male	Three y/o	Treatment (Wk 3)	Treatment	Feed Day	No	22:00	22:00 - 22:59	Night	38,1	45	30,0	30,0	19,26	530,47	114,20
	2019/05/30	CH2205	Male	Three y/o	Treatment (Wk 3)	Treatment	Feed Day	No	22:05	22:00 - 22:59	Night	37,6	71	20,4	20,4	18,75	568,10	100,99
	2019/05/30	CH2206	Male	Three y/o	Treatment (Wk 3)	Treatment	Feed Day	No	22:05	22:00 - 22:59	Night	38,1	96	24,6	24,6	19,26	590,91	107,39
	2019/05/30	CH2205	Male	Three y/o	Treatment (Wk 3)	Treatment	Feed Day	No	22:10	22:00 - 22:59	Night	37,4		22,4	22,4	18,55		104,19
	2019/05/30	CH2206	Male	Three y/o	Treatment (Wk 3)	Treatment	Feed Day	No	22:10	22:00 - 22:59	Night	38,1	95	28,8	28,8	19,26	590,14	112,79
	2019/05/30	CH2205	Male	Three y/o	Treatment (Wk 3)	Treatment	Feed Day	No	22:15	22:00 - 22:59	Night	37,4	92	17,8	17,8	18,55	587,78	96,34
	2019/05/30	CH2206	Male	Three y/o	Treatment (Wk 3)	Treatment	Feed Day	No	22:15	22:00 - 22:59	Night	38,0	90	27,0	27,0	19,15	586,16	110,58
	2019/05/30	CH2205	Male	Three y/o	Treatment (Wk 3)	Treatment	Feed Day	No	22:20	22:00 - 22:59	Night	37,3	73	20,6	20,6	18,46	570,27	101,32
	2019/05/30	CH2206	Male	Three y/o	Treatment (Wk 3)	Treatment	Feed Day	No	22:20	22:00 - 22:59	Night	38,0	67	28,0	28,0	19,15	563,53	111,83
	2019/05/30	CH2205	Male	Three y/o	Treatment (Wk 3)	Treatment	Feed Day	No	22:25	22:00 - 22:59	Night	37,3	69	9,4	9,4	18,46	565,86	74,66
	2019/05/30	CH2206	Male	Three y/o	Treatment (Wk 3)	Treatment	Feed Day	No	22:25	22:00 - 22:59	Night	38,0	103	29,4	29,4	19,15	596,00	113,50
	2019/05/30	CH2205	Male	Three y/o	Treatment (Wk 3)	Treatment	Feed Day	No	22:30	22:00 - 22:59	Night	37,6	73	10,0	10,0	18,75	570,27	76,76
	2019/05/30	CH2206	Male	Three y/o	Treatment (Wk 3)	Treatment	Feed Day	No	22:30	22:00 - 22:59	Night	38,0	71	25,8	25,8	19,15	568,10	109,02
	2019/05/30	CH2205	Male	Three y/o	Treatment (Wk 3)	Treatment	Feed Day	No	22:35	22:00 - 22:59	Night	37,7	48	13,2	13,2	18,85	536,04	86,17
	2019/05/30	CH2206	Male	Three y/o	Treatment (Wk 3)	Treatment	Feed Day	No	22:35	22:00 - 22:59	Night	38,0	53	26,8	26,8	19,15	544,43	110,33
	2019/05/30	CH2205	Male	Three y/o	Treatment (Wk 3)	Treatment	Feed Day	No	22:40	22:00 - 22:59	Night	37,9	46	11,0	11,0	19,05	532,38	79,98
	2019/05/30	CH2206	Male	Three y/o	Treatment (Wk 3)	Treatment	Feed Day	No	22:40	22:00 - 22:59	Night	38,0	98	35,4	35,4	19,15	592,41	119,88
	2019/05/30	CH2205	Male	Three y/o	Treatment (Wk 3)	Treatment	Feed Day	No	22:45	22:00 - 22:59	Night	38,0	103	13,6	13,6	19,15	596,00	87,18
	2019/05/30	CH2206	Male	Three y/o	Treatment (Wk 3)	Treatment	Feed Day	No	22:45	22:00 - 22:59	Night	38,0	76	26,0	26,0	19,15	573,39	109,29
	2019/05/30	CH2205	Male	Three y/o	Treatment (Wk 3)	Treatment	Feed Day	No	22:50	22:00 - 22:59	Night	38,0	68	8,0	8,0	19,15	564,71	69,22
	2019/05/30	CH2206	Male	Three y/o	Treatment (Wk 3)	Treatment	Feed Day	No	22:50	22:00 - 22:59	Night	37,9	94	31,0	31,0	19,05	589,37	115,32
	2019/05/30	CH2205	Male	Three y/o	Treatment (Wk 3)	Treatment	Feed Day	No	22:55	22:00 - 22:59	Night	38,1	68	7,4	7,4	19,26	564,71	66,59
	2019/05/30	CH2206	Male	Three y/o	Treatment (Wk 3)	Treatment	Feed Day	No	22:55	22:00 - 22:59	Night	37,9	92	26,0	26,0	19,05	587,78	109,29
	2019/05/30	CH2205	Male	Three y/o	Treatment (Wk 3)	Treatment	Feed Day	No	23:00	23:00 - 23:59	Night	38,1	48	6,2	6,2	19,26	536,04	60,63
	2019/05/30	CH2206	Male	Three y/o	Treatment (Wk 3)	Treatment	Feed Day	No	23:00	23:00 - 23:59	Night	37,9	46	22,4	22,4	19,05	532,38	104,19
	2019/05/30	CH2205	Male	Three y/o	Treatment (Wk 3)	Treatment	Feed Day	No	23:05	23:00 - 23:59	Night	38,1	102	9,2	9,2	19,26	595,30	73,94
	2019/05/30	CH2206	Male	Three y/o	Treatment (Wk 3)	Treatment	Feed Day	No	23:05	23:00 - 23:59	Night	37,9	74	18,4	18,4	19,05	571,33	97,47
	2019/05/30	CH2205	Male	Three y/o	Treatment (Wk 3)	Treatment	Feed Day	No	23:10	23:00 - 23:59	Night	38,1	79	9,2	9,2	19,26	576,36	73,94
	2019/05/30	CH2206	Male	Three y/o	Treatment (Wk 3)	Treatment	Feed Day	No	23:10	23:00 - 23:59	Night	37,8	96	10,0	10,0	18,95	590,91	76,76
	2019/05/30	CH2205	Male	Three y/o	Treatment (Wk 3)	Treatment	Feed Day	No	23:15	23:00 - 23:59	Night	38,1	102	9,4	9,4	19,26	595,30	74,66
	2019/05/30	CH2206	Male	Three y/o	Treatment (Wk 3)	Treatment	Feed Day	No	23:15	23:00 - 23:59	Night	37,8	63	33,2	33,2	18,95	558,62	117,68
	2019/05/30	CH2205	Male	Three y/o	Treatment (Wk 3)	Treatment	Feed Day	No	23:20	23:00 - 23:59	Night	38,1	49	4,8	4,8	19,26	537,80	52,04
	2019/05/30	CH2206	Male	Three y/o	Treatment (Wk 3)	Treatment	Feed Day	No	23:20	23:00 - 23:59	Night	37,7	73	33,4	33,4	18,85	570,27	117,88
	2019/05/30	CH2205	Male	Three y/o	Treatment (Wk 3)	Treatment	Feed Day	No	23:25	23:00 - 23:59	Night	38,1	68	7,8	7,8	19,26	564,71	68,36
	2019/05/30	CH2206	Male	Three y/o	Treatment (Wk 3)	Treatment	Feed Day	No	23:25	23:00 - 23:59	Night	37,7	97	33,4	33,4	18,85	591,66	117,88
	2019/05/30	CH2205	Male	Three y/o	Treatment (Wk 3)	Treatment	Feed Day	No	23:30	23:00 - 23:59	Night	38,1	113	2,8	2,8	19,26	602,58	34,04
	2019/05/30	CH2206	Male	Three y/o	Treatment (Wk 3)	Treatment	Feed Day	No	23:30	23:00 - 23:59	Night	37,8	54	16,6	16,6	18,95	545,99	93,96
	2019/05/30	CH2205	Male	Three y/o	Treatment (Wk 3)	Treatment	Feed Day	No	23:35	23:00 - 23:59	Night	38,1	74	8,8	8,8	19,26	571,33	72,43
	2019/05/30	CH2206	Male	Three y/o	Treatment (Wk 3)	Treatment	Feed Day	No	23:35	23:00 - 23:59	Night	37,8	41	6,0	6,0	18,95	522,29	59,53
	2019/05/30	CH2205	Male	Three y/o	Treatment (Wk 3)	Treatment	Feed Day	No	23:40	23:00 - 23:59	Night	38,1	71	2,0	2,0	19,26	568,10	22,86
	2019/05/30	CH2206	Male	Three y/o	Treatment (Wk 3)	Treatment	Feed Day	No	23:40	23:00 - 23:59	Night	37,8	54	37,2	37,2	18,95	545,99	121,59
	2019/05/30	CH2205	Male	Three y/o	Treatment (Wk 3)	Treatment	Feed Day	No	23:45	23:00 - 23:59	Night	38,1	70	7,0	7,0	19,26	566,99	64,72
	2019/05/30	CH2206	Male	Three y/o	Treatment (Wk 3)	Treatment	Feed Day	No	23:45	23:00 - 23:59	Night	37,8	93	39,0	39,0	18,95	588,58	123,22
	2019/05/30	CH2205	Male	Three y/o	Treatment (Wk 3)	Treatment	Feed Day	No	23:50	23:00 - 23:59	Night	38,1	107	7,6	7,6	19,26	598,73	67,49
	2019/05/30	CH2206	Male	Three y/o	Treatment (Wk 3)	Treatment	Feed Day	No	23:50	23:00 - 23:59	Night	37,8	98	32,4	32,4	18,95	592,41	116,84
	2019/05/30	CH2205	Male	Three y/o	Treatment (Wk 3)	Treatment	Feed Day	No	23:55	23:00 - 23:59	Night	38,1	84	3,2	3,2	19,26	581,01	38,49
	2019/05/30	CH2206	Male	Three y/o	Treatment (Wk 3)	Treatment	Feed Day	No	23:55	23:00 - 23:59	Night	37,8	85	36,0	36,0	18,95	581,90	120,46
17	2019/05/31	CH2205	Male	Three y/o	Treatment (Wk 3)	Treatment	Feed Day	No	00:00	00:00 - 00:59	Early Morning	38,1	112	24,0	24,0	19,26	601,96	106,55
	2019/05/31	CH2206	Male	Three y/o	Treatment (Wk 3)	Treatment	Feed Day	No	00:00	00:00 - 00:59	Early Morning	37,8	98	34,8	34,8	18,95	592,41	119,29
	2019/05/31	CH2205	Male	Three y/o	Treatment (Wk 3)	Treatment	Feed Day	No	00:05	00:00 - 00:59	Early Morning	38,1	107	25,4	25,4	19,26	598,73	108,49
	2019/05/31	CH2206	Male	Three y/o	Treatment (Wk 3)	Treatment	Feed Day	No	00:05	00:00 - 00:59	Early Morning	37,8	92	31,8	31,8	18,95	587,78	116,20
	2019/05/31	CH2205	Male	Three y/o	Treatment (Wk 3)	Treatment	Feed Day	No	00:10	00:00 - 00:59	Early Morning	37,9	60	21,2	21,2	19,05	554,67	102,31
	2019/05/31	CH2206	Male	Three y/o	Treatment (Wk 3)	Treatment	Feed Day	No	00:10	00:00 - 00:59	Early Morning	37,9	60	32,0	32,0	19,05	554,67	116,41
	2019/05/31	CH2205	Male	Three y/o	Treatment (Wk 3)	Treatment	Feed Day	No	00:15	00:00 - 00:59	Early Morning	37,7	99	23,4	23,4	18,85	593,14	105,68
	2019/05/31	CH2206	Male	Three y/o	Treatment (Wk 3)	Treatment	Feed Day	No	00:15	00:00 - 00:59	Early Morning	37,8		30,0	30,0	18,95		114,20
	2019/05/31	CH2205	Male	Three y/o	Treatment (Wk 3)	Treatment	Feed Day	No	00:20	00:00 - 00:59	Early Morning	37,6	90	25,0	25,0	18,75	586,16	107,94
	2019/05/31	CH2206	Male	Three y/o	Treatment (Wk 3)	Treatment	Feed Day	No	00:20	00:00 - 00:59	Early Morning	37,8	90	11,8	11,8	18,95	586,16	82,36
	2019/05/31	CH2205	Male	Three y/o	Treatment (Wk 3)	Treatment	Feed Day	No	00:25	00:00 - 00:59	Early Morning	37,6	45	25,4	25,4	18,75	530,47	108,49
	2019/05/31	CH2206	Male	Three y/o	Treatment (Wk 3)	Treatment	Feed Day	No	00:25	00:00 - 00:59	Early Morning	37,8	84	2,2	2,2	18,95	581,01	26,02
	2019/05/31	CH2205	Male	Three y/o	Treatment (Wk 3)	Treatment	Feed Day	No	00:30	00:00 - 00:59	Early Morning	37,6	83	19,8	19,8	18,75	580,11	99,97
	2019/05/31	CH2206	Male	Three y/o	Treatment (Wk 3)	Treatment	Feed Day	No	00:30	00:00 - 00:59	Early Morning	37,8	51	2,2	2,2	18,95	541,20	26,02
	2019/05/31	CH2205	Male	Three y/o	Treatment (Wk 3)	Treatment	Feed Day	No	00:35	00:00 - 00:59	Early Morning	37,9	79	19,4	19,4	19,05	576,36	99,28
	2019/05/31	CH2206	Male	Three y/o	Treatment (Wk 3)	Treatment	Feed Day	No	00:35	00:00 - 00:59	Early Morning	37,8	46	15,4	15,4	18,95	532,38	91,41
	2019/05/31	CH2205	Male	Three y/o	Treatment (Wk 3)	Treatment	Feed Day	No	00:40	00:00 - 00:59	Early Morning	38,0	62	21,4	21,4	19,15	557,33	102,63
	2019/05/31	CH2206	Male	Three y/o	Treatment (Wk 3)	Treatment	Feed Day	No	00:40	00:00 - 00:59	Early Morning	37,8	86	18,4	18,4	18,95	582,77	97,47
	2019/05/31	CH2205	Male	Three y/o	Treatment (Wk 3)	Treatment	Feed Day	No	00:45	00:00 - 00:59	Early Morning	38,0	50	18,8	18,8	19,15	539,52	98,20
	2019/05/31	CH2206	Male	Three y/o	Treatment (Wk 3)	Treatment	Feed Day	No	00:45	00:00 - 00:59	Early Morning	37,8	85	9,4	9,4	18,95	581,90	74,66
	2019/05/31	CH2205	Male	Three y/o	Treatment (Wk 3)	Treatment	Feed Day	No	00:50	00:00 - 00:59	Early Morning	38,0	82	23,2	23,2	19,15	579,19	105,39

2019/05/31	CH2206	Male	Three y/o	Treatment (Wk 3)	Treatment	Feed Day	No	00:50	00:00 - 00:59	Early Morning	37,8	90	9,8	9,8	18,95	586,16	76,07
2019/05/31	CH2205	Male	Three y/o	Treatment (Wk 3)	Treatment	Feed Day	No	00:55	00:00 - 00:59	Early Morning	38,1	99	21,4	21,4	19,26	593,14	102,63
2019/05/31	CH2206	Male	Three y/o	Treatment (Wk 3)	Treatment	Feed Day	No	00:55	00:00 - 00:59	Early Morning	37,7	43	11,4	11,4	18,85	526,50	81,19
2019/05/31	CH2205	Male	Three y/o	Treatment (Wk 3)	Treatment	Feed Day	No	01:00	01:00 - 01:59	Early Morning	38,0	100	22,8	22,8	19,15	593,87	104,79
2019/05/31	CH2206	Male	Three y/o	Treatment (Wk 3)	Treatment	Feed Day	No	01:00	01:00 - 01:59	Early Morning	37,7	105	27,6	27,6	18,85	597,38	111,33
2019/05/31	CH2205	Male	Three y/o	Treatment (Wk 3)	Treatment	Feed Day	No	01:05	01:00 - 01:59	Early Morning	38,0	57	19,0	19,0	19,15	550,47	98,56
2019/05/31	CH2206	Male	Three y/o	Treatment (Wk 3)	Treatment	Feed Day	No	01:05	01:00 - 01:59	Early Morning	37,7	73	24,2	24,2	18,85	570,27	106,83
2019/05/31	CH2205	Male	Three y/o	Treatment (Wk 3)	Treatment	Feed Day	No	01:10	01:00 - 01:59	Early Morning	38,0	101	25,8	25,8	19,15	594,59	109,02
2019/05/31	CH2206	Male	Three y/o	Treatment (Wk 3)	Treatment	Feed Day	No	01:10	01:00 - 01:59	Early Morning	37,7	55	29,4	29,4	18,85	547,52	113,50
2019/05/31	CH2205	Male	Three y/o	Treatment (Wk 3)	Treatment	Feed Day	No	01:15	01:00 - 01:59	Early Morning	38,0	135	27,4	27,4	19,15	614,84	111,09
2019/05/31	CH2206	Male	Three y/o	Treatment (Wk 3)	Treatment	Feed Day	No	01:15	01:00 - 01:59	Early Morning	37,8	119	102,6	102,6	18,95	606,20	156,74
2019/05/31	CH2205	Male	Three y/o	Treatment (Wk 3)	Treatment	Feed Day	No	01:20	01:00 - 01:59	Early Morning	37,6	101	29,4	29,4	18,75	594,59	113,50
2019/05/31	CH2206	Male	Three y/o	Treatment (Wk 3)	Treatment	Feed Day	No	01:20	01:00 - 01:59	Early Morning	37,8	90	41,8	41,8	18,95	586,16	125,60
2019/05/31	CH2205	Male	Three y/o	Treatment (Wk 3)	Treatment	Feed Day	No	01:25	01:00 - 01:59	Early Morning	37,9	48	17,2	17,2	19,05	536,04	95,17
2019/05/31	CH2206	Male	Three y/o	Treatment (Wk 3)	Treatment	Feed Day	No	01:25	01:00 - 01:59	Early Morning	37,8	86	40,6	40,6	18,95	582,77	124,60
2019/05/31	CH2205	Male	Three y/o	Treatment (Wk 3)	Treatment	Feed Day	No	01:30	01:00 - 01:59	Early Morning	37,9		22,0	22,0	19,05		103,57
2019/05/31	CH2206	Male	Three y/o	Treatment (Wk 3)	Treatment	Feed Day	No	01:30	01:00 - 01:59	Early Morning	37,8	62	39,2	39,2	18,95	557,33	123,39
2019/05/31	CH2205	Male	Three y/o	Treatment (Wk 3)	Treatment	Feed Day	No	01:35	01:00 - 01:59	Early Morning	37,9	45	18,6	18,6	19,05	530,47	97,84
2019/05/31	CH2206	Male	Three y/o	Treatment (Wk 3)	Treatment	Feed Day	No	01:35	01:00 - 01:59	Early Morning	37,8	92	44,0	44,0	18,95	587,78	127,37
2019/05/31	CH2205	Male	Three y/o	Treatment (Wk 3)	Treatment	Feed Day	No	01:40	01:00 - 01:59	Early Morning	37,9		19,8	19,8	19,05		99,97
2019/05/31	CH2206	Male	Three y/o	Treatment (Wk 3)	Treatment	Feed Day	No	01:40	01:00 - 01:59	Early Morning	37,8	94	29,4	29,4	18,95	589,37	113,50
2019/05/31	CH2205	Male	Three y/o	Treatment (Wk 3)	Treatment	Feed Day	No	01:45	01:00 - 01:59	Early Morning	37,9	72	15,2	15,2	19,05	569,20	90,96
2019/05/31	CH2206	Male	Three y/o	Treatment (Wk 3)	Treatment	Feed Day	No	01:45	01:00 - 01:59	Early Morning	37,8	93	33,2	33,2	18,95	588,58	117,68
2019/05/31	CH2205	Male	Three y/o	Treatment (Wk 3)	Treatment	Feed Day	No	01:50	01:00 - 01:59	Early Morning	37,9	78	15,0	15,0	19,05	575,38	90,51
2019/05/31	CH2206	Male	Three y/o	Treatment (Wk 3)	Treatment	Feed Day	No	01:50	01:00 - 01:59	Early Morning	37,7	88	34,0	34,0	18,85	584,49	118,49
2019/05/31	CH2205	Male	Three y/o	Treatment (Wk 3)	Treatment	Feed Day	No	01:55	01:00 - 01:59	Early Morning	37,9	72	15,4	15,4	19,05	569,20	91,41
2019/05/31	CH2206	Male	Three y/o	Treatment (Wk 3)	Treatment	Feed Day	No	01:55	01:00 - 01:59	Early Morning	37,8	98	33,4	33,4	18,95	592,41	117,88
2019/05/31	CH2205	Male	Three y/o	Treatment (Wk 3)	Treatment	Feed Day	No	02:00	02:00 - 02:59	Early Morning	37,9	64	17,2	17,2	19,05	559,88	95,17
2019/05/31	CH2206	Male	Three y/o	Treatment (Wk 3)	Treatment	Feed Day	No	02:00	02:00 - 02:59	Early Morning	37,8	94	37,0	37,0	18,95	589,37	121,40
2019/05/31	CH2205	Male	Three y/o	Treatment (Wk 3)	Treatment	Feed Day	No	02:05	02:00 - 02:59	Early Morning	37,9	48	19,6	19,6	19,05	536,04	99,63
2019/05/31	CH2206	Male	Three y/o	Treatment (Wk 3)	Treatment	Feed Day	No	02:05	02:00 - 02:59	Early Morning	37,8	90	37,6	37,6	18,95	586,16	121,96
2019/05/31	CH2205	Male	Three y/o	Treatment (Wk 3)	Treatment	Feed Day	No	02:10	02:00 - 02:59	Early Morning	38,0		18,2	18,2	19,15		97,10
2019/05/31	CH2206	Male	Three y/o	Treatment (Wk 3)	Treatment	Feed Day	No	02:10	02:00 - 02:59	Early Morning	37,8	58	25,6	25,6	18,95	551,90	108,76
2019/05/31	CH2205	Male	Three y/o	Treatment (Wk 3)	Treatment	Feed Day	No	02:15	02:00 - 02:59	Early Morning	38,0	134	22,0	22,0	19,15	614,33	103,57
2019/05/31	CH2206	Male	Three y/o	Treatment (Wk 3)	Treatment	Feed Day	No	02:15	02:00 - 02:59	Early Morning	37,7	95	29,6	29,6	18,85	590,14	113,73
2019/05/31	CH2205	Male	Three y/o	Treatment (Wk 3)	Treatment	Feed Day	No	02:20	02:00 - 02:59	Early Morning	37,9	66	27,0	27,0	19,05	562,34	110,58
2019/05/31	CH2206	Male	Three y/o	Treatment (Wk 3)	Treatment	Feed Day	No	02:20	02:00 - 02:59	Early Morning	37,7	58	39,6	39,6	18,85	551,90	123,74
2019/05/31	CH2205	Male	Three y/o	Treatment (Wk 3)	Treatment	Feed Day	No	02:25	02:00 - 02:59	Early Morning	38,0	49	21,4	21,4	19,15	537,80	102,63
2019/05/31	CH2206	Male	Three y/o	Treatment (Wk 3)	Treatment	Feed Day	No	02:25	02:00 - 02:59	Early Morning	37,5	59	44,6	44,6	18,65	553,30	127,84
2019/05/31	CH2205	Male	Three y/o	Treatment (Wk 3)	Treatment	Feed Day	No	02:30	02:00 - 02:59	Early Morning	38,0	103	22,2	22,2	19,15	596,00	103,88
2019/05/31	CH2206	Male	Three y/o	Treatment (Wk 3)	Treatment	Feed Day	No	02:30	02:00 - 02:59	Early Morning	37,5	92	46,0	46,0	18,65	587,78	128,91
2019/05/31	CH2205	Male	Three y/o	Treatment (Wk 3)	Treatment	Feed Day	No	02:35	02:00 - 02:59	Early Morning	38,0	42	25,4	25,4	19,15	524,42	108,49
2019/05/31	CH2206	Male	Three y/o	Treatment (Wk 3)	Treatment	Feed Day	No	02:35	02:00 - 02:59	Early Morning	37,5	90	48,4	48,4	18,65	586,16	130,66
2019/05/31	CH2205	Male	Three y/o	Treatment (Wk 3)	Treatment	Feed Day	No	02:40	02:00 - 02:59	Early Morning	38,0	129	26,6	26,6	19,15	611,75	110,07
2019/05/31	CH2206	Male	Three y/o	Treatment (Wk 3)	Treatment	Feed Day	No	02:40	02:00 - 02:59	Early Morning	37,5	197	49,8	49,8	18,65	639,25	131,65
2019/05/31	CH2205	Male	Three y/o	Treatment (Wk 3)	Treatment	Feed Day	No	02:45	02:00 - 02:59	Early Morning	38,0	94	23,8	23,8	19,15	589,37	106,26
2019/05/31	CH2206	Male	Three y/o	Treatment (Wk 3)	Treatment	Feed Day	No	02:45	02:00 - 02:59	Early Morning	37,4	68	46,4	46,4	18,55	564,71	129,21
2019/05/31	CH2205	Male	Three y/o	Treatment (Wk 3)	Treatment	Feed Day	No	02:50	02:00 - 02:59	Early Morning	38,0	87	26,2	26,2	19,15	583,64	109,55
2019/05/31	CH2206	Male	Three y/o	Treatment (Wk 3)	Treatment	Feed Day	No	02:50	02:00 - 02:59	Early Morning	37,4	47	48,0	48,0	18,55	534,23	130,38
2019/05/31	CH2205	Male	Three y/o	Treatment (Wk 3)	Treatment	Feed Day	No	02:55	02:00 - 02:59	Early Morning	38,0	91	27,8	27,8	19,15	586,98	111,58
2019/05/31	CH2206	Male	Three y/o	Treatment (Wk 3)	Treatment	Feed Day	No	02:55	02:00 - 02:59	Early Morning	37,3	92	47,0	47,0	18,46	587,78	129,65
2019/05/31	CH2205	Male	Three y/o	Treatment (Wk 3)	Treatment	Feed Day	No	03:00	03:00 - 03:59	Early Morning	37,9	63	30,0	30,0	19,05	558,62	114,20
2019/05/31	CH2206	Male	Three y/o	Treatment (Wk 3)	Treatment	Feed Day	No	03:00	03:00 - 03:59	Early Morning	37,3	90	46,2	46,2	18,46	586,16	129,06
2019/05/31	CH2205	Male	Three y/o	Treatment (Wk 3)	Treatment	Feed Day	No	03:05	03:00 - 03:59	Early Morning	37,9	92	25,4	25,4	19,05	587,78	108,49
2019/05/31	CH2206	Male	Three y/o	Treatment (Wk 3)	Treatment	Feed Day	No	03:05	03:00 - 03:59	Early Morning	37,3	67	51,4	51,4	18,46	563,53	132,74
2019/05/31	CH2205	Male	Three y/o	Treatment (Wk 3)	Treatment	Feed Day	No	03:10	03:00 - 03:59	Early Morning	37,9	65	25,2	25,2	19,05	561,12	108,22
2019/05/31	CH2206	Male	Three y/o	Treatment (Wk 3)	Treatment	Feed Day	No	03:10	03:00 - 03:59	Early Morning	37,3	80	51,6	51,6	18,46	577,31	132,87
2019/05/31	CH2205	Male	Three y/o	Treatment (Wk 3)	Treatment	Feed Day	No	03:15	03:00 - 03:59	Early Morning	37,9	114	18,6	18,6	19,05	603,20	97,84
2019/05/31	CH2206	Male	Three y/o	Treatment (Wk 3)	Treatment	Feed Day	No	03:15	03:00 - 03:59	Early Morning	37,3	91	45,2	45,2	18,46	586,98	128,30
2019/05/31	CH2205	Male	Three y/o	Treatment (Wk 3)	Treatment	Feed Day	No	03:20	03:00 - 03:59	Early Morning	37,9	46	21,8	21,8	19,05	532,38	103,26
2019/05/31	CH2206	Male	Three y/o	Treatment (Wk 3)	Treatment	Feed Day	No	03:20	03:00 - 03:59	Early Morning	37,2	86	48,4	48,4	18,36	582,77	130,66
2019/05/31	CH2205	Male	Three y/o	Treatment (Wk 3)	Treatment	Feed Day	No	03:25	03:00 - 03:59	Early Morning	37,9	67	19,8	19,8	19,05	563,53	99,97
2019/05/31	CH2206	Male	Three y/o	Treatment (Wk 3)	Treatment	Feed Day	No	03:25	03:00 - 03:59	Early Morning	37,2	77	48,8	48,8	18,36	574,39	130,95
2019/05/31	CH2205	Male	Three y/o	Treatment (Wk 3)	Treatment	Feed Day	No	03:30	03:00 - 03:59	Early Morning	37,9	95	17,6	17,6	19,05	590,14	95,95
2019/05/31	CH2206	Male	Three y/o	Treatment (Wk 3)	Treatment	Feed Day	No	03:30	03:00 - 03:59	Early Morning	37,2	56	30,4	30,4	18,36	549,01	114,65
2019/05/31	CH2205	Male	Three y/o	Treatment (Wk 3)	Treatment	Feed Day	No	03:35	03:00 - 03:59	Early Morning	37,9	92	20,4	20,4	19,05	587,78	100,99
2019/05/31	CH2206	Male	Three y/o	Treatment (Wk 3)	Treatment	Feed Day	No	03:35	03:00 - 03:59	Early Morning	37,3	55	21,8	21,8	18,46	547,52	103,26
2019/05/31	CH2205	Male	Three y/o	Treatment (Wk 3)	Treatment	Feed Day	No	03:40	03:00 - 03:59	Early Morning	37,9	55	17,4	17,4	19,05	547,52	95,56
2019/05/31	CH2206	Male	Three y/o	Treatment (Wk 3)	Treatment	Feed Day	No	03:40	03:00 - 03:59	Early Morning	37,4	90	18,6	18,6	18,55	586,16	97,84
2019/05/31	CH2205	Male	Three y/o	Treatment (Wk 3)	Treatment	Feed Day	No	03:45	03:00 - 03:59	Early Morning	37,9	45	10,6	10,6	19,05	530,47	78,73

2019/05/31	CH2206	Male	Three y/o	Treatment (Wk 3)	Treatment	Feed Day	No	03:45	03:00 - 03:59	Early Morning	37,5	44	32,8	32,8	18,65	528,51	117,26
2019/05/31	CH2205	Male	Three y/o	Treatment (Wk 3)	Treatment	Feed Day	No	03:50	03:00 - 03:59	Early Morning	37,9	98	9,2	9,2	19,05	592,41	73,94
2019/05/31	CH2206	Male	Three y/o	Treatment (Wk 3)	Treatment	Feed Day	No	03:50	03:00 - 03:59	Early Morning	37,5	98	41,4	41,4	18,65	592,41	125,27
2019/05/31	CH2205	Male	Three y/o	Treatment (Wk 3)	Treatment	Feed Day	No	03:55	03:00 - 03:59	Early Morning	37,9	66	12,4	12,4	19,05	562,34	84,04
2019/05/31	CH2206	Male	Three y/o	Treatment (Wk 3)	Treatment	Feed Day	No	03:55	03:00 - 03:59	Early Morning	37,4	82	43,2	43,2	18,55	579,19	126,74
2019/05/31	CH2205	Male	Three y/o	Treatment (Wk 3)	Treatment	Feed Day	No	04:00	04:00 - 04:59	Morning	37,9	103	18,6	18,6	19,05	596,00	97,84
2019/05/31	CH2206	Male	Three y/o	Treatment (Wk 3)	Treatment	Feed Day	No	04:00	04:00 - 04:59	Morning	37,5	90	49,0	49,0	18,65	586,16	131,09
2019/05/31	CH2205	Male	Three y/o	Treatment (Wk 3)	Treatment	Feed Day	No	04:05	04:00 - 04:59	Morning	37,9	88	23,2	23,2	19,05	584,49	105,39
2019/05/31	CH2206	Male	Three y/o	Treatment (Wk 3)	Treatment	Feed Day	No	04:05	04:00 - 04:59	Morning	37,5	76	25,2	25,2	18,65	573,39	108,22
2019/05/31	CH2205	Male	Three y/o	Treatment (Wk 3)	Treatment	Feed Day	No	04:10	04:00 - 04:59	Morning	37,9	46	20,0	20,0	19,05	532,38	100,31
2019/05/31	CH2206	Male	Three y/o	Treatment (Wk 3)	Treatment	Feed Day	No	04:10	04:00 - 04:59	Morning	37,5	93	18,6	18,6	18,65	588,58	97,84
2019/05/31	CH2205	Male	Three y/o	Treatment (Wk 3)	Treatment	Feed Day	No	04:15	04:00 - 04:59	Morning	37,9	63	20,6	20,6	19,05	558,62	101,32
2019/05/31	CH2206	Male	Three y/o	Treatment (Wk 3)	Treatment	Feed Day	No	04:15	04:00 - 04:59	Morning	37,4	44	18,4	18,4	18,55	528,51	97,47
2019/05/31	CH2205	Male	Three y/o	Treatment (Wk 3)	Treatment	Feed Day	No	04:20	04:00 - 04:59	Morning	37,8	71	22,8	22,8	18,95	568,10	104,79
2019/05/31	CH2206	Male	Three y/o	Treatment (Wk 3)	Treatment	Feed Day	No	04:20	04:00 - 04:59	Morning	37,3	86	20,2	20,2	18,46	582,77	100,65
2019/05/31	CH2205	Male	Three y/o	Treatment (Wk 3)	Treatment	Feed Day	No	04:25	04:00 - 04:59	Morning	37,9	92	22,4	22,4	19,05	587,78	104,19
2019/05/31	CH2206	Male	Three y/o	Treatment (Wk 3)	Treatment	Feed Day	No	04:25	04:00 - 04:59	Morning	37,3	89	12,4	12,4	18,46	585,33	84,04
2019/05/31	CH2205	Male	Three y/o	Treatment (Wk 3)	Treatment	Feed Day	No	04:30	04:00 - 04:59	Morning	37,9	106	26,6	26,6	19,05	598,06	110,07
2019/05/31	CH2206	Male	Three y/o	Treatment (Wk 3)	Treatment	Feed Day	No	04:30	04:00 - 04:59	Morning	37,2	78	9,0	9,0	18,36	575,38	73,19
2019/05/31	CH2205	Male	Three y/o	Treatment (Wk 3)	Treatment	Feed Day	No	04:35	04:00 - 04:59	Morning	37,7	68	6,6	6,6	18,85	564,71	62,73
2019/05/31	CH2206	Male	Three y/o	Treatment (Wk 3)	Treatment	Feed Day	No	04:35	04:00 - 04:59	Morning	37,0	91	7,4	7,4	18,16	586,98	66,59
2019/05/31	CH2205	Male	Three y/o	Treatment (Wk 3)	Treatment	Feed Day	No	04:40	04:00 - 04:59	Morning	37,6	44	4,0	4,0	18,75	528,51	45,94
2019/05/31	CH2206	Male	Three y/o	Treatment (Wk 3)	Treatment	Feed Day	No	04:40	04:00 - 04:59	Morning	37,0	96	12,0	12,0	18,16	590,91	82,93
2019/05/31	CH2205	Male	Three y/o	Treatment (Wk 3)	Treatment	Feed Day	No	04:45	04:00 - 04:59	Morning	37,5	65	3,0	3,0	18,65	561,12	36,34
2019/05/31	CH2206	Male	Three y/o	Treatment (Wk 3)	Treatment	Feed Day	No	04:45	04:00 - 04:59	Morning	37,0	69	18,0	18,0	18,16	565,86	96,72
2019/05/31	CH2205	Male	Three y/o	Treatment (Wk 3)	Treatment	Feed Day	No	04:50	04:00 - 04:59	Morning	37,5	83	4,2	4,2	18,65	580,11	47,57
2019/05/31	CH2206	Male	Three y/o	Treatment (Wk 3)	Treatment	Feed Day	No	04:50	04:00 - 04:59	Morning	37,1	49	10,4	10,4	18,26	537,80	78,08
2019/05/31	CH2205	Male	Three y/o	Treatment (Wk 3)	Treatment	Feed Day	No	04:55	04:00 - 04:59	Morning	37,4	45	6,6	6,6	18,55	530,47	62,73
2019/05/31	CH2206	Male	Three y/o	Treatment (Wk 3)	Treatment	Feed Day	No	04:55	04:00 - 04:59	Morning	37,1	48	5,4	5,4	18,26	536,04	55,99
2019/05/31	CH2205	Male	Three y/o	Treatment (Wk 3)	Treatment	Feed Day	No	05:00	05:00 - 05:59	Morning	37,4	49	3,0	3,0	18,55	537,80	36,34
2019/05/31	CH2206	Male	Three y/o	Treatment (Wk 3)	Treatment	Feed Day	No	05:00	05:00 - 05:59	Morning	37,1	42	6,0	6,0	18,26	524,42	59,53
2019/05/31	CH2205	Male	Three y/o	Treatment (Wk 3)	Treatment	Feed Day	No	05:05	05:00 - 05:59	Morning	37,5	57	9,8	9,8	18,65	550,47	76,07
2019/05/31	CH2206	Male	Three y/o	Treatment (Wk 3)	Treatment	Feed Day	No	05:05	05:00 - 05:59	Morning	37,1	88	14,6	14,6	18,26	584,49	89,59
2019/05/31	CH2205	Male	Three y/o	Treatment (Wk 3)	Treatment	Feed Day	No	05:10	05:00 - 05:59	Morning	37,5	49	3,8	3,8	18,65	537,80	44,22
2019/05/31	CH2206	Male	Three y/o	Treatment (Wk 3)	Treatment	Feed Day	No	05:10	05:00 - 05:59	Morning	37,1	103	16,4	16,4	18,26	596,00	93,55
2019/05/31	CH2205	Male	Three y/o	Treatment (Wk 3)	Treatment	Feed Day	No	05:15	05:00 - 05:59	Morning	37,6	57	11,4	11,4	18,75	550,47	81,19
2019/05/31	CH2206	Male	Three y/o	Treatment (Wk 3)	Treatment	Feed Day	No	05:15	05:00 - 05:59	Morning	37,1	49	18,2	18,2	18,26	537,80	97,10
2019/05/31	CH2205	Male	Three y/o	Treatment (Wk 3)	Treatment	Feed Day	No	05:20	05:00 - 05:59	Morning	37,6	88	12,4	12,4	18,75	584,49	84,04
2019/05/31	CH2206	Male	Three y/o	Treatment (Wk 3)	Treatment	Feed Day	No	05:20	05:00 - 05:59	Morning	37,2	76	24,6	24,6	18,36	573,39	107,39
2019/05/31	CH2205	Male	Three y/o	Treatment (Wk 3)	Treatment	Feed Day	No	05:25	05:00 - 05:59	Morning	37,7	74	9,2	9,2	18,85	571,33	73,94
2019/05/31	CH2206	Male	Three y/o	Treatment (Wk 3)	Treatment	Feed Day	No	05:25	05:00 - 05:59	Morning	37,2	98	10,6	10,6	18,36	592,41	78,73
2019/05/31	CH2205	Male	Three y/o	Treatment (Wk 3)	Treatment	Feed Day	No	05:30	05:00 - 05:59	Morning	37,7	34	7,8	7,8	18,85	505,29	68,36
2019/05/31	CH2206	Male	Three y/o	Treatment (Wk 3)	Treatment	Feed Day	No	05:30	05:00 - 05:59	Morning	37,2	49	21,2	21,2	18,36	537,80	102,31
2019/05/31	CH2205	Male	Three y/o	Treatment (Wk 3)	Treatment	Feed Day	No	05:35	05:00 - 05:59	Morning	37,7	94	5,0	5,0	18,85	589,37	53,41
2019/05/31	CH2206	Male	Three y/o	Treatment (Wk 3)	Treatment	Feed Day	No	05:35	05:00 - 05:59	Morning	37,3	88	5,0	5,0	18,46	584,49	53,41
2019/05/31	CH2205	Male	Three y/o	Treatment (Wk 3)	Treatment	Feed Day	No	05:40	05:00 - 05:59	Morning	37,7	53	11,4	11,4	18,85	544,43	81,19
2019/05/31	CH2206	Male	Three y/o	Treatment (Wk 3)	Treatment	Feed Day	No	05:40	05:00 - 05:59	Morning	37,2	98	9,2	9,2	18,36	592,41	73,94
2019/05/31	CH2205	Male	Three y/o	Treatment (Wk 3)	Treatment	Feed Day	No	05:45	05:00 - 05:59	Morning	37,7	132	193,4	193,4	18,85	613,32	178,94
2019/05/31	CH2206	Male	Three y/o	Treatment (Wk 3)	Treatment	Feed Day	No	05:45	05:00 - 05:59	Morning	37,1		46,0	46,0	18,26		128,91
2019/05/31	CH2205	Male	Three y/o	Treatment (Wk 3)	Treatment	Feed Day	No	05:50	05:00 - 05:59	Morning	37,6	118	21,8	21,8	18,75	605,61	103,26
2019/05/31	CH2206	Male	Three y/o	Treatment (Wk 3)	Treatment	Feed Day	No	05:50	05:00 - 05:59	Morning	37,2		18,0	18,0	18,36		96,72
2019/05/31	CH2205	Male	Three y/o	Treatment (Wk 3)	Treatment	Feed Day	No	05:55	05:00 - 05:59	Morning	37,4	60	20,6	20,6	18,55	554,67	101,32
2019/05/31	CH2206	Male	Three y/o	Treatment (Wk 3)	Treatment	Feed Day	No	05:55	05:00 - 05:59	Morning	37,2	76	40,6	40,6	18,36	573,39	124,60
2019/05/31	CH2205	Male	Three y/o	Treatment (Wk 3)	Treatment	Feed Day	No	06:00	06:00 - 06:59	Morning	37,3	74	22,6	22,6	18,46	571,33	104,49
2019/05/31	CH2206	Male	Three y/o	Treatment (Wk 3)	Treatment	Feed Day	No	06:00	06:00 - 06:59	Morning	37,3	140	102,2	102,2	18,46	617,28	156,60
2019/05/31	CH2205	Male	Three y/o	Treatment (Wk 3)	Treatment	Feed Day	No	06:05	06:00 - 06:59	Morning	37,3	84	19,2	19,2	18,46	581,01	98,92
2019/05/31	CH2206	Male	Three y/o	Treatment (Wk 3)	Treatment	Feed Day	No	06:05	06:00 - 06:59	Morning	37,4	124	124,0	124,0	18,55	609,04	163,35
2019/05/31	CH2205	Male	Three y/o	Treatment (Wk 3)	Treatment	Feed Day	No	06:10	06:00 - 06:59	Morning	37,4	60	27,6	27,6	18,55	554,67	111,33
2019/05/31	CH2206	Male	Three y/o	Treatment (Wk 3)	Treatment	Feed Day	No	06:10	06:00 - 06:59	Morning	37,3	90	21,8	21,8	18,46	586,16	103,26
2019/05/31	CH2205	Male	Three y/o	Treatment (Wk 3)	Treatment	Feed Day	No	06:15	06:00 - 06:59	Morning	37,4		87,8	87,8	18,55		151,31
2019/05/31	CH2206	Male	Three y/o	Treatment (Wk 3)	Treatment	Feed Day	No	06:15	06:00 - 06:59	Morning	37,1	121	37,4	37,4	18,26	607,35	121,77
2019/05/31	CH2205	Male	Three y/o	Treatment (Wk 3)	Treatment	Feed Day	No	06:20	06:00 - 06:59	Morning	37,4		32,0	32,0	18,55		116,41
2019/05/31	CH2206	Male	Three y/o	Treatment (Wk 3)	Treatment	Feed Day	No	06:20	06:00 - 06:59	Morning	37,3	91	46,6	46,6	18,46	586,98	129,35
2019/05/31	CH2205	Male	Three y/o	Treatment (Wk 3)	Treatment	Feed Day	No	06:25	06:00 - 06:59	Morning	37,4	117	46,8	46,8	18,55	605,02	129,50
2019/05/31	CH2206	Male	Three y/o	Treatment (Wk 3)	Treatment	Feed Day	No	06:25	06:00 - 06:59	Morning	37,3	148	34,2	34,2	18,46	620,97	118,70
2019/05/31	CH2205	Male	Three y/o	Treatment (Wk 3)	Treatment	Feed Day	No	06:30	06:00 - 06:59	Morning	37,3		34,8	34,8	18,46		119,29
2019/05/31	CH2206	Male	Three y/o	Treatment (Wk 3)	Treatment	Feed Day	No	06:30	06:00 - 06:59	Morning	37,3	107	47,6	47,6	18,46	598,73	130,09
2019/05/31	CH2205	Male	Three y/o	Treatment (Wk 3)	Treatment	Feed Day	No	06:35	06:00 - 06:59	Morning	37,3	78	36,2	36,2	18,46	575,38	120,65
2019/05/31	CH2206	Male	Three y/o	Treatment (Wk 3)	Treatment	Feed Day	No	06:35	06:00 - 06:59	Morning	37,3	119	100,8	100,8	18,46	606,20	156,12
2019/05/31	CH2205	Male	Three y/o	Treatment (Wk 3)	Treatment	Feed Day	No	06:40	06:00 - 06:59	Morning	37,3	135	40,8	40,8	18,46	614,84	124,77

2019/05/31	CH2206	Male	Three y/o	Treatment (Wk 3)	Treatment	Feed Day	No	06:40	06:00 - 06:59	Morning	37,4	119	83,4	83,4	18,55	606,20	149,52
2019/05/31	CH2205	Male	Three y/o	Treatment (Wk 3)	Treatment	Feed Day	No	06:45	06:00 - 06:59	Morning	37,1		37,6	37,6	18,26		121,96
2019/05/31	CH2206	Male	Three y/o	Treatment (Wk 3)	Treatment	Feed Day	No	06:45	06:00 - 06:59	Morning	37,4	111	36,8	36,8	18,55	601,33	121,22
2019/05/31	CH2205	Male	Three y/o	Treatment (Wk 3)	Treatment	Feed Day	No	06:50	06:00 - 06:59	Morning	37,1		14,6	14,6	18,26		89,59
2019/05/31	CH2206	Male	Three y/o	Treatment (Wk 3)	Treatment	Feed Day	No	06:50	06:00 - 06:59	Morning	37,4	107	221,0	221,0	18,55	598,73	183,63
2019/05/31	CH2205	Male	Three y/o	Treatment (Wk 3)	Treatment	Feed Day	No	06:55	06:00 - 06:59	Morning	37,1	79	25,6	25,6	18,26	576,36	108,76
2019/05/31	CH2206	Male	Three y/o	Treatment (Wk 3)	Treatment	Feed Day	No	06:55	06:00 - 06:59	Morning	37,4	182	49,4	49,4	18,55	634,31	131,37
2019/05/31	CH2205	Male	Three y/o	Treatment (Wk 3)	Treatment	Feed Day	No	07:00	07:00 - 07:59	Morning	37,1	89	57,0	57,0	18,26	585,33	136,32
2019/05/31	CH2206	Male	Three y/o	Treatment (Wk 3)	Treatment	Feed Day	No	07:00	07:00 - 07:59	Morning	37,5	140	339,2	339,2	18,65	617,28	198,77
2019/05/31	CH2205	Male	Three y/o	Treatment (Wk 3)	Treatment	Feed Day	No	07:05	07:00 - 07:59	Morning	37,2		99,2	99,2	18,36		155,56
2019/05/31	CH2206	Male	Three y/o	Treatment (Wk 3)	Treatment	Feed Day	No	07:05	07:00 - 07:59	Morning	37,5	135	264,4	264,4	18,65	614,84	189,96
2019/05/31	CH2205	Male	Three y/o	Treatment (Wk 3)	Treatment	Feed Day	No	07:10	07:00 - 07:59	Morning	37,3	54	115,6	115,6	18,46	545,99	160,90
2019/05/31	CH2206	Male	Three y/o	Treatment (Wk 3)	Treatment	Feed Day	No	07:10	07:00 - 07:59	Morning	37,5	120	107,0	107,0	18,65	606,78	158,20
2019/05/31	CH2205	Male	Three y/o	Treatment (Wk 3)	Treatment	Feed Day	No	07:15	07:00 - 07:59	Morning	37,3	86	81,2	81,2	18,46	582,77	148,59
2019/05/31	CH2206	Male	Three y/o	Treatment (Wk 3)	Treatment	Feed Day	No	07:15	07:00 - 07:59	Morning	37,5	113	210,0	210,0	18,65	602,58	181,84
2019/05/31	CH2205	Male	Three y/o	Treatment (Wk 3)	Treatment	Feed Day	No	07:20	07:00 - 07:59	Morning	37,3	93	53,8	53,8	18,46	588,58	134,32
2019/05/31	CH2206	Male	Three y/o	Treatment (Wk 3)	Treatment	Feed Day	No	07:20	07:00 - 07:59	Morning	37,7	126	271,4	271,4	18,85	610,14	190,88
2019/05/31	CH2205	Male	Three y/o	Treatment (Wk 3)	Treatment	Feed Day	No	07:25	07:00 - 07:59	Morning	37,4	118	134,4	134,4	18,55	605,61	166,17
2019/05/31	CH2206	Male	Three y/o	Treatment (Wk 3)	Treatment	Feed Day	No	07:25	07:00 - 07:59	Morning	37,7	129	128,4	128,4	18,85	611,75	164,57
2019/05/31	CH2205	Male	Three y/o	Treatment (Wk 3)	Treatment	Feed Day	No	07:30	07:00 - 07:59	Morning	37,4	120	121,0	121,0	18,55	606,78	162,50
2019/05/31	CH2206	Male	Three y/o	Treatment (Wk 3)	Treatment	Feed Day	No	07:30	07:00 - 07:59	Morning	37,7	147	61,4	61,4	18,85	620,52	138,89
2019/05/31	CH2205	Male	Three y/o	Treatment (Wk 3)	Treatment	Feed Day	No	07:35	07:00 - 07:59	Morning	37,5	123	226,2	226,2	18,65	608,49	184,45
2019/05/31	CH2206	Male	Three y/o	Treatment (Wk 3)	Treatment	Feed Day	No	07:35	07:00 - 07:59	Morning	37,7		84,2	84,2	18,85		149,85
2019/05/31	CH2205	Male	Three y/o	Treatment (Wk 3)	Treatment	Feed Day	No	07:40	07:00 - 07:59	Morning	37,5	101	29,6	29,6	18,65	594,59	113,73
2019/05/31	CH2206	Male	Three y/o	Treatment (Wk 3)	Treatment	Feed Day	No	07:40	07:00 - 07:59	Morning	37,7		224,8	224,8	18,85		184,23
2019/05/31	CH2205	Male	Three y/o	Treatment (Wk 3)	Treatment	Feed Day	No	07:45	07:00 - 07:59	Morning	37,4	65	16,0	16,0	18,55	561,12	92,71
2019/05/31	CH2206	Male	Three y/o	Treatment (Wk 3)	Treatment	Feed Day	No	07:45	07:00 - 07:59	Morning	37,7	107	57,0	57,0	18,85	598,73	136,32
2019/05/31	CH2205	Male	Three y/o	Treatment (Wk 3)	Treatment	Feed Day	No	07:50	07:00 - 07:59	Morning	37,4	62	22,6	22,6	18,55	557,33	104,49
2019/05/31	CH2206	Male	Three y/o	Treatment (Wk 3)	Treatment	Feed Day	No	07:50	07:00 - 07:59	Morning	37,7	121	119,2	119,2	18,85	607,35	161,97
2019/05/31	CH2205	Male	Three y/o	Treatment (Wk 3)	Treatment	Feed Day	No	07:55	07:00 - 07:59	Morning	37,3	169	35,0	35,0	18,46	629,60	119,49
2019/05/31	CH2206	Male	Three y/o	Treatment (Wk 3)	Treatment	Feed Day	No	07:55	07:00 - 07:59	Morning	37,8	117	224,4	224,4	18,95	605,02	184,17
2019/05/31	CH2205	Male	Three y/o	Treatment (Wk 3)	Treatment	Feed Day	No	08:00	08:00 - 08:59	Late Morning	37,4	93	29,4	29,4	18,55	588,58	113,50
2019/05/31	CH2206	Male	Three y/o	Treatment (Wk 3)	Treatment	Feed Day	No	08:00	08:00 - 08:59	Late Morning	37,7	125	206,6	206,6	18,85	609,60	181,26
2019/05/31	CH2205	Male	Three y/o	Treatment (Wk 3)	Treatment	Feed Day	No	08:05	08:00 - 08:59	Late Morning	37,4	69	24,8	24,8	18,55	565,86	107,67
2019/05/31	CH2206	Male	Three y/o	Treatment (Wk 3)	Treatment	Feed Day	No	08:05	08:00 - 08:59	Late Morning	37,7		66,6	66,6	18,85		141,71
2019/05/31	CH2205	Male	Three y/o	Treatment (Wk 3)	Treatment	Feed Day	No	08:10	08:00 - 08:59	Late Morning	37,3	85	24,6	24,6	18,46	581,90	107,39
2019/05/31	CH2206	Male	Three y/o	Treatment (Wk 3)	Treatment	Feed Day	No	08:10	08:00 - 08:59	Late Morning	37,7	83	127,4	127,4	18,85	580,11	164,30
2019/05/31	CH2205	Male	Three y/o	Treatment (Wk 3)	Treatment	Feed Day	No	08:15	08:00 - 08:59	Late Morning	37,2	76	30,4	30,4	18,36	573,39	114,65
2019/05/31	CH2206	Male	Three y/o	Treatment (Wk 3)	Treatment	Feed Day	No	08:15	08:00 - 08:59	Late Morning	37,7	115	148,0	148,0	18,85	603,82	169,55
2019/05/31	CH2205	Male	Three y/o	Treatment (Wk 3)	Treatment	Feed Day	No	08:20	08:00 - 08:59	Late Morning	37,1		89,8	89,8	18,26		152,10
2019/05/31	CH2206	Male	Three y/o	Treatment (Wk 3)	Treatment	Feed Day	No	08:20	08:00 - 08:59	Late Morning	37,7		431,2	431,2	18,85		207,28
2019/05/31	CH2205	Male	Three y/o	Treatment (Wk 3)	Treatment	Feed Day	No	08:25	08:00 - 08:59	Late Morning	37,2	102	240,6	240,6	18,36	595,30	186,63
2019/05/31	CH2206	Male	Three y/o	Treatment (Wk 3)	Treatment	Feed Day	No	08:25	08:00 - 08:59	Late Morning	37,7		145,2	145,2	18,85		168,88
2019/05/31	CH2205	Male	Three y/o	Treatment (Wk 3)	Treatment	Feed Day	No	08:30	08:00 - 08:59	Late Morning	37,3	154	175,4	175,4	18,46	623,58	175,51
2019/05/31	CH2206	Male	Three y/o	Treatment (Wk 3)	Treatment	Feed Day	No	08:30	08:00 - 08:59	Late Morning	37,7	107	167,6	167,6	18,85	598,73	173,91
2019/05/31	CH2205	Male	Three y/o	Treatment (Wk 3)	Treatment	Feed Day	No	08:35	08:00 - 08:59	Late Morning	37,3	114	173,4	173,4	18,46	603,20	175,10
2019/05/31	CH2206	Male	Three y/o	Treatment (Wk 3)	Treatment	Feed Day	No	08:35	08:00 - 08:59	Late Morning	37,8	124	138,8	138,8	18,95	609,04	167,30
2019/05/31	CH2205	Male	Three y/o	Treatment (Wk 3)	Treatment	Feed Day	No	08:40	08:00 - 08:59	Late Morning	37,4	89	130,8	130,8	18,55	585,33	165,22
2019/05/31	CH2206	Male	Three y/o	Treatment (Wk 3)	Treatment	Feed Day	No	08:40	08:00 - 08:59	Late Morning	37,7		76,2	76,2	18,85		146,38
2019/05/31	CH2205	Male	Three y/o	Treatment (Wk 3)	Treatment	Feed Day	No	08:45	08:00 - 08:59	Late Morning	37,3	66	18,0	18,0	18,46	562,34	96,72
2019/05/31	CH2206	Male	Three y/o	Treatment (Wk 3)	Treatment	Feed Day	No	08:45	08:00 - 08:59	Late Morning	37,7	89	63,0	63,0	18,85	585,33	139,78
2019/05/31	CH2205	Male	Three y/o	Treatment (Wk 3)	Treatment	Feed Day	No	08:50	08:00 - 08:59	Late Morning	37,2		20,4	20,4	18,36		100,99
2019/05/31	CH2206	Male	Three y/o	Treatment (Wk 3)	Treatment	Feed Day	No	08:50	08:00 - 08:59	Late Morning	37,7	86	154,2	154,2	18,85	582,77	170,99
2019/05/31	CH2205	Male	Three y/o	Treatment (Wk 3)	Treatment	Feed Day	No	08:55	08:00 - 08:59	Late Morning	37,1		61,2	61,2	18,26		138,78
2019/05/31	CH2206	Male	Three y/o	Treatment (Wk 3)	Treatment	Feed Day	No	08:55	08:00 - 08:59	Late Morning	37,7	109	90,8	90,8	18,85	600,04	152,48
2019/05/31	CH2205	Male	Three y/o	Treatment (Wk 3)	Treatment	Feed Day	No	09:00	09:00 - 09:59	Late Morning	37,2	121	16,2	16,2	18,36	607,35	93,13
2019/05/31	CH2206	Male	Three y/o	Treatment (Wk 3)	Treatment	Feed Day	No	09:00	09:00 - 09:59	Late Morning	37,7	107	217,0	217,0	18,85	598,73	182,99
2019/05/31	CH2205	Male	Three y/o	Treatment (Wk 3)	Treatment	Feed Day	No	09:05	09:00 - 09:59	Late Morning	37,3	91	88,8	88,8	18,46	586,98	151,71
2019/05/31	CH2206	Male	Three y/o	Treatment (Wk 3)	Treatment	Feed Day	No	09:05	09:00 - 09:59	Late Morning	37,7	107	81,8	81,8	18,85	598,73	148,85
2019/05/31	CH2205	Male	Three y/o	Treatment (Wk 3)	Treatment	Feed Day	No	09:10	09:00 - 09:59	Late Morning	37,4	95	21,6	21,6	18,55	590,14	102,94
2019/05/31	CH2206	Male	Three y/o	Treatment (Wk 3)	Treatment	Feed Day	No	09:10	09:00 - 09:59	Late Morning	37,7	158	45,0	45,0	18,85	625,26	128,15
2019/05/31	CH2205	Male	Three y/o	Treatment (Wk 3)	Treatment	Feed Day	No	09:15	09:00 - 09:59	Late Morning	37,4	107	233,2	233,2	18,55	598,73	185,53
2019/05/31	CH2206	Male	Three y/o	Treatment (Wk 3)	Treatment	Feed Day	No	09:15	09:00 - 09:59	Late Morning	37,7	105	71,6	71,6	18,85	597,38	144,22
2019/05/31	CH2205	Male	Three y/o	Treatment (Wk 3)	Treatment	Feed Day	No	09:20	09:00 - 09:59	Late Morning	37,4	78	24,4	24,4	18,55	575,38	107,11
2019/05/31	CH2206	Male	Three y/o	Treatment (Wk 3)	Treatment	Feed Day	No	09:20	09:00 - 09:59	Late Morning	37,7	106	250,4	250,4	18,85	598,06	188,04
2019/05/31	CH2205	Male	Three y/o	Treatment (Wk 3)	Treatment	Feed Day	No	09:25	09:00 - 09:59	Late Morning	37,4	56	24,2	24,2	18,55	549,01	106,83
2019/05/31	CH2206	Male	Three y/o	Treatment (Wk 3)	Treatment	Feed Day	No	09:25	09:00 - 09:59	Late Morning	37,7	90	31,8	31,8	18,85	586,16	116,20
2019/05/31	CH2205	Male	Three y/o	Treatment (Wk 3)	Treatment	Feed Day	No	09:30	09:00 - 09:59	Late Morning	37,5	64	33,4	33,4	18,65	559,88	117,88
2019/05/31	CH2206	Male	Three y/o	Treatment (Wk 3)	Treatment	Feed Day	No	09:30	09:00 - 09:59	Late Morning	37,5	73	82,2	82,2	18,65	570,27	149,02
2019/05/31	CH2205	Male	Three y/o	Treatment (Wk 3)	Treatment	Feed Day	No	09:35	09:00 - 09:59	Late Morning	37,4	90	18,2	18,2	18,55	586,16	97,10

2019/05/31	CH2206	Male	Three y/o	Treatment (Wk 3)	Treatment	Feed Day	No	09:35	09:00 - 09:59	Late Morning	37,5	135	312,8	312,8	18,65	614,84	195,90
2019/05/31	CH2205	Male	Three y/o	Treatment (Wk 3)	Treatment	Feed Day	No	09:40	09:00 - 09:59	Late Morning	37,4	60	13,2	13,2	18,55	554,67	86,17
2019/05/31	CH2206	Male	Three y/o	Treatment (Wk 3)	Treatment	Feed Day	No	09:40	09:00 - 09:59	Late Morning	37,7		158,2	158,2	18,85		171,88
2019/05/31	CH2205	Male	Three y/o	Treatment (Wk 3)	Treatment	Feed Day	No	09:45	09:00 - 09:59	Late Morning	37,4		30,4	30,4	18,55		114,65
2019/05/31	CH2206	Male	Three y/o	Treatment (Wk 3)	Treatment	Feed Day	No	09:45	09:00 - 09:59	Late Morning	37,8	105	62,4	62,4	18,95	597,38	139,45
2019/05/31	CH2205	Male	Three y/o	Treatment (Wk 3)	Treatment	Feed Day	No	09:50	09:00 - 09:59	Late Morning	37,4	87	24,2	24,2	18,55	583,64	106,83
2019/05/31	CH2206	Male	Three y/o	Treatment (Wk 3)	Treatment	Feed Day	No	09:50	09:00 - 09:59	Late Morning	37,9	74	30,8	30,8	19,05	571,33	115,10
2019/05/31	CH2205	Male	Three y/o	Treatment (Wk 3)	Treatment	Feed Day	No	09:55	09:00 - 09:59	Late Morning	37,4		23,2	23,2	18,55		105,39
2019/05/31	CH2206	Male	Three y/o	Treatment (Wk 3)	Treatment	Feed Day	No	09:55	09:00 - 09:59	Late Morning	37,9	92	113,0	113,0	19,05	587,78	160,11
2019/05/31	CH2205	Male	Three y/o	Treatment (Wk 3)	Treatment	Feed Day	No	10:00	10:00 - 10:59	Late Morning	37,3	128	25,8	25,8	18,46	611,22	109,02
2019/05/31	CH2206	Male	Three y/o	Treatment (Wk 3)	Treatment	Feed Day	No	10:00	10:00 - 10:59	Late Morning	37,9	106	233,4	233,4	19,05	598,06	185,56
2019/05/31	CH2205	Male	Three y/o	Treatment (Wk 3)	Treatment	Feed Day	No	10:05	10:00 - 10:59	Late Morning	37,3		21,8	21,8	18,46		103,26
2019/05/31	CH2206	Male	Three y/o	Treatment (Wk 3)	Treatment	Feed Day	No	10:05	10:00 - 10:59	Late Morning	37,8		30,2	30,2	18,95		114,42
2019/05/31	CH2205	Male	Three y/o	Treatment (Wk 3)	Treatment	Feed Day	No	10:10	10:00 - 10:59	Late Morning	37,2	138	102,0	102,0	18,36	616,31	156,53
2019/05/31	CH2206	Male	Three y/o	Treatment (Wk 3)	Treatment	Feed Day	No	10:10	10:00 - 10:59	Late Morning	37,7	108	100,0	100,0	18,85	599,39	155,84
2019/05/31	CH2205	Male	Three y/o	Treatment (Wk 3)	Treatment	Feed Day	No	10:15	10:00 - 10:59	Late Morning	37,3	70	284,8	284,8	18,46	566,99	192,58
2019/05/31	CH2206	Male	Three y/o	Treatment (Wk 3)	Treatment	Feed Day	No	10:15	10:00 - 10:59	Late Morning	37,7	191	24,6	24,6	18,85	637,33	107,39
2019/05/31	CH2205	Male	Three y/o	Treatment (Wk 3)	Treatment	Feed Day	No	10:20	10:00 - 10:59	Late Morning	37,4	105	241,2	241,2	18,55	597,38	186,72
2019/05/31	CH2206	Male	Three y/o	Treatment (Wk 3)	Treatment	Feed Day	No	10:20	10:00 - 10:59	Late Morning	37,7		88,4	88,4	18,85		151,55
2019/05/31	CH2205	Male	Three y/o	Treatment (Wk 3)	Treatment	Feed Day	No	10:25	10:00 - 10:59	Late Morning	37,4	118	53,0	53,0	18,55	605,61	133,80
2019/05/31	CH2206	Male	Three y/o	Treatment (Wk 3)	Treatment	Feed Day	No	10:25	10:00 - 10:59	Late Morning	37,7	90	19,2	19,2	18,85	586,16	98,92
2019/05/31	CH2205	Male	Three y/o	Treatment (Wk 3)	Treatment	Feed Day	Yes	10:30	10:00 - 10:59	Late Morning	37,5	67	52,4	52,4	18,65	563,53	133,41
2019/05/31	CH2206	Male	Three y/o	Treatment (Wk 3)	Treatment	Feed Day	Yes	10:30	10:00 - 10:59	Late Morning	37,7	103	45,6	45,6	18,85	596,00	128,61
2019/05/31	CH2205	Male	Three y/o	Treatment (Wk 3)	Treatment	Feed Day	Yes	10:35	10:00 - 10:59	Late Morning	37,5		97,6	97,6	18,65		155,00
2019/05/31	CH2206	Male	Three y/o	Treatment (Wk 3)	Treatment	Feed Day	Yes	10:35	10:00 - 10:59	Late Morning	37,7	45	114,8	114,8	18,85	530,47	160,66
2019/05/31	CH2205	Male	Three y/o	Treatment (Wk 3)	Treatment	Feed Day	Yes	10:40	10:00 - 10:59	Late Morning	37,7	147	103,0	103,0	18,85	620,52	156,87
2019/05/31	CH2206	Male	Three y/o	Treatment (Wk 3)	Treatment	Feed Day	Yes	10:40	10:00 - 10:59	Late Morning	37,7	180	70,2	70,2	18,85	633,61	143,54
2019/05/31	CH2205	Male	Three y/o	Treatment (Wk 3)	Treatment	Feed Day	Yes	10:45	10:00 - 10:59	Late Morning	37,7	120	511,0	511,0	18,85	606,78	213,32
2019/05/31	CH2206	Male	Three y/o	Treatment (Wk 3)	Treatment	Feed Day	Yes	10:45	10:00 - 10:59	Late Morning	37,9	158	227,8	227,8	19,05	625,26	184,70
2019/05/31	CH2205	Male	Three y/o	Treatment (Wk 3)	Treatment	Feed Day	Yes	10:50	10:00 - 10:59	Late Morning	37,7	58	182,0	182,0	18,85	551,90	176,80
2019/05/31	CH2206	Male	Three y/o	Treatment (Wk 3)	Treatment	Feed Day	Yes	10:50	10:00 - 10:59	Late Morning	38,0	197	384,8	384,8	19,15	639,25	203,24
2019/05/31	CH2205	Male	Three y/o	Treatment (Wk 3)	Treatment	Feed Day	Yes	10:55	10:00 - 10:59	Late Morning	37,9		166,6	166,6	19,05		173,70
2019/05/31	CH2206	Male	Three y/o	Treatment (Wk 3)	Treatment	Feed Day	Yes	10:55	10:00 - 10:59	Late Morning	38,2		196,6	196,6	19,36		179,52
2019/05/31	CH2205	Male	Three y/o	Treatment (Wk 3)	Treatment	Feed Day	Yes	11:00	11:00 - 11:59	Late Morning	37,9	119	85,4	85,4	19,05	606,20	150,35
2019/05/31	CH2206	Male	Three y/o	Treatment (Wk 3)	Treatment	Feed Day	Yes	11:00	11:00 - 11:59	Late Morning	38,2	117	498,4	498,4	19,36	605,02	212,43
2019/05/31	CH2205	Male	Three y/o	Treatment (Wk 3)	Treatment	Feed Day	Yes	11:05	11:00 - 11:59	Late Morning	37,9		188,8	188,8	19,05		178,09
2019/05/31	CH2206	Male	Three y/o	Treatment (Wk 3)	Treatment	Feed Day	Yes	11:05	11:00 - 11:59	Late Morning	38,2		289,2	289,2	19,36		193,12
2019/05/31	CH2205	Male	Three y/o	Treatment (Wk 3)	Treatment	Feed Day	Yes	11:10	11:00 - 11:59	Late Morning	38,0	88	22,6	22,6	19,15	584,49	104,49
2019/05/31	CH2206	Male	Three y/o	Treatment (Wk 3)	Treatment	Feed Day	Yes	11:10	11:00 - 11:59	Late Morning	38,7	150	103,2	103,2	19,87	621,86	156,94
2019/05/31	CH2205	Male	Three y/o	Treatment (Wk 3)	Treatment	Feed Day	Yes	11:15	11:00 - 11:59	Late Morning	37,9	100	54,0	54,0	19,05	593,87	134,45
2019/05/31	CH2206	Male	Three y/o	Treatment (Wk 3)	Treatment	Feed Day	Yes	11:15	11:00 - 11:59	Late Morning	38,6	152	97,8	97,8	19,76	622,73	155,07
2019/05/31	CH2205	Male	Three y/o	Treatment (Wk 3)	Treatment	Feed Day	Yes	11:20	11:00 - 11:59	Late Morning	37,9	92	42,2	42,2	19,05	587,78	125,93
2019/05/31	CH2206	Male	Three y/o	Treatment (Wk 3)	Treatment	Feed Day	Yes	11:20	11:00 - 11:59	Late Morning	38,4	88	52,0	52,0	19,56	584,49	133,14
2019/05/31	CH2205	Male	Three y/o	Treatment (Wk 3)	Treatment	Feed Day	Yes	11:25	11:00 - 11:59	Late Morning	37,9	95	51,4	51,4	19,05	590,14	132,74
2019/05/31	CH2206	Male	Three y/o	Treatment (Wk 3)	Treatment	Feed Day	Yes	11:25	11:00 - 11:59	Late Morning	38,3	126	50,4	50,4	19,46	610,14	132,06
2019/05/31	CH2205	Male	Three y/o	Treatment (Wk 3)	Treatment	Feed Day	No	11:30	11:00 - 11:59	Late Morning	37,9	80	24,2	24,2	19,05	577,31	106,83
2019/05/31	CH2206	Male	Three y/o	Treatment (Wk 3)	Treatment	Feed Day	No	11:30	11:00 - 11:59	Late Morning	38,3	135	65,6	65,6	19,46	614,84	141,19
2019/05/31	CH2205	Male	Three y/o	Treatment (Wk 3)	Treatment	Feed Day	No	11:35	11:00 - 11:59	Late Morning	37,7		23,2	23,2	18,85		105,39
2019/05/31	CH2206	Male	Three y/o	Treatment (Wk 3)	Treatment	Feed Day	No	11:35	11:00 - 11:59	Late Morning	38,3	123	75,8	75,8	19,46	608,49	146,20
2019/05/31	CH2205	Male	Three y/o	Treatment (Wk 3)	Treatment	Feed Day	No	11:40	11:00 - 11:59	Late Morning	37,6		143,6	143,6	18,75		168,49
2019/05/31	CH2206	Male	Three y/o	Treatment (Wk 3)	Treatment	Feed Day	No	11:40	11:00 - 11:59	Late Morning	38,3		311,4	311,4	19,46		195,74
2019/05/31	CH2205	Male	Three y/o	Treatment (Wk 3)	Treatment	Feed Day	No	11:45	11:00 - 11:59	Late Morning	37,8		56,2	56,2	18,95		135,83
2019/05/31	CH2206	Male	Three y/o	Treatment (Wk 3)	Treatment	Feed Day	No	11:45	11:00 - 11:59	Late Morning	38,3		32,6	32,6	19,46		117,05
2019/05/31	CH2205	Male	Three y/o	Treatment (Wk 3)	Treatment	Feed Day	No	11:50	11:00 - 11:59	Late Morning	37,5		63,0	63,0	18,65		139,78
2019/05/31	CH2206	Male	Three y/o	Treatment (Wk 3)	Treatment	Feed Day	No	11:50	11:00 - 11:59	Late Morning	38,0	114	123,2	123,2	19,15	603,20	163,13
2019/05/31	CH2205	Male	Three y/o	Treatment (Wk 3)	Treatment	Feed Day	No	11:55	11:00 - 11:59	Late Morning	37,5	102	99,0	99,0	18,65	595,30	155,49
2019/05/31	CH2206	Male	Three y/o	Treatment (Wk 3)	Treatment	Feed Day	No	11:55	11:00 - 11:59	Late Morning	38,0	137	211,4	211,4	19,15	615,83	182,07
2019/05/31	CH2205	Male	Three y/o	Treatment (Wk 3)	Treatment	Feed Day	No	12:00	12:00 - 12:59	Afternoon	37,6	84	169,4	169,4	18,75	581,01	174,28
2019/05/31	CH2206	Male	Three y/o	Treatment (Wk 3)	Treatment	Feed Day	No	12:00	12:00 - 12:59	Afternoon	38,1	118	62,6	62,6	19,26	605,61	139,56
2019/05/31	CH2205	Male	Three y/o	Treatment (Wk 3)	Treatment	Feed Day	No	12:05	12:00 - 12:59	Afternoon	37,6	92	155,2	155,2	18,75	587,78	171,21
2019/05/31	CH2206	Male	Three y/o	Treatment (Wk 3)	Treatment	Feed Day	No	12:05	12:00 - 12:59	Afternoon	38,1	172	103,0	103,0	19,26	630,73	156,87
2019/05/31	CH2205	Male	Three y/o	Treatment (Wk 3)	Treatment	Feed Day	No	12:10	12:00 - 12:59	Afternoon	37,6		67,6	67,6	18,75		142,23
2019/05/31	CH2206	Male	Three y/o	Treatment (Wk 3)	Treatment	Feed Day	No	12:10	12:00 - 12:59	Afternoon	38,1	128	257,6	257,6	19,26	611,22	189,04
2019/05/31	CH2205	Male	Three y/o	Treatment (Wk 3)	Treatment	Feed Day	No	12:15	12:00 - 12:59	Afternoon	37,6		35,4	35,4	18,75		119,88
2019/05/31	CH2206	Male	Three y/o	Treatment (Wk 3)	Treatment	Feed Day	No	12:15	12:00 - 12:59	Afternoon	38,2	143	21,8	21,8	19,36	618,69	103,26
2019/05/31	CH2205	Male	Three y/o	Treatment (Wk 3)	Treatment	Feed Day	No	12:20	12:00 - 12:59	Afternoon	37,6	88	89,0	89,0	18,75	584,49	151,78
2019/05/31	CH2206	Male	Three y/o	Treatment (Wk 3)	Treatment	Feed Day	No	12:20	12:00 - 12:59	Afternoon	38,1	124	54,0	54,0	19,26	609,04	134,45
2019/05/31	CH2205	Male	Three y/o	Treatment (Wk 3)	Treatment	Feed Day	No	12:25	12:00 - 12:59	Afternoon	37,7	92	99,4	99,4	18,85	587,78	155,63
2019/05/31	CH2206	Male	Three y/o	Treatment (Wk 3)	Treatment	Feed Day	No	12:25	12:00 - 12:59	Afternoon	38,1		116,6	116,6	19,26		161,20
2019/05/31	CH2205	Male	Three y/o	Treatment (Wk 3)	Treatment	Feed Day	No	12:30	12:00 - 12:59	Afternoon	37,6		34,8	34,8	18,75		119,29

2019/05/31	CH2206	Male	Three y/o	Treatment (Wk 3)	Treatment	Feed Day	No	12:30	12:00 - 12:59	Afternoon	38,0	49	33,0	33,0	19,15	537,80	117,47
2019/05/31	CH2205	Male	Three y/o	Treatment (Wk 3)	Treatment	Feed Day	No	12:35	12:00 - 12:59	Afternoon	37,5	90	33,8	33,8	18,65	586,16	118,29
2019/05/31	CH2206	Male	Three y/o	Treatment (Wk 3)	Treatment	Feed Day	No	12:35	12:00 - 12:59	Afternoon	37,9	109	25,4	25,4	19,05	600,04	108,49
2019/05/31	CH2205	Male	Three y/o	Treatment (Wk 3)	Treatment	Feed Day	No	12:40	12:00 - 12:59	Afternoon	37,4		22,0	22,0	18,55		103,57
2019/05/31	CH2206	Male	Three y/o	Treatment (Wk 3)	Treatment	Feed Day	No	12:40	12:00 - 12:59	Afternoon	37,8	74	15,6	15,6	18,95	571,33	91,85
2019/05/31	CH2205	Male	Three y/o	Treatment (Wk 3)	Treatment	Feed Day	No	12:45	12:00 - 12:59	Afternoon	37,5	70	18,8	18,8	18,65	566,99	98,20
2019/05/31	CH2206	Male	Three y/o	Treatment (Wk 3)	Treatment	Feed Day	No	12:45	12:00 - 12:59	Afternoon	37,7	76	13,8	13,8	18,85	573,39	87,68
2019/05/31	CH2205	Male	Three y/o	Treatment (Wk 3)	Treatment	Feed Day	No	12:50	12:00 - 12:59	Afternoon	37,5	61	18,4	18,4	18,65	556,01	97,47
2019/05/31	CH2206	Male	Three y/o	Treatment (Wk 3)	Treatment	Feed Day	No	12:50	12:00 - 12:59	Afternoon	37,7	89	12,2	12,2	18,85	585,33	83,49
2019/05/31	CH2205	Male	Three y/o	Treatment (Wk 3)	Treatment	Feed Day	No	12:55	12:00 - 12:59	Afternoon	37,6	62	17,6	17,6	18,75	557,33	95,95
2019/05/31	CH2206	Male	Three y/o	Treatment (Wk 3)	Treatment	Feed Day	No	12:55	12:00 - 12:59	Afternoon	37,7	88	15,2	15,2	18,85	584,49	90,96
2019/05/31	CH2205	Male	Three y/o	Treatment (Wk 3)	Treatment	Feed Day	No	13:00	13:00 - 13:59	Afternoon	37,6	48	17,8	17,8	18,75	536,04	96,34
2019/05/31	CH2206	Male	Three y/o	Treatment (Wk 3)	Treatment	Feed Day	No	13:00	13:00 - 13:59	Afternoon	37,5	80	38,0	38,0	18,65	577,31	122,32
2019/05/31	CH2205	Male	Three y/o	Treatment (Wk 3)	Treatment	Feed Day	No	13:05	13:00 - 13:59	Afternoon	37,6	145	185,2	185,2	18,75	619,62	177,42
2019/05/31	CH2206	Male	Three y/o	Treatment (Wk 3)	Treatment	Feed Day	No	13:05	13:00 - 13:59	Afternoon	37,5	135	31,4	31,4	18,65	614,84	115,76
2019/05/31	CH2205	Male	Three y/o	Treatment (Wk 3)	Treatment	Feed Day	No	13:10	13:00 - 13:59	Afternoon	37,6	111	71,0	71,0	18,75	601,33	143,93
2019/05/31	CH2206	Male	Three y/o	Treatment (Wk 3)	Treatment	Feed Day	No	13:10	13:00 - 13:59	Afternoon	37,5	98	28,8	28,8	18,65	592,41	112,79
2019/05/31	CH2205	Male	Three y/o	Treatment (Wk 3)	Treatment	Feed Day	No	13:15	13:00 - 13:59	Afternoon	37,6	108	45,2	45,2	18,75	599,39	128,30
2019/05/31	CH2206	Male	Three y/o	Treatment (Wk 3)	Treatment	Feed Day	No	13:15	13:00 - 13:59	Afternoon	37,4		43,2	43,2	18,55		126,74
2019/05/31	CH2205	Male	Three y/o	Treatment (Wk 3)	Treatment	Feed Day	No	13:20	13:00 - 13:59	Afternoon	37,6	35	32,8	32,8	18,75	507,97	117,26
2019/05/31	CH2206	Male	Three y/o	Treatment (Wk 3)	Treatment	Feed Day	No	13:20	13:00 - 13:59	Afternoon	37,5	120	64,4	64,4	18,65	606,78	140,55
2019/05/31	CH2205	Male	Three y/o	Treatment (Wk 3)	Treatment	Feed Day	No	13:25	13:00 - 13:59	Afternoon	37,6	87	30,6	30,6	18,75	583,64	114,87
2019/05/31	CH2206	Male	Three y/o	Treatment (Wk 3)	Treatment	Feed Day	No	13:25	13:00 - 13:59	Afternoon	37,8	119	45,2	45,2	18,95	606,20	128,30
2019/05/31	CH2205	Male	Three y/o	Treatment (Wk 3)	Treatment	Feed Day	No	13:30	13:00 - 13:59	Afternoon	37,6		249,6	249,6	18,75		187,92
2019/05/31	CH2206	Male	Three y/o	Treatment (Wk 3)	Treatment	Feed Day	No	13:30	13:00 - 13:59	Afternoon	37,8	150	66,8	66,8	18,95	621,86	141,81
2019/05/31	CH2205	Male	Three y/o	Treatment (Wk 3)	Treatment	Feed Day	No	13:35	13:00 - 13:59	Afternoon	37,7	86	93,8	93,8	18,85	582,77	153,61
2019/05/31	CH2206	Male	Three y/o	Treatment (Wk 3)	Treatment	Feed Day	No	13:35	13:00 - 13:59	Afternoon	37,8	107	21,0	21,0	18,95	598,73	101,98
2019/05/31	CH2205	Male	Three y/o	Treatment (Wk 3)	Treatment	Feed Day	No	13:40	13:00 - 13:59	Afternoon	37,7	75	24,0	24,0	18,85	572,36	106,55
2019/05/31	CH2206	Male	Three y/o	Treatment (Wk 3)	Treatment	Feed Day	No	13:40	13:00 - 13:59	Afternoon	38,0	88	40,8	40,8	19,15	584,49	124,77
2019/05/31	CH2205	Male	Three y/o	Treatment (Wk 3)	Treatment	Feed Day	No	13:45	13:00 - 13:59	Afternoon	37,8	197	24,2	24,2	18,95	639,25	106,83
2019/05/31	CH2206	Male	Three y/o	Treatment (Wk 3)	Treatment	Feed Day	No	13:45	13:00 - 13:59	Afternoon	37,9	143	159,6	159,6	19,05	618,69	172,19
2019/05/31	CH2205	Male	Three y/o	Treatment (Wk 3)	Treatment	Feed Day	No	13:50	13:00 - 13:59	Afternoon	38,0		27,0	27,0	19,15		110,58
2019/05/31	CH2206	Male	Three y/o	Treatment (Wk 3)	Treatment	Feed Day	No	13:50	13:00 - 13:59	Afternoon	38,0	91	6,0	6,0	19,15	586,98	59,53
2019/05/31	CH2205	Male	Three y/o	Treatment (Wk 3)	Treatment	Feed Day	No	13:55	13:00 - 13:59	Afternoon	38,0		10,0	10,0	19,15		76,76
2019/05/31	CH2206	Male	Three y/o	Treatment (Wk 3)	Treatment	Feed Day	No	13:55	13:00 - 13:59	Afternoon	37,9	91	5,8	5,8	19,05	586,98	58,39
2019/05/31	CH2205	Male	Three y/o	Treatment (Wk 3)	Treatment	Feed Day	No	14:00	14:00 - 14:59	Afternoon	38,0	72	8,4	8,4	19,15	569,20	70,86
2019/05/31	CH2206	Male	Three y/o	Treatment (Wk 3)	Treatment	Feed Day	No	14:00	14:00 - 14:59	Afternoon	37,9	97	24,6	24,6	19,05	591,66	107,39
2019/05/31	CH2205	Male	Three y/o	Treatment (Wk 3)	Treatment	Feed Day	No	14:05	14:00 - 14:59	Afternoon	38,1	62	3,4	3,4	19,26	557,33	40,51
2019/05/31	CH2206	Male	Three y/o	Treatment (Wk 3)	Treatment	Feed Day	No	14:05	14:00 - 14:59	Afternoon	37,9	93	32,8	32,8	19,05	588,58	117,26
2019/05/31	CH2205	Male	Three y/o	Treatment (Wk 3)	Treatment	Feed Day	No	14:10	14:00 - 14:59	Afternoon	38,1		45,8	45,8	19,26		128,76
2019/05/31	CH2206	Male	Three y/o	Treatment (Wk 3)	Treatment	Feed Day	No	14:10	14:00 - 14:59	Afternoon	37,9	99	27,4	27,4	19,05	593,14	111,09
2019/05/31	CH2205	Male	Three y/o	Treatment (Wk 3)	Treatment	Feed Day	No	14:15	14:00 - 14:59	Afternoon	38,0	134	66,0	66,0	19,15	614,33	141,40
2019/05/31	CH2206	Male	Three y/o	Treatment (Wk 3)	Treatment	Feed Day	No	14:15	14:00 - 14:59	Afternoon	37,9	105	27,8	27,8	19,05	597,38	111,58
2019/05/31	CH2205	Male	Three y/o	Treatment (Wk 3)	Treatment	Feed Day	No	14:20	14:00 - 14:59	Afternoon	37,9	105	55,4	55,4	19,05	597,38	135,33
2019/05/31	CH2206	Male	Three y/o	Treatment (Wk 3)	Treatment	Feed Day	No	14:20	14:00 - 14:59	Afternoon	38,0	126	43,8	43,8	19,15	610,14	127,22
2019/05/31	CH2205	Male	Three y/o	Treatment (Wk 3)	Treatment	Feed Day	No	14:25	14:00 - 14:59	Afternoon	37,9	94	32,8	32,8	19,05	589,37	117,26
2019/05/31	CH2206	Male	Three y/o	Treatment (Wk 3)	Treatment	Feed Day	No	14:25	14:00 - 14:59	Afternoon	38,0	114	5,4	5,4	19,15	603,20	55,99
2019/05/31	CH2205	Male	Three y/o	Treatment (Wk 3)	Treatment	Feed Day	No	14:30	14:00 - 14:59	Afternoon	37,9	86	10,8	10,8	19,05	582,77	79,36
2019/05/31	CH2206	Male	Three y/o	Treatment (Wk 3)	Treatment	Feed Day	No	14:30	14:00 - 14:59	Afternoon	38,0	92	2,4	2,4	19,15	587,78	28,91
2019/05/31	CH2205	Male	Three y/o	Treatment (Wk 3)	Treatment	Feed Day	No	14:35	14:00 - 14:59	Afternoon	38,0	68	6,8	6,8	19,15	564,71	63,74
2019/05/31	CH2206	Male	Three y/o	Treatment (Wk 3)	Treatment	Feed Day	No	14:35	14:00 - 14:59	Afternoon	38,0	92	9,0	9,0	19,15	587,78	73,19
2019/05/31	CH2205	Male	Three y/o	Treatment (Wk 3)	Treatment	Feed Day	No	14:40	14:00 - 14:59	Afternoon	38,0	98	106,6	106,6	19,15	592,41	158,07
2019/05/31	CH2206	Male	Three y/o	Treatment (Wk 3)	Treatment	Feed Day	No	14:40	14:00 - 14:59	Afternoon	38,0	135	33,4	33,4	19,15	614,84	117,88
2019/05/31	CH2205	Male	Three y/o	Treatment (Wk 3)	Treatment	Feed Day	No	14:45	14:00 - 14:59	Afternoon	38,0	125	6,0	6,0	19,15	609,60	59,53
2019/05/31	CH2206	Male	Three y/o	Treatment (Wk 3)	Treatment	Feed Day	No	14:45	14:00 - 14:59	Afternoon	38,0	125	143,6	143,6	19,15	609,60	168,49
2019/05/31	CH2205	Male	Three y/o	Treatment (Wk 3)	Treatment	Feed Day	No	14:50	14:00 - 14:59	Afternoon	38,0	88	7,6	7,6	19,15	584,49	67,49
2019/05/31	CH2206	Male	Three y/o	Treatment (Wk 3)	Treatment	Feed Day	No	14:50	14:00 - 14:59	Afternoon	38,0		77,8	77,8	19,15		147,11
2019/05/31	CH2205	Male	Three y/o	Treatment (Wk 3)	Treatment	Feed Day	No	14:55	14:00 - 14:59	Afternoon	38,0	152	21,0	21,0	19,15	622,73	101,98
2019/05/31	CH2206	Male	Three y/o	Treatment (Wk 3)	Treatment	Feed Day	No	14:55	14:00 - 14:59	Afternoon	38,0	94	33,2	33,2	19,15	589,37	117,68
2019/05/31	CH2205	Male	Three y/o	Treatment (Wk 3)	Treatment	Feed Day	No	15:00	15:00 - 15:59	Afternoon	38,0		10,6	10,6	19,15		78,73
2019/05/31	CH2206	Male	Three y/o	Treatment (Wk 3)	Treatment	Feed Day	No	15:00	15:00 - 15:59	Afternoon	37,9	94	50,4	50,4	19,05	589,37	132,06
2019/05/31	CH2205	Male	Three y/o	Treatment (Wk 3)	Treatment	Feed Day	No	15:05	15:00 - 15:59	Afternoon	38,0	65	6,0	6,0	19,15	561,12	59,53
2019/05/31	CH2206	Male	Three y/o	Treatment (Wk 3)	Treatment	Feed Day	No	15:05	15:00 - 15:59	Afternoon	38,0	180	44,6	44,6	19,15	633,61	127,84
2019/05/31	CH2205	Male	Three y/o	Treatment (Wk 3)	Treatment	Feed Day	No	15:10	15:00 - 15:59	Afternoon	38,1	80	4,8	4,8	19,26	577,31	52,04
[truncated: 4,121,953 more chars]
